# Unique high Arctic methane metabolizing community revealed through in situ ^13^CH_4_-DNA-SIP enrichment in concert with genome binning

**DOI:** 10.1038/s41598-021-04486-z

**Published:** 2022-01-21

**Authors:** Ianina Altshuler, Isabelle Raymond-Bouchard, Elisse Magnuson, Julien Tremblay, Charles W. Greer, Lyle G. Whyte

**Affiliations:** 1 Department of Natural Resource Sciences, McGill University, 21,111 Lakeshore Rd., Ste. Anne de Bellevue, QC H9X 3V9 Canada; 2 Energy, Mining and Environment Research Centre, National Research Council of Canada, 6100 Royalmount Ave., Montreal, QC H4P 2R2 Canada; 3 Department of Animal and Aquacultural Sciences, Norwegian University of Life Sciences NMBU, Universitetstunet 3, 1430 Ås, Norway

**Keywords:** Environmental microbiology, Metagenomics, Microbial ecology, Microbiome, Carbon cycle, Biogeochemistry

## Abstract

Greenhouse gas (GHG) emissions from Arctic permafrost soils create a positive feedback loop of climate warming and further GHG emissions. Active methane uptake in these soils can reduce the impact of GHG on future Arctic warming potential. Aerobic methane oxidizers are thought to be responsible for this apparent methane sink, though Arctic representatives of these organisms have resisted culturing efforts. Here, we first used in situ gas flux measurements and qPCR to identify relative methane sink hotspots at a high Arctic cytosol site, we then labeled the active microbiome in situ using DNA Stable Isotope Probing (SIP) with heavy ^13^CH_4_ (at 100 ppm and 1000 ppm). This was followed by amplicon and metagenome sequencing to identify active organisms involved in CH_4_ metabolism in these high Arctic cryosols. Sequencing of ^13^C-labeled *pmoA* genes demonstrated that type II methanotrophs (*Methylocapsa*) were overall the dominant active methane oxidizers in these mineral cryosols, while type I methanotrophs (*Methylomarinovum*) were only detected in the 100 ppm SIP treatment. From the SIP-^13^C-labeled DNA, we retrieved nine high to intermediate quality metagenome-assembled genomes (MAGs) belonging to the *Proteobacteria*, *Gemmatimonadetes*, and *Chloroflexi*, with three of these MAGs containing genes associated with methanotrophy. A novel *Chloroflexi* MAG contained a *mmoX* gene along with other methane oxidation pathway genes, identifying it as a potential uncultured methane oxidizer. This MAG also contained genes for copper import, synthesis of biopolymers, mercury detoxification, and ammonia uptake, indicating that this bacterium is strongly adapted to conditions in active layer permafrost and providing new insights into methane biogeochemical cycling. In addition, *Betaproteobacterial* MAGs were also identified as potential cross-feeders with methanotrophs in these Arctic cryosols. Overall, in situ SIP labeling combined with metagenomics and genome binning demonstrated to be a useful tool for discovering and characterizing novel organisms related to specific microbial functions or biogeochemical cycles of interest. Our findings reveal a unique and active Arctic cryosol microbial community potentially involved in CH_4_ cycling.

## Introduction

Linking microbial function to phylogeny and biogeochemical processes is challenging as the bulk of microbial species are resistant to isolation and laboratory cultivation^1,2^. Nevertheless, this “microbial dark matter” likely has a significant contribution to biogeochemical cycling in many ecosystems^3,4^. This remains one of the biggest hurdles in linking function and biological processes to specific microorganisms and furthering the field of microbial ecology. Genome binning has been used to reconstruct genomes from metagenomic data of organisms that have resisted laboratory culturing^5^. This allows researchers to characterize and study the identity, physiology, and metabolism of these uncultivated microbial species^6^. SIP (stable isotope probing) relies on the incorporation of heavy stable isotopes of elements, usually via a ^13^C-labeled substrate into biological molecules of microorganisms, labeling them in the process^7^. In this way, organisms that can grow and utilize a target ^13^C-labeled substrate or the metabolic products of the original metabolised substrate can be labeled and analyzed (sequenced)^8^. SIP identification of nucleic acids (RNA- and DNA-SIP) is especially useful in microbial ecology as it allows for phylogenetic identification of active organisms that can utilize targeted substrates or their downstream by-products in complex natural communities^9,10^. SIP ex situ labeling has been previously used to identify methanotrophic communities in high Arctic wetlands^11^, Arctic lake sediments^12^, and Arctic soils^13^, though to date in situ SIP labeling directly in the environment has not been reported in cryoenvironments. Combining in situ ^13^CH_4_-SIP with metagenome sequencing followed by genome binning allows potential identification of previously uncultured methanotrophic organism that are active in situ soils.

Methanotrophic organisms play a key role in regulating global methane (CH_4_) emissions to the atmosphere and reducing the CH_4_ atmospheric load. Methanotrophs are characterized by their ability to oxidize CH_4_ and assimilate it as organic carbon^14^. These organisms are phylogenetically diverse, belonging to the phyla NC10, *Verrucomicrobia* and *Proteobacteria* and are further classified as either type I methanotrophs within the *Gammaproteobacteria* or Type II methanotrophs within the *Alphaproteobacteria*^15,16^. All methanotrophs utilize a methane monooxygenase (MMO) enzyme to convert methane into methanol^15^. The two forms of the enzyme are a soluble cytoplasmic form (sMMO) coded by a gene cluster containing an *mmoX* gene, and a particulate membrane bound form (pMMO)^17^. The pMMO is encoded by three consecutive conserved open reading frames: *pmoC*, *pmoA*, and *pmoB*, with *pmoA* coding for the active site^17^. While the soluble methane monooxygenase is only found in some methanotrophs, the pMMO is ubiquitous in all known methanotrophs, with the notable exception of *Methylocella* and *Methyloferula* species, which only contain a soluble form of the enzyme^18^.

Some Arctic soils can act as methane sinks^13,19–24^. This has largely been attributed to a group of methanotrophs with divergent *pmoA* genes, part of the USCα (*Alphaproteobacteria*) and USC_ϒ_ (*Gammaproteobacteria*) clusters of high affinity methanotrophs^25–27^. One member of the USCα clade, *Methylocapsa gorgona* strain MG08, was very recently isolated from landfill soil and definitively shown to be able to oxidize methane at atmospheric concentrations^28^. Culturable strains of this genus with a lower affinity for CH_4_, *Methylocapsa acidiphila* and *Methylocapsa aurea,* were also able to grow on and oxidize CH_4_ at atmospheric concentrations^28^. This is concurrent with an earlier study which demonstrated that culturable low-affinity methanotrophs isolated from rice paddy soils could be responsible for atmospheric methane oxidation as well^29^. Arctic soils range in their negative methane flux from − 0.02 ± 0.01 to − 3.1 ± 1 mg CH_4_ m^2^-d^−1^^19,20,23^; we also previously observed negative methane fluxes in mineral cryosols located on Axel Heiberg Island in the Canadian high Arctic^23,24^. These Arctic soils are predicted to increase in methane consumption due to projected temperature increases in Arctic soils coupled with increased methanotrophy rates^19,30^. Previous studies have shown that *pmoA* genes detected in high Arctic mineral cryosols acting as methane sinks at atmospheric methane consentrations (~ 2 ppm) are phylogenetically related to high affinity methane oxidizers (USCα and USCϒ) from upland forest soils^21,23^. However, to date, these particular Arctic atmospheric methane oxidizers have not been cultured, thus limiting our understanding of their physiology and metabolic potential and ultimately their role in the global methane cycle.

Here, our objective focused on identifying active in situ organisms involved in methane cycling in remote high Arctic ice wedge polygon mineral cryosols that were previously shown to act as methane sinks, this is crucial for understanding future GHG emissions from widespread Arctic permafrost soils that are highly impacted by climate change^23,24^. We used both qPCR (*pmoA* gene abundances) and gas flux measurements to identify candidate soils with high methane oxidation rates; these soils were then used for in situ ^13^CH_4_-SIP labeling to identify organisms that are responsible for the negative methane flux in these Arctic cryosols. We performed *pmoA* and 16S rRNA targeted gene amplicon sequencing of the ^13^C-labeled extracted DNA from the cryosols to identify microbiota involved in methane metabolism at the site. Following this, metagenome sequencing of the ^13^C-labeled DNA was performed in concert with genome binning to yield high and intermediate quality novel MAGs (based on completeness and contamination). These MAGs were analyzed to identify the metabolic potential of non-culturable organisms involved with the methane cycle in high Arctic ice wedge polygon mineral cryosol site.

## Methods

### Study site and selecting target soils via gas flux measurements

The study site (Fig. S1) is adjacent to the McGill Arctic Research Station (MARS), at Expedition Fjord, Nunavut on Axel Heiberg Island in the Canadian high Arctic (coordinates- 79º26'N, 90º46'W). During the summer the active soil layer ranges from 60 to 73 cm in depth, with the top 5 cm at 9 °C ± 0.8 °C^23^. The site is characterized by a high centered ice-wedge polygon terrain. The soils are low in organic and water content and are sparsely vegetated with *Sphagnum*, sedges, and cotton grass^23,31^. The in situ CH_4_ soil gas flux measurements were performed using a static chamber system and analyzed as previously described^24,32^. Gas flux measurements were performed at both the trough and polygon interior soils to identify which soils were hot spots of methane oxidation. Four replicates were collected per each soil type, at two polygons/troughs and over two separate days, the samples were collected over an eight-hour period in 20 ml evacuated glass vials and brought back to the laboratory.

### QPCR analysis of the pmoA gene

Quantitative PCR of the *pmoA* gene and the control 16S rRNA gene was performed using iQ SYBR Green Supermix from BioRad using the manufacturer’s specifications. The qPCR was performed with DNA extracted from the top 0–5 cm of the soil (top) and the soil at 25–30 cm (bottom) collected from both throughs and polygon interiors of the ice-wedge polygon terrain. This resulted in a total of four soil types (each in triplicate); trough top 5 cm; polygon interior top 5 cm; trough bottom 25 cm; polygon interior bottom 25 cm. All soil samples were collected into sterile 50 ml falcon tubes using sterile spatulas. The DNA was extracted from 250 mg of soil per replicate using the UltraClean Soil DNA Isolation Kit (MoBio Laboratories Inc) following the manufacturer’s instructions. Quantitative PCR was performed on three biological replicates from different troughs and polygon interiors per soil type with three total technical replicates per biological replicate. The qPCR reactions were performed on the BioRad iQ5 Multicolour qPCR Detection System and the CT values were acquired. The cycler program used was 3 min at 95.0 °C, followed by 40X of 10 s at 95.0 °C; 45 s at 54.0 s; 45 s at 72.0 °C. The melt curve protocol included increasing the temperature by 0.5 °C every 30 s from 55.0 C-95.0 °C and was ran following the amplification protocol to check for fidelity of the primers and ensure that only one product was produced during the amplification. Primers used for *pmoA* gene qPCR were forward- 5’CCTTCTATCCGATGACCTCT’3 and reverse- 5’CATGAGCGTCCCATATTGCT’3 based on sequences recovered from similar cryosols^23,31^, primers used for the control 16S rRNA gene were forward 5′‐TCCTACGGGAGGCAGCAGT‐3′, and the reverse 5′‐GGACTACCAGGGTATCTAATCCTGTT‐3′^33^. Analysis of the qPCR data included first normalizing the target gene *pmoA* to the control 16S rRNA gene in each sample to control for DNA quality, quantity, extraction contaminants, and inhibition, thus producing the ∆CT (delta cycle threshold) values^34^. The CT used was an average of the three technical replicates for each sample. The 5 cm trough soil was used to calibrate and calculate the relative changes in gene abundance between all the soil types via the standard 2^−∆∆CT^ method^34^, this method first normalized the target gene’s (*pmoA* in our case) CT by a control gene’s CT (16S rRNA) for each individual sample giving ∆CT, then there is a second normalization step that uses one of the treatment’s ∆CT (soils) to which all the other samples are calibrated against to give a relative change in abundance (hence the ∆∆CT)^34^. ANOVA and Tukey t-test were used to check for significance between samples. This approach does not provide the total gene copy numbers of the target genes in the soil, rather it is a method to identify any relative differences in the amount of each target gene between the soil samples.

### In situ ^13^CH_4_-SIP enrichments and soil collection

In situ enrichments of the trough soils were performed by placing a closed chamber over the trough soils and injecting CH_4_ into the headspace. The chambers were constructed by outfitting inverted plastic flowerpots (12 cm in diameter, 11.5 cm height). The pots were wedged into the soil a depth of 2 cm and packed around with soil to help reduce gas loss; the pots were opaque to reduce any warming from a greenhouse gas effect. The pots were outfitted with rubber stoppers for injecting the gas. The headspace of the soils was injected with ≥ 99.0% CH_4_ gas (Sigma-Aldrich) to a final concentration of 100 ppm and 1000 ppm of ^13^CH_4_ gas to ensure sufficient in situ labeling, in triplicate for each treatment, with no CH_4_ augmentation as sequencing comparison control to the ^12^C and ^13^C bands, and a ^12^CH_4_ labeling control. The gas was refilled every other day for a total incubation time of 12 days in situ, the maximum amount of time possible given the logistical constraints of the field season. A previous ex situ study with high Arctic soils in laboratory microcosms reported sufficient labeling of DNA in as little as 8–12 days; however these were performed with supplemented nitrate mineral salts medium (NMS) media and in sealed vials with shaking^13^. Refilling ensured that there would still be sufficient ^13^CH_4_ to label active microorganisms in the event that gas was to diffuse out of the chamber through the soil and be lost to the atmosphere. The soils from the ^13^C-CH_4_ enrichment treatments and controls were collected in 50 ml Falcon tubes and immediately frozen at − 20 °C for transport to the laboratory. For the top 5 cm soil samples, the soil was collected by first measuring depth and then homogenizing soil at 0–5 cm. The bottom 25 cm soil sample was collected by digging a cross section in the soil, measuring depth, and homogenizing the soil at 25–30 cm.

### DNA extraction, CsCl gradient centrifugation, 16S rRNA/*pmoA* and metagenomic sequencing

The DNA was extracted from 10 g of soil per replicate using the DNeasy PowerMax Soil Kit (MoBio) following the manufacturer’s instructions. Separation of the CsCl gradient followed the method from Martineau et al.^13^. A concentration of 1.72 g mL-1 CsCl was used to create a gradient during ultracentrifugation at which separation of the heavy and light band could be achieved in high Arctic soils^35^. The heavy and light DNA bands were visualized using the Safe Imager blue light transilluminator and extracted with an 18-gauge needle as previously described for high Arctic soils^13^. The DNA samples were further purified to remove residual salt using the QIAEX II Gel Extraction Kit (Qiagen, Germany). Illumina libraries for *pmoA* and *16S rRNA* targeted gene sequencing were prepared using the Illumina targeted amplicon sequencing protocol with Nextera XT DNA indices. Primers used for *pmoA* amplification were A189F (5′-GGN GAC TGG GAC TTC TGG-3′) and Forest675R (CCY ACS ACA TCC TTA CCG AA′)^36^ and primers used for 16S were 515F-Y (5′-GTG YCA GCM GCC GCG GTA A-3′) and 926R (5′-CCG YCA ATT YMT TTR AGT TT-3′)^37^. Sequencing was performed on an Illumina MiSeq, using the V2 chemistry with the 500-cycle kit, generating 2 × 250 paired-end reads. For metagenome sequencing, the Nextera XT DNA Library Preparation Kit (Illumina, California, USA) was used to prepare three metagenome libraries using the heavy ^13^C-DNA from the 100 ppm ^13^CH_4_-SIP enrichment with the 600-cycle V3 kit (Illumina). Sequencing was performed on an Illumina MiSeq, generating 2 × 300 paired end reads as summarized in Table S2.

### Bioinformatics

Metagenomic reads were trimmed with Trimmomatic (v0.36, with settings LEADING:3, TRAILING:3, SLIDINGWINDOW:4:15, MINLEN:36)^38^. Raw reads were mapped back to contigs using BBMap^39^. Trimmed reads from triplicate samples were merged and assembled with Megahit (v1.1.3, default settings)^40^ with 50% of reads being mapped to the assembly. Assembled contigs were binned with MetaBAT2 (v2.12.1, default settings)^41^. The completeness, contamination, taxonomic classification, and taxonomic novelty of the MAGs were estimated with three independent tools to corroborate the results, we used CheckM (v1.0.13)^42^, the Microbial Genomes Atlas Online (MiGA 0.7.23.0)^43^, and GTDB (v 1.0.2, R89) analysis^44^. Gene prediction in the Megahit assembly was carried out with MetaGeneMark (v3.25)^45^. HMM models of *pmoA*, *pmoB*, and *pmoC,* and the *mmoX* genes were created with HMMER (v3.2.1) (hmmer.org), these models are available in the Supplemental File S1. These HMM models were added to the existing Pfam database (v31.0) to create a custom database, since all the *pmoCAB* and the *mmoX* genes were not part of the current Pfam database. Predicted genes identified by MetaGeneMark were annotated with this database using the HMMER hmmscan function. Annotation of the MAGs was corroborated with the RAST pipeline^46^ and the JGI MGAP pipeline^47^, and annotation of the metagenomes was performed with the DOE-JGI MAP Pipeline^48^. The predicted 3D protein structure modeling of the *mmoX* gene in MAG #21 and other key genes on the same contig were performed with SWISS-MODEL using the default setting to match the sequence to protein structures in the PDB (protein data bank)^49^. For the *mmoX* gene, following modeling, we constructed a dendrogram based on the predicted protein structure of the MAG #21 *mmoX* and publicly available sMMO, pMMO, and AMO 3D protein templates and predicted models with the DALI algorithm^50^. In addition, we used the I-TASSER/COFACTOR algorithms^51^ with the MAG #21 *mmoX* gene and other key genes on the same contig to deduce protein function through Enzyme Commission (EC) number and active sites using structure comparisons^51^.

For amplicon sequencing, the forward and reverse reads of the 16S rRNA gene and *pmoA* gene were merged and clustered by 97% and 90% identity respectively^52,53^. The 16S rRNA gene amplicons were analyzed using a published python pipeline by Trembley and Yergeau^54^. The *pmoA* taxonomic assignments were performed with a *pmoA* custom database from the Fungene Database^55^. Principal Coordinate Analysis was performed using Bray–Curtis dissimilarity distance matrix analysis of the 16S rRNA gene sequences from heavy and light bands of the SIP-CH_4_ enrichment at 100 ppm and 1000 ppm, and the control soils that were not enriched in CH_4_.

## Results

### Methane oxidation hotspot identification

**Figure 1:**
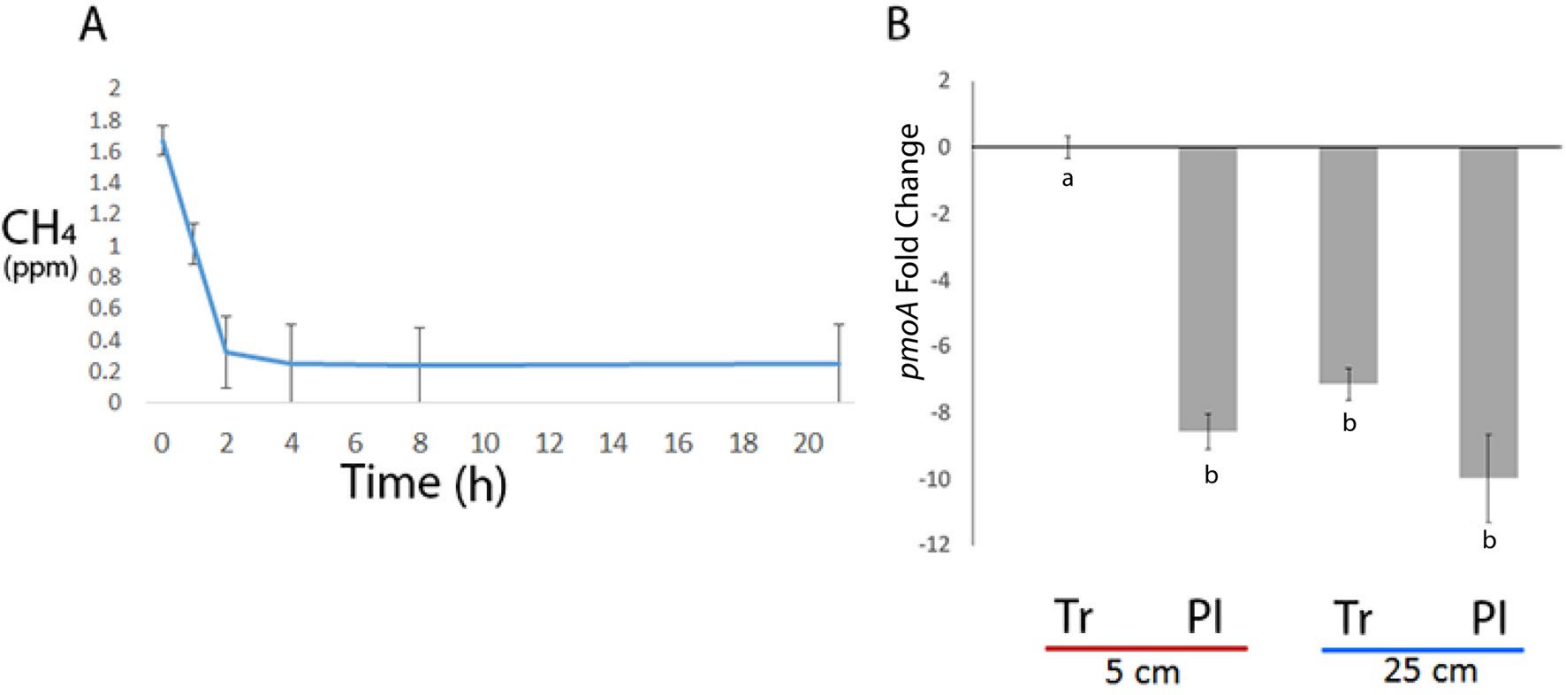
Methane gas flux and qPCR at the ice-wedge polygon terrain. (**A**) The average CH_4_ gas measurements in the static chambers across the entire ice-wedge polygon terrain. The CH_4_ gas flux across the entire site (taking into consideration both polygon interiors and troughs) was − 6.23 (± 1.39) mg CH_4_ m^−2^ day^−1^. Specifically, the flux it was − 8.47 mg CH_4_ m^−2^ day^−1^ in trough soils and − 3.99 mg CH_4_ m^−2^ day^−1^ in polygon interior soils. (**B**) Quantitative PCR of the relative abundance of the *pmoA* particulate methane monooxygenase gene in the trough (Tr) and polygon interior (PI) soils at the top 0–5 cm and bottom 25–30 cm of the ice-wedge polygon terrain. The fold-change axis is relative to the 5 cm trough samples. Bars represent the SEM. Letters represent statistically different fold-change of *pmoA* across all soil comparisons based on Tukey t-test.

The flux of CH_4_ at the overall ice-wedge polygon site was negative, with CH_4_ concentrations in some of the replicates going below the detection limit of the GC at 0.1 ppm. The CH_4_ flux from the soils across the entire ice-wedge polygon site was on average -6.23 (± 1.39) mg CH_4_ m^−2^ day^−1^ across the overall study site (Fig. 1) with the negative flux being more pronounced in trough soils at − 8.47 mg CH_4_ m^−2^ day^−1^, compared to polygon interior soils at − 3.99 mg CH_4_ m^−2^ day^−1^. The qPCR analysis was performed in corroboration with the gas flux measurements to identify relative methane oxidation hot spots. We detected relatively higher levels of *pmoA* genes in trough soils exhibiting higher negative methane flux and measured higher levels of *pmoA* genes in top 5 cm of trough soils relative to polygon interior soils and deeper soils (Fig. 1; Table S1), though it is possible that the absolute values could be higher in either soil.

### Community analysis and *pmoA* gene analyses of the ^13^C labeled DNA

**Figure 2:**
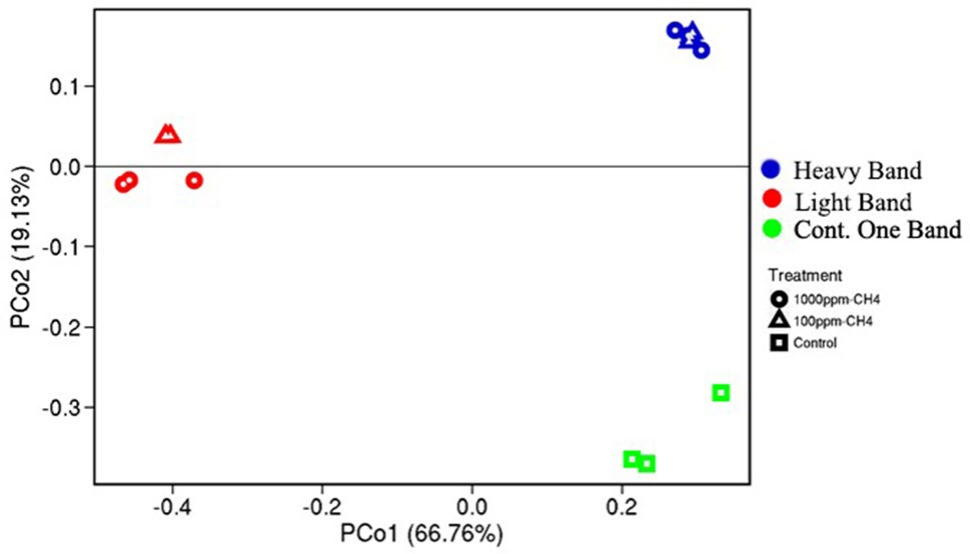
Beta-diversity measure of the microbial communities based on 16S rRNA gene sequences. PCoA Bray–Curtis analysis of the microbial community composition in the heavy and light bands from the ^13^CH_4_ SIP enrichment at 100 ppm and 1000 ppm, as well as the composition of the control soils that were not enriched in CH_4_.

**Figure 3:**
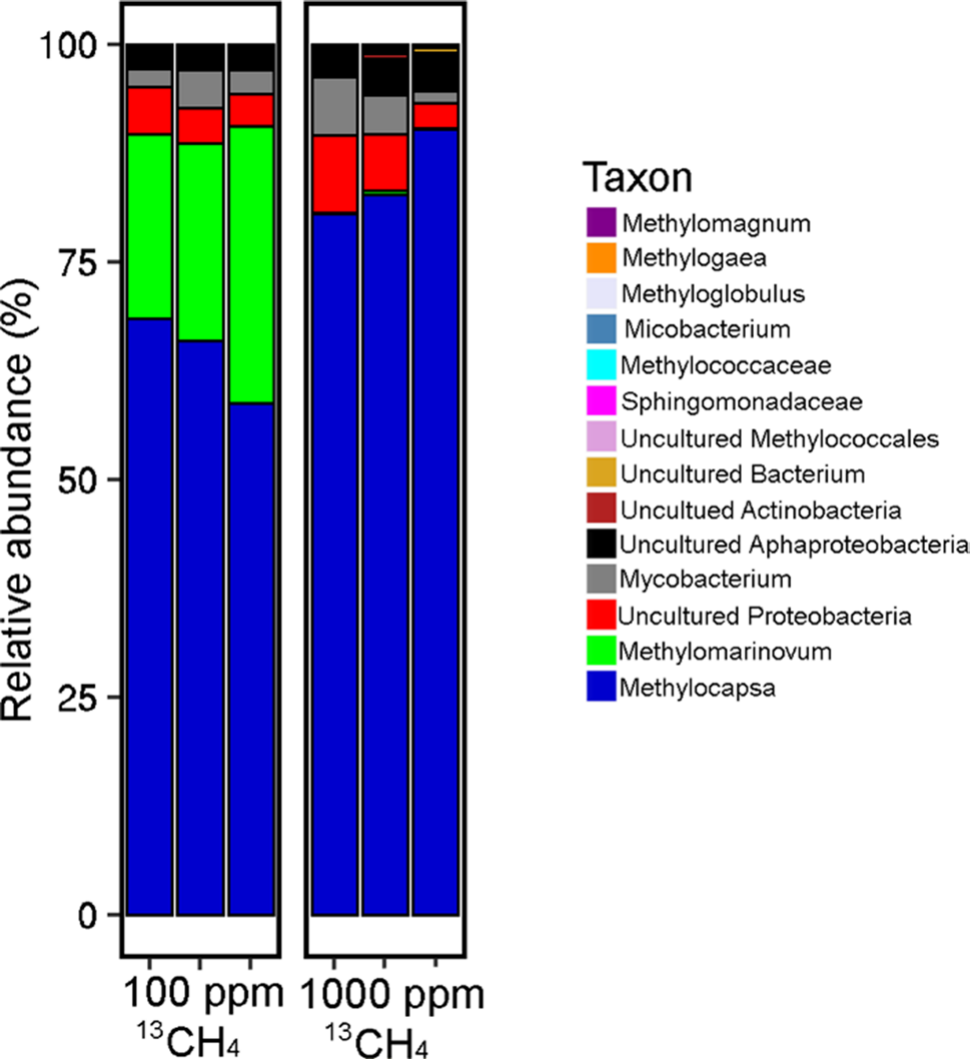
The *pmoA-*containing microbial community profile in the heavy ^13^C-DNA labeled band from the ^13^CH_4_ SIP enrichment at 100 ppm and 1000 ppm. The *pmoA* from the ^12^C-DNA band was not able to be amplified, suggesting no dormant *pmoA* containing methanotrophs in the SIP enrichments.

The in situ 100 ppm and 1000 ppm ^13^CH_4_-SIP enrichments resulted in a clear separation of the heavy labeled DNA from the light DNA band via the CsCl gradient, with only one band present in control samples (Fig. S2). To the best of our knowledge, this was the first successful attempt to perform in situ ^13^CH_4_-SIP in a polar permafrost environment. The microbial community composition in the ^13^C-DNA heavy labeled band and the ^12^C-DNA light band of the ^13^CH_4_-SIP enrichments grouped separately from each other and from the negative control non-enriched DNA samples (Figs. S3, 2), indicating separation of the ^13^C-enriched DNA from the ^12^C-DNA during the extraction. Beta diversity (16S rRNA gene) analysis separated the communities based on two principal components that explained 85.89% of the variation between the samples (Fig. 2). The heavy ^13^C-DNA sequences were enriched in *Proteobacteria* and *Verrucomicrobia* based on the 16S rRNA gene (Fig. S3). The *pmoA* gene was only amplified in the ^13^C-DNA heavy band, but not in the ^12^C-DNA light band. The majority of the *pmoA* sequences in the 100 ppm and 1000 ppm SIP ^13^CH_4_ enrichments belonged to *Alphaproteobacteria* type II methanotrophs (*Methylocapsa* genus) (Fig. 3). The 100 ppm treatment was also enriched for *Gammaproteobacteria* type I methanotrophs (*Methylomarinovum)* compared to the 1000 ppm treatment (Fig. 3).

### Metagenome binning of ^13^C labeled DNA

To further characterize the functional potential, in general, and methane metabolism of the microbial communities within these representative mineral cryosols more specifically, we performed metagenomic sequencing of the 100 ppm, ^13^CH_4_-SIP labeled DNA. Since we were able to label the DNA in situ with the lower ^13^CH_4_ concentrations of 100 ppm, (compared to 1000 ppm) we decided to focus on these samples because they also had a higher methanotroph diversity based on *pmoA* amplicon sequencing. Overall, the three biological replicate SIP metagenomes (~ 8 Gbp) contained genes involved in nitrogen fixation, denitrification, ammonia assimilation, inorganic sulfur assimilation, degradation of aromatic compounds, and fermentation processes that are common in soil metagenomes (please find the fully annotated metagenomes on JGI-GOLD #Ga0374936). Based on the DOE-JGI Metagenome Annotation Pipeline, genes for soluble MMO were detected in the metagenomes, but not genes for the particulate MMO, with the exception of one *pmoB* (related to *Bradyrhizobium)* (Table S4). Genes for formaldehyde assimilation via both the serine and the ribulose monophosphate pathways, as well as methanol dehydrogenases, were also detected (Table S4).

Genome binning with MetaBAT from the ^13^C-DNA band of the 100 ppm ^13^CH_4_ SIP labeled metagenomes resulted in 28 bins, with nine bins of intermediate to very high quality (assigned by MiGa algorithm): these MAGs (Metagenome-Assembled Genomes) ranged in completeness between 88.3 and 30.6%, with contamination ranging between 0.9 and 4.5%, based on copy number of essential genes^43^ (Table 1). To our best knowledge, combining in situ SIP metagenomic sequencing with genome binning has not been attempted before. Based on the lowest taxonomic level able to be classified, the high to intermediate quality MAGs included members of *Proteobacteria, Chloroflexi, Betaproteobacteria*, *Burkholderiales*, *Thiobacillaceae*, *Gemmatimonadetes*, and *Acidobacteria*. Seven of the nine MAGs were only classified with confidence to the Phylum, Class, and Order levels, suggesting these are likely novel clades (Tables 1, S2). These assignments were based on a combination of CheckM and average amino acid identity (AAI), which has been recommended to phylogenetically assign distantly related genomes, as the 16S rRNA genes are not always detected in binned genomes^43,56^. Using this approach, our analyses indicated that the MAGs may constitute novel non-cultured microbial clades at the class (MAGs 21, 22, 27), family (MAGs 8, 20), genus (MAGs 6, 24), and species (MAGs 15, 16) levels (Table 1), suggesting that combining SIP with genome binning is a useful approach for identifying and characterizing novel microorganisms and pathways.

**Table 1:** High to intermediate quality MAGs from the 100 ppm ^13^CH_4_-SIP enrichment from ice-wedge polygon soils.

MetaBAT Bin (MAG #)	Complet. (% via MiGA)	Contam. (% via MiGA)	Classification based on AAI (MiGA)	GTDB classification	Quality (via MiGA)	Predicted proteins (MiGA)	Methanotrophic gene markers
6	79.3	0.9	Betaproteobacteria* (*p* = 0.000)	*Gammaproteobacteria;* *Burkholderiales*	High (74.8)	3430	*pmoB*
8	80.2	3.6	Betaproteobacteria* (*p* = 0.00) *Nitrosomonadales* (*p* = 0.278)	*Gammaproteobacteria;* *Burkholderiales*	High (62.2)	4027	*pmoB*
15	88.3	0.9	*Comamonadaceae* (*p* = 0.000) *Ramlibacter* (p = 0.188)	*Gammaproteobacteria*; *Burkholderiaceae; Ramlibacter*	Very high (83.8)	4334	
16	53.2	2.7	*Thiobacillaceae* (*p* = 0.001) *Thiobacillus* (p = 0.293)	Hydrogenophilaceae; *Thiobacillus*	Intermediate (39.7)	3435	
20	30.6	0.9	*Gemmatimonadetes* (*p* = 0.000)	*Gemmatimonadetes;* *Gemmatimonadales*	Intermediate (26.1)	2476	
21	62.2	4.5	*Chloroflexi* (*p* = 0.007) Thermomicrobia (p = 0.271)	*Chloroflexota;* *Dehalococcoidia*	Intermediate (39.7)	2990	*mmoX*
22	33.3	1.8	*Proteobacteria* (*p* = 0.000) *Betaproteobacteria* (*p* = 0.214)	*Gammaproteobacteria*; *Burkholderiales*	Intermediate (24.3)	1913	
24	30.6	0.9	*Betaproteobacteria* (*p* = 0.000) *Burkholderiales* (*p* = 0.176)	*Gammaproteobacteria;* *Burkholderiales; Rhizobacter*	Intermediate (26.1)	2023	
27	20.7	0.0	*Acidobacteria* (*p* = 0.046) *Solibacteres* (*p* = 0.347)	*Acidobacteriota*; *Thermoanaerobaculia*	Intermediate (20.7)	3442	

*The former class *Betaproteobacteria* was reclassified as an order within the class *Gammaproteobacteria*, thus the discrepancy between MiGa and GTDB results for MAGS #6, 8, 24. Other inconsistencies may be due to ongoing rearrangement of microbial phylogeny clades at different levels.

The HMMER algorithm uses probabilistic models to detect new homologous sequences and novel proteins with similar function to previously identified proteins. It is therefore able to identify divergent homologs that may not be detected with BLAST^57^. The sequence similarity significance is assessed using low *E*-values (E < 0.001) to infer homology^57,58^. The ^13^CH_4_-SIP labeled metagenomes contained both *pmoA* and *mmoX* methane monooxygenase genes based on HMMER scans with the Pfam database. Three of the nine high – intermediate quality MAGs (# 8, #16, and #21; Table 1) contained methane oxidation (*mmoX,* or *pmoB)* genes that were also identified with the HMMER algorithm, but which were not detected through homology annotation.

**Figure 4:**
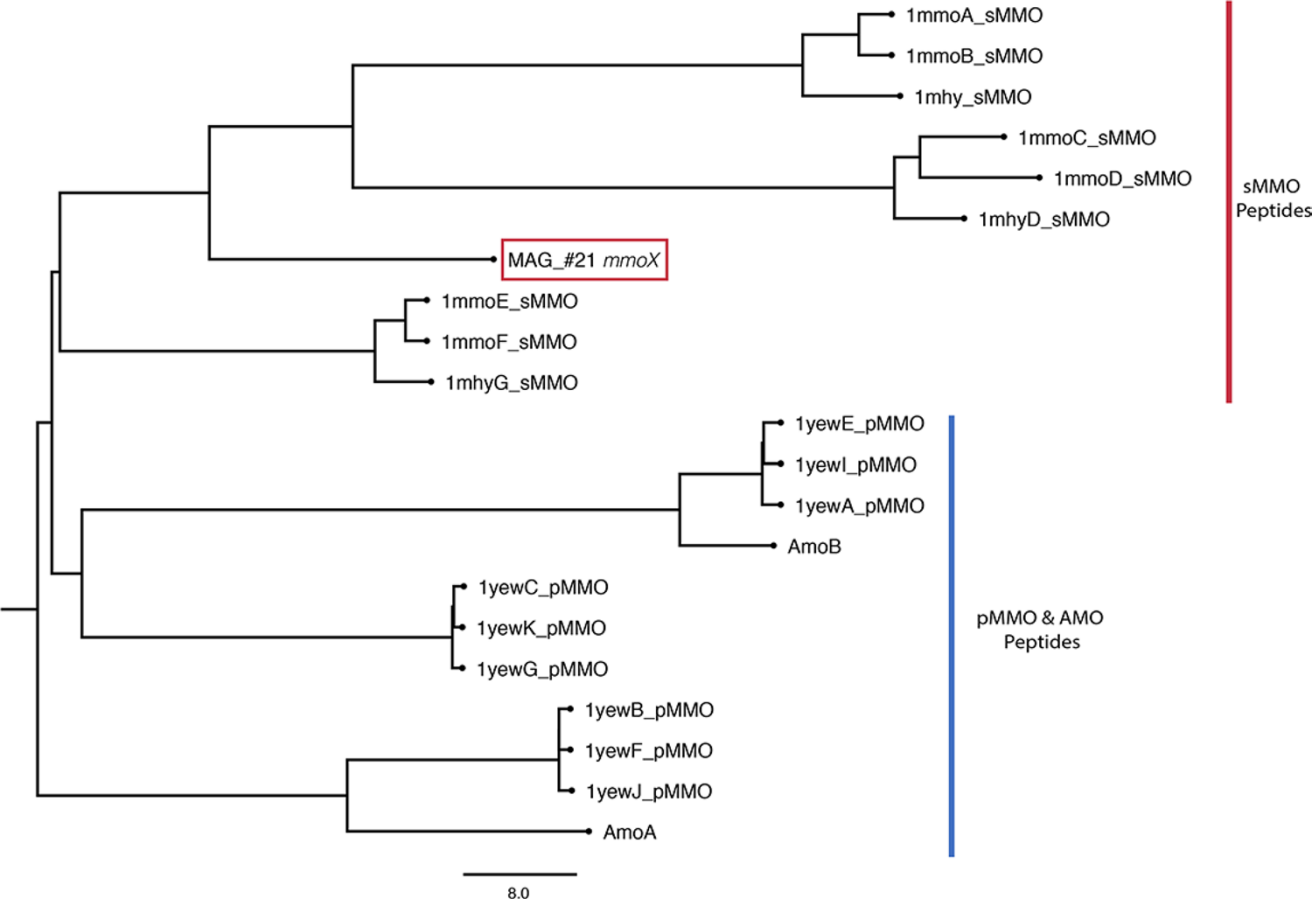
Dendrogram based on the predicted protein structure of the *mmoX* with MMO protein templates from PDB (1mmo-sMMO, 1mhy-sMMO, 1yew-pMMO) and predicted 3D models of AMO genes (Q04507-AmoA, Q04508-AmoB), preformed with the DALI algorithm^50^.

**Figure 5:**
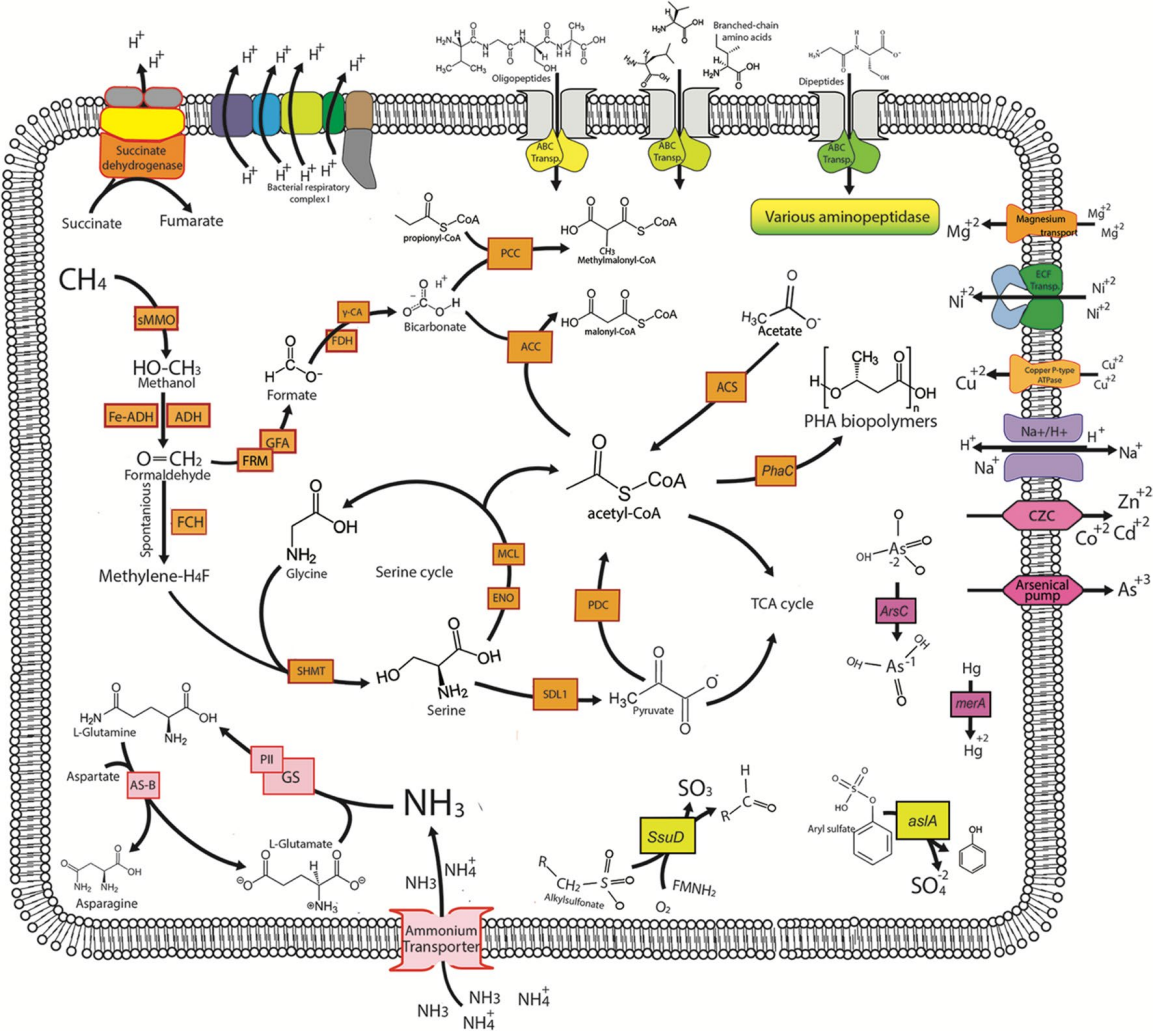
Schematic of theoretical cell involved in methane cycling in Arctic cryosols, based on MAG #21 that was binned from the 100 ppm ^13^CH_4_ SIP in situ enrichment.

Though we did not identify any high-quality MAGs with all three *pmoCAB* genes, an *mmoX* gene was identified in MAG #21 through the Pfam database, thus identifying it as a candidate potential methane oxidizer. Subsequent 3D protein modeling overall corroborated this result, with SWISS-MODEL confirming the structure and I-TASSER/COFACTOR confirming the EC number and active site but not structure (File S3; Fig. 4). This MAG could only be confidently classified down to the *Chloroflexi* phylum (*p* value: 0.006) and thus appears to be a novel order (*p* = 0.05), or perhaps even a novel class (*p* = 0.11) within the *Chloroflexi* phyla (Tables 1, S3). The JGI pipeline was able to identify a small fragment of the 16S gene in MAG #21. This partial 16S sequence covers the beginning of the 16S gene and includes the first and second conserved regions as well as the first variable region. Based on a subsequent NCBI BLAST search this gene fragment most closely matched to uncultured bacteria, with one close match to an uncultured *Chloroflexi* bacterium (File S2). This also corroborates the MiGa, GTDB, and JGI classifications. Through a combination of RAST, MGAP, and HMMER analysis we determined that MAG #21 also contains other genes involved in methane metabolism and 1-C carbon assimilation, including genes (*Eno*, *Mdh*, *Hpr*, *Ppc*, *Mcl1*, and *sgaA*) for the serine pathway of assimilating formaldehyde (Fig. 5).

## Discussion

Soil CH_4_ flux ranges from positive to negative across the Arctic. For example, wet, low centered polygonal Siberian tundra and peat lands act as sources of CH_4_^59^, while upland polar desert soils act as CH_4_ sinks^20,21,23,60^. Furthermore, CH_4_ uptake (− 0.15 to − 0.23 mg CH_4_/m^−2^ day^−1^) has been previously observed in ice-wedge polygonal terrain, though the effect of terrain topology was not studied^61^. As flux of CH_4_ is related to soil moisture and thaw depth of the permafrost^62^, understanding the flux of CH_4_ at a local scale is important in identifying methane oxidation hot spots. During the 2015 summer season, the flux was more negative in the wetter trough soils possibly because very low moisture content of the even drier polygon interior soils inhibited microbial growth and activity overall, including methanotrophs. The qPCR analysis supported this hypothesis as higher levels of *pmoA* genes relative to 16S rRNA genes were detected in trough soils which exhibited higher negative methane flux. Overall, the qPCR results in combination with gas flux results indicated that the trough top 5 cm soils were hotspots for atmospheric methane oxidation in ice wedge polygon soils during the 2015 summer season. Therefore, the trough soils were targeted for in situ SIP analyses with ^13^C-labeled CH_4_ to identify active methanotrophic members.

Despite the atmospheric (2 ppm) methane oxidation present in these soils^23^, we performed the in situ labeling experiments at 100 ppm and 1000 ppm of CH_4_ to ensure adequate labeling of any active methanotrophs and organisms that were using downstream methanotrophic metabolic by-products (ie cross-feeding) in the soils. These soils have also been previously shown to contain methanogens, especially in deeper soils layers^31,61^, suggesting that these soils may have methanotrophs specialized in obtaining methane from pulses of higher methane produced from deeper soils as well as uptake of atmospheric methane. Based on 16S rRNA gene amplicon sequencing, the heavy ^13^C-DNA bands were enriched in *Proteobacteria* and *Verrucomicrobia* (Fig. S3), while the light ^12^C-DNA band was enriched for *Bacteroidetes* (Fig. S3). Similar results were previously reported in other studies with laboratory ^13^CH_4_-SIP labeling^13,63,64^. Our study detected both type I and type II methanotrophs in ^13^CH_4_-SIP in situ, while Martineau et al.^13^ detected only type I methanotrophs (*Methylobacter and Methylosarcina; Gammaproteobacteria*) in Eureka (Ellesmere Island, Nunavut) ^13^CH_4_-SIP laboratory soil microcosm incubations. Esson et al.^64^ found SIP-labelled DNA enriched in *Methylocystis* (type II), *Methylomonas*, and *Methylovulum* (both type I) in organic Boreal peat bog ^13^CH_4_-SIP laboratory soil microcosms while specific actively labeled methanotroph genera in our mineral cryosoils belonged to *Methylocapsa* (type II) and *Methylomarinovum* (type I) indicating that unique methanotrophic populations exist in these Arctic cryosols. This difference may be due to differences in organic content between the soils and the high organic content of peat favoring methanogenesis and, therefore, likely favoring lower affinity methanotrophs.

The *pmoA* gene was only amplifiable in heavy ^13^C-DNA and was not detected in the light non-labeled DNA, indicating that we were likely not able to identify any dormant/inactive methanotrophs. The observed amplification of *pmoA* was unlikely to be caused by cross-amplification with *amoA* since the primers used for sequencing of the *pmoA* have been shown to be very specific and do not amplify *amoA* from ammonia oxidizers^65^. The dominant *pmoA* sequences in the 100 ppm and 1000 ppm SIP ^13^CH_4_ enrichments were assigned as the *Methylocapsa* genus (*Alphaproteobacteria* type II methanotroph) (Fig. 3). This genus is phylogenetically closest to the uncultured USCα cluster, members of which are hypothesized to be high affinity methanotrophs capable of atmospheric methane oxidation^66^. Significantly, Tveit et al.^28^ demonstrated the ability of culturable members of the *Methylocapsa* genus to oxidize methane at atmospheric concentrations and Belova et al. have demonstrated presence of *Methylocapsa* in forest tundra^67^. These studies taken together with our results suggests that these organisms are likely responsible for the methane sink observed in Arctic cryosols.

Interestingly, the 100 ppm ^13^CH_4_-SIP labeled treatment was also enriched for *Gammaproteobacteria* type I methanotrophs compared to the 1000 ppm treatment (Fig. 3). These most dominant type I *pmoA* sequences were taxonomically assigned to *Methylomarinovum* (Fig. 3), a methanotrophic genus that is very rarely detected in environmental samples^18^, although a member of this genus has been isolated from a submarine hydrothermal system^68^. The enrichment of this genus at a lower methane concentration SIP suggests its potential importance in methane cycling in Arctic cryosols: successful isolation and characterization of culturable *Methylomarinovum* strains would help confirm its contribution to methane fluxes in Arctic ecosystems. The overall apparent low diversity of *pmoA* methanotroph sequences in our Arctic cryosol samples is consistent with another studdy that demonstrated Arctic habitats (wetlands) having a lower diversity of *pmoA* compared to their more temperate counterparts^11^.

While the type II methanotrophs (based on *pmoA* gene amplicon sequencing) were primarily labeled in the CH_4_-SIP experiment, the consistent SIP enrichment of type I methanotrophs, at lower CH_4_ concentrations (100 ppm) compared to higher (1000 ppm) concentrations in situ*,* is intriguing. Laboratory competition experiments with type I and type II pure cultures of methanotrophs have shown type I methanotrophs to be more competitive at lower methane concentrations^18,69^ while rice field studies have shown conventional type II methanotrophs to be more positively correlated with higher methane concentrations compared to type I methanotrophs^70^. However, type II methanotrophs have also been shown to have a higher potential to be active when methane concentrations drop below 1000–100 ppmv^18^. While some type II methanotrophs (*Methylocapsa*) were shown to be atmospheric methane oxidizers, this is yet to be definitively shown for any type I methanotrophs*.* Our in situ ^13^CH_4_-SIP results also suggest that the type II *Methylocapsa* is active in Arctic cryosols, but the additional labeling of type I methanotrophs at the lower CH_4_ concentrations in situ suggests that these organisms may also contribute to methane oxidation in Arctic cryosol soils.

The MAGs reconstructed from the labeled ^13^CH_4_ metagenomes in this study belong to microbial clades that have not previously been associated with methanotrophy. This could be due to (1) these MAGs constitute novel organisms that are able to oxidize methane; (2) they are tangentially involved in the methane cycle and are acquiring the labeled ^13^C through syntrophy (cross-feeding)^71^, or (3) unlabeled DNA diffused throughout the gradient during handling. Detection of methanotrophy marker genes in the MAGs would suggest the first option, while lack of these marker genes in MAGs would suggest the second, although it is also possible that the marker genes were not binned into the MAGs retrieved. Detecting methanotrophy marker genes in the three MAGs (# 8, #16, and #21) suggested that these are potentially novel lineages that could be involved in methane cycling, with the *Chloroflexi* MAG #21 being a potential novel methane oxidizer due to presence of additional methane metabolism and 1-C carbon assimilation genes.

The serine pathway assimilation genes identified in a *Chloroflexi* MAG #21 are typically utilized by Type II *Alphaproteobacteria* methanotrophs. However, we did also identify genes coding for two carboxylating enzymes, acetyl-CoA and propionyl-CoA carboxylases, which are otherwise present as part of the 3-Hydroxypropionate Bi-cycle pathway of fixing CO_2_ in two other *Cloroflexi* bacterium, *C. aurantiacus*^72^ and *Ca. C. photoalkanotrophicum*^73^. Methanotrophic *Verrucomicrobia* and the candidate phylum NC10 rely on the Calvin-Benson-Bassham (CBB) cycle for autotrophic growth following oxidation of methane through to CO_2_^74,75^. A select few *Proteobacterial* methanotrophic species rely on a combination of the serine/RuMP pathways and the CBB pathway to assimilate carbon following CH_4_ oxidation^76^, similarly this *Chloroflexi* MAG #21 appears to have potential to use multiple pathways to assimilate carbon once it has been oxidized from CH_4_. Nitrogen acquisition in this MAG appears to be through ammonia uptake and assimilation. Genes for uptake and utilization of amino peptides and amino acids were also present, indicating that if this organism does utilize CH_4_, it may also use other carbon sources, indicating a mixotrophic metabolism strategy. Interestingly, MAG #21 also contained genes coding for export of cadmium and arsenic and detoxification of mercury via a mercuric reductase (coded by *merA*). Mercury(II) can interfere with methanotrophy rates^77^ as it irreversibly inhibits the sMMO enzyme (coded partially by *mmoX*)^78^: therefore, detoxification of mercury (II) via *merA* is advantageous for *mmoX*-containing methanotrophs. Genes coding for a copper importing p-type ATPase were also present in this MAG: sMMO expression is tightly linked to the availability of copper, with some mutants unable to produce active sMMO in the absence of copper^79^. This MAG also contained genes for synthesis of polyhydroxyalkanoate biopolymers such as poly-3-hydroxybutyrate (PHB)^80^; this pathway is present and expressed in other methanotrophic organisms under nutrient limited conditions, where instead of entering the TCA cycle, serine is funneled into the PHB cycle to generate intracellular storage granules that serve as a C source^80^. Specifically, N-limiting and P-limiting conditions increase the PHB production as a way of storing carbon for future growth once N and P limitation is removed. Stored PHBs can also serve as a source of carbon for synthesis or they can facilitate further methane consumption by providing a source of reducing power^80^. MAG #21 did not appear to contain a particulate methane monooxygenase (pMMO) genes. However, it did contain evidence for a soluble methane monooxygenase enzyme. While the sMMO methanotrophy marker is found in several methanotrophs, the pMMO is ubiquitous to all methanotrophs, with the notable exception of *Methylocella* and *Methyloferula,* methanotrophic lineages which lack a pMMO and rely solely on an sMMO^21,66^. Since MAG #21’s *mmoX* 3D protein structure prediction was more closely related to sMMO than AMO or other proteins (Fig. 4; File S3), this suggests that it may not be a cross-feeder, but potentially a novel methanotrophic lineage lacking pMMO as the *Methylocella* and *Methyloferula* lineages.

Currently, no members of *Chloroflexi* are known to be methanotrophic; however, GTDB analysis identified a close microbial relative to MAG #21 (75% identity) as being another uncultured *Chloroflexi* MAG (FeB_14; GCA_003104995.1) which was originally identified in a laboratory bioreactor where novel anaerobic methanotrophic (ANME) archaea was also sequenced^81^; this non-SIP study was focused on the ANME and did not indicate evidence of methanotrophy in the *Chloroflexi* MAG FeB_14^81^. We performed HMMER analysis with our Pfam database for MAG FeB_14 and identified a potential distant *mmoX-*like gene (though NCBI annotation was a hypothetical protein PWB45011.1). We did not identify *pmoCAB* genes, suggesting that this genus (*G225*, GTDB), that encompasses our MAG #21 and a previously binned MAG FeB_14, does potentially have only a distant *mmoX* gene. *Chloroflexi* members have been previously been suggested as containing pathways for the oxidation of methane and/or other small alkanes^73^, and have also been detected with 16S rRNA gene primers that target Type I methanotrophs^12^. Taken together these studies help corroborate our result that the *Chloroflexi* MAG #21 is a potential methanotroph.

The *mmoX* operon is usually characterized by the presence of additional genes besides the *mmoX*, namely *mmoYBZDC*^82,83^, which encode different components of the enzyme complex, in addition to *mmoGQSR* genes*,* which appear to be involved in regulation of the operon^84^. The genes that are located upstream and downstream of the *mmoX* gene from MAG#21 are presented in Table S5. Many of the genes are hypothetical or have unknown functional assignment. The presence of these specific additional *mmo* genes is not immediately apparent on this contig; however, there are several genes on the contig that do exhibit similarity to characterized components of the soluble methane monooxygenase operon. The gene immediately upstream of *mmoX* in our contig is a hypothetical protein containing an iron-sulfur binding domain (Table S5), this was corroborated by the gene’s 3D protein structure using both SWISS-MODEL and I-TASSER programs (File S3). This is characteristic of *mmoC*, an iron-sulfur flavoprotein which acts as the reductase component of methane monooxygenase^85,86^ as iron is a necessary co-factor for most soluble methane monooxygenases. The gene preceding this iron-sulfur binding protein is a predicted phenylacetic acid catabolic type protein^87^. Enzymes involved in phenylacetic acid catabolism belong to a subgroup of monooxygenases, which includes methane monooxygenases, within the family of bacterial di-iron multicomponent oxygenases^87^. This gene’s 3D protein structure corroborated these results. The SWISS-MODEL, similar to Pfam classification, predicted this upstream gene as being a phenylacetic acid catabolic type protein. The I-TASSER/COFACTOR 3D modeling results predicted it to potentially be part of an actual sMMO like protein based on its structural analogs and predicted function (File S3). Several genes with potential roles in transcriptional regulation and signal transduction, not unlike *mmoQSR*, were also seen further downstream on the contig. The MAG #21 *mmoX* contig also contains a sigma-70 factor, known to be necessary for methanotrophy. However, to date, sigma-70 has been shown to be specific to particulate methane monooxygenase, while the soluble methane monooxygenase appears to be under the control of sigma-54^88–90^. While the presence of these *mmoX* associated genes does not conclusively prove that the *Chloreflexi* MAG #21 is a methanotroph, it does suggest that it possesses a methane monooxygenase system. The presence of a *rpoD* (sigma 70) gene instead of an expected *rpoN* (sigma 54) gene suggest a potentially different regulation of sMMO synthesis in this putative methanotroph. In summary, the combination of showing potential methanotrophic activity through SIP labelling with the presence of *mmoX* plus other potential methane oxidation pathway genes within the *Chloroflexi* MAG #21 indicates that this MAG potentially represents a new methanotroph candidate active in Arctic mineral cryosols.

MAG #8 and MAG #16 both contained a *pmoB* methanotrophy gene (Table 1). Based on AAI, the lowest resolved classification showed that MAG #8 may be related to members of the family *Gallionellaceae* (*p* = 0.377), order *Nitrosomonadales* (*p* = 0.278), in the class *Betaproteobacteria* (*p* < 0.001) and that MAG #16 is related to genus *Thiobacillus* (p-value 0.313), family *Thiobacillaceae* (*p* value 0.185), order *Nitrosomonadales* (*p* value 0.0108) in the class *Betaproteobacteria* (*p* value 0.0107). We did not find *pmoA* and *pmoC* gene sequences which normally make up the particulate methane monooxygenase gene cluster (*pmoA* and *pmoC*), suggesting that these organisms may not be capable of methanotrophic metabolism and instead more likely acquired ^13^C through cross feeding, though this could also be due to the 80% completeness of the MAG #8 and 53% completeness of MAG #16. We did not find any other genes of interest up- or down-stream of the *pmoB* genes in these two MAGs (Table S6). There are currently no members of the *Betaproteobacteria* known to be capable of methane oxidation^15^ although previous SIP studies have identified some *Betaproteobacteria* 16S rRNA gene sequences in the heavy ^13^CH_4_-RNA labeled fraction from rice field soils^63^ although this was potentially due to cross-feeding as well. A more recent lake sediment DNA-SIP study demonstrated that *Burkholderiales* (former *Betaproteobacteria*, now *Gammaproteobacteria*; see Table 1) are persistent members in methane-oxidizing communities and are likely involved in carbon transfer from the methanotrophs^91^. Thus MAGs #8 and #16 likely acquired the ^13^C through cross-feeding. MAG #8 possesses genes with homology to genes involved in denitrification, including nitric oxide reductase and activation proteins (*nor, norD, norQ*) (Table S7). However, we did not detect other key dentrifier marker genes (*norB and norC)* in MAG #8, though several members of *Nitrosomonadales* are known denitrifiers^92–94^. As members of this family are also known for methylotrophy^95^, MAG #8 may be cross-feeding with other methanotrophs. MAG #8 also possesses the nitrite reductase gene (*nir*) potentially involved in nitrate/nitrite ammonification and genes for ammonia assimilation (Table S7). Generally, bacteria capable of ammonification or dissimilatory nitrate reduction to ammonium (DNRA) are distinct from those capable of denitrification, as both reduction pathways compete for the same source. However, some organisms do possess both pathways^96^. It is common for DNRA bacteria to be also capable of fermentation^97,98^, and MAG #8 possesses numerous fermentative pathways genes (Table S7). While *pmoA* and *pmoC* are lacking in this organism, presence of both *nor* and *nir* is characteristic of aerobic methanotrophs^99^, many of whom utilize ammonium or nitrate as nitrogen sources^100^. Some methanotrophs also utilize these enzymes to detoxify nitrite, a potential inhibitor of methane oxidation^100^. The high Arctic ice wedge polygon soils have low levels (< 0.7 mg/kg) of nitrate and nitrite^24^; therefore, denitrification and DNRA are likely important in these communities although DNRA is often dominate over denitrification in nitrate limiting soils^101^.

In contrast to our results, three recent metagenome binning attempts to identify high affinity methanotroph genomes were performed on an Antarctic mineral cryosoil (Taylor Dry Valley)^27,102^, forest soil (Marburg, Germany)^27^, and permafrost thaw gradient soils (Stordalen Mire)^103^, although without the use of SIP labeling. The Antarctic mineral soil contained a MAG likely belonging to *Gammaproteobacteria* (USCγ) (Type I methanotroph), while the forest and the Stordalen Mire soils contained MAGs most likely related to *Alphaproteobacteria* (USCα), but also *Gammaproteobacteria*^27,102,103^. In contrast to our ice wedge polygon MAG #21 which contained an *mmoX* gene, the forest, Antarctic, and Stordalen Mire soil MAGs mostly contained *pmoCAB* genes (with exception of a *Methylobacter* MAG)^27,102,103^. Methanotroph SIP (and *pmoA* sequencing) labelling previously identified methanotrophs in high Arctic wetlands^11^, Arctic lake sediments^12^, active layer soils^13^, and peatlands^104^. However, these studies all involved taking soils out of the natural environment and performing laboratory incubations in sealed glass microcosms, thus potentially creating artificial selection. Nevertheless, these studies are valuable in helping us understand the biological component of polar biogeochemical methane cycle and the active diversity of methanotrophs found across different polar ecological niches. In this in situ study, both genome binning and *pmoA* sequencing of in situ labeled DNA indicated that type II methanotrophs are likely the dominant active methanotrophs in the high Arctic mineral cryosols examined. Type II methanotrophs utilize the serine pathway of formaldehyde assimilation versus the RuMP pathway that type I methanotrophs utilize^15^. Genes indicative of the serine pathway were more abundant in the triplicate SIP metagenomes and in the binned genomes, compared to genes indicative of the Ribulose bisphosphate (RuBP) cycle. This is consistent with previous SIP studies that showed type II methanotrophs (using the serine cycle) as the dominant methane oxidizers in acidic peat soils^63,64,104^. Type I methanotrophs which use the RuBP cycle have also been shown to contribute to methane consumption, albeit in smaller proportion^64^. However, in contrast to our results, *Gammaproteobacteria* (type I) *Methylobacter* methanotrophs appear to be the dominant active methane oxidizers in Arctic wetlands^11^.

## Conclusions

This is the first study to perform in situ ^13^CH_4_ SIP labeling in high Arctic soils and to then successfully combine SIP labeling with genome binning, which allowed us to infer the unculturable active microorganisms involved in the methane biogeochemical cycling in Arctic cryosol environments. Overall, ^13^CH_4_-SIP in situ labeling followed with amplicon sequencing and in situ gas flux measurements identified the *Methylocapsa* genus (type II methanotroph) as contributing to atmospheric methane oxidation in the oligotrophic Arctic cryosols. This was consistent with the recent Tveit et al.^28^ study that demonstrated *Methylocapsa*’s ability to oxidize atmospheric methane in laboratory pure cultures. Our results demonstrated that this is likely also true in situ in Arctic cryosols. Furthermore, SIP also labeled the *Methylomarinovum* genus (type I methanotroph), suggesting it also has a role in methane oxidation in these Arctic cryosol sites. Further culturing studies are needed to confirm this hypothesis. Metagenomic binning and SIP have both been used to target the uncultured microbial dark matter, while SIP is also useful in linking phylogeny to physiological function. Here we successfully demonstrated that both approaches can be used in concert to first label the microbial community involved in the methane oxidation cycle with SIP, thus reducing the DNA diversity in the sample and allowing for a more robust and targeted approach for metagenome binning. Several high to intermediate quality MAGs were recovered with this strategy, a few of which appeared to contain methane oxidation genes. A majority of the MAGs recovered from the metagenomes contained genes for the serine cycle of assimilating formaldehyde, which could be indicative of type II methanotrophs. A *Chloroflexi* MAG contained many of the methane cycling genes including *mmoX* and serine cycle genes, and also contained genes for biopolymer production and mercury detoxification. Finally, we demonstrated how SIP in conjunction with genome binning is a useful tool for characterizing novel/unique organisms that are related to a specific function or biogeochemical cycle.

## Supplementary Information

Supplementary Information 1.

Supplementary Information 2.

Supplementary Information 3.

Supplementary Figures.

Supplementary Tables.

## Data Availability

Amplicon and metagenome sequencing data used in this study was submitted to the NCBI database under BioProject PRJNA588281. In addition, MAGs #21, #8, and 16 have been deposited to JGI-GOLD under accession numbers Ga0376100, Ga0376102, and Ga0376103. All the MAGs above 15% completeness and below 30% contamination have been submitted to NCBI under BioProject PRJNA588281.
