# Supplementary material for: Unique high Arctic methane metabolizing community revealed through in situ 13CH4-DNA-SIP enrichment in concert with genome binning
Source: Sci Rep. 2022 Jan 21;12:1160. doi: 10.1038/s41598-021-04486-z (PMC8782848; doi:10.1038/s41598-021-04486-z)
Supplement: Supplementary file 1 — Supplementary Information 1. [file 41598_2021_4486_MOESM1_ESM.pdf]

```
HMMER3/f [3.1b2 | February 2015]
NAME  pmoB_IA_loose Protein
LENG  415
ALPH  amino
RF    no
MM    no
CONS  yes
CS    no
MAP   yes
DATE  Tue Jan  2 10:15:21 2018
NSEQ  36
EFFN  0.988770
CKSUM 2327598343
STATS LOCAL MSV      -11.5451  0.69904
STATS LOCAL VITERBI  -12.2042  0.69904
STATS LOCAL FORWARD   -5.9414  0.69904
HMM          A        C        D        E        F        G        H        I        K        L        M        N        P        Q        R        S        T        V        W        Y
            m->m     m->i     m->d     i->m     i->i     d->m     d->d
  COMPO   2.53791  4.58482  3.03978  2.74189  3.20351  2.80189  3.82550  2.73007  2.75598  2.45744  3.54826  3.11791  3.24571  3.13152  2.98379  2.72986  2.78886  2.51096  4.13037  3.48257
          2.68618  4.42198  2.77545  2.73145  3.46356  2.40538  3.72520  3.29351  2.67713  2.69362  4.24506  2.90335  2.73746  3.18161  2.89794  2.37905  2.77468  2.98508  4.58441  3.61529
          0.48378  1.72992  1.57865  1.85227  0.17065  0.00000        *
      1   2.45411  4.14442  3.78559  3.21114  2.99139  3.64704  3.92100  2.32942  2.96253  2.19677  2.28347  3.51819  4.01395  3.38271  3.37476  2.92002  2.82161  2.33017  4.72786  3.18206     19 l - - -
          2.68618  4.42225  2.77519  2.73123  3.46354  2.40513  3.72494  3.29354  2.67741  2.69355  4.24690  2.90347  2.73739  3.18146  2.89801  2.37887  2.77519  2.98518  4.58477  3.61503
          0.02867  3.96202  4.68437  0.61958  0.77255  0.70721  0.67928
      2   2.67667  5.00053  2.71787  2.11850  4.33560  2.99817  3.61413  3.77225  1.80210  3.31092  4.11420  2.86131  3.34195  2.74978  2.75725  2.52539  2.91932  3.39980  5.48653  4.13080     20 k - - -
          2.68618  4.42225  2.77519  2.73123  3.46354  2.40513  3.72494  3.29354  2.67741  2.69355  4.24690  2.90347  2.73739  3.18146  2.89801  2.37887  2.77519  2.98518  4.58477  3.61503
          0.02867  3.96202  4.68437  0.61958  0.77255  0.70721  0.67928
      3   2.55443  4.43205  3.21842  2.66221  3.59787  3.46492  3.29239  2.76585  2.60964  2.34294  3.45265  2.77253  3.85523  2.95436  3.00292  2.50829  2.56267  2.72277  4.99179  3.72431     21 l - - -
          2.68618  4.42225  2.77519  2.73123  3.46354  2.40513  3.72494  3.29354  2.67741  2.69355  4.24690  2.90347  2.73739  3.18146  2.89801  2.37887  2.77519  2.98518  4.58477  3.61503
          0.02867  3.96202  4.68437  0.61958  0.77255  0.65692  0.73074
      4   2.43485  4.19341  3.60597  3.03886  3.33883  3.17941  3.86997  2.16899  2.96851  2.20505  3.11241  3.16555  3.96188  3.25971  3.27899  2.84155  2.63254  2.29286  4.81940  3.60008     22 i - - -
          2.68618  4.42225  2.77519  2.73123  3.46354  2.40513  3.72494  3.29354  2.67741  2.69355  4.24690  2.90347  2.73739  3.18146  2.89801  2.37887  2.77519  2.98518  4.58477  3.61503
          0.02777  3.99330  4.71565  0.61958  0.77255  0.49201  0.94519
      5   2.55320  4.82278  3.05981  2.41961  4.07863  3.46861  3.64347  3.47728  1.78230  3.07676  2.85234  2.85399  3.86025  2.79864  2.67364  2.70295  2.81323  3.16410  5.30850  4.00254     23 k - - -
          2.68618  4.42225  2.77519  2.73123  3.46354  2.40513  3.72494  3.29354  2.67741  2.69355  4.24690  2.90347  2.73739  3.18146  2.89801  2.37887  2.77519  2.98518  4.58477  3.61503
          0.02466  4.11067  4.83302  0.61958  0.77255  0.47746  0.96853
      6   2.53268  4.97748  2.37589  2.27427  4.26815  3.30076  3.60013  3.28164  2.12749  3.25799  3.51721  2.83150  3.79961  2.71892  2.54259  2.60554  2.84454  3.33854  5.44304  4.07018     24 k - - -
          2.68618  4.42225  2.77519  2.73123  3.46354  2.40513  3.72494  3.29354  2.67741  2.69355  4.24690  2.90347  2.73739  3.18146  2.89801  2.37887  2.77519  2.98518  4.58477  3.61503
          0.02344  4.16084  4.88319  0.61958  0.77255  0.51359  0.91216
      7   2.79049  5.09970  3.11707  2.52538  4.45284  3.52982  3.62050  3.86578  1.64437  3.07128  4.16204  2.79029  3.90913  2.52954  2.07287  2.54992  2.99853  3.50062  5.48599  4.18643     25 k - - -
          2.68618  4.42225  2.77519  2.73123  3.46354  2.40513  3.72494  3.29354  2.67741  2.69355  4.24690  2.90347  2.73739  3.18146  2.89801  2.37887  2.77519  2.98518  4.58477  3.61503
          0.02344  4.16084  4.88319  0.61958  0.77255  0.51359  0.91216
      8   2.52601  4.17962  3.73411  3.16110  3.21244  3.36963  3.91869  2.11916  3.08261  2.09075  3.29114  3.26788  4.01188  3.09895  3.36356  2.90251  2.59809  2.15702  4.78813  3.57752     26 l - - -
          2.68618  4.42225  2.77519  2.73123  3.46354  2.40513  3.72494  3.29354  2.67741  2.69355  4.24690  2.90347  2.73739  3.18146  2.89801  2.37887  2.77519  2.98518  4.58477  3.61503
          0.02344  4.16084  4.88319  0.61958  0.77255  0.51359  0.91216
      9   2.20008  4.41581  3.32725  2.76481  3.19364  2.83598  3.77335  2.93915  2.55252  2.53699  3.51533  3.20815  3.91574  2.86716  3.11626  2.76642  2.76791  2.39738  4.98492  3.47608     27 a - - -
          2.68618  4.42225  2.77519  2.73123  3.46354  2.40513  3.72494  3.29354  2.67741  2.69355  4.24690  2.90347  2.73739  3.18146  2.89801  2.37887  2.77519  2.98518  4.58477  3.61503
          0.02344  4.16084  4.88319  0.61958  0.77255  0.51359  0.91216
     10   2.81269  5.01192  3.20862  2.60861  3.76993  3.56012  3.64942  3.73585  1.44457  3.27012  4.09869  3.08513  3.94200  2.78813  2.04016  2.68259  3.02187  3.40465  5.41530  4.13401     28 k - - -
          2.68618  4.42225  2.77519  2.73123  3.46354  2.40513  3.72494  3.29354  2.67741  2.69355  4.24690  2.90347  2.73739  3.18146  2.89801  2.37887  2.77519  2.98518  4.58477  3.61503
          0.02344  4.16084  4.88319  0.61958  0.77255  0.51359  0.91216
     11   2.44657  4.12673  4.05094  3.46687  3.17393  3.73867  4.03536  2.24657  3.34588  1.59062  3.01120  3.69835  3.45338  3.57660  3.54158  3.02454  2.87305  2.32918  3.63697  3.53455     29 l - - -
          2.68618  4.42225  2.77519  2.73123  3.46354  2.40513  3.72494  3.29354  2.67741  2.69355  4.24690  2.90347  2.73739  3.18146  2.89801  2.37887  2.77519  2.98518  4.58477  3.61503
          0.02344  4.16084  4.88319  0.61958  0.77255  0.51359  0.91216
     12   2.23911  4.38303  3.37056  2.81537  3.57093  3.31298  3.80516  2.94150  2.77326  2.41841  3.52214  3.06400  3.91529  3.09340  3.14967  2.22053  2.55610  2.69668  3.38399  3.74141     30 s - - -
          2.68618  4.42225  2.77519  2.73123  3.46354  2.40513  3.72494  3.29354  2.67741  2.69355  4.24690  2.90347  2.73739  3.18146  2.89801  2.37887  2.77519  2.98518  4.58477  3.61503
          0.02344  4.16084  4.88319  0.61958  0.77255  0.51359  0.91216
     13   2.23328  4.18167  3.72684  3.15405  2.60124  3.29070  3.91290  2.63321  2.87444  2.01062  2.96228  3.48039  4.00725  3.34810  3.35508  2.89697  2.70679  2.42918  4.78444  3.57326     31 l - - -
          2.68618  4.42225  2.77519  2.73123  3.46354  2.40513  3.72494  3.29354  2.67741  2.69355  4.24690  2.90347  2.73739  3.18146  2.89801  2.37887  2.77519  2.98518  4.58477  3.61503
          0.02344  4.16084  4.88319  0.61958  0.77255  0.51359  0.91216
     14   2.52545  4.11740  3.96516  3.38585  3.01000  3.33958  4.00659  2.31031  3.28372  1.94756  3.13470  3.63916  4.07192  3.52459  3.50359  2.61617  2.84602  1.87306  4.74805  3.54965     32 v - - -
          2.68618  4.42225  2.77519  2.73123  3.46354  2.40513  3.72494  3.29354  2.67741  2.69355  4.24690  2.90347  2.73739  3.18146  2.89801  2.37887  2.77519  2.98518  4.58477  3.61503
          0.02344  4.16084  4.88319  0.61958  0.77255  0.51359  0.91216
     15   1.95133  4.32481  3.48352  2.94141  3.59630  2.54932  3.89599  2.74994  2.89417  2.25025  3.54897  3.31760  3.92327  3.20549  3.25002  2.54849  2.82587  2.52386  5.03351  3.79644     33 a - - -
          2.68618  4.42225  2.77519  2.73123  3.46354  2.40513  3.72494  3.29354  2.67741  2.69355  4.24690  2.90347  2.73739  3.18146  2.89801  2.37887  2.77519  2.98518  4.58477  3.61503
          0.03649  4.16084  3.90005  0.61958  0.77255  0.51359  0.91216
     16   2.64018  4.35399  4.45276  3.89527  3.37354  4.13677  4.53178  1.84366  3.78005  1.31749  3.23690  4.13565  4.46835  4.01062  3.97829  3.45881  2.98824  1.80706  5.14956  3.98576     34 l - - -
```

	2.68618	4.42225	2.77519	2.73123	3.46354	2.40513	3.72494	3.29354	2.67741	2.69355	4.24690	2.90347	2.73739	3.18146	2.89801	2.37887	2.77519	2.98518	4.58477	3.61503	
	0.02374	4.14809	4.87044	0.61958	0.77255	0.50371	0.92706														
17	2.11198	4.26621	3.94499	3.39724	3.40163	3.72906	4.15106	2.30500	3.29327	1.52568	3.34364	3.68517	4.14785	3.57806	3.55613	2.86130	2.96057	2.17606	4.98796	3.78388	35 l - - -
	2.68618	4.42225	2.77519	2.73123	3.46354	2.40513	3.72494	3.29354	2.67741	2.69355	4.24690	2.90347	2.73739	3.18146	2.89801	2.37887	2.77519	2.98518	4.58477	3.61503	
	0.02344	4.16084	4.88319	0.61958	0.77255	0.51359	0.91216														
18	2.12861	3.39037	3.97900	3.39573	3.07904	3.45163	3.97826	2.45458	3.28685	1.98427	3.10671	3.63094	4.04777	3.51887	3.49097	2.79538	2.81854	2.11296	4.69715	3.26168	36 l - - -
	2.68618	4.42225	2.77519	2.73123	3.46354	2.40513	3.72494	3.29354	2.67741	2.69355	4.24690	2.90347	2.73739	3.18146	2.89801	2.37887	2.77519	2.98518	4.58477	3.61503	
	0.02344	4.16084	4.88319	0.61958	0.77255	0.46875	0.98292														
19	2.05940	4.36998	3.44051	2.91069	3.76334	2.20691	3.92052	2.84512	2.87332	2.72019	3.69557	3.28383	3.88995	3.19158	3.24769	2.64621	2.20486	2.65335	5.17118	3.92505	37 a - - -
	2.68618	4.42225	2.77519	2.73123	3.46354	2.40513	3.72494	3.29354	2.67741	2.69355	4.24690	2.90347	2.73739	3.18146	2.89801	2.37887	2.77519	2.98518	4.58477	3.61503	
	0.02290	4.18373	4.90608	0.61958	0.77255	0.48576	0.95510														
20	2.55925	4.14545	4.01485	3.43619	3.02031	3.75201	4.05854	2.33474	3.33375	1.99705	3.00264	3.22107	4.11810	3.57484	3.55298	3.03563	2.70486	1.68401	4.79262	3.59495	38 v - - -
	2.68618	4.42225	2.77519	2.73123	3.46354	2.40513	3.72494	3.29354	2.67741	2.69355	4.24690	2.90347	2.73739	3.18146	2.89801	2.37887	2.77519	2.98518	4.58477	3.61503	
	0.02290	4.18373	4.90608	0.61958	0.77255	0.48576	0.95510														
21	1.96761	4.24541	4.00880	3.44161	3.39696	3.82976	4.16962	1.93874	3.35648	2.15664	3.14451	3.73790	4.20108	3.62099	3.61674	3.11767	2.48806	1.98235	4.97143	3.76790	39 i - - -
	2.68618	4.42225	2.77519	2.73123	3.46354	2.40513	3.72494	3.29354	2.67741	2.69355	4.24690	2.90347	2.73739	3.18146	2.89801	2.37887	2.77519	2.98518	4.58477	3.61503	
	0.02290	4.18373	4.90608	0.61958	0.77255	0.48576	0.95510														
22	1.79596	4.24380	3.77676	3.21023	3.38292	3.67789	4.00422	2.43571	3.13230	2.06727	2.95583	3.54197	4.06809	3.41437	3.42439	2.77508	2.84285	2.25673	4.90075	3.68956	40 a - - -
	2.68618	4.42225	2.77519	2.73123	3.46354	2.40513	3.72494	3.29354	2.67741	2.69355	4.24690	2.90347	2.73739	3.18146	2.89801	2.37887	2.77519	2.98518	4.58477	3.61503	
	0.07307	4.18373	2.89630	0.61958	0.77255	0.48576	0.95510														
23	1.90326	4.33615	3.41761	2.94463	4.01993	2.97246	4.00101	3.39104	2.92619	2.83538	3.93177	3.26224	3.80655	3.24466	3.29968	1.86576	1.97798	3.01008	5.38190	4.13549	41 s - - -
	2.68618	4.42225	2.77519	2.73123	3.46354	2.40513	3.72494	3.29354	2.67741	2.69355	4.24690	2.90347	2.73739	3.18146	2.89801	2.37887	2.77519	2.98518	4.58477	3.61503	
	0.02407	4.13473	4.85707	0.61958	0.77255	0.54367	0.86896														
24	2.48924	3.71484	4.16068	3.56875	3.12531	3.70315	4.01622	2.13859	3.42941	2.09109	2.49708	3.72964	3.74059	3.63088	3.57186	2.81394	2.81316	2.00077	4.06510	3.45866	42 v - - -
	2.68618	4.42225	2.77519	2.73123	3.46354	2.40513	3.72494	3.29354	2.67741	2.69355	4.24690	2.90347	2.73739	3.18146	2.89801	2.37887	2.77519	2.98518	4.58477	3.61503	
	0.02407	4.13473	4.85707	0.61958	0.77255	0.45118	1.01301														
25	2.38315	4.11866	3.89976	3.31996	2.26692	3.17130	3.95806	2.47106	3.22255	2.13879	3.13705	3.33167	4.04126	3.46775	3.45325	2.94584	2.82729	2.22594	4.71841	3.21707	43 l - - -
	2.68618	4.42225	2.77519	2.73123	3.46354	2.40513	3.72494	3.29354	2.67741	2.69355	4.24690	2.90347	2.73739	3.18146	2.89801	2.37887	2.77519	2.98518	4.58477	3.61503	
	0.02290	4.18373	4.90608	0.61958	0.77255	0.48576	0.95510														
26	2.05921	4.42108	3.33345	2.79833	3.63396	3.31387	3.81630	3.02069	2.76771	2.56508	3.59186	3.22416	3.58786	3.09130	3.15164	2.28368	2.83734	2.76374	5.04507	2.55934	44 a - - -
	2.68618	4.42225	2.77519	2.73123	3.46354	2.40513	3.72494	3.29354	2.67741	2.69355	4.24690	2.90347	2.73739	3.18146	2.89801	2.37887	2.77519	2.98518	4.58477	3.61503	
	0.02290	4.18373	4.90608	0.61958	0.77255	0.48576	0.95510														
27	2.18103	4.27627	3.57888	3.01772	3.45827	3.56400	3.90059	2.37190	2.96241	2.39569	3.42609	3.38695	3.60579	3.25781	3.29511	2.37675	2.48870	2.27149	4.92033	3.69443	45 a - - -
	2.68618	4.42225	2.77519	2.73123	3.46354	2.40513	3.72494	3.29354	2.67741	2.69355	4.24690	2.90347	2.73739	3.18146	2.89801	2.37887	2.77519	2.98518	4.58477	3.61503	
	0.02290	4.18373	4.90608	0.61958	0.77255	0.48576	0.95510														
28	2.12626	4.43537	3.30881	2.84898	4.14065	2.49875	3.96921	3.54443	2.85769	2.98361	4.02812	3.21100	1.91973	3.18099	3.25743	2.32737	2.58337	3.13743	5.46569	4.19818	46 p - - -
	2.68623	4.42191	2.77524	2.73128	3.46333	2.40517	3.72499	3.29359	2.67745	2.69343	4.24694	2.90351	2.73744	3.18136	2.89805	2.37882	2.77524	2.98513	4.58482	3.61491	
	0.19574	1.76976	4.90608	0.55700	0.85079	0.48576	0.95510														
29	2.22601	4.54221	3.19348	2.67309	3.85052	3.41201	3.78269	3.24019	2.66217	2.90517	3.75700	3.01570	2.67626	2.99826	3.07910	2.57011	2.10112	2.94437	5.20565	3.30773	49 t - - -
	2.68618	4.42225	2.77519	2.73123	3.46354	2.40513	3.72494	3.29354	2.67741	2.69355	4.24690	2.90347	2.73739	3.18146	2.89801	2.37887	2.77519	2.98518	4.58477	3.61503	
	0.02290	4.18373	4.90608	0.61958	0.77255	0.48576	0.95510														
30	1.19029	4.23195	3.82972	3.42584	3.97414	3.22739	4.32326	2.98901	3.37155	2.97433	3.91739	3.55913	3.91061	3.66480	3.64846	2.45673	2.84978	1.98522	5.44990	4.24107	50 a - - -
	2.68618	4.42225	2.77519	2.73123	3.46354	2.40513	3.72494	3.29354	2.67741	2.69355	4.24690	2.90347	2.73739	3.18146	2.89801	2.37887	2.77519	2.98518	4.58477	3.61503	
	0.02290	4.18373	4.90608	0.61958	0.77255	0.48576	0.95510														
31	2.46240	4.80318	2.79889	2.41420	4.03863	3.44142	3.50854	3.45484	2.44848	3.06084	3.52154	2.97506	3.83331	2.45262	2.90827	2.03014	2.84430	3.13453	4.06741	3.97409	51 s - - -
	2.68618	4.42225	2.77519	2.73123	3.46354	2.40513	3.72494	3.29354	2.67741	2.69355	4.24690	2.90347	2.73739	3.18146	2.89801	2.37887	2.77519	2.98518	4.58477	3.61503	
	0.02290	4.18373	4.90608	0.61958	0.77255	0.48576	0.95510														
32	0.93746	4.26295	3.61442	3.38908	4.48471	2.02462	4.44923	3.85276	3.48183	3.59245	4.43502	3.48298	3.81644	3.74842	3.76227	2.52551	2.84640	3.29295	5.82705	4.64732	52 a - - -
	2.68618	4.42225	2.77519	2.73123	3.46354	2.40513	3.72494	3.29354	2.67741	2.69355	4.24690	2.90347	2.73739	3.18146	2.89801	2.37887	2.77519	2.98518	4.58477	3.61503	
	0.02290	4.18373	4.90608	0.61958	0.77255	0.48576	0.95510														
33	2.76405	4.51717	3.54061	2.99343	3.34137	3.68540	1.75130	2.58471	2.81696	2.48021	2.91635	3.39601	4.07120	3.21824	3.13753	2.95919	2.99977	2.70752	4.87819	3.50999	53 h - - -
	2.68618	4.42225	2.77519	2.73123	3.46354	2.40513	3.72494	3.29354	2.67741	2.69355	4.24690	2.90347	2.73739	3.18146	2.89801	2.37887	2.77519	2.98518	4.58477	3.61503	
	0.02290	4.18373	4.90608	0.61958	0.77255	0.48576	0.95510														
34	3.09995	4.83262	3.83645	3.78085	4.89495	0.37791	4.87055	4.58175	4.00380	4.19229	5.17870	4.00221	4.23262	4.30392	4.19473	3.28255	3.60515	4.04968	5.89622	5.01130	54 G - - -
	2.68618	4.42225	2.77519	2.73123	3.46354	2.40513	3.72494	3.29354	2.67741	2.69355	4.24690	2.90347	2.73739	3.18146	2.89801	2.37887	2.77519	2.98518	4.58477	3.61503	
	0.02290	4.18373	4.90608	0.61958	0.77255	0.48576	0.95510														
35	2.39368	5.14631	2.54245	1.09153	4.58777	3.35061	3.88331	3.94580	2.75996	3.58495	4.46470	2.93913	3.93944	3.05563	3.22589	2.85954	3.18347	3.57883	5.81097	4.42368	55 e - - -
	2.68618	4.42225	2.77519	2.73123	3.46354	2.40513	3.72494	3.29354	2.67741	2.69355	4.24690	2.90347	2.73739	3.18146	2.89801	2.37887	2.77519	2.98518	4.58477	3.61503	
	0.02290	4.18373	4.90608	0.61958	0.77255	0.48576	0.95510														
36	3.19846	5.30490	3.62062	2.92800	4.79854	3.78564	3.70028	4.13505	1.00238	3.54696	4.42289	3.31553	4.14880	2.83253	1.77666	3.18594	3.35380	3.81492	5.55867	4.41303	56 k - - -
	2.68618	4.42225	2.77519	2.73123	3.46354	2.40513	3.72494	3.29354	2.67741	2.69355	4.24690	2.90347	2.73739	3.18146	2.89801	2.37887	2.77519	2.98518	4.58477	3.61503	
	0.02290	4.18373	4.90608	0.61958	0.77255	0.48576	0.95510														
37	1.81727	4.37890	3.29855	3.00301	4.39940	3.09957	4.17311	3.82722	3.10296	3.49147	4.31108	2.85497	3.80645	3.40545	3.46726	1.19988	2.84364	3.30754	5.71438	4.45915	57 s - - -
	2.68618	4.42225	2.77519	2.73123	3.46354	2.40513	3.72494	3.29354	2.67741	2.69355	4.24690	2.90347	2.73739	3.18146	2.89801	2.37887	2.77519	2.98518	4.58477	3.61503	
	0.02290	4.18373	4.90608	0.61958	0.77255	0.48576	0.95510														
38	2.92269	4.91181	3.24767	2.81480	3.84717	3.63020	3.85719	3.34796	2.50723	2.32271	3.90895	3.26831	4.07644	1.33851	2.80496	2.99931	3.16996	3.17077	5.27684	3.92793	58 q - - -
	2.68618	4.42225	2.77519	2.73123	3.46354	2.40513	3.72494	3.29354	2.67741	2.69355	4.24690	2.90347	2.73739	3.18146	2.89801	2.37887	2.77519	2.98518	4.58477	3.61503	

```
          0.02290  4.18373  4.90608  0.61958  0.77255  0.48576  0.95510
     39   1.91872  5.09386  2.66560  1.66715  4.42473  3.38581  3.72755  3.85845  2.52482  3.41746  4.23383  2.90619  3.87762  2.48592  2.98412  2.74930  3.02499  3.48805  5.61497  4.23820     59 e - - -
          2.68618  4.42225  2.77519  2.73123  3.46354  2.40513  3.72494  3.29354  2.67741  2.69355  4.24690  2.90347  2.73739  3.18146  2.89801  2.37887  2.77519  2.98518  4.58477  3.61503
          0.02290  4.18373  4.90608  0.61958  0.77255  0.48576  0.95510
     40   1.02776  4.31716  3.54140  3.25129  4.34348  3.10831  4.32023  3.67317  3.29026  3.41197  4.28775  3.43276  2.25248  3.59786  3.59881  2.57053  2.86533  3.20190  5.70261  4.49320     60 a - - -
          2.68618  4.42225  2.77519  2.73123  3.46354  2.40513  3.72494  3.29354  2.67741  2.69355  4.24690  2.90347  2.73739  3.18146  2.89801  2.37887  2.77519  2.98518  4.58477  3.61503
          0.02290  4.18373  4.90608  0.61958  0.77255  0.48576  0.95510
     41   3.33551  4.67235  4.64848  4.19642  1.05507  4.32568  3.86352  2.40250  4.04368  2.20242  3.47554  4.16768  4.62928  4.11717  4.13116  3.66221  3.56223  2.76711  4.07979  2.23375     61 f - - -
          2.68618  4.42225  2.77519  2.73123  3.46354  2.40513  3.72494  3.29354  2.67741  2.69355  4.24690  2.90347  2.73739  3.18146  2.89801  2.37887  2.77519  2.98518  4.58477  3.61503
          0.02290  4.18373  4.90608  0.61958  0.77255  0.48576  0.95510
     42   3.36599  4.67392  5.03040  4.45588  3.15356  4.64852  4.96141  2.00648  4.32694  0.96330  2.09331  4.69162  4.81673  4.39359  4.41772  3.99384  3.58408  2.13718  5.23803  4.20224     62 l - - -
          2.68618  4.42225  2.77519  2.73123  3.46354  2.40513  3.72494  3.29354  2.67741  2.69355  4.24690  2.90347  2.73739  3.18146  2.89801  2.37887  2.77519  2.98518  4.58477  3.61503
          0.02290  4.18373  4.90608  0.61958  0.77255  0.48576  0.95510
     43   3.39326  5.18728  3.91904  3.37864  4.62317  3.80584  4.10075  4.19699  2.43055  3.65635  4.65808  3.70929  4.30210  3.31412  0.59204  3.46858  3.64356  3.90975  5.58474  4.49376     63 R - - -
          2.68618  4.42225  2.77519  2.73123  3.46354  2.40513  3.72494  3.29354  2.67741  2.69355  4.24690  2.90347  2.73739  3.18146  2.89801  2.37887  2.77519  2.98518  4.58477  3.61503
          0.02290  4.18373  4.90608  0.61958  0.77255  0.48576  0.95510
     44   3.24594  4.75110  4.40202  4.03065  3.38670  4.07751  4.69389  2.59557  3.77885  1.94357  0.88517  4.26617  4.54947  4.14531  3.94516  3.62226  3.56252  2.63588  5.26213  4.04679     64 m - - -
          2.68618  4.42225  2.77519  2.73123  3.46354  2.40513  3.72494  3.29354  2.67741  2.69355  4.24690  2.90347  2.73739  3.18146  2.89801  2.37887  2.77519  2.98518  4.58477  3.61503
          0.02290  4.18373  4.90608  0.61958  0.77255  0.48576  0.95510
     45   3.39326  5.18728  3.91904  3.37864  4.62317  3.80584  4.10075  4.19699  2.43055  3.65635  4.65808  3.70929  4.30210  3.31412  0.59204  3.46858  3.64356  3.90975  5.58474  4.49376     65 R - - -
          2.68618  4.42225  2.77519  2.73123  3.46354  2.40513  3.72494  3.29354  2.67741  2.69355  4.24690  2.90347  2.73739  3.18146  2.89801  2.37887  2.77519  2.98518  4.58477  3.61503
          0.02290  4.18373  4.90608  0.61958  0.77255  0.48576  0.95510
     46   2.61430  4.45611  3.74249  3.52400  4.28167  3.28148  4.51265  3.44901  3.47333  3.30093  4.34016  3.67203  4.01397  3.85451  3.70858  2.82024  0.73597  3.10825  5.69020  4.50189     66 t - - -
          2.68618  4.42225  2.77519  2.73123  3.46354  2.40513  3.72494  3.29354  2.67741  2.69355  4.24690  2.90347  2.73739  3.18146  2.89801  2.37887  2.77519  2.98518  4.58477  3.61503
          0.02290  4.18373  4.90608  0.61958  0.77255  0.48576  0.95510
     47   2.66152  4.40294  4.78361  4.24168  3.52932  4.39617  4.85299  1.29081  4.13286  1.75854  3.36056  4.44520  4.69492  4.35377  4.30582  3.74335  3.32295  1.50875  5.38211  4.20723     67 i - - -
          2.68618  4.42225  2.77519  2.73123  3.46354  2.40513  3.72494  3.29354  2.67741  2.69355  4.24690  2.90347  2.73739  3.18146  2.89801  2.37887  2.77519  2.98518  4.58477  3.61503
          0.02290  4.18373  4.90608  0.61958  0.77255  0.48576  0.95510
     48   2.65498  4.92419  2.94291  2.42868  4.19440  3.43395  2.57885  3.63154  2.39346  3.20252  4.00508  2.46800  3.84080  2.30456  2.83610  2.66588  2.58671  2.98752  5.40602  4.05709     68 q - - -
          2.68618  4.42225  2.77519  2.73123  3.46354  2.40513  3.72494  3.29354  2.67741  2.69355  4.24690  2.90347  2.73739  3.18146  2.89801  2.37887  2.77519  2.98518  4.58477  3.61503
          0.02290  4.18373  4.90608  0.61958  0.77255  0.48576  0.95510
     49   3.73233  4.95610  4.82855  4.51314  1.42845  4.49008  3.63608  3.36252  4.31370  2.65293  3.93975  4.23723  4.80515  4.28448  4.31858  3.89004  3.95844  3.31696  1.26956  2.05270     69 w - - -
          2.68618  4.42225  2.77519  2.73123  3.46354  2.40513  3.72494  3.29354  2.67741  2.69355  4.24690  2.90347  2.73739  3.18146  2.89801  2.37887  2.77519  2.98518  4.58477  3.61503
          0.02290  4.18373  4.90608  0.61958  0.77255  0.48576  0.95510
     50   3.89452  5.07044  4.82375  4.56277  1.52103  4.57647  3.54805  3.56224  4.39723  2.85169  4.14649  4.22454  4.87763  4.30990  4.39001  3.96045  4.11368  3.49431  3.66797  0.81260     70 y - - -
          2.68618  4.42225  2.77519  2.73123  3.46354  2.40513  3.72494  3.29354  2.67741  2.69355  4.24690  2.90347  2.73739  3.18146  2.89801  2.37887  2.77519  2.98518  4.58477  3.61503
          0.02290  4.18373  4.90608  0.61958  0.77255  0.48576  0.95510
     51   3.35817  5.49836  0.55745  2.58672  4.92516  3.47760  4.24948  4.59158  3.39895  4.14777  5.14393  3.15471  4.15297  3.49681  3.94916  3.29282  3.70707  4.20089  6.02892  4.76100     71 D - - -
          2.68618  4.42225  2.77519  2.73123  3.46354  2.40513  3.72494  3.29354  2.67741  2.69355  4.24690  2.90347  2.73739  3.18146  2.89801  2.37887  2.77519  2.98518  4.58477  3.61503
          0.02290  4.18373  4.90608  0.61958  0.77255  0.48576  0.95510
     52   2.88673  4.32920  4.33120  3.77156  3.40872  4.05106  4.43711  2.11997  3.65636  1.70952  3.07666  4.02659  4.40164  3.90907  3.87470  3.36514  2.40507  1.43369  5.11931  3.93654     72 v - - -
          2.68618  4.42225  2.77519  2.73123  3.46354  2.40513  3.72494  3.29354  2.67741  2.69355  4.24690  2.90347  2.73739  3.18146  2.89801  2.37887  2.77519  2.98518  4.58477  3.61503
          0.02290  4.18373  4.90608  0.61958  0.77255  0.48576  0.95510
     53   2.61812  5.04944  2.88284  2.38772  4.36530  3.43217  3.60975  3.81266  2.10779  3.33165  4.10659  2.39561  3.82839  2.18553  2.46942  2.61442  2.89298  3.07566  5.49148  4.12659     73 k - - -
          2.68618  4.42225  2.77519  2.73123  3.46354  2.40513  3.72494  3.29354  2.67741  2.69355  4.24690  2.90347  2.73739  3.18146  2.89801  2.37887  2.77519  2.98518  4.58477  3.61503
          0.02290  4.18373  4.90608  0.61958  0.77255  0.48576  0.95510
     54   3.70996  4.95028  4.79420  4.46976  1.58900  4.46675  3.65133  3.34769  4.26916  2.64097  3.92826  4.22659  4.78926  4.26555  4.28957  3.87234  3.93919  3.30293  1.15823  2.07746     74 w - - -
          2.68618  4.42225  2.77519  2.73123  3.46354  2.40513  3.72494  3.29354  2.67741  2.69355  4.24690  2.90347  2.73739  3.18146  2.89801  2.37887  2.77519  2.98518  4.58477  3.61503
          0.02290  4.18373  4.90608  0.61958  0.77255  0.48576  0.95510
     55   2.49856  4.41691  3.51172  3.35443  4.40621  3.14273  4.44894  3.97753  3.45020  3.67726  4.58399  3.51907  3.90964  3.77613  3.71759  0.70296  3.00416  3.44173  5.75515  4.49515     75 s - - -
          2.68609  4.42229  2.77523  2.73127  3.46318  2.40516  3.72498  3.29358  2.67744  2.69358  4.24693  2.90350  2.73724  3.18150  2.89804  2.37890  2.77517  2.98522  4.58480  3.61507
          0.09465  2.48998  4.90608  0.86042  0.54988  0.48576  0.95510
     56   2.69840  4.86669  3.06182  2.59433  4.34953  3.18701  3.74682  3.75040  1.45735  3.32420  4.14495  3.06688  3.89534  2.89685  2.73853  2.59549  2.27197  3.37831  5.50773  4.21049     79 k - - -
          2.68618  4.42225  2.77519  2.73123  3.46354  2.40513  3.72494  3.29354  2.67741  2.69355  4.24690  2.90347  2.73739  3.18146  2.89801  2.37887  2.77519  2.98518  4.58477  3.61503
          0.02290  4.18373  4.90608  0.61958  0.77255  0.48576  0.95510
     57   2.50409  5.17732  2.31485  2.06320  4.49174  3.37361  3.64765  3.96785  2.44318  3.47142  4.23980  2.34577  3.82431  2.42416  2.94777  2.66283  2.42362  3.55390  5.62748  4.21297     80 e - - -
          2.68618  4.42225  2.77519  2.73123  3.46354  2.40513  3.72494  3.29354  2.67741  2.69355  4.24690  2.90347  2.73739  3.18146  2.89801  2.37887  2.77519  2.98518  4.58477  3.61503
          0.02290  4.18373  4.90608  0.61958  0.77255  0.48576  0.95510
     58   2.64794  5.04036  2.94160  2.22827  4.35477  3.42788  3.19142  3.70447  2.02410  3.32292  3.97794  2.79059  3.81813  2.63495  2.36333  2.51948  2.50993  3.41532  5.48277  4.11405     81 k - - -
          2.68618  4.42225  2.77519  2.73123  3.46354  2.40513  3.72494  3.29354  2.67741  2.69355  4.24690  2.90347  2.73739  3.18146  2.89801  2.37887  2.77519  2.98518  4.58477  3.61503
          0.02290  4.18373  4.90608  0.61958  0.77255  0.48576  0.95510
     59   3.12283  4.45137  4.82630  4.26974  2.58971  4.40316  4.74048  1.95884  4.14887  1.60761  3.17068  4.45057  4.67112  4.30234  4.27624  3.74156  3.35849  1.21931  5.16057  3.96328     82 v - - -
          2.68618  4.42225  2.77519  2.73123  3.46354  2.40513  3.72494  3.29354  2.67741  2.69355  4.24690  2.90347  2.73739  3.18146  2.89801  2.37887  2.77519  2.98518  4.58477  3.61503
          0.02290  4.18373  4.90608  0.61958  0.77255  0.48576  0.95510
     60   2.31735  5.05213  2.85866  2.38225  4.38082  3.41538  3.63834  3.82855  1.91889  3.35482  4.13680  2.25299  3.46137  2.55616  2.80491  2.60288  2.91934  3.44232  5.52178  4.15392     83 k - - -
          2.68618  4.42225  2.77519  2.73123  3.46354  2.40513  3.72494  3.29354  2.67741  2.69355  4.24690  2.90347  2.73739  3.18146  2.89801  2.37887  2.77519  2.98518  4.58477  3.61503
          0.02290  4.18373  4.90608  0.61958  0.77255  0.48576  0.95510
```

61	2.83640	4.34742	4.23629	3.73749	3.58513	3.91424	4.49174	1.99842	3.62090	2.34904	3.48794	3.97729	3.38068	3.92142	3.86230	3.27393	3.13019	1.06320	5.27542	4.05697	84 v - - -
	2.68618	4.42225	2.77519	2.73123	3.46354	2.40513	3.72494	3.29354	2.67741	2.69355	4.24690	2.90347	2.73739	3.18146	2.89801	2.37887	2.77519	2.98518	4.58477	3.61503	
	0.02290	4.18373	4.90608	0.61958	0.77255	0.48576	0.95510														
62	2.70424	4.81521	2.75475	2.62472	4.59277	1.94189	4.05343	4.13537	2.98121	3.72596	4.56883	1.28286	3.90955	3.25980	3.41313	2.76526	3.10459	3.64112	5.83193	4.49224	85 n - - -
	2.68618	4.42225	2.77519	2.73123	3.46354	2.40513	3.72494	3.29354	2.67741	2.69355	4.24690	2.90347	2.73739	3.18146	2.89801	2.37887	2.77519	2.98518	4.58477	3.61503	
	0.02290	4.18373	4.90608	0.61958	0.77255	0.48576	0.95510														
63	3.07947	5.69793	1.45354	1.24604	4.98270	3.31329	3.87685	4.52026	2.91938	4.00205	4.85991	2.77035	3.94767	3.04450	3.53580	2.94763	3.37206	4.07791	6.14440	4.61890	86 e - - -
	2.68618	4.42225	2.77519	2.73123	3.46354	2.40513	3.72494	3.29354	2.67741	2.69355	4.24690	2.90347	2.73739	3.18146	2.89801	2.37887	2.77519	2.98518	4.58477	3.61503	
	0.02290	4.18373	4.90608	0.61958	0.77255	0.48576	0.95510														
64	2.79268	5.21622	2.25127	1.57957	4.54019	3.35228	3.73201	4.00145	2.59511	3.53795	4.33937	2.84411	3.86609	2.87216	3.11245	2.66121	2.09417	3.60348	5.71928	4.29921	87 e - - -
	2.68618	4.42225	2.77519	2.73123	3.46354	2.40513	3.72494	3.29354	2.67741	2.69355	4.24690	2.90347	2.73739	3.18146	2.89801	2.37887	2.77519	2.98518	4.58477	3.61503	
	0.02290	4.18373	4.90608	0.61958	0.77255	0.48576	0.95510														
65	2.83862	4.24865	4.43637	3.85139	2.62626	3.98735	4.25245	2.38881	3.71199	1.76171	1.95355	4.01275	4.31120	3.87580	3.83658	3.29108	3.06694	1.92851	4.76602	3.03980	88 l - - -
	2.68618	4.42225	2.77519	2.73123	3.46354	2.40513	3.72494	3.29354	2.67741	2.69355	4.24690	2.90347	2.73739	3.18146	2.89801	2.37887	2.77519	2.98518	4.58477	3.61503	
	0.02290	4.18373	4.90608	0.61958	0.77255	0.48576	0.95510														
66	2.61412	4.73235	2.79893	2.22122	3.96412	3.44919	3.68136	3.13252	2.50937	2.99296	3.82494	3.01158	3.85038	2.85195	2.96221	2.35660	2.34934	2.51013	5.26701	3.94936	89 e - - -
	2.68618	4.42225	2.77519	2.73123	3.46354	2.40513	3.72494	3.29354	2.67741	2.69355	4.24690	2.90347	2.73739	3.18146	2.89801	2.37887	2.77519	2.98518	4.58477	3.61503	
	0.02290	4.18373	4.90608	0.61958	0.77255	0.48576	0.95510														
67	3.00433	3.15952	4.82989	4.27595	3.47466	4.32016	4.76101	1.43067	4.14898	1.78766	3.34168	4.41483	4.62849	4.34243	4.27333	3.66375	3.24933	1.42628	5.25683	4.07725	90 v - - -
	2.68618	4.42225	2.77519	2.73123	3.46354	2.40513	3.72494	3.29354	2.67741	2.69355	4.24690	2.90347	2.73739	3.18146	2.89801	2.37887	2.77519	2.98518	4.58477	3.61503	
	0.02290	4.18373	4.90608	0.61958	0.77255	0.48576	0.95510														
68	2.42769	4.39074	3.35448	2.99856	4.24112	3.16497	4.10927	3.61430	2.90677	3.31172	4.15765	3.29435	3.83344	3.34754	3.34694	1.59943	1.52276	3.17800	5.57681	4.32713	91 t - - -
	2.68618	4.42225	2.77519	2.73123	3.46354	2.40513	3.72494	3.29354	2.67741	2.69355	4.24690	2.90347	2.73739	3.18146	2.89801	2.37887	2.77519	2.98518	4.58477	3.61503	
	0.02290	4.18373	4.90608	0.61958	0.77255	0.48576	0.95510														
69	3.09995	4.83262	3.83645	3.78085	4.89495	0.37791	4.87055	4.58175	4.00380	4.19229	5.17870	4.00221	4.23262	4.30392	4.19473	3.28255	3.60515	4.04968	5.89622	5.01130	92 G - - -
	2.68618	4.42225	2.77519	2.73123	3.46354	2.40513	3.72494	3.29354	2.67741	2.69355	4.24690	2.90347	2.73739	3.18146	2.89801	2.37887	2.77519	2.98518	4.58477	3.61503	
	0.02290	4.18373	4.90608	0.61958	0.77255	0.48576	0.95510														
70	3.28850	5.23796	3.48347	3.06091	4.69708	3.73021	3.97489	4.14953	0.67848	3.65183	4.60744	3.46717	4.21751	3.15652	2.54565	3.31659	3.52376	3.84381	5.63101	4.49490	93 K - - -
	2.68618	4.42225	2.77519	2.73123	3.46354	2.40513	3.72494	3.29354	2.67741	2.69355	4.24690	2.90347	2.73739	3.18146	2.89801	2.37887	2.77519	2.98518	4.58477	3.61503	
	0.02290	4.18373	4.90608	0.61958	0.77255	0.48576	0.95510														
71	3.12642	4.48042	4.73889	4.19023	1.63193	4.32978	4.52022	2.15486	4.05812	1.69903	3.13781	4.34816	4.61086	4.19702	4.17967	3.66323	3.36085	1.64758	4.89569	3.56530	94 f - - -
	2.68618	4.42225	2.77519	2.73123	3.46354	2.40513	3.72494	3.29354	2.67741	2.69355	4.24690	2.90347	2.73739	3.18146	2.89801	2.37887	2.77519	2.98518	4.58477	3.61503	
	0.02290	4.18373	4.90608	0.61958	0.77255	0.48576	0.95510														
72	2.69882	4.50316	3.42935	2.86595	3.26058	3.61608	1.95675	2.98993	2.72009	2.54741	2.69916	3.28967	3.99641	3.10880	2.91925	2.86743	2.92845	2.77676	4.87955	3.52047	95 h - - -
	2.68618	4.42225	2.77519	2.73123	3.46354	2.40513	3.72494	3.29354	2.67741	2.69355	4.24690	2.90347	2.73739	3.18146	2.89801	2.37887	2.77519	2.98518	4.58477	3.61503	
	0.02290	4.18373	4.90608	0.61958	0.77255	0.48576	0.95510														
73	3.05676	4.39191	4.73751	4.19901	3.58776	4.37544	4.84611	1.60479	4.09168	1.96395	3.42321	4.41364	4.68639	4.33577	4.28311	3.72255	2.84896	1.11229	5.41736	4.22581	96 v - - -
	2.68618	4.42225	2.77519	2.73123	3.46354	2.40513	3.72494	3.29354	2.67741	2.69355	4.24690	2.90347	2.73739	3.18146	2.89801	2.37887	2.77519	2.98518	4.58477	3.61503	
	0.02290	4.18373	4.90608	0.61958	0.77255	0.48576	0.95510														
74	2.41926	4.31949	4.34078	3.76937	1.81840	3.99809	4.27045	2.40146	3.64474	1.75745	2.40792	3.98321	4.32953	3.83816	3.81425	3.30255	3.10977	2.34727	4.82732	3.59809	97 l - - -
	2.68618	4.42225	2.77519	2.73123	3.46354	2.40513	3.72494	3.29354	2.67741	2.69355	4.24690	2.90347	2.73739	3.18146	2.89801	2.37887	2.77519	2.98518	4.58477	3.61503	
	0.02290	4.18373	4.90608	0.61958	0.77255	0.48576	0.95510														
75	2.39053	5.08334	2.64250	1.91620	4.40268	3.37715	3.65842	3.86308	2.42709	3.39413	4.17454	2.73376	2.82634	2.78358	2.95285	2.19308	2.93027	3.46896	5.57189	4.17715	98 e - - -
	2.68618	4.42225	2.77519	2.73123	3.46354	2.40513	3.72494	3.29354	2.67741	2.69355	4.24690	2.90347	2.73739	3.18146	2.89801	2.37887	2.77519	2.98518	4.58477	3.61503	
	0.02290	4.18373	4.90608	0.61958	0.77255	0.48576	0.95510														
76	2.33643	4.89571	2.47909	2.26989	4.18086	2.58192	3.65254	3.17870	2.45039	3.19127	3.99148	2.93494	3.82428	2.79174	2.92593	2.54082	2.58383	3.25899	5.41212	4.05690	99 e - - -
	2.68618	4.42225	2.77519	2.73123	3.46354	2.40513	3.72494	3.29354	2.67741	2.69355	4.24690	2.90347	2.73739	3.18146	2.89801	2.37887	2.77519	2.98518	4.58477	3.61503	
	0.02290	4.18373	4.90608	0.61958	0.77255	0.48576	0.95510														
77	3.90581	5.15933	4.60044	4.39164	3.05721	4.10803	4.33949	3.95678	4.11523	3.29253	4.57374	4.48179	4.65362	4.48590	4.18455	4.10625	4.22457	3.85500	0.43840	3.04495	100 W - - -
	2.68618	4.42225	2.77519	2.73123	3.46354	2.40513	3.72494	3.29354	2.67741	2.69355	4.24690	2.90347	2.73739	3.18146	2.89801	2.37887	2.77519	2.98518	4.58477	3.61503	
	0.02290	4.18373	4.90608	0.61958	0.77255	0.48576	0.95510														
78	3.22031	4.91301	3.86274	3.75476	4.70463	3.58351	4.79631	4.34360	3.84144	3.93066	5.00116	4.02490	0.41006	4.22005	4.04145	3.39999	3.68818	3.95083	5.79302	4.83303	101 P - - -
	2.68618	4.42225	2.77519	2.73123	3.46354	2.40513	3.72494	3.29354	2.67741	2.69355	4.24690	2.90347	2.73739	3.18146	2.89801	2.37887	2.77519	2.98518	4.58477	3.61503	
	0.02290	4.18373	4.90608	0.61958	0.77255	0.48576	0.95510														
79	2.53839	4.92239	2.96419	1.95483	4.19308	3.43269	3.21030	3.13183	2.37475	3.19109	3.98588	2.94043	3.82521	2.37539	2.63861	2.63995	2.86133	3.06303	5.39775	4.04647	102 e - - -
	2.68618	4.42225	2.77519	2.73123	3.46354	2.40513	3.72494	3.29354	2.67741	2.69355	4.24690	2.90347	2.73739	3.18146	2.89801	2.37887	2.77519	2.98518	4.58477	3.61503	
	0.02290	4.18373	4.90608	0.61958	0.77255	0.48576	0.95510														
80	1.94724	4.72156	2.97237	2.10301	4.18051	3.33571	3.77864	3.58494	2.61058	3.20810	4.02795	3.02755	3.83851	2.94567	3.05882	2.25163	2.23558	3.22040	5.45242	4.12886	103 a - - -
	2.68618	4.42225	2.77519	2.73123	3.46354	2.40513	3.72494	3.29354	2.67741	2.69355	4.24690	2.90347	2.73739	3.18146	2.89801	2.37887	2.77519	2.98518	4.58477	3.61503	
	0.02290	4.18373	4.90608	0.61958	0.77255	0.48576	0.95510														
81	3.21744	4.49515	5.00903	4.49428	3.62332	4.63538	5.16479	1.54230	4.38754	1.91587	3.41686	4.70534	4.89814	4.62211	4.56545	4.01557	3.47081	0.96500	5.61313	4.42541	104 v - - -
	2.68618	4.42225	2.77519	2.73123	3.46354	2.40513	3.72494	3.29354	2.67741	2.69355	4.24690	2.90347	2.73739	3.18146	2.89801	2.37887	2.77519	2.98518	4.58477	3.61503	
	0.02290	4.18373	4.90608	0.61958	0.77255	0.48576	0.95510														
82	2.22394	5.12948	2.26935	2.23675	4.44442	2.97873	3.65660	3.91201	2.36404	3.43199	4.20814	2.62537	2.67647	2.77916	2.96178	2.66438	2.94241	3.50988	5.60153	4.19664	105 a - - -
	2.68618	4.42225	2.77519	2.73123	3.46354	2.40513	3.72494	3.29354	2.67741	2.69355	4.24690	2.90347	2.73739	3.18146	2.89801	2.37887	2.77519	2.98518	4.58477	3.61503	
	0.02290	4.18373	4.90608	0.61958	0.77255	0.48576	0.95510														
83	2.62053	4.80021	3.02694	2.35122	3.02034	3.45011	3.65044	3.44882	2.01373	3.01035	3.87373	2.67808	3.69493	2.80472	2.73627	2.65948	2.65710	2.98209	5.30247	3.97520	106 k - - -

```
        2.68618  4.42225  2.77519  2.73123  3.46354  2.40513  3.72494  3.29354  2.67741  2.69355  4.24690  2.90347  2.73739  3.18146  2.89801  2.37887  2.77519  2.98518  4.58477  3.61503
        0.02290  4.18373  4.90608  0.61958  0.77255  0.48576  0.95510
     84   2.39187  4.36083  3.34972  3.07754  4.39656  3.09717  4.22190  3.80979  3.14585  3.48880  4.32624  3.32583  1.28736  3.46950  3.48036  1.99726  2.62694  3.29612  5.72352  4.48013    107 p - - -
        2.68618  4.42225  2.77519  2.73123  3.46354  2.40513  3.72494  3.29354  2.67741  2.69355  4.24690  2.90347  2.73739  3.18146  2.89801  2.37887  2.77519  2.98518  4.58477  3.61503
        0.02290  4.18373  4.90608  0.61958  0.77255  0.48576  0.95510
     85   2.72858  5.22094  2.25432  1.74132  4.53896  2.61162  3.40369  4.02033  2.34855  3.51277  4.28044  2.83998  3.83153  2.77090  2.70828  2.67803  2.96990  3.59939  5.65983  4.24011    108 e - - -
        2.68618  4.42225  2.77519  2.73123  3.46354  2.40513  3.72494  3.29354  2.67741  2.69355  4.24690  2.90347  2.73739  3.18146  2.89801  2.37887  2.77519  2.98518  4.58477  3.61503
        0.02290  4.18373  4.90608  0.61958  0.77255  0.48576  0.95510
     86   2.30231  4.23094  3.70070  3.14224  3.40344  3.61070  3.95613  2.13670  3.06984  2.45523  3.37802  3.47778  4.01774  3.13613  3.36972  2.50222  2.68836  1.93720  4.88791  3.67205    109 v - - -
        2.68618  4.42225  2.77519  2.73123  3.46354  2.40513  3.72494  3.29354  2.67741  2.69355  4.24690  2.90347  2.73739  3.18146  2.89801  2.37887  2.77519  2.98518  4.58477  3.61503
        0.02290  4.18373  4.90608  0.61958  0.77255  0.48576  0.95510
     87   1.89717  3.63901  3.61700  3.10918  3.84132  2.97970  4.05928  2.76055  3.06140  2.91427  3.78562  3.37913  3.85891  3.36070  3.40096  1.55784  2.79627  2.85006  5.25522  4.03045    110 s - - -
        2.68618  4.42225  2.77519  2.73123  3.46354  2.40513  3.72494  3.29354  2.67741  2.69355  4.24690  2.90347  2.73739  3.18146  2.89801  2.37887  2.77519  2.98518  4.58477  3.61503
        0.02290  4.18373  4.90608  0.61958  0.77255  0.48576  0.95510
     88   3.77125  4.99539  4.77556  4.48757  0.77500  4.51708  3.62015  3.36330  4.32546  2.65602  3.96338  4.22501  4.82896  4.28368  4.33980  3.90660  3.99887  3.32414  3.75304  1.78557    111 f - - -
        2.68618  4.42225  2.77519  2.73123  3.46354  2.40513  3.72494  3.29354  2.67741  2.69355  4.24690  2.90347  2.73739  3.18146  2.89801  2.37887  2.77519  2.98518  4.58477  3.61503
        0.02290  4.18373  4.90608  0.61958  0.77255  0.48576  0.95510
     89   3.44926  4.74483  5.08491  4.52772  2.49292  4.71031  4.89441  2.03000  4.39489  0.80536  2.92118  4.73965  4.86113  4.42890  4.46658  4.06493  3.66477  2.40368  5.10390  3.92301    112 l - - -
        2.68618  4.42225  2.77519  2.73123  3.46354  2.40513  3.72494  3.29354  2.67741  2.69355  4.24690  2.90347  2.73739  3.18146  2.89801  2.37887  2.77519  2.98518  4.58477  3.61503
        0.02290  4.18373  4.90608  0.61958  0.77255  0.48576  0.95510
     90   2.93036  5.13227  2.67309  2.50030  4.09539  3.41866  3.05918  4.03011  2.67676  3.56050  4.44513  1.16381  3.98618  3.07143  3.06854  2.91929  3.22505  3.66077  5.44182  3.95838    113 n - - -
        2.68618  4.42225  2.77519  2.73123  3.46354  2.40513  3.72494  3.29354  2.67741  2.69355  4.24690  2.90347  2.73739  3.18146  2.89801  2.37887  2.77519  2.98518  4.58477  3.61503
        0.02290  4.18373  4.90608  0.61958  0.77255  0.48576  0.95510
     91   2.29678  4.09856  4.20064  3.61553  2.51781  3.78280  4.09728  1.88969  3.48559  2.20488  3.18464  3.79516  4.14577  3.69352  3.64321  3.07814  2.74051  2.00964  4.73065  3.10763    114 i - - -
        2.68618  4.42225  2.77519  2.73123  3.46354  2.40513  3.72494  3.29354  2.67741  2.69355  4.24690  2.90347  2.73739  3.18146  2.89801  2.37887  2.77519  2.98518  4.58477  3.61503
        0.02290  4.18373  4.90608  0.61958  0.77255  0.48576  0.95510
     92   2.49275  4.35794  3.43653  3.01668  2.52508  1.75920  4.00846  3.25461  3.03544  2.94892  3.83575  3.33449  3.89057  3.33337  3.38726  2.04741  2.85896  2.93269  5.16664  3.84199    115 g - - -
        2.68618  4.42225  2.77519  2.73123  3.46354  2.40513  3.72494  3.29354  2.67741  2.69355  4.24690  2.90347  2.73739  3.18146  2.89801  2.37887  2.77519  2.98518  4.58477  3.61503
        0.02290  4.18373  4.90608  0.61958  0.77255  0.48576  0.95510
     93   2.61833  4.42501  3.36205  2.35134  3.58072  3.56230  3.80519  2.35829  2.75713  2.62088  3.29293  3.24228  3.94472  2.81731  3.13982  2.80005  2.48765  2.27005  5.00724  3.75609    116 v - - -
        2.68618  4.42225  2.77519  2.73123  3.46354  2.40513  3.72494  3.29354  2.67741  2.69355  4.24690  2.90347  2.73739  3.18146  2.89801  2.37887  2.77519  2.98518  4.58477  3.61503
        0.02290  4.18373  4.90608  0.61958  0.77255  0.48576  0.95510
     94   3.22031  4.91301  3.86274  3.75476  4.70463  3.58351  4.79631  4.34360  3.84144  3.93066  5.00116  4.02490  0.41006  4.22005  4.04145  3.39999  3.68818  3.95083  5.79302  4.83303    117 P - - -
        2.68618  4.42225  2.77519  2.73123  3.46354  2.40513  3.72494  3.29354  2.67741  2.69355  4.24690  2.90347  2.73739  3.18146  2.89801  2.37887  2.77519  2.98518  4.58477  3.61503
        0.02290  4.18373  4.90608  0.61958  0.77255  0.48576  0.95510
     95   3.09995  4.83262  3.83645  3.78085  4.89495  0.37791  4.87055  4.58175  4.00380  4.19229  5.17870  4.00221  4.23262  4.30392  4.19473  3.28255  3.60515  4.04968  5.89622  5.01130    118 G - - -
        2.68618  4.42225  2.77519  2.73123  3.46354  2.40513  3.72494  3.29354  2.67741  2.69355  4.24690  2.90347  2.73739  3.18146  2.89801  2.37887  2.77519  2.98518  4.58477  3.61503
        0.02290  4.18373  4.90608  0.61958  0.77255  0.48576  0.95510
     96   2.32620  4.47705  3.43545  3.26315  4.42807  3.20487  4.40108  3.79544  3.34515  3.51085  4.46638  3.49384  0.88177  3.69736  3.62678  2.73761  3.04670  3.35287  5.76526  4.56086    119 p - - -
        2.68618  4.42225  2.77519  2.73123  3.46354  2.40513  3.72494  3.29354  2.67741  2.69355  4.24690  2.90347  2.73739  3.18146  2.89801  2.37887  2.77519  2.98518  4.58477  3.61503
        0.02290  4.18373  4.90608  0.61958  0.77255  0.48576  0.95510
     97   2.63365  4.42219  3.41750  2.85359  3.58965  3.56468  3.81359  2.92247  2.71245  2.63893  3.28699  3.27383  3.95728  2.72521  2.76309  2.60422  2.86975  1.75690  5.00491  3.76268    120 v - - -
        2.68618  4.42225  2.77519  2.73123  3.46354  2.40513  3.72494  3.29354  2.67741  2.69355  4.24690  2.90347  2.73739  3.18146  2.89801  2.37887  2.77519  2.98518  4.58477  3.61503
        0.02290  4.18373  4.90608  0.61958  0.77255  0.48576  0.95510
     98   2.68541  4.26374  4.44183  3.85776  2.28604  4.01377  4.32986  2.31450  3.72582  1.43652  2.74173  4.04008  4.33697  3.89978  3.86347  3.31942  2.90882  2.02258  4.87660  3.71896    121 l - - -
        2.68618  4.42225  2.77519  2.73123  3.46354  2.40513  3.72494  3.29354  2.67741  2.69355  4.24690  2.90347  2.73739  3.18146  2.89801  2.37887  2.77519  2.98518  4.58477  3.61503
        0.02290  4.18373  4.90608  0.61958  0.77255  0.48576  0.95510
     99   2.68149  4.39929  4.79972  4.25830  3.54004  4.40572  4.86568  1.34384  4.15049  1.76908  3.37113  4.45796  4.70307  4.37096  4.32142  3.75382  3.32399  1.42229  5.39133  4.21439    122 i - - -
        2.68618  4.42225  2.77519  2.73123  3.46354  2.40513  3.72494  3.29354  2.67741  2.69355  4.24690  2.90347  2.73739  3.18146  2.89801  2.37887  2.77519  2.98518  4.58477  3.61503
        0.02290  4.18373  4.90608  0.61958  0.77255  0.48576  0.95510
    100   3.23628  5.26451  3.69869  3.01264  4.74573  3.80246  3.73806  4.09622  1.79105  3.52879  4.42709  3.37582  4.18094  2.88147  0.95327  3.24287  3.39839  3.79203  5.54400  4.41104    123 r - - -
        2.68618  4.42225  2.77519  2.73123  3.46354  2.40513  3.72494  3.29354  2.67741  2.69355  4.24690  2.90347  2.73739  3.18146  2.89801  2.37887  2.77519  2.98518  4.58477  3.61503
        0.02290  4.18373  4.90608  0.61958  0.77255  0.48576  0.95510
    101   2.34004  4.81976  3.05897  2.45555  4.08375  3.46992  3.67299  3.47522  1.86134  2.81046  3.91518  3.02154  3.87081  2.79043  2.80990  2.70603  2.30489  3.16098  5.33001  4.01831    124 k - - -
        2.68618  4.42225  2.77519  2.73123  3.46354  2.40513  3.72494  3.29354  2.67741  2.69355  4.24690  2.90347  2.73739  3.18146  2.89801  2.37887  2.77519  2.98518  4.58477  3.61503
        0.02290  4.18373  4.90608  0.61958  0.77255  0.48576  0.95510
    102   2.02902  4.88377  2.76294  1.74718  4.37082  2.37000  3.78695  3.79558  2.63136  3.38710  4.19885  2.95816  3.84942  2.94302  3.10293  2.68233  2.79817  3.40096  5.60204  4.24372    125 e - - -
        2.68618  4.42225  2.77519  2.73123  3.46354  2.40513  3.72494  3.29354  2.67741  2.69355  4.24690  2.90347  2.73739  3.18146  2.89801  2.37887  2.77519  2.98518  4.58477  3.61503
        0.02290  4.18373  4.90608  0.61958  0.77255  0.48576  0.95510
    103   2.44704  4.65730  3.12638  2.56875  3.88033  3.46825  3.69866  2.87657  2.52709  2.91702  3.75977  3.06034  3.86514  2.59263  2.60105  2.11706  2.62598  2.84607  5.20435  3.90662    126 s - - -
        2.68618  4.42225  2.77519  2.73123  3.46354  2.40513  3.72494  3.29354  2.67741  2.69355  4.24690  2.90347  2.73739  3.18146  2.89801  2.37887  2.77519  2.98518  4.58477  3.61503
        0.02290  4.18373  4.90608  0.61958  0.77255  0.48576  0.95510
    104   3.36591  4.66689  4.68090  4.26149  1.71387  4.29059  3.63792  3.14313  4.09823  2.63980  3.76313  4.09262  4.60978  4.11016  4.13679  3.62223  3.58755  2.51939  1.88424  1.54967    127 y - - -
        2.68618  4.42225  2.77519  2.73123  3.46354  2.40513  3.72494  3.29354  2.67741  2.69355  4.24690  2.90347  2.73739  3.18146  2.89801  2.37887  2.77519  2.98518  4.58477  3.61503
        0.02290  4.18373  4.90608  0.61958  0.77255  0.48576  0.95510
    105   3.20153  4.48707  5.00962  4.46087  3.41952  4.57269  5.00096  1.37192  4.34854  1.61627  2.77296  4.64714  4.80945  4.50085  4.47076  3.92884  3.43909  1.42009  5.38838  4.26377    128 i - - -
        2.68618  4.42225  2.77519  2.73123  3.46354  2.40513  3.72494  3.29354  2.67741  2.69355  4.24690  2.90347  2.73739  3.18146  2.89801  2.37887  2.77519  2.98518  4.58477  3.61503
```

```
      0.02290  4.18373  4.90608  0.61958  0.77255  0.48576  0.95510
106   2.68317  4.78280  2.77895  2.64639  4.58881  1.57459  4.06622  4.12640  2.99752  3.72073  4.56184  1.53885  3.90385  3.27480  3.42603  2.75138  3.08982  3.62601  5.83192  4.49859   129 n - - -
      2.68618  4.42225  2.77519  2.73123  3.46354  2.40513  3.72494  3.29354  2.67741  2.69355  4.24690  2.90347  2.73739  3.18146  2.89801  2.37887  2.77519  2.98518  4.58477  3.61503
      0.02290  4.18373  4.90608  0.61958  0.77255  0.48576  0.95510
107   2.63139  5.15156  2.40372  1.81584  4.58917  1.57235  3.83663  4.04414  2.76173  3.60895  4.43983  2.87544  3.89649  3.00237  3.27696  2.79963  3.12919  3.63958  5.80759  4.39745   130 g - - -
      2.68620  4.42227  2.77521  2.73125  3.46356  2.40509  3.72496  3.29356  2.67743  2.69357  4.24657  2.90348  2.73734  3.18136  2.89803  2.37889  2.77521  2.98520  4.58479  3.61505
      0.05113  3.15963  4.90608  0.86594  0.54586  0.48576  0.95510
108   2.74470  4.98206  3.08306  2.52357  4.28023  3.50903  3.64855  3.68779  1.84776  3.23901  3.22445  2.94668  3.89921  1.89277  2.61923  2.75517  2.88602  3.35081  5.42185  4.11451   135 k - - -
      2.68618  4.42225  2.77519  2.73123  3.46354  2.40513  3.72494  3.29354  2.67741  2.69355  4.24690  2.90347  2.73739  3.18146  2.89801  2.37887  2.77519  2.98518  4.58477  3.61503
      0.02290  4.18373  4.90608  0.61958  0.77255  0.48576  0.95510
109   2.77991  4.31075  3.96063  3.40876  2.18446  3.80994  3.90106  2.69818  3.31252  1.87493  3.33625  3.66744  2.47021  3.55183  3.55334  3.09251  3.01214  2.54313  4.49418  2.61694   136 l - - -
      2.68618  4.42225  2.77519  2.73123  3.46354  2.40513  3.72494  3.29354  2.67741  2.69355  4.24690  2.90347  2.73739  3.18146  2.89801  2.37887  2.77519  2.98518  4.58477  3.61503
      0.02290  4.18373  4.90608  0.61958  0.77255  0.48576  0.95510
110   2.35243  4.06644  4.14991  3.56192  2.73238  3.72770  4.02125  2.47521  3.42804  2.14868  3.16791  3.73631  4.09426  3.63462  3.58268  3.01960  2.70992  1.69251  3.72365  3.14807   137 v - - -
      2.68619  4.42226  2.77521  2.73124  3.46355  2.40514  3.72496  3.29355  2.67742  2.69356  4.24691  2.90348  2.73741  3.18118  2.89802  2.37888  2.77521  2.98519  4.58478  3.61504
      0.09465  2.48998  4.90608  0.39060  1.12902  0.48576  0.95510
111   2.65505  4.26327  3.74990  3.18422  2.59657  3.68062  3.97659  2.42034  3.10920  2.30790  3.04318  3.52424  2.19895  3.38868  3.40383  2.95275  2.65299  2.43643  4.86163  3.63847   139 p - - -
      2.68618  4.42225  2.77519  2.73123  3.46354  2.40513  3.72494  3.29354  2.67741  2.69355  4.24690  2.90347  2.73739  3.18146  2.89801  2.37887  2.77519  2.98518  4.58477  3.61503
      0.02290  4.18373  4.90608  0.61958  0.77255  0.48576  0.95510
112   2.78637  5.00858  3.02622  2.57202  4.44188  3.46586  3.71838  3.88256  2.29708  3.40870  4.22837  2.07036  3.92620  2.86033  1.66458  2.50047  3.03991  3.50622  5.53906  4.23445   140 r - - -
      2.68618  4.42225  2.77519  2.73123  3.46354  2.40513  3.72494  3.29354  2.67741  2.69355  4.24690  2.90347  2.73739  3.18146  2.89801  2.37887  2.77519  2.98518  4.58477  3.61503
      0.02290  4.18373  4.90608  0.61958  0.77255  0.48576  0.95510
113   2.04863  4.27901  3.52897  3.31016  4.47014  3.03678  4.39906  3.86420  3.38871  3.59779  4.44726  3.43610  3.80935  3.68437  3.67733  0.88431  2.84263  3.30215  5.82558  4.59976   141 s - - -
      2.68618  4.42225  2.77519  2.73123  3.46354  2.40513  3.72494  3.29354  2.67741  2.69355  4.24690  2.90347  2.73739  3.18146  2.89801  2.37887  2.77519  2.98518  4.58477  3.61503
      0.02290  4.18373  4.90608  0.61958  0.77255  0.48576  0.95510
114   2.86255  4.05872  4.41807  3.85887  3.45444  4.05573  4.47548  1.97198  3.74699  2.24386  2.70402  4.07568  4.41511  3.98767  3.94545  3.37707  2.64797  1.20684  5.13231  3.94012   142 v - - -
      2.68618  4.42225  2.77519  2.73123  3.46354  2.40513  3.72494  3.29354  2.67741  2.69355  4.24690  2.90347  2.73739  3.18146  2.89801  2.37887  2.77519  2.98518  4.58477  3.61503
      0.02290  4.18373  4.90608  0.61958  0.77255  0.48576  0.95510
115   2.34314  4.49486  3.27140  2.71880  3.74670  3.26489  3.78170  2.80561  2.67534  2.79968  3.66418  3.16663  3.88878  3.01669  2.64942  1.97418  2.78968  2.54039  5.12445  3.85658   143 s - - -
      2.68618  4.42225  2.77519  2.73123  3.46354  2.40513  3.72494  3.29354  2.67741  2.69355  4.24690  2.90347  2.73739  3.18146  2.89801  2.37887  2.77519  2.98518  4.58477  3.61503
      0.02290  4.18373  4.90608  0.61958  0.77255  0.48576  0.95510
116   3.09140  4.51378  4.54717  4.00050  3.30890  4.25543  4.65725  2.07202  3.84967  0.99733  3.14035  4.25960  4.57182  4.08530  4.04191  3.59437  2.85600  2.04811  5.21639  4.08130   144 l - - -
      2.68618  4.42225  2.77519  2.73123  3.46354  2.40513  3.72494  3.29354  2.67741  2.69355  4.24690  2.90347  2.73739  3.18146  2.89801  2.37887  2.77519  2.98518  4.58477  3.61503
      0.02290  4.18373  4.90608  0.61958  0.77255  0.48576  0.95510
117   2.74946  5.20386  2.53513  1.51638  4.51589  3.38818  3.66511  3.98391  2.25332  3.48878  4.26895  2.85986  3.84802  2.41501  2.91737  2.70404  2.98949  3.32329  5.64331  4.23908   145 e - - -
      2.68618  4.42225  2.77519  2.73123  3.46354  2.40513  3.72494  3.29354  2.67741  2.69355  4.24690  2.90347  2.73739  3.18146  2.89801  2.37887  2.77519  2.98518  4.58477  3.61503
      0.02290  4.18373  4.90608  0.61958  0.77255  0.48576  0.95510
118   2.65595  4.30538  3.64592  3.07752  3.41310  3.66366  3.93227  2.32665  2.63182  1.79841  3.36818  3.45037  2.98671  3.29713  3.28950  2.92632  2.88977  2.31741  4.91393  3.69541   146 l - - -
      2.68618  4.42225  2.77519  2.73123  3.46354  2.40513  3.72494  3.29354  2.67741  2.69355  4.24690  2.90347  2.73739  3.18146  2.89801  2.37887  2.77519  2.98518  4.58477  3.61503
      0.02290  4.18373  4.90608  0.61958  0.77255  0.48576  0.95510
119   3.09995  4.83262  3.83645  3.78085  4.89495  0.37791  4.87055  4.58175  4.00380  4.19229  5.17870  4.00221  4.23262  4.30392  4.19473  3.28255  3.60515  4.04968  5.89622  5.01130   147 G - - -
      2.68618  4.42225  2.77519  2.73123  3.46354  2.40513  3.72494  3.29354  2.67741  2.69355  4.24690  2.90347  2.73739  3.18146  2.89801  2.37887  2.77519  2.98518  4.58477  3.61503
      0.02290  4.18373  4.90608  0.61958  0.77255  0.48576  0.95510
120   2.79268  5.29231  2.31458  1.95985  4.60616  2.15268  3.69698  4.09091  2.04504  3.58514  4.36746  2.61816  3.85616  2.82421  3.03557  2.73102  3.04198  3.67124  5.73689  4.30531   148 e - - -
      2.68618  4.42225  2.77519  2.73123  3.46354  2.40513  3.72494  3.29354  2.67741  2.69355  4.24690  2.90347  2.73739  3.18146  2.89801  2.37887  2.77519  2.98518  4.58477  3.61503
      0.02290  4.18373  4.90608  0.61958  0.77255  0.48576  0.95510
121   2.66734  4.89835  1.94673  2.42712  4.27938  3.35701  3.74948  3.67460  2.59416  3.29848  4.11785  2.77625  3.85237  2.90475  3.07290  2.68855  1.93409  2.99487  5.52994  4.16868   149 t - - -
      2.68618  4.42225  2.77519  2.73123  3.46354  2.40513  3.72494  3.29354  2.67741  2.69355  4.24690  2.90347  2.73739  3.18146  2.89801  2.37887  2.77519  2.98518  4.58477  3.61503
      0.02290  4.18373  4.90608  0.61958  0.77255  0.48576  0.95510
122   3.72820  5.08951  4.31383  4.11156  2.37789  4.20534  3.81093  3.69832  3.96130  3.06883  4.34214  4.12878  4.67640  4.18956  4.07623  3.82923  4.01892  3.58482  4.03002  0.57233   150 Y - - -
      2.68618  4.42225  2.77519  2.73123  3.46354  2.40513  3.72494  3.29354  2.67741  2.69355  4.24690  2.90347  2.73739  3.18146  2.89801  2.37887  2.77519  2.98518  4.58477  3.61503
      0.02290  4.18373  4.90608  0.61958  0.77255  0.48576  0.95510
123   2.13686  5.31225  1.86638  1.62744  4.63715  3.33956  3.74974  4.10935  2.64063  3.63156  4.43380  2.81905  3.87452  2.89044  3.17859  2.77147  2.98767  3.69686  5.80392  4.36016   151 e - - -
      2.68618  4.42225  2.77519  2.73123  3.46354  2.40513  3.72494  3.29354  2.67741  2.69355  4.24690  2.90347  2.73739  3.18146  2.89801  2.37887  2.77519  2.98518  4.58477  3.61503
      0.02290  4.18373  4.90608  0.61958  0.77255  0.48576  0.95510
124   3.84224  5.02788  4.83788  4.56732  0.84747  4.56163  3.57358  3.47003  4.39922  2.76176  4.05732  4.23515  4.86188  4.31275  4.38636  3.94446  4.06246  3.41413  3.69511  1.51212   152 f - - -
      2.68618  4.42225  2.77519  2.73123  3.46354  2.40513  3.72494  3.29354  2.67741  2.69355  4.24690  2.90347  2.73739  3.18146  2.89801  2.37887  2.77519  2.98518  4.58477  3.61503
      0.02290  4.18373  4.90608  0.61958  0.77255  0.48576  0.95510
125   2.92483  5.20315  3.21879  2.51242  4.60778  3.60906  3.64683  3.99539  1.38844  3.45479  4.27257  3.10078  3.98714  2.69432  1.92213  2.55856  3.11761  3.63579  5.53812  4.28333   153 k - - -
      2.68618  4.42225  2.77519  2.73123  3.46354  2.40513  3.72494  3.29354  2.67741  2.69355  4.24690  2.90347  2.73739  3.18146  2.89801  2.37887  2.77519  2.98518  4.58477  3.61503
      0.02290  4.18373  4.90608  0.61958  0.77255  0.48576  0.95510
126   2.80137  4.26601  4.16830  3.06454  3.36913  3.94440  4.27259  1.92008  3.49498  2.20643  2.43129  3.87409  4.29268  3.75243  3.72419  3.23944  3.03802  1.50388  4.99866  3.80635   154 v - - -
      2.68618  4.42225  2.77519  2.73123  3.46354  2.40513  3.72494  3.29354  2.67741  2.69355  4.24690  2.90347  2.73739  3.18146  2.89801  2.37887  2.77519  2.98518  4.58477  3.61503
      0.02290  4.18373  4.90608  0.61958  0.77255  0.48576  0.95510
127   2.63906  4.35789  3.34055  2.96145  3.53255  3.59704  3.91053  2.70779  2.91977  2.09479  3.49058  2.69248  4.00434  3.23641  3.26776  2.87260  2.76443  1.80335  4.99896  3.75796   155 v - - -
      2.68618  4.42225  2.77519  2.73123  3.46354  2.40513  3.72494  3.29354  2.67741  2.69355  4.24690  2.90347  2.73739  3.18146  2.89801  2.37887  2.77519  2.98518  4.58477  3.61503
      0.02290  4.18373  4.90608  0.61958  0.77255  0.48576  0.95510
```

128	2.94447	4.40219	4.30863	3.74111	3.22661	4.06893	4.36506	2.14063	3.58120	1.10190	2.82339	3.60658	4.39074	3.83770	3.78325	3.37576	3.17799	2.27434	4.99446	3.81480	156 l - - -
	2.68618	4.42225	2.77519	2.73123	3.46354	2.40513	3.72494	3.29354	2.67741	2.69355	4.24690	2.90347	2.73739	3.18146	2.89801	2.37887	2.77519	2.98518	4.58477	3.61503	
	0.02290	4.18373	4.90608	0.61958	0.77255	0.48576	0.95510														
129	2.76784	5.02859	3.14048	2.54126	4.35037	3.53168	3.63440	3.75651	1.59291	3.28435	3.43960	3.03717	3.90929	2.38366	2.30399	2.77010	2.69051	3.41016	5.44193	4.14385	157 k - - -
	2.68618	4.42225	2.77519	2.73123	3.46354	2.40513	3.72494	3.29354	2.67741	2.69355	4.24690	2.90347	2.73739	3.18146	2.89801	2.37887	2.77519	2.98518	4.58477	3.61503	
	0.02290	4.18373	4.90608	0.61958	0.77255	0.48576	0.95510														
130	1.16976	4.26884	3.58566	3.36168	4.50315	1.55202	4.43854	3.88296	3.46661	3.61172	4.44876	3.46801	3.81270	3.73106	3.75483	2.52160	2.84524	3.31201	5.83933	4.65617	158 a - - -
	2.68618	4.42225	2.77519	2.73123	3.46354	2.40513	3.72494	3.29354	2.67741	2.69355	4.24690	2.90347	2.73739	3.18146	2.89801	2.37887	2.77519	2.98518	4.58477	3.61503	
	0.02290	4.18373	4.90608	0.61958	0.77255	0.48576	0.95510														
131	3.39326	5.18728	3.91904	3.37864	4.62317	3.80584	4.10075	4.19699	2.43055	3.65635	4.65808	3.70929	4.30210	3.31412	0.59204	3.46858	3.64356	3.90975	5.58474	4.49376	159 R - - -
	2.68618	4.42225	2.77519	2.73123	3.46354	2.40513	3.72494	3.29354	2.67741	2.69355	4.24690	2.90347	2.73739	3.18146	2.89801	2.37887	2.77519	2.98518	4.58477	3.61503	
	0.02290	4.18373	4.90608	0.61958	0.77255	0.48576	0.95510														
132	2.68572	4.72840	3.19663	2.61840	3.92771	3.52630	3.69180	3.33182	2.40957	2.57425	3.81155	2.83380	3.90893	2.74154	1.85190	2.76095	2.90875	2.78678	5.21788	3.64362	160 r - - -
	2.68618	4.42225	2.77519	2.73123	3.46354	2.40513	3.72494	3.29354	2.67741	2.69355	4.24690	2.90347	2.73739	3.18146	2.89801	2.37887	2.77519	2.98518	4.58477	3.61503	
	0.02290	4.18373	4.90608	0.61958	0.77255	0.48576	0.95510														
133	2.14040	4.48439	3.19155	2.82665	4.21332	3.07232	3.99483	3.60766	2.86758	3.26592	4.10438	3.19950	1.55212	3.01550	3.25076	2.61163	2.52671	3.19685	5.52945	4.26078	161 p - - -
	2.68618	4.42225	2.77519	2.73123	3.46354	2.40513	3.72494	3.29354	2.67741	2.69355	4.24690	2.90347	2.73739	3.18146	2.89801	2.37887	2.77519	2.98518	4.58477	3.61503	
	0.02290	4.18373	4.90608	0.61958	0.77255	0.48576	0.95510														
134	3.09995	4.83262	3.83645	3.78085	4.89495	0.37791	4.87055	4.58175	4.00380	4.19229	5.17870	4.00221	4.23262	4.30392	4.19473	3.28255	3.60515	4.04968	5.89622	5.01130	162 G - - -
	2.68618	4.42225	2.77519	2.73123	3.46354	2.40513	3.72494	3.29354	2.67741	2.69355	4.24690	2.90347	2.73739	3.18146	2.89801	2.37887	2.77519	2.98518	4.58477	3.61503	
	0.02290	4.18373	4.90608	0.61958	0.77255	0.48576	0.95510														
135	2.62806	4.99844	2.42163	2.30672	4.29153	3.41011	3.17275	3.33579	2.36373	3.27996	4.05585	2.78222	3.80834	2.72805	2.36341	2.61747	2.43931	3.36148	5.46195	4.08730	163 e - - -
	2.68618	4.42225	2.77519	2.73123	3.46354	2.40513	3.72494	3.29354	2.67741	2.69355	4.24690	2.90347	2.73739	3.18146	2.89801	2.37887	2.77519	2.98518	4.58477	3.61503	
	0.02290	4.18373	4.90608	0.61958	0.77255	0.48576	0.95510														
136	3.83564	5.02079	4.83244	4.53413	1.86339	4.55351	3.52694	3.58097	4.34624	2.90213	4.14742	4.19757	4.84680	4.27243	4.34362	3.91541	4.04740	3.48268	1.28119	1.38283	164 w - - -
	2.68618	4.42225	2.77519	2.73123	3.46354	2.40513	3.72494	3.29354	2.67741	2.69355	4.24690	2.90347	2.73739	3.18146	2.89801	2.37887	2.77519	2.98518	4.58477	3.61503	
	0.02290	4.18373	4.90608	0.61958	0.77255	0.48576	0.95510														
137	2.86363	4.89378	3.05423	2.74806	3.92799	3.47672	1.33751	3.78927	2.69610	3.35392	4.25364	3.20700	2.43906	3.15251	3.03925	2.92262	3.16481	3.45451	5.28716	3.86823	165 h - - -
	2.68618	4.42225	2.77519	2.73123	3.46354	2.40513	3.72494	3.29354	2.67741	2.69355	4.24690	2.90347	2.73739	3.18146	2.89801	2.37887	2.77519	2.98518	4.58477	3.61503	
	0.02290	4.18373	4.90608	0.61958	0.77255	0.48576	0.95510														
138	3.05011	4.39030	4.72568	4.18406	3.56161	4.35766	4.82155	1.65819	4.07409	2.17629	2.92198	4.39711	4.66962	4.31300	4.26185	3.70281	3.01705	1.05027	5.38945	4.20442	166 v - - -
	2.68618	4.42225	2.77519	2.73123	3.46354	2.40513	3.72494	3.29354	2.67741	2.69355	4.24690	2.90347	2.73739	3.18146	2.89801	2.37887	2.77519	2.98518	4.58477	3.61503	
	0.02290	4.18373	4.90608	0.61958	0.77255	0.48576	0.95510														
139	2.85906	4.88121	3.00751	2.76477	4.04416	2.22279	1.25572	3.89776	2.80827	3.47091	4.36052	3.21917	4.01240	3.22976	3.15920	2.92373	3.19060	3.52948	5.38470	3.98267	167 h - - -
	2.68618	4.42225	2.77519	2.73123	3.46354	2.40513	3.72494	3.29354	2.67741	2.69355	4.24690	2.90347	2.73739	3.18146	2.89801	2.37887	2.77519	2.98518	4.58477	3.61503	
	0.02290	4.18373	4.90608	0.61958	0.77255	0.48576	0.95510														
140	2.13996	4.35207	3.46088	2.96382	3.93498	3.26357	3.99596	3.27402	2.93660	2.99544	3.85559	3.29363	2.17994	3.25234	3.30862	2.38751	2.11332	2.42869	5.31800	4.07404	168 t - - -
	2.68618	4.42225	2.77519	2.73123	3.46354	2.40513	3.72494	3.29354	2.67741	2.69355	4.24690	2.90347	2.73739	3.18146	2.89801	2.37887	2.77519	2.98518	4.58477	3.61503	
	0.02290	4.18373	4.90608	0.61958	0.77255	0.48576	0.95510														
141	2.67771	4.30876	3.69652	3.12894	2.78142	3.69164	3.95106	2.67307	3.04010	2.20199	2.09849	3.49165	4.06085	2.62794	3.34444	2.95539	2.64119	2.51669	4.86948	3.64529	169 m - - -
	2.68618	4.42225	2.77519	2.73123	3.46354	2.40513	3.72494	3.29354	2.67741	2.69355	4.24690	2.90347	2.73739	3.18146	2.89801	2.37887	2.77519	2.98518	4.58477	3.61503	
	0.02290	4.18373	4.90608	0.61958	0.77255	0.48576	0.95510														
142	3.19245	4.51981	4.93452	4.34813	2.49407	4.43616	4.74082	2.02344	4.20988	1.24981	1.99569	4.51417	4.65842	4.27471	4.27280	3.76734	3.41238	2.07042	5.07170	3.99191	170 l - - -
	2.68618	4.42225	2.77519	2.73123	3.46354	2.40513	3.72494	3.29354	2.67741	2.69355	4.24690	2.90347	2.73739	3.18146	2.89801	2.37887	2.77519	2.98518	4.58477	3.61503	
	0.02290	4.18373	4.90608	0.61958	0.77255	0.48576	0.95510														
143	2.64202	4.75207	2.78698	2.62317	4.47054	3.22163	4.00440	4.02416	2.89952	3.62942	4.47019	1.25087	3.88155	3.20947	3.31595	2.04428	3.03866	3.54446	5.75065	4.38126	171 n - - -
	2.68618	4.42225	2.77519	2.73123	3.46354	2.40513	3.72494	3.29354	2.67741	2.69355	4.24690	2.90347	2.73739	3.18146	2.89801	2.37887	2.77519	2.98518	4.58477	3.61503	
	0.02290	4.18373	4.90608	0.61958	0.77255	0.48576	0.95510														
144	3.16941	4.46802	4.94256	4.39120	3.41721	4.51983	4.93908	1.64487	4.27777	1.67951	2.64397	4.58290	4.76919	4.44210	4.40998	3.86998	3.40728	1.21839	5.35780	4.22518	172 v - - -
	2.68618	4.42225	2.77519	2.73123	3.46354	2.40513	3.72494	3.29354	2.67741	2.69355	4.24690	2.90347	2.73739	3.18146	2.89801	2.37887	2.77519	2.98518	4.58477	3.61503	
	0.02290	4.18373	4.90608	0.61958	0.77255	0.48576	0.95510														
145	2.73640	5.11878	2.91789	1.79868	4.44927	3.45940	3.62826	3.88920	1.84611	3.39173	3.73647	2.94239	3.86551	2.36594	2.67296	2.71147	2.95948	3.50412	5.53434	4.18353	173 e - - -
	2.68618	4.42225	2.77519	2.73123	3.46354	2.40513	3.72494	3.29354	2.67741	2.69355	4.24690	2.90347	2.73739	3.18146	2.89801	2.37887	2.77519	2.98518	4.58477	3.61503	
	0.02290	4.18373	4.90608	0.61958	0.77255	0.48576	0.95510														
146	2.84541	5.26504	1.88576	2.09931	4.63344	1.74963	3.79211	4.10764	2.71446	3.64209	4.45798	2.83301	3.88609	2.94478	3.25461	2.79183	2.75138	3.69643	5.82626	4.38951	174 g - - -
	2.68618	4.42225	2.77519	2.73123	3.46354	2.40513	3.72494	3.29354	2.67741	2.69355	4.24690	2.90347	2.73739	3.18146	2.89801	2.37887	2.77519	2.98518	4.58477	3.61503	
	0.02290	4.18373	4.90608	0.61958	0.77255	0.48576	0.95510														
147	1.80082	4.31695	3.53813	3.13370	4.02202	1.53351	4.15407	2.71309	3.12366	3.06110	3.96001	3.39162	3.87093	3.43690	3.45752	2.62350	2.84411	2.89969	5.43643	4.20258	175 g - - -
	2.68618	4.42225	2.77519	2.73123	3.46354	2.40513	3.72494	3.29354	2.67741	2.69355	4.24690	2.90347	2.73739	3.18146	2.89801	2.37887	2.77519	2.98518	4.58477	3.61503	
	0.02290	4.18373	4.90608	0.61958	0.77255	0.48576	0.95510														
148	3.09995	4.83262	3.83645	3.78085	4.89495	0.37791	4.87055	4.58175	4.00380	4.19229	5.17870	4.00221	4.23262	4.30392	4.19473	3.28255	3.60515	4.04968	5.89622	5.01130	176 G - - -
	2.68618	4.42225	2.77519	2.73123	3.46354	2.40513	3.72494	3.29354	2.67741	2.69355	4.24690	2.90347	2.73739	3.18146	2.89801	2.37887	2.77519	2.98518	4.58477	3.61503	
	0.02290	4.18373	4.90608	0.61958	0.77255	0.48576	0.95510														
149	2.81532	4.86786	2.96362	2.71975	4.33485	3.39738	3.96597	3.84770	2.71451	3.41423	4.32444	3.17648	1.23873	2.40753	3.05402	2.88015	3.15003	3.48899	5.58871	4.28742	177 p - - -
	2.68618	4.42225	2.77519	2.73123	3.46354	2.40513	3.72494	3.29354	2.67741	2.69355	4.24690	2.90347	2.73739	3.18146	2.89801	2.37887	2.77519	2.98518	4.58477	3.61503	
	0.02290	4.18373	4.90608	0.61958	0.77255	0.48576	0.95510														
150	3.18312	4.48162	4.97162	4.41679	2.69013	4.51246	4.88376	1.24488	4.29731	1.64014	3.16102	4.58663	4.75492	4.42794	4.40308	3.86146	3.41770	1.68585	5.25545	4.09895	178 i - - -

```
          2.68618  4.42225  2.77519  2.73123  3.46354  2.40513  3.72494  3.29354  2.67741  2.69355  4.24690  2.90347  2.73739  3.18146  2.89801  2.37887  2.77519  2.98518  4.58477  3.61503
          0.02290  4.18373  4.90608  0.61958  0.77255  0.48576  0.95510
    151   2.91601  4.31123  4.49618  3.93227  3.36797  4.12425  4.50409  1.34206  3.80858  2.07540  2.63173  4.14174  3.78415  4.02861  3.98329  3.44371  3.15772  1.69435  5.09951  3.92698    179 i - - -
          2.68618  4.42225  2.77519  2.73123  3.46354  2.40513  3.72494  3.29354  2.67741  2.69355  4.24690  2.90347  2.73739  3.18146  2.89801  2.37887  2.77519  2.98518  4.58477  3.61503
          0.02290  4.18373  4.90608  0.61958  0.77255  0.48576  0.95510
    152   3.09995  4.83262  3.83645  3.78085  4.89495  0.37791  4.87055  4.58175  4.00380  4.19229  5.17870  4.00221  4.23262  4.30392  4.19473  3.28255  3.60515  4.04968  5.89622  5.01130    180 G - - -
          2.68618  4.42225  2.77519  2.73123  3.46354  2.40513  3.72494  3.29354  2.67741  2.69355  4.24690  2.90347  2.73739  3.18146  2.89801  2.37887  2.77519  2.98518  4.58477  3.61503
          0.02290  4.18373  4.90608  0.61958  0.77255  0.48576  0.95510
    153   2.70519  4.59994  3.44258  3.29056  4.63854  2.51645  4.47219  4.16318  3.45464  3.80619  4.69480  3.56134  0.77239  3.77471  3.74877  2.86369  3.18216  3.63929  5.82721  4.71807    181 p - - -
          2.68618  4.42225  2.77519  2.73123  3.46354  2.40513  3.72494  3.29354  2.67741  2.69355  4.24690  2.90347  2.73739  3.18146  2.89801  2.37887  2.77519  2.98518  4.58477  3.61503
          0.02290  4.18373  4.90608  0.61958  0.77255  0.48576  0.95510
    154   2.70226  4.85600  2.66153  2.14720  4.52348  1.35105  3.94582  3.97357  2.85800  3.57333  4.41190  2.99966  3.88716  3.13544  3.31074  2.38789  3.06488  3.53626  5.77554  4.42379    182 g - - -
          2.68618  4.42225  2.77519  2.73123  3.46354  2.40513  3.72494  3.29354  2.67741  2.69355  4.24690  2.90347  2.73739  3.18146  2.89801  2.37887  2.77519  2.98518  4.58477  3.61503
          0.02290  4.18373  4.90608  0.61958  0.77255  0.48576  0.95510
    155   2.64776  4.95805  2.95841  2.11648  4.24130  3.43665  3.62264  3.23075  1.94958  3.22917  4.02095  2.93950  3.83073  2.35631  2.79549  2.52638  2.87631  3.01663  5.42423  4.07211    183 k - - -
          2.68618  4.42225  2.77519  2.73123  3.46354  2.40513  3.72494  3.29354  2.67741  2.69355  4.24690  2.90347  2.73739  3.18146  2.89801  2.37887  2.77519  2.98518  4.58477  3.61503
          0.02290  4.18373  4.90608  0.61958  0.77255  0.48576  0.95510
    156   3.09182  4.54223  4.22874  3.72561  1.96433  4.04337  3.68651  3.03808  2.90453  2.62206  3.65808  3.83188  4.38923  3.76776  3.75842  3.34397  3.31736  2.86634  1.70070  2.04200    184 w - - -
          2.68618  4.42225  2.77519  2.73123  3.46354  2.40513  3.72494  3.29354  2.67741  2.69355  4.24690  2.90347  2.73739  3.18146  2.89801  2.37887  2.77519  2.98518  4.58477  3.61503
          0.02290  4.18373  4.90608  0.61958  0.77255  0.48576  0.95510
    157   3.23554  4.50828  5.05212  4.52217  3.51326  4.64530  5.12464  1.44109  4.41436  1.55271  3.30305  4.72112  4.88308  4.59846  4.55905  4.01711  3.48021  1.24727  5.51570  4.36803    185 v - - -
          2.68618  4.42225  2.77519  2.73123  3.46354  2.40513  3.72494  3.29354  2.67741  2.69355  4.24690  2.90347  2.73739  3.18146  2.89801  2.37887  2.77519  2.98518  4.58477  3.61503
          0.02290  4.18373  4.90608  0.61958  0.77255  0.48576  0.95510
    158   2.56479  4.99260  2.37633  2.06391  4.29225  3.38992  3.68468  3.55246  2.50087  3.29546  4.10014  2.90800  3.84276  2.82488  2.98601  2.68165  1.89498  3.35039  5.51121  4.13881    186 t - - -
          2.68618  4.42225  2.77519  2.73123  3.46354  2.40513  3.72494  3.29354  2.67741  2.69355  4.24690  2.90347  2.73739  3.18146  2.89801  2.37887  2.77519  2.98518  4.58477  3.61503
          0.02290  4.18373  4.90608  0.61958  0.77255  0.48576  0.95510
    159   3.22894  4.52070  5.00986  4.53227  3.65406  4.60754  5.23256  0.93314  4.41346  2.11684  3.44809  4.72957  4.91073  4.67072  4.59283  4.01668  3.49902  1.45023  5.67759  4.47617    187 i - - -
          2.68618  4.42225  2.77519  2.73123  3.46354  2.40513  3.72494  3.29354  2.67741  2.69355  4.24690  2.90347  2.73739  3.18146  2.89801  2.37887  2.77519  2.98518  4.58477  3.61503
          0.02290  4.18373  4.90608  0.61958  0.77255  0.48576  0.95510
    160   2.54429  5.14012  2.34312  1.99219  4.45692  3.03347  3.63687  3.92787  2.09067  3.43619  4.20364  2.85676  3.81895  2.75303  2.91539  2.64924  2.40662  3.51814  5.59451  4.18835    188 e - - -
          2.68618  4.42225  2.77519  2.73123  3.46354  2.40513  3.72494  3.29354  2.67741  2.69355  4.24690  2.90347  2.73739  3.18146  2.89801  2.37887  2.77519  2.98518  4.58477  3.61503
          0.02290  4.18373  4.90608  0.61958  0.77255  0.48576  0.95510
    161   2.61022  4.52805  3.40842  3.26703  4.66584  0.77609  4.46854  4.18382  3.47635  3.82585  4.69060  3.51468  2.74206  3.76373  3.77992  2.77091  3.10140  3.61346  5.87248  4.74842    189 g - - -
          2.68618  4.42225  2.77519  2.73123  3.46354  2.40513  3.72494  3.29354  2.67741  2.69355  4.24690  2.90347  2.73739  3.18146  2.89801  2.37887  2.77519  2.98518  4.58477  3.61503
          0.02290  4.18373  4.90608  0.61958  0.77255  0.48576  0.95510
    162   2.74798  5.14588  1.82785  2.30726  4.51670  3.34256  3.72772  3.98628  2.51609  3.51685  4.31026  2.60622  3.85223  2.86523  3.09105  1.93171  2.66588  3.57697  5.69416  4.28254    190 d - - -
          2.68618  4.42225  2.77519  2.73123  3.46354  2.40513  3.72494  3.29354  2.67741  2.69355  4.24690  2.90347  2.73739  3.18146  2.89801  2.37887  2.77519  2.98518  4.58477  3.61503
          0.02290  4.18373  4.90608  0.61958  0.77255  0.48576  0.95510
    163   2.47108  4.23985  3.60519  3.03423  3.16172  3.60620  3.87077  2.72117  2.84354  2.40385  2.24146  3.05571  3.44389  3.25884  3.28746  2.74326  2.82614  2.50674  3.83942  3.40186    191 m - - -
          2.68618  4.42225  2.77519  2.73123  3.46354  2.40513  3.72494  3.29354  2.67741  2.69355  4.24690  2.90347  2.73739  3.18146  2.89801  2.37887  2.77519  2.98518  4.58477  3.61503
          0.02290  4.18373  4.90608  0.61958  0.77255  0.48576  0.95510
    164   2.07973  4.64601  3.06151  2.63623  4.26758  2.68132  3.84630  3.69085  2.27393  3.29889  4.11120  3.08177  3.82938  3.01672  3.08060  1.67856  2.87371  3.28111  5.52372  4.21526    192 s - - -
          2.68618  4.42225  2.77519  2.73123  3.46354  2.40513  3.72494  3.29354  2.67741  2.69355  4.24690  2.90347  2.73739  3.18146  2.89801  2.37887  2.77519  2.98518  4.58477  3.61503
          0.02290  4.18373  4.90608  0.61958  0.77255  0.48576  0.95510
    165   2.75781  5.26925  1.77055  2.02493  4.58463  3.16252  3.67583  4.07314  2.49995  3.56344  4.33506  2.57659  3.22259  2.70022  2.87856  2.54237  3.00455  3.64722  5.71212  4.27810    193 d - - -
          2.68618  4.42225  2.77519  2.73123  3.46354  2.40513  3.72494  3.29354  2.67741  2.69355  4.24690  2.90347  2.73739  3.18146  2.89801  2.37887  2.77519  2.98518  4.58477  3.61503
          0.02290  4.18373  4.90608  0.61958  0.77255  0.48576  0.95510
    166   3.68410  4.99736  4.66912  4.45276  0.61317  4.27914  4.05653  3.18184  4.35515  2.48406  3.87144  4.38573  4.73817  4.43916  4.39718  3.93054  3.98917  3.19377  4.23206  2.56778    194 F - - -
          2.68618  4.42225  2.77519  2.73123  3.46354  2.40513  3.72494  3.29354  2.67741  2.69355  4.24690  2.90347  2.73739  3.18146  2.89801  2.37887  2.77519  2.98518  4.58477  3.61503
          0.02290  4.18373  4.90608  0.61958  0.77255  0.48576  0.95510
    167   2.53458  4.71037  3.09366  2.47586  3.96417  3.45959  3.70055  3.35111  2.16848  2.99061  3.82854  3.04998  3.86862  2.87815  2.86369  2.57391  1.99150  2.69951  5.26212  3.96053    195 t - - -
          2.68618  4.42225  2.77519  2.73123  3.46354  2.40513  3.72494  3.29354  2.67741  2.69355  4.24690  2.90347  2.73739  3.18146  2.89801  2.37887  2.77519  2.98518  4.58477  3.61503
          0.02290  4.18373  4.90608  0.61958  0.77255  0.48576  0.95510
    168   2.81267  5.20873  2.11942  2.29373  4.45122  3.35690  3.74545  3.98586  2.62810  3.52565  4.33954  1.52822  3.87930  2.89856  3.14344  2.63279  3.07579  3.59823  5.67482  3.67149    196 n - - -
          2.68618  4.42225  2.77519  2.73123  3.46354  2.40513  3.72494  3.29354  2.67741  2.69355  4.24690  2.90347  2.73739  3.18146  2.89801  2.37887  2.77519  2.98518  4.58477  3.61503
          0.02290  4.18373  4.90608  0.61958  0.77255  0.48576  0.95510
    169   2.64038  4.86663  2.76982  2.43525  4.15527  3.41052  3.67605  3.32283  2.39960  3.17046  3.98174  2.57374  2.18314  2.82486  2.93741  2.66433  2.56166  3.23497  5.40145  4.05728    197 p - - -
          2.68618  4.42225  2.77519  2.73123  3.46354  2.40513  3.72494  3.29354  2.67741  2.69355  4.24690  2.90347  2.73739  3.18146  2.89801  2.37887  2.77519  2.98518  4.58477  3.61503
          0.02290  4.18373  4.90608  0.61958  0.77255  0.48576  0.95510
    170   3.22553  4.49247  5.05857  4.55584  3.68281  4.66894  5.24413  1.32517  4.45400  2.08912  3.46818  4.75777  4.93536  4.69934  4.63376  4.06016  3.48307  1.01376  5.68633  4.48776    198 v - - -
          2.68618  4.42225  2.77519  2.73123  3.46354  2.40513  3.72494  3.29354  2.67741  2.69355  4.24690  2.90347  2.73739  3.18146  2.89801  2.37887  2.77519  2.98518  4.58477  3.61503
          0.02290  4.18373  4.90608  0.61958  0.77255  0.48576  0.95510
    171   2.77661  4.97157  3.04509  2.55823  4.31389  3.49670  3.69501  3.71069  1.89356  3.27305  4.10402  3.04981  3.92327  2.39239  2.64154  2.79551  1.81450  3.37449  5.45932  4.16277    199 t - - -
          2.68618  4.42225  2.77519  2.73123  3.46354  2.40513  3.72494  3.29354  2.67741  2.69355  4.24690  2.90347  2.73739  3.18146  2.89801  2.37887  2.77519  2.98518  4.58477  3.61503
          0.02290  4.18373  4.90608  0.61958  0.77255  0.48576  0.95510
    172   2.64206  4.48302  3.10416  2.88993  3.74533  3.48759  3.95056  2.92939  2.87894  2.20495  3.69938  3.30135  3.97785  3.22247  3.24439  2.82040  1.54916  2.69663  5.20168  3.93102    200 t - - -
          2.68618  4.42225  2.77519  2.73123  3.46354  2.40513  3.72494  3.29354  2.67741  2.69355  4.24690  2.90347  2.73739  3.18146  2.89801  2.37887  2.77519  2.98518  4.58477  3.61503
```

```
        0.02290  4.18373  4.90608  0.61958  0.77255  0.48576  0.95510
    173   3.43666  4.73226  5.06383  4.53359  3.21179  4.72769  5.07087  1.67479  4.35987  0.85757  3.01183  4.77830  4.90670  4.49103  4.46828  4.11202  3.66809  2.17814  5.32979  4.23026    201 l - - -
        2.68618  4.42225  2.77519  2.73123  3.46354  2.40513  3.72494  3.29354  2.67741  2.69355  4.24690  2.90347  2.73739  3.18146  2.89801  2.37887  2.77519  2.98518  4.58477  3.61503
        0.02290  4.18373  4.90608  0.61958  0.77255  0.48576  0.95510
    174   2.65196  4.91605  2.36336  2.40359  4.21194  3.39675  3.67789  3.63835  2.48818  2.77008  4.03240  2.39000  3.83652  2.82208  2.96389  2.55923  2.16934  3.28720  5.44742  4.08878    202 t - - -
        2.68618  4.42225  2.77519  2.73123  3.46354  2.40513  3.72494  3.29354  2.67741  2.69355  4.24690  2.90347  2.73739  3.18146  2.89801  2.37887  2.77519  2.98518  4.58477  3.61503
        0.02290  4.18373  4.90608  0.61958  0.77255  0.48576  0.95510
    175   3.09995  4.83262  3.83645  3.78085  4.89495  0.37791  4.87055  4.58175  4.00380  4.19229  5.17870  4.00221  4.23262  4.30392  4.19473  3.28255  3.60515  4.04968  5.89622  5.01130    203 G - - -
        2.68618  4.42225  2.77519  2.73123  3.46354  2.40513  3.72494  3.29354  2.67741  2.69355  4.24690  2.90347  2.73739  3.18146  2.89801  2.37887  2.77519  2.98518  4.58477  3.61503
        0.02290  4.18373  4.90608  0.61958  0.77255  0.48576  0.95510
    176   2.67642  5.12135  2.63458  1.98665  4.43503  3.38465  3.63075  3.90356  2.40650  3.41482  4.18236  2.48335  3.81642  2.25041  2.89961  2.47914  2.47692  3.41867  5.57516  4.17336    204 e - - -
        2.68618  4.42225  2.77519  2.73123  3.46354  2.40513  3.72494  3.29354  2.67741  2.69355  4.24690  2.90347  2.73739  3.18146  2.89801  2.37887  2.77519  2.98518  4.58477  3.61503
        0.02290  4.18373  4.90608  0.61958  0.77255  0.48576  0.95510
    177   2.61999  4.34526  3.62697  3.08401  3.57017  3.56197  3.99473  2.70886  2.98922  2.32908  3.19675  3.44120  4.01609  3.32238  3.31651  2.71278  1.57787  2.42391  5.06541  3.83315    205 t - - -
        2.68618  4.42225  2.77519  2.73123  3.46354  2.40513  3.72494  3.29354  2.67741  2.69355  4.24690  2.90347  2.73739  3.18146  2.89801  2.37887  2.77519  2.98518  4.58477  3.61503
        0.02290  4.18373  4.90608  0.61958  0.77255  0.48576  0.95510
    178   3.21558  4.49150  5.04566  4.56042  3.71703  4.63778  5.26719  1.26870  4.45886  2.20000  3.50276  4.75342  4.93026  4.71700  4.64251  4.04127  3.48176  1.01622  5.72267  4.51416    206 v - - -
        2.68618  4.42225  2.77519  2.73123  3.46354  2.40513  3.72494  3.29354  2.67741  2.69355  4.24690  2.90347  2.73739  3.18146  2.89801  2.37887  2.77519  2.98518  4.58477  3.61503
        0.02290  4.18373  4.90608  0.61958  0.77255  0.48576  0.95510
    179   3.05681  5.59378  1.10720  2.20679  4.91103  3.30359  3.90215  4.48932  2.95948  3.98986  4.85596  1.93410  3.95033  3.07964  3.56914  2.94394  3.36656  4.04344  6.11067  4.59018    207 d - - -
        2.68618  4.42225  2.77519  2.73123  3.46354  2.40513  3.72494  3.29354  2.67741  2.69355  4.24690  2.90347  2.73739  3.18146  2.89801  2.37887  2.77519  2.98518  4.58477  3.61503
        0.02290  4.18373  4.90608  0.61958  0.77255  0.48576  0.95510
    180   3.46558  4.77158  5.06734  4.49610  3.11598  4.73496  5.01536  2.12620  4.33994  0.82850  2.10253  4.75952  4.87281  4.41180  4.43267  4.08898  3.67971  2.41083  5.25118  4.22075    208 l - - -
        2.68618  4.42225  2.77519  2.73123  3.46354  2.40513  3.72494  3.29354  2.67741  2.69355  4.24690  2.90347  2.73739  3.18146  2.89801  2.37887  2.77519  2.98518  4.58477  3.61503
        0.02290  4.18373  4.90608  0.61958  0.77255  0.48576  0.95510
    181   3.09018  5.40893  2.48023  0.86451  4.74794  3.41255  3.93523  4.21658  2.71228  3.77992  4.68861  2.95736  4.01702  3.11811  3.24108  3.02491  3.38099  3.85137  5.90234  4.52457    209 e - - -
        2.68618  4.42225  2.77519  2.73123  3.46354  2.40513  3.72494  3.29354  2.67741  2.69355  4.24690  2.90347  2.73739  3.18146  2.89801  2.37887  2.77519  2.98518  4.58477  3.61503
        0.02290  4.18373  4.90608  0.61958  0.77255  0.48576  0.95510
    182   2.61727  4.74068  3.01409  2.52310  4.04034  3.41485  3.56864  3.45109  2.52530  3.07273  3.90115  2.23105  3.85292  2.79032  2.96327  2.48487  2.07454  2.84026  5.32754  4.00979    210 t - - -
        2.68618  4.42225  2.77519  2.73123  3.46354  2.40513  3.72494  3.29354  2.67741  2.69355  4.24690  2.90347  2.73739  3.18146  2.89801  2.37887  2.77519  2.98518  4.58477  3.61503
        0.02290  4.18373  4.90608  0.61958  0.77255  0.48576  0.95510
    183   3.40091  4.75831  4.47999  4.04425  1.79591  4.26950  2.88014  3.29456  3.91127  2.77852  3.38318  3.99226  4.59343  3.98872  4.03478  3.58758  3.62115  3.14324  3.58652  1.05006    211 y - - -
        2.68618  4.42225  2.77519  2.73123  3.46354  2.40513  3.72494  3.29354  2.67741  2.69355  4.24690  2.90347  2.73739  3.18146  2.89801  2.37887  2.77519  2.98518  4.58477  3.61503
        0.02290  4.18373  4.90608  0.61958  0.77255  0.48576  0.95510
    184   2.18267  4.71592  2.85636  2.59275  4.39830  1.58646  3.91588  3.83144  2.65097  3.43963  4.26316  2.37545  3.85443  3.09886  3.21015  2.66978  2.95906  3.40023  5.65644  4.33314    212 g - - -
        2.68618  4.42225  2.77519  2.73123  3.46354  2.40513  3.72494  3.29354  2.67741  2.69355  4.24690  2.90347  2.73739  3.18146  2.89801  2.37887  2.77519  2.98518  4.58477  3.61503
        0.02290  4.18373  4.90608  0.61958  0.77255  0.48576  0.95510
    185   2.61851  4.26385  3.61802  2.72053  3.03595  3.63025  3.89502  2.23862  2.98239  1.97286  3.34634  3.42007  4.00536  2.96141  3.29923  2.88766  2.50541  2.49060  4.85683  3.63650    213 l - - -
        2.68618  4.42225  2.77519  2.73123  3.46354  2.40513  3.72494  3.29354  2.67741  2.69355  4.24690  2.90347  2.73739  3.18146  2.89801  2.37887  2.77519  2.98518  4.58477  3.61503
        0.02290  4.18373  4.90608  0.61958  0.77255  0.48576  0.95510
    186   2.03457  4.78824  2.25261  2.50088  4.44433  2.03727  3.85404  3.89573  2.73213  3.47539  4.27606  2.98848  3.82935  3.01458  3.21298  2.14408  2.93499  3.44763  5.67760  4.32159    214 a - - -
        2.68618  4.42225  2.77519  2.73123  3.46354  2.40513  3.72494  3.29354  2.67741  2.69355  4.24690  2.90347  2.73739  3.18146  2.89801  2.37887  2.77519  2.98518  4.58477  3.61503
        0.02290  4.18373  4.90608  0.61958  0.77255  0.48576  0.95510
    187   2.75959  5.05171  2.98977  2.49217  4.41954  3.31717  3.66264  3.84232  2.18597  3.36380  4.16689  2.07183  3.89545  2.79171  1.87123  2.66461  2.84617  3.47239  5.51436  4.19480    215 r - - -
        2.68618  4.42225  2.77519  2.73123  3.46354  2.40513  3.72494  3.29354  2.67741  2.69355  4.24690  2.90347  2.73739  3.18146  2.89801  2.37887  2.77519  2.98518  4.58477  3.61503
        0.02290  4.18373  4.90608  0.61958  0.77255  0.48576  0.95510
    188   2.42217  4.18136  3.90467  3.33586  3.33849  2.97198  4.04407  1.91869  3.24864  2.36571  2.99036  3.62219  4.09116  3.50985  3.50414  2.98805  2.50763  1.98574  4.85677  3.65174    216 i - - -
        2.68618  4.42225  2.77519  2.73123  3.46354  2.40513  3.72494  3.29354  2.67741  2.69355  4.24690  2.90347  2.73739  3.18146  2.89801  2.37887  2.77519  2.98518  4.58477  3.61503
        0.02290  4.18373  4.90608  0.61958  0.77255  0.48576  0.95510
    189   2.80586  4.22425  4.36179  3.78550  2.86062  3.94719  4.17927  1.83373  3.65180  2.05222  2.96143  3.95035  4.28548  3.83637  3.79555  3.24929  3.03711  2.04703  4.70735  2.04276    217 i - - -
        2.68618  4.42225  2.77519  2.73123  3.46354  2.40513  3.72494  3.29354  2.67741  2.69355  4.24690  2.90347  2.73739  3.18146  2.89801  2.37887  2.77519  2.98518  4.58477  3.61503
        0.02290  4.18373  4.90608  0.61958  0.77255  0.48576  0.95510
    190   2.30832  4.22198  3.66469  3.11362  2.20665  2.61291  3.93125  2.74285  3.04942  2.48001  3.00626  3.44220  3.97863  3.33185  3.35171  2.72778  2.70330  2.52081  4.87198  3.65000    218 f - - -
        2.68618  4.42225  2.77519  2.73123  3.46354  2.40513  3.72494  3.29354  2.67741  2.69355  4.24690  2.90347  2.73739  3.18146  2.89801  2.37887  2.77519  2.98518  4.58477  3.61503
        0.02290  4.18373  4.90608  0.61958  0.77255  0.48576  0.95510
    191   3.90581  5.15933  4.60044  4.39164  3.05721  4.10803  4.33949  3.95678  4.11523  3.29253  4.57374  4.48179  4.65362  4.48590  4.18455  4.10625  4.22457  3.85500  0.43840  3.04495    219 W - - -
        2.68618  4.42225  2.77519  2.73123  3.46354  2.40513  3.72494  3.29354  2.67741  2.69355  4.24690  2.90347  2.73739  3.18146  2.89801  2.37887  2.77519  2.98518  4.58477  3.61503
        0.02290  4.18373  4.90608  0.61958  0.77255  0.48576  0.95510
    192   2.66125  4.57276  3.27216  2.73981  3.56699  3.52485  1.95011  3.17561  2.63525  2.83218  3.71070  3.18673  3.93730  3.02391  3.00767  2.50644  2.56795  2.91261  3.80073  3.62047    220 h - - -
        2.68618  4.42225  2.77520  2.73123  3.46354  2.40510  3.72495  3.29354  2.67741  2.69355  4.24690  2.90347  2.73740  3.18146  2.89801  2.37887  2.77520  2.98518  4.58477  3.61503
        0.03975  3.45565  4.90608  0.54229  0.87088  0.48576  0.95510
    193   2.27534  4.14258  4.03615  3.45600  2.89688  3.74886  4.05755  2.36176  3.34954  1.65382  3.01299  3.70003  4.11509  3.58301  3.55899  2.82732  2.78755  2.19004  4.77255  3.58038    222 l - - -
        2.68618  4.42225  2.77519  2.73123  3.46354  2.40513  3.72494  3.29354  2.67741  2.69355  4.24690  2.90347  2.73739  3.18146  2.89801  2.37887  2.77519  2.98518  4.58477  3.61503
        0.02290  4.18373  4.90608  0.61958  0.77255  0.48576  0.95510
    194   2.68569  4.20036  4.01178  3.44651  2.06029  3.75315  4.04238  2.52872  3.33943  1.78423  3.23090  3.69640  3.20977  3.58036  3.55907  2.93383  2.92654  2.22648  4.73705  3.47898    223 l - - -
        2.68618  4.42225  2.77519  2.73123  3.46354  2.40513  3.72494  3.29354  2.67741  2.69355  4.24690  2.90347  2.73739  3.18146  2.89801  2.37887  2.77519  2.98518  4.58477  3.61503
        0.02290  4.18373  4.90608  0.61958  0.77255  0.48576  0.95510
```

195	2.68926	4.40136	3.74376	3.30372	3.31289	3.26597	4.00679	3.08202	3.09565	2.76650	3.73235	3.55575	4.05908	3.49719	3.34294	2.91720	2.47369	2.83841	1.39596	3.41754	224 w - - -
	2.68618	4.42225	2.77519	2.73123	3.46354	2.40513	3.72494	3.29354	2.67741	2.69355	4.24690	2.90347	2.73739	3.18146	2.89801	2.37887	2.77519	2.98518	4.58477	3.61503	
	0.02290	4.18373	4.90608	0.61958	0.77255	0.48576	0.95510														
196	2.59227	4.13740	3.83760	3.25947	2.96765	3.22928	3.94171	2.48976	3.17080	2.17808	3.08411	3.54873	4.02844	3.42473	3.42028	2.73932	2.33571	2.23391	4.74022	2.72035	225 l - - -
	2.68618	4.42225	2.77519	2.73123	3.46354	2.40513	3.72494	3.29354	2.67741	2.69355	4.24690	2.90347	2.73739	3.18146	2.89801	2.37887	2.77519	2.98518	4.58477	3.61503	
	0.02290	4.18373	4.90608	0.61958	0.77255	0.48576	0.95510														
197	1.94498	4.17759	4.07880	3.50387	2.64254	3.66359	4.11452	2.27462	3.39955	1.88692	3.23128	3.74910	4.16016	3.63461	3.61223	3.08317	2.92715	2.05849	4.82407	3.62425	226 l - - -
	2.68618	4.42225	2.77519	2.73123	3.46354	2.40513	3.72494	3.29354	2.67741	2.69355	4.24690	2.90347	2.73739	3.18146	2.89801	2.37887	2.77519	2.98518	4.58477	3.61503	
	0.02290	4.18373	4.90608	0.61958	0.77255	0.48576	0.95510														
198	2.80192	4.40137	4.79333	4.23915	3.31079	4.38001	4.79197	1.60077	4.12711	1.50395	3.26571	4.43072	4.66471	4.31487	4.27613	3.71910	3.31404	1.43436	5.28203	4.12440	227 v - - -
	2.68618	4.42225	2.77519	2.73123	3.46354	2.40513	3.72494	3.29354	2.67741	2.69355	4.24690	2.90347	2.73739	3.18146	2.89801	2.37887	2.77519	2.98518	4.58477	3.61503	
	0.02290	4.18373	4.90608	0.61958	0.77255	0.48576	0.95510														
199	1.79454	4.30480	3.51540	3.34963	4.56937	0.99193	4.47264	3.95705	3.50685	3.68889	4.53570	3.47208	3.83241	3.77018	3.78855	2.55358	2.88733	3.37149	5.89229	4.71325	228 g - - -
	2.68618	4.42225	2.77519	2.73123	3.46354	2.40513	3.72494	3.29354	2.67741	2.69355	4.24690	2.90347	2.73739	3.18146	2.89801	2.37887	2.77519	2.98518	4.58477	3.61503	
	0.02290	4.18373	4.90608	0.61958	0.77255	0.48576	0.95510														
200	2.22569	4.14312	4.05214	3.47430	3.14598	3.44616	4.08579	2.06953	3.37109	2.11099	3.12841	3.71920	4.13544	3.60941	3.58473	3.05474	2.66633	1.80946	4.81037	3.61299	229 v - - -
	2.68618	4.42225	2.77519	2.73123	3.46354	2.40513	3.72494	3.29354	2.67741	2.69355	4.24690	2.90347	2.73739	3.18146	2.89801	2.37887	2.77519	2.98518	4.58477	3.61503	
	0.02290	4.18373	4.90608	0.61958	0.77255	0.48576	0.95510														
201	1.64154	3.90137	3.73039	3.16555	2.98574	3.11262	3.93483	2.69646	3.09044	2.43426	3.34638	3.47885	3.99405	3.36403	3.37667	2.86340	2.82796	2.39239	4.09844	3.59891	230 a - - -
	2.68618	4.42225	2.77520	2.73124	3.46354	2.40510	3.72495	3.29354	2.67741	2.69351	4.24690	2.90347	2.73740	3.18147	2.89801	2.37887	2.77520	2.98519	4.58477	3.61503	
	0.03975	3.45565	4.90608	0.63180	0.75850	0.48576	0.95510														
202	3.60986	4.98319	4.44333	4.11568	2.39877	4.21320	3.81027	3.50308	3.77848	2.84891	4.10217	4.12148	4.64149	4.09197	3.89659	3.76199	3.87464	3.41432	0.81678	2.29051	233 w - - -
	2.68618	4.42225	2.77519	2.73123	3.46354	2.40513	3.72494	3.29354	2.67741	2.69355	4.24690	2.90347	2.73739	3.18146	2.89801	2.37887	2.77519	2.98518	4.58477	3.61503	
	0.02290	4.18373	4.90608	0.61958	0.77255	0.48576	0.95510														
203	3.09217	4.44966	4.76873	4.18112	3.11044	4.30100	4.59684	1.71683	4.03330	1.12100	2.66916	4.35453	4.55716	4.14554	4.12441	3.62211	3.31467	2.25001	4.26942	3.88748	234 l - - -
	2.68618	4.42225	2.77519	2.73123	3.46354	2.40513	3.72494	3.29354	2.67741	2.69355	4.24690	2.90347	2.73739	3.18146	2.89801	2.37887	2.77519	2.98518	4.58477	3.61503	
	0.03975	4.18373	3.74115	0.61958	0.77255	0.48576	0.95510														
204	2.56998	4.07042	4.10609	3.51909	2.92894	3.16774	4.00492	2.41569	3.39131	1.68249	3.16496	3.70901	4.08167	3.60314	3.55777	3.00461	2.83892	2.07814	4.10087	3.02557	235 l - - -
	2.68618	4.42225	2.77519	2.73123	3.46354	2.40513	3.72494	3.29354	2.67741	2.69355	4.24690	2.90347	2.73739	3.18146	2.89801	2.37887	2.77519	2.98518	4.58477	3.61503	
	0.02329	4.16727	4.88962	0.61958	0.77255	0.47337	0.97525														
205	3.97450	5.07815	4.98313	4.75448	1.53797	4.64533	3.50205	3.67162	4.56228	2.96040	4.22903	4.26378	4.92316	4.37787	4.48113	4.01795	4.17987	3.57965	2.00357	1.01757	236 y - - -
	2.68618	4.42225	2.77519	2.73123	3.46354	2.40513	3.72494	3.29354	2.67741	2.69355	4.24690	2.90347	2.73739	3.18146	2.89801	2.37887	2.77519	2.98518	4.58477	3.61503	
	0.02290	4.18373	4.90608	0.61958	0.77255	0.48576	0.95510														
206	3.60996	4.95921	4.56569	4.23562	2.26988	4.25249	3.83080	3.38723	3.97713	2.71464	4.00182	4.18930	4.67233	4.18521	4.07486	3.78835	3.87972	3.32848	0.83527	2.35238	237 w - - -
	2.68619	4.42226	2.77521	2.73124	3.46355	2.40511	3.72496	3.29355	2.67742	2.69342	4.24691	2.90348	2.73741	3.18147	2.89802	2.37888	2.77521	2.98519	4.58478	3.61504	
	0.09623	2.47283	4.90608	0.38759	1.13536	0.48576	0.95510														
207	2.49377	3.53015	4.37199	3.78086	2.53051	3.83124	4.16662	2.09839	3.62851	2.04654	3.05979	3.90003	4.18985	3.81333	3.73843	3.06962	2.80807	1.68516	4.74379	3.55989	239 v - - -
	2.68618	4.42225	2.77519	2.73123	3.46354	2.40513	3.72494	3.29354	2.67741	2.69355	4.24690	2.90347	2.73739	3.18146	2.89801	2.37887	2.77519	2.98518	4.58477	3.61503	
	0.02290	4.18373	4.90608	0.61958	0.77255	0.48576	0.95510														
208	2.53059	4.62243	3.16315	2.59885	3.65472	3.27788	3.70499	3.01335	2.36116	2.63979	3.71104	3.08497	3.87896	2.90565	2.13214	2.55271	2.84780	2.74361	5.15909	3.87096	240 r - - -
	2.68618	4.42225	2.77519	2.73123	3.46354	2.40513	3.72494	3.29354	2.67741	2.69355	4.24690	2.90347	2.73739	3.18146	2.89801	2.37887	2.77519	2.98518	4.58477	3.61503	
	0.02290	4.18373	4.90608	0.61958	0.77255	0.48576	0.95510														
209	3.02528	5.21262	3.43853	2.77106	4.64824	3.69284	3.66291	4.00645	1.45378	3.45896	4.30098	2.99421	4.05578	2.79018	1.48749	3.01565	3.20198	3.42684	5.52134	4.31765	241 k - - -
	2.68618	4.42225	2.77519	2.73123	3.46354	2.40513	3.72494	3.29354	2.67741	2.69355	4.24690	2.90347	2.73739	3.18146	2.89801	2.37887	2.77519	2.98518	4.58477	3.61503	
	0.02290	4.18373	4.90608	0.61958	0.77255	0.48576	0.95510														
210	2.84884	4.98534	3.14570	2.66448	4.38362	3.53206	3.73531	3.78335	2.00820	3.33030	4.17682	3.12776	1.64537	2.88885	2.26353	2.87755	3.08770	3.44722	5.48563	4.22383	242 p - - -
	2.68618	4.42225	2.77519	2.73123	3.46354	2.40513	3.72494	3.29354	2.67741	2.69355	4.24690	2.90347	2.73739	3.18146	2.89801	2.37887	2.77519	2.98518	4.58477	3.61503	
	0.21185	4.18373	1.73911	0.61958	0.77255	0.48576	0.95510														
211	2.50946	4.21604	3.60216	3.03338	3.34031	2.74026	3.87323	2.46142	2.95586	2.09248	2.96021	3.39616	3.97039	3.25403	3.14003	2.85209	2.81246	2.22360	4.83166	3.61318	243 l - - -
	2.68618	4.42225	2.77519	2.73123	3.46354	2.40513	3.72494	3.29354	2.67741	2.69355	4.24690	2.90347	2.73739	3.18146	2.89801	2.37887	2.77519	2.98518	4.58477	3.61503	
	0.02760	3.99948	4.72183	0.61958	0.77255	0.38281	1.14553														
212	2.94571	4.39407	4.38242	3.85628	1.44937	4.03724	4.27897	2.07280	3.72838	2.01269	3.23864	4.04170	4.40256	3.93502	3.90249	3.36946	2.74320	2.27922	4.77363	3.39996	244 f - - -
	2.68618	4.42225	2.77519	2.73123	3.46354	2.40513	3.72494	3.29354	2.67741	2.69355	4.24690	2.90347	2.73739	3.18146	2.89801	2.37887	2.77519	2.98518	4.58477	3.61503	
	0.02290	4.18373	4.90608	0.61958	0.77255	0.48576	0.95510														
213	2.54322	4.27977	4.33662	3.77844	3.47024	4.03466	4.43502	1.51844	3.67729	2.04839	3.37608	4.02079	4.39254	3.92783	3.89549	3.34857	2.40169	1.70413	5.12981	3.93211	245 i - - -
	2.68618	4.42225	2.77519	2.73123	3.46354	2.40513	3.72494	3.29354	2.67741	2.69355	4.24690	2.90347	2.73739	3.18146	2.89801	2.37887	2.77519	2.98518	4.58477	3.61503	
	0.02290	4.18373	4.90608	0.61958	0.77255	0.48576	0.95510														
214	2.41101	4.88014	2.94473	2.33384	4.23193	3.05554	3.67446	3.66243	2.43719	3.23482	4.03442	2.96326	2.39714	2.81370	2.39894	2.42236	2.88229	3.29834	5.44298	4.09822	246 e - - -
	2.68618	4.42225	2.77519	2.73123	3.46354	2.40513	3.72494	3.29354	2.67741	2.69355	4.24690	2.90347	2.73739	3.18146	2.89801	2.37887	2.77519	2.98518	4.58477	3.61503	
	0.02290	4.18373	4.90608	0.61958	0.77255	0.48576	0.95510														
215	2.87022	4.90233	3.43042	2.91032	4.44705	3.52696	3.82693	3.89084	2.24352	3.42962	4.30468	3.28801	4.02886	2.99664	1.11031	2.41274	3.15654	3.52835	5.51896	4.28956	247 r - - -
	2.68618	4.42225	2.77519	2.73123	3.46354	2.40513	3.72494	3.29354	2.67741	2.69355	4.24690	2.90347	2.73739	3.18146	2.89801	2.37887	2.77519	2.98518	4.58477	3.61503	
	0.02290	4.18373	4.90608	0.61958	0.77255	0.48576	0.95510														
216	2.68954	4.26874	3.79366	3.22883	2.85007	3.70837	3.18806	2.73997	3.13160	1.86050	3.25307	3.53233	4.07837	3.40468	3.25924	2.87904	2.92012	2.53957	4.56476	2.18341	248 l - - -
	2.68618	4.42225	2.77519	2.73123	3.46354	2.40513	3.72494	3.29354	2.67741	2.69355	4.24690	2.90347	2.73739	3.18146	2.89801	2.37887	2.77519	2.98518	4.58477	3.61503	
	0.02290	4.18373	4.90608	0.61958	0.77255	0.48576	0.95510														
217	2.49735	4.20998	3.67565	3.10268	3.00586	2.79392	3.89832	2.29841	3.03236	2.19727	3.15140	3.44833	3.77311	3.31146	2.82646	2.88583	2.82920	2.32160	4.80816	3.59335	249 l - - -

	2.68618	4.42225	2.77519	2.73123	3.46354	2.40513	3.72494	3.29354	2.67741	2.69355	4.24690	2.90347	2.73739	3.18146	2.89801	2.37887	2.77519	2.98518	4.58477	3.61503	
	0.02290	4.18373	4.90608	0.61958	0.77255	0.48576	0.95510														
218	2.29065	4.40503	3.23965	2.79451	3.55568	3.54712	3.78620	2.92369	2.74660	2.24881	2.76702	3.23157	3.92881	2.82328	2.71950	2.70727	2.77742	2.68664	4.24527	3.72483	250 l - - -
	2.68618	4.42225	2.77519	2.73123	3.46354	2.40513	3.72494	3.29354	2.67741	2.69355	4.24690	2.90347	2.73739	3.18146	2.89801	2.37887	2.77519	2.98518	4.58477	3.61503	
	0.02290	4.18373	4.90608	0.61958	0.77255	0.48576	0.95510														
219	2.50115	4.01780	4.30027	3.74033	3.43940	3.66153	4.36164	1.99430	3.63032	2.04645	3.37198	3.95874	4.32152	3.87686	3.83630	3.25823	3.03949	1.28177	5.05635	3.85591	251 v - - -
	2.68618	4.42225	2.77519	2.73123	3.46354	2.40513	3.72494	3.29354	2.67741	2.69355	4.24690	2.90347	2.73739	3.18146	2.89801	2.37887	2.77519	2.98518	4.58477	3.61503	
	0.02290	4.18373	4.90608	0.61958	0.77255	0.48576	0.95510														
220	2.15565	5.01133	2.57810	2.15238	4.30368	3.08365	3.61512	3.75562	2.38504	3.29457	4.07011	2.83224	3.80755	2.60420	2.79762	2.46030	2.82485	3.37410	5.47779	3.49414	252 e - - -
	2.68618	4.42225	2.77519	2.73123	3.46354	2.40513	3.72494	3.29354	2.67741	2.69355	4.24690	2.90347	2.73739	3.18146	2.89801	2.37887	2.77519	2.98518	4.58477	3.61503	
	0.02290	4.18373	4.90608	0.61958	0.77255	0.48576	0.95510														
221	1.91880	4.59222	3.06816	2.44873	3.83229	3.45676	3.73940	3.19982	2.60930	2.63804	3.72944	3.09456	3.87378	2.94584	3.03747	2.33340	2.71629	2.69218	5.18373	3.89518	253 a - - -
	2.68618	4.42225	2.77519	2.73123	3.46354	2.40513	3.72494	3.29354	2.67741	2.69355	4.24690	2.90347	2.73739	3.18146	2.89801	2.37887	2.77519	2.98518	4.58477	3.61503	
	0.02290	4.18373	4.90608	0.61958	0.77255	0.48576	0.95510														
222	2.63635	4.66796	2.94058	2.35820	4.21534	1.38590	3.94534	3.55463	2.82061	3.25851	4.13064	3.12131	3.89885	3.15618	3.22304	2.73200	2.98337	2.75259	5.53737	4.23536	254 g - - -
	2.68618	4.42225	2.77519	2.73123	3.46354	2.40513	3.72494	3.29354	2.67741	2.69355	4.24690	2.90347	2.73739	3.18146	2.89801	2.37887	2.77519	2.98518	4.58477	3.61503	
	0.02290	4.18373	4.90608	0.61958	0.77255	0.48576	0.95510														
223	2.75742	5.02147	3.11339	2.44073	4.33923	3.52088	3.63533	3.74857	1.71317	2.75393	4.08863	3.02671	3.33992	2.76436	2.08106	2.68077	2.97093	3.40106	5.44140	4.13783	255 k - - -
	2.68618	4.42225	2.77519	2.73123	3.46354	2.40513	3.72494	3.29354	2.67741	2.69355	4.24690	2.90347	2.73739	3.18146	2.89801	2.37887	2.77519	2.98518	4.58477	3.61503	
	0.02290	4.18373	4.90608	0.61958	0.77255	0.48576	0.95510														
224	2.10705	4.46790	2.85127	1.97204	4.22283	3.19096	3.62790	3.66066	2.22340	3.22338	4.01151	2.92211	3.49549	2.75640	2.87576	2.62342	2.85569	3.21775	5.42655	4.06376	256 e - - -
	2.68618	4.42225	2.77519	2.73123	3.46354	2.40513	3.72494	3.29354	2.67741	2.69355	4.24690	2.90347	2.73739	3.18146	2.89801	2.37887	2.77519	2.98518	4.58477	3.61503	
	0.02290	4.18373	4.90608	0.61958	0.77255	0.48576	0.95510														
225	2.83983	5.37153	1.68565	1.65536	4.67000	3.16072	3.30362	4.16875	2.60160	3.44302	4.44937	2.66500	3.86868	2.86035	3.13984	2.76415	3.09574	3.74358	5.81131	4.35714	257 e - - -
	2.68618	4.42225	2.77519	2.73123	3.46354	2.40513	3.72494	3.29354	2.67741	2.69355	4.24690	2.90347	2.73739	3.18146	2.89801	2.37887	2.77519	2.98518	4.58477	3.61503	
	0.02290	4.18373	4.90608	0.61958	0.77255	0.48576	0.95510														
226	2.78307	5.25276	2.23359	1.45386	4.57035	3.35722	3.70533	4.04831	2.54605	3.55790	4.34535	2.83327	3.85569	2.83630	2.93972	2.41297	2.80177	3.63642	5.72115	4.29420	258 e - - -
	2.68618	4.42225	2.77519	2.73123	3.46354	2.40513	3.72494	3.29354	2.67741	2.69355	4.24690	2.90347	2.73739	3.18146	2.89801	2.37887	2.77519	2.98518	4.58477	3.61503	
	0.02290	4.18373	4.90608	0.61958	0.77255	0.48576	0.95510														
227	2.68167	4.42009	4.04791	3.51997	3.32523	3.90866	4.26859	2.44502	3.35144	1.16104	3.22294	3.82644	4.28783	3.31458	3.59215	3.22962	3.12541	2.14238	5.04644	3.86515	259 l - - -
	2.68618	4.42225	2.77515	2.73123	3.46354	2.40513	3.72495	3.29354	2.67741	2.69355	4.24690	2.90347	2.73740	3.18146	2.89801	2.37887	2.77520	2.98518	4.58477	3.61503	
	0.03975	3.45565	4.90608	0.54229	0.87088	0.48576	0.95510														
228	3.13461	4.43684	4.92894	4.38718	2.62859	4.47025	4.87334	1.28866	4.27176	1.98156	3.27276	4.54896	4.74225	4.43870	4.39685	3.82401	3.37731	1.39799	5.28856	4.08584	261 i - - -
	2.68618	4.42225	2.77519	2.73123	3.46354	2.40513	3.72494	3.29354	2.67741	2.69355	4.24690	2.90347	2.73739	3.18146	2.89801	2.37887	2.77519	2.98518	4.58477	3.61503	
	0.02290	4.18373	4.90608	0.61958	0.77255	0.48576	0.95510														
229	2.43880	4.46610	3.20609	2.82990	4.29717	2.61845	4.01331	3.72369	2.90223	3.35931	4.17539	2.75178	3.81086	3.21674	3.30600	1.84993	1.64647	3.26364	5.59591	4.31938	262 t - - -
	2.68618	4.42225	2.77519	2.73123	3.46354	2.40513	3.72494	3.29354	2.67741	2.69355	4.24690	2.90347	2.73739	3.18146	2.89801	2.37887	2.77519	2.98518	4.58477	3.61503	
	0.02290	4.18373	4.90608	0.61958	0.77255	0.48576	0.95510														
230	2.26257	5.02089	2.32034	2.35688	4.37849	3.03824	3.68741	3.83131	2.30570	3.37836	4.16757	2.89570	2.32105	2.82050	2.98704	2.62895	2.93377	3.44037	5.56772	4.18407	263 a - - -
	2.68618	4.42225	2.77519	2.73123	3.46354	2.40513	3.72494	3.29354	2.67741	2.69355	4.24690	2.90347	2.73739	3.18146	2.89801	2.37887	2.77519	2.98518	4.58477	3.61503	
	0.02290	4.18373	4.90608	0.61958	0.77255	0.48576	0.95510														
231	2.58242	4.84753	2.72930	2.39610	4.09672	2.90722	3.63567	3.51862	2.34993	2.74910	3.48220	2.95460	3.82440	2.37587	2.89053	2.63675	2.52206	3.09064	5.34322	4.00034	264 k - - -
	2.68618	4.42225	2.77519	2.73123	3.46354	2.40513	3.72494	3.29354	2.67741	2.69355	4.24690	2.90347	2.73739	3.18146	2.89801	2.37887	2.77519	2.98518	4.58477	3.61503	
	0.02290	4.18373	4.90608	0.61958	0.77255	0.48576	0.95510														
232	3.06184	5.63171	1.01028	1.93729	4.93530	3.30634	3.88263	4.43733	2.93043	3.95516	4.82203	2.77365	3.94585	3.05479	3.54099	2.94137	3.13309	4.01085	6.12622	4.60522	265 d - - -
	2.68618	4.42225	2.77519	2.73123	3.46354	2.40513	3.72494	3.29354	2.67741	2.69355	4.24690	2.90347	2.73739	3.18146	2.89801	2.37887	2.77519	2.98518	4.58477	3.61503	
	0.02290	4.18373	4.90608	0.61958	0.77255	0.48576	0.95510														
233	3.07390	5.25848	3.49111	2.80269	4.71761	3.72446	3.66096	4.06032	1.45363	3.49048	4.33640	3.22541	4.07875	2.61873	1.44641	3.05786	2.97136	3.72491	5.53685	4.35167	266 r - - -
	2.68618	4.42225	2.77519	2.73123	3.46354	2.40513	3.72494	3.29354	2.67741	2.69355	4.24690	2.90347	2.73739	3.18146	2.89801	2.37887	2.77519	2.98518	4.58477	3.61503	
	0.02290	4.18373	4.90608	0.61958	0.77255	0.48576	0.95510														
234	2.70382	5.02816	3.03215	2.45701	4.34488	3.47642	3.61865	3.77271	1.80988	3.17765	4.08936	2.79259	3.86186	2.32813	2.39191	2.69700	2.56853	3.19167	5.45932	4.12580	267 k - - -
	2.68618	4.42225	2.77519	2.73123	3.46354	2.40513	3.72494	3.29354	2.67741	2.69355	4.24690	2.90347	2.73739	3.18146	2.89801	2.37887	2.77519	2.98518	4.58477	3.61503	
	0.02290	4.18373	4.90608	0.61958	0.77255	0.48576	0.95510														
235	3.14422	4.44256	4.94615	4.39505	2.84711	4.48653	4.88719	1.52906	4.27937	1.72736	3.23099	4.56224	4.74439	4.43404	4.39734	3.83587	3.38233	1.26917	5.29556	4.13118	268 v - - -
	2.68618	4.42225	2.77519	2.73123	3.46354	2.40513	3.72494	3.29354	2.67741	2.69355	4.24690	2.90347	2.73739	3.18146	2.89801	2.37887	2.77519	2.98518	4.58477	3.61503	
	0.02290	4.18373	4.90608	0.61958	0.77255	0.48576	0.95510														
236	1.71947	2.85197	3.91768	3.52045	4.21642	1.39506	4.39461	3.58219	3.46581	3.31554	4.15627	3.52842	3.78894	3.72507	3.72879	2.41699	2.55757	3.09272	5.61088	4.43492	269 g - - -
	2.68618	4.42225	2.77519	2.73123	3.46354	2.40513	3.72494	3.29354	2.67741	2.69355	4.24690	2.90347	2.73739	3.18146	2.89801	2.37887	2.77519	2.98518	4.58477	3.61503	
	0.02290	4.18373	4.90608	0.61958	0.77255	0.48576	0.95510														
237	2.23270	4.17276	3.87152	3.29380	2.70416	3.69898	3.98514	2.40274	2.72334	2.06955	2.58397	3.58815	4.06655	3.45844	3.45285	2.97241	2.86322	2.31407	4.78031	3.58018	270 l - - -
	2.68618	4.42225	2.77519	2.73123	3.46354	2.40513	3.72494	3.29354	2.67741	2.69355	4.24690	2.90347	2.73739	3.18146	2.89801	2.37887	2.77519	2.98518	4.58477	3.61503	
	0.02290	4.18373	4.90608	0.61958	0.77255	0.48576	0.95510														
238	2.15962	4.22763	3.83586	3.27569	3.39000	2.75423	4.04453	2.15563	3.19557	1.94977	3.35431	3.58601	4.08457	3.47158	3.47461	2.97086	2.89653	2.14771	4.91362	3.70414	271 l - - -
	2.68618	4.42225	2.77519	2.73123	3.46354	2.40513	3.72494	3.29354	2.67741	2.69355	4.24690	2.90347	2.73739	3.18146	2.89801	2.37887	2.77519	2.98518	4.58477	3.61503	
	0.02290	4.18373	4.90608	0.61958	0.77255	0.48576	0.95510														
239	2.73158	4.24991	4.43934	3.86520	1.92899	4.02192	4.35716	2.08024	3.73841	1.90982	3.19016	4.04795	4.35821	3.93405	3.89078	3.33269	2.87426	1.71304	4.92766	3.73409	272 v - - -
	2.68618	4.42225	2.77519	2.73123	3.46354	2.40513	3.72494	3.29354	2.67741	2.69355	4.24690	2.90347	2.73739	3.18146	2.89801	2.37887	2.77519	2.98518	4.58477	3.61503	

	0.02290	4.18373	4.90608	0.61958	0.77255	0.48576	0.95510														
240	2.18394	4.21274	3.80299	3.23758	3.35669	3.42001	3.99927	2.56212	3.15263	1.75248	2.96971	3.54951	4.05335	3.42913	3.43007	2.93824	2.54475	2.25509	4.86844	3.66139	273 l - - -
	2.68618	4.42225	2.77519	2.73123	3.46354	2.40513	3.72494	3.29354	2.67741	2.69355	4.24690	2.90347	2.73739	3.18146	2.89801	2.37887	2.77519	2.98518	4.58477	3.61503	
	0.02290	4.18373	4.90608	0.61958	0.77255	0.48576	0.95510														
241	1.90538	4.33284	4.38979	3.84208	3.52846	4.10065	4.54032	1.68555	3.74672	2.10015	3.40841	4.09415	4.46173	4.00511	3.97695	3.42516	3.16198	1.51298	5.24007	4.04070	274 v - - -
	2.68618	4.42225	2.77519	2.73123	3.46354	2.40513	3.72494	3.29354	2.67741	2.69355	4.24690	2.90347	2.73739	3.18146	2.89801	2.37887	2.77519	2.98518	4.58477	3.61503	
	0.02290	4.18373	4.90608	0.61958	0.77255	0.48576	0.95510														
242	2.01074	4.34069	3.55006	3.02050	3.63784	2.06494	3.97159	2.53270	2.96640	2.39586	3.58226	3.38146	3.96482	3.28246	3.31396	2.79078	2.86573	2.50736	5.09995	3.86353	275 a - - -
	2.68618	4.42225	2.77519	2.73123	3.46354	2.40513	3.72494	3.29354	2.67741	2.69355	4.24690	2.90347	2.73739	3.18146	2.89801	2.37887	2.77519	2.98518	4.58477	3.61503	
	0.02290	4.18373	4.90608	0.61958	0.77255	0.48576	0.95510														
243	2.61682	4.32024	3.97574	3.54557	3.81295	3.50762	4.41198	2.48949	3.44506	2.64798	3.72838	3.73490	4.11485	3.77029	3.71128	2.91671	1.45375	1.62389	5.41329	4.19298	276 t - - -
	2.68618	4.42225	2.77519	2.73123	3.46354	2.40513	3.72494	3.29354	2.67741	2.69355	4.24690	2.90347	2.73739	3.18146	2.89801	2.37887	2.77519	2.98518	4.58477	3.61503	
	0.02290	4.18373	4.90608	0.61958	0.77255	0.48576	0.95510														
244	2.84061	4.26956	4.33279	3.75836	3.29390	4.00738	4.34152	1.59554	3.64358	1.81362	2.87647	3.98808	3.11939	3.86256	3.83164	3.30953	3.07567	1.85033	4.97487	3.80248	277 i - - -
	2.68618	4.42225	2.77519	2.73123	3.46354	2.40513	3.72494	3.29354	2.67741	2.69355	4.24690	2.90347	2.73739	3.18146	2.89801	2.37887	2.77519	2.98518	4.58477	3.61503	
	0.02290	4.18373	4.90608	0.61958	0.77255	0.48576	0.95510														
245	2.56989	4.29146	4.35800	3.80273	3.41767	3.83766	4.43280	2.01464	3.68476	1.55117	3.32291	4.03183	4.38748	3.93230	3.88838	3.34515	3.10845	1.48880	5.10093	3.91139	278 v - - -
	2.68618	4.42225	2.77519	2.73123	3.46354	2.40513	3.72494	3.29354	2.67741	2.69355	4.24690	2.90347	2.73739	3.18146	2.89801	2.37887	2.77519	2.98518	4.58477	3.61503	
	0.02290	4.18373	4.90608	0.61958	0.77255	0.48576	0.95510														
246	2.53961	4.38726	4.66981	4.11090	3.40379	4.29563	4.69185	1.48071	3.99605	1.50508	3.15603	4.32241	4.59479	4.19923	4.16313	3.62562	3.27103	1.74039	5.22848	4.07179	279 i - - -
	2.68618	4.42225	2.77519	2.73123	3.46354	2.40513	3.72494	3.29354	2.67741	2.69355	4.24690	2.90347	2.73739	3.18146	2.89801	2.37887	2.77519	2.98518	4.58477	3.61503	
	0.02290	4.18373	4.90608	0.61958	0.77255	0.48576	0.95510														
247	2.98903	4.38872	4.54672	4.02150	3.63756	4.21757	4.74651	1.81199	3.90657	2.20210	3.48572	4.25819	4.58807	4.19086	4.13361	3.57021	2.65936	1.01727	5.42609	4.21709	280 v - - -
	2.68618	4.42225	2.77519	2.73123	3.46354	2.40513	3.72494	3.29354	2.67741	2.69355	4.24690	2.90347	2.73739	3.18146	2.89801	2.37887	2.77519	2.98518	4.58477	3.61503	
	0.02290	4.18373	4.90608	0.61958	0.77255	0.48576	0.95510														
248	2.01048	4.16932	4.17104	3.59266	2.67253	3.70361	4.15583	1.88375	3.47846	1.98644	3.13742	3.81396	4.19408	3.70183	3.66859	3.12908	2.94451	2.14914	4.82491	3.63129	281 i - - -
	2.68618	4.42225	2.77519	2.73123	3.46354	2.40513	3.72494	3.29354	2.67741	2.69355	4.24690	2.90347	2.73739	3.18146	2.89801	2.37887	2.77519	2.98518	4.58477	3.61503	
	0.02290	4.18373	4.90608	0.61958	0.77255	0.48576	0.95510														
249	2.10911	4.11595	4.05087	3.47020	2.75188	3.20612	4.04887	2.09062	3.36032	2.25926	2.83507	3.69978	4.10361	3.59029	3.56042	3.02082	2.86631	2.03558	4.75438	3.56005	282 v - - -
	2.68618	4.42225	2.77519	2.73123	3.46354	2.40513	3.72494	3.29354	2.67741	2.69355	4.24690	2.90347	2.73739	3.18146	2.89801	2.37887	2.77519	2.98518	4.58477	3.61503	
	0.02290	4.18373	4.90608	0.61958	0.77255	0.48576	0.95510														
250	2.15690	4.36640	3.41408	2.87777	3.06339	2.25964	3.87114	3.01260	2.84610	2.50632	3.60176	3.27085	3.90381	3.15852	3.21941	2.40202	2.64423	2.74773	5.06988	3.82287	283 a - - -
	2.68618	4.42225	2.77519	2.73123	3.46354	2.40513	3.72494	3.29354	2.67741	2.69355	4.24690	2.90347	2.73739	3.18146	2.89801	2.37887	2.77519	2.98518	4.58477	3.61503	
	0.02290	4.18373	4.90608	0.61958	0.77255	0.48576	0.95510														
251	2.21456	4.25039	3.65495	3.09208	3.11015	3.63087	3.88028	2.71673	3.02142	2.43761	2.55764	3.07292	4.01192	3.30713	3.32591	2.89498	2.85577	2.50862	4.77107	2.36181	284 a - - -
	2.68618	4.42225	2.77519	2.73123	3.46354	2.40513	3.72494	3.29354	2.67741	2.69355	4.24690	2.90347	2.73739	3.18146	2.89801	2.37887	2.77519	2.98518	4.58477	3.61503	
	0.02290	4.18373	4.90608	0.61958	0.77255	0.48576	0.95510														
252	2.08320	4.34203	3.43692	2.87172	3.09707	3.16332	3.81885	2.84975	2.72733	2.47604	3.13042	3.28643	3.94449	3.08006	3.19161	2.49684	2.67733	2.45259	4.92682	3.68787	285 a - - -
	2.68618	4.42225	2.77519	2.73123	3.46354	2.40513	3.72494	3.29354	2.67741	2.69355	4.24690	2.90347	2.73739	3.18146	2.89801	2.37887	2.77519	2.98518	4.58477	3.61503	
	0.02290	4.18373	4.90608	0.61958	0.77255	0.48576	0.95510														
253	2.58516	4.19347	3.78375	3.21131	3.32305	3.66592	3.96228	2.10429	3.12966	2.31634	3.05349	3.53039	4.04419	3.27117	3.40641	2.36060	2.56210	2.04135	4.82140	3.61225	286 v - - -
	2.68618	4.42225	2.77519	2.73123	3.46354	2.40513	3.72494	3.29354	2.67741	2.69355	4.24690	2.90347	2.73739	3.18146	2.89801	2.37887	2.77519	2.98518	4.58477	3.61503	
	0.02290	4.18373	4.90608	0.61958	0.77255	0.48576	0.95510														
254	1.94477	4.26795	3.69246	3.39334	4.26375	3.12111	4.38786	3.38189	3.36687	3.27045	4.19859	3.50912	3.86109	3.68924	3.64018	2.58105	1.05213	2.98409	5.68540	4.48331	287 t - - -
	2.68618	4.42225	2.77519	2.73123	3.46354	2.40513	3.72494	3.29354	2.67741	2.69355	4.24690	2.90347	2.73739	3.18146	2.89801	2.37887	2.77519	2.98518	4.58477	3.61503	
	0.02290	4.18373	4.90608	0.61958	0.77255	0.48576	0.95510														
255	2.41561	4.88692	2.96039	2.35915	4.15274	3.42985	3.63733	3.57792	2.22720	3.15908	3.56910	2.11492	3.82859	2.72224	2.86570	2.46622	2.86110	3.06834	5.37919	4.03292	288 n - - -
	2.68618	4.42225	2.77519	2.73123	3.46354	2.40513	3.72494	3.29354	2.67741	2.69355	4.24690	2.90347	2.73739	3.18146	2.89801	2.37887	2.77519	2.98518	4.58477	3.61503	
	0.02290	4.18373	4.90608	0.61958	0.77255	0.48576	0.95510														
256	2.36672	5.11091	2.62952	2.09022	4.42609	3.02800	3.64281	3.89223	2.26493	3.41091	4.18352	2.50574	3.82056	2.76233	2.92683	2.10595	2.92277	3.49029	5.57819	4.17768	289 e - - -
	2.68618	4.42225	2.77519	2.73123	3.46354	2.40513	3.72494	3.29354	2.67741	2.69355	4.24690	2.90347	2.73739	3.18146	2.89801	2.37887	2.77519	2.98518	4.58477	3.61503	
	0.02290	4.18373	4.90608	0.61958	0.77255	0.48576	0.95510														
257	2.46482	5.03518	2.89800	2.31151	4.35066	3.21093	3.62718	3.79419	1.91577	3.32424	4.10656	2.92252	3.83492	2.18676	2.78084	2.59605	2.59133	3.41424	5.49436	4.13153	290 k - - -
	2.68618	4.42225	2.77519	2.73123	3.46354	2.40513	3.72494	3.29354	2.67741	2.69355	4.24690	2.90347	2.73739	3.18146	2.89801	2.37887	2.77519	2.98518	4.58477	3.61503	
	0.02290	4.18373	4.90608	0.61958	0.77255	0.48576	0.95510														
258	3.56290	4.88515	4.51719	4.15694	1.42671	4.35236	3.56577	3.43564	4.03102	2.86558	4.03324	3.35459	4.68364	4.07827	4.13055	3.69814	3.78882	3.29820	3.74566	1.05990	291 y - - -
	2.68618	4.42225	2.77519	2.73123	3.46354	2.40513	3.72494	3.29354	2.67741	2.69355	4.24690	2.90347	2.73739	3.18146	2.89801	2.37887	2.77519	2.98518	4.58477	3.61503	
	0.02290	4.18373	4.90608	0.61958	0.77255	0.48576	0.95510														
259	3.22031	4.91301	3.86274	3.75476	4.70463	3.58351	4.79631	4.34360	3.84144	3.93066	5.00116	4.02490	0.41006	4.22005	4.04145	3.39999	3.68818	3.95083	5.79302	4.83303	292 P - - -
	2.68618	4.42225	2.77519	2.73123	3.46354	2.40513	3.72494	3.29354	2.67741	2.69355	4.24690	2.90347	2.73739	3.18146	2.89801	2.37887	2.77519	2.98518	4.58477	3.61503	
	0.02290	4.18373	4.90608	0.61958	0.77255	0.48576	0.95510														
260	2.61841	4.51925	2.95626	2.69043	3.69382	3.52170	3.75057	2.30237	2.51385	2.74470	3.61511	2.99363	3.90898	2.98897	2.62759	2.75496	2.85003	2.26721	5.07696	3.80870	293 v - - -
	2.68618	4.42225	2.77519	2.73123	3.46354	2.40513	3.72494	3.29354	2.67741	2.69355	4.24690	2.90347	2.73739	3.18146	2.89801	2.37887	2.77519	2.98518	4.58477	3.61503	
	0.02290	4.18373	4.90608	0.61958	0.77255	0.48576	0.95510														
261	2.02843	4.26632	3.78329	3.35757	3.99626	3.25020	4.29090	2.97163	3.29255	2.95701	3.91566	3.53224	3.91887	3.60412	3.58843	2.66317	1.28612	2.32903	5.47109	4.26068	294 t - - -
	2.68618	4.42225	2.77519	2.73123	3.46354	2.40513	3.72494	3.29354	2.67741	2.69355	4.24690	2.90347	2.73739	3.18146	2.89801	2.37887	2.77519	2.98518	4.58477	3.61503	
	0.02290	4.18373	4.90608	0.61958	0.77255	0.48576	0.95510														

262	2.97110	4.39540	4.52766	4.02545	3.62655	4.15252	4.74775	1.09628	3.90001	2.26447	3.47497	4.24176	4.55804	4.18843	4.11750	3.52236	2.47287	1.72410	5.42706	4.22469	295 i - - -
	2.68618	4.42225	2.77519	2.73123	3.46354	2.40513	3.72494	3.29354	2.67741	2.69355	4.24690	2.90347	2.73739	3.18146	2.89801	2.37887	2.77519	2.98518	4.58477	3.61503	
	0.02290	4.18373	4.90608	0.61958	0.77255	0.48576	0.95510														
263	3.22031	4.91301	3.86274	3.75476	4.70463	3.58351	4.79631	4.34360	3.84144	3.93066	5.00116	4.02490	0.41006	4.22005	4.04145	3.39999	3.68818	3.95083	5.79302	4.83303	296 P - - -
	2.68618	4.42225	2.77519	2.73123	3.46354	2.40513	3.72494	3.29354	2.67741	2.69355	4.24690	2.90347	2.73739	3.18146	2.89801	2.37887	2.77519	2.98518	4.58477	3.61503	
	0.02290	4.18373	4.90608	0.61958	0.77255	0.48576	0.95510														
264	3.46170	4.77263	5.02135	4.49902	3.17908	4.71252	5.03922	1.90253	4.30604	0.76063	2.99077	4.75424	4.89811	4.45378	4.42015	4.10296	3.69461	2.26529	5.30135	4.18908	297 l - - -
	2.68618	4.42225	2.77519	2.73123	3.46354	2.40513	3.72494	3.29354	2.67741	2.69355	4.24690	2.90347	2.73739	3.18146	2.89801	2.37887	2.77519	2.98518	4.58477	3.61503	
	0.02290	4.18373	4.90608	0.61958	0.77255	0.48576	0.95510														
265	3.17358	5.14304	3.15325	2.92559	4.35195	3.60187	4.08213	4.04395	2.72706	3.51336	4.54452	3.38355	4.16743	0.76699	2.99970	3.21374	3.47600	3.75749	5.57252	4.29319	298 q - - -
	2.68618	4.42225	2.77519	2.73123	3.46354	2.40513	3.72494	3.29354	2.67741	2.69355	4.24690	2.90347	2.73739	3.18146	2.89801	2.37887	2.77519	2.98518	4.58477	3.61503	
	0.02290	4.18373	4.90608	0.61958	0.77255	0.48576	0.95510														
266	1.60334	4.82087	2.91900	2.25377	4.16936	3.40708	3.74595	3.55194	2.31876	3.18082	4.01491	3.01430	3.87729	2.91336	2.94826	2.72044	2.66253	3.22103	5.42849	4.10919	299 a - - -
	2.68618	4.42225	2.77519	2.73123	3.46354	2.40513	3.72494	3.29354	2.67741	2.69355	4.24690	2.90347	2.73739	3.18146	2.89801	2.37887	2.77519	2.98518	4.58477	3.61503	
	0.02290	4.18373	4.90608	0.61958	0.77255	0.48576	0.95510														
267	2.33055	4.28549	3.46653	3.21421	4.46627	1.22126	4.32859	3.88052	3.31033	3.56891	4.38906	3.38457	3.79482	3.59451	3.62769	2.26464	1.99282	3.31214	5.79765	4.58345	300 g - - -
	2.68618	4.42225	2.77519	2.73123	3.46354	2.40513	3.72494	3.29354	2.67741	2.69355	4.24690	2.90347	2.73739	3.18146	2.89801	2.37887	2.77519	2.98518	4.58477	3.61503	
	0.02290	4.18373	4.90608	0.61958	0.77255	0.48576	0.95510														
268	2.61363	4.51347	3.07161	2.68904	3.22963	3.51919	3.74592	3.05815	2.40977	1.97017	3.60716	3.04577	3.90518	2.98621	2.79849	2.75026	2.61681	2.80476	5.06567	3.79679	301 l - - -
	2.68618	4.42225	2.77519	2.73123	3.46354	2.40513	3.72494	3.29354	2.67741	2.69355	4.24690	2.90347	2.73739	3.18146	2.89801	2.37887	2.77519	2.98518	4.58477	3.61503	
	0.02290	4.18373	4.90608	0.61958	0.77255	0.48576	0.95510														
269	2.60471	4.31349	2.93353	2.93118	3.21994	3.58901	3.84193	2.71383	2.88320	2.15598	2.76193	3.33232	3.96685	2.79293	3.23209	2.83531	2.83632	2.39853	4.89059	3.10453	302 l - - -
	2.68618	4.42225	2.77519	2.73123	3.46354	2.40513	3.72494	3.29354	2.67741	2.69355	4.24690	2.90347	2.73739	3.18146	2.89801	2.37887	2.77519	2.98518	4.58477	3.61503	
	0.02290	4.18373	4.90608	0.61958	0.77255	0.48576	0.95510														
270	2.68505	5.03131	2.72166	2.41567	4.33524	3.26449	3.62011	3.77543	2.12022	3.30489	4.09092	2.94186	3.84508	2.59893	1.89183	2.67428	2.91020	3.40334	5.47098	3.33731	303 r - - -
	2.68618	4.42225	2.77519	2.73123	3.46354	2.40513	3.72494	3.29354	2.67741	2.69355	4.24690	2.90347	2.73739	3.18146	2.89801	2.37887	2.77519	2.98518	4.58477	3.61503	
	0.25478	4.18373	1.56221	0.61958	0.77255	0.48576	0.95510														
271	2.26747	4.39542	3.04859	2.68658	4.17203	2.05631	3.88572	3.58877	2.77097	3.22874	4.05102	2.79562	2.77955	3.08857	3.17739	2.48384	2.28866	3.14928	5.47263	4.19195	304 g - - -
	2.68618	4.42225	2.77519	2.73123	3.46354	2.40513	3.72494	3.29354	2.67741	2.69355	4.24690	2.90347	2.73739	3.18146	2.89801	2.37887	2.77519	2.98518	4.58477	3.61503	
	0.02879	3.95775	4.68009	0.61958	0.77255	0.36715	1.17996														
272	2.91470	4.34249	4.38203	3.81236	3.28543	4.07969	4.41369	1.35862	3.68116	1.77582	2.71955	4.05463	3.45603	3.90971	3.87109	3.38822	3.15043	2.11689	5.02978	3.86572	305 i - - -
	2.68618	4.42225	2.77519	2.73123	3.46354	2.40513	3.72494	3.29354	2.67741	2.69355	4.24690	2.90347	2.73739	3.18146	2.89801	2.37887	2.77519	2.98518	4.58477	3.61503	
	0.02290	4.18373	4.90608	0.61958	0.77255	0.48576	0.95510														
273	2.64609	5.04816	2.58512	2.22425	4.35241	3.40601	3.61154	3.80790	1.94958	2.85632	4.10362	2.77535	3.81174	2.56996	2.83302	2.62622	2.49808	3.41726	5.50193	4.11974	306 k - - -
	2.68618	4.42225	2.77519	2.73123	3.46354	2.40513	3.72494	3.29354	2.67741	2.69355	4.24690	2.90347	2.73739	3.18146	2.89801	2.37887	2.77519	2.98518	4.58477	3.61503	
	0.02290	4.18373	4.90608	0.61958	0.77255	0.48576	0.95510														
274	2.38098	4.45273	3.32835	2.79865	3.74718	3.42987	3.83713	3.12083	2.74104	2.57155	3.68554	3.21653	1.92472	3.08939	3.12154	2.47264	2.75493	2.84468	4.71318	3.87470	307 p - - -
	2.68618	4.42225	2.77519	2.73123	3.46354	2.40513	3.72494	3.29354	2.67741	2.69355	4.24690	2.90347	2.73739	3.18146	2.89801	2.37887	2.77519	2.98518	4.58477	3.61503	
	0.02290	4.18373	4.90608	0.61958	0.77255	0.48576	0.95510														
275	2.80424	4.29988	4.09588	3.52113	3.25088	3.89612	4.16500	1.87580	2.93176	1.43492	3.20842	3.79940	4.24078	3.63922	3.55196	3.18960	3.03448	2.29796	4.89077	3.47377	308 l - - -
	2.68618	4.42225	2.77519	2.73123	3.46354	2.40513	3.72494	3.29354	2.67741	2.69355	4.24690	2.90347	2.73739	3.18146	2.89801	2.37887	2.77519	2.98518	4.58477	3.61503	
	0.02290	4.18373	4.90608	0.61958	0.77255	0.48576	0.95510														
276	2.54099	4.97622	2.81843	1.94962	4.26666	3.40400	3.63429	3.55765	2.41251	3.26448	4.05144	2.82239	2.53023	2.66081	2.89065	2.51188	2.81909	3.25377	5.46164	4.09138	309 e - - -
	2.68618	4.42225	2.77519	2.73123	3.46354	2.40513	3.72494	3.29354	2.67741	2.69355	4.24690	2.90347	2.73739	3.18146	2.89801	2.37887	2.77519	2.98518	4.58477	3.61503	
	0.02290	4.18373	4.90608	0.61958	0.77255	0.48576	0.95510														
277	2.34547	4.52672	3.22178	2.54977	3.70235	3.50627	3.73571	2.98058	2.54397	2.65974	2.86765	2.97935	3.08744	2.96709	3.04888	2.73069	2.45062	2.72411	5.08113	3.80721	310 a - - -
	2.68618	4.42225	2.77519	2.73123	3.46354	2.40513	3.72494	3.29354	2.67741	2.69355	4.24690	2.90347	2.73739	3.18146	2.89801	2.37887	2.77519	2.98518	4.58477	3.61503	
	0.02290	4.18373	4.90608	0.61958	0.77255	0.48576	0.95510														
278	2.62514	5.14309	2.56164	1.81572	4.45369	3.38212	3.64310	3.92246	2.37269	3.43437	4.20601	2.85830	2.66908	2.48652	2.92244	2.65954	2.69258	3.51743	5.59614	4.19173	311 e - - -
	2.68628	4.42242	2.77529	2.73127	3.46343	2.40487	3.72512	3.29371	2.67758	2.69372	4.24707	2.90337	2.73743	3.18164	2.89818	2.37895	2.77502	2.98455	4.58494	3.61472	
	0.31824	1.32738	4.90608	1.19659	0.35986	0.48576	0.95510														
279	2.20658	4.99316	2.89100	2.23474	4.28511	3.41142	3.23350	3.73118	2.18889	3.27509	4.05542	2.80334	2.89742	2.67887	2.84563	2.45231	2.86865	3.29957	5.46140	4.09010	318 k - - -
	2.68618	4.42225	2.77519	2.73123	3.46354	2.40513	3.72494	3.29354	2.67741	2.69355	4.24690	2.90347	2.73739	3.18146	2.89801	2.37887	2.77519	2.98518	4.58477	3.61503	
	0.02290	4.18373	4.90608	0.61958	0.77255	0.48576	0.95510														
280	2.30155	4.88073	2.93548	2.26116	4.17491	2.76869	3.50632	3.60572	2.41097	3.18431	3.98284	2.94295	3.24980	2.78669	2.86177	2.37319	2.45467	3.25352	5.40271	4.05094	319 e - - -
	2.68618	4.42225	2.77519	2.73123	3.46354	2.40513	3.72494	3.29354	2.67741	2.69355	4.24690	2.90347	2.73739	3.18146	2.89801	2.37887	2.77519	2.98518	4.58477	3.61503	
	0.02290	4.18373	4.90608	0.61958	0.77255	0.48576	0.95510														
281	2.59607	4.28199	3.53294	2.96396	3.04251	3.59002	3.84993	2.67498	2.90984	2.25699	3.21435	3.35268	3.45749	2.94062	3.16624	2.71128	2.18816	2.38602	4.86941	3.64158	320 t - - -
	2.68618	4.42225	2.77519	2.73123	3.46354	2.40513	3.72494	3.29354	2.67741	2.69355	4.24690	2.90347	2.73739	3.18146	2.89801	2.37887	2.77519	2.98518	4.58477	3.61503	
	0.02290	4.18373	4.90608	0.61958	0.77255	0.48576	0.95510														
282	3.20334	4.49065	5.00580	4.52268	3.71432	4.60239	5.23568	1.42204	4.41680	2.20566	3.50631	4.71836	4.90804	4.68210	4.60546	4.00589	3.47247	0.92100	5.71025	4.49682	321 v - - -
	2.68618	4.42225	2.77519	2.73123	3.46354	2.40513	3.72494	3.29354	2.67741	2.69355	4.24690	2.90347	2.73739	3.18146	2.89801	2.37887	2.77519	2.98518	4.58477	3.61503	
	0.02290	4.18373	4.90608	0.61958	0.77255	0.48576	0.95510														
283	2.42462	5.09118	2.61582	2.18633	4.40634	3.39045	3.60991	3.87409	2.19795	3.38387	4.14524	2.60233	3.80407	2.47729	2.86070	2.38298	2.47049	3.46672	5.54189	4.14410	322 e - - -
	2.68618	4.42225	2.77519	2.73123	3.46354	2.40513	3.72494	3.29354	2.67741	2.69355	4.24690	2.90347	2.73739	3.18146	2.89801	2.37887	2.77519	2.98518	4.58477	3.61503	
	0.02290	4.18373	4.90608	0.61958	0.77255	0.48576	0.95510														
284	1.81372	4.25821	3.97204	3.45329	3.61455	3.62102	4.25551	2.26556	3.37250	2.54580	3.54554	3.70021	4.12604	3.65765	3.64806	2.87731	2.72469	1.51523	5.16841	3.95712	323 v - - -

	2.68618	4.42225	2.77519	2.73123	3.46354	2.40513	3.72494	3.29354	2.67741	2.69355	4.24690	2.90347	2.73739	3.18146	2.89801	2.37887	2.77519	2.98518	4.58477	3.61503	
	0.02290	4.18373	4.90608	0.61958	0.77255	0.48576	0.95510														
285	2.74024	5.21918	2.46139	1.85685	4.55438	3.40168	3.64249	4.02674	1.77600	3.50775	4.27611	2.74741	3.84405	2.75662	2.59493	2.69222	2.97480	3.60738	5.63961	4.24056	324 k - - -
	2.68618	4.42225	2.77519	2.73123	3.46354	2.40513	3.72494	3.29354	2.67741	2.69355	4.24690	2.90347	2.73739	3.18146	2.89801	2.37887	2.77519	2.98518	4.58477	3.61503	
	0.02290	4.18373	4.90608	0.61958	0.77255	0.48576	0.95510														
286	2.63248	4.20692	3.80712	3.24134	3.32600	3.67621	3.98606	2.54803	3.15014	2.15108	3.31476	3.55415	3.43120	3.42869	3.42178	2.95515	2.49618	1.66687	4.83674	3.19429	325 v - - -
	2.68618	4.42225	2.77519	2.73123	3.46354	2.40513	3.72494	3.29354	2.67741	2.69355	4.24690	2.90347	2.73739	3.18146	2.89801	2.37887	2.77519	2.98518	4.58477	3.61503	
	0.02290	4.18373	4.90608	0.61958	0.77255	0.48576	0.95510														
287	2.61187	4.78631	2.76006	2.28231	4.01784	3.44369	3.65309	3.30836	2.41834	2.61920	3.86036	2.71889	3.83595	2.75864	2.92087	2.56495	2.49589	2.66803	5.29466	3.96543	326 e - - -
	2.68618	4.42225	2.77519	2.73123	3.46354	2.40513	3.72494	3.29354	2.67741	2.69355	4.24690	2.90347	2.73739	3.18146	2.89801	2.37887	2.77519	2.98518	4.58477	3.61503	
	0.02290	4.18373	4.90608	0.61958	0.77255	0.48576	0.95510														
288	2.67386	5.13592	2.11757	2.27571	4.45903	2.91911	3.62147	3.93111	2.05851	3.43103	4.19265	2.73433	3.81390	2.73310	2.63211	2.60203	2.68525	3.51678	5.58086	4.17814	327 k - - -
	2.68618	4.42225	2.77519	2.73123	3.46354	2.40513	3.72494	3.29354	2.67741	2.69355	4.24690	2.90347	2.73739	3.18146	2.89801	2.37887	2.77519	2.98518	4.58477	3.61503	
	0.02290	4.18373	4.90608	0.61958	0.77255	0.48576	0.95510														
289	1.04342	4.23095	3.63849	3.34647	4.41164	2.09893	4.38077	3.76750	3.39333	3.50149	4.33273	3.44820	3.79057	3.66611	3.68979	2.48761	2.48571	3.22562	5.76729	4.57442	328 a - - -
	2.68618	4.42225	2.77519	2.73123	3.46354	2.40513	3.72494	3.29354	2.67741	2.69355	4.24690	2.90347	2.73739	3.18146	2.89801	2.37887	2.77519	2.98518	4.58477	3.61503	
	0.02290	4.18373	4.90608	0.61958	0.77255	0.48576	0.95510														
290	2.60498	4.62520	3.09138	2.30598	3.91352	3.42747	3.76038	3.28044	2.61804	2.95413	3.80442	2.93923	3.87350	2.96072	3.04338	2.46420	1.96597	2.43121	5.25096	3.95757	329 t - - -
	2.68618	4.42225	2.77519	2.73123	3.46354	2.40513	3.72494	3.29354	2.67741	2.69355	4.24690	2.90347	2.73739	3.18146	2.89801	2.37887	2.77519	2.98518	4.58477	3.61503	
	0.02290	4.18373	4.90608	0.61958	0.77255	0.48576	0.95510														
291	3.72820	5.08951	4.31383	4.11156	2.37789	4.20534	3.81093	3.69832	3.96130	3.06883	4.34214	4.12878	4.67640	4.18956	4.07623	3.82923	4.01892	3.58482	4.03002	0.57233	330 Y - - -
	2.68618	4.42225	2.77519	2.73123	3.46354	2.40513	3.72494	3.29354	2.67741	2.69355	4.24690	2.90347	2.73739	3.18146	2.89801	2.37887	2.77519	2.98518	4.58477	3.61503	
	0.02290	4.18373	4.90608	0.61958	0.77255	0.48576	0.95510														
292	2.65676	5.06635	2.61279	2.36564	4.38422	3.41963	3.60530	3.83978	2.04380	3.35218	4.12048	2.59143	3.81854	2.71752	2.12512	2.63471	2.67827	3.44397	5.50759	3.77654	331 k - - -
	2.68618	4.42225	2.77519	2.73123	3.46354	2.40513	3.72494	3.29354	2.67741	2.69355	4.24690	2.90347	2.73739	3.18146	2.89801	2.37887	2.77519	2.98518	4.58477	3.61503	
	0.02290	4.18373	4.90608	0.61958	0.77255	0.48576	0.95510														
293	3.06283	4.53251	4.54850	4.23222	3.79962	4.03738	4.98744	2.08998	4.10550	2.43889	3.72731	4.36948	4.58862	4.44783	4.28676	3.56937	3.42179	0.70554	5.63303	4.37970	332 v - - -
	2.68618	4.42225	2.77519	2.73123	3.46354	2.40513	3.72494	3.29354	2.67741	2.69355	4.24690	2.90347	2.73739	3.18146	2.89801	2.37887	2.77519	2.98518	4.58477	3.61503	
	0.02290	4.18373	4.90608	0.61958	0.77255	0.48576	0.95510														
294	2.34949	4.67537	2.99560	2.70673	4.27696	3.29901	3.94119	3.68777	2.74204	3.31256	4.18407	3.13821	1.43008	2.66954	3.10950	2.72265	2.98578	3.30823	5.56651	4.27011	333 p - - -
	2.68618	4.42225	2.77519	2.73123	3.46354	2.40513	3.72494	3.29354	2.67741	2.69355	4.24690	2.90347	2.73739	3.18146	2.89801	2.37887	2.77519	2.98518	4.58477	3.61503	
	0.02290	4.18373	4.90608	0.61958	0.77255	0.48576	0.95510														
295	2.62171	4.94895	2.80634	2.49853	4.39156	1.62245	3.78547	3.81657	2.35066	3.38922	4.22128	3.00162	3.90438	2.35617	2.90671	2.77532	3.03718	3.44615	5.58460	4.25733	334 g - - -
	2.68618	4.42225	2.77519	2.73123	3.46354	2.40513	3.72494	3.29354	2.67741	2.69355	4.24690	2.90347	2.73739	3.18146	2.89801	2.37887	2.77519	2.98518	4.58477	3.61503	
	0.02290	4.18373	4.90608	0.61958	0.77255	0.48576	0.95510														
296	3.39326	5.18728	3.91904	3.37864	4.62317	3.80584	4.10075	4.19699	2.43055	3.65635	4.65808	3.70929	4.30210	3.31412	0.59204	3.46858	3.64356	3.90975	5.58474	4.49376	335 R - - -
	2.68618	4.42225	2.77519	2.73123	3.46354	2.40513	3.72494	3.29354	2.67741	2.69355	4.24690	2.90347	2.73739	3.18146	2.89801	2.37887	2.77519	2.98518	4.58477	3.61503	
	0.02290	4.18373	4.90608	0.61958	0.77255	0.48576	0.95510														
297	1.90253	4.72205	2.96895	2.11403	4.19604	3.32907	3.78300	3.60315	2.61500	3.22347	4.04165	3.02715	3.83676	2.94912	3.06390	2.11472	2.41889	3.23322	5.46481	4.14015	336 a - - -
	2.68618	4.42225	2.77519	2.73123	3.46354	2.40513	3.72494	3.29354	2.67741	2.69355	4.24690	2.90347	2.73739	3.18146	2.89801	2.37887	2.77519	2.98518	4.58477	3.61503	
	0.02290	4.18373	4.90608	0.61958	0.77255	0.48576	0.95510														
298	3.42628	4.74570	5.00699	4.43592	3.11890	4.67918	4.96086	2.15444	4.27631	0.98852	1.70907	4.69717	4.83609	4.36779	4.37908	4.02863	3.64240	2.40879	5.22991	4.18613	337 l - - -
	2.68618	4.42225	2.77519	2.73123	3.46354	2.40513	3.72494	3.29354	2.67741	2.69355	4.24690	2.90347	2.73739	3.18146	2.89801	2.37887	2.77519	2.98518	4.58477	3.61503	
	0.02290	4.18373	4.90608	0.61958	0.77255	0.48576	0.95510														
299	2.61539	4.91133	2.72024	2.39965	4.18343	3.10535	3.62311	3.61734	2.36130	3.09636	3.97886	2.92952	3.81481	2.42220	2.69852	2.31830	2.50862	2.85390	5.39727	4.03995	338 s - - -
	2.68618	4.42225	2.77519	2.73123	3.46354	2.40513	3.72494	3.29354	2.67741	2.69355	4.24690	2.90347	2.73739	3.18146	2.89801	2.37887	2.77519	2.98518	4.58477	3.61503	
	0.02290	4.18373	4.90608	0.61958	0.77255	0.48576	0.95510														
300	2.62038	4.29470	4.54757	3.96118	2.39845	4.08456	4.40036	2.08294	3.82476	1.68331	1.98860	4.12839	4.39242	3.97802	3.94197	3.39447	3.13266	2.11603	4.90482	3.76085	339 l - - -
	2.68618	4.42225	2.77519	2.73123	3.46354	2.40513	3.72494	3.29354	2.67741	2.69355	4.24690	2.90347	2.73739	3.18146	2.89801	2.37887	2.77519	2.98518	4.58477	3.61503	
	0.02290	4.18373	4.90608	0.61958	0.77255	0.48576	0.95510														
301	2.62495	4.99537	2.67552	2.36087	4.28749	3.40689	3.11757	3.73705	2.26378	3.27773	4.05347	2.61681	3.80630	2.51327	2.85040	2.46313	2.37544	2.99013	5.46165	4.08553	340 k - - -
	2.68618	4.42225	2.77519	2.73123	3.46354	2.40513	3.72494	3.29354	2.67741	2.69355	4.24690	2.90347	2.73739	3.18146	2.89801	2.37887	2.77519	2.98518	4.58477	3.61503	
	0.02290	4.18373	4.90608	0.61958	0.77255	0.48576	0.95510														
302	3.01569	4.37187	4.72541	4.14430	2.68843	4.23285	4.53425	1.84231	4.00382	1.29144	3.04641	4.29420	4.51429	4.13042	4.09792	3.55383	3.24327	1.83637	4.96481	3.50665	341 l - - -
	2.68618	4.42225	2.77519	2.73123	3.46354	2.40513	3.72494	3.29354	2.67741	2.69355	4.24690	2.90347	2.73739	3.18146	2.89801	2.37887	2.77519	2.98518	4.58477	3.61503	
	0.02290	4.18373	4.90608	0.61958	0.77255	0.48576	0.95510														
303	2.65383	4.99828	2.92848	2.16763	4.29520	3.42908	3.61901	3.48116	1.99002	3.27654	4.06068	2.92551	3.82754	2.40566	2.78997	2.48109	2.37020	3.36386	5.45787	4.09745	342 k - - -
	2.68618	4.42225	2.77519	2.73123	3.46354	2.40513	3.72494	3.29354	2.67741	2.69355	4.24690	2.90347	2.73739	3.18146	2.89801	2.37887	2.77519	2.98518	4.58477	3.61503	
	0.02290	4.18373	4.90608	0.61958	0.77255	0.48576	0.95510														
304	3.21811	4.49241	5.05209	4.56667	3.71589	4.64339	5.27217	1.22526	4.46548	2.19672	3.50067	4.75925	4.93377	4.72223	4.64808	4.04705	3.48388	1.05048	5.72385	4.51648	343 v - - -
	2.68618	4.42225	2.77519	2.73123	3.46354	2.40513	3.72494	3.29354	2.67741	2.69355	4.24690	2.90347	2.73739	3.18146	2.89801	2.37887	2.77519	2.98518	4.58477	3.61503	
	0.02290	4.18373	4.90608	0.61958	0.77255	0.48576	0.95510														
305	2.71558	4.96398	2.56761	2.43888	4.31177	3.40311	3.72086	3.72017	1.99570	3.31020	4.12920	2.96042	3.87354	2.86982	2.91682	2.72552	1.73543	3.36488	5.51964	4.17212	344 t - - -
	2.68618	4.42225	2.77519	2.73123	3.46354	2.40513	3.72494	3.29354	2.67741	2.69355	4.24690	2.90347	2.73739	3.18146	2.89801	2.37887	2.77519	2.98518	4.58477	3.61503	
	0.02290	4.18373	4.90608	0.61958	0.77255	0.48576	0.95510														
306	3.01823	5.00456	2.93444	2.85747	4.42134	3.42302	4.22356	4.21256	3.16132	3.82609	4.78843	0.70604	4.09507	3.50667	3.51832	3.07955	3.41628	3.80198	5.69101	4.35493	345 n - - -
	2.68618	4.42225	2.77519	2.73123	3.46354	2.40513	3.72494	3.29354	2.67741	2.69355	4.24690	2.90347	2.73739	3.18146	2.89801	2.37887	2.77519	2.98518	4.58477	3.61503	

	0.02290	4.18373	4.90608	0.61958	0.77255	0.48576	0.95510													
307	2.62430	4.91896	2.96059	2.40849	4.19342	2.78499	3.35789	3.43984	2.17904	3.19275	3.98540	2.37728	3.82048	2.75319	2.65215	2.53716	2.77876	2.99980	5.39924	4.04559	346 k - - -
	2.68618	4.42225	2.77519	2.73123	3.46354	2.40513	3.72494	3.29354	2.67741	2.69355	4.24690	2.90347	2.73739	3.18146	2.89801	2.37887	2.77519	2.98518	4.58477	3.61503	
	0.02290	4.18373	4.90608	0.61958	0.77255	0.48576	0.95510														
308	2.32590	4.29277	3.46618	3.17269	4.45871	1.40476	4.28725	3.88743	3.25552	3.55552	4.36606	3.36244	3.78650	3.53878	3.59174	1.81503	2.13845	3.31638	5.78005	4.55931	347 g - - -
	2.68618	4.42225	2.77519	2.73123	3.46354	2.40513	3.72494	3.29354	2.67741	2.69355	4.24690	2.90347	2.73739	3.18146	2.89801	2.37887	2.77519	2.98518	4.58477	3.61503	
	0.02290	4.18373	4.90608	0.61958	0.77255	0.48576	0.95510														
309	2.77107	5.26195	1.71846	2.26678	4.57686	3.36137	3.68789	4.06012	2.33189	3.55831	4.33755	2.42482	3.84814	2.56535	3.02715	2.45049	2.83683	3.64177	5.71277	4.28409	348 d - - -
	2.68618	4.42225	2.77519	2.73123	3.46354	2.40513	3.72494	3.29354	2.67741	2.69355	4.24690	2.90347	2.73739	3.18146	2.89801	2.37887	2.77519	2.98518	4.58477	3.61503	
	0.02290	4.18373	4.90608	0.61958	0.77255	0.48576	0.95510														
310	2.66044	5.12330	2.70433	2.06020	4.45227	3.07326	3.60533	3.92271	2.01485	3.41670	4.17380	2.87252	3.80977	2.51119	2.56779	2.28374	2.89241	3.50642	5.55948	4.16334	349 k - - -
	2.68618	4.42225	2.77519	2.73123	3.46354	2.40513	3.72494	3.29354	2.67741	2.69355	4.24690	2.90347	2.73739	3.18146	2.89801	2.37887	2.77519	2.98518	4.58477	3.61503	
	0.02290	4.18373	4.90608	0.61958	0.77255	0.48576	0.95510														
311	2.11478	4.59440	3.15021	2.68008	4.08433	3.33352	3.83089	3.47136	2.39957	3.12083	3.95752	3.12017	1.93972	3.02137	3.05494	2.47768	2.86576	2.94192	5.38699	4.09800	350 p - - -
	2.68618	4.42225	2.77519	2.73123	3.46354	2.40513	3.72494	3.29354	2.67741	2.69355	4.24690	2.90347	2.73739	3.18146	2.89801	2.37887	2.77519	2.98518	4.58477	3.61503	
	0.02290	4.18373	4.90608	0.61958	0.77255	0.48576	0.95510														
312	2.88242	4.33361	4.25918	3.69648	3.37848	4.03683	4.37020	1.91240	3.56052	1.92575	3.28265	3.97029	4.37552	3.20703	3.78444	3.34164	3.12074	1.32027	5.07291	3.88179	351 v - - -
	2.68618	4.42225	2.77519	2.73123	3.46354	2.40513	3.72494	3.29354	2.67741	2.69355	4.24690	2.90347	2.73739	3.18146	2.89801	2.37887	2.77519	2.98518	4.58477	3.61503	
	0.02290	4.18373	4.90608	0.61958	0.77255	0.48576	0.95510														
313	2.70119	4.83266	3.14378	2.56522	4.07470	3.51353	3.66552	3.48066	2.29331	3.07898	3.91143	3.05723	3.89686	2.43078	1.87362	2.64559	2.92208	2.77861	5.30448	3.38044	352 r - - -
	2.68618	4.42225	2.77519	2.73123	3.46354	2.40513	3.72494	3.29354	2.67741	2.69355	4.24690	2.90347	2.73739	3.18146	2.89801	2.37887	2.77519	2.98518	4.58477	3.61503	
	0.02290	4.18373	4.90608	0.61958	0.77255	0.48576	0.95510														
314	3.39833	4.69017	5.07713	4.53302	3.24463	4.71869	5.08403	1.64989	4.38470	0.90886	3.02890	4.77246	4.89796	4.50127	4.49538	4.09024	3.62955	2.03756	5.35599	4.27589	353 l - - -
	2.68618	4.42225	2.77519	2.73123	3.46354	2.40513	3.72494	3.29354	2.67741	2.69355	4.24690	2.90347	2.73739	3.18146	2.89801	2.37887	2.77519	2.98518	4.58477	3.61503	
	0.02290	4.18373	4.90608	0.61958	0.77255	0.48576	0.95510														
315	2.54344	4.26844	3.16498	2.66023	4.15609	1.70566	3.77932	3.55312	2.13323	3.16925	4.00186	3.11114	3.87760	2.95237	2.70321	2.69784	2.90967	3.20452	5.40182	4.11663	354 g - - -
	2.68618	4.42225	2.77519	2.73123	3.46354	2.40513	3.72494	3.29354	2.67741	2.69355	4.24690	2.90347	2.73739	3.18146	2.89801	2.37887	2.77519	2.98518	4.58477	3.61503	
	0.02290	4.18373	4.90608	0.61958	0.77255	0.48576	0.95510														
316	2.81646	5.21390	2.66816	1.38025	4.54312	2.97231	3.70055	3.99784	2.42858	3.50700	4.30943	2.89904	3.89009	2.65166	2.55663	2.77480	3.05832	3.60855	5.65825	4.28034	355 e - - -
	2.68618	4.42225	2.77519	2.73123	3.46354	2.40513	3.72494	3.29354	2.67741	2.69355	4.24690	2.90347	2.73739	3.18146	2.89801	2.37887	2.77519	2.98518	4.58477	3.61503	
	0.02290	4.18373	4.90608	0.61958	0.77255	0.48576	0.95510														
317	3.79615	5.00615	4.79879	4.51600	0.79258	4.53336	3.60083	3.40393	4.35166	2.69741	3.99888	4.22760	4.84046	4.29324	4.35614	3.91936	4.02068	3.35764	3.72941	1.69547	356 f - - -
	2.68618	4.42225	2.77519	2.73123	3.46354	2.40513	3.72494	3.29354	2.67741	2.69355	4.24690	2.90347	2.73739	3.18146	2.89801	2.37887	2.77519	2.98518	4.58477	3.61503	
	0.02290	4.18373	4.90608	0.61958	0.77255	0.48576	0.95510														
318	2.43092	4.53145	3.21677	2.65585	3.70592	3.50555	3.73292	2.84075	2.62949	2.51129	3.42599	2.51531	3.89113	2.72354	3.04568	2.55418	2.55910	2.82590	5.08330	3.40532	357 a - - -
	2.68618	4.42225	2.77519	2.73123	3.46354	2.40513	3.72494	3.29354	2.67741	2.69355	4.24690	2.90347	2.73739	3.18146	2.89801	2.37887	2.77519	2.98518	4.58477	3.61503	
	0.02290	4.18373	4.90608	0.61958	0.77255	0.48576	0.95510														
319	1.96604	4.24673	3.64698	3.32408	4.28685	3.08000	4.33111	3.56225	3.31563	3.35470	4.21689	3.45303	3.81791	3.62186	3.60709	2.31584	1.10787	3.10120	5.66772	4.46449	358 t - - -
	2.68618	4.42225	2.77519	2.73123	3.46354	2.40513	3.72494	3.29354	2.67741	2.69355	4.24690	2.90347	2.73739	3.18146	2.89801	2.37887	2.77519	2.98518	4.58477	3.61503	
	0.02290	4.18373	4.90608	0.61958	0.77255	0.48576	0.95510														
320	1.05750	4.33232	3.21329	3.06823	4.37068	2.52933	4.22372	3.78176	3.20594	3.46024	4.28452	3.31685	3.80261	3.47511	3.56424	2.22931	2.83038	3.26745	5.70718	4.47140	359 a - - -
	2.68618	4.42225	2.77519	2.73123	3.46354	2.40513	3.72494	3.29354	2.67741	2.69355	4.24690	2.90347	2.73739	3.18146	2.89801	2.37887	2.77519	2.98518	4.58477	3.61503	
	0.02290	4.18373	4.90608	0.61958	0.77255	0.48576	0.95510														
321	2.53521	4.65234	2.90602	2.63653	4.47914	1.94901	3.96041	3.93578	2.84393	3.52990	4.33539	1.83135	3.82734	3.13964	3.28589	2.12641	2.76983	3.44679	5.72718	4.40157	360 n - - -
	2.68618	4.42225	2.77519	2.73123	3.46354	2.40513	3.72494	3.29354	2.67741	2.69355	4.24690	2.90347	2.73739	3.18146	2.89801	2.37887	2.77519	2.98518	4.58477	3.61503	
	0.02290	4.18373	4.90608	0.61958	0.77255	0.48576	0.95510														
322	3.01599	4.39315	4.60747	4.06862	3.48181	4.02081	4.69833	1.89444	3.93490	1.64939	3.35104	4.28825	4.59022	4.18803	4.12569	3.59982	3.26975	1.15749	5.28804	4.09123	361 v - - -
	2.68618	4.42225	2.77519	2.73123	3.46354	2.40513	3.72494	3.29354	2.67741	2.69355	4.24690	2.90347	2.73739	3.18146	2.89801	2.37887	2.77519	2.98518	4.58477	3.61503	
	0.02290	4.18373	4.90608	0.61958	0.77255	0.48576	0.95510														
323	2.56526	5.06319	3.20931	2.63331	4.40391	3.58179	3.13925	3.80920	2.15077	3.32710	4.15688	3.10524	3.97030	2.54341	1.46030	2.81614	3.07029	3.47427	5.45807	4.19103	362 r - - -
	2.68618	4.42225	2.77519	2.73123	3.46354	2.40513	3.72494	3.29354	2.67741	2.69355	4.24690	2.90347	2.73739	3.18146	2.89801	2.37887	2.77519	2.98518	4.58477	3.61503	
	0.02290	4.18373	4.90608	0.61958	0.77255	0.48576	0.95510														
324	3.68410	4.99736	4.66912	4.45276	0.61317	4.27914	4.05653	3.18184	4.35515	2.48406	3.87144	4.38573	4.73817	4.43916	4.39718	3.93054	3.98917	3.19377	4.23206	2.56778	363 F - - -
	2.68618	4.42225	2.77519	2.73123	3.46354	2.40513	3.72494	3.29354	2.67741	2.69355	4.24690	2.90347	2.73739	3.18146	2.89801	2.37887	2.77519	2.98518	4.58477	3.61503	
	0.02290	4.18373	4.90608	0.61958	0.77255	0.48576	0.95510														
325	2.98029	4.49203	4.19894	3.61504	3.22586	4.04435	4.29338	2.49139	3.33871	1.10204	2.48947	3.92202	4.36267	3.69688	3.01945	3.34989	3.20772	2.50085	5.00810	3.84611	364 l - - -
	2.68618	4.42225	2.77519	2.73123	3.46354	2.40513	3.72494	3.29354	2.67741	2.69355	4.24690	2.90347	2.73739	3.18146	2.89801	2.37887	2.77519	2.98518	4.58477	3.61503	
	0.02290	4.18373	4.90608	0.61958	0.77255	0.48576	0.95510														
326	3.00464	5.55758	1.64332	2.15440	4.87786	3.19516	3.85134	4.40603	2.85886	3.90068	4.74207	1.40852	3.92698	3.01534	3.44989	2.89736	3.29707	3.96808	6.05527	4.55041	365 n - - -
	2.68618	4.42225	2.77519	2.73123	3.46354	2.40513	3.72494	3.29354	2.67741	2.69355	4.24690	2.90347	2.73739	3.18146	2.89801	2.37887	2.77519	2.98518	4.58477	3.61503	
	0.02290	4.18373	4.90608	0.61958	0.77255	0.48576	0.95510														
327	2.00902	4.56552	3.09432	2.73001	4.22443	3.25229	3.93128	3.63656	2.78147	3.27367	4.10772	3.14270	1.71084	2.82025	3.17991	2.39838	2.88919	3.23465	5.52229	4.23404	366 p - - -
	2.68618	4.42225	2.77519	2.73123	3.46354	2.40513	3.72494	3.29354	2.67741	2.69355	4.24690	2.90347	2.73739	3.18146	2.89801	2.37887	2.77519	2.98518	4.58477	3.61503	
	0.02290	4.18373	4.90608	0.61958	0.77255	0.48576	0.95510														
328	2.27489	5.15890	1.75518	2.25199	4.49274	3.35531	3.70456	3.96026	2.50982	3.48878	4.27859	2.59365	3.84710	2.83811	3.05947	2.41802	2.79427	3.55808	5.66676	4.25516	367 d - - -
	2.68618	4.42225	2.77519	2.73123	3.46354	2.40513	3.72494	3.29354	2.67741	2.69355	4.24690	2.90347	2.73739	3.18146	2.89801	2.37887	2.77519	2.98518	4.58477	3.61503	
	0.02290	4.18373	4.90608	0.61958	0.77255	0.48576	0.95510														

```
329   2.64059  4.39480  4.67449  4.13702  3.57067  4.32787  4.80062  1.75581  4.03043  1.86975  3.41034  4.36105  4.65141  4.27802  4.23069  3.67338  3.29394  1.10553  5.39377  4.20301    368 v - - -
      2.68618  4.42225  2.77519  2.73123  3.46354  2.40513  3.72494  3.29354  2.67741  2.69355  4.24690  2.90347  2.73739  3.18146  2.89801  2.37887  2.77519  2.98518  4.58477  3.61503
      0.07307  4.18373  2.89630  0.61958  0.77255  0.48576  0.95510
330   2.42641  4.08134  3.43481  2.86874  2.96199  3.55652  3.37942  2.83053  2.82144  2.44868  3.42986  3.28113  2.76081  3.12946  3.03809  2.72702  2.81905  2.60300  4.89041  2.90044    369 a - - -
      2.68618  4.42225  2.77519  2.73123  3.46354  2.40513  3.72494  3.29354  2.67741  2.69355  4.24690  2.90347  2.73739  3.18146  2.89801  2.37887  2.77519  2.98518  4.58477  3.61503
      0.02407  4.13473  4.85707  0.61958  0.77255  0.54367  0.86896
331   2.44957  4.99082  2.73853  2.12339  4.28402  2.96089  3.60615  3.73405  2.26397  3.27607  4.05377  2.69712  3.79702  2.66534  2.85987  2.55541  2.42665  3.14376  5.46183  4.08361    370 e - - -
      2.68618  4.42225  2.77519  2.73123  3.46354  2.40513  3.72494  3.29354  2.67741  2.69355  4.24690  2.90347  2.73739  3.18146  2.89801  2.37887  2.77519  2.98518  4.58477  3.61503
      0.02407  4.13473  4.85707  0.61958  0.77255  0.54367  0.86896
332   2.54167  5.04161  2.18549  2.32208  4.34021  3.37950  3.30321  3.79729  2.39595  3.32998  4.10642  2.39400  3.80273  2.54021  2.88447  2.62467  2.70195  3.06795  5.50781  4.11960    371 d - - -
      2.68618  4.42225  2.77519  2.73123  3.46354  2.40513  3.72494  3.29354  2.67741  2.69355  4.24690  2.90347  2.73739  3.18146  2.89801  2.37887  2.77519  2.98518  4.58477  3.61503
      0.02407  4.13473  4.85707  0.61958  0.77255  0.54367  0.86896
333   2.68113  5.16529  2.35228  2.06253  4.48684  3.23003  3.61855  3.96528  2.06596  3.45880  4.22050  2.59375  3.80463  2.50976  2.88927  2.63701  2.51435  3.54483  5.60516  4.19217    372 e - - -
      2.68617  4.42231  2.77525  2.73129  3.46360  2.40519  3.72500  3.29360  2.67739  2.69361  4.24696  2.90325  2.73738  3.18152  2.89807  2.37887  2.77525  2.98518  4.58268  3.61509
      0.22288  1.65019  4.85707  0.66898  0.71791  0.54367  0.86896
334   2.46600  4.53591  3.18451  2.55694  3.81042  3.42417  3.75061  3.18007  2.62618  2.68780  3.71284  3.10914  2.71504  2.96494  3.04452  2.37968  2.27378  2.67225  5.16798  3.88710    375 t - - -
      2.68618  4.42225  2.77519  2.73123  3.46354  2.40513  3.72494  3.29354  2.67741  2.69355  4.24690  2.90347  2.73739  3.18146  2.89801  2.37887  2.77519  2.98518  4.58477  3.61503
      0.02407  4.13473  4.85707  0.61958  0.77255  0.54367  0.86896
335   2.69535  5.09967  2.13949  2.20836  4.42606  2.37720  3.66544  3.88975  2.48746  3.42083  4.20588  2.46259  3.81984  2.79497  2.99248  2.51751  2.81763  3.22672  5.59981  4.19713    376 d - - -
      2.68618  4.42225  2.77519  2.73123  3.46354  2.40513  3.72494  3.29354  2.67741  2.69355  4.24690  2.90347  2.73739  3.18146  2.89801  2.37887  2.77519  2.98518  4.58477  3.61503
      0.02407  4.13473  4.85707  0.61958  0.77255  0.45118  1.01301
336   2.69790  4.39416  3.53944  3.00579  2.30987  3.64880  3.79074  2.90370  2.67751  2.59124  3.50803  3.08655  4.03309  3.24911  3.27162  2.62943  2.93125  2.68393  4.64822  2.10367    377 y - - -
      2.68618  4.42225  2.77519  2.73123  3.46354  2.40513  3.72494  3.29354  2.67741  2.69355  4.24690  2.90347  2.73739  3.18146  2.89801  2.37887  2.77519  2.98518  4.58477  3.61503
      0.02290  4.18373  4.90608  0.61958  0.77255  0.48576  0.95510
337   2.43599  4.78894  2.41131  2.56539  4.44544  3.25776  3.97443  3.85677  2.91080  3.50306  4.35775  3.04357  1.32537  3.17178  3.36864  2.73429  3.04196  3.44140  5.72919  4.38866    378 p - - -
      2.68618  4.42225  2.77519  2.73123  3.46354  2.40513  3.72494  3.29354  2.67741  2.69355  4.24690  2.90347  2.73739  3.18146  2.89801  2.37887  2.77519  2.98518  4.58477  3.61503
      0.02290  4.18373  4.90608  0.61958  0.77255  0.48576  0.95510
338   3.09065  5.71624  1.29733  1.37565  4.99894  3.31126  3.88305  4.53936  2.93596  4.01996  4.88172  2.76758  3.95056  3.05234  3.55867  2.95546  3.38520  4.09563  6.16247  4.63162    379 d - - -
      2.68618  4.42225  2.77519  2.73123  3.46354  2.40513  3.72494  3.29354  2.67741  2.69355  4.24690  2.90347  2.73739  3.18146  2.89801  2.37887  2.77519  2.98518  4.58477  3.61503
      0.21101  4.18373  1.74302  0.61958  0.77255  0.48576  0.95510
339   2.67503  4.93839  2.04902  2.23006  4.13784  3.33828  3.65448  3.62615  2.51542  3.21422  4.03905  2.85293  3.81023  2.81779  3.00218  2.30735  2.92344  3.28720  5.41077  2.80292    380 d - - -
      2.68618  4.42225  2.77519  2.73123  3.46354  2.40513  3.72494  3.29354  2.67741  2.69355  4.24690  2.90347  2.73739  3.18146  2.89801  2.37887  2.77519  2.98518  4.58477  3.61503
      0.02758  4.00031  4.72265  0.61958  0.77255  0.67548  0.71113
340   2.81890  4.39075  3.92814  3.35491  3.27352  3.85334  4.12454  2.32350  3.11617  1.35732  3.18477  3.69311  4.20630  3.49647  2.90184  3.15116  3.05595  2.18784  4.97075  3.76808    381 l - - -
      2.68618  4.42225  2.77519  2.73123  3.46354  2.40513  3.72494  3.29354  2.67741  2.69355  4.24690  2.90347  2.73739  3.18146  2.89801  2.37887  2.77519  2.98518  4.58477  3.61503
      0.02758  4.00031  4.72265  0.61958  0.77255  0.38314  1.14482
341   3.27403  4.58078  4.96471  4.40770  3.29986  4.59329  4.97010  1.91342  4.28486  1.08248  2.93020  4.63545  4.80733  4.42005  4.41343  3.94421  3.50576  1.53896  5.32955  4.23801    382 l - - -
      2.68618  4.42225  2.77519  2.73123  3.46354  2.40513  3.72494  3.29354  2.67741  2.69355  4.24690  2.90347  2.73739  3.18146  2.89801  2.37887  2.77519  2.98518  4.58477  3.61503
      0.02290  4.18373  4.90608  0.61958  0.77255  0.48576  0.95510
342   1.60164  4.76080  2.93077  2.55029  4.25590  3.33256  3.80038  3.67465  2.37751  3.28100  4.10256  2.34651  3.85529  2.96854  3.02950  2.43691  2.92472  3.29745  5.50719  4.18511    383 a - - -
      2.68618  4.42225  2.77519  2.73123  3.46354  2.40513  3.72494  3.29354  2.67741  2.69355  4.24690  2.90347  2.73739  3.18146  2.89801  2.37887  2.77519  2.98518  4.58477  3.61503
      0.07307  4.18373  2.89630  0.61958  0.77255  0.48576  0.95510
343   2.79290  5.32280  2.03480  1.78069  4.63314  2.49058  3.69113  4.12811  2.38921  3.61584  4.39796  2.52808  3.49496  2.81978  3.08245  2.72214  3.04618  3.69950  5.76597  4.31730    384 e - - -
      2.68618  4.42225  2.77519  2.73123  3.46354  2.40513  3.72494  3.29354  2.67741  2.69355  4.24690  2.90347  2.73739  3.18146  2.89801  2.37887  2.77519  2.98518  4.58477  3.61503
      0.02407  4.13473  4.85707  0.61958  0.77255  0.54367  0.86896
344   2.70548  5.17072  2.49132  1.85426  4.48958  2.61048  3.63399  3.96238  2.35001  3.46217  4.23331  2.68261  3.81824  2.75197  2.37659  2.66183  2.94479  3.55026  5.61318  4.20605    385 e - - -
      2.68618  4.42225  2.77519  2.73123  3.46354  2.40513  3.72494  3.29354  2.67741  2.69355  4.24690  2.90347  2.73739  3.18146  2.89801  2.37887  2.77519  2.98518  4.58477  3.61503
      0.02407  4.13473  4.85707  0.61958  0.77255  0.54367  0.86896
345   2.49778  4.77691  2.72823  2.23017  4.39113  1.50382  3.87965  3.81801  2.76566  3.42748  4.26022  2.99143  3.12711  3.06324  3.21376  2.68944  2.98236  3.40591  5.65122  4.31512    386 g - - -
      2.68618  4.42225  2.77519  2.73123  3.46354  2.40513  3.72494  3.29354  2.67741  2.69355  4.24690  2.90347  2.73739  3.18146  2.89801  2.37887  2.77519  2.98518  4.58477  3.61503
      0.02407  4.13473  4.85707  0.61958  0.77255  0.45118  1.01301
346   3.42800  4.72786  5.06884  4.49957  3.16097  4.72320  5.03426  1.98960  4.36517  0.81360  2.56837  4.75590  4.87176  4.43581  4.46302  4.07639  3.64597  2.18767  5.28544  4.25484    387 l - - -
      2.68618  4.42225  2.77519  2.73123  3.46354  2.40513  3.72494  3.29354  2.67741  2.69355  4.24690  2.90347  2.73739  3.18146  2.89801  2.37887  2.77519  2.98518  4.58477  3.61503
      0.05585  4.18373  3.24227  0.61958  0.77255  0.48576  0.95510
347   2.62874  4.87250  2.96611  2.27276  4.13455  3.43033  3.63259  3.12799  2.14927  3.13990  3.94581  2.95123  3.82779  2.56807  2.84158  2.18297  2.48375  3.21832  5.36239  4.02204    388 k - - -
      2.68618  4.42225  2.77519  2.73123  3.46354  2.40513  3.72494  3.29354  2.67741  2.69355  4.24690  2.90347  2.73739  3.18146  2.89801  2.37887  2.77519  2.98518  4.58477  3.61503
      0.06553  4.15155  3.04308  0.61958  0.77255  0.52449  0.89614
348   3.17129  4.47653  4.87432  4.34337  3.46701  4.52655  4.98801  1.68957  4.22693  1.57943  3.27255  4.57025  4.78860  4.44221  4.40091  3.89035  3.41887  1.13971  5.44332  4.27888    389 v - - -
      2.68618  4.42225  2.77519  2.73123  3.46354  2.40513  3.72494  3.29354  2.67741  2.69355  4.24690  2.90347  2.73739  3.18146  2.89801  2.37887  2.77519  2.98518  4.58477  3.61503
      0.02466  4.11068  4.83303  0.61958  0.77255  0.43651  1.03928
349   2.67007  5.04050  2.49565  2.20486  4.34572  3.38777  3.65096  3.79772  2.37158  3.34056  4.12492  2.75104  3.82493  2.77796  2.93216  1.88278  2.50524  3.30419  5.52775  4.14341    390 s - - -
      2.68618  4.42225  2.77519  2.73123  3.46354  2.40513  3.72494  3.29354  2.67741  2.69355  4.24690  2.90347  2.73739  3.18146  2.89801  2.37887  2.77519  2.98518  4.58477  3.61503
      0.02290  4.18373  4.90608  0.61958  0.77255  0.48576  0.95510
350   2.86180  5.35908  1.64914  2.23916  4.68009  3.33498  3.76090  4.17800  2.66184  3.68081  4.48447  2.28055  2.31971  2.74087  3.20372  2.78935  3.12940  3.75602  5.84139  4.38741    391 d - - -
      2.68618  4.42225  2.77519  2.73123  3.46354  2.40513  3.72494  3.29354  2.67741  2.69355  4.24690  2.90347  2.73739  3.18146  2.89801  2.37887  2.77519  2.98518  4.58477  3.61503
      0.02290  4.18373  4.90608  0.61958  0.77255  0.48576  0.95510
351   2.43345  5.07565  2.49269  2.33096  4.42501  3.36339  3.67983  3.88736  2.49082  3.41998  4.20314  1.96080  3.17412  2.69181  2.98831  2.29525  2.94468  3.48723  5.59837  4.20258    392 n - - -
```

```
        2.68618  4.42225  2.77519  2.73123  3.46354  2.40513  3.72494  3.29354  2.67741  2.69355  4.24690  2.90347  2.73739  3.18146  2.89801  2.37887  2.77519  2.98518  4.58477  3.61503
        0.02290  4.18373  4.90608  0.61958  0.77255  0.48576  0.95510
352     2.50978  4.98314  2.55623  2.05556  4.30296  3.05009  3.64351  3.75066  2.42986  3.30042  4.08314  2.90221  3.81484  2.76968  2.91534  2.12317  2.57948  3.11871  5.49201  4.11548    393 e - - -
        2.68618  4.42225  2.77519  2.73123  3.46354  2.40513  3.72494  3.29354  2.67741  2.69355  4.24690  2.90347  2.73739  3.18146  2.89801  2.37887  2.77519  2.98518  4.58477  3.61503
        0.04633  4.18373  3.50543  0.61958  0.77255  0.48576  0.95510
353     2.56800  4.44405  3.55565  3.28413  4.09839  3.26534  4.31567  3.38222  3.24607  3.12301  4.16277  3.51441  1.02171  3.62523  3.52640  2.75555  3.01567  2.67501  5.55092  4.28875    394 p - - -
        2.68618  4.42225  2.77519  2.73123  3.46354  2.40513  3.72494  3.29354  2.67741  2.69355  4.24690  2.90347  2.73739  3.18146  2.89801  2.37887  2.77519  2.98518  4.58477  3.61503
        0.02344  4.16084  4.88319  0.61958  0.77255  0.46875  0.98292
354     3.32864  4.61633  5.01759  4.51088  3.39831  4.67247  5.10595  1.01746  4.34538  1.51572  3.21653  4.73539  4.90608  4.55475  4.48892  4.06679  3.57561  1.88054  5.45541  4.28651    395 i - - -
        2.68618  4.42225  2.77519  2.73123  3.46354  2.40513  3.72494  3.29354  2.67741  2.69355  4.24690  2.90347  2.73739  3.18146  2.89801  2.37887  2.77519  2.98518  4.58477  3.61503
        0.02290  4.18373  4.90608  0.61958  0.77255  0.48576  0.95510
355     1.86209  4.80759  2.97172  2.42179  4.07064  3.43323  3.68112  3.48136  2.47818  2.80209  3.91441  2.66101  3.35006  2.55326  2.92292  2.67880  2.87949  3.16043  5.33945  4.01253    396 a - - -
        2.68618  4.42225  2.77519  2.73123  3.46354  2.40513  3.72494  3.29354  2.67741  2.69355  4.24690  2.90347  2.73739  3.18146  2.89801  2.37887  2.77519  2.98518  4.58477  3.61503
        0.02290  4.18373  4.90608  0.61958  0.77255  0.48576  0.95510
356     2.32491  4.47660  3.43537  3.26292  4.42799  3.20452  4.40083  3.79509  3.34490  3.51065  4.46597  3.49352  0.88247  3.69702  3.62660  2.73706  3.04615  3.35243  5.76527  4.56081    397 p - - -
        2.68618  4.42225  2.77519  2.73123  3.46354  2.40513  3.72494  3.29354  2.67741  2.69355  4.24690  2.90347  2.73739  3.18146  2.89801  2.37887  2.77519  2.98518  4.58477  3.61503
        0.02290  4.18373  4.90608  0.61958  0.77255  0.48576  0.95510
357     3.09995  4.83262  3.83645  3.78085  4.89495  0.37791  4.87055  4.58175  4.00380  4.19229  5.17870  4.00221  4.23262  4.30392  4.19473  3.28255  3.60515  4.04968  5.89622  5.01130    398 G - - -
        2.68618  4.42225  2.77519  2.73123  3.46354  2.40513  3.72494  3.29354  2.67741  2.69355  4.24690  2.90347  2.73739  3.18146  2.89801  2.37887  2.77519  2.98518  4.58477  3.61503
        0.02290  4.18373  4.90608  0.61958  0.77255  0.48576  0.95510
358     2.93744  5.35688  2.48412  1.17641  4.66779  3.38682  3.78263  4.13479  2.57925  3.64620  4.48928  2.87256  3.93314  2.40125  3.02418  2.87151  3.11011  3.74954  5.80771  4.39886    399 e - - -
        2.68618  4.42225  2.77519  2.73123  3.46354  2.40513  3.72494  3.29354  2.67741  2.69355  4.24690  2.90347  2.73739  3.18146  2.89801  2.37887  2.77519  2.98518  4.58477  3.61503
        0.02290  4.18373  4.90608  0.61958  0.77255  0.48576  0.95510
359     2.57653  4.61666  3.09784  2.70096  4.15930  3.31489  3.87567  3.56002  2.68113  3.20151  4.04624  3.13237  3.86779  2.50831  3.06454  2.24730  1.53756  3.19486  5.45454  4.16418    400 t - - -
        2.68618  4.42225  2.77519  2.73123  3.46354  2.40513  3.72494  3.29354  2.67741  2.69355  4.24690  2.90347  2.73739  3.18146  2.89801  2.37887  2.77519  2.98518  4.58477  3.61503
        0.02290  4.18373  4.90608  0.61958  0.77255  0.48576  0.95510
360     2.95112  5.08869  3.42034  2.75891  4.46183  3.66349  3.67498  3.80736  1.52588  3.32802  4.18184  3.19601  4.03114  2.81288  1.60468  2.96668  3.14117  2.82868  5.45705  4.23649    401 k - - -
        2.68618  4.42225  2.77519  2.73123  3.46354  2.40513  3.72494  3.29354  2.67741  2.69355  4.24690  2.90347  2.73739  3.18146  2.89801  2.37887  2.77519  2.98518  4.58477  3.61503
        0.02290  4.18373  4.90608  0.61958  0.77255  0.48576  0.95510
361     2.68818  4.90047  2.67847  2.18530  4.17779  3.41007  3.70835  3.58481  2.52839  3.19391  4.01899  2.95307  3.86264  2.73020  2.98873  2.70926  1.70588  3.07873  5.43619  4.09119    402 t - - -
        2.68618  4.42225  2.77519  2.73123  3.46354  2.40513  3.72494  3.29354  2.67741  2.69355  4.24690  2.90347  2.73739  3.18146  2.89801  2.37887  2.77519  2.98518  4.58477  3.61503
        0.02290  4.18373  4.90608  0.61958  0.77255  0.48576  0.95510
362     3.06970  4.40049  4.80166  4.23505  3.32674  4.35446  4.72786  1.65465  4.10906  1.60370  2.76417  4.41165  4.62889  4.26992  4.23215  3.68728  3.30381  1.38558  5.18451  3.85070    403 v - - -
        2.68618  4.42225  2.77519  2.73123  3.46354  2.40513  3.72494  3.29354  2.67741  2.69355  4.24690  2.90347  2.73739  3.18146  2.89801  2.37887  2.77519  2.98518  4.58477  3.61503
        0.02290  4.18373  4.90608  0.61958  0.77255  0.48576  0.95510
363     2.49527  5.00999  2.63520  2.02592  4.30689  3.40292  3.20769  3.76066  2.36525  3.08363  4.06541  2.85374  3.80154  2.56325  2.84877  2.43093  2.60432  3.16585  5.47269  4.09148    404 e - - -
        2.68618  4.42225  2.77519  2.73123  3.46354  2.40513  3.72494  3.29354  2.67741  2.69355  4.24690  2.90347  2.73739  3.18146  2.89801  2.37887  2.77519  2.98518  4.58477  3.61503
        0.02290  4.18373  4.90608  0.61958  0.77255  0.48576  0.95510
364     3.18155  4.47170  4.95781  4.43255  3.59455  4.57348  5.08191  1.52692  4.32553  2.09522  2.99598  4.63979  4.84429  4.55123  4.49898  3.94315  3.43277  0.98539  5.54976  4.37411    405 v - - -
        2.68618  4.42225  2.77519  2.73123  3.46354  2.40513  3.72494  3.29354  2.67741  2.69355  4.24690  2.90347  2.73739  3.18146  2.89801  2.37887  2.77519  2.98518  4.58477  3.61503
        0.02290  4.18373  4.90608  0.61958  0.77255  0.48576  0.95510
365     2.67878  5.05971  2.57373  2.20916  4.36738  3.39538  3.64589  3.81937  2.03121  3.35503  4.13758  2.88937  3.82854  2.77045  2.89648  2.33295  2.13261  3.43471  5.53435  4.15148    406 k - - -
        2.68618  4.42225  2.77519  2.73123  3.46354  2.40513  3.72494  3.29354  2.67741  2.69355  4.24690  2.90347  2.73739  3.18146  2.89801  2.37887  2.77519  2.98518  4.58477  3.61503
        0.02290  4.18373  4.90608  0.61958  0.77255  0.48576  0.95510
366     1.70618  4.30226  4.10076  3.54466  3.44896  3.88688  4.29073  2.07811  3.45590  2.26197  2.80012  3.83317  4.27365  3.72419  3.71604  3.19289  3.04404  1.75813  5.08450  3.88629    407 a - - -
        2.68618  4.42225  2.77519  2.73123  3.46354  2.40513  3.72494  3.29354  2.67741  2.69355  4.24690  2.90347  2.73739  3.18146  2.89801  2.37887  2.77519  2.98518  4.58477  3.61503
        0.02290  4.18373  4.90608  0.61958  0.77255  0.48576  0.95510
367     2.64593  4.90274  2.96673  2.46172  4.26555  3.12748  3.67237  3.69585  2.40170  3.25899  4.05811  2.97318  3.84231  2.01451  2.60817  2.14218  2.56381  3.32839  5.45555  4.11568    408 q - - -
        2.68618  4.42225  2.77519  2.73123  3.46354  2.40513  3.72494  3.29354  2.67741  2.69355  4.24690  2.90347  2.73739  3.18146  2.89801  2.37887  2.77519  2.98518  4.58477  3.61503
        0.02290  4.18373  4.90608  0.61958  0.77255  0.48576  0.95510
368     2.87363  5.24635  1.14669  2.28931  4.78857  2.71746  3.90129  4.29846  2.91007  3.83250  4.66790  2.84631  3.90536  3.07368  3.47765  2.35389  3.20830  3.83426  5.99865  4.53931    409 d - - -
        2.68618  4.42225  2.77519  2.73123  3.46354  2.40513  3.72494  3.29354  2.67741  2.69355  4.24690  2.90347  2.73739  3.18146  2.89801  2.37887  2.77519  2.98518  4.58477  3.61503
        0.02290  4.18373  4.90608  0.61958  0.77255  0.48576  0.95510
369     0.89272  4.32084  3.25549  3.16880  4.34776  3.09122  4.30449  3.67127  3.30386  3.44574  4.30768  3.37918  3.83126  3.58506  3.62384  2.36128  2.86292  3.19474  5.71925  4.48281    410 a - - -
        2.68618  4.42225  2.77519  2.73123  3.46354  2.40513  3.72494  3.29354  2.67741  2.69355  4.24690  2.90347  2.73739  3.18146  2.89801  2.37887  2.77519  2.98518  4.58477  3.61503
        0.02290  4.18373  4.90608  0.61958  0.77255  0.48576  0.95510
370     1.87305  4.85609  3.02311  2.14677  4.13813  3.46123  3.68034  3.53050  2.40270  2.91628  3.96343  3.01412  3.87429  2.83359  2.34370  2.71303  2.91487  3.20877  5.37058  4.05335    411 a - - -
        2.68618  4.42225  2.77519  2.73123  3.46354  2.40513  3.72494  3.29354  2.67741  2.69355  4.24690  2.90347  2.73739  3.18146  2.89801  2.37887  2.77519  2.98518  4.58477  3.61503
        0.02290  4.18373  4.90608  0.61958  0.77255  0.48576  0.95510
371     3.90581  5.15933  4.60044  4.39164  3.05721  4.10803  4.33949  3.95678  4.11523  3.29253  4.57374  4.48179  4.65362  4.48590  4.18455  4.10625  4.22457  3.85500  0.43840  3.04495    412 W - - -
        2.68618  4.42225  2.77519  2.73123  3.46354  2.40513  3.72494  3.29354  2.67741  2.69355  4.24690  2.90347  2.73739  3.18146  2.89801  2.37887  2.77519  2.98518  4.58477  3.61503
        0.02290  4.18373  4.90608  0.61958  0.77255  0.48576  0.95510
372     3.06741  5.65566  1.76780  1.06251  4.94610  3.32011  3.87478  4.47572  2.90103  3.96723  4.82413  2.78300  3.94818  3.04217  3.50268  2.94335  3.35830  4.04111  6.10866  4.59871    413 e - - -
        2.68618  4.42225  2.77519  2.73123  3.46354  2.40513  3.72494  3.29354  2.67741  2.69355  4.24690  2.90347  2.73739  3.18146  2.89801  2.37887  2.77519  2.98518  4.58477  3.61503
        0.02290  4.18373  4.90608  0.61958  0.77255  0.48576  0.95510
373     2.59898  4.25897  3.59102  3.02806  3.39756  3.59924  3.88373  2.44584  2.96618  2.47254  3.37800  2.93281  3.98652  3.26022  3.28801  2.51124  2.63383  1.90360  4.86408  3.21186    414 v - - -
        2.68618  4.42225  2.77519  2.73123  3.46354  2.40513  3.72494  3.29354  2.67741  2.69355  4.24690  2.90347  2.73739  3.18146  2.89801  2.37887  2.77519  2.98518  4.58477  3.61503
```

```
     0.02290  4.18373  4.90608  0.61958  0.77255  0.48576  0.95510
374  2.75023  5.02965  2.80633  1.74718  4.16948  3.44228  3.68010  3.72470  2.44925  3.28177  4.10643  2.94548  3.88332  2.24996  2.88350  2.74447  2.98543  3.38328  5.41975  2.65913   415 e - - -
     2.68618  4.42225  2.77519  2.73123  3.46354  2.40513  3.72494  3.29354  2.67741  2.69355  4.24690  2.90347  2.73739  3.18146  2.89801  2.37887  2.77519  2.98518  4.58477  3.61503
     0.02290  4.18373  4.90608  0.61958  0.77255  0.48576  0.95510
375  3.24033  5.29254  3.71102  2.99640  4.78716  3.81329  3.71667  4.12052  1.67040  3.53861  4.42845  3.36232  4.17730  2.85433  1.01392  3.23787  3.39204  3.81293  5.54861  4.41983   416 r - - -
     2.68618  4.42225  2.77519  2.73123  3.46354  2.40513  3.72494  3.29354  2.67741  2.69355  4.24690  2.90347  2.73739  3.18146  2.89801  2.37887  2.77519  2.98518  4.58477  3.61503
     0.02290  4.18373  4.90608  0.61958  0.77255  0.48576  0.95510
376  3.48100  4.83088  4.91836  4.40491  3.12939  4.64598  4.94984  2.29579  4.15593  0.67860  2.74776  4.67271  4.85565  4.35628  4.28083  4.04580  3.71758  2.44981  5.24343  4.11265   417 L - - -
     2.68618  4.42225  2.77519  2.73123  3.46354  2.40513  3.72494  3.29354  2.67741  2.69355  4.24690  2.90347  2.73739  3.18146  2.89801  2.37887  2.77519  2.98518  4.58477  3.61503
     0.02290  4.18373  4.90608  0.61958  0.77255  0.48576  0.95510
377  1.83333  4.68064  2.47106  2.57779  4.28645  3.26964  3.83818  3.70580  2.69245  3.32006  4.12924  3.04259  3.82215  3.00471  3.14921  1.88941  2.48027  3.29465  5.54877  4.22152   418 a - - -
     2.68618  4.42225  2.77519  2.73123  3.46354  2.40513  3.72494  3.29354  2.67741  2.69355  4.24690  2.90347  2.73739  3.18146  2.89801  2.37887  2.77519  2.98518  4.58477  3.61503
     0.09465  4.18373  2.58931  0.61958  0.77255  0.48576  0.95510
378  2.83557  5.29880  1.54222  2.03387  4.63984  2.44527  3.75258  4.12158  2.67624  3.64363  4.45655  2.78631  3.85696  2.90193  3.22440  2.76923  2.67931  3.70666  5.82024  4.37095   419 d - - -
     2.68618  4.42225  2.77519  2.73123  3.46354  2.40513  3.72494  3.29354  2.67741  2.69355  4.24690  2.90347  2.73739  3.18146  2.89801  2.37887  2.77519  2.98518  4.58477  3.61503
     0.02459  4.11367  4.83601  0.61958  0.77255  0.43826  1.03609
379  3.39102  4.68267  5.08845  4.52005  3.18727  4.70481  5.03440  1.76452  4.39207  0.91343  2.59170  4.75546  4.86342  4.45856  4.48132  4.05857  3.61120  2.11427  5.29134  4.25396   420 l - - -
     2.68618  4.42225  2.77519  2.73123  3.46354  2.40513  3.72494  3.29354  2.67741  2.69355  4.24690  2.90347  2.73739  3.18146  2.89801  2.37887  2.77519  2.98518  4.58477  3.61503
     0.02290  4.18373  4.90608  0.61958  0.77255  0.48576  0.95510
380  2.22983  4.24276  4.03921  3.49585  3.24864  3.80769  4.12267  1.58662  3.37889  2.28352  3.31894  3.75222  4.19377  3.64653  3.60559  3.11345  2.98192  2.22227  4.83472  2.61944   421 i - - -
     2.68618  4.42225  2.77519  2.73123  3.46354  2.40513  3.72494  3.29354  2.67741  2.69355  4.24690  2.90347  2.73739  3.18146  2.89801  2.37887  2.77519  2.98518  4.58477  3.61503
     0.02290  4.18373  4.90608  0.61958  0.77255  0.48576  0.95510
381  2.78252  4.83570  3.11750  2.63258  3.64240  3.55692  3.21193  3.46547  2.11780  3.06007  3.92999  2.72598  3.96135  2.93174  2.82423  2.83570  3.00746  3.18051  5.03751  1.91317   422 y - - -
     2.68618  4.42225  2.77519  2.73123  3.46354  2.40513  3.72494  3.29354  2.67741  2.69355  4.24690  2.90347  2.73739  3.18146  2.89801  2.37887  2.77519  2.98518  4.58477  3.61503
     0.02290  4.18373  4.90608  0.61958  0.77255  0.48576  0.95510
382  3.12176  5.74102  1.03354  1.69905  5.02286  3.31047  3.90620  4.56644  2.98421  4.05377  4.93047  2.77062  3.96261  3.08188  3.61689  2.98191  3.42271  4.12566  6.19502  4.66027   423 d - - -
     2.68618  4.42225  2.77519  2.73123  3.46354  2.40513  3.72494  3.29354  2.67741  2.69355  4.24690  2.90347  2.73739  3.18146  2.89801  2.37887  2.77519  2.98518  4.58477  3.61503
     0.02290  4.18373  4.90608  0.61958  0.77255  0.48576  0.95510
383  2.08311  4.37815  3.41899  2.92355  4.02592  3.23270  3.98726  3.39787  2.90355  3.08755  3.93193  3.26143  2.30109  3.22220  3.28859  2.13910  2.08404  2.67970  5.38384  4.13122   424 a - - -
     2.68618  4.42225  2.77519  2.73123  3.46354  2.40513  3.72494  3.29354  2.67741  2.69355  4.24690  2.90347  2.73739  3.18146  2.89801  2.37887  2.77519  2.98518  4.58477  3.61503
     0.02290  4.18373  4.90608  0.61958  0.77255  0.48576  0.95510
384  2.57444  5.04936  1.26869  2.36361  4.59527  3.28053  3.86371  4.02123  2.80087  3.61866  4.44589  2.89910  3.87906  3.02936  3.32200  2.58099  2.55298  3.59657  5.82409  4.41553   425 d - - -
     2.68618  4.42225  2.77519  2.73123  3.46354  2.40513  3.72494  3.29354  2.67741  2.69355  4.24690  2.90347  2.73739  3.18146  2.89801  2.37887  2.77519  2.98518  4.58477  3.61503
     0.02290  4.18373  4.90608  0.61958  0.77255  0.48576  0.95510
385  2.51262  4.54030  3.03359  2.83756  4.42028  3.15479  4.11696  3.95671  3.02801  3.59278  4.42922  2.51434  3.85263  3.34700  3.40283  1.04027  2.95198  3.44306  5.73029  4.40707   426 s - - -
     2.68618  4.42225  2.77519  2.73123  3.46354  2.40513  3.72494  3.29354  2.67741  2.69355  4.24690  2.90347  2.73739  3.18146  2.89801  2.37887  2.77519  2.98518  4.58477  3.61503
     0.02290  4.18373  4.90608  0.61958  0.77255  0.48576  0.95510
386  2.97032  5.11692  3.32077  2.75263  4.53831  3.62861  3.70199  3.93437  2.08824  3.42127  4.27253  3.18879  4.03617  2.41578  1.25490  2.65017  3.17776  3.59756  5.51046  4.28358   427 r - - -
     2.68618  4.42225  2.77519  2.73123  3.46354  2.40513  3.72494  3.29354  2.67741  2.69355  4.24690  2.90347  2.73739  3.18146  2.89801  2.37887  2.77519  2.98518  4.58477  3.61503
     0.02290  4.18373  4.90608  0.61958  0.77255  0.48576  0.95510
387  2.90229  4.29596  4.50832  3.93415  1.67943  4.04815  4.22058  2.16143  3.79120  1.78483  2.99222  4.06544  4.36532  3.93701  3.90200  3.35649  3.12996  2.17253  4.66195  2.89940   428 f - - -
     2.68618  4.42225  2.77519  2.73123  3.46354  2.40513  3.72494  3.29354  2.67741  2.69355  4.24690  2.90347  2.73739  3.18146  2.89801  2.37887  2.77519  2.98518  4.58477  3.61503
     0.02290  4.18373  4.90608  0.61958  0.77255  0.48576  0.95510
388  1.41440  4.27760  3.56236  3.35528  4.52683  1.26203  4.44731  3.91015  3.47683  3.63809  4.47589  3.46636  3.81604  3.73988  3.76444  2.52728  2.85455  3.33150  5.85858  4.67607   429 g - - -
     2.68618  4.42225  2.77519  2.73123  3.46354  2.40513  3.72494  3.29354  2.67741  2.69355  4.24690  2.90347  2.73739  3.18146  2.89801  2.37887  2.77519  2.98518  4.58477  3.61503
     0.02290  4.18373  4.90608  0.61958  0.77255  0.48576  0.95510
389  3.09995  4.83262  3.83645  3.78085  4.89495  0.37791  4.87055  4.58175  4.00380  4.19229  5.17870  4.00221  4.23262  4.30392  4.19473  3.28255  3.60515  4.04968  5.89622  5.01130   430 G - - -
     2.68618  4.42225  2.77519  2.73123  3.46354  2.40513  3.72494  3.29354  2.67741  2.69355  4.24690  2.90347  2.73739  3.18146  2.89801  2.37887  2.77519  2.98518  4.58477  3.61503
     0.02290  4.18373  4.90608  0.61958  0.77255  0.48576  0.95510
390  3.40610  4.75377  4.93045  4.40970  3.18869  4.61600  4.98032  2.13821  4.20977  0.74516  3.00687  4.66311  4.84350  4.38753  4.34280  4.00053  3.65068  2.14981  5.29339  4.16939   431 l - - -
     2.68618  4.42225  2.77519  2.73123  3.46354  2.40513  3.72494  3.29354  2.67741  2.69355  4.24690  2.90347  2.73739  3.18146  2.89801  2.37887  2.77519  2.98518  4.58477  3.61503
     0.02290  4.18373  4.90608  0.61958  0.77255  0.48576  0.95510
391  3.35240  4.65812  5.04430  4.46751  2.60016  4.62632  4.90915  2.02909  4.34392  0.88250  2.76480  4.67988  4.79663  4.38604  4.41632  3.96890  3.56857  2.17516  5.17085  4.10077   432 l - - -
     2.68618  4.42225  2.77519  2.73123  3.46354  2.40513  3.72494  3.29354  2.67741  2.69355  4.24690  2.90347  2.73739  3.18146  2.89801  2.37887  2.77519  2.98518  4.58477  3.61503
     0.02290  4.18373  4.90608  0.61958  0.77255  0.48576  0.95510
392  2.83933  4.39431  3.88814  2.92558  1.44179  3.83776  3.89950  2.73229  3.26499  2.31577  3.11402  3.65376  4.20339  3.53561  3.52088  3.12861  3.07172  2.59102  4.50594  2.90526   433 f - - -
     2.68618  4.42225  2.77519  2.73123  3.46354  2.40513  3.72494  3.29354  2.67741  2.69355  4.24690  2.90347  2.73739  3.18146  2.89801  2.37887  2.77519  2.98518  4.58477  3.61503
     0.02290  4.18373  4.90608  0.61958  0.77255  0.48576  0.95510
393  3.53719  4.85745  4.77310  4.39477  0.92797  4.46480  4.04203  2.74024  4.22762  1.81590  3.41652  4.36205  4.77799  4.29883  4.30051  3.87735  3.78599  2.80139  4.21925  2.57548   434 f - - -
     2.68618  4.42225  2.77519  2.73123  3.46354  2.40513  3.72494  3.29354  2.67741  2.69355  4.24690  2.90347  2.73739  3.18146  2.89801  2.37887  2.77519  2.98518  4.58477  3.61503
     0.02290  4.18373  4.90608  0.61958  0.77255  0.48576  0.95510
394  2.60717  4.11843  3.94403  3.36545  2.06884  3.41467  3.94999  2.51416  3.26138  2.31132  3.23267  3.61331  4.05528  3.49920  3.47898  2.70937  2.55467  2.30455  4.24516  2.88311   435 f - - -
     2.68618  4.42225  2.77519  2.73123  3.46354  2.40513  3.72494  3.29354  2.67741  2.69355  4.24690  2.90347  2.73739  3.18146  2.89801  2.37887  2.77519  2.98518  4.58477  3.61503
     0.02290  4.18373  4.90608  0.61958  0.77255  0.48576  0.95510
395  2.80145  5.11562  1.22042  2.33815  4.64416  3.28001  3.87320  4.09847  2.82918  3.67878  4.51069  2.88144  3.88949  3.04143  3.36131  2.42676  2.59956  3.66652  5.87052  4.44484   436 d - - -
     2.68618  4.42225  2.77519  2.73123  3.46354  2.40513  3.72494  3.29354  2.67741  2.69355  4.24690  2.90347  2.73739  3.18146  2.89801  2.37887  2.77519  2.98518  4.58477  3.61503
     0.02290  4.18373  4.90608  0.61958  0.77255  0.48576  0.95510
```

```
    396   2.41895  5.05944  2.37085  2.07841  4.38623  3.37472  3.66091  3.84341  2.46246  3.38018  4.16172  2.87902  2.54055  2.78716  2.95798  2.51271  2.56569  3.45089  5.56191  4.17028    437 e - - -
          2.68618  4.42225  2.77519  2.73123  3.46354  2.40513  3.72494  3.29354  2.67741  2.69355  4.24690  2.90347  2.73739  3.18146  2.89801  2.37887  2.77519  2.98518  4.58477  3.61503
          0.02290  4.18373  4.90608  0.61958  0.77255  0.48576  0.95510
    397   2.35268  5.03124  2.36691  2.12392  4.33427  3.32389  3.63041  3.78853  2.41158  3.32599  4.10309  2.80869  3.81321  2.68327  2.90092  2.20013  2.78777  3.07117  5.50821  4.12312    438 e - - -
          2.68619  4.42226  2.77520  2.73124  3.46355  2.40513  3.72495  3.29355  2.67741  2.69356  4.24630  2.90347  2.73740  3.18147  2.89801  2.37887  2.77520  2.98519  4.58478  3.61504
          0.07234  2.77449  4.90608  0.43961  1.03364  0.48576  0.95510
    398   2.78915  4.78555  3.03573  2.86082  4.55438  0.94820  4.13419  4.02776  2.43496  3.64759  4.52814  3.27559  3.99189  3.36165  3.25077  2.88368  3.18476  3.59171  5.74491  4.51670    440 g - - -
          2.68618  4.42225  2.77519  2.73123  3.46354  2.40513  3.72494  3.29354  2.67741  2.69355  4.24690  2.90347  2.73739  3.18146  2.89801  2.37887  2.77519  2.98518  4.58477  3.61503
          0.02290  4.18373  4.90608  0.61958  0.77255  0.48576  0.95510
    399   2.49137  5.03709  2.88544  2.27206  4.35020  3.16802  3.61546  3.80152  2.07762  3.32746  4.10152  2.27629  3.81846  2.73229  2.59929  2.57504  2.57078  3.41357  5.49607  4.12190    441 k - - -
          2.68618  4.42225  2.77519  2.73123  3.46354  2.40513  3.72494  3.29354  2.67741  2.69355  4.24690  2.90347  2.73739  3.18146  2.89801  2.37887  2.77519  2.98518  4.58477  3.61503
          0.02290  4.18373  4.90608  0.61958  0.77255  0.48576  0.95510
    400   3.18265  5.20389  3.48914  2.72793  4.64391  3.72888  3.80670  4.03853  2.11371  3.51709  4.42682  3.35238  4.15800  2.96473  0.90586  3.20109  3.38247  3.73247  5.55689  4.39391    442 r - - -
          2.68618  4.42225  2.77519  2.73123  3.46354  2.40513  3.72494  3.29354  2.67741  2.69355  4.24690  2.90347  2.73739  3.18146  2.89801  2.37887  2.77519  2.98518  4.58477  3.61503
          0.02290  4.18373  4.90608  0.61958  0.77255  0.48576  0.95510
    401   2.67830  4.59462  3.26468  2.71812  2.99004  3.56147  3.71235  3.00078  2.67225  2.79699  3.68594  2.84904  3.94707  2.02737  3.07360  2.79514  2.90929  2.89855  4.86952  2.58438    443 q - - -
          2.68618  4.42225  2.77519  2.73123  3.46354  2.40513  3.72494  3.29354  2.67741  2.69355  4.24690  2.90347  2.73739  3.18146  2.89801  2.37887  2.77519  2.98518  4.58477  3.61503
          0.02290  4.18373  4.90608  0.61958  0.77255  0.48576  0.95510
    402   2.37578  4.18169  3.90219  3.18263  3.29871  3.72450  4.02508  1.78939  3.23500  2.23764  2.72345  3.62048  3.43171  3.49610  3.48638  3.00222  2.88289  2.18950  4.82395  3.62206    444 i - - -
          2.68618  4.42225  2.77519  2.73123  3.46354  2.40513  3.72494  3.29354  2.67741  2.69355  4.24690  2.90347  2.73739  3.18146  2.89801  2.37887  2.77519  2.98518  4.58477  3.61503
          0.02290  4.18373  4.90608  0.61958  0.77255  0.48576  0.95510
    403   2.40235  4.13416  3.90754  3.32986  3.25349  3.68408  3.98590  2.20239  3.23381  2.32092  3.05756  3.60197  4.05848  3.48358  3.46981  2.57201  2.69848  1.88021  4.75922  3.01719    445 v - - -
          2.68618  4.42225  2.77519  2.73123  3.46354  2.40513  3.72494  3.29354  2.67741  2.69355  4.24690  2.90347  2.73739  3.18146  2.89801  2.37887  2.77519  2.98518  4.58477  3.61503
          0.02290  4.18373  4.90608  0.61958  0.77255  0.48576  0.95510
    404   2.25772  4.83996  2.97590  2.04062  4.09912  3.42804  3.64698  3.51928  2.43907  3.11432  3.31661  2.96047  3.58783  2.62923  2.90214  2.38026  2.65019  3.18621  5.34903  4.00844    446 e - - -
          2.68618  4.42225  2.77519  2.73123  3.46354  2.40513  3.72494  3.29354  2.67741  2.69355  4.24690  2.90347  2.73739  3.18146  2.89801  2.37887  2.77519  2.98518  4.58477  3.61503
          0.02290  4.18373  4.90608  0.61958  0.77255  0.48576  0.95510
    405   3.14774  4.45479  4.92175  4.42461  3.70261  4.51378  5.12390  1.05290  4.31657  2.22755  3.49854  4.61964  4.83404  4.57988  4.50634  3.90172  3.18480  1.31564  5.64411  4.44379    447 i - - -
          2.68588  4.42227  2.77522  2.73125  3.46356  2.40515  3.72497  3.29356  2.67743  2.69357  4.24692  2.90349  2.73742  3.18148  2.89803  2.37889  2.77522  2.98520  4.58479  3.61505
          0.15442  1.99738  4.90608  0.30389  1.33918  0.48576  0.95510
    406   2.74101  5.05073  1.57645  2.35620  4.61510  2.08577  3.84409  4.08856  2.76750  3.63826  4.44758  2.89152  3.86506  3.00236  3.29095  2.44186  2.76800  3.63938  5.82231  4.41135    449 d - - -
          2.68618  4.42225  2.77519  2.73123  3.46354  2.40513  3.72494  3.29354  2.67741  2.69355  4.24690  2.90347  2.73739  3.18146  2.89801  2.37887  2.77519  2.98518  4.58477  3.61503
          0.02290  4.18373  4.90608  0.61958  0.77255  0.48576  0.95510
    407   1.81348  4.65443  3.01267  2.62648  4.27156  2.14997  3.86049  3.69694  2.69248  3.30815  4.12531  3.07591  3.83525  2.31318  3.11850  2.44426  2.88948  3.28936  5.53674  4.22552    450 a - - -
          2.68618  4.42225  2.77519  2.73123  3.46354  2.40513  3.72494  3.29354  2.67741  2.69355  4.24690  2.90347  2.73739  3.18146  2.89801  2.37887  2.77519  2.98518  4.58477  3.61503
          0.02290  4.18373  4.90608  0.61958  0.77255  0.48576  0.95510
    408   2.29070  4.33383  3.61005  3.07447  3.01280  3.57768  3.97299  2.41129  3.01941  2.48554  3.46744  3.43675  1.98521  3.32252  3.35203  2.87079  2.89563  2.51798  4.99442  3.74678    451 p - - -
          2.68618  4.42225  2.77519  2.73123  3.46354  2.40513  3.72494  3.29354  2.67741  2.69355  4.24690  2.90347  2.73739  3.18146  2.89801  2.37887  2.77519  2.98518  4.58477  3.61503
          0.02290  4.18373  4.90608  0.61958  0.77255  0.48576  0.95510
    409   3.18407  4.53198  4.80864  4.30960  3.46547  4.44241  4.94945  1.95174  4.15641  1.47957  3.30674  4.52439  4.76169  4.40587  4.33038  3.82947  3.44910  1.08794  5.42835  4.23412    452 v - - -
          2.68618  4.42225  2.77519  2.73123  3.46354  2.40513  3.72494  3.29354  2.67741  2.69355  4.24690  2.90347  2.73739  3.18146  2.89801  2.37887  2.77519  2.98518  4.58477  3.61503
          0.02290  4.18373  4.90608  0.61958  0.77255  0.48576  0.95510
    410   3.22903  4.52517  4.99807  4.52296  3.64476  4.59678  5.22264  0.91817  4.40013  2.10693  3.44181  4.72065  4.90437  4.65941  4.57936  4.00742  3.50044  1.48786  5.66898  4.46663    453 i - - -
          2.68618  4.42225  2.77519  2.73123  3.46354  2.40513  3.72494  3.29354  2.67741  2.69355  4.24690  2.90347  2.73739  3.18146  2.89801  2.37887  2.77519  2.98518  4.58477  3.61503
          0.02290  4.18373  4.90608  0.61958  0.77255  0.48576  0.95510
    411   3.22031  4.91301  3.86274  3.75476  4.70463  3.58351  4.79631  4.34360  3.84144  3.93066  5.00116  4.02490  0.41006  4.22005  4.04145  3.39999  3.68818  3.95083  5.79302  4.83303    454 P - - -
          2.68618  4.42225  2.77519  2.73123  3.46354  2.40513  3.72494  3.29354  2.67741  2.69355  4.24690  2.90347  2.73739  3.18146  2.89801  2.37887  2.77519  2.98518  4.58477  3.61503
          0.02290  4.18373  4.90608  0.61958  0.77255  0.48576  0.95510
    412   2.64663  4.85570  3.02635  2.42117  4.13697  3.44894  3.65562  3.54896  1.91337  3.13919  3.95169  2.99065  3.85059  2.80141  2.71901  2.24318  2.40931  2.90012  5.36305  4.03672    455 k - - -
          2.68618  4.42225  2.77519  2.73123  3.46354  2.40513  3.72494  3.29354  2.67741  2.69355  4.24690  2.90347  2.73739  3.18146  2.89801  2.37887  2.77519  2.98518  4.58477  3.61503
          0.02290  4.18373  4.90608  0.61958  0.77255  0.48576  0.95510
    413   3.68410  4.99736  4.66912  4.45276  0.61317  4.27914  4.05653  3.18184  4.35515  2.48406  3.87144  4.38573  4.73817  4.43916  4.39718  3.93054  3.98917  3.19377  4.23206  2.56778    456 F - - -
          2.68618  4.42225  2.77519  2.73123  3.46354  2.40513  3.72494  3.29354  2.67741  2.69355  4.24690  2.90347  2.73739  3.18146  2.89801  2.37887  2.77519  2.98518  4.58477  3.61503
          0.02290  4.18373  4.90608  0.61958  0.77255  0.48576  0.95510
    414   2.60476  4.59194  2.96465  2.36614  3.77811  3.03198  3.71110  2.68454  2.58375  2.75030  3.06571  2.84823  3.87599  2.80368  3.01371  2.70799  2.83641  2.52836  5.13424  3.84664    457 e - - -
          2.68618  4.42225  2.77519  2.73123  3.46354  2.40513  3.72494  3.29354  2.67741  2.69355  4.24690  2.90347  2.73739  3.18146  2.89801  2.37887  2.77519  2.98518  4.58477  3.61503
          0.02290  4.18373  4.90608  0.61958  0.77255  0.48576  0.95510
    415   1.63404  4.15683  3.15476  2.86181  4.12812  2.26370  3.99997  3.51080  2.94860  3.20658  4.04810  3.12345  2.82671  3.25143  3.29559  2.36116  2.65189  3.03284  5.46503  4.23075    458 a - - -
          2.68613  4.42252  2.77526  2.73151  3.46381  2.40450  3.72522  3.29350  2.67743  2.69369  4.24541  2.90374  2.73734  3.18153  2.89812  2.37905  2.77505  2.98536  4.58419  3.61498
          0.28512  1.39401        *  1.79251  0.18217  0.00000        *
//
HMMER3/f [3.1b2 | February 2015]
NAME  pmoB_IA_strict Protein
LENG  415
ALPH  amino
RF    no
MM    no
```

```
CONS  yes
CS    no
MAP   yes
DATE  Tue Jan  2 10:16:23 2018
NSEQ  33
EFFN  0.954712
CKSUM 989184870
STATS LOCAL MSV      -11.4998  0.69901
STATS LOCAL VITERBI  -12.2907  0.69901
STATS LOCAL FORWARD   -6.0573  0.69901
HMM          A        C        D        E        F        G        H        I        K        L        M        N        P        Q        R        S        T        V        W        Y
            m->m     m->i     m->d     i->m     i->i     d->m     d->d
  COMPO   2.53589  4.57913  3.04421  2.74772  3.19902  2.80752  3.83449  2.73088  2.75551  2.45141  3.55085  3.12427  3.22409  3.13744  2.98003  2.72534  2.79067  2.50991  4.15916  3.48310
          2.68613  4.42239  2.77534  2.73132  3.46340  2.40527  3.72509  3.29353  2.67713  2.69358  4.24590  2.90345  2.73754  3.18147  2.89789  2.37892  2.77518  2.98504  4.58491  3.61517
          0.38717  1.98344  1.69592  1.53347  0.24307  0.00000        *
      1   2.46449  4.27048  3.73987  3.18451  3.34605  3.68741  3.99901  2.22631  2.85361  2.20861  2.06966  3.52910  4.07075  3.37708  3.31756  2.97634  2.90994  2.28635  4.91962  3.71320     12 m - - -
          2.68618  4.42225  2.77519  2.73123  3.46354  2.40513  3.72494  3.29354  2.67741  2.69355  4.24690  2.90347  2.73739  3.18146  2.89801  2.37887  2.77519  2.98518  4.58477  3.61503
          0.02840  3.97144  4.69378  0.61958  0.77255  0.68064  0.70581
      2   2.79226  5.09971  2.69333  2.06535  4.45990  2.89507  3.64152  3.88211  1.57386  3.40325  4.22835  2.89123  3.85546  2.78327  2.64093  2.76534  3.03033  3.51539  5.54711  4.21754     13 k - - -
          2.68618  4.42225  2.77519  2.73123  3.46354  2.40513  3.72494  3.29354  2.67741  2.69355  4.24690  2.90347  2.73739  3.18146  2.89801  2.37887  2.77519  2.98518  4.58477  3.61503
          0.02840  3.97144  4.69378  0.61958  0.77255  0.68064  0.70581
      3   2.55430  4.31400  3.37006  2.80771  3.45445  3.50995  3.23814  2.50030  2.73740  2.20458  3.33537  3.02042  3.89508  3.06854  3.09493  2.64922  2.52808  2.58248  4.88859  3.64276     14 l - - -
          2.68618  4.42225  2.77519  2.73123  3.46354  2.40513  3.72494  3.29354  2.67741  2.69355  4.24690  2.90347  2.73739  3.18146  2.89801  2.37887  2.77519  2.98518  4.58477  3.61503
          0.02840  3.97144  4.69378  0.61958  0.77255  0.68064  0.70581
      4   2.38600  4.27259  3.45163  2.90772  3.46537  3.06644  3.83898  2.16812  2.84990  2.36872  3.42322  2.87364  3.92074  3.16661  3.19505  2.77882  2.80076  2.35044  4.92336  3.68720     15 i - - -
          2.68618  4.42225  2.77519  2.73123  3.46354  2.40513  3.72494  3.29354  2.67741  2.69355  4.24690  2.90347  2.73739  3.18146  2.89801  2.37887  2.77519  2.98518  4.58477  3.61503
          0.02840  3.97144  4.69378  0.61958  0.77255  0.46042  0.99702
      5   2.67449  4.85866  3.04287  2.38889  4.12125  3.46813  3.63738  3.52482  1.83111  3.11275  2.74701  2.69615  3.86012  2.78699  2.64527  2.70439  2.80457  3.20484  5.33306  4.02320     16 k - - -
          2.68618  4.42225  2.77519  2.73123  3.46354  2.40513  3.72494  3.29354  2.67741  2.69355  4.24690  2.90347  2.73739  3.18146  2.89801  2.37887  2.77519  2.98518  4.58477  3.61503
          0.02464  4.11150  4.83384  0.61958  0.77255  0.44905  1.01676
      6   2.51326  4.93290  2.34032  2.38275  4.20910  3.28652  3.60910  3.39468  2.10800  3.20752  3.46820  2.83311  3.80563  2.73489  2.61389  2.61274  2.84162  3.28605  5.40839  3.71129     17 k - - -
          2.68618  4.42225  2.77519  2.73123  3.46354  2.40513  3.72494  3.29354  2.67741  2.69355  4.24690  2.90347  2.73739  3.18146  2.89801  2.37887  2.77519  2.98518  4.58477  3.61503
          0.02334  4.16489  4.88723  0.61958  0.77255  0.48576  0.95510
      7   2.87933  5.16128  3.18722  2.59753  4.55565  3.57923  3.63769  3.95184  1.50445  3.42330  4.23662  2.96569  3.96047  2.63170  1.92858  2.41399  3.07872  3.59004  5.51877  4.25170     18 k - - -
          2.68618  4.42225  2.77519  2.73123  3.46354  2.40513  3.72494  3.29354  2.67741  2.69355  4.24690  2.90347  2.73739  3.18146  2.89801  2.37887  2.77519  2.98518  4.58477  3.61503
          0.02334  4.16489  4.88723  0.61958  0.77255  0.48576  0.95510
      8   2.60902  4.16767  4.23077  3.65618  3.17954  3.46150  4.22083  1.70370  3.54134  1.90023  3.26330  3.86891  4.23566  3.76904  3.72872  3.17570  2.77635  1.80027  4.88902  3.69627     19 i - - -
          2.68618  4.42225  2.77519  2.73123  3.46354  2.40513  3.72494  3.29354  2.67741  2.69355  4.24690  2.90347  2.73739  3.18146  2.89801  2.37887  2.77519  2.98518  4.58477  3.61503
          0.02334  4.16489  4.88723  0.61958  0.77255  0.48576  0.95510
      9   2.14975  4.41168  3.33585  2.77718  3.12374  2.89818  3.78445  2.94053  2.52982  2.53215  3.52388  3.03861  3.91688  3.06200  3.12674  2.76531  2.76038  2.35330  4.99435  3.74154     20 a - - -
          2.68618  4.42225  2.77519  2.73123  3.46354  2.40513  3.72494  3.29354  2.67741  2.69355  4.24690  2.90347  2.73739  3.18146  2.89801  2.37887  2.77519  2.98518  4.58477  3.61503
          0.02334  4.16489  4.88723  0.61958  0.77255  0.48576  0.95510
     10   2.80220  4.98532  3.21483  2.61256  3.71692  3.55977  3.65461  3.68740  1.44208  3.23466  4.06933  3.08906  3.94075  2.79586  2.09884  2.82121  2.83088  3.36441  5.40209  4.12315     21 k - - -
          2.68618  4.42225  2.77519  2.73123  3.46354  2.40513  3.72494  3.29354  2.67741  2.69355  4.24690  2.90347  2.73739  3.18146  2.89801  2.37887  2.77519  2.98518  4.58477  3.61503
          0.02334  4.16489  4.88723  0.61958  0.77255  0.48576  0.95510
     11   2.43243  4.13145  4.01396  3.43102  3.18484  3.72646  4.02093  2.31692  3.31463  1.61191  3.00242  3.67383  3.35469  3.55085  3.52034  3.01015  2.86757  2.34238  3.56639  3.53568     22 l - - -
          2.68618  4.42225  2.77519  2.73123  3.46354  2.40513  3.72494  3.29354  2.67741  2.69355  4.24690  2.90347  2.73739  3.18146  2.89801  2.37887  2.77519  2.98518  4.58477  3.61503
          0.02334  4.16489  4.88723  0.61958  0.77255  0.48576  0.95510
     12   2.21221  4.27111  3.54267  2.97773  3.42070  3.33394  3.85856  2.65343  2.91821  2.27054  3.39694  3.35631  3.95965  3.21719  3.25124  2.20640  2.68414  2.55350  3.46827  3.64830     23 s - - -
          2.68618  4.42225  2.77519  2.73123  3.46354  2.40513  3.72494  3.29354  2.67741  2.69355  4.24690  2.90347  2.73739  3.18146  2.89801  2.37887  2.77519  2.98518  4.58477  3.61503
          0.05279  4.16489  3.32740  0.61958  0.77255  0.48576  0.95510
     13   2.26622  4.14781  3.95389  3.37950  2.44355  3.25509  4.01108  2.50661  3.27704  1.82506  2.82986  3.64029  4.07916  3.52028  3.50208  2.99051  2.86717  2.33749  4.74920  3.54203     24 l - - -
          2.68618  4.42225  2.77519  2.73123  3.46354  2.40513  3.72494  3.29354  2.67741  2.69355  4.24690  2.90347  2.73739  3.18146  2.89801  2.37887  2.77519  2.98518  4.58477  3.61503
          0.02403  4.13613  4.85848  0.61958  0.77255  0.51985  0.90291
     14   2.56424  4.14370  4.11443  3.53585  2.97115  3.80002  4.11926  2.14604  3.42625  1.94429  3.10268  3.76702  4.16090  3.65595  3.62574  2.61718  2.91148  1.71065  4.81032  3.61680     25 v - - -
          2.68618  4.42225  2.77519  2.73123  3.46354  2.40513  3.72494  3.29354  2.67741  2.69355  4.24690  2.90347  2.73739  3.18146  2.89801  2.37887  2.77519  2.98518  4.58477  3.61503
          0.02403  4.13613  4.85848  0.61958  0.77255  0.51985  0.90291
     15   1.66713  4.33086  3.40994  2.96362  3.95285  2.16433  4.00940  3.30809  2.95380  2.45014  3.88371  3.28100  3.82807  3.27159  3.31542  2.32659  2.80159  2.95361  5.33608  4.09159     26 a - - -
          2.68618  4.42225  2.77519  2.73123  3.46354  2.40513  3.72494  3.29354  2.67741  2.69355  4.24690  2.90347  2.73739  3.18146  2.89801  2.37887  2.77519  2.98518  4.58477  3.61503
          0.03948  4.13613  3.78410  0.61958  0.77255  0.46502  0.98919
     16   2.59105  4.33175  4.43433  3.87552  3.38418  4.11421  4.50735  1.82660  3.76291  1.38793  3.25678  4.11261  4.44981  3.99473  3.96180  3.43400  2.94999  1.76713  5.13530  3.96516     27 l - - -
          2.68618  4.42225  2.77519  2.73123  3.46354  2.40513  3.72494  3.29354  2.67741  2.69355  4.24690  2.90347  2.73739  3.18146  2.89801  2.37887  2.77519  2.98518  4.58477  3.61503
          0.02369  4.15024  4.87258  0.61958  0.77255  0.47489  0.97274
     17   2.14254  4.25771  3.94134  3.39095  3.39468  3.72683  4.13958  2.30138  3.28892  1.53511  3.34000  3.67882  4.14218  3.57085  3.55100  2.82481  2.95252  2.17081  4.97352  3.76977     28 l - - -
          2.68618  4.42225  2.77519  2.73123  3.46354  2.40513  3.72494  3.29354  2.67741  2.69355  4.24690  2.90347  2.73739  3.18146  2.89801  2.37887  2.77519  2.98518  4.58477  3.61503
          0.02334  4.16489  4.88723  0.61958  0.77255  0.48576  0.95510
     18   2.15439  3.31017  3.99932  3.41504  3.04997  3.42938  3.98244  2.44468  3.30312  2.03508  3.08952  3.64234  4.05078  3.53170  3.50047  2.77803  2.81830  2.08098  4.69061  3.23671     29 l - - -
```

```
     2.68618  4.42225  2.77519  2.73123  3.46354  2.40513  3.72494  3.29354  2.67741  2.69355  4.24690  2.90347  2.73739  3.18146  2.89801  2.37887  2.77519  2.98518  4.58477  3.61503
     0.02334  4.16489  4.88723  0.61958  0.77255  0.48576  0.95510
 19  2.07164  4.37148  3.41498  2.89828  3.79953  2.20828  3.92164  2.82857  2.86406  2.72847  3.72847  3.26861  3.87394  3.18581  3.23930  2.61768  2.12913  2.81680  5.19925  3.95126    30 a - - -
     2.68618  4.42225  2.77519  2.73123  3.46354  2.40513  3.72494  3.29354  2.67741  2.69355  4.24690  2.90347  2.73739  3.18146  2.89801  2.37887  2.77519  2.98518  4.58477  3.61503
     0.02334  4.16489  4.88723  0.61958  0.77255  0.48576  0.95510
 20  2.56044  4.18006  3.97329  3.39955  3.00061  3.75880  4.06351  2.40799  3.30187  2.00779  2.98102  3.11852  4.12590  3.55438  3.53913  3.04165  2.90353  1.62110  4.82401  3.61828    31 v - - -
     2.68618  4.42225  2.77519  2.73123  3.46354  2.40513  3.72494  3.29354  2.67741  2.69355  4.24690  2.90347  2.73739  3.18146  2.89801  2.37887  2.77519  2.98518  4.58477  3.61503
     0.02334  4.16489  4.88723  0.61958  0.77255  0.48576  0.95510
 21  2.01998  4.23703  3.97884  3.41093  3.38406  3.81059  4.14393  2.01931  3.32724  2.13665  3.11072  3.71145  4.18146  3.59245  3.59044  3.09613  2.43369  1.96875  4.95282  3.74886    32 v - - -
     2.68618  4.42225  2.77519  2.73123  3.46354  2.40513  3.72494  3.29354  2.67741  2.69355  4.24690  2.90347  2.73739  3.18146  2.89801  2.37887  2.77519  2.98518  4.58477  3.61503
     0.02334  4.16489  4.88723  0.61958  0.77255  0.48576  0.95510
 22  1.69208  4.28201  3.78142  3.22096  3.41605  3.69197  4.04049  2.39400  3.13524  2.08681  2.89275  3.55904  4.09034  3.43022  3.43317  2.90697  2.86389  2.32717  4.95900  3.74711    33 a - - -
     2.68618  4.42225  2.77519  2.73123  3.46354  2.40513  3.72494  3.29354  2.67741  2.69355  4.24690  2.90347  2.73739  3.18146  2.89801  2.37887  2.77519  2.98518  4.58477  3.61503
     0.05520  4.16489  3.26552  0.61958  0.77255  0.48576  0.95510
 23  1.80857  4.27226  3.53198  3.05848  4.01762  2.90449  4.07162  3.37817  3.03324  3.08964  3.93851  3.32093  3.79858  3.33641  3.38830  1.93790  1.96160  2.57313  5.39483  4.16425    34 a - - -
     2.68618  4.42225  2.77519  2.73123  3.46354  2.40513  3.72494  3.29354  2.67741  2.69355  4.24690  2.90347  2.73739  3.18146  2.89801  2.37887  2.77519  2.98518  4.58477  3.61503
     0.08060  4.13377  2.79017  0.61958  0.77255  0.46342  0.99189
 24  2.46536  3.68056  4.11779  3.52725  3.12521  3.68830  3.99852  2.18851  3.39342  2.00583  2.54152  3.70193  3.68875  3.60040  3.54690  2.78024  2.80417  2.12843  3.97004  3.45442    35 l - - -
     2.68618  4.42225  2.77519  2.73123  3.46354  2.40513  3.72494  3.29354  2.67741  2.69355  4.24690  2.90347  2.73739  3.18146  2.89801  2.37887  2.77519  2.98518  4.58477  3.61503
     0.02464  4.11150  4.83384  0.61958  0.77255  0.54742  0.86379
 25  2.46454  4.13819  3.99805  3.42163  2.16616  3.09196  4.02672  2.25051  3.31494  1.98859  3.07820  3.67081  4.09505  3.55235  3.52962  3.01308  2.87325  2.28591  4.73992  3.52831    36 l - - -
     2.68618  4.42225  2.77519  2.73123  3.46354  2.40513  3.72494  3.29354  2.67741  2.69355  4.24690  2.90347  2.73739  3.18146  2.89801  2.37887  2.77519  2.98518  4.58477  3.61503
     0.02464  4.11150  4.83384  0.61958  0.77255  0.44905  1.01676
 26  2.13412  4.54697  3.15711  2.65535  3.83842  3.20884  3.77529  3.23841  2.66172  2.90074  3.75355  2.91715  3.48434  2.99304  3.08112  2.18278  2.84142  2.94440  5.19667  2.57895    37 a - - -
     2.68618  4.42225  2.77519  2.73123  3.46354  2.40513  3.72494  3.29354  2.67741  2.69355  4.24690  2.90347  2.73739  3.18146  2.89801  2.37887  2.77519  2.98518  4.58477  3.61503
     0.02334  4.16489  4.88723  0.61958  0.77255  0.48576  0.95510
 27  1.96048  4.28797  3.58879  3.05114  3.61667  3.44651  3.97435  2.54581  3.00014  2.65868  3.56714  3.38926  3.94079  3.30167  3.34032  2.19278  2.38358  2.21098  5.07085  3.84243    38 a - - -
     2.68618  4.42225  2.77519  2.73123  3.46354  2.40513  3.72494  3.29354  2.67741  2.69355  4.24690  2.90347  2.73739  3.18146  2.89801  2.37887  2.77519  2.98518  4.58477  3.61503
     0.02334  4.16489  4.88723  0.61958  0.77255  0.48576  0.95510
 28  2.06239  4.41774  3.29416  2.86400  4.14985  2.57627  3.98891  3.55285  2.88465  2.95391  4.04438  3.21551  1.83117  3.20698  3.27668  2.28820  2.81611  3.13977  5.47862  4.21412    39 p - - -
     2.68618  4.42225  2.77519  2.73123  3.46354  2.40513  3.72494  3.29354  2.67741  2.69355  4.24690  2.90347  2.73739  3.18146  2.89801  2.37887  2.77519  2.98518  4.58477  3.61503
     0.02334  4.16489  4.88723  0.61958  0.77255  0.48576  0.95510
 29  2.37900  3.82436  3.35620  2.81403  3.43740  3.38428  3.84942  3.12682  2.78451  2.81548  3.67973  3.06982  2.69715  3.10437  3.17609  2.32808  2.04185  2.83788  5.14170  3.88572    40 t - - -
     2.68618  4.42225  2.77519  2.73123  3.46354  2.40513  3.72494  3.29354  2.67741  2.69355  4.24690  2.90347  2.73739  3.18146  2.89801  2.37887  2.77519  2.98518  4.58477  3.61503
     0.02334  4.16489  4.88723  0.61958  0.77255  0.48576  0.95510
 30  1.54536  4.32235  3.69117  3.14132  3.12556  3.65545  3.96565  2.63925  3.07113  2.29563  3.40296  3.49332  4.06145  3.36662  3.38637  2.93739  2.92073  2.34100  4.87902  3.26599    41 a - - -
     2.68618  4.42225  2.77519  2.73123  3.46354  2.40513  3.72494  3.29354  2.67741  2.69355  4.24690  2.90347  2.73739  3.18146  2.89801  2.37887  2.77519  2.98518  4.58477  3.61503
     0.02334  4.16489  4.88723  0.61958  0.77255  0.48576  0.95510
 31  2.34986  4.62684  2.99459  2.50688  3.81801  3.47446  3.51441  3.21319  2.54920  2.86531  3.71192  3.05899  3.86260  2.72160  2.98512  2.09760  2.83366  2.81798  3.95858  3.31999    42 s - - -
     2.68618  4.42225  2.77519  2.73123  3.46354  2.40513  3.72494  3.29354  2.67741  2.69355  4.24690  2.90347  2.73739  3.18146  2.89801  2.37887  2.77519  2.98518  4.58477  3.61503
     0.02334  4.16489  4.88723  0.61958  0.77255  0.48576  0.95510
 32  1.26981  4.35622  3.43017  2.99955  3.94607  3.26259  4.03514  3.24445  2.96259  2.99591  3.88144  3.31616  3.86701  3.02443  3.30720  2.45914  2.84083  2.52769  5.34269  4.09960    43 a - - -
     2.68618  4.42225  2.77519  2.73123  3.46354  2.40513  3.72494  3.29354  2.67741  2.69355  4.24690  2.90347  2.73739  3.18146  2.89801  2.37887  2.77519  2.98518  4.58477  3.61503
     0.02334  4.16489  4.88723  0.61958  0.77255  0.48576  0.95510
 33  2.75583  4.94353  2.84207  2.48920  4.20564  3.15865  1.73886  3.71696  2.47125  3.29122  4.12715  2.99675  3.90254  2.57710  2.86067  2.46430  3.01458  3.37333  5.44677  4.09170    44 h - - -
     2.68622  4.42229  2.77524  2.73128  3.46358  2.40494  3.72499  3.29330  2.67745  2.69359  4.24593  2.90351  2.73744  3.18151  2.89805  2.37891  2.77524  2.98523  4.58481  3.61507
     0.17761  1.86314  4.88723  0.60588  0.78876  0.48576  0.95510
 34  3.07087  4.80683  3.80366  3.74507  4.86220  0.39323  4.83738  4.54296  3.96555  4.15653  5.14181  3.96927  4.20642  4.26711  4.15990  3.25273  3.57468  4.01458  5.86989  4.97743    47 G - - -
     2.68618  4.42225  2.77519  2.73123  3.46354  2.40513  3.72494  3.29354  2.67741  2.69355  4.24690  2.90347  2.73739  3.18146  2.89801  2.37887  2.77519  2.98518  4.58477  3.61503
     0.02334  4.16489  4.88723  0.61958  0.77255  0.48576  0.95510
 35  2.31850  5.11109  2.55095  1.14426  4.55453  3.34060  3.86723  3.90936  2.73960  3.55159  4.42743  2.93339  3.92439  3.03871  3.20407  2.83745  3.15561  3.54347  5.78105  4.39754    48 e - - -
     2.68618  4.42225  2.77519  2.73123  3.46354  2.40513  3.72494  3.29354  2.67741  2.69355  4.24690  2.90347  2.73739  3.18146  2.89801  2.37887  2.77519  2.98518  4.58477  3.61503
     0.02334  4.16489  4.88723  0.61958  0.77255  0.48576  0.95510
 36  3.18425  5.29258  3.60848  2.91479  4.78379  3.77453  3.68930  4.12029  1.03398  3.53368  4.40869  3.30335  4.13701  2.82139  1.74528  3.17194  3.33985  3.80009  5.54736  4.40030    49 k - - -
     2.68618  4.42225  2.77519  2.73123  3.46354  2.40513  3.72494  3.29354  2.67741  2.69355  4.24690  2.90347  2.73739  3.18146  2.89801  2.37887  2.77519  2.98518  4.58477  3.61503
     0.02334  4.16489  4.88723  0.61958  0.77255  0.48576  0.95510
 37  1.73592  4.24856  3.56099  3.30132  4.43698  3.02097  4.36845  3.81738  3.36006  3.54753  4.38612  3.42239  3.78642  3.64840  3.65699  1.03904  2.80768  3.26110  5.78915  4.57431    50 s - - -
     2.68618  4.42225  2.77519  2.73123  3.46354  2.40513  3.72494  3.29354  2.67741  2.69355  4.24690  2.90347  2.73739  3.18146  2.89801  2.37887  2.77519  2.98518  4.58477  3.61503
     0.02334  4.16489  4.88723  0.61958  0.77255  0.48576  0.95510
 38  2.92339  4.93234  3.20735  2.79171  3.88769  3.60629  3.84399  3.41472  2.48015  2.48543  3.96843  3.24613  4.06362  1.29339  2.77483  2.99013  3.17443  3.22068  5.29209  3.93586    51 q - - -
     2.68618  4.42225  2.77519  2.73123  3.46354  2.40513  3.72494  3.29354  2.67741  2.69355  4.24690  2.90347  2.73739  3.18146  2.89801  2.37887  2.77519  2.98518  4.58477  3.61503
     0.02334  4.16489  4.88723  0.61958  0.77255  0.48576  0.95510
 39  1.98383  5.09528  2.65918  1.67077  4.42341  3.38069  3.71621  3.85868  2.50929  3.41428  4.22906  2.89708  3.86995  2.42642  2.96790  2.74151  3.01744  3.48712  5.60901  4.23130    52 e - - -
     2.68618  4.42225  2.77519  2.73123  3.46354  2.40513  3.72494  3.29354  2.67741  2.69355  4.24690  2.90347  2.73739  3.18146  2.89801  2.37887  2.77519  2.98518  4.58477  3.61503
     0.02334  4.16489  4.88723  0.61958  0.77255  0.48576  0.95510
 40  1.01078  4.30481  3.53135  3.24298  4.32729  3.09769  4.31040  3.65281  3.28130  3.39482  4.27290  3.42331  2.37873  3.58946  3.58836  2.56061  2.85474  3.18462  5.68847  4.47931    53 a - - -
     2.68618  4.42225  2.77519  2.73123  3.46354  2.40513  3.72494  3.29354  2.67741  2.69355  4.24690  2.90347  2.73739  3.18146  2.89801  2.37887  2.77519  2.98518  4.58477  3.61503
```

	0.02334	4.16489	4.88723	0.61958	0.77255	0.48576	0.95510														
41	3.35254	4.70153	4.74022	4.29557	1.09028	4.39831	4.21115	2.15307	4.14776	1.89824	3.27474	4.35056	4.70446	4.24965	4.24808	3.77762	3.60037	2.50910	4.44096	2.86663	54 f - - -
	2.68618	4.42225	2.77519	2.73123	3.46354	2.40513	3.72494	3.29354	2.67741	2.69355	4.24690	2.90347	2.73739	3.18146	2.89801	2.37887	2.77519	2.98518	4.58477	3.61503	
	0.02334	4.16489	4.88723	0.61958	0.77255	0.48576	0.95510														
42	3.41259	4.71823	5.04550	4.47212	3.12063	4.68927	4.98186	1.97970	4.32805	0.93717	1.99423	4.72234	4.83913	4.39552	4.41829	4.03895	3.62792	2.35159	5.23369	4.20135	55 l - - -
	2.68618	4.42225	2.77519	2.73123	3.46354	2.40513	3.72494	3.29354	2.67741	2.69355	4.24690	2.90347	2.73739	3.18146	2.89801	2.37887	2.77519	2.98518	4.58477	3.61503	
	0.02334	4.16489	4.88723	0.61958	0.77255	0.48576	0.95510														
43	3.36714	5.16534	3.89059	3.35258	4.59608	3.78529	4.08066	4.16726	2.41459	3.63009	4.63119	3.68530	4.28143	3.29398	0.61106	3.44264	3.61809	3.88059	5.56480	4.46945	56 R - - -
	2.68618	4.42225	2.77519	2.73123	3.46354	2.40513	3.72494	3.29354	2.67741	2.69355	4.24690	2.90347	2.73739	3.18146	2.89801	2.37887	2.77519	2.98518	4.58477	3.61503	
	0.02334	4.16489	4.88723	0.61958	0.77255	0.48576	0.95510														
44	3.22688	4.73442	4.37915	4.00493	3.37297	4.06173	4.67254	2.57964	3.75443	1.93166	0.91011	4.24321	4.53245	4.12159	3.92326	3.60187	3.54248	2.61796	5.24675	4.03019	57 m - - -
	2.68618	4.42225	2.77519	2.73123	3.46354	2.40513	3.72494	3.29354	2.67741	2.69355	4.24690	2.90347	2.73739	3.18146	2.89801	2.37887	2.77519	2.98518	4.58477	3.61503	
	0.02334	4.16489	4.88723	0.61958	0.77255	0.48576	0.95510														
45	3.36714	5.16534	3.89059	3.35258	4.59608	3.78529	4.08066	4.16726	2.41459	3.63009	4.63119	3.68530	4.28143	3.29398	0.61106	3.44264	3.61809	3.88059	5.56480	4.46945	58 R - - -
	2.68618	4.42225	2.77519	2.73123	3.46354	2.40513	3.72494	3.29354	2.67741	2.69355	4.24690	2.90347	2.73739	3.18146	2.89801	2.37887	2.77519	2.98518	4.58477	3.61503	
	0.02334	4.16489	4.88723	0.61958	0.77255	0.48576	0.95510														
46	2.59973	4.44195	3.72071	3.49953	4.25995	3.26836	4.49028	3.42461	3.44844	3.27730	4.31678	3.65206	3.99891	3.83040	3.68565	2.80533	0.75786	3.08627	5.67051	4.47976	59 t - - -
	2.68618	4.42225	2.77519	2.73123	3.46354	2.40513	3.72494	3.29354	2.67741	2.69355	4.24690	2.90347	2.73739	3.18146	2.89801	2.37887	2.77519	2.98518	4.58477	3.61503	
	0.02334	4.16489	4.88723	0.61958	0.77255	0.48576	0.95510														
47	2.61540	4.39451	4.73261	4.19038	3.51655	4.36190	4.81557	1.27552	4.08153	1.77998	3.35046	4.40249	4.66658	4.30814	4.26231	3.70682	3.30481	1.56170	5.36218	4.18612	60 i - - -
	2.68618	4.42225	2.77519	2.73123	3.46354	2.40513	3.72494	3.29354	2.67741	2.69355	4.24690	2.90347	2.73739	3.18146	2.89801	2.37887	2.77519	2.98518	4.58477	3.61503	
	0.02334	4.16489	4.88723	0.61958	0.77255	0.48576	0.95510														
48	2.64612	4.89132	2.95268	2.43577	4.15448	3.43215	2.58943	3.58672	2.40012	3.16699	3.97449	2.50641	3.83819	2.36223	2.84133	2.66368	2.55568	2.91535	5.37970	4.03613	61 q - - -
	2.68618	4.42225	2.77519	2.73123	3.46354	2.40513	3.72494	3.29354	2.67741	2.69355	4.24690	2.90347	2.73739	3.18146	2.89801	2.37887	2.77519	2.98518	4.58477	3.61503	
	0.02334	4.16489	4.88723	0.61958	0.77255	0.48576	0.95510														
49	3.71335	4.93743	4.81607	4.49860	1.36154	4.47673	3.62109	3.34481	4.30082	2.63835	3.92223	4.22207	4.79024	4.26922	4.30509	3.87309	3.93871	3.29765	1.35972	2.03810	62 w - - -
	2.68618	4.42225	2.77519	2.73123	3.46354	2.40513	3.72494	3.29354	2.67741	2.69355	4.24690	2.90347	2.73739	3.18146	2.89801	2.37887	2.77519	2.98518	4.58477	3.61503	
	0.02334	4.16489	4.88723	0.61958	0.77255	0.48576	0.95510														
50	3.86962	5.04878	4.80881	4.54350	1.46223	4.55891	3.53655	3.53793	4.37862	2.83098	4.12252	4.20925	4.85963	4.29276	4.37262	3.94082	4.08849	3.46921	3.65740	0.85848	63 y - - -
	2.68618	4.42225	2.77519	2.73123	3.46354	2.40513	3.72494	3.29354	2.67741	2.69355	4.24690	2.90347	2.73739	3.18146	2.89801	2.37887	2.77519	2.98518	4.58477	3.61503	
	0.02334	4.16489	4.88723	0.61958	0.77255	0.48576	0.95510														
51	3.33348	5.47501	0.57507	2.56798	4.89825	3.45924	4.22737	4.55940	3.37252	4.11869	5.11310	3.13547	4.13326	3.47379	3.92113	3.26941	3.68157	4.17038	6.00633	4.73552	64 D - - -
	2.68618	4.42225	2.77519	2.73123	3.46354	2.40513	3.72494	3.29354	2.67741	2.69355	4.24690	2.90347	2.73739	3.18146	2.89801	2.37887	2.77519	2.98518	4.58477	3.61503	
	0.02334	4.16489	4.88723	0.61958	0.77255	0.48576	0.95510														
52	2.86341	4.34235	4.27488	3.73909	3.47078	3.98333	4.45315	2.12478	3.61847	1.68244	3.35931	3.99344	4.37820	3.89649	3.85097	3.31733	2.31001	1.46219	5.18377	3.98940	65 v - - -
	2.68618	4.42225	2.77519	2.73123	3.46354	2.40513	3.72494	3.29354	2.67741	2.69355	4.24690	2.90347	2.73739	3.18146	2.89801	2.37887	2.77519	2.98518	4.58477	3.61503	
	0.02334	4.16489	4.88723	0.61958	0.77255	0.48576	0.95510														
53	2.65150	5.12660	2.84149	2.39058	4.46751	3.44181	3.61367	3.91680	2.02878	3.40884	4.18350	2.29841	3.84715	2.16515	2.36468	2.64148	2.93969	3.51847	5.54234	4.18182	66 k - - -
	2.68618	4.42225	2.77519	2.73123	3.46354	2.40513	3.72494	3.29354	2.67741	2.69355	4.24690	2.90347	2.73739	3.18146	2.89801	2.37887	2.77519	2.98518	4.58477	3.61503	
	0.02334	4.16489	4.88723	0.61958	0.77255	0.48576	0.95510														
54	3.69248	4.93163	4.78460	4.45851	1.52137	4.45579	3.63445	3.33056	4.26009	2.62675	3.91090	4.21194	4.77568	4.25144	4.27866	3.85655	3.92050	3.28414	1.23085	2.06010	67 w - - -
	2.68618	4.42225	2.77519	2.73123	3.46354	2.40513	3.72494	3.29354	2.67741	2.69355	4.24690	2.90347	2.73739	3.18146	2.89801	2.37887	2.77519	2.98518	4.58477	3.61503	
	0.02334	4.16489	4.88723	0.61958	0.77255	0.48576	0.95510														
55	2.48543	4.40425	3.49079	3.33109	4.38463	3.13020	4.42700	3.95254	3.42541	3.65336	4.56094	3.50011	3.89528	3.75279	3.69453	0.72247	2.98936	3.42132	5.73499	4.47297	68 s - - -
	2.68608	4.42229	2.77524	2.73127	3.46312	2.40517	3.72499	3.29358	2.67745	2.69359	4.24694	2.90351	2.73721	3.18150	2.89805	2.37891	2.77516	2.98523	4.58481	3.61507	
	0.10953	2.34126	4.88723	0.87908	0.53643	0.48576	0.95510														
56	2.67231	4.83686	3.04608	2.57882	4.31196	3.14498	3.74080	3.71545	1.55415	3.29552	4.11534	3.05330	3.87864	2.89268	2.76828	2.56508	2.18315	3.34491	5.48807	4.18664	72 k - - -
	2.68618	4.42225	2.77519	2.73123	3.46354	2.40513	3.72494	3.29354	2.67741	2.69355	4.24690	2.90347	2.73739	3.18146	2.89801	2.37887	2.77519	2.98518	4.58477	3.61503	
	0.02334	4.16489	4.88723	0.61958	0.77255	0.48576	0.95510														
57	2.48799	5.17363	2.33857	2.02957	4.48603	3.36676	3.64722	3.96118	2.44765	3.46733	4.23831	2.28007	3.82148	2.51000	2.95318	2.66310	2.45477	3.54939	5.62586	4.21135	73 e - - -
	2.68618	4.42225	2.77519	2.73123	3.46354	2.40513	3.72494	3.29354	2.67741	2.69355	4.24690	2.90347	2.73739	3.18146	2.89801	2.37887	2.77519	2.98518	4.58477	3.61503	
	0.02334	4.16489	4.88723	0.61958	0.77255	0.48576	0.95510														
58	2.64958	5.01854	2.95576	2.21715	4.32749	3.43174	3.15226	3.66188	2.05352	3.29706	3.94230	2.92157	3.82087	2.62619	2.30600	2.49624	2.54503	3.39069	5.46281	4.10223	74 k - - -
	2.68618	4.42225	2.77519	2.73123	3.46354	2.40513	3.72494	3.29354	2.67741	2.69355	4.24690	2.90347	2.73739	3.18146	2.89801	2.37887	2.77519	2.98518	4.58477	3.61503	
	0.02334	4.16489	4.88723	0.61958	0.77255	0.48576	0.95510														
59	3.12836	4.47200	4.76585	4.22215	2.72359	4.38339	4.71007	2.04543	4.10105	1.56848	3.18288	4.41719	4.66398	4.27200	4.24725	3.72678	3.36927	1.18938	5.13787	3.89953	75 v - - -
	2.68618	4.42225	2.77519	2.73123	3.46354	2.40513	3.72494	3.29354	2.67741	2.69355	4.24690	2.90347	2.73739	3.18146	2.89801	2.37887	2.77519	2.98518	4.58477	3.61503	
	0.02334	4.16489	4.88723	0.61958	0.77255	0.48576	0.95510														
60	2.45922	5.06609	2.85010	2.37787	4.39785	3.41444	3.63285	3.84604	1.85308	3.36625	4.14794	2.26277	3.41741	2.53438	2.78089	2.59566	2.92444	3.45761	5.52715	4.16016	76 k - - -
	2.68618	4.42225	2.77519	2.73123	3.46354	2.40513	3.72494	3.29354	2.67741	2.69355	4.24690	2.90347	2.73739	3.18146	2.89801	2.37887	2.77519	2.98518	4.58477	3.61503	
	0.02334	4.16489	4.88723	0.61958	0.77255	0.48576	0.95510														
61	2.82509	4.33692	4.21152	3.70750	3.56722	3.90479	4.46404	1.98281	3.59352	2.33620	3.47081	3.95417	3.33651	3.89243	3.83794	3.25945	3.11522	1.09958	5.25181	4.03442	77 v - - -
	2.68618	4.42225	2.77519	2.73123	3.46354	2.40513	3.72494	3.29354	2.67741	2.69355	4.24690	2.90347	2.73739	3.18146	2.89801	2.37887	2.77519	2.98518	4.58477	3.61503	
	0.02334	4.16489	4.88723	0.61958	0.77255	0.48576	0.95510														
62	2.68622	4.79514	2.75027	2.61639	4.57338	1.85190	4.04026	4.11219	2.96667	3.70444	4.54659	1.36580	3.89566	3.24647	3.39782	2.74882	3.08648	3.61931	5.81443	4.47591	78 n - - -
	2.68618	4.42225	2.77519	2.73123	3.46354	2.40513	3.72494	3.29354	2.67741	2.69355	4.24690	2.90347	2.73739	3.18146	2.89801	2.37887	2.77519	2.98518	4.58477	3.61503	
	0.02334	4.16489	4.88723	0.61958	0.77255	0.48576	0.95510														

63	3.06603	5.67994	1.45983	1.26293	4.96494	3.30380	3.86529	4.50064	2.90511	3.98431	4.84165	2.76146	3.93686	3.03276	3.51937	2.93555	3.35815	4.05979	6.12735	4.60409	79 e - - -
	2.68618	4.42225	2.77519	2.73123	3.46354	2.40513	3.72494	3.29354	2.67741	2.69355	4.24690	2.90347	2.73739	3.18146	2.89801	2.37887	2.77519	2.98518	4.58477	3.61503	
	0.02334	4.16489	4.88723	0.61958	0.77255	0.48576	0.95510														
64	2.78199	5.20545	2.22351	1.62470	4.52846	3.34560	3.72217	3.99039	2.58385	3.52614	4.32664	2.83710	3.85739	2.86180	3.10110	2.63443	2.10091	3.59244	5.70734	4.28786	80 e - - -
	2.68618	4.42225	2.77519	2.73123	3.46354	2.40513	3.72494	3.29354	2.67741	2.69355	4.24690	2.90347	2.73739	3.18146	2.89801	2.37887	2.77519	2.98518	4.58477	3.61503	
	0.02334	4.16489	4.88723	0.61958	0.77255	0.48576	0.95510														
65	3.12009	4.48236	4.73820	4.16080	2.51753	4.32843	4.61566	2.25712	4.03145	1.50329	1.71486	4.35927	4.58192	4.15013	4.14488	3.65115	3.34466	1.90633	5.02118	3.88355	81 l - - -
	2.68618	4.42225	2.77519	2.73123	3.46354	2.40513	3.72494	3.29354	2.67741	2.69355	4.24690	2.90347	2.73739	3.18146	2.89801	2.37887	2.77519	2.98518	4.58477	3.61503	
	0.02334	4.16489	4.88723	0.61958	0.77255	0.48576	0.95510														
66	2.60857	4.73151	2.77850	2.25299	3.96493	3.44272	3.67474	3.10759	2.50117	2.99337	3.82422	3.00417	3.84403	2.84410	2.95465	2.29859	2.36497	2.58664	5.26559	3.94694	82 e - - -
	2.68618	4.42225	2.77519	2.73123	3.46354	2.40513	3.72494	3.29354	2.67741	2.69355	4.24690	2.90347	2.73739	3.18146	2.89801	2.37887	2.77519	2.98518	4.58477	3.61503	
	0.02334	4.16489	4.88723	0.61958	0.77255	0.48576	0.95510														
67	2.97719	3.04586	4.78274	4.22507	3.43739	4.27536	4.70443	1.49700	4.09570	1.73376	3.31132	4.36572	4.58771	4.28724	4.22007	3.61507	3.22136	1.47631	5.20728	4.02992	83 v - - -
	2.68618	4.42225	2.77519	2.73123	3.46354	2.40513	3.72494	3.29354	2.67741	2.69355	4.24690	2.90347	2.73739	3.18146	2.89801	2.37887	2.77519	2.98518	4.58477	3.61503	
	0.02334	4.16489	4.88723	0.61958	0.77255	0.48576	0.95510														
68	2.42687	4.39428	3.32457	2.96479	4.22495	3.16449	4.08135	3.59914	2.86488	3.29319	4.13878	3.27221	3.82557	3.31594	3.31604	1.57098	1.60441	3.16801	5.55770	4.30501	84 s - - -
	2.68618	4.42225	2.77519	2.73123	3.46354	2.40513	3.72494	3.29354	2.67741	2.69355	4.24690	2.90347	2.73739	3.18146	2.89801	2.37887	2.77519	2.98518	4.58477	3.61503	
	0.02334	4.16489	4.88723	0.61958	0.77255	0.48576	0.95510														
69	3.07087	4.80683	3.80366	3.74507	4.86220	0.39323	4.83738	4.54296	3.96555	4.15653	5.14181	3.96927	4.20642	4.26711	4.15990	3.25273	3.57468	4.01458	5.86989	4.97743	85 G - - -
	2.68618	4.42225	2.77519	2.73123	3.46354	2.40513	3.72494	3.29354	2.67741	2.69355	4.24690	2.90347	2.73739	3.18146	2.89801	2.37887	2.77519	2.98518	4.58477	3.61503	
	0.02334	4.16489	4.88723	0.61958	0.77255	0.48576	0.95510														
70	3.26687	5.21885	3.46078	3.04013	4.67338	3.71239	3.95890	4.12415	0.69708	3.63003	4.58538	3.44777	4.19999	3.14070	2.53526	3.29544	3.50296	3.81902	5.61432	4.47465	86 k - - -
	2.68618	4.42225	2.77519	2.73123	3.46354	2.40513	3.72494	3.29354	2.67741	2.69355	4.24690	2.90347	2.73739	3.18146	2.89801	2.37887	2.77519	2.98518	4.58477	3.61503	
	0.02334	4.16489	4.88723	0.61958	0.77255	0.48576	0.95510														
71	3.10062	4.45987	4.70258	4.15844	1.68490	4.30085	4.50044	2.12010	4.02807	1.79376	3.16393	4.31815	4.59288	4.17948	4.15858	3.63569	3.33843	1.56264	4.88981	3.54680	87 v - - -
	2.68618	4.42225	2.77519	2.73123	3.46354	2.40513	3.72494	3.29354	2.67741	2.69355	4.24690	2.90347	2.73739	3.18146	2.89801	2.37887	2.77519	2.98518	4.58477	3.61503	
	0.02334	4.16489	4.88723	0.61958	0.77255	0.48576	0.95510														
72	2.78219	4.49862	3.59690	3.04856	2.98498	3.71906	1.88174	2.88295	2.91998	2.35825	2.50372	3.43327	4.09391	3.27106	3.24377	2.98516	3.01465	2.72468	4.69660	3.25658	88 h - - -
	2.68618	4.42225	2.77519	2.73123	3.46354	2.40513	3.72494	3.29354	2.67741	2.69355	4.24690	2.90347	2.73739	3.18146	2.89801	2.37887	2.77519	2.98518	4.58477	3.61503	
	0.02334	4.16489	4.88723	0.61958	0.77255	0.48576	0.95510														
73	3.03126	4.37869	4.67870	4.13703	3.55748	4.33012	4.78761	1.65804	4.02887	1.91259	3.39886	4.35850	4.64567	4.27381	4.22456	3.67249	2.80876	1.14575	5.37375	4.18380	89 v - - -
	2.68618	4.42225	2.77519	2.73123	3.46354	2.40513	3.72494	3.29354	2.67741	2.69355	4.24690	2.90347	2.73739	3.18146	2.89801	2.37887	2.77519	2.98518	4.58477	3.61503	
	0.02334	4.16489	4.88723	0.61958	0.77255	0.48576	0.95510														
74	2.55034	4.34850	4.38888	3.81897	1.75462	4.04358	4.30158	2.38459	3.69189	1.69370	2.42535	4.02988	4.36483	3.87679	3.85500	3.35081	3.14773	2.35058	4.82904	3.59119	90 l - - -
	2.68618	4.42225	2.77519	2.73123	3.46354	2.40513	3.72494	3.29354	2.67741	2.69355	4.24690	2.90347	2.73739	3.18146	2.89801	2.37887	2.77519	2.98518	4.58477	3.61503	
	0.02334	4.16489	4.88723	0.61958	0.77255	0.48576	0.95510														
75	2.34364	5.02902	2.63206	2.00985	4.35410	3.37090	3.66558	3.80577	2.43567	3.35246	4.14103	2.88378	2.72705	2.79681	2.96139	2.13753	2.92208	3.42184	5.54286	4.15855	91 e - - -
	2.68618	4.42225	2.77519	2.73123	3.46354	2.40513	3.72494	3.29354	2.67741	2.69355	4.24690	2.90347	2.73739	3.18146	2.89801	2.37887	2.77519	2.98518	4.58477	3.61503	
	0.02334	4.16489	4.88723	0.61958	0.77255	0.48576	0.95510														
76	2.27713	4.81312	2.67060	2.29138	4.08427	2.50127	3.66977	3.06260	2.48529	3.10518	3.92155	2.96417	3.83209	2.82419	2.95108	2.65258	2.53082	3.16537	5.35141	4.01416	92 a - - -
	2.68618	4.42225	2.77519	2.73123	3.46354	2.40513	3.72494	3.29354	2.67741	2.69355	4.24690	2.90347	2.73739	3.18146	2.89801	2.37887	2.77519	2.98518	4.58477	3.61503	
	0.02334	4.16489	4.88723	0.61958	0.77255	0.48576	0.95510														
77	3.86835	5.12987	4.56789	4.35347	3.02935	4.08352	4.30963	3.91661	4.07628	3.25628	4.53479	4.44682	4.62737	4.44811	4.14946	4.06898	4.18585	3.81445	0.45737	3.01784	93 W - - -
	2.68618	4.42225	2.77519	2.73123	3.46354	2.40513	3.72494	3.29354	2.67741	2.69355	4.24690	2.90347	2.73739	3.18146	2.89801	2.37887	2.77519	2.98518	4.58477	3.61503	
	0.02334	4.16489	4.88723	0.61958	0.77255	0.48576	0.95510														
78	3.18863	4.88603	3.82888	3.71728	4.67093	3.55752	4.76170	4.30328	3.80198	3.89323	4.96207	3.98991	0.42795	4.18131	4.00541	3.36743	3.65485	3.91304	5.76614	4.79867	94 P - - -
	2.68618	4.42225	2.77519	2.73123	3.46354	2.40513	3.72494	3.29354	2.67741	2.69355	4.24690	2.90347	2.73739	3.18146	2.89801	2.37887	2.77519	2.98518	4.58477	3.61503	
	0.02334	4.16489	4.88723	0.61958	0.77255	0.48576	0.95510														
79	2.64107	4.96185	2.95118	1.93333	4.24529	3.43002	3.16143	3.34697	2.34164	3.23346	4.02252	2.93026	3.82306	2.30774	2.58769	2.64046	2.86932	3.04713	5.42364	4.06846	95 e - - -
	2.68618	4.42225	2.77519	2.73123	3.46354	2.40513	3.72494	3.29354	2.67741	2.69355	4.24690	2.90347	2.73739	3.18146	2.89801	2.37887	2.77519	2.98518	4.58477	3.61503	
	0.02334	4.16489	4.88723	0.61958	0.77255	0.48576	0.95510														
80	2.02788	4.73502	2.94382	2.04104	4.18289	3.33413	3.76641	3.58611	2.59658	3.20846	4.02838	3.01191	3.83420	2.93189	3.04640	2.33092	2.17667	3.22309	5.45150	4.12483	96 a - - -
	2.68618	4.42225	2.77519	2.73123	3.46354	2.40513	3.72494	3.29354	2.67741	2.69355	4.24690	2.90347	2.73739	3.18146	2.89801	2.37887	2.77519	2.98518	4.58477	3.61503	
	0.02334	4.16489	4.88723	0.61958	0.77255	0.48576	0.95510														
81	3.20249	4.48763	4.96945	4.45237	3.59775	4.60525	5.12354	1.58169	4.34286	1.87945	3.39533	4.66842	4.87147	4.57854	4.52343	3.98236	3.45563	0.97596	5.58145	4.39445	97 v - - -
	2.68618	4.42225	2.77519	2.73123	3.46354	2.40513	3.72494	3.29354	2.67741	2.69355	4.24690	2.90347	2.73739	3.18146	2.89801	2.37887	2.77519	2.98518	4.58477	3.61503	
	0.02334	4.16489	4.88723	0.61958	0.77255	0.48576	0.95510														
82	2.08764	5.01457	2.16748	2.34983	4.43336	2.86612	3.71673	3.89414	2.55245	3.43789	4.22747	2.59942	2.63807	2.85363	3.05255	2.66475	2.95000	3.48446	5.62347	4.23223	98 a - - -
	2.68618	4.42225	2.77519	2.73123	3.46354	2.40513	3.72494	3.29354	2.67741	2.69355	4.24690	2.90347	2.73739	3.18146	2.89801	2.37887	2.77519	2.98518	4.58477	3.61503	
	0.02334	4.16489	4.88723	0.61958	0.77255	0.48576	0.95510														
83	2.62738	4.80158	3.00411	2.33024	2.90792	3.44567	3.65380	3.44941	1.99311	3.00479	3.87887	2.62147	3.67145	2.81064	2.88464	2.66642	2.64386	3.13341	5.30432	3.97738	99 k - - -
	2.68618	4.42225	2.77519	2.73123	3.46354	2.40513	3.72494	3.29354	2.67741	2.69355	4.24690	2.90347	2.73739	3.18146	2.89801	2.37887	2.77519	2.98518	4.58477	3.61503	
	0.02334	4.16489	4.88723	0.61958	0.77255	0.48576	0.95510														
84	2.38059	4.35229	3.34057	3.05023	4.36958	3.09169	4.19114	3.78030	3.11134	3.45697	4.29188	3.30641	1.37337	3.43449	3.45221	1.94538	2.57139	3.27346	5.69486	4.45054	100 p - - -
	2.68618	4.42225	2.77519	2.73123	3.46354	2.40513	3.72494	3.29354	2.67741	2.69355	4.24690	2.90347	2.73739	3.18146	2.89801	2.37887	2.77519	2.98518	4.58477	3.61503	
	0.02334	4.16489	4.88723	0.61958	0.77255	0.48576	0.95510														
85	2.74748	5.21981	2.41377	1.65439	4.53524	2.50382	3.66555	4.01078	2.33819	3.51112	4.28893	2.83984	3.83917	2.78851	2.66870	2.69737	2.99096	3.59850	5.66438	4.24955	101 e - - -

```
       2.68618  4.42225  2.77519  2.73123  3.46354  2.40513  3.72494  3.29354  2.67741  2.69355  4.24690  2.90347  2.73739  3.18146  2.89801  2.37887  2.77519  2.98518  4.58477  3.61503
       0.02334  4.16489  4.88723  0.61958  0.77255  0.48576  0.95510
 86    2.38097  4.20922  3.95778  3.41047  3.44923  3.68376  4.14884  1.86843  3.32547  2.43160  3.40581  3.67672  4.12067  3.59479  3.58537  2.47440  2.69403  1.72440  4.98763  3.78033    102 v - - -
       2.68618  4.42225  2.77519  2.73123  3.46354  2.40513  3.72494  3.29354  2.67741  2.69355  4.24690  2.90347  2.73739  3.18146  2.89801  2.37887  2.77519  2.98518  4.58477  3.61503
       0.02334  4.16489  4.88723  0.61958  0.77255  0.48576  0.95510
 87    1.93512  4.33167  3.45817  3.00303  4.00332  2.90603  4.04444  2.81621  2.98803  3.06898  3.92805  3.30549  3.83267  3.30313  3.34985  1.45961  2.80615  2.97549  5.38291  4.14051    103 s - - -
       2.68618  4.42225  2.77519  2.73123  3.46354  2.40513  3.72494  3.29354  2.67741  2.69355  4.24690  2.90347  2.73739  3.18146  2.89801  2.37887  2.77519  2.98518  4.58477  3.61503
       0.02334  4.16489  4.88723  0.61958  0.77255  0.48576  0.95510
 88    3.75093  4.97771  4.75667  4.46427  0.79991  4.50000  3.60427  3.35253  4.30308  2.65022  3.95258  4.20611  4.81145  4.26361  4.32022  3.88655  3.97785  3.30961  3.73850  1.75164    104 f - - -
       2.68618  4.42225  2.77519  2.73123  3.46354  2.40513  3.72494  3.29354  2.67741  2.69355  4.24690  2.90347  2.73739  3.18146  2.89801  2.37887  2.77519  2.98518  4.58477  3.61503
       0.02334  4.16489  4.88723  0.61958  0.77255  0.48576  0.95510
 89    3.41684  4.71632  5.05415  4.49591  2.41629  4.67352  4.85686  2.00271  4.36353  0.84735  2.91814  4.70253  4.83290  4.40232  4.43684  4.02532  3.63295  2.38659  5.07803  3.88940    105 l - - -
       2.68618  4.42225  2.77519  2.73123  3.46354  2.40513  3.72494  3.29354  2.67741  2.69355  4.24690  2.90347  2.73739  3.18146  2.89801  2.37887  2.77519  2.98518  4.58477  3.61503
       0.02334  4.16489  4.88723  0.61958  0.77255  0.48576  0.95510
 90    2.91580  5.11872  2.66847  2.48929  4.08036  3.41069  3.01735  4.00785  2.65783  3.53938  4.42225  1.20117  3.97408  3.05413  3.04977  2.90510  3.20810  3.64038  5.42593  3.94385    106 n - - -
       2.68618  4.42225  2.77519  2.73123  3.46354  2.40513  3.72494  3.29354  2.67741  2.69355  4.24690  2.90347  2.73739  3.18146  2.89801  2.37887  2.77519  2.98518  4.58477  3.61503
       0.02334  4.16489  4.88723  0.61958  0.77255  0.48576  0.95510
 91    2.34455  4.12246  4.26970  3.68641  2.40707  3.83114  4.13229  1.87044  3.55210  2.14989  3.17218  3.85248  4.18734  3.75040  3.69814  3.13037  2.92538  1.95456  4.72822  3.04892    107 i - - -
       2.68618  4.42225  2.77519  2.73123  3.46354  2.40513  3.72494  3.29354  2.67741  2.69355  4.24690  2.90347  2.73739  3.18146  2.89801  2.37887  2.77519  2.98518  4.58477  3.61503
       0.02334  4.16489  4.88723  0.61958  0.77255  0.48576  0.95510
 92    2.49349  4.34344  3.44377  3.02470  2.41470  1.79629  3.99805  3.21282  3.04128  2.90980  3.80225  3.33913  3.89199  3.33730  3.38739  2.12800  2.85575  2.90016  5.12088  3.78921    108 g - - -
       2.68618  4.42225  2.77519  2.73123  3.46354  2.40513  3.72494  3.29354  2.67741  2.69355  4.24690  2.90347  2.73739  3.18146  2.89801  2.37887  2.77519  2.98518  4.58477  3.61503
       0.02334  4.16489  4.88723  0.61958  0.77255  0.48576  0.95510
 93    2.67938  4.33149  3.63752  2.39994  3.48925  3.70014  3.97955  2.02897  3.01807  2.45495  3.14647  3.46750  4.07424  3.32417  3.35555  2.95949  2.58211  1.95430  4.99796  3.76954    109 v - - -
       2.68618  4.42225  2.77519  2.73123  3.46354  2.40513  3.72494  3.29354  2.67741  2.69355  4.24690  2.90347  2.73739  3.18146  2.89801  2.37887  2.77519  2.98518  4.58477  3.61503
       0.02334  4.16489  4.88723  0.61958  0.77255  0.48576  0.95510
 94    3.18863  4.88603  3.82888  3.71728  4.67093  3.55752  4.76170  4.30328  3.80198  3.89323  4.96207  3.98991  0.42795  4.18131  4.00541  3.36743  3.65485  3.91304  5.76614  4.79867    110 P - - -
       2.68618  4.42225  2.77519  2.73123  3.46354  2.40513  3.72494  3.29354  2.67741  2.69355  4.24690  2.90347  2.73739  3.18146  2.89801  2.37887  2.77519  2.98518  4.58477  3.61503
       0.02334  4.16489  4.88723  0.61958  0.77255  0.48576  0.95510
 95    3.07087  4.80683  3.80366  3.74507  4.86220  0.39323  4.83738  4.54296  3.96555  4.15653  5.14181  3.96927  4.20642  4.26711  4.15990  3.25273  3.57468  4.01458  5.86989  4.97743    111 G - - -
       2.68618  4.42225  2.77519  2.73123  3.46354  2.40513  3.72494  3.29354  2.67741  2.69355  4.24690  2.90347  2.73739  3.18146  2.89801  2.37887  2.77519  2.98518  4.58477  3.61503
       0.02334  4.16489  4.88723  0.61958  0.77255  0.48576  0.95510
 96    3.18863  4.88603  3.82888  3.71728  4.67093  3.55752  4.76170  4.30328  3.80198  3.89323  4.96207  3.98991  0.42795  4.18131  4.00541  3.36743  3.65485  3.91304  5.76614  4.79867    112 P - - -
       2.68618  4.42225  2.77519  2.73123  3.46354  2.40513  3.72494  3.29354  2.67741  2.69355  4.24690  2.90347  2.73739  3.18146  2.89801  2.37887  2.77519  2.98518  4.58477  3.61503
       0.02334  4.16489  4.88723  0.61958  0.77255  0.48576  0.95510
 97    2.65388  4.52820  3.31310  2.77936  3.74979  3.52096  3.78985  3.09185  2.58995  2.79049  3.67516  3.21341  3.93970  2.59593  2.61883  2.55330  2.90025  1.76880  5.11835  3.86444    113 v - - -
       2.68618  4.42225  2.77519  2.73123  3.46354  2.40513  3.72494  3.29354  2.67741  2.69355  4.24690  2.90347  2.73739  3.18146  2.89801  2.37887  2.77519  2.98518  4.58477  3.61503
       0.02334  4.16489  4.88723  0.61958  0.77255  0.48576  0.95510
 98    3.19717  4.53671  4.86469  4.28564  2.05223  4.42046  4.68282  2.27383  4.15158  1.15498  2.56079  4.46854  4.64639  4.23143  4.23564  3.74961  3.41758  2.01993  5.02079  3.88263    114 l - - -
       2.68618  4.42225  2.77519  2.73123  3.46354  2.40513  3.72494  3.29354  2.67741  2.69355  4.24690  2.90347  2.73739  3.18146  2.89801  2.37887  2.77519  2.98518  4.58477  3.61503
       0.02334  4.16489  4.88723  0.61958  0.77255  0.48576  0.95510
 99    3.23387  4.50575  5.04694  4.51797  3.50470  4.64564  5.12622  1.30021  4.41019  1.58371  3.29265  4.72001  4.88132  4.59500  4.55642  4.01857  3.47864  1.35879  5.51443  4.36755    115 i - - -
       2.68618  4.42225  2.77519  2.73123  3.46354  2.40513  3.72494  3.29354  2.67741  2.69355  4.24690  2.90347  2.73739  3.18146  2.89801  2.37887  2.77519  2.98518  4.58477  3.61503
       0.02334  4.16489  4.88723  0.61958  0.77255  0.48576  0.95510
100    3.21882  5.25214  3.67805  2.99330  4.73034  3.78914  3.72480  4.07985  1.77232  3.51405  4.41051  3.35919  4.16664  2.86775  0.98092  3.22490  3.38107  3.77521  5.53220  4.39683    116 r - - -
       2.68618  4.42225  2.77519  2.73123  3.46354  2.40513  3.72494  3.29354  2.67741  2.69355  4.24690  2.90347  2.73739  3.18146  2.89801  2.37887  2.77519  2.98518  4.58477  3.61503
       0.02334  4.16489  4.88723  0.61958  0.77255  0.48576  0.95510
101    2.29418  4.92260  2.97526  2.39746  4.24515  3.44628  3.67025  3.65133  1.74145  3.22772  4.04247  2.99099  3.86938  2.76479  2.75715  2.71302  2.31785  3.30771  5.43135  4.10855    117 k - - -
       2.68618  4.42225  2.77519  2.73123  3.46354  2.40513  3.72494  3.29354  2.67741  2.69355  4.24690  2.90347  2.73739  3.18146  2.89801  2.37887  2.77519  2.98518  4.58477  3.61503
       0.02334  4.16489  4.88723  0.61958  0.77255  0.48576  0.95510
102    1.92654  4.90745  2.69634  1.72350  4.41152  2.35652  3.80910  3.83744  2.67158  3.43113  4.25045  2.94644  3.85588  2.97101  3.14516  2.70305  2.99066  3.43884  5.64400  4.28144    118 e - - -
       2.68618  4.42225  2.77519  2.73123  3.46354  2.40513  3.72494  3.29354  2.67741  2.69355  4.24690  2.90347  2.73739  3.18146  2.89801  2.37887  2.77519  2.98518  4.58477  3.61503
       0.02334  4.16489  4.88723  0.61958  0.77255  0.48576  0.95510
103    2.43141  4.67138  3.11019  2.55835  3.90918  3.45652  3.69379  2.83155  2.50857  2.94249  3.78258  3.05024  3.85911  2.53661  2.67018  2.09669  2.60182  3.00697  5.22279  3.92297    119 s - - -
       2.68618  4.42225  2.77519  2.73123  3.46354  2.40513  3.72494  3.29354  2.67741  2.69355  4.24690  2.90347  2.73739  3.18146  2.89801  2.37887  2.77519  2.98518  4.58477  3.61503
       0.02334  4.16489  4.88723  0.61958  0.77255  0.48576  0.95510
104    3.29617  4.61156  4.63881  4.20313  1.67322  4.24165  3.64855  3.07571  4.04150  2.58788  3.70045  4.06310  4.56440  4.07040  4.08927  3.56996  3.51892  2.44741  1.91438  1.69514    120 f - - -
       2.68618  4.42225  2.77519  2.73123  3.46354  2.40513  3.72494  3.29354  2.67741  2.69355  4.24690  2.90347  2.73739  3.18146  2.89801  2.37887  2.77519  2.98518  4.58477  3.61503
       0.02334  4.16489  4.88723  0.61958  0.77255  0.48576  0.95510
105    3.19471  4.48264  4.98799  4.43938  3.41106  4.56300  4.98962  1.30884  4.32760  1.65253  2.73998  4.63179  4.79995  4.48347  4.45480  3.91850  3.43233  1.48036  5.38213  4.25728    121 i - - -
       2.68618  4.42225  2.77519  2.73123  3.46354  2.40513  3.72494  3.29354  2.67741  2.69355  4.24690  2.90347  2.73739  3.18146  2.89801  2.37887  2.77519  2.98518  4.58477  3.61503
       0.02334  4.16489  4.88723  0.61958  0.77255  0.48576  0.95510
106    2.66776  4.76343  2.77512  2.64111  4.56973  1.53758  4.05665  4.10557  2.98744  3.70223  4.54385  1.61002  3.89176  3.26625  3.41384  2.73790  3.07513  3.60660  5.81519  4.48356    122 g - - -
       2.68618  4.42225  2.77519  2.73123  3.46354  2.40513  3.72494  3.29354  2.67741  2.69355  4.24690  2.90347  2.73739  3.18146  2.89801  2.37887  2.77519  2.98518  4.58477  3.61503
       0.02334  4.16489  4.88723  0.61958  0.77255  0.48576  0.95510
107    2.90551  5.27532  2.31316  1.68723  4.68516  1.52673  3.85548  4.16494  2.80726  3.71163  4.55350  2.85241  3.91485  3.02534  3.33663  2.85311  3.20650  3.75472  5.88420  4.45895    123 g - - -
       2.68618  4.42225  2.77519  2.73123  3.46354  2.40513  3.72494  3.29354  2.67741  2.69355  4.24690  2.90347  2.73739  3.18146  2.89801  2.37887  2.77519  2.98518  4.58477  3.61503
```

```
      0.02334  4.16489  4.88723  0.61958  0.77255  0.48576  0.95510
108   2.74313  4.99341  3.06861  2.51230  4.29859  3.50174  3.64074  3.70825  1.85101  3.25350  3.39370  2.92492  3.89308  1.87083  2.60579  2.74970  2.87324  3.36653  5.42910  4.11999    124 k - - -
      2.68618  4.42225  2.77519  2.73123  3.46354  2.40513  3.72494  3.29354  2.67741  2.69355  4.24690  2.90347  2.73739  3.18146  2.89801  2.37887  2.77519  2.98518  4.58477  3.61503
      0.02334  4.16489  4.88723  0.61958  0.77255  0.48576  0.95510
109   2.76405  4.29787  3.94252  3.38906  2.30614  3.79526  3.89778  2.68555  3.29364  1.82825  3.32383  3.65235  2.54569  3.53509  3.53682  3.07683  2.99619  2.52951  4.50359  2.57247    125 l - - -
      2.68618  4.42225  2.77519  2.73123  3.46354  2.40513  3.72494  3.29354  2.67741  2.69355  4.24690  2.90347  2.73739  3.18146  2.89801  2.37887  2.77519  2.98518  4.58477  3.61503
      0.02334  4.16489  4.88723  0.61958  0.77255  0.48576  0.95510
110   2.33410  4.11822  4.20982  3.62760  2.63278  3.79140  4.07248  2.42258  3.49251  2.08018  3.17280  3.80028  4.15332  3.69651  3.64671  3.08833  2.90624  1.62208  3.54530  3.42832    126 v - - -
      2.68619  4.42226  2.77521  2.73125  3.46355  2.40514  3.72496  3.29355  2.67742  2.69356  4.24691  2.90348  2.73741  3.18113  2.89802  2.37888  2.77521  2.98520  4.58478  3.61504
      0.10953  2.34126  4.88723  0.36770  1.17871  0.48576  0.95510
111   2.63595  4.29151  3.68164  3.13716  2.50415  3.61933  3.96668  2.44331  3.06891  2.39562  3.38704  3.48128  2.17491  3.36130  3.37809  2.90734  2.58752  2.48200  4.89524  3.63996    128 p - - -
      2.68618  4.42225  2.77519  2.73123  3.46354  2.40513  3.72494  3.29354  2.67741  2.69355  4.24690  2.90347  2.73739  3.18146  2.89801  2.37887  2.77519  2.98518  4.58477  3.61503
      0.02334  4.16489  4.88723  0.61958  0.77255  0.48576  0.95510
112   2.77084  4.99229  3.01464  2.56052  4.42392  3.45379  3.71108  3.86440  2.29812  3.39368  4.21271  2.09711  3.91467  2.85340  1.69675  2.46538  3.02549  3.48837  5.52806  4.22164    129 r - - -
      2.68618  4.42225  2.77519  2.73123  3.46354  2.40513  3.72494  3.29354  2.67741  2.69355  4.24690  2.90347  2.73739  3.18146  2.89801  2.37887  2.77519  2.98518  4.58477  3.61503
      0.02334  4.16489  4.88723  0.61958  0.77255  0.48576  0.95510
113   2.02179  4.26760  3.51420  3.29039  4.45052  3.02666  4.37915  3.84086  3.36677  3.57509  4.42458  3.42048  3.79694  3.66307  3.65755  0.91257  2.82923  3.28339  5.80601  4.57999    130 s - - -
      2.68618  4.42225  2.77519  2.73123  3.46354  2.40513  3.72494  3.29354  2.67741  2.69355  4.24690  2.90347  2.73739  3.18146  2.89801  2.37887  2.77519  2.98518  4.58477  3.61503
      0.02334  4.16489  4.88723  0.61958  0.77255  0.48576  0.95510
114   2.80756  4.02395  4.32162  3.77544  3.49656  3.93862  4.44256  2.08943  3.66015  2.29840  2.85679  3.99328  4.34822  3.92158  3.87804  3.27406  2.56783  1.18594  5.15931  3.96167    131 v - - -
      2.68618  4.42225  2.77519  2.73123  3.46354  2.40513  3.72494  3.29354  2.67741  2.69355  4.24690  2.90347  2.73739  3.18146  2.89801  2.37887  2.77519  2.98518  4.58477  3.61503
      0.02334  4.16489  4.88723  0.61958  0.77255  0.48576  0.95510
115   2.42790  4.43033  3.35321  2.80094  3.67368  3.48056  3.81960  2.67901  2.73900  2.72146  3.60498  3.22670  3.91150  3.08160  2.75614  1.89125  2.78362  2.40881  5.07864  3.82444    132 s - - -
      2.68618  4.42225  2.77519  2.73123  3.46354  2.40513  3.72494  3.29354  2.67741  2.69355  4.24690  2.90347  2.73739  3.18146  2.89801  2.37887  2.77519  2.98518  4.58477  3.61503
      0.02334  4.16489  4.88723  0.61958  0.77255  0.48576  0.95510
116   3.06446  4.48935  4.51392  3.96522  3.30988  4.22928  4.62715  2.04938  3.82026  1.04077  3.14462  4.22666  4.54785  4.05749  4.01710  3.56456  2.81522  2.01823  5.20101  4.06191    133 l - - -
      2.68618  4.42225  2.77519  2.73123  3.46354  2.40513  3.72494  3.29354  2.67741  2.69355  4.24690  2.90347  2.73739  3.18146  2.89801  2.37887  2.77519  2.98518  4.58477  3.61503
      0.02334  4.16489  4.88723  0.61958  0.77255  0.48576  0.95510
117   2.78433  5.15556  2.74010  1.45917  4.46737  3.42588  3.67346  3.90581  2.17511  3.42830  4.23280  2.91176  3.87908  2.53962  2.79496  2.75274  3.01867  3.23443  5.59070  4.22597    134 e - - -
      2.68618  4.42225  2.77519  2.73123  3.46354  2.40513  3.72494  3.29354  2.67741  2.69355  4.24690  2.90347  2.73739  3.18146  2.89801  2.37887  2.77519  2.98518  4.58477  3.61503
      0.02334  4.16489  4.88723  0.61958  0.77255  0.48576  0.95510
118   2.65595  4.32792  3.60268  3.03527  3.43507  3.64979  3.91142  2.31024  2.54216  1.79641  3.38581  3.41878  3.10914  3.25879  3.23894  2.91165  2.88949  2.42400  4.92962  3.70769    135 l - - -
      2.68618  4.42225  2.77519  2.73123  3.46354  2.40513  3.72494  3.29354  2.67741  2.69355  4.24690  2.90347  2.73739  3.18146  2.89801  2.37887  2.77519  2.98518  4.58477  3.61503
      0.02334  4.16489  4.88723  0.61958  0.77255  0.48576  0.95510
119   3.07087  4.80683  3.80366  3.74507  4.86220  0.39323  4.83738  4.54296  3.96555  4.15653  5.14181  3.96927  4.20642  4.26711  4.15990  3.25273  3.57468  4.01458  5.86989  4.97743    136 G - - -
      2.68618  4.42225  2.77519  2.73123  3.46354  2.40513  3.72494  3.29354  2.67741  2.69355  4.24690  2.90347  2.73739  3.18146  2.89801  2.37887  2.77519  2.98518  4.58477  3.61503
      0.02334  4.16489  4.88723  0.61958  0.77255  0.48576  0.95510
120   2.78549  5.29201  2.28509  1.91817  4.60576  2.25446  3.68927  4.09255  2.06781  3.58458  4.36550  2.58051  3.84794  2.81588  3.03362  2.72247  3.03487  3.67052  5.73498  4.30070    137 e - - -
      2.68618  4.42225  2.77519  2.73123  3.46354  2.40513  3.72494  3.29354  2.67741  2.69355  4.24690  2.90347  2.73739  3.18146  2.89801  2.37887  2.77519  2.98518  4.58477  3.61503
      0.02334  4.16489  4.88723  0.61958  0.77255  0.48576  0.95510
121   2.65194  4.86746  2.04903  2.43315  4.23988  3.35619  3.73982  3.63032  2.58209  3.26007  4.08134  2.76506  3.84527  2.89687  3.05683  2.67863  1.92140  2.93543  5.49723  4.14194    138 t - - -
      2.68618  4.42225  2.77519  2.73123  3.46354  2.40513  3.72494  3.29354  2.67741  2.69355  4.24690  2.90347  2.73739  3.18146  2.89801  2.37887  2.77519  2.98518  4.58477  3.61503
      0.02334  4.16489  4.88723  0.61958  0.77255  0.48576  0.95510
122   3.69721  5.06637  4.28399  4.07704  2.36838  4.18181  3.79648  3.66935  3.92712  3.04510  4.31508  4.10292  4.65281  4.16038  4.04653  3.80070  3.98756  3.55483  4.02021  0.59046    139 Y - - -
      2.68618  4.42225  2.77519  2.73123  3.46354  2.40513  3.72494  3.29354  2.67741  2.69355  4.24690  2.90347  2.73739  3.18146  2.89801  2.37887  2.77519  2.98518  4.58477  3.61503
      0.02334  4.16489  4.88723  0.61958  0.77255  0.48576  0.95510
123   2.15685  5.29013  1.92608  1.60985  4.61532  3.33359  3.73935  4.08472  2.62623  3.60988  4.41194  2.81482  3.86542  2.87985  3.16139  2.75973  2.95998  3.67451  5.78390  4.34361    140 e - - -
      2.68618  4.42225  2.77519  2.73123  3.46354  2.40513  3.72494  3.29354  2.67741  2.69355  4.24690  2.90347  2.73739  3.18146  2.89801  2.37887  2.77519  2.98518  4.58477  3.61503
      0.02334  4.16489  4.88723  0.61958  0.77255  0.48576  0.95510
124   3.82451  5.01223  4.81989  4.54622  0.89060  4.54553  3.55821  3.46035  4.37909  2.75605  4.04774  4.21768  4.84572  4.29457  4.36861  3.92645  4.04434  3.40130  3.68063  1.45376    141 f - - -
      2.68618  4.42225  2.77519  2.73123  3.46354  2.40513  3.72494  3.29354  2.67741  2.69355  4.24690  2.90347  2.73739  3.18146  2.89801  2.37887  2.77519  2.98518  4.58477  3.61503
      0.02334  4.16489  4.88723  0.61958  0.77255  0.48576  0.95510
125   2.95146  5.18246  3.29213  2.68004  4.60268  3.62907  3.65121  3.98013  1.33580  3.44164  4.27078  3.13621  4.00628  2.68687  1.90837  2.51672  3.14166  3.63054  5.52075  4.28709    142 k - - -
      2.68618  4.42225  2.77519  2.73123  3.46354  2.40513  3.72494  3.29354  2.67741  2.69355  4.24690  2.90347  2.73739  3.18146  2.89801  2.37887  2.77519  2.98518  4.58477  3.61503
      0.02334  4.16489  4.88723  0.61958  0.77255  0.48576  0.95510
126   2.78426  4.26727  4.09863  2.98801  3.37125  3.91266  4.23464  1.97829  3.43115  2.21600  2.46892  3.82219  4.26397  3.69678  3.67436  3.20390  3.02080  1.51735  4.99147  3.79606    143 v - - -
      2.68618  4.42225  2.77519  2.73123  3.46354  2.40513  3.72494  3.29354  2.67741  2.69355  4.24690  2.90347  2.73739  3.18146  2.89801  2.37887  2.77519  2.98518  4.58477  3.61503
      0.02334  4.16489  4.88723  0.61958  0.77255  0.48576  0.95510
127   2.65157  4.39199  3.25797  2.92374  3.55365  3.59124  3.90276  2.71997  2.89431  2.12192  3.52012  2.61114  4.00303  3.21661  3.25048  2.87244  2.89951  1.79566  5.02230  3.76671    144 v - - -
      2.68618  4.42225  2.77519  2.73123  3.46354  2.40513  3.72494  3.29354  2.67741  2.69355  4.24690  2.90347  2.73739  3.18146  2.89801  2.37887  2.77519  2.98518  4.58477  3.61503
      0.02334  4.16489  4.88723  0.61958  0.77255  0.48576  0.95510
128   2.92006  4.39675  4.23187  3.67075  3.24263  4.02747  4.32337  2.12933  3.50364  1.12947  2.99335  3.51888  4.36103  3.78428  3.71954  3.33500  3.15654  2.25969  4.99030  3.79136    145 l - - -
      2.68618  4.42225  2.77519  2.73123  3.46354  2.40513  3.72494  3.29354  2.67741  2.69355  4.24690  2.90347  2.73739  3.18146  2.89801  2.37887  2.77519  2.98518  4.58477  3.61503
      0.02334  4.16489  4.88723  0.61958  0.77255  0.48576  0.95510
129   2.75523  5.00768  3.13283  2.53482  4.32345  3.52314  3.62993  3.72907  1.63639  3.26288  3.39878  3.03097  3.90111  2.42192  2.28032  2.76033  2.65849  3.38573  5.42652  4.12798    146 k - - -
      2.68618  4.42225  2.77519  2.73123  3.46354  2.40513  3.72494  3.29354  2.67741  2.69355  4.24690  2.90347  2.73739  3.18146  2.89801  2.37887  2.77519  2.98518  4.58477  3.61503
      0.02334  4.16489  4.88723  0.61958  0.77255  0.48576  0.95510
```

```
130   1.06629  4.25576  3.57776  3.34883  4.47365  1.77408  4.42038  3.84730  3.44691  3.58038  4.42024  3.45636  3.80161  3.71393  3.73468  2.51146  2.83250  3.28583  5.81309  4.62905    147 a - - -
      2.68618  4.42225  2.77519  2.73123  3.46354  2.40513  3.72494  3.29354  2.67741  2.69355  4.24690  2.90347  2.73739  3.18146  2.89801  2.37887  2.77519  2.98518  4.58477  3.61503
      0.02334  4.16489  4.88723  0.61958  0.77255  0.48576  0.95510
131   3.36714  5.16534  3.89059  3.35258  4.59608  3.78529  4.08066  4.16726  2.41459  3.63009  4.63119  3.68530  4.28143  3.29398  0.61106  3.44264  3.61809  3.88059  5.56480  4.46945    148 R - - -
      2.68618  4.42225  2.77519  2.73123  3.46354  2.40513  3.72494  3.29354  2.67741  2.69355  4.24690  2.90347  2.73739  3.18146  2.89801  2.37887  2.77519  2.98518  4.58477  3.61503
      0.02334  4.16489  4.88723  0.61958  0.77255  0.48576  0.95510
132   2.67789  4.75160  3.16313  2.58728  3.95903  3.51110  3.67660  3.36785  2.39078  2.69639  3.83326  2.77556  3.89469  2.70941  1.87211  2.74295  2.90121  2.79372  5.23623  3.61625    149 r - - -
      2.68618  4.42225  2.77519  2.73123  3.46354  2.40513  3.72494  3.29354  2.67741  2.69355  4.24690  2.90347  2.73739  3.18146  2.89801  2.37887  2.77519  2.98518  4.58477  3.61503
      0.02334  4.16489  4.88723  0.61958  0.77255  0.48576  0.95510
133   2.11312  4.50454  3.15830  2.77133  4.17703  3.07331  3.94283  3.57464  2.80677  3.22635  4.06258  3.16480  1.68648  2.92977  3.20037  2.61015  2.51389  3.17708  5.48898  4.21297    150 p - - -
      2.68618  4.42225  2.77519  2.73123  3.46354  2.40513  3.72494  3.29354  2.67741  2.69355  4.24690  2.90347  2.73739  3.18146  2.89801  2.37887  2.77519  2.98518  4.58477  3.61503
      0.02334  4.16489  4.88723  0.61958  0.77255  0.48576  0.95510
134   3.07087  4.80683  3.80366  3.74507  4.86220  0.39323  4.83738  4.54296  3.96555  4.15653  5.14181  3.96927  4.20642  4.26711  4.15990  3.25273  3.57468  4.01458  5.86989  4.97743    151 G - - -
      2.68618  4.42225  2.77519  2.73123  3.46354  2.40513  3.72494  3.29354  2.67741  2.69355  4.24690  2.90347  2.73739  3.18146  2.89801  2.37887  2.77519  2.98518  4.58477  3.61503
      0.02334  4.16489  4.88723  0.61958  0.77255  0.48576  0.95510
135   2.62429  4.98811  2.36689  2.29633  4.27847  3.40562  3.30124  3.26201  2.36468  3.26860  4.04643  2.76691  3.80492  2.72724  2.40153  2.61500  2.47670  3.34961  5.45356  4.08010    152 e - - -
      2.68618  4.42225  2.77519  2.73123  3.46354  2.40513  3.72494  3.29354  2.67741  2.69355  4.24690  2.90347  2.73739  3.18146  2.89801  2.37887  2.77519  2.98518  4.58477  3.61503
      0.02334  4.16489  4.88723  0.61958  0.77255  0.48576  0.95510
136   3.80232  4.99441  4.81117  4.50654  1.83269  4.53046  3.51797  3.54867  4.32198  2.87633  4.11701  4.17994  4.82456  4.25191  4.32273  3.89037  4.01448  3.44948  1.28803  1.42818    153 w - - -
      2.68618  4.42225  2.77519  2.73123  3.46354  2.40513  3.72494  3.29354  2.67741  2.69355  4.24690  2.90347  2.73739  3.18146  2.89801  2.37887  2.77519  2.98518  4.58477  3.61503
      0.02334  4.16489  4.88723  0.61958  0.77255  0.48576  0.95510
137   2.84259  4.87830  3.03855  2.72635  3.92476  3.46250  1.42342  3.76637  2.67708  3.33336  4.22974  3.18720  2.33842  3.12978  3.02387  2.90112  3.14202  3.43177  5.28091  3.86528    154 h - - -
      2.68618  4.42225  2.77519  2.73123  3.46354  2.40513  3.72494  3.29354  2.67741  2.69355  4.24690  2.90347  2.73739  3.18146  2.89801  2.37887  2.77519  2.98518  4.58477  3.61503
      0.02334  4.16489  4.88723  0.61958  0.77255  0.48576  0.95510
138   3.14546  4.46014  4.85404  4.32140  3.53253  4.49003  4.96880  1.69151  4.20689  2.04505  2.79913  4.53988  4.77264  4.43417  4.38609  3.85019  3.39591  0.99044  5.46443  4.29328    155 v - - -
      2.68618  4.42225  2.77519  2.73123  3.46354  2.40513  3.72494  3.29354  2.67741  2.69355  4.24690  2.90347  2.73739  3.18146  2.89801  2.37887  2.77519  2.98518  4.58477  3.61503
      0.02334  4.16489  4.88723  0.61958  0.77255  0.48576  0.95510
139   2.83413  4.86274  2.98826  2.74016  4.04146  2.13113  1.34254  3.87256  2.78762  3.44809  4.33401  3.19634  3.99233  3.20456  3.14297  2.89842  3.16436  3.50409  5.37917  3.98028    156 h - - -
      2.68618  4.42225  2.77519  2.73123  3.46354  2.40513  3.72494  3.29354  2.67741  2.69355  4.24690  2.90347  2.73739  3.18146  2.89801  2.37887  2.77519  2.98518  4.58477  3.61503
      0.02334  4.16489  4.88723  0.61958  0.77255  0.48576  0.95510
140   2.07786  4.35132  3.43981  2.94684  3.97270  3.23643  3.99181  3.32404  2.92260  3.03375  3.88742  3.27571  2.18521  3.23964  3.29848  2.34089  2.11245  2.55876  5.34532  4.09879    157 a - - -
      2.68618  4.42225  2.77519  2.73123  3.46354  2.40513  3.72494  3.29354  2.67741  2.69355  4.24690  2.90347  2.73739  3.18146  2.89801  2.37887  2.77519  2.98518  4.58477  3.61503
      0.02334  4.16489  4.88723  0.61958  0.77255  0.48576  0.95510
141   2.67068  4.34023  3.61661  3.05854  2.93805  3.65946  3.92358  2.71820  2.96683  2.32092  2.01413  3.43837  4.03819  2.64066  3.28494  2.92491  2.61186  2.55379  4.90034  3.66655    158 m - - -
      2.68618  4.42225  2.77519  2.73123  3.46354  2.40513  3.72494  3.29354  2.67741  2.69355  4.24690  2.90347  2.73739  3.18146  2.89801  2.37887  2.77519  2.98518  4.58477  3.61503
      0.02334  4.16489  4.88723  0.61958  0.77255  0.48576  0.95510
142   3.17238  4.50279  4.89914  4.31511  2.64637  4.41780  4.72868  1.96709  4.18068  1.31063  1.92898  4.48841  4.64524  4.25753  4.25445  3.74820  3.39366  2.01560  5.07543  3.99227    159 l - - -
      2.68618  4.42225  2.77519  2.73123  3.46354  2.40513  3.72494  3.29354  2.67741  2.69355  4.24690  2.90347  2.73739  3.18146  2.89801  2.37887  2.77519  2.98518  4.58477  3.61503
      0.02334  4.16489  4.88723  0.61958  0.77255  0.48576  0.95510
143   2.63666  4.74600  2.77324  2.61171  4.45459  3.21397  3.99354  4.01016  2.88818  3.61700  4.45978  1.25466  3.87360  3.19967  3.30330  2.08315  3.03256  3.53352  5.73664  4.36593    160 n - - -
      2.68618  4.42225  2.77519  2.73123  3.46354  2.40513  3.72494  3.29354  2.67741  2.69355  4.24690  2.90347  2.73739  3.18146  2.89801  2.37887  2.77519  2.98518  4.58477  3.61503
      0.02334  4.16489  4.88723  0.61958  0.77255  0.48576  0.95510
144   3.15186  4.45397  4.91368  4.36249  3.41528  4.49831  4.91769  1.61625  4.24938  1.74594  2.56952  4.55718  4.75177  4.41975  4.38663  3.84727  3.39022  1.23103  5.34792  4.21148    161 v - - -
      2.68618  4.42225  2.77519  2.73123  3.46354  2.40513  3.72494  3.29354  2.67741  2.69355  4.24690  2.90347  2.73739  3.18146  2.89801  2.37887  2.77519  2.98518  4.58477  3.61503
      0.02334  4.16489  4.88723  0.61958  0.77255  0.48576  0.95510
145   2.86459  5.27930  2.73588  1.60319  4.63061  3.45294  3.67763  4.07512  1.81226  3.54854  4.35163  2.92510  3.91201  2.36746  2.68407  2.81319  3.09159  3.68143  5.66352  4.30990    162 e - - -
      2.68618  4.42225  2.77519  2.73123  3.46354  2.40513  3.72494  3.29354  2.67741  2.69355  4.24690  2.90347  2.73739  3.18146  2.89801  2.37887  2.77519  2.98518  4.58477  3.61503
      0.02334  4.16489  4.88723  0.61958  0.77255  0.48576  0.95510
146   2.87235  5.30761  1.88660  2.04939  4.67403  1.66411  3.80292  4.15371  2.74797  3.68520  4.50851  2.81435  3.88845  2.95993  3.29833  2.80957  2.93444  3.73934  5.86821  4.42012    163 g - - -
      2.68618  4.42225  2.77519  2.73123  3.46354  2.40513  3.72494  3.29354  2.67741  2.69355  4.24690  2.90347  2.73739  3.18146  2.89801  2.37887  2.77519  2.98518  4.58477  3.61503
      0.02334  4.16489  4.88723  0.61958  0.77255  0.48576  0.95510
147   1.84427  4.31109  3.52603  3.11729  3.98835  1.56532  4.13506  2.65082  3.10518  3.02380  3.92713  3.38219  3.86845  3.41961  3.43955  2.62538  2.83845  2.86519  5.40740  4.17274    164 g - - -
      2.68618  4.42225  2.77519  2.73123  3.46354  2.40513  3.72494  3.29354  2.67741  2.69355  4.24690  2.90347  2.73739  3.18146  2.89801  2.37887  2.77519  2.98518  4.58477  3.61503
      0.02334  4.16489  4.88723  0.61958  0.77255  0.48576  0.95510
148   3.07087  4.80683  3.80366  3.74507  4.86220  0.39323  4.83738  4.54296  3.96555  4.15653  5.14181  3.96927  4.20642  4.26711  4.15990  3.25273  3.57468  4.01458  5.86989  4.97743    165 G - - -
      2.68618  4.42225  2.77519  2.73123  3.46354  2.40513  3.72494  3.29354  2.67741  2.69355  4.24690  2.90347  2.73739  3.18146  2.89801  2.37887  2.77519  2.98518  4.58477  3.61503
      0.02334  4.16489  4.88723  0.61958  0.77255  0.48576  0.95510
149   2.80052  4.82649  2.98006  2.75270  4.31147  3.37784  3.99526  3.83291  2.76055  3.40462  4.32709  3.19755  1.17316  2.57634  3.09406  2.87666  3.14915  3.47346  5.58050  4.28419    166 p - - -
      2.68618  4.42225  2.77519  2.73123  3.46354  2.40513  3.72494  3.29354  2.67741  2.69355  4.24690  2.90347  2.73739  3.18146  2.89801  2.37887  2.77519  2.98518  4.58477  3.61503
      0.02334  4.16489  4.88723  0.61958  0.77255  0.48576  0.95510
150   3.17715  4.48118  4.93890  4.38473  2.59247  4.49451  4.84810  1.22179  4.26487  1.66945  3.14481  4.55958  4.73829  4.39709  4.37526  3.84201  3.41150  1.76082  5.22054  4.04847    167 i - - -
      2.68618  4.42225  2.77519  2.73123  3.46354  2.40513  3.72494  3.29354  2.67741  2.69355  4.24690  2.90347  2.73739  3.18146  2.89801  2.37887  2.77519  2.98518  4.58477  3.61503
      0.02334  4.16489  4.88723  0.61958  0.77255  0.48576  0.95510
151   3.19156  4.48261  4.96470  4.43031  3.49652  4.56850  5.04049  1.11554  4.31774  1.96863  2.73905  4.63495  4.82349  4.51355  4.47183  3.93329  3.43730  1.48351  5.46967  4.32088    168 i - - -
      2.68618  4.42225  2.77519  2.73123  3.46354  2.40513  3.72494  3.29354  2.67741  2.69355  4.24690  2.90347  2.73739  3.18146  2.89801  2.37887  2.77519  2.98518  4.58477  3.61503
      0.02334  4.16489  4.88723  0.61958  0.77255  0.48576  0.95510
152   3.07087  4.80683  3.80366  3.74507  4.86220  0.39323  4.83738  4.54296  3.96555  4.15653  5.14181  3.96927  4.20642  4.26711  4.15990  3.25273  3.57468  4.01458  5.86989  4.97743    169 G - - -
```

```
     2.68618 4.42225 2.77519 2.73123 3.46354 2.40513 3.72494 3.29354 2.67741 2.69355 4.24690 2.90347 2.73739 3.18146 2.89801 2.37887 2.77519 2.98518 4.58477 3.61503
     0.02334 4.16489 4.88723 0.61958 0.77255 0.48576 0.95510
153  2.67773 4.57748 3.41205 3.25471 4.60978 2.47055 4.43895 4.12904 3.41766 3.77299 4.65887 3.52922 0.81301 3.73729 3.71622 2.83507 3.15254 3.60769 5.80468 4.68747  170 p - - -
     2.68618 4.42225 2.77519 2.73123 3.46354 2.40513 3.72494 3.29354 2.67741 2.69355 4.24690 2.90347 2.73739 3.18146 2.89801 2.37887 2.77519 2.98518 4.58477 3.61503
     0.02334 4.16489 4.88723 0.61958 0.77255 0.48576 0.95510
154  2.70273 4.87605 2.63752 2.08020 4.50851 1.44044 3.91194 3.95882 2.81805 3.55280 4.38838 2.97369 3.87635 3.09622 3.27764 2.35983 3.05462 3.52816 5.75507 4.39717  171 g - - -
     2.68618 4.42225 2.77519 2.73123 3.46354 2.40513 3.72494 3.29354 2.67741 2.69355 4.24690 2.90347 2.73739 3.18146 2.89801 2.37887 2.77519 2.98518 4.58477 3.61503
     0.02334 4.16489 4.88723 0.61958 0.77255 0.48576 0.95510
155  2.65668 4.93609 2.97834 2.08842 4.21272 3.44602 3.62703 3.15701 1.93900 3.19851 4.00061 2.95433 3.83937 2.38263 2.77291 2.66480 2.88380 2.92963 5.40346 4.06206  172 k - - -
     2.68618 4.42225 2.77519 2.73123 3.46354 2.40513 3.72494 3.29354 2.67741 2.69355 4.24690 2.90347 2.73739 3.18146 2.89801 2.37887 2.77519 2.98518 4.58477 3.61503
     0.02334 4.16489 4.88723 0.61958 0.77255 0.48576 0.95510
156  3.00976 4.49761 4.11304 3.59444 1.99718 3.96884 3.70734 2.98028 2.80785 2.57446 3.60022 3.75737 4.32076 3.67007 3.64035 3.26431 3.23631 2.80667 1.71864 2.33983  173 w - - -
     2.68618 4.42225 2.77519 2.73123 3.46354 2.40513 3.72494 3.29354 2.67741 2.69355 4.24690 2.90347 2.73739 3.18146 2.89801 2.37887 2.77519 2.98518 4.58477 3.61503
     0.02334 4.16489 4.88723 0.61958 0.77255 0.48576 0.95510
157  3.21786 4.49615 5.01367 4.48392 3.51081 4.61962 5.09794 1.50331 4.37568 1.58341 3.30356 4.68854 4.86322 4.56848 4.52806 3.98981 3.46332 1.19348 5.50542 4.35228  174 v - - -
     2.68618 4.42225 2.77519 2.73123 3.46354 2.40513 3.72494 3.29354 2.67741 2.69355 4.24690 2.90347 2.73739 3.18146 2.89801 2.37887 2.77519 2.98518 4.58477 3.61503
     0.02334 4.16489 4.88723 0.61958 0.77255 0.48576 0.95510
158  2.53407 4.89726 2.63916 2.04500 4.19116 3.39609 3.69285 3.42712 2.51256 3.20200 4.02048 2.93940 3.84473 2.84422 2.98348 2.68106 1.86348 3.24635 5.44177 4.08993  175 t - - -
     2.68618 4.42225 2.77519 2.73123 3.46354 2.40513 3.72494 3.29354 2.67741 2.69355 4.24690 2.90347 2.73739 3.18146 2.89801 2.37887 2.77519 2.98518 4.58477 3.61503
     0.02334 4.16489 4.88723 0.61958 0.77255 0.48576 0.95510
159  3.21520 4.50801 4.99172 4.51168 3.64541 4.59335 5.21291 0.96034 4.39431 2.11157 3.43975 4.71096 4.89617 4.65153 4.57532 4.00008 3.48455 1.42359 5.66376 4.46257  176 i - - -
     2.68618 4.42225 2.77519 2.73123 3.46354 2.40513 3.72494 3.29354 2.67741 2.69355 4.24690 2.90347 2.73739 3.18146 2.89801 2.37887 2.77519 2.98518 4.58477 3.61503
     0.02334 4.16489 4.88723 0.61958 0.77255 0.48576 0.95510
160  2.71560 5.18775 2.23843 1.91113 4.50400 2.93303 3.65077 3.98006 2.11583 3.48288 4.25419 2.83877 3.82498 2.77021 2.95107 2.66995 2.42694 3.56596 5.63827 4.22275  177 e - - -
     2.68618 4.42225 2.77519 2.73123 3.46354 2.40513 3.72494 3.29354 2.67741 2.69355 4.24690 2.90347 2.73739 3.18146 2.89801 2.37887 2.77519 2.98518 4.58477 3.61503
     0.02334 4.16489 4.88723 0.61958 0.77255 0.48576 0.95510
161  2.58874 4.51058 3.37981 3.23107 4.63520 0.81844 4.43397 4.14804 3.43548 3.79063 4.65384 3.48479 2.66952 3.72475 3.74387 2.74796 3.07663 3.58362 5.84717 4.71499  178 g - - -
     2.68618 4.42225 2.77519 2.73123 3.46354 2.40513 3.72494 3.29354 2.67741 2.69355 4.24690 2.90347 2.73739 3.18146 2.89801 2.37887 2.77519 2.98518 4.58477 3.61503
     0.02334 4.16489 4.88723 0.61958 0.77255 0.48576 0.95510
162  2.72156 5.08301 1.91779 2.32395 4.46777 3.33480 3.72572 3.92955 2.50254 3.47231 4.26909 2.74470 3.84359 2.86614 3.07788 1.86097 2.60093 3.52627 5.65762 4.25742  179 s - - -
     2.68618 4.42225 2.77519 2.73123 3.46354 2.40513 3.72494 3.29354 2.67741 2.69355 4.24690 2.90347 2.73739 3.18146 2.89801 2.37887 2.77519 2.98518 4.58477 3.61503
     0.02334 4.16489 4.88723 0.61958 0.77255 0.48576 0.95510
163  2.59685 4.32024 3.46813 2.90073 3.45009 3.57202 3.82036 2.82001 2.70266 2.48022 2.23890 2.95910 3.34117 3.15256 3.18662 2.69531 2.82835 2.59494 3.82404 3.41411  180 m - - -
     2.68618 4.42225 2.77519 2.73123 3.46354 2.40513 3.72494 3.29354 2.67741 2.69355 4.24690 2.90347 2.73739 3.18146 2.89801 2.37887 2.77519 2.98518 4.58477 3.61503
     0.02334 4.16489 4.88723 0.61958 0.77255 0.48576 0.95510
164  2.10522 4.66191 3.03858 2.60786 4.26029 2.62075 3.82099 3.68430 2.21393 3.28821 4.09929 3.06151 3.82303 2.98771 3.05038 1.77103 2.86913 3.27864 5.51062 4.19820  181 s - - -
     2.68618 4.42225 2.77519 2.73123 3.46354 2.40513 3.72494 3.29354 2.67741 2.69355 4.24690 2.90347 2.73739 3.18146 2.89801 2.37887 2.77519 2.98518 4.58477 3.61503
     0.02334 4.16489 4.88723 0.61958 0.77255 0.48576 0.95510
165  2.72671 5.23108 1.92236 2.01247 4.54923 3.14390 3.65352 4.03522 2.46221 3.52607 4.29286 2.57276 3.19262 2.67157 2.83065 2.49984 2.97055 3.60992 5.67365 4.24577  182 d - - -
     2.68618 4.42225 2.77519 2.73123 3.46354 2.40513 3.72494 3.29354 2.67741 2.69355 4.24690 2.90347 2.73739 3.18146 2.89801 2.37887 2.77519 2.98518 4.58477 3.61503
     0.02334 4.16489 4.88723 0.61958 0.77255 0.48576 0.95510
166  3.65633 4.97487 4.64261 4.42152 0.63212 4.25844 4.04309 3.15482 4.32396 2.46101 3.84666 4.36241 4.71705 4.41299 4.37075 3.90466 3.96096 3.16555 4.22202 2.55920  183 F - - -
     2.68618 4.42225 2.77519 2.73123 3.46354 2.40513 3.72494 3.29354 2.67741 2.69355 4.24690 2.90347 2.73739 3.18146 2.89801 2.37887 2.77519 2.98518 4.58477 3.61503
     0.02334 4.16489 4.88723 0.61958 0.77255 0.48576 0.95510
167  2.48155 4.65207 3.14895 2.61194 3.96814 3.42919 3.73375 3.34392 2.11231 2.99538 3.83769 3.08410 3.86617 2.91878 2.86394 2.52654 1.94465 2.76508 5.27101 3.98034  184 t - - -
     2.68618 4.42225 2.77519 2.73123 3.46354 2.40513 3.72494 3.29354 2.67741 2.69355 4.24690 2.90347 2.73739 3.18146 2.89801 2.37887 2.77519 2.98518 4.58477 3.61503
     0.02334 4.16489 4.88723 0.61958 0.77255 0.48576 0.95510
168  2.83919 5.21547 2.13941 2.29707 4.36097 3.35703 3.75562 3.97833 2.65870 3.52469 4.35515 1.45080 3.89098 2.92375 3.17058 2.79404 3.10517 3.60097 5.62747 3.56256  185 n - - -
     2.68618 4.42225 2.77519 2.73123 3.46354 2.40513 3.72494 3.29354 2.67741 2.69355 4.24690 2.90347 2.73739 3.18146 2.89801 2.37887 2.77519 2.98518 4.58477 3.61503
     0.02334 4.16489 4.88723 0.61958 0.77255 0.48576 0.95510
169  2.54849 4.53942 3.17961 2.72904 3.99443 3.33774 3.86138 3.05503 2.71397 3.04104 3.90065 2.76589 1.81437 3.07374 3.10826 2.66754 2.39942 3.02217 5.33677 4.05311  186 p - - -
     2.68618 4.42225 2.77519 2.73123 3.46354 2.40513 3.72494 3.29354 2.67741 2.69355 4.24690 2.90347 2.73739 3.18146 2.89801 2.37887 2.77519 2.98518 4.58477 3.61503
     0.02334 4.16489 4.88723 0.61958 0.77255 0.48576 0.95510
170  3.21399 4.48357 5.03630 4.53099 3.66231 4.65155 5.21679 1.31734 4.42835 2.06140 3.44934 4.73555 4.91757 4.67192 4.60841 4.04020 3.47068 1.04214 5.66213 4.46580  187 v - - -
     2.68618 4.42225 2.77519 2.73123 3.46354 2.40513 3.72494 3.29354 2.67741 2.69355 4.24690 2.90347 2.73739 3.18146 2.89801 2.37887 2.77519 2.98518 4.58477 3.61503
     0.02334 4.16489 4.88723 0.61958 0.77255 0.48576 0.95510
171  2.76898 4.93559 3.04578 2.56975 4.27908 3.48821 3.70236 3.67045 1.91621 3.24457 4.08245 3.05644 3.92193 2.50068 2.65019 2.79634 1.75829 3.34056 5.44124 4.14970  188 t - - -
     2.68618 4.42225 2.77519 2.73123 3.46354 2.40513 3.72494 3.29354 2.67741 2.69355 4.24690 2.90347 2.73739 3.18146 2.89801 2.37887 2.77519 2.98518 4.58477 3.61503
     0.02334 4.16489 4.88723 0.61958 0.77255 0.48576 0.95510
172  2.69349 4.42649 3.76844 3.32748 3.65681 3.55756 4.22806 2.67787 3.17701 1.99582 3.62304 3.61478 4.10757 3.56137 3.45785 2.94952 1.35138 2.50555 5.26185 4.00801  189 t - - -
     2.68618 4.42225 2.77519 2.73123 3.46354 2.40513 3.72494 3.29354 2.67741 2.69355 4.24690 2.90347 2.73739 3.18146 2.89801 2.37887 2.77519 2.98518 4.58477 3.61503
     0.02334 4.16489 4.88723 0.61958 0.77255 0.48576 0.95510
173  3.42557 4.72859 5.02549 4.49681 3.19545 4.70266 5.04062 1.73495 4.31880 0.84232 2.99992 4.74643 4.88689 4.45926 4.43279 4.08666 3.65760 2.18868 5.30986 4.20452  190 l - - -
     2.68618 4.42225 2.77519 2.73123 3.46354 2.40513 3.72494 3.29354 2.67741 2.69355 4.24690 2.90347 2.73739 3.18146 2.89801 2.37887 2.77519 2.98518 4.58477 3.61503
     0.02334 4.16489 4.88723 0.61958 0.77255 0.48576 0.95510
174  2.64359 4.88130 2.32156 2.41312 4.17019 3.39553 3.67850 3.59043 2.49435 2.68609 4.00036 2.55923 3.83472 2.82779 2.96662 2.54839 2.16629 3.24768 5.41896 4.06703  191 t - - -
     2.68618 4.42225 2.77519 2.73123 3.46354 2.40513 3.72494 3.29354 2.67741 2.69355 4.24690 2.90347 2.73739 3.18146 2.89801 2.37887 2.77519 2.98518 4.58477 3.61503
```

	0.02334	4.16489	4.88723	0.61958	0.77255	0.48576	0.95510														
175	3.07087	4.80683	3.80366	3.74507	4.86220	0.39323	4.83738	4.54296	3.96555	4.15653	5.14181	3.96927	4.20642	4.26711	4.15990	3.25273	3.57468	4.01458	5.86989	4.97743	192 G - - -
	2.68618	4.42225	2.77519	2.73123	3.46354	2.40513	3.72494	3.29354	2.67741	2.69355	4.24690	2.90347	2.73739	3.18146	2.89801	2.37887	2.77519	2.98518	4.58477	3.61503	
	0.02334	4.16489	4.88723	0.61958	0.77255	0.48576	0.95510														
176	2.67079	5.11454	2.61541	2.01454	4.42782	3.37939	3.62541	3.89604	2.40128	3.40784	4.17564	2.51793	3.81106	2.20289	2.89425	2.45182	2.53943	3.40248	5.56863	4.16715	193 e - - -
	2.68618	4.42225	2.77519	2.73123	3.46354	2.40513	3.72494	3.29354	2.67741	2.69355	4.24690	2.90347	2.73739	3.18146	2.89801	2.37887	2.77519	2.98518	4.58477	3.61503	
	0.02334	4.16489	4.88723	0.61958	0.77255	0.48576	0.95510														
177	2.58857	4.34157	3.59864	3.06363	3.61476	3.50845	3.99102	2.75938	2.97073	2.49990	3.15348	3.41520	3.98643	3.30763	3.30193	2.65696	1.54564	2.43384	5.09644	3.86174	194 t - - -
	2.68618	4.42225	2.77519	2.73123	3.46354	2.40513	3.72494	3.29354	2.67741	2.69355	4.24690	2.90347	2.73739	3.18146	2.89801	2.37887	2.77519	2.98518	4.58477	3.61503	
	0.02334	4.16489	4.88723	0.61958	0.77255	0.48576	0.95510														
178	3.20599	4.48379	5.02812	4.54152	3.69798	4.62373	5.24642	1.20542	4.43897	2.17994	3.48468	4.73646	4.91578	4.69556	4.62244	4.02585	3.47170	1.08596	5.70266	4.49639	195 v - - -
	2.68618	4.42225	2.77519	2.73123	3.46354	2.40513	3.72494	3.29354	2.67741	2.69355	4.24690	2.90347	2.73739	3.18146	2.89801	2.37887	2.77519	2.98518	4.58477	3.61503	
	0.02334	4.16489	4.88723	0.61958	0.77255	0.48576	0.95510														
179	3.04383	5.57535	1.11034	2.19862	4.89270	3.29356	3.89162	4.47015	2.94745	3.97304	4.83925	1.97069	3.93972	3.06936	3.55523	2.93246	3.35366	4.02576	6.09396	4.57539	196 d - - -
	2.68618	4.42225	2.77519	2.73123	3.46354	2.40513	3.72494	3.29354	2.67741	2.69355	4.24690	2.90347	2.73739	3.18146	2.89801	2.37887	2.77519	2.98518	4.58477	3.61503	
	0.02334	4.16489	4.88723	0.61958	0.77255	0.48576	0.95510														
180	3.45698	4.79061	4.95896	4.41050	3.10374	4.66842	4.95200	2.28314	4.21043	0.78252	2.18490	4.68296	4.84001	4.34739	4.32435	4.03813	3.67934	2.45512	5.22658	4.15535	197 l - - -
	2.68618	4.42225	2.77519	2.73123	3.46354	2.40513	3.72494	3.29354	2.67741	2.69355	4.24690	2.90347	2.73739	3.18146	2.89801	2.37887	2.77519	2.98518	4.58477	3.61503	
	0.02334	4.16489	4.88723	0.61958	0.77255	0.48576	0.95510														
181	3.06991	5.39185	2.46895	0.89362	4.72814	3.39996	3.91449	4.19283	2.67961	3.75634	4.66169	2.94130	4.00127	3.09584	3.21229	3.00522	3.35877	3.82815	5.88348	4.50408	198 e - - -
	2.68618	4.42225	2.77519	2.73123	3.46354	2.40513	3.72494	3.29354	2.67741	2.69355	4.24690	2.90347	2.73739	3.18146	2.89801	2.37887	2.77519	2.98518	4.58477	3.61503	
	0.02334	4.16489	4.88723	0.61958	0.77255	0.48576	0.95510														
182	2.61679	4.76701	2.98898	2.49363	4.05798	3.41248	3.53342	3.47212	2.49676	3.08585	3.90945	2.16071	3.84314	2.74339	2.93990	2.44374	2.25459	2.84198	5.33395	4.00992	199 n - - -
	2.68618	4.42225	2.77519	2.73123	3.46354	2.40513	3.72494	3.29354	2.67741	2.69355	4.24690	2.90347	2.73739	3.18146	2.89801	2.37887	2.77519	2.98518	4.58477	3.61503	
	0.02334	4.16489	4.88723	0.61958	0.77255	0.48576	0.95510														
183	3.77097	5.01017	4.67748	4.33788	1.72489	4.48890	2.73122	3.61724	4.18310	2.95923	4.17475	4.11004	4.78819	4.16387	4.24291	3.83343	3.98202	3.49427	3.41184	0.85393	200 y - - -
	2.68618	4.42225	2.77519	2.73123	3.46354	2.40513	3.72494	3.29354	2.67741	2.69355	4.24690	2.90347	2.73739	3.18146	2.89801	2.37887	2.77519	2.98518	4.58477	3.61503	
	0.02334	4.16489	4.88723	0.61958	0.77255	0.48576	0.95510														
184	2.26979	4.76049	2.81121	2.54370	4.39158	1.65580	3.87511	3.82596	2.57547	3.42611	4.24896	2.31189	3.85118	3.05193	3.16052	2.67688	2.96367	3.40540	5.63939	4.30784	201 g - - -
	2.68618	4.42225	2.77519	2.73123	3.46354	2.40513	3.72494	3.29354	2.67741	2.69355	4.24690	2.90347	2.73739	3.18146	2.89801	2.37887	2.77519	2.98518	4.58477	3.61503	
	0.02334	4.16489	4.88723	0.61958	0.77255	0.48576	0.95510														
185	2.61217	4.27585	3.57683	2.65656	2.98486	3.61518	3.87648	2.27622	2.94620	2.02487	3.35848	3.39017	3.99113	2.92080	3.27235	2.86992	2.54721	2.50757	4.86550	3.64155	202 l - - -
	2.68618	4.42225	2.77519	2.73123	3.46354	2.40513	3.72494	3.29354	2.67741	2.69355	4.24690	2.90347	2.73739	3.18146	2.89801	2.37887	2.77519	2.98518	4.58477	3.61503	
	0.02334	4.16489	4.88723	0.61958	0.77255	0.48576	0.95510														
186	1.96667	4.79887	2.19730	2.47823	4.42617	2.31566	3.82708	3.87735	2.69706	3.45400	4.25319	2.97127	3.82086	2.98424	3.17935	2.10272	2.92471	3.43562	5.65525	4.29639	203 a - - -
	2.68618	4.42225	2.77519	2.73123	3.46354	2.40513	3.72494	3.29354	2.67741	2.69355	4.24690	2.90347	2.73739	3.18146	2.89801	2.37887	2.77519	2.98518	4.58477	3.61503	
	0.02334	4.16489	4.88723	0.61958	0.77255	0.48576	0.95510														
187	2.77066	5.00132	2.98016	2.54413	4.44322	3.24548	3.71386	3.87806	2.31197	3.40599	4.22307	2.00091	3.91114	2.85540	1.72010	2.66251	3.02694	3.49868	5.54485	4.23661	204 r - - -
	2.68618	4.42225	2.77519	2.73123	3.46354	2.40513	3.72494	3.29354	2.67741	2.69355	4.24690	2.90347	2.73739	3.18146	2.89801	2.37887	2.77519	2.98518	4.58477	3.61503	
	0.02334	4.16489	4.88723	0.61958	0.77255	0.48576	0.95510														
188	2.39909	4.17303	3.90816	3.33876	3.32816	3.13577	4.04107	1.92137	3.25025	2.35419	2.94330	3.62267	4.08835	3.51033	3.50318	2.98675	2.46409	1.99886	4.84791	3.64347	205 i - - -
	2.68618	4.42225	2.77519	2.73123	3.46354	2.40513	3.72494	3.29354	2.67741	2.69355	4.24690	2.90347	2.73739	3.18146	2.89801	2.37887	2.77519	2.98518	4.58477	3.61503	
	0.02334	4.16489	4.88723	0.61958	0.77255	0.48576	0.95510														
189	2.78972	4.21347	4.33007	3.75366	2.83477	3.92597	4.15634	1.94621	3.62212	2.05241	2.93967	3.92371	4.26596	3.80930	3.77044	3.22655	3.02086	2.01807	4.69190	2.02001	206 i - - -
	2.68618	4.42225	2.77519	2.73123	3.46354	2.40513	3.72494	3.29354	2.67741	2.69355	4.24690	2.90347	2.73739	3.18146	2.89801	2.37887	2.77519	2.98518	4.58477	3.61503	
	0.02334	4.16489	4.88723	0.61958	0.77255	0.48576	0.95510														
190	2.25021	4.23680	3.61677	3.07231	2.20181	2.63152	3.92293	2.78819	3.01467	2.52332	3.19894	3.40808	3.95613	3.30286	3.32937	2.68052	2.66587	2.55738	4.90603	3.67986	207 f - - -
	2.68618	4.42225	2.77519	2.73123	3.46354	2.40513	3.72494	3.29354	2.67741	2.69355	4.24690	2.90347	2.73739	3.18146	2.89801	2.37887	2.77519	2.98518	4.58477	3.61503	
	0.02334	4.16489	4.88723	0.61958	0.77255	0.48576	0.95510														
191	3.86835	5.12987	4.56789	4.35347	3.02935	4.08352	4.30963	3.91661	4.07628	3.25628	4.53479	4.44682	4.62737	4.44811	4.14946	4.06898	4.18585	3.81445	0.45737	3.01784	208 W - - -
	2.68618	4.42225	2.77519	2.73123	3.46354	2.40513	3.72494	3.29354	2.67741	2.69355	4.24690	2.90347	2.73739	3.18146	2.89801	2.37887	2.77519	2.98518	4.58477	3.61503	
	0.02334	4.16489	4.88723	0.61958	0.77255	0.48576	0.95510														
192	2.64604	4.55911	3.26026	2.72472	3.58236	3.51417	2.05591	3.15816	2.62974	2.81762	3.69378	3.17425	3.92477	3.01167	3.00695	2.47512	2.51420	2.89518	3.76858	3.64233	209 h - - -
	2.68618	4.42225	2.77520	2.73123	3.46354	2.40509	3.72495	3.29354	2.67741	2.69355	4.24690	2.90347	2.73740	3.18146	2.89801	2.37887	2.77520	2.98518	4.58477	3.61503	
	0.04227	3.38577	4.88723	0.53555	0.88031	0.48576	0.95510														
193	2.26688	4.21670	4.24582	3.66551	2.80818	3.90730	4.22392	2.21454	3.55170	1.51929	2.91960	3.88853	4.24933	3.76056	3.73358	3.20291	2.84789	2.09845	4.85983	3.68828	211 l - - -
	2.68618	4.42225	2.77519	2.73123	3.46354	2.40513	3.72494	3.29354	2.67741	2.69355	4.24690	2.90347	2.73739	3.18146	2.89801	2.37887	2.77519	2.98518	4.58477	3.61503	
	0.02334	4.16489	4.88723	0.61958	0.77255	0.48576	0.95510														
194	2.66653	4.18869	3.97391	3.40684	2.24506	3.73254	4.02867	2.52491	3.30379	1.75918	3.22765	3.66697	3.11830	3.54879	3.53103	2.90395	2.90670	2.19718	4.74813	3.50550	212 l - - -
	2.68618	4.42225	2.77519	2.73123	3.46354	2.40513	3.72494	3.29354	2.67741	2.69355	4.24690	2.90347	2.73739	3.18146	2.89801	2.37887	2.77519	2.98518	4.58477	3.61503	
	0.02334	4.16489	4.88723	0.61958	0.77255	0.48576	0.95510														
195	2.65857	4.37572	3.70929	3.25935	3.33573	3.23477	3.99037	3.05185	3.06559	2.74282	3.69857	3.52249	4.03296	3.45935	3.32097	2.88665	2.39885	2.80654	1.49408	3.45145	213 w - - -
	2.68618	4.42225	2.77519	2.73123	3.46354	2.40513	3.72494	3.29354	2.67741	2.69355	4.24690	2.90347	2.73739	3.18146	2.89801	2.37887	2.77519	2.98518	4.58477	3.61503	
	0.02334	4.16489	4.88723	0.61958	0.77255	0.48576	0.95510														
196	2.58734	4.15641	3.77114	3.19550	2.93843	3.14418	3.91898	2.61640	3.11443	2.17492	3.07055	3.50564	4.01192	3.37748	3.38236	2.69906	2.40510	2.23960	4.75500	2.76640	214 l - - -
	2.68618	4.42225	2.77519	2.73123	3.46354	2.40513	3.72494	3.29354	2.67741	2.69355	4.24690	2.90347	2.73739	3.18146	2.89801	2.37887	2.77519	2.98518	4.58477	3.61503	
	0.02334	4.16489	4.88723	0.61958	0.77255	0.48576	0.95510														

197	1.86413	4.19607	3.97897	3.40953	2.60196	3.60766	4.07100	2.29678	3.31355	1.94440	3.25005	3.68103	4.12720	3.56279	3.55079	3.03885	2.91515	2.24961	4.82317	3.60894	215 a - - -
	2.68618	4.42225	2.77519	2.73123	3.46354	2.40513	3.72494	3.29354	2.67741	2.69355	4.24690	2.90347	2.73739	3.18146	2.89801	2.37887	2.77519	2.98518	4.58477	3.61503	
	0.02334	4.16489	4.88723	0.61958	0.77255	0.48576	0.95510														
198	2.72165	4.37520	4.74470	4.19018	3.30341	4.33950	4.75092	1.55456	4.07966	1.60228	3.27804	4.38441	4.63242	4.27593	4.23565	3.67584	3.28180	1.44064	5.26283	4.09771	216 v - - -
	2.68618	4.42225	2.77519	2.73123	3.46354	2.40513	3.72494	3.29354	2.67741	2.69355	4.24690	2.90347	2.73739	3.18146	2.89801	2.37887	2.77519	2.98518	4.58477	3.61503	
	0.02334	4.16489	4.88723	0.61958	0.77255	0.48576	0.95510														
199	1.70577	4.28588	3.50732	3.32828	4.53830	1.06704	4.44491	3.92235	3.47489	3.65386	4.49877	3.45263	3.81463	3.73960	3.76034	2.53372	2.86478	3.34270	5.86454	4.68232	217 g - - -
	2.68618	4.42225	2.77519	2.73123	3.46354	2.40513	3.72494	3.29354	2.67741	2.69355	4.24690	2.90347	2.73739	3.18146	2.89801	2.37887	2.77519	2.98518	4.58477	3.61503	
	0.02334	4.16489	4.88723	0.61958	0.77255	0.48576	0.95510														
200	2.18377	4.23853	4.35497	3.78423	3.17590	4.01344	4.36665	1.86321	3.67664	1.96263	3.10801	4.00447	4.35351	3.89976	3.86567	3.31891	2.97867	1.55448	5.00849	3.82216	218 v - - -
	2.68618	4.42225	2.77519	2.73123	3.46354	2.40513	3.72494	3.29354	2.67741	2.69355	4.24690	2.90347	2.73739	3.18146	2.89801	2.37887	2.77519	2.98518	4.58477	3.61503	
	0.02334	4.16489	4.88723	0.61958	0.77255	0.48576	0.95510														
201	1.45119	3.80891	3.72762	3.20122	3.02185	2.85307	4.04471	2.86451	3.13820	2.65172	3.56195	3.46309	3.92589	3.41927	3.44109	2.72666	2.81303	2.45069	5.05145	3.83152	219 a - - -
	2.68618	4.42226	2.77520	2.73124	3.46354	2.40510	3.72495	3.29354	2.67741	2.69351	4.24690	2.90347	2.73740	3.18147	2.89801	2.37887	2.77520	2.98519	4.58477	3.61504	
	0.04227	3.38577	4.88723	0.63282	0.75735	0.48576	0.95510														
202	3.58086	4.95839	4.41963	4.08414	2.36814	4.20131	3.77994	3.47178	3.75090	2.82204	4.07037	4.09198	4.62314	4.06026	3.87227	3.73282	3.84247	3.38135	0.85612	2.25455	222 w - - -
	2.68618	4.42225	2.77519	2.73123	3.46354	2.40513	3.72494	3.29354	2.67741	2.69355	4.24690	2.90347	2.73739	3.18146	2.89801	2.37887	2.77519	2.98518	4.58477	3.61503	
	0.02334	4.16489	4.88723	0.61958	0.77255	0.48576	0.95510														
203	3.06361	4.42692	4.72964	4.14154	3.09998	4.26575	4.56121	1.80026	3.99521	1.12169	2.63326	4.31605	4.52756	4.11183	4.09006	3.58471	3.28635	2.23011	4.20869	3.86150	223 l - - -
	2.68618	4.42225	2.77519	2.73123	3.46354	2.40513	3.72494	3.29354	2.67741	2.69355	4.24690	2.90347	2.73739	3.18146	2.89801	2.37887	2.77519	2.98518	4.58477	3.61503	
	0.04227	4.16489	3.65497	0.61958	0.77255	0.48576	0.95510														
204	2.57929	4.11372	4.01084	3.43010	2.92565	3.08919	4.00691	2.39566	3.32003	1.73618	3.19184	3.66691	4.08258	3.55140	3.52422	3.00020	2.85469	2.04162	4.70777	3.02331	224 l - - -
	2.68618	4.42225	2.77519	2.73123	3.46354	2.40513	3.72494	3.29354	2.67741	2.69355	4.24690	2.90347	2.73739	3.18146	2.89801	2.37887	2.77519	2.98518	4.58477	3.61503	
	0.02379	4.14640	4.86874	0.61958	0.77255	0.47215	0.97726														
205	3.95439	5.06073	4.96686	4.73578	1.48265	4.62868	3.48983	3.65252	4.54454	2.94363	4.21059	4.24895	4.90689	4.36206	4.46487	4.00070	4.15995	3.56009	2.25232	0.98366	225 y - - -
	2.68618	4.42225	2.77519	2.73123	3.46354	2.40513	3.72494	3.29354	2.67741	2.69355	4.24690	2.90347	2.73739	3.18146	2.89801	2.37887	2.77519	2.98518	4.58477	3.61503	
	0.02334	4.16489	4.88723	0.61958	0.77255	0.48576	0.95510														
206	3.58330	4.93383	4.54895	4.21088	2.22764	4.24603	3.80067	3.34975	3.95948	2.67887	3.96360	4.16369	4.65746	4.15804	4.05926	3.76294	3.84927	3.29162	0.87851	2.31973	226 w - - -
	2.68618	4.42225	2.77520	2.73123	3.46354	2.40509	3.72495	3.29354	2.67741	2.69355	4.24690	2.90347	2.73740	3.18146	2.89801	2.37887	2.77520	2.98518	4.58477	3.61503	
	0.04227	3.38577	4.88723	0.53555	0.88031	0.48576	0.95510														
207	2.57120	3.46902	4.32656	3.73629	2.56821	3.81202	4.14372	2.27264	3.58861	1.87178	3.03359	3.86939	4.17173	3.77759	3.70879	3.04170	2.79029	1.72808	4.73212	3.54828	228 v - - -
	2.68618	4.42225	2.77519	2.73123	3.46354	2.40513	3.72494	3.29354	2.67741	2.69355	4.24690	2.90347	2.73739	3.18146	2.89801	2.37887	2.77519	2.98518	4.58477	3.61503	
	0.02334	4.16489	4.88723	0.61958	0.77255	0.48576	0.95510														
208	2.50878	4.55483	3.22595	2.65931	3.55123	3.50961	3.72769	2.64756	2.36613	2.78442	3.64911	3.12992	3.89564	2.95020	2.18912	2.54744	2.84776	2.64658	5.10410	3.83076	229 r - - -
	2.68618	4.42225	2.77519	2.73123	3.46354	2.40513	3.72494	3.29354	2.67741	2.69355	4.24690	2.90347	2.73739	3.18146	2.89801	2.37887	2.77519	2.98518	4.58477	3.61503	
	0.02334	4.16489	4.88723	0.61958	0.77255	0.48576	0.95510														
209	2.86363	5.04355	3.29516	2.65977	4.37983	3.60443	3.64938	3.76048	1.68365	2.83195	4.12004	2.93304	3.97294	2.78582	1.69263	2.87600	3.06115	3.24759	5.42680	4.17410	230 k - - -
	2.68618	4.42225	2.77519	2.73123	3.46354	2.40513	3.72494	3.29354	2.67741	2.69355	4.24690	2.90347	2.73739	3.18146	2.89801	2.37887	2.77519	2.98518	4.58477	3.61503	
	0.02334	4.16489	4.88723	0.61958	0.77255	0.48576	0.95510														
210	2.84554	5.01757	3.12846	2.63182	4.41209	3.53047	3.70694	3.81245	1.79402	3.34542	4.18423	3.10209	1.82313	2.85272	2.32856	2.86279	3.07663	3.47040	5.48992	4.22310	231 k - - -
	2.68618	4.42225	2.77519	2.73123	3.46354	2.40513	3.72494	3.29354	2.67741	2.69355	4.24690	2.90347	2.73739	3.18146	2.89801	2.37887	2.77519	2.98518	4.58477	3.61503	
	0.16929	4.16489	1.96466	0.61958	0.77255	0.48576	0.95510														
211	2.48923	4.29049	3.42922	2.86387	3.43888	2.80417	3.79648	2.60432	2.80958	2.27963	3.17436	3.27050	3.29185	3.12078	3.04457	2.77802	2.79447	2.33652	4.88545	3.64992	232 l - - -
	2.68618	4.42225	2.77519	2.73123	3.46354	2.40513	3.72494	3.29354	2.67741	2.69355	4.24690	2.90347	2.73739	3.18146	2.89801	2.37887	2.77519	2.98518	4.58477	3.61503	
	0.02696	4.02256	4.74491	0.61958	0.77255	0.40204	1.10549														
212	2.91829	4.36627	4.36197	3.82936	1.53403	4.01795	4.27788	2.01781	3.70336	2.00908	3.22397	4.02136	4.38108	3.91235	3.87976	3.34641	2.69513	2.24748	4.79234	3.44254	233 f - - -
	2.68618	4.42225	2.77519	2.73123	3.46354	2.40513	3.72494	3.29354	2.67741	2.69355	4.24690	2.90347	2.73739	3.18146	2.89801	2.37887	2.77519	2.98518	4.58477	3.61503	
	0.02334	4.16489	4.88723	0.61958	0.77255	0.48576	0.95510														
213	2.49953	4.27039	4.26593	3.70818	3.46853	3.98958	4.38899	1.59363	3.61146	2.11614	3.37888	3.96305	4.35465	3.86831	3.84184	3.30058	2.32985	1.69239	5.11261	3.91176	234 i - - -
	2.68618	4.42225	2.77519	2.73123	3.46354	2.40513	3.72494	3.29354	2.67741	2.69355	4.24690	2.90347	2.73739	3.18146	2.89801	2.37887	2.77519	2.98518	4.58477	3.61503	
	0.02334	4.16489	4.88723	0.61958	0.77255	0.48576	0.95510														
214	2.27493	4.68023	3.09549	2.60072	4.20059	3.11403	3.77394	3.62022	2.54541	3.22150	4.03423	3.06099	2.23191	2.93610	2.30104	2.31173	2.86012	3.23788	5.44592	4.13758	235 p - - -
	2.68618	4.42225	2.77519	2.73123	3.46354	2.40513	3.72494	3.29354	2.67741	2.69355	4.24690	2.90347	2.73739	3.18146	2.89801	2.37887	2.77519	2.98518	4.58477	3.61503	
	0.02334	4.16489	4.88723	0.61958	0.77255	0.48576	0.95510														
215	2.83764	4.87614	3.39408	2.87961	4.41992	3.50244	3.81594	3.86211	2.25267	3.40670	4.27744	3.26337	4.00606	2.98569	1.16900	2.33388	3.12654	3.49785	5.50579	4.27049	236 r - - -
	2.68618	4.42225	2.77519	2.73123	3.46354	2.40513	3.72494	3.29354	2.67741	2.69355	4.24690	2.90347	2.73739	3.18146	2.89801	2.37887	2.77519	2.98518	4.58477	3.61503	
	0.02334	4.16489	4.88723	0.61958	0.77255	0.48576	0.95510														
216	2.66573	4.31398	3.63832	3.07135	3.18135	3.65358	3.13795	2.79778	2.94516	1.85871	3.27972	3.42571	4.02802	3.27394	3.10966	2.81608	2.89561	2.58789	4.69019	2.36141	237 l - - -
	2.68618	4.42225	2.77519	2.73123	3.46354	2.40513	3.72494	3.29354	2.67741	2.69355	4.24690	2.90347	2.73739	3.18146	2.89801	2.37887	2.77519	2.98518	4.58477	3.61503	
	0.02334	4.16489	4.88723	0.61958	0.77255	0.48576	0.95510														
217	2.59656	4.19861	3.70076	3.12776	2.97042	2.70797	3.90710	2.22872	3.05091	2.14685	3.30251	3.46488	3.75176	3.32948	2.95216	2.89337	2.82973	2.28197	4.79929	3.58530	238 l - - -
	2.68618	4.42225	2.77519	2.73123	3.46354	2.40513	3.72494	3.29354	2.67741	2.69355	4.24690	2.90347	2.73739	3.18146	2.89801	2.37887	2.77519	2.98518	4.58477	3.61503	
	0.02334	4.16489	4.88723	0.61958	0.77255	0.48576	0.95510														
218	2.26107	4.52212	3.11050	2.66573	3.69736	3.50630	3.73345	3.07371	2.62254	2.29220	2.91429	3.13478	3.89258	2.72859	2.70813	2.64292	2.77192	2.81663	5.07543	3.80396	239 a - - -
	2.68618	4.42225	2.77519	2.73123	3.46354	2.40513	3.72494	3.29354	2.67741	2.69355	4.24690	2.90347	2.73739	3.18146	2.89801	2.37887	2.77519	2.98518	4.58477	3.61503	
	0.02334	4.16489	4.88723	0.61958	0.77255	0.48576	0.95510														
219	2.44047	3.99037	4.20515	3.65215	3.46062	3.56799	4.31765	2.09832	3.54589	2.14828	3.39632	3.88112	4.26417	3.80510	3.77058	3.17720	3.00821	1.27168	5.06132	3.85730	240 v - - -

	2.68618	4.42225	2.77519	2.73123	3.46354	2.40513	3.72494	3.29354	2.67741	2.69355	4.24690	2.90347	2.73739	3.18146	2.89801	2.37887	2.77519	2.98518	4.58477	3.61503	
	0.02334	4.16489	4.88723	0.61958	0.77255	0.48576	0.95510														
220	2.15001	4.97190	2.52547	2.19225	4.25483	3.05724	3.62211	3.69914	2.40246	3.25317	4.03815	2.82948	3.80946	2.74837	2.80240	2.43938	2.81613	3.33047	5.45016	3.42912	241 a - - -
	2.68618	4.42225	2.77519	2.73123	3.46354	2.40513	3.72494	3.29354	2.67741	2.69355	4.24690	2.90347	2.73739	3.18146	2.89801	2.37887	2.77519	2.98518	4.58477	3.61503	
	0.02334	4.16489	4.88723	0.61958	0.77255	0.48576	0.95510														
221	1.97374	4.60005	3.04212	2.41061	3.83171	3.45548	3.72534	3.20123	2.59151	2.61915	3.72641	3.08028	3.86725	2.92864	3.02232	2.37845	2.69717	2.68239	5.17992	3.88894	242 a - - -
	2.68618	4.42225	2.77519	2.73123	3.46354	2.40513	3.72494	3.29354	2.67741	2.69355	4.24690	2.90347	2.73739	3.18146	2.89801	2.37887	2.77519	2.98518	4.58477	3.61503	
	0.02334	4.16489	4.88723	0.61958	0.77255	0.48576	0.95510														
222	2.63264	4.67071	2.92426	2.31862	4.19313	1.45437	3.91805	3.53181	2.79005	3.23356	4.10395	3.10164	3.88932	3.12577	3.19675	2.72453	2.97154	2.72422	5.51387	4.20965	243 g - - -
	2.68618	4.42225	2.77519	2.73123	3.46354	2.40513	3.72494	3.29354	2.67741	2.69355	4.24690	2.90347	2.73739	3.18146	2.89801	2.37887	2.77519	2.98518	4.58477	3.61503	
	0.02334	4.16489	4.88723	0.61958	0.77255	0.48576	0.95510														
223	2.79816	5.02571	3.18701	2.58241	4.35980	3.55050	3.64031	3.75737	1.65019	2.85938	4.10367	3.06592	3.28927	2.77249	1.94257	2.70747	3.00680	3.41792	5.43559	4.15505	244 k - - -
	2.68618	4.42225	2.77519	2.73123	3.46354	2.40513	3.72494	3.29354	2.67741	2.69355	4.24690	2.90347	2.73739	3.18146	2.89801	2.37887	2.77519	2.98518	4.58477	3.61503	
	0.02334	4.16489	4.88723	0.61958	0.77255	0.48576	0.95510														
224	2.17714	4.43695	2.83156	1.99748	4.22802	3.14678	3.61813	3.66808	2.18723	3.22672	4.01215	2.91272	3.45546	2.74447	2.86482	2.61500	2.84886	3.20939	5.42632	4.06098	245 e - - -
	2.68618	4.42225	2.77519	2.73123	3.46354	2.40513	3.72494	3.29354	2.67741	2.69355	4.24690	2.90347	2.73739	3.18146	2.89801	2.37887	2.77519	2.98518	4.58477	3.61503	
	0.02334	4.16489	4.88723	0.61958	0.77255	0.48576	0.95510														
225	2.81751	5.32678	1.71911	1.67260	4.62108	3.11659	3.22906	4.11547	2.57803	3.36760	4.40505	2.80643	3.85876	2.84654	3.10870	2.74846	3.07162	3.69786	5.76975	4.32444	246 e - - -
	2.68618	4.42225	2.77519	2.73123	3.46354	2.40513	3.72494	3.29354	2.67741	2.69355	4.24690	2.90347	2.73739	3.18146	2.89801	2.37887	2.77519	2.98518	4.58477	3.61503	
	0.02334	4.16489	4.88723	0.61958	0.77255	0.48576	0.95510														
226	2.75787	5.20973	2.31458	1.49837	4.52763	3.35569	3.68970	4.00051	2.51938	3.51562	4.30225	2.83585	3.84438	2.81976	2.90174	2.37392	2.75919	3.59358	5.68216	4.26345	247 e - - -
	2.68618	4.42225	2.77519	2.73123	3.46354	2.40513	3.72494	3.29354	2.67741	2.69355	4.24690	2.90347	2.73739	3.18146	2.89801	2.37887	2.77519	2.98518	4.58477	3.61503	
	0.02334	4.16489	4.88723	0.61958	0.77255	0.48576	0.95510														
227	2.63824	4.42710	3.94210	3.42251	3.34265	3.85280	4.20865	2.50182	3.24690	1.18161	3.24728	3.74647	4.24279	3.20227	3.50023	3.17410	3.10052	2.27105	5.04189	3.85133	248 l - - -
	2.68618	4.42225	2.77514	2.73123	3.46354	2.40513	3.72495	3.29354	2.67741	2.69355	4.24690	2.90347	2.73740	3.18146	2.89801	2.37887	2.77520	2.98518	4.58477	3.61503	
	0.04227	3.38577	4.88723	0.53555	0.88031	0.48576	0.95510														
228	3.12420	4.43323	4.89684	4.35263	2.52233	4.44298	4.82600	1.27588	4.23495	1.93931	3.23665	4.51614	4.71530	4.39641	4.35858	3.79378	3.36568	1.50571	5.23782	4.03045	250 i - - -
	2.68618	4.42225	2.77519	2.73123	3.46354	2.40513	3.72494	3.29354	2.67741	2.69355	4.24690	2.90347	2.73739	3.18146	2.89801	2.37887	2.77519	2.98518	4.58477	3.61503	
	0.02334	4.16489	4.88723	0.61958	0.77255	0.48576	0.95510														
229	2.44070	4.48262	3.16595	2.78499	4.29452	2.56450	3.97966	3.72339	2.86029	3.35269	4.16616	2.67710	3.80183	3.17617	3.27175	1.79918	1.80295	3.26562	5.58547	4.30172	251 s - - -
	2.68618	4.42225	2.77519	2.73123	3.46354	2.40513	3.72494	3.29354	2.67741	2.69355	4.24690	2.90347	2.73739	3.18146	2.89801	2.37887	2.77519	2.98518	4.58477	3.61503	
	0.02334	4.16489	4.88723	0.61958	0.77255	0.48576	0.95510														
230	2.29683	5.03696	2.25409	2.34004	4.38843	3.00395	3.67360	3.84468	2.27609	3.38516	4.17083	2.88165	2.45700	2.80378	2.97213	2.61719	2.92804	3.45028	5.56926	4.18102	252 d - - -
	2.68618	4.42225	2.77519	2.73123	3.46354	2.40513	3.72494	3.29354	2.67741	2.69355	4.24690	2.90347	2.73739	3.18146	2.89801	2.37887	2.77519	2.98518	4.58477	3.61503	
	0.02334	4.16489	4.88723	0.61958	0.77255	0.48576	0.95510														
231	2.57489	4.85418	2.68342	2.38004	4.10659	2.86403	3.62825	3.53058	2.33357	2.85503	3.43632	2.94477	3.81761	2.47373	2.88248	2.62922	2.48239	3.08890	5.34772	4.00209	253 k - - -
	2.68618	4.42225	2.77519	2.73123	3.46354	2.40513	3.72494	3.29354	2.67741	2.69355	4.24690	2.90347	2.73739	3.18146	2.89801	2.37887	2.77519	2.98518	4.58477	3.61503	
	0.02334	4.16489	4.88723	0.61958	0.77255	0.48576	0.95510														
232	3.04300	5.58218	1.00083	2.01699	4.89826	3.29697	3.87826	4.38775	2.92389	3.92182	4.79267	2.77300	3.93706	3.05250	3.52760	2.93001	3.09710	3.96803	6.09825	4.58593	254 d - - -
	2.68618	4.42225	2.77519	2.73123	3.46354	2.40513	3.72494	3.29354	2.67741	2.69355	4.24690	2.90347	2.73739	3.18146	2.89801	2.37887	2.77519	2.98518	4.58477	3.61503	
	0.02334	4.16489	4.88723	0.61958	0.77255	0.48576	0.95510														
233	3.04501	5.23814	3.45332	2.77471	4.68827	3.70266	3.65117	4.03639	1.44832	3.47251	4.31534	3.20347	4.05926	2.59755	1.51255	3.02958	2.93103	3.69909	5.52528	4.33193	255 k - - -
	2.68618	4.42225	2.77519	2.73123	3.46354	2.40513	3.72494	3.29354	2.67741	2.69355	4.24690	2.90347	2.73739	3.18146	2.89801	2.37887	2.77519	2.98518	4.58477	3.61503	
	0.02334	4.16489	4.88723	0.61958	0.77255	0.48576	0.95510														
234	2.80407	5.08653	3.15536	2.55294	4.43757	3.54547	3.62692	3.84210	1.68248	3.16798	4.15196	3.04388	3.92213	2.21513	2.18046	2.79611	2.53009	3.48594	5.47382	4.18471	256 k - - -
	2.68618	4.42225	2.77519	2.73123	3.46354	2.40513	3.72494	3.29354	2.67741	2.69355	4.24690	2.90347	2.73739	3.18146	2.89801	2.37887	2.77519	2.98518	4.58477	3.61503	
	0.02334	4.16489	4.88723	0.61958	0.77255	0.48576	0.95510														
235	3.12639	4.42914	4.91189	4.36147	2.80193	4.46290	4.86007	1.54555	4.24678	1.78514	3.23296	4.53258	4.72551	4.40806	4.37118	3.81087	3.36511	1.24930	5.27973	4.10620	257 v - - -
	2.68618	4.42225	2.77519	2.73123	3.46354	2.40513	3.72494	3.29354	2.67741	2.69355	4.24690	2.90347	2.73739	3.18146	2.89801	2.37887	2.77519	2.98518	4.58477	3.61503	
	0.02334	4.16489	4.88723	0.61958	0.77255	0.48576	0.95510														
236	1.62996	2.70737	3.92842	3.54934	4.21993	1.44030	4.41175	3.58372	3.49481	3.32326	4.16533	3.53232	3.77707	3.75040	3.74624	2.39438	2.74756	3.08739	5.61708	4.44443	258 g - - -
	2.68618	4.42225	2.77519	2.73123	3.46354	2.40513	3.72494	3.29354	2.67741	2.69355	4.24690	2.90347	2.73739	3.18146	2.89801	2.37887	2.77519	2.98518	4.58477	3.61503	
	0.02334	4.16489	4.88723	0.61958	0.77255	0.48576	0.95510														
237	2.25905	4.15922	3.91480	3.33584	2.62059	3.70942	3.99876	2.44791	2.86205	2.02134	2.50386	3.61585	4.07522	3.48793	3.47586	2.98608	2.86512	2.28860	4.76647	3.56923	259 l - - -
	2.68618	4.42225	2.77519	2.73123	3.46354	2.40513	3.72494	3.29354	2.67741	2.69355	4.24690	2.90347	2.73739	3.18146	2.89801	2.37887	2.77519	2.98518	4.58477	3.61503	
	0.02334	4.16489	4.88723	0.61958	0.77255	0.48576	0.95510														
238	2.20697	4.22269	3.83685	3.27676	3.38182	2.79482	4.04321	2.19116	3.19542	1.89083	3.34506	3.58604	4.08268	3.47137	3.47310	2.97015	2.89379	2.12892	4.90873	3.69982	260 l - - -
	2.68618	4.42225	2.77519	2.73123	3.46354	2.40513	3.72494	3.29354	2.67741	2.69355	4.24690	2.90347	2.73739	3.18146	2.89801	2.37887	2.77519	2.98518	4.58477	3.61503	
	0.02334	4.16489	4.88723	0.61958	0.77255	0.48576	0.95510														
239	2.78382	4.36868	4.49924	3.94901	1.71429	4.11432	4.38935	2.23612	3.82271	1.79340	3.17791	4.13186	4.44703	4.00858	3.97903	3.43950	3.20375	1.73283	4.87943	3.58302	261 f - - -
	2.68618	4.42225	2.77519	2.73123	3.46354	2.40513	3.72494	3.29354	2.67741	2.69355	4.24690	2.90347	2.73739	3.18146	2.89801	2.37887	2.77519	2.98518	4.58477	3.61503	
	0.02334	4.16489	4.88723	0.61958	0.77255	0.48576	0.95510														
240	2.11766	4.24210	3.69782	3.14194	3.41065	3.34191	3.96326	2.65003	3.06082	1.80685	2.97606	3.47535	4.01262	3.35529	3.36061	2.88159	2.48183	2.44579	4.90553	3.69160	262 l - - -
	2.68618	4.42225	2.77519	2.73123	3.46354	2.40513	3.72494	3.29354	2.67741	2.69355	4.24690	2.90347	2.73739	3.18146	2.89801	2.37887	2.77519	2.98518	4.58477	3.61503	
	0.02334	4.16489	4.88723	0.61958	0.77255	0.48576	0.95510														
241	1.79394	4.33076	4.28293	3.74305	3.55479	4.01125	4.48968	1.76998	3.65207	2.22621	3.43729	4.00815	4.40265	3.92519	3.90288	3.34012	3.13015	1.53019	5.24894	4.04299	263 v - - -
	2.68618	4.42225	2.77519	2.73123	3.46354	2.40513	3.72494	3.29354	2.67741	2.69355	4.24690	2.90347	2.73739	3.18146	2.89801	2.37887	2.77519	2.98518	4.58477	3.61503	

	0.02334	4.16489	4.88723	0.61958	0.77255	0.48576	0.95510														
242	2.05447	4.32388	3.56662	3.03081	3.59060	2.15756	3.96478	2.47061	2.97428	2.32964	3.53755	3.39247	3.97403	3.28684	3.31691	2.80927	2.86358	2.44866	5.06147	3.82804	264 a - - -
	2.68618	4.42225	2.77519	2.73123	3.46354	2.40513	3.72494	3.29354	2.67741	2.69355	4.24690	2.90347	2.73739	3.18146	2.89801	2.37887	2.77519	2.98518	4.58477	3.61503	
	0.02334	4.16489	4.88723	0.61958	0.77255	0.48576	0.95510														
243	2.56492	4.31402	3.89044	3.47393	3.85307	3.42020	4.36867	2.61905	3.37365	2.70702	3.77729	3.66259	4.05087	3.70869	3.64657	2.83624	1.30820	1.87736	5.42922	4.20488	265 t - - -
	2.68618	4.42225	2.77519	2.73123	3.46354	2.40513	3.72494	3.29354	2.67741	2.69355	4.24690	2.90347	2.73739	3.18146	2.89801	2.37887	2.77519	2.98518	4.58477	3.61503	
	0.02334	4.16489	4.88723	0.61958	0.77255	0.48576	0.95510														
244	2.85384	4.27336	4.36450	3.79251	3.31441	4.03370	4.37773	1.49226	3.67669	1.92008	2.84586	4.01981	3.34780	3.89799	3.86398	3.33947	3.09031	1.78371	5.00584	3.83166	266 i - - -
	2.68618	4.42225	2.77519	2.73123	3.46354	2.40513	3.72494	3.29354	2.67741	2.69355	4.24690	2.90347	2.73739	3.18146	2.89801	2.37887	2.77519	2.98518	4.58477	3.61503	
	0.02334	4.16489	4.88723	0.61958	0.77255	0.48576	0.95510														
245	2.51789	4.27164	4.30164	3.74521	3.40879	3.77904	4.38550	2.02014	3.63061	1.60891	3.32159	3.98076	4.34993	3.88132	3.84081	3.29966	3.07742	1.50986	5.07227	3.87990	267 v - - -
	2.68618	4.42225	2.77519	2.73123	3.46354	2.40513	3.72494	3.29354	2.67741	2.69355	4.24690	2.90347	2.73739	3.18146	2.89801	2.37887	2.77519	2.98518	4.58477	3.61503	
	0.02334	4.16489	4.88723	0.61958	0.77255	0.48576	0.95510														
246	2.46435	4.37826	4.59982	4.03991	3.38428	4.25072	4.63916	1.49337	3.92533	1.51724	3.13290	4.26366	4.55612	4.13610	4.10297	3.57674	3.24672	1.81652	5.19839	4.04047	268 i - - -
	2.68618	4.42225	2.77519	2.73123	3.46354	2.40513	3.72494	3.29354	2.67741	2.69355	4.24690	2.90347	2.73739	3.18146	2.89801	2.37887	2.77519	2.98518	4.58477	3.61503	
	0.02334	4.16489	4.88723	0.61958	0.77255	0.48576	0.95510														
247	2.92310	4.37347	4.41543	3.90027	3.63189	4.08206	4.65153	1.93312	3.77591	2.20955	3.49439	4.13899	4.49399	4.07721	4.01355	3.44044	2.55470	1.05314	5.38930	4.17514	269 v - - -
	2.68618	4.42225	2.77519	2.73123	3.46354	2.40513	3.72494	3.29354	2.67741	2.69355	4.24690	2.90347	2.73739	3.18146	2.89801	2.37887	2.77519	2.98518	4.58477	3.61503	
	0.02334	4.16489	4.88723	0.61958	0.77255	0.48576	0.95510														
248	1.94376	4.17921	4.09641	3.52099	2.84663	3.67207	4.13417	1.89705	3.41632	2.04120	3.14501	3.76748	4.17608	3.65236	3.62881	3.10406	2.93592	2.14427	4.84185	3.64501	270 i - - -
	2.68618	4.42225	2.77519	2.73123	3.46354	2.40513	3.72494	3.29354	2.67741	2.69355	4.24690	2.90347	2.73739	3.18146	2.89801	2.37887	2.77519	2.98518	4.58477	3.61503	
	0.02334	4.16489	4.88723	0.61958	0.77255	0.48576	0.95510														
249	2.03573	4.14228	3.95527	3.37955	2.85672	3.15064	4.02765	2.13778	3.28352	2.29549	3.04578	3.64363	4.08615	3.52930	3.51451	2.99412	2.86435	2.01480	4.78672	3.58654	271 v - - -
	2.68618	4.42225	2.77519	2.73123	3.46354	2.40513	3.72494	3.29354	2.67741	2.69355	4.24690	2.90347	2.73739	3.18146	2.89801	2.37887	2.77519	2.98518	4.58477	3.61503	
	0.02334	4.16489	4.88723	0.61958	0.77255	0.48576	0.95510														
250	1.92395	4.36686	3.39510	2.92508	4.01642	1.98010	3.99235	3.39202	2.91556	2.62841	3.92843	3.25909	3.82438	3.23358	3.29469	2.16168	2.54563	3.01937	5.37828	4.12783	272 a - - -
	2.68618	4.42225	2.77519	2.73123	3.46354	2.40513	3.72494	3.29354	2.67741	2.69355	4.24690	2.90347	2.73739	3.18146	2.89801	2.37887	2.77519	2.98518	4.58477	3.61503	
	0.02334	4.16489	4.88723	0.61958	0.77255	0.48576	0.95510														
251	2.15902	4.31948	3.50189	2.95577	3.40297	3.57684	3.84702	2.81157	2.89008	2.52810	2.51677	2.93904	3.97296	3.20538	3.22587	2.84170	2.85218	2.59080	4.86954	2.53072	273 a - - -
	2.68618	4.42225	2.77519	2.73123	3.46354	2.40513	3.72494	3.29354	2.67741	2.69355	4.24690	2.90347	2.73739	3.18146	2.89801	2.37887	2.77519	2.98518	4.58477	3.61503	
	0.02334	4.16489	4.88723	0.61958	0.77255	0.48576	0.95510														
252	2.05821	4.26668	3.54764	2.98120	2.99979	3.11739	3.85851	2.75817	2.92659	2.38460	3.03467	3.36089	3.96407	3.15089	3.25969	2.47273	2.82246	2.36705	4.86765	3.64102	274 a - - -
	2.68618	4.42225	2.77519	2.73123	3.46354	2.40513	3.72494	3.29354	2.67741	2.69355	4.24690	2.90347	2.73739	3.18146	2.89801	2.37887	2.77519	2.98518	4.58477	3.61503	
	0.02334	4.16489	4.88723	0.61958	0.77255	0.48576	0.95510														
253	2.57443	4.22611	3.71122	3.14692	3.38071	3.63381	3.95227	2.13555	3.07037	2.34628	3.35449	3.48537	4.02681	3.21116	3.36782	2.35644	2.53417	1.99475	4.87149	3.65605	275 v - - -
	2.68618	4.42225	2.77519	2.73123	3.46354	2.40513	3.72494	3.29354	2.67741	2.69355	4.24690	2.90347	2.73739	3.18146	2.89801	2.37887	2.77519	2.98518	4.58477	3.61503	
	0.02334	4.16489	4.88723	0.61958	0.77255	0.48576	0.95510														
254	2.00961	4.26467	3.66922	3.37625	4.24894	3.11707	4.37569	3.35629	3.34873	3.24987	4.18593	3.49808	3.85587	3.67668	3.62112	2.57890	1.04599	2.96521	5.67403	4.46968	276 t - - -
	2.68618	4.42225	2.77519	2.73123	3.46354	2.40513	3.72494	3.29354	2.67741	2.69355	4.24690	2.90347	2.73739	3.18146	2.89801	2.37887	2.77519	2.98518	4.58477	3.61503	
	0.02334	4.16489	4.88723	0.61958	0.77255	0.48576	0.95510														
255	2.35179	5.00038	2.79462	2.40455	4.36327	3.38839	3.69283	3.80262	2.16764	3.35408	4.15656	1.86117	3.85366	2.74740	2.88357	2.43616	2.95765	3.42593	5.54059	4.18287	277 n - - -
	2.68618	4.42225	2.77519	2.73123	3.46354	2.40513	3.72494	3.29354	2.67741	2.69355	4.24690	2.90347	2.73739	3.18146	2.89801	2.37887	2.77519	2.98518	4.58477	3.61503	
	0.02334	4.16489	4.88723	0.61958	0.77255	0.48576	0.95510														
256	2.30658	5.03206	2.77311	2.08569	4.35719	3.00111	3.65120	3.80982	2.24975	3.34920	4.13330	2.61217	3.82195	2.77816	2.91527	2.10666	2.91149	3.42334	5.53226	4.15048	278 e - - -
	2.68618	4.42225	2.77519	2.73123	3.46354	2.40513	3.72494	3.29354	2.67741	2.69355	4.24690	2.90347	2.73739	3.18146	2.89801	2.37887	2.77519	2.98518	4.58477	3.61503	
	0.02334	4.16489	4.88723	0.61958	0.77255	0.48576	0.95510														
257	2.45086	5.02589	2.90430	2.30040	4.33897	3.43054	3.62797	3.77640	1.98382	3.31071	4.09885	2.92944	3.83999	2.11044	2.75737	2.59022	2.54351	3.40324	5.48314	4.12815	279 k - - -
	2.68618	4.42225	2.77519	2.73123	3.46354	2.40513	3.72494	3.29354	2.67741	2.69355	4.24690	2.90347	2.73739	3.18146	2.89801	2.37887	2.77519	2.98518	4.58477	3.61503	
	0.02334	4.16489	4.88723	0.61958	0.77255	0.48576	0.95510														
258	3.88017	5.04882	4.83415	4.57404	1.31961	4.57018	3.53373	3.54064	4.40643	2.83045	4.12238	4.21880	4.86736	4.30603	4.38917	3.95150	4.09748	3.47294	3.65146	0.94068	280 y - - -
	2.68618	4.42225	2.77519	2.73123	3.46354	2.40513	3.72494	3.29354	2.67741	2.69355	4.24690	2.90347	2.73739	3.18146	2.89801	2.37887	2.77519	2.98518	4.58477	3.61503	
	0.02334	4.16489	4.88723	0.61958	0.77255	0.48576	0.95510														
259	3.18863	4.88603	3.82888	3.71728	4.67093	3.55752	4.76170	4.30328	3.80198	3.89323	4.96207	3.98991	0.42795	4.18131	4.00541	3.36743	3.65485	3.91304	5.76614	4.79867	281 P - - -
	2.68618	4.42225	2.77519	2.73123	3.46354	2.40513	3.72494	3.29354	2.67741	2.69355	4.24690	2.90347	2.73739	3.18146	2.89801	2.37887	2.77519	2.98518	4.58477	3.61503	
	0.02334	4.16489	4.88723	0.61958	0.77255	0.48576	0.95510														
260	2.64766	4.40228	3.00379	2.89275	3.56455	3.60624	3.86842	2.04012	2.82775	2.57511	3.50459	3.31726	3.99277	3.16417	2.61425	2.86009	2.88374	2.06150	5.01976	3.77387	282 i - - -
	2.68618	4.42225	2.77519	2.73123	3.46354	2.40513	3.72494	3.29354	2.67741	2.69355	4.24690	2.90347	2.73739	3.18146	2.89801	2.37887	2.77519	2.98518	4.58477	3.61503	
	0.02334	4.16489	4.88723	0.61958	0.77255	0.48576	0.95510														
261	2.13614	4.26382	3.78053	3.35727	3.97245	3.26065	4.28897	2.91169	3.28811	2.91756	3.89031	3.53555	3.92485	3.60397	3.58162	2.67381	1.28076	2.26576	5.45888	4.24743	283 t - - -
	2.68618	4.42225	2.77519	2.73123	3.46354	2.40513	3.72494	3.29354	2.67741	2.69355	4.24690	2.90347	2.73739	3.18146	2.89801	2.37887	2.77519	2.98518	4.58477	3.61503	
	0.02334	4.16489	4.88723	0.61958	0.77255	0.48576	0.95510														
262	2.94548	4.37902	4.47893	3.97188	3.60951	4.11731	4.69883	1.14932	3.84979	2.26281	3.46300	4.19514	4.52391	4.13764	4.07233	3.48203	2.39655	1.72405	5.39349	4.19086	284 i - - -
	2.68618	4.42225	2.77519	2.73123	3.46354	2.40513	3.72494	3.29354	2.67741	2.69355	4.24690	2.90347	2.73739	3.18146	2.89801	2.37887	2.77519	2.98518	4.58477	3.61503	
	0.02334	4.16489	4.88723	0.61958	0.77255	0.48576	0.95510														
263	3.18863	4.88603	3.82888	3.71728	4.67093	3.55752	4.76170	4.30328	3.80198	3.89323	4.96207	3.98991	0.42795	4.18131	4.00541	3.36743	3.65485	3.91304	5.76614	4.79867	285 P - - -
	2.68618	4.42225	2.77519	2.73123	3.46354	2.40513	3.72494	3.29354	2.67741	2.69355	4.24690	2.90347	2.73739	3.18146	2.89801	2.37887	2.77519	2.98518	4.58477	3.61503	
	0.02334	4.16489	4.88723	0.61958	0.77255	0.48576	0.95510														

```
264   3.43734  4.74801  5.00420  4.47935  3.17954  4.69483  5.02483  1.84448  4.29201  0.79547  2.98979  4.73411  4.88240  4.44072  4.40886  4.08174  3.67011  2.23084  5.29594  4.18426    286 l - - -
      2.68618  4.42225  2.77519  2.73123  3.46354  2.40513  3.72494  3.29354  2.67741  2.69355  4.24690  2.90347  2.73739  3.18146  2.89801  2.37887  2.77519  2.98518  4.58477  3.61503
      0.02334  4.16489  4.88723  0.61958  0.77255  0.48576  0.95510
265   3.15245  5.12453  3.13482  2.90531  4.33089  3.58519  4.06225  4.01824  2.70785  3.48982  4.52006  3.36365  4.14957  0.79098  2.98153  3.19274  3.45419  3.73260  5.55451  4.27303    287 q - - -
      2.68618  4.42225  2.77519  2.73123  3.46354  2.40513  3.72494  3.29354  2.67741  2.69355  4.24690  2.90347  2.73739  3.18146  2.89801  2.37887  2.77519  2.98518  4.58477  3.61503
      0.02334  4.16489  4.88723  0.61958  0.77255  0.48576  0.95510
266   1.68418  4.84313  2.89841  2.19950  4.18044  3.40401  3.72339  3.56997  2.28227  3.18882  4.01773  2.99226  3.86615  2.88492  2.92619  2.70882  2.64479  3.23570  5.42905  4.10342    288 a - - -
      2.68618  4.42225  2.77519  2.73123  3.46354  2.40513  3.72494  3.29354  2.67741  2.69355  4.24690  2.90347  2.73739  3.18146  2.89801  2.37887  2.77519  2.98518  4.58477  3.61503
      0.02334  4.16489  4.88723  0.61958  0.77255  0.48576  0.95510
267   2.37749  4.31552  3.46584  3.24397  4.45839  1.15648  4.35841  3.83287  3.33814  3.55957  4.41172  3.41743  3.82428  3.63791  3.63860  2.55406  1.88550  3.29978  5.78732  4.58611    289 g - - -
      2.68618  4.42225  2.77519  2.73123  3.46354  2.40513  3.72494  3.29354  2.67741  2.69355  4.24690  2.90347  2.73739  3.18146  2.89801  2.37887  2.77519  2.98518  4.58477  3.61503
      0.02334  4.16489  4.88723  0.61958  0.77255  0.48576  0.95510
268   2.62025  4.42342  3.37467  2.80658  3.10686  3.55552  3.78307  2.93870  2.40562  1.93814  3.52157  3.08430  3.93774  3.06411  2.78307  2.79926  2.58384  2.70322  4.98406  3.73477    290 l - - -
      2.68618  4.42225  2.77519  2.73123  3.46354  2.40513  3.72494  3.29354  2.67741  2.69355  4.24690  2.90347  2.73739  3.18146  2.89801  2.37887  2.77519  2.98518  4.58477  3.61503
      0.02334  4.16489  4.88723  0.61958  0.77255  0.48576  0.95510
269   2.60152  4.33814  3.03873  2.88416  3.46298  3.57380  3.82265  2.71822  2.83801  2.20281  2.72522  3.29772  3.95266  2.71705  3.19762  2.81699  2.83309  2.40258  4.91313  3.04454    291 l - - -
      2.68618  4.42225  2.77519  2.73123  3.46354  2.40513  3.72494  3.29354  2.67741  2.69355  4.24690  2.90347  2.73739  3.18146  2.89801  2.37887  2.77519  2.98518  4.58477  3.61503
      0.02334  4.16489  4.88723  0.61958  0.77255  0.48576  0.95510
270   2.72180  5.00920  2.72603  2.46681  4.28699  3.47780  3.63196  3.73280  2.05602  3.27240  4.07642  2.98051  3.87325  2.76401  1.79205  2.72047  2.94303  3.38036  5.43290  3.18915    292 r - - -
      2.68618  4.42225  2.77519  2.73123  3.46354  2.40513  3.72494  3.29354  2.67741  2.69355  4.24690  2.90347  2.73739  3.18146  2.89801  2.37887  2.77519  2.98518  4.58477  3.61503
      0.25017  4.16489  1.58085  0.61958  0.77255  0.48576  0.95510
271   2.14129  4.25120  3.20186  2.86726  4.19979  1.84223  4.01321  3.59634  2.93730  3.26942  4.09760  3.15677  2.57113  3.24516  3.30446  2.42274  2.31754  3.11623  5.52303  4.27638    293 g - - -
      2.68618  4.42225  2.77519  2.73123  3.46354  2.40513  3.72494  3.29354  2.67741  2.69355  4.24690  2.90347  2.73739  3.18146  2.89801  2.37887  2.77519  2.98518  4.58477  3.61503
      0.02920  3.94392  4.66626  0.61958  0.77255  0.37059  1.17225
272   2.89529  4.32842  4.35194  3.78052  3.26434  4.05371  4.38186  1.43316  3.65198  1.73710  2.66771  4.02470  3.40820  3.87824  3.84301  3.35960  3.13001  2.13682  5.00194  3.83920    294 i - - -
      2.68618  4.42225  2.77519  2.73123  3.46354  2.40513  3.72494  3.29354  2.67741  2.69355  4.24690  2.90347  2.73739  3.18146  2.89801  2.37887  2.77519  2.98518  4.58477  3.61503
      0.02334  4.16489  4.88723  0.61958  0.77255  0.48576  0.95510
273   2.64729  5.05981  2.62691  2.20786  4.36921  3.40138  3.60624  3.82679  1.88939  2.97950  4.11455  2.75726  3.80836  2.54325  2.81860  2.62447  2.54624  3.43189  5.50915  4.12543    295 k - - -
      2.68618  4.42225  2.77519  2.73123  3.46354  2.40513  3.72494  3.29354  2.67741  2.69355  4.24690  2.90347  2.73739  3.18146  2.89801  2.37887  2.77519  2.98518  4.58477  3.61503
      0.02334  4.16489  4.88723  0.61958  0.77255  0.48576  0.95510
274   2.47092  4.43750  3.34708  2.81916  3.70412  3.44195  3.83845  3.07980  2.74484  2.52455  3.65775  3.23135  1.87686  3.10169  3.11381  2.56006  2.74732  2.81290  4.63505  3.84220    296 p - - -
      2.68618  4.42225  2.77519  2.73123  3.46354  2.40513  3.72494  3.29354  2.67741  2.69355  4.24690  2.90347  2.73739  3.18146  2.89801  2.37887  2.77519  2.98518  4.58477  3.61503
      0.02334  4.16489  4.88723  0.61958  0.77255  0.48576  0.95510
275   2.77920  4.28229  4.05608  3.48113  3.25305  3.86658  4.13489  1.82600  2.88957  1.53154  3.21738  3.76369  4.21479  3.60606  3.52137  3.15791  3.00968  2.28967  4.87633  3.44002    297 l - - -
      2.68611  4.42241  2.77532  2.73091  3.46341  2.40513  3.72511  3.29362  2.67757  2.69371  4.24706  2.90363  2.73725  3.18163  2.89817  2.37903  2.77444  2.98512  4.58493  3.61520
      0.24478  1.56264  4.88723  1.35746  0.29748  0.48576  0.95510
276   2.56297  5.13083  2.65480  1.95412  4.44058  2.82257  3.63991  3.90801  2.35906  3.42277  4.19625  2.73933  2.64911  2.48380  2.92041  2.65589  2.93112  3.50542  5.58707  4.18434    304 e - - -
      2.68618  4.42225  2.77519  2.73123  3.46354  2.40513  3.72494  3.29354  2.67741  2.69355  4.24690  2.90347  2.73739  3.18146  2.89801  2.37887  2.77519  2.98518  4.58477  3.61503
      0.02334  4.16489  4.88723  0.61958  0.77255  0.48576  0.95510
277   2.59463  4.22730  3.62960  2.95346  3.34864  3.61285  3.87993  2.59749  2.84012  2.33504  2.62556  3.41676  2.99773  3.27551  3.29623  2.87072  2.82643  2.08885  4.82143  3.17248    305 v - - -
      2.68618  4.42225  2.77519  2.73123  3.46354  2.40513  3.72494  3.29354  2.67741  2.69355  4.24690  2.90347  2.73739  3.18146  2.89801  2.37887  2.77519  2.98518  4.58477  3.61503
      0.02334  4.16489  4.88723  0.61958  0.77255  0.48576  0.95510
278   2.59699  5.04532  2.68977  1.93716  4.41731  2.75460  3.69442  3.87582  2.51253  3.41608  4.20554  2.57557  2.48278  2.82845  3.00556  2.44272  2.95008  3.47699  5.60030  4.21013    306 e - - -
      2.68618  4.42225  2.77519  2.73123  3.46354  2.40513  3.72494  3.29354  2.67741  2.69355  4.24690  2.90347  2.73739  3.18146  2.89801  2.37887  2.77519  2.98518  4.58477  3.61503
      0.02334  4.16489  4.88723  0.61958  0.77255  0.48576  0.95510
279   2.24010  4.97640  2.89602  2.21512  4.26488  3.41284  3.20484  3.70629  2.15831  3.25585  4.04140  2.91185  2.79306  2.67282  2.83386  2.53419  2.87048  3.27396  5.44663  4.08170    307 k - - -
      2.68618  4.42225  2.77519  2.73123  3.46354  2.40513  3.72494  3.29354  2.67741  2.69355  4.24690  2.90347  2.73739  3.18146  2.89801  2.37887  2.77519  2.98518  4.58477  3.61503
      0.02334  4.16489  4.88723  0.61958  0.77255  0.48576  0.95510
280   2.27880  4.88127  2.93033  2.23406  4.16685  3.02287  3.47124  3.59742  2.39724  3.17561  3.97408  2.93594  3.19346  2.77683  2.84659  2.40824  2.40796  3.24689  5.39390  4.04181    308 e - - -
      2.68618  4.42225  2.77519  2.73123  3.46354  2.40513  3.72494  3.29354  2.67741  2.69355  4.24690  2.90347  2.73739  3.18146  2.89801  2.37887  2.77519  2.98518  4.58477  3.61503
      0.02334  4.16489  4.88723  0.61958  0.77255  0.48576  0.95510
281   2.59337  4.32826  3.45112  2.88482  3.04928  3.56497  3.81946  2.71433  2.83432  2.25619  3.43137  3.29530  3.66126  2.86089  3.10163  2.66956  2.13281  2.53541  4.91014  3.67303    309 t - - -
      2.68618  4.42225  2.77519  2.73123  3.46354  2.40513  3.72494  3.29354  2.67741  2.69355  4.24690  2.90347  2.73739  3.18146  2.89801  2.37887  2.77519  2.98518  4.58477  3.61503
      0.02334  4.16489  4.88723  0.61958  0.77255  0.48576  0.95510
282   3.18868  4.48118  4.97579  4.49183  3.69805  4.57746  5.20584  1.43324  4.38435  2.19266  3.49288  4.69023  4.88678  4.65096  4.57493  3.97942  3.45821  0.93082  5.68859  4.47501    310 v - - -
      2.68618  4.42225  2.77519  2.73123  3.46354  2.40513  3.72494  3.29354  2.67741  2.69355  4.24690  2.90347  2.73739  3.18146  2.89801  2.37887  2.77519  2.98518  4.58477  3.61503
      0.02334  4.16489  4.88723  0.61958  0.77255  0.48576  0.95510
283   2.39564  5.06374  2.75633  2.18000  4.37362  3.39133  3.60815  3.83517  2.17328  3.35375  4.12065  2.55865  3.80324  2.44008  2.84961  2.43134  2.51209  3.43669  5.51870  4.12833    311 k - - -
      2.68618  4.42225  2.77519  2.73123  3.46354  2.40513  3.72494  3.29354  2.67741  2.69355  4.24690  2.90347  2.73739  3.18146  2.89801  2.37887  2.77519  2.98518  4.58477  3.61503
      0.02334  4.16489  4.88723  0.61958  0.77255  0.48576  0.95510
284   1.63995  4.22322  3.89957  3.44821  3.80320  3.35782  4.29465  2.67679  3.37939  2.76974  3.73667  3.61493  3.98303  3.66650  3.65093  2.65337  2.59763  1.53276  5.31556  4.11087    312 v - - -
      2.68618  4.42225  2.77519  2.73123  3.46354  2.40513  3.72494  3.29354  2.67741  2.69355  4.24690  2.90347  2.73739  3.18146  2.89801  2.37887  2.77519  2.98518  4.58477  3.61503
      0.02334  4.16489  4.88723  0.61958  0.77255  0.48576  0.95510
285   2.74742  5.21712  2.40604  1.86636  4.55299  3.40191  3.64144  4.02153  1.76230  3.50350  4.27609  2.87028  3.84608  2.75723  2.55860  2.69965  2.98131  3.60605  5.63480  4.24111    313 k - - -
      2.68618  4.42225  2.77519  2.73123  3.46354  2.40513  3.72494  3.29354  2.67741  2.69355  4.24690  2.90347  2.73739  3.18146  2.89801  2.37887  2.77519  2.98518  4.58477  3.61503
      0.02334  4.16489  4.88723  0.61958  0.77255  0.48576  0.95510
286   2.64680  4.21865  3.82720  3.26540  3.34385  3.69192  4.01605  2.49839  3.17044  2.10037  3.32203  3.57718  3.38918  3.45348  3.44171  2.97581  2.59668  1.60171  4.86818  3.39893    314 v - - -
```

	2.68618	4.42225	2.77519	2.73123	3.46354	2.40513	3.72494	3.29354	2.67741	2.69355	4.24690	2.90347	2.73739	3.18146	2.89801	2.37887	2.77519	2.98518	4.58477	3.61503	
	0.02334	4.16489	4.88723	0.61958	0.77255	0.48576	0.95510														
287	2.60945	4.87253	2.69219	2.21629	4.12917	3.42121	3.62526	3.39401	2.36132	2.83710	3.93903	2.65658	3.81506	2.70726	2.88070	2.53348	2.45372	2.85570	5.36277	4.01272	315 e - - -
	2.68618	4.42225	2.77519	2.73123	3.46354	2.40513	3.72494	3.29354	2.67741	2.69355	4.24690	2.90347	2.73739	3.18146	2.89801	2.37887	2.77519	2.98518	4.58477	3.61503	
	0.02334	4.16489	4.88723	0.61958	0.77255	0.48576	0.95510														
288	2.69621	5.14380	1.97919	2.25773	4.46179	2.81794	3.64025	3.93100	2.07923	3.43996	4.21171	2.85465	3.82168	2.75883	2.78648	2.61514	2.67381	3.52371	5.59855	4.19490	316 d - - -
	2.68618	4.42225	2.77519	2.73123	3.46354	2.40513	3.72494	3.29354	2.67741	2.69355	4.24690	2.90347	2.73739	3.18146	2.89801	2.37887	2.77519	2.98518	4.58477	3.61503	
	0.02334	4.16489	4.88723	0.61958	0.77255	0.48576	0.95510														
289	0.93139	4.25285	3.59803	3.37249	4.46348	2.10095	4.43275	3.82650	3.46348	3.57000	4.41534	3.46988	3.80608	3.73237	3.74388	2.51677	2.83628	3.27329	5.80735	4.62659	317 a - - -
	2.68618	4.42225	2.77519	2.73123	3.46354	2.40513	3.72494	3.29354	2.67741	2.69355	4.24690	2.90347	2.73739	3.18146	2.89801	2.37887	2.77519	2.98518	4.58477	3.61503	
	0.02334	4.16489	4.88723	0.61958	0.77255	0.48576	0.95510														
290	2.57723	4.49125	3.23206	2.47580	3.81100	3.42351	3.83909	3.10281	2.73863	2.85129	3.72798	3.18426	3.89620	3.07777	3.13301	2.42963	1.88821	2.24817	5.19509	3.92797	318 t - - -
	2.68618	4.42225	2.77519	2.73123	3.46354	2.40513	3.72494	3.29354	2.67741	2.69355	4.24690	2.90347	2.73739	3.18146	2.89801	2.37887	2.77519	2.98518	4.58477	3.61503	
	0.02334	4.16489	4.88723	0.61958	0.77255	0.48576	0.95510														
291	3.69721	5.06637	4.28399	4.07704	2.36838	4.18181	3.79648	3.66935	3.92712	3.04510	4.31508	4.10292	4.65281	4.16038	4.04653	3.80070	3.98756	3.55483	4.02021	0.59046	319 Y - - -
	2.68618	4.42225	2.77519	2.73123	3.46354	2.40513	3.72494	3.29354	2.67741	2.69355	4.24690	2.90347	2.73739	3.18146	2.89801	2.37887	2.77519	2.98518	4.58477	3.61503	
	0.02334	4.16489	4.88723	0.61958	0.77255	0.48576	0.95510														
292	2.64522	5.05917	2.56140	2.35255	4.37500	3.40877	3.59951	3.83257	2.07174	3.34609	4.11296	2.53956	3.80885	2.71146	2.22491	2.62282	2.65870	3.43547	5.50341	3.74584	320 k - - -
	2.68618	4.42225	2.77519	2.73123	3.46354	2.40513	3.72494	3.29354	2.67741	2.69355	4.24690	2.90347	2.73739	3.18146	2.89801	2.37887	2.77519	2.98518	4.58477	3.61503	
	0.02334	4.16489	4.88723	0.61958	0.77255	0.48576	0.95510														
293	3.04661	4.51992	4.52316	4.20502	3.78297	4.01865	4.96252	2.08431	4.07785	2.42483	3.71244	4.34556	4.57000	4.42096	4.26104	3.54840	3.40546	0.72156	5.61364	4.35958	321 v - - -
	2.68618	4.42225	2.77519	2.73123	3.46354	2.40513	3.72494	3.29354	2.67741	2.69355	4.24690	2.90347	2.73739	3.18146	2.89801	2.37887	2.77519	2.98518	4.58477	3.61503	
	0.02334	4.16489	4.88723	0.61958	0.77255	0.48576	0.95510														
294	2.29362	4.66881	2.98908	2.68206	4.25357	3.29569	3.91243	3.66138	2.71343	3.28663	4.15198	3.11879	1.52282	2.62286	3.08762	2.70631	2.96475	3.28505	5.54071	4.24313	322 p - - -
	2.68618	4.42225	2.77519	2.73123	3.46354	2.40513	3.72494	3.29354	2.67741	2.69355	4.24690	2.90347	2.73739	3.18146	2.89801	2.37887	2.77519	2.98518	4.58477	3.61503	
	0.02334	4.16489	4.88723	0.61958	0.77255	0.48576	0.95510														
295	2.48486	4.74284	2.86372	2.62141	4.40270	1.44635	3.92597	3.84441	2.73774	3.44870	4.29461	3.07518	3.87965	2.26672	3.12576	2.71543	3.00477	3.42533	5.65186	4.33655	323 g - - -
	2.68618	4.42225	2.77519	2.73123	3.46354	2.40513	3.72494	3.29354	2.67741	2.69355	4.24690	2.90347	2.73739	3.18146	2.89801	2.37887	2.77519	2.98518	4.58477	3.61503	
	0.02334	4.16489	4.88723	0.61958	0.77255	0.48576	0.95510														
296	3.36714	5.16534	3.89059	3.35258	4.59608	3.78529	4.08066	4.16726	2.41459	3.63009	4.63119	3.68530	4.28143	3.29398	0.61106	3.44264	3.61809	3.88059	5.56480	4.46945	324 R - - -
	2.68618	4.42225	2.77519	2.73123	3.46354	2.40513	3.72494	3.29354	2.67741	2.69355	4.24690	2.90347	2.73739	3.18146	2.89801	2.37887	2.77519	2.98518	4.58477	3.61503	
	0.02334	4.16489	4.88723	0.61958	0.77255	0.48576	0.95510														
297	1.81105	4.69290	2.97320	2.14550	4.17709	3.31747	3.79197	3.57881	2.63046	3.20726	4.02968	3.03370	3.83316	2.96348	3.07479	2.18460	2.45176	3.21118	5.45447	4.13518	325 a - - -
	2.68618	4.42225	2.77519	2.73123	3.46354	2.40513	3.72494	3.29354	2.67741	2.69355	4.24690	2.90347	2.73739	3.18146	2.89801	2.37887	2.77519	2.98518	4.58477	3.61503	
	0.02334	4.16489	4.88723	0.61958	0.77255	0.48576	0.95510														
298	3.41116	4.75632	4.90616	4.35478	3.10785	4.61256	4.89905	2.28912	4.15884	0.97281	1.70138	4.62306	4.80057	4.30570	4.27933	3.97521	3.63465	2.44817	5.20473	4.12621	326 l - - -
	2.68618	4.42225	2.77519	2.73123	3.46354	2.40513	3.72494	3.29354	2.67741	2.69355	4.24690	2.90347	2.73739	3.18146	2.89801	2.37887	2.77519	2.98518	4.58477	3.61503	
	0.02334	4.16489	4.88723	0.61958	0.77255	0.48576	0.95510														
299	2.61211	4.93380	2.68658	2.38226	4.21340	3.08097	3.61225	3.65240	2.33996	3.09903	3.99812	2.91474	3.80631	2.37608	2.66713	2.33461	2.58667	2.94234	5.41308	4.04967	327 s - - -
	2.68618	4.42225	2.77519	2.73123	3.46354	2.40513	3.72494	3.29354	2.67741	2.69355	4.24690	2.90347	2.73739	3.18146	2.89801	2.37887	2.77519	2.98518	4.58477	3.61503	
	0.02334	4.16489	4.88723	0.61958	0.77255	0.48576	0.95510														
300	2.83799	4.39661	4.66694	4.08238	2.26070	4.21659	4.51219	2.03575	3.94599	1.52215	1.91054	4.25936	4.49218	4.07212	4.05210	3.53276	3.24814	2.30426	4.95185	3.81745	328 l - - -
	2.68618	4.42225	2.77519	2.73123	3.46354	2.40513	3.72494	3.29354	2.67741	2.69355	4.24690	2.90347	2.73739	3.18146	2.89801	2.37887	2.77519	2.98518	4.58477	3.61503	
	0.02334	4.16489	4.88723	0.61958	0.77255	0.48576	0.95510														
301	2.62296	4.98773	2.65117	2.35931	4.27806	3.40394	3.04681	3.72621	2.24726	3.26882	4.04634	2.68800	3.80387	2.46763	2.84495	2.56462	2.38390	2.95542	5.45431	4.07996	329 k - - -
	2.68618	4.42225	2.77519	2.73123	3.46354	2.40513	3.72494	3.29354	2.67741	2.69355	4.24690	2.90347	2.73739	3.18146	2.89801	2.37887	2.77519	2.98518	4.58477	3.61503	
	0.02334	4.16489	4.88723	0.61958	0.77255	0.48576	0.95510														
302	2.99272	4.35502	4.67891	4.09911	2.83052	4.20631	4.50991	1.79391	3.96203	1.32005	3.05533	4.25822	4.49298	4.09970	4.06722	3.52545	3.22109	1.83916	4.95890	3.43189	330 l - - -
	2.68618	4.42225	2.77519	2.73123	3.46354	2.40513	3.72494	3.29354	2.67741	2.69355	4.24690	2.90347	2.73739	3.18146	2.89801	2.37887	2.77519	2.98518	4.58477	3.61503	
	0.02334	4.16489	4.88723	0.61958	0.77255	0.48576	0.95510														
303	2.64411	4.96008	2.93382	2.21369	4.24653	3.42606	3.62020	3.41863	2.09321	3.23535	4.02591	2.92909	3.82466	2.36729	2.80287	2.44557	2.31559	3.31987	5.42892	4.07396	331 k - - -
	2.68618	4.42225	2.77519	2.73123	3.46354	2.40513	3.72494	3.29354	2.67741	2.69355	4.24690	2.90347	2.73739	3.18146	2.89801	2.37887	2.77519	2.98518	4.58477	3.61503	
	0.02334	4.16489	4.88723	0.61958	0.77255	0.48576	0.95510														
304	3.19826	4.48136	5.00775	4.52183	3.70083	4.60598	5.23077	1.32764	4.41797	2.18914	3.49056	4.71814	4.90464	4.67886	4.60467	4.00769	3.46531	0.99174	5.69863	4.48893	332 v - - -
	2.68618	4.42225	2.77519	2.73123	3.46354	2.40513	3.72494	3.29354	2.67741	2.69355	4.24690	2.90347	2.73739	3.18146	2.89801	2.37887	2.77519	2.98518	4.58477	3.61503	
	0.02334	4.16489	4.88723	0.61958	0.77255	0.48576	0.95510														
305	2.71034	4.97015	2.49566	2.41780	4.31382	3.39149	3.71347	3.72560	2.04182	3.31353	4.13101	2.94308	3.86412	2.86171	2.92916	2.71668	1.75498	3.36825	5.52266	4.16964	333 t - - -
	2.68618	4.42225	2.77519	2.73123	3.46354	2.40513	3.72494	3.29354	2.67741	2.69355	4.24690	2.90347	2.73739	3.18146	2.89801	2.37887	2.77519	2.98518	4.58477	3.61503	
	0.02334	4.16489	4.88723	0.61958	0.77255	0.48576	0.95510														
306	2.99769	4.98587	2.91719	2.83759	4.39907	3.40625	4.20177	4.18447	3.13691	3.80001	4.76139	0.72854	4.07683	3.48367	3.49425	3.05901	3.39442	3.77601	5.67109	4.33309	334 n - - -
	2.68618	4.42225	2.77519	2.73123	3.46354	2.40513	3.72494	3.29354	2.67741	2.69355	4.24690	2.90347	2.73739	3.18146	2.89801	2.37887	2.77519	2.98518	4.58477	3.61503	
	0.02334	4.16489	4.88723	0.61958	0.77255	0.48576	0.95510														
307	2.61903	4.88823	2.97123	2.41797	4.15653	2.89986	3.53053	3.38216	2.15885	3.15994	3.95825	2.40800	3.82017	2.75943	2.61452	2.52348	2.75307	2.92806	5.37496	4.02762	335 k - - -
	2.68618	4.42225	2.77519	2.73123	3.46354	2.40513	3.72494	3.29354	2.67741	2.69355	4.24690	2.90347	2.73739	3.18146	2.89801	2.37887	2.77519	2.98518	4.58477	3.61503	
	0.02334	4.16489	4.88723	0.61958	0.77255	0.48576	0.95510														
308	2.31574	4.28064	3.44754	3.16724	4.45344	1.35210	4.28561	3.88095	3.25779	3.55204	4.36417	3.35393	3.77655	3.54013	3.59033	1.88459	2.18555	3.30815	5.77633	4.55676	336 g - - -
	2.68618	4.42225	2.77519	2.73123	3.46354	2.40513	3.72494	3.29354	2.67741	2.69355	4.24690	2.90347	2.73739	3.18146	2.89801	2.37887	2.77519	2.98518	4.58477	3.61503	

```
      0.02334  4.16489  4.88723  0.61958  0.77255  0.48576  0.95510
309   2.76648  5.25189  1.75423  2.25992  4.56648  3.35279  3.68561  4.04895  2.41956  3.55034  4.33161  2.38829  3.84272  2.51358  3.03312  2.40977  2.81470  3.63239  5.70769  4.27897    337 d - - -
      2.68618  4.42225  2.77519  2.73123  3.46354  2.40513  3.72494  3.29354  2.67741  2.69355  4.24690  2.90347  2.73739  3.18146  2.89801  2.37887  2.77519  2.98518  4.58477  3.61503
      0.02334  4.16489  4.88723  0.61958  0.77255  0.48576  0.95510
310   2.65588  5.11249  2.68355  2.08225  4.43815  3.00679  3.60293  3.90721  2.05437  3.40514  4.16428  2.86785  3.80549  2.57235  2.55074  2.24248  2.88833  3.49390  5.55149  4.15598    338 k - - -
      2.68618  4.42225  2.77519  2.73123  3.46354  2.40513  3.72494  3.29354  2.67741  2.69355  4.24690  2.90347  2.73739  3.18146  2.89801  2.37887  2.77519  2.98518  4.58477  3.61503
      0.02334  4.16489  4.88723  0.61958  0.77255  0.48576  0.95510
311   2.02817  4.63384  3.06388  2.65083  4.19700  3.29947  3.84294  3.60084  2.35370  3.22896  4.05800  3.09415  1.93116  3.02519  3.04762  2.43832  2.88741  3.22134  5.47101  4.17440    339 p - - -
      2.68618  4.42225  2.77519  2.73123  3.46354  2.40513  3.72494  3.29354  2.67741  2.69355  4.24690  2.90347  2.73739  3.18146  2.89801  2.37887  2.77519  2.98518  4.58477  3.61503
      0.02334  4.16489  4.88723  0.61958  0.77255  0.48576  0.95510
312   2.85646  4.36116  4.09240  3.53934  3.40987  3.95992  4.28734  2.12480  3.38529  2.02782  3.32601  3.84971  4.31722  3.10433  3.63814  3.26384  3.09906  1.26893  5.08093  3.87081    340 v - - -
      2.68618  4.42225  2.77519  2.73123  3.46354  2.40513  3.72494  3.29354  2.67741  2.69355  4.24690  2.90347  2.73739  3.18146  2.89801  2.37887  2.77519  2.98518  4.58477  3.61503
      0.02334  4.16489  4.88723  0.61958  0.77255  0.48576  0.95510
313   2.72759  4.80224  3.21349  2.62314  4.02492  3.54255  3.67543  3.42966  2.26013  3.03538  3.88303  3.09952  3.92166  2.53785  1.78948  2.78692  2.94428  2.67955  5.26483  3.29355    341 r - - -
      2.68618  4.42225  2.77519  2.73123  3.46354  2.40513  3.72494  3.29354  2.67741  2.69355  4.24690  2.90347  2.73739  3.18146  2.89801  2.37887  2.77519  2.98518  4.58477  3.61503
      0.02334  4.16489  4.88723  0.61958  0.77255  0.48576  0.95510
314   3.39872  4.71161  5.00308  4.46102  3.20471  4.67853  5.02925  1.90056  4.29986  0.81810  2.99667  4.71618  4.86695  4.43407  4.42320  4.04914  3.63132  2.08129  5.31807  4.23077    342 l - - -
      2.68618  4.42225  2.77519  2.73123  3.46354  2.40513  3.72494  3.29354  2.67741  2.69355  4.24690  2.90347  2.73739  3.18146  2.89801  2.37887  2.77519  2.98518  4.58477  3.61503
      0.02334  4.16489  4.88723  0.61958  0.77255  0.48576  0.95510
315   2.53429  4.24186  3.14669  2.62938  4.14148  1.81805  3.75211  3.53972  2.08122  3.15224  3.98240  3.08914  3.86837  2.92105  2.67698  2.68992  2.89872  3.19512  5.38408  4.09439    343 g - - -
      2.68618  4.42225  2.77519  2.73123  3.46354  2.40513  3.72494  3.29354  2.67741  2.69355  4.24690  2.90347  2.73739  3.18146  2.89801  2.37887  2.77519  2.98518  4.58477  3.61503
      0.02334  4.16489  4.88723  0.61958  0.77255  0.48576  0.95510
316   2.79717  5.19410  2.68546  1.44295  4.52263  2.95370  3.68400  3.97600  2.40433  3.48464  4.28402  2.89520  3.87855  2.60396  2.51805  2.75756  3.03675  3.58686  5.63535  4.25990    344 e - - -
      2.68618  4.42225  2.77519  2.73123  3.46354  2.40513  3.72494  3.29354  2.67741  2.69355  4.24690  2.90347  2.73739  3.18146  2.89801  2.37887  2.77519  2.98518  4.58477  3.61503
      0.02334  4.16489  4.88723  0.61958  0.77255  0.48576  0.95510
317   3.78144  4.99131  4.78462  4.49912  0.82614  4.51959  3.58211  3.40055  4.33532  2.69864  3.99470  4.21009  4.82567  4.27602  4.34054  3.90265  4.00493  3.34959  3.71105  1.63696    345 f - - -
      2.68618  4.42225  2.77519  2.73123  3.46354  2.40513  3.72494  3.29354  2.67741  2.69355  4.24690  2.90347  2.73739  3.18146  2.89801  2.37887  2.77519  2.98518  4.58477  3.61503
      0.02334  4.16489  4.88723  0.61958  0.77255  0.48576  0.95510
318   2.51810  4.48177  3.25667  2.69465  3.64730  3.51388  3.74559  2.77428  2.66396  2.43018  3.36282  2.66107  3.89828  2.72313  3.06946  2.52668  2.51247  2.77105  5.04011  3.33887    346 l - - -
      2.68618  4.42225  2.77519  2.73123  3.46354  2.40513  3.72494  3.29354  2.67741  2.69355  4.24690  2.90347  2.73739  3.18146  2.89801  2.37887  2.77519  2.98518  4.58477  3.61503
      0.02334  4.16489  4.88723  0.61958  0.77255  0.48576  0.95510
319   1.90898  4.23690  3.62956  3.29418  4.26600  3.07041  4.30318  3.55678  3.28675  3.33625  4.19360  3.43220  3.80358  3.59051  3.58465  2.26351  1.17576  3.09441  5.64363  4.43887    347 t - - -
      2.68618  4.42225  2.77519  2.73123  3.46354  2.40513  3.72494  3.29354  2.67741  2.69355  4.24690  2.90347  2.73739  3.18146  2.89801  2.37887  2.77519  2.98518  4.58477  3.61503
      0.02334  4.16489  4.88723  0.61958  0.77255  0.48576  0.95510
320   0.99289  4.32607  3.18539  3.08666  4.32385  3.08797  4.23651  3.68077  3.22247  3.41678  4.26495  3.32896  3.81197  3.50194  3.56379  2.17641  2.84238  3.20158  5.68189  4.44115    348 a - - -
      2.68618  4.42225  2.77519  2.73123  3.46354  2.40513  3.72494  3.29354  2.67741  2.69355  4.24690  2.90347  2.73739  3.18146  2.89801  2.37887  2.77519  2.98518  4.58477  3.61503
      0.02334  4.16489  4.88723  0.61958  0.77255  0.48576  0.95510
321   2.53000  4.65385  2.89964  2.61510  4.45218  2.22753  3.93195  3.90614  2.80734  3.49925  4.30412  1.74361  3.81784  3.10802  3.25096  2.08027  2.72328  3.42630  5.69820  4.37103    349 n - - -
      2.68618  4.42225  2.77519  2.73123  3.46354  2.40513  3.72494  3.29354  2.67741  2.69355  4.24690  2.90347  2.73739  3.18146  2.89801  2.37887  2.77519  2.98518  4.58477  3.61503
      0.02334  4.16489  4.88723  0.61958  0.77255  0.48576  0.95510
322   2.95166  4.38658  4.44772  3.94339  3.48847  3.86229  4.61055  2.03891  3.79318  1.68357  3.39058  4.16252  4.49574  4.08296  3.99692  3.46446  3.22696  1.15539  5.26229  4.04159    350 v - - -
      2.68618  4.42225  2.77519  2.73123  3.46354  2.40513  3.72494  3.29354  2.67741  2.69355  4.24690  2.90347  2.73739  3.18146  2.89801  2.37887  2.77519  2.98518  4.58477  3.61503
      0.02334  4.16489  4.88723  0.61958  0.77255  0.48576  0.95510
323   2.52261  5.04165  3.16968  2.59861  4.37430  3.55684  3.05913  3.78312  2.16646  3.30667  4.13174  3.07803  3.94630  2.52530  1.55283  2.77765  3.03872  3.44553  5.44613  4.16870    351 r - - -
      2.68618  4.42225  2.77519  2.73123  3.46354  2.40513  3.72494  3.29354  2.67741  2.69355  4.24690  2.90347  2.73739  3.18146  2.89801  2.37887  2.77519  2.98518  4.58477  3.61503
      0.02334  4.16489  4.88723  0.61958  0.77255  0.48576  0.95510
324   3.65633  4.97487  4.64261  4.42152  0.63212  4.25844  4.04309  3.15482  4.32396  2.46101  3.84666  4.36241  4.71705  4.41299  4.37075  3.90466  3.96096  3.16555  4.22202  2.55920    352 F - - -
      2.68618  4.42225  2.77519  2.73123  3.46354  2.40513  3.72494  3.29354  2.67741  2.69355  4.24690  2.90347  2.73739  3.18146  2.89801  2.37887  2.77519  2.98518  4.58477  3.61503
      0.02334  4.16489  4.88723  0.61958  0.77255  0.48576  0.95510
325   2.95841  4.48277  4.15023  3.56598  3.23233  4.01353  4.25919  2.49798  3.28488  1.12819  2.54531  3.87976  4.33650  3.65502  2.96503  3.31731  3.18610  2.49861  4.99998  3.83241    353 l - - -
      2.68618  4.42225  2.77519  2.73123  3.46354  2.40513  3.72494  3.29354  2.67741  2.69355  4.24690  2.90347  2.73739  3.18146  2.89801  2.37887  2.77519  2.98518  4.58477  3.61503
      0.02334  4.16489  4.88723  0.61958  0.77255  0.48576  0.95510
326   2.99432  5.55373  1.58839  2.13600  4.87003  3.17249  3.83822  4.39686  2.84449  3.88969  4.72961  1.48888  3.91610  3.00122  3.43659  2.88591  3.28519  3.95890  6.04536  4.53970    354 n - - -
      2.68618  4.42225  2.77519  2.73123  3.46354  2.40513  3.72494  3.29354  2.67741  2.69355  4.24690  2.90347  2.73739  3.18146  2.89801  2.37887  2.77519  2.98518  4.58477  3.61503
      0.02334  4.16489  4.88723  0.61958  0.77255  0.48576  0.95510
327   1.81355  4.31054  3.41362  3.11756  4.36342  3.07171  4.23281  3.75748  3.18234  3.45029  4.28429  3.33901  1.40980  3.48676  3.51698  2.21540  2.82069  3.24564  5.70022  4.46921    355 p - - -
      2.68618  4.42225  2.77519  2.73123  3.46354  2.40513  3.72494  3.29354  2.67741  2.69355  4.24690  2.90347  2.73739  3.18146  2.89801  2.37887  2.77519  2.98518  4.58477  3.61503
      0.02334  4.16489  4.88723  0.61958  0.77255  0.48576  0.95510
328   2.23093  5.15838  1.68377  2.23607  4.49038  3.34574  3.71267  3.95493  2.52382  3.48989  4.28629  2.67819  3.84821  2.85084  3.08272  2.55797  2.76964  3.55716  5.67352  4.26174    356 d - - -
      2.68618  4.42225  2.77519  2.73123  3.46354  2.40513  3.72494  3.29354  2.67741  2.69355  4.24690  2.90347  2.73739  3.18146  2.89801  2.37887  2.77519  2.98518  4.58477  3.61503
      0.02334  4.16489  4.88723  0.61958  0.77255  0.48576  0.95510
329   2.59652  4.37862  4.64340  4.10183  3.53931  4.29961  4.75676  1.74332  3.99579  1.81604  3.38371  4.32681  4.62194  4.23872  4.19444  3.64062  3.27132  1.17570  5.35137  4.16369    357 v - - -
      2.68618  4.42225  2.77519  2.73123  3.46354  2.40513  3.72494  3.29354  2.67741  2.69355  4.24690  2.90347  2.73739  3.18146  2.89801  2.37887  2.77519  2.98518  4.58477  3.61503
      0.07803  4.16489  2.82128  0.61958  0.77255  0.48576  0.95510
330   2.38921  4.00189  3.51256  2.94548  2.84255  3.57118  3.34192  2.76272  2.89151  2.38005  3.37701  3.33297  2.83058  3.18809  3.22934  2.72952  2.81411  2.54249  4.82833  2.93437    358 l - - -
      2.68618  4.42225  2.77519  2.73123  3.46354  2.40513  3.72494  3.29354  2.67741  2.69355  4.24690  2.90347  2.73739  3.18146  2.89801  2.37887  2.77519  2.98518  4.58477  3.61503
      0.02464  4.11150  4.83384  0.61958  0.77255  0.54742  0.86379
```

331	2.56062	5.04896	2.65796	2.06001	4.35346	2.86952	3.60444	3.81375	2.24430	3.33862	4.11009	2.63967	3.79285	2.64884	2.86643	2.54304	2.45086	3.34317	5.51009	4.11868	359 e - - -
	2.68618	4.42225	2.77519	2.73123	3.46354	2.40513	3.72494	3.29354	2.67741	2.69355	4.24690	2.90347	2.73739	3.18146	2.89801	2.37887	2.77519	2.98518	4.58477	3.61503	
	0.02464	4.11150	4.83384	0.61958	0.77255	0.54742	0.86379														
332	2.66968	5.04890	2.10088	2.31132	4.35237	3.36186	3.64122	3.80417	2.44260	3.34804	4.13564	2.28995	3.81076	2.50728	2.93405	2.64813	2.67426	2.97428	5.53404	4.14414	360 d - - -
	2.68618	4.42225	2.77519	2.73123	3.46354	2.40513	3.72494	3.29354	2.67741	2.69355	4.24690	2.90347	2.73739	3.18146	2.89801	2.37887	2.77519	2.98518	4.58477	3.61503	
	0.02464	4.11150	4.83384	0.61958	0.77255	0.54742	0.86379														
333	2.66859	5.13788	2.45802	2.10313	4.45625	3.20188	3.61016	3.93039	2.08538	3.42991	4.19385	2.55728	3.79797	2.48632	2.87343	2.62835	2.45764	3.51568	5.58068	4.17304	361 k - - -
	2.68615	4.42230	2.77524	2.73128	3.46359	2.40518	3.72499	3.29359	2.67746	2.69360	4.24695	2.90325	2.73737	3.18151	2.89806	2.37892	2.77524	2.98523	4.58237	3.61508	
	0.18832	1.80975	4.83384	0.66562	0.72146	0.54742	0.86379														
334	2.43294	4.64510	3.04592	2.44612	4.03297	3.34532	3.72900	3.43934	2.56087	3.06760	3.89310	2.89807	2.69595	2.90332	3.00251	2.22091	2.26281	2.94394	5.32730	4.01670	364 s - - -
	2.68618	4.42225	2.77519	2.73123	3.46354	2.40513	3.72494	3.29354	2.67741	2.69355	4.24690	2.90347	2.73739	3.18146	2.89801	2.37887	2.77519	2.98518	4.58477	3.61503	
	0.02464	4.11150	4.83384	0.61958	0.77255	0.54742	0.86379														
335	2.67184	5.10290	2.20986	2.19134	4.41332	2.54719	3.62869	3.87987	2.29982	3.39988	4.17511	2.59260	3.80313	2.75019	2.92570	2.48015	2.78322	3.29419	5.56911	4.16597	365 e - - -
	2.68618	4.42225	2.77519	2.73123	3.46354	2.40513	3.72494	3.29354	2.67741	2.69355	4.24690	2.90347	2.73739	3.18146	2.89801	2.37887	2.77519	2.98518	4.58477	3.61503	
	0.02464	4.11150	4.83384	0.61958	0.77255	0.44905	1.01676														
336	2.78257	4.36350	3.82816	3.30680	1.98467	3.75420	3.78966	2.86603	3.23329	2.53547	3.49553	3.57704	3.46956	3.48250	3.50111	2.53573	3.02434	2.66511	4.37097	1.91300	366 y - - -
	2.68618	4.42225	2.77519	2.73123	3.46354	2.40513	3.72494	3.29354	2.67741	2.69355	4.24690	2.90347	2.73739	3.18146	2.89801	2.37887	2.77519	2.98518	4.58477	3.61503	
	0.02334	4.16489	4.88723	0.61958	0.77255	0.48576	0.95510														
337	2.66453	4.56919	3.40174	3.24140	4.60581	2.40736	4.42860	4.12193	3.40658	3.76559	4.64762	3.51668	0.83785	3.72363	3.70871	2.82122	3.13902	3.59824	5.80480	4.68224	367 p - - -
	2.68618	4.42225	2.77519	2.73123	3.46354	2.40513	3.72494	3.29354	2.67741	2.69355	4.24690	2.90347	2.73739	3.18146	2.89801	2.37887	2.77519	2.98518	4.58477	3.61503	
	0.02334	4.16489	4.88723	0.61958	0.77255	0.48576	0.95510														
338	2.95717	5.54735	1.52290	1.52211	4.84057	3.31907	3.78808	4.35462	2.37701	3.83149	4.64958	2.77073	3.90072	2.93669	3.29238	2.85019	3.22833	3.91783	5.97928	4.48837	368 e - - -
	2.68618	4.42225	2.77519	2.73123	3.46354	2.40513	3.72494	3.29354	2.67741	2.69355	4.24690	2.90347	2.73739	3.18146	2.89801	2.37887	2.77519	2.98518	4.58477	3.61503	
	0.17761	4.16489	1.91599	0.61958	0.77255	0.48576	0.95510														
339	2.70767	4.93219	1.78204	2.34256	3.96986	3.33864	3.68422	3.63542	2.59397	3.22916	4.07721	2.87760	3.83643	2.88091	3.07840	2.43562	2.96906	3.30221	5.31886	2.74808	369 d - - -
	2.68618	4.42225	2.77519	2.73123	3.46354	2.40513	3.72494	3.29354	2.67741	2.69355	4.24690	2.90347	2.73739	3.18146	2.89801	2.37887	2.77519	2.98518	4.58477	3.61503	
	0.02719	4.01447	4.73681	0.61958	0.77255	0.64348	0.74541														
340	2.63108	4.49795	2.84175	2.71688	3.60808	3.52135	3.76197	2.79141	2.63867	1.83883	3.55029	3.17288	3.91758	3.01239	2.71746	2.78112	2.86641	2.70779	5.04390	3.76977	370 l - - -
	2.68618	4.42225	2.77519	2.73123	3.46354	2.40513	3.72494	3.29354	2.67741	2.69355	4.24690	2.90347	2.73739	3.18146	2.89801	2.37887	2.77519	2.98518	4.58477	3.61503	
	0.02719	4.01447	4.73681	0.61958	0.77255	0.39844	1.11282														
341	3.23958	4.57657	4.85251	4.30356	3.28696	4.50963	4.89316	2.06166	4.15832	1.09903	2.91127	4.54206	4.75391	4.33421	4.30726	3.86277	3.47819	1.49218	5.29843	4.18324	371 l - - -
	2.68618	4.42225	2.77519	2.73123	3.46354	2.40513	3.72494	3.29354	2.67741	2.69355	4.24690	2.90347	2.73739	3.18146	2.89801	2.37887	2.77519	2.98518	4.58477	3.61503	
	0.02334	4.16489	4.88723	0.61958	0.77255	0.48576	0.95510														
342	1.71415	4.78043	2.90976	2.52036	4.25719	3.33304	3.77458	3.67921	2.34412	3.27844	4.09612	2.28076	3.84535	2.93736	3.00433	2.38971	2.91610	3.30199	5.50012	4.17220	372 a - - -
	2.68618	4.42225	2.77519	2.73123	3.46354	2.40513	3.72494	3.29354	2.67741	2.69355	4.24690	2.90347	2.73739	3.18146	2.89801	2.37887	2.77519	2.98518	4.58477	3.61503	
	0.07803	4.16489	2.82128	0.61958	0.77255	0.48576	0.95510														
343	2.90163	5.46990	1.79737	1.69304	4.77375	2.27398	3.75835	4.28616	2.70892	3.77104	4.58390	2.39025	3.86602	2.90620	3.28320	2.80350	3.17455	3.85054	5.92726	4.43994	373 e - - -
	2.68618	4.42225	2.77519	2.73123	3.46354	2.40513	3.72494	3.29354	2.67741	2.69355	4.24690	2.90347	2.73739	3.18146	2.89801	2.37887	2.77519	2.98518	4.58477	3.61503	
	0.02464	4.11150	4.83384	0.61958	0.77255	0.54742	0.86379														
344	2.69659	5.15931	2.46673	1.94188	4.47674	2.53458	3.62670	3.94893	2.33220	3.45031	4.22181	2.64460	3.81063	2.74491	2.41044	2.65366	2.93588	3.53796	5.60274	4.19605	374 e - - -
	2.68618	4.42225	2.77519	2.73123	3.46354	2.40513	3.72494	3.29354	2.67741	2.69355	4.24690	2.90347	2.73739	3.18146	2.89801	2.37887	2.77519	2.98518	4.58477	3.61503	
	0.02464	4.11150	4.83384	0.61958	0.77255	0.54742	0.86379														
345	2.47484	4.78137	2.72036	2.19450	4.36368	1.61395	3.84607	3.79093	2.72435	3.39681	4.22636	2.97118	3.04793	3.02496	3.17679	2.67619	2.96402	3.38532	5.62067	4.28118	375 g - - -
	2.68618	4.42225	2.77519	2.73123	3.46354	2.40513	3.72494	3.29354	2.67741	2.69355	4.24690	2.90347	2.73739	3.18146	2.89801	2.37887	2.77519	2.98518	4.58477	3.61503	
	0.02464	4.11150	4.83384	0.61958	0.77255	0.44905	1.01676														
346	3.39551	4.69922	5.03687	4.46688	3.15638	4.68758	5.00134	1.96706	4.33602	0.85379	2.51339	4.71972	4.84444	4.41042	4.43644	4.03782	3.61408	2.15321	5.26723	4.23298	376 l - - -
	2.68618	4.42225	2.77519	2.73123	3.46354	2.40513	3.72494	3.29354	2.67741	2.69355	4.24690	2.90347	2.73739	3.18146	2.89801	2.37887	2.77519	2.98518	4.58477	3.61503	
	0.06167	4.16489	3.11731	0.61958	0.77255	0.48576	0.95510														
347	2.63348	4.90161	2.94701	2.30787	4.17766	3.42268	3.62342	3.37462	2.09544	3.17509	3.97640	2.93893	3.82293	2.53279	2.80877	2.17330	2.43787	3.25660	5.38509	4.04138	377 k - - -
	2.68618	4.42225	2.77519	2.73123	3.46354	2.40513	3.72494	3.29354	2.67741	2.69355	4.24690	2.90347	2.73739	3.18146	2.89801	2.37887	2.77519	2.98518	4.58477	3.61503	
	0.07522	4.12746	2.87641	0.61958	0.77255	0.52972	0.88860														
348	3.14518	4.45420	4.83341	4.30724	3.49441	4.50104	4.97819	1.64254	4.19181	1.72710	3.30260	4.53990	4.77356	4.42583	4.37897	3.86668	3.39598	1.09004	5.46112	4.28151	378 v - - -
	2.68618	4.42225	2.77519	2.73123	3.46354	2.40513	3.72494	3.29354	2.67741	2.69355	4.24690	2.90347	2.73739	3.18146	2.89801	2.37887	2.77519	2.98518	4.58477	3.61503	
	0.02550	4.07774	4.80008	0.61958	0.77255	0.42944	1.05231														
349	2.65327	4.94814	2.63720	2.22642	4.25182	3.38929	3.66370	3.68634	2.37708	3.25754	4.05682	2.91484	3.82816	2.80206	2.93856	1.84299	2.44638	3.20744	5.46786	4.10387	379 s - - -
	2.68618	4.42225	2.77519	2.73123	3.46354	2.40513	3.72494	3.29354	2.67741	2.69355	4.24690	2.90347	2.73739	3.18146	2.89801	2.37887	2.77519	2.98518	4.58477	3.61503	
	0.02334	4.16489	4.88723	0.61958	0.77255	0.48576	0.95510														
350	2.87236	5.32707	1.56817	2.24497	4.69735	3.30850	3.80051	4.20229	2.73882	3.71860	4.53671	2.35004	2.15232	2.95472	3.28907	2.80597	3.15966	3.77444	5.87999	4.42360	380 d - - -
	2.68618	4.42225	2.77519	2.73123	3.46354	2.40513	3.72494	3.29354	2.67741	2.69355	4.24690	2.90347	2.73739	3.18146	2.89801	2.37887	2.77519	2.98518	4.58477	3.61503	
	0.02334	4.16489	4.88723	0.61958	0.77255	0.48576	0.95510														
351	2.37560	5.01357	2.41510	2.35292	4.44293	3.32462	3.72512	3.90434	2.56313	3.44873	4.23990	1.87643	3.39815	2.86359	3.06177	2.20812	2.95721	3.49264	5.63376	4.24262	381 n - - -
	2.68618	4.42225	2.77519	2.73123	3.46354	2.40513	3.72494	3.29354	2.67741	2.69355	4.24690	2.90347	2.73739	3.18146	2.89801	2.37887	2.77519	2.98518	4.58477	3.61503	
	0.02334	4.16489	4.88723	0.61958	0.77255	0.48576	0.95510														
352	2.48852	5.04111	2.50582	1.96080	4.38435	2.97606	3.66569	3.84175	2.47274	3.38057	4.16377	2.87722	3.82008	2.79398	2.96849	2.10984	2.51819	3.44711	5.56390	4.17345	382 e - - -
	2.68618	4.42225	2.77519	2.73123	3.46354	2.40513	3.72494	3.29354	2.67741	2.69355	4.24690	2.90347	2.73739	3.18146	2.89801	2.37887	2.77519	2.98518	4.58477	3.61503	
	0.02334	4.16489	4.88723	0.61958	0.77255	0.48576	0.95510														
353	2.56664	4.44026	3.56593	3.28594	4.08668	3.27085	4.31300	3.35221	3.24458	3.10181	4.14249	3.51643	1.03916	3.62187	3.52636	2.75602	3.01169	2.62179	5.54475	4.28320	383 p - - -

	2.68618	4.42225	2.77519	2.73123	3.46354	2.40513	3.72494	3.29354	2.67741	2.69355	4.24690	2.90347	2.73739	3.18146	2.89801	2.37887	2.77519	2.98518	4.58477	3.61503	
	0.02334	4.16489	4.88723	0.61958	0.77255	0.48576	0.95510														
354	3.32041	4.61053	5.00153	4.49063	3.37222	4.65885	5.08196	1.06414	4.32636	1.45701	3.18895	4.71755	4.88909	4.52930	4.46849	4.04957	3.56569	1.89205	5.42977	4.26753	384 i - - -
	2.68618	4.42225	2.77519	2.73123	3.46354	2.40513	3.72494	3.29354	2.67741	2.69355	4.24690	2.90347	2.73739	3.18146	2.89801	2.37887	2.77519	2.98518	4.58477	3.61503	
	0.02334	4.16489	4.88723	0.61958	0.77255	0.48576	0.95510														
355	1.87202	4.77859	2.97403	2.41643	4.03766	3.42945	3.68466	3.44467	2.49142	2.75276	3.89014	2.61294	3.31701	2.68022	2.93455	2.67910	2.87436	3.12946	5.31825	3.99599	385 a - - -
	2.68618	4.42225	2.77519	2.73123	3.46354	2.40513	3.72494	3.29354	2.67741	2.69355	4.24690	2.90347	2.73739	3.18146	2.89801	2.37887	2.77519	2.98518	4.58477	3.61503	
	0.02334	4.16489	4.88723	0.61958	0.77255	0.48576	0.95510														
356	2.28464	4.45408	3.41437	3.23484	4.40453	3.18481	4.37284	3.76357	3.31465	3.48268	4.43338	3.46740	0.92509	3.66549	3.59994	2.71105	3.01858	3.32214	5.74532	4.53714	386 p - - -
	2.68618	4.42225	2.77519	2.73123	3.46354	2.40513	3.72494	3.29354	2.67741	2.69355	4.24690	2.90347	2.73739	3.18146	2.89801	2.37887	2.77519	2.98518	4.58477	3.61503	
	0.02334	4.16489	4.88723	0.61958	0.77255	0.48576	0.95510														
357	3.07087	4.80683	3.80366	3.74507	4.86220	0.39323	4.83738	4.54296	3.96555	4.15653	5.14181	3.96927	4.20642	4.26711	4.15990	3.25273	3.57468	4.01458	5.86989	4.97743	387 G - - -
	2.68618	4.42225	2.77519	2.73123	3.46354	2.40513	3.72494	3.29354	2.67741	2.69355	4.24690	2.90347	2.73739	3.18146	2.89801	2.37887	2.77519	2.98518	4.58477	3.61503	
	0.02334	4.16489	4.88723	0.61958	0.77255	0.48576	0.95510														
358	2.91846	5.33400	2.48939	1.21994	4.64591	3.38046	3.76761	4.10994	2.55842	3.62271	4.46286	2.86649	3.92128	2.35843	3.00166	2.85511	3.08148	3.72548	5.78512	4.37944	388 e - - -
	2.68618	4.42225	2.77519	2.73123	3.46354	2.40513	3.72494	3.29354	2.67741	2.69355	4.24690	2.90347	2.73739	3.18146	2.89801	2.37887	2.77519	2.98518	4.58477	3.61503	
	0.02334	4.16489	4.88723	0.61958	0.77255	0.48576	0.95510														
359	2.54860	4.56785	3.12125	2.73348	4.14292	3.28769	3.89932	3.54102	2.72012	3.19125	4.03918	3.14875	3.85756	2.65074	3.09837	2.17483	1.51342	3.17146	5.45013	4.16622	389 t - - -
	2.68618	4.42225	2.77519	2.73123	3.46354	2.40513	3.72494	3.29354	2.67741	2.69355	4.24690	2.90347	2.73739	3.18146	2.89801	2.37887	2.77519	2.98518	4.58477	3.61503	
	0.02334	4.16489	4.88723	0.61958	0.77255	0.48576	0.95510														
360	2.96757	5.11481	3.42938	2.76183	4.50455	3.66714	3.66520	3.85184	1.43370	3.35490	4.20806	3.19539	4.03329	2.80023	1.64401	2.97700	3.15387	2.98439	5.46709	4.25258	390 k - - -
	2.68618	4.42225	2.77519	2.73123	3.46354	2.40513	3.72494	3.29354	2.67741	2.69355	4.24690	2.90347	2.73739	3.18146	2.89801	2.37887	2.77519	2.98518	4.58477	3.61503	
	0.02334	4.16489	4.88723	0.61958	0.77255	0.48576	0.95510														
361	2.74721	4.89722	2.79081	2.13196	4.23925	3.39952	3.78002	3.62526	2.56667	3.24778	4.10323	2.99728	3.90383	2.75820	2.97396	2.78008	1.53290	3.29846	5.49755	4.17107	391 t - - -
	2.68618	4.42225	2.77519	2.73123	3.46354	2.40513	3.72494	3.29354	2.67741	2.69355	4.24690	2.90347	2.73739	3.18146	2.89801	2.37887	2.77519	2.98518	4.58477	3.61503	
	0.02334	4.16489	4.88723	0.61958	0.77255	0.48576	0.95510														
362	3.05798	4.39407	4.77925	4.20929	3.28665	4.33008	4.69262	1.62105	4.08137	1.53480	2.71466	4.38596	4.60350	4.23545	4.20069	3.66035	3.29051	1.52172	5.14517	3.80092	392 v - - -
	2.68618	4.42225	2.77519	2.73123	3.46354	2.40513	3.72494	3.29354	2.67741	2.69355	4.24690	2.90347	2.73739	3.18146	2.89801	2.37887	2.77519	2.98518	4.58477	3.61503	
	0.02334	4.16489	4.88723	0.61958	0.77255	0.48576	0.95510														
363	2.58300	5.04239	2.64707	1.95585	4.34561	3.39184	3.15991	3.80480	2.36949	3.32998	4.09835	2.83185	3.79997	2.53611	2.85627	2.41390	2.54962	3.15803	5.50094	4.11259	393 e - - -
	2.68618	4.42225	2.77519	2.73123	3.46354	2.40513	3.72494	3.29354	2.67741	2.69355	4.24690	2.90347	2.73739	3.18146	2.89801	2.37887	2.77519	2.98518	4.58477	3.61503	
	0.02334	4.16489	4.88723	0.61958	0.77255	0.48576	0.95510														
364	3.17884	4.48184	4.94283	4.46176	3.69403	4.54650	5.18049	1.51136	4.34987	2.19473	3.49463	4.66155	4.86776	4.62251	4.54387	3.94945	3.45143	0.89536	5.67782	4.46006	394 v - - -
	2.68618	4.42225	2.77519	2.73123	3.46354	2.40513	3.72494	3.29354	2.67741	2.69355	4.24690	2.90347	2.73739	3.18146	2.89801	2.37887	2.77519	2.98518	4.58477	3.61503	
	0.02334	4.16489	4.88723	0.61958	0.77255	0.48576	0.95510														
365	2.67650	5.07641	2.54681	2.16820	4.38676	3.38817	3.63666	3.84296	1.97369	3.37076	4.14944	2.87686	3.82139	2.75861	2.88612	2.36132	2.24208	3.45214	5.54344	4.15580	395 k - - -
	2.68618	4.42225	2.77519	2.73123	3.46354	2.40513	3.72494	3.29354	2.67741	2.69355	4.24690	2.90347	2.73739	3.18146	2.89801	2.37887	2.77519	2.98518	4.58477	3.61503	
	0.02334	4.16489	4.88723	0.61958	0.77255	0.48576	0.95510														
366	1.76855	4.29108	4.11268	3.55469	3.42659	3.89336	4.28724	2.04736	3.46438	2.23673	2.71658	3.83953	4.27376	3.72869	3.71892	3.19809	3.03950	1.76440	5.06562	3.86963	396 v - - -
	2.68618	4.42225	2.77519	2.73123	3.46354	2.40513	3.72494	3.29354	2.67741	2.69355	4.24690	2.90347	2.73739	3.18146	2.89801	2.37887	2.77519	2.98518	4.58477	3.61503	
	0.02334	4.16489	4.88723	0.61958	0.77255	0.48576	0.95510														
367	2.68014	4.91694	2.99959	2.49349	4.28144	3.43096	3.67373	3.70482	2.35332	3.26395	4.07206	2.99629	3.86271	1.96529	2.48539	2.08212	2.65276	3.34454	5.45198	4.12750	397 q - - -
	2.68618	4.42225	2.77519	2.73123	3.46354	2.40513	3.72494	3.29354	2.67741	2.69355	4.24690	2.90347	2.73739	3.18146	2.89801	2.37887	2.77519	2.98518	4.58477	3.61503	
	0.02334	4.16489	4.88723	0.61958	0.77255	0.48576	0.95510														
368	2.84401	5.20792	1.21816	2.28815	4.75132	2.67706	3.88171	4.25614	2.87614	3.79185	4.62240	2.84241	3.88956	3.05164	3.43633	2.28138	3.17620	3.79462	5.96033	4.50864	398 d - - -
	2.68618	4.42225	2.77519	2.73123	3.46354	2.40513	3.72494	3.29354	2.67741	2.69355	4.24690	2.90347	2.73739	3.18146	2.89801	2.37887	2.77519	2.98518	4.58477	3.61503	
	0.02334	4.16489	4.88723	0.61958	0.77255	0.48576	0.95510														
369	0.92980	4.32167	3.20391	3.12147	4.32689	3.08753	4.26847	3.65629	3.26238	3.42241	4.28228	3.34982	3.82041	3.54358	3.59116	2.33835	2.85344	3.18456	5.69522	4.45489	399 a - - -
	2.68618	4.42225	2.77519	2.73123	3.46354	2.40513	3.72494	3.29354	2.67741	2.69355	4.24690	2.90347	2.73739	3.18146	2.89801	2.37887	2.77519	2.98518	4.58477	3.61503	
	0.02334	4.16489	4.88723	0.61958	0.77255	0.48576	0.95510														
370	1.78165	4.92458	2.92159	2.04539	4.27087	3.42712	3.70034	3.67502	2.40977	3.25833	4.08042	2.99143	3.87641	2.84867	2.33089	2.72654	2.95700	3.32833	5.46602	4.14011	400 a - - -
	2.68618	4.42225	2.77519	2.73123	3.46354	2.40513	3.72494	3.29354	2.67741	2.69355	4.24690	2.90347	2.73739	3.18146	2.89801	2.37887	2.77519	2.98518	4.58477	3.61503	
	0.02334	4.16489	4.88723	0.61958	0.77255	0.48576	0.95510														
371	3.86835	5.12987	4.56789	4.35347	3.02935	4.08352	4.30963	3.91661	4.07628	3.25628	4.53479	4.44682	4.62737	4.44811	4.14946	4.06898	4.18585	3.81445	0.45737	3.01784	401 W - - -
	2.68618	4.42225	2.77519	2.73123	3.46354	2.40513	3.72494	3.29354	2.67741	2.69355	4.24690	2.90347	2.73739	3.18146	2.89801	2.37887	2.77519	2.98518	4.58477	3.61503	
	0.02334	4.16489	4.88723	0.61958	0.77255	0.48576	0.95510														
372	3.05332	5.63254	1.79785	1.06720	4.92382	3.31167	3.86373	4.45049	2.88564	3.94549	4.80230	2.77617	3.93792	3.03113	3.48327	2.93162	3.34371	4.01858	6.08757	4.58187	402 e - - -
	2.68618	4.42225	2.77519	2.73123	3.46354	2.40513	3.72494	3.29354	2.67741	2.69355	4.24690	2.90347	2.73739	3.18146	2.89801	2.37887	2.77519	2.98518	4.58477	3.61503	
	0.02334	4.16489	4.88723	0.61958	0.77255	0.48576	0.95510														
373	2.59794	4.24021	3.61941	3.05694	3.37370	3.60643	3.89214	2.37760	2.99023	2.44924	3.35908	2.87671	3.99245	3.28182	3.30285	2.60720	2.72613	1.87541	4.84664	3.17298	403 v - - -
	2.68618	4.42225	2.77519	2.73123	3.46354	2.40513	3.72494	3.29354	2.67741	2.69355	4.24690	2.90347	2.73739	3.18146	2.89801	2.37887	2.77519	2.98518	4.58477	3.61503	
	0.02334	4.16489	4.88723	0.61958	0.77255	0.48576	0.95510														
374	2.74575	5.00256	2.81417	1.80602	4.12153	3.44219	3.67775	3.68737	2.45069	3.25102	4.08117	2.94995	3.88245	2.27288	2.88061	2.74537	2.98074	3.35311	5.38671	2.55310	404 e - - -
	2.68618	4.42225	2.77519	2.73123	3.46354	2.40513	3.72494	3.29354	2.67741	2.69355	4.24690	2.90347	2.73739	3.18146	2.89801	2.37887	2.77519	2.98518	4.58477	3.61503	
	0.02334	4.16489	4.88723	0.61958	0.77255	0.48576	0.95510														
375	3.21996	5.25869	3.68075	2.98953	4.73988	3.79183	3.71975	4.08528	1.74721	3.51623	4.41070	3.35605	4.16591	2.86135	0.99330	3.22395	3.37972	3.77993	5.53329	4.39895	405 r - - -
	2.68618	4.42225	2.77519	2.73123	3.46354	2.40513	3.72494	3.29354	2.67741	2.69355	4.24690	2.90347	2.73739	3.18146	2.89801	2.37887	2.77519	2.98518	4.58477	3.61503	

```
      0.02334  4.16489  4.88723  0.61958  0.77255  0.48576  0.95510
376   3.50060  4.88187  4.65844  4.30955  3.27444  4.35030  4.87486  2.50384  4.07499  0.58360  3.22348  4.55706  4.74162  4.37185  4.19907  3.97103  3.78494  2.61525  5.24914  4.03668    406 L - - -
      2.68618  4.42225  2.77519  2.73123  3.46354  2.40513  3.72494  3.29354  2.67741  2.69355  4.24690  2.90347  2.73739  3.18146  2.89801  2.37887  2.77519  2.98518  4.58477  3.61503
      0.02334  4.16489  4.88723  0.61958  0.77255  0.48576  0.95510
377   1.80081  4.61409  2.62005  2.62315  4.24459  3.24957  3.85944  3.65650  2.72249  3.28439  4.09906  3.06935  3.81404  3.03529  3.16863  1.87901  2.40058  3.24760  5.52331  4.20947    407 a - - -
      2.68618  4.42225  2.77519  2.73123  3.46354  2.40513  3.72494  3.29354  2.67741  2.69355  4.24690  2.90347  2.73739  3.18146  2.89801  2.37887  2.77519  2.98518  4.58477  3.61503
      0.10953  4.16489  2.42796  0.61958  0.77255  0.48576  0.95510
378   2.83148  5.29697  1.49038  2.00429  4.63160  2.74305  3.74247  4.11204  2.67119  3.63604  4.45182  2.77009  3.84622  2.89324  3.22099  2.76296  2.63444  3.69928  5.81409  4.36305    408 d - - -
      2.68618  4.42225  2.77519  2.73123  3.46354  2.40513  3.72494  3.29354  2.67741  2.69355  4.24690  2.90347  2.73739  3.18146  2.89801  2.37887  2.77519  2.98518  4.58477  3.61503
      0.02542  4.08077  4.80312  0.61958  0.77255  0.43111  1.04922
379   3.41348  4.70561  5.08623  4.51813  3.15620  4.71878  5.03106  1.73492  4.37727  0.90292  2.51207  4.76206  4.86723  4.44616  4.46593  4.07471  3.63170  2.25442  5.27456  4.23689    409 l - - -
      2.68618  4.42225  2.77519  2.73123  3.46354  2.40513  3.72494  3.29354  2.67741  2.69355  4.24690  2.90347  2.73739  3.18146  2.89801  2.37887  2.77519  2.98518  4.58477  3.61503
      0.02334  4.16489  4.88723  0.61958  0.77255  0.48576  0.95510
380   2.26097  4.23965  4.02127  3.47833  3.22837  3.79780  4.10362  1.63154  3.36135  2.27924  3.31399  3.73683  4.18356  3.63010  3.58986  3.10270  2.97521  2.23044  4.81402  2.52328    410 i - - -
      2.68618  4.42225  2.77519  2.73123  3.46354  2.40513  3.72494  3.29354  2.67741  2.69355  4.24690  2.90347  2.73739  3.18146  2.89801  2.37887  2.77519  2.98518  4.58477  3.61503
      0.02334  4.16489  4.88723  0.61958  0.77255  0.48576  0.95510
381   2.79618  4.82233  3.16083  2.66541  3.57192  3.57372  3.14297  3.44758  2.05471  3.04093  3.91882  2.90919  3.97403  2.94207  2.79719  2.85505  3.01890  3.16873  4.98164  1.89946    411 y - - -
      2.68618  4.42225  2.77519  2.73123  3.46354  2.40513  3.72494  3.29354  2.67741  2.69355  4.24690  2.90347  2.73739  3.18146  2.89801  2.37887  2.77519  2.98518  4.58477  3.61503
      0.02334  4.16489  4.88723  0.61958  0.77255  0.48576  0.95510
382   3.10099  5.71893  1.09086  1.64210  5.00068  3.30035  3.88867  4.54182  2.95856  4.02894  4.90141  2.75978  3.94845  3.06261  3.58726  2.96323  3.39996  4.10152  6.17152  4.63908    412 d - - -
      2.68618  4.42225  2.77519  2.73123  3.46354  2.40513  3.72494  3.29354  2.67741  2.69355  4.24690  2.90347  2.73739  3.18146  2.89801  2.37887  2.77519  2.98518  4.58477  3.61503
      0.02334  4.16489  4.88723  0.61958  0.77255  0.48576  0.95510
383   2.14107  4.36843  3.40787  2.92942  4.09369  3.19179  4.00591  3.47545  2.91480  3.15598  3.99259  3.25488  2.26634  3.23432  3.29888  2.06659  1.97068  2.86574  5.43866  4.18567    413 t - - -
      2.68618  4.42225  2.77519  2.73123  3.46354  2.40513  3.72494  3.29354  2.67741  2.69355  4.24690  2.90347  2.73739  3.18146  2.89801  2.37887  2.77519  2.98518  4.58477  3.61503
      0.02334  4.16489  4.88723  0.61958  0.77255  0.48576  0.95510
384   2.53577  5.00794  1.35305  2.36834  4.54840  3.27577  3.84237  3.97300  2.76432  3.57048  4.39346  2.89980  3.86356  3.00578  3.27807  2.54900  2.47483  3.55224  5.77750  4.37773    414 d - - -
      2.68618  4.42225  2.77519  2.73123  3.46354  2.40513  3.72494  3.29354  2.67741  2.69355  4.24690  2.90347  2.73739  3.18146  2.89801  2.37887  2.77519  2.98518  4.58477  3.61503
      0.02334  4.16489  4.88723  0.61958  0.77255  0.48576  0.95510
385   2.51369  4.55072  2.99651  2.79554  4.40693  3.15400  4.08335  3.93878  2.98890  3.57104  4.40669  2.42366  3.84323  3.30800  3.37076  1.09670  2.94538  3.43333  5.71239  4.38562    415 s - - -
      2.68618  4.42225  2.77519  2.73123  3.46354  2.40513  3.72494  3.29354  2.67741  2.69355  4.24690  2.90347  2.73739  3.18146  2.89801  2.37887  2.77519  2.98518  4.58477  3.61503
      0.02334  4.16489  4.88723  0.61958  0.77255  0.48576  0.95510
386   3.01263  5.14621  3.36452  2.78209  4.56666  3.65410  3.69396  3.96045  2.04462  3.43350  4.29314  3.20780  4.05434  2.34022  1.22092  2.82501  3.21008  3.62981  5.50692  4.29336    416 r - - -
      2.68618  4.42225  2.77519  2.73123  3.46354  2.40513  3.72494  3.29354  2.67741  2.69355  4.24690  2.90347  2.73739  3.18146  2.89801  2.37887  2.77519  2.98518  4.58477  3.61503
      0.02334  4.16489  4.88723  0.61958  0.77255  0.48576  0.95510
387   2.89820  4.29814  4.47110  3.89961  1.68551  4.03473  4.18647  2.28576  3.76024  1.76188  2.97893  4.03804  4.35314  3.91007  3.87953  3.34145  3.12569  2.16313  4.62808  2.83726    417 f - - -
      2.68618  4.42225  2.77519  2.73123  3.46354  2.40513  3.72494  3.29354  2.67741  2.69355  4.24690  2.90347  2.73739  3.18146  2.89801  2.37887  2.77519  2.98518  4.58477  3.61503
      0.02334  4.16489  4.88723  0.61958  0.77255  0.48576  0.95510
388   1.48597  4.27093  3.53464  3.33162  4.51373  1.22705  4.43046  3.89557  3.45773  3.62471  4.46459  3.44979  3.80580  3.72236  3.74659  2.51938  2.84662  3.32012  5.84485  4.66075    418 g - - -
      2.68618  4.42225  2.77519  2.73123  3.46354  2.40513  3.72494  3.29354  2.67741  2.69355  4.24690  2.90347  2.73739  3.18146  2.89801  2.37887  2.77519  2.98518  4.58477  3.61503
      0.02334  4.16489  4.88723  0.61958  0.77255  0.48576  0.95510
389   3.07087  4.80683  3.80366  3.74507  4.86220  0.39323  4.83738  4.54296  3.96555  4.15653  5.14181  3.96927  4.20642  4.26711  4.15990  3.25273  3.57468  4.01458  5.86989  4.97743    419 G - - -
      2.68618  4.42225  2.77519  2.73123  3.46354  2.40513  3.72494  3.29354  2.67741  2.69355  4.24690  2.90347  2.73739  3.18146  2.89801  2.37887  2.77519  2.98518  4.58477  3.61503
      0.02334  4.16489  4.88723  0.61958  0.77255  0.48576  0.95510
390   3.43828  4.80223  4.83737  4.36333  3.14509  4.56679  4.90734  2.19251  4.11589  0.69867  3.01636  4.61659  4.82392  4.34632  4.24842  3.99065  3.68984  2.38194  5.22667  4.03890    420 l - - -
      2.68618  4.42225  2.77519  2.73123  3.46354  2.40513  3.72494  3.29354  2.67741  2.69355  4.24690  2.90347  2.73739  3.18146  2.89801  2.37887  2.77519  2.98518  4.58477  3.61503
      0.02334  4.16489  4.88723  0.61958  0.77255  0.48576  0.95510
391   3.43216  4.73428  5.06263  4.49094  2.51066  4.69285  4.90921  2.14359  4.36312  0.79888  2.68786  4.72112  4.83567  4.39236  4.43691  4.03939  3.64372  2.44169  5.12937  4.01609    421 l - - -
      2.68618  4.42225  2.77519  2.73123  3.46354  2.40513  3.72494  3.29354  2.67741  2.69355  4.24690  2.90347  2.73739  3.18146  2.89801  2.37887  2.77519  2.98518  4.58477  3.61503
      0.02334  4.16489  4.88723  0.61958  0.77255  0.48576  0.95510
392   2.80482  4.36659  3.85875  2.88714  1.51904  3.80826  3.89477  2.71566  3.23137  2.30915  3.06031  3.62473  4.17405  3.50303  3.49257  3.09473  3.03678  2.56890  4.52981  2.93061    422 f - - -
      2.68618  4.42225  2.77519  2.73123  3.46354  2.40513  3.72494  3.29354  2.67741  2.69355  4.24690  2.90347  2.73739  3.18146  2.89801  2.37887  2.77519  2.98518  4.58477  3.61503
      0.02334  4.16489  4.88723  0.61958  0.77255  0.48576  0.95510
393   3.50454  4.82814  4.76185  4.36683  0.98550  4.45654  4.06233  2.68958  4.20141  1.73591  3.36311  4.35520  4.76047  4.27721  4.27987  3.86010  3.74977  2.75495  4.24614  2.61409    423 f - - -
      2.68618  4.42225  2.77519  2.73123  3.46354  2.40513  3.72494  3.29354  2.67741  2.69355  4.24690  2.90347  2.73739  3.18146  2.89801  2.37887  2.77519  2.98518  4.58477  3.61503
      0.02334  4.16489  4.88723  0.61958  0.77255  0.48576  0.95510
394   2.61183  4.13997  3.89152  3.31675  2.13238  3.38229  3.92306  2.52996  3.21950  2.33340  3.25445  3.58146  4.04816  3.46464  3.45302  2.66931  2.51345  2.40119  4.19096  2.77697    424 f - - -
      2.68618  4.42225  2.77519  2.73123  3.46354  2.40513  3.72494  3.29354  2.67741  2.69355  4.24690  2.90347  2.73739  3.18146  2.89801  2.37887  2.77519  2.98518  4.58477  3.61503
      0.02334  4.16489  4.88723  0.61958  0.77255  0.48576  0.95510
395   2.80191  5.11277  1.21557  2.32883  4.63249  3.27289  3.86967  4.07752  2.83071  3.66902  4.50664  2.87289  3.88510  3.04013  3.36304  2.55392  2.51990  3.65182  5.86469  4.43785    425 d - - -
      2.68618  4.42225  2.77519  2.73123  3.46354  2.40513  3.72494  3.29354  2.67741  2.69355  4.24690  2.90347  2.73739  3.18146  2.89801  2.37887  2.77519  2.98518  4.58477  3.61503
      0.02334  4.16489  4.88723  0.61958  0.77255  0.48576  0.95510
396   2.40044  5.08447  2.27257  2.02588  4.40778  3.36575  3.66438  3.86532  2.47488  3.40121  4.18500  2.86486  2.61404  2.79215  2.97442  2.66354  2.52776  3.47175  5.58217  4.18515    426 e - - -
      2.68618  4.42225  2.77519  2.73123  3.46354  2.40513  3.72494  3.29354  2.67741  2.69355  4.24690  2.90347  2.73739  3.18146  2.89801  2.37887  2.77519  2.98518  4.58477  3.61503
      0.02334  4.16489  4.88723  0.61958  0.77255  0.48576  0.95510
397   2.31157  4.96571  2.34511  2.27906  4.26076  3.31613  3.63531  3.70389  2.42377  3.26141  4.04924  2.81442  3.81304  2.68783  2.90714  2.21241  2.76226  2.96271  5.46118  4.08961    427 s - - -
      2.68619  4.42226  2.77520  2.73124  3.46355  2.40514  3.72495  3.29355  2.67742  2.69356  4.24617  2.90348  2.73740  3.18147  2.89802  2.37888  2.77520  2.98519  4.58478  3.61504
      0.08521  2.60186  4.88723  0.41339  1.08295  0.48576  0.95510
```

```
    398   2.77658  4.77953  3.01377  2.83061  4.53001  0.99389  4.10136  3.99878  2.38685  3.61723  4.49608  3.25002  3.97622  3.32497  3.21076  2.86797  3.16603  3.56846  5.72096  4.48781    429 g - - -
          2.68618  4.42225  2.77519  2.73123  3.46354  2.40513  3.72494  3.29354  2.67741  2.69355  4.24690  2.90347  2.73739  3.18146  2.89801  2.37887  2.77519  2.98518  4.58477  3.61503
          0.02334  4.16489  4.88723  0.61958  0.77255  0.48576  0.95510
    399   2.56527  4.99296  2.92373  2.40176  4.32064  3.13417  3.62604  3.76155  2.01496  3.29835  4.08245  2.28053  3.82726  2.74889  2.54083  2.57592  2.51486  3.38343  5.47367  4.11604    430 k - - -
          2.68618  4.42225  2.77519  2.73123  3.46354  2.40513  3.72494  3.29354  2.67741  2.69355  4.24690  2.90347  2.73739  3.18146  2.89801  2.37887  2.77519  2.98518  4.58477  3.61503
          0.02334  4.16489  4.88723  0.61958  0.77255  0.48576  0.95510
    400   3.36714  5.16534  3.89059  3.35258  4.59608  3.78529  4.08066  4.16726  2.41459  3.63009  4.63119  3.68530  4.28143  3.29398  0.61106  3.44264  3.61809  3.88059  5.56480  4.46945    431 R - - -
          2.68618  4.42225  2.77519  2.73123  3.46354  2.40513  3.72494  3.29354  2.67741  2.69355  4.24690  2.90347  2.73739  3.18146  2.89801  2.37887  2.77519  2.98518  4.58477  3.61503
          0.02334  4.16489  4.88723  0.61958  0.77255  0.48576  0.95510
    401   2.71354  4.70248  3.18462  2.66455  2.89627  3.55288  3.67725  3.29418  2.62187  2.90793  3.79516  2.77266  3.94662  1.89334  3.02973  2.79506  2.94527  3.02705  4.84813  2.70469    432 q - - -
          2.68618  4.42225  2.77519  2.73123  3.46354  2.40513  3.72494  3.29354  2.67741  2.69355  4.24690  2.90347  2.73739  3.18146  2.89801  2.37887  2.77519  2.98518  4.58477  3.61503
          0.02334  4.16489  4.88723  0.61958  0.77255  0.48576  0.95510
    402   2.37260  4.21657  4.13385  3.31046  3.32523  3.87997  4.20452  1.62336  3.45472  2.13282  2.60919  3.82092  4.23423  3.70290  3.67580  3.17204  2.98306  1.98871  4.92425  3.73206    433 i - - -
          2.68618  4.42225  2.77519  2.73123  3.46354  2.40513  3.72494  3.29354  2.67741  2.69355  4.24690  2.90347  2.73739  3.18146  2.89801  2.37887  2.77519  2.98518  4.58477  3.61503
          0.02334  4.16489  4.88723  0.61958  0.77255  0.48576  0.95510
    403   2.36649  4.15375  3.85747  3.28633  3.28713  3.66471  3.98252  2.23976  3.19615  2.34974  3.27961  3.57368  4.04913  3.45634  3.44843  2.65293  2.67197  1.82721  4.79075  2.94955    434 v - - -
          2.68618  4.42225  2.77519  2.73123  3.46354  2.40513  3.72494  3.29354  2.67741  2.69355  4.24690  2.90347  2.73739  3.18146  2.89801  2.37887  2.77519  2.98518  4.58477  3.61503
          0.02334  4.16489  4.88723  0.61958  0.77255  0.48576  0.95510
    404   2.20659  4.79341  2.99312  2.06242  4.04292  3.43202  3.65754  3.45294  2.45869  3.06362  3.22274  2.97772  3.83466  2.62350  2.91333  2.45532  2.62269  3.13294  5.31319  3.98360    435 e - - -
          2.68618  4.42225  2.77519  2.73123  3.46354  2.40513  3.72494  3.29354  2.67741  2.69355  4.24690  2.90347  2.73739  3.18146  2.89801  2.37887  2.77519  2.98518  4.58477  3.61503
          0.02334  4.16489  4.88723  0.61958  0.77255  0.48576  0.95510
    405   3.11766  4.43704  4.86305  4.36257  3.68284  4.46205  5.06230  1.06812  4.25285  2.22276  3.48513  4.56161  4.79115  4.51824  4.44650  3.84538  3.10662  1.34646  5.60394  4.40391    436 i - - -
          2.68584  4.42227  2.77522  2.73126  3.46356  2.40515  3.72497  3.29356  2.67743  2.69357  4.24692  2.90349  2.73742  3.18149  2.89803  2.37889  2.77522  2.98521  4.58479  3.61505
          0.17761  1.86314  4.88723  0.28412  1.39707  0.48576  0.95510
    406   2.81943  5.16940  1.38350  2.30147  4.72783  2.12943  3.87622  4.22821  2.85793  3.76540  4.59088  2.85146  3.88271  3.04413  3.41076  2.39303  3.15196  3.76572  5.93577  4.49275    438 d - - -
          2.68618  4.42225  2.77519  2.73123  3.46354  2.40513  3.72494  3.29354  2.67741  2.69355  4.24690  2.90347  2.73739  3.18146  2.89801  2.37887  2.77519  2.98518  4.58477  3.61503
          0.02334  4.16489  4.88723  0.61958  0.77255  0.48576  0.95510
    407   1.72311  4.66258  2.98879  2.62701  4.26818  2.24006  3.86460  3.68635  2.68695  3.30416  4.13158  3.07640  3.84297  2.22496  3.09979  2.64745  2.90742  3.28726  5.53511  4.22677    439 a - - -
          2.68618  4.42225  2.77519  2.73123  3.46354  2.40513  3.72494  3.29354  2.67741  2.69355  4.24690  2.90347  2.73739  3.18146  2.89801  2.37887  2.77519  2.98518  4.58477  3.61503
          0.02334  4.16489  4.88723  0.61958  0.77255  0.48576  0.95510
    408   2.38473  4.31932  3.64540  3.10847  2.94836  3.60162  3.98369  2.32819  3.04900  2.43377  3.43133  3.46391  2.00782  3.34823  3.37244  2.89444  2.90143  2.47621  4.96803  3.72078    440 p - - -
          2.68618  4.42225  2.77519  2.73123  3.46354  2.40513  3.72494  3.29354  2.67741  2.69355  4.24690  2.90347  2.73739  3.18146  2.89801  2.37887  2.77519  2.98518  4.58477  3.61503
          0.02334  4.16489  4.88723  0.61958  0.77255  0.48576  0.95510
    409   3.17097  4.52311  4.78279  4.28345  3.45331  4.42313  4.92731  1.94913  4.12989  1.47753  3.29641  4.50129  4.74454  4.38174  4.30640  3.80913  3.43621  1.10531  5.41299  4.21765    441 v - - -
          2.68618  4.42225  2.77519  2.73123  3.46354  2.40513  3.72494  3.29354  2.67741  2.69355  4.24690  2.90347  2.73739  3.18146  2.89801  2.37887  2.77519  2.98518  4.58477  3.61503
          0.02334  4.16489  4.88723  0.61958  0.77255  0.48576  0.95510
    410   3.21519  4.52199  4.95395  4.48240  3.61695  4.55899  5.18177  0.91562  4.35212  2.08201  3.42136  4.68265  4.87603  4.61649  4.53265  3.97083  3.48890  1.53802  5.63704  4.43195    442 i - - -
          2.68618  4.42225  2.77519  2.73123  3.46354  2.40513  3.72494  3.29354  2.67741  2.69355  4.24690  2.90347  2.73739  3.18146  2.89801  2.37887  2.77519  2.98518  4.58477  3.61503
          0.02334  4.16489  4.88723  0.61958  0.77255  0.48576  0.95510
    411   3.18863  4.88603  3.82888  3.71728  4.67093  3.55752  4.76170  4.30328  3.80198  3.89323  4.96207  3.98991  0.42795  4.18131  4.00541  3.36743  3.65485  3.91304  5.76614  4.79867    443 P - - -
          2.68618  4.42225  2.77519  2.73123  3.46354  2.40513  3.72494  3.29354  2.67741  2.69355  4.24690  2.90347  2.73739  3.18146  2.89801  2.37887  2.77519  2.98518  4.58477  3.61503
          0.02334  4.16489  4.88723  0.61958  0.77255  0.48576  0.95510
    412   2.64450  4.86403  3.01421  2.39446  4.14453  3.44444  3.64649  3.55820  1.91225  3.14451  3.95518  2.98057  3.84470  2.79019  2.69571  2.20476  2.54197  2.87870  5.36474  4.03639    444 k - - -
          2.68618  4.42225  2.77519  2.73123  3.46354  2.40513  3.72494  3.29354  2.67741  2.69355  4.24690  2.90347  2.73739  3.18146  2.89801  2.37887  2.77519  2.98518  4.58477  3.61503
          0.02334  4.16489  4.88723  0.61958  0.77255  0.48576  0.95510
    413   3.65633  4.97487  4.64261  4.42152  0.63212  4.25844  4.04309  3.15482  4.32396  2.46101  3.84666  4.36241  4.71705  4.41299  4.37075  3.90466  3.96096  3.16555  4.22202  2.55920    445 F - - -
          2.68618  4.42225  2.77519  2.73123  3.46354  2.40513  3.72494  3.29354  2.67741  2.69355  4.24690  2.90347  2.73739  3.18146  2.89801  2.37887  2.77519  2.98518  4.58477  3.61503
          0.02334  4.16489  4.88723  0.61958  0.77255  0.48576  0.95510
    414   2.59866  4.47107  3.26822  2.42388  3.63518  2.97544  3.75239  2.52765  2.67636  2.60644  3.10412  2.85305  3.90325  2.86354  3.07839  2.74826  2.83042  2.45596  5.03241  3.76881    446 e - - -
          2.68618  4.42225  2.77519  2.73123  3.46354  2.40513  3.72494  3.29354  2.67741  2.69355  4.24690  2.90347  2.73739  3.18146  2.89801  2.37887  2.77519  2.98518  4.58477  3.61503
          0.02334  4.16489  4.88723  0.61958  0.77255  0.48576  0.95510
    415   1.53830  4.13433  3.18399  2.90743  4.14210  2.19765  4.03609  3.51700  2.99740  3.22406  4.06852  3.14506  3.12731  3.29654  3.33265  2.35294  2.64815  3.03054  5.48376  4.25726    447 a - - -
          2.68612  4.42255  2.77525  2.73153  3.46384  2.40441  3.72524  3.29346  2.67744  2.69371  4.24514  2.90377  2.73732  3.18154  2.89814  2.37907  2.77505  2.98548  4.58416  3.61498
          0.29681  1.35939        *  1.85293  0.17052  0.00000        *
//
HMMER3/f [3.1b2 | February 2015]
NAME  pmoC_IA_strict Protein
LENG  255
ALPH  amino
RF    no
MM    no
CONS  yes
CS    no
MAP   yes
DATE  Mon Dec  4 12:55:44 2017
NSEQ  34
EFFN  0.734619
```

```
CKSUM 1819627275
STATS LOCAL MSV      -11.0699  0.70271
STATS LOCAL VITERBI  -11.7000  0.70271
STATS LOCAL FORWARD   -5.5153  0.70271
HMM          A        C        D        E        F        G        H        I        K        L        M        N        P        Q        R        S        T        V        W        Y
            m->m     m->i     m->d     i->m     i->i     d->m     d->d
  COMPO   2.53527  4.42005  3.21194  2.80239  2.91962  2.99124  3.63706  2.66894  2.93420  2.34697  3.43547  3.27359  3.42625  3.25323  3.08554  2.79326  2.80424  2.52518  3.71308  3.08016
          2.68618  4.42225  2.77519  2.73123  3.46354  2.40513  3.72494  3.29354  2.67741  2.69355  4.24690  2.90347  2.73739  3.18146  2.89801  2.37887  2.77519  2.98518  4.58477  3.61503
          0.02666  4.03368  4.75603  0.61958  0.77255  0.00000        *
      1   3.10282  4.62768  4.22590  3.83428  3.28796  3.95614  4.53220  2.48008  3.59269  1.86074  1.09964  4.09103  4.42003  3.96536  3.77853  3.46749  3.41256  2.50432  5.14866  3.92331      1 m - - -
          2.68618  4.42225  2.77519  2.73123  3.46354  2.40513  3.72494  3.29354  2.67741  2.69355  4.24690  2.90347  2.73739  3.18146  2.89801  2.37887  2.77519  2.98518  4.58477  3.61503
          0.02666  4.03368  4.75603  0.61958  0.77255  0.48576  0.95510
      2   1.16312  4.22670  3.33571  3.06090  4.24759  2.99995  4.16937  3.63393  3.13312  3.35086  4.19138  3.02976  3.72798  3.43715  3.45697  2.03203  2.75133  3.13824  5.59990  4.36531      2 a - - -
          2.68618  4.42225  2.77519  2.73123  3.46354  2.40513  3.72494  3.29354  2.67741  2.69355  4.24690  2.90347  2.73739  3.18146  2.89801  2.37887  2.77519  2.98518  4.58477  3.61503
          0.02666  4.03368  4.75603  0.61958  0.77255  0.48576  0.95510
      3   2.18129  4.74642  2.95690  2.40235  4.02303  3.38734  3.64218  3.43191  2.43934  2.94064  3.86615  2.67725  3.80607  2.44578  2.88373  2.51906  2.53701  3.04506  5.29321  3.96852      3 a - - -
          2.68618  4.42225  2.77519  2.73123  3.46354  2.40513  3.72494  3.29354  2.67741  2.69355  4.24690  2.90347  2.73739  3.18146  2.89801  2.37887  2.77519  2.98518  4.58477  3.61503
          0.02666  4.03368  4.75603  0.61958  0.77255  0.48576  0.95510
      4   2.40526  4.52790  3.13553  2.40671  3.74567  3.44496  3.72897  2.95752  2.59871  2.78372  3.65991  3.09577  3.86428  2.95271  3.00254  2.70564  1.95330  2.80874  5.11920  3.54074      4 t - - -
          2.68618  4.42225  2.77519  2.73123  3.46354  2.40513  3.72494  3.29354  2.67741  2.69355  4.24690  2.90347  2.73739  3.18146  2.89801  2.37887  2.77519  2.98518  4.58477  3.61503
          0.02666  4.03368  4.75603  0.61958  0.77255  0.48576  0.95510
      5   2.59111  4.54133  3.30717  2.84761  4.05213  3.33394  3.86326  3.38181  2.52914  3.06206  3.96705  3.22045  3.88236  3.07774  2.28777  2.71991  1.49505  3.06283  5.34719  4.09831      5 t - - -
          2.68618  4.42225  2.77519  2.73123  3.46354  2.40513  3.72494  3.29354  2.67741  2.69355  4.24690  2.90347  2.73739  3.18146  2.89801  2.37887  2.77519  2.98518  4.58477  3.61503
          0.02666  4.03368  4.75603  0.61958  0.77255  0.48576  0.95510
      6   2.50899  4.77052  2.84292  1.97848  4.01204  3.40131  3.62556  3.10398  2.43218  3.03136  3.85211  2.93977  3.80405  2.65364  2.89069  2.59388  2.58737  2.84607  5.28473  3.95285      6 e - - -
          2.68618  4.42225  2.77519  2.73123  3.46354  2.40513  3.72494  3.29354  2.67741  2.69355  4.24690  2.90347  2.73739  3.18146  2.89801  2.37887  2.77519  2.98518  4.58477  3.61503
          0.09795  4.03368  2.58240  0.61958  0.77255  0.48576  0.95510
      7   2.29650  4.80172  2.88376  2.32122  4.06556  3.37854  3.23149  3.48159  2.27641  3.07764  3.89562  2.91647  3.79501  2.67719  2.82511  2.30454  2.57998  3.15407  5.31170  3.98024      7 k - - -
          2.68574  4.42236  2.77518  2.73127  3.46365  2.40513  3.72506  3.29365  2.67752  2.69366  4.24630  2.90358  2.73739  3.18158  2.89806  2.37888  2.77506  2.98514  4.58488  3.61514
          0.29435  1.40338  4.68668  1.07800  0.41593  0.44468  1.02451
      8   2.02422  4.20322  3.70960  3.20053  3.59484  2.98068  4.07016  2.48607  3.13519  2.59002  3.53888  3.47547  3.94783  3.43146  3.43929  2.76233  2.62846  1.68904  5.09056  3.87375     14 v - - -
          2.68618  4.42225  2.77519  2.73123  3.46354  2.40513  3.72494  3.29354  2.67741  2.69355  4.24690  2.90347  2.73739  3.18146  2.89801  2.37887  2.77519  2.98518  4.58477  3.61503
          0.02666  4.03368  4.75603  0.61958  0.77255  0.48576  0.95510
      9   2.43024  4.71966  3.00442  2.47961  4.07347  2.80476  3.65926  3.48645  2.09317  3.09045  3.90649  2.97419  3.79791  2.81583  2.76845  2.28895  2.55417  3.02337  5.32683  4.00693     15 k - - -
          2.68618  4.42225  2.77519  2.73123  3.46354  2.40513  3.72494  3.29354  2.67741  2.69355  4.24690  2.90347  2.73739  3.18146  2.89801  2.37887  2.77519  2.98518  4.58477  3.61503
          0.02666  4.03368  4.75603  0.61958  0.77255  0.48576  0.95510
     10   2.00859  4.32235  3.39925  2.83796  3.49224  3.34996  3.79346  2.56328  2.77344  2.40273  3.44499  3.25074  3.90925  2.99741  2.98040  2.76560  2.66997  2.44470  4.92776  3.68799     16 a - - -
          2.68618  4.42225  2.77519  2.73123  3.46354  2.40513  3.72494  3.29354  2.67741  2.69355  4.24690  2.90347  2.73739  3.18146  2.89801  2.37887  2.77519  2.98518  4.58477  3.61503
          0.02666  4.03368  4.75603  0.61958  0.77255  0.48576  0.95510
     11   2.13353  4.66834  2.64300  2.42570  3.91262  3.40574  3.65921  3.15273  2.49429  2.78831  3.78018  2.98812  3.81624  2.83555  2.94280  2.33829  2.81445  2.83952  5.22457  3.91152     17 a - - -
          2.68618  4.42225  2.77519  2.73123  3.46354  2.40513  3.72494  3.29354  2.67741  2.69355  4.24690  2.90347  2.73739  3.18146  2.89801  2.37887  2.77519  2.98518  4.58477  3.61503
          0.02666  4.03368  4.75603  0.61958  0.77255  0.48576  0.95510
     12   1.97115  4.45870  3.18327  2.44430  3.72808  3.25915  3.76157  2.62687  2.66456  2.77557  3.64604  3.12438  3.85281  2.99907  3.06669  2.60077  2.52757  2.78567  5.11040  3.84173     18 a - - -
          2.68618  4.42225  2.77519  2.73123  3.46354  2.40513  3.72494  3.29354  2.67741  2.69355  4.24690  2.90347  2.73739  3.18146  2.89801  2.37887  2.77519  2.98518  4.58477  3.61503
          0.02666  4.03368  4.75603  0.61958  0.77255  0.48576  0.95510
     13   2.24701  4.90195  2.83467  1.97605  4.19402  3.20071  3.61138  3.54101  2.38928  3.19619  3.99358  2.88801  3.30337  2.59582  2.78881  2.52287  2.84874  3.27087  5.40419  4.04591     19 e - - -
          2.68618  4.42225  2.77519  2.73123  3.46354  2.40513  3.72494  3.29354  2.67741  2.69355  4.24690  2.90347  2.73739  3.18146  2.89801  2.37887  2.77519  2.98518  4.58477  3.61503
          0.02666  4.03368  4.75603  0.61958  0.77255  0.48576  0.95510
     14   2.48967  4.87077  2.74862  1.98688  4.13205  3.38281  3.60385  3.55951  2.35147  2.97488  3.94151  2.76922  3.78967  2.66518  2.86197  2.57292  2.71091  2.82760  5.36083  4.00718     20 e - - -
          2.68618  4.42225  2.77519  2.73123  3.46354  2.40513  3.72494  3.29354  2.67741  2.69355  4.24690  2.90347  2.73739  3.18146  2.89801  2.37887  2.77519  2.98518  4.58477  3.61503
          0.02666  4.03368  4.75603  0.61958  0.77255  0.48576  0.95510
     15   2.21101  4.84015  2.79123  2.20995  4.10310  3.38911  3.60387  3.52705  2.33419  3.11272  3.91628  2.91079  3.39360  2.74445  2.61563  2.60702  2.74519  2.87185  5.33760  3.99104     21 e - - -
          2.68618  4.42225  2.77519  2.73123  3.46354  2.40513  3.72494  3.29354  2.67741  2.69355  4.24690  2.90347  2.73739  3.18146  2.89801  2.37887  2.77519  2.98518  4.58477  3.61503
          0.02666  4.03368  4.75603  0.61958  0.77255  0.48576  0.95510
     16   2.50877  4.77658  2.90623  2.46577  4.15327  3.34987  3.68100  3.56734  2.21883  3.16281  3.98463  2.96345  2.27966  2.70310  2.85219  2.36300  2.77709  3.21887  5.38856  4.06817     22 k - - -
          2.68618  4.42225  2.77519  2.73123  3.46354  2.40513  3.72494  3.29354  2.67741  2.69355  4.24690  2.90347  2.73739  3.18146  2.89801  2.37887  2.77519  2.98518  4.58477  3.61503
          0.02666  4.03368  4.75603  0.61958  0.77255  0.48576  0.95510
     17   2.51611  4.21920  3.63081  3.05905  3.33981  3.61337  3.88595  2.43301  2.67584  1.92901  2.99883  3.41866  3.98852  3.27310  3.27696  2.67306  2.82498  2.24191  4.83209  3.61538     23 l - - -
          2.68618  4.42225  2.77519  2.73123  3.46354  2.40513  3.72494  3.29354  2.67741  2.69355  4.24690  2.90347  2.73739  3.18146  2.89801  2.37887  2.77519  2.98518  4.58477  3.61503
          0.02666  4.03368  4.75603  0.61958  0.77255  0.48576  0.95510
     18   2.58367  4.13569  3.79816  3.22104  2.75252  3.64003  3.90926  2.55533  2.78099  1.86725  3.21107  3.51871  3.72816  3.38646  3.37310  2.91274  2.81538  2.11821  4.22775  3.48089     24 l - - -
          2.68618  4.42225  2.77519  2.73123  3.46354  2.40513  3.72494  3.29354  2.67741  2.69355  4.24690  2.90347  2.73739  3.18146  2.89801  2.37887  2.77519  2.98518  4.58477  3.61503
          0.02666  4.03368  4.75603  0.61958  0.77255  0.48576  0.95510
     19   2.71007  5.19133  2.11092  2.04292  4.49918  3.09684  3.41870  3.98018  2.46849  3.48518  4.26500  2.34427  3.11792  2.76117  2.98764  2.43304  2.95834  3.56655  5.64597  4.21870     25 e - - -
          2.68618  4.42225  2.77519  2.73123  3.46354  2.40513  3.72494  3.29354  2.67741  2.69355  4.24690  2.90347  2.73739  3.18146  2.89801  2.37887  2.77519  2.98518  4.58477  3.61503
          0.02666  4.03368  4.75603  0.61958  0.77255  0.48576  0.95510
     20   2.58548  4.34009  3.40647  2.83535  3.41836  3.54156  3.76069  2.80373  2.39122  2.36706  3.43930  3.24968  3.68560  2.91959  3.08257  2.78686  2.81512  2.58228  3.06324  3.01011     26 l - - -
```

```
     2.68626  4.42233  2.77511  2.73131  3.46330  2.40521  3.72503  3.29356  2.67744  2.69331  4.24698  2.90318  2.73748  3.18155  2.89809  2.37895  2.77520  2.98527  4.58485  3.61472
     0.17361  1.89195  4.75603  1.13192  0.38922  0.48576  0.95510
 21  2.61183  4.78762  3.02384  2.45878  4.02940  3.12690  3.61291  3.44291  1.99479  3.04175  3.86175  2.81967  3.40086  2.76609  2.47368  2.64892  2.83853  3.12562  3.86370  3.71252    32 k - - -
     2.68618  4.42225  2.77519  2.73123  3.46354  2.40513  3.72494  3.29354  2.67741  2.69355  4.24690  2.90347  2.73739  3.18146  2.89801  2.37887  2.77519  2.98518  4.58477  3.61503
     0.02666  4.03368  4.75603  0.61958  0.77255  0.48576  0.95510
 22  2.60299  4.79173  2.99886  2.44350  4.04248  3.05630  3.61311  3.45581  2.25086  3.05332  3.87012  2.54301  3.45776  2.76407  2.40357  2.63740  2.72616  3.13384  3.84815  3.96367    33 k - - -
     2.68618  4.42225  2.77519  2.73123  3.46354  2.40513  3.72494  3.29354  2.67741  2.69355  4.24690  2.90347  2.73739  3.18146  2.89801  2.37887  2.77519  2.98518  4.58477  3.61503
     0.02666  4.03368  4.75603  0.61958  0.77255  0.48576  0.95510
 23  2.67200  4.20582  4.26060  3.68030  2.42438  3.89204  4.12952  2.33964  3.55379  1.60282  2.43553  3.87578  4.22210  3.73756  3.71074  3.18879  2.99660  2.32641  4.67621  2.88333    34 l - - -
     2.68618  4.42225  2.77519  2.73123  3.46354  2.40513  3.72494  3.29354  2.67741  2.69355  4.24690  2.90347  2.73739  3.18146  2.89801  2.37887  2.77519  2.98518  4.58477  3.61503
     0.02666  4.03368  4.75603  0.61958  0.77255  0.48576  0.95510
 24  2.52188  4.06845  4.04322  3.46196  2.82635  3.69333  3.93980  2.31841  3.33939  2.03249  3.15466  3.66251  4.05812  3.55514  3.51846  2.98099  2.62716  2.27765  2.99114  2.72411    35 l - - -
     2.68618  4.42225  2.77519  2.73123  3.46354  2.40513  3.72494  3.29354  2.67741  2.69355  4.24690  2.90347  2.73739  3.18146  2.89801  2.37887  2.77519  2.98518  4.58477  3.61503
     0.02666  4.03368  4.75603  0.61958  0.77255  0.48576  0.95510
 25  2.45782  4.16954  3.73807  3.16534  2.83407  3.63913  3.91971  2.20984  2.70956  1.97819  3.25465  3.49033  4.01153  3.35473  3.35133  2.80573  2.82527  2.30389  4.77996  3.56658    36 l - - -
     2.68618  4.42225  2.77519  2.73123  3.46354  2.40513  3.72494  3.29354  2.67741  2.69355  4.24690  2.90347  2.73739  3.18146  2.89801  2.37887  2.77519  2.98518  4.58477  3.61503
     0.02666  4.03368  4.75603  0.61958  0.77255  0.48576  0.95510
 26  2.04912  3.75277  3.42708  2.91021  3.66530  2.11565  3.88779  3.02727  2.86723  2.74502  3.62357  3.25887  3.83472  3.18226  3.22415  2.63203  2.66709  2.73940  5.08318  3.11549    37 a - - -
     2.68618  4.42225  2.77519  2.73123  3.46354  2.40513  3.72494  3.29354  2.67741  2.69355  4.24690  2.90347  2.73739  3.18146  2.89801  2.37887  2.77519  2.98518  4.58477  3.61503
     0.02666  4.03368  4.75603  0.61958  0.77255  0.48576  0.95510
 27  2.46533  3.33481  4.06800  3.48094  2.68475  3.68405  3.99346  2.25582  3.35644  1.99211  2.97837  3.67803  4.05058  3.33470  3.52768  2.93281  2.71503  2.07956  4.65767  3.46810    38 l - - -
     2.68618  4.42225  2.77519  2.73123  3.46354  2.40513  3.72494  3.29354  2.67741  2.69355  4.24690  2.90347  2.73739  3.18146  2.89801  2.37887  2.77519  2.98518  4.58477  3.61503
     0.02666  4.03368  4.75603  0.61958  0.77255  0.48576  0.95510
 28  2.11251  4.26385  3.51091  2.96924  3.52272  2.55373  3.89415  2.36880  2.91413  2.40823  3.47633  3.33282  3.91767  3.22288  3.25597  2.68184  2.76394  2.39113  4.98064  3.74922    39 a - - -
     2.68618  4.42225  2.77519  2.73123  3.46354  2.40513  3.72494  3.29354  2.67741  2.69355  4.24690  2.90347  2.73739  3.18146  2.89801  2.37887  2.77519  2.98518  4.58477  3.61503
     0.02666  4.03368  4.75603  0.61958  0.77255  0.48576  0.95510
 29  2.52510  4.18702  4.23194  3.65897  2.89914  3.72822  4.23778  1.91431  3.54746  1.66672  3.17243  3.88531  4.24880  3.76892  3.73836  3.20433  2.98536  1.84042  4.88423  3.69672    40 l - - -
     2.68618  4.42225  2.77519  2.73123  3.46354  2.40513  3.72494  3.29354  2.67741  2.69355  4.24690  2.90347  2.73739  3.18146  2.89801  2.37887  2.77519  2.98518  4.58477  3.61503
     0.02666  4.03368  4.75603  0.61958  0.77255  0.48576  0.95510
 30  2.88109  4.32631  4.26471  3.69572  2.99233  3.99826  4.18528  2.07092  3.55949  1.43068  2.93405  3.93037  4.31395  3.77361  3.74675  3.29764  3.10899  2.30278  4.72381  2.39493    41 l - - -
     2.68618  4.42225  2.77519  2.73123  3.46354  2.40513  3.72494  3.29354  2.67741  2.69355  4.24690  2.90347  2.73739  3.18146  2.89801  2.37887  2.77519  2.98518  4.58477  3.61503
     0.02666  4.03368  4.75603  0.61958  0.77255  0.48576  0.95510
 31  2.51468  4.02684  3.32174  2.77252  3.63650  3.41237  3.79030  2.89446  2.72730  2.44973  3.57344  2.84358  3.85910  3.05620  3.11062  2.25866  2.18366  2.68085  5.04176  3.78713    42 t - - -
     2.68618  4.42225  2.77519  2.73123  3.46354  2.40513  3.72494  3.29354  2.67741  2.69355  4.24690  2.90347  2.73739  3.18146  2.89801  2.37887  2.77519  2.98518  4.58477  3.61503
     0.02666  4.03368  4.75603  0.61958  0.77255  0.48576  0.95510
 32  2.59554  4.21764  4.00720  3.45635  3.40688  3.13232  4.19470  1.98672  3.35967  2.09790  3.34055  3.74011  4.18750  3.63844  3.61696  3.10026  2.95688  1.51099  5.00174  3.79231    43 v - - -
     2.68618  4.42225  2.77519  2.73123  3.46354  2.40513  3.72494  3.29354  2.67741  2.69355  4.24690  2.90347  2.73739  3.18146  2.89801  2.37887  2.77519  2.98518  4.58477  3.61503
     0.02666  4.03368  4.75603  0.61958  0.77255  0.48576  0.95510
 33  2.99380  4.38630  4.44535  3.90550  1.47598  4.07520  3.98058  2.42838  3.75947  1.88240  2.75976  4.00784  4.38353  3.89186  3.87995  3.38659  3.21870  2.51474  3.58378  2.81059    44 f - - -
     2.68618  4.42225  2.77519  2.73123  3.46354  2.40513  3.72494  3.29354  2.67741  2.69355  4.24690  2.90347  2.73739  3.18146  2.89801  2.37887  2.77519  2.98518  4.58477  3.61503
     0.02666  4.03368  4.75603  0.61958  0.77255  0.48576  0.95510
 34  3.06855  4.74058  3.44681  2.89865  2.67439  3.75052  3.71633  3.26651  3.07325  2.83390  3.91219  3.48791  4.21575  3.45673  3.33897  3.19830  3.33126  3.07949  4.28441  1.11112    45 y - - -
     2.68618  4.42225  2.77519  2.73123  3.46354  2.40513  3.72494  3.29354  2.67741  2.69355  4.24690  2.90347  2.73739  3.18146  2.89801  2.37887  2.77519  2.98518  4.58477  3.61503
     0.02666  4.03368  4.75603  0.61958  0.77255  0.48576  0.95510
 35  2.46416  4.15601  3.75326  3.18244  3.28676  2.97729  3.93390  2.21007  3.09977  2.03471  2.94703  3.49936  4.00934  3.37253  3.37575  2.75492  2.81891  2.18708  4.79160  3.58275    46 l - - -
     2.68618  4.42225  2.77519  2.73123  3.46354  2.40513  3.72494  3.29354  2.67741  2.69355  4.24690  2.90347  2.73739  3.18146  2.89801  2.37887  2.77519  2.98518  4.58477  3.61503
     0.02666  4.03368  4.75603  0.61958  0.77255  0.48576  0.95510
 36  2.29688  4.11293  3.75925  3.18279  3.03377  3.05673  3.89078  2.46914  3.09757  2.22341  3.05877  3.48489  3.98105  3.35825  3.35858  2.74192  2.78534  2.29048  3.18707  3.50245    47 l - - -
     2.68618  4.42225  2.77519  2.73123  3.46354  2.40513  3.72494  3.29354  2.67741  2.69355  4.24690  2.90347  2.73739  3.18146  2.89801  2.37887  2.77519  2.98518  4.58477  3.61503
     0.02666  4.03368  4.75603  0.61958  0.77255  0.48576  0.95510
 37  2.87247  3.24561  4.56359  4.00200  3.14751  4.12635  4.52562  1.73858  3.88328  1.96566  3.25639  4.17687  4.45262  4.08978  4.03756  3.45352  3.11574  1.32058  5.08360  3.89298    48 v - - -
     2.68618  4.42225  2.77519  2.73123  3.46354  2.40513  3.72494  3.29354  2.67741  2.69355  4.24690  2.90347  2.73739  3.18146  2.89801  2.37887  2.77519  2.98518  4.58477  3.61503
     0.02666  4.03368  4.75603  0.61958  0.77255  0.48576  0.95510
 38  2.75244  4.80407  3.24926  2.66728  4.06186  3.52301  3.66319  3.46152  2.21538  3.05607  3.17517  2.76334  3.92373  2.83561  1.60842  2.81371  2.97646  3.17262  5.26957  3.99933    49 r - - -
     2.68618  4.42225  2.77519  2.73123  3.46354  2.40513  3.72494  3.29354  2.67741  2.69355  4.24690  2.90347  2.73739  3.18146  2.89801  2.37887  2.77519  2.98518  4.58477  3.61503
     0.02666  4.03368  4.75603  0.61958  0.77255  0.48576  0.95510
 39  2.84434  4.26178  4.36814  3.79020  3.02528  3.98795  4.22877  1.91559  3.63881  1.54650  3.04801  3.98072  4.30685  3.83293  3.78832  3.29398  3.07390  2.11107  2.72347  3.49664    50 l - - -
     2.68618  4.42225  2.77519  2.73123  3.46354  2.40513  3.72494  3.29354  2.67741  2.69355  4.24690  2.90347  2.73739  3.18146  2.89801  2.37887  2.77519  2.98518  4.58477  3.61503
     0.02666  4.03368  4.75603  0.61958  0.77255  0.48576  0.95510
 40  3.48747  4.91087  4.07975  3.84179  2.31656  4.02068  3.70522  3.47503  3.69483  2.88668  4.13379  3.92717  4.49191  3.96171  3.84439  3.60710  3.77558  3.35354  3.96564  0.73471    51 y - - -
     2.68618  4.42225  2.77519  2.73123  3.46354  2.40513  3.72494  3.29354  2.67741  2.69355  4.24690  2.90347  2.73739  3.18146  2.89801  2.37887  2.77519  2.98518  4.58477  3.61503
     0.02666  4.03368  4.75603  0.61958  0.77255  0.48576  0.95510
 41  2.87388  5.26432  2.47309  1.45270  4.56040  3.34294  3.71127  4.03402  2.47519  3.54388  4.39038  2.83303  3.87569  2.07393  2.89881  2.81458  3.12858  3.65853  5.69562  4.30453    52 e - - -
     2.68618  4.42225  2.77519  2.73123  3.46354  2.40513  3.72494  3.29354  2.67741  2.69355  4.24690  2.90347  2.73739  3.18146  2.89801  2.37887  2.77519  2.98518  4.58477  3.61503
     0.02666  4.03368  4.75603  0.61958  0.77255  0.48576  0.95510
 42  2.73270  4.93347  2.96397  2.51027  4.31805  2.18746  3.66036  3.74212  2.26040  3.28559  4.11328  2.99848  3.87253  2.25374  2.14223  2.74901  2.97785  3.38819  5.44547  4.14684    53 r - - -
     2.68618  4.42225  2.77519  2.73123  3.46354  2.40513  3.72494  3.29354  2.67741  2.69355  4.24690  2.90347  2.73739  3.18146  2.89801  2.37887  2.77519  2.98518  4.58477  3.61503
```

	0.02666	4.03368	4.75603	0.61958	0.77255	0.48576	0.95510														
43	2.08700	4.20651	3.80167	3.24506	3.36495	3.67168	4.02705	2.25513	3.16166	2.34204	3.32963	3.56312	4.06950	3.44650	3.44525	2.96106	2.76543	1.75980	4.90250	3.23511	54 v - - -
	2.68618	4.42225	2.77519	2.73123	3.46354	2.40513	3.72494	3.29354	2.67741	2.69355	4.24690	2.90347	2.73739	3.18146	2.89801	2.37887	2.77519	2.98518	4.58477	3.61503	
	0.02666	4.03368	4.75603	0.61958	0.77255	0.48576	0.95510														
44	3.64830	4.88297	4.63911	4.32727	1.18901	4.39924	3.48359	3.32796	4.17276	2.65672	3.92474	4.08231	4.70769	4.13891	4.20410	3.76890	3.87029	3.25528	3.62297	1.24187	55 f - - -
	2.68618	4.42225	2.77519	2.73123	3.46354	2.40513	3.72494	3.29354	2.67741	2.69355	4.24690	2.90347	2.73739	3.18146	2.89801	2.37887	2.77519	2.98518	4.58477	3.61503	
	0.02666	4.03368	4.75603	0.61958	0.77255	0.48576	0.95510														
45	1.56470	4.18035	3.38711	3.14467	4.37136	1.51398	4.25650	3.77500	3.25868	3.47596	4.29861	3.30218	3.69992	3.52903	3.57334	2.23787	2.72671	3.20737	5.71031	4.49928	56 g - - -
	2.68618	4.42225	2.77519	2.73123	3.46354	2.40513	3.72494	3.29354	2.67741	2.69355	4.24690	2.90347	2.73739	3.18146	2.89801	2.37887	2.77519	2.98518	4.58477	3.61503	
	0.02666	4.03368	4.75603	0.61958	0.77255	0.48576	0.95510														
46	2.88028	4.30545	4.31656	3.76378	2.58342	3.97381	4.02731	1.96730	3.61962	1.98166	3.16412	3.92050	4.30535	3.80387	3.77420	3.28109	3.11166	2.27527	2.09982	3.01474	57 i - - -
	2.68618	4.42225	2.77519	2.73123	3.46354	2.40513	3.72494	3.29354	2.67741	2.69355	4.24690	2.90347	2.73739	3.18146	2.89801	2.37887	2.77519	2.98518	4.58477	3.61503	
	0.02666	4.03368	4.75603	0.61958	0.77255	0.48576	0.95510														
47	2.35223	4.63732	3.07093	2.54161	3.92592	3.39620	3.67916	3.32184	2.41707	2.95797	3.79668	3.02410	3.82416	2.86152	2.63174	1.99953	2.55882	3.01578	5.22764	3.68116	58 s - - -
	2.68618	4.42225	2.77519	2.73123	3.46354	2.40513	3.72494	3.29354	2.67741	2.69355	4.24690	2.90347	2.73739	3.18146	2.89801	2.37887	2.77519	2.98518	4.58477	3.61503	
	0.02666	4.03368	4.75603	0.61958	0.77255	0.48576	0.95510														
48	1.89693	4.78041	2.99765	2.51987	4.10069	3.40985	3.21450	3.50349	1.96425	3.10817	3.94486	3.00705	3.85009	2.83619	2.73596	2.70201	2.78665	3.18071	5.33275	4.03120	59 a - - -
	2.68618	4.42225	2.77519	2.73123	3.46354	2.40513	3.72494	3.29354	2.67741	2.69355	4.24690	2.90347	2.73739	3.18146	2.89801	2.37887	2.77519	2.98518	4.58477	3.61503	
	0.02666	4.03368	4.75603	0.61958	0.77255	0.48576	0.95510														
49	2.87735	4.63403	3.58392	3.50518	4.63929	0.51867	4.61309	4.28057	3.70822	3.91370	4.89265	3.74860	4.03020	4.01983	3.92413	3.05432	3.37115	3.77778	5.68928	4.74753	60 G - - -
	2.68618	4.42225	2.77519	2.73123	3.46354	2.40513	3.72494	3.29354	2.67741	2.69355	4.24690	2.90347	2.73739	3.18146	2.89801	2.37887	2.77519	2.98518	4.58477	3.61503	
	0.02666	4.03368	4.75603	0.61958	0.77255	0.48576	0.95510														
50	3.31852	4.65618	4.82204	4.27471	3.12220	4.56293	4.86777	1.87895	4.09294	0.88955	2.85176	4.55290	4.75985	4.26993	4.24051	3.92244	3.54777	2.17180	5.21011	4.09726	61 l - - -
	2.68618	4.42225	2.77519	2.73123	3.46354	2.40513	3.72494	3.29354	2.67741	2.69355	4.24690	2.90347	2.73739	3.18146	2.89801	2.37887	2.77519	2.98518	4.58477	3.61503	
	0.02666	4.03368	4.75603	0.61958	0.77255	0.48576	0.95510														
51	3.17015	5.31440	0.71172	2.44939	4.71481	3.33942	4.08322	4.34124	3.19942	3.92193	4.90624	3.01284	4.00386	3.32453	3.73547	3.11617	3.51310	3.96414	5.85135	4.56456	62 d - - -
	2.68618	4.42225	2.77519	2.73123	3.46354	2.40513	3.72494	3.29354	2.67741	2.69355	4.24690	2.90347	2.73739	3.18146	2.89801	2.37887	2.77519	2.98518	4.58477	3.61503	
	0.02666	4.03368	4.75603	0.61958	0.77255	0.48576	0.95510														
52	2.69080	4.46538	3.44885	2.96041	2.78800	3.55985	3.72484	3.04406	2.91772	2.69072	3.64515	3.32709	4.00056	3.22808	3.26407	1.72816	2.95909	2.81220	4.52917	2.35457	63 s - - -
	2.68618	4.42225	2.77519	2.73123	3.46354	2.40513	3.72494	3.29354	2.67741	2.69355	4.24690	2.90347	2.73739	3.18146	2.89801	2.37887	2.77519	2.98518	4.58477	3.61503	
	0.02666	4.03368	4.75603	0.61958	0.77255	0.48576	0.95510														
53	2.54627	4.23137	3.68670	3.22160	1.84395	3.45122	3.96502	2.76924	3.15649	2.50900	3.48950	3.48107	3.96925	3.44382	3.43245	2.53910	2.25923	2.54934	4.79241	3.42880	64 f - - -
	2.68618	4.42225	2.77519	2.73123	3.46354	2.40513	3.72494	3.29354	2.67741	2.69355	4.24690	2.90347	2.73739	3.18146	2.89801	2.37887	2.77519	2.98518	4.58477	3.61503	
	0.02666	4.03368	4.75603	0.61958	0.77255	0.48576	0.95510														
54	1.75973	4.57156	2.93843	2.28002	4.13539	3.22961	3.81785	3.51881	2.68020	3.17380	4.00991	3.02995	3.79136	3.00909	3.10442	2.02994	2.70693	3.14503	5.43485	4.13222	65 a - - -
	2.68618	4.42225	2.77519	2.73123	3.46354	2.40513	3.72494	3.29354	2.67741	2.69355	4.24690	2.90347	2.73739	3.18146	2.89801	2.37887	2.77519	2.98518	4.58477	3.61503	
	0.02666	4.03368	4.75603	0.61958	0.77255	0.48576	0.95510														
55	2.76061	4.93480	2.68169	2.30764	4.29191	3.34598	3.74784	3.72719	2.50244	3.30780	4.17021	2.94158	1.72358	2.51410	2.88810	2.77646	3.03766	3.38554	5.51594	4.18423	66 p - - -
	2.68618	4.42225	2.77519	2.73123	3.46354	2.40513	3.72494	3.29354	2.67741	2.69355	4.24690	2.90347	2.73739	3.18146	2.89801	2.37887	2.77519	2.98518	4.58477	3.61503	
	0.02666	4.03368	4.75603	0.61958	0.77255	0.48576	0.95510														
56	2.55977	5.36372	1.91614	1.45503	4.65971	3.27081	3.71487	4.14994	2.63768	3.65688	4.47266	2.73502	3.83266	2.71766	3.18795	2.76100	3.11346	3.73489	5.82478	4.36098	67 e - - -
	2.68618	4.42225	2.77519	2.73123	3.46354	2.40513	3.72494	3.29354	2.67741	2.69355	4.24690	2.90347	2.73739	3.18146	2.89801	2.37887	2.77519	2.98518	4.58477	3.61503	
	0.02666	4.03368	4.75603	0.61958	0.77255	0.48576	0.95510														
57	3.54653	4.83189	4.55742	4.22407	0.94569	4.33755	3.56073	3.16611	4.07556	2.49390	3.78149	4.06994	4.66077	4.09894	4.13977	3.71521	3.77790	3.11894	3.72038	1.77817	68 f - - -
	2.68618	4.42225	2.77519	2.73123	3.46354	2.40513	3.72494	3.29354	2.67741	2.69355	4.24690	2.90347	2.73739	3.18146	2.89801	2.37887	2.77519	2.98518	4.58477	3.61503	
	0.02666	4.03368	4.75603	0.61958	0.77255	0.48576	0.95510														
58	2.76250	5.19581	2.47195	1.75590	4.50622	3.34589	3.64405	3.97032	2.40240	3.47540	4.27354	2.81963	3.82765	1.98734	2.70381	2.71217	3.00434	3.57533	5.62725	4.23197	69 e - - -
	2.68618	4.42225	2.77519	2.73123	3.46354	2.40513	3.72494	3.29354	2.67741	2.69355	4.24690	2.90347	2.73739	3.18146	2.89801	2.37887	2.77519	2.98518	4.58477	3.61503	
	0.02666	4.03368	4.75603	0.61958	0.77255	0.48576	0.95510														
59	2.52959	4.54757	3.17240	2.64152	3.79227	3.44439	3.72458	2.95645	2.47194	2.81382	3.69056	3.10340	3.86573	2.58509	2.92550	2.70645	1.93515	2.74983	5.14638	3.87261	70 t - - -
	2.68618	4.42225	2.77519	2.73123	3.46354	2.40513	3.72494	3.29354	2.67741	2.69355	4.24690	2.90347	2.73739	3.18146	2.89801	2.37887	2.77519	2.98518	4.58477	3.61503	
	0.02666	4.03368	4.75603	0.61958	0.77255	0.48576	0.95510														
60	3.48747	4.91087	4.07975	3.84179	2.31656	4.02068	3.70522	3.47503	3.69483	2.88668	4.13379	3.92717	4.49191	3.96171	3.84439	3.60710	3.77558	3.35354	3.96564	0.73471	71 y - - -
	2.68618	4.42225	2.77519	2.73123	3.46354	2.40513	3.72494	3.29354	2.67741	2.69355	4.24690	2.90347	2.73739	3.18146	2.89801	2.37887	2.77519	2.98518	4.58477	3.61503	
	0.02666	4.03368	4.75603	0.61958	0.77255	0.48576	0.95510														
61	3.61679	4.93173	4.34628	4.09412	2.84921	3.91802	4.11223	3.65007	3.81429	3.01594	4.27597	4.21130	4.44976	4.19354	3.91337	3.81884	3.92595	3.54480	0.61461	2.84332	72 W - - -
	2.68618	4.42225	2.77519	2.73123	3.46354	2.40513	3.72494	3.29354	2.67741	2.69355	4.24690	2.90347	2.73739	3.18146	2.89801	2.37887	2.77519	2.98518	4.58477	3.61503	
	0.02666	4.03368	4.75603	0.61958	0.77255	0.48576	0.95510														
62	2.96187	4.44933	4.21646	3.66033	2.95532	4.00640	4.16431	2.48232	3.42719	1.77341	1.48539	3.91510	4.33734	3.72781	3.62496	3.32817	3.19724	2.49214	3.89089	3.36614	73 m - - -
	2.68618	4.42225	2.77519	2.73123	3.46354	2.40513	3.72494	3.29354	2.67741	2.69355	4.24690	2.90347	2.73739	3.18146	2.89801	2.37887	2.77519	2.98518	4.58477	3.61503	
	0.02666	4.03368	4.75603	0.61958	0.77255	0.48576	0.95510														
63	2.60244	4.78553	2.90141	2.47654	4.23408	3.32209	3.69740	3.66140	2.43508	3.24353	4.05927	1.95313	3.81150	2.85210	2.50557	2.31587	2.60707	3.28735	5.44477	4.11985	74 n - - -
	2.68618	4.42225	2.77519	2.73123	3.46354	2.40513	3.72494	3.29354	2.67741	2.69355	4.24690	2.90347	2.73739	3.18146	2.89801	2.37887	2.77519	2.98518	4.58477	3.61503	
	0.02666	4.03368	4.75603	0.61958	0.77255	0.48576	0.95510														
64	3.18943	4.52186	4.83675	4.25909	2.27283	4.42622	4.68873	1.85922	4.13191	1.16715	2.49340	4.46277	4.63347	4.20700	4.22228	3.75739	3.40782	2.28638	5.01147	3.89205	75 l - - -
	2.68618	4.42225	2.77519	2.73123	3.46354	2.40513	3.72494	3.29354	2.67741	2.69355	4.24690	2.90347	2.73739	3.18146	2.89801	2.37887	2.77519	2.98518	4.58477	3.61503	
	0.02666	4.03368	4.75603	0.61958	0.77255	0.48576	0.95510														

65	3.30041	4.64377	4.78641	4.23934	2.13137	4.48239	4.57680	2.28352	4.10045	0.96619	2.74727	4.45287	4.68378	4.19360	4.21536	3.82267	3.51971	2.42181	4.85549	3.55473	76 l - - -
	2.68618	4.42225	2.77519	2.73123	3.46354	2.40513	3.72494	3.29354	2.67741	2.69355	4.24690	2.90347	2.73739	3.18146	2.89801	2.37887	2.77519	2.98518	4.58477	3.61503	
	0.02666	4.03368	4.75603	0.61958	0.77255	0.48576	0.95510														
66	2.94347	4.41954	4.08349	3.56523	2.39553	3.91164	3.64774	2.70323	3.42146	2.34660	3.53275	3.38327	4.26356	3.63460	3.62098	3.20683	3.17089	2.72465	2.51373	1.61828	77 y - - -
	2.68618	4.42225	2.77519	2.73123	3.46354	2.40513	3.72494	3.29354	2.67741	2.69355	4.24690	2.90347	2.73739	3.18146	2.89801	2.37887	2.77519	2.98518	4.58477	3.61503	
	0.02666	4.03368	4.75603	0.61958	0.77255	0.48576	0.95510														
67	2.41439	4.23345	3.58106	3.04224	3.26981	2.83067	3.92502	2.13015	2.98523	2.49649	3.42705	3.38838	3.94664	3.28338	3.30927	2.79535	2.19088	2.42953	4.94276	3.71656	78 i - - -
	2.68618	4.42225	2.77519	2.73123	3.46354	2.40513	3.72494	3.29354	2.67741	2.69355	4.24690	2.90347	2.73739	3.18146	2.89801	2.37887	2.77519	2.98518	4.58477	3.61503	
	0.02666	4.03368	4.75603	0.61958	0.77255	0.48576	0.95510														
68	2.72185	4.90172	2.72668	1.49913	4.14212	3.38423	3.71209	2.92231	2.51505	3.15988	4.03365	2.80158	3.86451	2.88370	2.94424	2.74385	2.97616	3.16958	5.42755	4.07197	79 e - - -
	2.68618	4.42225	2.77519	2.73123	3.46354	2.40513	3.72494	3.29354	2.67741	2.69355	4.24690	2.90347	2.73739	3.18146	2.89801	2.37887	2.77519	2.98518	4.58477	3.61503	
	0.02666	4.03368	4.75603	0.61958	0.77255	0.48576	0.95510														
69	2.70315	4.17819	4.07983	3.31017	2.94251	3.82623	4.13503	1.87728	3.39993	1.76592	3.05549	3.76382	4.17521	3.63655	3.61712	3.11483	2.60825	2.07507	4.83860	3.65207	80 l - - -
	2.68618	4.42225	2.77519	2.73123	3.46354	2.40513	3.72494	3.29354	2.67741	2.69355	4.24690	2.90347	2.73739	3.18146	2.89801	2.37887	2.77519	2.98518	4.58477	3.61503	
	0.02666	4.03368	4.75603	0.61958	0.77255	0.48576	0.95510														
70	2.35316	4.22573	3.77362	3.23028	3.46106	3.62017	4.06097	2.04085	3.15720	2.41372	3.40174	3.54803	3.00019	3.44897	3.45580	2.92551	2.76085	1.77930	4.99525	3.77950	81 v - - -
	2.68618	4.42225	2.77519	2.73123	3.46354	2.40513	3.72494	3.29354	2.67741	2.69355	4.24690	2.90347	2.73739	3.18146	2.89801	2.37887	2.77519	2.98518	4.58477	3.61503	
	0.02666	4.03368	4.75603	0.61958	0.77255	0.48576	0.95510														
71	2.65454	4.05216	4.31685	3.73885	3.02207	3.96797	4.29444	2.01828	3.62469	1.47021	2.68737	3.95917	4.29445	3.82445	3.79884	3.27016	2.89720	2.00285	4.89565	3.73518	82 l - - -
	2.68618	4.42225	2.77519	2.73123	3.46354	2.40513	3.72494	3.29354	2.67741	2.69355	4.24690	2.90347	2.73739	3.18146	2.89801	2.37887	2.77519	2.98518	4.58477	3.61503	
	0.02666	4.03368	4.75603	0.61958	0.77255	0.48576	0.95510														
72	2.65980	4.86694	2.80519	1.65542	4.12637	3.38528	3.66635	3.10693	2.45953	3.13641	3.97830	2.61611	3.83210	2.82395	2.90321	2.68223	2.90558	3.02346	5.38929	4.04038	83 e - - -
	2.68618	4.42225	2.77519	2.73123	3.46354	2.40513	3.72494	3.29354	2.67741	2.69355	4.24690	2.90347	2.73739	3.18146	2.89801	2.37887	2.77519	2.98518	4.58477	3.61503	
	0.02666	4.03368	4.75603	0.61958	0.77255	0.48576	0.95510														
73	2.31708	4.16756	3.73953	3.17281	3.18265	3.36535	3.94418	2.40652	3.09660	2.13947	3.29326	3.49391	3.71725	3.37148	3.38060	2.89630	2.72547	1.85905	4.82147	3.60975	84 v - - -
	2.68618	4.42225	2.77519	2.73123	3.46354	2.40513	3.72494	3.29354	2.67741	2.69355	4.24690	2.90347	2.73739	3.18146	2.89801	2.37887	2.77519	2.98518	4.58477	3.61503	
	0.02666	4.03368	4.75603	0.61958	0.77255	0.48576	0.95510														
74	2.34725	4.19240	3.97780	3.41764	3.37963	3.76719	4.14492	1.97727	3.32613	2.05883	3.32264	3.70266	4.15510	3.59694	3.58313	2.85727	2.80096	1.70246	4.95096	3.74417	85 v - - -
	2.68618	4.42225	2.77519	2.73123	3.46354	2.40513	3.72494	3.29354	2.67741	2.69355	4.24690	2.90347	2.73739	3.18146	2.89801	2.37887	2.77519	2.98518	4.58477	3.61503	
	0.02666	4.03368	4.75603	0.61958	0.77255	0.48576	0.95510														
75	2.38925	4.20822	3.65027	3.08623	3.20872	3.59202	3.91861	2.33263	3.02129	2.30990	3.33984	3.43515	3.98890	3.30792	3.33187	2.54440	2.29735	2.14116	4.86460	3.64499	86 v - - -
	2.68618	4.42225	2.77519	2.73123	3.46354	2.40513	3.72494	3.29354	2.67741	2.69355	4.24690	2.90347	2.73739	3.18146	2.89801	2.37887	2.77519	2.98518	4.58477	3.61503	
	0.02666	4.03368	4.75603	0.61958	0.77255	0.48576	0.95510														
76	2.03837	4.15810	3.73233	3.16606	2.64216	3.12591	3.87149	2.62131	3.08084	2.20803	3.27137	3.47337	3.98956	3.35006	3.35250	2.87926	2.81365	2.41939	3.59044	3.40335	87 a - - -
	2.68618	4.42225	2.77519	2.73123	3.46354	2.40513	3.72494	3.29354	2.67741	2.69355	4.24690	2.90347	2.73739	3.18146	2.89801	2.37887	2.77519	2.98518	4.58477	3.61503	
	0.02666	4.03368	4.75603	0.61958	0.77255	0.48576	0.95510														
77	2.31080	3.92418	3.48189	2.93157	3.51418	2.84471	3.85858	2.47662	2.88031	2.39573	3.47347	3.29716	3.88603	3.18458	3.22740	2.20496	2.65364	2.58580	4.95618	3.72467	88 s - - -
	2.68618	4.42225	2.77519	2.73123	3.46354	2.40513	3.72494	3.29354	2.67741	2.69355	4.24690	2.90347	2.73739	3.18146	2.89801	2.37887	2.77519	2.98518	4.58477	3.61503	
	0.02666	4.03368	4.75603	0.61958	0.77255	0.48576	0.95510														
78	2.26148	4.22025	3.66110	3.10130	3.16900	2.58551	3.93738	2.22796	3.03668	2.29111	3.34462	3.44915	4.00046	3.32428	3.34753	2.87631	2.83666	2.19069	4.88279	3.66072	89 v - - -
	2.68618	4.42225	2.77519	2.73123	3.46354	2.40513	3.72494	3.29354	2.67741	2.69355	4.24690	2.90347	2.73739	3.18146	2.89801	2.37887	2.77519	2.98518	4.58477	3.61503	
	0.02666	4.03368	4.75603	0.61958	0.77255	0.48576	0.95510														
79	2.53908	4.15796	3.97459	3.40225	2.93197	3.74479	4.06582	2.19161	3.30446	1.70456	3.20707	3.67397	4.11294	3.55343	3.53878	2.90419	2.58382	2.08874	4.81603	3.61775	90 l - - -
	2.68618	4.42225	2.77519	2.73123	3.46354	2.40513	3.72494	3.29354	2.67741	2.69355	4.24690	2.90347	2.73739	3.18146	2.89801	2.37887	2.77519	2.98518	4.58477	3.61503	
	0.02666	4.03368	4.75603	0.61958	0.77255	0.48576	0.95510														
80	2.35565	3.46319	3.73805	3.16753	3.24640	3.40578	3.89073	2.53724	3.06710	2.27635	3.26617	3.22267	3.97000	3.34607	3.33300	2.85419	2.78966	2.40161	2.52774	3.51094	91 l - - -
	2.68618	4.42225	2.77519	2.73123	3.46354	2.40513	3.72494	3.29354	2.67741	2.69355	4.24690	2.90347	2.73739	3.18146	2.89801	2.37887	2.77519	2.98518	4.58477	3.61503	
	0.02666	4.03368	4.75603	0.61958	0.77255	0.48576	0.95510														
81	2.27662	4.23248	3.25853	3.07823	4.40417	1.16976	4.24740	3.81450	3.22601	3.52238	4.36017	3.27506	3.72125	3.51981	3.53475	2.30057	2.48429	3.25132	5.73810	4.50905	92 g - - -
	2.68618	4.42225	2.77519	2.73123	3.46354	2.40513	3.72494	3.29354	2.67741	2.69355	4.24690	2.90347	2.73739	3.18146	2.89801	2.37887	2.77519	2.98518	4.58477	3.61503	
	0.02666	4.03368	4.75603	0.61958	0.77255	0.48576	0.95510														
82	3.65111	4.88645	4.63060	4.29515	1.85687	4.39278	3.43958	3.44270	4.11268	2.79141	4.01454	4.04787	4.69597	4.09974	4.15464	3.74667	3.86598	3.33336	2.00012	1.07768	93 y - - -
	2.68618	4.42225	2.77519	2.73123	3.46354	2.40513	3.72494	3.29354	2.67741	2.69355	4.24690	2.90347	2.73739	3.18146	2.89801	2.37887	2.77519	2.98518	4.58477	3.61503	
	0.02666	4.03368	4.75603	0.61958	0.77255	0.48576	0.95510														
83	3.28229	4.60817	4.83234	4.30536	3.18105	4.54415	4.89367	1.58896	4.13301	1.03577	3.00117	4.56293	4.76449	4.31988	4.27861	3.91808	3.51934	2.04644	5.24619	4.10916	94 l - - -
	2.68618	4.42225	2.77519	2.73123	3.46354	2.40513	3.72494	3.29354	2.67741	2.69355	4.24690	2.90347	2.73739	3.18146	2.89801	2.37887	2.77519	2.98518	4.58477	3.61503	
	0.02666	4.03368	4.75603	0.61958	0.77255	0.48576	0.95510														
84	3.61679	4.93173	4.34628	4.09412	2.84921	3.91802	4.11223	3.65007	3.81429	3.01594	4.27597	4.21130	4.44976	4.19354	3.91337	3.81884	3.92595	3.54480	0.61461	2.84332	95 W - - -
	2.68618	4.42225	2.77519	2.73123	3.46354	2.40513	3.72494	3.29354	2.67741	2.69355	4.24690	2.90347	2.73739	3.18146	2.89801	2.37887	2.77519	2.98518	4.58477	3.61503	
	0.02666	4.03368	4.75603	0.61958	0.77255	0.48576	0.95510														
85	2.58737	4.49045	3.22707	2.66033	3.22763	3.49217	3.70733	2.93805	1.96493	2.64058	3.57649	3.12362	3.87522	2.94620	2.88181	2.72211	2.69610	2.62723	5.02806	3.45597	96 k - - -
	2.68618	4.42225	2.77519	2.73123	3.46354	2.40513	3.72494	3.29354	2.67741	2.69355	4.24690	2.90347	2.73739	3.18146	2.89801	2.37887	2.77519	2.98518	4.58477	3.61503	
	0.02666	4.03368	4.75603	0.61958	0.77255	0.48576	0.95510														
86	2.35682	4.21659	3.56833	3.14758	3.91685	3.15658	4.11759	3.07102	3.08591	2.94335	3.85976	3.37042	3.81208	3.41571	3.39650	2.25598	1.46765	2.34569	5.35050	4.12250	97 t - - -
	2.68619	4.42226	2.77520	2.73124	3.46355	2.40514	3.72495	3.29355	2.67742	2.69356	4.24691	2.90348	2.73740	3.18147	2.89802	2.37888	2.77520	2.98519	4.58478	3.61466	
	0.09562	2.49385	4.75603	0.41739	1.07518	0.48576	0.95510														
87	3.19496	5.01753	3.70281	3.18361	4.41333	3.64851	3.95253	3.96851	2.31809	3.45525	4.45357	3.52980	4.14536	3.16690	0.75800	3.27280	3.45129	3.68607	5.43230	4.30818	99 r - - -

	2.68618	4.42225	2.77519	2.73123	3.46354	2.40513	3.72494	3.29354	2.67741	2.69355	4.24690	2.90347	2.73739	3.18146	2.89801	2.37887	2.77519	2.98518	4.58477	3.61503	
	0.02666	4.03368	4.75603	0.61958	0.77255	0.48576	0.95510														
88	2.74828	5.03942	1.28482	2.28460	4.47999	3.23872	3.79846	3.88468	2.74284	3.51102	4.37199	2.82819	3.48391	2.97086	3.26174	2.73292	2.94558	3.29522	5.73810	4.31913	100 d - - -
	2.68618	4.42225	2.77519	2.73123	3.46354	2.40513	3.72494	3.29354	2.67741	2.69355	4.24690	2.90347	2.73739	3.18146	2.89801	2.37887	2.77519	2.98518	4.58477	3.61503	
	0.02666	4.03368	4.75603	0.61958	0.77255	0.48576	0.95510														
89	3.02252	5.11324	3.38128	2.78841	4.54578	3.63760	3.64505	3.91997	1.83940	3.39346	4.26455	2.99675	4.03367	2.78939	1.21445	3.03121	3.20904	3.60093	5.45011	4.26205	101 r - - -
	2.68618	4.42225	2.77519	2.73123	3.46354	2.40513	3.72494	3.29354	2.67741	2.69355	4.24690	2.90347	2.73739	3.18146	2.89801	2.37887	2.77519	2.98518	4.58477	3.61503	
	0.02666	4.03368	4.75603	0.61958	0.77255	0.48576	0.95510														
90	2.60488	5.12338	2.44105	2.27078	4.42556	3.34477	3.06853	3.89117	2.13865	3.40702	4.19292	2.04626	3.80302	2.67537	2.86920	2.65928	2.93822	3.49680	5.56912	4.17237	102 n - - -
	2.68618	4.42225	2.77519	2.73123	3.46354	2.40513	3.72494	3.29354	2.67741	2.69355	4.24690	2.90347	2.73739	3.18146	2.89801	2.37887	2.77519	2.98518	4.58477	3.61503	
	0.02666	4.03368	4.75603	0.61958	0.77255	0.48576	0.95510														
91	3.15699	4.50519	4.72964	4.16079	3.02128	4.40716	4.72769	2.03050	4.04887	1.13736	2.56209	4.40946	4.63382	4.18196	4.19334	3.73664	3.38256	1.75962	5.13344	4.03813	103 l - - -
	2.68618	4.42225	2.77519	2.73123	3.46354	2.40513	3.72494	3.29354	2.67741	2.69355	4.24690	2.90347	2.73739	3.18146	2.89801	2.37887	2.77519	2.98518	4.58477	3.61503	
	0.02666	4.03368	4.75603	0.61958	0.77255	0.48576	0.95510														
92	2.47115	4.94059	2.08188	2.09547	4.21545	3.35553	3.63107	3.63947	2.44617	3.22166	3.16572	2.86506	3.79818	2.77223	2.93016	2.53262	2.88148	3.28753	5.44040	4.07112	104 d - - -
	2.68618	4.42225	2.77519	2.73123	3.46354	2.40513	3.72494	3.29354	2.67741	2.69355	4.24690	2.90347	2.73739	3.18146	2.89801	2.37887	2.77519	2.98518	4.58477	3.61503	
	0.02666	4.03368	4.75603	0.61958	0.77255	0.48576	0.95510														
93	2.25075	4.86244	2.87182	2.39572	4.18240	3.36837	3.62947	3.60948	2.25464	3.18448	3.98927	2.66952	3.80019	2.37361	2.83411	2.33999	2.64893	3.25531	5.39583	4.05183	105 a - - -
	2.68618	4.42225	2.77519	2.73123	3.46354	2.40513	3.72494	3.29354	2.67741	2.69355	4.24690	2.90347	2.73739	3.18146	2.89801	2.37887	2.77519	2.98518	4.58477	3.61503	
	0.02666	4.03368	4.75603	0.61958	0.77255	0.48576	0.95510														
94	3.16080	4.46778	4.84600	4.30650	3.36046	4.50396	4.93211	1.50748	4.19084	1.40679	3.15941	4.53965	4.74839	4.38040	4.35383	3.86208	3.40261	1.47240	5.35791	4.21957	106 l - - -
	2.68618	4.42225	2.77519	2.73123	3.46354	2.40513	3.72494	3.29354	2.67741	2.69355	4.24690	2.90347	2.73739	3.18146	2.89801	2.37887	2.77519	2.98518	4.58477	3.61503	
	0.02666	4.03368	4.75603	0.61958	0.77255	0.48576	0.95510														
95	1.99686	4.58724	2.96365	2.38882	4.05079	3.27794	3.77331	3.44285	2.62796	3.09049	3.92752	2.81378	3.80069	2.96421	3.05413	2.46391	2.04230	3.09502	5.35754	4.05634	107 a - - -
	2.68618	4.42225	2.77520	2.73123	3.46354	2.40509	3.72495	3.29354	2.67741	2.69355	4.24690	2.90347	2.73740	3.18146	2.89801	2.37887	2.77520	2.98518	4.58477	3.61503	
	0.04739	3.27838	4.75603	0.53872	0.87586	0.48576	0.95510														
96	2.30881	4.33028	3.25090	3.02625	4.23758	3.07587	4.16989	3.53517	3.06991	3.27384	4.20731	3.28546	1.25887	3.43469	3.37540	2.55674	2.66252	3.11653	5.61176	4.36503	109 p - - -
	2.68618	4.42225	2.77519	2.73123	3.46354	2.40513	3.72494	3.29354	2.67741	2.69355	4.24690	2.90347	2.73739	3.18146	2.89801	2.37887	2.77519	2.98518	4.58477	3.61503	
	0.02666	4.03368	4.75603	0.61958	0.77255	0.48576	0.95510														
97	2.60468	4.91159	3.03762	2.39649	4.22345	2.98524	3.61271	3.62686	2.05996	3.18632	4.00374	2.98593	3.85440	2.75257	1.90242	2.71091	2.91738	3.05712	5.37243	4.07010	110 r - - -
	2.68618	4.42225	2.77519	2.73123	3.46354	2.40513	3.72494	3.29354	2.67741	2.69355	4.24690	2.90347	2.73739	3.18146	2.89801	2.37887	2.77519	2.98518	4.58477	3.61503	
	0.02666	4.03368	4.75603	0.61958	0.77255	0.48576	0.95510														
98	2.95408	5.28640	2.39007	1.08164	4.56757	3.31877	3.80445	4.05979	2.65322	3.60078	4.50654	2.84622	3.90942	2.83667	3.09344	2.89344	3.23770	3.70313	5.76091	4.36075	111 e - - -
	2.68618	4.42225	2.77519	2.73123	3.46354	2.40513	3.72494	3.29354	2.67741	2.69355	4.24690	2.90347	2.73739	3.18146	2.89801	2.37887	2.77519	2.98518	4.58477	3.61503	
	0.02666	4.03368	4.75603	0.61958	0.77255	0.48576	0.95510														
99	3.09938	5.23515	2.56707	0.81610	4.60288	3.38409	3.98855	4.08925	2.89003	3.68170	4.65157	3.03581	3.99722	3.20272	3.31344	3.06710	3.40581	3.75755	5.74405	4.46989	112 e - - -
	2.68618	4.42225	2.77519	2.73123	3.46354	2.40513	3.72494	3.29354	2.67741	2.69355	4.24690	2.90347	2.73739	3.18146	2.89801	2.37887	2.77519	2.98518	4.58477	3.61503	
	0.02666	4.03368	4.75603	0.61958	0.77255	0.48576	0.95510														
100	3.30721	4.63648	4.85547	4.28936	3.09760	4.56642	4.86387	1.89428	4.14317	0.94282	2.44723	4.56268	4.74358	4.26705	4.27454	3.91120	3.52771	2.22283	5.18635	4.12317	113 l - - -
	2.68618	4.42225	2.77519	2.73123	3.46354	2.40513	3.72494	3.29354	2.67741	2.69355	4.24690	2.90347	2.73739	3.18146	2.89801	2.37887	2.77519	2.98518	4.58477	3.61503	
	0.02666	4.03368	4.75603	0.61958	0.77255	0.48576	0.95510														
101	3.10330	5.17385	3.54046	2.86551	4.63267	3.70113	3.64132	3.97895	1.61762	3.42285	4.30633	3.25055	4.07313	2.78091	1.21811	3.10560	3.26771	3.66973	5.45910	4.30626	114 r - - -
	2.68618	4.42225	2.77519	2.73123	3.46354	2.40513	3.72494	3.29354	2.67741	2.69355	4.24690	2.90347	2.73739	3.18146	2.89801	2.37887	2.77519	2.98518	4.58477	3.61503	
	0.02666	4.03368	4.75603	0.61958	0.77255	0.48576	0.95510														
102	2.78590	4.89462	3.49775	2.96470	4.34149	3.54699	3.80102	3.74797	2.16810	3.29411	4.24728	3.32200	4.03634	2.98324	1.04817	3.01301	3.20647	3.44726	5.42478	4.22507	115 r - - -
	2.68618	4.42225	2.77519	2.73123	3.46354	2.40513	3.72494	3.29354	2.67741	2.69355	4.24690	2.90347	2.73739	3.18146	2.89801	2.37887	2.77519	2.98518	4.58477	3.61503	
	0.02666	4.03368	4.75603	0.61958	0.77255	0.48576	0.95510														
103	2.68896	4.30213	3.68970	3.13373	2.71519	3.67713	2.88168	2.54276	3.06169	2.36019	3.41439	2.92924	4.04656	3.32877	3.35929	2.94001	2.91913	2.58837	3.40705	2.37466	116 l - - -
	2.68618	4.42225	2.77519	2.73123	3.46354	2.40513	3.72494	3.29354	2.67741	2.69355	4.24690	2.90347	2.73739	3.18146	2.89801	2.37887	2.77519	2.98518	4.58477	3.61503	
	0.02666	4.03368	4.75603	0.61958	0.77255	0.48576	0.95510														
104	2.65109	4.15793	4.23396	3.67209	1.95143	3.81840	3.87537	2.55350	3.53245	2.10696	3.04058	3.78891	4.17082	3.70267	3.66616	3.11728	2.96040	2.24067	3.16007	2.53257	117 f - - -
	2.68618	4.42225	2.77519	2.73123	3.46354	2.40513	3.72494	3.29354	2.67741	2.69355	4.24690	2.90347	2.73739	3.18146	2.89801	2.37887	2.77519	2.98518	4.58477	3.61503	
	0.02666	4.03368	4.75603	0.61958	0.77255	0.48576	0.95510														
105	2.56587	4.62223	2.95155	2.51689	3.86843	3.20949	3.59223	3.26757	2.52507	2.91153	3.75261	2.75614	3.82040	2.86520	2.96384	2.43394	2.11966	2.77191	5.19450	3.66350	118 t - - -
	2.68618	4.42225	2.77519	2.73123	3.46354	2.40513	3.72494	3.29354	2.67741	2.69355	4.24690	2.90347	2.73739	3.18146	2.89801	2.37887	2.77519	2.98518	4.58477	3.61503	
	0.02666	4.03368	4.75603	0.61958	0.77255	0.48576	0.95510														
106	2.67522	4.65088	3.05960	2.54770	3.71033	3.47787	2.62759	3.23244	2.50048	2.09031	3.75473	2.64997	3.89494	2.92574	2.86816	2.76714	2.90904	2.96652	5.08232	3.74034	119 l - - -
	2.68618	4.42225	2.77519	2.73123	3.46354	2.40513	3.72494	3.29354	2.67741	2.69355	4.24690	2.90347	2.73739	3.18146	2.89801	2.37887	2.77519	2.98518	4.58477	3.61503	
	0.02666	4.03368	4.75603	0.61958	0.77255	0.48576	0.95510														
107	2.50812	4.12988	3.74543	3.17098	3.10276	3.01322	3.89288	2.51132	3.08483	2.14730	3.23742	3.47939	3.98408	3.35153	3.35197	2.87748	2.53378	2.25493	3.00389	3.50969	120 l - - -
	2.68618	4.42225	2.77519	2.73123	3.46354	2.40513	3.72494	3.29354	2.67741	2.69355	4.24690	2.90347	2.73739	3.18146	2.89801	2.37887	2.77519	2.98518	4.58477	3.61503	
	0.02666	4.03368	4.75603	0.61958	0.77255	0.48576	0.95510														
108	2.51331	4.27299	3.45395	2.83624	3.41059	3.39465	3.79937	2.57787	2.82865	2.17146	2.96989	3.15973	3.92359	2.84010	3.17533	2.79146	2.58619	2.38486	4.85961	3.62741	121 l - - -
	2.68618	4.42225	2.77519	2.73123	3.46354	2.40513	3.72494	3.29354	2.67741	2.69355	4.24690	2.90347	2.73739	3.18146	2.89801	2.37887	2.77519	2.98518	4.58477	3.61503	
	0.02666	4.03368	4.75603	0.61958	0.77255	0.48576	0.95510														
109	3.10172	4.48955	4.42270	3.91868	2.06520	4.11842	3.81249	2.48780	3.76316	1.97885	3.32423	3.97883	4.43336	3.89640	3.89165	3.43521	3.32742	2.64438	1.75493	2.49057	122 w - - -
	2.68619	4.42226	2.77520	2.73124	3.46355	2.40514	3.72495	3.29355	2.67742	2.69356	4.24691	2.90348	2.73740	3.18147	2.89802	2.37888	2.77504	2.98519	4.58478	3.61504	

	0.09562	2.49385	4.75603	0.41739	1.07518	0.48576	0.95510													
110	3.25003	4.57064	4.85123	4.30989	3.21829	4.54224	4.91359	1.58177	4.16418	1.08764	3.01881	4.56410	4.76040	4.33417	4.31314	3.90364	3.48640	1.91284	5.27835	4.16350	124 l - - -
	2.68618	4.42225	2.77519	2.73123	3.46354	2.40513	3.72494	3.29354	2.67741	2.69355	4.24690	2.90347	2.73739	3.18146	2.89801	2.37887	2.77519	2.98518	4.58477	3.61503	
	0.02666	4.03368	4.75603	0.61958	0.77255	0.48576	0.95510														
111	2.36849	4.19414	3.61683	3.06639	3.08602	3.00587	3.90590	2.66544	3.00351	2.46031	3.24832	3.39925	3.93969	3.29097	3.31371	2.50687	2.79416	1.85558	4.87325	3.65143	125 v - - -
	2.68618	4.42225	2.77519	2.73123	3.46354	2.40513	3.72494	3.29354	2.67741	2.69355	4.24690	2.90347	2.73739	3.18146	2.89801	2.37887	2.77519	2.98518	4.58477	3.61503	
	0.02666	4.03368	4.75603	0.61958	0.77255	0.48576	0.95510														
112	2.38050	3.52796	4.18655	3.62217	3.35684	3.84660	4.24889	1.97966	3.51845	2.26593	2.99630	3.84937	4.22646	3.76149	3.72972	3.15687	2.79254	1.48319	4.95600	3.75750	126 v - - -
	2.68618	4.42225	2.77519	2.73123	3.46354	2.40513	3.72494	3.29354	2.67741	2.69355	4.24690	2.90347	2.73739	3.18146	2.89801	2.37887	2.77519	2.98518	4.58477	3.61503	
	0.02666	4.03368	4.75603	0.61958	0.77255	0.48576	0.95510														
113	3.54816	4.88521	4.38666	4.06078	1.90360	4.27103	3.52122	3.32021	3.91569	2.68934	3.95029	3.98054	4.62408	4.00990	4.03012	3.66107	3.78900	3.23199	3.70259	0.86436	127 y - - -
	2.68618	4.42225	2.77519	2.73123	3.46354	2.40513	3.72494	3.29354	2.67741	2.69355	4.24690	2.90347	2.73739	3.18146	2.89801	2.37887	2.77519	2.98518	4.58477	3.61503	
	0.02666	4.03368	4.75603	0.61958	0.77255	0.48576	0.95510														
114	1.48103	4.18237	3.47892	3.13453	4.22545	2.10735	4.18545	3.57534	3.16300	3.30321	4.14039	3.31080	3.71507	3.45371	3.48790	2.42669	2.00177	3.08558	5.58139	4.36887	128 a - - -
	2.68618	4.42225	2.77519	2.73123	3.46354	2.40513	3.72494	3.29354	2.67741	2.69355	4.24690	2.90347	2.73739	3.18146	2.89801	2.37887	2.77519	2.98518	4.58477	3.61503	
	0.02666	4.03368	4.75603	0.61958	0.77255	0.48576	0.95510														
115	2.53260	4.10305	4.14861	3.56859	2.56056	3.76230	4.02462	2.24464	3.43774	2.10045	3.13031	3.75424	4.12120	3.64473	3.60189	3.05749	2.83232	2.00372	2.67320	3.36145	129 v - - -
	2.68618	4.42225	2.77519	2.73123	3.46354	2.40513	3.72494	3.29354	2.67741	2.69355	4.24690	2.90347	2.73739	3.18146	2.89801	2.37887	2.77519	2.98518	4.58477	3.61503	
	0.02666	4.03368	4.75603	0.61958	0.77255	0.48576	0.95510														
116	1.23179	3.43124	3.72869	3.27740	3.88651	2.92198	4.16231	3.15285	3.19872	2.76796	3.84163	3.41618	3.77365	3.49555	3.48508	2.39629	2.71954	2.79140	5.31496	4.10918	130 a - - -
	2.68618	4.42225	2.77519	2.73123	3.46354	2.40513	3.72494	3.29354	2.67741	2.69355	4.24690	2.90347	2.73739	3.18146	2.89801	2.37887	2.77519	2.98518	4.58477	3.61503	
	0.02666	4.03368	4.75603	0.61958	0.77255	0.48576	0.95510														
117	3.15876	4.45676	4.86867	4.33744	3.42377	4.52593	4.98385	1.29080	4.22370	1.61636	3.22129	4.56783	4.77603	4.43090	4.39467	3.89100	3.40449	1.46312	5.42058	4.26550	131 i - - -
	2.68618	4.42225	2.77519	2.73123	3.46354	2.40513	3.72494	3.29354	2.67741	2.69355	4.24690	2.90347	2.73739	3.18146	2.89801	2.37887	2.77519	2.98518	4.58477	3.61503	
	0.02666	4.03368	4.75603	0.61958	0.77255	0.48576	0.95510														
118	3.02615	4.56994	3.98790	3.56665	2.50981	3.89463	3.76717	2.84779	3.40681	2.46712	3.63210	3.74726	4.31809	3.69321	3.62432	3.26096	3.29788	2.30459	4.16716	1.24440	132 y - - -
	2.68618	4.42225	2.77519	2.73123	3.46354	2.40513	3.72494	3.29354	2.67741	2.69355	4.24690	2.90347	2.73739	3.18146	2.89801	2.37887	2.77519	2.98518	4.58477	3.61503	
	0.02666	4.03368	4.75603	0.61958	0.77255	0.48576	0.95510														
119	3.46653	4.78742	4.48814	4.09543	1.88973	4.27823	3.52186	3.23444	3.90713	2.59990	3.82158	3.99014	4.59895	3.99252	4.00546	3.62670	3.69020	3.14438	1.30017	1.89715	133 w - - -
	2.68618	4.42225	2.77519	2.73123	3.46354	2.40513	3.72494	3.29354	2.67741	2.69355	4.24690	2.90347	2.73739	3.18146	2.89801	2.37887	2.77519	2.98518	4.58477	3.61503	
	0.09562	4.03368	2.61072	0.61958	0.77255	0.48576	0.95510														
120	1.88763	4.21098	3.27201	3.09659	4.35356	1.23225	4.25012	3.72221	3.24971	3.46334	4.33005	3.28566	3.70748	3.53687	3.54967	2.45003	2.77286	3.19259	5.68057	4.47898	134 g - - -
	2.68618	4.42225	2.77519	2.73123	3.46354	2.40513	3.72494	3.29354	2.67741	2.69355	4.24690	2.90347	2.73739	3.18146	2.89801	2.37887	2.77519	2.98518	4.58477	3.61503	
	0.02854	3.96660	4.68894	0.61958	0.77255	0.44587	1.02240														
121	1.00341	4.18942	3.47504	3.24680	4.32833	2.34285	4.31523	3.66096	3.32951	3.42609	4.28808	3.37436	3.73561	3.61384	3.61384	2.45764	2.76857	3.14727	5.67995	4.48994	135 a - - -
	2.68618	4.42225	2.77519	2.73123	3.46354	2.40513	3.72494	3.29354	2.67741	2.69355	4.24690	2.90347	2.73739	3.18146	2.89801	2.37887	2.77519	2.98518	4.58477	3.61503	
	0.02666	4.03368	4.75603	0.61958	0.77255	0.48576	0.95510														
122	2.33863	4.23760	3.50397	3.16391	4.01368	3.09020	4.17284	3.27515	3.13319	3.09951	4.03333	3.36191	3.79132	3.46986	3.43615	1.19915	2.78937	2.40199	5.45048	4.17799	136 s - - -
	2.68618	4.42225	2.77519	2.73123	3.46354	2.40513	3.72494	3.29354	2.67741	2.69355	4.24690	2.90347	2.73739	3.18146	2.89801	2.37887	2.77519	2.98518	4.58477	3.61503	
	0.02666	4.03368	4.75603	0.61958	0.77255	0.48576	0.95510														
123	3.65176	4.89365	4.61335	4.30020	1.42565	4.39309	3.47915	3.34766	4.14850	2.67875	3.94601	4.07047	4.70455	4.12574	4.19023	3.76326	3.87469	3.27134	3.62121	1.03705	137 y - - -
	2.68618	4.42225	2.77519	2.73123	3.46354	2.40513	3.72494	3.29354	2.67741	2.69355	4.24690	2.90347	2.73739	3.18146	2.89801	2.37887	2.77519	2.98518	4.58477	3.61503	
	0.02666	4.03368	4.75603	0.61958	0.77255	0.48576	0.95510														
124	3.49474	4.81514	4.49908	4.16896	0.92131	4.29487	3.64452	3.04603	4.02660	2.36461	3.68333	4.07813	4.63771	4.09136	4.11092	3.69496	3.73881	3.02501	3.81973	2.01123	138 f - - -
	2.68618	4.42225	2.77519	2.73123	3.46354	2.40513	3.72494	3.29354	2.67741	2.69355	4.24690	2.90347	2.73739	3.18146	2.89801	2.37887	2.77519	2.98518	4.58477	3.61503	
	0.02666	4.03368	4.75603	0.61958	0.77255	0.48576	0.95510														
125	2.08455	4.21411	3.68357	3.25316	3.88763	3.21810	4.19722	2.81970	3.18549	2.82757	3.80351	3.45622	3.86851	3.50623	3.48683	2.62468	1.50045	2.20475	5.37490	4.15891	139 t - - -
	2.68618	4.42225	2.77519	2.73123	3.46354	2.40513	3.72494	3.29354	2.67741	2.69355	4.24690	2.90347	2.73739	3.18146	2.89801	2.37887	2.77519	2.98518	4.58477	3.61503	
	0.02666	4.03368	4.75603	0.61958	0.77255	0.48576	0.95510														
126	3.09938	5.23515	2.56707	0.81610	4.60288	3.38409	3.98855	4.08925	2.89003	3.68170	4.65157	3.03581	3.99722	3.20272	3.31344	3.06710	3.40581	3.75755	5.74405	4.46989	140 e - - -
	2.68618	4.42225	2.77519	2.73123	3.46354	2.40513	3.72494	3.29354	2.67741	2.69355	4.24690	2.90347	2.73739	3.18146	2.89801	2.37887	2.77519	2.98518	4.58477	3.61503	
	0.02666	4.03368	4.75603	0.61958	0.77255	0.48576	0.95510														
127	3.01814	5.00291	3.01880	2.77892	4.19230	3.47838	3.93765	3.85058	2.58916	3.33659	4.36268	3.23903	4.03591	0.97244	2.86897	3.06036	3.31628	3.57104	5.43693	4.14251	141 q - - -
	2.68618	4.42225	2.77519	2.73123	3.46354	2.40513	3.72494	3.29354	2.67741	2.69355	4.24690	2.90347	2.73739	3.18146	2.89801	2.37887	2.77519	2.98518	4.58477	3.61503	
	0.02666	4.03368	4.75603	0.61958	0.77255	0.48576	0.95510														
128	3.17015	5.31440	0.71172	2.44939	4.71481	3.33942	4.08322	4.34124	3.19942	3.92193	4.90624	3.01284	4.00386	3.32453	3.73547	3.11617	3.51310	3.96414	5.85135	4.56456	142 d - - -
	2.68618	4.42225	2.77519	2.73123	3.46354	2.40513	3.72494	3.29354	2.67741	2.69355	4.24690	2.90347	2.73739	3.18146	2.89801	2.37887	2.77519	2.98518	4.58477	3.61503	
	0.02666	4.03368	4.75603	0.61958	0.77255	0.48576	0.95510														
129	2.53063	4.54715	2.85394	2.72988	4.42819	1.19437	4.08073	3.96845	3.02766	3.60138	4.45727	2.42915	3.81228	3.32296	3.40559	2.63943	2.97133	3.45989	5.70207	4.40964	143 g - - -
	2.68618	4.42225	2.77519	2.73123	3.46354	2.40513	3.72494	3.29354	2.67741	2.69355	4.24690	2.90347	2.73739	3.18146	2.89801	2.37887	2.77519	2.98518	4.58477	3.61503	
	0.02666	4.03368	4.75603	0.61958	0.77255	0.48576	0.95510														
130	2.29824	4.21223	3.55939	3.12806	3.92361	3.14831	4.10307	3.10109	3.07478	2.95560	3.85786	3.35604	3.80124	3.39692	3.39246	2.20008	1.51935	2.36453	5.34689	4.12328	144 t - - -
	2.68618	4.42225	2.77519	2.73123	3.46354	2.40513	3.72494	3.29354	2.67741	2.69355	4.24690	2.90347	2.73739	3.18146	2.89801	2.37887	2.77519	2.98518	4.58477	3.61503	
	0.02666	4.03368	4.75603	0.61958	0.77255	0.48576	0.95510														
131	3.61679	4.93173	4.34628	4.09412	2.84921	3.91802	4.11223	3.65007	3.81429	3.01594	4.27597	4.21130	4.44976	4.19354	3.91337	3.81884	3.92595	3.54480	0.61461	2.84332	145 W - - -
	2.68618	4.42225	2.77519	2.73123	3.46354	2.40513	3.72494	3.29354	2.67741	2.69355	4.24690	2.90347	2.73739	3.18146	2.89801	2.37887	2.77519	2.98518	4.58477	3.61503	
	0.02666	4.03368	4.75603	0.61958	0.77255	0.48576	0.95510														

132	3.18610	4.97021	3.29694	3.07575	3.40855	3.59320	0.84342	3.91399	2.91490	3.38647	4.44416	3.47748	4.15596	3.44485	3.16957	3.25612	3.49607	3.64618	4.87088	3.35543	146 h - - -
	2.68618	4.42225	2.77519	2.73123	3.46354	2.40513	3.72494	3.29354	2.67741	2.69355	4.24690	2.90347	2.73739	3.18146	2.89801	2.37887	2.77519	2.98518	4.58477	3.61503	
	0.02666	4.03368	4.75603	0.61958	0.77255	0.48576	0.95510														
133	2.64529	4.71326	3.06095	2.57326	3.95810	3.42491	3.69823	3.36819	2.42784	2.97801	3.22314	3.04972	3.86523	1.79932	2.79711	2.44355	2.89895	3.07827	5.26176	3.95280	147 q - - -
	2.68618	4.42225	2.77519	2.73123	3.46354	2.40513	3.72494	3.29354	2.67741	2.69355	4.24690	2.90347	2.73739	3.18146	2.89801	2.37887	2.77519	2.98518	4.58477	3.61503	
	0.02666	4.03368	4.75603	0.61958	0.77255	0.48576	0.95510														
134	2.35686	4.21731	3.56350	3.13835	3.91202	3.15820	4.10961	3.07506	3.07879	2.94093	3.85406	3.36537	3.81072	3.40680	3.39195	2.22174	1.49681	2.33414	5.34357	4.11562	148 t - - -
	2.68618	4.42225	2.77519	2.73123	3.46354	2.40513	3.72494	3.29354	2.67741	2.69355	4.24690	2.90347	2.73739	3.18146	2.89801	2.37887	2.77519	2.98518	4.58477	3.61503	
	0.02666	4.03368	4.75603	0.61958	0.77255	0.48576	0.95510														
135	2.51001	4.29254	4.44739	3.89458	3.42663	4.13407	4.54998	1.57704	3.79140	2.10464	2.77689	4.13352	4.46598	4.03252	3.99889	3.45924	3.14855	1.43218	5.18461	4.00056	149 v - - -
	2.68618	4.42225	2.77519	2.73123	3.46354	2.40513	3.72494	3.29354	2.67741	2.69355	4.24690	2.90347	2.73739	3.18146	2.89801	2.37887	2.77519	2.98518	4.58477	3.61503	
	0.02666	4.03368	4.75603	0.61958	0.77255	0.48576	0.95510														
136	3.11758	4.42869	4.83519	4.34296	3.57714	4.46922	5.05471	1.19433	4.22950	2.07071	3.38033	4.55813	4.78044	4.49223	4.42435	3.86142	3.38480	1.25260	5.55585	4.35242	150 i - - -
	2.68618	4.42225	2.77519	2.73123	3.46354	2.40513	3.72494	3.29354	2.67741	2.69355	4.24690	2.90347	2.73739	3.18146	2.89801	2.37887	2.77519	2.98518	4.58477	3.61503	
	0.02666	4.03368	4.75603	0.61958	0.77255	0.48576	0.95510														
137	3.19496	5.01753	3.70281	3.18361	4.41333	3.64851	3.95253	3.96851	2.31809	3.45525	4.45357	3.52980	4.14536	3.16690	0.75800	3.27280	3.45129	3.68607	5.43230	4.30818	151 r - - -
	2.68618	4.42225	2.77519	2.73123	3.46354	2.40513	3.72494	3.29354	2.67741	2.69355	4.24690	2.90347	2.73739	3.18146	2.89801	2.37887	2.77519	2.98518	4.58477	3.61503	
	0.02666	4.03368	4.75603	0.61958	0.77255	0.48576	0.95510														
138	3.17015	5.31440	0.71172	2.44939	4.71481	3.33942	4.08322	4.34124	3.19942	3.92193	4.90624	3.01284	4.00386	3.32453	3.73547	3.11617	3.51310	3.96414	5.85135	4.56456	152 d - - -
	2.68618	4.42225	2.77519	2.73123	3.46354	2.40513	3.72494	3.29354	2.67741	2.69355	4.24690	2.90347	2.73739	3.18146	2.89801	2.37887	2.77519	2.98518	4.58477	3.61503	
	0.02666	4.03368	4.75603	0.61958	0.77255	0.48576	0.95510														
139	2.50691	4.35134	3.57919	3.34222	4.11908	3.18440	4.34621	3.26619	3.28838	3.12388	4.16631	3.52330	3.90192	3.67573	3.53740	2.71028	0.92524	2.94357	5.54255	4.33660	153 t - - -
	2.68618	4.42225	2.77519	2.73123	3.46354	2.40513	3.72494	3.29354	2.67741	2.69355	4.24690	2.90347	2.73739	3.18146	2.89801	2.37887	2.77519	2.98518	4.58477	3.61503	
	0.02666	4.03368	4.75603	0.61958	0.77255	0.48576	0.95510														
140	2.89120	5.40647	1.35219	1.92124	4.71603	3.25291	3.75455	4.21554	2.72620	3.72725	4.56047	2.72634	2.95009	2.91329	3.29699	2.79851	3.17261	3.79690	5.89448	4.41804	154 d - - -
	2.68618	4.42225	2.77519	2.73123	3.46354	2.40513	3.72494	3.29354	2.67741	2.69355	4.24690	2.90347	2.73739	3.18146	2.89801	2.37887	2.77519	2.98518	4.58477	3.61503	
	0.02666	4.03368	4.75603	0.61958	0.77255	0.48576	0.95510														
141	3.47001	4.82537	4.45997	4.20753	0.78069	4.11698	3.95839	2.97664	4.11116	2.31068	3.68451	4.20413	4.57381	4.23529	4.19093	3.72996	3.77217	2.97875	4.16427	2.51296	155 f - - -
	2.68618	4.42225	2.77519	2.73123	3.46354	2.40513	3.72494	3.29354	2.67741	2.69355	4.24690	2.90347	2.73739	3.18146	2.89801	2.37887	2.77519	2.98518	4.58477	3.61503	
	0.02666	4.03368	4.75603	0.61958	0.77255	0.48576	0.95510														
142	2.50691	4.35134	3.57919	3.34222	4.11908	3.18440	4.34621	3.26619	3.28838	3.12388	4.16631	3.52330	3.90192	3.67573	3.53740	2.71028	0.92524	2.94357	5.54255	4.33660	156 t - - -
	2.68618	4.42225	2.77519	2.73123	3.46354	2.40513	3.72494	3.29354	2.67741	2.69355	4.24690	2.90347	2.73739	3.18146	2.89801	2.37887	2.77519	2.98518	4.58477	3.61503	
	0.02666	4.03368	4.75603	0.61958	0.77255	0.48576	0.95510														
143	2.00367	4.32510	3.30465	3.07209	4.25609	3.07649	4.20344	3.57730	3.13527	3.31320	4.23460	3.31880	1.27695	3.47670	3.44358	2.56467	2.85983	3.14530	5.61150	4.38614	157 p - - -
	2.68618	4.42225	2.77519	2.73123	3.46354	2.40513	3.72494	3.29354	2.67741	2.69355	4.24690	2.90347	2.73739	3.18146	2.89801	2.37887	2.77519	2.98518	4.58477	3.61503	
	0.02666	4.03368	4.75603	0.61958	0.77255	0.48576	0.95510														
144	2.44309	4.46826	2.94544	2.73966	4.29064	3.08882	4.01014	3.81762	2.91262	3.46120	4.30390	2.50466	3.77533	3.24078	3.28609	1.22137	2.87265	3.32893	5.60706	4.28148	158 s - - -
	2.68618	4.42225	2.77519	2.73123	3.46354	2.40513	3.72494	3.29354	2.67741	2.69355	4.24690	2.90347	2.73739	3.18146	2.89801	2.37887	2.77519	2.98518	4.58477	3.61503	
	0.02666	4.03368	4.75603	0.61958	0.77255	0.48576	0.95510														
145	3.18610	4.97021	3.29694	3.07575	3.40855	3.59320	0.84342	3.91399	2.91490	3.38647	4.44416	3.47748	4.15596	3.44485	3.16957	3.25612	3.49607	3.64618	4.87088	3.35543	159 h - - -
	2.68618	4.42225	2.77519	2.73123	3.46354	2.40513	3.72494	3.29354	2.67741	2.69355	4.24690	2.90347	2.73739	3.18146	2.89801	2.37887	2.77519	2.98518	4.58477	3.61503	
	0.02666	4.03368	4.75603	0.61958	0.77255	0.48576	0.95510														
146	3.11700	4.48285	4.67995	4.23530	3.47788	4.33737	4.94053	0.98686	4.07131	1.98289	3.33263	4.45128	4.70544	4.37349	4.26177	3.75885	3.40306	1.69575	5.45735	4.22217	160 i - - -
	2.68618	4.42225	2.77519	2.73123	3.46354	2.40513	3.72494	3.29354	2.67741	2.69355	4.24690	2.90347	2.73739	3.18146	2.89801	2.37887	2.77519	2.98518	4.58477	3.61503	
	0.02666	4.03368	4.75603	0.61958	0.77255	0.48576	0.95510														
147	3.16598	4.49674	4.75518	4.22905	2.61793	4.42333	4.68356	1.17367	4.10211	1.70346	3.11273	4.43393	4.68246	4.27039	4.25049	3.77796	3.40587	1.95266	5.05487	3.75779	161 i - - -
	2.68618	4.42225	2.77519	2.73123	3.46354	2.40513	3.72494	3.29354	2.67741	2.69355	4.24690	2.90347	2.73739	3.18146	2.89801	2.37887	2.77519	2.98518	4.58477	3.61503	
	0.02666	4.03368	4.75603	0.61958	0.77255	0.48576	0.95510														
148	2.91015	5.31212	2.32401	1.19111	4.63198	3.28148	3.77815	4.13482	2.67024	3.67582	4.53495	2.37597	3.87598	2.94847	3.15433	2.83938	3.19683	3.74012	5.81402	4.37957	162 e - - -
	2.68618	4.42225	2.77519	2.73123	3.46354	2.40513	3.72494	3.29354	2.67741	2.69355	4.24690	2.90347	2.73739	3.18146	2.89801	2.37887	2.77519	2.98518	4.58477	3.61503	
	0.02666	4.03368	4.75603	0.61958	0.77255	0.48576	0.95510														
149	3.47001	4.82537	4.45997	4.20753	0.78069	4.11698	3.95839	2.97664	4.11116	2.31068	3.68451	4.20413	4.57381	4.23529	4.19093	3.72996	3.77217	2.97875	4.16427	2.51296	163 f - - -
	2.68618	4.42225	2.77519	2.73123	3.46354	2.40513	3.72494	3.29354	2.67741	2.69355	4.24690	2.90347	2.73739	3.18146	2.89801	2.37887	2.77519	2.98518	4.58477	3.61503	
	0.02666	4.03368	4.75603	0.61958	0.77255	0.48576	0.95510														
150	3.48747	4.91087	4.07975	3.84179	2.31656	4.02068	3.70522	3.47503	3.69483	2.88668	4.13379	3.92717	4.49191	3.96171	3.84439	3.60710	3.77558	3.35354	3.96564	0.73471	164 y - - -
	2.68618	4.42225	2.77519	2.73123	3.46354	2.40513	3.72494	3.29354	2.67741	2.69355	4.24690	2.90347	2.73739	3.18146	2.89801	2.37887	2.77519	2.98518	4.58477	3.61503	
	0.02666	4.03368	4.75603	0.61958	0.77255	0.48576	0.95510														
151	2.88134	4.38147	4.11734	3.54613	2.64338	3.96704	4.20271	2.43645	3.42686	1.30181	2.54845	3.84969	4.27968	3.08040	3.65585	3.25520	3.10682	2.44367	4.84757	3.64262	165 l - - -
	2.68618	4.42225	2.77519	2.73123	3.46354	2.40513	3.72494	3.29354	2.67741	2.69355	4.24690	2.90347	2.73739	3.18146	2.89801	2.37887	2.77519	2.98518	4.58477	3.61503	
	0.02666	4.03368	4.75603	0.61958	0.77255	0.48576	0.95510														
152	2.44309	4.46826	2.94544	2.73966	4.29064	3.08882	4.01014	3.81762	2.91262	3.46120	4.30390	2.50466	3.77533	3.24078	3.28609	1.22137	2.87265	3.32893	5.60706	4.28148	166 s - - -
	2.68618	4.42225	2.77519	2.73123	3.46354	2.40513	3.72494	3.29354	2.67741	2.69355	4.24690	2.90347	2.73739	3.18146	2.89801	2.37887	2.77519	2.98518	4.58477	3.61503	
	0.02666	4.03368	4.75603	0.61958	0.77255	0.48576	0.95510														
153	3.71584	4.90327	4.73458	4.43163	1.62507	4.44743	3.42692	3.46414	4.25878	2.79672	4.03257	4.09508	4.73931	4.17125	4.25325	3.80469	3.92691	3.36163	2.64839	0.96804	167 y - - -
	2.68618	4.42225	2.77519	2.73123	3.46354	2.40513	3.72494	3.29354	2.67741	2.69355	4.24690	2.90347	2.73739	3.18146	2.89801	2.37887	2.77519	2.98518	4.58477	3.61503	
	0.02666	4.03368	4.75603	0.61958	0.77255	0.48576	0.95510														
154	2.97886	4.70647	3.60392	3.46821	4.44510	3.38498	4.53021	4.03474	3.53984	3.64297	4.70148	3.75741	0.57586	3.92368	3.76486	3.15199	3.43364	3.66126	5.58479	4.56881	168 P - - -

```
        2.68618  4.42225  2.77519  2.73123  3.46354  2.40513  3.72494  3.29354  2.67741  2.69355  4.24690  2.90347  2.73739  3.18146  2.89801  2.37887  2.77519  2.98518  4.58477  3.61503
        0.02666  4.03368  4.75603  0.61958  0.77255  0.48576  0.95510
  155   3.18240  4.49509  4.82481  4.28535  3.35381  4.51669  4.93214  1.12525  4.14913  1.67104  2.95202  4.53452  4.75830  4.36146  4.32248  3.87628  3.42440  1.74960  5.36676  4.22103    169 i - - -
        2.68618  4.42225  2.77519  2.73123  3.46354  2.40513  3.72494  3.29354  2.67741  2.69355  4.24690  2.90347  2.73739  3.18146  2.89801  2.37887  2.77519  2.98518  4.58477  3.61503
        0.02666  4.03368  4.75603  0.61958  0.77255  0.48576  0.95510
  156   3.60758  4.89159  4.51262  4.18789  1.71429  4.34382  3.49443  3.33464  4.04243  2.68101  3.94587  4.02975  4.67075  4.07159  4.12099  3.71838  3.83705  3.25376  3.65270  0.90642    170 y - - -
        2.68618  4.42225  2.77519  2.73123  3.46354  2.40513  3.72494  3.29354  2.67741  2.69355  4.24690  2.90347  2.73739  3.18146  2.89801  2.37887  2.77519  2.98518  4.58477  3.61503
        0.02666  4.03368  4.75603  0.61958  0.77255  0.48576  0.95510
  157   2.75725  4.27539  4.14152  3.64507  3.49410  3.82487  4.38798  1.30855  3.52191  2.29009  3.41855  3.88356  4.27628  3.82624  3.76064  2.72791  3.05070  1.80246  5.17007  3.94123    171 i - - -
        2.68618  4.42225  2.77519  2.73123  3.46354  2.40513  3.72494  3.29354  2.67741  2.69355  4.24690  2.90347  2.73739  3.18146  2.89801  2.37887  2.77519  2.98518  4.58477  3.61503
        0.02666  4.03368  4.75603  0.61958  0.77255  0.48576  0.95510
  158   2.74539  4.30038  3.90623  3.41143  3.47609  3.78842  4.22536  1.41238  3.29853  2.34609  3.43057  3.02355  4.21233  3.62923  3.57093  3.12223  3.01701  1.87234  5.09685  3.84814    172 i - - -
        2.68618  4.42225  2.77519  2.73123  3.46354  2.40513  3.72494  3.29354  2.67741  2.69355  4.24690  2.90347  2.73739  3.18146  2.89801  2.37887  2.77519  2.98518  4.58477  3.61503
        0.02666  4.03368  4.75603  0.61958  0.77255  0.48576  0.95510
  159   2.55698  3.82702  4.05607  3.47673  2.80395  3.75853  4.07193  2.16248  3.36941  1.87279  2.95768  3.71664  4.11832  3.59876  3.57393  3.04732  2.27494  2.17356  4.76844  3.57909    173 l - - -
        2.68618  4.42225  2.77519  2.73123  3.46354  2.40513  3.72494  3.29354  2.67741  2.69355  4.24690  2.90347  2.73739  3.18146  2.89801  2.37887  2.77519  2.98518  4.58477  3.61503
        0.02666  4.03368  4.75603  0.61958  0.77255  0.48576  0.95510
  160   1.96825  4.25463  3.32326  3.16477  4.42896  1.08524  4.32194  3.80936  3.33133  3.54709  4.41393  3.34298  3.75329  3.61393  3.62449  2.49653  2.82479  3.26413  5.74940  4.55547    174 g - - -
        2.68618  4.42225  2.77519  2.73123  3.46354  2.40513  3.72494  3.29354  2.67741  2.69355  4.24690  2.90347  2.73739  3.18146  2.89801  2.37887  2.77519  2.98518  4.58477  3.61503
        0.02666  4.03368  4.75603  0.61958  0.77255  0.48576  0.95510
  161   2.55871  4.12734  3.75814  3.18570  2.95841  2.70984  3.90310  2.42143  3.10018  2.31870  3.24275  3.48903  3.98751  3.36509  3.36503  2.80582  2.73003  1.93502  4.28191  3.51450    175 v - - -
        2.68618  4.42225  2.77519  2.73123  3.46354  2.40513  3.72494  3.29354  2.67741  2.69355  4.24690  2.90347  2.73739  3.18146  2.89801  2.37887  2.77519  2.98518  4.58477  3.61503
        0.02666  4.03368  4.75603  0.61958  0.77255  0.48576  0.95510
  162   1.51519  2.88750  3.77748  3.37216  4.09197  1.86298  4.26524  3.46227  3.32269  3.19026  4.03397  3.41910  3.70714  3.58956  3.59766  2.24489  2.67551  2.98978  5.48679  4.30212    176 a - - -
        2.68618  4.42225  2.77519  2.73123  3.46354  2.40513  3.72494  3.29354  2.67741  2.69355  4.24690  2.90347  2.73739  3.18146  2.89801  2.37887  2.77519  2.98518  4.58477  3.61503
        0.02666  4.03368  4.75603  0.61958  0.77255  0.48576  0.95510
  163   1.34632  3.68859  3.67533  3.27397  4.20111  2.22353  4.23634  3.59650  3.24476  3.29824  4.12402  3.36776  3.69387  3.52342  3.54822  1.92656  2.67755  3.08058  5.56520  4.36985    177 a - - -
        2.68618  4.42225  2.77519  2.73123  3.46354  2.40513  3.72494  3.29354  2.67741  2.69355  4.24690  2.90347  2.73739  3.18146  2.89801  2.37887  2.77519  2.98518  4.58477  3.61503
        0.02666  4.03368  4.75603  0.61958  0.77255  0.48576  0.95510
  164   2.95284  4.33764  4.41505  3.89041  1.60223  4.00695  3.81081  2.66544  3.74122  1.92441  2.88445  3.92978  4.33510  3.85833  3.84324  3.31753  3.17853  2.56920  3.07951  2.41284    178 f - - -
        2.68618  4.42225  2.77519  2.73123  3.46354  2.40513  3.72494  3.29354  2.67741  2.69355  4.24690  2.90347  2.73739  3.18146  2.89801  2.37887  2.77519  2.98518  4.58477  3.61503
        0.02666  4.03368  4.75603  0.61958  0.77255  0.48576  0.95510
  165   3.13432  4.48372  4.75058  4.17106  2.63025  4.36158  4.65514  2.02580  4.05049  1.25932  2.03312  4.38836  4.58717  4.14945  4.16081  3.68649  3.35489  2.09778  5.03008  3.93843    179 l - - -
        2.68618  4.42225  2.77519  2.73123  3.46354  2.40513  3.72494  3.29354  2.67741  2.69355  4.24690  2.90347  2.73739  3.18146  2.89801  2.37887  2.77519  2.98518  4.58477  3.61503
        0.02666  4.03368  4.75603  0.61958  0.77255  0.48576  0.95510
  166   3.28853  4.69058  4.28342  3.83020  2.10303  4.14329  3.26571  3.21932  3.64322  2.26783  3.80905  3.84861  4.47363  3.81226  3.80188  3.46030  3.50903  3.06932  3.26068  1.11864    180 y - - -
        2.68618  4.42225  2.77519  2.73123  3.46354  2.40513  3.72494  3.29354  2.67741  2.69355  4.24690  2.90347  2.73739  3.18146  2.89801  2.37887  2.77519  2.98518  4.58477  3.61503
        0.02666  4.03368  4.75603  0.61958  0.77255  0.48576  0.95510
  167   1.09243  4.15633  3.55427  3.25593  4.22649  2.81317  4.27143  3.47174  3.26593  3.28739  4.15644  3.37435  3.73066  3.56565  3.55234  2.44081  2.26706  3.00969  5.60986  4.41129    181 a - - -
        2.68618  4.42225  2.77519  2.73123  3.46354  2.40513  3.72494  3.29354  2.67741  2.69355  4.24690  2.90347  2.73739  3.18146  2.89801  2.37887  2.77519  2.98518  4.58477  3.61503
        0.02666  4.03368  4.75603  0.61958  0.77255  0.48576  0.95510
  168   2.74127  4.91449  3.17586  2.56114  4.20060  3.51746  3.10089  3.28658  1.78285  3.15532  3.68418  3.04078  3.88974  2.74883  1.97437  2.76470  2.94880  3.28204  5.33210  4.05345    182 k - - -
        2.68618  4.42225  2.77519  2.73123  3.46354  2.40513  3.72494  3.29354  2.67741  2.69355  4.24690  2.90347  2.73739  3.18146  2.89801  2.37887  2.77519  2.98518  4.58477  3.61503
        0.02666  4.03368  4.75603  0.61958  0.77255  0.48576  0.95510
  169   2.50691  4.35134  3.57919  3.34222  4.11908  3.18440  4.34621  3.26619  3.28838  3.12388  4.16631  3.52330  3.90192  3.67573  3.53740  2.71028  0.92524  2.94357  5.54255  4.33660    183 t - - -
        2.68618  4.42225  2.77519  2.73123  3.46354  2.40513  3.72494  3.29354  2.67741  2.69355  4.24690  2.90347  2.73739  3.18146  2.89801  2.37887  2.77519  2.98518  4.58477  3.61503
        0.02666  4.03368  4.75603  0.61958  0.77255  0.48576  0.95510
  170   3.19496  5.01753  3.70281  3.18361  4.41333  3.64851  3.95253  3.96851  2.31809  3.45525  4.45357  3.52980  4.14536  3.16690  0.75800  3.27280  3.45129  3.68607  5.43230  4.30818    184 r - - -
        2.68618  4.42225  2.77519  2.73123  3.46354  2.40513  3.72494  3.29354  2.67741  2.69355  4.24690  2.90347  2.73739  3.18146  2.89801  2.37887  2.77519  2.98518  4.58477  3.61503
        0.02666  4.03368  4.75603  0.61958  0.77255  0.48576  0.95510
  171   3.30840  4.65891  4.76797  4.25084  3.13053  4.51435  4.84193  1.88055  4.05377  0.88628  2.96846  4.52105  4.74502  4.26133  4.20519  3.89542  3.54774  2.16706  5.20038  4.04646    185 l - - -
        2.68618  4.42225  2.77519  2.73123  3.46354  2.40513  3.72494  3.29354  2.67741  2.69355  4.24690  2.90347  2.73739  3.18146  2.89801  2.37887  2.77519  2.98518  4.58477  3.61503
        0.02666  4.03368  4.75603  0.61958  0.77255  0.48576  0.95510
  172   2.97886  4.70647  3.60392  3.46821  4.44510  3.38498  4.53021  4.03474  3.53984  3.64297  4.70148  3.75741  0.57586  3.92368  3.76486  3.15199  3.43364  3.66126  5.58479  4.56881    186 P - - -
        2.68618  4.42225  2.77519  2.73123  3.46354  2.40513  3.72494  3.29354  2.67741  2.69355  4.24690  2.90347  2.73739  3.18146  2.89801  2.37887  2.77519  2.98518  4.58477  3.61503
        0.02666  4.03368  4.75603  0.61958  0.77255  0.48576  0.95510
  173   2.50864  4.17711  3.62040  3.04795  3.12189  3.58151  3.73491  2.64540  2.97723  2.13723  3.07515  3.39767  3.95712  3.02145  3.27886  2.80619  2.48608  2.26229  4.76554  2.95802    187 l - - -
        2.68618  4.42225  2.77519  2.73123  3.46354  2.40513  3.72494  3.29354  2.67741  2.69355  4.24690  2.90347  2.73739  3.18146  2.89801  2.37887  2.77519  2.98518  4.58477  3.61503
        0.02666  4.03368  4.75603  0.61958  0.77255  0.48576  0.95510
  174   3.11211  4.50081  4.38380  3.89628  1.57017  4.10146  3.72931  2.79243  3.75896  2.22676  2.73910  3.93818  4.42440  3.87816  3.88911  3.41735  3.33786  2.71428  4.00065  1.71225    188 f - - -
        2.68618  4.42227  2.77521  2.73125  3.46356  2.40509  3.72496  3.29356  2.67743  2.69357  4.24692  2.90343  2.73741  3.18137  2.89803  2.37889  2.77516  2.98520  4.58479  3.61493
        0.09128  2.54287  4.75603  0.68179  0.70464  0.48576  0.95510
  175   1.95704  4.74629  2.90836  2.45561  4.07358  2.92266  3.50684  3.48784  2.40425  3.09581  3.92007  2.95872  3.81036  2.53000  2.90623  2.42779  2.80039  3.15394  5.33905  4.01637    192 a - - -
        2.68618  4.42225  2.77519  2.73123  3.46354  2.40513  3.72494  3.29354  2.67741  2.69355  4.24690  2.90347  2.73739  3.18146  2.89801  2.37887  2.77519  2.98518  4.58477  3.61503
        0.02666  4.03368  4.75603  0.61958  0.77255  0.48576  0.95510
  176   2.88322  5.15840  3.11826  2.42507  4.54806  3.55085  3.25706  3.93895  1.40504  3.40150  4.22357  3.03868  3.93575  2.45202  2.19323  2.85993  3.07555  3.58379  5.48712  4.23010    193 k - - -
        2.68618  4.42225  2.77519  2.73123  3.46354  2.40513  3.72494  3.29354  2.67741  2.69355  4.24690  2.90347  2.73739  3.18146  2.89801  2.37887  2.77519  2.98518  4.58477  3.61503
```

	0.02666	4.03368	4.75603	0.61958	0.77255	0.48576	0.95510														
177	2.28865	4.66457	2.95057	2.56269	4.18635	1.88302	3.76578	3.59436	2.25721	3.20587	4.03749	3.02210	3.81532	2.94275	2.78742	2.55461	2.88128	3.22307	5.43382	4.13611	194 g - - -
	2.68618	4.42225	2.77519	2.73123	3.46354	2.40513	3.72494	3.29354	2.67741	2.69355	4.24690	2.90347	2.73739	3.18146	2.89801	2.37887	2.77519	2.98518	4.58477	3.61503	
	0.02666	4.03368	4.75603	0.61958	0.77255	0.48576	0.95510														
178	2.65330	4.11097	4.09573	3.52678	2.50954	3.73478	3.51038	2.17554	3.39908	1.97709	3.22233	3.48145	4.09776	3.59811	3.56433	3.02594	2.88409	2.37436	3.39998	2.61919	195 l - - -
	2.68618	4.42225	2.77519	2.73123	3.46354	2.40513	3.72494	3.29354	2.67741	2.69355	4.24690	2.90347	2.73739	3.18146	2.89801	2.37887	2.77519	2.98518	4.58477	3.61503	
	0.02666	4.03368	4.75603	0.61958	0.77255	0.48576	0.95510														
179	2.59754	4.55325	3.13957	2.70300	3.67938	3.38003	3.28735	3.28318	2.62408	2.56280	3.82161	3.13355	3.87454	3.02965	2.98535	1.61208	2.88966	2.99027	5.08755	3.71410	196 s - - -
	2.68618	4.42225	2.77519	2.73123	3.46354	2.40513	3.72494	3.29354	2.67741	2.69355	4.24690	2.90347	2.73739	3.18146	2.89801	2.37887	2.77519	2.98518	4.58477	3.61503	
	0.02666	4.03368	4.75603	0.61958	0.77255	0.48576	0.95510														
180	3.21716	4.54615	4.81033	4.26501	3.23610	4.50455	4.88341	1.75152	4.13169	1.08077	3.03495	4.52129	4.73333	4.30452	4.28963	3.85955	3.45430	1.76047	5.27683	4.16340	197 l - - -
	2.68618	4.42225	2.77519	2.73123	3.46354	2.40513	3.72494	3.29354	2.67741	2.69355	4.24690	2.90347	2.73739	3.18146	2.89801	2.37887	2.77519	2.98518	4.58477	3.61503	
	0.02666	4.03368	4.75603	0.61958	0.77255	0.48576	0.95510														
181	2.16862	4.56596	3.10879	2.61501	3.88940	3.37115	3.74058	3.25885	2.56242	2.91102	3.42574	3.07417	2.27478	2.65536	2.96034	2.65893	2.83339	2.96328	5.22756	3.94118	198 a - - -
	2.68618	4.42225	2.77519	2.73123	3.46354	2.40513	3.72494	3.29354	2.67741	2.69355	4.24690	2.90347	2.73739	3.18146	2.89801	2.37887	2.77519	2.98518	4.58477	3.61503	
	0.02666	4.03368	4.75603	0.61958	0.77255	0.48576	0.95510														
182	3.24638	4.61360	4.43032	3.97245	1.91173	4.19185	3.67973	2.87627	3.83162	1.75402	3.50455	3.97919	4.50502	3.93332	3.95664	3.51572	3.47004	2.81577	3.91305	1.41897	199 y - - -
	2.68618	4.42225	2.77519	2.73123	3.46354	2.40513	3.72494	3.29354	2.67741	2.69355	4.24690	2.90347	2.73739	3.18146	2.89801	2.37887	2.77519	2.98518	4.58477	3.61503	
	0.02666	4.03368	4.75603	0.61958	0.77255	0.48576	0.95510														
183	2.47743	4.25444	3.99477	3.43139	3.31790	3.81522	4.16957	2.27277	3.33175	1.61891	2.85544	3.73146	4.18656	3.60121	3.58912	3.11075	2.60194	1.96526	4.95766	3.77014	200 l - - -
	2.68618	4.42225	2.77519	2.73123	3.46354	2.40513	3.72494	3.29354	2.67741	2.69355	4.24690	2.90347	2.73739	3.18146	2.89801	2.37887	2.77519	2.98518	4.58477	3.61503	
	0.02666	4.03368	4.75603	0.61958	0.77255	0.48576	0.95510														
184	3.10741	4.43333	4.77380	4.21349	3.26799	4.40750	4.78374	1.65412	4.10034	1.48893	2.50588	4.43637	4.65368	4.26010	4.24591	3.74571	3.34060	1.53387	5.21793	4.10268	201 l - - -
	2.68618	4.42225	2.77519	2.73123	3.46354	2.40513	3.72494	3.29354	2.67741	2.69355	4.24690	2.90347	2.73739	3.18146	2.89801	2.37887	2.77519	2.98518	4.58477	3.61503	
	0.02666	4.03368	4.75603	0.61958	0.77255	0.48576	0.95510														
185	2.25547	4.31713	3.41748	2.59966	3.33255	3.53816	3.81591	2.73498	2.81551	2.18357	3.43144	3.27483	3.92970	3.12907	3.17665	2.72803	2.62441	2.25178	4.92946	3.68970	202 l - - -
	2.68618	4.42225	2.77519	2.73123	3.46354	2.40513	3.72494	3.29354	2.67741	2.69355	4.24690	2.90347	2.73739	3.18146	2.89801	2.37887	2.77519	2.98518	4.58477	3.61503	
	0.02666	4.03368	4.75603	0.61958	0.77255	0.48576	0.95510														
186	2.43036	4.12119	3.91403	3.34658	2.95950	3.65630	3.99041	2.44262	3.23981	2.26837	3.23473	3.60485	4.04781	3.49572	3.47127	2.86588	2.75007	1.58930	4.20687	3.51965	203 v - - -
	2.68618	4.42225	2.77519	2.73123	3.46354	2.40513	3.72494	3.29354	2.67741	2.69355	4.24690	2.90347	2.73739	3.18146	2.89801	2.37887	2.77519	2.98518	4.58477	3.61503	
	0.02666	4.03368	4.75603	0.61958	0.77255	0.48576	0.95510														
187	2.30023	4.15669	3.80402	3.24149	2.99640	2.97561	3.98808	2.17143	3.16317	2.34343	3.29602	3.54195	4.02971	3.43095	3.43469	2.91688	2.73544	1.90346	4.83714	3.62728	204 v - - -
	2.68618	4.42225	2.77519	2.73123	3.46354	2.40513	3.72494	3.29354	2.67741	2.69355	4.24690	2.90347	2.73739	3.18146	2.89801	2.37887	2.77519	2.98518	4.58477	3.61503	
	0.02666	4.03368	4.75603	0.61958	0.77255	0.48576	0.95510														
188	2.49679	4.37120	3.32383	2.88829	3.62513	1.70234	3.87865	3.22022	2.83500	2.89363	3.77891	3.24221	3.85282	3.19183	3.17750	2.37688	2.83612	2.90984	3.39801	3.71483	205 g - - -
	2.68618	4.42225	2.77519	2.73123	3.46354	2.40513	3.72494	3.29354	2.67741	2.69355	4.24690	2.90347	2.73739	3.18146	2.89801	2.37887	2.77519	2.98518	4.58477	3.61503	
	0.02666	4.03368	4.75603	0.61958	0.77255	0.48576	0.95510														
189	2.97886	4.70647	3.60392	3.46821	4.44510	3.38498	4.53021	4.03474	3.53984	3.64297	4.70148	3.75741	0.57586	3.92368	3.76486	3.15199	3.43364	3.66126	5.58479	4.56881	206 P - - -
	2.68618	4.42225	2.77519	2.73123	3.46354	2.40513	3.72494	3.29354	2.67741	2.69355	4.24690	2.90347	2.73739	3.18146	2.89801	2.37887	2.77519	2.98518	4.58477	3.61503	
	0.02666	4.03368	4.75603	0.61958	0.77255	0.48576	0.95510														
190	2.78601	4.39908	4.44011	3.89280	1.69264	4.13016	4.31965	2.01857	3.76750	1.66170	3.08222	4.09991	4.43820	3.94976	3.93469	3.45102	3.22487	2.21124	4.77491	3.43778	207 l - - -
	2.68618	4.42225	2.77519	2.73123	3.46354	2.40513	3.72494	3.29354	2.67741	2.69355	4.24690	2.90347	2.73739	3.18146	2.89801	2.37887	2.77519	2.98518	4.58477	3.61503	
	0.02666	4.03368	4.75603	0.61958	0.77255	0.48576	0.95510														
191	3.10282	4.62768	4.22590	3.83428	3.28796	3.95614	4.53220	2.48008	3.59269	1.86074	1.09964	4.09103	4.42003	3.96536	3.77853	3.46749	3.41256	2.50432	5.14866	3.92331	208 m - - -
	2.68618	4.42225	2.77519	2.73123	3.46354	2.40513	3.72494	3.29354	2.67741	2.69355	4.24690	2.90347	2.73739	3.18146	2.89801	2.37887	2.77519	2.98518	4.58477	3.61503	
	0.02666	4.03368	4.75603	0.61958	0.77255	0.48576	0.95510														
192	3.15515	4.49729	4.71145	4.20910	2.60803	4.38080	4.65923	1.13516	4.08457	1.79741	3.15468	4.40707	4.66793	4.26636	4.23819	3.75148	3.40390	1.94953	5.03763	3.70824	209 i - - -
	2.68618	4.42225	2.77519	2.73123	3.46354	2.40513	3.72494	3.29354	2.67741	2.69355	4.24690	2.90347	2.73739	3.18146	2.89801	2.37887	2.77519	2.98518	4.58477	3.61503	
	0.02666	4.03368	4.75603	0.61958	0.77255	0.48576	0.95510														
193	3.17423	4.50779	4.80389	4.23746	2.29758	4.41816	4.67881	1.84214	4.11511	1.16694	2.93236	4.44351	4.63994	4.21501	4.22460	3.75250	3.39807	2.05797	5.02109	3.85163	210 l - - -
	2.68618	4.42225	2.77519	2.73123	3.46354	2.40513	3.72494	3.29354	2.67741	2.69355	4.24690	2.90347	2.73739	3.18146	2.89801	2.37887	2.77519	2.98518	4.58477	3.61503	
	0.02666	4.03368	4.75603	0.61958	0.77255	0.48576	0.95510														
194	2.50014	4.34409	3.50915	3.15031	3.86605	3.26587	4.15580	2.99384	3.09307	2.82046	3.86040	3.41987	1.49908	3.45675	3.39637	2.70292	2.90422	2.14420	5.35874	4.10072	211 p - - -
	2.68618	4.42225	2.77519	2.73123	3.46354	2.40513	3.72494	3.29354	2.67741	2.69355	4.24690	2.90347	2.73739	3.18146	2.89801	2.37887	2.77519	2.98518	4.58477	3.61503	
	0.02666	4.03368	4.75603	0.61958	0.77255	0.48576	0.95510														
195	2.59890	4.66049	2.91517	2.57237	3.99984	3.30418	3.78526	3.47711	2.61382	2.67730	4.00574	1.71924	3.84032	2.99326	3.00874	2.34909	2.91042	3.14112	5.33600	3.98207	212 n - - -
	2.68618	4.42225	2.77519	2.73123	3.46354	2.40513	3.72494	3.29354	2.67741	2.69355	4.24690	2.90347	2.73739	3.18146	2.89801	2.37887	2.77519	2.98518	4.58477	3.61503	
	0.02666	4.03368	4.75603	0.61958	0.77255	0.48576	0.95510														
196	3.07706	4.42111	4.72980	4.24585	3.58846	4.37199	4.97960	1.56354	4.12403	2.11260	3.40976	4.46537	4.72020	4.40904	4.33283	3.76639	3.35392	0.99172	5.53658	4.31465	213 v - - -
	2.68618	4.42225	2.77519	2.73123	3.46354	2.40513	3.72494	3.29354	2.67741	2.69355	4.24690	2.90347	2.73739	3.18146	2.89801	2.37887	2.77519	2.98518	4.58477	3.61503	
	0.02666	4.03368	4.75603	0.61958	0.77255	0.48576	0.95510														
197	2.03530	4.26823	3.31155	3.16251	4.43538	1.04707	4.32702	3.82206	3.33628	3.55874	4.43083	3.34669	3.76131	3.62121	3.62773	2.51094	2.84035	3.27840	5.75133	4.55885	214 g - - -
	2.68618	4.42225	2.77519	2.73123	3.46354	2.40513	3.72494	3.29354	2.67741	2.69355	4.24690	2.90347	2.73739	3.18146	2.89801	2.37887	2.77519	2.98518	4.58477	3.61503	
	0.02666	4.03368	4.75603	0.61958	0.77255	0.48576	0.95510														
198	2.80995	4.40056	3.77106	2.78453	2.70557	3.81473	4.04232	2.51717	3.16930	1.37798	3.21839	3.61805	4.17586	3.47117	3.45348	3.11217	3.04463	2.43729	4.83570	3.53675	215 l - - -
	2.68618	4.42225	2.77519	2.73123	3.46354	2.40513	3.72494	3.29354	2.67741	2.69355	4.24690	2.90347	2.73739	3.18146	2.89801	2.37887	2.77519	2.98518	4.58477	3.61503	
	0.02666	4.03368	4.75603	0.61958	0.77255	0.48576	0.95510														

```
199   2.86470  4.86179  2.80974  2.71294  4.25143  3.29821  4.06248  3.99953  2.98136  3.62880  4.58520  0.90139  3.95921  3.33781  3.33953  2.92718  3.25330  3.60527  5.53900  4.18997    216 n - - -
      2.68618  4.42225  2.77519  2.73123  3.46354  2.40513  3.72494  3.29354  2.67741  2.69355  4.24690  2.90347  2.73739  3.18146  2.89801  2.37887  2.77519  2.98518  4.58477  3.61503
      0.02666  4.03368  4.75603  0.61958  0.77255  0.48576  0.95510
200   3.09938  5.23515  2.56707  0.81610  4.60288  3.38409  3.98855  4.08925  2.89003  3.68170  4.65157  3.03581  3.99722  3.20272  3.31344  3.06710  3.40581  3.75755  5.74405  4.46989    217 e - - -
      2.68618  4.42225  2.77519  2.73123  3.46354  2.40513  3.72494  3.29354  2.67741  2.69355  4.24690  2.90347  2.73739  3.18146  2.89801  2.37887  2.77519  2.98518  4.58477  3.61503
      0.02666  4.03368  4.75603  0.61958  0.77255  0.48576  0.95510
201   3.45215  4.78433  4.49911  4.12156  1.87272  4.23271  3.62110  3.15280  3.92249  2.48779  3.75413  4.04037  4.58549  4.03524  4.01720  3.64307  3.69256  3.09647  1.24431  2.11433    218 w - - -
      2.68618  4.42225  2.77519  2.73123  3.46354  2.40513  3.72494  3.29354  2.67741  2.69355  4.24690  2.90347  2.73739  3.18146  2.89801  2.37887  2.77519  2.98518  4.58477  3.61503
      0.02666  4.03368  4.75603  0.61958  0.77255  0.48576  0.95510
202   2.87735  4.63403  3.58392  3.50518  4.63929  0.51867  4.61309  4.28057  3.70822  3.91370  4.89265  3.74860  4.03020  4.01983  3.92413  3.05432  3.37115  3.77778  5.68928  4.74753    219 G - - -
      2.68618  4.42225  2.77519  2.73123  3.46354  2.40513  3.72494  3.29354  2.67741  2.69355  4.24690  2.90347  2.73739  3.18146  2.89801  2.37887  2.77519  2.98518  4.58477  3.61503
      0.02666  4.03368  4.75603  0.61958  0.77255  0.48576  0.95510
203   3.18610  4.97021  3.29694  3.07575  3.40855  3.59320  0.84342  3.91399  2.91490  3.38647  4.44416  3.47748  4.15596  3.44485  3.16957  3.25612  3.49607  3.64618  4.87088  3.35543    220 h - - -
      2.68618  4.42225  2.77519  2.73123  3.46354  2.40513  3.72494  3.29354  2.67741  2.69355  4.24690  2.90347  2.73739  3.18146  2.89801  2.37887  2.77519  2.98518  4.58477  3.61503
      0.02666  4.03368  4.75603  0.61958  0.77255  0.48576  0.95510
204   2.30555  4.22350  3.43374  3.14611  4.17372  3.03257  4.19656  3.47386  3.13834  3.25780  4.14269  3.33259  3.75787  3.48089  3.43255  2.11670  1.21981  3.03536  5.55675  4.32198    221 t - - -
      2.68618  4.42225  2.77519  2.73123  3.46354  2.40513  3.72494  3.29354  2.67741  2.69355  4.24690  2.90347  2.73739  3.18146  2.89801  2.37887  2.77519  2.98518  4.58477  3.61503
      0.02666  4.03368  4.75603  0.61958  0.77255  0.48576  0.95510
205   2.36574  4.32930  3.65872  3.15815  1.75263  3.59081  3.88799  2.76633  3.00646  2.41702  3.44077  3.47213  4.03436  3.35966  2.84416  2.91542  2.93719  2.58360  4.70945  3.31533    222 f - - -
      2.68618  4.42225  2.77519  2.73123  3.46354  2.40513  3.72494  3.29354  2.67741  2.69355  4.24690  2.90347  2.73739  3.18146  2.89801  2.37887  2.77519  2.98518  4.58477  3.61503
      0.02666  4.03368  4.75603  0.61958  0.77255  0.48576  0.95510
206   3.61679  4.93173  4.34628  4.09412  2.84921  3.91802  4.11223  3.65007  3.81429  3.01594  4.27597  4.21130  4.44976  4.19354  3.91337  3.81884  3.92595  3.54480  0.61461  2.84332    223 W - - -
      2.68618  4.42225  2.77519  2.73123  3.46354  2.40513  3.72494  3.29354  2.67741  2.69355  4.24690  2.90347  2.73739  3.18146  2.89801  2.37887  2.77519  2.98518  4.58477  3.61503
      0.02666  4.03368  4.75603  0.61958  0.77255  0.48576  0.95510
207   3.20319  4.57064  4.61006  4.13213  1.37470  4.29070  4.23367  1.94497  3.99453  1.79608  3.15748  4.25106  4.59328  4.12791  4.12181  3.65394  3.44761  2.33521  4.51696  2.99787    224 f - - -
      2.68618  4.42225  2.77519  2.73123  3.46354  2.40513  3.72494  3.29354  2.67741  2.69355  4.24690  2.90347  2.73739  3.18146  2.89801  2.37887  2.77519  2.98518  4.58477  3.61503
      0.02666  4.03368  4.75603  0.61958  0.77255  0.48576  0.95510
208   2.79804  4.42881  3.80155  3.22058  3.32060  3.77932  4.01150  2.59907  2.90605  1.90478  1.84177  3.57538  4.14196  3.35482  2.70712  3.07598  3.03082  2.51820  4.95504  3.73980    225 m - - -
      2.68618  4.42225  2.77519  2.73123  3.46354  2.40513  3.72494  3.29354  2.67741  2.69355  4.24690  2.90347  2.73739  3.18146  2.89801  2.37887  2.77519  2.98518  4.58477  3.61503
      0.02666  4.03368  4.75603  0.61958  0.77255  0.48576  0.95510
209   3.09938  5.23515  2.56707  0.81610  4.60288  3.38409  3.98855  4.08925  2.89003  3.68170  4.65157  3.03581  3.99722  3.20272  3.31344  3.06710  3.40581  3.75755  5.74405  4.46989    226 e - - -
      2.68618  4.42225  2.77519  2.73123  3.46354  2.40513  3.72494  3.29354  2.67741  2.69355  4.24690  2.90347  2.73739  3.18146  2.89801  2.37887  2.77519  2.98518  4.58477  3.61503
      0.02666  4.03368  4.75603  0.61958  0.77255  0.48576  0.95510
210   3.09938  5.23515  2.56707  0.81610  4.60288  3.38409  3.98855  4.08925  2.89003  3.68170  4.65157  3.03581  3.99722  3.20272  3.31344  3.06710  3.40581  3.75755  5.74405  4.46989    227 e - - -
      2.68618  4.42225  2.77519  2.73123  3.46354  2.40513  3.72494  3.29354  2.67741  2.69355  4.24690  2.90347  2.73739  3.18146  2.89801  2.37887  2.77519  2.98518  4.58477  3.61503
      0.02666  4.03368  4.75603  0.61958  0.77255  0.48576  0.95510
211   3.10813  4.48498  4.52586  3.99929  2.22741  4.17968  4.01910  2.54768  3.84104  1.14906  3.12173  4.09534  4.46864  3.96382  3.95498  3.50086  3.33101  2.57603  3.13821  2.78988    228 l - - -
      2.68618  4.42225  2.77519  2.73123  3.46354  2.40513  3.72494  3.29354  2.67741  2.69355  4.24690  2.90347  2.73739  3.18146  2.89801  2.37887  2.77519  2.98518  4.58477  3.61503
      0.02666  4.03368  4.75603  0.61958  0.77255  0.48576  0.95510
212   3.47001  4.82537  4.45997  4.20753  0.78069  4.11698  3.95839  2.97664  4.11116  2.31068  3.68451  4.20413  4.57381  4.23529  4.19093  3.72996  3.77217  2.97875  4.16427  2.51296    229 f - - -
      2.68618  4.42225  2.77519  2.73123  3.46354  2.40513  3.72494  3.29354  2.67741  2.69355  4.24690  2.90347  2.73739  3.18146  2.89801  2.37887  2.77519  2.98518  4.58477  3.61503
      0.02666  4.03368  4.75603  0.61958  0.77255  0.48576  0.95510
213   1.96876  4.23110  4.00352  3.57987  3.75717  3.48792  4.42772  2.27611  3.49751  2.56255  3.64793  3.74435  4.09288  3.80206  3.75368  2.89941  2.79389  1.28940  5.38488  4.17461    230 v - - -
      2.68618  4.42225  2.77519  2.73123  3.46354  2.40513  3.72494  3.29354  2.67741  2.69355  4.24690  2.90347  2.73739  3.18146  2.89801  2.37887  2.77519  2.98518  4.58477  3.61503
      0.02666  4.03368  4.75603  0.61958  0.77255  0.48576  0.95510
214   0.80423  4.27961  3.63271  3.43471  4.21913  3.09921  4.43680  3.36924  3.45528  3.24313  4.25956  3.54150  3.85398  3.78215  3.69276  2.62970  2.91839  2.99555  5.63008  4.44920    231 a - - -
      2.68618  4.42225  2.77519  2.73123  3.46354  2.40513  3.72494  3.29354  2.67741  2.69355  4.24690  2.90347  2.73739  3.18146  2.89801  2.37887  2.77519  2.98518  4.58477  3.61503
      0.02666  4.03368  4.75603  0.61958  0.77255  0.48576  0.95510
215   2.97886  4.70647  3.60392  3.46821  4.44510  3.38498  4.53021  4.03474  3.53984  3.64297  4.70148  3.75741  0.57586  3.92368  3.76486  3.15199  3.43364  3.66126  5.58479  4.56881    232 P - - -
      2.68618  4.42225  2.77519  2.73123  3.46354  2.40513  3.72494  3.29354  2.67741  2.69355  4.24690  2.90347  2.73739  3.18146  2.89801  2.37887  2.77519  2.98518  4.58477  3.61503
      0.02666  4.03368  4.75603  0.61958  0.77255  0.48576  0.95510
216   3.30772  4.65641  4.77318  4.25495  3.13269  4.51763  4.84586  1.86578  4.05984  0.89196  2.96886  4.52472  4.74677  4.26515  4.21073  3.89791  3.54667  2.16082  5.20308  4.05124    233 l - - -
      2.68618  4.42225  2.77519  2.73123  3.46354  2.40513  3.72494  3.29354  2.67741  2.69355  4.24690  2.90347  2.73739  3.18146  2.89801  2.37887  2.77519  2.98518  4.58477  3.61503
      0.02666  4.03368  4.75603  0.61958  0.77255  0.48576  0.95510
217   3.18610  4.97021  3.29694  3.07575  3.40855  3.59320  0.84342  3.91399  2.91490  3.38647  4.44416  3.47748  4.15596  3.44485  3.16957  3.25612  3.49607  3.64618  4.87088  3.35543    234 h - - -
      2.68618  4.42225  2.77519  2.73123  3.46354  2.40513  3.72494  3.29354  2.67741  2.69355  4.24690  2.90347  2.73739  3.18146  2.89801  2.37887  2.77519  2.98518  4.58477  3.61503
      0.02666  4.03368  4.75603  0.61958  0.77255  0.48576  0.95510
218   3.57035  4.86206  4.50299  4.14468  1.98093  4.30544  3.47621  3.41515  3.91612  2.78295  3.99171  3.99752  4.63367  4.01634  4.00177  3.68098  3.79279  3.29915  1.54470  1.34970    235 y - - -
      2.68618  4.42225  2.77519  2.73123  3.46354  2.40513  3.72494  3.29354  2.67741  2.69355  4.24690  2.90347  2.73739  3.18146  2.89801  2.37887  2.77519  2.98518  4.58477  3.61503
      0.02666  4.03368  4.75603  0.61958  0.77255  0.48576  0.95510
219   2.37312  4.29767  3.35995  2.99151  4.05114  1.44582  4.05759  3.34531  2.96209  3.09759  3.55490  3.27126  3.78735  3.31654  3.29331  2.54371  2.36654  2.96921  5.43442  4.18623    236 g - - -
      2.68618  4.42225  2.77519  2.73123  3.46354  2.40513  3.72494  3.29354  2.67741  2.69355  4.24690  2.90347  2.73739  3.18146  2.89801  2.37887  2.77519  2.98518  4.58477  3.61503
      0.02666  4.03368  4.75603  0.61958  0.77255  0.48576  0.95510
220   3.47001  4.82537  4.45997  4.20753  0.78069  4.11698  3.95839  2.97664  4.11116  2.31068  3.68451  4.20413  4.57381  4.23529  4.19093  3.72996  3.77217  2.97875  4.16427  2.51296    237 f - - -
      2.68618  4.42225  2.77519  2.73123  3.46354  2.40513  3.72494  3.29354  2.67741  2.69355  4.24690  2.90347  2.73739  3.18146  2.89801  2.37887  2.77519  2.98518  4.58477  3.61503
      0.02666  4.03368  4.75603  0.61958  0.77255  0.48576  0.95510
221   2.93687  4.43793  4.34616  4.01693  3.67265  3.88798  4.79231  2.05576  3.88719  2.33338  3.61587  4.18014  4.44146  4.23659  4.08377  3.40376  3.29572  0.84356  5.48345  4.22394    238 v - - -
```

	2.68618	4.42225	2.77519	2.73123	3.46354	2.40513	3.72494	3.29354	2.67741	2.69355	4.24690	2.90347	2.73739	3.18146	2.89801	2.37887	2.77519	2.98518	4.58477	3.61503	
	0.02666	4.03368	4.75603	0.61958	0.77255	0.48576	0.95510														
222	2.48494	4.13125	3.90961	3.33522	2.36425	3.68734	3.99787	2.10892	3.23952	2.20238	3.05274	3.60936	3.32151	3.48806	3.47624	2.97035	2.71827	2.20476	4.76015	3.55960	239 i - - -
	2.68618	4.42225	2.77519	2.73123	3.46354	2.40513	3.72494	3.29354	2.67741	2.69355	4.24690	2.90347	2.73739	3.18146	2.89801	2.37887	2.77519	2.98518	4.58477	3.61503	
	0.02666	4.03368	4.75603	0.61958	0.77255	0.48576	0.95510														
223	2.78811	4.37985	3.87063	3.42927	1.45577	2.87468	3.88551	2.77233	3.34546	2.26319	3.48801	3.65054	4.15575	3.60547	3.58328	3.05641	3.07697	2.61551	4.42825	2.89467	240 f - - -
	2.68618	4.42225	2.77519	2.73123	3.46354	2.40513	3.72494	3.29354	2.67741	2.69355	4.24690	2.90347	2.73739	3.18146	2.89801	2.37887	2.77519	2.98518	4.58477	3.61503	
	0.02666	4.03368	4.75603	0.61958	0.77255	0.48576	0.95510														
224	2.07716	4.27646	3.38025	2.98137	4.01057	1.62596	4.03372	3.31381	2.95741	3.06482	3.93830	3.26466	3.77940	3.29530	3.14181	2.53366	2.76907	2.46132	5.38981	4.15091	241 g - - -
	2.68618	4.42225	2.77519	2.73123	3.46354	2.40513	3.72494	3.29354	2.67741	2.69355	4.24690	2.90347	2.73739	3.18146	2.89801	2.37887	2.77519	2.98518	4.58477	3.61503	
	0.02666	4.03368	4.75603	0.61958	0.77255	0.48576	0.95510														
225	3.61679	4.93173	4.34628	4.09412	2.84921	3.91802	4.11223	3.65007	3.81429	3.01594	4.27597	4.21130	4.44976	4.19354	3.91337	3.81884	3.92595	3.54480	0.61461	2.84332	242 W - - -
	2.68618	4.42225	2.77519	2.73123	3.46354	2.40513	3.72494	3.29354	2.67741	2.69355	4.24690	2.90347	2.73739	3.18146	2.89801	2.37887	2.77519	2.98518	4.58477	3.61503	
	0.02666	4.03368	4.75603	0.61958	0.77255	0.48576	0.95510														
226	2.19927	3.73934	3.77067	3.19789	2.60887	3.15545	3.90705	2.56967	3.11247	2.19795	2.89523	3.49158	3.97781	3.37275	3.37216	2.71784	2.78526	2.36489	4.73207	3.52446	243 l - - -
	2.68618	4.42225	2.77519	2.73123	3.46354	2.40513	3.72494	3.29354	2.67741	2.69355	4.24690	2.90347	2.73739	3.18146	2.89801	2.37887	2.77519	2.98518	4.58477	3.61503	
	0.02666	4.03368	4.75603	0.61958	0.77255	0.48576	0.95510														
227	1.33546	4.33456	3.66500	3.20188	3.66650	3.42236	4.13525	2.66038	3.07949	2.37870	3.32645	3.49038	3.98801	3.44506	3.38268	2.80031	2.91670	2.46225	5.23969	3.98806	244 a - - -
	2.68618	4.42225	2.77519	2.73123	3.46354	2.40513	3.72494	3.29354	2.67741	2.69355	4.24690	2.90347	2.73739	3.18146	2.89801	2.37887	2.77519	2.98518	4.58477	3.61503	
	0.02666	4.03368	4.75603	0.61958	0.77255	0.48576	0.95510														
228	2.84593	4.34822	4.08315	3.53047	2.82932	3.91171	3.71040	2.43776	3.39603	1.27177	3.12926	3.80529	4.25292	3.65216	3.62079	3.21096	3.08046	2.14755	4.72538	3.40207	245 l - - -
	2.68618	4.42225	2.77519	2.73123	3.46354	2.40513	3.72494	3.29354	2.67741	2.69355	4.24690	2.90347	2.73739	3.18146	2.89801	2.37887	2.77519	2.98518	4.58477	3.61503	
	0.02666	4.03368	4.75603	0.61958	0.77255	0.48576	0.95510														
229	1.97621	4.32021	3.34914	2.86044	3.70961	2.15084	3.87155	3.12084	2.82265	2.81278	3.68466	3.22341	3.82709	3.15223	3.18447	2.44139	2.77872	2.81909	3.52205	3.85216	246 a - - -
	2.68618	4.42225	2.77519	2.73123	3.46354	2.40513	3.72494	3.29354	2.67741	2.69355	4.24690	2.90347	2.73739	3.18146	2.89801	2.37887	2.77519	2.98518	4.58477	3.61503	
	0.02666	4.03368	4.75603	0.61958	0.77255	0.48576	0.95510														
230	3.04394	4.37808	4.71254	4.17249	2.76532	4.33777	4.73000	1.59649	4.06600	1.93867	3.22321	4.37570	4.62764	4.26170	4.23109	3.68372	3.28925	1.23908	5.20619	3.98721	247 v - - -
	2.68618	4.42225	2.77519	2.73123	3.46354	2.40513	3.72494	3.29354	2.67741	2.69355	4.24690	2.90347	2.73739	3.18146	2.89801	2.37887	2.77519	2.98518	4.58477	3.61503	
	0.02666	4.03368	4.75603	0.61958	0.77255	0.48576	0.95510														
231	2.63421	4.14246	3.94981	3.37466	2.59488	3.10587	3.96674	2.49953	3.26847	1.81798	2.52034	3.63199	4.07349	3.50629	3.48878	2.99398	2.86607	2.34645	4.66008	3.18713	248 l - - -
	2.68618	4.42225	2.77519	2.73123	3.46354	2.40513	3.72494	3.29354	2.67741	2.69355	4.24690	2.90347	2.73739	3.18146	2.89801	2.37887	2.77519	2.98518	4.58477	3.61503	
	0.02666	4.03368	4.75603	0.61958	0.77255	0.48576	0.95510														
232	2.87735	4.63403	3.58392	3.50518	4.63929	0.51867	4.61309	4.28057	3.70822	3.91370	4.89265	3.74860	4.03020	4.01983	3.92413	3.05432	3.37115	3.77778	5.68928	4.74753	249 G - - -
	2.68618	4.42225	2.77519	2.73123	3.46354	2.40513	3.72494	3.29354	2.67741	2.69355	4.24690	2.90347	2.73739	3.18146	2.89801	2.37887	2.77519	2.98518	4.58477	3.61503	
	0.02666	4.03368	4.75603	0.61958	0.77255	0.48576	0.95510														
233	2.66232	4.25542	3.77653	3.23015	3.45558	3.70284	4.06673	2.10976	3.14159	2.33064	3.39811	3.01286	4.10313	3.45108	3.44146	2.99539	2.66429	1.57834	4.99997	3.77472	250 v - - -
	2.68618	4.42225	2.77519	2.73123	3.46354	2.40513	3.72494	3.29354	2.67741	2.69355	4.24690	2.90347	2.73739	3.18146	2.89801	2.37887	2.77519	2.98518	4.58477	3.61503	
	0.02666	4.03368	4.75603	0.61958	0.77255	0.48576	0.95510														
234	2.40842	4.11706	3.77667	3.20245	3.23038	3.10809	3.90637	2.50398	3.10770	1.96272	3.23215	3.49852	3.98795	3.37416	3.36486	2.78916	2.79325	2.07398	3.74027	3.51608	251 l - - -
	2.68618	4.42225	2.77519	2.73123	3.46354	2.40513	3.72494	3.29354	2.67741	2.69355	4.24690	2.90347	2.73739	3.18146	2.89801	2.37887	2.77519	2.98518	4.58477	3.61503	
	0.02666	4.03368	4.75603	0.61958	0.77255	0.48576	0.95510														
235	2.47784	4.25649	3.49718	2.93608	2.97554	3.47593	3.82169	2.66623	2.86668	1.92494	3.36426	2.86640	3.93783	3.17685	3.08639	2.80953	2.69512	2.50911	4.84436	3.60923	252 l - - -
	2.68618	4.42225	2.77519	2.73123	3.46354	2.40513	3.72494	3.29354	2.67741	2.69355	4.24690	2.90347	2.73739	3.18146	2.89801	2.37887	2.77519	2.98518	4.58477	3.61503	
	0.02666	4.03368	4.75603	0.61958	0.77255	0.48576	0.95510														
236	2.52056	4.85155	2.61209	2.38789	4.11366	3.38586	3.62335	3.26717	2.40978	3.12241	3.93784	2.91318	3.80297	1.98877	2.86734	2.63053	2.66313	3.19078	5.35611	3.90029	253 q - - -
	2.68618	4.42225	2.77519	2.73123	3.46354	2.40513	3.72494	3.29354	2.67741	2.69355	4.24690	2.90347	2.73739	3.18146	2.89801	2.37887	2.77519	2.98518	4.58477	3.61503	
	0.02666	4.03368	4.75603	0.61958	0.77255	0.48576	0.95510														
237	2.46525	4.31540	3.40741	2.51984	3.46484	3.07136	3.79623	2.47152	2.79727	2.43153	2.87980	3.26112	3.92174	3.10951	3.16038	2.78414	2.69970	2.39213	4.90946	3.66998	254 v - - -
	2.68618	4.42225	2.77519	2.73123	3.46354	2.40513	3.72494	3.29354	2.67741	2.69355	4.24690	2.90347	2.73739	3.18146	2.89801	2.37887	2.77519	2.98518	4.58477	3.61503	
	0.02666	4.03368	4.75603	0.61958	0.77255	0.48576	0.95510														
238	2.77422	3.11693	4.42271	3.84497	2.41518	3.95011	4.28008	2.13707	3.70815	1.72092	3.11348	3.99946	4.28895	3.88969	3.83903	3.26313	3.01142	1.74153	4.82194	3.61936	255 l - - -
	2.68618	4.42225	2.77519	2.73123	3.46354	2.40513	3.72494	3.29354	2.67741	2.69355	4.24690	2.90347	2.73739	3.18146	2.89801	2.37887	2.77519	2.98518	4.58477	3.61503	
	0.02666	4.03368	4.75603	0.61958	0.77255	0.48576	0.95510														
239	2.35834	4.51838	3.05332	2.59673	3.71214	3.10053	3.69120	3.02570	2.57634	2.70712	3.29599	3.07336	2.86788	2.91063	2.99882	2.64405	2.55911	2.82539	5.07894	3.54955	256 a - - -
	2.68618	4.42225	2.77519	2.73123	3.46354	2.40513	3.72494	3.29354	2.67741	2.69355	4.24690	2.90347	2.73739	3.18146	2.89801	2.37887	2.77519	2.98518	4.58477	3.61503	
	0.02666	4.03368	4.75603	0.61958	0.77255	0.48576	0.95510														
240	2.30222	4.39321	3.12438	2.59377	4.10354	3.22635	3.71620	3.50986	2.44273	3.11959	3.94362	2.86801	3.80805	2.88397	1.90176	2.30216	2.83187	3.15234	5.34862	4.05663	257 r - - -
	2.68618	4.42225	2.77519	2.73123	3.46354	2.40513	3.72494	3.29354	2.67741	2.69355	4.24690	2.90347	2.73739	3.18146	2.89801	2.37887	2.77519	2.98518	4.58477	3.61503	
	0.02666	4.03368	4.75603	0.61958	0.77255	0.48576	0.95510														
241	3.10294	4.42309	4.79876	4.24208	2.76092	4.40331	4.75936	1.43934	4.12953	1.54395	3.08535	4.44390	4.65285	4.27787	4.26271	3.74399	3.33690	1.65142	5.16993	4.01220	258 i - - -
	2.68618	4.42225	2.77519	2.73123	3.46354	2.40513	3.72494	3.29354	2.67741	2.69355	4.24690	2.90347	2.73739	3.18146	2.89801	2.37887	2.77519	2.98518	4.58477	3.61503	
	0.02666	4.03368	4.75603	0.61958	0.77255	0.48576	0.95510														
242	2.21064	4.31179	3.38766	2.82188	3.25957	3.52475	3.45712	2.51107	2.65714	2.46535	3.41371	3.24052	3.90557	3.01857	3.13940	2.55241	2.76013	2.51877	4.88770	3.37151	259 a - - -
	2.68618	4.42225	2.77519	2.73123	3.46354	2.40513	3.72494	3.29354	2.67741	2.69355	4.24690	2.90347	2.73739	3.18146	2.89801	2.37887	2.77519	2.98518	4.58477	3.61503	
	0.02666	4.03368	4.75603	0.61958	0.77255	0.48576	0.95510														
243	2.38353	5.06080	2.80064	1.90103	4.37644	3.39103	3.50261	3.82146	2.07127	3.33770	4.12358	2.69082	3.81112	2.64719	2.55839	2.65586	2.90903	3.43866	5.49490	4.13201	260 e - - -
	2.68618	4.42225	2.77519	2.73123	3.46354	2.40513	3.72494	3.29354	2.67741	2.69355	4.24690	2.90347	2.73739	3.18146	2.89801	2.37887	2.77519	2.98518	4.58477	3.61503	

```
          0.02666  4.03368  4.75603  0.61958  0.77255  0.48576  0.95510
    244   2.83601  4.36687  4.12507  3.62830  3.31936  3.57464  4.34750  2.25608  3.46652  1.23278  3.24926  3.88853  4.29262  3.77910  3.69339  3.22776  3.11821  1.90217  5.07301  3.85136    261 l - - -
          2.68618  4.42225  2.77519  2.73123  3.46354  2.40513  3.72494  3.29354  2.67741  2.69355  4.24690  2.90347  2.73739  3.18146  2.89801  2.37887  2.77519  2.98518  4.58477  3.61503
          0.02666  4.03368  4.75603  0.61958  0.77255  0.48576  0.95510
    245   2.63810  3.78440  4.11867  3.53718  3.11353  3.56382  4.08318  1.94377  3.41480  1.71855  2.83650  3.74910  4.12375  3.63841  3.59750  2.94406  2.87351  2.14457  4.74965  3.55947    262 l - - -
          2.68616  4.42238  2.77527  2.73132  3.46335  2.40521  3.72508  3.29356  2.67722  2.69356  4.24703  2.90360  2.73753  3.18139  2.89806  2.37895  2.77504  2.98514  4.58490  3.61423
          0.35283  1.41865  2.89563  1.16291  0.37480  0.48576  0.95510
    246   2.74753  4.93972  2.65366  2.27844  4.36057  1.76984  3.72844  3.77884  2.08797  3.35762  4.20460  2.91715  3.85208  2.89787  2.83842  2.75749  3.02780  3.41870  5.54298  4.21973    268 g - - -
          2.68618  4.42225  2.77519  2.73123  3.46354  2.40513  3.72494  3.29354  2.67741  2.69355  4.24690  2.90347  2.73739  3.18146  2.89801  2.37887  2.77519  2.98518  4.58477  3.61503
          0.02798  3.98589  4.70824  0.61958  0.77255  0.53462  0.88163
    247   2.78392  5.06033  2.95915  2.23530  4.38866  3.47035  3.59338  3.80247  1.53195  3.31109  4.13014  2.96561  3.87549  2.54399  2.42125  2.77015  2.99412  3.45277  5.44433  3.78653    269 k - - -
          2.68618  4.42225  2.77519  2.73123  3.46354  2.40513  3.72494  3.29354  2.67741  2.69355  4.24690  2.90347  2.73739  3.18146  2.89801  2.37887  2.77519  2.98518  4.58477  3.61503
          0.02798  3.98589  4.70824  0.61958  0.77255  0.45638  1.00395
    248   2.51165  5.15173  2.03968  1.96199  4.46118  3.32063  3.62571  3.93442  2.23578  3.44583  4.22517  2.79488  3.34358  2.75060  2.94880  2.57567  2.84434  3.52700  5.60962  4.19335    270 e - - -
          2.68618  4.42225  2.77519  2.73123  3.46354  2.40513  3.72494  3.29354  2.67741  2.69355  4.24690  2.90347  2.73739  3.18146  2.89801  2.37887  2.77519  2.98518  4.58477  3.61503
          0.02666  4.03368  4.75603  0.61958  0.77255  0.48576  0.95510
    249   2.34363  3.45477  3.04944  2.74002  3.55175  3.15940  3.75029  2.81676  2.54349  2.23758  3.49421  3.17976  3.88200  3.02585  3.09124  2.72975  2.68886  2.66496  4.96559  3.71242    271 l - - -
          2.68618  4.42225  2.77519  2.73123  3.46354  2.40513  3.72494  3.29354  2.67741  2.69355  4.24690  2.90347  2.73739  3.18146  2.89801  2.37887  2.77519  2.98518  4.58477  3.61503
          0.02666  4.03368  4.75603  0.61958  0.77255  0.48576  0.95510
    250   2.23411  3.30500  4.03895  3.46507  3.26339  3.74016  4.08674  2.14418  3.36224  2.20025  2.82805  3.70704  4.11608  3.60303  3.57804  3.03420  2.61237  1.86882  4.81383  3.61665    272 v - - -
          2.68618  4.42225  2.77519  2.73123  3.46354  2.40513  3.72494  3.29354  2.67741  2.69355  4.24690  2.90347  2.73739  3.18146  2.89801  2.37887  2.77519  2.98518  4.58477  3.61503
          0.15232  4.03368  2.09087  0.61958  0.77255  0.48576  0.95510
    251   2.14482  4.63010  2.86896  2.12281  4.05280  3.09710  3.68935  3.45910  2.52917  3.08468  3.91127  2.94025  3.43784  2.86329  2.97376  2.26337  2.80227  2.96488  5.33690  4.01776    273 e - - -
          2.68618  4.42225  2.77519  2.73123  3.46354  2.40513  3.72494  3.29354  2.67741  2.69355  4.24690  2.90347  2.73739  3.18146  2.89801  2.37887  2.77519  2.98518  4.58477  3.61503
          0.05556  3.91154  3.38037  0.61958  0.77255  0.60209  0.79334
    252   2.15189  4.68552  2.57939  2.34183  3.96409  3.17168  3.64297  3.35765  2.49023  2.66184  3.83135  2.91567  3.36873  2.81882  2.94447  2.61396  2.81603  3.04414  5.26426  3.94093    274 a - - -
          2.68618  4.42225  2.77519  2.73123  3.46354  2.40513  3.72494  3.29354  2.67741  2.69355  4.24690  2.90347  2.73739  3.18146  2.89801  2.37887  2.77519  2.98518  4.58477  3.61503
          0.05326  3.88692  3.46220  0.61958  0.77255  0.62243  0.76925
    253   2.07510  4.25711  3.80619  3.27890  3.47873  3.64195  4.15149  2.17579  3.18250  1.99651  3.37383  3.59791  4.09352  3.50125  3.48412  2.96927  2.81256  1.83422  5.10686  3.88983    275 v - - -
          2.68618  4.42225  2.77519  2.73123  3.46354  2.40513  3.72494  3.29354  2.67741  2.69355  4.24690  2.90347  2.73739  3.18146  2.89801  2.37887  2.77519  2.98518  4.58477  3.61503
          0.03163  3.86528  4.58763  0.61958  0.77255  0.63958  0.74975
    254   2.57727  4.82712  2.66412  2.02922  4.08300  3.34888  3.57583  3.50444  2.31259  3.09222  3.90179  2.86554  3.76214  2.72026  2.70763  2.59197  2.62081  2.97886  5.31752  3.72338    276 e - - -
          2.68618  4.42225  2.77519  2.73123  3.46354  2.40513  3.72494  3.29354  2.67741  2.69355  4.24690  2.90347  2.73739  3.18146  2.89801  2.37887  2.77519  2.98518  4.58477  3.61503
          0.10260  3.86528  2.56975  0.61958  0.77255  0.63958  0.74975
    255   2.32147  4.68325  2.68851  2.22064  3.95631  3.10345  3.61689  3.35674  2.45857  2.98605  3.82116  2.88483  3.51940  2.69220  2.91032  2.59867  2.80316  2.73662  5.25067  3.92633    277 e - - -
          2.68576  4.42192  2.77530  2.73061  3.46393  2.40530  3.72491  3.29368  2.67758  2.69360  4.24549  2.90342  2.73704  3.18164  2.89834  2.37923  2.77526  2.98509  4.58485  3.61522
          0.35056  1.21838        *  2.14233  0.12486  0.00000        *
//
HMMER3/f [3.1b2 | February 2015]
NAME  pmoC_IA_loose Protein
LENG  255
ALPH  amino
RF    no
MM    no
CONS  yes
CS    no
MAP   yes
DATE  Mon Dec  4 12:52:11 2017
NSEQ  37
EFFN  0.754272
CKSUM 1455442439
STATS LOCAL MSV       -11.0459  0.70267
STATS LOCAL VITERBI   -11.6744  0.70267
STATS LOCAL FORWARD    -5.4963  0.70267
HMM          A        C        D        E        F        G        H        I        K        L        M        N        P        Q        R        S        T        V        W        Y
            m->m     m->i     m->d     i->m     i->i     d->m     d->d
  COMPO   2.54205  4.42220  3.21281  2.79803  2.90927  2.98574  3.63302  2.67397  2.93890  2.34776  3.42199  3.27569  3.42042  3.25298  3.08565  2.79525  2.80266  2.52638  3.73963  3.07432
          2.68618  4.42225  2.77519  2.73123  3.46354  2.40513  3.72494  3.29354  2.67741  2.69355  4.24690  2.90347  2.73739  3.18146  2.89801  2.37887  2.77519  2.98518  4.58477  3.61503
          0.02633  4.04612  4.76847  0.61958  0.77255  0.00000        *
      1   3.11396  4.63711  4.24001  3.84985  3.29524  3.96585  4.54491  2.48869  3.60744  1.86658  1.08039  4.10491  4.43026  3.97953  3.79171  3.47969  3.42418  2.51429  5.15731  3.93282      1 m - - -
          2.68618  4.42225  2.77519  2.73123  3.46354  2.40513  3.72494  3.29354  2.67741  2.69355  4.24690  2.90347  2.73739  3.18146  2.89801  2.37887  2.77519  2.98518  4.58477  3.61503
          0.02633  4.04612  4.76847  0.61958  0.77255  0.48576  0.95510
      2   1.16770  4.22750  3.35690  3.07798  4.25974  3.00208  4.18166  3.64841  3.14762  3.36295  4.20149  3.05819  3.73186  3.44951  3.47108  1.97214  2.75369  3.14784  5.61124  4.37874      2 a - - -
          2.68618  4.42225  2.77519  2.73123  3.46354  2.40513  3.72494  3.29354  2.67741  2.69355  4.24690  2.90347  2.73739  3.18146  2.89801  2.37887  2.77519  2.98518  4.58477  3.61503
          0.02633  4.04612  4.76847  0.61958  0.77255  0.48576  0.95510
      3   2.16531  4.72726  2.98031  2.42163  3.99353  3.39708  3.64791  3.39906  2.45095  2.85833  3.84354  2.69904  3.81209  2.47718  2.89360  2.51218  2.55025  3.02937  5.27398  3.95384      3 a - - -
          2.68618  4.42225  2.77519  2.73123  3.46354  2.40513  3.72494  3.29354  2.67741  2.69355  4.24690  2.90347  2.73739  3.18146  2.89801  2.37887  2.77519  2.98518  4.58477  3.61503
```

	0.02633	4.04612	4.76847	0.61958	0.77255	0.48576	0.95510														
4	2.41715	4.50135	3.18225	2.44100	3.71315	3.45906	3.73844	2.93776	2.62069	2.75020	3.62985	3.11857	3.87201	2.96940	3.02287	2.71222	1.95284	2.70005	5.09655	3.54985	4 t - - -
	2.68618	4.42225	2.77519	2.73123	3.46354	2.40513	3.72494	3.29354	2.67741	2.69355	4.24690	2.90347	2.73739	3.18146	2.89801	2.37887	2.77519	2.98518	4.58477	3.61503	
	0.02633	4.04612	4.76847	0.61958	0.77255	0.48576	0.95510														
5	2.54184	4.51226	3.26450	2.80377	4.05884	3.29874	3.84798	3.41644	2.55038	3.08885	3.95886	3.18298	3.85087	3.05847	2.36362	2.56186	1.55158	3.07430	5.35068	4.09447	5 t - - -
	2.68618	4.42225	2.77519	2.73123	3.46354	2.40513	3.72494	3.29354	2.67741	2.69355	4.24690	2.90347	2.73739	3.18146	2.89801	2.37887	2.77519	2.98518	4.58477	3.61503	
	0.02633	4.04612	4.76847	0.61958	0.77255	0.48576	0.95510														
6	2.51484	4.80288	2.85725	1.99047	4.05031	3.28975	3.61347	3.16728	2.40795	3.06663	3.87781	2.92853	3.79816	2.64574	2.79651	2.58974	2.60455	2.89878	5.30536	3.96669	6 e - - -
	2.68618	4.42225	2.77519	2.73123	3.46354	2.40513	3.72494	3.29354	2.67741	2.69355	4.24690	2.90347	2.73739	3.18146	2.89801	2.37887	2.77519	2.98518	4.58477	3.61503	
	0.02633	4.04612	4.76847	0.61958	0.77255	0.48576	0.95510														
7	2.23201	4.82392	2.90396	2.33501	4.09212	3.10991	3.27088	3.51324	2.31191	3.10551	3.91658	2.92483	3.80259	2.69468	2.86701	2.33926	2.54403	3.17876	5.33723	3.99762	7 a - - -
	2.68569	4.42238	2.77520	2.73128	3.46366	2.40511	3.72496	3.29366	2.67753	2.69367	4.24633	2.90359	2.73742	3.18152	2.89808	2.37891	2.77504	2.98510	4.58489	3.61516	
	0.39040	1.37558	2.65187	1.07891	0.41546	0.48576	0.95510														
8	2.16549	4.24721	3.55129	2.92130	3.53159	3.29838	3.94888	2.48837	2.97203	2.53674	3.47545	3.38243	3.94842	3.28676	3.30048	2.79376	2.65834	1.76888	5.01201	3.78404	14 v - - -
	2.68618	4.42225	2.77519	2.73123	3.46354	2.40513	3.72494	3.29354	2.67741	2.69355	4.24690	2.90347	2.73739	3.18146	2.89801	2.37887	2.77519	2.98518	4.58477	3.61503	
	0.02809	3.98209	4.70444	0.61958	0.77255	0.44704	1.02031														
9	2.39509	4.69246	3.02717	2.49554	4.02817	2.82221	3.66677	3.43660	2.13387	3.05133	3.87242	2.98780	3.80304	2.82959	2.80246	2.30910	2.56669	2.89813	5.29942	3.98394	15 k - - -
	2.68618	4.42225	2.77519	2.73123	3.46354	2.40513	3.72494	3.29354	2.67741	2.69355	4.24690	2.90347	2.73739	3.18146	2.89801	2.37887	2.77519	2.98518	4.58477	3.61503	
	0.02633	4.04612	4.76847	0.61958	0.77255	0.48576	0.95510														
10	1.95875	4.32770	3.40091	2.84059	3.50220	3.35320	3.79834	2.58252	2.77574	2.41426	3.45408	3.25302	3.91133	3.00833	2.99081	2.76635	2.67734	2.45712	4.93707	3.69694	16 a - - -
	2.68618	4.42225	2.77519	2.73123	3.46354	2.40513	3.72494	3.29354	2.67741	2.69355	4.24690	2.90347	2.73739	3.18146	2.89801	2.37887	2.77519	2.98518	4.58477	3.61503	
	0.02633	4.04612	4.76847	0.61958	0.77255	0.48576	0.95510														
11	2.11879	4.71142	2.64785	2.35790	3.95846	3.40347	3.64877	3.21006	2.47342	2.84685	3.81501	2.96975	3.81246	2.81617	2.92772	2.34927	2.81964	2.88800	5.25451	3.93323	17 a - - -
	2.68618	4.42225	2.77519	2.73123	3.46354	2.40513	3.72494	3.29354	2.67741	2.69355	4.24690	2.90347	2.73739	3.18146	2.89801	2.37887	2.77519	2.98518	4.58477	3.61503	
	0.02633	4.04612	4.76847	0.61958	0.77255	0.48576	0.95510														
12	2.04312	4.49217	3.17678	2.44731	3.70754	3.31888	3.72455	2.68202	2.55184	2.75669	3.62256	3.10545	3.85819	2.95498	3.02409	2.63234	2.56102	2.72656	5.08377	3.81075	18 a - - -
	2.68618	4.42225	2.77519	2.73123	3.46354	2.40513	3.72494	3.29354	2.67741	2.69355	4.24690	2.90347	2.73739	3.18146	2.89801	2.37887	2.77519	2.98518	4.58477	3.61503	
	0.02633	4.04612	4.76847	0.61958	0.77255	0.48576	0.95510														
13	2.23372	4.91000	2.82978	1.95771	4.20496	3.16302	3.61546	3.55928	2.39489	3.20686	4.00352	2.88855	3.32743	2.60605	2.80063	2.53060	2.85352	3.28108	5.41364	4.05378	19 e - - -
	2.68618	4.42225	2.77519	2.73123	3.46354	2.40513	3.72494	3.29354	2.67741	2.69355	4.24690	2.90347	2.73739	3.18146	2.89801	2.37887	2.77519	2.98518	4.58477	3.61503	
	0.02633	4.04612	4.76847	0.61958	0.77255	0.48576	0.95510														
14	2.50548	4.87113	2.76630	1.98240	4.13269	3.38639	3.60590	3.56004	2.35413	2.98099	3.94189	2.77786	3.79208	2.67031	2.86296	2.57952	2.62809	2.85546	5.36151	4.00821	20 e - - -
	2.68618	4.42225	2.77519	2.73123	3.46354	2.40513	3.72494	3.29354	2.67741	2.69355	4.24690	2.90347	2.73739	3.18146	2.89801	2.37887	2.77519	2.98518	4.58477	3.61503	
	0.02633	4.04612	4.76847	0.61958	0.77255	0.48576	0.95510														
15	2.15651	4.84303	2.76869	2.22778	4.10636	3.39097	3.60772	3.53036	2.34323	3.11633	3.92004	2.91311	3.40621	2.74852	2.63419	2.61008	2.76166	2.88316	5.34155	3.99470	21 a - - -
	2.68618	4.42225	2.77519	2.73123	3.46354	2.40513	3.72494	3.29354	2.67741	2.69355	4.24690	2.90347	2.73739	3.18146	2.89801	2.37887	2.77519	2.98518	4.58477	3.61503	
	0.02633	4.04612	4.76847	0.61958	0.77255	0.48576	0.95510														
16	2.54181	4.87394	2.73010	2.38422	4.19666	3.35749	3.64808	3.62514	2.23880	3.20376	4.01196	2.90746	2.35041	2.68082	2.88733	2.39738	2.75194	3.27053	5.41979	4.07097	22 k - - -
	2.68618	4.42225	2.77519	2.73123	3.46354	2.40513	3.72494	3.29354	2.67741	2.69355	4.24690	2.90347	2.73739	3.18146	2.89801	2.37887	2.77519	2.98518	4.58477	3.61503	
	0.02633	4.04612	4.76847	0.61958	0.77255	0.48576	0.95510														
17	2.52637	4.21800	3.65052	3.07840	3.33596	3.62249	3.89652	2.43985	2.69594	1.87995	3.00898	3.43376	3.99719	3.28867	3.28927	2.68715	2.83081	2.23478	4.83204	3.61678	23 l - - -
	2.68618	4.42225	2.77519	2.73123	3.46354	2.40513	3.72494	3.29354	2.67741	2.69355	4.24690	2.90347	2.73739	3.18146	2.89801	2.37887	2.77519	2.98518	4.58477	3.61503	
	0.02633	4.04612	4.76847	0.61958	0.77255	0.48576	0.95510														
18	2.58931	4.13146	3.82915	3.25145	2.62184	3.65081	3.92040	2.54700	2.80929	1.87856	3.20474	3.53992	3.74424	3.40957	3.39298	2.92535	2.82102	2.11390	4.29169	3.47262	24 l - - -
	2.68618	4.42225	2.77519	2.73123	3.46354	2.40513	3.72494	3.29354	2.67741	2.69355	4.24690	2.90347	2.73739	3.18146	2.89801	2.37887	2.77519	2.98518	4.58477	3.61503	
	0.02633	4.04612	4.76847	0.61958	0.77255	0.48576	0.95510														
19	2.61439	5.16557	2.16994	2.06857	4.47508	3.12552	3.42810	3.95280	2.45044	3.46037	4.23725	2.33854	3.15639	2.75257	2.96459	2.44569	2.94083	3.54132	5.62210	4.20100	25 e - - -
	2.68618	4.42225	2.77519	2.73123	3.46354	2.40513	3.72494	3.29354	2.67741	2.69355	4.24690	2.90347	2.73739	3.18146	2.89801	2.37887	2.77519	2.98518	4.58477	3.61503	
	0.02633	4.04612	4.76847	0.61958	0.77255	0.48576	0.95510														
20	2.58012	4.34083	3.39376	2.82412	3.44992	3.39981	3.76460	2.81123	2.41606	2.37145	3.44154	3.24245	3.72340	2.92837	3.08972	2.77987	2.81026	2.58616	3.02442	3.06084	26 l - - -
	2.68626	4.42233	2.77511	2.73131	3.46330	2.40521	3.72502	3.29356	2.67744	2.69332	4.24698	2.90319	2.73748	3.18154	2.89809	2.37895	2.77520	2.98526	4.58485	3.61473	
	0.16607	1.93434	4.76847	1.12597	0.39206	0.48576	0.95510														
21	2.60068	4.75082	3.03468	2.46886	3.98242	3.14791	3.62250	3.39102	2.05601	3.00183	3.65816	2.83879	3.42212	2.78219	2.46077	2.64681	2.82866	3.08119	3.90407	3.70894	32 k - - -
	2.68618	4.42225	2.77519	2.73123	3.46354	2.40513	3.72494	3.29354	2.67741	2.69355	4.24690	2.90347	2.73739	3.18146	2.89801	2.37887	2.77519	2.98518	4.58477	3.61503	
	0.02633	4.04612	4.76847	0.61958	0.77255	0.48576	0.95510														
22	2.60219	4.79990	2.99272	2.43838	4.05227	2.96059	3.61400	3.46731	2.26047	3.06310	3.87779	2.56727	3.37589	2.76326	2.42768	2.63450	2.73328	3.14277	3.90834	3.96904	33 k - - -
	2.68618	4.42225	2.77519	2.73123	3.46354	2.40513	3.72494	3.29354	2.67741	2.69355	4.24690	2.90347	2.73739	3.18146	2.89801	2.37887	2.77519	2.98518	4.58477	3.61503	
	0.02633	4.04612	4.76847	0.61958	0.77255	0.48576	0.95510														
23	2.69319	4.21729	4.28412	3.70337	2.45381	3.90975	4.14874	2.34273	3.57574	1.57618	2.36627	3.89690	4.23706	3.75615	3.72930	3.20739	3.01121	2.33290	4.68880	2.90628	34 l - - -
	2.68618	4.42225	2.77519	2.73123	3.46354	2.40513	3.72494	3.29354	2.67741	2.69355	4.24690	2.90347	2.73739	3.18146	2.89801	2.37887	2.77519	2.98518	4.58477	3.61503	
	0.02633	4.04612	4.76847	0.61958	0.77255	0.48576	0.95510														
24	2.52858	4.06934	4.06158	3.47981	2.83801	3.70058	3.94795	2.28472	3.35505	2.03529	3.15398	3.67502	4.06493	3.56849	3.52965	2.98912	2.64438	2.27900	2.90720	2.74731	35 l - - -
	2.68618	4.42225	2.77519	2.73123	3.46354	2.40513	3.72494	3.29354	2.67741	2.69355	4.24690	2.90347	2.73739	3.18146	2.89801	2.37887	2.77519	2.98518	4.58477	3.61503	
	0.02633	4.04612	4.76847	0.61958	0.77255	0.48576	0.95510														
25	2.47202	4.16776	3.76484	3.19161	2.85069	3.65079	3.93288	2.21042	2.73791	1.91289	3.24852	3.51019	4.02228	3.37548	3.36863	2.82108	2.83219	2.30154	4.77887	3.56707	36 l - - -
	2.68618	4.42225	2.77519	2.73123	3.46354	2.40513	3.72494	3.29354	2.67741	2.69355	4.24690	2.90347	2.73739	3.18146	2.89801	2.37887	2.77519	2.98518	4.58477	3.61503	
	0.02633	4.04612	4.76847	0.61958	0.77255	0.48576	0.95510														

```
26   2.01724  3.78021  3.42821  2.91290  3.68107  2.09408  3.89419  3.04381  2.87082  2.76071  3.63826  3.26081  3.83606  3.18623  3.22875  2.63132  2.67808  2.75297  5.09709  3.13712     37 a - - -
     2.68618  4.42225  2.77519  2.73123  3.46354  2.40513  3.72494  3.29354  2.67741  2.69355  4.24690  2.90347  2.73739  3.18146  2.89801  2.37887  2.77519  2.98518  4.58477  3.61503
     0.02633  4.04612  4.76847  0.61958  0.77255  0.48576  0.95510
27   2.47464  3.35461  4.07314  3.48597  2.70452  3.68660  3.99647  2.26579  3.36115  1.99759  2.99397  3.68179  4.05336  3.35216  3.53153  2.93817  2.66394  2.05042  4.65992  3.47029     38 l - - -
     2.68618  4.42225  2.77519  2.73123  3.46354  2.40513  3.72494  3.29354  2.67741  2.69355  4.24690  2.90347  2.73739  3.18146  2.89801  2.37887  2.77519  2.98518  4.58477  3.61503
     0.02633  4.04612  4.76847  0.61958  0.77255  0.48576  0.95510
28   2.10466  4.25586  3.54150  2.99796  3.50537  2.59503  3.90747  2.30879  2.94078  2.40201  3.46131  3.35610  3.93205  3.24621  3.27673  2.70486  2.77315  2.35386  4.96970  3.74085     39 a - - -
     2.68618  4.42225  2.77519  2.73123  3.46354  2.40513  3.72494  3.29354  2.67741  2.69355  4.24690  2.90347  2.73739  3.18146  2.89801  2.37887  2.77519  2.98518  4.58477  3.61503
     0.02633  4.04612  4.76847  0.61958  0.77255  0.48576  0.95510
29   2.40079  4.18083  4.19392  3.62095  2.92222  3.73239  4.21300  1.94811  3.51234  1.71545  3.18822  3.85364  4.22930  3.73873  3.71036  3.17781  2.96998  1.85447  4.87763  3.68793     40 l - - -
     2.68618  4.42225  2.77519  2.73123  3.46354  2.40513  3.72494  3.29354  2.67741  2.69355  4.24690  2.90347  2.73739  3.18146  2.89801  2.37887  2.77519  2.98518  4.58477  3.61503
     0.02633  4.04612  4.76847  0.61958  0.77255  0.48576  0.95510
30   2.87479  4.30164  4.33045  3.75919  2.83372  4.00302  4.18568  2.04419  3.63396  1.43802  2.93054  3.96337  4.31670  3.81507  3.79768  3.30378  3.10189  2.31563  4.68604  2.42817     41 l - - -
     2.68618  4.42225  2.77519  2.73123  3.46354  2.40513  3.72494  3.29354  2.67741  2.69355  4.24690  2.90347  2.73739  3.18146  2.89801  2.37887  2.77519  2.98518  4.58477  3.61503
     0.02633  4.04612  4.76847  0.61958  0.77255  0.48576  0.95510
31   2.55236  4.11321  3.20910  2.70863  3.44704  3.46832  3.74356  2.90010  2.67728  2.46816  3.54164  2.81460  3.87061  3.00202  3.07501  2.33483  2.27478  2.68824  5.00816  3.74678     42 t - - -
     2.68618  4.42225  2.77519  2.73123  3.46354  2.40513  3.72494  3.29354  2.67741  2.69355  4.24690  2.90347  2.73739  3.18146  2.89801  2.37887  2.77519  2.98518  4.58477  3.61503
     0.02633  4.04612  4.76847  0.61958  0.77255  0.48576  0.95510
32   2.43847  4.20651  3.96162  3.40799  3.40091  3.16154  4.15151  2.02446  3.31707  2.13198  3.34270  3.69580  4.15338  3.59345  3.57914  3.05809  2.93114  1.58182  4.97359  3.76435     43 v - - -
     2.68618  4.42225  2.77519  2.73123  3.46354  2.40513  3.72494  3.29354  2.67741  2.69355  4.24690  2.90347  2.73739  3.18146  2.89801  2.37887  2.77519  2.98518  4.58477  3.61503
     0.02633  4.04612  4.76847  0.61958  0.77255  0.48576  0.95510
33   3.01334  4.40320  4.46056  3.92353  1.43681  4.09180  3.97926  2.44333  3.77648  1.89085  2.77643  4.02071  4.39876  3.90599  3.89522  3.40414  3.23808  2.53048  3.60493  2.79267     44 f - - -
     2.68618  4.42225  2.77519  2.73123  3.46354  2.40513  3.72494  3.29354  2.67741  2.69355  4.24690  2.90347  2.73739  3.18146  2.89801  2.37887  2.77519  2.98518  4.58477  3.61503
     0.02633  4.04612  4.76847  0.61958  0.77255  0.48576  0.95510
34   3.09418  4.75438  3.48095  2.94133  2.65331  3.77165  3.72084  3.28182  3.10617  2.84318  3.92911  3.51528  4.23726  3.48703  3.36675  3.22420  3.35800  3.09741  4.26683  1.07424     45 y - - -
     2.68618  4.42225  2.77519  2.73123  3.46354  2.40513  3.72494  3.29354  2.67741  2.69355  4.24690  2.90347  2.73739  3.18146  2.89801  2.37887  2.77519  2.98518  4.58477  3.61503
     0.02633  4.04612  4.76847  0.61958  0.77255  0.48576  0.95510
35   2.47832  4.14880  3.79672  3.22499  3.27768  3.00416  3.95418  2.12098  3.13804  2.02862  2.95177  3.53068  4.02530  3.40624  3.40405  2.78043  2.82698  2.17397  4.78865  3.58215     46 l - - -
     2.68618  4.42225  2.77519  2.73123  3.46354  2.40513  3.72494  3.29354  2.67741  2.69355  4.24690  2.90347  2.73739  3.18146  2.89801  2.37887  2.77519  2.98518  4.58477  3.61503
     0.02633  4.04612  4.76847  0.61958  0.77255  0.48576  0.95510
36   2.30542  3.82571  3.85252  3.27250  3.01623  3.00979  3.91750  2.42179  3.17613  2.19751  3.04083  3.54162  3.99742  3.42218  3.40877  2.76697  2.78446  2.26428  3.23105  3.48373     47 l - - -
     2.68618  4.42225  2.77519  2.73123  3.46354  2.40513  3.72494  3.29354  2.67741  2.69355  4.24690  2.90347  2.73739  3.18146  2.89801  2.37887  2.77519  2.98518  4.58477  3.61503
     0.02633  4.04612  4.76847  0.61958  0.77255  0.48576  0.95510
37   2.88261  3.26965  4.57708  4.01666  3.16594  4.14014  4.54357  1.74586  3.89857  1.97874  3.26830  4.19177  4.46618  4.10625  4.05375  3.46851  3.12646  1.28638  5.10103  3.90945     48 v - - -
     2.68618  4.42225  2.77519  2.73123  3.46354  2.40513  3.72494  3.29354  2.67741  2.69355  4.24690  2.90347  2.73739  3.18146  2.89801  2.37887  2.77519  2.98518  4.58477  3.61503
     0.02633  4.04612  4.76847  0.61958  0.77255  0.48576  0.95510
38   2.76439  4.81500  3.26412  2.67907  4.07531  3.53174  3.66756  3.47511  2.20949  3.06735  3.19506  2.78619  3.93225  2.83946  1.57016  2.82512  2.98772  3.18617  5.27726  4.00893     49 r - - -
     2.68618  4.42225  2.77519  2.73123  3.46354  2.40513  3.72494  3.29354  2.67741  2.69355  4.24690  2.90347  2.73739  3.18146  2.89801  2.37887  2.77519  2.98518  4.58477  3.61503
     0.02633  4.04612  4.76847  0.61958  0.77255  0.48576  0.95510
39   2.85057  4.26588  4.38136  3.80347  3.02703  3.99551  4.23508  1.88717  3.65057  1.56014  3.05285  3.99074  4.31411  3.84352  3.79768  3.30216  3.08016  2.12212  2.67851  3.49658     50 l - - -
     2.68618  4.42225  2.77519  2.73123  3.46354  2.40513  3.72494  3.29354  2.67741  2.69355  4.24690  2.90347  2.73739  3.18146  2.89801  2.37887  2.77519  2.98518  4.58477  3.61503
     0.02633  4.04612  4.76847  0.61958  0.77255  0.48576  0.95510
40   3.50686  4.92515  4.09883  3.86368  2.32032  4.03574  3.71314  3.49287  3.71639  2.90117  4.15040  3.94349  4.50689  3.98017  3.86318  3.62504  3.79515  3.37202  3.96972  0.71954     51 y - - -
     2.68618  4.42225  2.77519  2.73123  3.46354  2.40513  3.72494  3.29354  2.67741  2.69355  4.24690  2.90347  2.73739  3.18146  2.89801  2.37887  2.77519  2.98518  4.58477  3.61503
     0.02633  4.04612  4.76847  0.61958  0.77255  0.48576  0.95510
41   2.88121  5.27551  2.47161  1.42107  4.57178  3.34629  3.71789  4.04676  2.48530  3.55567  4.40217  2.83574  3.88084  2.09909  2.91099  2.82064  3.13650  3.67008  5.70676  4.31377     52 e - - -
     2.68618  4.42225  2.77519  2.73123  3.46354  2.40513  3.72494  3.29354  2.67741  2.69355  4.24690  2.90347  2.73739  3.18146  2.89801  2.37887  2.77519  2.98518  4.58477  3.61503
     0.02633  4.04612  4.76847  0.61958  0.77255  0.48576  0.95510
42   2.73567  4.94082  2.96244  2.51003  4.32547  2.17348  3.66391  3.75044  2.26672  3.29306  4.11973  2.99951  3.87527  2.21994  2.16704  2.75090  2.98092  3.39530  5.45252  4.15217     53 r - - -
     2.68618  4.42225  2.77519  2.73123  3.46354  2.40513  3.72494  3.29354  2.67741  2.69355  4.24690  2.90347  2.73739  3.18146  2.89801  2.37887  2.77519  2.98518  4.58477  3.61503
     0.02633  4.04612  4.76847  0.61958  0.77255  0.48576  0.95510
43   2.10508  4.21024  3.82799  3.27264  3.37247  3.68588  4.04859  2.24944  3.18738  2.34216  3.33538  3.58584  4.08488  3.47164  3.46752  2.97802  2.78236  1.69646  4.91572  3.26680     54 v - - -
     2.68618  4.42225  2.77519  2.73123  3.46354  2.40513  3.72494  3.29354  2.67741  2.69355  4.24690  2.90347  2.73739  3.18146  2.89801  2.37887  2.77519  2.98518  4.58477  3.61503
     0.02633  4.04612  4.76847  0.61958  0.77255  0.48576  0.95510
44   3.66896  4.89712  4.65947  4.35232  1.17392  4.41544  3.48814  3.34555  4.19636  2.67071  3.94088  4.09597  4.72261  4.15556  4.22237  3.78615  3.89048  3.27360  3.62505  1.23634     55 f - - -
     2.68618  4.42225  2.77519  2.73123  3.46354  2.40513  3.72494  3.29354  2.67741  2.69355  4.24690  2.90347  2.73739  3.18146  2.89801  2.37887  2.77519  2.98518  4.58477  3.61503
     0.02633  4.04612  4.76847  0.61958  0.77255  0.48576  0.95510
45   1.58956  4.18668  3.39390  3.15686  4.38659  1.45975  4.27032  3.79052  3.27453  3.49234  4.31487  3.31145  3.70702  3.54381  3.58754  2.25342  2.73483  3.21917  5.72527  4.51501     56 g - - -
     2.68618  4.42225  2.77519  2.73123  3.46354  2.40513  3.72494  3.29354  2.67741  2.69355  4.24690  2.90347  2.73739  3.18146  2.89801  2.37887  2.77519  2.98518  4.58477  3.61503
     0.02633  4.04612  4.76847  0.61958  0.77255  0.48576  0.95510
46   2.75293  4.22463  4.11413  3.54863  2.70559  3.82917  3.96484  2.07405  3.41021  2.09121  3.18287  3.75630  4.17958  3.43517  3.59924  3.12288  2.98406  2.31449  2.21070  3.12394     57 i - - -
     2.68618  4.42225  2.77519  2.73123  3.46354  2.40513  3.72494  3.29354  2.67741  2.69355  4.24690  2.90347  2.73739  3.18146  2.89801  2.37887  2.77519  2.98518  4.58477  3.61503
     0.02633  4.04612  4.76847  0.61958  0.77255  0.48576  0.95510
47   2.38434  4.63406  3.07880  2.52932  3.68508  3.42695  3.66396  3.26035  2.38458  2.90260  3.74359  3.02096  3.82906  2.84969  2.66681  2.07756  2.58499  2.96822  5.18342  3.70148     58 s - - -
     2.68618  4.42225  2.77519  2.73123  3.46354  2.40513  3.72494  3.29354  2.67741  2.69355  4.24690  2.90347  2.73739  3.18146  2.89801  2.37887  2.77519  2.98518  4.58477  3.61503
     0.02633  4.04612  4.76847  0.61958  0.77255  0.48576  0.95510
48   1.94986  4.74644  3.02957  2.51206  3.98928  3.43776  3.26016  3.39975  2.03852  3.01654  3.85437  3.00705  3.84727  2.82499  2.78283  2.69217  2.77601  3.09531  5.25843  3.71479     59 a - - -
```

```
         2.68618  4.42225  2.77519  2.73123  3.46354  2.40513  3.72494  3.29354  2.67741  2.69355  4.24690  2.90347  2.73739  3.18146  2.89801  2.37887  2.77519  2.98518  4.58477  3.61503
         0.02633  4.04612  4.76847  0.61958  0.77255  0.48576  0.95510
     49  2.89498  4.64988  3.60405  3.52716  4.66000  0.50522  4.63379  4.30482  3.73186  3.93620  4.91564  3.76881  4.04640  4.04253  3.94591  3.07238  3.38974  3.79962  5.70617  4.76884     60 G - - -
         2.68618  4.42225  2.77519  2.73123  3.46354  2.40513  3.72494  3.29354  2.67741  2.69355  4.24690  2.90347  2.73739  3.18146  2.89801  2.37887  2.77519  2.98518  4.58477  3.61503
         0.02633  4.04612  4.76847  0.61958  0.77255  0.48576  0.95510
     50  3.33144  4.66746  4.83782  4.29143  3.12412  4.57589  4.88023  1.89274  4.10723  0.87331  2.85896  4.56852  4.77098  4.28256  4.25236  3.93711  3.56073  2.18374  5.21579  4.10316     61 l - - -
         2.68618  4.42225  2.77519  2.73123  3.46354  2.40513  3.72494  3.29354  2.67741  2.69355  4.24690  2.90347  2.73739  3.18146  2.89801  2.37887  2.77519  2.98518  4.58477  3.61503
         0.02633  4.04612  4.76847  0.61958  0.77255  0.48576  0.95510
     51  3.18499  5.32951  0.69765  2.45972  4.73194  3.35018  4.09614  4.36153  3.21499  3.94017  4.92528  3.02360  4.01554  3.33783  3.75234  3.12996  3.52838  3.98326  5.86590  4.58030     62 d - - -
         2.68618  4.42225  2.77519  2.73123  3.46354  2.40513  3.72494  3.29354  2.67741  2.69355  4.24690  2.90347  2.73739  3.18146  2.89801  2.37887  2.77519  2.98518  4.58477  3.61503
         0.02633  4.04612  4.76847  0.61958  0.77255  0.48576  0.95510
     52  2.52695  4.41412  3.37588  2.87381  3.05463  3.48904  3.77103  3.00132  2.83790  2.68204  3.60243  3.26588  3.93628  3.15709  3.19922  1.77564  2.87850  2.75725  4.76066  2.60337     63 s - - -
         2.68618  4.42225  2.77519  2.73123  3.46354  2.40513  3.72494  3.29354  2.67741  2.69355  4.24690  2.90347  2.73739  3.18146  2.89801  2.37887  2.77519  2.98518  4.58477  3.61503
         0.02633  4.04612  4.76847  0.61958  0.77255  0.48576  0.95510
     53  2.55579  4.23614  3.70494  3.24248  1.79242  3.46107  3.97210  2.77018  3.17601  2.50829  3.49294  3.49622  3.97990  3.46162  3.44804  2.55496  2.28162  2.55210  4.78374  3.41288     64 f - - -
         2.68618  4.42225  2.77519  2.73123  3.46354  2.40513  3.72494  3.29354  2.67741  2.69355  4.24690  2.90347  2.73739  3.18146  2.89801  2.37887  2.77519  2.98518  4.58477  3.61503
         0.02633  4.04612  4.76847  0.61958  0.77255  0.48576  0.95510
     54  1.74357  4.56976  2.94901  2.29654  4.14196  3.23032  3.82705  3.52589  2.69053  3.18109  4.01701  3.03811  3.79491  3.01898  3.11393  2.00298  2.71809  3.15009  5.44196  4.14029     65 a - - -
         2.68618  4.42225  2.77519  2.73123  3.46354  2.40513  3.72494  3.29354  2.67741  2.69355  4.24690  2.90347  2.73739  3.18146  2.89801  2.37887  2.77519  2.98518  4.58477  3.61503
         0.02633  4.04612  4.76847  0.61958  0.77255  0.48576  0.95510
     55  2.76572  4.93295  2.69000  2.32451  4.29567  3.34911  3.75906  3.73189  2.51389  3.31359  4.17827  2.95160  1.68033  2.53148  2.89718  2.78379  3.04545  3.39013  5.52187  4.19157     66 p - - -
         2.68618  4.42225  2.77519  2.73123  3.46354  2.40513  3.72494  3.29354  2.67741  2.69355  4.24690  2.90347  2.73739  3.18146  2.89801  2.37887  2.77519  2.98518  4.58477  3.61503
         0.02633  4.04612  4.76847  0.61958  0.77255  0.48576  0.95510
     56  2.57440  5.37501  1.92861  1.42119  4.67125  3.27470  3.72219  4.16218  2.64770  3.66866  4.48537  2.73876  3.83872  2.73377  3.19921  2.76885  3.12313  3.74648  5.83630  4.37091     67 e - - -
         2.68618  4.42225  2.77519  2.73123  3.46354  2.40513  3.72494  3.29354  2.67741  2.69355  4.24690  2.90347  2.73739  3.18146  2.89801  2.37887  2.77519  2.98518  4.58477  3.61503
         0.02633  4.04612  4.76847  0.61958  0.77255  0.48576  0.95510
     57  3.55970  4.84294  4.57042  4.24011  0.92661  4.34871  3.57058  3.17225  4.09089  2.49657  3.78776  4.08253  4.67216  4.11248  4.15293  3.72845  3.79153  3.12766  3.72896  1.79834     68 f - - -
         2.68618  4.42225  2.77519  2.73123  3.46354  2.40513  3.72494  3.29354  2.67741  2.69355  4.24690  2.90347  2.73739  3.18146  2.89801  2.37887  2.77519  2.98518  4.58477  3.61503
         0.02633  4.04612  4.76847  0.61958  0.77255  0.48576  0.95510
     58  2.73432  5.15604  2.52769  1.81259  4.46755  3.35448  3.63502  3.92654  2.39035  3.43803  4.23081  2.83165  3.82160  1.99341  2.71289  2.69166  2.87384  3.53375  5.59586  4.20522     69 e - - -
         2.68618  4.42225  2.77519  2.73123  3.46354  2.40513  3.72494  3.29354  2.67741  2.69355  4.24690  2.90347  2.73739  3.18146  2.89801  2.37887  2.77519  2.98518  4.58477  3.61503
         0.02633  4.04612  4.76847  0.61958  0.77255  0.48576  0.95510
     59  2.53300  4.54134  3.18474  2.65776  3.79308  3.44429  3.73743  2.95904  2.49731  2.81328  3.69347  3.11552  3.87120  2.60198  2.93759  2.71151  1.88499  2.74810  5.15105  3.87902     70 t - - -
         2.68618  4.42225  2.77519  2.73123  3.46354  2.40513  3.72494  3.29354  2.67741  2.69355  4.24690  2.90347  2.73739  3.18146  2.89801  2.37887  2.77519  2.98518  4.58477  3.61503
         0.02633  4.04612  4.76847  0.61958  0.77255  0.48576  0.95510
     60  3.50686  4.92515  4.09883  3.86368  2.32032  4.03574  3.71314  3.49287  3.71639  2.90117  4.15040  3.94349  4.50689  3.98017  3.86318  3.62504  3.79515  3.37202  3.96972  0.71954     71 y - - -
         2.68618  4.42225  2.77519  2.73123  3.46354  2.40513  3.72494  3.29354  2.67741  2.69355  4.24690  2.90347  2.73739  3.18146  2.89801  2.37887  2.77519  2.98518  4.58477  3.61503
         0.02633  4.04612  4.76847  0.61958  0.77255  0.48576  0.95510
     61  3.63987  4.94992  4.36684  4.11814  2.86519  3.93328  4.13009  3.67429  3.83837  3.03776  4.29951  4.23296  4.46616  4.21695  3.93507  3.84178  3.94978  3.56933  0.59760  2.85873     72 W - - -
         2.68618  4.42225  2.77519  2.73123  3.46354  2.40513  3.72494  3.29354  2.67741  2.69355  4.24690  2.90347  2.73739  3.18146  2.89801  2.37887  2.77519  2.98518  4.58477  3.61503
         0.02633  4.04612  4.76847  0.61958  0.77255  0.48576  0.95510
     62  2.97486  4.45501  4.24493  3.68458  2.96563  4.03121  4.19097  2.46921  3.45704  1.73437  1.48045  3.94016  4.35413  3.74853  3.65268  3.34952  3.20796  2.48628  3.94284  3.40148     73 m - - -
         2.68618  4.42225  2.77519  2.73123  3.46354  2.40513  3.72494  3.29354  2.67741  2.69355  4.24690  2.90347  2.73739  3.18146  2.89801  2.37887  2.77519  2.98518  4.58477  3.61503
         0.02633  4.04612  4.76847  0.61958  0.77255  0.48576  0.95510
     63  2.60074  4.78343  2.90987  2.48018  4.23111  3.32484  3.70060  3.65788  2.44310  3.24130  4.05627  1.95684  3.81306  2.85514  2.52730  2.31849  2.55622  3.28407  5.44477  4.11942     74 n - - -
         2.68618  4.42225  2.77519  2.73123  3.46354  2.40513  3.72494  3.29354  2.67741  2.69355  4.24690  2.90347  2.73739  3.18146  2.89801  2.37887  2.77519  2.98518  4.58477  3.61503
         0.02633  4.04612  4.76847  0.61958  0.77255  0.48576  0.95510
     64  3.20125  4.52973  4.86109  4.28327  2.31351  4.44348  4.71050  1.82118  4.15553  1.15491  2.51714  4.48478  4.64783  4.22676  4.24208  3.77609  3.41960  2.28278  5.02683  3.91339     75 l - - -
         2.68618  4.42225  2.77519  2.73123  3.46354  2.40513  3.72494  3.29354  2.67741  2.69355  4.24690  2.90347  2.73739  3.18146  2.89801  2.37887  2.77519  2.98518  4.58477  3.61503
         0.02633  4.04612  4.76847  0.61958  0.77255  0.48576  0.95510
     65  3.31681  4.65701  4.80721  4.26106  2.15230  4.50088  4.59332  2.28785  4.12113  0.94383  2.76145  4.47346  4.69888  4.21072  4.23317  3.84286  3.53593  2.42990  4.86503  3.56491     76 l - - -
         2.68618  4.42225  2.77519  2.73123  3.46354  2.40513  3.72494  3.29354  2.67741  2.69355  4.24690  2.90347  2.73739  3.18146  2.89801  2.37887  2.77519  2.98518  4.58477  3.61503
         0.02633  4.04612  4.76847  0.61958  0.77255  0.48576  0.95510
     66  2.97788  4.44634  4.11288  3.60007  2.36664  3.93865  3.63984  2.73607  3.45335  2.37035  3.56118  3.40403  4.28887  3.66057  3.64774  3.23588  3.20493  2.75669  2.50469  1.55101     77 y - - -
         2.68618  4.42225  2.77519  2.73123  3.46354  2.40513  3.72494  3.29354  2.67741  2.69355  4.24690  2.90347  2.73739  3.18146  2.89801  2.37887  2.77519  2.98518  4.58477  3.61503
         0.02633  4.04612  4.76847  0.61958  0.77255  0.48576  0.95510
     67  2.42861  4.22663  3.61481  3.07570  3.26689  2.85989  3.94232  2.05223  3.01568  2.48171  3.41693  3.41434  3.96151  3.31098  3.33263  2.81380  2.21099  2.40525  4.93751  3.71374     78 i - - -
         2.68618  4.42225  2.77519  2.73123  3.46354  2.40513  3.72494  3.29354  2.67741  2.69355  4.24690  2.90347  2.73739  3.18146  2.89801  2.37887  2.77519  2.98518  4.58477  3.61503
         0.02633  4.04612  4.76847  0.61958  0.77255  0.48576  0.95510
     68  2.72905  4.91477  2.72293  1.47426  4.15647  3.38649  3.71673  2.94517  2.51938  3.17430  4.04786  2.80702  3.86870  2.88758  2.94930  2.74892  2.98390  3.18454  5.43957  4.08198     79 e - - -
         2.68618  4.42225  2.77519  2.73123  3.46354  2.40513  3.72494  3.29354  2.67741  2.69355  4.24690  2.90347  2.73739  3.18146  2.89801  2.37887  2.77519  2.98518  4.58477  3.61503
         0.02633  4.04612  4.76847  0.61958  0.77255  0.48576  0.95510
     69  2.68032  4.17816  3.99846  3.15021  2.97732  3.78582  4.08758  1.93397  3.32541  1.79072  3.08609  3.70102  4.14025  3.57205  3.55910  3.06918  2.61032  2.12007  4.82877  3.63719     80 l - - -
         2.68618  4.42225  2.77519  2.73123  3.46354  2.40513  3.72494  3.29354  2.67741  2.69355  4.24690  2.90347  2.73739  3.18146  2.89801  2.37887  2.77519  2.98518  4.58477  3.61503
         0.02633  4.04612  4.76847  0.61958  0.77255  0.48576  0.95510
     70  2.36641  4.23163  3.77114  3.22811  3.46786  3.62097  4.06220  2.06261  3.15559  2.42101  3.40836  3.54735  2.91884  3.44806  3.45560  2.92618  2.76840  1.77079  5.00091  3.78473     81 v - - -
         2.68618  4.42225  2.77519  2.73123  3.46354  2.40513  3.72494  3.29354  2.67741  2.69355  4.24690  2.90347  2.73739  3.18146  2.89801  2.37887  2.77519  2.98518  4.58477  3.61503
```

```
     0.02633  4.04612  4.76847  0.61958  0.77255  0.48576  0.95510
 71  2.66996  4.06247  4.36122  3.78345  3.03880  3.99138  4.32337  2.00302  3.66698  1.49420  2.70580  3.99356  4.31605  3.86279  3.83316  3.29622  2.93075  1.89037  4.91176  3.75118     82 l - - -
     2.68618  4.42225  2.77519  2.73123  3.46354  2.40513  3.72494  3.29354  2.67741  2.69355  4.24690  2.90347  2.73739  3.18146  2.89801  2.37887  2.77519  2.98518  4.58477  3.61503
     0.02633  4.04612  4.76847  0.61958  0.77255  0.48576  0.95510
 72  2.66661  4.88051  2.79875  1.62661  4.14189  3.38662  3.67015  3.12853  2.46268  3.15171  3.99282  2.62385  3.83559  2.82687  2.90701  2.68667  2.91287  3.04199  5.40166  4.05065     83 e - - -
     2.68618  4.42225  2.77519  2.73123  3.46354  2.40513  3.72494  3.29354  2.67741  2.69355  4.24690  2.90347  2.73739  3.18146  2.89801  2.37887  2.77519  2.98518  4.58477  3.61503
     0.02633  4.04612  4.76847  0.61958  0.77255  0.48576  0.95510
 73  2.27723  4.17023  3.74582  3.17908  3.19605  3.38247  3.94887  2.41587  3.10252  2.10619  3.29576  3.49919  3.73853  3.37705  3.38578  2.90030  2.73248  1.88032  4.82452  3.61310     84 v - - -
     2.68618  4.42225  2.77519  2.73123  3.46354  2.40513  3.72494  3.29354  2.67741  2.69355  4.24690  2.90347  2.73739  3.18146  2.89801  2.37887  2.77519  2.98518  4.58477  3.61503
     0.02633  4.04612  4.76847  0.61958  0.77255  0.48576  0.95510
 74  2.37074  4.19290  4.02456  3.46332  3.37510  3.79576  4.17135  1.93744  3.36861  2.01288  3.31657  3.73946  4.17845  3.63489  3.61685  2.89318  2.82147  1.69295  4.95525  3.75052     85 v - - -
     2.68618  4.42225  2.77519  2.73123  3.46354  2.40513  3.72494  3.29354  2.67741  2.69355  4.24690  2.90347  2.73739  3.18146  2.89801  2.37887  2.77519  2.98518  4.58477  3.61503
     0.02633  4.04612  4.76847  0.61958  0.77255  0.48576  0.95510
 75  2.35025  4.22133  3.61950  3.05626  3.23073  3.57824  3.90577  2.37277  2.99460  2.33774  3.35651  3.41271  3.97829  3.28430  3.31272  2.47603  2.32666  2.17996  4.87454  3.65268     86 v - - -
     2.68618  4.42225  2.77519  2.73123  3.46354  2.40513  3.72494  3.29354  2.67741  2.69355  4.24690  2.90347  2.73739  3.18146  2.89801  2.37887  2.77519  2.98518  4.58477  3.61503
     0.02633  4.04612  4.76847  0.61958  0.77255  0.48576  0.95510
 76  2.06756  4.14928  3.71914  3.15036  2.65847  3.12278  3.88114  2.61660  3.06768  2.22360  3.26615  3.46341  3.97982  3.33829  3.34240  2.86747  2.72097  2.41167  3.65031  3.48003     87 a - - -
     2.68618  4.42225  2.77519  2.73123  3.46354  2.40513  3.72494  3.29354  2.67741  2.69355  4.24690  2.90347  2.73739  3.18146  2.89801  2.37887  2.77519  2.98518  4.58477  3.61503
     0.02633  4.04612  4.76847  0.61958  0.77255  0.48576  0.95510
 77  2.27379  3.95138  3.49496  2.94309  3.50082  2.87923  3.86208  2.46038  2.89095  2.36042  3.46211  3.30807  3.89541  3.19361  3.23529  2.24088  2.66690  2.57475  4.94590  3.71560     88 s - - -
     2.68618  4.42225  2.77519  2.73123  3.46354  2.40513  3.72494  3.29354  2.67741  2.69355  4.24690  2.90347  2.73739  3.18146  2.89801  2.37887  2.77519  2.98518  4.58477  3.61503
     0.02633  4.04612  4.76847  0.61958  0.77255  0.48576  0.95510
 78  2.18273  4.23039  3.63947  3.08051  3.19540  2.60660  3.92975  2.26977  3.01826  2.31402  3.36048  3.43342  3.99292  3.30836  3.33475  2.86469  2.83518  2.21902  4.89325  3.66961     89 a - - -
     2.68618  4.42225  2.77519  2.73123  3.46354  2.40513  3.72494  3.29354  2.67741  2.69355  4.24690  2.90347  2.73739  3.18146  2.89801  2.37887  2.77519  2.98518  4.58477  3.61503
     0.02633  4.04612  4.76847  0.61958  0.77255  0.48576  0.95510
 79  2.55461  4.16577  3.99197  3.41981  2.93998  3.75717  4.07948  2.19909  3.32069  1.65641  3.20566  3.68947  4.12449  3.56853  3.55282  2.91995  2.59919  2.09444  4.82367  3.62636     90 l - - -
     2.68618  4.42225  2.77519  2.73123  3.46354  2.40513  3.72494  3.29354  2.67741  2.69355  4.24690  2.90347  2.73739  3.18146  2.89801  2.37887  2.77519  2.98518  4.58477  3.61503
     0.02633  4.04612  4.76847  0.61958  0.77255  0.48576  0.95510
 80  2.35685  3.49087  3.79023  3.21596  3.22845  3.43957  3.90830  2.51722  3.11763  2.25446  3.23852  3.26779  3.98350  3.38289  3.37025  2.87425  2.78949  2.27192  2.58724  3.50863     91 l - - -
     2.68618  4.42225  2.77519  2.73123  3.46354  2.40513  3.72494  3.29354  2.67741  2.69355  4.24690  2.90347  2.73739  3.18146  2.89801  2.37887  2.77519  2.98518  4.58477  3.61503
     0.02633  4.04612  4.76847  0.61958  0.77255  0.48576  0.95510
 81  2.27341  4.23195  3.27666  3.08619  4.41785  1.18887  4.25202  3.83808  3.23415  3.53363  4.36330  3.28006  3.72185  3.52148  3.54740  2.20005  2.49941  3.26471  5.74827  4.52219     92 g - - -
     2.68618  4.42225  2.77519  2.73123  3.46354  2.40513  3.72494  3.29354  2.67741  2.69355  4.24690  2.90347  2.73739  3.18146  2.89801  2.37887  2.77519  2.98518  4.58477  3.61503
     0.02633  4.04612  4.76847  0.61958  0.77255  0.48576  0.95510
 82  3.66818  4.90012  4.64363  4.31131  1.86733  4.40534  3.44590  3.45855  4.12602  2.80411  4.02960  4.05876  4.70831  4.11197  4.16591  3.76059  3.88295  3.34985  1.96920  1.07089     93 y - - -
     2.68618  4.42225  2.77519  2.73123  3.46354  2.40513  3.72494  3.29354  2.67741  2.69355  4.24690  2.90347  2.73739  3.18146  2.89801  2.37887  2.77519  2.98518  4.58477  3.61503
     0.02633  4.04612  4.76847  0.61958  0.77255  0.48576  0.95510
 83  3.30052  4.61604  4.89227  4.33712  3.16029  4.58887  4.91527  1.63133  4.18359  1.01106  2.81519  4.59889  4.77668  4.32860  4.31810  3.94401  3.52750  2.09251  5.24293  4.15368     94 l - - -
     2.68618  4.42225  2.77519  2.73123  3.46354  2.40513  3.72494  3.29354  2.67741  2.69355  4.24690  2.90347  2.73739  3.18146  2.89801  2.37887  2.77519  2.98518  4.58477  3.61503
     0.02633  4.04612  4.76847  0.61958  0.77255  0.48576  0.95510
 84  3.19070  4.67608  4.22168  3.79476  2.51060  3.98119  3.82735  2.77636  3.46559  2.41171  3.65564  3.90852  4.40756  3.82190  3.63021  3.42533  3.46391  2.90522  1.19165  2.54149     95 w - - -
     2.68618  4.42225  2.77519  2.73123  3.46354  2.40513  3.72494  3.29354  2.67741  2.69355  4.24690  2.90347  2.73739  3.18146  2.89801  2.37887  2.77519  2.98518  4.58477  3.61503
     0.02633  4.04612  4.76847  0.61958  0.77255  0.48576  0.95510
 85  2.59324  4.51407  3.21463  2.64776  3.25666  3.49149  3.70334  2.96897  1.92101  2.66552  3.59887  3.11492  3.87485  2.93508  2.87131  2.72042  2.70527  2.65850  5.04725  3.47791     96 k - - -
     2.68618  4.42225  2.77519  2.73123  3.46354  2.40513  3.72494  3.29354  2.67741  2.69355  4.24690  2.90347  2.73739  3.18146  2.89801  2.37887  2.77519  2.98518  4.58477  3.61503
     0.02633  4.04612  4.76847  0.61958  0.77255  0.48576  0.95510
 86  2.36056  4.22072  3.57852  3.16080  3.92858  3.15873  4.12977  3.08065  3.09820  2.95472  3.87206  3.37946  3.81728  3.42852  3.40703  2.27358  1.43163  2.36180  5.36273  4.13505     97 t - - -
     2.68619  4.42226  2.77520  2.73124  3.46355  2.40514  3.72495  3.29355  2.67742  2.69356  4.24691  2.90347  2.73740  3.18147  2.89802  2.37888  2.77520  2.98519  4.58478  3.61467
     0.09207  2.53243  4.76847  0.42188  1.06657  0.48576  0.95510
 87  3.21057  5.03121  3.71984  3.19868  4.43025  3.66102  3.96379  3.98677  2.32612  3.47126  4.46970  3.54368  4.15769  3.17794  0.74292  3.28809  3.46631  3.70390  5.44441  4.32290     99 r - - -
     2.68618  4.42225  2.77519  2.73123  3.46354  2.40513  3.72494  3.29354  2.67741  2.69355  4.24690  2.90347  2.73739  3.18146  2.89801  2.37887  2.77519  2.98518  4.58477  3.61503
     0.02633  4.04612  4.76847  0.61958  0.77255  0.48576  0.95510
 88  2.77066  5.06794  1.23433  2.28174  4.50752  3.24005  3.81440  3.91557  2.77160  3.54330  4.41067  2.82768  3.53993  2.98939  3.29578  2.75097  2.97815  3.33123  5.76875  4.34230    100 d - - -
     2.68618  4.42225  2.77519  2.73123  3.46354  2.40513  3.72494  3.29354  2.67741  2.69355  4.24690  2.90347  2.73739  3.18146  2.89801  2.37887  2.77519  2.98518  4.58477  3.61503
     0.02633  4.04612  4.76847  0.61958  0.77255  0.48576  0.95510
 89  2.93065  5.01767  3.40013  2.75483  4.37819  3.62477  3.63633  3.74720  1.90313  3.26787  3.78896  3.03077  4.00085  2.78378  1.31848  2.95609  3.12082  3.44160  5.38453  4.17226    101 r - - -
     2.68618  4.42225  2.77519  2.73123  3.46354  2.40513  3.72494  3.29354  2.67741  2.69355  4.24690  2.90347  2.73739  3.18146  2.89801  2.37887  2.77519  2.98518  4.58477  3.61503
     0.02633  4.04612  4.76847  0.61958  0.77255  0.48576  0.95510
 90  2.61761  5.14167  2.39039  2.26539  4.44445  3.34339  3.08335  3.91308  2.16283  3.42620  4.21114  2.01393  3.80602  2.68247  2.88869  2.66401  2.94707  3.51535  5.58685  4.18516    102 n - - -
     2.68618  4.42225  2.77519  2.73123  3.46354  2.40513  3.72494  3.29354  2.67741  2.69355  4.24690  2.90347  2.73739  3.18146  2.89801  2.37887  2.77519  2.98518  4.58477  3.61503
     0.02633  4.04612  4.76847  0.61958  0.77255  0.48576  0.95510
 91  3.15112  4.49889  4.73437  4.16506  3.04226  4.40178  4.72556  2.03806  4.05184  1.17016  2.49501  4.40857  4.63181  4.18548  4.19392  3.73115  3.37710  1.72271  5.13421  4.03824    103 l - - -
     2.68618  4.42225  2.77519  2.73123  3.46354  2.40513  3.72494  3.29354  2.67741  2.69355  4.24690  2.90347  2.73739  3.18146  2.89801  2.37887  2.77519  2.98518  4.58477  3.61503
     0.02633  4.04612  4.76847  0.61958  0.77255  0.48576  0.95510
 92  2.43207  4.95239  2.10684  2.06585  4.23030  3.35826  3.62995  3.65731  2.44122  3.23469  3.21599  2.86534  3.79890  2.76863  2.92623  2.54175  2.88188  3.30152  5.44909  4.07753    104 e - - -
     2.68618  4.42225  2.77519  2.73123  3.46354  2.40513  3.72494  3.29354  2.67741  2.69355  4.24690  2.90347  2.73739  3.18146  2.89801  2.37887  2.77519  2.98518  4.58477  3.61503
     0.02633  4.04612  4.76847  0.61958  0.77255  0.48576  0.95510
```

```
 93   2.21093  4.87132  2.86802  2.39459  4.19201  3.36996  3.63221  3.62032  2.26543  3.19384  3.99760  2.61862  3.80248  2.38688  2.84076  2.35885  2.67684  3.26457  5.40399  4.05811    105 a - - -
      2.68618  4.42225  2.77519  2.73123  3.46354  2.40513  3.72494  3.29354  2.67741  2.69355  4.24690  2.90347  2.73739  3.18146  2.89801  2.37887  2.77519  2.98518  4.58477  3.61503
      0.02633  4.04612  4.76847  0.61958  0.77255  0.48576  0.95510
 94   3.15331  4.46170  4.84525  4.29429  3.32758  4.48967  4.89283  1.55720  4.18332  1.40861  2.95686  4.52258  4.72671  4.35249  4.33535  3.83853  3.38976  1.48635  5.31195  4.19082    106 l - - -
      2.68618  4.42225  2.77519  2.73123  3.46354  2.40513  3.72494  3.29354  2.67741  2.69355  4.24690  2.90347  2.73739  3.18146  2.89801  2.37887  2.77519  2.98518  4.58477  3.61503
      0.02633  4.04612  4.76847  0.61958  0.77255  0.48576  0.95510
 95   1.96705  4.58136  2.97797  2.40520  4.05099  3.27917  3.78276  3.44185  2.63924  3.09157  3.92926  2.82691  3.80457  2.97531  3.06376  2.47330  2.02779  3.09369  5.35999  4.06067    107 a - - -
      2.68618  4.42225  2.77520  2.73123  3.46354  2.40510  3.72495  3.29354  2.67741  2.69355  4.24690  2.90347  2.73740  3.18146  2.89801  2.37887  2.77520  2.98518  4.58477  3.61503
      0.04617  3.30696  4.76847  0.54084  0.87289  0.48576  0.95510
 96   2.32704  4.34040  3.25835  3.03873  4.24761  3.08393  4.18310  3.54973  3.08351  3.28612  4.22291  3.29670  1.22679  3.44953  3.38698  2.56795  2.67762  3.13057  5.62212  4.37516    109 p - - -
      2.68618  4.42225  2.77519  2.73123  3.46354  2.40513  3.72494  3.29354  2.67741  2.69355  4.24690  2.90347  2.73739  3.18146  2.89801  2.37887  2.77519  2.98518  4.58477  3.61503
      0.02633  4.04612  4.76847  0.61958  0.77255  0.48576  0.95510
 97   2.62189  4.92227  3.05227  2.41240  4.23891  2.99896  3.61708  3.64050  2.05597  3.19726  4.01587  2.99609  3.86370  2.75679  1.84594  2.72393  2.92971  3.07697  5.37962  4.08118    110 r - - -
      2.68618  4.42225  2.77519  2.73123  3.46354  2.40513  3.72494  3.29354  2.67741  2.69355  4.24690  2.90347  2.73739  3.18146  2.89801  2.37887  2.77519  2.98518  4.58477  3.61503
      0.02633  4.04612  4.76847  0.61958  0.77255  0.48576  0.95510
 98   2.96462  5.29539  2.39501  1.05992  4.57799  3.32474  3.81567  4.07181  2.66815  3.61335  4.52136  2.85432  3.91760  2.85796  3.10947  2.90368  3.24960  3.71497  5.77161  4.37163    111 e - - -
      2.68618  4.42225  2.77519  2.73123  3.46354  2.40513  3.72494  3.29354  2.67741  2.69355  4.24690  2.90347  2.73739  3.18146  2.89801  2.37887  2.77519  2.98518  4.58477  3.61503
      0.02633  4.04612  4.76847  0.61958  0.77255  0.48576  0.95510
 99   3.11182  5.24854  2.57453  0.80143  4.61810  3.39338  3.99965  4.10707  2.90285  3.69774  4.66764  3.04509  4.00728  3.21392  3.32714  3.07859  3.41853  3.77429  5.75689  4.48345    112 e - - -
      2.68618  4.42225  2.77519  2.73123  3.46354  2.40513  3.72494  3.29354  2.67741  2.69355  4.24690  2.90347  2.73739  3.18146  2.89801  2.37887  2.77519  2.98518  4.58477  3.61503
      0.02633  4.04612  4.76847  0.61958  0.77255  0.48576  0.95510
100   3.31505  4.64247  4.87279  4.30503  3.09557  4.57460  4.87058  1.91341  4.16035  0.94681  2.35362  4.57520  4.74898  4.27607  4.28651  3.91897  3.53460  2.24331  5.18548  4.12750    113 l - - -
      2.68618  4.42225  2.77519  2.73123  3.46354  2.40513  3.72494  3.29354  2.67741  2.69355  4.24690  2.90347  2.73739  3.18146  2.89801  2.37887  2.77519  2.98518  4.58477  3.61503
      0.02633  4.04612  4.76847  0.61958  0.77255  0.48576  0.95510
101   3.11399  5.18135  3.55355  2.87696  4.64219  3.70944  3.64824  3.98823  1.63246  3.43099  4.31555  3.26020  4.08156  2.78803  1.19236  3.11649  3.27791  3.67946  5.46553  4.31452    114 r - - -
      2.68618  4.42225  2.77519  2.73123  3.46354  2.40513  3.72494  3.29354  2.67741  2.69355  4.24690  2.90347  2.73739  3.18146  2.89801  2.37887  2.77519  2.98518  4.58477  3.61503
      0.02633  4.04612  4.76847  0.61958  0.77255  0.48576  0.95510
102   2.81368  4.91079  3.51718  2.98142  4.35589  3.56072  3.81015  3.76809  2.17112  3.30935  4.26459  3.33675  4.04922  2.99260  1.02179  3.03184  3.22460  3.46800  5.43297  4.23602    115 r - - -
      2.68618  4.42225  2.77519  2.73123  3.46354  2.40513  3.72494  3.29354  2.67741  2.69355  4.24690  2.90347  2.73739  3.18146  2.89801  2.37887  2.77519  2.98518  4.58477  3.61503
      0.02633  4.04612  4.76847  0.61958  0.77255  0.48576  0.95510
103   2.65033  4.25399  3.69554  3.13382  2.82198  3.65576  2.98006  2.53665  3.06059  2.31693  3.36508  2.98175  4.02669  3.32791  3.35273  2.91925  2.88105  2.46964  3.50222  2.43789    116 l - - -
      2.68618  4.42225  2.77519  2.73123  3.46354  2.40513  3.72494  3.29354  2.67741  2.69355  4.24690  2.90347  2.73739  3.18146  2.89801  2.37887  2.77519  2.98518  4.58477  3.61503
      0.02633  4.04612  4.76847  0.61958  0.77255  0.48576  0.95510
104   2.56746  3.77034  4.22452  3.64196  2.11443  3.74106  3.95129  2.46574  3.49414  2.10467  3.01078  3.76425  4.10176  3.67384  3.61603  3.03899  2.85600  2.15998  3.35379  2.78685    117 l - - -
      2.68618  4.42225  2.77519  2.73123  3.46354  2.40513  3.72494  3.29354  2.67741  2.69355  4.24690  2.90347  2.73739  3.18146  2.89801  2.37887  2.77519  2.98518  4.58477  3.61503
      0.02633  4.04612  4.76847  0.61958  0.77255  0.48576  0.95510
105   2.57068  4.65368  2.93582  2.50032  3.91244  3.21397  3.59402  3.31561  2.51401  2.95205  3.78819  2.65869  3.81801  2.85539  2.95712  2.43582  2.10880  2.82972  5.22656  3.72053    118 t - - -
      2.68618  4.42225  2.77519  2.73123  3.46354  2.40513  3.72494  3.29354  2.67741  2.69355  4.24690  2.90347  2.73739  3.18146  2.89801  2.37887  2.77519  2.98518  4.58477  3.61503
      0.02633  4.04612  4.76847  0.61958  0.77255  0.48576  0.95510
106   2.67909  4.63016  3.08768  2.57868  3.67790  3.48935  2.67322  3.20260  2.52144  2.01327  3.73504  2.67807  3.90579  2.94713  2.88359  2.78029  2.91316  2.94219  5.06102  3.72012    119 l - - -
      2.68618  4.42225  2.77519  2.73123  3.46354  2.40513  3.72494  3.29354  2.67741  2.69355  4.24690  2.90347  2.73739  3.18146  2.89801  2.37887  2.77519  2.98518  4.58477  3.61503
      0.02633  4.04612  4.76847  0.61958  0.77255  0.48576  0.95510
107   2.51285  4.10500  3.80250  3.22479  3.09894  3.05495  3.90436  2.44547  3.13343  2.14046  3.21279  3.51383  3.99437  3.38926  3.38278  2.89406  2.54280  2.23197  3.05267  3.33864    120 l - - -
      2.68618  4.42225  2.77519  2.73123  3.46354  2.40513  3.72494  3.29354  2.67741  2.69355  4.24690  2.90347  2.73739  3.18146  2.89801  2.37887  2.77519  2.98518  4.58477  3.61503
      0.02633  4.04612  4.76847  0.61958  0.77255  0.48576  0.95510
108   2.51843  4.27521  3.45390  2.84317  3.41321  3.40776  3.79998  2.59541  2.83017  2.18936  2.89778  3.18206  3.92429  2.83023  3.17751  2.72552  2.60085  2.40238  4.86094  3.62853    121 l - - -
      2.68618  4.42225  2.77519  2.73123  3.46354  2.40513  3.72494  3.29354  2.67741  2.69355  4.24690  2.90347  2.73739  3.18146  2.89801  2.37887  2.77519  2.98518  4.58477  3.61503
      0.02633  4.04612  4.76847  0.61958  0.77255  0.48576  0.95510
109   3.11852  4.50539  4.43142  3.92985  2.07731  4.13091  3.81218  2.50486  3.77267  1.99382  3.33916  3.98709  4.44559  3.90585  3.90112  3.44850  3.34427  2.66031  1.70240  2.48229    122 w - - -
      2.68619  4.42226  2.77520  2.73124  3.46355  2.40514  3.72495  3.29355  2.67742  2.69356  4.24691  2.90347  2.73740  3.18147  2.89802  2.37888  2.77505  2.98519  4.58478  3.61504
      0.09207  2.53243  4.76847  0.42188  1.06657  0.48576  0.95510
110   3.02573  4.41228  4.56462  3.98860  3.06568  4.20746  4.47024  1.76342  3.83518  1.17501  2.97289  4.20411  4.48014  4.00695  3.97677  3.52516  3.25198  2.16554  4.21358  3.74626    124 l - - -
      2.68618  4.42225  2.77519  2.73123  3.46354  2.40513  3.72494  3.29354  2.67741  2.69355  4.24690  2.90347  2.73739  3.18146  2.89801  2.37887  2.77519  2.98518  4.58477  3.61503
      0.02633  4.04612  4.76847  0.61958  0.77255  0.48576  0.95510
111   2.39529  4.18923  3.63342  3.07611  3.11045  3.05144  3.90140  2.64661  3.01067  2.43565  3.12324  3.41089  3.95190  3.29525  3.31666  2.54062  2.75626  1.86351  4.84990  3.63152    125 v - - -
      2.68618  4.42225  2.77519  2.73123  3.46354  2.40513  3.72494  3.29354  2.67741  2.69355  4.24690  2.90347  2.73739  3.18146  2.89801  2.37887  2.77519  2.98518  4.58477  3.61503
      0.02633  4.04612  4.76847  0.61958  0.77255  0.48576  0.95510
112   2.40481  3.56199  4.20767  3.64180  3.34897  3.85934  4.25461  1.98569  3.53564  2.25690  2.90282  3.86396  4.23490  3.77519  3.74089  3.16899  2.80713  1.47495  4.94997  3.75289    126 v - - -
      2.68618  4.42225  2.77519  2.73123  3.46354  2.40513  3.72494  3.29354  2.67741  2.69355  4.24690  2.90347  2.73739  3.18146  2.89801  2.37887  2.77519  2.98518  4.58477  3.61503
      0.02633  4.04612  4.76847  0.61958  0.77255  0.48576  0.95510
113   3.56282  4.89813  4.39599  4.07418  1.91667  4.28030  3.52853  3.33600  3.92781  2.70343  3.96626  3.99085  4.63483  4.02209  4.04064  3.67339  3.80448  3.24790  3.70878  0.84709    127 y - - -
      2.68618  4.42225  2.77519  2.73123  3.46354  2.40513  3.72494  3.29354  2.67741  2.69355  4.24690  2.90347  2.73739  3.18146  2.89801  2.37887  2.77519  2.98518  4.58477  3.61503
      0.02633  4.04612  4.76847  0.61958  0.77255  0.48576  0.95510
114   1.51864  4.18927  3.48205  3.14090  4.24526  1.97291  4.19557  3.59962  3.17306  3.32401  4.15909  3.31615  3.71978  3.46255  3.49862  2.43039  2.02963  3.10325  5.59866  4.38618    128 a - - -
      2.68618  4.42225  2.77519  2.73123  3.46354  2.40513  3.72494  3.29354  2.67741  2.69355  4.24690  2.90347  2.73739  3.18146  2.89801  2.37887  2.77519  2.98518  4.58477  3.61503
      0.02633  4.04612  4.76847  0.61958  0.77255  0.48576  0.95510
115   2.54671  4.09699  4.18922  3.60692  2.58757  3.77483  4.04654  2.17286  3.47180  2.08977  3.12218  3.78060  4.13188  3.67262  3.62490  3.07165  2.83907  1.97021  2.72623  3.38764    129 v - - -
```

	2.68618	4.42225	2.77519	2.73123	3.46354	2.40513	3.72494	3.29354	2.67741	2.69355	4.24690	2.90347	2.73739	3.18146	2.89801	2.37887	2.77519	2.98518	4.58477	3.61503	
	0.02633	4.04612	4.76847	0.61958	0.77255	0.48576	0.95510														
116	1.23330	3.44968	3.71459	3.26424	3.91763	2.76373	4.16251	3.21592	3.19450	2.81096	3.86885	3.40689	3.76810	3.48834	3.48697	2.39705	2.71848	2.83940	5.33672	4.13162	130 a - - -
	2.68618	4.42225	2.77519	2.73123	3.46354	2.40513	3.72494	3.29354	2.67741	2.69355	4.24690	2.90347	2.73739	3.18146	2.89801	2.37887	2.77519	2.98518	4.58477	3.61503	
	0.02633	4.04612	4.76847	0.61958	0.77255	0.48576	0.95510														
117	3.17009	4.46623	4.88620	4.35385	3.41697	4.53811	4.99227	1.29272	4.23963	1.57740	3.21322	4.58234	4.78432	4.44059	4.40607	3.90321	3.41492	1.48696	5.41873	4.26850	131 i - - -
	2.68618	4.42225	2.77519	2.73123	3.46354	2.40513	3.72494	3.29354	2.67741	2.69355	4.24690	2.90347	2.73739	3.18146	2.89801	2.37887	2.77519	2.98518	4.58477	3.61503	
	0.02633	4.04612	4.76847	0.61958	0.77255	0.48576	0.95510														
118	3.10552	4.57763	4.16454	3.70188	2.27608	4.02754	3.71277	2.84453	3.57390	2.41149	3.58270	3.82966	4.39336	3.77298	3.77254	3.35459	3.35032	2.33581	4.03617	1.23219	132 y - - -
	2.68618	4.42225	2.77519	2.73123	3.46354	2.40513	3.72494	3.29354	2.67741	2.69355	4.24690	2.90347	2.73739	3.18146	2.89801	2.37887	2.77519	2.98518	4.58477	3.61503	
	0.02633	4.04612	4.76847	0.61958	0.77255	0.48576	0.95510														
119	3.47590	4.79830	4.49275	4.10097	1.90829	4.28475	3.53179	3.24186	3.90864	2.60438	3.82925	3.99793	4.60680	3.99921	4.00800	3.63526	3.70028	3.15364	1.27056	1.91339	133 w - - -
	2.68618	4.42225	2.77519	2.73123	3.46354	2.40513	3.72494	3.29354	2.67741	2.69355	4.24690	2.90347	2.73739	3.18146	2.89801	2.37887	2.77519	2.98518	4.58477	3.61503	
	0.09207	4.04612	2.65258	0.61958	0.77255	0.48576	0.95510														
120	1.90666	4.22076	3.28327	3.11176	4.37076	1.19592	4.26626	3.74209	3.26802	3.48240	4.34910	3.29842	3.71769	3.55414	3.56649	2.46037	2.78449	3.20886	5.69629	4.49636	134 g - - -
	2.68618	4.42225	2.77519	2.73123	3.46354	2.40513	3.72494	3.29354	2.67741	2.69355	4.24690	2.90347	2.73739	3.18146	2.89801	2.37887	2.77519	2.98518	4.58477	3.61503	
	0.02809	3.98214	4.70449	0.61958	0.77255	0.44707	1.02026														
121	0.98212	4.19532	3.48963	3.26284	4.33926	2.36436	4.32875	3.67168	3.34498	3.43767	4.29974	3.38606	3.74339	3.62867	3.62744	2.46484	2.77633	3.15637	5.69092	4.50235	135 a - - -
	2.68618	4.42225	2.77519	2.73123	3.46354	2.40513	3.72494	3.29354	2.67741	2.69355	4.24690	2.90347	2.73739	3.18146	2.89801	2.37887	2.77519	2.98518	4.58477	3.61503	
	0.02633	4.04612	4.76847	0.61958	0.77255	0.48576	0.95510														
122	2.34176	4.24262	3.51102	3.17390	4.02908	3.09145	4.18367	3.29708	3.14430	3.11798	4.05027	3.36869	3.79537	3.48059	3.44653	1.17409	2.79473	2.42588	5.46434	4.19167	136 s - - -
	2.68618	4.42225	2.77519	2.73123	3.46354	2.40513	3.72494	3.29354	2.67741	2.69355	4.24690	2.90347	2.73739	3.18146	2.89801	2.37887	2.77519	2.98518	4.58477	3.61503	
	0.02633	4.04612	4.76847	0.61958	0.77255	0.48576	0.95510														
123	3.67496	4.90748	4.64076	4.33325	1.38451	4.41235	3.48318	3.36525	4.17950	2.69174	3.96140	4.08714	4.72161	4.14632	4.21316	3.78345	3.89704	3.29007	3.62174	1.04641	137 y - - -
	2.68618	4.42225	2.77519	2.73123	3.46354	2.40513	3.72494	3.29354	2.67741	2.69355	4.24690	2.90347	2.73739	3.18146	2.89801	2.37887	2.77519	2.98518	4.58477	3.61503	
	0.02633	4.04612	4.76847	0.61958	0.77255	0.48576	0.95510														
124	3.50811	4.82665	4.51241	4.18639	0.90470	4.30516	3.65631	3.05166	4.04376	2.36699	3.68971	4.09220	4.64926	4.10682	4.12563	3.70889	3.75328	3.03337	3.82995	2.02782	138 f - - -
	2.68618	4.42225	2.77519	2.73123	3.46354	2.40513	3.72494	3.29354	2.67741	2.69355	4.24690	2.90347	2.73739	3.18146	2.89801	2.37887	2.77519	2.98518	4.58477	3.61503	
	0.02633	4.04612	4.76847	0.61958	0.77255	0.48576	0.95510														
125	2.09565	4.21870	3.69045	3.26258	3.90004	3.21859	4.20676	2.83475	3.19452	2.84029	3.81611	3.46236	3.87194	3.51561	3.49484	2.62672	1.46553	2.22368	5.38663	4.17099	139 t - - -
	2.68618	4.42225	2.77519	2.73123	3.46354	2.40513	3.72494	3.29354	2.67741	2.69355	4.24690	2.90347	2.73739	3.18146	2.89801	2.37887	2.77519	2.98518	4.58477	3.61503	
	0.02633	4.04612	4.76847	0.61958	0.77255	0.48576	0.95510														
126	3.11182	5.24854	2.57453	0.80143	4.61810	3.39338	3.99965	4.10707	2.90285	3.69774	4.66764	3.04509	4.00728	3.21392	3.32714	3.07859	3.41853	3.77429	5.75689	4.48345	140 e - - -
	2.68618	4.42225	2.77519	2.73123	3.46354	2.40513	3.72494	3.29354	2.67741	2.69355	4.24690	2.90347	2.73739	3.18146	2.89801	2.37887	2.77519	2.98518	4.58477	3.61503	
	0.02633	4.04612	4.76847	0.61958	0.77255	0.48576	0.95510														
127	3.02995	5.01395	3.02891	2.78981	4.20492	3.48785	3.94846	3.86570	2.59930	3.35039	4.37669	3.24983	4.04592	0.95411	2.87863	3.07190	3.32835	3.58555	5.44754	4.15420	141 q - - -
	2.68618	4.42225	2.77519	2.73123	3.46354	2.40513	3.72494	3.29354	2.67741	2.69355	4.24690	2.90347	2.73739	3.18146	2.89801	2.37887	2.77519	2.98518	4.58477	3.61503	
	0.02633	4.04612	4.76847	0.61958	0.77255	0.48576	0.95510														
128	3.18499	5.32951	0.69765	2.45972	4.73194	3.35018	4.09614	4.36153	3.21499	3.94017	4.92528	3.02360	4.01554	3.33783	3.75234	3.12996	3.52838	3.98326	5.86590	4.58030	142 d - - -
	2.68618	4.42225	2.77519	2.73123	3.46354	2.40513	3.72494	3.29354	2.67741	2.69355	4.24690	2.90347	2.73739	3.18146	2.89801	2.37887	2.77519	2.98518	4.58477	3.61503	
	0.02633	4.04612	4.76847	0.61958	0.77255	0.48576	0.95510														
129	2.53618	4.54988	2.86844	2.74683	4.44004	1.16384	4.09658	3.98252	3.04598	3.61594	4.47214	2.45308	3.82016	3.34057	3.42190	2.64655	2.97940	3.47051	5.71364	4.42345	143 g - - -
	2.68618	4.42225	2.77519	2.73123	3.46354	2.40513	3.72494	3.29354	2.67741	2.69355	4.24690	2.90347	2.73739	3.18146	2.89801	2.37887	2.77519	2.98518	4.58477	3.61503	
	0.02633	4.04612	4.76847	0.61958	0.77255	0.48576	0.95510														
130	2.21943	4.21193	3.56907	3.13060	3.93241	3.14675	4.10490	3.12530	3.08192	2.96942	3.86351	3.35734	3.79993	3.39789	3.40282	2.21033	1.52566	2.39100	5.35081	4.12967	144 t - - -
	2.68618	4.42225	2.77519	2.73123	3.46354	2.40513	3.72494	3.29354	2.67741	2.69355	4.24690	2.90347	2.73739	3.18146	2.89801	2.37887	2.77519	2.98518	4.58477	3.61503	
	0.02633	4.04612	4.76847	0.61958	0.77255	0.48576	0.95510														
131	3.63987	4.94992	4.36684	4.11814	2.86519	3.93328	4.13009	3.67429	3.83837	3.03776	4.29951	4.23296	4.46616	4.21695	3.93507	3.84178	3.94978	3.56933	0.59760	2.85873	145 W - - -
	2.68618	4.42225	2.77519	2.73123	3.46354	2.40513	3.72494	3.29354	2.67741	2.69355	4.24690	2.90347	2.73739	3.18146	2.89801	2.37887	2.77519	2.98518	4.58477	3.61503	
	0.02633	4.04612	4.76847	0.61958	0.77255	0.48576	0.95510														
132	3.20253	4.98347	3.31174	3.09232	3.41943	3.60558	0.82340	3.93238	2.93123	3.40304	4.46212	3.49333	4.16927	3.46173	3.18487	3.27259	3.51314	3.66436	4.88146	3.36606	146 h - - -
	2.68618	4.42225	2.77519	2.73123	3.46354	2.40513	3.72494	3.29354	2.67741	2.69355	4.24690	2.90347	2.73739	3.18146	2.89801	2.37887	2.77519	2.98518	4.58477	3.61503	
	0.02633	4.04612	4.76847	0.61958	0.77255	0.48576	0.95510														
133	2.64597	4.69814	3.08038	2.58636	3.93131	3.43579	3.70445	3.33549	2.44355	2.94845	3.08441	3.06154	3.87149	1.82288	2.81364	2.46073	2.89762	3.05243	5.24586	3.94103	147 q - - -
	2.68618	4.42225	2.77519	2.73123	3.46354	2.40513	3.72494	3.29354	2.67741	2.69355	4.24690	2.90347	2.73739	3.18146	2.89801	2.37887	2.77519	2.98518	4.58477	3.61503	
	0.02633	4.04612	4.76847	0.61958	0.77255	0.48576	0.95510														
134	2.35997	4.22142	3.57232	3.14972	3.92423	3.15963	4.12049	3.08780	3.08970	2.95350	3.86664	3.37310	3.81509	3.41794	3.40158	2.23335	1.46204	2.35265	5.35551	4.12791	148 t - - -
	2.68618	4.42225	2.77519	2.73123	3.46354	2.40513	3.72494	3.29354	2.67741	2.69355	4.24690	2.90347	2.73739	3.18146	2.89801	2.37887	2.77519	2.98518	4.58477	3.61503	
	0.02633	4.04612	4.76847	0.61958	0.77255	0.48576	0.95510														
135	2.52862	4.29834	4.47110	3.91919	3.43969	4.15167	4.57202	1.59007	3.81578	2.11527	2.79730	4.15517	4.48239	4.05659	4.02140	3.47849	3.15965	1.38370	5.20147	4.01671	149 v - - -
	2.68618	4.42225	2.77519	2.73123	3.46354	2.40513	3.72494	3.29354	2.67741	2.69355	4.24690	2.90347	2.73739	3.18146	2.89801	2.37887	2.77519	2.98518	4.58477	3.61503	
	0.02633	4.04612	4.76847	0.61958	0.77255	0.48576	0.95510														
136	3.12613	4.43366	4.85449	4.36264	3.58789	4.48441	5.07302	1.18883	4.25017	2.08010	3.38941	4.57572	4.79353	4.51190	4.44365	3.87749	3.39311	1.24175	5.56923	4.36586	150 i - - -
	2.68618	4.42225	2.77519	2.73123	3.46354	2.40513	3.72494	3.29354	2.67741	2.69355	4.24690	2.90347	2.73739	3.18146	2.89801	2.37887	2.77519	2.98518	4.58477	3.61503	
	0.02633	4.04612	4.76847	0.61958	0.77255	0.48576	0.95510														
137	3.21057	5.03121	3.71984	3.19868	4.43025	3.66102	3.96379	3.98677	2.32612	3.47126	4.46970	3.54368	4.15769	3.17794	0.74292	3.28809	3.46631	3.70390	5.44441	4.32290	151 r - - -
	2.68618	4.42225	2.77519	2.73123	3.46354	2.40513	3.72494	3.29354	2.67741	2.69355	4.24690	2.90347	2.73739	3.18146	2.89801	2.37887	2.77519	2.98518	4.58477	3.61503	

```
      0.02633  4.04612  4.76847  0.61958  0.77255  0.48576  0.95510
138   3.18499  5.32951  0.69765  2.45972  4.73194  3.35018  4.09614  4.36153  3.21499  3.94017  4.92528  3.02360  4.01554  3.33783  3.75234  3.12996  3.52838  3.98326  5.86590  4.58030    152 d - - -
      2.68618  4.42225  2.77519  2.73123  3.46354  2.40513  3.72494  3.29354  2.67741  2.69355  4.24690  2.90347  2.73739  3.18146  2.89801  2.37887  2.77519  2.98518  4.58477  3.61503
      0.02633  4.04612  4.76847  0.61958  0.77255  0.48576  0.95510
139   2.51508  4.35935  3.59186  3.35616  4.13168  3.19183  4.35900  3.28039  3.30256  3.13762  4.17966  3.53474  3.91053  3.68940  3.55061  2.71864  0.90819  2.95635  5.55400  4.34937    153 t - - -
      2.68618  4.42225  2.77519  2.73123  3.46354  2.40513  3.72494  3.29354  2.67741  2.69355  4.24690  2.90347  2.73739  3.18146  2.89801  2.37887  2.77519  2.98518  4.58477  3.61503
      0.02633  4.04612  4.76847  0.61958  0.77255  0.48576  0.95510
140   2.90225  5.42059  1.32115  1.93014  4.73049  3.25698  3.76333  4.23139  2.73940  3.74237  4.57705  2.73021  2.97207  2.92283  3.31248  2.80797  3.18469  3.81178  5.90909  4.43041    154 d - - -
      2.68618  4.42225  2.77519  2.73123  3.46354  2.40513  3.72494  3.29354  2.67741  2.69355  4.24690  2.90347  2.73739  3.18146  2.89801  2.37887  2.77519  2.98518  4.58477  3.61503
      0.02633  4.04612  4.76847  0.61958  0.77255  0.48576  0.95510
141   3.48713  4.83899  4.47715  4.22754  0.76525  4.13020  3.96574  2.99278  4.13100  2.32415  3.69909  4.21883  4.58710  4.25179  4.20766  3.74611  3.78948  2.99573  4.16879  2.51628    155 f - - -
      2.68618  4.42225  2.77519  2.73123  3.46354  2.40513  3.72494  3.29354  2.67741  2.69355  4.24690  2.90347  2.73739  3.18146  2.89801  2.37887  2.77519  2.98518  4.58477  3.61503
      0.02633  4.04612  4.76847  0.61958  0.77255  0.48576  0.95510
142   2.51508  4.35935  3.59186  3.35616  4.13168  3.19183  4.35900  3.28039  3.30256  3.13762  4.17966  3.53474  3.91053  3.68940  3.55061  2.71864  0.90819  2.95635  5.55400  4.34937    156 t - - -
      2.68618  4.42225  2.77519  2.73123  3.46354  2.40513  3.72494  3.29354  2.67741  2.69355  4.24690  2.90347  2.73739  3.18146  2.89801  2.37887  2.77519  2.98518  4.58477  3.61503
      0.02633  4.04612  4.76847  0.61958  0.77255  0.48576  0.95510
143   2.02123  4.33366  3.31383  3.08507  4.26882  3.08375  4.21686  3.59181  3.14959  3.32703  4.24976  3.32995  1.24449  3.49128  3.45632  2.57421  2.87050  3.15832  5.62349  4.39924    157 p - - -
      2.68618  4.42225  2.77519  2.73123  3.46354  2.40513  3.72494  3.29354  2.67741  2.69355  4.24690  2.90347  2.73739  3.18146  2.89801  2.37887  2.77519  2.98518  4.58477  3.61503
      0.02633  4.04612  4.76847  0.61958  0.77255  0.48576  0.95510
144   2.44557  4.46870  2.95890  2.75437  4.29965  3.09175  4.02292  3.82808  2.92703  3.47221  4.31476  2.52682  3.78086  3.25478  3.29892  1.19624  2.87746  3.33628  5.61680  4.29215    158 s - - -
      2.68618  4.42225  2.77519  2.73123  3.46354  2.40513  3.72494  3.29354  2.67741  2.69355  4.24690  2.90347  2.73739  3.18146  2.89801  2.37887  2.77519  2.98518  4.58477  3.61503
      0.02633  4.04612  4.76847  0.61958  0.77255  0.48576  0.95510
145   3.20253  4.98347  3.31174  3.09232  3.41943  3.60558  0.82340  3.93238  2.93123  3.40304  4.46212  3.49333  4.16927  3.46173  3.18487  3.27259  3.51314  3.66436  4.88146  3.36606    159 h - - -
      2.68618  4.42225  2.77519  2.73123  3.46354  2.40513  3.72494  3.29354  2.67741  2.69355  4.24690  2.90347  2.73739  3.18146  2.89801  2.37887  2.77519  2.98518  4.58477  3.61503
      0.02633  4.04612  4.76847  0.61958  0.77255  0.48576  0.95510
146   3.12570  4.49004  4.69177  4.24964  3.48450  4.34647  4.95290  0.97316  4.08478  1.98910  3.33991  4.46374  4.71504  4.38732  4.27377  3.77010  3.41222  1.70303  5.46578  4.22958    160 i - - -
      2.68618  4.42225  2.77519  2.73123  3.46354  2.40513  3.72494  3.29354  2.67741  2.69355  4.24690  2.90347  2.73739  3.18146  2.89801  2.37887  2.77519  2.98518  4.58477  3.61503
      0.02633  4.04612  4.76847  0.61958  0.77255  0.48576  0.95510
147   3.17417  4.50297  4.76881  4.24458  2.63988  4.43462  4.69975  1.15129  4.11735  1.71744  3.12352  4.44822  4.69414  4.28646  4.26524  3.79115  3.41481  1.94850  5.06922  3.77169    161 i - - -
      2.68618  4.42225  2.77519  2.73123  3.46354  2.40513  3.72494  3.29354  2.67741  2.69355  4.24690  2.90347  2.73739  3.18146  2.89801  2.37887  2.77519  2.98518  4.58477  3.61503
      0.02633  4.04612  4.76847  0.61958  0.77255  0.48576  0.95510
148   2.91853  5.32206  2.32738  1.17127  4.64252  3.28690  3.78556  4.14659  2.67906  3.68690  4.54656  2.39159  3.88256  2.95609  3.16378  2.84701  3.20570  3.75114  5.82397  4.38869    162 e - - -
      2.68618  4.42225  2.77519  2.73123  3.46354  2.40513  3.72494  3.29354  2.67741  2.69355  4.24690  2.90347  2.73739  3.18146  2.89801  2.37887  2.77519  2.98518  4.58477  3.61503
      0.02633  4.04612  4.76847  0.61958  0.77255  0.48576  0.95510
149   3.48713  4.83899  4.47715  4.22754  0.76525  4.13020  3.96574  2.99278  4.13100  2.32415  3.69909  4.21883  4.58710  4.25179  4.20766  3.74611  3.78948  2.99573  4.16879  2.51628    163 f - - -
      2.68618  4.42225  2.77519  2.73123  3.46354  2.40513  3.72494  3.29354  2.67741  2.69355  4.24690  2.90347  2.73739  3.18146  2.89801  2.37887  2.77519  2.98518  4.58477  3.61503
      0.02633  4.04612  4.76847  0.61958  0.77255  0.48576  0.95510
150   3.50686  4.92515  4.09883  3.86368  2.32032  4.03574  3.71314  3.49287  3.71639  2.90117  4.15040  3.94349  4.50689  3.98017  3.86318  3.62504  3.79515  3.37202  3.96972  0.71954    164 y - - -
      2.68618  4.42225  2.77519  2.73123  3.46354  2.40513  3.72494  3.29354  2.67741  2.69355  4.24690  2.90347  2.73739  3.18146  2.89801  2.37887  2.77519  2.98518  4.58477  3.61503
      0.02633  4.04612  4.76847  0.61958  0.77255  0.48576  0.95510
151   2.88961  4.37950  4.15479  3.58211  2.65625  3.98269  4.22385  2.43039  3.46215  1.31603  2.39841  3.87750  4.29276  3.12379  3.68287  3.27248  3.11484  2.44116  4.85349  3.65807    165 l - - -
      2.68618  4.42225  2.77519  2.73123  3.46354  2.40513  3.72494  3.29354  2.67741  2.69355  4.24690  2.90347  2.73739  3.18146  2.89801  2.37887  2.77519  2.98518  4.58477  3.61503
      0.02633  4.04612  4.76847  0.61958  0.77255  0.48576  0.95510
152   2.44557  4.46870  2.95890  2.75437  4.29965  3.09175  4.02292  3.82808  2.92703  3.47221  4.31476  2.52682  3.78086  3.25478  3.29892  1.19624  2.87746  3.33628  5.61680  4.29215    166 s - - -
      2.68618  4.42225  2.77519  2.73123  3.46354  2.40513  3.72494  3.29354  2.67741  2.69355  4.24690  2.90347  2.73739  3.18146  2.89801  2.37887  2.77519  2.98518  4.58477  3.61503
      0.02633  4.04612  4.76847  0.61958  0.77255  0.48576  0.95510
153   3.73503  4.91820  4.74855  4.44926  1.63732  4.46159  3.43288  3.48109  4.27543  2.80960  4.04842  4.10629  4.75271  4.18425  4.26733  3.81978  3.94580  3.37959  2.67303  0.94406    167 y - - -
      2.68618  4.42225  2.77519  2.73123  3.46354  2.40513  3.72494  3.29354  2.67741  2.69355  4.24690  2.90347  2.73739  3.18146  2.89801  2.37887  2.77519  2.98518  4.58477  3.61503
      0.02633  4.04612  4.76847  0.61958  0.77255  0.48576  0.95510
154   2.99788  4.72282  3.62437  3.49084  4.46579  3.40066  4.55136  4.05922  3.56366  3.66585  4.72523  3.77854  0.55991  3.94710  3.78680  3.17151  3.45373  3.68421  5.60149  4.58984    168 P - - -
      2.68618  4.42225  2.77519  2.73123  3.46354  2.40513  3.72494  3.29354  2.67741  2.69355  4.24690  2.90347  2.73739  3.18146  2.89801  2.37887  2.77519  2.98518  4.58477  3.61503
      0.02633  4.04612  4.76847  0.61958  0.77255  0.48576  0.95510
155   3.18556  4.49864  4.83311  4.28840  3.33283  4.51526  4.91869  1.14419  4.15656  1.65347  2.81020  4.53438  4.75174  4.35353  4.32231  3.87065  3.42471  1.78592  5.34327  4.21016    169 i - - -
      2.68618  4.42225  2.77519  2.73123  3.46354  2.40513  3.72494  3.29354  2.67741  2.69355  4.24690  2.90347  2.73739  3.18146  2.89801  2.37887  2.77519  2.98518  4.58477  3.61503
      0.02633  4.04612  4.76847  0.61958  0.77255  0.48576  0.95510
156   3.62479  4.90513  4.52641  4.20520  1.72603  4.35654  3.50028  3.35058  4.05877  2.69434  3.96129  4.04122  4.68339  4.08508  4.13477  3.73265  3.85434  3.27023  3.65685  0.88757    170 y - - -
      2.68618  4.42225  2.77519  2.73123  3.46354  2.40513  3.72494  3.29354  2.67741  2.69355  4.24690  2.90347  2.73739  3.18146  2.89801  2.37887  2.77519  2.98518  4.58477  3.61503
      0.02633  4.04612  4.76847  0.61958  0.77255  0.48576  0.95510
157   2.76935  4.27411  4.17504  3.67079  3.49538  3.85356  4.40301  1.31848  3.55102  2.28975  3.41464  3.90785  4.29468  3.84795  3.78723  2.73913  3.05790  1.74841  5.17195  3.94768    171 i - - -
      2.68618  4.42225  2.77519  2.73123  3.46354  2.40513  3.72494  3.29354  2.67741  2.69355  4.24690  2.90347  2.73739  3.18146  2.89801  2.37887  2.77519  2.98518  4.58477  3.61503
      0.02633  4.04612  4.76847  0.61958  0.77255  0.48576  0.95510
158   2.75357  4.26689  4.00365  3.45643  3.41442  3.85422  4.21601  1.47783  3.34288  2.27217  3.18093  3.10157  4.23144  3.64395  3.60665  3.15814  3.00319  1.82031  5.03342  3.81157    172 i - - -
      2.68618  4.42225  2.77519  2.73123  3.46354  2.40513  3.72494  3.29354  2.67741  2.69355  4.24690  2.90347  2.73739  3.18146  2.89801  2.37887  2.77519  2.98518  4.58477  3.61503
      0.02633  4.04612  4.76847  0.61958  0.77255  0.48576  0.95510
159   2.57385  3.84467  4.11571  3.53530  2.81392  3.78457  4.10253  2.08222  3.42326  1.88005  2.98711  3.75934  4.14161  3.64548  3.61440  3.07639  2.30822  2.09097  4.77635  3.58946    173 l - - -
      2.68618  4.42225  2.77519  2.73123  3.46354  2.40513  3.72494  3.29354  2.67741  2.69355  4.24690  2.90347  2.73739  3.18146  2.89801  2.37887  2.77519  2.98518  4.58477  3.61503
      0.02633  4.04612  4.76847  0.61958  0.77255  0.48576  0.95510
```

```
160   1.96585  4.25997  3.33657  3.17883  4.44159  1.07011  4.33464  3.82290  3.34599  3.56019  4.42587  3.35348  3.76040  3.62719  3.63819  2.50237  2.83130  3.27444  5.76175  4.56909    174 g - - -
      2.68618  4.42225  2.77519  2.73123  3.46354  2.40513  3.72494  3.29354  2.67741  2.69355  4.24690  2.90347  2.73739  3.18146  2.89801  2.37887  2.77519  2.98518  4.58477  3.61503
      0.02633  4.04612  4.76847  0.61958  0.77255  0.48576  0.95510
161   2.56549  4.12019  3.79127  3.21637  2.96558  2.75263  3.91443  2.41555  3.12747  2.25483  3.23065  3.51176  3.99924  3.38786  3.38395  2.82604  2.73893  1.90822  4.31117  3.51053    175 v - - -
      2.68618  4.42225  2.77519  2.73123  3.46354  2.40513  3.72494  3.29354  2.67741  2.69355  4.24690  2.90347  2.73739  3.18146  2.89801  2.37887  2.77519  2.98518  4.58477  3.61503
      0.02633  4.04612  4.76847  0.61958  0.77255  0.48576  0.95510
162   1.54404  2.91401  3.78588  3.38377  4.11200  1.77138  4.27831  3.48302  3.33509  3.21083  4.05321  3.42667  3.71206  3.60161  3.60941  2.25706  2.68173  3.00495  5.50516  4.32123    176 a - - -
      2.68618  4.42225  2.77519  2.73123  3.46354  2.40513  3.72494  3.29354  2.67741  2.69355  4.24690  2.90347  2.73739  3.18146  2.89801  2.37887  2.77519  2.98518  4.58477  3.61503
      0.02633  4.04612  4.76847  0.61958  0.77255  0.48576  0.95510
163   1.37245  3.72720  3.67186  3.27033  4.22035  2.18004  4.23990  3.61966  3.24380  3.31740  4.14081  3.36913  3.69939  3.52305  3.55058  1.88430  2.68461  3.09828  5.58072  4.38408    177 a - - -
      2.68618  4.42225  2.77519  2.73123  3.46354  2.40513  3.72494  3.29354  2.67741  2.69355  4.24690  2.90347  2.73739  3.18146  2.89801  2.37887  2.77519  2.98518  4.58477  3.61503
      0.02633  4.04612  4.76847  0.61958  0.77255  0.48576  0.95510
164   2.97591  4.35689  4.43385  3.91298  1.54768  4.02624  3.80837  2.68209  3.76275  1.93653  2.90857  3.94449  4.35279  3.87527  3.86186  3.33789  3.20131  2.58881  3.09217  2.40324    178 f - - -
      2.68618  4.42225  2.77519  2.73123  3.46354  2.40513  3.72494  3.29354  2.67741  2.69355  4.24690  2.90347  2.73739  3.18146  2.89801  2.37887  2.77519  2.98518  4.58477  3.61503
      0.02633  4.04612  4.76847  0.61958  0.77255  0.48576  0.95510
165   2.98698  4.37154  4.56156  3.98072  2.67516  4.18029  4.48671  2.06812  3.85953  1.39513  2.08656  4.19388  4.45465  4.00402  3.99536  3.49329  3.04560  2.09423  4.96068  3.84191    179 l - - -
      2.68618  4.42225  2.77519  2.73123  3.46354  2.40513  3.72494  3.29354  2.67741  2.69355  4.24690  2.90347  2.73739  3.18146  2.89801  2.37887  2.77519  2.98518  4.58477  3.61503
      0.02633  4.04612  4.76847  0.61958  0.77255  0.48576  0.95510
166   3.31495  4.71042  4.30545  3.85688  2.09824  4.16340  3.28515  3.24027  3.66623  2.28702  3.82859  3.86519  4.49255  3.83178  3.82080  3.48236  3.53515  3.09256  3.28163  1.08541    180 y - - -
      2.68618  4.42225  2.77519  2.73123  3.46354  2.40513  3.72494  3.29354  2.67741  2.69355  4.24690  2.90347  2.73739  3.18146  2.89801  2.37887  2.77519  2.98518  4.58477  3.61503
      0.02633  4.04612  4.76847  0.61958  0.77255  0.48576  0.95510
167   1.06945  4.16127  3.56705  3.27147  4.23840  2.82295  4.28516  3.48192  3.28149  3.29941  4.16887  3.38502  3.73735  3.58057  3.56591  2.44666  2.28470  3.01814  5.62224  4.42474    181 a - - -
      2.68618  4.42225  2.77519  2.73123  3.46354  2.40513  3.72494  3.29354  2.67741  2.69355  4.24690  2.90347  2.73739  3.18146  2.89801  2.37887  2.77519  2.98518  4.58477  3.61503
      0.02633  4.04612  4.76847  0.61958  0.77255  0.48576  0.95510
168   2.75682  4.93709  3.18846  2.56961  4.23097  3.52713  3.12294  3.32043  1.74795  3.17802  3.71608  3.04765  3.89827  2.74819  1.94329  2.77688  2.96255  3.30939  5.34671  4.07067    182 k - - -
      2.68618  4.42225  2.77519  2.73123  3.46354  2.40513  3.72494  3.29354  2.67741  2.69355  4.24690  2.90347  2.73739  3.18146  2.89801  2.37887  2.77519  2.98518  4.58477  3.61503
      0.02633  4.04612  4.76847  0.61958  0.77255  0.48576  0.95510
169   2.51508  4.35935  3.59186  3.35616  4.13168  3.19183  4.35900  3.28039  3.30256  3.13762  4.17966  3.53474  3.91053  3.68940  3.55061  2.71864  0.90819  2.95635  5.55400  4.34937    183 t - - -
      2.68618  4.42225  2.77519  2.73123  3.46354  2.40513  3.72494  3.29354  2.67741  2.69355  4.24690  2.90347  2.73739  3.18146  2.89801  2.37887  2.77519  2.98518  4.58477  3.61503
      0.02633  4.04612  4.76847  0.61958  0.77255  0.48576  0.95510
170   3.21057  5.03121  3.71984  3.19868  4.43025  3.66102  3.96379  3.98677  2.32612  3.47126  4.46970  3.54368  4.15769  3.17794  0.74292  3.28809  3.46631  3.70390  5.44441  4.32290    184 r - - -
      2.68618  4.42225  2.77519  2.73123  3.46354  2.40513  3.72494  3.29354  2.67741  2.69355  4.24690  2.90347  2.73739  3.18146  2.89801  2.37887  2.77519  2.98518  4.58477  3.61503
      0.02633  4.04612  4.76847  0.61958  0.77255  0.48576  0.95510
171   3.31642  4.65545  4.81165  4.28955  3.14481  4.54380  4.87520  1.81024  4.10143  0.90410  2.97305  4.55585  4.76476  4.29462  4.24740  3.92238  3.55369  2.14483  5.22065  4.07885    185 l - - -
      2.68618  4.42225  2.77519  2.73123  3.46354  2.40513  3.72494  3.29354  2.67741  2.69355  4.24690  2.90347  2.73739  3.18146  2.89801  2.37887  2.77519  2.98518  4.58477  3.61503
      0.02633  4.04612  4.76847  0.61958  0.77255  0.48576  0.95510
172   2.99788  4.72282  3.62437  3.49084  4.46579  3.40066  4.55136  4.05922  3.56366  3.66585  4.72523  3.77854  0.55991  3.94710  3.78680  3.17151  3.45373  3.68421  5.60149  4.58984    186 P - - -
      2.68618  4.42225  2.77519  2.73123  3.46354  2.40513  3.72494  3.29354  2.67741  2.69355  4.24690  2.90347  2.73739  3.18146  2.89801  2.37887  2.77519  2.98518  4.58477  3.61503
      0.02633  4.04612  4.76847  0.61958  0.77255  0.48576  0.95510
173   2.51458  4.16754  3.65036  3.07716  3.07183  3.59055  3.75446  2.63315  3.00379  2.13707  3.07762  3.41818  3.96564  3.05570  3.29780  2.81892  2.46065  2.25848  4.75638  2.90495    187 l - - -
      2.68618  4.42225  2.77519  2.73123  3.46354  2.40513  3.72494  3.29354  2.67741  2.69355  4.24690  2.90347  2.73739  3.18146  2.89801  2.37887  2.77519  2.98518  4.58477  3.61503
      0.02633  4.04612  4.76847  0.61958  0.77255  0.48576  0.95510
174   3.13348  4.51681  4.40434  3.92107  1.54477  4.11837  3.72511  2.81274  3.78240  2.24502  2.76317  3.95181  4.44040  3.89576  3.90796  3.43555  3.35898  2.73455  3.98863  1.68173    188 f - - -
      2.68614  4.42227  2.77522  2.73125  3.46356  2.40510  3.72497  3.29356  2.67743  2.69357  4.24692  2.90343  2.73742  3.18137  2.89803  2.37889  2.77517  2.98520  4.58479  3.61494
      0.10210  2.42401  4.76847  0.63885  0.75056  0.48576  0.95510
175   1.94983  4.24296  3.06254  2.53629  3.92514  2.97509  3.54024  3.32612  2.45024  2.96169  3.79680  3.01950  3.81440  2.59183  2.94247  2.45429  2.76743  3.01442  5.23442  3.93264    192 a - - -
      2.68618  4.42225  2.77519  2.73123  3.46354  2.40513  3.72494  3.29354  2.67741  2.69355  4.24690  2.90347  2.73739  3.18146  2.89801  2.37887  2.77519  2.98518  4.58477  3.61503
      0.02633  4.04612  4.76847  0.61958  0.77255  0.48576  0.95510
176   2.89089  5.16424  3.12796  2.44041  4.55471  3.55776  3.22146  3.94512  1.38349  3.40688  4.22954  3.04571  3.94214  2.47166  2.19923  2.86763  3.08263  3.59042  5.49135  4.23575    193 k - - -
      2.68618  4.42225  2.77519  2.73123  3.46354  2.40513  3.72494  3.29354  2.67741  2.69355  4.24690  2.90347  2.73739  3.18146  2.89801  2.37887  2.77519  2.98518  4.58477  3.61503
      0.02633  4.04612  4.76847  0.61958  0.77255  0.48576  0.95510
177   2.30058  4.66035  2.95161  2.57521  4.20005  1.81682  3.78185  3.60803  2.27548  3.22109  4.05373  3.03096  3.81958  2.96094  2.81051  2.56127  2.88821  3.23289  5.44883  4.15251    194 g - - -
      2.68618  4.42225  2.77519  2.73123  3.46354  2.40513  3.72494  3.29354  2.67741  2.69355  4.24690  2.90347  2.73739  3.18146  2.89801  2.37887  2.77519  2.98518  4.58477  3.61503
      0.02633  4.04612  4.76847  0.61958  0.77255  0.48576  0.95510
178   2.66991  4.12344  4.11633  3.54957  2.50466  3.74979  3.52032  2.19680  3.42015  1.99691  3.23538  3.50174  4.11195  3.61513  3.58130  3.04199  2.90056  2.38838  3.10447  2.58897    195 l - - -
      2.68618  4.42225  2.77519  2.73123  3.46354  2.40513  3.72494  3.29354  2.67741  2.69355  4.24690  2.90347  2.73739  3.18146  2.89801  2.37887  2.77519  2.98518  4.58477  3.61503
      0.02633  4.04612  4.76847  0.61958  0.77255  0.48576  0.95510
179   2.59879  4.55534  3.14384  2.71135  3.68998  3.37800  3.31524  3.29617  2.63391  2.57960  3.83456  3.13966  3.87764  3.03951  2.99442  1.57667  2.89439  3.00076  5.09815  3.72398    196 s - - -
      2.68618  4.42225  2.77519  2.73123  3.46354  2.40513  3.72494  3.29354  2.67741  2.69355  4.24690  2.90347  2.73739  3.18146  2.89801  2.37887  2.77519  2.98518  4.58477  3.61503
      0.02633  4.04612  4.76847  0.61958  0.77255  0.48576  0.95510
180   3.21637  4.53818  4.83646  4.29090  3.25717  4.51724  4.90346  1.71033  4.16193  1.11346  3.05398  4.54060  4.74515  4.33090  4.31640  3.87273  3.45349  1.71392  5.29378  4.18089    197 l - - -
      2.68618  4.42225  2.77519  2.73123  3.46354  2.40513  3.72494  3.29354  2.67741  2.69355  4.24690  2.90347  2.73739  3.18146  2.89801  2.37887  2.77519  2.98518  4.58477  3.61503
      0.02633  4.04612  4.76847  0.61958  0.77255  0.48576  0.95510
181   2.13417  4.56273  3.11643  2.62478  3.89574  3.36887  3.74982  3.26487  2.57391  2.91848  3.44794  3.08124  2.25012  2.67803  2.97134  2.65968  2.83572  2.96741  5.23466  3.94897    198 a - - -
      2.68618  4.42225  2.77519  2.73123  3.46354  2.40513  3.72494  3.29354  2.67741  2.69355  4.24690  2.90347  2.73739  3.18146  2.89801  2.37887  2.77519  2.98518  4.58477  3.61503
      0.02633  4.04612  4.76847  0.61958  0.77255  0.48576  0.95510
182   3.27045  4.63161  4.45038  3.99770  1.89862  4.20939  3.67447  2.90153  3.85561  1.78024  3.52885  3.99205  4.52178  3.95090  3.97595  3.53468  3.49382  2.84032  3.90103  1.37555    199 y - - -
```

```
     2.68618  4.42225  2.77519  2.73123  3.46354  2.40513  3.72494  3.29354  2.67741  2.69355  4.24690  2.90347  2.73739  3.18146  2.89801  2.37887  2.77519  2.98518  4.58477  3.61503
     0.02633  4.04612  4.76847  0.61958  0.77255  0.48576  0.95510
183  2.50074  4.25616  4.03187  3.46808  3.31895  3.83767  4.19333  2.25352  3.36701  1.60350  2.87372  3.76160  4.20601  3.63259  3.61837  3.13471  2.62058  1.91503  4.96536  3.77937    200 l - - -
     2.68618  4.42225  2.77519  2.73123  3.46354  2.40513  3.72494  3.29354  2.67741  2.69355  4.24690  2.90347  2.73739  3.18146  2.89801  2.37887  2.77519  2.98518  4.58477  3.61503
     0.02633  4.04612  4.76847  0.61958  0.77255  0.48576  0.95510
184  3.11690  4.44291  4.78698  4.22468  3.25338  4.41267  4.78305  1.67531  4.10980  1.45724  2.42513  4.44466  4.65586  4.26181  4.24928  3.75033  3.34891  1.57750  5.20719  4.09744    201 l - - -
     2.68618  4.42225  2.77519  2.73123  3.46354  2.40513  3.72494  3.29354  2.67741  2.69355  4.24690  2.90347  2.73739  3.18146  2.89801  2.37887  2.77519  2.98518  4.58477  3.61503
     0.02633  4.04612  4.76847  0.61958  0.77255  0.48576  0.95510
185  2.27038  4.30561  3.44745  2.62929  3.33099  3.55049  3.83074  2.71207  2.84217  2.11447  3.41923  3.29768  3.94124  3.15340  3.19687  2.74553  2.63638  2.23721  4.92104  3.68440    202 l - - -
     2.68618  4.42225  2.77519  2.73123  3.46354  2.40513  3.72494  3.29354  2.67741  2.69355  4.24690  2.90347  2.73739  3.18146  2.89801  2.37887  2.77519  2.98518  4.58477  3.61503
     0.02633  4.04612  4.76847  0.61958  0.77255  0.48576  0.95510
186  2.42625  4.11775  3.94235  3.37348  2.88806  3.66863  3.99894  2.43671  3.26473  2.25868  3.22733  3.62372  4.05729  3.51554  3.48887  2.88451  2.75989  1.58668  4.23569  3.51191    203 v - - -
     2.68618  4.42225  2.77519  2.73123  3.46354  2.40513  3.72494  3.29354  2.67741  2.69355  4.24690  2.90347  2.73739  3.18146  2.89801  2.37887  2.77519  2.98518  4.58477  3.61503
     0.02633  4.04612  4.76847  0.61958  0.77255  0.48576  0.95510
187  2.31791  4.14342  3.85053  3.28543  2.88703  3.01868  4.00018  2.16302  3.20146  2.32207  3.27661  3.57223  4.04264  3.46244  3.45949  2.93581  2.74647  1.88562  4.81791  3.61081    204 v - - -
     2.68618  4.42225  2.77519  2.73123  3.46354  2.40513  3.72494  3.29354  2.67741  2.69355  4.24690  2.90347  2.73739  3.18146  2.89801  2.37887  2.77519  2.98518  4.58477  3.61503
     0.02633  4.04612  4.76847  0.61958  0.77255  0.48576  0.95510
188  2.50148  4.37634  3.33054  2.89847  3.63154  1.65741  3.88783  3.23329  2.84475  2.90607  3.79187  3.24998  3.85804  3.20250  3.18584  2.39408  2.84314  2.92111  3.42143  3.71975    205 g - - -
     2.68618  4.42225  2.77519  2.73123  3.46354  2.40513  3.72494  3.29354  2.67741  2.69355  4.24690  2.90347  2.73739  3.18146  2.89801  2.37887  2.77519  2.98518  4.58477  3.61503
     0.02633  4.04612  4.76847  0.61958  0.77255  0.48576  0.95510
189  2.99788  4.72282  3.62437  3.49084  4.46579  3.40066  4.55136  4.05922  3.56366  3.66585  4.72523  3.77854  0.55991  3.94710  3.78680  3.17151  3.45373  3.68421  5.60149  4.58984    206 P - - -
     2.68618  4.42225  2.77519  2.73123  3.46354  2.40513  3.72494  3.29354  2.67741  2.69355  4.24690  2.90347  2.73739  3.18146  2.89801  2.37887  2.77519  2.98518  4.58477  3.61503
     0.02633  4.04612  4.76847  0.61958  0.77255  0.48576  0.95510
190  2.81008  4.41344  4.45629  3.91153  1.64236  4.14461  4.32017  2.03302  3.78473  1.67068  3.08919  4.11406  4.45184  3.96446  3.94950  3.46704  3.24165  2.22519  4.76332  3.41266    207 f - - -
     2.68618  4.42225  2.77519  2.73123  3.46354  2.40513  3.72494  3.29354  2.67741  2.69355  4.24690  2.90347  2.73739  3.18146  2.89801  2.37887  2.77519  2.98518  4.58477  3.61503
     0.02633  4.04612  4.76847  0.61958  0.77255  0.48576  0.95510
191  3.11396  4.63711  4.24001  3.84985  3.29524  3.96585  4.54491  2.48869  3.60744  1.86658  1.08039  4.10491  4.43026  3.97953  3.79171  3.47969  3.42418  2.51429  5.15731  3.93282    208 m - - -
     2.68618  4.42225  2.77519  2.73123  3.46354  2.40513  3.72494  3.29354  2.67741  2.69355  4.24690  2.90347  2.73739  3.18146  2.89801  2.37887  2.77519  2.98518  4.58477  3.61503
     0.02633  4.04612  4.76847  0.61958  0.77255  0.48576  0.95510
192  3.16398  4.50368  4.72717  4.22584  2.63088  4.39356  4.67741  1.11604  4.10114  1.80481  3.16295  4.42290  4.68006  4.28290  4.25383  3.76580  3.41310  1.94660  5.05378  3.72643    209 i - - -
     2.68618  4.42225  2.77519  2.73123  3.46354  2.40513  3.72494  3.29354  2.67741  2.69355  4.24690  2.90347  2.73739  3.18146  2.89801  2.37887  2.77519  2.98518  4.58477  3.61503
     0.02633  4.04612  4.76847  0.61958  0.77255  0.48576  0.95510
193  3.17485  4.50809  4.81116  4.24454  2.19402  4.41596  4.66866  1.85144  4.12019  1.19125  2.93707  4.44387  4.63944  4.21753  4.22563  3.75011  3.39855  2.07534  5.00751  3.82825    210 l - - -
     2.68618  4.42225  2.77519  2.73123  3.46354  2.40513  3.72494  3.29354  2.67741  2.69355  4.24690  2.90347  2.73739  3.18146  2.89801  2.37887  2.77519  2.98518  4.58477  3.61503
     0.02633  4.04612  4.76847  0.61958  0.77255  0.48576  0.95510
194  2.50446  4.35095  3.51450  3.16003  3.88146  3.26632  4.16682  3.01683  3.10342  2.83859  3.87865  3.42684  1.45675  3.46786  3.40557  2.70623  2.91140  2.17273  5.37236  4.11405    211 p - - -
     2.68618  4.42225  2.77519  2.73123  3.46354  2.40513  3.72494  3.29354  2.67741  2.69355  4.24690  2.90347  2.73739  3.18146  2.89801  2.37887  2.77519  2.98518  4.58477  3.61503
     0.02633  4.04612  4.76847  0.61958  0.77255  0.48576  0.95510
195  2.60538  4.67005  2.91267  2.57491  4.01227  3.30628  3.79178  3.49362  2.61990  2.69825  4.02107  1.68142  3.84511  2.99948  3.01482  2.36166  2.91834  3.15542  5.34742  3.99133    212 n - - -
     2.68618  4.42225  2.77519  2.73123  3.46354  2.40513  3.72494  3.29354  2.67741  2.69355  4.24690  2.90347  2.73739  3.18146  2.89801  2.37887  2.77519  2.98518  4.58477  3.61503
     0.02633  4.04612  4.76847  0.61958  0.77255  0.48576  0.95510
196  3.08325  4.42662  4.74060  4.25809  3.59748  4.37878  4.99144  1.57502  4.13580  2.12156  3.41876  4.47565  4.72825  4.42147  4.34392  3.77457  3.36081  0.97550  5.54684  4.32386    213 v - - -
     2.68618  4.42225  2.77519  2.73123  3.46354  2.40513  3.72494  3.29354  2.67741  2.69355  4.24690  2.90347  2.73739  3.18146  2.89801  2.37887  2.77519  2.98518  4.58477  3.61503
     0.02633  4.04612  4.76847  0.61958  0.77255  0.48576  0.95510
197  2.07589  4.28337  3.31825  3.17586  4.45215  1.00884  4.34348  3.84461  3.35473  3.57985  4.45442  3.36056  3.77446  3.63988  3.64405  2.52739  2.85814  3.29897  5.76445  4.57456    214 g - - -
     2.68618  4.42225  2.77519  2.73123  3.46354  2.40513  3.72494  3.29354  2.67741  2.69355  4.24690  2.90347  2.73739  3.18146  2.89801  2.37887  2.77519  2.98518  4.58477  3.61503
     0.02633  4.04612  4.76847  0.61958  0.77255  0.48576  0.95510
198  2.82210  4.40584  3.79521  2.81121  2.71627  3.83042  4.05894  2.51201  3.19132  1.34623  3.21317  3.63904  4.18995  3.49150  3.47168  3.12940  3.05676  2.43675  4.84135  3.54485    215 l - - -
     2.68618  4.42225  2.77519  2.73123  3.46354  2.40513  3.72494  3.29354  2.67741  2.69355  4.24690  2.90347  2.73739  3.18146  2.89801  2.37887  2.77519  2.98518  4.58477  3.61503
     0.02633  4.04612  4.76847  0.61958  0.77255  0.48576  0.95510
199  2.87658  4.87314  2.81898  2.72372  4.26492  3.30782  4.07476  4.01631  2.99504  3.64428  4.60102  0.88372  3.96968  3.35056  3.35324  2.93886  3.26586  3.62075  5.55106  4.20291    216 n - - -
     2.68618  4.42225  2.77519  2.73123  3.46354  2.40513  3.72494  3.29354  2.67741  2.69355  4.24690  2.90347  2.73739  3.18146  2.89801  2.37887  2.77519  2.98518  4.58477  3.61503
     0.02633  4.04612  4.76847  0.61958  0.77255  0.48576  0.95510
200  3.11182  5.24854  2.57453  0.80143  4.61810  3.39338  3.99965  4.10707  2.90285  3.69774  4.66764  3.04509  4.00728  3.21392  3.32714  3.07859  3.41853  3.77429  5.75689  4.48345    217 e - - -
     2.68618  4.42225  2.77519  2.73123  3.46354  2.40513  3.72494  3.29354  2.67741  2.69355  4.24690  2.90347  2.73739  3.18146  2.89801  2.37887  2.77519  2.98518  4.58477  3.61503
     0.02633  4.04612  4.76847  0.61958  0.77255  0.48576  0.95510
201  3.46437  4.79662  4.50679  4.13159  1.89524  4.23913  3.63149  3.16609  3.92995  2.49962  3.76783  4.05045  4.59421  4.04566  4.02452  3.65398  3.70582  3.11032  1.21161  2.12460    218 w - - -
     2.68618  4.42225  2.77519  2.73123  3.46354  2.40513  3.72494  3.29354  2.67741  2.69355  4.24690  2.90347  2.73739  3.18146  2.89801  2.37887  2.77519  2.98518  4.58477  3.61503
     0.02633  4.04612  4.76847  0.61958  0.77255  0.48576  0.95510
202  2.89498  4.64988  3.60405  3.52716  4.66000  0.50522  4.63379  4.30482  3.73186  3.93620  4.91564  3.76881  4.04640  4.04253  3.94591  3.07238  3.38974  3.79962  5.70617  4.76884    219 G - - -
     2.68618  4.42225  2.77519  2.73123  3.46354  2.40513  3.72494  3.29354  2.67741  2.69355  4.24690  2.90347  2.73739  3.18146  2.89801  2.37887  2.77519  2.98518  4.58477  3.61503
     0.02633  4.04612  4.76847  0.61958  0.77255  0.48576  0.95510
203  3.20253  4.98347  3.31174  3.09232  3.41943  3.60558  0.82340  3.93238  2.93123  3.40304  4.46212  3.49333  4.16927  3.46173  3.18487  3.27259  3.51314  3.66436  4.88146  3.36606    220 h - - -
     2.68618  4.42225  2.77519  2.73123  3.46354  2.40513  3.72494  3.29354  2.67741  2.69355  4.24690  2.90347  2.73739  3.18146  2.89801  2.37887  2.77519  2.98518  4.58477  3.61503
     0.02633  4.04612  4.76847  0.61958  0.77255  0.48576  0.95510
204  2.31159  4.22890  3.44470  3.15902  4.18274  3.03819  4.20802  3.48194  3.15078  3.26726  4.15277  3.34245  3.76491  3.49339  3.44350  2.13264  1.19591  3.04268  5.56639  4.33208    221 t - - -
     2.68618  4.42225  2.77519  2.73123  3.46354  2.40513  3.72494  3.29354  2.67741  2.69355  4.24690  2.90347  2.73739  3.18146  2.89801  2.37887  2.77519  2.98518  4.58477  3.61503
```

```
      0.02633  4.04612  4.76847  0.61958  0.77255  0.48576  0.95510
205   2.38026  4.33368  3.68177  3.18512  1.70244  3.60224  3.89710  2.76616  3.03296  2.41282  3.44323  3.49186  4.04751  3.38316  2.87683  2.93012  2.95002  2.58632  4.69719  3.29436    222 f - - -
      2.68618  4.42225  2.77519  2.73123  3.46354  2.40513  3.72494  3.29354  2.67741  2.69355  4.24690  2.90347  2.73739  3.18146  2.89801  2.37887  2.77519  2.98518  4.58477  3.61503
      0.02633  4.04612  4.76847  0.61958  0.77255  0.48576  0.95510
206   3.63987  4.94992  4.36684  4.11814  2.86519  3.93328  4.13009  3.67429  3.83837  3.03776  4.29951  4.23296  4.46616  4.21695  3.93507  3.84178  3.94978  3.56933  0.59760  2.85873    223 W - - -
      2.68618  4.42225  2.77519  2.73123  3.46354  2.40513  3.72494  3.29354  2.67741  2.69355  4.24690  2.90347  2.73739  3.18146  2.89801  2.37887  2.77519  2.98518  4.58477  3.61503
      0.02633  4.04612  4.76847  0.61958  0.77255  0.48576  0.95510
207   3.21705  4.58307  4.62097  4.14629  1.34071  4.29989  4.22867  1.96927  4.00768  1.80547  3.16837  4.25912  4.60299  4.13819  4.13252  3.66465  3.46181  2.35323  4.50598  2.98002    224 f - - -
      2.68618  4.42225  2.77519  2.73123  3.46354  2.40513  3.72494  3.29354  2.67741  2.69355  4.24690  2.90347  2.73739  3.18146  2.89801  2.37887  2.77519  2.98518  4.58477  3.61503
      0.02633  4.04612  4.76847  0.61958  0.77255  0.48576  0.95510
208   2.80659  4.43317  3.81756  3.23721  3.31973  3.78913  4.02303  2.59779  2.92005  1.90566  1.80286  3.58947  4.15176  3.36885  2.72667  3.08740  3.03978  2.51953  4.95883  3.74394    225 m - - -
      2.68618  4.42225  2.77519  2.73123  3.46354  2.40513  3.72494  3.29354  2.67741  2.69355  4.24690  2.90347  2.73739  3.18146  2.89801  2.37887  2.77519  2.98518  4.58477  3.61503
      0.02633  4.04612  4.76847  0.61958  0.77255  0.48576  0.95510
209   3.11182  5.24854  2.57453  0.80143  4.61810  3.39338  3.99965  4.10707  2.90285  3.69774  4.66764  3.04509  4.00728  3.21392  3.32714  3.07859  3.41853  3.77429  5.75689  4.48345    226 e - - -
      2.68618  4.42225  2.77519  2.73123  3.46354  2.40513  3.72494  3.29354  2.67741  2.69355  4.24690  2.90347  2.73739  3.18146  2.89801  2.37887  2.77519  2.98518  4.58477  3.61503
      0.02633  4.04612  4.76847  0.61958  0.77255  0.48576  0.95510
210   3.11182  5.24854  2.57453  0.80143  4.61810  3.39338  3.99965  4.10707  2.90285  3.69774  4.66764  3.04509  4.00728  3.21392  3.32714  3.07859  3.41853  3.77429  5.75689  4.48345    227 e - - -
      2.68618  4.42225  2.77519  2.73123  3.46354  2.40513  3.72494  3.29354  2.67741  2.69355  4.24690  2.90347  2.73739  3.18146  2.89801  2.37887  2.77519  2.98518  4.58477  3.61503
      0.02633  4.04612  4.76847  0.61958  0.77255  0.48576  0.95510
211   3.11811  4.49358  4.54108  4.01362  2.24901  4.19262  4.03825  2.54571  3.85472  1.12293  3.11763  4.11104  4.47944  3.97633  3.96738  3.51436  3.34080  2.57903  3.17085  2.81297    228 l - - -
      2.68618  4.42225  2.77519  2.73123  3.46354  2.40513  3.72494  3.29354  2.67741  2.69355  4.24690  2.90347  2.73739  3.18146  2.89801  2.37887  2.77519  2.98518  4.58477  3.61503
      0.02633  4.04612  4.76847  0.61958  0.77255  0.48576  0.95510
212   3.48713  4.83899  4.47715  4.22754  0.76525  4.13020  3.96574  2.99278  4.13100  2.32415  3.69909  4.21883  4.58710  4.25179  4.20766  3.74611  3.78948  2.99573  4.16879  2.51628    229 f - - -
      2.68618  4.42225  2.77519  2.73123  3.46354  2.40513  3.72494  3.29354  2.67741  2.69355  4.24690  2.90347  2.73739  3.18146  2.89801  2.37887  2.77519  2.98518  4.58477  3.61503
      0.02633  4.04612  4.76847  0.61958  0.77255  0.48576  0.95510
213   1.99441  4.23912  4.03044  3.60921  3.76208  3.50899  4.45228  2.25914  3.52568  2.55897  3.65076  3.76963  4.11273  3.82989  3.77863  2.92195  2.81990  1.25359  5.39849  4.18819    230 v - - -
      2.68618  4.42225  2.77519  2.73123  3.46354  2.40513  3.72494  3.29354  2.67741  2.69355  4.24690  2.90347  2.73739  3.18146  2.89801  2.37887  2.77519  2.98518  4.58477  3.61503
      0.02633  4.04612  4.76847  0.61958  0.77255  0.48576  0.95510
214   1.03356  4.39698  3.20412  2.80235  4.16708  3.16646  4.14442  3.34861  3.05899  3.18799  4.13259  3.29029  3.85094  3.41354  3.37892  2.64947  2.91336  2.99641  5.55002  4.30710    231 a - - -
      2.68618  4.42225  2.77519  2.73123  3.46354  2.40513  3.72494  3.29354  2.67741  2.69355  4.24690  2.90347  2.73739  3.18146  2.89801  2.37887  2.77519  2.98518  4.58477  3.61503
      0.02633  4.04612  4.76847  0.61958  0.77255  0.48576  0.95510
215   2.99788  4.72282  3.62437  3.49084  4.46579  3.40066  4.55136  4.05922  3.56366  3.66585  4.72523  3.77854  0.55991  3.94710  3.78680  3.17151  3.45373  3.68421  5.60149  4.58984    232 P - - -
      2.68618  4.42225  2.77519  2.73123  3.46354  2.40513  3.72494  3.29354  2.67741  2.69355  4.24690  2.90347  2.73739  3.18146  2.89801  2.37887  2.77519  2.98518  4.58477  3.61503
      0.02633  4.04612  4.76847  0.61958  0.77255  0.48576  0.95510
216   3.32072  4.66760  4.78943  4.27171  3.13421  4.53087  4.85842  1.88095  4.07517  0.87536  2.96957  4.54052  4.75771  4.27767  4.22352  3.91255  3.55946  2.17420  5.20861  4.05848    233 l - - -
      2.68618  4.42225  2.77519  2.73123  3.46354  2.40513  3.72494  3.29354  2.67741  2.69355  4.24690  2.90347  2.73739  3.18146  2.89801  2.37887  2.77519  2.98518  4.58477  3.61503
      0.02633  4.04612  4.76847  0.61958  0.77255  0.48576  0.95510
217   3.20253  4.98347  3.31174  3.09232  3.41943  3.60558  0.82340  3.93238  2.93123  3.40304  4.46212  3.49333  4.16927  3.46173  3.18487  3.27259  3.51314  3.66436  4.88146  3.36606    234 h - - -
      2.68618  4.42225  2.77519  2.73123  3.46354  2.40513  3.72494  3.29354  2.67741  2.69355  4.24690  2.90347  2.73739  3.18146  2.89801  2.37887  2.77519  2.98518  4.58477  3.61503
      0.02633  4.04612  4.76847  0.61958  0.77255  0.48576  0.95510
218   3.58013  4.87139  4.51168  4.15441  1.98999  4.31330  3.48535  3.42404  3.92331  2.79055  4.00054  4.00679  4.64221  4.02546  4.00853  3.69061  3.80285  3.30872  1.48343  1.38436    235 y - - -
      2.68618  4.42225  2.77519  2.73123  3.46354  2.40513  3.72494  3.29354  2.67741  2.69355  4.24690  2.90347  2.73739  3.18146  2.89801  2.37887  2.77519  2.98518  4.58477  3.61503
      0.02633  4.04612  4.76847  0.61958  0.77255  0.48576  0.95510
219   2.40413  4.31697  3.37025  2.97047  3.97967  1.50803  4.02418  3.26518  2.92929  3.01565  3.33853  3.27192  3.80929  3.28423  3.26686  2.57579  2.40782  2.91861  5.37380  4.12459    236 g - - -
      2.68618  4.42225  2.77519  2.73123  3.46354  2.40513  3.72494  3.29354  2.67741  2.69355  4.24690  2.90347  2.73739  3.18146  2.89801  2.37887  2.77519  2.98518  4.58477  3.61503
      0.02633  4.04612  4.76847  0.61958  0.77255  0.48576  0.95510
220   3.48713  4.83899  4.47715  4.22754  0.76525  4.13020  3.96574  2.99278  4.13100  2.32415  3.69909  4.21883  4.58710  4.25179  4.20766  3.74611  3.78948  2.99573  4.16879  2.51628    237 f - - -
      2.68618  4.42225  2.77519  2.73123  3.46354  2.40513  3.72494  3.29354  2.67741  2.69355  4.24690  2.90347  2.73739  3.18146  2.89801  2.37887  2.77519  2.98518  4.58477  3.61503
      0.02633  4.04612  4.76847  0.61958  0.77255  0.48576  0.95510
221   2.94705  4.44528  4.36301  4.03467  3.68270  3.90043  4.80821  2.05762  3.90514  2.34157  3.62449  4.19576  4.45358  4.25388  4.10046  3.41740  3.30584  0.83114  5.49544  4.23648    238 v - - -
      2.68618  4.42225  2.77519  2.73123  3.46354  2.40513  3.72494  3.29354  2.67741  2.69355  4.24690  2.90347  2.73739  3.18146  2.89801  2.37887  2.77519  2.98518  4.58477  3.61503
      0.02633  4.04612  4.76847  0.61958  0.77255  0.48576  0.95510
222   2.49670  4.13438  3.92580  3.35145  2.28452  3.69520  4.00691  2.12241  3.25412  2.19709  3.06115  3.62187  3.34138  3.50111  3.48764  2.97931  2.73123  2.20800  4.76200  3.56118    239 i - - -
      2.68618  4.42225  2.77519  2.73123  3.46354  2.40513  3.72494  3.29354  2.67741  2.69355  4.24690  2.90347  2.73739  3.18146  2.89801  2.37887  2.77519  2.98518  4.58477  3.61503
      0.02633  4.04612  4.76847  0.61958  0.77255  0.48576  0.95510
223   2.80266  4.39142  3.88820  3.45198  1.41395  2.89363  3.88923  2.78581  3.36780  2.27846  3.50188  3.66620  4.16861  3.62532  3.60187  3.07060  3.09322  2.62992  4.41891  2.87847    240 f - - -
      2.68618  4.42225  2.77519  2.73123  3.46354  2.40513  3.72494  3.29354  2.67741  2.69355  4.24690  2.90347  2.73739  3.18146  2.89801  2.37887  2.77519  2.98518  4.58477  3.61503
      0.02633  4.04612  4.76847  0.61958  0.77255  0.48576  0.95510
224   2.01192  4.27191  3.39984  3.00098  4.02572  1.62214  4.05060  3.33082  2.98111  3.08162  3.95272  3.27580  3.77999  3.31345  3.17653  2.53014  2.76887  2.47953  5.40490  4.16803    241 g - - -
      2.68618  4.42225  2.77519  2.73123  3.46354  2.40513  3.72494  3.29354  2.67741  2.69355  4.24690  2.90347  2.73739  3.18146  2.89801  2.37887  2.77519  2.98518  4.58477  3.61503
      0.02633  4.04612  4.76847  0.61958  0.77255  0.48576  0.95510
225   3.63987  4.94992  4.36684  4.11814  2.86519  3.93328  4.13009  3.67429  3.83837  3.03776  4.29951  4.23296  4.46616  4.21695  3.93507  3.84178  3.94978  3.56933  0.59760  2.85873    242 W - - -
      2.68618  4.42225  2.77519  2.73123  3.46354  2.40513  3.72494  3.29354  2.67741  2.69355  4.24690  2.90347  2.73739  3.18146  2.89801  2.37887  2.77519  2.98518  4.58477  3.61503
      0.02633  4.04612  4.76847  0.61958  0.77255  0.48576  0.95510
226   2.21592  3.75431  3.80003  3.22647  2.50883  3.18291  3.91764  2.56026  3.13751  2.19934  2.88245  3.51110  3.98628  3.39413  3.38932  2.73645  2.78900  2.35696  4.72610  3.51957    243 l - - -
      2.68618  4.42225  2.77519  2.73123  3.46354  2.40513  3.72494  3.29354  2.67741  2.69355  4.24690  2.90347  2.73739  3.18146  2.89801  2.37887  2.77519  2.98518  4.58477  3.61503
      0.02633  4.04612  4.76847  0.61958  0.77255  0.48576  0.95510
```

227	1.47731	4.31573	3.58974	3.07518	3.62843	3.45241	4.01310	2.58062	2.97667	2.45700	3.37281	3.41015	3.96147	3.32255	3.30380	2.68867	2.86780	2.45817	5.13307	3.88890	244 a - - -
	2.68618	4.42225	2.77519	2.73123	3.46354	2.40513	3.72494	3.29354	2.67741	2.69355	4.24690	2.90347	2.73739	3.18146	2.89801	2.37887	2.77519	2.98518	4.58477	3.61503	
	0.02633	4.04612	4.76847	0.61958	0.77255	0.48576	0.95510														
228	2.86112	4.36034	4.10323	3.55139	2.84218	3.92829	3.73338	2.43670	3.41440	1.24138	3.12699	3.82475	4.26798	3.66994	3.63678	3.22915	3.09582	2.15729	4.73675	3.41534	245 l - - -
	2.68618	4.42225	2.77519	2.73123	3.46354	2.40513	3.72494	3.29354	2.67741	2.69355	4.24690	2.90347	2.73739	3.18146	2.89801	2.37887	2.77519	2.98518	4.58477	3.61503	
	0.02633	4.04612	4.76847	0.61958	0.77255	0.48576	0.95510														
229	1.91434	4.32174	3.35831	2.86884	3.71434	2.17392	3.87834	3.12426	2.83076	2.81676	3.68869	3.23007	3.83106	3.15963	3.19213	2.46211	2.78209	2.82218	3.55149	3.85816	246 a - - -
	2.68618	4.42225	2.77519	2.73123	3.46354	2.40513	3.72494	3.29354	2.67741	2.69355	4.24690	2.90347	2.73739	3.18146	2.89801	2.37887	2.77519	2.98518	4.58477	3.61503	
	0.02633	4.04612	4.76847	0.61958	0.77255	0.48576	0.95510														
230	3.05113	4.38367	4.72414	4.18517	2.78871	4.34826	4.74478	1.61042	4.07898	1.95104	3.23514	4.38783	4.63838	4.27594	4.24448	3.69535	3.29698	1.20764	5.22074	4.00087	247 v - - -
	2.68618	4.42225	2.77519	2.73123	3.46354	2.40513	3.72494	3.29354	2.67741	2.69355	4.24690	2.90347	2.73739	3.18146	2.89801	2.37887	2.77519	2.98518	4.58477	3.61503	
	0.02633	4.04612	4.76847	0.61958	0.77255	0.48576	0.95510														
231	2.64156	4.14137	3.98082	3.40531	2.46512	3.12578	3.97420	2.49713	3.29584	1.82654	2.54888	3.65243	4.08398	3.52876	3.50808	3.00702	2.87334	2.34551	4.64903	3.18428	248 l - - -
	2.68618	4.42225	2.77519	2.73123	3.46354	2.40513	3.72494	3.29354	2.67741	2.69355	4.24690	2.90347	2.73739	3.18146	2.89801	2.37887	2.77519	2.98518	4.58477	3.61503	
	0.02633	4.04612	4.76847	0.61958	0.77255	0.48576	0.95510														
232	2.89498	4.64988	3.60405	3.52716	4.66000	0.50522	4.63379	4.30482	3.73186	3.93620	4.91564	3.76881	4.04640	4.04253	3.94591	3.07238	3.38974	3.79962	5.70617	4.76884	249 G - - -
	2.68618	4.42225	2.77519	2.73123	3.46354	2.40513	3.72494	3.29354	2.67741	2.69355	4.24690	2.90347	2.73739	3.18146	2.89801	2.37887	2.77519	2.98518	4.58477	3.61503	
	0.02633	4.04612	4.76847	0.61958	0.77255	0.48576	0.95510														
233	2.67274	4.25707	3.80513	3.25977	3.45974	3.71868	4.08834	2.10008	3.16881	2.33057	3.40136	3.03828	4.11924	3.47766	3.46452	3.01382	2.68837	1.53352	5.01010	3.78548	250 v - - -
	2.68618	4.42225	2.77519	2.73123	3.46354	2.40513	3.72494	3.29354	2.67741	2.69355	4.24690	2.90347	2.73739	3.18146	2.89801	2.37887	2.77519	2.98518	4.58477	3.61503	
	0.02633	4.04612	4.76847	0.61958	0.77255	0.48576	0.95510														
234	2.42149	4.09210	3.83716	3.25886	3.08592	3.13935	3.91979	2.48413	3.16299	1.96261	3.20371	3.53548	3.99993	3.41457	3.40235	2.82395	2.72123	2.06442	3.77919	3.49560	251 l - - -
	2.68618	4.42225	2.77519	2.73123	3.46354	2.40513	3.72494	3.29354	2.67741	2.69355	4.24690	2.90347	2.73739	3.18146	2.89801	2.37887	2.77519	2.98518	4.58477	3.61503	
	0.02633	4.04612	4.76847	0.61958	0.77255	0.48576	0.95510														
235	2.48702	4.24684	3.52750	2.96667	2.97477	3.48928	3.83552	2.65465	2.89316	1.86649	3.35408	2.90330	3.94853	3.20132	3.11209	2.82298	2.70724	2.49521	4.83579	3.60208	252 l - - -
	2.68618	4.42225	2.77519	2.73123	3.46354	2.40513	3.72494	3.29354	2.67741	2.69355	4.24690	2.90347	2.73739	3.18146	2.89801	2.37887	2.77519	2.98518	4.58477	3.61503	
	0.02633	4.04612	4.76847	0.61958	0.77255	0.48576	0.95510														
236	2.53151	4.86366	2.61371	2.38810	4.12827	3.38748	3.62727	3.29284	2.41172	3.13594	3.95111	2.91399	3.80675	1.94535	2.86869	2.63547	2.67913	3.20468	5.36753	3.91486	253 q - - -
	2.68618	4.42225	2.77519	2.73123	3.46354	2.40513	3.72494	3.29354	2.67741	2.69355	4.24690	2.90347	2.73739	3.18146	2.89801	2.37887	2.77519	2.98518	4.58477	3.61503	
	0.02633	4.04612	4.76847	0.61958	0.77255	0.48576	0.95510														
237	2.47359	4.28548	3.46307	2.57600	3.42888	3.09417	3.81881	2.44129	2.84655	2.35983	2.87754	3.30103	3.93844	3.15238	3.19693	2.80638	2.71006	2.31670	4.88429	3.65094	254 v - - -
	2.68618	4.42225	2.77519	2.73123	3.46354	2.40513	3.72494	3.29354	2.67741	2.69355	4.24690	2.90347	2.73739	3.18146	2.89801	2.37887	2.77519	2.98518	4.58477	3.61503	
	0.02633	4.04612	4.76847	0.61958	0.77255	0.48576	0.95510														
238	2.66484	3.20383	4.24531	3.65937	2.48741	3.80488	4.11871	2.23397	3.52323	1.81836	3.10469	3.82942	4.15783	3.50668	3.66826	3.10443	2.89835	1.87665	4.71753	3.52566	255 l - - -
	2.68618	4.42225	2.77519	2.73123	3.46354	2.40513	3.72494	3.29354	2.67741	2.69355	4.24690	2.90347	2.73739	3.18146	2.89801	2.37887	2.77519	2.98518	4.58477	3.61503	
	0.02633	4.04612	4.76847	0.61958	0.77255	0.48576	0.95510														
239	2.34783	4.53191	3.05518	2.59094	3.73138	3.05242	3.69146	3.05170	2.57154	2.72871	3.33100	3.06932	2.82031	2.90687	2.99616	2.64422	2.56888	2.84297	5.09375	3.57665	256 a - - -
	2.68618	4.42225	2.77519	2.73123	3.46354	2.40513	3.72494	3.29354	2.67741	2.69355	4.24690	2.90347	2.73739	3.18146	2.89801	2.37887	2.77519	2.98518	4.58477	3.61503	
	0.02633	4.04612	4.76847	0.61958	0.77255	0.48576	0.95510														
240	2.32233	4.41043	3.13982	2.60835	4.11997	3.24485	3.72234	3.52502	2.43352	3.13302	3.95806	2.89042	3.81693	2.88994	1.82968	2.32438	2.84362	3.16652	5.35811	4.06951	257 r - - -
	2.68618	4.42225	2.77519	2.73123	3.46354	2.40513	3.72494	3.29354	2.67741	2.69355	4.24690	2.90347	2.73739	3.18146	2.89801	2.37887	2.77519	2.98518	4.58477	3.61503	
	0.02633	4.04612	4.76847	0.61958	0.77255	0.48576	0.95510														
241	3.08971	4.41307	4.79803	4.23375	2.78417	4.38143	4.73821	1.48141	4.11817	1.53726	2.90612	4.42888	4.63082	4.25653	4.24095	3.71774	3.32118	1.66637	5.15049	4.01928	258 i - - -
	2.68618	4.42225	2.77519	2.73123	3.46354	2.40513	3.72494	3.29354	2.67741	2.69355	4.24690	2.90347	2.73739	3.18146	2.89801	2.37887	2.77519	2.98518	4.58477	3.61503	
	0.02633	4.04612	4.76847	0.61958	0.77255	0.48576	0.95510														
242	2.22971	4.32816	3.37243	2.80684	3.28741	3.52322	3.42174	2.53938	2.60091	2.48391	3.42924	3.23066	3.90427	3.01532	3.13118	2.56546	2.76547	2.48952	4.90268	3.39627	259 a - - -
	2.68618	4.42225	2.77519	2.73123	3.46354	2.40513	3.72494	3.29354	2.67741	2.69355	4.24690	2.90347	2.73739	3.18146	2.89801	2.37887	2.77519	2.98518	4.58477	3.61503	
	0.02633	4.04612	4.76847	0.61958	0.77255	0.48576	0.95510														
243	2.37497	5.06727	2.78863	1.85623	4.38157	3.39068	3.51579	3.82748	2.09403	3.34470	4.13109	2.69872	3.81425	2.65833	2.58742	2.66012	2.91505	3.44453	5.50328	4.13822	260 e - - -
	2.68618	4.42225	2.77519	2.73123	3.46354	2.40513	3.72494	3.29354	2.67741	2.69355	4.24690	2.90347	2.73739	3.18146	2.89801	2.37887	2.77519	2.98518	4.58477	3.61503	
	0.02633	4.04612	4.76847	0.61958	0.77255	0.48576	0.95510														
244	2.85611	4.38143	4.15374	3.65817	3.31576	3.60950	4.37056	2.25565	3.49289	1.19805	3.24293	3.91568	4.31284	3.80405	3.71603	3.25288	3.13817	1.91554	5.08237	3.86204	261 l - - -
	2.68618	4.42225	2.77519	2.73123	3.46354	2.40513	3.72494	3.29354	2.67741	2.69355	4.24690	2.90347	2.73739	3.18146	2.89801	2.37887	2.77519	2.98518	4.58477	3.61503	
	0.02633	4.04612	4.76847	0.61958	0.77255	0.48576	0.95510														
245	2.56515	3.83360	4.10918	3.52784	3.13229	3.57444	4.07878	1.93497	3.40834	1.76066	2.86663	3.74129	4.11935	3.63293	3.59343	2.94850	2.86776	2.11544	4.75253	3.56234	262 l - - -
	2.68616	4.42238	2.77527	2.73132	3.46335	2.40521	3.72496	3.29356	2.67723	2.69357	4.24702	2.90359	2.73752	3.18139	2.89806	2.37895	2.77506	2.98515	4.58490	3.61427	
	0.34896	1.42094	2.93577	1.12668	0.39172	0.48576	0.95510														
246	2.75053	4.93211	2.66018	2.29966	4.36638	1.70346	3.74476	3.78466	2.11891	3.36693	4.21611	2.92852	3.85857	2.91687	2.85786	2.76413	3.03599	3.42302	5.55270	4.23163	268 g - - -
	2.68618	4.42225	2.77519	2.73123	3.46354	2.40513	3.72494	3.29354	2.67741	2.69355	4.24690	2.90347	2.73739	3.18146	2.89801	2.37887	2.77519	2.98518	4.58477	3.61503	
	0.02757	4.00050	4.72285	0.61958	0.77255	0.53307	0.88382														
247	2.77014	5.06509	3.01234	2.25785	4.40539	3.48004	3.40555	3.81933	1.56346	3.31903	4.12863	2.97190	3.87184	2.54703	2.34621	2.75526	2.97803	3.46171	5.44499	3.85472	269 k - - -
	2.68618	4.42225	2.77519	2.73123	3.46354	2.40513	3.72494	3.29354	2.67741	2.69355	4.24690	2.90347	2.73739	3.18146	2.89801	2.37887	2.77519	2.98518	4.58477	3.61503	
	0.02757	4.00050	4.72285	0.61958	0.77255	0.45728	1.00241														
248	2.52756	5.16857	2.05987	1.89108	4.47889	3.32237	3.63067	3.95439	2.24662	3.46258	4.24117	2.79481	3.36304	2.75524	2.95752	2.58586	2.85700	3.54421	5.62403	4.20474	270 e - - -
	2.68618	4.42225	2.77519	2.73123	3.46354	2.40513	3.72494	3.29354	2.67741	2.69355	4.24690	2.90347	2.73739	3.18146	2.89801	2.37887	2.77519	2.98518	4.58477	3.61503	
	0.02633	4.04612	4.76847	0.61958	0.77255	0.48576	0.95510														
249	2.36791	3.51777	3.04160	2.63647	3.59331	3.14653	3.73157	2.87305	2.51749	2.24902	3.52755	3.14862	3.87522	2.99010	3.06220	2.72043	2.70176	2.71170	4.99470	3.73405	271 l - - -

```
          2.68618  4.42225  2.77519  2.73123  3.46354  2.40513  3.72494  3.29354  2.67741  2.69355  4.24690  2.90347  2.73739  3.18146  2.89801  2.37887  2.77519  2.98518  4.58477  3.61503
          0.02633  4.04612  4.76847  0.61958  0.77255  0.48576  0.95510
    250   2.25059  3.38158  3.90517  3.32755  3.22376  3.66744  3.97751  2.29986  3.22971  2.17976  2.85218  3.59444  4.04141  3.32308  3.46187  2.94897  2.60422  2.01408  4.74130  3.54198    272 v - - -
          2.68618  4.42225  2.77519  2.73123  3.46354  2.40513  3.72494  3.29354  2.67741  2.69355  4.24690  2.90347  2.73739  3.18146  2.89801  2.37887  2.77519  2.98518  4.58477  3.61503
          0.14186  4.04612  2.16484  0.61958  0.77255  0.48576  0.95510
    251   2.23664  4.71025  2.89415  2.16067  4.00559  3.19971  3.63458  3.41495  2.44965  2.93789  3.85538  2.92816  3.51902  2.71691  2.89830  2.34351  2.81060  2.99164  5.28315  3.95811    273 e - - -
          2.68618  4.42225  2.77519  2.73123  3.46354  2.40513  3.72494  3.29354  2.67741  2.69355  4.24690  2.90347  2.73739  3.18146  2.89801  2.37887  2.77519  2.98518  4.58477  3.61503
          0.05336  3.93376  3.42977  0.61958  0.77255  0.59502  0.80196
    252   2.17968  4.69318  2.61300  2.34940  3.97571  3.09329  3.64094  3.37960  2.48319  2.67285  3.83763  2.91966  3.40143  2.81293  2.93994  2.53858  2.81231  3.06125  5.26994  3.94520    274 a - - -
          2.68618  4.42225  2.77519  2.73123  3.46354  2.40513  3.72494  3.29354  2.67741  2.69355  4.24690  2.90347  2.73739  3.18146  2.89801  2.37887  2.77519  2.98518  4.58477  3.61503
          0.05010  3.91061  3.54611  0.61958  0.77255  0.61475  0.77822
    253   2.13927  4.27925  3.55351  2.89628  3.48024  3.57423  3.94104  2.40456  2.93432  2.10260  3.40440  3.39492  3.99141  3.26597  3.26648  2.86288  2.76895  2.04365  5.00138  3.77144    275 v - - -
          2.68618  4.42225  2.77519  2.73123  3.46354  2.40513  3.72494  3.29354  2.67741  2.69355  4.24690  2.90347  2.73739  3.18146  2.89801  2.37887  2.77519  2.98518  4.58477  3.61503
          0.03080  3.89131  4.61366  0.61958  0.77255  0.63056  0.75992
    254   2.58488  4.87102  2.58654  1.99953  4.13582  3.34540  3.57533  3.56487  2.31411  3.14050  3.94265  2.85355  3.76162  2.71356  2.72111  2.58994  2.61691  3.03706  5.35492  3.75271    276 e - - -
          2.68618  4.42225  2.77519  2.73123  3.46354  2.40513  3.72494  3.29354  2.67741  2.69355  4.24690  2.90347  2.73739  3.18146  2.89801  2.37887  2.77519  2.98518  4.58477  3.61503
          0.10829  3.89131  2.49848  0.61958  0.77255  0.63056  0.75992
    255   2.34772  4.64633  2.80239  2.28830  3.87510  3.18314  3.60764  3.27473  2.44532  2.90950  3.74652  2.92088  3.43763  2.70512  2.89208  2.60433  2.78366  2.73162  5.18381  3.65250    277 e - - -
          2.68572  4.42196  2.77531  2.73061  3.46392  2.40531  3.72492  3.29369  2.67759  2.69361  4.24556  2.90343  2.73707  3.18164  2.89828  2.37919  2.77526  2.98510  4.58485  3.61523
          0.38147  1.14840        *  2.06445  0.13569  0.00000        *
//
HMMER3/f [3.1b2 | February 2015]
NAME  mmoX_loose
LENG  519
ALPH  amino
RF    no
MM    no
CONS  yes
CS    no
MAP   yes
DATE  Wed Mar 28 11:40:54 2018
NSEQ  205
EFFN  1.654739
CKSUM 4003712988
STATS LOCAL MSV      -11.7524  0.69784
STATS LOCAL VITERBI  -12.7209  0.69784
STATS LOCAL FORWARD   -6.3533  0.69784
HMM          A        C        D        E        F        G        H        I        K        L        M        N        P        Q        R        S        T        V        W        Y   
            m->m     m->i     m->d     i->m     i->i     d->m     d->d
  COMPO   2.50695  4.35371  2.87717  2.57714  3.23079  2.98877  3.53991  2.96335  2.70128  2.59040  3.55212  3.07618  3.34217  3.02962  2.90626  2.73479  2.82016  2.74569  3.95917  3.22557
          2.68522  4.42293  2.77522  2.73181  3.46405  2.40556  3.72563  3.29361  2.67717  2.69307  4.24136  2.90367  2.73762  3.18179  2.89831  2.37905  2.77441  2.98559  4.58545  3.61571
          0.81050  1.09933  1.50385  2.01078  0.14374  0.00000        *
      1   2.69544  4.18642  4.06093  3.48319  3.29303  3.80171  4.11029  1.98021  3.37763  2.09669  2.36829  2.94376  4.16498  3.62154  3.59718  3.08682  2.76717  1.96609  4.84210  3.64674     22 v - - -
          2.68618  4.42225  2.77519  2.73123  3.46354  2.40513  3.72494  3.29354  2.67741  2.69355  4.24690  2.90347  2.73739  3.18146  2.89801  2.37887  2.77519  2.98518  4.58477  3.61503
          0.02141  4.25055  4.97289  0.61958  0.77255  0.79986  0.59673
      2   2.32183  4.70708  3.11648  2.56123  3.94795  3.47076  3.70832  3.32181  2.52876  2.69327  3.81631  3.05784  3.67283  2.75418  2.64405  2.15697  2.35091  3.02838  5.25681  3.95066     23 s - - -
          2.68618  4.42225  2.77519  2.73123  3.46354  2.40513  3.72494  3.29354  2.67741  2.69355  4.24690  2.90347  2.73739  3.18146  2.89801  2.37887  2.77519  2.98518  4.58477  3.61503
          0.05415  4.25055  3.25839  0.61958  0.77255  0.79986  0.59673
      3   2.21148  4.96579  3.08916  2.53643  4.31251  3.49094  3.67149  3.72545  2.09955  3.27518  4.08447  3.03426  3.89809  2.75839  1.91744  2.27048  2.96073  3.37356  5.45274  4.14328     24 r - - -
          2.68618  4.42225  2.77519  2.73123  3.46354  2.40513  3.72494  3.29354  2.67741  2.69355  4.24690  2.90347  2.73739  3.18146  2.89801  2.37887  2.77519  2.98518  4.58477  3.61503
          0.02211  4.21851  4.94086  0.61958  0.77255  0.71011  0.67647
      4   2.68108  5.04788  2.95057  2.32959  4.35397  3.45327  3.63951  3.79900  2.10645  3.32957  3.72462  2.94532  2.94885  1.95455  2.80226  2.45199  2.91005  3.41925  5.50091  4.13612     25 q - - -
          2.68618  4.42225  2.77519  2.73123  3.46354  2.40513  3.72494  3.29354  2.67741  2.69355  4.24690  2.90347  2.73739  3.18146  2.89801  2.37887  2.77519  2.98518  4.58477  3.61503
          0.19790  4.27750  1.79773  0.61958  0.77255  0.53561  0.88023
      5   2.57264  4.76035  2.77236  2.51149  3.98306  3.47183  3.67733  3.18397  2.12255  3.00940  2.90080  3.02109  3.86049  2.84271  2.51944  2.68075  2.72000  2.75189  5.27481  3.95674     26 k - - -
          2.68618  4.42225  2.77519  2.73123  3.46354  2.40513  3.72494  3.29354  2.67741  2.69355  4.24690  2.90347  2.73739  3.18146  2.89801  2.37887  2.77519  2.98518  4.58477  3.61503
          0.02168  4.23814  4.96049  0.61958  0.77255  0.69200  0.69430
      6   2.41079  4.41677  3.41107  2.84661  3.56802  2.54999  3.82791  2.93482  2.68377  2.31604  3.24520  3.17091  3.96350  3.12200  3.18301  2.61049  2.45989  2.69938  3.74144  3.74588     27 l - - -
          2.68618  4.42225  2.77519  2.73123  3.46354  2.40513  3.72494  3.29354  2.67741  2.69355  4.24690  2.90347  2.73739  3.18146  2.89801  2.37887  2.77519  2.98518  4.58477  3.61503
          0.02045  4.29592  5.01827  0.61958  0.77255  0.75362  0.63613
      7   2.36975  4.37605  3.46586  2.89934  3.51755  2.90833  3.64906  2.33986  2.74244  2.58966  3.44196  3.31609  3.57590  3.16313  2.92611  2.83868  2.79200  2.29074  4.95450  3.18611     28 v - - -
          2.68618  4.42225  2.77519  2.73123  3.46354  2.40513  3.72494  3.29354  2.67741  2.69355  4.24690  2.90347  2.73739  3.18146  2.89801  2.37887  2.77519  2.98518  4.58477  3.61503
          0.02045  4.29592  5.01827  0.61958  0.77255  0.49286  0.94385
      8   2.67952  5.02701  2.72161  2.35316  3.91952  3.09534  3.67483  3.75562  2.34369  2.80562  4.08660  2.72846  3.51428  2.79693  2.92289  1.86524  2.84511  3.38479  5.50001  4.12915     29 s - - -
          2.68618  4.42225  2.77519  2.73123  3.46354  2.40513  3.72494  3.29354  2.67741  2.69355  4.24690  2.90347  2.73739  3.18146  2.89801  2.37887  2.77519  2.98518  4.58477  3.61503
          0.01805  4.41940  5.14174  0.61958  0.77255  0.53509  0.88097
      9   2.33129  4.23055  3.96949  3.39072  3.34129  3.77094  4.06197  2.30374  2.73159  1.73376  3.09806  3.67329  3.60953  3.54933  3.53728  3.04611  2.91332  2.06144  4.84773  3.64677     30 l - - -
```

```
    2.68618  4.42225  2.77519  2.73123  3.46354  2.40513  3.72494  3.29354  2.67741  2.69355  4.24690  2.90347  2.73739  3.18146  2.89801  2.37887  2.77519  2.98518  4.58477  3.61503
    0.01757  4.44625  5.16859  0.61958  0.77255  0.56359  0.84202
10  2.40936  5.01205  3.00424  2.15158  4.29573  2.89400  3.68698  3.44151  2.45639  3.29428  4.07799  2.86880  3.87947  2.42993  2.93361  2.46695  2.22814  3.21174  5.49353  4.12772    31 e - - -
    2.68618  4.42225  2.77519  2.73123  3.46354  2.40513  3.72494  3.29354  2.67741  2.69355  4.24690  2.90347  2.73739  3.18146  2.89801  2.37887  2.77519  2.98518  4.58477  3.61503
    0.01757  4.44625  5.16859  0.61958  0.77255  0.56359  0.84202
11  2.73150  5.20304  2.70834  1.91039  4.54410  3.48532  3.66951  4.01497  1.85329  3.49824  4.24999  2.95721  3.88481  2.77283  2.20075  2.69685  2.71747  3.45893  5.62996  4.23813    32 k - - -
    2.68618  4.42225  2.77519  2.73123  3.46354  2.40513  3.72494  3.29354  2.67741  2.69355  4.24690  2.90347  2.73739  3.18146  2.89801  2.37887  2.77519  2.98518  4.58477  3.61503
    0.01757  4.44625  5.16859  0.61958  0.77255  0.56359  0.84202
12  1.77321  4.58919  2.93791  2.75981  3.76633  3.58476  3.83188  3.13675  2.74504  2.82383  3.69212  3.22634  3.97899  2.85284  3.15635  2.82027  2.79327  2.23014  5.15402  3.06855    33 a - - -
    2.68618  4.42225  2.77519  2.73123  3.46354  2.40513  3.72494  3.29354  2.67741  2.69355  4.24690  2.90347  2.73739  3.18146  2.89801  2.37887  2.77519  2.98518  4.58477  3.61503
    0.01757  4.44625  5.16859  0.61958  0.77255  0.56359  0.84202
13  2.24406  4.77408  3.16161  2.64973  3.98884  3.53753  1.85364  3.42292  2.62654  3.06068  3.90333  2.94840  3.95662  2.98941  3.05153  2.78686  2.41597  3.12569  5.31454  3.59811    34 h - - -
    2.68618  4.42225  2.77519  2.73123  3.46354  2.40513  3.72494  3.29354  2.67741  2.69355  4.24690  2.90347  2.73739  3.18146  2.89801  2.37887  2.77519  2.98518  4.58477  3.61503
    0.01757  4.44625  5.16859  0.61958  0.77255  0.56359  0.84202
14  2.28748  5.16237  2.82792  1.99300  4.49346  3.46553  3.65361  3.96763  2.12969  3.24161  4.20758  2.64066  3.85987  2.58301  2.41250  2.64805  2.92129  3.31344  5.60194  4.20246    35 e - - -
    2.68618  4.42225  2.77519  2.73123  3.46354  2.40513  3.72494  3.29354  2.67741  2.69355  4.24690  2.90347  2.73739  3.18146  2.89801  2.37887  2.77519  2.98518  4.58477  3.61503
    0.01757  4.44625  5.16859  0.61958  0.77255  0.56359  0.84202
15  2.82677  4.81442  3.40588  2.79474  4.01195  3.66947  3.79543  3.40594  1.65888  2.86913  2.99341  3.25270  4.04128  2.98823  2.17753  2.91599  3.04122  3.13700  2.83362  4.02620    36 k - - -
    2.68618  4.42225  2.77519  2.73123  3.46354  2.40513  3.72494  3.29354  2.67741  2.69355  4.24690  2.90347  2.73739  3.18146  2.89801  2.37887  2.77519  2.98518  4.58477  3.61503
    0.01757  4.44625  5.16859  0.61958  0.77255  0.56359  0.84202
16  2.69402  4.24396  4.40549  3.82694  3.39532  3.98678  4.33553  1.50536  3.70076  2.17249  3.13480  4.00824  4.34566  3.91726  3.86345  3.06907  2.22250  1.84361  4.97142  3.77981    37 i - - -
    2.68618  4.42225  2.77519  2.73123  3.46354  2.40513  3.72494  3.29354  2.67741  2.69355  4.24690  2.90347  2.73739  3.18146  2.89801  2.37887  2.77519  2.98518  4.58477  3.61503
    0.01757  4.44625  5.16859  0.61958  0.77255  0.56359  0.84202
17  2.38759  5.04445  3.00464  2.44733  3.58133  3.48620  3.67758  3.78028  2.01380  3.32504  4.10256  2.98188  3.61023  2.29420  2.54464  2.44746  2.57200  3.40589  5.51181  4.14219    38 k - - -
    2.68618  4.42225  2.77519  2.73123  3.46354  2.40513  3.72494  3.29354  2.67741  2.69355  4.24690  2.90347  2.73739  3.18146  2.89801  2.37887  2.77519  2.98518  4.58477  3.61503
    0.01757  4.44625  5.16859  0.61958  0.77255  0.56359  0.84202
18  2.72370  5.21946  2.13851  1.98375  4.55212  3.03058  3.67686  4.03405  2.43188  3.31314  4.26692  2.77727  3.87328  2.67264  2.57141  2.22566  2.95805  3.60412  5.65728  4.24564    39 e - - -
    2.68618  4.42225  2.77519  2.73123  3.46354  2.40513  3.72494  3.29354  2.67741  2.69355  4.24690  2.90347  2.73739  3.18146  2.89801  2.37887  2.77519  2.98518  4.58477  3.61503
    0.01757  4.44625  5.16859  0.61958  0.77255  0.56359  0.84202
19  3.15174  4.50520  4.87017  4.29661  2.14587  4.31288  4.42557  2.41160  4.13235  1.09440  2.90879  4.36669  4.59794  4.21367  4.18191  3.63255  3.37446  2.58683  4.76369  2.40513    40 l - - -
    2.68618  4.42225  2.77519  2.73123  3.46354  2.40513  3.72494  3.29354  2.67741  2.69355  4.24690  2.90347  2.73739  3.18146  2.89801  2.37887  2.77519  2.98518  4.58477  3.61503
    0.01757  4.44625  5.16859  0.61958  0.77255  0.56359  0.84202
20  2.57064  5.03701  2.18275  2.46462  4.41155  2.42206  3.73382  3.86459  2.51465  3.40769  4.18367  2.72459  3.88879  2.85719  2.99873  2.23430  2.56100  3.03393  5.59123  4.21487    41 d - - -
    2.68618  4.42225  2.77519  2.73123  3.46354  2.40513  3.72494  3.29354  2.67741  2.69355  4.24690  2.90347  2.73739  3.18146  2.89801  2.37887  2.77519  2.98518  4.58477  3.61503
    0.01757  4.44625  5.16859  0.61958  0.77255  0.56359  0.84202
21  3.16348  4.79483  4.27813  3.90921  3.18933  3.89858  4.26126  3.42405  3.58313  2.97931  4.10478  4.04119  4.46265  4.01553  3.75942  3.39258  2.70416  3.23964  0.77071  3.23371    42 w - - -
    2.68618  4.42225  2.77519  2.73123  3.46354  2.40513  3.72494  3.29354  2.67741  2.69355  4.24690  2.90347  2.73739  3.18146  2.89801  2.37887  2.77519  2.98518  4.58477  3.61503
    0.01757  4.44625  5.16859  0.61958  0.77255  0.56359  0.84202
22  2.86272  5.38308  2.08601  1.50628  4.70264  3.19785  3.77955  4.19431  2.60858  3.59200  4.45181  2.54752  3.94019  2.87748  3.13213  2.80140  2.35592  3.76398  5.82736  4.38898    43 e - - -
    2.68618  4.42225  2.77519  2.73123  3.46354  2.40513  3.72494  3.29354  2.67741  2.69355  4.24690  2.90347  2.73739  3.18146  2.89801  2.37887  2.77519  2.98518  4.58477  3.61503
    0.11663  4.44625  2.31911  0.61958  0.77255  0.56359  0.84202
23  2.70076  4.75758  3.07861  2.38265  3.35283  3.51305  3.40776  3.37863  2.61473  3.01860  3.86850  3.10066  1.76023  2.96707  3.04370  2.77433  2.76752  3.08931  5.28152  3.96228    44 p - - -
    2.68618  4.42225  2.77519  2.73123  3.46354  2.40513  3.72494  3.29354  2.67741  2.69355  4.24690  2.90347  2.73739  3.18146  2.89801  2.37887  2.77519  2.98518  4.58477  3.61503
    0.01938  4.34900  5.07134  0.61958  0.77255  0.69370  0.69260
24  2.60217  4.99061  2.78280  2.25853  4.28989  3.44886  3.69827  3.72758  2.48691  3.29365  4.08666  2.96962  3.87169  2.65375  2.96394  1.99429  2.02133  3.25643  5.50031  4.13620    45 s - - -
    2.68618  4.42225  2.77519  2.73123  3.46354  2.40513  3.72494  3.29354  2.67741  2.69355  4.24690  2.90347  2.73739  3.18146  2.89801  2.37887  2.77519  2.98518  4.58477  3.61503
    0.01938  4.34900  5.07134  0.61958  0.77255  0.47526  0.97212
25  3.14489  4.55023  4.52180  4.02479  1.71216  3.57688  3.90760  2.98616  3.88662  2.62469  3.66024  4.05523  4.51106  4.01741  4.00606  3.46986  3.37939  2.30873  4.21615  1.28713    46 y - - -
    2.68618  4.42225  2.77519  2.73123  3.46354  2.40513  3.72494  3.29354  2.67741  2.69355  4.24690  2.90347  2.73739  3.18146  2.89801  2.37887  2.77519  2.98518  4.58477  3.61503
    0.01757  4.44625  5.16859  0.61958  0.77255  0.56359  0.84202
26  2.02610  4.68697  3.08092  2.63145  3.06161  3.55803  3.34043  3.06498  2.48576  2.92345  3.77257  3.14854  3.39963  2.58539  3.07546  2.77411  2.91003  2.47717  5.22515  3.93307    47 a - - -
    2.68618  4.42225  2.77519  2.73123  3.46354  2.40513  3.72494  3.29354  2.67741  2.69355  4.24690  2.90347  2.73739  3.18146  2.89801  2.37887  2.77519  2.98518  4.58477  3.61503
    0.01757  4.44625  5.16859  0.61958  0.77255  0.56359  0.84202
27  2.48720  5.17120  2.80530  2.16070  4.50438  3.26663  3.35705  3.98207  2.19060  3.47099  4.21560  2.85924  2.99247  2.31843  2.79831  2.65566  2.39828  3.55556  5.61037  4.20647    48 e - - -
    2.68618  4.42225  2.77519  2.73123  3.46354  2.40513  3.72494  3.29354  2.67741  2.69355  4.24690  2.90347  2.73739  3.18146  2.89801  2.37887  2.77519  2.98518  4.58477  3.61503
    0.01757  4.44625  5.16859  0.61958  0.77255  0.56359  0.84202
28  2.68989  5.11406  2.93155  1.98861  4.42627  3.27494  3.66357  3.88804  2.32193  3.19515  3.46767  2.61845  2.48484  2.68310  2.62856  2.66879  2.92080  3.48710  5.56563  4.17784    49 e - - -
    2.68618  4.42225  2.77519  2.73123  3.46354  2.40513  3.72494  3.29354  2.67741  2.69355  4.24690  2.90347  2.73739  3.18146  2.89801  2.37887  2.77519  2.98518  4.58477  3.61503
    0.01757  4.44625  5.16859  0.61958  0.77255  0.56359  0.84202
29  1.99038  5.11656  2.56509  2.25203  4.43005  3.08573  3.66189  3.89409  2.32928  3.40849  4.16686  2.94918  3.34405  2.77010  2.49432  2.56145  2.91733  3.24703  5.56929  3.97443    50 a - - -
    2.68624  4.42231  2.77506  2.73112  3.46360  2.40518  3.72500  3.29343  2.67728  2.69361  4.24695  2.90352  2.73745  3.18152  2.89806  2.37892  2.77525  2.98490  4.58483  3.61509
    0.21081  2.00028  2.90450  0.66830  0.71863  0.56359  0.84202
30  2.38405  4.57119  3.08183  2.63247  3.19759  3.36709  3.79999  3.11983  2.67057  2.76864  2.85789  3.16154  3.63390  3.03731  3.12306  2.50722  2.24817  2.86408  5.12579  3.27962    53 t - - -
    2.68618  4.42225  2.77519  2.73123  3.46354  2.40513  3.72494  3.29354  2.67741  2.69355  4.24690  2.90347  2.73739  3.18146  2.89801  2.37887  2.77519  2.98518  4.58477  3.61503
    0.01848  4.39592  5.11826  0.61958  0.77255  0.63461  0.75533
31  2.67825  4.99263  3.05153  2.48592  4.27092  3.50271  3.50728  3.70030  1.97447  3.00836  4.05571  3.00976  3.08803  2.74105  2.24996  2.70795  2.43933  2.98745  5.46129  4.11502    54 k - - -
    2.68618  4.42225  2.77519  2.73123  3.46354  2.40513  3.72494  3.29354  2.67741  2.69355  4.24690  2.90347  2.73739  3.18146  2.89801  2.37887  2.77519  2.98518  4.58477  3.61503
```

```
    0.01848  4.39592  5.11826  0.61958  0.77255  0.50452  0.92582
32  3.24787  4.64850  4.52225  3.43091  1.48699  4.23349  3.87943  3.07606  3.89213  2.30487  3.63204  4.07333  4.57244  4.02730  4.02599  3.54416  3.47395  2.93086  4.16976  1.33699     55 y - - -
    2.68618  4.42225  2.77519  2.73123  3.46354  2.40513  3.72494  3.29354  2.67741  2.69355  4.24690  2.90347  2.73739  3.18146  2.89801  2.37887  2.77519  2.98518  4.58477  3.61503
    0.01750  4.44989  5.17224  0.61958  0.77255  0.55811  0.84932
33  2.53523  4.89450  2.89433  2.43702  4.15835  2.63448  3.73038  3.32422  2.53150  3.17731  3.62159  3.04387  2.11534  2.87910  2.99651  2.44503  2.92347  3.24598  5.41680  4.07868     56 p - - -
    2.68618  4.42225  2.77519  2.73123  3.46354  2.40513  3.72494  3.29354  2.67741  2.69355  4.24690  2.90347  2.73739  3.18146  2.89801  2.37887  2.77519  2.98518  4.58477  3.61503
    0.01750  4.44989  5.17224  0.61958  0.77255  0.55811  0.84932
34  2.46981  4.63361  3.28112  2.66793  4.09595  2.85545  4.03500  3.60261  2.92449  3.26347  4.10309  3.28010  3.96444  3.24668  3.33429  2.52912  1.19447  3.23505  5.54056  4.26166     57 t - - -
    2.68618  4.42225  2.77519  2.73123  3.46354  2.40513  3.72494  3.29354  2.67741  2.69355  4.24690  2.90347  2.73739  3.18146  2.89801  2.37887  2.77519  2.98518  4.58477  3.61503
    0.01750  4.44989  5.17224  0.61958  0.77255  0.55811  0.84932
35  2.61411  5.13200  2.03539  2.41929  4.44664  3.47900  3.67310  3.90834  1.98651  3.42196  4.18415  2.96046  3.87739  2.78308  2.48443  2.66824  2.68729  3.50712  5.58075  3.23780     58 k - - -
    2.68618  4.42225  2.77519  2.73123  3.46354  2.40513  3.72494  3.29354  2.67741  2.69355  4.24690  2.90347  2.73739  3.18146  2.89801  2.37887  2.77519  2.98518  4.58477  3.61503
    0.01750  4.44989  5.17224  0.61958  0.77255  0.55811  0.84932
36  3.01985  4.80901  3.56362  2.48349  2.71518  3.78076  3.07151  3.37308  3.06047  2.96559  3.92285  3.48531  4.24978  3.37421  3.41822  3.13805  3.25011  3.14564  4.50454  1.21986     59 y - - -
    2.68618  4.42225  2.77519  2.73123  3.46354  2.40513  3.72494  3.29354  2.67741  2.69355  4.24690  2.90347  2.73739  3.18146  2.89801  2.37887  2.77519  2.98518  4.58477  3.61503
    0.01750  4.44989  5.17224  0.61958  0.77255  0.55811  0.84932
37  2.70227  5.09099  3.00226  2.36574  4.39628  2.91275  3.12476  3.57025  1.86655  3.37455  4.14441  2.97810  3.88125  2.68294  2.50759  2.68804  2.39274  3.22757  5.54400  4.16991     60 k - - -
    2.68618  4.42225  2.77519  2.73123  3.46354  2.40513  3.72494  3.29354  2.67741  2.69355  4.24690  2.90347  2.73739  3.18146  2.89801  2.37887  2.77519  2.98518  4.58477  3.61503
    0.01750  4.44989  5.17224  0.61958  0.77255  0.55811  0.84932
38  3.09944  4.43431  4.95743  4.23796  2.04739  4.33989  4.67851  1.42496  4.20535  1.77585  2.57921  4.45798  4.62680  4.31177  4.25343  3.66727  3.32847  2.01153  5.08656  3.95117     61 i - - -
    2.68618  4.42225  2.77519  2.73123  3.46354  2.40513  3.72494  3.29354  2.67741  2.69355  4.24690  2.90347  2.73739  3.18146  2.89801  2.37887  2.77519  2.98518  4.58477  3.61503
    0.01750  4.44989  5.17224  0.61958  0.77255  0.55811  0.84932
39  2.41409  5.14531  2.75899  2.14969  4.37819  3.43614  3.53684  3.93885  2.14886  3.44111  4.19293  2.87850  2.44673  2.76361  2.80157  2.45103  2.73275  3.46876  5.59163  4.19411     62 k - - -
    2.68618  4.42225  2.77519  2.73123  3.46354  2.40513  3.72494  3.29354  2.67741  2.69355  4.24690  2.90347  2.73739  3.18146  2.89801  2.37887  2.77519  2.98518  4.58477  3.61503
    0.01750  4.44989  5.17224  0.61958  0.77255  0.55811  0.84932
40  2.48744  5.06875  3.00418  2.37610  4.36617  3.28564  3.68147  3.07438  1.67684  3.35036  4.12674  2.63165  3.26973  2.79963  2.73174  2.69538  2.93439  3.43433  5.52992  4.16078     63 k - - -
    2.68618  4.42225  2.77519  2.73123  3.46354  2.40513  3.72494  3.29354  2.67741  2.69355  4.24690  2.90347  2.73739  3.18146  2.89801  2.37887  2.77519  2.98518  4.58477  3.61503
    0.01750  4.44989  5.17224  0.61958  0.77255  0.55811  0.84932
41  2.03276  5.15386  2.80873  2.16658  4.48023  3.27114  2.99076  3.95181  2.03890  3.41640  4.20044  2.94260  3.86263  2.68700  2.68735  2.62920  2.80812  3.48726  5.59663  4.19913     64 a - - -
    2.68618  4.42225  2.77519  2.73123  3.46354  2.40513  3.72494  3.29354  2.67741  2.69355  4.24690  2.90347  2.73739  3.18146  2.89801  2.37887  2.77519  2.98518  4.58477  3.61503
    0.01750  4.44989  5.17224  0.61958  0.77255  0.47218  0.97721
42  2.54731  5.08661  2.52971  2.45844  4.43575  2.66823  3.73876  3.89099  2.45039  3.42931  4.20499  2.92062  2.37664  2.84806  3.00634  2.61291  2.16101  3.49614  5.61043  4.22916     65 t - - -
    2.68618  4.42225  2.77519  2.73123  3.46354  2.40513  3.72494  3.29354  2.67741  2.69355  4.24690  2.90347  2.73739  3.18146  2.89801  2.37887  2.77519  2.98518  4.58477  3.61503
    0.01692  4.48326  5.20561  0.61958  0.77255  0.50539  0.92450
43  2.91230  5.05162  3.37964  2.75437  4.32613  3.69238  3.77292  3.73081  1.38916  3.26593  4.12856  3.22520  4.06284  2.92259  2.08458  2.79654  3.11857  3.15146  2.90550  4.19651     66 k - - -
    2.68634  4.42241  2.77487  2.73120  3.46350  2.40528  3.72510  3.29370  2.67657  2.69353  4.24705  2.90362  2.73755  3.18147  2.89816  2.37903  2.77535  2.98534  4.58257  3.61519
    0.34901  1.24089  5.20561  0.80674  0.59115  0.50539  0.92450
44  3.37067  6.07027  0.85647  1.55063  5.34253  3.48734  4.11955  4.91939  3.25176  4.37517  5.26830  2.93775  4.16249  3.30003  3.92810  3.20611  3.68078  4.45382  6.50761  4.93043     71 d - - -
    2.68618  4.42225  2.77519  2.73123  3.46354  2.40513  3.72494  3.29354  2.67741  2.69355  4.24690  2.90347  2.73739  3.18146  2.89801  2.37887  2.77519  2.98518  4.58477  3.61503
    0.01692  4.48326  5.20561  0.61958  0.77255  0.50539  0.92450
45  3.05260  5.12573  3.13786  2.91268  4.64063  3.59854  4.17500  4.18046  2.89683  3.72061  4.62450  3.37376  0.95885  2.11488  3.22849  3.10494  3.39051  3.79785  5.84782  4.55385     72 p - - -
    2.68618  4.42225  2.77519  2.73123  3.46354  2.40513  3.72494  3.29354  2.67741  2.69355  4.24690  2.90347  2.73739  3.18146  2.89801  2.37887  2.77519  2.98518  4.58477  3.61503
    0.01692  4.48326  5.20561  0.61958  0.77255  0.50539  0.92450
46  3.23236  4.57784  4.91599  4.35888  1.42281  4.37511  4.37998  2.64749  4.19198  1.52334  2.19631  4.40321  4.65691  4.26099  4.23656  3.69763  3.45420  2.67449  4.67881  2.75006     73 f - - -
    2.68618  4.42225  2.77519  2.73123  3.46354  2.40513  3.72494  3.29354  2.67741  2.69355  4.24690  2.90347  2.73739  3.18146  2.89801  2.37887  2.77519  2.98518  4.58477  3.61503
    0.01692  4.48326  5.20561  0.61958  0.77255  0.50539  0.92450
47  3.50298  5.57709  4.04203  3.21725  5.15880  4.04893  3.85910  4.44472  0.85512  3.79316  4.67881  3.56191  4.38070  2.98530  1.52497  3.47672  3.62132  4.12995  5.75237  4.66173     74 k - - -
    2.68618  4.42225  2.77519  2.73123  3.46354  2.40513  3.72494  3.29354  2.67741  2.69355  4.24690  2.90347  2.73739  3.18146  2.89801  2.37887  2.77519  2.98518  4.58477  3.61503
    0.01692  4.48326  5.20561  0.61958  0.77255  0.49248  0.94446
48  2.70030  4.49372  3.47067  2.90500  3.64102  3.64789  3.52835  3.00179  2.84952  2.14438  3.29606  3.33830  4.02971  2.02437  3.22338  2.81652  2.61885  2.24202  5.06460  3.81709     75 q - - -
    2.68618  4.42225  2.77519  2.73123  3.46354  2.40513  3.72494  3.29354  2.67741  2.69355  4.24690  2.90347  2.73739  3.18146  2.89801  2.37887  2.77519  2.98518  4.58477  3.61503
    0.01684  4.48800  5.21035  0.61958  0.77255  0.49752  0.93659
49  2.81962  4.23110  4.46621  3.88224  3.36323  3.98868  4.33190  1.62236  3.74342  1.98734  3.11516  4.03379  4.34573  3.94496  3.88028  2.70682  2.54761  1.72321  4.93573  3.74852     76 i - - -
    2.68618  4.42225  2.77519  2.73123  3.46354  2.40513  3.72494  3.29354  2.67741  2.69355  4.24690  2.90347  2.73739  3.18146  2.89801  2.37887  2.77519  2.98518  4.58477  3.61503
    0.01684  4.48800  5.21035  0.61958  0.77255  0.49752  0.93659
50  2.25266  4.18673  4.55228  3.95240  3.01990  3.94862  4.27118  2.19618  3.78379  1.64163  2.21050  4.04107  4.29857  3.94151  3.86147  3.25417  3.02032  2.13757  4.80384  3.09122     77 l - - -
    2.68618  4.42225  2.77519  2.73123  3.46354  2.40513  3.72494  3.29354  2.67741  2.69355  4.24690  2.90347  2.73739  3.18146  2.89801  2.37887  2.77519  2.98518  4.58477  3.61503
    0.01684  4.48800  5.21035  0.61958  0.77255  0.49752  0.93659
51  2.87563  5.28926  2.55762  2.53416  4.65015  3.31198  3.44447  4.09536  1.97314  3.56803  4.34624  3.05832  3.98684  2.84573  1.43413  2.84080  3.09504  3.69398  5.68052  4.33380     78 r - - -
    2.68618  4.42225  2.77519  2.73123  3.46354  2.40513  3.72494  3.29354  2.67741  2.69355  4.24690  2.90347  2.73739  3.18146  2.89801  2.37887  2.77519  2.98518  4.58477  3.61503
    0.01684  4.48800  5.21035  0.61958  0.77255  0.49752  0.93659
52  2.54730  5.35654  2.31683  1.64522  4.70723  3.20374  3.82146  4.19315  2.66178  3.69330  4.47081  2.95354  3.96516  2.94794  3.18443  1.68140  3.13589  3.76547  5.84893  4.41748     79 e - - -
    2.68618  4.42225  2.77519  2.73123  3.46354  2.40513  3.72494  3.29354  2.67741  2.69355  4.24690  2.90347  2.73739  3.18146  2.89801  2.37887  2.77519  2.98518  4.58477  3.61503
    0.01684  4.48800  5.21035  0.61958  0.77255  0.49752  0.93659
53  4.24687  5.48126  4.80485  4.68128  2.58962  4.59230  4.08272  4.19272  4.53013  3.47795  4.80362  4.55969  5.06617  4.67242  4.56961  4.30493  4.54417  4.09518  4.24412  0.34891     80 Y - - -
    2.68618  4.42225  2.77519  2.73123  3.46354  2.40513  3.72494  3.29354  2.67741  2.69355  4.24690  2.90347  2.73739  3.18146  2.89801  2.37887  2.77519  2.98518  4.58477  3.61503
    0.01684  4.48800  5.21035  0.61958  0.77255  0.49752  0.93659
```

54	2.08763	3.70925	4.45557	3.85886	1.87655	3.85959	4.16832	2.53308	3.69071	2.21551	3.23383	3.94512	4.22578	3.86097	3.77755	3.16325	2.95221	1.99532	3.27246	3.50694	81 f - - -
	2.68618	4.42225	2.77519	2.73123	3.46354	2.40513	3.72494	3.29354	2.67741	2.69355	4.24690	2.90347	2.73739	3.18146	2.89801	2.37887	2.77519	2.98518	4.58477	3.61503	
	0.01684	4.48800	5.21035	0.61958	0.77255	0.49752	0.93659														
55	2.63204	5.12146	3.02657	2.47150	4.44053	3.20387	3.21243	3.89049	2.11053	3.41114	4.18245	3.00406	2.25582	2.80805	2.23386	2.57233	2.82052	3.50095	5.57336	4.20462	82 k - - -
	2.68618	4.42225	2.77519	2.73123	3.46354	2.40513	3.72494	3.29354	2.67741	2.69355	4.24690	2.90347	2.73739	3.18146	2.89801	2.37887	2.77519	2.98518	4.58477	3.61503	
	0.01684	4.48800	5.21035	0.61958	0.77255	0.47848	0.96686														
56	2.88850	4.48847	3.87808	3.37968	3.19863	3.85262	4.02829	2.91706	3.23189	2.56962	1.27446	3.06395	4.25265	3.56758	3.49026	3.16135	3.13234	2.73716	4.76210	2.66136	83 m - - -
	2.68618	4.42225	2.77519	2.73123	3.46354	2.40513	3.72494	3.29354	2.67741	2.69355	4.24690	2.90347	2.73739	3.18146	2.89801	2.37887	2.77519	2.98518	4.58477	3.61503	
	0.01673	4.49498	5.21732	0.61958	0.77255	0.48576	0.95510														
57	3.11979	5.58993	2.64130	1.19386	4.90919	3.55295	3.93750	4.40707	2.71716	3.88172	4.71315	3.02900	4.09647	1.63963	3.15963	3.04025	3.37657	3.99976	6.00366	4.58971	84 e - - -
	2.68618	4.42225	2.77519	2.73123	3.46354	2.40513	3.72494	3.29354	2.67741	2.69355	4.24690	2.90347	2.73739	3.18146	2.89801	2.37887	2.77519	2.98518	4.58477	3.61503	
	0.01673	4.49498	5.21732	0.61958	0.77255	0.48576	0.95510														
58	2.17608	5.04778	3.00615	1.74729	4.33811	3.25906	3.70182	3.78228	2.46736	3.30907	4.11380	2.93896	3.89484	2.69573	2.80935	2.51135	2.75615	2.91163	5.52632	4.15677	85 e - - -
	2.68618	4.42225	2.77519	2.73123	3.46354	2.40513	3.72494	3.29354	2.67741	2.69355	4.24690	2.90347	2.73739	3.18146	2.89801	2.37887	2.77519	2.98518	4.58477	3.61503	
	0.01673	4.49498	5.21732	0.61958	0.77255	0.48576	0.95510														
59	2.47307	4.96013	3.01353	1.24244	4.34641	3.50524	3.87041	3.62740	2.68197	3.33308	4.17154	3.11248	3.98117	3.02424	3.13892	2.80791	2.69794	2.80608	5.60901	4.26596	86 e - - -
	2.68618	4.42225	2.77519	2.73123	3.46354	2.40513	3.72494	3.29354	2.67741	2.69355	4.24690	2.90347	2.73739	3.18146	2.89801	2.37887	2.77519	2.98518	4.58477	3.61503	
	0.01673	4.49498	5.21732	0.61958	0.77255	0.48576	0.95510														
60	3.69684	5.57969	3.90978	3.46348	5.11985	4.06053	4.29329	4.60701	0.42127	4.05074	5.01781	3.84275	4.54853	3.47720	2.77927	3.72184	3.92081	4.29420	5.93905	4.87104	87 K - - -
	2.68618	4.42225	2.77519	2.73123	3.46354	2.40513	3.72494	3.29354	2.67741	2.69355	4.24690	2.90347	2.73739	3.18146	2.89801	2.37887	2.77519	2.98518	4.58477	3.61503	
	0.01673	4.49498	5.21732	0.61958	0.77255	0.48576	0.95510														
61	3.40819	6.10003	0.72100	1.81497	5.37510	3.50118	4.15112	4.95744	3.30298	4.41712	5.32370	2.95562	4.18515	3.33660	3.98776	3.24014	3.72372	4.49318	6.54439	4.96638	88 d - - -
	2.68618	4.42225	2.77519	2.73123	3.46354	2.40513	3.72494	3.29354	2.67741	2.69355	4.24690	2.90347	2.73739	3.18146	2.89801	2.37887	2.77519	2.98518	4.58477	3.61503	
	0.01673	4.49498	5.21732	0.61958	0.77255	0.48576	0.95510														
62	2.71026	5.20081	2.54320	2.08557	4.53612	3.47465	3.28854	4.01614	2.32678	3.30536	4.24507	2.09726	3.87405	2.77152	2.65910	2.47664	2.61032	3.58640	5.63817	4.23148	89 e - - -
	2.68618	4.42225	2.77519	2.73123	3.46354	2.40513	3.72494	3.29354	2.67741	2.69355	4.24690	2.90347	2.73739	3.18146	2.89801	2.37887	2.77519	2.98518	4.58477	3.61503	
	0.01673	4.49498	5.21732	0.61958	0.77255	0.48576	0.95510														
63	3.31707	5.41541	3.82537	3.09391	4.93460	3.93570	3.85128	4.24572	1.79728	3.67383	4.54259	3.47418	4.29604	2.98246	0.90158	3.31520	2.86422	3.92353	5.70614	4.56341	90 r - - -
	2.68618	4.42225	2.77519	2.73123	3.46354	2.40513	3.72494	3.29354	2.67741	2.69355	4.24690	2.90347	2.73739	3.18146	2.89801	2.37887	2.77519	2.98518	4.58477	3.61503	
	0.01673	4.49498	5.21732	0.61958	0.77255	0.48576	0.95510														
64	2.72486	4.49505	3.51813	2.95767	3.63901	3.67273	3.55530	2.97756	2.86762	2.35986	3.59425	3.37973	4.05789	2.20908	3.22174	2.92211	2.58496	1.75194	5.07043	3.82597	91 v - - -
	2.68618	4.42225	2.77519	2.73123	3.46354	2.40513	3.72494	3.29354	2.67741	2.69355	4.24690	2.90347	2.73739	3.18146	2.89801	2.37887	2.77519	2.98518	4.58477	3.61503	
	0.01673	4.49498	5.21732	0.61958	0.77255	0.48576	0.95510														
65	4.06451	5.25616	5.00161	4.72352	1.48879	4.76936	3.75325	3.81383	4.54930	3.12481	4.37752	4.41055	5.06930	3.61049	4.55451	4.13798	4.27883	3.71947	3.88086	0.72443	92 y - - -
	2.68618	4.42225	2.77519	2.73123	3.46354	2.40513	3.72494	3.29354	2.67741	2.69355	4.24690	2.90347	2.73739	3.18146	2.89801	2.37887	2.77519	2.98518	4.58477	3.61503	
	0.01673	4.49498	5.21732	0.61958	0.77255	0.48576	0.95510														
66	1.73491	4.42053	3.90356	3.71682	4.87871	0.85647	4.77160	4.33021	3.85228	4.01267	4.80329	3.71805	3.99187	4.07271	4.11434	2.24983	3.01477	3.62587	6.20882	5.04621	93 g - - -
	2.68591	4.42233	2.77528	2.73131	3.46347	2.40517	3.72503	3.29329	2.67749	2.69363	4.24698	2.90355	2.73748	3.18154	2.89809	2.37876	2.77504	2.98514	4.58485	3.61511	
	0.42615	1.07423	5.21732	0.12763	2.12179	0.48576	0.95510														
67	1.83070	4.25922	4.27960	3.69958	3.33752	3.56788	4.24372	2.09679	3.57616	1.58095	2.88769	3.90804	4.27889	3.79804	3.75641	3.20906	3.03287	2.33523	4.90944	3.72498	95 l - - -
	2.68618	4.42225	2.77519	2.73123	3.46354	2.40513	3.72494	3.29354	2.67741	2.69355	4.24690	2.90347	2.73739	3.18146	2.89801	2.37887	2.77519	2.98518	4.58477	3.61503	
	0.01673	4.49498	5.21732	0.61958	0.77255	0.48576	0.95510														
68	2.64576	5.00034	2.57059	2.09559	4.26576	3.50991	3.71220	3.69835	2.32439	2.18967	3.05503	3.01769	3.90498	2.57378	2.95970	2.69989	2.93867	3.34719	5.48264	4.12582	96 e - - -
	2.68618	4.42225	2.77519	2.73123	3.46354	2.40513	3.72494	3.29354	2.67741	2.69355	4.24690	2.90347	2.73739	3.18146	2.89801	2.37887	2.77519	2.98518	4.58477	3.61503	
	0.01673	4.49498	5.21732	0.61958	0.77255	0.48576	0.95510														
69	2.32128	5.58660	1.12313	2.23855	4.92225	3.19421	3.94001	4.42825	2.88106	3.92058	4.72880	2.55805	4.04344	3.08650	3.44924	2.76753	3.32915	3.99178	6.08065	4.60455	97 d - - -
	2.68618	4.42225	2.77519	2.73123	3.46354	2.40513	3.72494	3.29354	2.67741	2.69355	4.24690	2.90347	2.73739	3.18146	2.89801	2.37887	2.77519	2.98518	4.58477	3.61503	
	0.01673	4.49498	5.21732	0.61958	0.77255	0.48576	0.95510														
70	1.68905	4.47263	3.54304	3.00285	2.92407	2.22963	3.99273	3.14396	2.97164	2.84604	3.72188	3.15795	4.01372	3.28073	3.34485	2.68811	2.62487	2.65644	5.19053	3.94657	98 a - - -
	2.68618	4.42225	2.77519	2.73123	3.46354	2.40513	3.72494	3.29354	2.67741	2.69355	4.24690	2.90347	2.73739	3.18146	2.89801	2.37887	2.77519	2.98518	4.58477	3.61503	
	0.01673	4.49498	5.21732	0.61958	0.77255	0.48576	0.95510														
71	1.58725	4.33937	3.83529	3.29075	3.55794	3.58312	4.10193	2.80288	3.21637	1.82105	3.53385	3.28758	4.11482	3.50322	3.50997	2.96993	2.78198	2.26454	5.03993	3.82622	99 a - - -
	2.68618	4.42225	2.77519	2.73123	3.46354	2.40513	3.72494	3.29354	2.67741	2.69355	4.24690	2.90347	2.73739	3.18146	2.89801	2.37887	2.77519	2.98518	4.58477	3.61503	
	0.01673	4.49498	5.21732	0.61958	0.77255	0.48576	0.95510														
72	2.48432	4.54467	3.40304	2.83871	3.70587	3.62630	3.86226	2.40204	2.79449	2.23726	3.64256	3.28905	4.01028	2.67228	2.65079	2.43509	2.43100	2.82215	5.10990	3.85228	100 l - - -
	2.68618	4.42225	2.77519	2.73123	3.46354	2.40513	3.72494	3.29354	2.67741	2.69355	4.24690	2.90347	2.73739	3.18146	2.89801	2.37887	2.77519	2.98518	4.58477	3.61503	
	0.08095	4.49498	2.70912	0.61958	0.77255	0.48576	0.95510														
73	3.22727	5.31586	3.52853	3.01691	4.78672	3.79737	3.91347	4.25380	2.24163	3.70800	4.58483	2.51953	4.24557	3.06598	0.85764	3.23968	3.44943	3.90355	5.70674	4.49878	101 r - - -
	2.68618	4.42225	2.77519	2.73123	3.46354	2.40513	3.72494	3.29354	2.67741	2.69355	4.24690	2.90347	2.73739	3.18146	2.89801	2.37887	2.77519	2.98518	4.58477	3.61503	
	0.01783	4.43185	5.15420	0.61958	0.77255	0.42847	1.05413														
74	2.59096	4.46056	3.49766	2.93253	3.34172	2.15930	3.89953	2.97139	2.89372	2.27330	3.17332	2.80986	4.03258	3.20401	3.26598	2.52265	2.81192	2.60107	5.03759	3.51411	102 g - - -
	2.68618	4.42225	2.77519	2.73123	3.46354	2.40513	3.72494	3.29354	2.67741	2.69355	4.24690	2.90347	2.73739	3.18146	2.89801	2.37887	2.77519	2.98518	4.58477	3.61503	
	0.01673	4.49498	5.21732	0.61958	0.77255	0.48576	0.95510														
75	2.78443	5.29363	2.20613	2.36617	4.62773	2.57442	3.72052	4.11317	2.23442	3.59222	4.34401	1.98147	3.91193	2.58145	2.71578	2.73735	3.02108	3.67973	5.72611	4.30872	103 n - - -
	2.68618	4.42225	2.77519	2.73123	3.46354	2.40513	3.72494	3.29354	2.67741	2.69355	4.24690	2.90347	2.73739	3.18146	2.89801	2.37887	2.77519	2.98518	4.58477	3.61503	
	0.01673	4.49498	5.21732	0.61958	0.77255	0.48576	0.95510														
76	2.20429	4.60739	3.32708	2.76852	3.78059	3.60324	3.27251	3.15915	2.73758	2.60139	2.13207	2.81867	3.99015	2.90175	3.14737	2.58157	2.80630	2.90291	5.16345	3.89260	104 m - - -

```
        2.68618  4.42225  2.77519  2.73123  3.46354  2.40513  3.72494  3.29354  2.67741  2.69355  4.24690  2.90347  2.73739  3.18146  2.89801  2.37887  2.77519  2.98518  4.58477  3.61503
        0.01673  4.49498  5.21732  0.61958  0.77255  0.48576  0.95510
     77 2.24538  4.28390  3.86848  3.31173  1.87780  2.73881  4.06339  2.80015  3.23760  2.22405  3.44887  3.60836  4.10290  3.50434  3.51223  2.70602  2.64556  2.58308  4.93416  3.72212    105 f - - -
        2.68618  4.42225  2.77519  2.73123  3.46354  2.40513  3.72494  3.29354  2.67741  2.69355  4.24690  2.90347  2.73739  3.18146  2.89801  2.37887  2.77519  2.98518  4.58477  3.61503
        0.04167  4.49498  3.51819  0.61958  0.77255  0.48576  0.95510
     78 2.46968  5.16884  2.58924  2.40986  4.49841  2.74282  3.68344  3.96842  2.43023  3.47082  4.22647  2.34719  3.88137  2.68865  2.01198  2.68865  2.88066  3.46597  5.62125  4.22430    106 r - - -
        2.68618  4.42225  2.77519  2.73123  3.46354  2.40513  3.72494  3.29354  2.67741  2.69355  4.24690  2.90347  2.73739  3.18146  2.89801  2.37887  2.77519  2.98518  4.58477  3.61503
        0.01714  4.47045  5.19279  0.61958  0.77255  0.46130  0.99551
     79 2.71257  5.11092  3.00804  2.44899  4.41869  3.30282  3.68385  3.54537  2.16199  3.31019  4.16368  2.46658  3.89126  1.99916  2.46972  2.64361  2.80803  3.07816  5.56357  4.18599    107 q - - -
        2.68618  4.42225  2.77519  2.73123  3.46354  2.40513  3.72494  3.29354  2.67741  2.69355  4.24690  2.90347  2.73739  3.18146  2.89801  2.37887  2.77519  2.98518  4.58477  3.61503
        0.01673  4.49498  5.21732  0.61958  0.77255  0.48576  0.95510
     80 2.42343  4.21619  4.22322  3.64280  3.35055  3.86126  4.19330  2.19078  3.52574  2.14335  3.29315  3.85232  3.73113  3.75187  3.71218  2.94225  2.64674  1.42898  4.88018  3.68629    108 v - - -
        2.68618  4.42225  2.77519  2.73123  3.46354  2.40513  3.72494  3.29354  2.67741  2.69355  4.24690  2.90347  2.73739  3.18146  2.89801  2.37887  2.77519  2.98518  4.58477  3.61503
        0.01673  4.49498  5.21732  0.61958  0.77255  0.48576  0.95510
     81 2.86271  5.39862  2.06566  1.73083  4.72396  3.47771  2.70609  4.21932  2.56651  3.69445  4.45635  2.93739  3.94838  2.03530  3.09722  2.67357  3.10676  3.78127  5.82952  4.39265    109 e - - -
        2.68618  4.42225  2.77519  2.73123  3.46354  2.40513  3.72494  3.29354  2.67741  2.69355  4.24690  2.90347  2.73739  3.18146  2.89801  2.37887  2.77519  2.98518  4.58477  3.61503
        0.01673  4.49498  5.21732  0.61958  0.77255  0.48576  0.95510
     82 2.40453  5.17037  2.83142  2.15326  4.52340  3.50537  3.84849  3.96432  2.64411  3.52065  4.32972  3.04132  1.52762  2.55563  3.10332  2.83607  3.12213  3.58855  5.71637  4.34595    110 p - - -
        2.68618  4.42225  2.77519  2.73123  3.46354  2.40513  3.72494  3.29354  2.67741  2.69355  4.24690  2.90347  2.73739  3.18146  2.89801  2.37887  2.77519  2.98518  4.58477  3.61503
        0.01673  4.49498  5.21732  0.61958  0.77255  0.48576  0.95510
     83 3.29472  5.40322  3.70436  3.06140  4.93929  3.05346  3.87793  4.27820  1.92724  3.70673  4.57119  3.45546  4.28485  3.01232  0.86248  3.29383  3.47540  3.94176  5.73384  4.57921    111 r - - -
        2.68618  4.42225  2.77519  2.73123  3.46354  2.40513  3.72494  3.29354  2.67741  2.69355  4.24690  2.90347  2.73739  3.18146  2.89801  2.37887  2.77519  2.98518  4.58477  3.61503
        0.01673  4.49498  5.21732  0.61958  0.77255  0.48576  0.95510
     84 4.35918  5.39306  5.31148  5.14767  1.70893  4.96030  3.72240  4.00201  4.93462  3.23231  4.54356  4.54570  5.22669  4.68430  4.80226  4.34748  4.55934  3.93041  0.90135  1.60148    112 w - - -
        2.68618  4.42225  2.77519  2.73123  3.46354  2.40513  3.72494  3.29354  2.67741  2.69355  4.24690  2.90347  2.73739  3.18146  2.89801  2.37887  2.77519  2.98518  4.58477  3.61503
        0.01673  4.49498  5.21732  0.61958  0.77255  0.48576  0.95510
     85 2.58389  4.23230  3.95367  3.37480  3.34496  3.01995  4.04779  2.30459  2.91271  2.07226  2.44189  3.24997  4.13221  3.53571  3.52749  3.03292  2.79843  2.02970  4.83922  3.63690    113 v - - -
        2.68618  4.42225  2.77519  2.73123  3.46354  2.40513  3.72494  3.29354  2.67741  2.69355  4.24690  2.90347  2.73739  3.18146  2.89801  2.37887  2.77519  2.98518  4.58477  3.61503
        0.01673  4.49498  5.21732  0.61958  0.77255  0.48576  0.95510
     86 3.09789  5.59195  2.51293  0.92641  4.96064  3.48507  3.98130  4.47128  2.90384  3.96791  4.79276  2.37452  4.07526  3.13619  3.43740  2.58562  3.38442  4.03504  6.11600  4.64882    114 e - - -
        2.68618  4.42225  2.77519  2.73123  3.46354  2.40513  3.72494  3.29354  2.67741  2.69355  4.24690  2.90347  2.73739  3.18146  2.89801  2.37887  2.77519  2.98518  4.58477  3.61503
        0.01673  4.49498  5.21732  0.61958  0.77255  0.48576  0.95510
     87 2.32477  3.81878  4.07183  3.48890  3.29938  3.13021  4.07719  2.45337  3.38128  2.38967  3.30703  3.72682  4.14760  3.61447  3.58812  2.85900  2.39241  2.18481  2.33701  3.36696    115 v - - -
        2.68618  4.42225  2.77519  2.73123  3.46354  2.40513  3.72494  3.29354  2.67741  2.69355  4.24690  2.90347  2.73739  3.18146  2.89801  2.37887  2.77519  2.98518  4.58477  3.61503
        0.01673  4.49498  5.21732  0.61958  0.77255  0.48576  0.95510
     88 3.05281  4.53422  4.36043  3.77963  3.31889  4.15843  4.43179  2.21548  3.63991  1.51240  1.69979  4.06894  4.46973  2.48361  3.85226  3.44903  3.27793  2.57705  5.08485  3.94409    116 l - - -
        2.68618  4.42225  2.77519  2.73123  3.46354  2.40513  3.72494  3.29354  2.67741  2.69355  4.24690  2.90347  2.73739  3.18146  2.89801  2.37887  2.77519  2.98518  4.58477  3.61503
        0.01673  4.49498  5.21732  0.61958  0.77255  0.48576  0.95510
     89 3.15542  5.19092  3.61763  3.06501  4.59019  3.81280  3.97244  3.82078  0.79257  3.46354  4.41083  3.46890  4.25666  3.13168  2.58758  3.20848  3.39379  2.88236  5.68101  4.45533    117 k - - -
        2.68618  4.42225  2.77519  2.73123  3.46354  2.40513  3.72494  3.29354  2.67741  2.69355  4.24690  2.90347  2.73739  3.18146  2.89801  2.37887  2.77519  2.98518  4.58477  3.61503
        0.01673  4.49498  5.21732  0.61958  0.77255  0.48576  0.95510
     90 2.53894  4.24672  4.43493  3.84667  2.72285  3.96428  4.28782  2.38264  3.70687  1.34556  3.24612  4.00382  3.26460  3.89484  3.83973  3.26525  3.05427  1.94496  4.87702  3.70150    118 l - - -
        2.68618  4.42225  2.77519  2.73123  3.46354  2.40513  3.72494  3.29354  2.67741  2.69355  4.24690  2.90347  2.73739  3.18146  2.89801  2.37887  2.77519  2.98518  4.58477  3.61503
        0.01673  4.49498  5.21732  0.61958  0.77255  0.48576  0.95510
     91 2.34765  4.18689  4.10791  3.52574  1.82207  3.32556  3.38327  2.28762  3.41255  2.38140  3.30352  3.75122  4.15909  3.64199  3.60923  3.07034  2.80724  2.17714  4.79750  3.59917    119 f - - -
        2.68618  4.42225  2.77519  2.73123  3.46354  2.40513  3.72494  3.29354  2.67741  2.69355  4.24690  2.90347  2.73739  3.18146  2.89801  2.37887  2.77519  2.98518  4.58477  3.61503
        0.01673  4.49498  5.21732  0.61958  0.77255  0.48576  0.95510
     92 2.74344  4.22950  4.16240  3.58194  3.29936  3.83908  4.14470  2.31691  3.45983  1.35017  3.30376  3.80719  4.20873  3.69298  3.65393  2.46124  2.82522  2.37891  4.83799  3.11413    120 l - - -
        2.68618  4.42225  2.77519  2.73123  3.46354  2.40513  3.72494  3.29354  2.67741  2.69355  4.24690  2.90347  2.73739  3.18146  2.89801  2.37887  2.77519  2.98518  4.58477  3.61503
        0.01673  4.49498  5.21732  0.61958  0.77255  0.48576  0.95510
     93 2.08477  4.97395  3.04869  2.53221  4.39636  3.02483  3.77366  3.84410  2.55336  3.39902  4.17875  2.44673  2.95914  2.74375  3.02767  1.97045  2.67017  3.44523  5.59048  4.22821    121 s - - -
        2.68618  4.42225  2.77519  2.73123  3.46354  2.40513  3.72494  3.29354  2.67741  2.69355  4.24690  2.90347  2.73739  3.18146  2.89801  2.37887  2.77519  2.98518  4.58477  3.61503
        0.01673  4.49498  5.21732  0.61958  0.77255  0.48576  0.95510
     94 2.42841  4.22349  3.66955  3.40226  2.39562  2.98914  4.05782  1.77540  3.30982  2.36281  3.33922  3.37816  4.13666  3.55715  3.54633  2.95344  2.92580  2.33861  4.83349  3.63209    122 i - - -
        2.68618  4.42225  2.77519  2.73123  3.46354  2.40513  3.72494  3.29354  2.67741  2.69355  4.24690  2.90347  2.73739  3.18146  2.89801  2.37887  2.77519  2.98518  4.58477  3.61503
        0.01673  4.49498  5.21732  0.61958  0.77255  0.48576  0.95510
     95 3.24005  4.53430  5.14194  4.58101  3.07144  4.57658  4.98870  1.24354  4.44465  1.51778  3.36137  4.69540  4.84508  4.57986  4.52123  3.92472  3.17135  1.62056  5.38579  4.24287    123 i - - -
        2.68618  4.42225  2.77519  2.73123  3.46354  2.40513  3.72494  3.29354  2.67741  2.69355  4.24690  2.90347  2.73739  3.18146  2.89801  2.37887  2.77519  2.98518  4.58477  3.61503
        0.01673  4.49498  5.21732  0.61958  0.77255  0.48576  0.95510
     96 2.65002  5.19973  2.63630  2.00044  4.52814  3.47802  3.73259  3.99737  2.51319  3.51052  4.27641  2.80028  2.17109  2.84805  3.01030  2.39484  2.40966  3.58793  5.67224  4.27052    124 e - - -
        2.68618  4.42225  2.77519  2.73123  3.46354  2.40513  3.72494  3.29354  2.67741  2.69355  4.24690  2.90347  2.73739  3.18146  2.89801  2.37887  2.77519  2.98518  4.58477  3.61503
        0.01673  4.49498  5.21732  0.61958  0.77255  0.48576  0.95510
     97 2.57208  4.27473  3.53534  3.08471  1.98961  3.73424  4.01282  2.73924  3.18778  2.24988  2.98630  3.37136  3.14797  3.45537  3.46663  2.99977  2.92357  2.14177  4.87446  3.66441    125 f - - -
        2.68618  4.42225  2.77519  2.73123  3.46354  2.40513  3.72494  3.29354  2.67741  2.69355  4.24690  2.90347  2.73739  3.18146  2.89801  2.37887  2.77519  2.98518  4.58477  3.61503
        0.01673  4.49498  5.21732  0.61958  0.77255  0.48576  0.95510
     98 2.34468  4.53055  3.49097  2.96804  3.85936  2.19019  3.99697  3.00628  2.93510  2.33744  3.79269  3.36873  1.95757  3.26087  3.31587  2.82941  2.95673  2.92222  5.26473  4.01175    126 p - - -
        2.68618  4.42225  2.77519  2.73123  3.46354  2.40513  3.72494  3.29354  2.67741  2.69355  4.24690  2.90347  2.73739  3.18146  2.89801  2.37887  2.77519  2.98518  4.58477  3.61503
```

	0.01673	4.49498	5.21732	0.61958	0.77255	0.48576	0.95510														
99	3.67893	5.78035	2.96685	0.38898	5.23627	3.81976	4.52201	4.83724	3.50242	4.36431	5.36081	3.50588	4.46708	3.75565	3.94507	3.62108	4.00343	4.47105	6.27448	5.06823	127 E - - -
	2.68618	4.42225	2.77519	2.73123	3.46354	2.40513	3.72494	3.29354	2.67741	2.69355	4.24690	2.90347	2.73739	3.18146	2.89801	2.37887	2.77519	2.98518	4.58477	3.61503	
	0.01673	4.49498	5.21732	0.61958	0.77255	0.48576	0.95510														
100	3.23351	4.58955	4.78069	4.25984	2.64676	4.33811	4.19486	1.59179	4.10105	2.18344	3.48138	4.29275	4.65337	4.21670	4.18933	3.66055	3.46236	2.54562	4.49542	1.32576	128 y - - -
	2.68618	4.42225	2.77519	2.73123	3.46354	2.40513	3.72494	3.29354	2.67741	2.69355	4.24690	2.90347	2.73739	3.18146	2.89801	2.37887	2.77519	2.98518	4.58477	3.61503	
	0.01673	4.49498	5.21732	0.61958	0.77255	0.48576	0.95510														
101	2.45160	4.89299	3.10057	2.54667	4.14212	3.52454	3.73834	3.55770	2.53626	2.85985	3.62442	2.41702	3.81359	2.50579	2.99660	1.84924	2.93120	3.05369	5.40607	4.07235	129 s - - -
	2.68618	4.42225	2.77519	2.73123	3.46354	2.40513	3.72494	3.29354	2.67741	2.69355	4.24690	2.90347	2.73739	3.18146	2.89801	2.37887	2.77519	2.98518	4.58477	3.61503	
	0.01673	4.49498	5.21732	0.61958	0.77255	0.48576	0.95510														
102	0.53063	4.42403	4.05181	3.89016	4.75822	3.22253	4.84791	4.08225	3.93721	3.88898	4.75135	3.81460	4.03772	4.19503	4.14134	2.48869	3.05522	3.49594	6.14469	4.97091	130 A - - -
	2.68618	4.42225	2.77519	2.73123	3.46354	2.40513	3.72494	3.29354	2.67741	2.69355	4.24690	2.90347	2.73739	3.18146	2.89801	2.37887	2.77519	2.98518	4.58477	3.61503	
	0.01673	4.49498	5.21732	0.61958	0.77255	0.48576	0.95510														
103	1.82741	4.33120	3.71948	3.14951	3.46185	3.40441	3.09912	2.24839	3.08647	2.54163	2.77211	3.50963	4.08364	3.37167	3.39966	2.68436	2.85020	2.47157	4.92985	3.71056	131 a - - -
	2.68618	4.42225	2.77519	2.73123	3.46354	2.40513	3.72494	3.29354	2.67741	2.69355	4.24690	2.90347	2.73739	3.18146	2.89801	2.37887	2.77519	2.98518	4.58477	3.61503	
	0.01673	4.49498	5.21732	0.61958	0.77255	0.48576	0.95510														
104	2.24433	4.22195	3.16235	2.59500	4.13128	3.33255	3.49820	3.10862	2.20376	3.14567	3.96773	3.10239	3.94762	2.90657	1.78716	2.75276	2.95827	3.22463	5.39049	4.07394	132 r - - -
	2.68618	4.42225	2.77519	2.73123	3.46354	2.40513	3.72494	3.29354	2.67741	2.69355	4.24690	2.90347	2.73739	3.18146	2.89801	2.37887	2.77519	2.98518	4.58477	3.61503	
	0.01673	4.49498	5.21732	0.61958	0.77255	0.48576	0.95510														
105	1.43347	3.03794	3.67848	3.18928	4.21396	2.05459	4.21967	3.61471	3.16392	3.28926	4.12879	3.47426	3.97633	3.10988	3.53193	2.01097	2.94826	3.21350	5.57796	4.33982	133 a - - -
	2.68618	4.42225	2.77519	2.73123	3.46354	2.40513	3.72494	3.29354	2.67741	2.69355	4.24690	2.90347	2.73739	3.18146	2.89801	2.37887	2.77519	2.98518	4.58477	3.61503	
	0.01673	4.49498	5.21732	0.61958	0.77255	0.48576	0.95510														
106	2.45755	3.76958	3.82600	2.89362	2.81473	3.72455	4.01324	2.75320	3.17895	2.48861	1.99309	3.57865	4.10487	3.44891	3.46189	2.46938	2.47929	2.54486	4.88672	3.67602	134 m - - -
	2.68618	4.42225	2.77519	2.73123	3.46354	2.40513	3.72494	3.29354	2.67741	2.69355	4.24690	2.90347	2.73739	3.18146	2.89801	2.37887	2.77519	2.98518	4.58477	3.61503	
	0.01673	4.49498	5.21732	0.61958	0.77255	0.48576	0.95510														
107	1.09449	4.47078	3.74865	3.40341	4.66048	2.01769	4.48803	4.10091	3.45943	3.75509	4.55435	3.57422	2.21078	3.73385	3.80329	2.45086	2.98528	3.51434	5.97747	4.76154	135 a - - -
	2.68618	4.42225	2.77519	2.73123	3.46354	2.40513	3.72494	3.29354	2.67741	2.69355	4.24690	2.90347	2.73739	3.18146	2.89801	2.37887	2.77519	2.98518	4.58477	3.61503	
	0.01673	4.49498	5.21732	0.61958	0.77255	0.48576	0.95510														
108	2.70916	4.50703	3.47482	2.90520	3.40058	3.65301	3.07211	2.98706	2.82623	2.49560	1.85610	3.15958	4.03333	2.97810	2.63209	2.89159	2.91020	2.78911	5.06813	3.81755	136 m - - -
	2.68618	4.42225	2.77519	2.73123	3.46354	2.40513	3.72494	3.29354	2.67741	2.69355	4.24690	2.90347	2.73739	3.18146	2.89801	2.37887	2.77519	2.98518	4.58477	3.61503	
	0.01673	4.49498	5.21732	0.61958	0.77255	0.48576	0.95510														
109	2.23615	4.32231	4.54594	3.84358	3.34651	4.08926	4.42520	2.27250	3.82501	1.34840	2.67257	4.12795	4.42768	4.01415	3.96097	3.39581	3.15000	1.83033	4.99901	3.83183	137 l - - -
	2.68618	4.42225	2.77519	2.73123	3.46354	2.40513	3.72494	3.29354	2.67741	2.69355	4.24690	2.90347	2.73739	3.18146	2.89801	2.37887	2.77519	2.98518	4.58477	3.61503	
	0.01673	4.49498	5.21732	0.61958	0.77255	0.48576	0.95510														
110	2.36203	4.15169	4.26649	3.67605	3.01329	2.40614	4.13293	1.88748	3.53941	2.25085	3.26340	3.84249	4.18935	3.74332	3.68695	3.11386	2.93466	2.18968	3.02692	3.57609	138 i - - -
	2.68618	4.42225	2.77519	2.73123	3.46354	2.40513	3.72494	3.29354	2.67741	2.69355	4.24690	2.90347	2.73739	3.18146	2.89801	2.37887	2.77519	2.98518	4.58477	3.61503	
	0.01673	4.49498	5.21732	0.61958	0.77255	0.48576	0.95510														
111	2.75128	5.28210	1.70269	2.42466	4.61207	3.43757	3.58662	4.08465	2.51644	3.58284	4.35330	2.93451	3.94378	2.83209	1.88763	2.51357	3.06508	3.67121	5.73011	4.32790	139 d - - -
	2.68618	4.42225	2.77519	2.73123	3.46354	2.40513	3.72494	3.29354	2.67741	2.69355	4.24690	2.90347	2.73739	3.18146	2.89801	2.37887	2.77519	2.98518	4.58477	3.61503	
	0.01673	4.49498	5.21732	0.61958	0.77255	0.48576	0.95510														
112	2.08884	4.24997	3.64246	3.07371	3.07238	3.68660	3.95290	2.77349	3.02058	2.23442	3.47983	3.45807	4.06739	2.91979	3.35556	2.20781	2.45391	2.45759	4.96309	3.73642	140 a - - -
	2.68618	4.42225	2.77519	2.73123	3.46354	2.40513	3.72494	3.29354	2.67741	2.69355	4.24690	2.90347	2.73739	3.18146	2.89801	2.37887	2.77519	2.98518	4.58477	3.61503	
	0.01673	4.49498	5.21732	0.61958	0.77255	0.48576	0.95510														
113	1.94135	4.17182	4.18958	3.60324	2.87729	3.23111	4.12058	2.40159	3.48050	2.36040	3.17789	3.80223	4.18039	3.69800	3.65489	3.05035	2.93567	1.63085	4.79295	3.31093	141 v - - -
	2.68618	4.42225	2.77519	2.73123	3.46354	2.40513	3.72494	3.29354	2.67741	2.69355	4.24690	2.90347	2.73739	3.18146	2.89801	2.37887	2.77519	2.98518	4.58477	3.61503	
	0.01673	4.49498	5.21732	0.61958	0.77255	0.48576	0.95510														
114	2.59983	5.12118	2.85746	2.24063	4.42977	3.48747	3.68374	3.88952	2.36108	3.41070	4.17411	2.81703	2.19506	2.75481	2.59770	2.63616	2.42804	3.29224	5.57720	4.19073	142 p - - -
	2.68618	4.42225	2.77519	2.73123	3.46354	2.40513	3.72494	3.29354	2.67741	2.69355	4.24690	2.90347	2.73739	3.18146	2.89801	2.37887	2.77519	2.98518	4.58477	3.61503	
	0.01673	4.49498	5.21732	0.61958	0.77255	0.48576	0.95510														
115	1.79993	4.76938	3.17486	2.81027	4.59438	2.00217	4.07749	4.04780	2.93627	3.63446	4.42481	1.71686	3.43626	3.24341	3.38424	2.56060	3.02135	3.55629	5.83177	4.51018	143 n - - -
	2.68618	4.42225	2.77519	2.73123	3.46354	2.40513	3.72494	3.29354	2.67741	2.69355	4.24690	2.90347	2.73739	3.18146	2.89801	2.37887	2.77519	2.98518	4.58477	3.61503	
	0.01673	4.49498	5.21732	0.61958	0.77255	0.48576	0.95510														
116	2.11387	5.07326	2.88407	2.08915	4.35961	3.49920	3.64467	3.80436	2.48673	3.35501	3.73311	2.99715	2.14129	2.63617	2.96527	2.71965	2.95997	3.43352	5.54831	4.17643	144 e - - -
	2.68618	4.42225	2.77519	2.73123	3.46354	2.40513	3.72494	3.29354	2.67741	2.69355	4.24690	2.90347	2.73739	3.18146	2.89801	2.37887	2.77519	2.98518	4.58477	3.61503	
	0.01673	4.49498	5.21732	0.61958	0.77255	0.48576	0.95510														
117	2.63067	5.59190	2.18984	1.06161	4.91834	2.65844	3.92517	4.42424	2.85102	3.91185	4.71556	2.94187	4.03783	3.03587	3.41296	2.95822	3.31673	3.98698	6.06663	4.59148	145 e - - -
	2.68618	4.42225	2.77519	2.73123	3.46354	2.40513	3.72494	3.29354	2.67741	2.69355	4.24690	2.90347	2.73739	3.18146	2.89801	2.37887	2.77519	2.98518	4.58477	3.61503	
	0.01673	4.49498	5.21732	0.61958	0.77255	0.48576	0.95510														
118	2.57848	4.27264	3.90707	3.32961	3.36821	3.76332	4.04229	2.31688	3.23233	1.57993	3.36117	3.63557	3.38616	2.56284	3.49286	3.03374	2.94245	2.50905	3.70013	3.66243	146 l - - -
	2.68618	4.42225	2.77519	2.73123	3.46354	2.40513	3.72494	3.29354	2.67741	2.69355	4.24690	2.90347	2.73739	3.18146	2.89801	2.37887	2.77519	2.98518	4.58477	3.61503	
	0.01673	4.49498	5.21732	0.61958	0.77255	0.48576	0.95510														
119	3.29221	5.48724	3.73368	2.99806	4.97632	3.92159	2.26588	4.31381	1.69259	3.70344	4.55083	3.40777	4.26098	2.73732	1.18140	3.26362	3.44125	3.97407	5.71068	4.54382	147 r - - -
	2.68618	4.42225	2.77519	2.73123	3.46354	2.40513	3.72494	3.29354	2.67741	2.69355	4.24690	2.90347	2.73739	3.18146	2.89801	2.37887	2.77519	2.98518	4.58477	3.61503	
	0.01673	4.49498	5.21732	0.61958	0.77255	0.48576	0.95510														
120	2.74251	4.97228	3.05207	2.56027	4.29043	3.51507	3.78259	3.70321	2.55325	3.29819	4.10887	1.78472	3.94606	2.21248	2.99900	2.76730	2.39749	2.77208	5.52118	4.17913	148 n - - -
	2.68618	4.42225	2.77519	2.73123	3.46354	2.40513	3.72494	3.29354	2.67741	2.69355	4.24690	2.90347	2.73739	3.18146	2.89801	2.37887	2.77519	2.98518	4.58477	3.61503	
	0.01673	4.49498	5.21732	0.61958	0.77255	0.48576	0.95510														

121	1.96335	4.41878	3.93802	3.72732	4.81749	0.80643	4.75381	4.20719	3.81627	3.93003	4.73979	3.72763	3.99883	4.05732	4.07360	2.67742	2.37694	3.55990	6.15944	4.99773	149 g - - -
	2.68618	4.42225	2.77519	2.73123	3.46354	2.40513	3.72494	3.29354	2.67741	2.69355	4.24690	2.90347	2.73739	3.18146	2.89801	2.37887	2.77519	2.98518	4.58477	3.61503	
	0.01673	4.49498	5.21732	0.61958	0.77255	0.48576	0.95510														
122	2.36968	3.37524	4.27800	3.69103	2.50414	3.83609	4.10966	2.60675	3.55205	1.99133	3.28012	3.85045	4.20340	3.38206	3.69753	3.12950	2.95560	2.41945	4.71298	1.90760	150 y - - -
	2.68618	4.42225	2.77519	2.73123	3.46354	2.40513	3.72494	3.29354	2.67741	2.69355	4.24690	2.90347	2.73739	3.18146	2.89801	2.37887	2.77519	2.98518	4.58477	3.61503	
	0.01673	4.49498	5.21732	0.61958	0.77255	0.48576	0.95510														
123	1.99224	4.37634	3.65510	3.08763	3.50714	3.69357	3.66221	2.85974	3.01938	1.87672	3.02641	3.46884	4.07523	3.09717	3.34773	2.94864	2.21933	2.64366	4.96753	3.74238	151 l - - -
	2.68618	4.42225	2.77519	2.73123	3.46354	2.40513	3.72494	3.29354	2.67741	2.69355	4.24690	2.90347	2.73739	3.18146	2.89801	2.37887	2.77519	2.98518	4.58477	3.61503	
	0.01673	4.49498	5.21732	0.61958	0.77255	0.48576	0.95510														
124	1.94500	4.18095	4.12075	3.53581	2.90854	3.20092	3.72673	2.44429	3.42133	2.34850	2.19261	3.75790	4.16077	3.64768	3.61392	3.07445	2.92663	2.09503	4.79312	3.59924	152 a - - -
	2.68618	4.42225	2.77519	2.73123	3.46354	2.40513	3.72494	3.29354	2.67741	2.69355	4.24690	2.90347	2.73739	3.18146	2.89801	2.37887	2.77519	2.98518	4.58477	3.61503	
	0.01673	4.49498	5.21732	0.61958	0.77255	0.48576	0.95510														
125	3.13260	5.24268	3.10316	2.90177	4.80823	2.80995	4.19713	4.37356	2.89874	3.89822	4.77900	3.37707	4.22619	0.73732	3.22980	3.16296	3.46683	3.95698	5.94282	4.65565	153 q - - -
	2.68618	4.42225	2.77519	2.73123	3.46354	2.40513	3.72494	3.29354	2.67741	2.69355	4.24690	2.90347	2.73739	3.18146	2.89801	2.37887	2.77519	2.98518	4.58477	3.61503	
	0.01673	4.49498	5.21732	0.61958	0.77255	0.48576	0.95510														
126	2.58329	4.27315	4.08286	3.52896	3.46889	3.73741	4.19554	2.65199	3.42515	2.50122	1.66595	3.76233	4.17908	3.68126	3.65588	2.53724	2.65118	1.88662	4.98641	3.78877	154 m - - -
	2.68618	4.42225	2.77519	2.73123	3.46354	2.40513	3.72494	3.29354	2.67741	2.69355	4.24690	2.90347	2.73739	3.18146	2.89801	2.37887	2.77519	2.98518	4.58477	3.61503	
	0.01673	4.49498	5.21732	0.61958	0.77255	0.48576	0.95510														
127	3.12193	4.46301	4.88654	3.72233	3.45236	4.38441	4.75519	1.40893	4.17133	1.36059	3.09442	4.45901	4.68174	4.33800	4.27948	3.71226	3.35655	1.77711	5.23579	4.08623	155 l - - -
	2.68618	4.42225	2.77519	2.73123	3.46354	2.40513	3.72494	3.29354	2.67741	2.69355	4.24690	2.90347	2.73739	3.18146	2.89801	2.37887	2.77519	2.98518	4.58477	3.61503	
	0.01673	4.49498	5.21732	0.61958	0.77255	0.48576	0.95510														
128	3.80564	5.89450	0.32817	2.95132	5.39014	3.82026	4.65849	5.15285	3.88212	4.65495	5.68746	3.52525	4.51516	3.92562	4.45143	3.72439	4.16903	4.73584	6.41264	5.21317	156 D - - -
	2.68618	4.42225	2.77519	2.73123	3.46354	2.40513	3.72494	3.29354	2.67741	2.69355	4.24690	2.90347	2.73739	3.18146	2.89801	2.37887	2.77519	2.98518	4.58477	3.61503	
	0.01673	4.49498	5.21732	0.61958	0.77255	0.48576	0.95510														
129	3.67893	5.78035	2.96685	0.38898	5.23627	3.81976	4.52201	4.83724	3.50242	4.36431	5.36081	3.50588	4.46708	3.75565	3.94507	3.62108	4.00343	4.47105	6.27448	5.06823	157 E - - -
	2.68618	4.42225	2.77519	2.73123	3.46354	2.40513	3.72494	3.29354	2.67741	2.69355	4.24690	2.90347	2.73739	3.18146	2.89801	2.37887	2.77519	2.98518	4.58477	3.61503	
	0.01673	4.49498	5.21732	0.61958	0.77255	0.48576	0.95510														
130	2.88091	4.40731	4.97790	4.40256	2.37658	4.36470	4.74938	1.65745	4.24791	2.05893	3.00197	4.48681	4.67005	4.39412	4.31551	3.69799	3.32065	1.25262	5.20023	4.03750	158 v - - -
	2.68618	4.42225	2.77519	2.73123	3.46354	2.40513	3.72494	3.29354	2.67741	2.69355	4.24690	2.90347	2.73739	3.18146	2.89801	2.37887	2.77519	2.98518	4.58477	3.61503	
	0.01673	4.49498	5.21732	0.61958	0.77255	0.48576	0.95510														
131	3.87087	5.57570	4.43644	3.86558	5.10196	4.17715	4.48854	4.73143	2.76424	4.13163	5.14919	4.15934	4.68077	3.70881	0.34313	3.94815	4.11413	4.43503	5.94720	4.93659	159 R - - -
	2.68618	4.42225	2.77519	2.73123	3.46354	2.40513	3.72494	3.29354	2.67741	2.69355	4.24690	2.90347	2.73739	3.18146	2.89801	2.37887	2.77519	2.98518	4.58477	3.61503	
	0.01673	4.49498	5.21732	0.61958	0.77255	0.48576	0.95510														
132	3.94060	5.56047	3.99214	3.85233	3.94950	4.15562	0.32649	4.75510	3.69252	4.14335	5.26938	4.21957	4.75772	4.23947	3.89783	4.02004	4.28299	4.47690	5.38709	3.89157	160 H - - -
	2.68618	4.42225	2.77519	2.73123	3.46354	2.40513	3.72494	3.29354	2.67741	2.69355	4.24690	2.90347	2.73739	3.18146	2.89801	2.37887	2.77519	2.98518	4.58477	3.61503	
	0.01673	4.49498	5.21732	0.61958	0.77255	0.48576	0.95510														
133	2.38532	4.61923	3.35683	2.82274	3.96319	3.02063	3.91676	3.35080	2.80513	2.85988	3.87047	3.26277	3.97412	3.13661	3.21937	1.77636	1.87999	3.04901	5.32246	3.58215	161 s - - -
	2.68618	4.42225	2.77519	2.73123	3.46354	2.40513	3.72494	3.29354	2.67741	2.69355	4.24690	2.90347	2.73739	3.18146	2.89801	2.37887	2.77519	2.98518	4.58477	3.61503	
	0.01673	4.49498	5.21732	0.61958	0.77255	0.48576	0.95510														
134	2.78591	5.15033	2.95534	2.48286	4.45807	3.51879	3.03965	3.91590	2.47371	3.44624	4.23049	2.26821	3.94026	2.01886	2.92241	2.77037	1.98158	3.53375	5.61244	4.24057	162 t - - -
	2.68618	4.42225	2.77519	2.73123	3.46354	2.40513	3.72494	3.29354	2.67741	2.69355	4.24690	2.90347	2.73739	3.18146	2.89801	2.37887	2.77519	2.98518	4.58477	3.61503	
	0.01673	4.49498	5.21732	0.61958	0.77255	0.48576	0.95510														
135	2.82404	4.44904	3.83757	3.27568	3.54062	3.84256	4.11918	1.60880	3.15055	2.15699	3.48038	3.64142	4.21593	2.21144	3.45087	3.11535	2.56076	2.49547	5.07920	3.86490	163 i - - -
	2.68618	4.42225	2.77519	2.73123	3.46354	2.40513	3.72494	3.29354	2.67741	2.69355	4.24690	2.90347	2.73739	3.18146	2.89801	2.37887	2.77519	2.98518	4.58477	3.61503	
	0.01673	4.49498	5.21732	0.61958	0.77255	0.48576	0.95510														
136	2.72938	3.12787	3.12172	2.05057	4.15084	3.54707	3.76005	3.54528	2.53633	3.00122	3.98885	3.09212	3.94604	1.73681	2.97477	2.76929	2.96340	3.06452	5.41674	4.09038	164 q - - -
	2.68618	4.42225	2.77519	2.73123	3.46354	2.40513	3.72494	3.29354	2.67741	2.69355	4.24690	2.90347	2.73739	3.18146	2.89801	2.37887	2.77519	2.98518	4.58477	3.61503	
	0.01673	4.49498	5.21732	0.61958	0.77255	0.48576	0.95510														
137	2.15661	4.38211	3.63448	3.06690	3.51967	2.74205	3.95426	2.67302	3.01180	2.53134	2.14220	3.45405	4.06798	2.86447	3.34907	2.93756	2.77225	2.48185	4.97646	3.74891	165 m - - -
	2.68618	4.42225	2.77519	2.73123	3.46354	2.40513	3.72494	3.29354	2.67741	2.69355	4.24690	2.90347	2.73739	3.18146	2.89801	2.37887	2.77519	2.98518	4.58477	3.61503	
	0.01673	4.49498	5.21732	0.61958	0.77255	0.48576	0.95510														
138	2.52774	4.69974	3.43769	2.90190	2.54406	3.71466	2.95608	3.25931	2.87787	2.90196	3.80242	1.86513	4.10018	3.19669	3.27546	2.95404	3.06035	3.01255	4.82079	2.39909	166 n - - -
	2.68618	4.42225	2.77519	2.73123	3.46354	2.40513	3.72494	3.29354	2.67741	2.69355	4.24690	2.90347	2.73739	3.18146	2.89801	2.37887	2.77519	2.98518	4.58477	3.61503	
	0.01673	4.49498	5.21732	0.61958	0.77255	0.48576	0.95510														
139	2.62601	4.20670	4.41664	3.82736	2.87228	3.92400	4.25296	2.15002	3.68290	1.30531	3.26285	3.97261	4.28396	3.87354	3.81071	2.82206	2.83392	2.20579	4.84786	3.66750	167 l - - -
	2.68618	4.42225	2.77519	2.73123	3.46354	2.40513	3.72494	3.29354	2.67741	2.69355	4.24690	2.90347	2.73739	3.18146	2.89801	2.37887	2.77519	2.98518	4.58477	3.61503	
	0.01673	4.49498	5.21732	0.61958	0.77255	0.48576	0.95510														
140	2.43274	4.94358	3.07728	2.53291	4.19597	3.53155	3.72434	3.14884	1.78832	2.87920	3.48038	2.42055	3.92061	2.86659	2.62807	2.73597	2.94118	3.28234	5.43370	4.09558	168 k - - -
	2.68618	4.42225	2.77519	2.73123	3.46354	2.40513	3.72494	3.29354	2.67741	2.69355	4.24690	2.90347	2.73739	3.18146	2.89801	2.37887	2.77519	2.98518	4.58477	3.61503	
	0.01673	4.49498	5.21732	0.61958	0.77255	0.48576	0.95510														
141	2.75260	5.16480	2.91337	2.47327	4.48864	3.52473	2.85524	3.94524	1.76288	3.44956	4.21581	3.00619	3.91481	2.79995	2.12412	2.40807	2.97830	3.54700	5.59490	3.40828	169 k - - -
	2.68618	4.42225	2.77519	2.73123	3.46354	2.40513	3.72494	3.29354	2.67741	2.69355	4.24690	2.90347	2.73739	3.18146	2.89801	2.37887	2.77519	2.98518	4.58477	3.61503	
	0.01673	4.49498	5.21732	0.61958	0.77255	0.48576	0.95510														
142	2.77484	4.38729	3.81717	3.25261	3.21117	3.19130	3.05368	2.79878	3.17595	2.08876	3.49830	3.05779	4.15661	3.45308	3.47182	3.04609	3.00583	2.66196	2.95530	2.00303	170 y - - -
	2.68618	4.42225	2.77519	2.73123	3.46354	2.40513	3.72494	3.29354	2.67741	2.69355	4.24690	2.90347	2.73739	3.18146	2.89801	2.37887	2.77519	2.98518	4.58477	3.61503	
	0.01673	4.49498	5.21732	0.61958	0.77255	0.48576	0.95510														
143	4.09407	5.24930	5.11677	4.84305	1.53319	4.80692	3.16980	3.83027	4.65974	2.55374	4.39690	4.44540	5.09392	4.53747	4.62343	4.16969	4.30276	3.72621	3.85601	0.76727	171 y - - -

```
      2.68618  4.42225  2.77519  2.73123  3.46354  2.40513  3.72494  3.29354  2.67741  2.69355  4.24690  2.90347  2.73739  3.18146  2.89801  2.37887  2.77519  2.98518  4.58477  3.61503
      0.01673  4.49498  5.21732  0.61958  0.77255  0.48576  0.95510
144   2.16839  4.37401  3.66389  3.10122  3.52495  3.67876  3.97643  2.86398  3.04179  2.23525  2.00663  2.84703  4.07394  3.33915  3.37143  2.58151  2.91405  2.55265  4.98502  3.75973   172 m - - -
      2.68618  4.42225  2.77519  2.73123  3.46354  2.40513  3.72494  3.29354  2.67741  2.69355  4.24690  2.90347  2.73739  3.18146  2.89801  2.37887  2.77519  2.98518  4.58477  3.61503
      0.01673  4.49498  5.21732  0.61958  0.77255  0.48576  0.95510
145   2.76905  5.27644  2.61077  1.99310  4.61586  3.48343  3.70575  4.09979  1.68034  3.57555  4.32406  2.36273  3.90469  2.74531  2.78957  2.51988  2.99458  3.66515  5.70520  4.29292   173 k - - -
      2.68618  4.42225  2.77519  2.73123  3.46354  2.40513  3.72494  3.29354  2.67741  2.69355  4.24690  2.90347  2.73739  3.18146  2.89801  2.37887  2.77519  2.98518  4.58477  3.61503
      0.01673  4.49498  5.21732  0.61958  0.77255  0.48576  0.95510
146   2.75359  5.10360  3.03350  2.49269  4.38265  3.52910  2.33993  3.84393  2.42537  3.37947  4.16291  1.94601  3.92473  2.57476  2.62229  2.74454  2.83205  3.47111  5.54496  3.43596   174 n - - -
      2.68618  4.42225  2.77519  2.73123  3.46354  2.40513  3.72494  3.29354  2.67741  2.69355  4.24690  2.90347  2.73739  3.18146  2.89801  2.37887  2.77519  2.98518  4.58477  3.61503
      0.01673  4.49498  5.21732  0.61958  0.77255  0.48576  0.95510
147   2.33006  4.36485  3.75543  2.83838  3.02249  3.29075  3.96243  2.84801  3.12831  2.56435  3.47537  3.54286  4.12080  3.40950  3.43459  3.00489  2.96806  2.63634  3.72531  1.68010   175 y - - -
      2.68618  4.42225  2.77519  2.73123  3.46354  2.40513  3.72494  3.29354  2.67741  2.69355  4.24690  2.90347  2.73739  3.18146  2.89801  2.37887  2.77519  2.98518  4.58477  3.61503
      0.01673  4.49498  5.21732  0.61958  0.77255  0.48576  0.95510
148   2.57805  4.68915  3.24586  2.68694  3.87684  3.57801  2.94870  2.14410  2.66735  2.92458  3.77624  2.71637  3.05591  2.69759  3.09786  2.68978  2.78969  2.86961  5.22985  3.63136   176 i - - -
      2.68618  4.42225  2.77519  2.73123  3.46354  2.40513  3.72494  3.29354  2.67741  2.69355  4.24690  2.90347  2.73739  3.18146  2.89801  2.37887  2.77519  2.98518  4.58477  3.61503
      0.09578  4.49498  2.52364  0.61958  0.77255  0.48576  0.95510
149   3.21096  5.60439  0.75816  2.48575  4.94822  3.52083  4.07663  4.52150  3.02429  4.02980  4.90437  3.05480  4.13631  3.25610  2.76810  3.13096  3.50993  4.10720  6.10533  4.68577   177 d - - -
      2.68618  4.42225  2.77519  2.73123  3.46354  2.40513  3.72494  3.29354  2.67741  2.69355  4.24690  2.90347  2.73739  3.18146  2.89801  2.37887  2.77519  2.98518  4.58477  3.61503
      0.07091  4.41729  2.87555  0.61958  0.77255  0.60550  0.78923
150   2.34892  4.57933  3.63920  3.49267  4.64655  3.30995  4.61836  4.02283  3.59115  3.74114  4.68353  3.67310  0.69899  3.92694  3.85526  2.84930  3.17168  3.53600  5.97384  4.79146   178 p - - -
      2.68618  4.42225  2.77519  2.73123  3.46354  2.40513  3.72494  3.29354  2.67741  2.69355  4.24690  2.90347  2.73739  3.18146  2.89801  2.37887  2.77519  2.98518  4.58477  3.61503
      0.01906  4.36545  5.08779  0.61958  0.77255  0.67369  0.71299
151   1.32957  4.69070  2.93835  2.52718  3.92458  3.50713  3.83010  3.32476  2.70760  2.98998  3.84942  3.15219  3.95297  3.04504  3.12806  2.77741  2.95270  3.04080  4.30632  3.98128   179 a - - -
      2.68618  4.42225  2.77519  2.73123  3.46354  2.40513  3.72494  3.29354  2.67741  2.69355  4.24690  2.90347  2.73739  3.18146  2.89801  2.37887  2.77519  2.98518  4.58477  3.61503
      0.01906  4.36545  5.08779  0.61958  0.77255  0.48577  0.95510
152   3.53173  5.21181  4.31733  4.30328  5.36309  0.21799  5.34713  5.14208  4.55874  4.70405  5.71060  4.48470  4.61482  4.83833  4.69497  3.72524  4.05447  4.55901  6.26822  5.49691   180 G - - -
      2.68618  4.42225  2.77519  2.73123  3.46354  2.40513  3.72494  3.29354  2.67741  2.69355  4.24690  2.90347  2.73739  3.18146  2.89801  2.37887  2.77519  2.98518  4.58477  3.61503
      0.01755  4.44710  5.16945  0.61958  0.77255  0.44071  1.03165
153   2.53872  4.29274  4.27267  3.70504  1.50078  3.91475  2.74442  2.74377  3.57788  2.34947  2.90030  3.86722  4.27726  3.77572  3.74335  3.20605  3.06599  2.55763  3.97802  2.91473   181 f - - -
      2.68618  4.42225  2.77519  2.73123  3.46354  2.40513  3.72494  3.29354  2.67741  2.69355  4.24690  2.90347  2.73739  3.18146  2.89801  2.37887  2.77519  2.98518  4.58477  3.61503
      0.01673  4.49498  5.21732  0.61958  0.77255  0.48576  0.95510
154   2.71847  5.40790  1.58590  2.37852  4.72275  3.47964  2.90056  4.21422  2.63979  3.70421  4.47905  1.94722  3.96792  2.77440  3.16099  2.83363  2.94689  3.78782  5.85222  4.41513   182 d - - -
      2.68618  4.42225  2.77519  2.73123  3.46354  2.40513  3.72494  3.29354  2.67741  2.69355  4.24690  2.90347  2.73739  3.18146  2.89801  2.37887  2.77519  2.98518  4.58477  3.61503
      0.01673  4.49498  5.21732  0.61958  0.77255  0.48576  0.95510
155   2.69230  4.50754  2.57256  2.87023  3.65905  3.39092  3.87452  2.08125  2.83748  2.60917  3.21362  2.87887  4.01670  3.15448  3.22543  2.43187  2.91681  2.78991  3.82007  3.54289   183 i - - -
      2.68618  4.42225  2.77519  2.73123  3.46354  2.40513  3.72494  3.29354  2.67741  2.69355  4.24690  2.90347  2.73739  3.18146  2.89801  2.37887  2.77519  2.98518  4.58477  3.61503
      0.01673  4.49498  5.21732  0.61958  0.77255  0.48576  0.95510
156   2.04029  4.60952  3.33088  2.77391  3.49672  2.72849  3.84921  3.20216  2.75177  2.87687  3.73814  3.07367  3.59121  3.08074  3.16706  2.48930  2.05091  2.93549  4.26482  3.92457   184 a - - -
      2.68618  4.42225  2.77519  2.73123  3.46354  2.40513  3.72494  3.29354  2.67741  2.69355  4.24690  2.90347  2.73739  3.18146  2.89801  2.37887  2.77519  2.98518  4.58477  3.61503
      0.01673  4.49498  5.21732  0.61958  0.77255  0.48576  0.95510
157   2.70056  5.01677  3.03506  1.91462  3.64903  3.50623  3.43490  3.72868  2.46550  2.64396  3.69039  2.75569  3.89741  2.63644  2.29651  2.61128  2.93131  3.36896  5.49289  4.13160   185 e - - -
      2.68618  4.42225  2.77519  2.73123  3.46354  2.40513  3.72494  3.29354  2.67741  2.69355  4.24690  2.90347  2.73739  3.18146  2.89801  2.37887  2.77519  2.98518  4.58477  3.61503
      0.01673  4.49498  5.21732  0.61958  0.77255  0.48576  0.95510
158   2.52738  5.18058  3.00852  2.18493  4.51513  3.29855  3.18299  3.66679  1.84181  3.28305  4.22757  2.98322  3.89720  2.54135  2.18649  2.70538  2.96060  3.56635  5.61094  4.22813   186 k - - -
      2.68618  4.42225  2.77519  2.73123  3.46354  2.40513  3.72494  3.29354  2.67741  2.69355  4.24690  2.90347  2.73739  3.18146  2.89801  2.37887  2.77519  2.98518  4.58477  3.61503
      0.01673  4.49498  5.21732  0.61958  0.77255  0.48576  0.95510
159   1.63556  4.20874  3.46018  2.89566  3.64245  3.49851  3.57950  3.01057  2.85709  2.71049  3.04004  3.09973  4.02299  2.99179  3.23791  2.87620  2.40834  2.77358  4.19589  3.81505   187 a - - -
      2.68618  4.42225  2.77519  2.73123  3.46354  2.40513  3.72494  3.29354  2.67741  2.69355  4.24690  2.90347  2.73739  3.18146  2.89801  2.37887  2.77519  2.98518  4.58477  3.61503
      0.01673  4.49498  5.21732  0.61958  0.77255  0.48576  0.95510
160   2.75131  4.50009  3.56840  3.00268  2.02642  3.70500  3.25579  3.01239  2.92465  2.41357  3.60322  3.20712  4.08304  3.25317  2.20968  2.79173  2.98148  2.78439  4.90825  3.08468   188 f - - -
      2.68618  4.42225  2.77519  2.73123  3.46354  2.40513  3.72494  3.29354  2.67741  2.69355  4.24690  2.90347  2.73739  3.18146  2.89801  2.37887  2.77519  2.98518  4.58477  3.61503
      0.01673  4.49498  5.21732  0.61958  0.77255  0.48576  0.95510
161   1.88308  5.03284  2.61355  2.46930  4.31246  2.52318  3.03739  3.75335  2.48176  3.31252  4.09817  3.00333  3.89898  2.67143  2.95993  2.70205  2.84758  3.21579  5.51339  3.82838   189 a - - -
      2.68618  4.42225  2.77519  2.73123  3.46354  2.40513  3.72494  3.29354  2.67741  2.69355  4.24690  2.90347  2.73739  3.18146  2.89801  2.37887  2.77519  2.98518  4.58477  3.61503
      0.01673  4.49498  5.21732  0.61958  0.77255  0.48576  0.95510
162   2.69858  4.48593  3.08304  2.52464  3.90321  3.31554  3.72317  2.79636  2.15654  3.18868  3.99541  2.26234  3.91294  2.76892  2.51158  2.67316  2.50139  3.26374  5.42262  4.08215   190 k - - -
      2.68625  4.42232  2.77511  2.73131  3.46361  2.40491  3.72439  3.29361  2.67748  2.69362  4.24697  2.90311  2.73747  3.18154  2.89808  2.37894  2.77527  2.98526  4.58484  3.61510
      0.38417  1.15975  5.21732  0.13678  2.05699  0.48576  0.95510
163   2.69016  3.06674  2.72072  2.63575  3.99304  3.54477  3.78533  3.38925  2.62665  2.86814  3.86822  2.57775  2.34966  2.96771  3.07170  2.63197  2.81184  2.65849  5.31374  4.00788   192 p - - -
      2.68618  4.42225  2.77519  2.73123  3.46354  2.40513  3.72494  3.29354  2.67741  2.69355  4.24690  2.90347  2.73739  3.18146  2.89801  2.37887  2.77519  2.98518  4.58477  3.61503
      0.01673  4.49498  5.21732  0.61958  0.77255  0.48576  0.95510
164   2.71543  4.12938  4.42913  3.83317  2.94309  3.56132  4.13892  2.21997  3.66772  1.90311  3.24099  3.92492  3.79558  3.83980  3.75948  3.15328  2.94732  2.28184  2.88651  2.08201   193 l - - -
      2.68618  4.42225  2.77519  2.73123  3.46354  2.40513  3.72494  3.29354  2.67741  2.69355  4.24690  2.90347  2.73739  3.18146  2.89801  2.37887  2.77519  2.98518  4.58477  3.61503
      0.01673  4.49498  5.21732  0.61958  0.77255  0.48576  0.95510
165   1.91799  3.65151  4.21910  3.62943  2.88612  2.55410  4.10898  2.38791  3.49885  2.34389  3.26605  3.80969  4.17162  3.70783  3.65747  3.09236  2.92275  2.32335  3.03820  3.06181   194 a - - -
      2.68618  4.42225  2.77519  2.73123  3.46354  2.40513  3.72494  3.29354  2.67741  2.69355  4.24690  2.90347  2.73739  3.18146  2.89801  2.37887  2.77519  2.98518  4.58477  3.61503
```

	0.01673	4.49498	5.21732	0.61958	0.77255	0.48576	0.95510														
166	2.59922	4.65558	3.28312	2.71102	3.83729	2.46350	3.81245	3.22069	2.27948	2.44938	3.74562	3.20026	3.97563	3.03066	2.85421	2.73310	2.33686	2.63566	4.37199	3.92130	195 k - - -
	2.68618	4.42225	2.77519	2.73123	3.46354	2.40513	3.72494	3.29354	2.67741	2.69355	4.24690	2.90347	2.73739	3.18146	2.89801	2.37887	2.77519	2.98518	4.58477	3.61503	
	0.01830	4.49498	4.96589	0.61958	0.77255	0.48576	0.95510														
167	2.41638	4.56939	3.41396	2.86857	3.89533	2.50023	3.93148	3.27479	2.84457	2.53533	3.81379	3.29564	3.36735	3.16915	3.24955	2.29406	1.90711	2.71239	5.27326	4.00782	196 t - - -
	2.68618	4.42225	2.77519	2.73123	3.46354	2.40513	3.72494	3.29354	2.67741	2.69355	4.24690	2.90347	2.73739	3.18146	2.89801	2.37887	2.77519	2.98518	4.58477	3.61503	
	0.08590	4.49343	2.64320	0.61958	0.77255	0.48840	0.95090														
168	2.28557	4.18907	4.22108	3.64143	3.32102	3.84217	4.17801	1.59994	3.51867	2.36034	2.52799	3.49875	4.21606	3.74369	3.69805	2.97684	2.96340	2.00934	4.85474	3.66194	197 i - - -
	2.68618	4.42225	2.77519	2.73123	3.46354	2.40513	3.72494	3.29354	2.67741	2.69355	4.24690	2.90347	2.73739	3.18146	2.89801	2.37887	2.77519	2.98518	4.58477	3.61503	
	0.01794	4.42547	5.14781	0.61958	0.77255	0.42722	1.05647														
169	2.58892	4.85390	3.13139	2.57305	3.70693	2.07080	3.74714	3.31495	2.18637	3.11202	3.93402	3.08493	3.13252	2.90738	2.65631	2.74674	2.93166	2.87505	5.36879	4.04566	198 g - - -
	2.68618	4.42225	2.77519	2.73123	3.46354	2.40513	3.72494	3.29354	2.67741	2.69355	4.24690	2.90347	2.73739	3.18146	2.89801	2.37887	2.77519	2.98518	4.58477	3.61503	
	0.01675	4.49343	5.21577	0.61958	0.77255	0.48840	0.95090														
170	2.63418	5.39527	3.64900	2.96586	4.86307	3.87352	3.81790	4.20316	1.83319	3.63782	4.48653	3.38417	4.23290	2.74899	1.01667	3.21451	3.39471	3.86925	5.68661	4.50552	199 r - - -
	2.68618	4.42225	2.77519	2.73123	3.46354	2.40513	3.72494	3.29354	2.67741	2.69355	4.24690	2.90347	2.73739	3.18146	2.89801	2.37887	2.77519	2.98518	4.58477	3.61503	
	0.01675	4.49343	5.21577	0.61958	0.77255	0.48840	0.95090														
171	2.38068	4.68782	3.25545	2.69896	3.88388	3.57660	3.80803	3.26768	2.67330	2.82098	3.78495	3.18294	3.96900	1.94318	3.09970	2.29408	2.86449	2.45700	5.23746	3.35761	200 q - - -
	2.68618	4.42225	2.77519	2.73123	3.46354	2.40513	3.72494	3.29354	2.67741	2.69355	4.24690	2.90347	2.73739	3.18146	2.89801	2.37887	2.77519	2.98518	4.58477	3.61503	
	0.01675	4.49343	5.21577	0.61958	0.77255	0.48840	0.95090														
172	2.66062	4.46694	4.68494	4.11136	1.24399	4.22975	4.50913	2.45453	3.97108	1.83473	2.96357	4.27010	4.54857	4.12940	4.09350	3.54625	3.30336	2.20708	4.98692	3.75611	201 f - - -
	2.68618	4.42225	2.77519	2.73123	3.46354	2.40513	3.72494	3.29354	2.67741	2.69355	4.24690	2.90347	2.73739	3.18146	2.89801	2.37887	2.77519	2.98518	4.58477	3.61503	
	0.01675	4.49343	5.21577	0.61958	0.77255	0.48840	0.95090														
173	2.00980	3.52097	3.68486	3.12036	2.42368	2.49140	3.98041	2.86822	3.06362	2.47242	3.48894	3.48558	4.06886	3.00431	3.38859	2.62507	2.92474	2.64557	4.97049	3.74680	202 a - - -
	2.68618	4.42225	2.77519	2.73123	3.46354	2.40513	3.72494	3.29354	2.67741	2.69355	4.24690	2.90347	2.73739	3.18146	2.89801	2.37887	2.77519	2.98518	4.58477	3.61503	
	0.01675	4.49343	5.21577	0.61958	0.77255	0.48840	0.95090														
174	3.13387	5.75972	1.60212	1.14059	5.05896	3.47490	3.96557	4.58927	2.93895	4.05335	4.86859	2.74638	3.37269	3.11423	3.53157	2.89328	3.41024	4.13624	6.19832	4.68596	203 e - - -
	2.68618	4.42225	2.77519	2.73123	3.46354	2.40513	3.72494	3.29354	2.67741	2.69355	4.24690	2.90347	2.73739	3.18146	2.89801	2.37887	2.77519	2.98518	4.58477	3.61503	
	0.08108	4.49343	2.70757	0.61958	0.77255	0.48840	0.95090														
175	2.18802	4.96784	1.96454	2.58767	4.64690	1.50974	3.99327	4.09793	2.89290	3.67541	4.47834	3.08616	3.96051	3.15460	3.38697	2.78355	2.74087	3.63725	5.87181	4.50047	204 g - - -
	2.68618	4.42225	2.77519	2.73123	3.46354	2.40513	3.72494	3.29354	2.67741	2.69355	4.24690	2.90347	2.73739	3.18146	2.89801	2.37887	2.77519	2.98518	4.58477	3.61503	
	0.01786	4.43020	5.15255	0.61958	0.77255	0.43086	1.04969														
176	2.36524	4.33971	4.13461	3.60166	1.29236	3.83834	4.13893	2.73080	3.48885	2.43502	2.93466	3.46068	4.25182	3.73414	3.69933	3.15254	3.06782	2.55620	4.78963	3.44999	205 f - - -
	2.68618	4.42225	2.77519	2.73123	3.46354	2.40513	3.72494	3.29354	2.67741	2.69355	4.24690	2.90347	2.73739	3.18146	2.89801	2.37887	2.77519	2.98518	4.58477	3.61503	
	0.01675	4.49343	5.21577	0.61958	0.77255	0.48840	0.95090														
177	2.70325	3.64709	4.44419	3.84452	2.69483	3.85073	4.17493	1.58860	3.67744	2.14150	2.90939	3.36282	4.21566	3.85124	3.76590	2.98243	2.66197	2.14858	4.74132	3.55911	206 i - - -
	2.68618	4.42225	2.77519	2.73123	3.46354	2.40513	3.72494	3.29354	2.67741	2.69355	4.24690	2.90347	2.73739	3.18146	2.89801	2.37887	2.77519	2.98518	4.58477	3.61503	
	0.01675	4.49343	5.21577	0.61958	0.77255	0.48840	0.95090														
178	2.47532	4.52654	3.53787	3.04296	4.07367	3.42239	4.09787	3.43305	3.02556	3.13611	3.99132	2.93498	3.98987	3.34430	3.40845	1.97026	1.47544	2.38811	5.44806	4.19598	207 t - - -
	2.68618	4.42225	2.77519	2.73123	3.46354	2.40513	3.72494	3.29354	2.67741	2.69355	4.24690	2.90347	2.73739	3.18146	2.89801	2.37887	2.77519	2.98518	4.58477	3.61503	
	0.01675	4.49343	5.21577	0.61958	0.77255	0.48840	0.95090														
179	1.97961	3.79871	2.86021	2.91141	4.38891	1.28114	4.10078	3.80833	2.98770	3.44441	4.26120	3.03920	3.96431	3.29509	3.41276	2.72525	2.98868	3.37824	5.68953	4.40085	208 g - - -
	2.68618	4.42225	2.77519	2.73123	3.46354	2.40513	3.72494	3.29354	2.67741	2.69355	4.24690	2.90347	2.73739	3.18146	2.89801	2.37887	2.77519	2.98518	4.58477	3.61503	
	0.01675	4.49343	5.21577	0.61958	0.77255	0.48840	0.95090														
180	3.25370	5.81754	0.81003	2.37346	5.22994	2.52912	4.11211	4.79404	3.21245	4.27208	5.13940	2.49955	4.13550	3.29042	3.85712	3.13416	3.57903	4.31490	6.41661	4.87188	209 d - - -
	2.68618	4.42225	2.77519	2.73123	3.46354	2.40513	3.72494	3.29354	2.67741	2.69355	4.24690	2.90347	2.73739	3.18146	2.89801	2.37887	2.77519	2.98518	4.58477	3.61503	
	0.01675	4.49343	5.21577	0.61958	0.77255	0.48840	0.95090														
181	1.54464	4.78292	2.79620	2.68942	4.20532	3.46692	3.88326	3.59605	2.72143	3.05779	4.06223	3.16568	2.27016	3.05548	3.16871	2.74995	2.71479	3.19349	5.49823	4.18378	210 a - - -
	2.68618	4.42225	2.77519	2.73123	3.46354	2.40513	3.72494	3.29354	2.67741	2.69355	4.24690	2.90347	2.73739	3.18146	2.89801	2.37887	2.77519	2.98518	4.58477	3.61503	
	0.01675	4.49343	5.21577	0.61958	0.77255	0.48840	0.95090														
182	2.66117	4.38058	4.92776	4.35432	2.70409	4.32864	4.72005	1.37446	4.20306	2.22243	2.92134	4.44431	4.64418	4.36458	4.28250	3.66032	3.28973	1.41022	5.19707	4.02536	211 i - - -
	2.68618	4.42225	2.77519	2.73123	3.46354	2.40513	3.72494	3.29354	2.67741	2.69355	4.24690	2.90347	2.73739	3.18146	2.89801	2.37887	2.77519	2.98518	4.58477	3.61503	
	0.01675	4.49343	5.21577	0.61958	0.77255	0.48840	0.95090														
183	2.52873	5.12918	2.64636	1.71616	4.43589	3.49219	3.76137	3.87196	2.55784	3.20923	4.22049	2.99677	3.93162	2.89056	3.04578	2.76071	1.82884	3.49637	5.62653	4.24485	212 e - - -
	2.68618	4.42225	2.77519	2.73123	3.46354	2.40513	3.72494	3.29354	2.67741	2.69355	4.24690	2.90347	2.73739	3.18146	2.89801	2.37887	2.77519	2.98518	4.58477	3.61503	
	0.01675	4.49343	5.21577	0.61958	0.77255	0.48840	0.95090														
184	1.84675	2.81016	3.80882	3.24019	2.82070	3.17127	4.02107	2.67265	3.17035	2.52813	3.43317	3.14665	4.09241	3.44297	3.46124	2.56513	2.76895	2.48151	4.92042	3.70820	213 a - - -
	2.68618	4.42225	2.77519	2.73123	3.46354	2.40513	3.72494	3.29354	2.67741	2.69355	4.24690	2.90347	2.73739	3.18146	2.89801	2.37887	2.77519	2.98518	4.58477	3.61503	
	0.01675	4.49343	5.21577	0.61958	0.77255	0.48840	0.95090														
185	1.78954	4.36002	3.76922	3.20467	3.52449	3.71786	4.04627	2.32002	3.14136	2.44591	3.22793	3.55748	4.11864	3.42928	3.45637	1.97501	2.92161	2.44353	5.00760	3.78795	214 a - - -
	2.68608	4.42147	2.77527	2.73131	3.46361	2.40520	3.72502	3.29361	2.67748	2.69362	4.24697	2.90276	2.73747	3.18154	2.89808	2.37881	2.77527	2.98526	4.58484	3.61511	
	0.39376	1.13926	5.21577	0.13471	2.07122	0.48840	0.95090														
186	3.21579	4.51171	5.11608	4.55626	3.55056	4.55523	4.97681	1.33539	4.42049	1.64952	2.72898	4.67140	4.83162	4.57001	4.50479	3.90249	3.10032	1.46558	5.39520	4.24662	216 i - - -
	2.68618	4.42225	2.77519	2.73123	3.46354	2.40513	3.72494	3.29354	2.67741	2.69355	4.24690	2.90347	2.73739	3.18146	2.89801	2.37887	2.77519	2.98518	4.58477	3.61503	
	0.01675	4.49343	5.21577	0.61958	0.77255	0.48412	0.95774														
187	2.39282	4.07435	3.31300	2.75478	3.34923	3.33871	3.82837	3.17465	2.73210	2.85013	3.71284	2.20277	3.98344	2.77586	3.14783	2.69508	2.92702	2.91563	5.16537	2.35612	217 n - - -
	2.68618	4.42225	2.77519	2.73123	3.46354	2.40513	3.72494	3.29354	2.67741	2.69355	4.24690	2.90347	2.73739	3.18146	2.89801	2.37887	2.77519	2.98518	4.58477	3.61503	
	0.01673	4.49498	5.21732	0.61958	0.77255	0.48576	0.95510														

```
188   3.74594  4.99348  5.52193  4.97439  3.34322  5.10409  5.45263  2.05218  4.82005  0.58553  3.10124  5.20927  5.20038  4.82867  4.86241  4.49980  3.96780  2.26909  5.55428  4.53993    218 L - - -
      2.68618  4.42225  2.77519  2.73123  3.46354  2.40513  3.72494  3.29354  2.67741  2.69355  4.24690  2.90347  2.73739  3.18146  2.89801  2.37887  2.77519  2.98518  4.58477  3.61503
      0.01673  4.49498  5.21732  0.61958  0.77255  0.48576  0.95510
189   2.74783  5.04146  2.97816  2.53328  4.45322  2.91453  3.80406  3.89956  2.57765  3.45197  4.24099  2.69236  3.94008  1.78893  3.03823  2.60703  1.92879  3.50552  5.64020  4.27813    219 q - - -
      2.68618  4.42225  2.77519  2.73123  3.46354  2.40513  3.72494  3.29354  2.67741  2.69355  4.24690  2.90347  2.73739  3.18146  2.89801  2.37887  2.77519  2.98518  4.58477  3.61503
      0.01673  4.49498  5.21732  0.61958  0.77255  0.48576  0.95510
190   3.05719  4.39494  4.92285  4.33797  2.37338  4.30433  4.66362  2.00995  4.17673  1.68151  3.02365  4.42240  4.61037  4.30921  4.23689  3.63098  3.01699  1.31023  5.11115  3.96033    220 v - - -
      2.68618  4.42225  2.77519  2.73123  3.46354  2.40513  3.72494  3.29354  2.67741  2.69355  4.24690  2.90347  2.73739  3.18146  2.89801  2.37887  2.77519  2.98518  4.58477  3.61503
      0.01673  4.49498  5.21732  0.61958  0.77255  0.48576  0.95510
191   2.57579  3.78600  4.30376  3.74521  3.16141  3.79497  4.29335  2.51441  3.62607  2.47064  3.43889  3.90291  4.23934  3.84860  3.80405  2.71779  2.65892  1.14609  4.98812  3.79556    221 v - - -
      2.68618  4.42225  2.77519  2.73123  3.46354  2.40513  3.72494  3.29354  2.67741  2.69355  4.24690  2.90347  2.73739  3.18146  2.89801  2.37887  2.77519  2.98518  4.58477  3.61503
      0.01673  4.49498  5.21732  0.61958  0.77255  0.48576  0.95510
192   1.64964  4.28209  3.92978  3.35979  2.78567  2.63323  4.07999  2.56474  3.27760  2.12328  3.39649  3.65771  4.14640  3.53885  3.54138  3.03604  2.95515  2.41236  4.90891  3.69952    222 a - - -
      2.68618  4.42225  2.77519  2.73123  3.46354  2.40513  3.72494  3.29354  2.67741  2.69355  4.24690  2.90347  2.73739  3.18146  2.89801  2.37887  2.77519  2.98518  4.58477  3.61503
      0.01673  4.49498  5.21732  0.61958  0.77255  0.48576  0.95510
193   3.67893  5.78035  2.96685  0.38898  5.23627  3.81976  4.52201  4.83724  3.50242  4.36431  5.36081  3.50588  4.46708  3.75565  3.94507  3.62108  4.00343  4.47105  6.27448  5.06823    223 E - - -
      2.68618  4.42225  2.77519  2.73123  3.46354  2.40513  3.72494  3.29354  2.67741  2.69355  4.24690  2.90347  2.73739  3.18146  2.89801  2.37887  2.77519  2.98518  4.58477  3.61503
      0.01673  4.49498  5.21732  0.61958  0.77255  0.48576  0.95510
194   2.02484  4.47139  3.63038  3.14542  3.81391  3.51850  4.10454  3.18397  3.09933  2.92135  3.81616  3.48299  4.05147  3.42091  3.43987  2.82620  1.30916  2.90643  5.25231  2.91196    224 t - - -
      2.68618  4.42225  2.77519  2.73123  3.46354  2.40513  3.72494  3.29354  2.67741  2.69355  4.24690  2.90347  2.73739  3.18146  2.89801  2.37887  2.77519  2.98518  4.58477  3.61503
      0.01673  4.49498  5.21732  0.61958  0.77255  0.48576  0.95510
195   1.08683  2.36195  4.27091  3.82233  4.00183  2.72096  4.52594  3.25001  3.72829  3.07823  3.97569  3.81473  4.05385  3.96097  3.93835  2.78277  2.96378  2.29422  5.47132  4.29471    225 a - - -
      2.68618  4.42225  2.77519  2.73123  3.46354  2.40513  3.72494  3.29354  2.67741  2.69355  4.24690  2.90347  2.73739  3.18146  2.89801  2.37887  2.77519  2.98518  4.58477  3.61503
      0.01673  4.49498  5.21732  0.61958  0.77255  0.48576  0.95510
196   3.98363  5.15888  5.18087  4.88096  0.78730  4.79803  3.84175  3.49452  4.68998  2.14256  4.07238  4.50731  5.07223  4.56575  4.63655  4.16375  4.19579  3.47193  3.95786  1.68446    226 f - - -
      2.68618  4.42225  2.77519  2.73123  3.46354  2.40513  3.72494  3.29354  2.67741  2.69355  4.24690  2.90347  2.73739  3.18146  2.89801  2.37887  2.77519  2.98518  4.58477  3.61503
      0.01673  4.49498  5.21732  0.61958  0.77255  0.48576  0.95510
197   2.56873  4.47570  3.91297  3.71120  4.62741  3.27741  4.70604  3.93655  3.70191  3.74266  4.63185  3.75428  4.06080  4.03039  3.93602  2.51128  0.63250  3.42866  6.01754  4.80930    227 T - - -
      2.68618  4.42225  2.77519  2.73123  3.46354  2.40513  3.72494  3.29354  2.67741  2.69355  4.24690  2.90347  2.73739  3.18146  2.89801  2.37887  2.77519  2.98518  4.58477  3.61503
      0.01673  4.49498  5.21732  0.61958  0.77255  0.48576  0.95510
198   3.41024  5.34842  3.28470  3.25330  4.83180  3.74694  4.64426  4.73370  3.63548  4.31034  5.29557  0.40869  4.44400  3.95587  3.98042  3.47564  3.83335  4.28425  6.05478  4.76441    228 N - - -
      2.68618  4.42225  2.77519  2.73123  3.46354  2.40513  3.72494  3.29354  2.67741  2.69355  4.24690  2.90347  2.73739  3.18146  2.89801  2.37887  2.77519  2.98518  4.58477  3.61503
      0.01673  4.49498  5.21732  0.61958  0.77255  0.48576  0.95510
199   2.74806  4.39866  3.80068  3.24086  3.55251  3.74874  4.09318  2.61623  3.17011  2.05649  3.50612  3.59473  2.20385  3.46583  3.48519  3.02677  1.94785  2.28546  5.06081  3.84300    229 t - - -
      2.68618  4.42225  2.77519  2.73123  3.46354  2.40513  3.72494  3.29354  2.67741  2.69355  4.24690  2.90347  2.73739  3.18146  2.89801  2.37887  2.77519  2.98518  4.58477  3.61503
      0.01673  4.49498  5.21732  0.61958  0.77255  0.48576  0.95510
200   3.33922  4.74416  4.74985  4.18296  2.94179  4.48732  4.71105  2.39028  4.02101  0.72485  3.11581  4.44139  4.73590  3.20948  4.17630  3.80531  3.55912  2.66880  5.15561  3.98850    230 l - - -
      2.68618  4.42225  2.77519  2.73123  3.46354  2.40513  3.72494  3.29354  2.67741  2.69355  4.24690  2.90347  2.73739  3.18146  2.89801  2.37887  2.77519  2.98518  4.58477  3.61503
      0.01673  4.49498  5.21732  0.61958  0.77255  0.48576  0.95510
201   3.15693  4.50519  4.90431  4.33623  1.27458  4.38339  4.68502  1.86123  4.18980  1.94694  3.27531  4.46053  4.68090  4.32788  4.28158  3.71368  3.11374  2.03945  5.10599  3.87968    231 f - - -
      2.68618  4.42225  2.77519  2.73123  3.46354  2.40513  3.72494  3.29354  2.67741  2.69355  4.24690  2.90347  2.73739  3.18146  2.89801  2.37887  2.77519  2.98518  4.58477  3.61503
      0.01673  4.49498  5.21732  0.61958  0.77255  0.48576  0.95510
202   3.42563  4.68033  5.31272  4.82289  3.85202  4.88775  5.50070  1.81224  4.70833  2.07201  3.63677  5.00782  5.15097  4.95608  4.87623  4.29565  3.68748  0.67332  5.89479  4.68740    232 V - - -
      2.68618  4.42225  2.77519  2.73123  3.46354  2.40513  3.72494  3.29354  2.67741  2.69355  4.24690  2.90347  2.73739  3.18146  2.89801  2.37887  2.77519  2.98518  4.58477  3.61503
      0.01673  4.49498  5.21732  0.61958  0.77255  0.48576  0.95510
203   1.03852  4.52151  3.59689  3.16501  4.38340  2.54877  4.25688  3.79704  3.18277  3.45645  4.28005  3.45556  2.76731  3.48725  3.56152  2.53975  2.70996  3.34273  5.71714  4.27050    233 a - - -
      2.68618  4.42225  2.77519  2.73123  3.46354  2.40513  3.72494  3.29354  2.67741  2.69355  4.24690  2.90347  2.73739  3.18146  2.89801  2.37887  2.77519  2.98518  4.58477  3.61503
      0.01673  4.49498  5.21732  0.61958  0.77255  0.48576  0.95510
204   2.79610  4.20401  4.50021  3.90487  2.66520  3.94997  4.27100  2.41849  3.74680  1.83329  1.79208  3.64395  4.30166  3.91904  3.84699  3.25332  2.88174  1.95696  4.82733  3.65227    234 m - - -
      2.68618  4.42225  2.77519  2.73123  3.46354  2.40513  3.72494  3.29354  2.67741  2.69355  4.24690  2.90347  2.73739  3.18146  2.89801  2.37887  2.77519  2.98518  4.58477  3.61503
      0.01673  4.49498  5.21732  0.61958  0.77255  0.48576  0.95510
205   2.44143  4.48761  3.51976  2.96592  3.72821  3.59096  3.95659  2.87089  2.92548  2.78572  3.34273  3.37468  1.94956  3.24211  3.30153  2.70880  2.05895  2.66060  5.15000  3.90384    235 p - - -
      2.68618  4.42225  2.77519  2.73123  3.46354  2.40513  3.72494  3.29354  2.67741  2.69355  4.24690  2.90347  2.73739  3.18146  2.89801  2.37887  2.77519  2.98518  4.58477  3.61503
      0.01673  4.49498  5.21732  0.61958  0.77255  0.48576  0.95510
206   2.42457  5.31261  2.35120  1.79258  4.64017  3.32217  3.75248  4.12514  2.54716  3.61503  4.37591  2.94467  3.92817  2.52125  3.05668  1.87190  3.05598  3.69743  5.76017  4.33703    236 e - - -
      2.68618  4.42225  2.77519  2.73123  3.46354  2.40513  3.72494  3.29354  2.67741  2.69355  4.24690  2.90347  2.73739  3.18146  2.89801  2.37887  2.77519  2.98518  4.58477  3.61503
      0.01673  4.49498  5.21732  0.61958  0.77255  0.48576  0.95510
207   2.59527  4.90205  2.79236  1.80900  4.14180  2.92733  3.74204  3.55717  2.54818  3.16336  3.97820  3.05981  3.92338  2.89454  3.01211  2.74050  2.83111  2.60060  3.07779  4.07429    237 e - - -
      2.68618  4.42225  2.77519  2.73123  3.46354  2.40513  3.72494  3.29354  2.67741  2.69355  4.24690  2.90347  2.73739  3.18146  2.89801  2.37887  2.77519  2.98518  4.58477  3.61503
      0.01673  4.49498  5.21732  0.61958  0.77255  0.48576  0.95510
208   0.39798  4.63801  4.23390  4.10679  4.81136  3.43797  5.03204  4.04599  4.14366  3.89197  4.88183  4.05511  4.23734  4.42680  4.31469  2.99812  3.32018  3.57693  6.16276  5.05849    238 A - - -
      2.68618  4.42225  2.77519  2.73123  3.46354  2.40513  3.72494  3.29354  2.67741  2.69355  4.24690  2.90347  2.73739  3.18146  2.89801  2.37887  2.77519  2.98518  4.58477  3.61503
      0.01673  4.49498  5.21732  0.61958  0.77255  0.48576  0.95510
209   1.14354  4.42905  3.75211  3.23536  3.86840  3.50222  4.17017  2.69085  3.17852  2.91238  3.61984  3.53883  4.05352  3.48579  3.51372  2.31706  2.76102  2.82853  5.31020  4.08383    239 a - - -
      2.68618  4.42225  2.77519  2.73123  3.46354  2.40513  3.72494  3.29354  2.67741  2.69355  4.24690  2.90347  2.73739  3.18146  2.89801  2.37887  2.77519  2.98518  4.58477  3.61503
      0.01673  4.49498  5.21732  0.61958  0.77255  0.48576  0.95510
210   1.68499  4.82336  2.84847  2.59235  4.03818  3.54750  3.45297  3.44682  2.57364  2.81211  3.90184  3.10068  3.93728  2.92565  2.47647  2.75819  2.93269  2.99685  5.33764  3.39106    240 a - - -
```

```
        2.68618  4.42225  2.77519  2.73123  3.46354  2.40513  3.72494  3.29354  2.67741  2.69355  4.24690  2.90347  2.73739  3.18146  2.89801  2.37887  2.77519  2.98518  4.58477  3.61503
        0.01673  4.49498  5.21732  0.61958  0.77255  0.48576  0.95510
  211   2.74977  4.78601  3.17512  2.93183  4.44883  3.41032  4.19413  3.87997  3.07303  3.59036  4.45594  0.92794  4.05508  3.41497  3.45887  2.62861  3.14484  2.82136  5.78372  4.44070    241 n - - -
        2.68618  4.42225  2.77519  2.73123  3.46354  2.40513  3.72494  3.29354  2.67741  2.69355  4.24690  2.90347  2.73739  3.18146  2.89801  2.37887  2.77519  2.98518  4.58477  3.61503
        0.01673  4.49498  5.21732  0.61958  0.77255  0.48576  0.95510
  212   3.61409  5.28345  4.40777  4.40090  5.44890  0.19756  5.43468  5.24582  4.66158  4.79772  5.80869  4.57530  4.68639  4.93749  4.78679  3.80965  4.13953  4.65390  6.33571  5.58617    242 G - - -
        2.68618  4.42225  2.77519  2.73123  3.46354  2.40513  3.72494  3.29354  2.67741  2.69355  4.24690  2.90347  2.73739  3.18146  2.89801  2.37887  2.77519  2.98518  4.58477  3.61503
        0.01673  4.49498  5.21732  0.61958  0.77255  0.48576  0.95510
  213   3.50040  6.00443  0.52303  2.38735  5.35607  3.57193  4.28144  4.96644  3.46042  4.46936  5.41986  3.08539  4.26879  3.48839  4.12075  3.35602  3.83638  4.52253  6.51247  5.02715    243 D - - -
        2.68618  4.42225  2.77519  2.73123  3.46354  2.40513  3.72494  3.29354  2.67741  2.69355  4.24690  2.90347  2.73739  3.18146  2.89801  2.37887  2.77519  2.98518  4.58477  3.61503
        0.01673  4.49498  5.21732  0.61958  0.77255  0.48576  0.95510
  214   2.69592  4.84648  2.98491  2.14290  4.06989  3.53791  3.31594  3.29191  2.55948  2.81535  3.13675  2.91842  3.92728  2.70035  3.01609  2.63209  2.92725  3.17062  5.35794  2.35951    244 e - - -
        2.68618  4.42225  2.77519  2.73123  3.46354  2.40513  3.72494  3.29354  2.67741  2.69355  4.24690  2.90347  2.73739  3.18146  2.89801  2.37887  2.77519  2.98518  4.58477  3.61503
        0.01673  4.49498  5.21732  0.61958  0.77255  0.48576  0.95510
  215   1.89910  4.29695  3.79972  2.87714  3.41988  3.18417  4.00703  2.31341  3.15618  2.04419  3.14355  3.56429  4.10541  3.43059  3.44800  2.82432  2.92809  2.33058  4.89962  3.68655    245 a - - -
        2.68618  4.42225  2.77519  2.73123  3.46354  2.40513  3.72494  3.29354  2.67741  2.69355  4.24690  2.90347  2.73739  3.18146  2.89801  2.37887  2.77519  2.98518  4.58477  3.61503
        0.01673  4.49498  5.21732  0.61958  0.77255  0.48576  0.95510
  216   2.62764  4.23628  4.25561  3.66982  2.87147  3.88489  4.18663  2.57871  3.54550  1.51082  2.91118  3.87372  4.24380  3.75684  3.71763  3.17534  1.92243  2.42777  4.83531  3.36959    246 l - - -
        2.68618  4.42225  2.77519  2.73123  3.46354  2.40513  3.72494  3.29354  2.67741  2.69355  4.24690  2.90347  2.73739  3.18146  2.89801  2.37887  2.77519  2.98518  4.58477  3.61503
        0.01673  4.49498  5.21732  0.61958  0.77255  0.48576  0.95510
  217   2.26875  4.49605  3.58503  3.07653  3.95623  2.92576  4.08974  3.29857  3.03887  3.01416  3.12598  3.42866  1.40257  3.35982  3.40605  2.77348  2.95756  2.77292  5.35687  4.11354    247 p - - -
        2.68618  4.42225  2.77519  2.73123  3.46354  2.40513  3.72494  3.29354  2.67741  2.69355  4.24690  2.90347  2.73739  3.18146  2.89801  2.37887  2.77519  2.98518  4.58477  3.61503
        0.01673  4.49498  5.21732  0.61958  0.77255  0.48576  0.95510
  218   2.01625  4.41746  3.93758  3.59150  4.58661  3.23224  4.57997  3.96126  3.58764  3.68056  4.50543  3.66996  3.98927  3.86567  3.88201  1.82644  0.98272  3.41787  5.94336  4.75312    248 t - - -
        2.68618  4.42225  2.77519  2.73123  3.46354  2.40513  3.72494  3.29354  2.67741  2.69355  4.24690  2.90347  2.73739  3.18146  2.89801  2.37887  2.77519  2.98518  4.58477  3.61503
        0.01673  4.49498  5.21732  0.61958  0.77255  0.48576  0.95510
  219   2.68140  4.32386  4.59286  4.02167  2.72473  4.11928  4.49992  2.23956  3.88950  2.13345  3.36399  4.17770  4.47371  4.09422  4.03298  3.43656  2.71953  1.10370  5.08817  3.89709    249 v - - -
        2.68618  4.42225  2.77519  2.73123  3.46354  2.40513  3.72494  3.29354  2.67741  2.69355  4.24690  2.90347  2.73739  3.18146  2.89801  2.37887  2.77519  2.98518  4.58477  3.61503
        0.01673  4.49498  5.21732  0.61958  0.77255  0.48576  0.95510
  220   2.84322  4.29683  4.31360  3.74528  1.33637  2.95616  4.11255  2.41170  3.61324  2.34196  3.08835  3.91181  4.29728  3.81344  3.77342  3.23159  3.07671  2.50779  4.65306  3.07114    250 f - - -
        2.68618  4.42225  2.77519  2.73123  3.46354  2.40513  3.72494  3.29354  2.67741  2.69355  4.24690  2.90347  2.73739  3.18146  2.89801  2.37887  2.77519  2.98518  4.58477  3.61503
        0.01673  4.49498  5.21732  0.61958  0.77255  0.48576  0.95510
  221   2.84329  4.38830  4.01295  3.46349  2.56463  3.86936  2.58446  2.82332  3.35058  1.34310  3.46203  3.73407  4.24306  3.61626  3.59362  2.76656  3.07753  2.64463  4.67531  3.27363    251 l - - -
        2.68618  4.42225  2.77519  2.73123  3.46354  2.40513  3.72494  3.29354  2.67741  2.69355  4.24690  2.90347  2.73739  3.18146  2.89801  2.37887  2.77519  2.98518  4.58477  3.61503
        0.01673  4.49498  5.21732  0.61958  0.77255  0.48576  0.95510
  222   2.53952  4.47870  3.84319  3.66983  4.79093  3.23209  4.73020  4.23006  3.74190  3.95158  4.79301  3.71848  4.03219  4.03475  3.99465  0.57890  2.73074  3.60082  6.14203  4.93109    252 S - - -
        2.68618  4.42225  2.77519  2.73123  3.46354  2.40513  3.72494  3.29354  2.67741  2.69355  4.24690  2.90347  2.73739  3.18146  2.89801  2.37887  2.77519  2.98518  4.58477  3.61503
        0.01673  4.49498  5.21732  0.61958  0.77255  0.48576  0.95510
  223   2.54257  4.56487  5.17628  4.67894  3.93090  4.68532  5.33121  1.26871  4.58752  2.51126  3.73018  4.82984  5.00830  4.83782  4.75787  4.07596  3.54688  0.97817  5.83242  4.61839    253 v - - -
        2.68618  4.42225  2.77519  2.73123  3.46354  2.40513  3.72494  3.29354  2.67741  2.69355  4.24690  2.90347  2.73739  3.18146  2.89801  2.37887  2.77519  2.98518  4.58477  3.61503
        0.01673  4.49498  5.21732  0.61958  0.77255  0.48576  0.95510
  224   3.17436  5.56313  2.73069  1.96547  4.88922  3.60238  3.96506  4.37885  2.67162  3.85805  4.71373  3.09784  4.14283  1.00172  3.05703  3.10653  3.42790  3.99506  5.96971  4.59581    254 q - - -
        2.68618  4.42225  2.77519  2.73123  3.46354  2.40513  3.72494  3.29354  2.67741  2.69355  4.24690  2.90347  2.73739  3.18146  2.89801  2.37887  2.77519  2.98518  4.58477  3.61503
        0.01673  4.49498  5.21732  0.61958  0.77255  0.48576  0.95510
  225   2.51479  4.45802  3.85907  3.58563  4.70159  3.23278  4.62390  4.10502  3.61068  3.81757  4.64428  3.67189  4.00592  3.90474  3.89686  0.82054  1.82610  3.51763  6.04421  4.84079    255 s - - -
        2.68618  4.42225  2.77519  2.73123  3.46354  2.40513  3.72494  3.29354  2.67741  2.69355  4.24690  2.90347  2.73739  3.18146  2.89801  2.37887  2.77519  2.98518  4.58477  3.61503
        0.01673  4.49498  5.21732  0.61958  0.77255  0.48576  0.95510
  226   3.80564  5.89450  0.32817  2.95132  5.39014  3.82026  4.65849  5.15285  3.88212  4.65495  5.68746  3.52525  4.51516  3.92562  4.45143  3.72439  4.16903  4.73584  6.41264  5.21317    256 D - - -
        2.68618  4.42225  2.77519  2.73123  3.46354  2.40513  3.72494  3.29354  2.67741  2.69355  4.24690  2.90347  2.73739  3.18146  2.89801  2.37887  2.77519  2.98518  4.58477  3.61503
        0.01673  4.49498  5.21732  0.61958  0.77255  0.48576  0.95510
  227   3.67893  5.78035  2.96685  0.38898  5.23627  3.81976  4.52201  4.83724  3.50242  4.36431  5.36081  3.50588  4.46708  3.75565  3.94507  3.62108  4.00343  4.47105  6.27448  5.06823    257 E - - -
        2.68618  4.42225  2.77519  2.73123  3.46354  2.40513  3.72494  3.29354  2.67741  2.69355  4.24690  2.90347  2.73739  3.18146  2.89801  2.37887  2.77519  2.98518  4.58477  3.61503
        0.01673  4.49498  5.21732  0.61958  0.77255  0.48576  0.95510
  228   1.90020  4.44845  3.72429  3.21610  3.88169  3.49818  4.16668  3.14450  3.16393  2.13763  3.83022  3.52686  4.05215  3.47449  3.50368  1.44562  2.97668  2.69994  5.32565  4.09342    258 s - - -
        2.68618  4.42225  2.77519  2.73123  3.46354  2.40513  3.72494  3.29354  2.67741  2.69355  4.24690  2.90347  2.73739  3.18146  2.89801  2.37887  2.77519  2.98518  4.58477  3.61503
        0.01673  4.49498  5.21732  0.61958  0.77255  0.48576  0.95510
  229   3.87087  5.57570  4.43644  3.86558  5.10196  4.17715  4.48854  4.73143  2.76424  4.13163  5.14919  4.15934  4.68077  3.70881  0.34313  3.94815  4.11413  4.43503  5.94720  4.93659    259 R - - -
        2.68618  4.42225  2.77519  2.73123  3.46354  2.40513  3.72494  3.29354  2.67741  2.69355  4.24690  2.90347  2.73739  3.18146  2.89801  2.37887  2.77519  2.98518  4.58477  3.61503
        0.01673  4.49498  5.21732  0.61958  0.77255  0.48576  0.95510
  230   3.63920  5.46289  3.39003  3.39042  3.93494  3.96516  0.45867  4.54156  3.39723  4.00302  5.04319  3.82386  4.56514  3.88370  3.66829  3.68235  3.96812  4.23004  5.37949  3.86173    260 H - - -
        2.68618  4.42225  2.77519  2.73123  3.46354  2.40513  3.72494  3.29354  2.67741  2.69355  4.24690  2.90347  2.73739  3.18146  2.89801  2.37887  2.77519  2.98518  4.58477  3.61503
        0.01673  4.49498  5.21732  0.61958  0.77255  0.48576  0.95510
  231   2.71591  4.54091  4.86829  4.31602  3.48877  4.40626  4.83264  1.40295  4.16849  2.06702  1.51166  4.48813  4.72316  4.36050  4.30216  3.74964  3.42374  2.11727  5.32816  4.18465    261 i - - -
        2.68618  4.42225  2.77519  2.73123  3.46354  2.40513  3.72494  3.29354  2.67741  2.69355  4.24690  2.90347  2.73739  3.18146  2.89801  2.37887  2.77519  2.98518  4.58477  3.61503
        0.01673  4.49498  5.21732  0.61958  0.77255  0.48576  0.95510
  232   1.66211  4.81147  3.17509  2.68220  4.34090  2.53325  3.89064  3.77205  2.70562  3.36759  4.16873  2.97071  3.93218  2.69576  3.15816  2.09700  2.48153  3.37587  5.59082  4.26419    262 a - - -
        2.68618  4.42225  2.77519  2.73123  3.46354  2.40513  3.72494  3.29354  2.67741  2.69355  4.24690  2.90347  2.73739  3.18146  2.89801  2.37887  2.77519  2.98518  4.58477  3.61503
```

```
      0.01673  4.49498  5.21732  0.61958  0.77255  0.48576  0.95510
233   2.94636  5.11062  2.99239  2.66123  4.26957  3.59672  3.90031  3.80674  2.64043  2.65510  4.26470  1.18387  4.07957  2.68110  3.01934  2.96763  3.20288  3.49408  5.55798  4.18385    263 n - - -
      2.68618  4.42225  2.77519  2.73123  3.46354  2.40513  3.72494  3.29354  2.67741  2.69355  4.24690  2.90347  2.73739  3.18146  2.89801  2.37887  2.77519  2.98518  4.58477  3.61503
      0.01673  4.49498  5.21732  0.61958  0.77255  0.48576  0.95510
234   3.61409  5.28345  4.40777  4.40090  5.44890  0.19756  5.43468  5.24582  4.66158  4.79772  5.80869  4.57530  4.68639  4.93749  4.78679  3.80965  4.13953  4.65390  6.33571  5.58617    264 G - - -
      2.68618  4.42225  2.77519  2.73123  3.46354  2.40513  3.72494  3.29354  2.67741  2.69355  4.24690  2.90347  2.73739  3.18146  2.89801  2.37887  2.77519  2.98518  4.58477  3.61503
      0.01673  4.49498  5.21732  0.61958  0.77255  0.48576  0.95510
235   2.84467  4.52919  3.71832  2.78375  2.95997  3.80442  2.58509  2.82798  3.09848  2.43592  3.63290  3.53128  4.17780  3.39376  3.42969  3.05894  3.07475  2.81579  4.68133  1.58745    265 y - - -
      2.68618  4.42225  2.77519  2.73123  3.46354  2.40513  3.72494  3.29354  2.67741  2.69355  4.24690  2.90347  2.73739  3.18146  2.89801  2.37887  2.77519  2.98518  4.58477  3.61503
      0.01673  4.49498  5.21732  0.61958  0.77255  0.48576  0.95510
236   2.25234  5.12830  2.93583  2.18552  4.46773  3.02874  3.71960  3.92904  2.48485  3.45229  4.21953  2.98227  3.31974  2.27680  2.97050  1.89498  2.96936  3.52635  5.62032  4.23226    266 s - - -
      2.68618  4.42225  2.77519  2.73123  3.46354  2.40513  3.72494  3.29354  2.67741  2.69355  4.24690  2.90347  2.73739  3.18146  2.89801  2.37887  2.77519  2.98518  4.58477  3.61503
      0.01673  4.49498  5.21732  0.61958  0.77255  0.48576  0.95510
237   2.73649  3.68527  4.26923  3.68316  3.26455  3.85993  4.18177  2.05752  3.55574  2.12783  2.95682  3.86872  4.22771  3.76848  3.71959  2.91440  1.65960  2.22842  4.83487  3.64533    267 t - - -
      2.68618  4.42225  2.77519  2.73123  3.46354  2.40513  3.72494  3.29354  2.67741  2.69355  4.24690  2.90347  2.73739  3.18146  2.89801  2.37887  2.77519  2.98518  4.58477  3.61503
      0.01673  4.49498  5.21732  0.61958  0.77255  0.48576  0.95510
238   3.52549  4.76877  5.44331  4.89747  3.50600  4.94874  5.37911  1.58151  4.77983  0.98535  3.19108  5.07000  5.11634  4.84638  4.84203  4.32822  3.75735  1.60392  5.60362  4.53698    268 l - - -
      2.68618  4.42225  2.77519  2.73123  3.46354  2.40513  3.72494  3.29354  2.67741  2.69355  4.24690  2.90347  2.73739  3.18146  2.89801  2.37887  2.77519  2.98518  4.58477  3.61503
      0.01673  4.49498  5.21732  0.61958  0.77255  0.48576  0.95510
239   2.33056  4.33538  3.76962  3.19409  3.45512  3.73433  4.00059  2.77738  2.41983  2.04851  2.35285  3.54598  4.10916  3.39964  3.19131  2.99559  2.94437  2.09741  4.93311  3.71842    269 l - - -
      2.68618  4.42225  2.77519  2.73123  3.46354  2.40513  3.72494  3.29354  2.67741  2.69355  4.24690  2.90347  2.73739  3.18146  2.89801  2.37887  2.77519  2.98518  4.58477  3.61503
      0.01673  4.49498  5.21732  0.61958  0.77255  0.48576  0.95510
240   2.08267  4.24364  3.95607  3.37994  2.77838  3.75563  4.06185  2.68556  3.29091  2.35433  2.40225  3.66387  4.13633  3.54402  3.53883  2.13238  2.77863  2.29811  4.85987  3.65712    270 a - - -
      2.68618  4.42225  2.77519  2.73123  3.46354  2.40513  3.72494  3.29354  2.67741  2.69355  4.24690  2.90347  2.73739  3.18146  2.89801  2.37887  2.77519  2.98518  4.58477  3.61503
      0.01673  4.49498  5.21732  0.61958  0.77255  0.48576  0.95510
241   1.96127  3.97859  4.65861  4.06828  2.89289  4.05245  4.40379  1.74152  3.90511  2.18778  2.84179  4.15342  4.40012  4.07411  3.98703  3.36592  3.08784  1.66646  4.93658  3.75746    271 v - - -
      2.68618  4.42225  2.77519  2.73123  3.46354  2.40513  3.72494  3.29354  2.67741  2.69355  4.24690  2.90347  2.73739  3.18146  2.89801  2.37887  2.77519  2.98518  4.58477  3.61503
      0.01673  4.49498  5.21732  0.61958  0.77255  0.48576  0.95510
242   2.22389  4.52834  4.84263  4.28097  3.46676  4.39651  4.79810  1.80326  4.15133  1.10027  3.31918  4.46151  4.70441  4.32875  4.28823  3.73049  3.40570  2.01974  5.29650  4.15591    272 l - - -
      2.68618  4.42225  2.77519  2.73123  3.46354  2.40513  3.72494  3.29354  2.67741  2.69355  4.24690  2.90347  2.73739  3.18146  2.89801  2.37887  2.77519  2.98518  4.58477  3.61503
      0.01673  4.49498  5.21732  0.61958  0.77255  0.48576  0.95510
243   2.27852  5.26130  2.59966  1.94794  4.59258  3.47491  3.67320  4.07646  2.25117  3.56081  4.31075  2.25152  3.89912  2.73720  2.97211  2.20503  2.99590  3.64552  5.69900  4.28318    273 e - - -
      2.68620  4.42227  2.77521  2.73125  3.46356  2.40514  3.72496  3.29356  2.67742  2.69357  4.24691  2.90348  2.73741  3.18103  2.89802  2.37888  2.77521  2.98520  4.58479  3.61505
      0.14859  2.45445  2.95323  0.32936  1.27076  0.48576  0.95510
244   2.98102  5.53237  1.30262  1.81599  4.83530  3.45555  3.85994  3.74213  2.75455  3.82421  4.61741  2.37289  3.99286  2.99579  3.30470  2.81316  3.24123  3.90582  5.97512  4.50925    275 d - - -
      2.68618  4.42225  2.77519  2.73123  3.46354  2.40513  3.72494  3.29354  2.67741  2.69355  4.24690  2.90347  2.73739  3.18146  2.89801  2.37887  2.77519  2.98518  4.58477  3.61503
      0.01755  4.44710  5.16945  0.61958  0.77255  0.44071  1.03165
245   2.81659  5.39252  2.52761  1.66762  4.70337  3.48268  3.60676  4.18765  2.65019  3.68704  4.46743  2.62146  1.79849  2.91938  3.16903  2.84119  3.05497  3.76982  5.84344  4.41197    276 e - - -
      2.68618  4.42225  2.77519  2.73123  3.46354  2.40513  3.72494  3.29354  2.67741  2.69355  4.24690  2.90347  2.73739  3.18146  2.89801  2.37887  2.77519  2.98518  4.58477  3.61503
      0.01673  4.49498  5.21732  0.61958  0.77255  0.48576  0.95510
246   2.20777  5.22775  2.00373  2.12413  4.55676  3.06700  3.44138  4.03551  2.45737  3.52683  4.27836  2.94814  3.89341  2.80501  2.41358  2.57184  2.97894  3.61090  5.66980  4.26078    277 d - - -
      2.68618  4.42225  2.77519  2.73123  3.46354  2.40513  3.72494  3.29354  2.67741  2.69355  4.24690  2.90347  2.73739  3.18146  2.89801  2.37887  2.77519  2.98518  4.58477  3.61503
      0.01673  4.49498  5.21732  0.61958  0.77255  0.48576  0.95510
247   2.31412  5.24532  2.70146  2.24318  4.72112  3.46620  3.96672  4.18035  2.84248  3.73455  4.55233  1.14962  4.03346  3.12426  3.33293  2.72972  3.23276  3.76372  5.92030  4.52148    278 n - - -
      2.68618  4.42225  2.77519  2.73123  3.46354  2.40513  3.72494  3.29354  2.67741  2.69355  4.24690  2.90347  2.73739  3.18146  2.89801  2.37887  2.77519  2.98518  4.58477  3.61503
      0.01673  4.49498  5.21732  0.61958  0.77255  0.48576  0.95510
248   2.42949  4.61920  3.31836  2.75514  3.79230  3.60159  2.65755  2.86698  2.51056  2.38957  3.45381  3.22630  3.98592  2.98382  2.50045  2.65420  2.92798  2.52389  5.17034  3.89722    279 l - - -
      2.68618  4.42225  2.77519  2.73123  3.46354  2.40513  3.72494  3.29354  2.67741  2.69355  4.24690  2.90347  2.73739  3.18146  2.89801  2.37887  2.77519  2.98518  4.58477  3.61503
      0.01673  4.49498  5.21732  0.61958  0.77255  0.48576  0.95510
249   2.77252  5.14518  3.05047  2.22263  4.46080  3.54050  3.70696  3.90370  1.97807  2.73816  4.20092  3.02839  2.18432  2.48380  2.70316  2.75695  2.99745  3.52148  5.58134  4.22398    280 k - - -
      2.68618  4.42225  2.77519  2.73123  3.46354  2.40513  3.72494  3.29354  2.67741  2.69355  4.24690  2.90347  2.73739  3.18146  2.89801  2.37887  2.77519  2.98518  4.58477  3.61503
      0.01673  4.49498  5.21732  0.61958  0.77255  0.48576  0.95510
250   2.71937  4.11967  4.50377  3.90350  2.83701  3.86620  3.88076  2.23272  3.72469  1.50654  2.89660  3.96421  3.82651  3.88302  3.79175  3.17056  2.95112  2.36578  3.60201  2.54009    281 l - - -
      2.68618  4.42225  2.77519  2.73123  3.46354  2.40513  3.72494  3.29354  2.67741  2.69355  4.24690  2.90347  2.73739  3.18146  2.89801  2.37887  2.77519  2.98518  4.58477  3.61503
      0.01673  4.49498  5.21732  0.61958  0.77255  0.48576  0.95510
251   3.00205  4.41590  4.59906  4.03386  3.40876  3.27374  4.54787  2.13847  3.89325  1.05965  3.31748  4.21556  4.51453  4.09905  4.04453  3.48909  3.24639  1.87389  5.12273  3.95584    282 l - - -
      2.68618  4.42225  2.77519  2.73123  3.46354  2.40513  3.72494  3.29354  2.67741  2.69355  4.24690  2.90347  2.73739  3.18146  2.89801  2.37887  2.77519  2.98518  4.58477  3.61503
      0.01673  4.49498  5.21732  0.61958  0.77255  0.48576  0.95510
252   3.00500  5.52789  2.62623  1.51983  4.85071  3.50781  3.86433  4.34541  2.67268  3.81928  4.61250  2.18290  4.02544  1.69133  3.15924  2.92866  3.25672  3.91779  5.95041  4.51479    283 e - - -
      2.68618  4.42225  2.77519  2.73123  3.46354  2.40513  3.72494  3.29354  2.67741  2.69355  4.24690  2.90347  2.73739  3.18146  2.89801  2.37887  2.77519  2.98518  4.58477  3.61503
      0.01673  4.49498  5.21732  0.61958  0.77255  0.48576  0.95510
253   2.59715  5.02855  2.87424  2.21352  3.56617  3.30445  3.70679  3.74657  2.47277  3.30657  4.09328  3.00901  3.90116  2.83339  1.88503  2.71014  2.28425  3.38448  5.50748  4.14462    284 r - - -
      2.68618  4.42225  2.77519  2.73123  3.46354  2.40513  3.72494  3.29354  2.67741  2.69355  4.24690  2.90347  2.73739  3.18146  2.89801  2.37887  2.77519  2.98518  4.58477  3.61503
      0.01673  4.49498  5.21732  0.61958  0.77255  0.48576  0.95510
254   2.62430  4.78714  1.61082  2.63700  3.97242  3.55732  3.79551  3.39323  2.64566  2.74924  3.88441  3.13706  3.96197  2.98478  3.08978  2.57701  2.95526  2.93367  3.48581  3.98619    285 d - - -
      2.68618  4.42225  2.77519  2.73123  3.46354  2.40513  3.72494  3.29354  2.67741  2.69355  4.24690  2.90347  2.73739  3.18146  2.89801  2.37887  2.77519  2.98518  4.58477  3.61503
      0.01673  4.49498  5.21732  0.61958  0.77255  0.48576  0.95510
```

```
255   3.69113  4.93962  5.54716  4.96282  2.37353  5.01633  5.27274  1.84712  4.82274  0.74478  2.81889  5.13788  5.10652  4.74528  4.80165  4.38270  3.89733  2.62256  5.37355  4.34473    286 l - - -
      2.68618  4.42225  2.77519  2.73123  3.46354  2.40513  3.72494  3.29354  2.67741  2.69355  4.24690  2.90347  2.73739  3.18146  2.89801  2.37887  2.77519  2.98518  4.58477  3.61503
      0.01673  4.49498  5.21732  0.61958  0.77255  0.48576  0.95510
256   2.86810  5.38581  1.80611  2.19304  4.71797  3.49332  3.77259  4.20480  2.45240  3.67910  4.44403  2.34387  3.95597  2.88774  1.90835  2.80989  3.10871  3.77241  5.81030  4.38791    287 d - - -
      2.68618  4.42225  2.77519  2.73123  3.46354  2.40513  3.72494  3.29354  2.67741  2.69355  4.24690  2.90347  2.73739  3.18146  2.89801  2.37887  2.77519  2.98518  4.58477  3.61503
      0.01673  4.49498  5.21732  0.61958  0.77255  0.48576  0.95510
257   2.74099  5.06220  3.09184  2.53000  4.34184  3.54903  3.21384  3.78030  2.09595  3.33053  4.12426  2.39606  3.93796  2.84480  2.42917  2.76202  2.76716  3.42374  5.51308  2.25427    288 k - - -
      2.68618  4.42225  2.77519  2.73123  3.46354  2.40513  3.72494  3.29354  2.67741  2.69355  4.24690  2.90347  2.73739  3.18146  2.89801  2.37887  2.77519  2.98518  4.58477  3.61503
      0.01673  4.49498  5.21732  0.61958  0.77255  0.48576  0.95510
258   1.41005  4.38820  3.70749  3.14421  3.44162  3.70846  3.38201  2.88693  3.04969  2.59851  3.04609  3.50683  4.09942  3.36437  3.36228  2.94174  2.96120  2.67005  3.11143  3.64513    289 a - - -
      2.68618  4.42225  2.77519  2.73123  3.46354  2.40513  3.72494  3.29354  2.67741  2.69355  4.24690  2.90347  2.73739  3.18146  2.89801  2.37887  2.77519  2.98518  4.58477  3.61503
      0.01673  4.49498  5.21732  0.61958  0.77255  0.48576  0.95510
259   3.18849  4.59918  4.58547  4.10187  1.03223  4.17555  3.93573  3.07208  3.93590  2.67615  3.72096  4.10249  4.55021  4.07470  4.04317  3.11911  3.06986  2.90822  2.38610  2.61532    290 f - - -
      2.68618  4.42225  2.77519  2.73123  3.46354  2.40513  3.72494  3.29354  2.67741  2.69355  4.24690  2.90347  2.73739  3.18146  2.89801  2.37887  2.77519  2.98518  4.58477  3.61503
      0.01673  4.49498  5.21732  0.61958  0.77255  0.48576  0.95510
260   4.55074  5.66303  5.14562  5.03051  3.57310  4.52665  4.87079  4.66840  4.78479  3.93538  5.26067  5.08051  5.09913  5.13272  4.78716  4.75014  4.89137  4.56938  0.21966  3.55049    291 W - - -
      2.68618  4.42225  2.77519  2.73123  3.46354  2.40513  3.72494  3.29354  2.67741  2.69355  4.24690  2.90347  2.73739  3.18146  2.89801  2.37887  2.77519  2.98518  4.58477  3.61503
      0.01673  4.49498  5.21732  0.61958  0.77255  0.48576  0.95510
261   2.73702  5.10845  3.04079  2.48001  4.41443  3.52108  3.10636  3.36114  2.40562  3.38879  4.16430  2.38007  3.91150  2.34374  2.06641  2.72495  2.44739  3.47938  5.55715  4.19253    292 r - - -
      2.68618  4.42225  2.77519  2.73123  3.46354  2.40513  3.72494  3.29354  2.67741  2.69355  4.24690  2.90347  2.73739  3.18146  2.89801  2.37887  2.77519  2.98518  4.58477  3.61503
      0.01673  4.49498  5.21732  0.61958  0.77255  0.48576  0.95510
262   2.70694  4.34197  3.03112  2.50262  4.35331  2.74497  3.73626  3.79628  2.50066  3.17312  4.13719  2.03042  3.90815  1.98171  2.96810  2.49849  2.95305  3.41972  5.54656  4.18452    293 q - - -
      2.68618  4.42225  2.77519  2.73123  3.46354  2.40513  3.72494  3.29354  2.67741  2.69355  4.24690  2.90347  2.73739  3.18146  2.89801  2.37887  2.77519  2.98518  4.58477  3.61503
      0.01673  4.49498  5.21732  0.61958  0.77255  0.48576  0.95510
263   3.11064  4.95619  3.66685  3.13073  2.89112  3.93345  1.39707  3.55879  2.97209  3.09822  4.06649  3.51857  4.30328  2.40776  3.31435  3.18902  3.33335  3.32309  3.34054  2.33395    294 h - - -
      2.68618  4.42225  2.77519  2.73123  3.46354  2.40513  3.72494  3.29354  2.67741  2.69355  4.24690  2.90347  2.73739  3.18146  2.89801  2.37887  2.77519  2.98518  4.58477  3.61503
      0.01673  4.49498  5.21732  0.61958  0.77255  0.48576  0.95510
264   2.18088  3.19115  3.14601  2.58451  4.07870  3.48649  3.75006  3.48555  2.18240  3.10347  3.43980  3.09414  3.93541  2.73481  2.24476  2.58341  2.79903  3.17704  5.36162  4.04257    295 a - - -
      2.68618  4.42225  2.77519  2.73123  3.46354  2.40513  3.72494  3.29354  2.67741  2.69355  4.24690  2.90347  2.73739  3.18146  2.89801  2.37887  2.77519  2.98518  4.58477  3.61503
      0.01673  4.49498  5.21732  0.61958  0.77255  0.48576  0.95510
265   2.59142  4.12441  4.36110  3.76532  2.74242  2.92565  4.14818  1.98683  3.61162  2.21919  3.24089  3.88928  4.19817  3.79823  3.72535  2.96176  2.92970  1.81529  4.74218  2.68036    296 v - - -
      2.68618  4.42225  2.77519  2.73123  3.46354  2.40513  3.72494  3.29354  2.67741  2.69355  4.24690  2.90347  2.73739  3.18146  2.89801  2.37887  2.77519  2.98518  4.58477  3.61503
      0.01673  4.49498  5.21732  0.61958  0.77255  0.48576  0.95510
266   2.87618  4.41980  4.91755  4.33064  2.01519  4.31250  4.65692  2.18308  4.17030  1.60490  2.99208  4.42387  4.61231  4.29395  4.23060  3.63756  3.30826  1.43862  5.09347  3.94688    297 v - - -
      2.68618  4.42225  2.77519  2.73123  3.46354  2.40513  3.72494  3.29354  2.67741  2.69355  4.24690  2.90347  2.73739  3.18146  2.89801  2.37887  2.77519  2.98518  4.58477  3.61503
      0.01673  4.49498  5.21732  0.61958  0.77255  0.48576  0.95510
267   2.56451  5.30069  1.37899  2.31020  4.67304  3.47076  3.87749  4.13654  2.74877  3.67435  4.47289  2.98720  3.99295  3.01845  3.26999  2.69936  2.06060  3.72830  5.85700  4.43982    298 d - - -
      2.68618  4.42225  2.77519  2.73123  3.46354  2.40513  3.72494  3.29354  2.67741  2.69355  4.24690  2.90347  2.73739  3.18146  2.89801  2.37887  2.77519  2.98518  4.58477  3.61503
      0.09578  4.49498  2.52364  0.61958  0.77255  0.48576  0.95510
268   1.74134  4.62973  3.27910  2.73776  3.95674  3.25034  3.85125  3.34728  2.72266  2.68947  3.85150  2.87075  2.63091  3.05706  3.14779  2.61582  2.63913  2.86066  5.30191  4.01683    299 a - - -
      2.68618  4.42225  2.77519  2.73123  3.46354  2.40513  3.72494  3.29354  2.67741  2.69355  4.24690  2.90347  2.73739  3.18146  2.89801  2.37887  2.77519  2.98518  4.58477  3.61503
      0.01809  4.41729  5.13964  0.61958  0.77255  0.41757  1.07483
269   1.70063  4.18538  4.30620  3.71745  2.76237  3.49827  4.18597  2.31848  3.58397  1.88216  3.07314  3.88888  4.23250  3.78737  3.73401  3.16317  2.97404  2.10689  4.81722  3.63131    300 a - - -
      2.68618  4.42225  2.77519  2.73123  3.46354  2.40513  3.72494  3.29354  2.67741  2.69355  4.24690  2.90347  2.73739  3.18146  2.89801  2.37887  2.77519  2.98518  4.58477  3.61503
      0.01673  4.49498  5.21732  0.61958  0.77255  0.48576  0.95510
270   2.91878  4.30163  4.64072  4.05533  3.36247  4.11630  4.46220  1.58388  3.90533  1.65640  3.08454  4.18525  4.45315  3.74589  4.01556  3.42829  2.61907  1.72024  5.00753  3.83681    301 i - - -
      2.68618  4.42225  2.77519  2.73123  3.46354  2.40513  3.72494  3.29354  2.67741  2.69355  4.24690  2.90347  2.73739  3.18146  2.89801  2.37887  2.77519  2.98518  4.58477  3.61503
      0.01673  4.49498  5.21732  0.61958  0.77255  0.48576  0.95510
271   1.73300  4.42598  3.89007  3.73321  4.89498  0.79932  4.79703  4.33902  3.88852  4.03394  4.82971  3.72811  3.99993  4.10620  4.13975  2.47154  3.02729  3.63382  6.22855  5.06817    302 g - - -
      2.68618  4.42225  2.77519  2.73123  3.46354  2.40513  3.72494  3.29354  2.67741  2.69355  4.24690  2.90347  2.73739  3.18146  2.89801  2.37887  2.77519  2.98518  4.58477  3.61503
      0.01673  4.49498  5.21732  0.61958  0.77255  0.48576  0.95510
272   2.49168  4.19494  4.05411  3.47098  3.30140  3.44870  4.07041  2.64757  3.36495  2.14381  2.49233  3.71676  4.14595  3.60130  3.32972  3.05474  1.95416  2.21704  4.20162  3.06259    303 t - - -
      2.68618  4.42225  2.77519  2.73123  3.46354  2.40513  3.72494  3.29354  2.67741  2.69355  4.24690  2.90347  2.73739  3.18146  2.89801  2.37887  2.77519  2.98518  4.58477  3.61503
      0.01673  4.49498  5.21732  0.61958  0.77255  0.48576  0.95510
273   2.59270  4.12716  4.38620  3.78890  2.03025  3.84173  4.15996  2.35642  3.63178  1.98458  2.56200  3.90603  4.20648  3.53759  3.73887  2.80005  2.79365  2.09662  4.74544  3.56308    304 l - - -
      2.68618  4.42225  2.77519  2.73123  3.46354  2.40513  3.72494  3.29354  2.67741  2.69355  4.24690  2.90347  2.73739  3.18146  2.89801  2.37887  2.77519  2.98518  4.58477  3.61503
      0.01673  4.49498  5.21732  0.61958  0.77255  0.48576  0.95510
274   2.47081  4.13864  4.26299  3.67087  2.44574  3.80982  4.11962  1.80940  3.53236  2.25641  2.60639  3.48570  4.17776  3.40011  3.67618  2.81842  2.92222  2.37343  4.75176  3.13591    305 i - - -
      2.68618  4.42225  2.77519  2.73123  3.46354  2.40513  3.72494  3.29354  2.67741  2.69355  4.24690  2.90347  2.73739  3.18146  2.89801  2.37887  2.77519  2.98518  4.58477  3.61503
      0.01673  4.49498  5.21732  0.61958  0.77255  0.48576  0.95510
275   3.33001  6.01059  1.45459  0.93967  5.28996  3.49373  4.09638  4.85601  3.18951  4.31361  5.18751  2.94450  4.15196  3.26989  3.84208  3.17673  3.63200  4.39370  6.44625  4.88666    306 e - - -
      2.68618  4.42225  2.77519  2.73123  3.46354  2.40513  3.72494  3.29354  2.67741  2.69355  4.24690  2.90347  2.73739  3.18146  2.89801  2.37887  2.77519  2.98518  4.58477  3.61503
      0.01673  4.49498  5.21732  0.61958  0.77255  0.48576  0.95510
276   4.25897  5.49049  4.81617  4.69440  2.59558  4.60122  4.08969  4.20446  4.54335  3.48777  4.81457  4.56975  5.07518  4.68358  4.58107  4.31602  4.55642  4.10727  4.25009  0.34504    307 Y - - -
      2.68618  4.42225  2.77519  2.73123  3.46354  2.40513  3.72494  3.29354  2.67741  2.69355  4.24690  2.90347  2.73739  3.18146  2.89801  2.37887  2.77519  2.98518  4.58477  3.61503
      0.01673  4.49498  5.21732  0.61958  0.77255  0.48576  0.95510
277   2.82217  4.37563  3.99546  3.44210  2.43263  1.62631  4.00906  2.82473  3.35354  2.50536  2.76439  3.71564  4.22590  3.60373  3.60571  3.12928  3.05914  2.63683  4.67302  2.64451    308 g - - -
```

```
      2.68618  4.42225  2.77519  2.73123  3.46354  2.40513  3.72494  3.29354  2.67741  2.69355  4.24690  2.90347  2.73739  3.18146  2.89801  2.37887  2.77519  2.98518  4.58477  3.61503
      0.01673  4.49498  5.21732  0.61958  0.77255  0.48576  0.95510
278   2.41974  4.67308  3.10015  2.71474  3.88859  3.56631  3.82372  3.26988  2.69876  2.54492  3.79026  3.19358  3.96956  2.86633  3.12615  2.18063  1.89717  2.72585  5.24530  3.95952    309 t - - -
      2.68618  4.42225  2.77519  2.73123  3.46354  2.40513  3.72494  3.29354  2.67741  2.69355  4.24690  2.90347  2.73739  3.18146  2.89801  2.37887  2.77519  2.98518  4.58477  3.61503
      0.01673  4.49498  5.21732  0.61958  0.77255  0.48576  0.95510
279   2.72244  5.10065  3.04395  2.40717  4.40540  3.52168  3.40920  3.84955  1.67788  3.38070  4.15776  3.01375  2.82009  2.81452  2.62610  2.72577  2.44536  3.08576  5.55224  4.18936    310 k - - -
      2.68618  4.42225  2.77519  2.73123  3.46354  2.40513  3.72494  3.29354  2.67741  2.69355  4.24690  2.90347  2.73739  3.18146  2.89801  2.37887  2.77519  2.98518  4.58477  3.61503
      0.01673  4.49498  5.21732  0.61958  0.77255  0.48576  0.95510
280   2.59221  5.04137  2.24771  2.41904  3.09593  3.49788  3.35640  3.76632  2.22271  2.96283  4.09967  2.40724  3.89103  2.81748  2.94102  2.60785  2.92991  3.12485  5.51385  4.14455    311 k - - -
      2.68618  4.42225  2.77519  2.73123  3.46354  2.40513  3.72494  3.29354  2.67741  2.69355  4.24690  2.90347  2.73739  3.18146  2.89801  2.37887  2.77519  2.98518  4.58477  3.61503
      0.01673  4.49498  5.21732  0.61958  0.77255  0.48576  0.95510
281   3.37072  5.53998  3.74258  2.60638  5.06791  3.95413  3.83157  4.37824  1.60470  3.75301  4.61331  3.44285  4.30187  2.95418  0.97689  3.33727  3.51196  4.04469  5.74745  4.60449    312 r - - -
      2.68640  4.42247  2.77432  2.73141  3.46376  2.40535  3.72516  3.29376  2.67669  2.69377  4.24712  2.90342  2.73754  3.18168  2.89730  2.37893  2.77541  2.98540  4.58499  3.61525
      0.39531  1.13598  5.21732  0.98706  0.46628  0.48576  0.95510
282   2.61442  4.89386  3.13609  2.57363  4.13972  3.28801  3.74397  3.54921  2.09191  3.04426  3.97328  3.08551  3.22971  2.89755  2.19831  2.75617  2.68303  2.31058  5.39903  4.07512    316 k - - -
      2.68618  4.42225  2.77519  2.73123  3.46354  2.40513  3.72494  3.29354  2.67741  2.69355  4.24690  2.90347  2.73739  3.18146  2.89801  2.37887  2.77519  2.98518  4.58477  3.61503
      0.01673  4.49498  5.21732  0.61958  0.77255  0.48576  0.95510
283   2.70843  4.95647  3.02888  1.58557  4.20783  3.52274  3.26080  3.63460  2.35605  3.22006  2.87333  3.04087  3.63611  2.86201  2.96015  2.72862  2.93913  3.29741  4.02772  4.09924    317 e - - -
      2.68618  4.42225  2.77519  2.73123  3.46354  2.40513  3.72494  3.29354  2.67741  2.69355  4.24690  2.90347  2.73739  3.18146  2.89801  2.37887  2.77519  2.98518  4.58477  3.61503
      0.01673  4.49498  5.21732  0.61958  0.77255  0.48576  0.95510
284   2.08371  4.48736  3.72150  3.40381  4.70558  3.23429  4.50435  4.14425  3.46375  3.80302  4.60436  3.57658  2.22496  3.74879  3.80128  0.92453  3.00249  3.54526  6.01627  4.79700    318 s - - -
      2.68618  4.42225  2.77519  2.73123  3.46354  2.40513  3.72494  3.29354  2.67741  2.69355  4.24690  2.90347  2.73739  3.18146  2.89801  2.37887  2.77519  2.98518  4.58477  3.61503
      0.01673  4.49498  5.21732  0.61958  0.77255  0.48576  0.95510
285   3.28669  5.12707  5.08636  4.77648  1.85362  4.71806  3.76071  3.68330  4.59379  3.07881  4.27273  4.41578  5.01669  4.49462  4.56242  4.07360  4.14481  3.56278  1.77476  0.94949    319 y - - -
      2.68618  4.42225  2.77519  2.73123  3.46354  2.40513  3.72494  3.29354  2.67741  2.69355  4.24690  2.90347  2.73739  3.18146  2.89801  2.37887  2.77519  2.98518  4.58477  3.61503
      0.01673  4.49498  5.21732  0.61958  0.77255  0.48576  0.95510
286   1.86145  4.73970  3.26374  2.69689  3.94144  3.59339  3.79855  3.32002  1.93892  2.81759  3.82803  3.18228  3.97991  2.99469  2.77204  2.81660  2.95502  2.47632  5.27123  3.67026    320 a - - -
      2.68618  4.42225  2.77519  2.73123  3.46354  2.40513  3.72494  3.29354  2.67741  2.69355  4.24690  2.90347  2.73739  3.18146  2.89801  2.37887  2.77519  2.98518  4.58477  3.61503
      0.01673  4.49498  5.21732  0.61958  0.77255  0.48576  0.95510
287   3.02026  5.57566  2.33486  1.08865  4.89236  3.49742  3.87493  4.39201  2.12668  3.86095  4.65309  2.95195  4.02624  2.81072  3.22501  2.93519  3.27470  3.95686  5.99492  4.54272    321 e - - -
      2.68618  4.42225  2.77519  2.73123  3.46354  2.40513  3.72494  3.29354  2.67741  2.69355  4.24690  2.90347  2.73739  3.18146  2.89801  2.37887  2.77519  2.98518  4.58477  3.61503
      0.01673  4.49498  5.21732  0.61958  0.77255  0.48576  0.95510
288   2.27845  4.18659  4.11361  3.52870  2.75743  3.79277  4.09260  2.62257  3.16215  2.11036  2.10350  3.75529  4.16223  3.64310  3.61068  3.07618  2.29668  2.34515  4.79707  3.41659    322 m - - -
      2.68618  4.42225  2.77519  2.73123  3.46354  2.40513  3.72494  3.29354  2.67741  2.69355  4.24690  2.90347  2.73739  3.18146  2.89801  2.37887  2.77519  2.98518  4.58477  3.61503
      0.01673  4.49498  5.21732  0.61958  0.77255  0.48576  0.95510
289   3.02484  4.56295  4.26425  3.76936  2.95366  3.96449  4.06534  2.57067  3.50175  2.63656  3.69424  3.92748  4.40048  3.84092  3.68834  2.99986  3.29931  2.81885  1.08050  3.03170    323 w - - -
      2.68618  4.42225  2.77519  2.73123  3.46354  2.40513  3.72494  3.29354  2.67741  2.69355  4.24690  2.90347  2.73739  3.18146  2.89801  2.37887  2.77519  2.98518  4.58477  3.61503
      0.01673  4.49498  5.21732  0.61958  0.77255  0.48576  0.95510
290   2.73294  5.22814  2.80796  1.90170  4.56902  3.48380  2.70465  4.04898  2.33946  3.52780  4.27274  2.36205  3.88715  2.59500  2.34847  2.69460  2.84975  3.61713  5.65828  4.25355    324 e - - -
      2.68618  4.42225  2.77519  2.73123  3.46354  2.40513  3.72494  3.29354  2.67741  2.69355  4.24690  2.90347  2.73739  3.18146  2.89801  2.37887  2.77519  2.98518  4.58477  3.61503
      0.01673  4.49498  5.21732  0.61958  0.77255  0.48576  0.95510
291   2.72000  5.14377  2.62761  2.16351  4.46030  3.49617  3.68381  3.92038  2.41478  3.43286  3.76178  2.97750  3.89129  2.60611  1.71065  2.69769  2.81084  3.28878  5.59045  4.20600    325 r - - -
      2.68618  4.42225  2.77519  2.73123  3.46354  2.40513  3.72494  3.29354  2.67741  2.69355  4.24690  2.90347  2.73739  3.18146  2.89801  2.37887  2.77519  2.98518  4.58477  3.61503
      0.01673  4.49498  5.21732  0.61958  0.77255  0.48576  0.95510
292   4.21085  5.36572  5.07564  4.83255  2.25000  4.79787  3.79873  3.96982  4.53735  3.23073  4.52236  4.48211  5.12328  4.55652  4.53368  4.24398  4.43306  3.88753  0.70842  1.75672    326 w - - -
      2.68618  4.42225  2.77519  2.73123  3.46354  2.40513  3.72494  3.29354  2.67741  2.69355  4.24690  2.90347  2.73739  3.18146  2.89801  2.37887  2.77519  2.98518  4.58477  3.61503
      0.01673  4.49498  5.21732  0.61958  0.77255  0.48576  0.95510
293   2.71680  4.51659  5.13113  4.59913  3.32536  4.62551  5.12991  1.18478  4.49017  2.37677  3.60413  4.73634  4.91948  4.69898  4.62329  3.98829  3.48266  1.14547  5.60192  4.40408    327 v - - -
      2.68618  4.42225  2.77519  2.73123  3.46354  2.40513  3.72494  3.29354  2.67741  2.69355  4.24690  2.90347  2.73739  3.18146  2.89801  2.37887  2.77519  2.98518  4.58477  3.61503
      0.01673  4.49498  5.21732  0.61958  0.77255  0.48576  0.95510
294   2.68733  4.20612  4.14708  2.74801  2.77602  3.81664  4.07529  2.53895  3.44920  2.28909  3.31509  3.77801  4.18469  3.67047  3.63790  3.10160  2.95589  2.06610  3.73297  1.89708    328 y - - -
      2.68618  4.42225  2.77519  2.73123  3.46354  2.40513  3.72494  3.29354  2.67741  2.69355  4.24690  2.90347  2.73739  3.18146  2.89801  2.37887  2.77519  2.98518  4.58477  3.61503
      0.01673  4.49498  5.21732  0.61958  0.77255  0.48576  0.95510
295   3.05415  5.65576  1.59306  1.31986  4.96119  3.11998  3.90666  4.47920  2.47777  3.94344  4.73853  2.92825  4.03370  2.80680  3.37636  2.95540  3.31775  4.03081  6.08218  4.59668    329 e - - -
      2.68618  4.42225  2.77519  2.73123  3.46354  2.40513  3.72494  3.29354  2.67741  2.69355  4.24690  2.90347  2.73739  3.18146  2.89801  2.37887  2.77519  2.98518  4.58477  3.61503
      0.01673  4.49498  5.21732  0.61958  0.77255  0.48576  0.95510
296   3.27239  5.92049  0.83715  2.34132  5.19838  3.48245  4.06019  4.76485  3.11680  4.22770  5.08799  2.30585  4.12799  2.67846  3.74131  3.13283  3.57021  4.30890  6.36803  4.81758    330 d - - -
      2.68618  4.42225  2.77519  2.73123  3.46354  2.40513  3.72494  3.29354  2.67741  2.69355  4.24690  2.90347  2.73739  3.18146  2.89801  2.37887  2.77519  2.98518  4.58477  3.61503
      0.09578  4.49498  2.52364  0.61958  0.77255  0.48576  0.95510
297   4.36561  5.36483  5.29306  5.16121  1.52090  4.93410  3.65118  4.00252  4.94708  3.21871  4.53894  4.50194  5.19515  4.65483  4.78657  4.32497  4.56230  3.92941  1.67555  0.96996    331 y - - -
      2.68618  4.42225  2.77519  2.73123  3.46354  2.40513  3.72494  3.29354  2.67741  2.69355  4.24690  2.90347  2.73739  3.18146  2.89801  2.37887  2.77519  2.98518  4.58477  3.61503
      0.01809  4.41729  5.13964  0.61958  0.77255  0.41757  1.07483
298   2.29059  4.35326  3.75350  3.20164  3.49399  2.25855  4.02718  2.71276  3.13699  2.59678  3.50828  3.54290  4.09802  3.42517  3.44579  2.96229  2.95109  2.32158  4.97187  1.99602    332 y - - -
      2.68618  4.42225  2.77519  2.73123  3.46354  2.40513  3.72494  3.29354  2.67741  2.69355  4.24690  2.90347  2.73739  3.18146  2.89801  2.37887  2.77519  2.98518  4.58477  3.61503
      0.01673  4.49498  5.21732  0.61958  0.77255  0.48576  0.95510
299   2.90588  5.20144  2.56127  2.58852  4.65430  1.57754  3.89947  4.11526  2.63193  3.64358  4.44906  3.10899  4.03310  3.04145  1.72090  2.89915  3.17956  3.70952  5.78864  4.43741    333 g - - -
      2.68618  4.42225  2.77519  2.73123  3.46354  2.40513  3.72494  3.29354  2.67741  2.69355  4.24690  2.90347  2.73739  3.18146  2.89801  2.37887  2.77519  2.98518  4.58477  3.61503
```

```
      0.01673  4.49498  5.21732  0.61958  0.77255  0.48576  0.95510
300   2.32519  4.60367  3.35148  2.80719  3.86019  3.28179  3.88288  2.44294  2.78448  2.91581  3.77692  3.25833  3.98549  2.78665  3.19453  1.85167  2.31064  2.96093  5.23613  3.96524   334 s - - -
      2.68618  4.42225  2.77519  2.73123  3.46354  2.40513  3.72494  3.29354  2.67741  2.69355  4.24690  2.90347  2.73739  3.18146  2.89801  2.37887  2.77519  2.98518  4.58477  3.61503
      0.01673  4.49498  5.21732  0.61958  0.77255  0.48576  0.95510
301   4.10694  5.22413  5.25070  5.01463  1.70003  4.83973  3.75065  3.77612  4.81179  2.28721  4.34619  4.50220  5.11884  4.61367  4.70496  4.20999  4.31450  3.68448  2.13301  0.91932   335 y - - -
      2.68618  4.42225  2.77519  2.73123  3.46354  2.40513  3.72494  3.29354  2.67741  2.69355  4.24690  2.90347  2.73739  3.18146  2.89801  2.37887  2.77519  2.98518  4.58477  3.61503
      0.01673  4.49498  5.21732  0.61958  0.77255  0.48576  0.95510
302   2.71674  4.12542  4.46405  3.86293  2.66893  3.86340  4.18321  1.92746  3.69279  1.78389  2.35751  3.95117  4.22505  3.86147  3.20119  2.99156  2.94847  2.28341  4.73931  3.27852   336 l - - -
      2.68618  4.42225  2.77519  2.73123  3.46354  2.40513  3.72494  3.29354  2.67741  2.69355  4.24690  2.90347  2.73739  3.18146  2.89801  2.37887  2.77519  2.98518  4.58477  3.61503
      0.06543  4.49498  2.95323  0.61958  0.77255  0.48576  0.95510
303   2.57192  4.82230  2.99025  2.40794  4.04478  2.64646  3.73399  3.10473  2.28297  2.21555  3.89832  3.00791  3.91505  2.89828  2.87642  2.56281  2.91312  2.96115  5.33652  4.01547   337 l - - -
      2.68618  4.42225  2.77519  2.73123  3.46354  2.40513  3.72494  3.29354  2.67741  2.69355  4.24690  2.90347  2.73739  3.18146  2.89801  2.37887  2.77519  2.98518  4.58477  3.61503
      0.01755  4.44710  5.16945  0.61958  0.77255  0.56231  0.84372
304   2.81246  5.22171  2.50007  2.47165  4.56341  3.52090  3.72430  4.01670  2.20973  3.51538  4.29251  3.00607  2.10097  2.84055  1.90466  2.78277  3.04300  3.61745  5.65547  4.28720   338 r - - -
      2.68618  4.42225  2.77519  2.73123  3.46354  2.40513  3.72494  3.29354  2.67741  2.69355  4.24690  2.90347  2.73739  3.18146  2.89801  2.37887  2.77519  2.98518  4.58477  3.61503
      0.01755  4.44710  5.16945  0.61958  0.77255  0.56231  0.84372
305   3.79935  5.04613  5.50399  4.93961  2.66108  5.09913  5.25549  2.46450  4.79598  0.55350  2.69877  5.16390  5.15279  4.72780  4.80331  4.47688  4.00144  2.72711  5.32260  4.21810   339 L - - -
      2.68618  4.42225  2.77519  2.73123  3.46354  2.40513  3.72494  3.29354  2.67741  2.69355  4.24690  2.90347  2.73739  3.18146  2.89801  2.37887  2.77519  2.98518  4.58477  3.61503
      0.01755  4.44710  5.16945  0.61958  0.77255  0.56231  0.84372
306   2.25491  5.06792  2.84202  1.40863  4.55536  2.42267  3.85335  4.01120  2.68153  3.56065  4.35015  3.02660  3.93950  2.99245  3.16950  2.54770  2.88559  3.58966  5.74552  4.36443   340 e - - -
      2.68618  4.42225  2.77519  2.73123  3.46354  2.40513  3.72494  3.29354  2.67741  2.69355  4.24690  2.90347  2.73739  3.18146  2.89801  2.37887  2.77519  2.98518  4.58477  3.61503
      0.01755  4.44710  5.16945  0.61958  0.77255  0.44071  1.03165
307   2.81833  5.26466  2.68896  2.39955  4.61514  3.54017  3.71726  4.07400  1.40178  3.55243  4.31800  3.01385  2.82754  2.82581  2.42658  2.78217  3.04397  3.66128  5.67586  4.30381   341 k - - -
      2.68618  4.42225  2.77519  2.73123  3.46354  2.40513  3.72494  3.29354  2.67741  2.69355  4.24690  2.90347  2.73739  3.18146  2.89801  2.37887  2.77519  2.98518  4.58477  3.61503
      0.01673  4.49498  5.21732  0.61958  0.77255  0.48576  0.95510
308   4.41840  5.44741  5.27690  5.13595  1.57678  4.97201  3.73948  4.01566  4.93878  3.21455  4.56571  4.55851  5.24882  4.70067  4.81866  4.38062  4.62620  3.96872  3.82731  0.57328   342 Y - - -
      2.68618  4.42225  2.77519  2.73123  3.46354  2.40513  3.72494  3.29354  2.67741  2.69355  4.24690  2.90347  2.73739  3.18146  2.89801  2.37887  2.77519  2.98518  4.58477  3.61503
      0.01673  4.49498  5.21732  0.61958  0.77255  0.48576  0.95510
309   3.61409  5.28345  4.40777  4.40090  5.44890  0.19756  5.43468  5.24582  4.66158  4.79772  5.80869  4.57530  4.68639  4.93749  4.78679  3.80965  4.13953  4.65390  6.33571  5.58617   343 G - - -
      2.68618  4.42225  2.77519  2.73123  3.46354  2.40513  3.72494  3.29354  2.67741  2.69355  4.24690  2.90347  2.73739  3.18146  2.89801  2.37887  2.77519  2.98518  4.58477  3.61503
      0.01673  4.49498  5.21732  0.61958  0.77255  0.48576  0.95510
310   3.50281  4.73963  5.44779  4.91583  3.58840  4.95613  5.43110  1.38934  4.80479  1.13753  3.35470  5.08304  5.13848  4.90370  4.88360  4.34352  3.74080  1.51380  5.67697  4.58426   344 l - - -
      2.68618  4.42225  2.77519  2.73123  3.46354  2.40513  3.72494  3.29354  2.67741  2.69355  4.24690  2.90347  2.73739  3.18146  2.89801  2.37887  2.77519  2.98518  4.58477  3.61503
      0.01673  4.49498  5.21732  0.61958  0.77255  0.48576  0.95510
311   2.69784  5.16407  3.00434  1.97377  4.49112  3.50470  3.41021  3.95266  1.79418  3.45402  4.21269  2.94428  3.89599  2.75760  2.46066  2.70376  2.49542  3.20407  5.60051  4.08974   345 k - - -
      2.68618  4.42225  2.77519  2.73123  3.46354  2.40513  3.72494  3.29354  2.67741  2.69355  4.24690  2.90347  2.73739  3.18146  2.89801  2.37887  2.77519  2.98518  4.58477  3.61503
      0.01673  4.49498  5.21732  0.61958  0.77255  0.48576  0.95510
312   2.58575  4.30942  3.76260  2.71530  3.43211  3.71905  3.99145  1.94028  3.12384  2.51242  3.18673  3.53880  3.48079  3.40234  3.42491  2.42374  2.92489  2.12039  4.90675  3.18420   346 i - - -
      2.68618  4.42225  2.77519  2.73123  3.46354  2.40513  3.72494  3.29354  2.67741  2.69355  4.24690  2.90347  2.73739  3.18146  2.89801  2.37887  2.77519  2.98518  4.58477  3.61503
      0.01673  4.49498  5.21732  0.61958  0.77255  0.48576  0.95510
313   3.13693  5.11669  3.32293  3.08968  4.23963  3.68624  2.32820  4.17620  3.07771  3.72704  4.64536  3.52467  0.83759  3.53364  3.40003  3.21124  3.47953  3.81138  5.58937  4.18555   347 p - - -
      2.68618  4.42225  2.77519  2.73123  3.46354  2.40513  3.72494  3.29354  2.67741  2.69355  4.24690  2.90347  2.73739  3.18146  2.89801  2.37887  2.77519  2.98518  4.58477  3.61503
      0.01673  4.49498  5.21732  0.61958  0.77255  0.48576  0.95510
314   2.24765  5.02815  3.07311  2.37780  4.30618  3.53049  2.26829  3.73776  2.28287  3.29777  4.09036  3.03598  3.82139  2.83954  2.28045  2.63937  2.95773  3.38327  3.94739  4.14716   348 a - - -
      2.68618  4.42225  2.77519  2.73123  3.46354  2.40513  3.72494  3.29354  2.67741  2.69355  4.24690  2.90347  2.73739  3.18146  2.89801  2.37887  2.77519  2.98518  4.58477  3.61503
      0.01673  4.49498  5.21732  0.61958  0.77255  0.48576  0.95510
315   2.69559  3.76715  2.05605  2.53381  4.14305  3.09473  3.72908  3.56223  2.52753  3.16334  3.97439  2.91951  3.91515  2.87785  2.66845  2.32063  2.92724  3.21884  3.70727  3.44931   349 d - - -
      2.68618  4.42225  2.77519  2.73123  3.46354  2.40513  3.72494  3.29354  2.67741  2.69355  4.24690  2.90347  2.73739  3.18146  2.89801  2.37887  2.77519  2.98518  4.58477  3.61503
      0.01673  4.49498  5.21732  0.61958  0.77255  0.48576  0.95510
316   2.59878  4.25769  2.82529  3.42775  3.08544  3.80065  4.08379  2.59090  3.33510  1.47865  3.09636  3.70627  4.16787  3.58041  3.57299  3.07596  2.96010  2.44409  3.12034  3.64199   350 l - - -
      2.68618  4.42225  2.77519  2.73123  3.46354  2.40513  3.72494  3.29354  2.67741  2.69355  4.24690  2.90347  2.73739  3.18146  2.89801  2.37887  2.77519  2.98518  4.58477  3.61503
      0.01673  4.49498  5.21732  0.61958  0.77255  0.48576  0.95510
317   2.51891  5.03826  2.64715  2.29936  4.31953  3.22884  3.50251  3.34774  2.34525  3.31579  4.09653  2.92983  3.47869  2.81652  2.48415  2.63831  2.92818  2.27817  5.51107  4.14249   351 v - - -
      2.68618  4.42225  2.77519  2.73123  3.46354  2.40513  3.72494  3.29354  2.67741  2.69355  4.24690  2.90347  2.73739  3.18146  2.89801  2.37887  2.77519  2.98518  4.58477  3.61503
      0.01673  4.49498  5.21732  0.61958  0.77255  0.48576  0.95510
318   2.53156  5.23410  1.94290  1.88586  4.56132  3.39950  3.49319  3.82120  2.46197  3.53174  4.28344  2.94654  3.89465  2.27001  2.95890  2.69377  2.98252  3.44683  5.67468  4.26423   352 e - - -
      2.68618  4.42225  2.77519  2.73123  3.46354  2.40513  3.72494  3.29354  2.67741  2.69355  4.24690  2.90347  2.73739  3.18146  2.89801  2.37887  2.77519  2.98518  4.58477  3.61503
      0.01673  4.49498  5.21732  0.61958  0.77255  0.48576  0.95510
319   1.73675  4.57649  3.36215  2.11118  3.23412  3.61667  3.82223  2.79090  2.76253  2.78760  3.67232  3.26470  4.00305  3.05986  3.18254  2.84697  2.86875  2.63091  5.14119  3.87587   353 a - - -
      2.68618  4.42225  2.77519  2.73123  3.46354  2.40513  3.72494  3.29354  2.67741  2.69355  4.24690  2.90347  2.73739  3.18146  2.89801  2.37887  2.77519  2.98518  4.58477  3.61503
      0.01673  4.49498  5.21732  0.61958  0.77255  0.48576  0.95510
320   1.71842  3.52119  3.47456  2.91593  3.73410  3.60488  3.91944  2.52704  2.11063  2.79309  3.67329  3.34124  4.02285  3.19283  3.23693  2.58606  2.94044  2.82443  5.14436  3.89367   354 a - - -
      2.68618  4.42225  2.77519  2.73123  3.46354  2.40513  3.72494  3.29354  2.67741  2.69355  4.24690  2.90347  2.73739  3.18146  2.89801  2.37887  2.77519  2.98518  4.58477  3.61503
      0.01673  4.49498  5.21732  0.61958  0.77255  0.48576  0.95510
321   2.72078  4.96124  3.09866  2.28308  4.07557  3.53639  3.72324  3.63973  2.18286  3.04356  4.02828  2.77733  3.92475  2.81890  2.35155  2.74212  2.82843  3.18342  2.44062  4.10718   355 k - - -
      2.68618  4.42225  2.77519  2.73123  3.46354  2.40513  3.72494  3.29354  2.67741  2.69355  4.24690  2.90347  2.73739  3.18146  2.89801  2.37887  2.77519  2.98518  4.58477  3.61503
      0.01673  4.49498  5.21732  0.61958  0.77255  0.48576  0.95510
```

```
322   2.71786  5.22082  2.11340  2.08064  4.30338  3.08279  3.27294  4.04281  2.21928  3.52300  4.26467  2.36489  3.87622  2.65232  2.89673  2.59666  2.95061  3.60799  5.65608  4.01509    356 e - - -
      2.68618  4.42225  2.77519  2.73123  3.46354  2.40513  3.72494  3.29354  2.67741  2.69355  4.24690  2.90347  2.73739  3.18146  2.89801  2.37887  2.77519  2.98518  4.58477  3.61503
      0.01673  4.49498  5.21732  0.61958  0.77255  0.48576  0.95510
323   2.21219  4.67751  3.25192  2.71465  3.86303  3.59236  3.80799  3.05681  2.36238  2.65959  3.45151  3.19567  3.97725  2.94730  2.06659  2.78853  2.93546  2.47270  5.21896  3.93686    357 r - - -
      2.68618  4.42225  2.77519  2.73123  3.46354  2.40513  3.72494  3.29354  2.67741  2.69355  4.24690  2.90347  2.73739  3.18146  2.89801  2.37887  2.77519  2.98518  4.58477  3.61503
      0.01673  4.49498  5.21732  0.61958  0.77255  0.48576  0.95510
324   2.69434  4.66980  2.77031  2.32411  3.58297  3.58310  3.80538  2.21524  2.68346  2.74814  3.75759  3.09768  3.96928  3.01839  2.82962  2.80203  2.67616  2.96961  5.21303  2.78687    358 i - - -
      2.68618  4.42225  2.77519  2.73123  3.46354  2.40513  3.72494  3.29354  2.67741  2.69355  4.24690  2.90347  2.73739  3.18146  2.89801  2.37887  2.77519  2.98518  4.58477  3.61503
      0.01673  4.49498  5.21732  0.61958  0.77255  0.48576  0.95510
325   2.69439  4.39174  3.36763  3.04178  3.51635  3.68247  3.09263  2.85172  2.98844  2.60109  3.28927  3.43582  4.06152  3.28830  3.33067  2.80283  2.35107  2.37037  2.12394  3.62833    359 w - - -
      2.68618  4.42225  2.77519  2.73123  3.46354  2.40513  3.72494  3.29354  2.67741  2.69355  4.24690  2.90347  2.73739  3.18146  2.89801  2.37887  2.77519  2.98518  4.58477  3.61503
      0.01673  4.49498  5.21732  0.61958  0.77255  0.48576  0.95510
326   2.16571  4.99589  2.88122  2.33030  4.26375  3.16362  3.70315  3.42035  2.15165  2.93428  3.51869  2.55460  3.89673  2.72828  2.95315  2.70317  2.92643  3.18222  5.47774  3.48498    360 k - - -
      2.68618  4.42225  2.77519  2.73123  3.46354  2.40513  3.72494  3.29354  2.67741  2.69355  4.24690  2.90347  2.73739  3.18146  2.89801  2.37887  2.77519  2.98518  4.58477  3.61503
      0.01673  4.49498  5.21732  0.61958  0.77255  0.48576  0.95510
327   2.82255  5.20466  3.00881  2.49319  4.53775  3.13081  2.09769  3.98445  1.70819  3.49157  4.27294  3.01476  3.96067  2.76960  2.81514  2.31415  3.04998  3.59571  5.63727  4.27995    361 k - - -
      2.68637  4.42245  2.77528  2.73143  3.46369  2.40507  3.72428  3.29373  2.67758  2.69372  4.24709  2.90338  2.73759  3.18166  2.89820  2.37906  2.77539  2.98418  4.58447  3.61298
      0.32820  1.29331  5.21732  1.02298  0.44554  0.48576  0.95510
328   3.94060  5.56047  3.99214  3.85233  3.94950  4.15562  0.32649  4.75510  3.69252  4.14335  5.26938  4.21957  4.75772  4.23947  3.89783  4.02004  4.28299  4.47690  5.38709  3.89157    365 H - - -
      2.68618  4.42225  2.77519  2.73123  3.46354  2.40513  3.72494  3.29354  2.67741  2.69355  4.24690  2.90347  2.73739  3.18146  2.89801  2.37887  2.77519  2.98518  4.58477  3.61503
      0.01673  4.49498  5.21732  0.61958  0.77255  0.48576  0.95510
329   2.43451  5.19505  2.38286  2.04470  4.53480  3.47253  3.60351  4.01705  2.05870  3.50151  4.24252  2.94283  3.87039  2.45525  2.86718  2.43113  2.60073  3.58631  5.62848  4.11990    366 e - - -
      2.68618  4.42225  2.77519  2.73123  3.46354  2.40513  3.72494  3.29354  2.67741  2.69355  4.24690  2.90347  2.73739  3.18146  2.89801  2.37887  2.77519  2.98518  4.58477  3.61503
      0.01673  4.49498  5.21732  0.61958  0.77255  0.48576  0.95510
330   1.98745  4.32427  4.59722  4.02352  3.48069  4.14648  4.51615  1.93773  3.89664  1.94531  3.17325  4.18934  4.49078  4.10466  4.04562  3.45976  2.89708  1.41766  5.11178  3.92388    367 v - - -
      2.68618  4.42225  2.77519  2.73123  3.46354  2.40513  3.72494  3.29354  2.67741  2.69355  4.24690  2.90347  2.73739  3.18146  2.89801  2.37887  2.77519  2.98518  4.58477  3.61503
      0.01673  4.49498  5.21732  0.61958  0.77255  0.48576  0.95510
331   1.47169  4.36816  3.76157  3.19781  2.79270  3.33550  3.30643  2.85524  3.12949  2.57014  3.48155  3.54517  4.12199  3.41173  3.43562  3.00465  2.97060  2.64295  3.31761  3.40703    368 a - - -
      2.68618  4.42225  2.77519  2.73123  3.46354  2.40513  3.72494  3.29354  2.67741  2.69355  4.24690  2.90347  2.73739  3.18146  2.89801  2.37887  2.77519  2.98518  4.58477  3.61503
      0.01673  4.49498  5.21732  0.61958  0.77255  0.48576  0.95510
332   2.48277  4.51130  3.43756  2.87225  3.66422  3.63667  3.87485  2.66916  2.83029  2.17569  2.82873  3.10806  4.01860  2.33067  2.69621  2.73597  2.92681  2.66787  5.07939  3.82742    369 l - - -
      2.68618  4.42225  2.77519  2.73123  3.46354  2.40513  3.72494  3.29354  2.67741  2.69355  4.24690  2.90347  2.73739  3.18146  2.89801  2.37887  2.77519  2.98518  4.58477  3.61503
      0.01673  4.49498  5.21732  0.61958  0.77255  0.48576  0.95510
333   2.35027  4.17884  4.11476  3.30621  2.07169  3.05030  4.08811  2.50232  3.41606  2.18360  3.29311  3.75305  4.15757  3.64262  3.60949  3.07099  2.63156  2.06740  4.79022  3.31727    370 v - - -
      2.68618  4.42225  2.77519  2.73123  3.46354  2.40513  3.72494  3.29354  2.67741  2.69355  4.24690  2.90347  2.73739  3.18146  2.89801  2.37887  2.77519  2.98518  4.58477  3.61503
      0.01673  4.49498  5.21732  0.61958  0.77255  0.48576  0.95510
334   2.06177  4.23029  4.21443  3.64231  1.75304  3.82576  4.17485  2.59497  3.52314  2.04381  3.33425  3.83988  4.21780  3.74498  3.70621  2.81446  2.98842  2.22238  4.84591  3.62892    371 f - - -
      2.68618  4.42225  2.77519  2.73123  3.46354  2.40513  3.72494  3.29354  2.67741  2.69355  4.24690  2.90347  2.73739  3.18146  2.89801  2.37887  2.77519  2.98518  4.58477  3.61503
      0.01673  4.49498  5.21732  0.61958  0.77255  0.48576  0.95510
335   1.66900  4.29517  3.92668  3.35757  2.97133  3.77426  4.01092  2.76794  3.27216  2.49400  3.41332  3.64899  4.15270  3.52895  3.53154  2.66222  2.89711  2.56413  3.63330  2.24297    372 a - - -
      2.68618  4.42225  2.77519  2.73123  3.46354  2.40513  3.72494  3.29354  2.67741  2.69355  4.24690  2.90347  2.73739  3.18146  2.89801  2.37887  2.77519  2.98518  4.58477  3.61503
      0.01673  4.49498  5.21732  0.61958  0.77255  0.48576  0.95510
336   1.74005  4.54927  3.40356  2.85505  3.76687  3.30907  3.89432  3.13655  2.82935  2.82949  3.70084  2.74704  4.00260  3.15210  3.22671  2.83589  2.28853  2.41738  5.16592  3.29507    373 a - - -
      2.68618  4.42225  2.77519  2.73123  3.46354  2.40513  3.72494  3.29354  2.67741  2.69355  4.24690  2.90347  2.73739  3.18146  2.89801  2.37887  2.77519  2.98518  4.58477  3.61503
      0.01673  4.49498  5.21732  0.61958  0.77255  0.48576  0.95510
337   2.54446  4.24592  3.93254  3.35592  3.35323  2.19484  4.04418  2.55607  3.26546  1.89201  2.96132  3.64754  4.13062  3.52306  3.51702  2.89261  2.92973  2.49746  4.84825  2.65630    374 l - - -
      2.68618  4.42225  2.77519  2.73123  3.46354  2.40513  3.72494  3.29354  2.67741  2.69355  4.24690  2.90347  2.73739  3.18146  2.89801  2.37887  2.77519  2.98518  4.58477  3.61503
      0.01673  4.49498  5.21732  0.61958  0.77255  0.48576  0.95510
338   2.63100  4.59602  3.68900  3.17754  3.71666  2.97761  4.02952  3.27146  2.86263  2.94761  3.85266  3.52197  4.13976  3.36154  2.70400  3.00531  3.10012  3.01359  1.28389  3.82946    375 w - - -
      2.68618  4.42225  2.77519  2.73123  3.46354  2.40513  3.72494  3.29354  2.67741  2.69355  4.24690  2.90347  2.73739  3.18146  2.89801  2.37887  2.77519  2.98518  4.58477  3.61503
      0.01673  4.49498  5.21732  0.61958  0.77255  0.48576  0.95510
339   2.34556  4.20093  3.61188  3.04889  3.58482  3.64688  3.96288  2.92245  2.98855  2.57095  3.21849  3.43670  1.61416  3.29776  3.33380  2.75175  2.93487  2.71068  4.45392  3.79781    376 p - - -
      2.68618  4.42225  2.77519  2.73123  3.46354  2.40513  3.72494  3.29354  2.67741  2.69355  4.24690  2.90347  2.73739  3.18146  2.89801  2.37887  2.77519  2.98518  4.58477  3.61503
      0.01673  4.49498  5.21732  0.61958  0.77255  0.48576  0.95510
340   2.15437  4.15480  4.34442  3.75149  2.88496  3.85376  4.17120  2.52955  3.60623  1.90147  3.13298  3.89611  4.21827  3.79948  3.73502  3.15037  2.40744  1.68506  4.38195  3.59429    377 v - - -
      2.68618  4.42225  2.77519  2.73123  3.46354  2.40513  3.72494  3.29354  2.67741  2.69355  4.24690  2.90347  2.73739  3.18146  2.89801  2.37887  2.77519  2.98518  4.58477  3.61503
      0.01673  4.49498  5.21732  0.61958  0.77255  0.48576  0.95510
341   2.38418  4.82069  3.14290  2.59006  3.67526  2.76997  3.21169  3.25072  2.58085  3.07566  3.90386  1.90135  3.93474  2.85315  3.03313  2.75407  2.93030  2.84618  5.34223  4.02391    378 n - - -
      2.68618  4.42225  2.77519  2.73123  3.46354  2.40513  3.72494  3.29354  2.67741  2.69355  4.24690  2.90347  2.73739  3.18146  2.89801  2.37887  2.77519  2.98518  4.58477  3.61503
      0.01673  4.49498  5.21732  0.61958  0.77255  0.48576  0.95510
342   2.51327  4.24785  3.98538  3.41115  1.81656  3.47508  4.03514  2.71046  3.31773  2.44473  3.36271  3.37442  3.36416  3.56400  3.55502  2.90665  2.94845  2.50986  3.65373  2.55406    379 f - - -
      2.68618  4.42225  2.77519  2.73123  3.46354  2.40513  3.72494  3.29354  2.67741  2.69355  4.24690  2.90347  2.73739  3.18146  2.89801  2.37887  2.77519  2.98518  4.58477  3.61503
      0.01673  4.49498  5.21732  0.61958  0.77255  0.48576  0.95510
343   2.80412  4.56554  4.42110  3.90624  1.86894  4.13456  3.39427  3.05451  3.77422  2.69619  3.69415  3.99563  4.48742  3.93843  3.93084  3.10036  3.36062  2.87373  1.41726  2.48599    380 w - - -
      2.68618  4.42225  2.77519  2.73123  3.46354  2.40513  3.72494  3.29354  2.67741  2.69355  4.24690  2.90347  2.73739  3.18146  2.89801  2.37887  2.77519  2.98518  4.58477  3.61503
      0.01673  4.49498  5.21732  0.61958  0.77255  0.48576  0.95510
344   3.21796  5.38992  3.51762  2.92921  4.71126  3.84303  2.79003  4.20426  2.17860  3.64632  4.50022  2.82007  4.22808  2.97690  0.96913  3.20491  3.39963  3.86800  5.65028  4.40516    381 r - - -
```

```
      2.68618  4.42225  2.77519  2.73123  3.46354  2.40513  3.72494  3.29354  2.67741  2.69355  4.24690  2.90347  2.73739  3.18146  2.89801  2.37887  2.77519  2.98518  4.58477  3.61503
      0.01673  4.49498  5.21732  0.61958  0.77255  0.48576  0.95510
345   2.69083  4.14176  4.25365  3.66204  3.06609  3.80900  4.11817  1.74234  3.52535  1.99157  3.25626  3.82883  3.42564  3.72899  3.67260  2.71033  2.37190  2.37562  4.01117  3.29152    382 i - - -
      2.68618  4.42225  2.77519  2.73123  3.46354  2.40513  3.72494  3.29354  2.67741  2.69355  4.24690  2.90347  2.73739  3.18146  2.89801  2.37887  2.77519  2.98518  4.58477  3.61503
      0.01673  4.49498  5.21732  0.61958  0.77255  0.48576  0.95510
346   2.55699  5.16716  1.73098  1.97254  4.46508  3.48850  3.73532  3.92472  2.52211  3.45653  4.23330  2.97496  3.91742  2.85683  3.01542  2.47669  2.92842  3.53539  3.50316  4.24179    383 d - - -
      2.68618  4.42225  2.77519  2.73123  3.46354  2.40513  3.72494  3.29354  2.67741  2.69355  4.24690  2.90347  2.73739  3.18146  2.89801  2.37887  2.77519  2.98518  4.58477  3.61503
      0.01673  4.49498  5.21732  0.61958  0.77255  0.48576  0.95510
347   1.89368  4.35939  3.67081  3.10242  3.49403  3.46790  3.96558  2.84358  3.04489  2.10386  3.22809  3.47789  2.54823  3.33665  3.37224  2.94682  2.80596  2.50795  4.95601  3.27139    384 a - - -
      2.68619  4.42226  2.77521  2.73124  3.46355  2.40501  3.72495  3.29355  2.67742  2.69356  4.24691  2.90348  2.73741  3.18147  2.89802  2.37888  2.77521  2.98519  4.58478  3.61504
      0.06543  2.84880  5.21732  0.39893  1.11180  0.48576  0.95510
348   2.49311  4.44907  3.57429  3.00833  3.57151  3.68816  3.94223  2.76853  2.94815  2.27735  2.23193  3.41872  2.41644  2.65309  3.30133  2.93511  2.94947  2.70422  5.03030  3.79726    386 m - - -
      2.68618  4.42225  2.77519  2.73123  3.46354  2.40513  3.72494  3.29354  2.67741  2.69355  4.24690  2.90347  2.73739  3.18146  2.89801  2.37887  2.77519  2.98518  4.58477  3.61503
      0.01673  4.49498  5.21732  0.61958  0.77255  0.48576  0.95510
349   2.71457  5.08637  2.27493  2.45320  4.40667  3.27726  3.71395  3.69801  2.48205  3.39746  4.17104  2.74729  3.89726  2.83295  2.80347  2.31253  1.87632  3.42896  5.57807  4.19856    387 t - - -
      2.68618  4.42225  2.77519  2.73123  3.46354  2.40513  3.72494  3.29354  2.67741  2.69355  4.24690  2.90347  2.73739  3.18146  2.89801  2.37887  2.77519  2.98518  4.58477  3.61503
      0.01673  4.49498  5.21732  0.61958  0.77255  0.48576  0.95510
350   2.67167  5.51650  1.69722  1.48707  4.83130  3.47607  3.85068  4.33456  2.71860  3.81220  4.59285  2.93347  2.78775  2.76705  3.26013  2.88289  3.21628  3.89592  5.95565  4.49593    388 e - - -
      2.68618  4.42225  2.77519  2.73123  3.46354  2.40513  3.72494  3.29354  2.67741  2.69355  4.24690  2.90347  2.73739  3.18146  2.89801  2.37887  2.77519  2.98518  4.58477  3.61503
      0.01673  4.49498  5.21732  0.61958  0.77255  0.48576  0.95510
351   2.23211  5.18454  2.77520  1.86414  4.51573  3.47980  3.67246  3.99062  2.05600  3.48268  4.23051  2.95290  3.87720  2.74880  2.53513  2.56384  2.65846  3.56804  5.62433  4.14750    389 e - - -
      2.68618  4.42225  2.77519  2.73123  3.46354  2.40513  3.72494  3.29354  2.67741  2.69355  4.24690  2.90347  2.73739  3.18146  2.89801  2.37887  2.77519  2.98518  4.58477  3.61503
      0.01673  4.49498  5.21732  0.61958  0.77255  0.48576  0.95510
352   3.38343  6.08551  0.83806  1.56738  5.35739  3.49541  4.13045  4.93586  3.26644  4.39086  5.28586  2.94578  4.17216  3.31155  3.94526  3.21765  3.69434  4.46953  6.52277  4.94377    390 d - - -
      2.68618  4.42225  2.77519  2.73123  3.46354  2.40513  3.72494  3.29354  2.67741  2.69355  4.24690  2.90347  2.73739  3.18146  2.89801  2.37887  2.77519  2.98518  4.58477  3.61503
      0.01673  4.49498  5.21732  0.61958  0.77255  0.48576  0.95510
353   2.55396  4.33264  3.75136  3.17690  1.81013  3.72252  3.98119  2.80809  2.92377  2.52871  2.58437  3.52974  4.09785  2.93169  2.97999  2.98299  2.93640  2.59774  4.91280  3.42171    391 f - - -
      2.68618  4.42225  2.77519  2.73123  3.46354  2.40513  3.72494  3.29354  2.67741  2.69355  4.24690  2.90347  2.73739  3.18146  2.89801  2.37887  2.77519  2.98518  4.58477  3.61503
      0.01673  4.49498  5.21732  0.61958  0.77255  0.48576  0.95510
354   2.42297  5.20667  2.58267  1.46936  4.52145  3.48411  3.71276  3.99041  2.38243  3.07283  4.26086  2.95978  3.90447  2.66055  2.97069  2.72292  2.83607  3.58149  5.65541  4.25432    392 e - - -
      2.68618  4.42225  2.77519  2.73123  3.46354  2.40513  3.72494  3.29354  2.67741  2.69355  4.24690  2.90347  2.73739  3.18146  2.89801  2.37887  2.77519  2.98518  4.58477  3.61503
      0.01673  4.49498  5.21732  0.61958  0.77255  0.48576  0.95510
355   4.12678  5.31651  5.12356  4.87434  2.07069  4.74874  3.92508  3.74204  4.63530  2.97544  4.31379  4.55820  5.10043  4.62399  4.62042  4.24451  4.37051  3.72155  0.63881  2.32584    393 W - - -
      2.68618  4.42225  2.77519  2.73123  3.46354  2.40513  3.72494  3.29354  2.67741  2.69355  4.24690  2.90347  2.73739  3.18146  2.89801  2.37887  2.77519  2.98518  4.58477  3.61503
      0.01673  4.49498  5.21732  0.61958  0.77255  0.48576  0.95510
356   3.77198  5.01724  5.13058  4.73650  0.86138  4.73062  4.04607  3.09810  4.55533  1.64290  3.66891  4.54357  4.98963  4.50978  4.54995  4.08734  3.98794  3.15042  4.18788  2.30373    394 f - - -
      2.68618  4.42225  2.77519  2.73123  3.46354  2.40513  3.72494  3.29354  2.67741  2.69355  4.24690  2.90347  2.73739  3.18146  2.89801  2.37887  2.77519  2.98518  4.58477  3.61503
      0.01673  4.49498  5.21732  0.61958  0.77255  0.48576  0.95510
357   2.76476  5.35963  2.62471  0.86595  4.86646  3.46936  4.02535  4.32206  2.93059  3.88030  4.71551  3.04471  4.07175  3.18893  3.43026  2.52913  3.32993  3.89157  6.05657  4.63265    395 e - - -
      2.68618  4.42225  2.77519  2.73123  3.46354  2.40513  3.72494  3.29354  2.67741  2.69355  4.24690  2.90347  2.73739  3.18146  2.89801  2.37887  2.77519  2.98518  4.58477  3.61503
      0.01673  4.49498  5.21732  0.61958  0.77255  0.48576  0.95510
358   2.18790  5.17606  2.77656  1.96614  4.50443  3.47701  2.76972  3.97915  2.27139  3.47384  4.22172  2.69790  3.87358  2.72441  2.78800  2.54474  2.93593  3.55782  5.61804  3.54739    396 e - - -
      2.68618  4.42225  2.77519  2.73123  3.46354  2.40513  3.72494  3.29354  2.67741  2.69355  4.24690  2.90347  2.73739  3.18146  2.89801  2.37887  2.77519  2.98518  4.58477  3.61503
      0.01673  4.49498  5.21732  0.61958  0.77255  0.48576  0.95510
359   3.05820  5.39275  3.13978  2.67651  4.74973  3.68798  2.95887  4.20872  1.11561  3.66076  4.47513  2.27133  4.10571  2.92956  2.60117  3.02152  3.26613  3.83056  5.72338  4.41967    397 k - - -
      2.68618  4.42225  2.77519  2.73123  3.46354  2.40513  3.72494  3.29354  2.67741  2.69355  4.24690  2.90347  2.73739  3.18146  2.89801  2.37887  2.77519  2.98518  4.58477  3.61503
      0.01673  4.49498  5.21732  0.61958  0.77255  0.48576  0.95510
360   4.16711  5.42896  4.67682  4.51672  2.33860  4.64830  3.64838  4.13233  4.32478  3.38229  4.68017  4.37479  5.05480  4.46825  4.39195  4.16899  4.42765  4.02522  4.00567  0.43235    398 Y - - -
      2.68618  4.42225  2.77519  2.73123  3.46354  2.40513  3.72494  3.29354  2.67741  2.69355  4.24690  2.90347  2.73739  3.18146  2.89801  2.37887  2.77519  2.98518  4.58477  3.61503
      0.01673  4.49498  5.21732  0.61958  0.77255  0.48576  0.95510
361   3.78309  5.38732  4.45801  4.41099  5.28827  4.04248  5.39306  5.05232  4.53282  4.58140  5.68360  4.63927  0.20379  4.89668  4.66690  3.97842  4.27617  4.61438  6.25128  5.42935    399 P - - -
      2.68618  4.42225  2.77519  2.73123  3.46354  2.40513  3.72494  3.29354  2.67741  2.69355  4.24690  2.90347  2.73739  3.18146  2.89801  2.37887  2.77519  2.98518  4.58477  3.61503
      0.01673  4.49498  5.21732  0.61958  0.77255  0.48576  0.95510
362   2.61713  5.00285  2.36069  2.77592  4.94830  0.80609  4.32050  4.42265  3.37417  4.03720  4.88158  3.24489  4.09403  3.53324  3.86976  2.94304  3.31308  3.88660  6.20870  4.85384    400 g - - -
      2.68620  4.42227  2.77521  2.73125  3.46356  2.40514  3.72496  3.29356  2.67742  2.69357  4.24691  2.90348  2.73741  3.18148  2.89802  2.37875  2.77511  2.98520  4.58479  3.61505
      0.09578  2.45446  5.21732  0.32936  1.27076  0.48576  0.95510
363   4.16364  5.31832  5.18039  4.94359  1.83398  4.80559  3.87279  3.74947  4.71547  2.97387  4.31133  4.55854  5.12983  4.63860  4.67094  4.26606  4.39518  3.72971  0.71249  2.25570    402 w - - -
      2.68618  4.42225  2.77519  2.73123  3.46354  2.40513  3.72494  3.29354  2.67741  2.69355  4.24690  2.90347  2.73739  3.18146  2.89801  2.37887  2.77519  2.98518  4.58477  3.61503
      0.01673  4.49498  5.21732  0.61958  0.77255  0.48576  0.95510
364   2.49838  5.04843  2.46699  2.32381  4.30097  3.51140  3.77693  3.73543  2.60004  3.31925  4.12862  2.71948  3.94660  2.92390  3.08232  2.66559  2.86787  3.39324  5.54381  1.81274    403 y - - -
      2.68618  4.42225  2.77519  2.73123  3.46354  2.40513  3.72494  3.29354  2.67741  2.69355  4.24690  2.90347  2.73739  3.18146  2.89801  2.37887  2.77519  2.98518  4.58477  3.61503
      0.01673  4.49498  5.21732  0.61958  0.77255  0.48576  0.95510
365   2.23274  5.20523  2.24051  2.26356  4.54036  3.25475  3.67457  4.02076  2.15697  3.50645  4.25067  2.64963  3.87610  2.77586  2.67344  2.34855  2.80349  3.59096  5.64373  4.23602    404 k - - -
      2.68618  4.42225  2.77519  2.73123  3.46354  2.40513  3.72494  3.29354  2.67741  2.69355  4.24690  2.90347  2.73739  3.18146  2.89801  2.37887  2.77519  2.98518  4.58477  3.61503
      0.01673  4.49498  5.21732  0.61958  0.77255  0.48576  0.95510
366   2.69912  5.07791  3.00390  2.18491  4.37319  3.49379  2.70858  3.82433  2.01864  3.15570  3.73695  2.73496  3.88565  2.67946  2.81461  2.54811  2.92994  3.07604  5.54070  3.26929    405 k - - -
      2.68618  4.42225  2.77519  2.73123  3.46354  2.40513  3.72494  3.29354  2.67741  2.69355  4.24690  2.90347  2.73739  3.18146  2.89801  2.37887  2.77519  2.98518  4.58477  3.61503
```

	0.01673	4.49498	5.21732	0.61958	0.77255	0.48576	0.95510													
367	4.11661	5.25094	5.20394	4.95339	1.27953	4.84807	3.77967	3.19052	4.76197	2.99702	4.28970	4.50604	5.12661	4.60078	4.69035	4.22121	4.32760	3.63880	3.88613	0.83743	406 y - - -
	2.68618	4.42225	2.77519	2.73123	3.46354	2.40513	3.72494	3.29354	2.67741	2.69355	4.24690	2.90347	2.73739	3.18146	2.89801	2.37887	2.77519	2.98518	4.58477	3.61503	
	0.01673	4.49498	5.21732	0.61958	0.77255	0.48576	0.95510													
368	2.96506	5.04836	3.23019	2.90019	4.64671	0.99791	4.04885	4.09857	2.63961	3.65064	4.50394	3.33660	4.13862	2.94965	2.32198	3.02295	3.28248	3.70418	5.78239	4.51763	407 g - - -
	2.68618	4.42225	2.77519	2.73123	3.46354	2.40513	3.72494	3.29354	2.67741	2.69355	4.24690	2.90347	2.73739	3.18146	2.89801	2.37887	2.77519	2.98518	4.58477	3.61503	
	0.01673	4.49498	5.21732	0.61958	0.77255	0.48576	0.95510													
369	2.53967	5.20502	2.52758	2.29966	4.54207	3.05849	3.67263	4.02176	1.80345	3.50591	4.24989	2.81370	2.86918	2.77344	2.54856	2.67894	2.94640	3.54162	5.64135	4.23559	408 k - - -
	2.68618	4.42225	2.77519	2.73123	3.46354	2.40513	3.72494	3.29354	2.67741	2.69355	4.24690	2.90347	2.73739	3.18146	2.89801	2.37887	2.77519	2.98518	4.58477	3.61503	
	0.01673	4.49498	5.21732	0.61958	0.77255	0.48576	0.95510													
370	2.70456	4.13728	4.33738	3.74356	2.21728	3.83160	4.12821	2.16214	3.59213	2.16642	3.24825	3.87590	4.19766	3.21032	3.14182	3.12772	2.93645	2.20069	2.71627	3.10951	409 i - - -
	2.68618	4.42225	2.77519	2.73123	3.46354	2.40513	3.72494	3.29354	2.67741	2.69355	4.24690	2.90347	2.73739	3.18146	2.89801	2.37887	2.77519	2.98518	4.58477	3.61503	
	0.01673	4.49498	5.21732	0.61958	0.77255	0.48576	0.95510													
371	4.30729	5.36360	5.28642	5.10110	1.80569	4.93526	3.72681	3.95888	4.89134	3.09205	4.50589	4.53202	5.20411	4.66207	4.77381	4.31714	4.50895	3.88363	0.91702	1.54530	410 w - - -
	2.68618	4.42225	2.77519	2.73123	3.46354	2.40513	3.72494	3.29354	2.67741	2.69355	4.24690	2.90347	2.73739	3.18146	2.89801	2.37887	2.77519	2.98518	4.58477	3.61503	
	0.01673	4.49498	5.21732	0.61958	0.77255	0.48576	0.95510													
372	2.82895	5.35417	2.22804	1.46460	4.68705	3.48133	3.74998	4.17738	2.17426	3.65142	4.40788	2.60730	3.93320	2.86095	2.76150	2.77365	2.97955	3.74016	5.78310	4.35682	411 e - - -
	2.68618	4.42225	2.77519	2.73123	3.46354	2.40513	3.72494	3.29354	2.67741	2.69355	4.24690	2.90347	2.73739	3.18146	2.89801	2.37887	2.77519	2.98518	4.58477	3.61503	
	0.01673	4.49498	5.21732	0.61958	0.77255	0.48576	0.95510													
373	2.29539	4.82768	3.10481	2.15140	3.40205	3.54170	3.46365	3.45381	2.57046	3.07775	3.90403	2.31560	3.93067	2.91685	2.89270	2.74912	2.79266	2.85297	3.39579	4.02354	412 e - - -
	2.68618	4.42225	2.77519	2.73123	3.46354	2.40513	3.72494	3.29354	2.67741	2.69355	4.24690	2.90347	2.73739	3.18146	2.89801	2.37887	2.77519	2.98518	4.58477	3.61503	
	0.01673	4.49498	5.21732	0.61958	0.77255	0.48576	0.95510													
374	3.85410	5.06541	5.07306	4.74416	2.00973	4.67706	3.49867	2.80427	4.56100	3.04607	4.21384	4.39955	4.97817	4.47142	4.53041	4.02760	4.06902	3.48614	1.90364	0.92965	413 y - - -
	2.68618	4.42225	2.77519	2.73123	3.46354	2.40513	3.72494	3.29354	2.67741	2.69355	4.24690	2.90347	2.73739	3.18146	2.89801	2.37887	2.77519	2.98518	4.58477	3.61503	
	0.01673	4.49498	5.21732	0.61958	0.77255	0.48576	0.95510													
375	2.21450	5.16969	2.89861	2.35810	4.49679	3.38919	3.68286	3.96265	2.13658	3.46402	4.22058	2.41157	3.89097	2.78932	2.05416	2.68002	2.70311	3.55179	5.61205	4.22256	414 r - - -
	2.68618	4.42225	2.77519	2.73123	3.46354	2.40513	3.72494	3.29354	2.67741	2.69355	4.24690	2.90347	2.73739	3.18146	2.89801	2.37887	2.77519	2.98518	4.58477	3.61503	
	0.01673	4.49498	5.21732	0.61958	0.77255	0.48576	0.95510													
376	2.43259	5.23887	2.91882	2.00413	4.59013	3.52012	3.68851	4.05720	1.86273	3.53145	4.28497	2.99104	3.91598	2.37461	2.15649	2.73403	2.99572	3.63436	5.65318	4.27091	415 k - - -
	2.68618	4.42225	2.77519	2.73123	3.46354	2.40513	3.72494	3.29354	2.67741	2.69355	4.24690	2.90347	2.73739	3.18146	2.89801	2.37887	2.77519	2.98518	4.58477	3.61503	
	0.01673	4.49498	5.21732	0.61958	0.77255	0.48576	0.95510													
377	2.70781	4.47981	3.50865	2.78873	3.62664	3.66162	3.89708	2.70761	2.73848	1.73011	2.71009	2.97946	4.04122	3.00945	2.72875	2.90330	2.93787	2.75869	5.05036	3.80898	416 l - - -
	2.68618	4.42225	2.77519	2.73123	3.46354	2.40513	3.72494	3.29354	2.67741	2.69355	4.24690	2.90347	2.73739	3.18146	2.89801	2.37887	2.77519	2.98518	4.58477	3.61503	
	0.01673	4.49498	5.21732	0.61958	0.77255	0.48576	0.95510													
378	1.92870	4.71143	3.27455	2.74074	4.13202	2.41480	3.88817	3.53855	2.73101	3.17332	4.00048	3.19951	3.94521	2.87600	3.09651	1.96122	2.51766	2.81788	5.44024	4.14046	417 a - - -
	2.68618	4.42225	2.77519	2.73123	3.46354	2.40513	3.72494	3.29354	2.67741	2.69355	4.24690	2.90347	2.73739	3.18146	2.89801	2.37887	2.77519	2.98518	4.58477	3.61503	
	0.15474	4.49498	2.02346	0.61958	0.77255	0.48576	0.95510													
379	2.44384	3.61535	2.99159	2.49732	3.88930	3.52435	3.30221	3.28257	2.37024	2.93154	3.77537	3.10396	3.91148	2.93235	2.73030	2.73817	2.76940	2.58327	5.22458	2.68624	418 k - - -
	2.68618	4.42225	2.77519	2.73123	3.46354	2.40513	3.72494	3.29354	2.67741	2.69355	4.24690	2.90347	2.73739	3.18146	2.89801	2.37887	2.77519	2.98518	4.58477	3.61503	
	0.01918	4.35942	5.08176	0.61958	0.77255	0.68111	0.70533													
380	2.80466	5.23617	2.79058	1.79034	4.56183	3.46768	3.71690	4.02290	2.28122	3.52540	4.30573	2.62526	2.06017	2.83789	2.75048	2.76590	3.04102	3.62149	5.67432	4.28684	419 e - - -
	2.68618	4.42225	2.77519	2.73123	3.46354	2.40513	3.72494	3.29354	2.67741	2.69355	4.24690	2.90347	2.73739	3.18146	2.89801	2.37887	2.77519	2.98518	4.58477	3.61503	
	0.01918	4.35942	5.08176	0.61958	0.77255	0.38052	1.15045													
381	3.02958	5.55219	1.61527	2.37694	4.88258	1.56291	3.91583	4.38369	2.61885	3.87509	4.67828	2.55590	4.03437	3.05819	3.21678	2.94927	3.29876	3.95235	6.03037	4.56799	420 g - - -
	2.68615	4.42232	2.77526	2.73130	3.46361	2.40519	3.72501	3.29361	2.67747	2.69362	4.24696	2.90353	2.73650	3.18153	2.89807	2.37893	2.77526	2.98525	4.58484	3.61510	
	0.57648	1.24833	1.88950	0.14678	1.99130	0.48576	0.95510													
382	2.47532	5.12473	2.92443	2.14447	4.45249	2.97115	3.63106	3.82998	2.11593	3.41895	4.17315	2.74500	3.83981	2.60291	2.32866	2.32262	2.90303	3.50671	5.56493	4.17344	422 k - - -
	2.68618	4.42225	2.77519	2.73123	3.46354	2.40513	3.72494	3.29354	2.67741	2.69355	4.24690	2.90347	2.73739	3.18146	2.89801	2.37887	2.77519	2.98518	4.58477	3.61503	
	0.01961	4.33748	5.05982	0.61958	0.77255	0.64242	0.74659													
383	2.56325	5.09619	2.77554	2.38603	4.40857	3.44615	3.35150	3.82325	2.23191	3.38810	4.14938	2.20932	3.80877	2.65786	2.88336	2.13119	2.59370	3.38700	5.55111	4.16120	423 s - - -
	2.68618	4.42225	2.77519	2.73123	3.46354	2.40513	3.72494	3.29354	2.67741	2.69355	4.24690	2.90347	2.73739	3.18146	2.89801	2.37887	2.77519	2.98518	4.58477	3.61503	
	0.01906	4.36545	5.08779	0.61958	0.77255	0.67369	0.71299													
384	2.61504	4.99906	2.99313	2.43765	4.28284	2.26575	3.42006	3.62729	2.11108	3.18780	4.06072	2.71002	3.57512	2.78533	2.64530	2.57797	2.89965	3.05132	5.47196	4.10896	424 k - - -
	2.68618	4.42225	2.77519	2.73123	3.46354	2.40513	3.72494	3.29354	2.67741	2.69355	4.24690	2.90347	2.73739	3.18146	2.89801	2.37887	2.77519	2.98518	4.58477	3.61503	
	0.01906	4.36545	5.08779	0.61958	0.77255	0.67369	0.71299													
385	2.60901	4.29784	3.65190	3.08080	2.63654	3.65895	3.43756	2.39398	3.01963	2.44781	2.99419	3.44962	2.65151	3.08981	2.97741	2.91351	2.74846	2.46930	4.88851	3.66713	425 i - - -
	2.68618	4.42225	2.77519	2.73123	3.46354	2.40513	3.72494	3.29354	2.67741	2.69355	4.24690	2.90347	2.73739	3.18146	2.89801	2.37887	2.77519	2.98518	4.58477	3.61503	
	0.01906	4.36545	5.08779	0.61958	0.77255	0.55209	0.85742													
386	2.65793	4.35220	3.12280	3.04711	3.48921	3.66324	3.93298	1.98380	2.99790	1.89700	3.46229	3.17902	3.69755	3.29254	3.33344	2.78248	2.64814	2.55501	4.94763	3.72035	426 l - - -
	2.68618	4.42225	2.77519	2.73123	3.46354	2.40513	3.72494	3.29354	2.67741	2.69355	4.24690	2.90347	2.73739	3.18146	2.89801	2.37887	2.77519	2.98518	4.58477	3.61503	
	0.01809	4.41729	5.13964	0.61958	0.77255	0.60550	0.78923													
387	2.08543	3.74628	4.18633	3.59874	2.51008	3.79306	4.10382	2.33600	3.47357	2.16078	3.08101	3.79138	2.58977	3.68711	3.64187	3.08280	2.89379	2.17263	4.76605	3.57638	427 a - - -
	2.68618	4.42225	2.77519	2.73123	3.46354	2.40513	3.72494	3.29354	2.67741	2.69355	4.24690	2.90347	2.73739	3.18146	2.89801	2.37887	2.77519	2.98518	4.58477	3.61503	
	0.03951	4.41729	3.62421	0.61958	0.77255	0.41757	1.07483													
388	2.37441	4.31133	3.46930	3.15911	2.69214	3.70478	3.97381	2.56844	3.09530	2.03084	3.09559	2.77401	4.08091	3.37606	2.97465	2.96253	2.76919	2.57618	4.90489	3.03753	428 l - - -
	2.68618	4.42225	2.77519	2.73123	3.46354	2.40513	3.72494	3.29354	2.67741	2.69355	4.24690	2.90347	2.73739	3.18146	2.89801	2.37887	2.77519	2.98518	4.58477	3.61503	
	0.01706	4.47553	5.19788	0.61958	0.77255	0.46612	0.98734													

389	2.18249	5.15010	2.97522	1.90420	4.47132	3.25188	3.67500	3.93740	2.31744	3.44335	3.83690	2.96540	3.88071	2.27962	2.54324	2.53702	2.92853	3.50279	5.59605	4.20482	429 e - - -
	2.68618	4.42225	2.77519	2.73123	3.46354	2.40513	3.72494	3.29354	2.67741	2.69355	4.24690	2.90347	2.73739	3.18146	2.89801	2.37887	2.77519	2.98518	4.58477	3.61503	
	0.01673	4.49498	5.21732	0.61958	0.77255	0.48576	0.95510														
390	2.58342	4.92112	2.37535	2.01810	3.24680	3.31059	3.47670	3.58662	2.51939	2.92033	3.99001	3.00400	3.91204	2.86144	2.98624	2.72408	2.68651	3.25662	3.27181	4.07727	430 e - - -
	2.68618	4.42225	2.77519	2.73123	3.46354	2.40513	3.72494	3.29354	2.67741	2.69355	4.24690	2.90347	2.73739	3.18146	2.89801	2.37887	2.77519	2.98518	4.58477	3.61503	
	0.01673	4.49498	5.21732	0.61958	0.77255	0.48576	0.95510														
391	2.71645	4.08786	4.45076	3.85206	2.48171	3.86220	4.17676	2.42832	3.68510	1.69312	3.22943	3.63090	4.22546	3.85750	3.77394	3.16418	2.73920	1.77701	4.73570	2.85679	431 l - - -
	2.68618	4.42225	2.77519	2.73123	3.46354	2.40513	3.72494	3.29354	2.67741	2.69355	4.24690	2.90347	2.73739	3.18146	2.89801	2.37887	2.77519	2.98518	4.58477	3.61503	
	0.01673	4.49498	5.21732	0.61958	0.77255	0.48576	0.95510														
392	2.70122	4.96105	2.82578	2.40842	4.21927	2.25003	3.72033	3.04370	2.50802	2.94384	4.03065	2.58627	2.80003	2.85865	2.97923	2.63316	2.93390	3.15309	5.45498	4.10528	432 g - - -
	2.68618	4.42225	2.77519	2.73123	3.46354	2.40513	3.72494	3.29354	2.67741	2.69355	4.24690	2.90347	2.73739	3.18146	2.89801	2.37887	2.77519	2.98518	4.58477	3.61503	
	0.01673	4.49498	5.21732	0.61958	0.77255	0.48576	0.95510														
393	2.33145	5.06573	2.99575	2.04189	4.34950	3.10948	3.47153	3.79371	2.21648	3.34297	4.12512	2.99998	3.90269	2.66314	2.93763	2.65873	2.95088	3.42380	5.53405	2.36890	433 e - - -
	2.68618	4.42225	2.77519	2.73123	3.46354	2.40513	3.72494	3.29354	2.67741	2.69355	4.24690	2.90347	2.73739	3.18146	2.89801	2.37887	2.77519	2.98518	4.58477	3.61503	
	0.01673	4.49498	5.21732	0.61958	0.77255	0.48576	0.95510														
394	2.68550	4.56425	3.36938	2.60660	3.72688	3.61638	3.84951	3.09977	2.77731	2.31497	3.24488	2.59283	4.00010	2.79203	3.17992	2.78952	2.36444	2.17213	5.12485	3.70456	434 v - - -
	2.68618	4.42225	2.77519	2.73123	3.46354	2.40513	3.72494	3.29354	2.67741	2.69355	4.24690	2.90347	2.73739	3.18146	2.89801	2.37887	2.77519	2.98518	4.58477	3.61503	
	0.01673	4.49498	5.21732	0.61958	0.77255	0.48576	0.95510														
395	2.71370	4.55733	3.36264	2.85386	3.68397	2.89609	3.87927	3.10203	2.83087	2.19041	3.67008	2.92841	2.63454	3.15596	3.22091	2.87441	2.95184	2.85598	5.10111	2.21982	435 l - - -
	2.68618	4.42225	2.77519	2.73123	3.46354	2.40513	3.72494	3.29354	2.67741	2.69355	4.24690	2.90347	2.73739	3.18146	2.89801	2.37887	2.77519	2.98518	4.58477	3.61503	
	0.01673	4.49498	5.21732	0.61958	0.77255	0.48576	0.95510														
396	2.69102	5.01624	3.05689	2.69575	4.36376	3.55897	2.63310	3.82494	2.49454	3.41374	4.25663	3.18476	1.32871	3.08712	2.99974	2.92107	3.15385	3.48192	5.60077	4.27650	436 p - - -
	2.68624	4.42231	2.77511	2.73123	3.46360	2.40519	3.72501	3.29329	2.67747	2.69353	4.24696	2.90353	2.73729	3.18147	2.89807	2.37893	2.77526	2.98484	4.58483	3.61509	
	0.35164	1.96583	1.85516	0.66977	0.71709	0.48576	0.95510														
397	2.69955	4.36724	3.69523	3.13048	3.22848	3.71295	2.06778	2.54996	3.03364	2.52167	2.77003	3.48672	4.08658	3.28633	3.34157	2.97327	2.95109	2.63294	4.74559	2.43389	439 h - - -
	2.68618	4.42225	2.77519	2.73123	3.46354	2.40513	3.72494	3.29354	2.67741	2.69355	4.24690	2.90347	2.73739	3.18146	2.89801	2.37887	2.77519	2.98518	4.58477	3.61503	
	0.01973	4.33127	5.05362	0.61958	0.77255	0.36544	1.18380														
398	2.75402	4.27751	4.04180	3.46083	2.69158	3.83124	4.11821	2.01732	3.34693	2.01131	3.33455	3.73854	4.19648	3.60747	2.11239	3.10928	2.57524	2.40996	4.88555	3.68665	440 l - - -
	2.68618	4.42225	2.77519	2.73123	3.46354	2.40513	3.72494	3.29354	2.67741	2.69355	4.24690	2.90347	2.73739	3.18146	2.89801	2.37887	2.77519	2.98518	4.58477	3.61503	
	0.01673	4.49498	5.21732	0.61958	0.77255	0.48576	0.95510														
399	3.01165	0.72667	2.14378	3.47241	4.50346	3.62739	4.63144	3.82819	3.70565	3.65031	4.59719	3.79716	4.30452	3.96475	4.03572	3.18671	3.43557	3.48627	5.84464	4.64429	441 c - - -
	2.68618	4.42225	2.77519	2.73123	3.46354	2.40513	3.72494	3.29354	2.67741	2.69355	4.24690	2.90347	2.73739	3.18146	2.89801	2.37887	2.77519	2.98518	4.58477	3.61503	
	0.01673	4.49498	5.21732	0.61958	0.77255	0.48576	0.95510														
400	2.77820	4.93558	3.20554	2.63107	4.15250	3.59473	3.75497	3.58889	2.46025	3.18426	4.01035	2.80946	3.97866	2.19709	2.14010	2.81756	2.83239	3.27637	2.39974	3.72536	442 r - - -
	2.68618	4.42225	2.77519	2.73123	3.46354	2.40513	3.72494	3.29354	2.67741	2.69355	4.24690	2.90347	2.73739	3.18146	2.89801	2.37887	2.77519	2.98518	4.58477	3.61503	
	0.01673	4.49498	5.21732	0.61958	0.77255	0.48576	0.95510														
401	2.70932	4.35454	3.94192	3.40513	3.62550	3.68397	4.20371	2.71891	3.32399	2.38576	3.59139	3.68601	4.16019	3.60966	3.60758	2.29974	1.84674	1.60453	5.12852	3.91585	443 v - - -
	2.68618	4.42225	2.77519	2.73123	3.46354	2.40513	3.72494	3.29354	2.67741	2.69355	4.24690	2.90347	2.73739	3.18146	2.89801	2.37887	2.77519	2.98518	4.58477	3.61503	
	0.01673	4.49498	5.21732	0.61958	0.77255	0.48576	0.95510														
402	2.48117	0.86902	4.33692	4.06636	4.45206	3.23201	4.79653	3.85408	3.94810	3.62583	4.48482	3.58360	4.02820	4.21122	4.10759	1.86320	3.00430	3.33958	5.87936	4.69505	444 c - - -
	2.68618	4.42225	2.77519	2.73123	3.46354	2.40513	3.72494	3.29354	2.67741	2.69355	4.24690	2.90347	2.73739	3.18146	2.89801	2.37887	2.77519	2.98518	4.58477	3.61503	
	0.01673	4.49498	5.21732	0.61958	0.77255	0.48576	0.95510														
403	2.80556	4.67067	3.46195	2.89741	3.72617	3.70385	3.55775	3.14284	2.73088	2.21302	2.49265	3.34575	4.08251	1.66910	3.08213	2.95163	3.03215	2.93828	5.16338	3.88708	445 q - - -
	2.68618	4.42225	2.77519	2.73123	3.46354	2.40513	3.72494	3.29354	2.67741	2.69355	4.24690	2.90347	2.73739	3.18146	2.89801	2.37887	2.77519	2.98518	4.58477	3.61503	
	0.01673	4.49498	5.21732	0.61958	0.77255	0.48576	0.95510														
404	2.84243	4.25318	4.47467	3.54352	3.36842	4.01329	4.35065	2.14347	3.75012	1.93070	2.76222	4.05038	4.36545	3.95532	3.89048	3.09268	3.07748	1.26753	4.95442	3.76927	446 v - - -
	2.68618	4.42225	2.77519	2.73123	3.46354	2.40513	3.72494	3.29354	2.67741	2.69355	4.24690	2.90347	2.73739	3.18146	2.89801	2.37887	2.77519	2.98518	4.58477	3.61503	
	0.01673	4.49498	5.21732	0.61958	0.77255	0.48576	0.95510														
405	3.78309	5.38732	4.45801	4.41099	5.28827	4.04248	5.39306	5.05232	4.53282	4.58140	5.68360	4.63927	0.20379	4.89668	4.66690	3.97842	4.27617	4.61438	6.25128	5.42935	447 P - - -
	2.68618	4.42225	2.77519	2.73123	3.46354	2.40513	3.72494	3.29354	2.67741	2.69355	4.24690	2.90347	2.73739	3.18146	2.89801	2.37887	2.77519	2.98518	4.58477	3.61503	
	0.01673	4.49498	5.21732	0.61958	0.77255	0.48576	0.95510														
406	2.31604	2.08309	4.45078	3.85169	2.51577	3.49399	4.18207	2.31244	3.68453	2.21076	2.68977	3.94237	4.22037	3.85754	3.77261	3.15733	2.94171	2.25091	4.74704	3.56538	448 c - - -
	2.68618	4.42225	2.77519	2.73123	3.46354	2.40513	3.72494	3.29354	2.67741	2.69355	4.24690	2.90347	2.73739	3.18146	2.89801	2.37887	2.77519	2.98518	4.58477	3.61503	
	0.01673	4.49498	5.21732	0.61958	0.77255	0.48576	0.95510														
407	2.76391	3.27891	4.82980	4.23676	2.84378	4.14351	4.50290	1.76694	4.05713	1.53825	3.16217	4.28064	4.47738	4.19856	4.10120	3.46512	3.15262	1.67817	4.98034	3.81282	449 l - - -
	2.68618	4.42225	2.77519	2.73123	3.46354	2.40513	3.72494	3.29354	2.67741	2.69355	4.24690	2.90347	2.73739	3.18146	2.89801	2.37887	2.77519	2.98518	4.58477	3.61503	
	0.13211	4.49498	2.18396	0.61958	0.77255	0.48576	0.95510														
408	2.69363	4.13332	4.28219	3.69131	2.25746	3.48712	4.13590	1.71165	3.54691	2.25744	2.99352	3.85107	4.18624	3.74990	3.12775	3.11828	2.92637	2.08908	4.75549	3.56653	450 i - - -
	2.68618	4.42225	2.77519	2.73123	3.46354	2.40513	3.72494	3.29354	2.67741	2.69355	4.24690	2.90347	2.73739	3.18146	2.89801	2.37887	2.77519	2.98518	4.58477	3.61503	
	0.17503	4.38162	1.91010	0.61958	0.77255	0.65329	0.73466														
409	2.41220	4.76017	3.18386	2.62324	4.08057	3.47829	3.69541	3.46835	2.43170	2.89931	3.92374	3.09841	3.48274	2.89495	1.75005	2.73417	2.44826	3.15380	5.33368	4.04395	451 r - - -
	2.68618	4.42225	2.77519	2.73123	3.46354	2.40513	3.72494	3.29354	2.67741	2.69355	4.24690	2.90347	2.73739	3.18146	2.89801	2.37887	2.77519	2.98518	4.58477	3.61503	
	0.02189	4.22847	4.95082	0.61958	0.77255	0.82089	0.57989														
410	2.46004	5.12506	2.66278	1.41040	4.44371	3.37725	3.70142	3.89216	2.48676	3.43085	4.22624	2.90323	3.87017	2.79279	2.76261	2.72533	2.81492	3.50903	5.61164	4.22669	452 e - - -
	2.68618	4.42225	2.77519	2.73123	3.46354	2.40513	3.72494	3.29354	2.67741	2.69355	4.24690	2.90347	2.73739	3.18146	2.89801	2.37887	2.77519	2.98518	4.58477	3.61503	
	0.02189	4.22847	4.95082	0.61958	0.77255	0.32187	1.29022														
411	3.07578	5.60438	1.57484	2.23366	4.93160	2.85771	3.95444	4.43679	2.90426	3.93305	4.74857	2.94870	1.56239	3.02500	3.47245	2.98766	3.35332	4.00561	6.09622	4.61978	453 p - - -

```
     2.68618  4.42225  2.77519  2.73123  3.46354  2.40513  3.72494  3.29354  2.67741  2.69355  4.24690  2.90347  2.73739  3.18146  2.89801  2.37887  2.77519  2.98518  4.58477  3.61503
     0.01673  4.49498  5.21732  0.61958  0.77255  0.48576  0.95510
412  2.69246  4.56751  3.15524  2.80094  3.29000  2.96233  3.84657  2.97904  2.77363  2.66529  2.48229  3.26079  3.99743  2.95063  2.52902  2.40238  2.77018  2.61788  5.12741  3.86314    454 s - - -
     2.68618  4.42225  2.77519  2.73123  3.46354  2.40513  3.72494  3.29354  2.67741  2.69355  4.24690  2.90347  2.73739  3.18146  2.89801  2.37887  2.77519  2.98518  4.58477  3.61503
     0.01673  4.49498  5.21732  0.61958  0.77255  0.48576  0.95510
413  2.47449  4.02463  3.05971  3.32194  3.16699  3.74680  4.03006  2.31846  3.21076  2.04826  3.36041  3.62437  3.56272  3.24232  3.49959  3.01618  2.89825  1.84736  4.85221  3.34867    455 v - - -
     2.68618  4.42225  2.77519  2.73123  3.46354  2.40513  3.72494  3.29354  2.67741  2.69355  4.24690  2.90347  2.73739  3.18146  2.89801  2.37887  2.77519  2.98518  4.58477  3.61503
     0.01673  4.49498  5.21732  0.61958  0.77255  0.48576  0.95510
414  2.10218  3.46282  2.70349  2.65800  3.77722  3.60066  3.60725  2.95161  2.73900  2.83394  3.62239  3.23150  3.38589  3.06741  3.15350  2.69876  2.74796  2.21130  5.16079  3.88881    456 a - - -
     2.68618  4.42225  2.77519  2.73123  3.46354  2.40513  3.72494  3.29354  2.67741  2.69355  4.24690  2.90347  2.73739  3.18146  2.89801  2.37887  2.77519  2.98518  4.58477  3.61503
     0.01673  4.49498  5.21732  0.61958  0.77255  0.48576  0.95510
415  2.51315  4.44601  2.27958  2.17558  4.46617  3.01678  3.66997  3.72247  2.10552  3.44061  4.19354  2.95720  3.87281  2.59589  2.86894  2.67127  2.48347  3.29916  5.59395  4.19859    457 k - - -
     2.68618  4.42225  2.77519  2.73123  3.46354  2.40513  3.72494  3.29354  2.67741  2.69355  4.24690  2.90347  2.73739  3.18146  2.89801  2.37887  2.77519  2.98518  4.58477  3.61503
     0.01673  4.49498  5.21732  0.61958  0.77255  0.48576  0.95510
416  2.49180  4.87536  2.93211  2.04047  4.49885  2.68802  3.67295  3.97208  1.90250  3.46936  4.21877  2.74253  3.38597  2.74368  2.90458  2.41534  2.93674  3.51326  5.61556  4.21586    458 k - - -
     2.68618  4.42225  2.77519  2.73123  3.46354  2.40513  3.72494  3.29354  2.67741  2.69355  4.24690  2.90347  2.73739  3.18146  2.89801  2.37887  2.77519  2.98518  4.58477  3.61503
     0.01673  4.49498  5.21732  0.61958  0.77255  0.48576  0.95510
417  2.57488  4.72983  3.07033  2.19210  3.92641  3.56675  3.11640  2.54569  2.63835  2.96921  3.81404  3.14809  3.95470  2.97841  2.92024  2.54167  2.76036  2.22258  5.26396  3.96636    459 e - - -
     2.68618  4.42225  2.77519  2.73123  3.46354  2.40513  3.72494  3.29354  2.67741  2.69355  4.24690  2.90347  2.73739  3.18146  2.89801  2.37887  2.77519  2.98518  4.58477  3.61503
     0.04167  4.49498  3.51819  0.61958  0.77255  0.48576  0.95510
418  2.47734  4.20854  2.10494  2.42699  4.18048  2.97562  3.71294  3.22609  2.50299  3.19443  3.71994  2.88534  3.90043  2.85437  2.97165  2.51138  2.50077  3.11990  5.42524  4.08061    460 d - - -
     2.68618  4.42225  2.77519  2.73123  3.46354  2.40513  3.72494  3.29354  2.67741  2.69355  4.24690  2.90347  2.73739  3.18146  2.89801  2.37887  2.77519  2.98518  4.58477  3.61503
     0.01714  4.47045  5.19279  0.61958  0.77255  0.52617  0.89370
419  2.70282  5.10060  2.46121  2.39851  4.40408  2.40580  3.68452  3.86053  2.44265  3.38966  4.15798  2.91486  3.88111  2.79896  2.36542  2.36163  2.68950  3.46995  5.56332  3.24236    461 s - - -
     2.68618  4.42225  2.77519  2.73123  3.46354  2.40513  3.72494  3.29354  2.67741  2.69355  4.24690  2.90347  2.73739  3.18146  2.89801  2.37887  2.77519  2.98518  4.58477  3.61503
     0.01714  4.47045  5.19279  0.61958  0.77255  0.52617  0.89370
420  2.48727  4.69794  3.23354  2.58513  3.54239  3.57006  3.76836  2.96306  2.43727  2.28844  3.78314  3.16062  3.95605  2.24191  2.55665  2.78690  2.92202  2.74841  5.23463  3.94406    462 q - - -
     2.68618  4.42225  2.77519  2.73123  3.46354  2.40513  3.72494  3.29354  2.67741  2.69355  4.24690  2.90347  2.73739  3.18146  2.89801  2.37887  2.77519  2.98518  4.58477  3.61503
     0.01714  4.47045  5.19279  0.61958  0.77255  0.52617  0.89370
421  2.68431  4.48229  3.44943  2.51003  3.51481  3.40947  3.72350  2.78499  2.84691  2.33601  3.58118  3.32045  4.01448  3.16310  2.45905  2.65507  2.64275  2.51183  3.01042  3.80421    463 l - - -
     2.68618  4.42225  2.77519  2.73123  3.46354  2.40513  3.72494  3.29354  2.67741  2.69355  4.24690  2.90347  2.73739  3.18146  2.89801  2.37887  2.77519  2.98518  4.58477  3.61503
     0.34617  4.47045  1.26881  0.61958  0.77255  0.52617  0.89370
422  2.61085  4.61228  3.15672  2.59299  3.65215  3.48698  3.14763  3.06385  2.51072  2.84606  3.69849  3.07848  3.26807  2.89978  2.23437  2.70696  2.84084  2.76406  5.12815  3.02162    464 r - - -
     2.68626  4.42233  2.77528  2.73131  3.46362  2.40521  3.72503  3.29362  2.67749  2.69363  4.24698  2.90355  2.73730  3.18154  2.89799  2.37895  2.77422  2.98526  4.58485  3.61511
     0.79610  0.72362  2.75006  0.12724  2.12459  0.58045  0.82017
423  2.75235  4.19265  4.27012  3.69593  2.61745  3.86925  4.09295  2.34720  3.56428  2.23711  3.25196  3.86251  4.22755  3.76405  3.72000  3.16723  2.78264  1.75570  4.65154  2.01753    466 v - - -
     2.68618  4.42225  2.77519  2.73123  3.46354  2.40513  3.72494  3.29354  2.67741  2.69355  4.24690  2.90347  2.73739  3.18146  2.89801  2.37887  2.77519  2.98518  4.58477  3.61503
     0.02099  4.26993  4.99228  0.61958  0.77255  0.59269  0.80483
424  2.36024  2.43826  3.70880  3.13962  3.41365  3.52267  2.68832  2.63911  3.07030  2.38443  3.39890  3.48583  4.04151  3.35384  3.37457  2.61375  2.87961  2.33549  4.88464  3.66751    467 v - - -
     2.68618  4.42225  2.77519  2.73123  3.46354  2.40513  3.72494  3.29354  2.67741  2.69355  4.24690  2.90347  2.73739  3.18146  2.89801  2.37887  2.77519  2.98518  4.58477  3.61503
     0.01918  4.35942  5.08176  0.61958  0.77255  0.38052  1.15045
425  2.73463  5.18684  2.34465  1.82034  4.50197  3.48085  3.01729  3.97330  2.46029  3.48023  4.23892  2.95709  3.89288  2.80810  2.95326  2.04409  2.96890  3.12882  5.63530  3.99800    468 e - - -
     2.68618  4.42225  2.77519  2.73123  3.46354  2.40513  3.72494  3.29354  2.67741  2.69355  4.24690  2.90347  2.73739  3.18146  2.89801  2.37887  2.77519  2.98518  4.58477  3.61503
     0.01673  4.49498  5.21732  0.61958  0.77255  0.48576  0.95510
426  2.64699  4.89080  2.96804  1.86381  3.35079  3.41109  3.14274  3.54252  2.34564  3.11208  3.96230  3.06234  3.91984  2.88631  2.99822  2.73418  2.92935  3.22175  4.39357  2.62065    469 e - - -
     2.68618  4.42225  2.77519  2.73123  3.46354  2.40513  3.72494  3.29354  2.67741  2.69355  4.24690  2.90347  2.73739  3.18146  2.89801  2.37887  2.77519  2.98518  4.58477  3.61503
     0.01673  4.49498  5.21732  0.61958  0.77255  0.48576  0.95510
427  2.51150  5.04200  2.87438  2.31837  3.85706  3.00710  3.69208  3.68268  2.37411  2.84641  4.06913  2.29779  3.85813  2.68685  2.80342  2.69248  2.39898  3.22076  5.51382  4.14392    470 n - - -
     2.68618  4.42225  2.77519  2.73123  3.46354  2.40513  3.72494  3.29354  2.67741  2.69355  4.24690  2.90347  2.73739  3.18146  2.89801  2.37887  2.77519  2.98518  4.58477  3.61503
     0.01673  4.49498  5.21732  0.61958  0.77255  0.48576  0.95510
428  2.66776  2.00350  2.55933  3.13655  4.43137  1.22511  4.31656  3.83466  3.29147  3.52144  4.36731  3.47060  4.04464  3.56418  3.67838  2.81609  3.08573  3.40608  5.76758  4.51996    471 g - - -
     2.68618  4.42225  2.77519  2.73123  3.46354  2.40513  3.72494  3.29354  2.67741  2.69355  4.24690  2.90347  2.73739  3.18146  2.89801  2.37887  2.77519  2.98518  4.58477  3.61503
     0.01673  4.49498  5.21732  0.61958  0.77255  0.48576  0.95510
429  2.68358  5.18631  2.84989  2.41509  4.52200  3.50007  3.20339  3.98973  2.01858  3.48002  4.23241  2.82573  3.89178  2.41348  1.94922  2.69809  2.83514  3.57259  5.61737  3.46748    472 r - - -
     2.68618  4.42225  2.77519  2.73123  3.46354  2.40513  3.72494  3.29354  2.67741  2.69355  4.24690  2.90347  2.73739  3.18146  2.89801  2.37887  2.77519  2.98518  4.58477  3.61503
     0.01673  4.49498  5.21732  0.61958  0.77255  0.48576  0.95510
430  2.90713  4.97758  3.42418  2.79846  4.22836  3.70646  3.79576  3.62198  1.60952  3.21047  3.88345  3.25970  4.07665  2.96066  2.23319  2.96168  2.73232  3.32994  2.17897  4.15576    473 k - - -
     2.68618  4.42225  2.77519  2.73123  3.46354  2.40513  3.72494  3.29354  2.67741  2.69355  4.24690  2.90347  2.73739  3.18146  2.89801  2.37887  2.77519  2.98518  4.58477  3.61503
     0.01673  4.49498  5.21732  0.61958  0.77255  0.48576  0.95510
431  2.75555  4.38989  3.76381  3.19878  2.91747  3.75903  2.48040  2.86948  3.13125  2.46268  3.49607  3.54856  4.13418  3.41318  3.44077  3.01810  1.94225  2.49282  4.79468  2.40759    474 t - - -
     2.68618  4.42225  2.77519  2.73123  3.46354  2.40513  3.72494  3.29354  2.67741  2.69355  4.24690  2.90347  2.73739  3.18146  2.89801  2.37887  2.77519  2.98518  4.58477  3.61503
     0.01673  4.49498  5.21732  0.61958  0.77255  0.48576  0.95510
432  2.06001  4.21007  2.42193  2.51639  4.20570  3.51121  2.63962  3.63116  2.51507  2.90345  4.02107  3.03977  3.72130  2.86629  2.98401  2.32799  2.86824  3.29088  5.44675  4.10064    475 a - - -
     2.68618  4.42225  2.77519  2.73123  3.46354  2.40513  3.72494  3.29354  2.67741  2.69355  4.24690  2.90347  2.73739  3.18146  2.89801  2.37887  2.77519  2.98518  4.58477  3.61503
     0.01673  4.49498  5.21732  0.61958  0.77255  0.48576  0.95510
433  2.22443  4.25971  3.90369  3.14294  2.04306  3.75536  4.03945  2.69999  2.77945  2.10880  3.33986  3.63151  4.12898  3.50329  3.49980  3.02593  2.83426  2.17090  4.86535  3.65928    476 f - - -
     2.68618  4.42225  2.77519  2.73123  3.46354  2.40513  3.72494  3.29354  2.67741  2.69355  4.24690  2.90347  2.73739  3.18146  2.89801  2.37887  2.77519  2.98518  4.58477  3.61503
```

	0.01673	4.49498	5.21732	0.61958	0.77255	0.48576	0.95510														
434	2.67785	2.07762	3.56953	2.61187	3.59722	3.63384	3.99586	2.92984	3.04243	2.66938	3.56504	3.44156	4.06089	3.34134	3.38134	2.58419	2.47019	2.06721	5.04759	3.81923	477 v - - -
	2.68618	4.42225	2.77519	2.73123	3.46354	2.40513	3.72494	3.29354	2.67741	2.69355	4.24690	2.90347	2.73739	3.18146	2.89801	2.37887	2.77519	2.98518	4.58477	3.61503	
	0.01673	4.49498	5.21732	0.61958	0.77255	0.48576	0.95510														
435	1.68620	4.88842	2.30894	2.66179	4.54678	3.07793	3.95666	3.99839	2.79698	3.57050	4.35861	3.14043	3.94808	3.10633	3.26918	1.63904	2.83999	3.54653	5.76557	4.41580	478 s - - -
	2.68618	4.42225	2.77519	2.73123	3.46354	2.40513	3.72494	3.29354	2.67741	2.69355	4.24690	2.90347	2.73739	3.18146	2.89801	2.37887	2.77519	2.98518	4.58477	3.61503	
	0.01673	4.49498	5.21732	0.61958	0.77255	0.48576	0.95510														
436	2.61291	5.05865	2.27787	2.01101	2.37099	3.50158	3.72084	3.77328	2.50401	3.16158	4.12129	3.00046	3.71517	2.55444	2.96967	2.72557	2.96217	3.41063	5.53393	4.16405	479 e - - -
	2.68618	4.42225	2.77519	2.73123	3.46354	2.40513	3.72494	3.29354	2.67741	2.69355	4.24690	2.90347	2.73739	3.18146	2.89801	2.37887	2.77519	2.98518	4.58477	3.61503	
	0.01673	4.49498	5.21732	0.61958	0.77255	0.48576	0.95510														
437	2.24523	4.84578	3.13192	2.57321	4.07148	2.74977	3.23206	3.48002	2.55695	3.09869	3.57558	2.93799	3.36706	2.89450	2.39220	2.63811	2.92883	3.17104	3.08820	4.03604	480 a - - -
	2.68618	4.42225	2.77519	2.73123	3.46354	2.40513	3.72494	3.29354	2.67741	2.69355	4.24690	2.90347	2.73739	3.18146	2.89801	2.37887	2.77519	2.98518	4.58477	3.61503	
	0.03745	4.49498	3.66557	0.61958	0.77255	0.48576	0.95510														
438	2.46604	1.98696	2.62525	2.72841	4.18842	2.35910	3.91772	3.60039	2.77483	3.23170	4.05671	3.12248	3.13031	3.10054	3.21597	2.51612	2.95222	3.24300	5.49336	4.19030	481 c - - -
	2.68618	4.42225	2.77519	2.73123	3.46354	2.40513	3.72494	3.29354	2.67741	2.69355	4.24690	2.90347	2.73739	3.18146	2.89801	2.37887	2.77519	2.98518	4.58477	3.61503	
	0.01707	4.47460	5.19695	0.61958	0.77255	0.51951	0.90341														
439	2.76412	5.22672	2.87863	1.47049	4.56498	3.50214	3.69348	4.03267	2.14704	3.51854	4.27613	2.97452	3.90855	2.47932	2.60468	2.72881	2.81414	3.49511	5.65218	4.26361	482 e - - -
	2.68618	4.42225	2.77519	2.73123	3.46354	2.40513	3.72494	3.29354	2.67741	2.69355	4.24690	2.90347	2.73739	3.18146	2.89801	2.37887	2.77519	2.98518	4.58477	3.61503	
	0.01707	4.47460	5.19695	0.61958	0.77255	0.51951	0.90341														
440	2.72211	4.64542	3.34011	2.64554	3.68355	3.61828	2.92982	3.20313	2.56535	2.87006	3.73688	3.23822	4.00060	3.05820	2.40074	2.84594	2.95104	2.81462	3.11113	2.25537	483 y - - -
	2.68618	4.42225	2.77519	2.73123	3.46354	2.40513	3.72494	3.29354	2.67741	2.69355	4.24690	2.90347	2.73739	3.18146	2.89801	2.37887	2.77519	2.98518	4.58477	3.61503	
	0.01707	4.47460	5.19695	0.61958	0.77255	0.51951	0.90341														
441	2.69376	4.77362	2.96894	2.24629	3.98062	3.55016	3.76400	2.23150	2.56931	2.85859	2.85386	2.90601	3.93958	2.41241	3.04335	2.76419	2.92514	3.08607	5.29865	3.99121	484 i - - -
	2.68618	4.42225	2.77519	2.73123	3.46354	2.40513	3.72494	3.29354	2.67741	2.69355	4.24690	2.90347	2.73739	3.18146	2.89801	2.37887	2.77519	2.98518	4.58477	3.61503	
	0.01707	4.47460	5.19695	0.61958	0.77255	0.51951	0.90341														
442	3.30489	4.70246	4.55156	4.07420	1.43945	1.81201	3.84199	3.20781	3.94499	2.79371	3.83584	4.08716	4.60787	4.06024	4.07526	3.57539	3.53318	3.03804	2.49870	2.17217	485 f - - -
	2.68618	4.42225	2.77519	2.73123	3.46354	2.40513	3.72494	3.29354	2.67741	2.69355	4.24690	2.90347	2.73739	3.18146	2.89801	2.37887	2.77519	2.98518	4.58477	3.61503	
	0.01707	4.47460	5.19695	0.61958	0.77255	0.46522	0.98885														
443	2.50713	5.06188	2.64181	2.29499	3.86792	3.49799	3.69202	3.79705	2.31611	2.58658	4.11863	2.92236	3.89098	2.81141	1.98317	2.60382	2.93364	3.42190	5.52900	4.15703	486 r - - -
	2.68618	4.42225	2.77519	2.73123	3.46354	2.40513	3.72494	3.29354	2.67741	2.69355	4.24690	2.90347	2.73739	3.18146	2.89801	2.37887	2.77519	2.98518	4.58477	3.61503	
	0.01673	4.49498	5.21732	0.61958	0.77255	0.48576	0.95510														
444	2.60356	5.14946	2.66803	2.31512	4.47126	3.47879	3.41612	3.78446	2.33399	3.26035	4.13977	2.77252	2.63351	2.26735	2.80286	2.38026	2.85641	3.52755	4.59829	4.19991	487 q - - -
	2.68618	4.42225	2.77519	2.73123	3.46354	2.40513	3.72494	3.29354	2.67741	2.69355	4.24690	2.90347	2.73739	3.18146	2.89801	2.37887	2.77519	2.98518	4.58477	3.61503	
	0.01673	4.49498	5.21732	0.61958	0.77255	0.48576	0.95510														
445	2.53302	5.06926	2.85941	1.85002	4.36084	3.49422	3.69082	3.80979	2.45068	3.35164	4.12625	2.75594	3.88849	2.80896	2.69091	2.69272	2.05262	3.25487	4.63260	4.16136	488 e - - -
	2.68618	4.42225	2.77519	2.73123	3.46354	2.40513	3.72494	3.29354	2.67741	2.69355	4.24690	2.90347	2.73739	3.18146	2.89801	2.37887	2.77519	2.98518	4.58477	3.61503	
	0.01673	4.49498	5.21732	0.61958	0.77255	0.48576	0.95510														
446	3.78309	5.38732	4.45801	4.41099	5.28827	4.04248	5.39306	5.05232	4.53282	4.58140	5.68360	4.63927	0.20379	4.89668	4.66690	3.97842	4.27617	4.61438	6.25128	5.42935	489 P - - -
	2.68618	4.42225	2.77519	2.73123	3.46354	2.40513	3.72494	3.29354	2.67741	2.69355	4.24690	2.90347	2.73739	3.18146	2.89801	2.37887	2.77519	2.98518	4.58477	3.61503	
	0.03944	4.49498	3.59315	0.61958	0.77255	0.48576	0.95510														
447	2.28779	5.13208	2.69283	1.86545	4.44819	3.47450	3.40934	3.57962	2.41512	3.42490	3.78437	2.38513	3.86903	2.77408	2.79121	2.65645	2.71776	3.50714	5.58241	4.18929	490 e - - -
	2.68618	4.42225	2.77519	2.73123	3.46354	2.40513	3.72494	3.29354	2.67741	2.69355	4.24690	2.90347	2.73739	3.18146	2.89801	2.37887	2.77519	2.98518	4.58477	3.61503	
	0.01711	4.47264	5.19499	0.61958	0.77255	0.46838	0.98354														
448	2.66971	4.80256	3.30153	2.71833	4.01723	3.62242	3.46678	3.19590	2.23539	3.01973	2.16456	3.19838	4.00296	2.97862	1.96445	2.72942	2.99462	3.12229	5.30961	4.02998	491 r - - -
	2.68618	4.42225	2.77519	2.73123	3.46354	2.40513	3.72494	3.29354	2.67741	2.69355	4.24690	2.90347	2.73739	3.18146	2.89801	2.37887	2.77519	2.98518	4.58477	3.61503	
	0.01676	4.49305	5.21540	0.61958	0.77255	0.48903	0.94990														
449	2.94725	4.60551	3.81650	3.28541	2.58796	2.00088	3.02715	3.12737	3.22704	2.77693	3.72711	3.61646	4.26324	3.50057	3.54470	3.15266	3.18095	2.75395	4.51540	1.65792	492 y - - -
	2.68618	4.42225	2.77519	2.73123	3.46354	2.40513	3.72494	3.29354	2.67741	2.69355	4.24690	2.90347	2.73739	3.18146	2.89801	2.37887	2.77519	2.98518	4.58477	3.61503	
	0.01676	4.49305	5.21540	0.61958	0.77255	0.48903	0.94990														
450	2.35074	5.06119	3.02014	2.13701	4.35233	3.50307	3.69448	3.79687	2.36308	3.34173	4.12018	2.99924	3.89577	2.69412	2.10387	2.48770	2.56327	2.88136	5.52824	4.15992	493 r - - -
	2.68618	4.42225	2.77519	2.73123	3.46354	2.40513	3.72494	3.29354	2.67741	2.69355	4.24690	2.90347	2.73739	3.18146	2.89801	2.37887	2.77519	2.98518	4.58477	3.61503	
	0.01925	4.49305	4.84331	0.61958	0.77255	0.48903	0.94990														
451	2.66880	3.00379	3.82921	3.25774	2.60195	2.44699	4.01431	2.75718	3.17979	1.98607	3.39928	3.58081	4.10506	2.68765	3.46075	2.85037	2.92523	2.54884	4.88506	3.67164	494 l - - -
	2.68618	4.42225	2.77519	2.73123	3.46354	2.40513	3.72494	3.29354	2.67741	2.69355	4.24690	2.90347	2.73739	3.18146	2.89801	2.37887	2.77519	2.98518	4.58477	3.61503	
	0.20047	4.49060	1.76939	0.61958	0.77255	0.49317	0.94338														
452	2.42684	3.93021	3.39884	2.83546	3.12623	3.57094	3.25147	2.94686	2.79905	2.66456	3.54520	3.26954	3.96078	3.11537	3.02994	2.05605	2.35052	2.59625	5.01628	3.76642	495 s - - -
	2.68625	4.42232	2.77527	2.73123	3.46361	2.40453	3.72502	3.29361	2.67748	2.69362	4.24697	2.90354	2.73731	3.18154	2.89808	2.37894	2.77527	2.98526	4.58484	3.61510	
	0.74776	1.10863	1.62677	0.25321	1.49747	0.73806	0.65017														
453	2.38760	4.40423	3.36048	2.78997	2.76898	3.54020	3.52567	2.92664	2.40653	2.61926	3.50345	3.22146	3.92040	3.04835	2.62685	2.76436	2.83204	2.68983	3.29744	3.66083	499 a - - -
	2.68618	4.42225	2.77519	2.73123	3.46354	2.40513	3.72494	3.29354	2.67741	2.69355	4.24690	2.90347	2.73739	3.18146	2.89801	2.37887	2.77519	2.98518	4.58477	3.61503	
	0.02494	4.09952	4.82186	0.61958	0.77255	0.40600	1.09755														
454	2.65202	5.05785	3.12929	2.54583	4.35769	3.31558	3.44038	3.77916	2.11110	2.75623	3.82322	3.05814	3.93707	2.43364	1.76359	2.77272	2.98973	3.42605	5.49716	4.17113	500 r - - -
	2.68618	4.42225	2.77519	2.73123	3.46354	2.40513	3.72494	3.29354	2.67741	2.69355	4.24690	2.90347	2.73739	3.18146	2.89801	2.37887	2.77519	2.98518	4.58477	3.61503	
	0.02749	4.41271	4.19991	0.61958	0.77255	0.61186	0.78163														
455	2.68934	5.11796	2.91934	1.81188	4.43281	3.45673	3.66167	3.89648	2.41439	3.41151	3.81553	2.40340	3.08786	2.57455	2.89978	2.52264	2.55149	3.49349	5.57296	4.18162	501 e - - -
	2.68618	4.42225	2.77519	2.73123	3.46354	2.40513	3.72494	3.29354	2.67741	2.69355	4.24690	2.90347	2.73739	3.18146	2.89801	2.37887	2.77519	2.98518	4.58477	3.61503	
	0.01834	4.40356	5.12591	0.61958	0.77255	0.62437	0.76701														

456	2.94894	4.32911	4.64673	4.07206	2.14823	4.09235	4.23524	2.34611	3.90629	2.04532	3.12511	4.14109	4.42125	4.03667	3.98076	3.40424	3.17770	2.02024	1.82313	3.28372	502 w - - -
	2.68618	4.42225	2.77519	2.73123	3.46354	2.40513	3.72494	3.29354	2.67741	2.69355	4.24690	2.90347	2.73739	3.18146	2.89801	2.37887	2.77519	2.98518	4.58477	3.61503	
	0.01834	4.40356	5.12591	0.61958	0.77255	0.62437	0.76701														
457	2.70743	4.58717	3.35736	1.89774	2.94690	3.61703	3.00117	3.13256	2.76694	2.67850	3.68180	3.25386	3.99966	3.09338	3.16957	2.84597	2.80782	2.88353	2.91154	3.32652	503 e - - -
	2.68618	4.42225	2.77519	2.73123	3.46354	2.40513	3.72494	3.29354	2.67741	2.69355	4.24690	2.90347	2.73739	3.18146	2.89801	2.37887	2.77519	2.98518	4.58477	3.61503	
	0.01834	4.40356	5.12591	0.61958	0.77255	0.62437	0.76701														
458	2.77367	5.24144	2.56723	1.69740	4.56071	3.44873	3.71770	4.03601	2.50630	3.53906	4.30638	2.92278	3.89614	2.32032	3.00691	2.49373	2.15627	3.62145	5.69378	4.28170	504 e - - -
	2.68618	4.42225	2.77519	2.73123	3.46354	2.40513	3.72494	3.29354	2.67741	2.69355	4.24690	2.90347	2.73739	3.18146	2.89801	2.37887	2.77519	2.98518	4.58477	3.61503	
	0.01834	4.40356	5.12591	0.61958	0.77255	0.62437	0.76701														
459	2.66211	3.62802	3.87375	3.29523	3.33272	3.71801	3.67014	2.26016	3.20195	1.84872	3.30445	3.59625	4.09103	2.84396	2.91815	2.98813	2.76760	2.14828	4.82209	3.61711	505 l - - -
	2.68618	4.42225	2.77519	2.73123	3.46354	2.40513	3.72494	3.29354	2.67741	2.69355	4.24690	2.90347	2.73739	3.18146	2.89801	2.37887	2.77519	2.98518	4.58477	3.61503	
	0.01834	4.40356	5.12591	0.61958	0.77255	0.62437	0.76701														
460	3.51055	5.02565	4.21949	3.77962	2.06392	4.30023	2.23555	3.62218	3.69660	3.07008	4.17580	3.04579	4.64525	3.88356	3.94127	3.60356	3.73324	3.44856	3.98699	1.00351	506 y - - -
	2.68618	4.42225	2.77519	2.73123	3.46354	2.40513	3.72494	3.29354	2.67741	2.69355	4.24690	2.90347	2.73739	3.18146	2.89801	2.37887	2.77519	2.98518	4.58477	3.61503	
	0.01834	4.40356	5.12591	0.61958	0.77255	0.62437	0.76701														
461	2.63425	5.31308	1.85938	2.15946	4.62822	3.44693	2.21488	4.11309	2.54006	3.54815	4.37350	2.91229	3.91122	2.42240	3.04791	2.72024	3.05696	3.69059	5.75221	4.32741	507 d - - -
	2.68618	4.42225	2.77519	2.73123	3.46354	2.40513	3.72494	3.29354	2.67741	2.69355	4.24690	2.90347	2.73739	3.18146	2.89801	2.37887	2.77519	2.98518	4.58477	3.61503	
	0.01834	4.40356	5.12591	0.61958	0.77255	0.62437	0.76701														
462	2.90030	5.05049	2.48301	2.65490	4.86460	0.84219	4.21909	4.39638	3.25836	3.98275	4.83603	2.80789	4.05628	3.43187	3.75410	2.93863	3.31173	3.88448	6.11982	4.74353	508 g - - -
	2.68618	4.42225	2.77519	2.73123	3.46354	2.40513	3.72494	3.29354	2.67741	2.69355	4.24690	2.90347	2.73739	3.18146	2.89801	2.37887	2.77519	2.98518	4.58477	3.61503	
	0.01834	4.40356	5.12591	0.61958	0.77255	0.50276	0.92851														
463	2.69521	4.76636	3.18660	2.62279	3.97406	3.53147	3.44690	3.15957	2.28086	3.00868	3.28346	2.80902	3.94040	2.57806	2.63776	2.76785	2.91412	2.89669	2.51749	3.98528	509 k - - -
	2.68618	4.42225	2.77519	2.73123	3.46354	2.40513	3.72494	3.29354	2.67741	2.69355	4.24690	2.90347	2.73739	3.18146	2.89801	2.37887	2.77519	2.98518	4.58477	3.61503	
	0.01744	4.45352	5.17586	0.61958	0.77255	0.55259	0.85674														
464	2.76914	3.39163	1.77753	1.97167	4.53304	3.19252	3.73324	4.00320	2.52558	3.51749	4.28700	2.69416	3.90718	2.85148	3.02698	2.40735	3.01219	3.59477	5.68114	4.27585	510 d - - -
	2.68618	4.42225	2.77519	2.73123	3.46354	2.40513	3.72494	3.29354	2.67741	2.69355	4.24690	2.90347	2.73739	3.18146	2.89801	2.37887	2.77519	2.98518	4.58477	3.61503	
	0.01744	4.45352	5.17586	0.61958	0.77255	0.55259	0.85674														
465	2.86929	4.27479	4.52077	3.93869	2.48671	3.99779	4.11493	2.53051	3.77488	1.10047	3.21841	3.79288	4.34196	3.93423	3.87205	3.30244	3.09881	2.39039	3.87524	3.33323	511 l - - -
	2.68618	4.42225	2.77519	2.73123	3.46354	2.40513	3.72494	3.29354	2.67741	2.69355	4.24690	2.90347	2.73739	3.18146	2.89801	2.37887	2.77519	2.98518	4.58477	3.61503	
	0.01744	4.45352	5.17586	0.61958	0.77255	0.55259	0.85674														
466	1.61302	4.53018	3.38796	2.71817	3.71318	3.59341	3.86233	2.77709	2.79939	2.77649	3.65056	3.27829	3.10512	3.12473	3.19394	2.40941	2.91639	2.83507	4.22435	3.85736	512 a - - -
	2.68618	4.42225	2.77519	2.73123	3.46354	2.40513	3.72494	3.29354	2.67741	2.69355	4.24690	2.90347	2.73739	3.18146	2.89801	2.37887	2.77519	2.98518	4.58477	3.61503	
	0.01744	4.45352	5.17586	0.61958	0.77255	0.55259	0.85674														
467	3.11093	5.70872	1.12882	1.63847	5.00624	3.46534	3.93944	4.52784	2.88251	3.99828	4.81652	2.91766	4.04998	3.08817	2.90934	3.00171	3.38350	4.08578	6.14030	4.64614	513 d - - -
	2.68618	4.42225	2.77519	2.73123	3.46354	2.40513	3.72494	3.29354	2.67741	2.69355	4.24690	2.90347	2.73739	3.18146	2.89801	2.37887	2.77519	2.98518	4.58477	3.61503	
	0.01744	4.45352	5.17586	0.61958	0.77255	0.55259	0.85674														
468	2.23226	3.81770	3.57338	3.37997	3.08633	3.17385	4.03584	2.20978	3.28660	2.36234	3.31933	3.65543	4.11842	3.53430	3.06809	3.02073	2.90737	1.86336	4.81261	3.18226	514 v - - -
	2.68618	4.42225	2.77519	2.73123	3.46354	2.40513	3.72494	3.29354	2.67741	2.69355	4.24690	2.90347	2.73739	3.18146	2.89801	2.37887	2.77519	2.98518	4.58477	3.61503	
	0.01744	4.45352	5.17586	0.61958	0.77255	0.55259	0.85674														
469	2.87905	4.45395	4.94056	4.39599	3.70940	4.48063	4.94793	1.08572	4.28318	2.28363	3.38597	4.55758	4.79208	4.50615	4.43639	3.82712	2.81517	1.42155	5.48351	4.28694	515 i - - -
	2.68618	4.42225	2.77519	2.73123	3.46354	2.40513	3.72494	3.29354	2.67741	2.69355	4.24690	2.90347	2.73739	3.18146	2.89801	2.37887	2.77519	2.98518	4.58477	3.61503	
	0.01744	4.45352	5.17586	0.61958	0.77255	0.55259	0.85674														
470	2.39553	5.10013	2.59836	2.19585	4.40715	3.47431	3.66622	3.86648	2.09359	2.59492	4.15141	2.95837	3.86763	2.63111	2.68818	2.48395	2.88389	3.16195	5.55581	4.16993	516 k - - -
	2.68618	4.42225	2.77519	2.73123	3.46354	2.40513	3.72494	3.29354	2.67741	2.69355	4.24690	2.90347	2.73739	3.18146	2.89801	2.37887	2.77519	2.98518	4.58477	3.61503	
	0.01744	4.45352	5.17586	0.61958	0.77255	0.55259	0.85674														
471	2.52856	5.21059	1.90659	2.08890	4.53510	3.46291	3.68946	3.63189	2.45407	3.50806	4.26208	2.75735	3.07506	2.66177	2.95170	2.40389	2.96841	3.59119	5.65510	4.24641	517 d - - -
	2.68618	4.42225	2.77519	2.73123	3.46354	2.40513	3.72494	3.29354	2.67741	2.69355	4.24690	2.90347	2.73739	3.18146	2.89801	2.37887	2.77519	2.98518	4.58477	3.61503	
	0.01744	4.45352	5.17586	0.61958	0.77255	0.55259	0.85674														
472	2.60153	4.47662	3.44991	2.89317	3.64250	2.74561	3.88377	3.00889	2.85487	1.79005	3.16731	2.92283	4.01077	3.09004	3.23316	2.61729	2.53187	2.76963	5.06359	3.81577	518 l - - -
	2.68618	4.42225	2.77519	2.73123	3.46354	2.40513	3.72494	3.29354	2.67741	2.69355	4.24690	2.90347	2.73739	3.18146	2.89801	2.37887	2.77519	2.98518	4.58477	3.61503	
	0.01744	4.45352	5.17586	0.61958	0.77255	0.55259	0.85674														
473	2.58767	5.00047	2.99591	2.30699	4.00607	1.87376	2.98506	3.70411	2.39471	3.27513	4.07059	2.76183	3.90089	2.84552	2.95879	2.71667	2.94258	3.14451	5.48657	4.12936	519 g - - -
	2.68618	4.42225	2.77519	2.73123	3.46354	2.40513	3.72494	3.29354	2.67741	2.69355	4.24690	2.90347	2.73739	3.18146	2.89801	2.37887	2.77519	2.98518	4.58477	3.61503	
	0.01744	4.45352	5.17586	0.61958	0.77255	0.55259	0.85674														
474	2.68298	4.24594	3.87531	2.89800	2.33017	2.88046	4.00914	2.48034	3.21829	2.17583	3.35590	3.60596	3.67100	3.47901	3.48183	3.00399	2.91498	2.50487	4.82875	2.40693	520 l - - -
	2.68618	4.42225	2.77519	2.73123	3.46354	2.40513	3.72494	3.29354	2.67741	2.69355	4.24690	2.90347	2.73739	3.18146	2.89801	2.37887	2.77519	2.98518	4.58477	3.61503	
	0.01744	4.45352	5.17586	0.61958	0.77255	0.55259	0.85674														
475	2.69749	4.29625	3.45955	3.25929	3.44164	3.14147	4.04142	2.59140	3.19204	2.05476	3.42549	3.58767	4.11715	3.46671	3.47981	2.69503	2.93930	1.50632	4.93052	3.71702	521 v - - -
	2.68618	4.42225	2.77519	2.73123	3.46354	2.40513	3.72494	3.29354	2.67741	2.69355	4.24690	2.90347	2.73739	3.18146	2.89801	2.37887	2.77519	2.98518	4.58477	3.61503	
	0.01744	4.45352	5.17586	0.61958	0.77255	0.55259	0.85674														
476	2.88504	4.84599	3.48119	2.91581	2.87712	3.68631	3.87942	3.48130	2.56099	3.07270	3.98785	3.34782	3.24627	3.11158	1.21065	2.98271	3.12815	3.21864	5.25019	3.90324	522 r - - -
	2.68618	4.42225	2.77519	2.73123	3.46354	2.40513	3.72494	3.29354	2.67741	2.69355	4.24690	2.90347	2.73739	3.18146	2.89801	2.37887	2.77519	2.98518	4.58477	3.61503	
	0.01744	4.45352	5.17586	0.61958	0.77255	0.55259	0.85674														
477	2.51896	5.20604	1.92295	2.29163	4.56235	3.43358	3.75834	4.03395	2.56097	3.54918	4.32073	2.95683	2.77021	2.87945	3.06431	1.92181	2.87771	3.61860	5.71296	4.30651	523 s - - -
	2.68618	4.42225	2.77519	2.73123	3.46354	2.40513	3.72494	3.29354	2.67741	2.69355	4.24690	2.90347	2.73739	3.18146	2.89801	2.37887	2.77519	2.98518	4.58477	3.61503	
	0.01744	4.45352	5.17586	0.61958	0.77255	0.55259	0.85674														
478	3.26819	5.64177	0.70127	2.51885	5.12935	3.51812	4.20121	4.70306	3.28187	4.21940	5.10865	3.09085	2.63178	3.39468	3.87363	3.18988	3.60624	4.24808	6.29253	4.86416	524 d - - -

	2.68618	4.42225	2.77519	2.73123	3.46354	2.40513	3.72494	3.29354	2.67741	2.69355	4.24690	2.90347	2.73739	3.18146	2.89801	2.37887	2.77519	2.98518	4.58477	3.61503	
	0.01744	4.45352	5.17586	0.61958	0.77255	0.55259	0.85674														
479	2.90317	4.71258	3.83009	3.55576	4.15801	0.83374	4.53432	3.53746	3.52804	1.97972	4.28127	3.80879	4.25456	3.89370	3.79670	3.10435	3.32053	3.26517	5.62254	4.36278	525 g - - -
	2.68618	4.42225	2.77519	2.73123	3.46354	2.40513	3.72494	3.29354	2.67741	2.69355	4.24690	2.90347	2.73739	3.18146	2.89801	2.37887	2.77519	2.98518	4.58477	3.61503	
	0.01744	4.45352	5.17586	0.61958	0.77255	0.55259	0.85674														
480	2.64251	5.11184	3.09382	2.47325	4.42707	3.09242	3.69759	3.86028	1.45894	3.38531	3.40031	3.03080	3.93381	2.69490	2.43484	2.76250	2.90523	3.48845	5.54675	4.20445	526 k - - -
	2.68618	4.42225	2.77519	2.73123	3.46354	2.40513	3.72494	3.29354	2.67741	2.69355	4.24690	2.90347	2.73739	3.18146	2.89801	2.37887	2.77519	2.98518	4.58477	3.61503	
	0.01744	4.45352	5.17586	0.61958	0.77255	0.55259	0.85674														
481	2.30385	4.62015	3.34477	2.77306	4.33612	3.38970	4.22120	3.58900	3.11434	3.36402	4.24654	3.40514	4.03025	3.46112	3.47055	2.81731	0.96254	3.22501	5.69304	4.43731	527 t - - -
	2.68618	4.42225	2.77519	2.73123	3.46354	2.40513	3.72494	3.29354	2.67741	2.69355	4.24690	2.90347	2.73739	3.18146	2.89801	2.37887	2.77519	2.98518	4.58477	3.61503	
	0.01744	4.45352	5.17586	0.61958	0.77255	0.55259	0.85674														
482	3.31203	4.15376	5.07675	4.50762	3.36698	4.58164	4.96014	2.24793	4.36432	0.86505	2.87271	4.67488	4.83067	4.47706	4.45313	3.92724	3.54587	1.74503	5.32481	4.22955	528 l - - -
	2.68618	4.42225	2.77519	2.73123	3.46354	2.40513	3.72494	3.29354	2.67741	2.69355	4.24690	2.90347	2.73739	3.18146	2.89801	2.37887	2.77519	2.98518	4.58477	3.61503	
	0.01744	4.45352	5.17586	0.61958	0.77255	0.55259	0.85674														
483	3.17196	4.47406	5.05135	4.49183	3.54399	4.50490	4.92653	1.37275	4.35820	1.72077	2.78898	4.61299	4.78932	4.51958	4.45266	3.84961	3.00924	1.40916	5.36716	4.21059	529 i - - -
	2.68618	4.42225	2.77519	2.73123	3.46354	2.40513	3.72494	3.29354	2.67741	2.69355	4.24690	2.90347	2.73739	3.18146	2.89801	2.37887	2.77519	2.98518	4.58477	3.61503	
	0.01744	4.45352	5.17586	0.61958	0.77255	0.55259	0.85674														
484	1.98784	5.07076	2.97173	2.35916	4.37450	2.49081	3.69633	3.82265	2.35759	3.36355	4.14130	2.98414	2.83549	2.58582	2.78298	2.61257	2.94386	3.44291	5.54686	4.17389	530 a - - -
	2.68618	4.42225	2.77519	2.73123	3.46354	2.40513	3.72494	3.29354	2.67741	2.69355	4.24690	2.90347	2.73739	3.18146	2.89801	2.37887	2.77519	2.98518	4.58477	3.61503	
	0.01744	4.45352	5.17586	0.61958	0.77255	0.55259	0.85674														
485	3.09568	4.95319	3.62734	3.10017	2.95396	3.90205	3.77553	3.54473	2.89195	3.06493	4.04866	3.49320	4.27929	1.19166	3.21876	3.17360	3.31920	3.31680	3.73353	2.24268	531 q - - -
	2.68618	4.42225	2.77519	2.73123	3.46354	2.40513	3.72494	3.29354	2.67741	2.69355	4.24690	2.90347	2.73739	3.18146	2.89801	2.37887	2.77519	2.98518	4.58477	3.61503	
	0.01744	4.45352	5.17586	0.61958	0.77255	0.55259	0.85674														
486	3.09361	4.73302	3.93498	3.47123	2.43104	3.96481	3.90297	3.24079	3.40627	2.80131	3.85180	3.74795	1.18787	3.67370	3.69371	3.28459	3.35227	3.06021	4.42194	2.26509	532 p - - -
	2.68618	4.42225	2.77519	2.73123	3.46354	2.40513	3.72494	3.29354	2.67741	2.69355	4.24690	2.90347	2.73739	3.18146	2.89801	2.37887	2.77519	2.98518	4.58477	3.61503	
	0.06351	4.45352	2.99771	0.61958	0.77255	0.55259	0.85674														
487	2.74124	5.00940	3.02107	2.39746	4.28895	3.05985	1.78554	3.71541	2.34525	3.28250	4.08605	3.02929	3.91952	2.85210	2.72306	2.52302	2.97186	3.13956	5.48599	4.14521	533 h - - -
	2.68618	4.42225	2.77519	2.73123	3.46354	2.40513	3.72494	3.29354	2.67741	2.69355	4.24690	2.90347	2.73739	3.18146	2.89801	2.37887	2.77519	2.98518	4.58477	3.61503	
	0.01825	4.40826	5.13060	0.61958	0.77255	0.61798	0.77443														
488	3.19868	4.56520	4.83566	4.26273	3.31961	4.41522	4.76789	2.29748	4.12153	0.95550	2.60799	4.46191	4.68970	4.27350	4.24664	3.74425	2.96785	1.90300	5.21707	4.11193	534 l - - -
	2.68618	4.42225	2.77519	2.73123	3.46354	2.40513	3.72494	3.29354	2.67741	2.69355	4.24690	2.90347	2.73739	3.18146	2.89801	2.37887	2.77519	2.98518	4.58477	3.61503	
	0.01825	4.40826	5.13060	0.61958	0.77255	0.50695	0.92212														
489	2.67552	5.19595	2.32898	2.12805	4.53126	3.46359	2.91003	4.01118	2.38620	3.30338	4.24080	2.62151	3.86564	2.75575	2.27330	2.38470	2.93734	3.58168	5.63243	4.22573	535 e - - -
	2.68618	4.42225	2.77519	2.73123	3.46354	2.40513	3.72494	3.29354	2.67741	2.69355	4.24690	2.90347	2.73739	3.18146	2.89801	2.37887	2.77519	2.98518	4.58477	3.61503	
	0.01744	4.45352	5.17586	0.61958	0.77255	0.55259	0.85674														
490	2.67662	4.30123	3.73068	3.15911	2.46115	3.21834	3.59245	2.64051	2.92194	1.87621	3.40997	3.51183	3.16365	3.37402	3.39755	2.81871	2.55222	2.56657	4.89546	3.67826	536 l - - -
	2.68618	4.42225	2.77519	2.73123	3.46354	2.40513	3.72494	3.29354	2.67741	2.69355	4.24690	2.90347	2.73739	3.18146	2.89801	2.37887	2.77519	2.98518	4.58477	3.61503	
	0.01744	4.45352	5.17586	0.61958	0.77255	0.55259	0.85674														
491	2.87056	5.41589	1.40319	2.15123	4.73389	2.74138	3.79074	4.22888	2.48600	3.71077	4.48237	2.69591	3.59250	2.91268	2.95253	2.73898	3.13183	3.79550	5.85401	4.41102	537 d - - -
	2.68615	4.42230	2.77512	2.73113	3.46359	2.40518	3.72499	3.29359	2.67746	2.69360	4.24695	2.90352	2.73737	3.18151	2.89772	2.37886	2.77525	2.98523	4.58482	3.61508	
	0.46955	1.47926	1.91792	0.18083	1.79923	0.55259	0.85674														
492	2.69623	5.21281	2.01816	2.14471	4.53672	3.16407	3.66690	4.01713	2.44528	3.50969	4.26900	2.78018	2.61207	2.71436	2.54753	2.61728	2.96335	3.59402	5.65558	4.24026	539 d - - -
	2.68618	4.42225	2.77519	2.73123	3.46354	2.40513	3.72494	3.29354	2.67741	2.69355	4.24690	2.90347	2.73739	3.18146	2.89801	2.37887	2.77519	2.98518	4.58477	3.61503	
	0.05287	4.30123	3.27163	0.61958	0.77255	0.58366	0.81611														
493	2.70061	5.05464	3.05487	2.48750	4.35940	3.25779	3.66664	3.79218	1.67018	3.32676	4.11601	2.43443	3.89777	2.75274	2.45108	2.63669	2.95376	3.42656	3.62700	4.15500	540 k - - -
	2.68618	4.42225	2.77519	2.73123	3.46354	2.40513	3.72494	3.29354	2.67741	2.69355	4.24690	2.90347	2.73739	3.18146	2.89801	2.37887	2.77519	2.98518	4.58477	3.61503	
	0.01942	4.34713	5.06948	0.61958	0.77255	0.56621	0.83857														
494	2.66874	4.80483	2.70725	2.42317	3.63957	3.51419	3.68224	3.43243	2.11495	2.13888	3.32326	3.02327	3.90303	2.85205	2.84477	2.72174	2.89990	3.12702	5.31901	3.69257	541 k - - -
	2.68618	4.42225	2.77519	2.73123	3.46354	2.40513	3.72494	3.29354	2.67741	2.69355	4.24690	2.90347	2.73739	3.18146	2.89801	2.37887	2.77519	2.98518	4.58477	3.61503	
	0.01834	4.40356	5.12591	0.61958	0.77255	0.62437	0.76701														
495	2.73109	4.39220	2.89734	3.11799	3.36651	3.71984	3.94858	2.83614	3.04762	2.39291	2.00291	3.49159	4.09795	3.29769	3.36662	2.98293	2.96351	2.64340	2.37640	3.58325	542 m - - -
	2.68618	4.42225	2.77519	2.73123	3.46354	2.40513	3.72494	3.29354	2.67741	2.69355	4.24690	2.90347	2.73739	3.18146	2.89801	2.37887	2.77519	2.98518	4.58477	3.61503	
	0.01834	4.40356	5.12591	0.61958	0.77255	0.62437	0.76701														
496	2.95029	4.43253	4.21428	3.67537	2.41471	3.97047	3.91539	2.88582	3.55318	2.53492	3.53155	3.84777	4.33463	3.76199	3.74897	3.26479	1.87256	2.70902	1.81541	2.63127	543 w - - -
	2.68618	4.42225	2.77519	2.73123	3.46354	2.40513	3.72494	3.29354	2.67741	2.69355	4.24690	2.90347	2.73739	3.18146	2.89801	2.37887	2.77519	2.98518	4.58477	3.61503	
	0.01834	4.40356	5.12591	0.61958	0.77255	0.62437	0.76701														
497	2.55251	4.51577	3.14778	2.66848	3.67659	3.59741	3.83483	2.30639	2.70336	2.45663	3.61323	3.25858	3.98178	3.09842	3.01253	2.83038	1.94227	2.75707	5.08233	3.82394	544 t - - -
	2.68618	4.42225	2.77519	2.73123	3.46354	2.40513	3.72494	3.29354	2.67741	2.69355	4.24690	2.90347	2.73739	3.18146	2.89801	2.37887	2.77519	2.98518	4.58477	3.61503	
	0.01834	4.40356	5.12591	0.61958	0.77255	0.50276	0.92851														
498	2.72899	4.84217	2.09091	2.35665	4.05924	3.23274	3.78101	3.43272	2.63395	1.92913	3.93136	3.07112	3.94098	2.95932	3.09168	2.78297	2.96790	2.78611	5.37439	4.05247	545 l - - -
	2.68618	4.42225	2.77519	2.73123	3.46354	2.40513	3.72494	3.29354	2.67741	2.69355	4.24690	2.90347	2.73739	3.18146	2.89801	2.37887	2.77519	2.98518	4.58477	3.61503	
	0.01744	4.45352	5.17586	0.61958	0.77255	0.55259	0.85674														
499	3.14979	5.81105	0.95806	1.91232	5.12186	3.45077	4.01181	4.65728	3.05173	4.13346	4.97901	2.90931	4.08241	3.17601	3.67248	2.37724	3.48747	4.20509	6.28941	4.75590	546 d - - -
	2.68618	4.42225	2.77519	2.73123	3.46354	2.40513	3.72494	3.29354	2.67741	2.69355	4.24690	2.90347	2.73739	3.18146	2.89801	2.37887	2.77519	2.98518	4.58477	3.61503	
	0.01744	4.45352	5.17586	0.61958	0.77255	0.55259	0.85674														
500	2.69757	5.00696	2.21879	2.21524	4.27556	3.49324	2.83957	2.51991	2.47431	3.06382	4.06911	2.99727	3.89079	2.60118	2.94936	2.70181	2.92954	3.24212	5.48580	4.12388	547 e - - -
	2.68618	4.42225	2.77519	2.73123	3.46354	2.40513	3.72494	3.29354	2.67741	2.69355	4.24690	2.90347	2.73739	3.18146	2.89801	2.37887	2.77519	2.98518	4.58477	3.61503	

```
          0.01744  4.45352  5.17586  0.61958  0.77255  0.55259  0.85674
    501   2.58722  4.82508  2.68775  2.54886  4.04813  3.11541  3.73585  3.24530  2.32712  2.84983  3.73780  3.07603  3.91772  2.89986  2.39584  2.73615  2.91567  2.18256  5.33906  4.01831    548 v - - -
          2.68618  4.42225  2.77519  2.73123  3.46354  2.40513  3.72494  3.29354  2.67741  2.69355  4.24690  2.90347  2.73739  3.18146  2.89801  2.37887  2.77519  2.98518  4.58477  3.61503
          0.01744  4.45352  5.17586  0.61958  0.77255  0.55259  0.85674
    502   2.45354  5.26757  3.33673  2.71904  4.66535  3.69805  3.74677  4.05924  1.79147  3.53002  4.34105  3.19589  3.64319  2.68005  1.37182  2.96939  3.18322  3.69657  5.62567  4.36016    549 r - - -
          2.68618  4.42225  2.77519  2.73123  3.46354  2.40513  3.72494  3.29354  2.67741  2.69355  4.24690  2.90347  2.73739  3.18146  2.89801  2.37887  2.77519  2.98518  4.58477  3.61503
          0.01744  4.45352  5.17586  0.61958  0.77255  0.55259  0.85674
    503   2.45938  4.88715  2.80094  2.52286  4.12665  2.36381  3.71641  3.20620  2.22573  2.77343  3.95778  2.91712  3.87109  2.86565  2.82038  2.71594  2.60731  3.21978  4.36190  4.05317    550 k - - -
          2.68618  4.42225  2.77519  2.73123  3.46354  2.40513  3.72494  3.29354  2.67741  2.69355  4.24690  2.90347  2.73739  3.18146  2.89801  2.37887  2.77519  2.98518  4.58477  3.61503
          0.01744  4.45352  5.17586  0.61958  0.77255  0.55259  0.85674
    504   2.44347  4.02866  3.12768  2.52122  4.03309  2.65042  3.06076  3.12045  2.51831  3.06334  3.59973  2.17245  3.91813  2.90454  2.87912  2.73668  2.91340  3.13480  5.32934  3.68868    551 n - - -
          2.68618  4.42225  2.77519  2.73123  3.46354  2.40513  3.72494  3.29354  2.67741  2.69355  4.24690  2.90347  2.73739  3.18146  2.89801  2.37887  2.77519  2.98518  4.58477  3.61503
          0.29009  4.45352  1.42641  0.61958  0.77255  0.55259  0.85674
    505   2.59149  3.17372  3.19234  3.20908  2.61125  3.64450  3.92640  2.42037  3.12760  2.35437  3.02997  3.51626  3.41096  3.38837  3.14296  2.91295  2.82347  2.23100  4.75702  3.29320    552 v - - -
          2.68618  4.42225  2.77519  2.73123  3.46354  2.40513  3.72494  3.29354  2.67741  2.69355  4.24690  2.90347  2.73739  3.18146  2.89801  2.37887  2.77519  2.98518  4.58477  3.61503
          0.02284  4.18627  4.90861  0.61958  0.77255  0.37484  1.16284
    506   2.38839  4.94226  2.77683  2.12462  4.19720  3.50111  3.60726  3.46901  2.47564  3.20820  3.97948  3.01751  3.60501  2.67623  2.94807  2.70176  2.30650  2.52866  5.43311  3.71087    553 e - - -
          2.68618  4.42225  2.77519  2.73123  3.46354  2.40513  3.72494  3.29354  2.67741  2.69355  4.24690  2.90347  2.73739  3.18146  2.89801  2.37887  2.77519  2.98518  4.58477  3.61503
          0.01744  4.45352  5.17586  0.61958  0.77255  0.55259  0.85674
    507   2.39916  4.13329  4.31550  3.15246  2.28593  3.82834  4.14088  1.99705  3.57728  1.93095  3.23268  3.86789  4.19292  3.77084  3.70671  3.12394  2.93023  2.17443  4.74954  3.07273    554 l - - -
          2.68618  4.42225  2.77519  2.73123  3.46354  2.40513  3.72494  3.29354  2.67741  2.69355  4.24690  2.90347  2.73739  3.18146  2.89801  2.37887  2.77519  2.98518  4.58477  3.61503
          0.01744  4.45352  5.17586  0.61958  0.77255  0.55259  0.85674
    508   2.41296  5.00901  3.01557  2.45827  4.28527  3.18668  3.63864  3.68896  2.18272  3.18216  3.90675  2.76374  3.59122  2.34795  2.58193  2.51612  2.81305  3.16518  3.59626  4.12080    555 k - - -
          2.68618  4.42225  2.77519  2.73123  3.46354  2.40513  3.72494  3.29354  2.67741  2.69355  4.24690  2.90347  2.73739  3.18146  2.89801  2.37887  2.77519  2.98518  4.58477  3.61503
          0.01744  4.45352  5.17586  0.61958  0.77255  0.55259  0.85674
    509   2.65699  5.00603  2.22058  2.46308  4.28532  3.48873  3.69318  3.72470  2.46457  3.28637  4.07303  2.82648  3.88506  2.82023  2.68183  1.90186  2.77978  3.16194  3.96301  4.12562    556 s - - -
          2.68618  4.42225  2.77519  2.73123  3.46354  2.40513  3.72494  3.29354  2.67741  2.69355  4.24690  2.90347  2.73739  3.18146  2.89801  2.37887  2.77519  2.98518  4.58477  3.61503
          0.01744  4.45352  5.17586  0.61958  0.77255  0.55259  0.85674
    510   2.15259  4.64660  3.27016  2.94392  4.48690  2.71724  4.16430  3.91720  3.05254  3.54590  4.36440  3.31959  1.22730  3.06745  3.45520  2.52948  3.01535  3.45195  5.77824  4.49786    557 p - - -
          2.68618  4.42225  2.77519  2.73123  3.46354  2.40513  3.72494  3.29354  2.67741  2.69355  4.24690  2.90347  2.73739  3.18146  2.89801  2.37887  2.77519  2.98518  4.58477  3.61503
          0.01744  4.45352  5.17586  0.61958  0.77255  0.55259  0.85674
    511   2.47900  4.66118  3.24741  2.56950  3.84536  3.56992  3.51527  3.10200  2.66445  1.97646  3.26322  2.45523  3.60670  2.72081  3.09111  2.78896  2.91393  2.89678  5.20385  3.91849    558 l - - -
          2.68618  4.42225  2.77519  2.73123  3.46354  2.40513  3.72494  3.29354  2.67741  2.69355  4.24690  2.90347  2.73739  3.18146  2.89801  2.37887  2.77519  2.98518  4.58477  3.61503
          0.01744  4.45352  5.17586  0.61958  0.77255  0.55259  0.85674
    512   2.38560  4.97428  2.87896  2.44600  4.23932  3.49517  3.23128  3.62203  2.18346  2.98303  3.98822  2.77337  3.55438  2.80349  2.82012  2.47558  2.82520  2.40865  5.45804  4.10172    559 k - - -
          2.68618  4.42225  2.77519  2.73123  3.46354  2.40513  3.72494  3.29354  2.67741  2.69355  4.24690  2.90347  2.73739  3.18146  2.89801  2.37887  2.77519  2.98518  4.58477  3.61503
          0.03308  4.45352  3.86785  0.61958  0.77255  0.55259  0.85674
    513   1.99349  4.69290  3.16834  2.65138  3.88568  3.38299  3.25980  3.21022  2.54455  2.29569  3.70467  3.14257  3.94128  2.85797  2.87670  2.62806  2.55974  2.91420  5.22975  3.93654    560 a - - -
          2.68618  4.42225  2.77519  2.73123  3.46354  2.40513  3.72494  3.29354  2.67741  2.69355  4.24690  2.90347  2.73739  3.18146  2.89801  2.37887  2.77519  2.98518  4.58477  3.61503
          0.13567  4.43815  2.16240  0.61958  0.77255  0.57562  0.82635
    514   2.58155  4.20448  3.83074  3.25644  2.85624  3.40317  3.97267  2.44402  2.96446  1.57877  3.31211  3.56283  4.06517  3.18059  3.42481  2.79371  2.87259  2.45818  4.80499  3.59746    561 l - - -
          2.68618  4.42225  2.77519  2.73123  3.46354  2.40513  3.72494  3.29354  2.67741  2.69355  4.24690  2.90347  2.73739  3.18146  2.89801  2.37887  2.77519  2.98518  4.58477  3.61503
          0.09974  4.32239  2.50523  0.61958  0.77255  0.72458  0.66267
    515   2.51255  4.88642  3.00514  2.47028  4.22456  3.38039  3.67668  3.65188  2.43105  3.22639  4.02431  2.06960  3.30670  2.81351  2.31717  2.65605  2.60860  3.08951  5.43543  4.09244    562 n - - -
          2.68618  4.42225  2.77519  2.73123  3.46354  2.40513  3.72494  3.29354  2.67741  2.69355  4.24690  2.90347  2.73739  3.18146  2.89801  2.37887  2.77519  2.98518  4.58477  3.61503
          0.08074  4.24420  2.76110  0.61958  0.77255  0.80600  0.59175
    516   2.19010  4.96747  2.87854  1.93264  4.25175  3.28775  3.62298  3.39386  2.37883  3.24693  4.03245  2.87473  3.81703  2.56188  2.69481  2.50424  2.86626  3.32644  5.44253  4.07625    563 e - - -
          2.68618  4.42225  2.77519  2.73123  3.46354  2.40513  3.72494  3.29354  2.67741  2.69355  4.24690  2.90347  2.73739  3.18146  2.89801  2.37887  2.77519  2.98518  4.58477  3.61503
          0.05722  4.18630  3.20870  0.61958  0.77255  0.85867  0.55117
    517   2.48608  4.39232  3.20220  2.66376  3.27770  3.55049  3.79745  2.88653  2.76944  2.48807  2.07608  3.24350  3.93528  2.89159  3.14315  2.79509  2.83836  2.65856  4.96313  3.71522    564 m - - -
          2.68618  4.42225  2.77519  2.73123  3.46354  2.40513  3.72494  3.29354  2.67741  2.69355  4.24690  2.90347  2.73739  3.18146  2.89801  2.37887  2.77519  2.98518  4.58477  3.61503
          0.02363  4.15271  4.87506  0.61958  0.77255  0.88670  0.53105
    518   2.42318  4.77548  2.95531  2.37588  4.03790  3.40146  3.67000  3.44878  2.46745  2.86882  3.88395  2.92342  3.83565  2.68344  2.93078  2.05743  2.34815  3.12874  5.31432  3.98804    565 s - - -
          2.68618  4.42225  2.77519  2.73123  3.46354  2.40513  3.72494  3.29354  2.67741  2.69355  4.24690  2.90347  2.73739  3.18146  2.89801  2.37887  2.77519  2.98518  4.58477  3.61503
          0.02363  4.15271  4.87506  0.61958  0.77255  0.88670  0.53105
    519   2.46950  4.96496  2.30442  2.28813  3.54058  3.38057  3.33351  3.68432  2.31803  3.24142  4.02876  2.88955  2.73829  2.74842  2.88381  2.61637  2.86373  3.31931  5.44086  4.07014    566 e - - -
          2.68298  4.42243  2.77585  2.73088  3.46368  2.40542  3.72329  3.29296  2.67824  2.69450  4.24821  2.90364  2.73742  3.18233  2.89643  2.38016  2.77536  2.98563  4.58475  3.61410
          0.71988  0.66711        *  2.78909  0.06345  0.00000        *
//
HMMER3/f [3.1b2 | February 2015]
NAME  mmoX_strict
LENG  526
ALPH  amino
RF    no
MM    no
CONS  yes
CS    no
```

```
MAP   yes
DATE  Wed Mar 28 11:52:53 2018
NSEQ  16
EFFN  0.449219
CKSUM 774237873
STATS LOCAL MSV      -12.0810  0.69782
STATS LOCAL VITERBI  -12.6726  0.69782
STATS LOCAL FORWARD   -6.4060  0.69782
HMM          A        C        D        E        F        G        H        I        K        L        M        N        P        Q        R        S        T        V        W        Y
            m->m     m->i     m->d     i->m     i->i     d->m     d->d
  COMPO   2.47825  4.35226  2.90000  2.67093  3.20748  2.83359  3.66996  2.88232  2.72161  2.49025  3.70251  3.07138  3.35290  3.14913  2.96306  2.71750  2.87915  2.66011  3.90628  3.30247
          2.68618  4.42225  2.77519  2.73123  3.46354  2.40513  3.72494  3.29354  2.67741  2.69355  4.24690  2.90347  2.73739  3.18146  2.89801  2.37887  2.77519  2.98518  4.58477  3.61503
          0.03268  3.83303  4.55537  0.61958  0.77255  0.00000        *
      1   2.93904  4.49431  4.00741  3.59844  3.19183  3.80469  4.34274  2.36355  3.36846  1.79296  1.45569  3.88063  4.26555  3.75324  3.57830  3.28378  3.24383  2.36495  5.02707  3.78505      1 m - - -
          2.68618  4.42225  2.77519  2.73123  3.46354  2.40513  3.72494  3.29354  2.67741  2.69355  4.24690  2.90347  2.73739  3.18146  2.89801  2.37887  2.77519  2.98518  4.58477  3.61503
          0.09366  3.83303  2.69170  0.61958  0.77255  0.48576  0.95510
      2   1.30355  4.12316  3.26253  3.01609  4.01780  2.92881  4.09074  3.23380  3.04453  3.04727  3.99766  3.22067  3.23520  3.38455  3.33350  2.41158  2.69096  2.84549  5.41830  4.19758      2 a - - -
          2.68618  4.42225  2.77519  2.73123  3.46354  2.40513  3.72494  3.29354  2.67741  2.69355  4.24690  2.90347  2.73739  3.18146  2.89801  2.37887  2.77519  2.98518  4.58477  3.61503
          0.03470  3.77407  4.49641  0.61958  0.77255  0.53480  0.88137
      3   3.03823  4.43784  4.39817  3.89388  3.17623  4.21674  4.58949  1.46972  3.70772  1.47996  3.06084  4.18254  4.52347  4.00741  3.91889  3.58800  3.29240  1.85241  5.13359  3.90207      3 i - - -
          2.68618  4.42225  2.77519  2.73123  3.46354  2.40513  3.72494  3.29354  2.67741  2.69355  4.24690  2.90347  2.73739  3.18146  2.89801  2.37887  2.77519  2.98518  4.58477  3.61503
          0.03470  3.77407  4.49641  0.61958  0.77255  0.45627  1.00414
      4   2.29672  4.21624  3.17324  2.98785  4.03735  2.94690  4.09847  3.55494  3.05893  3.27847  4.20833  3.21852  3.68259  3.41149  3.34573  1.15212  2.77402  3.09870  5.41316  4.12083      4 s - - -
          2.68618  4.42225  2.77519  2.73123  3.46354  2.40513  3.72494  3.29354  2.67741  2.69355  4.24690  2.90347  2.73739  3.18146  2.89801  2.37887  2.77519  2.98518  4.58477  3.61503
          0.03268  3.83303  4.55537  0.61958  0.77255  0.48576  0.95510
      5   2.28107  4.25779  3.55100  3.08958  3.56931  3.39575  4.02942  2.52059  2.97456  1.94476  3.49177  3.40341  3.92978  3.34122  3.27847  2.76180  2.18959  2.32625  5.12938  3.89625      5 l - - -
          2.68618  4.42225  2.77519  2.73123  3.46354  2.40513  3.72494  3.29354  2.67741  2.69355  4.24690  2.90347  2.73739  3.18146  2.89801  2.37887  2.77519  2.98518  4.58477  3.61503
          0.03268  3.83303  4.55537  0.61958  0.77255  0.48576  0.95510
      6   1.28840  4.08974  3.32133  3.07452  4.09757  2.89173  4.13766  3.36654  3.11400  3.18238  4.07592  3.23481  3.63658  3.43215  3.40172  2.07179  2.65674  2.91807  5.49184  4.25860      6 a - - -
          2.68618  4.42225  2.77519  2.73123  3.46354  2.40513  3.72494  3.29354  2.67741  2.69355  4.24690  2.90347  2.73739  3.18146  2.89801  2.37887  2.77519  2.98518  4.58477  3.61503
          0.03268  3.83303  4.55537  0.61958  0.77255  0.48576  0.95510
      7   2.37458  4.32793  3.02191  2.75292  4.01025  3.06898  3.91445  3.33981  2.77887  3.10516  4.00228  2.63116  3.72315  3.16399  3.11211  2.51910  1.61110  2.96639  5.37144  4.08300      7 t - - -
          2.68618  4.42225  2.77519  2.73123  3.46354  2.40513  3.72494  3.29354  2.67741  2.69355  4.24690  2.90347  2.73739  3.18146  2.89801  2.37887  2.77519  2.98518  4.58477  3.61503
          0.03268  3.83303  4.55537  0.61958  0.77255  0.48576  0.95510
      8   2.75788  4.81780  3.07448  2.59410  4.06201  3.45171  3.61636  3.45570  1.55381  2.71847  3.96895  2.89538  3.88340  2.79226  2.41136  2.80564  2.98689  3.16908  5.25507  3.96900      8 k - - -
          2.68618  4.42225  2.77519  2.73123  3.46354  2.40513  3.72494  3.29354  2.67741  2.69355  4.24690  2.90347  2.73739  3.18146  2.89801  2.37887  2.77519  2.98518  4.58477  3.61503
          0.03268  3.83303  4.55537  0.61958  0.77255  0.48576  0.95510
      9   1.07216  4.17353  3.42348  3.21371  4.01396  2.99897  4.24029  3.13365  3.22896  3.01700  4.05375  3.37300  3.73453  3.57391  3.48180  2.52446  2.79912  2.79493  5.44509  4.24110      9 a - - -
          2.68618  4.42225  2.77519  2.73123  3.46354  2.40513  3.72494  3.29354  2.67741  2.69355  4.24690  2.90347  2.73739  3.18146  2.89801  2.37887  2.77519  2.98518  4.58477  3.61503
          0.03268  3.83303  4.55537  0.61958  0.77255  0.48576  0.95510
     10   1.41346  4.13946  3.60406  3.25487  3.81760  3.11274  4.20815  2.68954  3.19125  2.69772  3.76143  3.41895  3.79883  3.52680  3.46723  2.55546  2.75923  2.14984  5.36576  4.13046     10 a - - -
          2.68618  4.42225  2.77519  2.73123  3.46354  2.40513  3.72494  3.29354  2.67741  2.69355  4.24690  2.90347  2.73739  3.18146  2.89801  2.37887  2.77519  2.98518  4.58477  3.61503
          0.03268  3.83303  4.55537  0.61958  0.77255  0.48576  0.95510
     11   2.46534  4.44659  3.01782  2.64902  3.99386  3.19334  3.77977  3.32267  2.30123  3.02596  3.89796  3.05351  3.76139  2.99425  2.87972  2.39647  1.86211  2.98267  5.30257  4.03374     11 t - - -
          2.68618  4.42225  2.77519  2.73123  3.46354  2.40513  3.72494  3.29354  2.67741  2.69355  4.24690  2.90347  2.73739  3.18146  2.89801  2.37887  2.77519  2.98518  4.58477  3.61503
          0.03268  3.83303  4.55537  0.61958  0.77255  0.48576  0.95510
     12   2.82312  5.22223  1.41080  2.11546  4.47857  3.17460  3.70336  4.02097  2.67069  3.56228  4.44220  2.68537  3.78090  2.50278  3.20064  2.75030  3.11604  3.63804  5.71322  4.25892     12 d - - -
          2.68618  4.42225  2.77519  2.73123  3.46354  2.40513  3.72494  3.29354  2.67741  2.69355  4.24690  2.90347  2.73739  3.18146  2.89801  2.37887  2.77519  2.98518  4.58477  3.61503
          0.03268  3.83303  4.55537  0.61958  0.77255  0.48576  0.95510
     13   1.28840  4.08974  3.32133  3.07452  4.09757  2.89173  4.13766  3.36654  3.11400  3.18238  4.07592  3.23481  3.63658  3.43215  3.40172  2.07179  2.65674  2.91807  5.49184  4.25860     13 a - - -
          2.68618  4.42225  2.77519  2.73123  3.46354  2.40513  3.72494  3.29354  2.67741  2.69355  4.24690  2.90347  2.73739  3.18146  2.89801  2.37887  2.77519  2.98518  4.58477  3.61503
          0.03268  3.83303  4.55537  0.61958  0.77255  0.48576  0.95510
     14   3.11165  4.58599  4.12585  3.76620  3.12182  3.93147  4.43434  2.32453  3.53431  0.92536  3.15834  4.04543  4.37407  3.91210  3.71656  3.49871  3.40796  2.35149  4.98612  3.70011     14 l - - -
          2.68618  4.42225  2.77519  2.73123  3.46354  2.40513  3.72494  3.29354  2.67741  2.69355  4.24690  2.90347  2.73739  3.18146  2.89801  2.37887  2.77519  2.98518  4.58477  3.61503
          0.03268  3.83303  4.55537  0.61958  0.77255  0.48576  0.95510
     15   2.15977  4.64120  2.89728  2.52437  4.15830  2.92070  3.69695  3.52223  1.79202  3.15708  4.00860  2.97680  3.77787  2.87472  2.71886  2.63053  2.86727  3.16702  5.37697  4.09405     15 k - - -
          2.68618  4.42225  2.77519  2.73123  3.46354  2.40513  3.72494  3.29354  2.67741  2.69355  4.24690  2.90347  2.73739  3.18146  2.89801  2.37887  2.77519  2.98518  4.58477  3.61503
          0.03268  3.83303  4.55537  0.61958  0.77255  0.48576  0.95510
     16   1.70362  4.16509  3.71980  3.33040  3.71709  3.26346  4.24896  2.42207  3.25607  2.54041  3.63542  3.51764  3.90392  3.58403  3.53157  2.68850  2.82087  1.73146  5.31689  4.08748     16 a - - -
          2.68618  4.42225  2.77519  2.73123  3.46354  2.40513  3.72494  3.29354  2.67741  2.69355  4.24690  2.90347  2.73739  3.18146  2.89801  2.37887  2.77519  2.98518  4.58477  3.61503
          0.03268  3.83303  4.55537  0.61958  0.77255  0.48576  0.95510
     17   2.73194  4.88682  2.58298  2.37354  3.86071  3.27100  3.00231  3.72742  2.48409  3.28526  4.17081  1.71943  3.81955  2.89769  2.86331  2.74028  3.01966  3.38080  5.21586  3.75130     17 n - - -
          2.68618  4.42225  2.77519  2.73123  3.46354  2.40513  3.72494  3.29354  2.67741  2.69355  4.24690  2.90347  2.73739  3.18146  2.89801  2.37887  2.77519  2.98518  4.58477  3.61503
          0.03268  3.83303  4.55537  0.61958  0.77255  0.48576  0.95510
     18   2.68107  4.70085  3.14662  2.67413  4.14011  3.34958  3.69155  3.57367  2.23823  3.14525  4.03418  3.09456  2.68976  2.87481  1.64584  2.75330  2.96168  3.24503  5.30264  4.06377     18 r - - -
          2.68618  4.42225  2.77519  2.73123  3.46354  2.40513  3.72494  3.29354  2.67741  2.69355  4.24690  2.90347  2.73739  3.18146  2.89801  2.37887  2.77519  2.98518  4.58477  3.61503
          0.03268  3.83303  4.55537  0.61958  0.77255  0.48576  0.95510
```

```
19   1.41346  4.13946  3.60406  3.25487  3.81760  3.11274  4.20815  2.68954  3.19125  2.69772  3.76143  3.41895  3.79883  3.52680  3.46723  2.55546  2.75923  2.14984  5.36576  4.13046     19 a - - -
     2.68618  4.42225  2.77519  2.73123  3.46354  2.40513  3.72494  3.29354  2.67741  2.69355  4.24690  2.90347  2.73739  3.18146  2.89801  2.37887  2.77519  2.98518  4.58477  3.61503
     0.03268  3.83303  4.55537  0.61958  0.77255  0.48576  0.95510
20   2.70125  4.46443  3.30428  3.13815  4.13555  3.15421  4.21835  3.67141  3.19219  3.30231  4.35093  3.44851  0.90433  3.58220  3.44165  2.86766  3.13986  3.32122  5.33234  4.25589     20 p - - -
     2.68618  4.42225  2.77519  2.73123  3.46354  2.40513  3.72494  3.29354  2.67741  2.69355  4.24690  2.90347  2.73739  3.18146  2.89801  2.37887  2.77519  2.98518  4.58477  3.61503
     0.03268  3.83303  4.55537  0.61958  0.77255  0.48576  0.95510
21   2.49678  4.20373  3.75665  3.33624  3.64888  3.38456  4.23915  2.30954  3.22895  2.44830  3.56093  3.56178  3.97869  3.57719  3.50471  2.79304  2.08903  1.59573  5.27006  4.03798     21 v - - -
     2.68618  4.42225  2.77519  2.73123  3.46354  2.40513  3.72494  3.29354  2.67741  2.69355  4.24690  2.90347  2.73739  3.18146  2.89801  2.37887  2.77519  2.98518  4.58477  3.61503
     0.03268  3.83303  4.55537  0.61958  0.77255  0.48576  0.95510
22   2.20553  4.17111  3.08384  2.87647  4.23131  1.97412  4.06180  3.69536  3.02680  3.37091  4.20799  3.12602  3.62103  3.32402  3.36003  1.56385  2.68276  3.15484  5.55537  4.30688     22 s - - -
     2.68618  4.42225  2.77519  2.73123  3.46354  2.40513  3.72494  3.29354  2.67741  2.69355  4.24690  2.90347  2.73739  3.18146  2.89801  2.37887  2.77519  2.98518  4.58477  3.61503
     0.03268  3.83303  4.55537  0.61958  0.77255  0.48576  0.95510
23   2.85705  4.33858  4.26805  3.82469  3.45243  3.94181  4.60904  1.81360  3.67074  2.06189  3.35133  4.05620  4.40129  4.01087  3.90428  3.35191  3.17119  1.15614  5.31647  4.04977     23 v - - -
     2.68618  4.42225  2.77519  2.73123  3.46354  2.40513  3.72494  3.29354  2.67741  2.69355  4.24690  2.90347  2.73739  3.18146  2.89801  2.37887  2.77519  2.98518  4.58477  3.61503
     0.03268  3.83303  4.55537  0.61958  0.77255  0.48576  0.95510
24   2.73876  5.18432  2.08330  1.75059  4.49657  2.85988  3.64357  3.97574  2.56457  3.50393  4.32388  2.41020  3.75437  2.79695  3.10367  2.47872  3.01030  3.57537  5.68767  4.24643     24 e - - -
     2.68618  4.42225  2.77519  2.73123  3.46354  2.40513  3.72494  3.29354  2.67741  2.69355  4.24690  2.90347  2.73739  3.18146  2.89801  2.37887  2.77519  2.98518  4.58477  3.61503
     0.03268  3.83303  4.55537  0.61958  0.77255  0.48576  0.95510
25   1.84080  4.21999  3.17136  2.90340  4.07893  2.98596  4.03125  3.37810  2.95108  3.12335  4.04060  3.17597  1.84165  3.29499  3.27361  2.45304  2.73323  2.97262  5.44455  4.20696     25 a - - -
     2.68618  4.42225  2.77519  2.73123  3.46354  2.40513  3.72494  3.29354  2.67741  2.69355  4.24690  2.90347  2.73739  3.18146  2.89801  2.37887  2.77519  2.98518  4.58477  3.61503
     0.03268  3.83303  4.55537  0.61958  0.77255  0.48576  0.95510
26   2.85084  4.83409  2.87832  2.63320  3.99721  3.34074  3.79090  3.62260  2.45691  3.12880  4.15852  3.09230  3.89411  1.30591  2.74155  2.90061  3.14819  3.35495  5.27660  3.96796     26 q - - -
     2.68618  4.42225  2.77519  2.73123  3.46354  2.40513  3.72494  3.29354  2.67741  2.69355  4.24690  2.90347  2.73739  3.18146  2.89801  2.37887  2.77519  2.98518  4.58477  3.61503
     0.03268  3.83303  4.55537  0.61958  0.77255  0.48576  0.95510
27   2.91965  5.02079  2.47306  1.08285  4.36227  3.24954  3.83381  3.80837  2.70946  3.43216  4.40865  2.91254  3.85246  3.05076  3.11366  2.90651  3.22382  3.49656  5.54134  4.26436     27 e - - -
     2.68618  4.42225  2.77519  2.73123  3.46354  2.40513  3.72494  3.29354  2.67741  2.69355  4.24690  2.90347  2.73739  3.18146  2.89801  2.37887  2.77519  2.98518  4.58477  3.61503
     0.03268  3.83303  4.55537  0.61958  0.77255  0.48576  0.95510
28   2.77713  4.33135  4.07016  3.73272  3.52199  3.68239  4.54148  2.05456  3.59987  2.21635  3.49380  3.92869  4.24556  3.96282  3.81605  3.18396  3.13994  1.07623  5.29954  4.02841     28 v - - -
     2.68618  4.42225  2.77519  2.73123  3.46354  2.40513  3.72494  3.29354  2.67741  2.69355  4.24690  2.90347  2.73739  3.18146  2.89801  2.37887  2.77519  2.98518  4.58477  3.61503
     0.03268  3.83303  4.55537  0.61958  0.77255  0.48576  0.95510
29   2.95371  4.77708  3.08911  2.84570  3.25855  3.41543  1.22850  3.65045  2.68990  3.14913  4.18951  3.25638  3.96612  3.21160  2.95766  3.02494  3.25600  3.38639  4.72403  3.21065     29 h - - -
     2.68618  4.42225  2.77519  2.73123  3.46354  2.40513  3.72494  3.29354  2.67741  2.69355  4.24690  2.90347  2.73739  3.18146  2.89801  2.37887  2.77519  2.98518  4.58477  3.61503
     0.03268  3.83303  4.55537  0.61958  0.77255  0.48576  0.95510
30   2.93342  5.02244  3.30360  2.70065  4.43096  3.55731  3.56515  3.79670  1.45615  3.27991  4.15794  3.11308  3.94750  2.70993  1.71111  2.94347  3.11800  3.48664  5.35577  4.16695     30 k - - -
     2.68618  4.42225  2.77519  2.73123  3.46354  2.40513  3.72494  3.29354  2.67741  2.69355  4.24690  2.90347  2.73739  3.18146  2.89801  2.37887  2.77519  2.98518  4.58477  3.61503
     0.03268  3.83303  4.55537  0.61958  0.77255  0.48576  0.95510
31   3.27202  4.66041  4.03210  3.72890  2.62865  3.68698  3.85349  3.29453  3.45219  2.69596  3.93010  3.88606  4.20159  3.84191  3.58729  3.47606  3.57058  3.18378  0.96876  2.63291     31 w - - -
     2.68618  4.42225  2.77519  2.73123  3.46354  2.40513  3.72494  3.29354  2.67741  2.69355  4.24690  2.90347  2.73739  3.18146  2.89801  2.37887  2.77519  2.98518  4.58477  3.61503
     0.03268  3.83303  4.55537  0.61958  0.77255  0.48576  0.95510
32   3.11742  4.56186  4.36339  3.84367  3.04064  4.21357  4.51543  2.15078  3.61412  1.04291  2.61240  4.15123  4.50273  3.91216  3.81428  3.57799  3.36270  2.21717  5.03322  3.83519     32 l - - -
     2.68618  4.42225  2.77519  2.73123  3.46354  2.40513  3.72494  3.29354  2.67741  2.69355  4.24690  2.90347  2.73739  3.18146  2.89801  2.37887  2.77519  2.98518  4.58477  3.61503
     0.03268  3.83303  4.55537  0.61958  0.77255  0.48576  0.95510
33   2.85084  4.83409  2.87832  2.63320  3.99721  3.34074  3.79090  3.62260  2.45691  3.12880  4.15852  3.09230  3.89411  1.30591  2.74155  2.90061  3.14819  3.35495  5.27660  3.96796     33 q - - -
     2.68618  4.42225  2.77519  2.73123  3.46354  2.40513  3.72494  3.29354  2.67741  2.69355  4.24690  2.90347  2.73739  3.18146  2.89801  2.37887  2.77519  2.98518  4.58477  3.61503
     0.03268  3.83303  4.55537  0.61958  0.77255  0.48576  0.95510
34   2.49822  4.59742  2.28567  2.41537  4.20952  3.08187  3.81220  3.67943  2.75674  3.33103  4.18997  2.88580  3.71965  3.02163  3.20469  1.55873  2.87299  3.26318  5.51679  4.15907     34 s - - -
     2.68618  4.42225  2.77519  2.73123  3.46354  2.40513  3.72494  3.29354  2.67741  2.69355  4.24690  2.90347  2.73739  3.18146  2.89801  2.37887  2.77519  2.98518  4.58477  3.61503
     0.03268  3.83303  4.55537  0.61958  0.77255  0.48576  0.95510
35   3.20912  4.62273  4.18444  3.89199  1.07857  3.90702  3.86137  2.73830  3.80015  2.11778  3.47472  3.97513  4.36694  3.97918  3.92960  3.48072  3.51047  2.72599  4.12320  2.49559     35 f - - -
     2.68618  4.42225  2.77519  2.73123  3.46354  2.40513  3.72494  3.29354  2.67741  2.69355  4.24690  2.90347  2.73739  3.18146  2.89801  2.37887  2.77519  2.98518  4.58477  3.61503
     0.03268  3.83303  4.55537  0.61958  0.77255  0.48576  0.95510
36   2.46876  4.50856  2.78195  2.54888  4.12058  3.10960  3.82273  3.51159  2.66673  3.21677  4.09677  1.82926  3.73573  3.03779  3.04206  2.56440  2.26507  3.12768  5.43662  4.10918     36 n - - -
     2.68618  4.42225  2.77519  2.73123  3.46354  2.40513  3.72494  3.29354  2.67741  2.69355  4.24690  2.90347  2.73739  3.18146  2.89801  2.37887  2.77519  2.98518  4.58477  3.61503
     0.03268  3.83303  4.55537  0.61958  0.77255  0.48576  0.95510
37   3.27202  4.66041  4.03210  3.72890  2.62865  3.68698  3.85349  3.29453  3.45219  2.69596  3.93010  3.88606  4.20159  3.84191  3.58729  3.47606  3.57058  3.18378  0.96876  2.63291     37 w - - -
     2.68618  4.42225  2.77519  2.73123  3.46354  2.40513  3.72494  3.29354  2.67741  2.69355  4.24690  2.90347  2.73739  3.18146  2.89801  2.37887  2.77519  2.98518  4.58477  3.61503
     0.03268  3.83303  4.55537  0.61958  0.77255  0.48576  0.95510
38   2.94831  5.06869  0.97632  2.31258  4.44069  3.18175  3.89799  4.02012  2.97493  3.63580  4.61368  2.86669  3.83184  3.13755  3.48525  2.91560  3.28628  3.66374  5.61691  4.32095     38 d - - -
     2.68618  4.42225  2.77519  2.73123  3.46354  2.40513  3.72494  3.29354  2.67741  2.69355  4.24690  2.90347  2.73739  3.18146  2.89801  2.37887  2.77519  2.98518  4.58477  3.61503
     0.03268  3.83303  4.55537  0.61958  0.77255  0.48576  0.95510
39   3.20912  4.62273  4.18444  3.89199  1.07857  3.90702  3.86137  2.73830  3.80015  2.11778  3.47472  3.97513  4.36694  3.97918  3.92960  3.48072  3.51047  2.72599  4.12320  2.49559     39 f - - -
     2.68618  4.42225  2.77519  2.73123  3.46354  2.40513  3.72494  3.29354  2.67741  2.69355  4.24690  2.90347  2.73739  3.18146  2.89801  2.37887  2.77519  2.98518  4.58477  3.61503
     0.03268  3.83303  4.55537  0.61958  0.77255  0.48576  0.95510
40   2.49895  4.92839  2.42974  2.32466  4.28655  3.29087  3.63079  3.68004  1.69254  3.27320  4.11475  2.84532  3.79074  2.78703  2.74295  2.68342  2.94594  3.32995  5.46627  4.12430     40 k - - -
     2.68618  4.42225  2.77519  2.73123  3.46354  2.40513  3.72494  3.29354  2.67741  2.69355  4.24690  2.90347  2.73739  3.18146  2.89801  2.37887  2.77519  2.98518  4.58477  3.61503
     0.03268  3.83303  4.55537  0.61958  0.77255  0.48576  0.95510
41   2.46081  5.06380  2.42891  1.95567  4.35328  3.27578  3.59321  3.80404  2.25429  3.34094  4.14889  2.51421  3.76187  2.47946  2.86014  2.64494  2.92791  3.42951  5.52376  4.13475     41 e - - -
```

```
    2.68618  4.42225  2.77519  2.73123  3.46354  2.40513  3.72494  3.29354  2.67741  2.69355  4.24690  2.90347  2.73739  3.18146  2.89801  2.37887  2.77519  2.98518  4.58477  3.61503
    0.03268  3.83303  4.55537  0.61958  0.77255  0.48576  0.95510
42  2.73199  4.90690  2.56889  2.36304  4.25999  3.25389  3.68777  3.75671  2.16576  3.34482  4.21828  1.70484  3.81059  2.87229  2.76108  2.73900  3.02265  3.39953  5.46742  4.11506    42 n - - -
    2.68618  4.42225  2.77519  2.73123  3.46354  2.40513  3.72494  3.29354  2.67741  2.69355  4.24690  2.90347  2.73739  3.18146  2.89801  2.37887  2.77519  2.98518  4.58477  3.61503
    0.03268  3.83303  4.55537  0.61958  0.77255  0.48576  0.95510
43  2.66776  4.67730  3.19054  2.71832  4.13275  3.33977  3.68369  3.58399  2.17597  3.16867  4.05803  3.10883  3.85048  2.87043  1.54770  2.42675  2.96097  3.24550  5.30051  4.04159    43 r - - -
    2.68618  4.42225  2.77519  2.73123  3.46354  2.40513  3.72494  3.29354  2.67741  2.69355  4.24690  2.90347  2.73739  3.18146  2.89801  2.37887  2.77519  2.98518  4.58477  3.61503
    0.03268  3.83303  4.55537  0.61958  0.77255  0.48576  0.95510
44  2.00606  4.14036  3.38728  3.09061  3.99498  2.99929  4.12551  3.04944  3.05816  2.96339  3.93390  3.28399  3.70892  3.41349  3.34506  2.45586  1.52095  2.70746  5.43710  4.21211    44 t - - -
    2.68618  4.42225  2.77519  2.73123  3.46354  2.40513  3.72494  3.29354  2.67741  2.69355  4.24690  2.90347  2.73739  3.18146  2.89801  2.37887  2.77519  2.98518  4.58477  3.61503
    0.03268  3.83303  4.55537  0.61958  0.77255  0.48576  0.95510
45  2.93703  4.89791  3.11032  2.74002  4.27531  3.43004  3.73906  3.70226  1.09362  3.27630  4.24050  3.16601  3.93369  2.93324  2.42654  2.98198  3.19357  3.41249  5.34669  4.15401    45 k - - -
    2.68618  4.42225  2.77519  2.73123  3.46354  2.40513  3.72494  3.29354  2.67741  2.69355  4.24690  2.90347  2.73739  3.18146  2.89801  2.37887  2.77519  2.98518  4.58477  3.61503
    0.03268  3.83303  4.55537  0.61958  0.77255  0.48576  0.95510
46  3.19134  4.69630  3.78054  3.50310  2.29611  3.78460  3.60201  3.20618  3.36176  2.67014  3.88549  3.67575  4.25966  3.67727  3.55331  3.33175  3.47801  3.07495  3.93816  1.03399    46 y - - -
    2.68618  4.42225  2.77519  2.73123  3.46354  2.40513  3.72494  3.29354  2.67741  2.69355  4.24690  2.90347  2.73739  3.18146  2.89801  2.37887  2.77519  2.98518  4.58477  3.61503
    0.03268  3.83303  4.55537  0.61958  0.77255  0.48576  0.95510
47  1.80616  4.18609  3.16414  2.85822  4.10321  2.95138  3.98783  3.46891  2.91356  3.17814  4.03038  3.13126  2.15259  3.23682  3.25729  2.17428  2.67262  3.01464  5.44939  4.20320    47 a - - -
    2.68618  4.42225  2.77519  2.73123  3.46354  2.40513  3.72494  3.29354  2.67741  2.69355  4.24690  2.90347  2.73739  3.18146  2.89801  2.37887  2.77519  2.98518  4.58477  3.61503
    0.03268  3.83303  4.55537  0.61958  0.77255  0.48576  0.95510
48  2.39269  4.23746  3.39425  3.14521  3.93517  3.07784  4.16539  3.05754  3.08739  2.92240  3.97601  3.36046  3.77873  3.48436  3.34758  2.59359  1.24059  2.75673  5.37648  4.15199    48 t - - -
    2.68618  4.42225  2.77519  2.73123  3.46354  2.40513  3.72494  3.29354  2.67741  2.69355  4.24690  2.90347  2.73739  3.18146  2.89801  2.37887  2.77519  2.98518  4.58477  3.61503
    0.03268  3.83303  4.55537  0.61958  0.77255  0.48576  0.95510
49  2.93703  4.89791  3.11032  2.74002  4.27531  3.43004  3.73906  3.70226  1.09362  3.27630  4.24050  3.16601  3.93369  2.93324  2.42654  2.98198  3.19357  3.41249  5.34669  4.15401    49 k - - -
    2.68618  4.42225  2.77519  2.73123  3.46354  2.40513  3.72494  3.29354  2.67741  2.69355  4.24690  2.90347  2.73739  3.18146  2.89801  2.37887  2.77519  2.98518  4.58477  3.61503
    0.03268  3.83303  4.55537  0.61958  0.77255  0.48576  0.95510
50  3.19134  4.69630  3.78054  3.50310  2.29611  3.78460  3.60201  3.20618  3.36176  2.67014  3.88549  3.67575  4.25966  3.67727  3.55331  3.33175  3.47801  3.07495  3.93816  1.03399    50 y - - -
    2.68618  4.42225  2.77519  2.73123  3.46354  2.40513  3.72494  3.29354  2.67741  2.69355  4.24690  2.90347  2.73739  3.18146  2.89801  2.37887  2.77519  2.98518  4.58477  3.61503
    0.03268  3.83303  4.55537  0.61958  0.77255  0.48576  0.95510
51  2.67722  4.97604  2.48415  2.31384  4.23872  3.34176  2.59413  3.70252  2.01891  3.24456  4.06071  2.84173  3.78670  2.71719  2.58326  2.66284  2.90926  3.34924  5.40416  4.05055    51 k - - -
    2.68618  4.42225  2.77519  2.73123  3.46354  2.40513  3.72494  3.29354  2.67741  2.69355  4.24690  2.90347  2.73739  3.18146  2.89801  2.37887  2.77519  2.98518  4.58477  3.61503
    0.03268  3.83303  4.55537  0.61958  0.77255  0.48576  0.95510
52  2.93904  4.49431  4.00741  3.59844  3.19183  3.80469  4.34274  2.36355  3.36846  1.79296  1.45569  3.88063  4.26555  3.75324  3.57830  3.28378  3.24383  2.36495  5.02707  3.78505    52 m - - -
    2.68618  4.42225  2.77519  2.73123  3.46354  2.40513  3.72494  3.29354  2.67741  2.69355  4.24690  2.90347  2.73739  3.18146  2.89801  2.37887  2.77519  2.98518  4.58477  3.61503
    0.03268  3.83303  4.55537  0.61958  0.77255  0.48576  0.95510
53  1.50306  4.47874  2.39322  2.54364  4.11672  3.09613  3.87372  3.39328  2.82346  3.16904  4.06831  2.97986  3.73455  3.09887  3.24452  2.56670  2.84426  3.03329  5.46313  4.15376    53 a - - -
    2.68618  4.42225  2.77519  2.73123  3.46354  2.40513  3.72494  3.29354  2.67741  2.69355  4.24690  2.90347  2.73739  3.18146  2.89801  2.37887  2.77519  2.98518  4.58477  3.61503
    0.03268  3.83303  4.55537  0.61958  0.77255  0.48576  0.95510
54  2.51314  4.49640  2.91255  2.63515  3.86063  3.19383  3.82575  3.23749  2.68258  3.01939  3.96466  1.75306  3.78975  3.07180  3.04247  2.62769  2.86961  2.54934  5.26605  3.89698    54 n - - -
    2.68618  4.42225  2.77519  2.73123  3.46354  2.40513  3.72494  3.29354  2.67741  2.69355  4.24690  2.90347  2.73739  3.18146  2.89801  2.37887  2.77519  2.98518  4.58477  3.61503
    0.03268  3.83303  4.55537  0.61958  0.77255  0.48576  0.95510
55  2.78288  5.29147  1.95898  1.71988  4.57403  3.19764  3.63910  4.06721  2.55425  3.57355  4.39446  2.39535  3.76112  2.49020  3.09251  2.69714  3.04890  3.65879  5.74012  4.28161    55 e - - -
    2.68618  4.42225  2.77519  2.73123  3.46354  2.40513  3.72494  3.29354  2.67741  2.69355  4.24690  2.90347  2.73739  3.18146  2.89801  2.37887  2.77519  2.98518  4.58477  3.61503
    0.03268  3.83303  4.55537  0.61958  0.77255  0.48576  0.95510
56  2.39269  4.23746  3.39425  3.14521  3.93517  3.07784  4.16539  3.05754  3.08739  2.92240  3.97601  3.36046  3.77873  3.48436  3.34758  2.59359  1.24059  2.75673  5.37648  4.15199    56 t - - -
    2.68618  4.42225  2.77519  2.73123  3.46354  2.40513  3.72494  3.29354  2.67741  2.69355  4.24690  2.90347  2.73739  3.18146  2.89801  2.37887  2.77519  2.98518  4.58477  3.61503
    0.03268  3.83303  4.55537  0.61958  0.77255  0.48576  0.95510
57  2.91529  4.99693  3.22470  2.67995  4.39156  3.52540  3.58444  3.77191  1.32458  3.27138  4.15738  3.10007  3.93724  2.73544  1.96900  2.92975  3.11342  3.46273  5.35654  4.15483    57 k - - -
    2.68618  4.42225  2.77519  2.73123  3.46354  2.40513  3.72494  3.29354  2.67741  2.69355  4.24690  2.90347  2.73739  3.18146  2.89801  2.37887  2.77519  2.98518  4.58477  3.61503
    0.03268  3.83303  4.55537  0.61958  0.77255  0.48576  0.95510
58  2.91965  5.02079  2.47306  1.08285  4.36227  3.24954  3.83381  3.80837  2.70946  3.43216  4.40865  2.91254  3.85246  3.05076  3.11366  2.90651  3.22382  3.49656  5.54134  4.26436    58 e - - -
    2.68618  4.42225  2.77519  2.73123  3.46354  2.40513  3.72494  3.29354  2.67741  2.69355  4.24690  2.90347  2.73739  3.18146  2.89801  2.37887  2.77519  2.98518  4.58477  3.61503
    0.03268  3.83303  4.55537  0.61958  0.77255  0.48576  0.95510
59  2.85084  4.83409  2.87832  2.63320  3.99721  3.34074  3.79090  3.62260  2.45691  3.12880  4.15852  3.09230  3.89411  1.30591  2.74155  2.90061  3.14819  3.35495  5.27660  3.96796    59 q - - -
    2.68618  4.42225  2.77519  2.73123  3.46354  2.40513  3.72494  3.29354  2.67741  2.69355  4.24690  2.90347  2.73739  3.18146  2.89801  2.37887  2.77519  2.98518  4.58477  3.61503
    0.03268  3.83303  4.55537  0.61958  0.77255  0.48576  0.95510
60  3.20912  4.62273  4.18444  3.89199  1.07857  3.90702  3.86137  2.73830  3.80015  2.11778  3.47472  3.97513  4.36694  3.97918  3.92960  3.48072  3.51047  2.72599  4.12320  2.49559    60 f - - -
    2.68618  4.42225  2.77519  2.73123  3.46354  2.40513  3.72494  3.29354  2.67741  2.69355  4.24690  2.90347  2.73739  3.18146  2.89801  2.37887  2.77519  2.98518  4.58477  3.61503
    0.03268  3.83303  4.55537  0.61958  0.77255  0.48576  0.95510
61  2.93703  4.89791  3.11032  2.74002  4.27531  3.43004  3.73906  3.70226  1.09362  3.27630  4.24050  3.16601  3.93369  2.93324  2.42654  2.98198  3.19357  3.41249  5.34669  4.15401    61 k - - -
    2.68618  4.42225  2.77519  2.73123  3.46354  2.40513  3.72494  3.29354  2.67741  2.69355  4.24690  2.90347  2.73739  3.18146  2.89801  2.37887  2.77519  2.98518  4.58477  3.61503
    0.03268  3.83303  4.55537  0.61958  0.77255  0.48576  0.95510
62  2.97065  4.42481  4.30855  3.81542  3.22660  4.08255  4.53944  1.98866  3.64320  1.36231  3.11377  4.09156  4.44806  3.94717  3.86386  3.45882  3.24441  1.58147  5.14671  3.92065    62 l - - -
    2.68618  4.42225  2.77519  2.73123  3.46354  2.40513  3.72494  3.29354  2.67741  2.69355  4.24690  2.90347  2.73739  3.18146  2.89801  2.37887  2.77519  2.98518  4.58477  3.61503
    0.03268  3.83303  4.55537  0.61958  0.77255  0.48576  0.95510
63  2.78250  4.31026  4.05610  3.61307  3.46251  3.82012  4.43743  1.36293  3.46151  2.10764  3.34848  3.87781  4.28501  3.81712  3.70683  3.21401  2.67851  1.84418  5.24658  4.01482    63 i - - -
    2.68618  4.42225  2.77519  2.73123  3.46354  2.40513  3.72494  3.29354  2.67741  2.69355  4.24690  2.90347  2.73739  3.18146  2.89801  2.37887  2.77519  2.98518  4.58477  3.61503
```

```
        0.03268  3.83303  4.55537  0.61958  0.77255  0.48576  0.95510
 64     1.07216  4.17353  3.42348  3.21371  4.01396  2.99897  4.24029  3.13365  3.22896  3.01700  4.05375  3.37300  3.73453  3.57391  3.48180  2.52446  2.79912  2.79493  5.44509  4.24110     64 a - - -
        2.68618  4.42225  2.77519  2.73123  3.46354  2.40513  3.72494  3.29354  2.67741  2.69355  4.24690  2.90347  2.73739  3.18146  2.89801  2.37887  2.77519  2.98518  4.58477  3.61503
        0.03268  3.83303  4.55537  0.61958  0.77255  0.48576  0.95510
 65     2.93703  4.89791  3.11032  2.74002  4.27531  3.43004  3.73906  3.70226  1.09362  3.27630  4.24050  3.16601  3.93369  2.93324  2.42654  2.98198  3.19357  3.41249  5.34669  4.15401     65 k - - -
        2.68618  4.42225  2.77519  2.73123  3.46354  2.40513  3.72494  3.29354  2.67741  2.69355  4.24690  2.90347  2.73739  3.18146  2.89801  2.37887  2.77519  2.98518  4.58477  3.61503
        0.03268  3.83303  4.55537  0.61958  0.77255  0.48576  0.95510
 66     2.91965  5.02079  2.47306  1.08285  4.36227  3.24954  3.83381  3.80837  2.70946  3.43216  4.40865  2.91254  3.85246  3.05076  3.11366  2.90651  3.22382  3.49656  5.54134  4.26436     66 e - - -
        2.68618  4.42225  2.77519  2.73123  3.46354  2.40513  3.72494  3.29354  2.67741  2.69355  4.24690  2.90347  2.73739  3.18146  2.89801  2.37887  2.77519  2.98518  4.58477  3.61503
        0.03268  3.83303  4.55537  0.61958  0.77255  0.48576  0.95510
 67     3.19134  4.69630  3.78054  3.50310  2.29611  3.78460  3.60201  3.20618  3.36176  2.67014  3.88549  3.67575  4.25966  3.67727  3.55331  3.33175  3.47801  3.07495  3.93816  1.03399     67 y - - -
        2.68618  4.42225  2.77519  2.73123  3.46354  2.40513  3.72494  3.29354  2.67741  2.69355  4.24690  2.90347  2.73739  3.18146  2.89801  2.37887  2.77519  2.98518  4.58477  3.61503
        0.03268  3.83303  4.55537  0.61958  0.77255  0.48576  0.95510
 68     1.07216  4.17353  3.42348  3.21371  4.01396  2.99897  4.24029  3.13365  3.22896  3.01700  4.05375  3.37300  3.73453  3.57391  3.48180  2.52446  2.79912  2.79493  5.44509  4.24110     68 a - - -
        2.68618  4.42225  2.77519  2.73123  3.46354  2.40513  3.72494  3.29354  2.67741  2.69355  4.24690  2.90347  2.73739  3.18146  2.89801  2.37887  2.77519  2.98518  4.58477  3.61503
        0.03268  3.83303  4.55537  0.61958  0.77255  0.48576  0.95510
 69     2.96214  4.80276  3.44889  2.96907  4.14736  3.45685  3.79770  3.68689  2.22438  3.21005  4.21188  3.33151  3.96192  3.02053  1.04043  3.04858  3.23116  3.41270  5.24678  4.08393     69 r - - -
        2.68618  4.42225  2.77519  2.73123  3.46354  2.40513  3.72494  3.29354  2.67741  2.69355  4.24690  2.90347  2.73739  3.18146  2.89801  2.37887  2.77519  2.98518  4.58477  3.61503
        0.03268  3.83303  4.55537  0.61958  0.77255  0.48576  0.95510
 70     2.93904  4.49431  4.00741  3.59844  3.19183  3.80469  4.34274  2.36355  3.36846  1.79296  1.45569  3.88063  4.26555  3.75324  3.57830  3.28378  3.24383  2.36495  5.02707  3.78505     70 m - - -
        2.68618  4.42225  2.77519  2.73123  3.46354  2.40513  3.72494  3.29354  2.67741  2.69355  4.24690  2.90347  2.73739  3.18146  2.89801  2.37887  2.77519  2.98518  4.58477  3.61503
        0.03268  3.83303  4.55537  0.61958  0.77255  0.48576  0.95510
 71     2.91965  5.02079  2.47306  1.08285  4.36227  3.24954  3.83381  3.80837  2.70946  3.43216  4.40865  2.91254  3.85246  3.05076  3.11366  2.90651  3.22382  3.49656  5.54134  4.26436     71 e - - -
        2.68618  4.42225  2.77519  2.73123  3.46354  2.40513  3.72494  3.29354  2.67741  2.69355  4.24690  2.90347  2.73739  3.18146  2.89801  2.37887  2.77519  2.98518  4.58477  3.61503
        0.03268  3.83303  4.55537  0.61958  0.77255  0.48576  0.95510
 72     1.48669  4.09375  3.30062  3.03535  4.12165  2.88470  4.11123  3.43938  3.08293  3.21291  4.08357  3.21051  3.62477  3.39368  3.38530  1.75014  2.64464  2.96699  5.49764  4.26622     72 a - - -
        2.68618  4.42225  2.77519  2.73123  3.46354  2.40513  3.72494  3.29354  2.67741  2.69355  4.24690  2.90347  2.73739  3.18146  2.89801  2.37887  2.77519  2.98518  4.58477  3.61503
        0.03268  3.83303  4.55537  0.61958  0.77255  0.48576  0.95510
 73     2.08693  4.24267  3.94909  3.45118  3.52859  3.67786  4.31021  1.89945  3.35740  2.25787  3.40707  3.72692  4.16014  3.67115  3.64189  3.03716  2.96856  1.58050  5.21739  3.99659     73 v - - -
        2.68618  4.42225  2.77519  2.73123  3.46354  2.40513  3.72494  3.29354  2.67741  2.69355  4.24690  2.90347  2.73739  3.18146  2.89801  2.37887  2.77519  2.98518  4.58477  3.61503
        0.03268  3.83303  4.55537  0.61958  0.77255  0.48576  0.95510
 74     2.93703  4.89791  3.11032  2.74002  4.27531  3.43004  3.73906  3.70226  1.09362  3.27630  4.24050  3.16601  3.93369  2.93324  2.42654  2.98198  3.19357  3.41249  5.34669  4.15401     74 k - - -
        2.68618  4.42225  2.77519  2.73123  3.46354  2.40513  3.72494  3.29354  2.67741  2.69355  4.24690  2.90347  2.73739  3.18146  2.89801  2.37887  2.77519  2.98518  4.58477  3.61503
        0.03268  3.83303  4.55537  0.61958  0.77255  0.48576  0.95510
 75     2.87759  5.33786  1.33387  1.86786  4.63127  3.15364  3.72174  4.12359  2.74483  3.67936  4.56019  2.64720  3.78205  2.90177  3.31840  2.77979  3.17457  3.72921  5.83535  4.36780     75 d - - -
        2.68618  4.42225  2.77519  2.73123  3.46354  2.40513  3.72494  3.29354  2.67741  2.69355  4.24690  2.90347  2.73739  3.18146  2.89801  2.37887  2.77519  2.98518  4.58477  3.61503
        0.03268  3.83303  4.55537  0.61958  0.77255  0.48576  0.95510
 76     2.91965  5.02079  2.47306  1.08285  4.36227  3.24954  3.83381  3.80837  2.70946  3.43216  4.40865  2.91254  3.85246  3.05076  3.11366  2.90651  3.22382  3.49656  5.54134  4.26436     76 e - - -
        2.68618  4.42225  2.77519  2.73123  3.46354  2.40513  3.72494  3.29354  2.67741  2.69355  4.24690  2.90347  2.73739  3.18146  2.89801  2.37887  2.77519  2.98518  4.58477  3.61503
        0.03268  3.83303  4.55537  0.61958  0.77255  0.48576  0.95510
 77     2.96214  4.80276  3.44889  2.96907  4.14736  3.45685  3.79770  3.68689  2.22438  3.21005  4.21188  3.33151  3.96192  3.02053  1.04043  3.04858  3.23116  3.41270  5.24678  4.08393     77 r - - -
        2.68618  4.42225  2.77519  2.73123  3.46354  2.40513  3.72494  3.29354  2.67741  2.69355  4.24690  2.90347  2.73739  3.18146  2.89801  2.37887  2.77519  2.98518  4.58477  3.61503
        0.03268  3.83303  4.55537  0.61958  0.77255  0.48576  0.95510
 78     2.85084  4.83409  2.87832  2.63320  3.99721  3.34074  3.79090  3.62260  2.45691  3.12880  4.15852  3.09230  3.89411  1.30591  2.74155  2.90061  3.14819  3.35495  5.27660  3.96796     78 q - - -
        2.68618  4.42225  2.77519  2.73123  3.46354  2.40513  3.72494  3.29354  2.67741  2.69355  4.24690  2.90347  2.73739  3.18146  2.89801  2.37887  2.77519  2.98518  4.58477  3.61503
        0.03268  3.83303  4.55537  0.61958  0.77255  0.48576  0.95510
 79     3.27048  4.64138  4.25823  3.87451  1.24758  4.10742  3.51349  2.92823  3.74392  2.30076  3.56638  3.86566  4.44830  3.85385  3.87583  3.47123  3.50746  2.87164  3.72551  1.81967     79 f - - -
        2.68618  4.42225  2.77519  2.73123  3.46354  2.40513  3.72494  3.29354  2.67741  2.69355  4.24690  2.90347  2.73739  3.18146  2.89801  2.37887  2.77519  2.98518  4.58477  3.61503
        0.03268  3.83303  4.55537  0.61958  0.77255  0.48576  0.95510
 80     2.61765  4.39623  3.28327  3.17805  4.31966  0.79266  4.30013  3.91025  3.35388  3.56921  4.54515  3.44774  3.78610  3.68127  3.59365  2.78855  3.09584  3.44624  5.42460  4.42045     80 g - - -
        2.68618  4.42225  2.77519  2.73123  3.46354  2.40513  3.72494  3.29354  2.67741  2.69355  4.24690  2.90347  2.73739  3.18146  2.89801  2.37887  2.77519  2.98518  4.58477  3.61503
        0.03268  3.83303  4.55537  0.61958  0.77255  0.48576  0.95510
 81     2.22167  4.14590  3.27252  2.97589  4.04732  2.94922  4.04682  3.34732  2.97338  3.12609  4.01188  3.19785  3.65932  3.32126  3.28022  1.93709  1.64328  2.92309  5.42707  4.18333     81 t - - -
        2.68618  4.42225  2.77519  2.73123  3.46354  2.40513  3.72494  3.29354  2.67741  2.69355  4.24690  2.90347  2.73739  3.18146  2.89801  2.37887  2.77519  2.98518  4.58477  3.61503
        0.03268  3.83303  4.55537  0.61958  0.77255  0.48576  0.95510
 82     3.11165  4.58599  4.12585  3.76620  3.12182  3.93147  4.43434  2.32453  3.53431  0.92536  3.15834  4.04543  4.37407  3.91210  3.71656  3.49871  3.40796  2.35149  4.98612  3.70011     82 l - - -
        2.68618  4.42225  2.77519  2.73123  3.46354  2.40513  3.72494  3.29354  2.67741  2.69355  4.24690  2.90347  2.73739  3.18146  2.89801  2.37887  2.77519  2.98518  4.58477  3.61503
        0.03268  3.83303  4.55537  0.61958  0.77255  0.48576  0.95510
 83     2.79290  4.56425  3.36875  2.93033  3.36969  3.60990  3.86671  2.76319  2.65521  1.59485  3.41131  3.33960  4.03536  2.49749  2.93346  2.97542  3.04375  2.67550  4.97460  3.65998     83 l - - -
        2.68618  4.42225  2.77519  2.73123  3.46354  2.40513  3.72494  3.29354  2.67741  2.69355  4.24690  2.90347  2.73739  3.18146  2.89801  2.37887  2.77519  2.98518  4.58477  3.61503
        0.03268  3.83303  4.55537  0.61958  0.77255  0.48576  0.95510
 84     2.94831  5.06869  0.97632  2.31258  4.44069  3.18175  3.89799  4.02012  2.97493  3.63580  4.61368  2.86669  3.83184  3.13755  3.48525  2.91560  3.28628  3.66374  5.61691  4.32095     84 d - - -
        2.68618  4.42225  2.77519  2.73123  3.46354  2.40513  3.72494  3.29354  2.67741  2.69355  4.24690  2.90347  2.73739  3.18146  2.89801  2.37887  2.77519  2.98518  4.58477  3.61503
        0.03268  3.83303  4.55537  0.61958  0.77255  0.48576  0.95510
 85     1.96982  4.12346  3.32254  3.01105  4.02420  1.73990  4.07149  3.25379  3.03469  3.07299  3.97043  3.22046  3.66136  3.35128  3.34888  2.39618  2.66644  2.51617  5.42236  4.18978     85 g - - -
        2.68618  4.42225  2.77519  2.73123  3.46354  2.40513  3.72494  3.29354  2.67741  2.69355  4.24690  2.90347  2.73739  3.18146  2.89801  2.37887  2.77519  2.98518  4.58477  3.61503
        0.03268  3.83303  4.55537  0.61958  0.77255  0.48576  0.95510
```

86	3.11165	4.58599	4.12585	3.76620	3.12182	3.93147	4.43434	2.32453	3.53431	0.92536	3.15834	4.04543	4.37407	3.91210	3.71656	3.49871	3.40796	2.35149	4.98612	3.70011	86 l - - -
	2.68618	4.42225	2.77519	2.73123	3.46354	2.40513	3.72494	3.29354	2.67741	2.69355	4.24690	2.90347	2.73739	3.18146	2.89801	2.37887	2.77519	2.98518	4.58477	3.61503	
	0.03268	3.83303	4.55537	0.61958	0.77255	0.48576	0.95510														
87	2.50040	4.52434	2.41981	2.52216	4.06778	3.14758	3.83050	3.34746	2.74622	3.11363	4.01543	2.97396	3.75466	3.04923	3.16253	2.59784	1.68565	3.01045	5.41310	4.09919	87 t - - -
	2.68618	4.42225	2.77519	2.73123	3.46354	2.40513	3.72494	3.29354	2.67741	2.69355	4.24690	2.90347	2.73739	3.18146	2.89801	2.37887	2.77519	2.98518	4.58477	3.61503	
	0.03268	3.83303	4.55537	0.61958	0.77255	0.48576	0.95510														
88	2.96214	4.80276	3.44889	2.96907	4.14736	3.45685	3.79770	3.68689	2.22438	3.21005	4.21188	3.33151	3.96192	3.02053	1.04043	3.04858	3.23116	3.41270	5.24678	4.08393	88 r - - -
	2.68618	4.42225	2.77519	2.73123	3.46354	2.40513	3.72494	3.29354	2.67741	2.69355	4.24690	2.90347	2.73739	3.18146	2.89801	2.37887	2.77519	2.98518	4.58477	3.61503	
	0.03268	3.83303	4.55537	0.61958	0.77255	0.48576	0.95510														
89	3.01681	4.49585	4.27346	3.80340	3.12930	4.06697	4.50357	2.10456	3.59520	1.12338	3.04442	4.08084	4.44056	3.91542	3.80393	3.46258	3.29608	1.94912	5.07659	3.83318	89 l - - -
	2.68618	4.42225	2.77519	2.73123	3.46354	2.40513	3.72494	3.29354	2.67741	2.69355	4.24690	2.90347	2.73739	3.18146	2.89801	2.37887	2.77519	2.98518	4.58477	3.61503	
	0.03268	3.83303	4.55537	0.61958	0.77255	0.48576	0.95510														
90	2.75458	5.18504	1.91320	1.98070	4.51468	2.32563	3.66529	3.99637	2.61474	3.53174	4.36239	2.38861	3.75698	2.82649	3.16184	2.68853	3.03673	3.59481	5.71857	4.27254	90 d - - -
	2.68618	4.42225	2.77519	2.73123	3.46354	2.40513	3.72494	3.29354	2.67741	2.69355	4.24690	2.90347	2.73739	3.18146	2.89801	2.37887	2.77519	2.98518	4.58477	3.61503	
	0.03268	3.83303	4.55537	0.61958	0.77255	0.48576	0.95510														
91	1.07216	4.17353	3.42348	3.21371	4.01396	2.99897	4.24029	3.13365	3.22896	3.01700	4.05375	3.37300	3.73453	3.57391	3.48180	2.52446	2.79912	2.79493	5.44509	4.24110	91 a - - -
	2.68618	4.42225	2.77519	2.73123	3.46354	2.40513	3.72494	3.29354	2.67741	2.69355	4.24690	2.90347	2.73739	3.18146	2.89801	2.37887	2.77519	2.98518	4.58477	3.61503	
	0.03268	3.83303	4.55537	0.61958	0.77255	0.48576	0.95510														
92	1.99808	4.17612	3.12803	2.96128	4.23292	1.37572	4.13449	3.60004	3.11575	3.34657	4.23431	3.18905	3.64798	3.42094	3.41914	2.41362	2.73088	3.10516	5.55818	4.34684	92 g - - -
	2.68618	4.42225	2.77519	2.73123	3.46354	2.40513	3.72494	3.29354	2.67741	2.69355	4.24690	2.90347	2.73739	3.18146	2.89801	2.37887	2.77519	2.98518	4.58477	3.61503	
	0.03268	3.83303	4.55537	0.61958	0.77255	0.48576	0.95510														
93	2.51253	4.49394	2.91762	2.63773	3.85772	3.19568	3.82615	3.22888	2.68333	3.01349	3.95911	1.76139	3.79049	3.07233	3.04322	2.62804	2.86839	2.53450	5.26400	3.89618	93 n - - -
	2.68618	4.42225	2.77519	2.73123	3.46354	2.40513	3.72494	3.29354	2.67741	2.69355	4.24690	2.90347	2.73739	3.18146	2.89801	2.37887	2.77519	2.98518	4.58477	3.61503	
	0.03268	3.83303	4.55537	0.61958	0.77255	0.48576	0.95510														
94	2.93342	5.02244	3.30360	2.70065	4.43096	3.55731	3.56515	3.79670	1.45615	3.27991	4.15794	3.11308	3.94750	2.70993	1.71111	2.94347	3.11800	3.48664	5.35577	4.16695	94 k - - -
	2.68618	4.42225	2.77519	2.73123	3.46354	2.40513	3.72494	3.29354	2.67741	2.69355	4.24690	2.90347	2.73739	3.18146	2.89801	2.37887	2.77519	2.98518	4.58477	3.61503	
	0.03268	3.83303	4.55537	0.61958	0.77255	0.48576	0.95510														
95	2.85705	4.33858	4.26805	3.82469	3.45243	3.94181	4.60904	1.81360	3.67074	2.06189	3.35133	4.05620	4.40129	4.01087	3.90428	3.35191	3.17119	1.15614	5.31647	4.04977	95 v - - -
	2.68618	4.42225	2.77519	2.73123	3.46354	2.40513	3.72494	3.29354	2.67741	2.69355	4.24690	2.90347	2.73739	3.18146	2.89801	2.37887	2.77519	2.98518	4.58477	3.61503	
	0.03268	3.83303	4.55537	0.61958	0.77255	0.48576	0.95510														
96	2.95371	4.77708	3.08911	2.84570	3.25855	3.41543	1.22850	3.65045	2.68990	3.14913	4.18951	3.25638	3.96612	3.21160	2.95766	3.02494	3.25600	3.38639	4.72403	3.21065	96 h - - -
	2.68618	4.42225	2.77519	2.73123	3.46354	2.40513	3.72494	3.29354	2.67741	2.69355	4.24690	2.90347	2.73739	3.18146	2.89801	2.37887	2.77519	2.98518	4.58477	3.61503	
	0.03268	3.83303	4.55537	0.61958	0.77255	0.48576	0.95510														
97	2.70125	4.46443	3.30428	3.13815	4.13555	3.15421	4.21835	3.67141	3.19219	3.30231	4.35093	3.44851	0.90433	3.58220	3.44165	2.86766	3.13986	3.32122	5.33234	4.25589	97 p - - -
	2.68618	4.42225	2.77519	2.73123	3.46354	2.40513	3.72494	3.29354	2.67741	2.69355	4.24690	2.90347	2.73739	3.18146	2.89801	2.37887	2.77519	2.98518	4.58477	3.61503	
	0.03268	3.83303	4.55537	0.61958	0.77255	0.48576	0.95510														
98	2.93734	5.02052	3.31837	2.70911	4.42950	3.56151	3.56518	3.79409	1.52573	3.27735	4.15650	3.11870	3.95039	2.71037	1.61828	2.94861	3.12094	3.48542	5.35287	4.16659	98 k - - -
	2.68618	4.42225	2.77519	2.73123	3.46354	2.40513	3.72494	3.29354	2.67741	2.69355	4.24690	2.90347	2.73739	3.18146	2.89801	2.37887	2.77519	2.98518	4.58477	3.61503	
	0.03268	3.83303	4.55537	0.61958	0.77255	0.48576	0.95510														
99	3.27202	4.66041	4.03210	3.72890	2.62865	3.68698	3.85349	3.29453	3.45219	2.69596	3.93010	3.88606	4.20159	3.84191	3.58729	3.47606	3.57058	3.18378	0.96876	2.63291	99 w - - -
	2.68618	4.42225	2.77519	2.73123	3.46354	2.40513	3.72494	3.29354	2.67741	2.69355	4.24690	2.90347	2.73739	3.18146	2.89801	2.37887	2.77519	2.98518	4.58477	3.61503	
	0.03268	3.83303	4.55537	0.61958	0.77255	0.48576	0.95510														
100	2.44835	4.48600	2.69530	2.54493	4.25377	1.70151	3.89389	3.77305	2.81346	3.40757	4.26503	2.21704	3.70580	3.12346	3.20321	2.54342	2.86170	3.30666	5.53410	4.22287	100 g - - -
	2.68618	4.42225	2.77519	2.73123	3.46354	2.40513	3.72494	3.29354	2.67741	2.69355	4.24690	2.90347	2.73739	3.18146	2.89801	2.37887	2.77519	2.98518	4.58477	3.61503	
	0.03268	3.83303	4.55537	0.61958	0.77255	0.48576	0.95510														
101	2.91965	5.02079	2.47306	1.08285	4.36227	3.24954	3.83381	3.80837	2.70946	3.43216	4.40865	2.91254	3.85246	3.05076	3.11366	2.90651	3.22382	3.49656	5.54134	4.26436	101 e - - -
	2.68618	4.42225	2.77519	2.73123	3.46354	2.40513	3.72494	3.29354	2.67741	2.69355	4.24690	2.90347	2.73739	3.18146	2.89801	2.37887	2.77519	2.98518	4.58477	3.61503	
	0.03268	3.83303	4.55537	0.61958	0.77255	0.48576	0.95510														
102	2.40030	4.22119	3.42470	2.97866	3.69049	3.23563	3.95654	2.47296	2.89012	2.68062	3.64330	3.28380	3.81529	3.25195	3.20956	2.40017	1.85254	2.51065	5.15711	3.90764	102 t - - -
	2.68618	4.42225	2.77519	2.73123	3.46354	2.40513	3.72494	3.29354	2.67741	2.69355	4.24690	2.90347	2.73739	3.18146	2.89801	2.37887	2.77519	2.98518	4.58477	3.61503	
	0.03268	3.83303	4.55537	0.61958	0.77255	0.48576	0.95510														
103	2.93904	4.49431	4.00741	3.59844	3.19183	3.80469	4.34274	2.36355	3.36846	1.79296	1.45569	3.88063	4.26555	3.75324	3.57830	3.28378	3.24383	2.36495	5.02707	3.78505	103 m - - -
	2.68618	4.42225	2.77519	2.73123	3.46354	2.40513	3.72494	3.29354	2.67741	2.69355	4.24690	2.90347	2.73739	3.18146	2.89801	2.37887	2.77519	2.98518	4.58477	3.61503	
	0.03268	3.83303	4.55537	0.61958	0.77255	0.48576	0.95510														
104	2.93703	4.89791	3.11032	2.74002	4.27531	3.43004	3.73906	3.70226	1.09362	3.27630	4.24050	3.16601	3.93369	2.93324	2.42654	2.98198	3.19357	3.41249	5.34669	4.15401	104 k - - -
	2.68618	4.42225	2.77519	2.73123	3.46354	2.40513	3.72494	3.29354	2.67741	2.69355	4.24690	2.90347	2.73739	3.18146	2.89801	2.37887	2.77519	2.98518	4.58477	3.61503	
	0.03268	3.83303	4.55537	0.61958	0.77255	0.48576	0.95510														
105	2.97780	4.37231	4.43474	3.91350	3.36165	4.20592	4.66164	1.71003	3.77685	1.75649	3.20838	4.19305	4.53346	4.06599	4.00971	3.56199	3.23892	1.28099	5.27745	4.06301	105 v - - -
	2.68618	4.42225	2.77519	2.73123	3.46354	2.40513	3.72494	3.29354	2.67741	2.69355	4.24690	2.90347	2.73739	3.18146	2.89801	2.37887	2.77519	2.98518	4.58477	3.61503	
	0.03268	3.83303	4.55537	0.61958	0.77255	0.48576	0.95510														
106	2.60965	4.29319	4.29626	3.79375	3.48040	4.01563	4.58319	1.45186	3.68340	2.08163	3.32040	4.05442	4.41403	3.98112	3.93024	3.37890	3.13111	1.46786	5.30718	4.09963	106 i - - -
	2.68618	4.42225	2.77519	2.73123	3.46354	2.40513	3.72494	3.29354	2.67741	2.69355	4.24690	2.90347	2.73739	3.18146	2.89801	2.37887	2.77519	2.98518	4.58477	3.61503	
	0.03268	3.83303	4.55537	0.61958	0.77255	0.48576	0.95510														
107	1.94020	4.12787	3.22040	2.98469	4.14082	2.89101	4.09593	3.49170	3.05042	3.25036	4.12838	3.18609	3.63166	3.37355	3.35554	1.36516	2.66799	3.01268	5.51269	4.26187	107 s - - -
	2.68618	4.42225	2.77519	2.73123	3.46354	2.40513	3.72494	3.29354	2.67741	2.69355	4.24690	2.90347	2.73739	3.18146	2.89801	2.37887	2.77519	2.98518	4.58477	3.61503	
	0.03268	3.83303	4.55537	0.61958	0.77255	0.48576	0.95510														
108	2.69182	4.68600	2.68850	2.57033	4.04344	3.15836	3.89101	3.74595	2.79175	3.39751	4.35350	1.22987	3.80860	3.16424	3.14460	2.76087	3.07272	3.37215	5.35398	3.99503	108 n - - -

```
     2.68618  4.42225  2.77519  2.73123  3.46354  2.40513  3.72494  3.29354  2.67741  2.69355  4.24690  2.90347  2.73739  3.18146  2.89801  2.37887  2.77519  2.98518  4.58477  3.61503
     0.03268  3.83303  4.55537  0.61958  0.77255  0.48576  0.95510
109  3.20912  4.62273  4.18444  3.89199  1.07857  3.90702  3.86137  2.73830  3.80015  2.11778  3.47472  3.97513  4.36694  3.97918  3.92960  3.48072  3.51047  2.72599  4.12320  2.49559    109 f - - -
     2.68618  4.42225  2.77519  2.73123  3.46354  2.40513  3.72494  3.29354  2.67741  2.69355  4.24690  2.90347  2.73739  3.18146  2.89801  2.37887  2.77519  2.98518  4.58477  3.61503
     0.03268  3.83303  4.55537  0.61958  0.77255  0.48576  0.95510
110  3.11165  4.58599  4.12585  3.76620  3.12182  3.93147  4.43434  2.32453  3.53431  0.92536  3.15834  4.04543  4.37407  3.91210  3.71656  3.49871  3.40796  2.35149  4.98612  3.70011    110 l - - -
     2.68618  4.42225  2.77519  2.73123  3.46354  2.40513  3.72494  3.29354  2.67741  2.69355  4.24690  2.90347  2.73739  3.18146  2.89801  2.37887  2.77519  2.98518  4.58477  3.61503
     0.03268  3.83303  4.55537  0.61958  0.77255  0.48576  0.95510
111  2.91965  5.02079  2.47306  1.08285  4.36227  3.24954  3.83381  3.80837  2.70946  3.43216  4.40865  2.91254  3.85246  3.05076  3.11366  2.90651  3.22382  3.49656  5.54134  4.26436    111 e - - -
     2.68618  4.42225  2.77519  2.73123  3.46354  2.40513  3.72494  3.29354  2.67741  2.69355  4.24690  2.90347  2.73739  3.18146  2.89801  2.37887  2.77519  2.98518  4.58477  3.61503
     0.03268  3.83303  4.55537  0.61958  0.77255  0.48576  0.95510
112  2.59084  4.23004  3.91003  3.51121  3.61948  3.51913  4.38316  2.12280  3.38997  2.34517  3.51940  3.71710  4.09862  3.74298  3.64338  2.94156  2.56552  1.30673  5.32127  4.08472    112 v - - -
     2.68618  4.42225  2.77519  2.73123  3.46354  2.40513  3.72494  3.29354  2.67741  2.69355  4.24690  2.90347  2.73739  3.18146  2.89801  2.37887  2.77519  2.98518  4.58477  3.61503
     0.03268  3.83303  4.55537  0.61958  0.77255  0.48576  0.95510
113  2.61765  4.39623  3.28327  3.17805  4.31966  0.79266  4.30013  3.91025  3.35388  3.56921  4.54515  3.44774  3.78610  3.68127  3.59365  2.78855  3.09584  3.44624  5.42460  4.42045    113 g - - -
     2.68618  4.42225  2.77519  2.73123  3.46354  2.40513  3.72494  3.29354  2.67741  2.69355  4.24690  2.90347  2.73739  3.18146  2.89801  2.37887  2.77519  2.98518  4.58477  3.61503
     0.03268  3.83303  4.55537  0.61958  0.77255  0.48576  0.95510
114  2.91965  5.02079  2.47306  1.08285  4.36227  3.24954  3.83381  3.80837  2.70946  3.43216  4.40865  2.91254  3.85246  3.05076  3.11366  2.90651  3.22382  3.49656  5.54134  4.26436    114 e - - -
     2.68618  4.42225  2.77519  2.73123  3.46354  2.40513  3.72494  3.29354  2.67741  2.69355  4.24690  2.90347  2.73739  3.18146  2.89801  2.37887  2.77519  2.98518  4.58477  3.61503
     0.03268  3.83303  4.55537  0.61958  0.77255  0.48576  0.95510
115  3.19134  4.69630  3.78054  3.50310  2.29611  3.78460  3.60201  3.20618  3.36176  2.67014  3.88549  3.67575  4.25966  3.67727  3.55331  3.33175  3.47801  3.07495  3.93816  1.03399    115 y - - -
     2.68618  4.42225  2.77519  2.73123  3.46354  2.40513  3.72494  3.29354  2.67741  2.69355  4.24690  2.90347  2.73739  3.18146  2.89801  2.37887  2.77519  2.98518  4.58477  3.61503
     0.03268  3.83303  4.55537  0.61958  0.77255  0.48576  0.95510
116  2.54104  4.61752  2.59982  2.45886  4.25898  2.56739  3.84224  3.80359  2.74127  3.42706  4.29359  1.55362  3.73528  3.06247  3.14212  2.60772  2.92747  3.36384  5.52727  4.18902    116 n - - -
     2.68618  4.42225  2.77519  2.73123  3.46354  2.40513  3.72494  3.29354  2.67741  2.69355  4.24690  2.90347  2.73739  3.18146  2.89801  2.37887  2.77519  2.98518  4.58477  3.61503
     0.03268  3.83303  4.55537  0.61958  0.77255  0.48576  0.95510
117  1.07216  4.17353  3.42348  3.21371  4.01396  2.99897  4.24029  3.13365  3.22896  3.01700  4.05375  3.37300  3.73453  3.57391  3.48180  2.52446  2.79912  2.79493  5.44509  4.24110    117 a - - -
     2.68618  4.42225  2.77519  2.73123  3.46354  2.40513  3.72494  3.29354  2.67741  2.69355  4.24690  2.90347  2.73739  3.18146  2.89801  2.37887  2.77519  2.98518  4.58477  3.61503
     0.03268  3.83303  4.55537  0.61958  0.77255  0.48576  0.95510
118  3.00047  4.43103  4.27833  3.89317  3.34832  4.02286  4.62907  1.12816  3.72049  1.94320  3.29060  4.14408  4.45846  4.07325  3.92162  3.51489  3.30059  1.84674  5.23051  3.96495    118 i - - -
     2.68618  4.42225  2.77519  2.73123  3.46354  2.40513  3.72494  3.29354  2.67741  2.69355  4.24690  2.90347  2.73739  3.18146  2.89801  2.37887  2.77519  2.98518  4.58477  3.61503
     0.03268  3.83303  4.55537  0.61958  0.77255  0.48576  0.95510
119  1.21371  4.10221  3.33541  3.10543  4.06531  2.91295  4.16078  3.27143  3.13474  3.13523  4.06620  3.26123  3.65879  3.46519  3.41087  2.26255  2.68247  2.85776  5.48330  4.24147    119 a - - -
     2.68618  4.42225  2.77519  2.73123  3.46354  2.40513  3.72494  3.29354  2.67741  2.69355  4.24690  2.90347  2.73739  3.18146  2.89801  2.37887  2.77519  2.98518  4.58477  3.61503
     0.03268  3.83303  4.55537  0.61958  0.77255  0.48576  0.95510
120  1.30094  4.10914  3.27132  3.04048  4.16230  2.27516  4.13820  3.47324  3.12634  3.24982  4.12575  3.22112  3.63246  3.42734  3.42464  2.36978  2.67156  2.99715  5.51804  4.31111    120 a - - -
     2.68618  4.42225  2.77519  2.73123  3.46354  2.40513  3.72494  3.29354  2.67741  2.69355  4.24690  2.90347  2.73739  3.18146  2.89801  2.37887  2.77519  2.98518  4.58477  3.61503
     0.03268  3.83303  4.55537  0.61958  0.77255  0.48576  0.95510
121  2.20774  4.14600  3.24421  2.95864  4.09598  2.92690  4.04945  3.43061  2.97673  3.18497  4.05914  3.18135  3.64493  3.31729  3.28939  1.51784  2.09157  2.97824  5.46315  4.21655    121 s - - -
     2.68618  4.42225  2.77519  2.73123  3.46354  2.40513  3.72494  3.29354  2.67741  2.69355  4.24690  2.90347  2.73739  3.18146  2.89801  2.37887  2.77519  2.98518  4.58477  3.61503
     0.03268  3.83303  4.55537  0.61958  0.77255  0.48576  0.95510
122  1.36072  4.11112  3.25844  3.02671  4.17114  2.14087  4.13177  3.49308  3.11931  3.25995  4.13075  3.21239  3.62929  3.41746  3.42206  2.36551  2.66879  3.01018  5.52343  4.31500    122 a - - -
     2.68618  4.42225  2.77519  2.73123  3.46354  2.40513  3.72494  3.29354  2.67741  2.69355  4.24690  2.90347  2.73739  3.18146  2.89801  2.37887  2.77519  2.98518  4.58477  3.61503
     0.03268  3.83303  4.55537  0.61958  0.77255  0.48576  0.95510
123  3.02068  4.51129  4.21262  3.69876  3.07702  4.07098  4.40123  2.20589  3.46987  1.47853  1.75611  4.00731  4.40350  3.79721  3.68555  3.43320  3.27475  2.24458  5.00500  3.79040    123 l - - -
     2.68618  4.42225  2.77519  2.73123  3.46354  2.40513  3.72494  3.29354  2.67741  2.69355  4.24690  2.90347  2.73739  3.18146  2.89801  2.37887  2.77519  2.98518  4.58477  3.61503
     0.03268  3.83303  4.55537  0.61958  0.77255  0.48576  0.95510
124  3.11165  4.58599  4.12585  3.76620  3.12182  3.93147  4.43434  2.32453  3.53431  0.92536  3.15834  4.04543  4.37407  3.91210  3.71656  3.49871  3.40796  2.35149  4.98612  3.70011    124 l - - -
     2.68618  4.42225  2.77519  2.73123  3.46354  2.40513  3.72494  3.29354  2.67741  2.69355  4.24690  2.90347  2.73739  3.18146  2.89801  2.37887  2.77519  2.98518  4.58477  3.61503
     0.03268  3.83303  4.55537  0.61958  0.77255  0.48576  0.95510
125  3.27202  4.66041  4.03210  3.72890  2.62865  3.68698  3.85349  3.29453  3.45219  2.69596  3.93010  3.88606  4.20159  3.84191  3.58729  3.47606  3.57058  3.18378  0.96876  2.63291    125 w - - -
     2.68618  4.42225  2.77519  2.73123  3.46354  2.40513  3.72494  3.29354  2.67741  2.69355  4.24690  2.90347  2.73739  3.18146  2.89801  2.37887  2.77519  2.98518  4.58477  3.61503
     0.03268  3.83303  4.55537  0.61958  0.77255  0.48576  0.95510
126  2.94831  5.06869  0.97632  2.31258  4.44069  3.18175  3.89799  4.02012  2.97493  3.63580  4.61368  2.86669  3.83184  3.13755  3.48525  2.91560  3.28628  3.66374  5.61691  4.32095    126 d - - -
     2.68618  4.42225  2.77519  2.73123  3.46354  2.40513  3.72494  3.29354  2.67741  2.69355  4.24690  2.90347  2.73739  3.18146  2.89801  2.37887  2.77519  2.98518  4.58477  3.61503
     0.03268  3.83303  4.55537  0.61958  0.77255  0.48576  0.95510
127  2.20851  4.14891  3.22021  2.96018  4.11307  2.91715  4.06271  3.46020  2.99058  3.21396  4.09390  3.17973  3.64367  3.33363  3.29812  1.39338  2.31025  2.99928  5.48211  4.22845    127 s - - -
     2.68618  4.42225  2.77519  2.73123  3.46354  2.40513  3.72494  3.29354  2.67741  2.69355  4.24690  2.90347  2.73739  3.18146  2.89801  2.37887  2.77519  2.98518  4.58477  3.61503
     0.03268  3.83303  4.55537  0.61958  0.77255  0.48576  0.95510
128  1.07216  4.17353  3.42348  3.21371  4.01396  2.99897  4.24029  3.13365  3.22896  3.01700  4.05375  3.37300  3.73453  3.57391  3.48180  2.52446  2.79912  2.79493  5.44509  4.24110    128 a - - -
     2.68618  4.42225  2.77519  2.73123  3.46354  2.40513  3.72494  3.29354  2.67741  2.69355  4.24690  2.90347  2.73739  3.18146  2.89801  2.37887  2.77519  2.98518  4.58477  3.61503
     0.03268  3.83303  4.55537  0.61958  0.77255  0.48576  0.95510
129  2.50245  4.53350  2.92109  2.55749  4.01433  3.21799  3.73307  3.39904  2.50332  3.05082  3.91689  2.99748  3.76313  2.35783  2.86333  2.41645  2.05907  3.05775  5.31070  4.01862    129 t - - -
     2.68618  4.42225  2.77519  2.73123  3.46354  2.40513  3.72494  3.29354  2.67741  2.69355  4.24690  2.90347  2.73739  3.18146  2.89801  2.37887  2.77519  2.98518  4.58477  3.61503
     0.03268  3.83303  4.55537  0.61958  0.77255  0.48576  0.95510
130  1.28840  4.08974  3.32133  3.07452  4.09757  2.89173  4.13766  3.36654  3.11400  3.18238  4.07592  3.23481  3.63658  3.43215  3.40172  2.07179  2.65674  2.91807  5.49184  4.25860    130 a - - -
     2.68618  4.42225  2.77519  2.73123  3.46354  2.40513  3.72494  3.29354  2.67741  2.69355  4.24690  2.90347  2.73739  3.18146  2.89801  2.37887  2.77519  2.98518  4.58477  3.61503
```

```
        0.03268  3.83303  4.55537  0.61958  0.77255  0.48576  0.95510
    131   1.43932  4.17497  3.23983  2.95111  4.06415  2.96621  4.04921  3.35124  2.98775  3.11025  4.01510  3.19545  2.52182  3.31897  3.30712  2.42398  2.70139  2.93904  5.43729  4.20787    131 a - - -
          2.68618  4.42225  2.77519  2.73123  3.46354  2.40513  3.72494  3.29354  2.67741  2.69355  4.24690  2.90347  2.73739  3.18146  2.89801  2.37887  2.77519  2.98518  4.58477  3.61503
          0.03268  3.83303  4.55537  0.61958  0.77255  0.48576  0.95510
    132   2.91965  5.02079  2.47306  1.08285  4.36227  3.24954  3.83381  3.80837  2.70946  3.43216  4.40865  2.91254  3.85246  3.05076  3.11366  2.90651  3.22382  3.49656  5.54134  4.26436    132 e - - -
          2.68618  4.42225  2.77519  2.73123  3.46354  2.40513  3.72494  3.29354  2.67741  2.69355  4.24690  2.90347  2.73739  3.18146  2.89801  2.37887  2.77519  2.98518  4.58477  3.61503
          0.03268  3.83303  4.55537  0.61958  0.77255  0.48576  0.95510
    133   2.85084  4.83409  2.87832  2.63320  3.99721  3.34074  3.79090  3.62260  2.45691  3.12880  4.15852  3.09230  3.89411  1.30591  2.74155  2.90061  3.14819  3.35495  5.27660  3.96796    133 q - - -
          2.68618  4.42225  2.77519  2.73123  3.46354  2.40513  3.72494  3.29354  2.67741  2.69355  4.24690  2.90347  2.73739  3.18146  2.89801  2.37887  2.77519  2.98518  4.58477  3.61503
          0.03268  3.83303  4.55537  0.61958  0.77255  0.48576  0.95510
    134   2.91529  4.99693  3.22470  2.67995  4.39156  3.52540  3.58444  3.77191  1.32458  3.27138  4.15738  3.10007  3.93724  2.73544  1.96900  2.92975  3.11342  3.46273  5.35654  4.15483    134 k - - -
          2.68618  4.42225  2.77519  2.73123  3.46354  2.40513  3.72494  3.29354  2.67741  2.69355  4.24690  2.90347  2.73739  3.18146  2.89801  2.37887  2.77519  2.98518  4.58477  3.61503
          0.03268  3.83303  4.55537  0.61958  0.77255  0.48576  0.95510
    135   2.69182  4.68600  2.68850  2.57033  4.04344  3.15836  3.89101  3.74595  2.79175  3.39751  4.35350  1.22987  3.80860  3.16424  3.14460  2.76087  3.07272  3.37215  5.35398  3.99503    135 n - - -
          2.68618  4.42225  2.77519  2.73123  3.46354  2.40513  3.72494  3.29354  2.67741  2.69355  4.24690  2.90347  2.73739  3.18146  2.89801  2.37887  2.77519  2.98518  4.58477  3.61503
          0.03268  3.83303  4.55537  0.61958  0.77255  0.48576  0.95510
    136   2.61765  4.39623  3.28327  3.17805  4.31966  0.79266  4.30013  3.91025  3.35388  3.56921  4.54515  3.44774  3.78610  3.68127  3.59365  2.78855  3.09584  3.44624  5.42460  4.42045    136 g - - -
          2.68618  4.42225  2.77519  2.73123  3.46354  2.40513  3.72494  3.29354  2.67741  2.69355  4.24690  2.90347  2.73739  3.18146  2.89801  2.37887  2.77519  2.98518  4.58477  3.61503
          0.03268  3.83303  4.55537  0.61958  0.77255  0.48576  0.95510
    137   3.19134  4.69630  3.78054  3.50310  2.29611  3.78460  3.60201  3.20618  3.36176  2.67014  3.88549  3.67575  4.25966  3.67727  3.55331  3.33175  3.47801  3.07495  3.93816  1.03399    137 y - - -
          2.68618  4.42225  2.77519  2.73123  3.46354  2.40513  3.72494  3.29354  2.67741  2.69355  4.24690  2.90347  2.73739  3.18146  2.89801  2.37887  2.77519  2.98518  4.58477  3.61503
          0.03268  3.83303  4.55537  0.61958  0.77255  0.48576  0.95510
    138   3.11165  4.58599  4.12585  3.76620  3.12182  3.93147  4.43434  2.32453  3.53431  0.92536  3.15834  4.04543  4.37407  3.91210  3.71656  3.49871  3.40796  2.35149  4.98612  3.70011    138 l - - -
          2.68618  4.42225  2.77519  2.73123  3.46354  2.40513  3.72494  3.29354  2.67741  2.69355  4.24690  2.90347  2.73739  3.18146  2.89801  2.37887  2.77519  2.98518  4.58477  3.61503
          0.03268  3.83303  4.55537  0.61958  0.77255  0.48576  0.95510
    139   1.22039  4.11016  3.29396  3.06808  4.14573  2.49108  4.15295  3.42889  3.14052  3.22967  4.12008  3.24046  3.64203  3.44943  3.42921  2.38385  2.68272  2.96940  5.50852  4.30395    139 a - - -
          2.68618  4.42225  2.77519  2.73123  3.46354  2.40513  3.72494  3.29354  2.67741  2.69355  4.24690  2.90347  2.73739  3.18146  2.89801  2.37887  2.77519  2.98518  4.58477  3.61503
          0.03268  3.83303  4.55537  0.61958  0.77255  0.48576  0.95510
    140   2.85084  4.83409  2.87832  2.63320  3.99721  3.34074  3.79090  3.62260  2.45691  3.12880  4.15852  3.09230  3.89411  1.30591  2.74155  2.90061  3.14819  3.35495  5.27660  3.96796    140 q - - -
          2.68618  4.42225  2.77519  2.73123  3.46354  2.40513  3.72494  3.29354  2.67741  2.69355  4.24690  2.90347  2.73739  3.18146  2.89801  2.37887  2.77519  2.98518  4.58477  3.61503
          0.03268  3.83303  4.55537  0.61958  0.77255  0.48576  0.95510
    141   2.77713  4.33135  4.07016  3.73272  3.52199  3.68239  4.54148  2.05456  3.59987  2.21635  3.49380  3.92869  4.24556  3.96282  3.81605  3.18396  3.13994  1.07623  5.29954  4.02841    141 v - - -
          2.68618  4.42225  2.77519  2.73123  3.46354  2.40513  3.72494  3.29354  2.67741  2.69355  4.24690  2.90347  2.73739  3.18146  2.89801  2.37887  2.77519  2.98518  4.58477  3.61503
          0.03268  3.83303  4.55537  0.61958  0.77255  0.48576  0.95510
    142   3.11614  4.53160  4.42181  3.91721  3.08504  4.24926  4.57977  1.87497  3.70961  1.09557  2.97430  4.21059  4.54268  3.99673  3.90718  3.62404  3.36665  2.06422  5.07604  3.85551    142 l - - -
          2.68618  4.42225  2.77519  2.73123  3.46354  2.40513  3.72494  3.29354  2.67741  2.69355  4.24690  2.90347  2.73739  3.18146  2.89801  2.37887  2.77519  2.98518  4.58477  3.61503
          0.03268  3.83303  4.55537  0.61958  0.77255  0.48576  0.95510
    143   2.94831  5.06869  0.97632  2.31258  4.44069  3.18175  3.89799  4.02012  2.97493  3.63580  4.61368  2.86669  3.83184  3.13755  3.48525  2.91560  3.28628  3.66374  5.61691  4.32095    143 d - - -
          2.68618  4.42225  2.77519  2.73123  3.46354  2.40513  3.72494  3.29354  2.67741  2.69355  4.24690  2.90347  2.73739  3.18146  2.89801  2.37887  2.77519  2.98518  4.58477  3.61503
          0.03268  3.83303  4.55537  0.61958  0.77255  0.48576  0.95510
    144   2.91965  5.02079  2.47306  1.08285  4.36227  3.24954  3.83381  3.80837  2.70946  3.43216  4.40865  2.91254  3.85246  3.05076  3.11366  2.90651  3.22382  3.49656  5.54134  4.26436    144 e - - -
          2.68618  4.42225  2.77519  2.73123  3.46354  2.40513  3.72494  3.29354  2.67741  2.69355  4.24690  2.90347  2.73739  3.18146  2.89801  2.37887  2.77519  2.98518  4.58477  3.61503
          0.03268  3.83303  4.55537  0.61958  0.77255  0.48576  0.95510
    145   3.00047  4.43103  4.27833  3.89317  3.34832  4.02286  4.62907  1.12816  3.72049  1.94320  3.29060  4.14408  4.45846  4.07325  3.92162  3.51489  3.30059  1.84674  5.23051  3.96495    145 i - - -
          2.68618  4.42225  2.77519  2.73123  3.46354  2.40513  3.72494  3.29354  2.67741  2.69355  4.24690  2.90347  2.73739  3.18146  2.89801  2.37887  2.77519  2.98518  4.58477  3.61503
          0.03268  3.83303  4.55537  0.61958  0.77255  0.48576  0.95510
    146   2.96214  4.80276  3.44889  2.96907  4.14736  3.45685  3.79770  3.68689  2.22438  3.21005  4.21188  3.33151  3.96192  3.02053  1.04043  3.04858  3.23116  3.41270  5.24678  4.08393    146 r - - -
          2.68618  4.42225  2.77519  2.73123  3.46354  2.40513  3.72494  3.29354  2.67741  2.69355  4.24690  2.90347  2.73739  3.18146  2.89801  2.37887  2.77519  2.98518  4.58477  3.61503
          0.03268  3.83303  4.55537  0.61958  0.77255  0.48576  0.95510
    147   2.95371  4.77708  3.08911  2.84570  3.25855  3.41543  1.22850  3.65045  2.68990  3.14913  4.18951  3.25638  3.96612  3.21160  2.95766  3.02494  3.25600  3.38639  4.72403  3.21065    147 h - - -
          2.68618  4.42225  2.77519  2.73123  3.46354  2.40513  3.72494  3.29354  2.67741  2.69355  4.24690  2.90347  2.73739  3.18146  2.89801  2.37887  2.77519  2.98518  4.58477  3.61503
          0.03268  3.83303  4.55537  0.61958  0.77255  0.48576  0.95510
    148   2.38510  4.19187  3.57380  3.19012  3.75470  3.19433  4.14548  2.64030  3.08537  2.62549  3.69832  3.40898  3.84127  3.45382  3.36939  2.62062  1.62712  2.14480  5.30472  4.06051    148 t - - -
          2.68618  4.42225  2.77519  2.73123  3.46354  2.40513  3.72494  3.29354  2.67741  2.69355  4.24690  2.90347  2.73739  3.18146  2.89801  2.37887  2.77519  2.98518  4.58477  3.61503
          0.03268  3.83303  4.55537  0.61958  0.77255  0.48576  0.95510
    149   2.74001  4.85496  2.69487  2.43608  3.69451  3.32052  2.06717  3.65469  2.43962  3.21142  4.10362  2.35061  3.84131  2.89318  2.79569  2.75888  3.01423  3.32673  5.08073  3.59893    149 h - - -
          2.68618  4.42225  2.77519  2.73123  3.46354  2.40513  3.72494  3.29354  2.67741  2.69355  4.24690  2.90347  2.73739  3.18146  2.89801  2.37887  2.77519  2.98518  4.58477  3.61503
          0.03268  3.83303  4.55537  0.61958  0.77255  0.48576  0.95510
    150   2.85084  4.83409  2.87832  2.63320  3.99721  3.34074  3.79090  3.62260  2.45691  3.12880  4.15852  3.09230  3.89411  1.30591  2.74155  2.90061  3.14819  3.35495  5.27660  3.96796    150 q - - -
          2.68618  4.42225  2.77519  2.73123  3.46354  2.40513  3.72494  3.29354  2.67741  2.69355  4.24690  2.90347  2.73739  3.18146  2.89801  2.37887  2.77519  2.98518  4.58477  3.61503
          0.03268  3.83303  4.55537  0.61958  0.77255  0.48576  0.95510
    151   2.25382  1.72330  3.90993  3.53466  3.73976  3.05415  4.28101  2.73675  3.36312  2.71837  3.73527  3.52960  3.76618  3.68854  3.56207  2.51405  2.32392  2.45176  5.26166  4.05675    151 c - - -
          2.68618  4.42225  2.77519  2.73123  3.46354  2.40513  3.72494  3.29354  2.67741  2.69355  4.24690  2.90347  2.73739  3.18146  2.89801  2.37887  2.77519  2.98518  4.58477  3.61503
          0.03268  3.83303  4.55537  0.61958  0.77255  0.48576  0.95510
    152   1.20986  4.11096  3.29782  3.07291  4.14272  2.52260  4.15565  3.42030  3.14292  3.22582  4.11931  3.24412  3.64413  3.45342  3.42993  2.38701  2.68533  2.96415  5.50682  4.30255    152 a - - -
          2.68618  4.42225  2.77519  2.73123  3.46354  2.40513  3.72494  3.29354  2.67741  2.69355  4.24690  2.90347  2.73739  3.18146  2.89801  2.37887  2.77519  2.98518  4.58477  3.61503
          0.03268  3.83303  4.55537  0.61958  0.77255  0.48576  0.95510
```

153	3.31901	4.66639	4.29375	3.91306	1.41332	4.13594	3.44289	3.04223	3.78137	2.42653	3.66551	3.85948	4.46762	3.86324	3.90114	3.48998	3.54945	2.96168	3.63788	1.45256	153 f - - -
	2.68618	4.42225	2.77519	2.73123	3.46354	2.40513	3.72494	3.29354	2.67741	2.69355	4.24690	2.90347	2.73739	3.18146	2.89801	2.37887	2.77519	2.98518	4.58477	3.61503	
	0.03268	3.83303	4.55537	0.61958	0.77255	0.48576	0.95510														
154	2.97390	4.35546	4.50185	4.00795	3.42747	4.21104	4.75918	1.39085	3.88068	1.95194	3.26146	4.25941	4.56325	4.16912	4.10367	3.59113	3.24789	1.35646	5.35839	4.14408	154 v - - -
	2.68618	4.42225	2.77519	2.73123	3.46354	2.40513	3.72494	3.29354	2.67741	2.69355	4.24690	2.90347	2.73739	3.18146	2.89801	2.37887	2.77519	2.98518	4.58477	3.61503	
	0.03268	3.83303	4.55537	0.61958	0.77255	0.48576	0.95510														
155	2.69182	4.68600	2.68850	2.57033	4.04344	3.15836	3.89101	3.74595	2.79175	3.39751	4.35350	1.22987	3.80860	3.16424	3.14460	2.76087	3.07272	3.37215	5.35398	3.99503	155 n - - -
	2.68618	4.42225	2.77519	2.73123	3.46354	2.40513	3.72494	3.29354	2.67741	2.69355	4.24690	2.90347	2.73739	3.18146	2.89801	2.37887	2.77519	2.98518	4.58477	3.61503	
	0.03268	3.83303	4.55537	0.61958	0.77255	0.48576	0.95510														
156	3.02035	4.71432	3.48686	3.08459	2.32507	3.75457	2.30259	3.31883	2.92383	2.79809	3.86135	3.38728	4.15546	3.29751	3.20342	3.10180	3.25548	3.12056	3.95038	1.61525	156 y - - -
	2.68618	4.42225	2.77519	2.73123	3.46354	2.40513	3.72494	3.29354	2.67741	2.69355	4.24690	2.90347	2.73739	3.18146	2.89801	2.37887	2.77519	2.98518	4.58477	3.61503	
	0.03268	3.83303	4.55537	0.61958	0.77255	0.48576	0.95510														
157	3.19134	4.69630	3.78054	3.50310	2.29611	3.78460	3.60201	3.20618	3.36176	2.67014	3.88549	3.67575	4.25966	3.67727	3.55331	3.33175	3.47801	3.07495	3.93816	1.03399	157 y - - -
	2.68618	4.42225	2.77519	2.73123	3.46354	2.40513	3.72494	3.29354	2.67741	2.69355	4.24690	2.90347	2.73739	3.18146	2.89801	2.37887	2.77519	2.98518	4.58477	3.61503	
	0.03268	3.83303	4.55537	0.61958	0.77255	0.48576	0.95510														
158	3.32226	4.67335	4.28046	3.90017	1.50716	4.13275	3.43593	3.05952	3.76945	2.44719	3.68323	3.85112	4.46587	3.85523	3.89346	3.48663	3.55293	2.97508	3.63222	1.35460	158 y - - -
	2.68618	4.42225	2.77519	2.73123	3.46354	2.40513	3.72494	3.29354	2.67741	2.69355	4.24690	2.90347	2.73739	3.18146	2.89801	2.37887	2.77519	2.98518	4.58477	3.61503	
	0.03268	3.83303	4.55537	0.61958	0.77255	0.48576	0.95510														
159	1.76331	4.10747	3.22457	2.95367	4.19321	2.44142	4.07422	3.59288	3.04599	3.28981	4.11939	3.16024	3.60441	3.33625	3.37564	1.62450	2.63034	3.06727	5.53288	4.30676	159 s - - -
	2.68618	4.42225	2.77519	2.73123	3.46354	2.40513	3.72494	3.29354	2.67741	2.69355	4.24690	2.90347	2.73739	3.18146	2.89801	2.37887	2.77519	2.98518	4.58477	3.61503	
	0.03268	3.83303	4.55537	0.61958	0.77255	0.48576	0.95510														
160	2.93703	4.89791	3.11032	2.74002	4.27531	3.43004	3.73906	3.70226	1.09362	3.27630	4.24050	3.16601	3.93369	2.93324	2.42654	2.98198	3.19357	3.41249	5.34669	4.15401	160 k - - -
	2.68618	4.42225	2.77519	2.73123	3.46354	2.40513	3.72494	3.29354	2.67741	2.69355	4.24690	2.90347	2.73739	3.18146	2.89801	2.37887	2.77519	2.98518	4.58477	3.61503	
	0.03268	3.83303	4.55537	0.61958	0.77255	0.48576	0.95510														
161	2.74499	4.91722	2.69580	2.39074	3.93096	3.35160	2.18634	3.65052	2.31993	3.20367	4.06853	2.57873	3.83418	2.57546	2.67173	2.74960	2.99036	3.32589	5.22449	3.81033	161 h - - -
	2.68618	4.42225	2.77519	2.73123	3.46354	2.40513	3.72494	3.29354	2.67741	2.69355	4.24690	2.90347	2.73739	3.18146	2.89801	2.37887	2.77519	2.98518	4.58477	3.61503	
	0.03268	3.83303	4.55537	0.61958	0.77255	0.48576	0.95510														
162	2.66770	4.45613	3.23590	2.89570	3.00507	2.47368	3.72331	3.15136	2.87959	2.79108	3.76485	3.25626	3.92144	3.23129	3.19462	2.81953	2.97568	2.89934	4.54995	1.78470	162 y - - -
	2.68618	4.42225	2.77519	2.73123	3.46354	2.40513	3.72494	3.29354	2.67741	2.69355	4.24690	2.90347	2.73739	3.18146	2.89801	2.37887	2.77519	2.98518	4.58477	3.61503	
	0.03268	3.83303	4.55537	0.61958	0.77255	0.48576	0.95510														
163	2.72038	4.76163	3.00688	2.55111	3.36547	3.46613	2.45236	3.39671	2.37533	2.96238	3.85997	3.02212	3.87926	2.40806	2.72246	2.76662	2.94709	3.11908	4.80948	2.91379	163 k - - -
	2.68618	4.42225	2.77519	2.73123	3.46354	2.40513	3.72494	3.29354	2.67741	2.69355	4.24690	2.90347	2.73739	3.18146	2.89801	2.37887	2.77519	2.98518	4.58477	3.61503	
	0.03268	3.83303	4.55537	0.61958	0.77255	0.48576	0.95510														
164	2.94831	5.06869	0.97632	2.31258	4.44069	3.18175	3.89799	4.02012	2.97493	3.63580	4.61368	2.86669	3.83184	3.13755	3.48525	2.91560	3.28628	3.66374	5.61691	4.32095	164 d - - -
	2.68618	4.42225	2.77519	2.73123	3.46354	2.40513	3.72494	3.29354	2.67741	2.69355	4.24690	2.90347	2.73739	3.18146	2.89801	2.37887	2.77519	2.98518	4.58477	3.61503	
	0.03268	3.83303	4.55537	0.61958	0.77255	0.48576	0.95510														
165	2.08981	4.26090	3.13291	2.89963	4.07966	3.00609	4.04294	3.39599	2.95310	3.12916	4.07660	3.18485	1.58879	3.31372	3.26529	2.49769	2.78149	3.00382	5.44365	4.20428	165 p - - -
	2.68618	4.42225	2.77519	2.73123	3.46354	2.40513	3.72494	3.29354	2.67741	2.69355	4.24690	2.90347	2.73739	3.18146	2.89801	2.37887	2.77519	2.98518	4.58477	3.61503	
	0.03268	3.83303	4.55537	0.61958	0.77255	0.48576	0.95510														
166	1.07216	4.17353	3.42348	3.21371	4.01396	2.99897	4.24029	3.13365	3.22896	3.01700	4.05375	3.37300	3.73453	3.57391	3.48180	2.52446	2.79912	2.79493	5.44509	4.24110	166 a - - -
	2.68618	4.42225	2.77519	2.73123	3.46354	2.40513	3.72494	3.29354	2.67741	2.69355	4.24690	2.90347	2.73739	3.18146	2.89801	2.37887	2.77519	2.98518	4.58477	3.61503	
	0.03268	3.83303	4.55537	0.61958	0.77255	0.48576	0.95510														
167	2.61765	4.39623	3.28327	3.17805	4.31966	0.79266	4.30013	3.91025	3.35388	3.56921	4.54515	3.44774	3.78610	3.68127	3.59365	2.78855	3.09584	3.44624	5.42460	4.42045	167 g - - -
	2.68618	4.42225	2.77519	2.73123	3.46354	2.40513	3.72494	3.29354	2.67741	2.69355	4.24690	2.90347	2.73739	3.18146	2.89801	2.37887	2.77519	2.98518	4.58477	3.61503	
	0.03268	3.83303	4.55537	0.61958	0.77255	0.48576	0.95510														
168	2.95371	4.77708	3.08911	2.84570	3.25855	3.41543	1.22850	3.65045	2.68990	3.14913	4.18951	3.25638	3.96612	3.21160	2.95766	3.02494	3.25600	3.38639	4.72403	3.21065	168 h - - -
	2.68618	4.42225	2.77519	2.73123	3.46354	2.40513	3.72494	3.29354	2.67741	2.69355	4.24690	2.90347	2.73739	3.18146	2.89801	2.37887	2.77519	2.98518	4.58477	3.61503	
	0.03268	3.83303	4.55537	0.61958	0.77255	0.48576	0.95510														
169	2.49756	4.54779	2.72370	2.52328	4.13979	3.10895	3.82491	3.55691	2.67027	3.25824	4.14779	1.67673	3.74304	3.04250	3.04440	2.58482	2.49051	3.17069	5.45626	4.11309	169 n - - -
	2.68618	4.42225	2.77519	2.73123	3.46354	2.40513	3.72494	3.29354	2.67741	2.69355	4.24690	2.90347	2.73739	3.18146	2.89801	2.37887	2.77519	2.98518	4.58477	3.61503	
	0.03268	3.83303	4.55537	0.61958	0.77255	0.48576	0.95510														
170	2.81624	5.21169	1.36059	2.09950	4.53004	3.13779	3.72956	4.09054	2.75109	3.65208	4.52578	2.33680	3.77138	2.91654	3.31144	2.74391	3.13023	3.68068	5.77175	4.29845	170 d - - -
	2.68618	4.42225	2.77519	2.73123	3.46354	2.40513	3.72494	3.29354	2.67741	2.69355	4.24690	2.90347	2.73739	3.18146	2.89801	2.37887	2.77519	2.98518	4.58477	3.61503	
	0.03268	3.83303	4.55537	0.61958	0.77255	0.48576	0.95510														
171	1.57850	4.24746	3.54787	3.11428	3.61541	3.30254	4.06319	2.62460	3.00478	2.45806	2.95188	3.39548	3.88677	3.37054	3.30456	2.69600	2.82952	2.42162	5.18399	3.94009	171 a - - -
	2.68618	4.42225	2.77519	2.73123	3.46354	2.40513	3.72494	3.29354	2.67741	2.69355	4.24690	2.90347	2.73739	3.18146	2.89801	2.37887	2.77519	2.98518	4.58477	3.61503	
	0.03268	3.83303	4.55537	0.61958	0.77255	0.48576	0.95510														
172	2.96214	4.80276	3.44889	2.96907	4.14736	3.45685	3.79770	3.68689	2.22438	3.21005	4.21188	3.33151	3.96192	3.02053	1.04043	3.04858	3.23116	3.41270	5.24678	4.08393	172 r - - -
	2.68618	4.42225	2.77519	2.73123	3.46354	2.40513	3.72494	3.29354	2.67741	2.69355	4.24690	2.90347	2.73739	3.18146	2.89801	2.37887	2.77519	2.98518	4.58477	3.61503	
	0.03268	3.83303	4.55537	0.61958	0.77255	0.48576	0.95510														
173	2.88604	4.93815	3.22752	2.69414	4.25547	3.51099	3.59853	3.70452	1.97399	3.19836	4.10527	3.10972	3.93299	2.44337	1.46106	2.91485	3.09584	3.40773	5.30653	4.07742	173 r - - -
	2.68618	4.42225	2.77519	2.73123	3.46354	2.40513	3.72494	3.29354	2.67741	2.69355	4.24690	2.90347	2.73739	3.18146	2.89801	2.37887	2.77519	2.98518	4.58477	3.61503	
	0.03268	3.83303	4.55537	0.61958	0.77255	0.48576	0.95510														
174	2.51358	4.30133	3.49751	3.09509	3.61542	3.32970	4.05108	2.63239	2.94548	2.22709	3.60531	3.39181	3.91196	3.35827	3.23354	2.74082	1.69028	2.43361	5.18285	3.90549	174 t - - -
	2.68618	4.42225	2.77519	2.73123	3.46354	2.40513	3.72494	3.29354	2.67741	2.69355	4.24690	2.90347	2.73739	3.18146	2.89801	2.37887	2.77519	2.98518	4.58477	3.61503	
	0.03268	3.83303	4.55537	0.61958	0.77255	0.48576	0.95510														
175	2.96214	4.80276	3.44889	2.96907	4.14736	3.45685	3.79770	3.68689	2.22438	3.21005	4.21188	3.33151	3.96192	3.02053	1.04043	3.04858	3.23116	3.41270	5.24678	4.08393	175 r - - -

```
      2.68618  4.42225  2.77519  2.73123  3.46354  2.40513  3.72494  3.29354  2.67741  2.69355  4.24690  2.90347  2.73739  3.18146  2.89801  2.37887  2.77519  2.98518  4.58477  3.61503
      0.03268  3.83303  4.55537  0.61958  0.77255  0.48576  0.95510
176   1.32678  4.10114  3.42292  3.13859  4.03559  2.95355  4.16500  3.09682  3.12591  3.01427  3.97205  3.29399  3.68265  3.45959  3.40703  2.41516  2.30146  2.73116  5.47416  4.25852    176 a - - -
      2.68618  4.42225  2.77519  2.73123  3.46354  2.40513  3.72494  3.29354  2.67741  2.69355  4.24690  2.90347  2.73739  3.18146  2.89801  2.37887  2.77519  2.98518  4.58477  3.61503
      0.03268  3.83303  4.55537  0.61958  0.77255  0.48576  0.95510
177   3.00047  4.43103  4.27833  3.89317  3.34832  4.02286  4.62907  1.12816  3.72049  1.94320  3.29060  4.14408  4.45846  4.07325  3.92162  3.51489  3.30059  1.84674  5.23051  3.96495    177 i - - -
      2.68618  4.42225  2.77519  2.73123  3.46354  2.40513  3.72494  3.29354  2.67741  2.69355  4.24690  2.90347  2.73739  3.18146  2.89801  2.37887  2.77519  2.98518  4.58477  3.61503
      0.03268  3.83303  4.55537  0.61958  0.77255  0.48576  0.95510
178   2.41332  4.41549  2.77153  2.62986  4.23629  1.50266  3.94781  3.76410  2.88008  3.41387  4.27813  2.49152  3.69773  3.19505  3.24663  2.52684  2.84667  3.28467  5.52428  4.23223    178 g - - -
      2.68618  4.42225  2.77519  2.73123  3.46354  2.40513  3.72494  3.29354  2.67741  2.69355  4.24690  2.90347  2.73739  3.18146  2.89801  2.37887  2.77519  2.98518  4.58477  3.61503
      0.03268  3.83303  4.55537  0.61958  0.77255  0.48576  0.95510
179   2.70125  4.46443  3.30428  3.13815  4.13555  3.15421  4.21835  3.67141  3.19219  3.30231  4.35093  3.44851  0.90433  3.58220  3.44165  2.86766  3.13986  3.32122  5.33234  4.25589    179 p - - -
      2.68618  4.42225  2.77519  2.73123  3.46354  2.40513  3.72494  3.29354  2.67741  2.69355  4.24690  2.90347  2.73739  3.18146  2.89801  2.37887  2.77519  2.98518  4.58477  3.61503
      0.03268  3.83303  4.55537  0.61958  0.77255  0.48576  0.95510
180   3.11165  4.58599  4.12585  3.76620  3.12182  3.93147  4.43434  2.32453  3.53431  0.92536  3.15834  4.04543  4.37407  3.91210  3.71656  3.49871  3.40796  2.35149  4.98612  3.70011    180 l - - -
      2.68618  4.42225  2.77519  2.73123  3.46354  2.40513  3.72494  3.29354  2.67741  2.69355  4.24690  2.90347  2.73739  3.18146  2.89801  2.37887  2.77519  2.98518  4.58477  3.61503
      0.03268  3.83303  4.55537  0.61958  0.77255  0.48576  0.95510
181   3.20630  4.63349  4.10661  3.68461  2.06886  3.99217  3.46070  3.12413  3.44188  2.54856  3.71924  3.74111  4.35515  3.69093  3.60684  3.36753  3.44015  3.00025  1.68288  1.81536    181 w - - -
      2.68618  4.42225  2.77519  2.73123  3.46354  2.40513  3.72494  3.29354  2.67741  2.69355  4.24690  2.90347  2.73739  3.18146  2.89801  2.37887  2.77519  2.98518  4.58477  3.61503
      0.03268  3.83303  4.55537  0.61958  0.77255  0.48576  0.95510
182   2.74028  4.83342  2.94330  2.55547  4.26279  3.35626  3.66284  3.66270  1.44282  3.23412  4.11456  3.01358  3.07756  2.83186  2.49512  2.77709  3.00480  3.32979  5.38218  4.12662    182 k - - -
      2.68618  4.42225  2.77519  2.73123  3.46354  2.40513  3.72494  3.29354  2.67741  2.69355  4.24690  2.90347  2.73739  3.18146  2.89801  2.37887  2.77519  2.98518  4.58477  3.61503
      0.03268  3.83303  4.55537  0.61958  0.77255  0.48576  0.95510
183   2.61765  4.39623  3.28327  3.17805  4.31966  0.79266  4.30013  3.91025  3.35388  3.56921  4.54515  3.44774  3.78610  3.68127  3.59365  2.78855  3.09584  3.44624  5.42460  4.42045    183 g - - -
      2.68618  4.42225  2.77519  2.73123  3.46354  2.40513  3.72494  3.29354  2.67741  2.69355  4.24690  2.90347  2.73739  3.18146  2.89801  2.37887  2.77519  2.98518  4.58477  3.61503
      0.03268  3.83303  4.55537  0.61958  0.77255  0.48576  0.95510
184   2.89298  4.41775  4.14943  3.64703  3.18758  3.92926  4.39511  2.16508  3.46360  1.71941  1.80299  3.93249  4.32382  3.77904  3.69275  3.29535  3.17324  1.96000  5.07234  3.87560    184 l - - -
      2.68618  4.42225  2.77519  2.73123  3.46354  2.40513  3.72494  3.29354  2.67741  2.69355  4.24690  2.90347  2.73739  3.18146  2.89801  2.37887  2.77519  2.98518  4.58477  3.61503
      0.03268  3.83303  4.55537  0.61958  0.77255  0.48576  0.95510
185   2.93703  4.89791  3.11032  2.74002  4.27531  3.43004  3.73906  3.70226  1.09362  3.27630  4.24050  3.16601  3.93369  2.93324  2.42654  2.98198  3.19357  3.41249  5.34669  4.15401    185 k - - -
      2.68618  4.42225  2.77519  2.73123  3.46354  2.40513  3.72494  3.29354  2.67741  2.69355  4.24690  2.90347  2.73739  3.18146  2.89801  2.37887  2.77519  2.98518  4.58477  3.61503
      0.03268  3.83303  4.55537  0.61958  0.77255  0.48576  0.95510
186   2.96214  4.80276  3.44889  2.96907  4.14736  3.45685  3.79770  3.68689  2.22438  3.21005  4.21188  3.33151  3.96192  3.02053  1.04043  3.04858  3.23116  3.41270  5.24678  4.08393    186 r - - -
      2.68618  4.42225  2.77519  2.73123  3.46354  2.40513  3.72494  3.29354  2.67741  2.69355  4.24690  2.90347  2.73739  3.18146  2.89801  2.37887  2.77519  2.98518  4.58477  3.61503
      0.03268  3.83303  4.55537  0.61958  0.77255  0.48576  0.95510
187   2.44789  4.17864  3.66308  3.29598  3.62255  3.29664  4.18737  2.43778  3.20351  2.55432  3.62493  3.50244  3.91975  3.55512  3.46653  2.39428  2.83925  1.42867  5.20392  3.93031    187 v - - -
      2.68618  4.42225  2.77519  2.73123  3.46354  2.40513  3.72494  3.29354  2.67741  2.69355  4.24690  2.90347  2.73739  3.18146  2.89801  2.37887  2.77519  2.98518  4.58477  3.61503
      0.03268  3.83303  4.55537  0.61958  0.77255  0.48576  0.95510
188   3.20912  4.62273  4.18444  3.89199  1.07857  3.90702  3.86137  2.73830  3.80015  2.11778  3.47472  3.97513  4.36694  3.97918  3.92960  3.48072  3.51047  2.72599  4.12320  2.49559    188 f - - -
      2.68618  4.42225  2.77519  2.73123  3.46354  2.40513  3.72494  3.29354  2.67741  2.69355  4.24690  2.90347  2.73739  3.18146  2.89801  2.37887  2.77519  2.98518  4.58477  3.61503
      0.03268  3.83303  4.55537  0.61958  0.77255  0.48576  0.95510
189   1.58884  4.09624  3.25158  2.97528  4.17806  2.44032  4.08212  3.57066  3.05984  3.27375  4.10517  3.17137  3.60394  3.34855  3.38533  1.80011  2.62562  3.05031  5.52282  4.30071    189 a - - -
      2.68618  4.42225  2.77519  2.73123  3.46354  2.40513  3.72494  3.29354  2.67741  2.69355  4.24690  2.90347  2.73739  3.18146  2.89801  2.37887  2.77519  2.98518  4.58477  3.61503
      0.03268  3.83303  4.55537  0.61958  0.77255  0.48576  0.95510
190   2.87759  5.33786  1.33387  1.86786  4.63127  3.15364  3.72174  4.12359  2.74483  3.67936  4.56019  2.64720  3.78205  2.90177  3.31840  2.77979  3.17457  3.72921  5.83535  4.36780    190 d - - -
      2.68618  4.42225  2.77519  2.73123  3.46354  2.40513  3.72494  3.29354  2.67741  2.69355  4.24690  2.90347  2.73739  3.18146  2.89801  2.37887  2.77519  2.98518  4.58477  3.61503
      0.03268  3.83303  4.55537  0.61958  0.77255  0.48576  0.95510
191   2.61765  4.39623  3.28327  3.17805  4.31966  0.79266  4.30013  3.91025  3.35388  3.56921  4.54515  3.44774  3.78610  3.68127  3.59365  2.78855  3.09584  3.44624  5.42460  4.42045    191 g - - -
      2.68618  4.42225  2.77519  2.73123  3.46354  2.40513  3.72494  3.29354  2.67741  2.69355  4.24690  2.90347  2.73739  3.18146  2.89801  2.37887  2.77519  2.98518  4.58477  3.61503
      0.03268  3.83303  4.55537  0.61958  0.77255  0.48576  0.95510
192   3.20912  4.62273  4.18444  3.89199  1.07857  3.90702  3.86137  2.73830  3.80015  2.11778  3.47472  3.97513  4.36694  3.97918  3.92960  3.48072  3.51047  2.72599  4.12320  2.49559    192 f - - -
      2.68618  4.42225  2.77519  2.73123  3.46354  2.40513  3.72494  3.29354  2.67741  2.69355  4.24690  2.90347  2.73739  3.18146  2.89801  2.37887  2.77519  2.98518  4.58477  3.61503
      0.03268  3.83303  4.55537  0.61958  0.77255  0.48576  0.95510
193   3.05842  4.44288  4.46438  3.97453  3.24535  4.25288  4.66494  1.28899  3.78539  1.63545  3.13223  4.24752  4.57064  4.09300  3.99079  3.63824  3.31861  1.80341  5.20134  3.95958    193 i - - -
      2.68618  4.42225  2.77519  2.73123  3.46354  2.40513  3.72494  3.29354  2.67741  2.69355  4.24690  2.90347  2.73739  3.18146  2.89801  2.37887  2.77519  2.98518  4.58477  3.61503
      0.03268  3.83303  4.55537  0.61958  0.77255  0.48576  0.95510
194   1.94020  4.12787  3.22040  2.98469  4.14082  2.89101  4.09593  3.49170  3.05042  3.25036  4.12838  3.18609  3.63166  3.37355  3.35554  1.36516  2.66799  3.01268  5.51269  4.26187    194 s - - -
      2.68618  4.42225  2.77519  2.73123  3.46354  2.40513  3.72494  3.29354  2.67741  2.69355  4.24690  2.90347  2.73739  3.18146  2.89801  2.37887  2.77519  2.98518  4.58477  3.61503
      0.03268  3.83303  4.55537  0.61958  0.77255  0.48576  0.95510
195   2.61765  4.39623  3.28327  3.17805  4.31966  0.79266  4.30013  3.91025  3.35388  3.56921  4.54515  3.44774  3.78610  3.68127  3.59365  2.78855  3.09584  3.44624  5.42460  4.42045    195 g - - -
      2.68618  4.42225  2.77519  2.73123  3.46354  2.40513  3.72494  3.29354  2.67741  2.69355  4.24690  2.90347  2.73739  3.18146  2.89801  2.37887  2.77519  2.98518  4.58477  3.61503
      0.03268  3.83303  4.55537  0.61958  0.77255  0.48576  0.95510
196   2.94831  5.06869  0.97632  2.31258  4.44069  3.18175  3.89799  4.02012  2.97493  3.63580  4.61368  2.86669  3.83184  3.13755  3.48525  2.91560  3.28628  3.66374  5.61691  4.32095    196 d - - -
      2.68618  4.42225  2.77519  2.73123  3.46354  2.40513  3.72494  3.29354  2.67741  2.69355  4.24690  2.90347  2.73739  3.18146  2.89801  2.37887  2.77519  2.98518  4.58477  3.61503
      0.03268  3.83303  4.55537  0.61958  0.77255  0.48576  0.95510
197   1.07216  4.17353  3.42348  3.21371  4.01396  2.99897  4.24029  3.13365  3.22896  3.01700  4.05375  3.37300  3.73453  3.57391  3.48180  2.52446  2.79912  2.79493  5.44509  4.24110    197 a - - -
      2.68618  4.42225  2.77519  2.73123  3.46354  2.40513  3.72494  3.29354  2.67741  2.69355  4.24690  2.90347  2.73739  3.18146  2.89801  2.37887  2.77519  2.98518  4.58477  3.61503
```

```
     0.03268  3.83303  4.55537  0.61958  0.77255  0.48576  0.95510
198  2.77713  4.33135  4.07016  3.73272  3.52199  3.68239  4.54148  2.05456  3.59987  2.21635  3.49380  3.92869  4.24556  3.96282  3.81605  3.18396  3.13994  1.07623  5.29954  4.02841   198 v - - -
     2.68618  4.42225  2.77519  2.73123  3.46354  2.40513  3.72494  3.29354  2.67741  2.69355  4.24690  2.90347  2.73739  3.18146  2.89801  2.37887  2.77519  2.98518  4.58477  3.61503
     0.03268  3.83303  4.55537  0.61958  0.77255  0.48576  0.95510
199  2.91965  5.02079  2.47306  1.08285  4.36227  3.24954  3.83381  3.80837  2.70946  3.43216  4.40865  2.91254  3.85246  3.05076  3.11366  2.90651  3.22382  3.49656  5.54134  4.26436   199 e - - -
     2.68618  4.42225  2.77519  2.73123  3.46354  2.40513  3.72494  3.29354  2.67741  2.69355  4.24690  2.90347  2.73739  3.18146  2.89801  2.37887  2.77519  2.98518  4.58477  3.61503
     0.03268  3.83303  4.55537  0.61958  0.77255  0.48576  0.95510
200  2.18222  1.69325  3.81778  3.46894  3.76930  2.95754  4.22784  3.00844  3.31950  2.88933  3.83386  3.45041  3.69124  3.63845  3.52135  2.10962  2.65636  2.65385  5.24351  4.01136   200 c - - -
     2.68618  4.42225  2.77519  2.73123  3.46354  2.40513  3.72494  3.29354  2.67741  2.69355  4.24690  2.90347  2.73739  3.18146  2.89801  2.37887  2.77519  2.98518  4.58477  3.61503
     0.03268  3.83303  4.55537  0.61958  0.77255  0.48576  0.95510
201  2.29672  4.21624  3.17324  2.98785  4.03735  2.94690  4.09847  3.55494  3.05893  3.27847  4.20833  3.21852  3.68259  3.41149  3.34573  1.15212  2.77402  3.09870  5.41316  4.12083   201 s - - -
     2.68618  4.42225  2.77519  2.73123  3.46354  2.40513  3.72494  3.29354  2.67741  2.69355  4.24690  2.90347  2.73739  3.18146  2.89801  2.37887  2.77519  2.98518  4.58477  3.61503
     0.03268  3.83303  4.55537  0.61958  0.77255  0.48576  0.95510
202  3.01028  4.37338  4.52665  3.99777  3.35334  4.28445  4.72611  1.53081  3.87469  1.71911  3.17897  4.27234  4.58213  4.13794  4.09572  3.63731  3.26327  1.38449  5.29423  4.10345   202 v - - -
     2.68618  4.42225  2.77519  2.73123  3.46354  2.40513  3.72494  3.29354  2.67741  2.69355  4.24690  2.90347  2.73739  3.18146  2.89801  2.37887  2.77519  2.98518  4.58477  3.61503
     0.03268  3.83303  4.55537  0.61958  0.77255  0.48576  0.95510
203  2.69182  4.68600  2.68850  2.57033  4.04344  3.15836  3.89101  3.74595  2.79175  3.39751  4.35350  1.22987  3.80860  3.16424  3.14460  2.76087  3.07272  3.37215  5.35398  3.99503   203 n - - -
     2.68618  4.42225  2.77519  2.73123  3.46354  2.40513  3.72494  3.29354  2.67741  2.69355  4.24690  2.90347  2.73739  3.18146  2.89801  2.37887  2.77519  2.98518  4.58477  3.61503
     0.03268  3.83303  4.55537  0.61958  0.77255  0.48576  0.95510
204  3.11165  4.58599  4.12585  3.76620  3.12182  3.93147  4.43434  2.32453  3.53431  0.92536  3.15834  4.04543  4.37407  3.91210  3.71656  3.49871  3.40796  2.35149  4.98612  3.70011   204 l - - -
     2.68618  4.42225  2.77519  2.73123  3.46354  2.40513  3.72494  3.29354  2.67741  2.69355  4.24690  2.90347  2.73739  3.18146  2.89801  2.37887  2.77519  2.98518  4.58477  3.61503
     0.03268  3.83303  4.55537  0.61958  0.77255  0.48576  0.95510
205  2.85084  4.83409  2.87832  2.63320  3.99721  3.34074  3.79090  3.62260  2.45691  3.12880  4.15852  3.09230  3.89411  1.30591  2.74155  2.90061  3.14819  3.35495  5.27660  3.96796   205 q - - -
     2.68618  4.42225  2.77519  2.73123  3.46354  2.40513  3.72494  3.29354  2.67741  2.69355  4.24690  2.90347  2.73739  3.18146  2.89801  2.37887  2.77519  2.98518  4.58477  3.61503
     0.03268  3.83303  4.55537  0.61958  0.77255  0.48576  0.95510
206  3.11165  4.58599  4.12585  3.76620  3.12182  3.93147  4.43434  2.32453  3.53431  0.92536  3.15834  4.04543  4.37407  3.91210  3.71656  3.49871  3.40796  2.35149  4.98612  3.70011   206 l - - -
     2.68618  4.42225  2.77519  2.73123  3.46354  2.40513  3.72494  3.29354  2.67741  2.69355  4.24690  2.90347  2.73739  3.18146  2.89801  2.37887  2.77519  2.98518  4.58477  3.61503
     0.03268  3.83303  4.55537  0.61958  0.77255  0.48576  0.95510
207  2.77713  4.33135  4.07016  3.73272  3.52199  3.68239  4.54148  2.05456  3.59987  2.21635  3.49380  3.92869  4.24556  3.96282  3.81605  3.18396  3.13994  1.07623  5.29954  4.02841   207 v - - -
     2.68618  4.42225  2.77519  2.73123  3.46354  2.40513  3.72494  3.29354  2.67741  2.69355  4.24690  2.90347  2.73739  3.18146  2.89801  2.37887  2.77519  2.98518  4.58477  3.61503
     0.03268  3.83303  4.55537  0.61958  0.77255  0.48576  0.95510
208  2.61765  4.39623  3.28327  3.17805  4.31966  0.79266  4.30013  3.91025  3.35388  3.56921  4.54515  3.44774  3.78610  3.68127  3.59365  2.78855  3.09584  3.44624  5.42460  4.42045   208 g - - -
     2.68618  4.42225  2.77519  2.73123  3.46354  2.40513  3.72494  3.29354  2.67741  2.69355  4.24690  2.90347  2.73739  3.18146  2.89801  2.37887  2.77519  2.98518  4.58477  3.61503
     0.03268  3.83303  4.55537  0.61958  0.77255  0.48576  0.95510
209  2.91965  5.02079  2.47306  1.08285  4.36227  3.24954  3.83381  3.80837  2.70946  3.43216  4.40865  2.91254  3.85246  3.05076  3.11366  2.90651  3.22382  3.49656  5.54134  4.26436   209 e - - -
     2.68618  4.42225  2.77519  2.73123  3.46354  2.40513  3.72494  3.29354  2.67741  2.69355  4.24690  2.90347  2.73739  3.18146  2.89801  2.37887  2.77519  2.98518  4.58477  3.61503
     0.03268  3.83303  4.55537  0.61958  0.77255  0.48576  0.95510
210  1.07216  4.17353  3.42348  3.21371  4.01396  2.99897  4.24029  3.13365  3.22896  3.01700  4.05375  3.37300  3.73453  3.57391  3.48180  2.52446  2.79912  2.79493  5.44509  4.24110   210 a - - -
     2.68618  4.42225  2.77519  2.73123  3.46354  2.40513  3.72494  3.29354  2.67741  2.69355  4.24690  2.90347  2.73739  3.18146  2.89801  2.37887  2.77519  2.98518  4.58477  3.61503
     0.03268  3.83303  4.55537  0.61958  0.77255  0.48576  0.95510
211  2.54347  1.02412  4.05233  3.78348  3.78454  3.17418  4.43931  2.92247  3.61038  2.83944  3.95970  3.78101  3.87800  3.95817  3.74153  2.81493  3.00701  2.66956  5.20830  4.07232   211 c - - -
     2.68618  4.42225  2.77519  2.73123  3.46354  2.40513  3.72494  3.29354  2.67741  2.69355  4.24690  2.90347  2.73739  3.18146  2.89801  2.37887  2.77519  2.98518  4.58477  3.61503
     0.03268  3.83303  4.55537  0.61958  0.77255  0.48576  0.95510
212  3.20912  4.62273  4.18444  3.89199  1.07857  3.90702  3.86137  2.73830  3.80015  2.11778  3.47472  3.97513  4.36694  3.97918  3.92960  3.48072  3.51047  2.72599  4.12320  2.49559   212 f - - -
     2.68618  4.42225  2.77519  2.73123  3.46354  2.40513  3.72494  3.29354  2.67741  2.69355  4.24690  2.90347  2.73739  3.18146  2.89801  2.37887  2.77519  2.98518  4.58477  3.61503
     0.03268  3.83303  4.55537  0.61958  0.77255  0.48576  0.95510
213  2.39269  4.23746  3.39425  3.14521  3.93517  3.07784  4.16539  3.05754  3.08739  2.92240  3.97601  3.36046  3.77873  3.48436  3.34758  2.59359  1.24059  2.75673  5.37648  4.15199   213 t - - -
     2.68618  4.42225  2.77519  2.73123  3.46354  2.40513  3.72494  3.29354  2.67741  2.69355  4.24690  2.90347  2.73739  3.18146  2.89801  2.37887  2.77519  2.98518  4.58477  3.61503
     0.03268  3.83303  4.55537  0.61958  0.77255  0.48576  0.95510
214  2.69182  4.68600  2.68850  2.57033  4.04344  3.15836  3.89101  3.74595  2.79175  3.39751  4.35350  1.22987  3.80860  3.16424  3.14460  2.76087  3.07272  3.37215  5.35398  3.99503   214 n - - -
     2.68618  4.42225  2.77519  2.73123  3.46354  2.40513  3.72494  3.29354  2.67741  2.69355  4.24690  2.90347  2.73739  3.18146  2.89801  2.37887  2.77519  2.98518  4.58477  3.61503
     0.03268  3.83303  4.55537  0.61958  0.77255  0.48576  0.95510
215  2.70125  4.46443  3.30428  3.13815  4.13555  3.15421  4.21835  3.67141  3.19219  3.30231  4.35093  3.44851  0.90433  3.58220  3.44165  2.86766  3.13986  3.32122  5.33234  4.25589   215 p - - -
     2.68618  4.42225  2.77519  2.73123  3.46354  2.40513  3.72494  3.29354  2.67741  2.69355  4.24690  2.90347  2.73739  3.18146  2.89801  2.37887  2.77519  2.98518  4.58477  3.61503
     0.03268  3.83303  4.55537  0.61958  0.77255  0.48576  0.95510
216  3.11165  4.58599  4.12585  3.76620  3.12182  3.93147  4.43434  2.32453  3.53431  0.92536  3.15834  4.04543  4.37407  3.91210  3.71656  3.49871  3.40796  2.35149  4.98612  3.70011   216 l - - -
     2.68618  4.42225  2.77519  2.73123  3.46354  2.40513  3.72494  3.29354  2.67741  2.69355  4.24690  2.90347  2.73739  3.18146  2.89801  2.37887  2.77519  2.98518  4.58477  3.61503
     0.03268  3.83303  4.55537  0.61958  0.77255  0.48576  0.95510
217  3.00047  4.43103  4.27833  3.89317  3.34832  4.02286  4.62907  1.12816  3.72049  1.94320  3.29060  4.14408  4.45846  4.07325  3.92162  3.51489  3.30059  1.84674  5.23051  3.96495   217 i - - -
     2.68618  4.42225  2.77519  2.73123  3.46354  2.40513  3.72494  3.29354  2.67741  2.69355  4.24690  2.90347  2.73739  3.18146  2.89801  2.37887  2.77519  2.98518  4.58477  3.61503
     0.03268  3.83303  4.55537  0.61958  0.77255  0.48576  0.95510
218  2.77713  4.33135  4.07016  3.73272  3.52199  3.68239  4.54148  2.05456  3.59987  2.21635  3.49380  3.92869  4.24556  3.96282  3.81605  3.18396  3.13994  1.07623  5.29954  4.02841   218 v - - -
     2.68618  4.42225  2.77519  2.73123  3.46354  2.40513  3.72494  3.29354  2.67741  2.69355  4.24690  2.90347  2.73739  3.18146  2.89801  2.37887  2.77519  2.98518  4.58477  3.61503
     0.03268  3.83303  4.55537  0.61958  0.77255  0.48576  0.95510
219  1.07216  4.17353  3.42348  3.21371  4.01396  2.99897  4.24029  3.13365  3.22896  3.01700  4.05375  3.37300  3.73453  3.57391  3.48180  2.52446  2.79912  2.79493  5.44509  4.24110   219 a - - -
     2.68618  4.42225  2.77519  2.73123  3.46354  2.40513  3.72494  3.29354  2.67741  2.69355  4.24690  2.90347  2.73739  3.18146  2.89801  2.37887  2.77519  2.98518  4.58477  3.61503
     0.03268  3.83303  4.55537  0.61958  0.77255  0.48576  0.95510
```

220	2.89507	4.36874	4.24868	3.78761	3.36571	3.98405	4.54844	1.91805	3.61702	1.81558	3.28181	4.04311	4.40936	3.95828	3.84886	3.38250	3.19214	1.24551	5.23093	3.96224	220 v - - -
	2.68618	4.42225	2.77519	2.73123	3.46354	2.40513	3.72494	3.29354	2.67741	2.69355	4.24690	2.90347	2.73739	3.18146	2.89801	2.37887	2.77519	2.98518	4.58477	3.61503	
	0.03268	3.83303	4.55537	0.61958	0.77255	0.48576	0.95510														
221	2.39269	4.23746	3.39425	3.14521	3.93517	3.07784	4.16539	3.05754	3.08739	2.92240	3.97601	3.36046	3.77873	3.48436	3.34758	2.59359	1.24059	2.75673	5.37648	4.15199	221 t - - -
	2.68618	4.42225	2.77519	2.73123	3.46354	2.40513	3.72494	3.29354	2.67741	2.69355	4.24690	2.90347	2.73739	3.18146	2.89801	2.37887	2.77519	2.98518	4.58477	3.61503	
	0.03268	3.83303	4.55537	0.61958	0.77255	0.48576	0.95510														
222	2.91965	5.02079	2.47306	1.08285	4.36227	3.24954	3.83381	3.80837	2.70946	3.43216	4.40865	2.91254	3.85246	3.05076	3.11366	2.90651	3.22382	3.49656	5.54134	4.26436	222 e - - -
	2.68618	4.42225	2.77519	2.73123	3.46354	2.40513	3.72494	3.29354	2.67741	2.69355	4.24690	2.90347	2.73739	3.18146	2.89801	2.37887	2.77519	2.98518	4.58477	3.61503	
	0.03268	3.83303	4.55537	0.61958	0.77255	0.48576	0.95510														
223	3.27202	4.66041	4.03210	3.72890	2.62865	3.68698	3.85349	3.29453	3.45219	2.69596	3.93010	3.88606	4.20159	3.84191	3.58729	3.47606	3.57058	3.18378	0.96876	2.63291	223 w - - -
	2.68618	4.42225	2.77519	2.73123	3.46354	2.40513	3.72494	3.29354	2.67741	2.69355	4.24690	2.90347	2.73739	3.18146	2.89801	2.37887	2.77519	2.98518	4.58477	3.61503	
	0.03268	3.83303	4.55537	0.61958	0.77255	0.48576	0.95510														
224	1.07216	4.17353	3.42348	3.21371	4.01396	2.99897	4.24029	3.13365	3.22896	3.01700	4.05375	3.37300	3.73453	3.57391	3.48180	2.52446	2.79912	2.79493	5.44509	4.24110	224 a - - -
	2.68618	4.42225	2.77519	2.73123	3.46354	2.40513	3.72494	3.29354	2.67741	2.69355	4.24690	2.90347	2.73739	3.18146	2.89801	2.37887	2.77519	2.98518	4.58477	3.61503	
	0.03268	3.83303	4.55537	0.61958	0.77255	0.48576	0.95510														
225	1.66413	4.10371	3.27609	3.01370	4.13402	2.88520	4.10185	3.46564	3.06729	3.22871	4.09669	3.19913	3.62419	3.37970	3.37403	1.56192	2.64793	2.98700	5.50483	4.26923	225 s - - -
	2.68618	4.42225	2.77519	2.73123	3.46354	2.40513	3.72494	3.29354	2.67741	2.69355	4.24690	2.90347	2.73739	3.18146	2.89801	2.37887	2.77519	2.98518	4.58477	3.61503	
	0.03268	3.83303	4.55537	0.61958	0.77255	0.48576	0.95510														
226	1.07216	4.17353	3.42348	3.21371	4.01396	2.99897	4.24029	3.13365	3.22896	3.01700	4.05375	3.37300	3.73453	3.57391	3.48180	2.52446	2.79912	2.79493	5.44509	4.24110	226 a - - -
	2.68618	4.42225	2.77519	2.73123	3.46354	2.40513	3.72494	3.29354	2.67741	2.69355	4.24690	2.90347	2.73739	3.18146	2.89801	2.37887	2.77519	2.98518	4.58477	3.61503	
	0.03268	3.83303	4.55537	0.61958	0.77255	0.48576	0.95510														
227	2.69182	4.68600	2.68850	2.57033	4.04344	3.15836	3.89101	3.74595	2.79175	3.39751	4.35350	1.22987	3.80860	3.16424	3.14460	2.76087	3.07272	3.37215	5.35398	3.99503	227 n - - -
	2.68618	4.42225	2.77519	2.73123	3.46354	2.40513	3.72494	3.29354	2.67741	2.69355	4.24690	2.90347	2.73739	3.18146	2.89801	2.37887	2.77519	2.98518	4.58477	3.61503	
	0.03268	3.83303	4.55537	0.61958	0.77255	0.48576	0.95510														
228	2.61765	4.39623	3.28327	3.17805	4.31966	0.79266	4.30013	3.91025	3.35388	3.56921	4.54515	3.44774	3.78610	3.68127	3.59365	2.78855	3.09584	3.44624	5.42460	4.42045	228 g - - -
	2.68618	4.42225	2.77519	2.73123	3.46354	2.40513	3.72494	3.29354	2.67741	2.69355	4.24690	2.90347	2.73739	3.18146	2.89801	2.37887	2.77519	2.98518	4.58477	3.61503	
	0.03268	3.83303	4.55537	0.61958	0.77255	0.48576	0.95510														
229	2.94831	5.06869	0.97632	2.31258	4.44069	3.18175	3.89799	4.02012	2.97493	3.63580	4.61368	2.86669	3.83184	3.13755	3.48525	2.91560	3.28628	3.66374	5.61691	4.32095	229 d - - -
	2.68618	4.42225	2.77519	2.73123	3.46354	2.40513	3.72494	3.29354	2.67741	2.69355	4.24690	2.90347	2.73739	3.18146	2.89801	2.37887	2.77519	2.98518	4.58477	3.61503	
	0.03268	3.83303	4.55537	0.61958	0.77255	0.48576	0.95510														
230	2.91965	5.02079	2.47306	1.08285	4.36227	3.24954	3.83381	3.80837	2.70946	3.43216	4.40865	2.91254	3.85246	3.05076	3.11366	2.90651	3.22382	3.49656	5.54134	4.26436	230 e - - -
	2.68618	4.42225	2.77519	2.73123	3.46354	2.40513	3.72494	3.29354	2.67741	2.69355	4.24690	2.90347	2.73739	3.18146	2.89801	2.37887	2.77519	2.98518	4.58477	3.61503	
	0.03268	3.83303	4.55537	0.61958	0.77255	0.48576	0.95510														
231	3.02614	4.42208	4.44790	3.93184	3.24605	4.22380	4.63664	1.30355	3.75694	1.73900	2.75597	4.21009	4.53513	4.04514	3.96589	3.58767	3.28169	1.83451	5.19675	4.00254	231 i - - -
	2.68618	4.42225	2.77519	2.73123	3.46354	2.40513	3.72494	3.29354	2.67741	2.69355	4.24690	2.90347	2.73739	3.18146	2.89801	2.37887	2.77519	2.98518	4.58477	3.61503	
	0.03268	3.83303	4.55537	0.61958	0.77255	0.48576	0.95510														
232	2.39269	4.23746	3.39425	3.14521	3.93517	3.07784	4.16539	3.05754	3.08739	2.92240	3.97601	3.36046	3.77873	3.48436	3.34758	2.59359	1.24059	2.75673	5.37648	4.15199	232 t - - -
	2.68618	4.42225	2.77519	2.73123	3.46354	2.40513	3.72494	3.29354	2.67741	2.69355	4.24690	2.90347	2.73739	3.18146	2.89801	2.37887	2.77519	2.98518	4.58477	3.61503	
	0.03268	3.83303	4.55537	0.61958	0.77255	0.48576	0.95510														
233	2.70125	4.46443	3.30428	3.13815	4.13555	3.15421	4.21835	3.67141	3.19219	3.30231	4.35093	3.44851	0.90433	3.58220	3.44165	2.86766	3.13986	3.32122	5.33234	4.25589	233 p - - -
	2.68618	4.42225	2.77519	2.73123	3.46354	2.40513	3.72494	3.29354	2.67741	2.69355	4.24690	2.90347	2.73739	3.18146	2.89801	2.37887	2.77519	2.98518	4.58477	3.61503	
	0.03268	3.83303	4.55537	0.61958	0.77255	0.48576	0.95510														
234	2.39269	4.23746	3.39425	3.14521	3.93517	3.07784	4.16539	3.05754	3.08739	2.92240	3.97601	3.36046	3.77873	3.48436	3.34758	2.59359	1.24059	2.75673	5.37648	4.15199	234 t - - -
	2.68618	4.42225	2.77519	2.73123	3.46354	2.40513	3.72494	3.29354	2.67741	2.69355	4.24690	2.90347	2.73739	3.18146	2.89801	2.37887	2.77519	2.98518	4.58477	3.61503	
	0.03268	3.83303	4.55537	0.61958	0.77255	0.48576	0.95510														
235	2.77713	4.33135	4.07016	3.73272	3.52199	3.68239	4.54148	2.05456	3.59987	2.21635	3.49380	3.92869	4.24556	3.96282	3.81605	3.18396	3.13994	1.07623	5.29954	4.02841	235 v - - -
	2.68618	4.42225	2.77519	2.73123	3.46354	2.40513	3.72494	3.29354	2.67741	2.69355	4.24690	2.90347	2.73739	3.18146	2.89801	2.37887	2.77519	2.98518	4.58477	3.61503	
	0.03268	3.83303	4.55537	0.61958	0.77255	0.48576	0.95510														
236	3.20912	4.62273	4.18444	3.89199	1.07857	3.90702	3.86137	2.73830	3.80015	2.11778	3.47472	3.97513	4.36694	3.97918	3.92960	3.48072	3.51047	2.72599	4.12320	2.49559	236 f - - -
	2.68618	4.42225	2.77519	2.73123	3.46354	2.40513	3.72494	3.29354	2.67741	2.69355	4.24690	2.90347	2.73739	3.18146	2.89801	2.37887	2.77519	2.98518	4.58477	3.61503	
	0.03268	3.83303	4.55537	0.61958	0.77255	0.48576	0.95510														
237	3.11165	4.58599	4.12585	3.76620	3.12182	3.93147	4.43434	2.32453	3.53431	0.92536	3.15834	4.04543	4.37407	3.91210	3.71656	3.49871	3.40796	2.35149	4.98612	3.70011	237 l - - -
	2.68618	4.42225	2.77519	2.73123	3.46354	2.40513	3.72494	3.29354	2.67741	2.69355	4.24690	2.90347	2.73739	3.18146	2.89801	2.37887	2.77519	2.98518	4.58477	3.61503	
	0.03268	3.83303	4.55537	0.61958	0.77255	0.48576	0.95510														
238	2.29672	4.21624	3.17324	2.98785	4.03735	2.94690	4.09847	3.55494	3.05893	3.27847	4.20833	3.21852	3.68259	3.41149	3.34573	1.15212	2.77402	3.09870	5.41316	4.12083	238 s - - -
	2.68618	4.42225	2.77519	2.73123	3.46354	2.40513	3.72494	3.29354	2.67741	2.69355	4.24690	2.90347	2.73739	3.18146	2.89801	2.37887	2.77519	2.98518	4.58477	3.61503	
	0.03268	3.83303	4.55537	0.61958	0.77255	0.48576	0.95510														
239	2.97830	4.36070	4.50257	4.01061	3.41784	4.21178	4.75955	1.34036	3.88027	1.93804	3.25287	4.26172	4.56420	4.16914	4.10155	3.59350	3.25272	1.41646	5.35369	4.13968	239 i - - -
	2.68618	4.42225	2.77519	2.73123	3.46354	2.40513	3.72494	3.29354	2.67741	2.69355	4.24690	2.90347	2.73739	3.18146	2.89801	2.37887	2.77519	2.98518	4.58477	3.61503	
	0.03268	3.83303	4.55537	0.61958	0.77255	0.48576	0.95510														
240	2.91965	5.02079	2.47306	1.08285	4.36227	3.24954	3.83381	3.80837	2.70946	3.43216	4.40865	2.91254	3.85246	3.05076	3.11366	2.90651	3.22382	3.49656	5.54134	4.26436	240 e - - -
	2.68618	4.42225	2.77519	2.73123	3.46354	2.40513	3.72494	3.29354	2.67741	2.69355	4.24690	2.90347	2.73739	3.18146	2.89801	2.37887	2.77519	2.98518	4.58477	3.61503	
	0.03268	3.83303	4.55537	0.61958	0.77255	0.48576	0.95510														
241	2.39269	4.23746	3.39425	3.14521	3.93517	3.07784	4.16539	3.05754	3.08739	2.92240	3.97601	3.36046	3.77873	3.48436	3.34758	2.59359	1.24059	2.75673	5.37648	4.15199	241 t - - -
	2.68618	4.42225	2.77519	2.73123	3.46354	2.40513	3.72494	3.29354	2.67741	2.69355	4.24690	2.90347	2.73739	3.18146	2.89801	2.37887	2.77519	2.98518	4.58477	3.61503	
	0.03268	3.83303	4.55537	0.61958	0.77255	0.48576	0.95510														
242	2.94831	5.06869	0.97632	2.31258	4.44069	3.18175	3.89799	4.02012	2.97493	3.63580	4.61368	2.86669	3.83184	3.13755	3.48525	2.91560	3.28628	3.66374	5.61691	4.32095	242 d - - -

```
        2.68618  4.42225  2.77519  2.73123  3.46354  2.40513  3.72494  3.29354  2.67741  2.69355  4.24690  2.90347  2.73739  3.18146  2.89801  2.37887  2.77519  2.98518  4.58477  3.61503
        0.03268  3.83303  4.55537  0.61958  0.77255  0.48576  0.95510
 243    2.91965  5.02079  2.47306  1.08285  4.36227  3.24954  3.83381  3.80837  2.70946  3.43216  4.40865  2.91254  3.85246  3.05076  3.11366  2.90651  3.22382  3.49656  5.54134  4.26436    243 e - - -
        2.68618  4.42225  2.77519  2.73123  3.46354  2.40513  3.72494  3.29354  2.67741  2.69355  4.24690  2.90347  2.73739  3.18146  2.89801  2.37887  2.77519  2.98518  4.58477  3.61503
        0.03268  3.83303  4.55537  0.61958  0.77255  0.48576  0.95510
 244    3.11165  4.58599  4.12585  3.76620  3.12182  3.93147  4.43434  2.32453  3.53431  0.92536  3.15834  4.04543  4.37407  3.91210  3.71656  3.49871  3.40796  2.35149  4.98612  3.70011    244 l - - -
        2.68618  4.42225  2.77519  2.73123  3.46354  2.40513  3.72494  3.29354  2.67741  2.69355  4.24690  2.90347  2.73739  3.18146  2.89801  2.37887  2.77519  2.98518  4.58477  3.61503
        0.03268  3.83303  4.55537  0.61958  0.77255  0.48576  0.95510
 245    2.96214  4.80276  3.44889  2.96907  4.14736  3.45685  3.79770  3.68689  2.22438  3.21005  4.21188  3.33151  3.96192  3.02053  1.04043  3.04858  3.23116  3.41270  5.24678  4.08393    245 r - - -
        2.68618  4.42225  2.77519  2.73123  3.46354  2.40513  3.72494  3.29354  2.67741  2.69355  4.24690  2.90347  2.73739  3.18146  2.89801  2.37887  2.77519  2.98518  4.58477  3.61503
        0.03268  3.83303  4.55537  0.61958  0.77255  0.48576  0.95510
 246    2.95371  4.77708  3.08911  2.84570  3.25855  3.41543  1.22850  3.65045  2.68990  3.14913  4.18951  3.25638  3.96612  3.21160  2.95766  3.02494  3.25600  3.38639  4.72403  3.21065    246 h - - -
        2.68618  4.42225  2.77519  2.73123  3.46354  2.40513  3.72494  3.29354  2.67741  2.69355  4.24690  2.90347  2.73739  3.18146  2.89801  2.37887  2.77519  2.98518  4.58477  3.61503
        0.03268  3.83303  4.55537  0.61958  0.77255  0.48576  0.95510
 247    2.93904  4.49431  4.00741  3.59844  3.19183  3.80469  4.34274  2.36355  3.36846  1.79296  1.45569  3.88063  4.26555  3.75324  3.57830  3.28378  3.24383  2.36495  5.02707  3.78505    247 m - - -
        2.68618  4.42225  2.77519  2.73123  3.46354  2.40513  3.72494  3.29354  2.67741  2.69355  4.24690  2.90347  2.73739  3.18146  2.89801  2.37887  2.77519  2.98518  4.58477  3.61503
        0.03268  3.83303  4.55537  0.61958  0.77255  0.48576  0.95510
 248    1.07216  4.17353  3.42348  3.21371  4.01396  2.99897  4.24029  3.13365  3.22896  3.01700  4.05375  3.37300  3.73453  3.57391  3.48180  2.52446  2.79912  2.79493  5.44509  4.24110    248 a - - -
        2.68618  4.42225  2.77519  2.73123  3.46354  2.40513  3.72494  3.29354  2.67741  2.69355  4.24690  2.90347  2.73739  3.18146  2.89801  2.37887  2.77519  2.98518  4.58477  3.61503
        0.03268  3.83303  4.55537  0.61958  0.77255  0.48576  0.95510
 249    2.69182  4.68600  2.68850  2.57033  4.04344  3.15836  3.89101  3.74595  2.79175  3.39751  4.35350  1.22987  3.80860  3.16424  3.14460  2.76087  3.07272  3.37215  5.35398  3.99503    249 n - - -
        2.68618  4.42225  2.77519  2.73123  3.46354  2.40513  3.72494  3.29354  2.67741  2.69355  4.24690  2.90347  2.73739  3.18146  2.89801  2.37887  2.77519  2.98518  4.58477  3.61503
        0.03268  3.83303  4.55537  0.61958  0.77255  0.48576  0.95510
 250    2.61765  4.39623  3.28327  3.17805  4.31966  0.79266  4.30013  3.91025  3.35388  3.56921  4.54515  3.44774  3.78610  3.68127  3.59365  2.78855  3.09584  3.44624  5.42460  4.42045    250 g - - -
        2.68618  4.42225  2.77519  2.73123  3.46354  2.40513  3.72494  3.29354  2.67741  2.69355  4.24690  2.90347  2.73739  3.18146  2.89801  2.37887  2.77519  2.98518  4.58477  3.61503
        0.03268  3.83303  4.55537  0.61958  0.77255  0.48576  0.95510
 251    3.19134  4.69630  3.78054  3.50310  2.29611  3.78460  3.60201  3.20618  3.36176  2.67014  3.88549  3.67575  4.25966  3.67727  3.55331  3.33175  3.47801  3.07495  3.93816  1.03399    251 y - - -
        2.68618  4.42225  2.77519  2.73123  3.46354  2.40513  3.72494  3.29354  2.67741  2.69355  4.24690  2.90347  2.73739  3.18146  2.89801  2.37887  2.77519  2.98518  4.58477  3.61503
        0.03268  3.83303  4.55537  0.61958  0.77255  0.48576  0.95510
 252    2.85084  4.83409  2.87832  2.63320  3.99721  3.34074  3.79090  3.62260  2.45691  3.12880  4.15852  3.09230  3.89411  1.30591  2.74155  2.90061  3.14819  3.35495  5.27660  3.96796    252 q - - -
        2.68618  4.42225  2.77519  2.73123  3.46354  2.40513  3.72494  3.29354  2.67741  2.69355  4.24690  2.90347  2.73739  3.18146  2.89801  2.37887  2.77519  2.98518  4.58477  3.61503
        0.03268  3.83303  4.55537  0.61958  0.77255  0.48576  0.95510
 253    2.39269  4.23746  3.39425  3.14521  3.93517  3.07784  4.16539  3.05754  3.08739  2.92240  3.97601  3.36046  3.77873  3.48436  3.34758  2.59359  1.24059  2.75673  5.37648  4.15199    253 t - - -
        2.68618  4.42225  2.77519  2.73123  3.46354  2.40513  3.72494  3.29354  2.67741  2.69355  4.24690  2.90347  2.73739  3.18146  2.89801  2.37887  2.77519  2.98518  4.58477  3.61503
        0.03268  3.83303  4.55537  0.61958  0.77255  0.48576  0.95510
 254    2.93575  4.34758  4.41945  3.93447  3.44348  4.12899  4.70350  1.61127  3.80150  1.99682  3.29431  4.18626  4.51214  4.10744  4.03367  3.51330  3.21946  1.20644  5.34803  4.11683    254 v - - -
        2.68618  4.42225  2.77519  2.73123  3.46354  2.40513  3.72494  3.29354  2.67741  2.69355  4.24690  2.90347  2.73739  3.18146  2.89801  2.37887  2.77519  2.98518  4.58477  3.61503
        0.03268  3.83303  4.55537  0.61958  0.77255  0.48576  0.95510
 255    2.77713  4.33135  4.07016  3.73272  3.52199  3.68239  4.54148  2.05456  3.59987  2.21635  3.49380  3.92869  4.24556  3.96282  3.81605  3.18396  3.13994  1.07623  5.29954  4.02841    255 v - - -
        2.68618  4.42225  2.77519  2.73123  3.46354  2.40513  3.72494  3.29354  2.67741  2.69355  4.24690  2.90347  2.73739  3.18146  2.89801  2.37887  2.77519  2.98518  4.58477  3.61503
        0.03268  3.83303  4.55537  0.61958  0.77255  0.48576  0.95510
 256    2.29672  4.21624  3.17324  2.98785  4.03735  2.94690  4.09847  3.55494  3.05893  3.27847  4.20833  3.21852  3.68259  3.41149  3.34573  1.15212  2.77402  3.09870  5.41316  4.12083    256 s - - -
        2.68618  4.42225  2.77519  2.73123  3.46354  2.40513  3.72494  3.29354  2.67741  2.69355  4.24690  2.90347  2.73739  3.18146  2.89801  2.37887  2.77519  2.98518  4.58477  3.61503
        0.03268  3.83303  4.55537  0.61958  0.77255  0.48576  0.95510
 257    3.00047  4.43103  4.27833  3.89317  3.34832  4.02286  4.62907  1.12816  3.72049  1.94320  3.29060  4.14408  4.45846  4.07325  3.92162  3.51489  3.30059  1.84674  5.23051  3.96495    257 i - - -
        2.68618  4.42225  2.77519  2.73123  3.46354  2.40513  3.72494  3.29354  2.67741  2.69355  4.24690  2.90347  2.73739  3.18146  2.89801  2.37887  2.77519  2.98518  4.58477  3.61503
        0.03268  3.83303  4.55537  0.61958  0.77255  0.48576  0.95510
 258    1.57850  4.24746  3.54787  3.11428  3.61541  3.30254  4.06319  2.62460  3.00478  2.45806  2.95188  3.39548  3.88677  3.37054  3.30456  2.69600  2.82952  2.42162  5.18399  3.94009    258 a - - -
        2.68618  4.42225  2.77519  2.73123  3.46354  2.40513  3.72494  3.29354  2.67741  2.69355  4.24690  2.90347  2.73739  3.18146  2.89801  2.37887  2.77519  2.98518  4.58477  3.61503
        0.03268  3.83303  4.55537  0.61958  0.77255  0.48576  0.95510
 259    2.69182  4.68600  2.68850  2.57033  4.04344  3.15836  3.89101  3.74595  2.79175  3.39751  4.35350  1.22987  3.80860  3.16424  3.14460  2.76087  3.07272  3.37215  5.35398  3.99503    259 n - - -
        2.68618  4.42225  2.77519  2.73123  3.46354  2.40513  3.72494  3.29354  2.67741  2.69355  4.24690  2.90347  2.73739  3.18146  2.89801  2.37887  2.77519  2.98518  4.58477  3.61503
        0.03268  3.83303  4.55537  0.61958  0.77255  0.48576  0.95510
 260    2.81624  5.21169  1.36059  2.09950  4.53004  3.13779  3.72956  4.09054  2.75109  3.65208  4.52578  2.33680  3.77138  2.91654  3.31144  2.74391  3.13023  3.68068  5.77175  4.29845    260 d - - -
        2.68618  4.42225  2.77519  2.73123  3.46354  2.40513  3.72494  3.29354  2.67741  2.69355  4.24690  2.90347  2.73739  3.18146  2.89801  2.37887  2.77519  2.98518  4.58477  3.61503
        0.03268  3.83303  4.55537  0.61958  0.77255  0.48576  0.95510
 261    2.65134  4.67722  2.70434  2.23781  4.16275  3.20190  3.81871  3.57174  2.65398  3.22028  4.13540  2.98106  1.65737  3.03762  3.02530  2.71920  2.98721  3.23283  5.42121  4.14678    261 p - - -
        2.68618  4.42225  2.77519  2.73123  3.46354  2.40513  3.72494  3.29354  2.67741  2.69355  4.24690  2.90347  2.73739  3.18146  2.89801  2.37887  2.77519  2.98518  4.58477  3.61503
        0.03268  3.83303  4.55537  0.61958  0.77255  0.48576  0.95510
 262    1.55070  4.48995  2.82281  2.29710  4.04957  3.16330  3.85261  3.27069  2.71762  3.05791  3.98053  3.02138  3.77293  3.07899  3.08856  2.62075  2.86732  2.95081  5.39863  4.11257    262 a - - -
        2.68618  4.42225  2.77519  2.73123  3.46354  2.40513  3.72494  3.29354  2.67741  2.69355  4.24690  2.90347  2.73739  3.18146  2.89801  2.37887  2.77519  2.98518  4.58477  3.61503
        0.03268  3.83303  4.55537  0.61958  0.77255  0.48576  0.95510
 263    1.66759  4.09319  3.34133  3.01786  4.10058  2.90235  4.07522  3.40569  3.03578  3.16925  4.02438  3.20529  3.62494  3.34622  3.35173  1.76670  2.29078  2.94325  5.46635  4.24765    263 a - - -
        2.68618  4.42225  2.77519  2.73123  3.46354  2.40513  3.72494  3.29354  2.67741  2.69355  4.24690  2.90347  2.73739  3.18146  2.89801  2.37887  2.77519  2.98518  4.58477  3.61503
        0.03268  3.83303  4.55537  0.61958  0.77255  0.48576  0.95510
 264    1.92860  4.36865  3.18733  2.70434  3.69712  3.30820  3.75803  3.02407  2.62753  2.72977  3.13420  3.11618  3.79869  2.78448  2.99000  2.45310  2.76717  2.75087  5.08908  3.82713    264 a - - -
        2.68618  4.42225  2.77519  2.73123  3.46354  2.40513  3.72494  3.29354  2.67741  2.69355  4.24690  2.90347  2.73739  3.18146  2.89801  2.37887  2.77519  2.98518  4.58477  3.61503
```

	0.03268	3.83303	4.55537	0.61958	0.77255	0.48576	0.95510														
265	2.93703	4.89791	3.11032	2.74002	4.27531	3.43004	3.73906	3.70226	1.09362	3.27630	4.24050	3.16601	3.93369	2.93324	2.42654	2.98198	3.19357	3.41249	5.34669	4.15401	265 k - - -
	2.68618	4.42225	2.77519	2.73123	3.46354	2.40513	3.72494	3.29354	2.67741	2.69355	4.24690	2.90347	2.73739	3.18146	2.89801	2.37887	2.77519	2.98518	4.58477	3.61503	
	0.03268	3.83303	4.55537	0.61958	0.77255	0.48576	0.95510														
266	3.30850	4.68017	4.22725	3.84567	1.67285	4.10948	3.43803	3.06991	3.71723	2.46671	3.69976	3.82562	4.45024	3.82505	3.85750	3.46537	3.54195	2.98044	3.64493	1.24451	266 y - - -
	2.68618	4.42225	2.77519	2.73123	3.46354	2.40513	3.72494	3.29354	2.67741	2.69355	4.24690	2.90347	2.73739	3.18146	2.89801	2.37887	2.77519	2.98518	4.58477	3.61503	
	0.03268	3.83303	4.55537	0.61958	0.77255	0.48576	0.95510														
267	3.11165	4.58599	4.12585	3.76620	3.12182	3.93147	4.43434	2.32453	3.53431	0.92536	3.15834	4.04543	4.37407	3.91210	3.71656	3.49871	3.40796	2.35149	4.98612	3.70011	267 l - - -
	2.68618	4.42225	2.77519	2.73123	3.46354	2.40513	3.72494	3.29354	2.67741	2.69355	4.24690	2.90347	2.73739	3.18146	2.89801	2.37887	2.77519	2.98518	4.58477	3.61503	
	0.03268	3.83303	4.55537	0.61958	0.77255	0.48576	0.95510														
268	2.72473	4.91338	2.50849	2.33063	4.19111	3.23132	3.69396	3.78014	2.47148	3.34744	4.23007	1.69633	3.80195	2.52397	2.85127	2.72722	3.02350	3.41943	5.44938	4.05730	268 n - - -
	2.68618	4.42225	2.77519	2.73123	3.46354	2.40513	3.72494	3.29354	2.67741	2.69355	4.24690	2.90347	2.73739	3.18146	2.89801	2.37887	2.77519	2.98518	4.58477	3.61503	
	0.03268	3.83303	4.55537	0.61958	0.77255	0.48576	0.95510														
269	2.39269	4.23746	3.39425	3.14521	3.93517	3.07784	4.16539	3.05754	3.08739	2.92240	3.97601	3.36046	3.77873	3.48436	3.34758	2.59359	1.24059	2.75673	5.37648	4.15199	269 t - - -
	2.68618	4.42225	2.77519	2.73123	3.46354	2.40513	3.72494	3.29354	2.67741	2.69355	4.24690	2.90347	2.73739	3.18146	2.89801	2.37887	2.77519	2.98518	4.58477	3.61503	
	0.03268	3.83303	4.55537	0.61958	0.77255	0.48576	0.95510														
270	2.94831	5.06869	0.97632	2.31258	4.44069	3.18175	3.89799	4.02012	2.97493	3.63580	4.61368	2.86669	3.83184	3.13755	3.48525	2.91560	3.28628	3.66374	5.61691	4.32095	270 d - - -
	2.68618	4.42225	2.77519	2.73123	3.46354	2.40513	3.72494	3.29354	2.67741	2.69355	4.24690	2.90347	2.73739	3.18146	2.89801	2.37887	2.77519	2.98518	4.58477	3.61503	
	0.03268	3.83303	4.55537	0.61958	0.77255	0.48576	0.95510														
271	3.11165	4.58599	4.12585	3.76620	3.12182	3.93147	4.43434	2.32453	3.53431	0.92536	3.15834	4.04543	4.37407	3.91210	3.71656	3.49871	3.40796	2.35149	4.98612	3.70011	271 l - - -
	2.68618	4.42225	2.77519	2.73123	3.46354	2.40513	3.72494	3.29354	2.67741	2.69355	4.24690	2.90347	2.73739	3.18146	2.89801	2.37887	2.77519	2.98518	4.58477	3.61503	
	0.03268	3.83303	4.55537	0.61958	0.77255	0.48576	0.95510														
272	2.74708	5.04402	2.04586	2.18135	4.38536	3.15677	3.72598	3.93810	2.68596	3.52178	4.38875	1.61091	3.76998	2.91578	3.19182	2.71228	3.06187	3.54286	5.64150	4.20748	272 n - - -
	2.68618	4.42225	2.77519	2.73123	3.46354	2.40513	3.72494	3.29354	2.67741	2.69355	4.24690	2.90347	2.73739	3.18146	2.89801	2.37887	2.77519	2.98518	4.58477	3.61503	
	0.03268	3.83303	4.55537	0.61958	0.77255	0.48576	0.95510														
273	2.69182	4.68600	2.68850	2.57033	4.04344	3.15836	3.89101	3.74595	2.79175	3.39751	4.35350	1.22987	3.80860	3.16424	3.14460	2.76087	3.07272	3.37215	5.35398	3.99503	273 n - - -
	2.68618	4.42225	2.77519	2.73123	3.46354	2.40513	3.72494	3.29354	2.67741	2.69355	4.24690	2.90347	2.73739	3.18146	2.89801	2.37887	2.77519	2.98518	4.58477	3.61503	
	0.03268	3.83303	4.55537	0.61958	0.77255	0.48576	0.95510														
274	1.07216	4.17353	3.42348	3.21371	4.01396	2.99897	4.24029	3.13365	3.22896	3.01700	4.05375	3.37300	3.73453	3.57391	3.48180	2.52446	2.79912	2.79493	5.44509	4.24110	274 a - - -
	2.68618	4.42225	2.77519	2.73123	3.46354	2.40513	3.72494	3.29354	2.67741	2.69355	4.24690	2.90347	2.73739	3.18146	2.89801	2.37887	2.77519	2.98518	4.58477	3.61503	
	0.03268	3.83303	4.55537	0.61958	0.77255	0.48576	0.95510														
275	3.20912	4.62273	4.18444	3.89199	1.07857	3.90702	3.86137	2.73830	3.80015	2.11778	3.47472	3.97513	4.36694	3.97918	3.92960	3.48072	3.51047	2.72599	4.12320	2.49559	275 f - - -
	2.68618	4.42225	2.77519	2.73123	3.46354	2.40513	3.72494	3.29354	2.67741	2.69355	4.24690	2.90347	2.73739	3.18146	2.89801	2.37887	2.77519	2.98518	4.58477	3.61503	
	0.03268	3.83303	4.55537	0.61958	0.77255	0.48576	0.95510														
276	3.27202	4.66041	4.03210	3.72890	2.62865	3.68698	3.85349	3.29453	3.45219	2.69596	3.93010	3.88606	4.20159	3.84191	3.58729	3.47606	3.57058	3.18378	0.96876	2.63291	276 w - - -
	2.68618	4.42225	2.77519	2.73123	3.46354	2.40513	3.72494	3.29354	2.67741	2.69355	4.24690	2.90347	2.73739	3.18146	2.89801	2.37887	2.77519	2.98518	4.58477	3.61503	
	0.03268	3.83303	4.55537	0.61958	0.77255	0.48576	0.95510														
277	2.39269	4.23746	3.39425	3.14521	3.93517	3.07784	4.16539	3.05754	3.08739	2.92240	3.97601	3.36046	3.77873	3.48436	3.34758	2.59359	1.24059	2.75673	5.37648	4.15199	277 t - - -
	2.68618	4.42225	2.77519	2.73123	3.46354	2.40513	3.72494	3.29354	2.67741	2.69355	4.24690	2.90347	2.73739	3.18146	2.89801	2.37887	2.77519	2.98518	4.58477	3.61503	
	0.03268	3.83303	4.55537	0.61958	0.77255	0.48576	0.95510														
278	2.85084	4.83409	2.87832	2.63320	3.99721	3.34074	3.79090	3.62260	2.45691	3.12880	4.15852	3.09230	3.89411	1.30591	2.74155	2.90061	3.14819	3.35495	5.27660	3.96796	278 q - - -
	2.68618	4.42225	2.77519	2.73123	3.46354	2.40513	3.72494	3.29354	2.67741	2.69355	4.24690	2.90347	2.73739	3.18146	2.89801	2.37887	2.77519	2.98518	4.58477	3.61503	
	0.03268	3.83303	4.55537	0.61958	0.77255	0.48576	0.95510														
279	2.79552	4.93468	2.79713	2.46526	3.91790	3.39040	3.01403	3.67483	2.24912	3.19408	4.09085	2.95263	3.86561	1.75570	2.56010	2.80296	3.03573	3.36394	5.19830	3.79529	279 q - - -
	2.68618	4.42225	2.77519	2.73123	3.46354	2.40513	3.72494	3.29354	2.67741	2.69355	4.24690	2.90347	2.73739	3.18146	2.89801	2.37887	2.77519	2.98518	4.58477	3.61503	
	0.03268	3.83303	4.55537	0.61958	0.77255	0.48576	0.95510														
280	2.93703	4.89791	3.11032	2.74002	4.27531	3.43004	3.73906	3.70226	1.09362	3.27630	4.24050	3.16601	3.93369	2.93324	2.42654	2.98198	3.19357	3.41249	5.34669	4.15401	280 k - - -
	2.68618	4.42225	2.77519	2.73123	3.46354	2.40513	3.72494	3.29354	2.67741	2.69355	4.24690	2.90347	2.73739	3.18146	2.89801	2.37887	2.77519	2.98518	4.58477	3.61503	
	0.03268	3.83303	4.55537	0.61958	0.77255	0.48576	0.95510														
281	3.28721	4.68247	4.16936	3.78810	1.78716	4.07978	3.44718	3.07007	3.66031	2.47715	3.70821	3.79946	4.43049	3.79350	3.81617	3.44017	3.52462	2.97669	3.66742	1.19862	281 y - - -
	2.68618	4.42225	2.77519	2.73123	3.46354	2.40513	3.72494	3.29354	2.67741	2.69355	4.24690	2.90347	2.73739	3.18146	2.89801	2.37887	2.77519	2.98518	4.58477	3.61503	
	0.03268	3.83303	4.55537	0.61958	0.77255	0.48576	0.95510														
282	3.16565	4.57542	4.32949	3.89837	1.46633	4.12523	3.93908	2.44916	3.75317	1.64553	3.17551	4.02445	4.46331	3.92659	3.90981	3.51146	3.41891	2.47589	4.21808	2.62273	282 f - - -
	2.68618	4.42225	2.77519	2.73123	3.46354	2.40513	3.72494	3.29354	2.67741	2.69355	4.24690	2.90347	2.73739	3.18146	2.89801	2.37887	2.77519	2.98518	4.58477	3.61503	
	0.03268	3.83303	4.55537	0.61958	0.77255	0.48576	0.95510														
283	2.39269	4.23746	3.39425	3.14521	3.93517	3.07784	4.16539	3.05754	3.08739	2.92240	3.97601	3.36046	3.77873	3.48436	3.34758	2.59359	1.24059	2.75673	5.37648	4.15199	283 t - - -
	2.68618	4.42225	2.77519	2.73123	3.46354	2.40513	3.72494	3.29354	2.67741	2.69355	4.24690	2.90347	2.73739	3.18146	2.89801	2.37887	2.77519	2.98518	4.58477	3.61503	
	0.03268	3.83303	4.55537	0.61958	0.77255	0.48576	0.95510														
284	2.70125	4.46443	3.30428	3.13815	4.13555	3.15421	4.21835	3.67141	3.19219	3.30231	4.35093	3.44851	0.90433	3.58220	3.44165	2.86766	3.13986	3.32122	5.33234	4.25589	284 p - - -
	2.68618	4.42225	2.77519	2.73123	3.46354	2.40513	3.72494	3.29354	2.67741	2.69355	4.24690	2.90347	2.73739	3.18146	2.89801	2.37887	2.77519	2.98518	4.58477	3.61503	
	0.03268	3.83303	4.55537	0.61958	0.77255	0.48576	0.95510														
285	2.42454	4.24461	4.00278	3.50278	2.86460	3.73729	4.25845	2.09998	3.41769	2.11561	3.29333	3.76543	4.18503	3.69601	3.67795	3.08782	2.99008	1.48503	5.02340	3.75428	285 v - - -
	2.68618	4.42225	2.77519	2.73123	3.46354	2.40513	3.72494	3.29354	2.67741	2.69355	4.24690	2.90347	2.73739	3.18146	2.89801	2.37887	2.77519	2.98518	4.58477	3.61503	
	0.03268	3.83303	4.55537	0.61958	0.77255	0.48576	0.95510														
286	3.01085	4.48483	4.28454	3.80764	3.14253	4.07424	4.51230	2.08521	3.60714	1.15008	3.04874	4.08584	4.44363	3.92137	3.81791	3.46449	3.28821	1.89343	5.08889	3.85273	286 l - - -
	2.68618	4.42225	2.77519	2.73123	3.46354	2.40513	3.72494	3.29354	2.67741	2.69355	4.24690	2.90347	2.73739	3.18146	2.89801	2.37887	2.77519	2.98518	4.58477	3.61503	
	0.03268	3.83303	4.55537	0.61958	0.77255	0.48576	0.95510														

287	2.61765	4.39623	3.28327	3.17805	4.31966	0.79266	4.30013	3.91025	3.35388	3.56921	4.54515	3.44774	3.78610	3.68127	3.59365	2.78855	3.09584	3.44624	5.42460	4.42045	287 g - - -
	2.68618	4.42225	2.77519	2.73123	3.46354	2.40513	3.72494	3.29354	2.67741	2.69355	4.24690	2.90347	2.73739	3.18146	2.89801	2.37887	2.77519	2.98518	4.58477	3.61503	
	0.03268	3.83303	4.55537	0.61958	0.77255	0.48576	0.95510														
288	2.41907	4.26330	3.62691	3.09372	3.11653	3.60922	3.85727	2.56557	2.97261	2.21088	2.51234	3.43506	4.00442	3.29799	3.26236	2.90196	2.87706	2.41237	4.69620	2.54988	288 l - - -
	2.68618	4.42225	2.77519	2.73123	3.46354	2.40513	3.72494	3.29354	2.67741	2.69355	4.24690	2.90347	2.73739	3.18146	2.89801	2.37887	2.77519	2.98518	4.58477	3.61503	
	0.03268	3.83303	4.55537	0.61958	0.77255	0.48576	0.95510														
289	2.53550	4.38902	4.00901	3.48158	3.21880	3.88718	4.27348	2.20437	3.31416	1.35813	2.77965	3.80497	4.26068	3.63970	3.56903	3.21863	3.10410	2.15734	5.03843	3.84802	289 l - - -
	2.68618	4.42225	2.77519	2.73123	3.46354	2.40513	3.72494	3.29354	2.67741	2.69355	4.24690	2.90347	2.73739	3.18146	2.89801	2.37887	2.77519	2.98518	4.58477	3.61503	
	0.03268	3.83303	4.55537	0.61958	0.77255	0.48576	0.95510														
290	3.16565	4.57542	4.32949	3.89837	1.46633	4.12523	3.93908	2.44916	3.75317	1.64553	3.17551	4.02445	4.46331	3.92659	3.90981	3.51146	3.41891	2.47589	4.21808	2.62273	290 f - - -
	2.68618	4.42225	2.77519	2.73123	3.46354	2.40513	3.72494	3.29354	2.67741	2.69355	4.24690	2.90347	2.73739	3.18146	2.89801	2.37887	2.77519	2.98518	4.58477	3.61503	
	0.03268	3.83303	4.55537	0.61958	0.77255	0.48576	0.95510														
291	2.91965	5.02079	2.47306	1.08285	4.36227	3.24954	3.83381	3.80837	2.70946	3.43216	4.40865	2.91254	3.85246	3.05076	3.11366	2.90651	3.22382	3.49656	5.54134	4.26436	291 e - - -
	2.68618	4.42225	2.77519	2.73123	3.46354	2.40513	3.72494	3.29354	2.67741	2.69355	4.24690	2.90347	2.73739	3.18146	2.89801	2.37887	2.77519	2.98518	4.58477	3.61503	
	0.03268	3.83303	4.55537	0.61958	0.77255	0.48576	0.95510														
292	3.19134	4.69630	3.78054	3.50310	2.29611	3.78460	3.60201	3.20618	3.36176	2.67014	3.88549	3.67575	4.25966	3.67727	3.55331	3.33175	3.47801	3.07495	3.93816	1.03399	292 y - - -
	2.68618	4.42225	2.77519	2.73123	3.46354	2.40513	3.72494	3.29354	2.67741	2.69355	4.24690	2.90347	2.73739	3.18146	2.89801	2.37887	2.77519	2.98518	4.58477	3.61503	
	0.03268	3.83303	4.55537	0.61958	0.77255	0.48576	0.95510														
293	2.61765	4.39623	3.28327	3.17805	4.31966	0.79266	4.30013	3.91025	3.35388	3.56921	4.54515	3.44774	3.78610	3.68127	3.59365	2.78855	3.09584	3.44624	5.42460	4.42045	293 g - - -
	2.68618	4.42225	2.77519	2.73123	3.46354	2.40513	3.72494	3.29354	2.67741	2.69355	4.24690	2.90347	2.73739	3.18146	2.89801	2.37887	2.77519	2.98518	4.58477	3.61503	
	0.03268	3.83303	4.55537	0.61958	0.77255	0.48576	0.95510														
294	2.29672	4.21624	3.17324	2.98785	4.03735	2.94690	4.09847	3.55494	3.05893	3.27847	4.20833	3.21852	3.68259	3.41149	3.34573	1.15212	2.77402	3.09870	5.41316	4.12083	294 s - - -
	2.68618	4.42225	2.77519	2.73123	3.46354	2.40513	3.72494	3.29354	2.67741	2.69355	4.24690	2.90347	2.73739	3.18146	2.89801	2.37887	2.77519	2.98518	4.58477	3.61503	
	0.03268	3.83303	4.55537	0.61958	0.77255	0.48576	0.95510														
295	2.74000	4.82297	3.05712	2.55017	3.76954	3.47136	3.16627	3.48219	1.66964	3.04635	3.91799	3.01766	3.87278	2.77624	2.49024	2.77669	2.95467	3.18995	5.06852	3.15018	295 k - - -
	2.68618	4.42225	2.77519	2.73123	3.46354	2.40513	3.72494	3.29354	2.67741	2.69355	4.24690	2.90347	2.73739	3.18146	2.89801	2.37887	2.77519	2.98518	4.58477	3.61503	
	0.03268	3.83303	4.55537	0.61958	0.77255	0.48576	0.95510														
296	3.27048	4.64138	4.25823	3.87451	1.24758	4.10742	3.51349	2.92823	3.74392	2.30076	3.56638	3.86566	4.44830	3.85385	3.87583	3.47123	3.50746	2.87164	3.72551	1.81967	296 f - - -
	2.68618	4.42225	2.77519	2.73123	3.46354	2.40513	3.72494	3.29354	2.67741	2.69355	4.24690	2.90347	2.73739	3.18146	2.89801	2.37887	2.77519	2.98518	4.58477	3.61503	
	0.03268	3.83303	4.55537	0.61958	0.77255	0.48576	0.95510														
297	2.93703	4.89791	3.11032	2.74002	4.27531	3.43004	3.73906	3.70226	1.09362	3.27630	4.24050	3.16601	3.93369	2.93324	2.42654	2.98198	3.19357	3.41249	5.34669	4.15401	297 k - - -
	2.68618	4.42225	2.77519	2.73123	3.46354	2.40513	3.72494	3.29354	2.67741	2.69355	4.24690	2.90347	2.73739	3.18146	2.89801	2.37887	2.77519	2.98518	4.58477	3.61503	
	0.03268	3.83303	4.55537	0.61958	0.77255	0.48576	0.95510														
298	2.77713	4.33135	4.07016	3.73272	3.52199	3.68239	4.54148	2.05456	3.59987	2.21635	3.49380	3.92869	4.24556	3.96282	3.81605	3.18396	3.13994	1.07623	5.29954	4.02841	298 v - - -
	2.68618	4.42225	2.77519	2.73123	3.46354	2.40513	3.72494	3.29354	2.67741	2.69355	4.24690	2.90347	2.73739	3.18146	2.89801	2.37887	2.77519	2.98518	4.58477	3.61503	
	0.03268	3.83303	4.55537	0.61958	0.77255	0.48576	0.95510														
299	2.84347	5.27224	1.96615	1.36141	4.56663	3.17767	3.70122	4.04262	2.66829	3.60143	4.46940	2.67385	3.78186	2.87491	3.20659	2.76041	3.12981	3.65752	5.76303	4.31947	299 e - - -
	2.68618	4.42225	2.77519	2.73123	3.46354	2.40513	3.72494	3.29354	2.67741	2.69355	4.24690	2.90347	2.73739	3.18146	2.89801	2.37887	2.77519	2.98518	4.58477	3.61503	
	0.03268	3.83303	4.55537	0.61958	0.77255	0.48576	0.95510														
300	2.70125	4.46443	3.30428	3.13815	4.13555	3.15421	4.21835	3.67141	3.19219	3.30231	4.35093	3.44851	0.90433	3.58220	3.44165	2.86766	3.13986	3.32122	5.33234	4.25589	300 p - - -
	2.68618	4.42225	2.77519	2.73123	3.46354	2.40513	3.72494	3.29354	2.67741	2.69355	4.24690	2.90347	2.73739	3.18146	2.89801	2.37887	2.77519	2.98518	4.58477	3.61503	
	0.03268	3.83303	4.55537	0.61958	0.77255	0.48576	0.95510														
301	3.27202	4.66041	4.03210	3.72890	2.62865	3.68698	3.85349	3.29453	3.45219	2.69596	3.93010	3.88606	4.20159	3.84191	3.58729	3.47606	3.57058	3.18378	0.96876	2.63291	301 w - - -
	2.68618	4.42225	2.77519	2.73123	3.46354	2.40513	3.72494	3.29354	2.67741	2.69355	4.24690	2.90347	2.73739	3.18146	2.89801	2.37887	2.77519	2.98518	4.58477	3.61503	
	0.03268	3.83303	4.55537	0.61958	0.77255	0.48576	0.95510														
302	2.28632	4.22189	3.91607	3.53692	3.63679	3.49676	4.41425	2.12052	3.43851	2.35800	3.53720	3.72686	4.09222	3.77802	3.68977	2.92844	2.95647	1.29149	5.34662	4.10917	302 v - - -
	2.68618	4.42225	2.77519	2.73123	3.46354	2.40513	3.72494	3.29354	2.67741	2.69355	4.24690	2.90347	2.73739	3.18146	2.89801	2.37887	2.77519	2.98518	4.58477	3.61503	
	0.03268	3.83303	4.55537	0.61958	0.77255	0.48576	0.95510														
303	2.93703	4.89791	3.11032	2.74002	4.27531	3.43004	3.73906	3.70226	1.09362	3.27630	4.24050	3.16601	3.93369	2.93324	2.42654	2.98198	3.19357	3.41249	5.34669	4.15401	303 k - - -
	2.68618	4.42225	2.77519	2.73123	3.46354	2.40513	3.72494	3.29354	2.67741	2.69355	4.24690	2.90347	2.73739	3.18146	2.89801	2.37887	2.77519	2.98518	4.58477	3.61503	
	0.03268	3.83303	4.55537	0.61958	0.77255	0.48576	0.95510														
304	2.51552	4.29020	3.53305	3.07325	3.57552	3.37263	4.01833	2.60319	2.92729	2.41160	2.95564	3.38734	3.91820	3.31845	3.22825	2.74787	1.76434	2.41491	5.14310	3.89603	304 t - - -
	2.68618	4.42225	2.77519	2.73123	3.46354	2.40513	3.72494	3.29354	2.67741	2.69355	4.24690	2.90347	2.73739	3.18146	2.89801	2.37887	2.77519	2.98518	4.58477	3.61503	
	0.03268	3.83303	4.55537	0.61958	0.77255	0.48576	0.95510														
305	3.27202	4.66041	4.03210	3.72890	2.62865	3.68698	3.85349	3.29453	3.45219	2.69596	3.93010	3.88606	4.20159	3.84191	3.58729	3.47606	3.57058	3.18378	0.96876	2.63291	305 w - - -
	2.68618	4.42225	2.77519	2.73123	3.46354	2.40513	3.72494	3.29354	2.67741	2.69355	4.24690	2.90347	2.73739	3.18146	2.89801	2.37887	2.77519	2.98518	4.58477	3.61503	
	0.03268	3.83303	4.55537	0.61958	0.77255	0.48576	0.95510														
306	2.73173	4.98547	2.13946	2.21704	4.33588	3.15608	3.73933	3.89769	2.69011	3.49479	4.36826	1.55901	3.77232	2.93622	3.17727	2.71099	3.05371	3.50620	5.60307	4.17957	306 n - - -
	2.68618	4.42225	2.77519	2.73123	3.46354	2.40513	3.72494	3.29354	2.67741	2.69355	4.24690	2.90347	2.73739	3.18146	2.89801	2.37887	2.77519	2.98518	4.58477	3.61503	
	0.03268	3.83303	4.55537	0.61958	0.77255	0.48576	0.95510														
307	2.96214	4.80276	3.44889	2.96907	4.14736	3.45685	3.79770	3.68689	2.22438	3.21005	4.21188	3.33151	3.96192	3.02053	1.04043	3.04858	3.23116	3.41270	5.24678	4.08393	307 r - - -
	2.68618	4.42225	2.77519	2.73123	3.46354	2.40513	3.72494	3.29354	2.67741	2.69355	4.24690	2.90347	2.73739	3.18146	2.89801	2.37887	2.77519	2.98518	4.58477	3.61503	
	0.03268	3.83303	4.55537	0.61958	0.77255	0.48576	0.95510														
308	3.27202	4.66041	4.03210	3.72890	2.62865	3.68698	3.85349	3.29453	3.45219	2.69596	3.93010	3.88606	4.20159	3.84191	3.58729	3.47606	3.57058	3.18378	0.96876	2.63291	308 w - - -
	2.68618	4.42225	2.77519	2.73123	3.46354	2.40513	3.72494	3.29354	2.67741	2.69355	4.24690	2.90347	2.73739	3.18146	2.89801	2.37887	2.77519	2.98518	4.58477	3.61503	
	0.03268	3.83303	4.55537	0.61958	0.77255	0.48576	0.95510														
309	2.77713	4.33135	4.07016	3.73272	3.52199	3.68239	4.54148	2.05456	3.59987	2.21635	3.49380	3.92869	4.24556	3.96282	3.81605	3.18396	3.13994	1.07623	5.29954	4.02841	309 v - - -

	2.68618	4.42225	2.77519	2.73123	3.46354	2.40513	3.72494	3.29354	2.67741	2.69355	4.24690	2.90347	2.73739	3.18146	2.89801	2.37887	2.77519	2.98518	4.58477	3.61503	
	0.03268	3.83303	4.55537	0.61958	0.77255	0.48576	0.95510														
310	3.19134	4.69630	3.78054	3.50310	2.29611	3.78460	3.60201	3.20618	3.36176	2.67014	3.88549	3.67575	4.25966	3.67727	3.55331	3.33175	3.47801	3.07495	3.93816	1.03399	310 y - - -
	2.68618	4.42225	2.77519	2.73123	3.46354	2.40513	3.72494	3.29354	2.67741	2.69355	4.24690	2.90347	2.73739	3.18146	2.89801	2.37887	2.77519	2.98518	4.58477	3.61503	
	0.03268	3.83303	4.55537	0.61958	0.77255	0.48576	0.95510														
311	2.91965	5.02079	2.47306	1.08285	4.36227	3.24954	3.83381	3.80837	2.70946	3.43216	4.40865	2.91254	3.85246	3.05076	3.11366	2.90651	3.22382	3.49656	5.54134	4.26436	311 e - - -
	2.68618	4.42225	2.77519	2.73123	3.46354	2.40513	3.72494	3.29354	2.67741	2.69355	4.24690	2.90347	2.73739	3.18146	2.89801	2.37887	2.77519	2.98518	4.58477	3.61503	
	0.03268	3.83303	4.55537	0.61958	0.77255	0.48576	0.95510														
312	2.94831	5.06869	0.97632	2.31258	4.44069	3.18175	3.89799	4.02012	2.97493	3.63580	4.61368	2.86669	3.83184	3.13755	3.48525	2.91560	3.28628	3.66374	5.61691	4.32095	312 d - - -
	2.68618	4.42225	2.77519	2.73123	3.46354	2.40513	3.72494	3.29354	2.67741	2.69355	4.24690	2.90347	2.73739	3.18146	2.89801	2.37887	2.77519	2.98518	4.58477	3.61503	
	0.03268	3.83303	4.55537	0.61958	0.77255	0.48576	0.95510														
313	3.27202	4.66041	4.03210	3.72890	2.62865	3.68698	3.85349	3.29453	3.45219	2.69596	3.93010	3.88606	4.20159	3.84191	3.58729	3.47606	3.57058	3.18378	0.96876	2.63291	313 w - - -
	2.68618	4.42225	2.77519	2.73123	3.46354	2.40513	3.72494	3.29354	2.67741	2.69355	4.24690	2.90347	2.73739	3.18146	2.89801	2.37887	2.77519	2.98518	4.58477	3.61503	
	0.03268	3.83303	4.55537	0.61958	0.77255	0.48576	0.95510														
314	1.93948	4.16463	3.14032	2.96492	4.23010	1.42234	4.13207	3.58946	3.11373	3.33790	4.22032	3.18743	3.64246	3.41632	3.41889	2.40132	2.71746	3.09294	5.55959	4.34719	314 g - - -
	2.68618	4.42225	2.77519	2.73123	3.46354	2.40513	3.72494	3.29354	2.67741	2.69355	4.24690	2.90347	2.73739	3.18146	2.89801	2.37887	2.77519	2.98518	4.58477	3.61503	
	0.03268	3.83303	4.55537	0.61958	0.77255	0.48576	0.95510														
315	2.61765	4.39623	3.28327	3.17805	4.31966	0.79266	4.30013	3.91025	3.35388	3.56921	4.54515	3.44774	3.78610	3.68127	3.59365	2.78855	3.09584	3.44624	5.42460	4.42045	315 g - - -
	2.68618	4.42225	2.77519	2.73123	3.46354	2.40513	3.72494	3.29354	2.67741	2.69355	4.24690	2.90347	2.73739	3.18146	2.89801	2.37887	2.77519	2.98518	4.58477	3.61503	
	0.03268	3.83303	4.55537	0.61958	0.77255	0.48576	0.95510														
316	3.00047	4.43103	4.27833	3.89317	3.34832	4.02286	4.62907	1.12816	3.72049	1.94320	3.29060	4.14408	4.45846	4.07325	3.92162	3.51489	3.30059	1.84674	5.23051	3.96495	316 i - - -
	2.68618	4.42225	2.77519	2.73123	3.46354	2.40513	3.72494	3.29354	2.67741	2.69355	4.24690	2.90347	2.73739	3.18146	2.89801	2.37887	2.77519	2.98518	4.58477	3.61503	
	0.03268	3.83303	4.55537	0.61958	0.77255	0.48576	0.95510														
317	3.27202	4.66041	4.03210	3.72890	2.62865	3.68698	3.85349	3.29453	3.45219	2.69596	3.93010	3.88606	4.20159	3.84191	3.58729	3.47606	3.57058	3.18378	0.96876	2.63291	317 w - - -
	2.68618	4.42225	2.77519	2.73123	3.46354	2.40513	3.72494	3.29354	2.67741	2.69355	4.24690	2.90347	2.73739	3.18146	2.89801	2.37887	2.77519	2.98518	4.58477	3.61503	
	0.03268	3.83303	4.55537	0.61958	0.77255	0.48576	0.95510														
318	3.05842	4.44288	4.46438	3.97453	3.24535	4.25288	4.66494	1.28899	3.78539	1.63545	3.13223	4.24752	4.57064	4.09300	3.99079	3.63824	3.31861	1.80341	5.20134	3.95958	318 i - - -
	2.68618	4.42225	2.77519	2.73123	3.46354	2.40513	3.72494	3.29354	2.67741	2.69355	4.24690	2.90347	2.73739	3.18146	2.89801	2.37887	2.77519	2.98518	4.58477	3.61503	
	0.03268	3.83303	4.55537	0.61958	0.77255	0.48576	0.95510														
319	2.61765	4.39623	3.28327	3.17805	4.31966	0.79266	4.30013	3.91025	3.35388	3.56921	4.54515	3.44774	3.78610	3.68127	3.59365	2.78855	3.09584	3.44624	5.42460	4.42045	319 g - - -
	2.68618	4.42225	2.77519	2.73123	3.46354	2.40513	3.72494	3.29354	2.67741	2.69355	4.24690	2.90347	2.73739	3.18146	2.89801	2.37887	2.77519	2.98518	4.58477	3.61503	
	0.03268	3.83303	4.55537	0.61958	0.77255	0.48576	0.95510														
320	2.96214	4.80276	3.44889	2.96907	4.14736	3.45685	3.79770	3.68689	2.22438	3.21005	4.21188	3.33151	3.96192	3.02053	1.04043	3.04858	3.23116	3.41270	5.24678	4.08393	320 r - - -
	2.68618	4.42225	2.77519	2.73123	3.46354	2.40513	3.72494	3.29354	2.67741	2.69355	4.24690	2.90347	2.73739	3.18146	2.89801	2.37887	2.77519	2.98518	4.58477	3.61503	
	0.03268	3.83303	4.55537	0.61958	0.77255	0.48576	0.95510														
321	3.11165	4.58599	4.12585	3.76620	3.12182	3.93147	4.43434	2.32453	3.53431	0.92536	3.15834	4.04543	4.37407	3.91210	3.71656	3.49871	3.40796	2.35149	4.98612	3.70011	321 l - - -
	2.68618	4.42225	2.77519	2.73123	3.46354	2.40513	3.72494	3.29354	2.67741	2.69355	4.24690	2.90347	2.73739	3.18146	2.89801	2.37887	2.77519	2.98518	4.58477	3.61503	
	0.03268	3.83303	4.55537	0.61958	0.77255	0.48576	0.95510														
322	2.25704	4.48151	2.78750	2.55041	4.14958	1.86259	3.82152	3.56794	2.66598	3.21083	4.07394	2.97389	3.72216	2.61482	3.03969	2.54577	2.82382	3.16596	5.44599	4.14831	322 g - - -
	2.68618	4.42225	2.77519	2.73123	3.46354	2.40513	3.72494	3.29354	2.67741	2.69355	4.24690	2.90347	2.73739	3.18146	2.89801	2.37887	2.77519	2.98518	4.58477	3.61503	
	0.03268	3.83303	4.55537	0.61958	0.77255	0.48576	0.95510														
323	2.88235	5.02060	2.97356	2.55662	4.36475	3.46181	3.59540	3.77900	1.38955	3.28143	4.16890	3.01438	3.90585	2.44411	2.27202	2.88239	3.09541	3.46410	5.39135	4.14329	323 k - - -
	2.68618	4.42225	2.77519	2.73123	3.46354	2.40513	3.72494	3.29354	2.67741	2.69355	4.24690	2.90347	2.73739	3.18146	2.89801	2.37887	2.77519	2.98518	4.58477	3.61503	
	0.03268	3.83303	4.55537	0.61958	0.77255	0.48576	0.95510														
324	3.28721	4.68247	4.16936	3.78810	1.78716	4.07978	3.44718	3.07007	3.66031	2.47715	3.70821	3.79946	4.43049	3.79350	3.81617	3.44017	3.52462	2.97669	3.66742	1.19862	324 y - - -
	2.68618	4.42225	2.77519	2.73123	3.46354	2.40513	3.72494	3.29354	2.67741	2.69355	4.24690	2.90347	2.73739	3.18146	2.89801	2.37887	2.77519	2.98518	4.58477	3.61503	
	0.03268	3.83303	4.55537	0.61958	0.77255	0.48576	0.95510														
325	2.61765	4.39623	3.28327	3.17805	4.31966	0.79266	4.30013	3.91025	3.35388	3.56921	4.54515	3.44774	3.78610	3.68127	3.59365	2.78855	3.09584	3.44624	5.42460	4.42045	325 g - - -
	2.68618	4.42225	2.77519	2.73123	3.46354	2.40513	3.72494	3.29354	2.67741	2.69355	4.24690	2.90347	2.73739	3.18146	2.89801	2.37887	2.77519	2.98518	4.58477	3.61503	
	0.03268	3.83303	4.55537	0.61958	0.77255	0.48576	0.95510														
326	2.77713	4.33135	4.07016	3.73272	3.52199	3.68239	4.54148	2.05456	3.59987	2.21635	3.49380	3.92869	4.24556	3.96282	3.81605	3.18396	3.13994	1.07623	5.29954	4.02841	326 v - - -
	2.68618	4.42225	2.77519	2.73123	3.46354	2.40513	3.72494	3.29354	2.67741	2.69355	4.24690	2.90347	2.73739	3.18146	2.89801	2.37887	2.77519	2.98518	4.58477	3.61503	
	0.03268	3.83303	4.55537	0.61958	0.77255	0.48576	0.95510														
327	2.78928	5.14486	2.39398	1.59159	4.45543	3.26281	3.63356	3.90172	2.18455	3.43890	4.28079	2.53947	3.79776	2.79038	2.80997	2.73801	3.04473	3.53364	5.59952	4.21909	327 e - - -
	2.68618	4.42225	2.77519	2.73123	3.46354	2.40513	3.72494	3.29354	2.67741	2.69355	4.24690	2.90347	2.73739	3.18146	2.89801	2.37887	2.77519	2.98518	4.58477	3.61503	
	0.03268	3.83303	4.55537	0.61958	0.77255	0.48576	0.95510														
328	2.20851	4.14891	3.22021	2.96018	4.11307	2.91715	4.06271	3.46020	2.99058	3.21396	4.09390	3.17973	3.64367	3.33363	3.29812	1.39338	2.31025	2.99928	5.48211	4.22845	328 s - - -
	2.68618	4.42225	2.77519	2.73123	3.46354	2.40513	3.72494	3.29354	2.67741	2.69355	4.24690	2.90347	2.73739	3.18146	2.89801	2.37887	2.77519	2.98518	4.58477	3.61503	
	0.03268	3.83303	4.55537	0.61958	0.77255	0.48576	0.95510														
329	2.70125	4.46443	3.30428	3.13815	4.13555	3.15421	4.21835	3.67141	3.19219	3.30231	4.35093	3.44851	0.90433	3.58220	3.44165	2.86766	3.13986	3.32122	5.33234	4.25589	329 p - - -
	2.68618	4.42225	2.77519	2.73123	3.46354	2.40513	3.72494	3.29354	2.67741	2.69355	4.24690	2.90347	2.73739	3.18146	2.89801	2.37887	2.77519	2.98518	4.58477	3.61503	
	0.03268	3.83303	4.55537	0.61958	0.77255	0.48576	0.95510														
330	2.25451	4.72872	3.03122	2.51960	4.09111	3.37378	3.61089	3.47509	2.08306	3.06983	3.91168	2.98765	3.37441	2.77356	2.12973	2.67517	2.87066	3.15196	5.28329	4.00719	330 k - - -
	2.68618	4.42225	2.77519	2.73123	3.46354	2.40513	3.72494	3.29354	2.67741	2.69355	4.24690	2.90347	2.73739	3.18146	2.89801	2.37887	2.77519	2.98518	4.58477	3.61503	
	0.03268	3.83303	4.55537	0.61958	0.77255	0.48576	0.95510														
331	2.17042	3.08180	3.64723	3.22769	3.84664	2.95686	4.11235	3.11660	3.13239	2.93600	3.83756	3.33097	3.66426	3.44417	3.40719	1.48237	2.62617	2.60727	5.28395	4.05561	331 s - - -
	2.68618	4.42225	2.77519	2.73123	3.46354	2.40513	3.72494	3.29354	2.67741	2.69355	4.24690	2.90347	2.73739	3.18146	2.89801	2.37887	2.77519	2.98518	4.58477	3.61503	

	0.03268	3.83303	4.55537	0.61958	0.77255	0.48576	0.95510														
332	3.11165	4.58599	4.12585	3.76620	3.12182	3.93147	4.43434	2.32453	3.53431	0.92536	3.15834	4.04543	4.37407	3.91210	3.71656	3.49871	3.40796	2.35149	4.98612	3.70011	332 l - - -
	2.68618	4.42225	2.77519	2.73123	3.46354	2.40513	3.72494	3.29354	2.67741	2.69355	4.24690	2.90347	2.73739	3.18146	2.89801	2.37887	2.77519	2.98518	4.58477	3.61503	
	0.03268	3.83303	4.55537	0.61958	0.77255	0.48576	0.95510														
333	2.48975	4.84138	3.17578	2.63095	4.23654	3.45973	3.59791	3.58944	1.89306	3.15312	4.02078	3.06362	3.88356	2.75511	1.70067	2.80948	2.99255	3.27787	5.31721	4.08800	333 r - - -
	2.68618	4.42225	2.77519	2.73123	3.46354	2.40513	3.72494	3.29354	2.67741	2.69355	4.24690	2.90347	2.73739	3.18146	2.89801	2.37887	2.77519	2.98518	4.58477	3.61503	
	0.03268	3.83303	4.55537	0.61958	0.77255	0.48576	0.95510														
334	2.94831	5.06869	0.97632	2.31258	4.44069	3.18175	3.89799	4.02012	2.97493	3.63580	4.61368	2.86669	3.83184	3.13755	3.48525	2.91560	3.28628	3.66374	5.61691	4.32095	334 d - - -
	2.68618	4.42225	2.77519	2.73123	3.46354	2.40513	3.72494	3.29354	2.67741	2.69355	4.24690	2.90347	2.73739	3.18146	2.89801	2.37887	2.77519	2.98518	4.58477	3.61503	
	0.03268	3.83303	4.55537	0.61958	0.77255	0.48576	0.95510														
335	1.07216	4.17353	3.42348	3.21371	4.01396	2.99897	4.24029	3.13365	3.22896	3.01700	4.05375	3.37300	3.73453	3.57391	3.48180	2.52446	2.79912	2.79493	5.44509	4.24110	335 a - - -
	2.68618	4.42225	2.77519	2.73123	3.46354	2.40513	3.72494	3.29354	2.67741	2.69355	4.24690	2.90347	2.73739	3.18146	2.89801	2.37887	2.77519	2.98518	4.58477	3.61503	
	0.03268	3.83303	4.55537	0.61958	0.77255	0.48576	0.95510														
336	2.93703	4.89791	3.11032	2.74002	4.27531	3.43004	3.73906	3.70226	1.09362	3.27630	4.24050	3.16601	3.93369	2.93324	2.42654	2.98198	3.19357	3.41249	5.34669	4.15401	336 k - - -
	2.68618	4.42225	2.77519	2.73123	3.46354	2.40513	3.72494	3.29354	2.67741	2.69355	4.24690	2.90347	2.73739	3.18146	2.89801	2.37887	2.77519	2.98518	4.58477	3.61503	
	0.03268	3.83303	4.55537	0.61958	0.77255	0.48576	0.95510														
337	2.81139	4.97827	3.14362	2.56684	4.34678	3.49968	3.55019	3.71944	1.68357	3.22755	4.07151	3.02269	3.88560	2.43944	1.94671	2.81787	2.79564	3.39513	5.35174	4.11609	337 k - - -
	2.68618	4.42225	2.77519	2.73123	3.46354	2.40513	3.72494	3.29354	2.67741	2.69355	4.24690	2.90347	2.73739	3.18146	2.89801	2.37887	2.77519	2.98518	4.58477	3.61503	
	0.03268	3.83303	4.55537	0.61958	0.77255	0.48576	0.95510														
338	2.94831	5.06869	0.97632	2.31258	4.44069	3.18175	3.89799	4.02012	2.97493	3.63580	4.61368	2.86669	3.83184	3.13755	3.48525	2.91560	3.28628	3.66374	5.61691	4.32095	338 d - - -
	2.68618	4.42225	2.77519	2.73123	3.46354	2.40513	3.72494	3.29354	2.67741	2.69355	4.24690	2.90347	2.73739	3.18146	2.89801	2.37887	2.77519	2.98518	4.58477	3.61503	
	0.03268	3.83303	4.55537	0.61958	0.77255	0.48576	0.95510														
339	1.07216	4.17353	3.42348	3.21371	4.01396	2.99897	4.24029	3.13365	3.22896	3.01700	4.05375	3.37300	3.73453	3.57391	3.48180	2.52446	2.79912	2.79493	5.44509	4.24110	339 a - - -
	2.68618	4.42225	2.77519	2.73123	3.46354	2.40513	3.72494	3.29354	2.67741	2.69355	4.24690	2.90347	2.73739	3.18146	2.89801	2.37887	2.77519	2.98518	4.58477	3.61503	
	0.03268	3.83303	4.55537	0.61958	0.77255	0.48576	0.95510														
340	2.87265	4.46656	3.76603	3.33692	2.53445	3.73639	3.70253	2.74281	3.18384	2.36535	3.52134	3.57518	4.16777	3.50368	3.42854	3.09791	3.14554	2.30811	4.18454	1.57362	340 y - - -
	2.68618	4.42225	2.77519	2.73123	3.46354	2.40513	3.72494	3.29354	2.67741	2.69355	4.24690	2.90347	2.73739	3.18146	2.89801	2.37887	2.77519	2.98518	4.58477	3.61503	
	0.03268	3.83303	4.55537	0.61958	0.77255	0.48576	0.95510														
341	3.27202	4.66041	4.03210	3.72890	2.62865	3.68698	3.85349	3.29453	3.45219	2.69596	3.93010	3.88606	4.20159	3.84191	3.58729	3.47606	3.57058	3.18378	0.96876	2.63291	341 w - - -
	2.68618	4.42225	2.77519	2.73123	3.46354	2.40513	3.72494	3.29354	2.67741	2.69355	4.24690	2.90347	2.73739	3.18146	2.89801	2.37887	2.77519	2.98518	4.58477	3.61503	
	0.03268	3.83303	4.55537	0.61958	0.77255	0.48576	0.95510														
342	1.28840	4.08974	3.32133	3.07452	4.09757	2.89173	4.13766	3.36654	3.11400	3.18238	4.07592	3.23481	3.63658	3.43215	3.40172	2.07179	2.65674	2.91807	5.49184	4.25860	342 a - - -
	2.68618	4.42225	2.77519	2.73123	3.46354	2.40513	3.72494	3.29354	2.67741	2.69355	4.24690	2.90347	2.73739	3.18146	2.89801	2.37887	2.77519	2.98518	4.58477	3.61503	
	0.03268	3.83303	4.55537	0.61958	0.77255	0.48576	0.95510														
343	2.95371	4.77708	3.08911	2.84570	3.25855	3.41543	1.22850	3.65045	2.68990	3.14913	4.18951	3.25638	3.96612	3.21160	2.95766	3.02494	3.25600	3.38639	4.72403	3.21065	343 h - - -
	2.68618	4.42225	2.77519	2.73123	3.46354	2.40513	3.72494	3.29354	2.67741	2.69355	4.24690	2.90347	2.73739	3.18146	2.89801	2.37887	2.77519	2.98518	4.58477	3.61503	
	0.03268	3.83303	4.55537	0.61958	0.77255	0.48576	0.95510														
344	2.95371	4.77708	3.08911	2.84570	3.25855	3.41543	1.22850	3.65045	2.68990	3.14913	4.18951	3.25638	3.96612	3.21160	2.95766	3.02494	3.25600	3.38639	4.72403	3.21065	344 h - - -
	2.68618	4.42225	2.77519	2.73123	3.46354	2.40513	3.72494	3.29354	2.67741	2.69355	4.24690	2.90347	2.73739	3.18146	2.89801	2.37887	2.77519	2.98518	4.58477	3.61503	
	0.03268	3.83303	4.55537	0.61958	0.77255	0.48576	0.95510														
345	2.94831	5.06869	0.97632	2.31258	4.44069	3.18175	3.89799	4.02012	2.97493	3.63580	4.61368	2.86669	3.83184	3.13755	3.48525	2.91560	3.28628	3.66374	5.61691	4.32095	345 d - - -
	2.68618	4.42225	2.77519	2.73123	3.46354	2.40513	3.72494	3.29354	2.67741	2.69355	4.24690	2.90347	2.73739	3.18146	2.89801	2.37887	2.77519	2.98518	4.58477	3.61503	
	0.03268	3.83303	4.55537	0.61958	0.77255	0.48576	0.95510														
346	2.43290	4.38874	3.84116	3.43130	3.31013	3.68524	4.26687	2.29587	3.25864	1.31093	3.25622	3.71998	4.17591	3.63740	3.50369	3.09755	3.08866	2.21783	5.09472	3.84229	346 l - - -
	2.68618	4.42225	2.77519	2.73123	3.46354	2.40513	3.72494	3.29354	2.67741	2.69355	4.24690	2.90347	2.73739	3.18146	2.89801	2.37887	2.77519	2.98518	4.58477	3.61503	
	0.03268	3.83303	4.55537	0.61958	0.77255	0.48576	0.95510														
347	2.17393	4.20872	3.68207	3.12606	2.65778	3.62440	3.85200	2.53301	3.04432	2.18864	2.89261	3.45614	4.00145	3.32263	3.33323	2.90051	2.84692	2.37427	4.64540	2.94679	347 a - - -
	2.68618	4.42225	2.77519	2.73123	3.46354	2.40513	3.72494	3.29354	2.67741	2.69355	4.24690	2.90347	2.73739	3.18146	2.89801	2.37887	2.77519	2.98518	4.58477	3.61503	
	0.03268	3.83303	4.55537	0.61958	0.77255	0.48576	0.95510														
348	3.11165	4.58599	4.12585	3.76620	3.12182	3.93147	4.43434	2.32453	3.53431	0.92536	3.15834	4.04543	4.37407	3.91210	3.71656	3.49871	3.40796	2.35149	4.98612	3.70011	348 l - - -
	2.68618	4.42225	2.77519	2.73123	3.46354	2.40513	3.72494	3.29354	2.67741	2.69355	4.24690	2.90347	2.73739	3.18146	2.89801	2.37887	2.77519	2.98518	4.58477	3.61503	
	0.03268	3.83303	4.55537	0.61958	0.77255	0.48576	0.95510														
349	2.14192	4.31771	3.86353	3.35164	3.36408	3.73740	4.20081	2.05279	3.22309	1.69244	3.26521	3.67508	4.16454	3.55693	3.50608	3.07333	3.00491	2.06499	5.08254	3.85833	349 l - - -
	2.68618	4.42225	2.77519	2.73123	3.46354	2.40513	3.72494	3.29354	2.67741	2.69355	4.24690	2.90347	2.73739	3.18146	2.89801	2.37887	2.77519	2.98518	4.58477	3.61503	
	0.03268	3.83303	4.55537	0.61958	0.77255	0.48576	0.95510														
350	1.07216	4.17353	3.42348	3.21371	4.01396	2.99897	4.24029	3.13365	3.22896	3.01700	4.05375	3.37300	3.73453	3.57391	3.48180	2.52446	2.79912	2.79493	5.44509	4.24110	350 a - - -
	2.68618	4.42225	2.77519	2.73123	3.46354	2.40513	3.72494	3.29354	2.67741	2.69355	4.24690	2.90347	2.73739	3.18146	2.89801	2.37887	2.77519	2.98518	4.58477	3.61503	
	0.03268	3.83303	4.55537	0.61958	0.77255	0.48576	0.95510														
351	3.23553	4.68340	4.03680	3.66877	1.96907	4.00043	3.47616	3.07352	3.53588	2.50553	3.73189	3.74249	4.38096	3.72911	3.71774	3.38275	3.48534	2.97023	3.73034	1.15812	351 y - - -
	2.68618	4.42225	2.77519	2.73123	3.46354	2.40513	3.72494	3.29354	2.67741	2.69355	4.24690	2.90347	2.73739	3.18146	2.89801	2.37887	2.77519	2.98518	4.58477	3.61503	
	0.03268	3.83303	4.55537	0.61958	0.77255	0.48576	0.95510														
352	1.07216	4.17353	3.42348	3.21371	4.01396	2.99897	4.24029	3.13365	3.22896	3.01700	4.05375	3.37300	3.73453	3.57391	3.48180	2.52446	2.79912	2.79493	5.44509	4.24110	352 a - - -
	2.68618	4.42225	2.77519	2.73123	3.46354	2.40513	3.72494	3.29354	2.67741	2.69355	4.24690	2.90347	2.73739	3.18146	2.89801	2.37887	2.77519	2.98518	4.58477	3.61503	
	0.03268	3.83303	4.55537	0.61958	0.77255	0.48576	0.95510														
353	3.11345	4.55080	4.38116	3.84858	3.03687	4.22749	4.52201	2.15178	3.63854	1.08899	2.38639	4.15907	4.50234	3.91407	3.83811	3.57999	3.35277	2.22530	5.03372	3.86146	353 l - - -
	2.68618	4.42225	2.77519	2.73123	3.46354	2.40513	3.72494	3.29354	2.67741	2.69355	4.24690	2.90347	2.73739	3.18146	2.89801	2.37887	2.77519	2.98518	4.58477	3.61503	
	0.03268	3.83303	4.55537	0.61958	0.77255	0.48576	0.95510														

354	3.27202	4.66041	4.03210	3.72890	2.62865	3.68698	3.85349	3.29453	3.45219	2.69596	3.93010	3.88606	4.20159	3.84191	3.58729	3.47606	3.57058	3.18378	0.96876	2.63291	354 w - - -
	2.68618	4.42225	2.77519	2.73123	3.46354	2.40513	3.72494	3.29354	2.67741	2.69355	4.24690	2.90347	2.73739	3.18146	2.89801	2.37887	2.77519	2.98518	4.58477	3.61503	
	0.03268	3.83303	4.55537	0.61958	0.77255	0.48576	0.95510														
355	2.70125	4.46443	3.30428	3.13815	4.13555	3.15421	4.21835	3.67141	3.19219	3.30231	4.35093	3.44851	0.90433	3.58220	3.44165	2.86766	3.13986	3.32122	5.33234	4.25589	355 p - - -
	2.68618	4.42225	2.77519	2.73123	3.46354	2.40513	3.72494	3.29354	2.67741	2.69355	4.24690	2.90347	2.73739	3.18146	2.89801	2.37887	2.77519	2.98518	4.58477	3.61503	
	0.03268	3.83303	4.55537	0.61958	0.77255	0.48576	0.95510														
356	2.65480	4.33513	3.68798	3.24104	3.41586	3.55568	4.12379	2.39932	3.07121	1.62563	3.36556	3.55080	4.05804	3.46355	3.34428	2.93861	2.20887	2.26966	5.09106	3.83875	356 l - - -
	2.68618	4.42225	2.77519	2.73123	3.46354	2.40513	3.72494	3.29354	2.67741	2.69355	4.24690	2.90347	2.73739	3.18146	2.89801	2.37887	2.77519	2.98518	4.58477	3.61503	
	0.03268	3.83303	4.55537	0.61958	0.77255	0.48576	0.95510														
357	2.28328	4.27527	3.18195	2.87341	3.86089	1.73562	3.95979	3.15559	2.86610	2.51535	3.88225	3.18628	3.74812	3.23813	3.18726	2.54166	2.77716	2.83074	5.28237	3.99851	357 g - - -
	2.68618	4.42225	2.77519	2.73123	3.46354	2.40513	3.72494	3.29354	2.67741	2.69355	4.24690	2.90347	2.73739	3.18146	2.89801	2.37887	2.77519	2.98518	4.58477	3.61503	
	0.03268	3.83303	4.55537	0.61958	0.77255	0.48576	0.95510														
358	2.69380	4.34082	3.64321	3.28805	1.51099	2.77082	3.84967	2.85647	3.25731	2.48690	3.58344	3.52340	4.01248	3.53320	3.50568	2.92699	3.02386	2.67377	4.44058	2.90809	358 f - - -
	2.68618	4.42225	2.77519	2.73123	3.46354	2.40513	3.72494	3.29354	2.67741	2.69355	4.24690	2.90347	2.73739	3.18146	2.89801	2.37887	2.77519	2.98518	4.58477	3.61503	
	0.03268	3.83303	4.55537	0.61958	0.77255	0.48576	0.95510														
359	2.16602	4.18429	3.71446	3.18864	2.47097	3.55749	3.95321	2.25221	3.10965	2.25848	3.29478	3.49088	3.99463	3.39495	3.39305	2.75791	2.84053	2.25703	4.79274	3.50133	359 a - - -
	2.68618	4.42225	2.77519	2.73123	3.46354	2.40513	3.72494	3.29354	2.67741	2.69355	4.24690	2.90347	2.73739	3.18146	2.89801	2.37887	2.77519	2.98518	4.58477	3.61503	
	0.03268	3.83303	4.55537	0.61958	0.77255	0.48576	0.95510														
360	2.96214	4.80276	3.44889	2.96907	4.14736	3.45685	3.79770	3.68689	2.22438	3.21005	4.21188	3.33151	3.96192	3.02053	1.04043	3.04858	3.23116	3.41270	5.24678	4.08393	360 r - - -
	2.68618	4.42225	2.77519	2.73123	3.46354	2.40513	3.72494	3.29354	2.67741	2.69355	4.24690	2.90347	2.73739	3.18146	2.89801	2.37887	2.77519	2.98518	4.58477	3.61503	
	0.03268	3.83303	4.55537	0.61958	0.77255	0.48576	0.95510														
361	3.00006	4.46650	4.29746	3.81230	3.16547	4.08204	4.52332	2.05483	3.62287	1.20124	3.06130	4.09095	4.44689	3.93010	3.83674	3.46561	3.27505	1.80060	5.10709	3.87892	361 l - - -
	2.68618	4.42225	2.77519	2.73123	3.46354	2.40513	3.72494	3.29354	2.67741	2.69355	4.24690	2.90347	2.73739	3.18146	2.89801	2.37887	2.77519	2.98518	4.58477	3.61503	
	0.03268	3.83303	4.55537	0.61958	0.77255	0.48576	0.95510														
362	1.80759	4.39298	2.96281	2.63437	4.02842	3.11015	3.81803	3.41831	2.66506	3.08613	3.94492	3.03027	3.71679	2.64892	3.03416	2.08608	2.76443	3.03810	5.35138	4.07005	362 a - - -
	2.68618	4.42225	2.77519	2.73123	3.46354	2.40513	3.72494	3.29354	2.67741	2.69355	4.24690	2.90347	2.73739	3.18146	2.89801	2.37887	2.77519	2.98518	4.58477	3.61503	
	0.03268	3.83303	4.55537	0.61958	0.77255	0.48576	0.95510														
363	3.11165	4.58599	4.12585	3.76620	3.12182	3.93147	4.43434	2.32453	3.53431	0.92536	3.15834	4.04543	4.37407	3.91210	3.71656	3.49871	3.40796	2.35149	4.98612	3.70011	363 l - - -
	2.68618	4.42225	2.77519	2.73123	3.46354	2.40513	3.72494	3.29354	2.67741	2.69355	4.24690	2.90347	2.73739	3.18146	2.89801	2.37887	2.77519	2.98518	4.58477	3.61503	
	0.03268	3.83303	4.55537	0.61958	0.77255	0.48576	0.95510														
364	2.70125	4.46443	3.30428	3.13815	4.13555	3.15421	4.21835	3.67141	3.19219	3.30231	4.35093	3.44851	0.90433	3.58220	3.44165	2.86766	3.13986	3.32122	5.33234	4.25589	364 p - - -
	2.68618	4.42225	2.77519	2.73123	3.46354	2.40513	3.72494	3.29354	2.67741	2.69355	4.24690	2.90347	2.73739	3.18146	2.89801	2.37887	2.77519	2.98518	4.58477	3.61503	
	0.03268	3.83303	4.55537	0.61958	0.77255	0.48576	0.95510														
365	2.70762	4.95365	1.42076	2.21732	4.38792	3.14514	3.76345	3.76540	2.74445	3.44264	4.34351	2.75966	3.76811	2.95373	3.25987	2.69599	2.67012	3.39914	5.66817	4.25531	365 d - - -
	2.68618	4.42225	2.77519	2.73123	3.46354	2.40513	3.72494	3.29354	2.67741	2.69355	4.24690	2.90347	2.73739	3.18146	2.89801	2.37887	2.77519	2.98518	4.58477	3.61503	
	0.03268	3.83303	4.55537	0.61958	0.77255	0.48576	0.95510														
366	2.79577	5.11012	2.44899	1.72521	4.39099	3.28196	3.63971	3.84872	2.37766	3.38213	4.24755	2.78957	3.81006	2.09224	2.77351	2.75408	3.04966	3.49894	5.55208	4.18440	366 e - - -
	2.68618	4.42225	2.77519	2.73123	3.46354	2.40513	3.72494	3.29354	2.67741	2.69355	4.24690	2.90347	2.73739	3.18146	2.89801	2.37887	2.77519	2.98518	4.58477	3.61503	
	0.03268	3.83303	4.55537	0.61958	0.77255	0.48576	0.95510														
367	2.76950	5.04431	2.60125	1.64309	4.34600	3.33075	3.60971	3.78158	2.26768	3.31533	4.16487	2.84183	3.81883	2.64692	2.38177	2.74509	3.00881	3.43699	5.47865	4.14370	367 e - - -
	2.68618	4.42225	2.77519	2.73123	3.46354	2.40513	3.72494	3.29354	2.67741	2.69355	4.24690	2.90347	2.73739	3.18146	2.89801	2.37887	2.77519	2.98518	4.58477	3.61503	
	0.03268	3.83303	4.55537	0.61958	0.77255	0.48576	0.95510														
368	2.86121	5.36258	1.57988	1.60203	4.64866	3.15977	3.69779	4.14405	2.69960	3.67784	4.53999	2.63722	3.77520	2.86856	3.27302	2.75932	3.14934	3.74024	5.83863	4.36189	368 d - - -
	2.68618	4.42225	2.77519	2.73123	3.46354	2.40513	3.72494	3.29354	2.67741	2.69355	4.24690	2.90347	2.73739	3.18146	2.89801	2.37887	2.77519	2.98518	4.58477	3.61503	
	0.03268	3.83303	4.55537	0.61958	0.77255	0.48576	0.95510														
369	2.44350	4.48299	3.21728	2.72067	3.61204	3.45729	3.75260	2.91499	2.55440	2.51657	2.58957	3.15796	3.88661	2.37954	2.89186	2.75706	2.86267	2.71891	5.06420	3.80328	369 q - - -
	2.68618	4.42225	2.77519	2.73123	3.46354	2.40513	3.72494	3.29354	2.67741	2.69355	4.24690	2.90347	2.73739	3.18146	2.89801	2.37887	2.77519	2.98518	4.58477	3.61503	
	0.03268	3.83303	4.55537	0.61958	0.77255	0.48576	0.95510														
370	2.24702	4.84383	2.49814	1.57429	4.26381	3.21197	3.73396	3.56488	2.58466	3.25930	4.15888	2.84087	3.78740	2.91826	3.01516	2.69416	2.98193	3.23441	5.53341	4.18084	370 e - - -
	2.68618	4.42225	2.77519	2.73123	3.46354	2.40513	3.72494	3.29354	2.67741	2.69355	4.24690	2.90347	2.73739	3.18146	2.89801	2.37887	2.77519	2.98518	4.58477	3.61503	
	0.03268	3.83303	4.55537	0.61958	0.77255	0.48576	0.95510														
371	3.27202	4.66041	4.03210	3.72890	2.62865	3.68698	3.85349	3.29453	3.45219	2.69596	3.93010	3.88606	4.20159	3.84191	3.58729	3.47606	3.57058	3.18378	0.96876	2.63291	371 w - - -
	2.68618	4.42225	2.77519	2.73123	3.46354	2.40513	3.72494	3.29354	2.67741	2.69355	4.24690	2.90347	2.73739	3.18146	2.89801	2.37887	2.77519	2.98518	4.58477	3.61503	
	0.03268	3.83303	4.55537	0.61958	0.77255	0.48576	0.95510														
372	3.26097	4.63851	4.24683	3.86459	1.23640	4.09979	3.53386	2.90086	3.73462	2.27022	3.54431	3.86785	4.44438	3.85264	3.87003	3.46806	3.50042	2.85067	3.75065	1.88345	372 f - - -
	2.68618	4.42225	2.77519	2.73123	3.46354	2.40513	3.72494	3.29354	2.67741	2.69355	4.24690	2.90347	2.73739	3.18146	2.89801	2.37887	2.77519	2.98518	4.58477	3.61503	
	0.03268	3.83303	4.55537	0.61958	0.77255	0.48576	0.95510														
373	2.91965	5.02079	2.47306	1.08285	4.36227	3.24954	3.83381	3.80837	2.70946	3.43216	4.40865	2.91254	3.85246	3.05076	3.11366	2.90651	3.22382	3.49656	5.54134	4.26436	373 e - - -
	2.68618	4.42225	2.77519	2.73123	3.46354	2.40513	3.72494	3.29354	2.67741	2.69355	4.24690	2.90347	2.73739	3.18146	2.89801	2.37887	2.77519	2.98518	4.58477	3.61503	
	0.03268	3.83303	4.55537	0.61958	0.77255	0.48576	0.95510														
374	1.07216	4.17353	3.42348	3.21371	4.01396	2.99897	4.24029	3.13365	3.22896	3.01700	4.05375	3.37300	3.73453	3.57391	3.48180	2.52446	2.79912	2.79493	5.44509	4.24110	374 a - - -
	2.68618	4.42225	2.77519	2.73123	3.46354	2.40513	3.72494	3.29354	2.67741	2.69355	4.24690	2.90347	2.73739	3.18146	2.89801	2.37887	2.77519	2.98518	4.58477	3.61503	
	0.03268	3.83303	4.55537	0.61958	0.77255	0.48576	0.95510														
375	2.69182	4.68600	2.68850	2.57033	4.04344	3.15836	3.89101	3.74595	2.79175	3.39751	4.35350	1.22987	3.80860	3.16424	3.14460	2.76087	3.07272	3.37215	5.35398	3.99503	375 n - - -
	2.68618	4.42225	2.77519	2.73123	3.46354	2.40513	3.72494	3.29354	2.67741	2.69355	4.24690	2.90347	2.73739	3.18146	2.89801	2.37887	2.77519	2.98518	4.58477	3.61503	
	0.03268	3.83303	4.55537	0.61958	0.77255	0.48576	0.95510														
376	3.19134	4.69630	3.78054	3.50310	2.29611	3.78460	3.60201	3.20618	3.36176	2.67014	3.88549	3.67575	4.25966	3.67727	3.55331	3.33175	3.47801	3.07495	3.93816	1.03399	376 y - - -

```
      2.68618  4.42225  2.77519  2.73123  3.46354  2.40513  3.72494  3.29354  2.67741  2.69355  4.24690  2.90347  2.73739  3.18146  2.89801  2.37887  2.77519  2.98518  4.58477  3.61503
      0.03268  3.83303  4.55537  0.61958  0.77255  0.48576  0.95510
377   2.70125  4.46443  3.30428  3.13815  4.13555  3.15421  4.21835  3.67141  3.19219  3.30231  4.35093  3.44851  0.90433  3.58220  3.44165  2.86766  3.13986  3.32122  5.33234  4.25589   377 p - - -
      2.68618  4.42225  2.77519  2.73123  3.46354  2.40513  3.72494  3.29354  2.67741  2.69355  4.24690  2.90347  2.73739  3.18146  2.89801  2.37887  2.77519  2.98518  4.58477  3.61503
      0.03268  3.83303  4.55537  0.61958  0.77255  0.48576  0.95510
378   2.61765  4.39623  3.28327  3.17805  4.31966  0.79266  4.30013  3.91025  3.35388  3.56921  4.54515  3.44774  3.78610  3.68127  3.59365  2.78855  3.09584  3.44624  5.42460  4.42045   378 g - - -
      2.68618  4.42225  2.77519  2.73123  3.46354  2.40513  3.72494  3.29354  2.67741  2.69355  4.24690  2.90347  2.73739  3.18146  2.89801  2.37887  2.77519  2.98518  4.58477  3.61503
      0.03268  3.83303  4.55537  0.61958  0.77255  0.48576  0.95510
379   3.27202  4.66041  4.03210  3.72890  2.62865  3.68698  3.85349  3.29453  3.45219  2.69596  3.93010  3.88606  4.20159  3.84191  3.58729  3.47606  3.57058  3.18378  0.96876  2.63291   379 w - - -
      2.68618  4.42225  2.77519  2.73123  3.46354  2.40513  3.72494  3.29354  2.67741  2.69355  4.24690  2.90347  2.73739  3.18146  2.89801  2.37887  2.77519  2.98518  4.58477  3.61503
      0.03268  3.83303  4.55537  0.61958  0.77255  0.48576  0.95510
380   2.11522  4.48846  2.75592  2.58657  3.53060  3.35666  3.70326  3.07416  2.66047  2.77292  3.67391  3.04620  3.82836  2.98284  3.06250  2.68565  2.83509  2.81119  4.97064  2.57861   380 a - - -
      2.68618  4.42225  2.77519  2.73123  3.46354  2.40513  3.72494  3.29354  2.67741  2.69355  4.24690  2.90347  2.73739  3.18146  2.89801  2.37887  2.77519  2.98518  4.58477  3.61503
      0.03268  3.83303  4.55537  0.61958  0.77255  0.48576  0.95510
381   2.49537  5.08792  1.54903  2.13297  4.48812  3.14538  3.70699  3.95931  2.68218  3.54101  4.39049  2.39043  3.75481  2.88128  3.22377  2.68401  3.03980  3.55432  5.72512  4.27923   381 d - - -
      2.68618  4.42225  2.77519  2.73123  3.46354  2.40513  3.72494  3.29354  2.67741  2.69355  4.24690  2.90347  2.73739  3.18146  2.89801  2.37887  2.77519  2.98518  4.58477  3.61503
      0.03268  3.83303  4.55537  0.61958  0.77255  0.48576  0.95510
382   2.72423  4.60671  3.22255  2.76472  3.22419  3.49503  1.97884  3.13627  2.51807  2.69789  3.18721  3.17983  3.93610  3.01485  2.82299  2.83640  2.97834  2.92375  4.73332  3.24750   382 h - - -
      2.68618  4.42225  2.77519  2.73123  3.46354  2.40513  3.72494  3.29354  2.67741  2.69355  4.24690  2.90347  2.73739  3.18146  2.89801  2.37887  2.77519  2.98518  4.58477  3.61503
      0.03268  3.83303  4.55537  0.61958  0.77255  0.48576  0.95510
383   3.19134  4.69630  3.78054  3.50310  2.29611  3.78460  3.60201  3.20618  3.36176  2.67014  3.88549  3.67575  4.25966  3.67727  3.55331  3.33175  3.47801  3.07495  3.93816  1.03399   383 y - - -
      2.68618  4.42225  2.77519  2.73123  3.46354  2.40513  3.72494  3.29354  2.67741  2.69355  4.24690  2.90347  2.73739  3.18146  2.89801  2.37887  2.77519  2.98518  4.58477  3.61503
      0.03268  3.83303  4.55537  0.61958  0.77255  0.48576  0.95510
384   2.61765  4.39623  3.28327  3.17805  4.31966  0.79266  4.30013  3.91025  3.35388  3.56921  4.54515  3.44774  3.78610  3.68127  3.59365  2.78855  3.09584  3.44624  5.42460  4.42045   384 g - - -
      2.68618  4.42225  2.77519  2.73123  3.46354  2.40513  3.72494  3.29354  2.67741  2.69355  4.24690  2.90347  2.73739  3.18146  2.89801  2.37887  2.77519  2.98518  4.58477  3.61503
      0.03268  3.83303  4.55537  0.61958  0.77255  0.48576  0.95510
385   2.93703  4.89791  3.11032  2.74002  4.27531  3.43004  3.73906  3.70226  1.09362  3.27630  4.24050  3.16601  3.93369  2.93324  2.42654  2.98198  3.19357  3.41249  5.34669  4.15401   385 k - - -
      2.68618  4.42225  2.77519  2.73123  3.46354  2.40513  3.72494  3.29354  2.67741  2.69355  4.24690  2.90347  2.73739  3.18146  2.89801  2.37887  2.77519  2.98518  4.58477  3.61503
      0.03268  3.83303  4.55537  0.61958  0.77255  0.48576  0.95510
386   2.97949  4.38276  4.45250  3.98020  3.37767  4.16698  4.72443  1.24023  3.82807  1.90116  3.23394  4.22901  4.53963  4.13383  4.04498  3.56175  3.26179  1.60514  5.31847  4.08962   386 i - - -
      2.68618  4.42225  2.77519  2.73123  3.46354  2.40513  3.72494  3.29354  2.67741  2.69355  4.24690  2.90347  2.73739  3.18146  2.89801  2.37887  2.77519  2.98518  4.58477  3.61503
      0.03268  3.83303  4.55537  0.61958  0.77255  0.48576  0.95510
387   3.18010  4.58262  4.20504  3.76132  1.78512  4.06897  3.51656  2.89037  3.62835  2.12506  3.53185  3.80653  4.39604  3.76548  3.79119  3.39387  3.40727  2.80916  3.76138  1.45177   387 y - - -
      2.68618  4.42225  2.77519  2.73123  3.46354  2.40513  3.72494  3.29354  2.67741  2.69355  4.24690  2.90347  2.73739  3.18146  2.89801  2.37887  2.77519  2.98518  4.58477  3.61503
      0.03268  3.83303  4.55537  0.61958  0.77255  0.48576  0.95510
388   2.71980  5.14244  2.22837  1.87334  4.43626  3.24234  3.61302  3.90473  2.45161  3.43135  4.24418  2.17283  3.76171  2.75825  2.71186  2.66515  2.97466  3.51673  5.60429  4.19082   388 e - - -
      2.68618  4.42225  2.77519  2.73123  3.46354  2.40513  3.72494  3.29354  2.67741  2.69355  4.24690  2.90347  2.73739  3.18146  2.89801  2.37887  2.77519  2.98518  4.58477  3.61503
      0.03268  3.83303  4.55537  0.61958  0.77255  0.48576  0.95510
389   2.91965  5.02079  2.47306  1.08285  4.36227  3.24954  3.83381  3.80837  2.70946  3.43216  4.40865  2.91254  3.85246  3.05076  3.11366  2.90651  3.22382  3.49656  5.54134  4.26436   389 e - - -
      2.68618  4.42225  2.77519  2.73123  3.46354  2.40513  3.72494  3.29354  2.67741  2.69355  4.24690  2.90347  2.73739  3.18146  2.89801  2.37887  2.77519  2.98518  4.58477  3.61503
      0.03268  3.83303  4.55537  0.61958  0.77255  0.48576  0.95510
390   3.27202  4.66041  4.03210  3.72890  2.62865  3.68698  3.85349  3.29453  3.45219  2.69596  3.93010  3.88606  4.20159  3.84191  3.58729  3.47606  3.57058  3.18378  0.96876  2.63291   390 w - - -
      2.68618  4.42225  2.77519  2.73123  3.46354  2.40513  3.72494  3.29354  2.67741  2.69355  4.24690  2.90347  2.73739  3.18146  2.89801  2.37887  2.77519  2.98518  4.58477  3.61503
      0.03268  3.83303  4.55537  0.61958  0.77255  0.48576  0.95510
391   2.93522  5.02259  3.31030  2.70388  4.43168  3.55945  3.56470  3.79667  1.48197  3.27945  4.15766  3.11521  3.94870  2.70946  1.67420  2.94553  3.11915  3.48702  5.35491  4.16723   391 k - - -
      2.68618  4.42225  2.77519  2.73123  3.46354  2.40513  3.72494  3.29354  2.67741  2.69355  4.24690  2.90347  2.73739  3.18146  2.89801  2.37887  2.77519  2.98518  4.58477  3.61503
      0.03268  3.83303  4.55537  0.61958  0.77255  0.48576  0.95510
392   2.09243  4.63509  2.97078  2.52791  4.06187  3.29826  3.66149  3.44364  2.00195  3.06630  3.91206  2.98592  3.78571  2.83872  2.59174  2.40875  2.84414  3.10907  5.29786  4.01648   392 k - - -
      2.68618  4.42225  2.77519  2.73123  3.46354  2.40513  3.72494  3.29354  2.67741  2.69355  4.24690  2.90347  2.73739  3.18146  2.89801  2.37887  2.77519  2.98518  4.58477  3.61503
      0.03268  3.83303  4.55537  0.61958  0.77255  0.48576  0.95510
393   2.78009  4.52604  3.54808  3.00938  3.43860  3.61299  3.84223  2.78179  2.53270  1.63204  3.46618  3.38121  4.02863  3.13146  2.26645  2.97517  3.02679  2.66027  4.97725  3.69263   393 l - - -
      2.68618  4.42225  2.77519  2.73123  3.46354  2.40513  3.72494  3.29354  2.67741  2.69355  4.24690  2.90347  2.73739  3.18146  2.89801  2.37887  2.77519  2.98518  4.58477  3.61503
      0.03268  3.83303  4.55537  0.61958  0.77255  0.48576  0.95510
394   2.61765  4.39623  3.28327  3.17805  4.31966  0.79266  4.30013  3.91025  3.35388  3.56921  4.54515  3.44774  3.78610  3.68127  3.59365  2.78855  3.09584  3.44624  5.42460  4.42045   394 g - - -
      2.68618  4.42225  2.77519  2.73123  3.46354  2.40513  3.72494  3.29354  2.67741  2.69355  4.24690  2.90347  2.73739  3.18146  2.89801  2.37887  2.77519  2.98518  4.58477  3.61503
      0.03268  3.83303  4.55537  0.61958  0.77255  0.48576  0.95510
395   2.69642  2.72638  3.96405  3.43042  2.39800  3.70756  3.76098  2.57463  3.31179  2.25968  3.29205  3.62102  4.09530  3.53659  3.52089  3.01502  2.94443  2.40790  4.25602  2.16375   395 y - - -
      2.68618  4.42225  2.77519  2.73123  3.46354  2.40513  3.72494  3.29354  2.67741  2.69355  4.24690  2.90347  2.73739  3.18146  2.89801  2.37887  2.77519  2.98518  4.58477  3.61503
      0.03268  3.83303  4.55537  0.61958  0.77255  0.48576  0.95510
396   2.91965  5.02079  2.47306  1.08285  4.36227  3.24954  3.83381  3.80837  2.70946  3.43216  4.40865  2.91254  3.85246  3.05076  3.11366  2.90651  3.22382  3.49656  5.54134  4.26436   396 e - - -
      2.68618  4.42225  2.77519  2.73123  3.46354  2.40513  3.72494  3.29354  2.67741  2.69355  4.24690  2.90347  2.73739  3.18146  2.89801  2.37887  2.77519  2.98518  4.58477  3.61503
      0.03268  3.83303  4.55537  0.61958  0.77255  0.48576  0.95510
397   2.94831  5.06869  0.97632  2.31258  4.44069  3.18175  3.89799  4.02012  2.97493  3.63580  4.61368  2.86669  3.83184  3.13755  3.48525  2.91560  3.28628  3.66374  5.61691  4.32095   397 d - - -
      2.68618  4.42225  2.77519  2.73123  3.46354  2.40513  3.72494  3.29354  2.67741  2.69355  4.24690  2.90347  2.73739  3.18146  2.89801  2.37887  2.77519  2.98518  4.58477  3.61503
      0.03268  3.83303  4.55537  0.61958  0.77255  0.48576  0.95510
398   2.70125  4.46443  3.30428  3.13815  4.13555  3.15421  4.21835  3.67141  3.19219  3.30231  4.35093  3.44851  0.90433  3.58220  3.44165  2.86766  3.13986  3.32122  5.33234  4.25589   398 p - - -
      2.68618  4.42225  2.77519  2.73123  3.46354  2.40513  3.72494  3.29354  2.67741  2.69355  4.24690  2.90347  2.73739  3.18146  2.89801  2.37887  2.77519  2.98518  4.58477  3.61503
```

	0.03268	3.83303	4.55537	0.61958	0.77255	0.48576	0.95510														
399	2.65928	4.75548	2.90106	2.53504	4.22019	3.30093	3.66967	3.63000	1.60907	3.22617	4.09106	2.98715	3.81793	2.84297	2.55489	2.23059	2.94705	3.27888	5.38630	4.10097	399 k - - -
	2.68618	4.42225	2.77519	2.73123	3.46354	2.40513	3.72494	3.29354	2.67741	2.69355	4.24690	2.90347	2.73739	3.18146	2.89801	2.37887	2.77519	2.98518	4.58477	3.61503	
	0.03268	3.83303	4.55537	0.61958	0.77255	0.48576	0.95510														
400	1.94020	4.12787	3.22040	2.98469	4.14082	2.89101	4.09593	3.49170	3.05042	3.25036	4.12838	3.18609	3.63166	3.37355	3.35554	1.36516	2.66799	3.01268	5.51269	4.26187	400 s - - -
	2.68618	4.42225	2.77519	2.73123	3.46354	2.40513	3.72494	3.29354	2.67741	2.69355	4.24690	2.90347	2.73739	3.18146	2.89801	2.37887	2.77519	2.98518	4.58477	3.61503	
	0.03268	3.83303	4.55537	0.61958	0.77255	0.48576	0.95510														
401	2.61765	4.39623	3.28327	3.17805	4.31966	0.79266	4.30013	3.91025	3.35388	3.56921	4.54515	3.44774	3.78610	3.68127	3.59365	2.78855	3.09584	3.44624	5.42460	4.42045	401 g - - -
	2.68618	4.42225	2.77519	2.73123	3.46354	2.40513	3.72494	3.29354	2.67741	2.69355	4.24690	2.90347	2.73739	3.18146	2.89801	2.37887	2.77519	2.98518	4.58477	3.61503	
	0.03268	3.83303	4.55537	0.61958	0.77255	0.48576	0.95510														
402	3.20912	4.62273	4.18444	3.89199	1.07857	3.90702	3.86137	2.73830	3.80015	2.11778	3.47472	3.97513	4.36694	3.97918	3.92960	3.48072	3.51047	2.72599	4.12320	2.49559	402 f - - -
	2.68618	4.42225	2.77519	2.73123	3.46354	2.40513	3.72494	3.29354	2.67741	2.69355	4.24690	2.90347	2.73739	3.18146	2.89801	2.37887	2.77519	2.98518	4.58477	3.61503	
	0.03268	3.83303	4.55537	0.61958	0.77255	0.48576	0.95510														
403	2.83586	3.36887	4.36815	3.81721	3.30485	4.03560	4.46512	1.53882	3.69414	1.76489	3.18340	4.05192	4.38153	3.94209	3.89966	3.36849	3.09068	1.72481	5.08822	3.89399	403 i - - -
	2.68618	4.42225	2.77519	2.73123	3.46354	2.40513	3.72494	3.29354	2.67741	2.69355	4.24690	2.90347	2.73739	3.18146	2.89801	2.37887	2.77519	2.98518	4.58477	3.61503	
	0.03268	3.83303	4.55537	0.61958	0.77255	0.48576	0.95510														
404	2.41170	4.33087	3.07142	2.86838	4.21684	2.39311	4.05516	3.69156	2.99187	3.35293	4.24190	3.18717	1.42594	3.33100	3.31393	2.55887	2.85599	3.23185	5.45854	4.28400	404 p - - -
	2.68618	4.42225	2.77519	2.73123	3.46354	2.40513	3.72494	3.29354	2.67741	2.69355	4.24690	2.90347	2.73739	3.18146	2.89801	2.37887	2.77519	2.98518	4.58477	3.61503	
	0.03268	3.83303	4.55537	0.61958	0.77255	0.48576	0.95510														
405	2.44715	4.24201	3.75129	3.20172	3.09429	3.69196	3.91788	2.16013	3.08584	2.01744	3.23736	3.52734	4.06427	3.38864	3.36370	2.97795	2.90318	2.26359	4.69649	2.55420	405 l - - -
	2.68618	4.42225	2.77519	2.73123	3.46354	2.40513	3.72494	3.29354	2.67741	2.69355	4.24690	2.90347	2.73739	3.18146	2.89801	2.37887	2.77519	2.98518	4.58477	3.61503	
	0.03268	3.83303	4.55537	0.61958	0.77255	0.48576	0.95510														
406	2.40775	4.48969	3.17424	2.62110	3.66416	3.45190	3.67193	2.74974	2.39129	2.66324	3.16862	3.08302	3.84346	2.43070	2.85581	2.69922	2.80380	2.74389	5.04254	3.77692	406 k - - -
	2.68618	4.42225	2.77519	2.73123	3.46354	2.40513	3.72494	3.29354	2.67741	2.69355	4.24690	2.90347	2.73739	3.18146	2.89801	2.37887	2.77519	2.98518	4.58477	3.61503	
	0.03268	3.83303	4.55537	0.61958	0.77255	0.48576	0.95510														
407	3.27202	4.66041	4.03210	3.72890	2.62865	3.68698	3.85349	3.29453	3.45219	2.69596	3.93010	3.88606	4.20159	3.84191	3.58729	3.47606	3.57058	3.18378	0.96876	2.63291	407 w - - -
	2.68618	4.42225	2.77519	2.73123	3.46354	2.40513	3.72494	3.29354	2.67741	2.69355	4.24690	2.90347	2.73739	3.18146	2.89801	2.37887	2.77519	2.98518	4.58477	3.61503	
	0.03268	3.83303	4.55537	0.61958	0.77255	0.48576	0.95510														
408	3.10159	4.50111	4.46641	3.91306	2.32053	4.25229	4.40866	2.20160	3.78800	1.18049	2.52234	4.17848	4.49412	3.94840	3.96218	3.57450	3.32542	2.29254	4.80917	3.53317	408 l - - -
	2.68618	4.42225	2.77519	2.73123	3.46354	2.40513	3.72494	3.29354	2.67741	2.69355	4.24690	2.90347	2.73739	3.18146	2.89801	2.37887	2.77519	2.98518	4.58477	3.61503	
	0.03268	3.83303	4.55537	0.61958	0.77255	0.48576	0.95510														
409	3.06143	4.43575	4.52108	3.96091	3.14778	4.30016	4.63560	1.71045	3.84253	1.33776	2.60141	4.25283	4.54611	4.04146	4.03857	3.62783	3.29302	1.84101	5.13062	4.01418	409 l - - -
	2.68618	4.42225	2.77519	2.73123	3.46354	2.40513	3.72494	3.29354	2.67741	2.69355	4.24690	2.90347	2.73739	3.18146	2.89801	2.37887	2.77519	2.98518	4.58477	3.61503	
	0.03268	3.83303	4.55537	0.61958	0.77255	0.48576	0.95510														
410	2.39658	4.98262	2.48477	1.71741	4.29964	3.26393	3.64414	3.71125	2.43678	3.29230	4.14392	2.79478	3.78623	2.49554	2.86527	2.69423	2.97410	3.36514	5.50954	4.14480	410 e - - -
	2.68618	4.42225	2.77519	2.73123	3.46354	2.40513	3.72494	3.29354	2.67741	2.69355	4.24690	2.90347	2.73739	3.18146	2.89801	2.37887	2.77519	2.98518	4.58477	3.61503	
	0.03268	3.83303	4.55537	0.61958	0.77255	0.48576	0.95510														
411	2.68417	4.79686	2.75388	2.47083	4.14066	3.27301	3.68875	3.68567	2.36404	3.26710	4.14203	1.72668	3.81314	2.88015	2.38276	2.71999	2.97912	3.33111	5.36593	4.02724	411 n - - -
	2.68618	4.42225	2.77519	2.73123	3.46354	2.40513	3.72494	3.29354	2.67741	2.69355	4.24690	2.90347	2.73739	3.18146	2.89801	2.37887	2.77519	2.98518	4.58477	3.61503	
	0.03268	3.83303	4.55537	0.61958	0.77255	0.48576	0.95510														
412	2.44835	4.48600	2.69530	2.54493	4.25377	1.70151	3.89389	3.77305	2.81346	3.40757	4.26503	2.21704	3.70580	3.12346	3.20321	2.54342	2.86170	3.30666	5.53410	4.22287	412 g - - -
	2.68618	4.42225	2.77519	2.73123	3.46354	2.40513	3.72494	3.29354	2.67741	2.69355	4.24690	2.90347	2.73739	3.18146	2.89801	2.37887	2.77519	2.98518	4.58477	3.61503	
	0.03268	3.83303	4.55537	0.61958	0.77255	0.48576	0.95510														
413	2.95371	4.77708	3.08911	2.84570	3.25855	3.41543	1.22850	3.65045	2.68990	3.14913	4.18951	3.25638	3.96612	3.21160	2.95766	3.02494	3.25600	3.38639	4.72403	3.21065	413 h - - -
	2.68618	4.42225	2.77519	2.73123	3.46354	2.40513	3.72494	3.29354	2.67741	2.69355	4.24690	2.90347	2.73739	3.18146	2.89801	2.37887	2.77519	2.98518	4.58477	3.61503	
	0.03268	3.83303	4.55537	0.61958	0.77255	0.48576	0.95510														
414	2.68474	5.07303	2.02378	2.10454	4.38290	2.84471	3.62347	3.84576	2.50110	3.38982	4.20303	2.72466	2.85826	2.57894	3.01451	2.64276	2.94772	3.46103	5.58276	4.17105	414 d - - -
	2.68618	4.42225	2.77519	2.73123	3.46354	2.40513	3.72494	3.29354	2.67741	2.69355	4.24690	2.90347	2.73739	3.18146	2.89801	2.37887	2.77519	2.98518	4.58477	3.61503	
	0.03268	3.83303	4.55537	0.61958	0.77255	0.48576	0.95510														
415	2.96821	4.35221	4.49314	3.99907	3.43371	4.20296	4.75329	1.43994	3.87274	1.96312	3.26880	4.25097	4.55793	4.16278	4.09760	3.58253	3.24283	1.31003	5.35956	4.14340	415 v - - -
	2.68618	4.42225	2.77519	2.73123	3.46354	2.40513	3.72494	3.29354	2.67741	2.69355	4.24690	2.90347	2.73739	3.18146	2.89801	2.37887	2.77519	2.98518	4.58477	3.61503	
	0.03268	3.83303	4.55537	0.61958	0.77255	0.48576	0.95510														
416	3.28721	4.68247	4.16936	3.78810	1.78716	4.07978	3.44718	3.07007	3.66031	2.47715	3.70821	3.79946	4.43049	3.79350	3.81617	3.44017	3.52462	2.97669	3.66742	1.19862	416 y - - -
	2.68618	4.42225	2.77519	2.73123	3.46354	2.40513	3.72494	3.29354	2.67741	2.69355	4.24690	2.90347	2.73739	3.18146	2.89801	2.37887	2.77519	2.98518	4.58477	3.61503	
	0.03268	3.83303	4.55537	0.61958	0.77255	0.48576	0.95510														
417	2.98073	4.37596	4.47380	3.99339	3.39018	4.18577	4.73926	1.26128	3.84917	1.90938	3.23721	4.24330	4.55003	4.14774	4.06749	3.57539	3.25991	1.55285	5.33162	4.10982	417 i - - -
	2.68618	4.42225	2.77519	2.73123	3.46354	2.40513	3.72494	3.29354	2.67741	2.69355	4.24690	2.90347	2.73739	3.18146	2.89801	2.37887	2.77519	2.98518	4.58477	3.61503	
	0.03268	3.83303	4.55537	0.61958	0.77255	0.48576	0.95510														
418	2.94831	5.06869	0.97632	2.31258	4.44069	3.18175	3.89799	4.02012	2.97493	3.63580	4.61368	2.86669	3.83184	3.13755	3.48525	2.91560	3.28628	3.66374	5.61691	4.32095	418 d - - -
	2.68618	4.42225	2.77519	2.73123	3.46354	2.40513	3.72494	3.29354	2.67741	2.69355	4.24690	2.90347	2.73739	3.18146	2.89801	2.37887	2.77519	2.98518	4.58477	3.61503	
	0.03268	3.83303	4.55537	0.61958	0.77255	0.48576	0.95510														
419	2.87082	4.90587	3.23870	2.69682	4.01683	3.51568	2.97334	3.66833	2.01850	3.17734	4.07358	3.11447	3.93125	2.77864	1.48881	2.90439	3.08052	3.37082	5.17815	3.87027	419 r - - -
	2.68618	4.42225	2.77519	2.73123	3.46354	2.40513	3.72494	3.29354	2.67741	2.69355	4.24690	2.90347	2.73739	3.18146	2.89801	2.37887	2.77519	2.98518	4.58477	3.61503	
	0.03268	3.83303	4.55537	0.61958	0.77255	0.48576	0.95510														
420	2.59084	4.23004	3.91003	3.51121	3.61948	3.51913	4.38316	2.12280	3.38997	2.34517	3.51940	3.71710	4.09862	3.74298	3.64338	2.94156	2.56552	1.30673	5.32127	4.08472	420 v - - -
	2.68618	4.42225	2.77519	2.73123	3.46354	2.40513	3.72494	3.29354	2.67741	2.69355	4.24690	2.90347	2.73739	3.18146	2.89801	2.37887	2.77519	2.98518	4.58477	3.61503	
	0.03268	3.83303	4.55537	0.61958	0.77255	0.48576	0.95510														

```
421   2.29672  4.21624  3.17324  2.98785  4.03735  2.94690  4.09847  3.55494  3.05893  3.27847  4.20833  3.21852  3.68259  3.41149  3.34573  1.15212  2.77402  3.09870  5.41316  4.12083    421 s - - -
      2.68618  4.42225  2.77519  2.73123  3.46354  2.40513  3.72494  3.29354  2.67741  2.69355  4.24690  2.90347  2.73739  3.18146  2.89801  2.37887  2.77519  2.98518  4.58477  3.61503
      0.03268  3.83303  4.55537  0.61958  0.77255  0.48576  0.95510
422   2.64384  4.74354  2.75258  2.47790  4.10896  3.24999  3.70328  3.62617  2.39989  3.21017  4.10270  2.94622  3.80271  1.74364  2.73267  2.39445  2.95169  3.27843  5.36875  4.02525    422 q - - -
      2.68618  4.42225  2.77519  2.73123  3.46354  2.40513  3.72494  3.29354  2.67741  2.69355  4.24690  2.90347  2.73739  3.18146  2.89801  2.37887  2.77519  2.98518  4.58477  3.61503
      0.03268  3.83303  4.55537  0.61958  0.77255  0.48576  0.95510
423   2.87606  4.34702  4.23479  3.75722  2.70830  3.95734  4.37747  2.01849  3.65226  1.93706  3.22436  3.99208  4.36390  3.91099  3.87731  3.33194  3.16084  1.32028  4.94730  3.58783    423 v - - -
      2.68618  4.42225  2.77519  2.73123  3.46354  2.40513  3.72494  3.29354  2.67741  2.69355  4.24690  2.90347  2.73739  3.18146  2.89801  2.37887  2.77519  2.98518  4.58477  3.61503
      0.03268  3.83303  4.55537  0.61958  0.77255  0.48576  0.95510
424   2.70125  4.46443  3.30428  3.13815  4.13555  3.15421  4.21835  3.67141  3.19219  3.30231  4.35093  3.44851  0.90433  3.58220  3.44165  2.86766  3.13986  3.32122  5.33234  4.25589    424 p - - -
      2.68618  4.42225  2.77519  2.73123  3.46354  2.40513  3.72494  3.29354  2.67741  2.69355  4.24690  2.90347  2.73739  3.18146  2.89801  2.37887  2.77519  2.98518  4.58477  3.61503
      0.03268  3.83303  4.55537  0.61958  0.77255  0.48576  0.95510
425   3.20912  4.62273  4.18444  3.89199  1.07857  3.90702  3.86137  2.73830  3.80015  2.11778  3.47472  3.97513  4.36694  3.97918  3.92960  3.48072  3.51047  2.72599  4.12320  2.49559    425 f - - -
      2.68618  4.42225  2.77519  2.73123  3.46354  2.40513  3.72494  3.29354  2.67741  2.69355  4.24690  2.90347  2.73739  3.18146  2.89801  2.37887  2.77519  2.98518  4.58477  3.61503
      0.03268  3.83303  4.55537  0.61958  0.77255  0.48576  0.95510
426   2.66464  2.33714  4.21398  3.71191  3.37060  3.69986  4.36628  1.67695  3.55289  2.15143  3.31881  3.87714  4.18912  3.84302  3.75697  3.08773  2.98157  1.89740  5.07326  3.84119    426 i - - -
      2.68618  4.42225  2.77519  2.73123  3.46354  2.40513  3.72494  3.29354  2.67741  2.69355  4.24690  2.90347  2.73739  3.18146  2.89801  2.37887  2.77519  2.98518  4.58477  3.61503
      0.03268  3.83303  4.55537  0.61958  0.77255  0.48576  0.95510
427   2.70125  4.46443  3.30428  3.13815  4.13555  3.15421  4.21835  3.67141  3.19219  3.30231  4.35093  3.44851  0.90433  3.58220  3.44165  2.86766  3.13986  3.32122  5.33234  4.25589    427 p - - -
      2.68618  4.42225  2.77519  2.73123  3.46354  2.40513  3.72494  3.29354  2.67741  2.69355  4.24690  2.90347  2.73739  3.18146  2.89801  2.37887  2.77519  2.98518  4.58477  3.61503
      0.03268  3.83303  4.55537  0.61958  0.77255  0.48576  0.95510
428   2.20861  4.14591  3.24917  2.95840  4.08985  2.93001  4.04641  3.42178  2.97367  3.17682  4.05054  3.18209  3.64596  3.31403  3.28714  1.56016  2.02959  2.97233  5.45737  4.21180    428 s - - -
      2.68618  4.42225  2.77519  2.73123  3.46354  2.40513  3.72494  3.29354  2.67741  2.69355  4.24690  2.90347  2.73739  3.18146  2.89801  2.37887  2.77519  2.98518  4.58477  3.61503
      0.03268  3.83303  4.55537  0.61958  0.77255  0.48576  0.95510
429   2.97882  4.50000  3.97274  3.50138  2.79602  3.93914  4.01350  2.43431  3.28800  1.24176  3.15765  3.78567  4.29688  3.62938  3.51247  3.29878  3.22396  2.42661  4.54873  2.72140    429 l - - -
      2.68618  4.42225  2.77519  2.73123  3.46354  2.40513  3.72494  3.29354  2.67741  2.69355  4.24690  2.90347  2.73739  3.18146  2.89801  2.37887  2.77519  2.98518  4.58477  3.61503
      0.03268  3.83303  4.55537  0.61958  0.77255  0.48576  0.95510
430   1.27301  3.14437  3.79065  3.41427  3.88595  2.96426  4.25270  2.92590  3.30774  2.88292  3.85140  3.43503  3.69555  3.60973  3.54460  2.41758  2.65718  2.58774  5.36355  4.16741    430 a - - -
      2.68618  4.42225  2.77519  2.73123  3.46354  2.40513  3.72494  3.29354  2.67741  2.69355  4.24690  2.90347  2.73739  3.18146  2.89801  2.37887  2.77519  2.98518  4.58477  3.61503
      0.03268  3.83303  4.55537  0.61958  0.77255  0.48576  0.95510
431   2.93703  4.89791  3.11032  2.74002  4.27531  3.43004  3.73906  3.70226  1.09362  3.27630  4.24050  3.16601  3.93369  2.93324  2.42654  2.98198  3.19357  3.41249  5.34669  4.15401    431 k - - -
      2.68618  4.42225  2.77519  2.73123  3.46354  2.40513  3.72494  3.29354  2.67741  2.69355  4.24690  2.90347  2.73739  3.18146  2.89801  2.37887  2.77519  2.98518  4.58477  3.61503
      0.03268  3.83303  4.55537  0.61958  0.77255  0.48576  0.95510
432   2.21183  3.07555  3.62008  3.30535  4.04724  1.30362  4.22002  3.39717  3.24902  3.17836  4.07699  3.38073  3.65840  3.56517  3.49596  2.41737  2.69749  2.95088  5.39501  4.23437    432 g - - -
      2.68618  4.42225  2.77519  2.73123  3.46354  2.40513  3.72494  3.29354  2.67741  2.69355  4.24690  2.90347  2.73739  3.18146  2.89801  2.37887  2.77519  2.98518  4.58477  3.61503
      0.03268  3.83303  4.55537  0.61958  0.77255  0.48576  0.95510
433   2.20547  4.43745  2.92273  2.37790  4.00996  3.15081  3.77836  3.36390  2.64467  3.05029  3.90050  2.99899  3.72533  2.98629  3.04496  2.05564  2.27831  3.00374  5.33092  4.04551    433 s - - -
      2.68618  4.42225  2.77519  2.73123  3.46354  2.40513  3.72494  3.29354  2.67741  2.69355  4.24690  2.90347  2.73739  3.18146  2.89801  2.37887  2.77519  2.98518  4.58477  3.61503
      0.03268  3.83303  4.55537  0.61958  0.77255  0.48576  0.95510
434   2.34404  4.26981  3.19907  2.78271  3.83765  2.15903  3.85560  3.20777  2.75896  2.91414  3.11414  3.12847  3.72225  3.10893  3.11603  2.10473  2.71491  2.86281  5.21734  3.95145    434 s - - -
      2.68618  4.42225  2.77519  2.73123  3.46354  2.40513  3.72494  3.29354  2.67741  2.69355  4.24690  2.90347  2.73739  3.18146  2.89801  2.37887  2.77519  2.98518  4.58477  3.61503
      0.03268  3.83303  4.55537  0.61958  0.77255  0.48576  0.95510
435   2.31572  4.31315  2.99558  2.69170  4.10425  3.02334  3.88249  3.51043  2.76533  3.18519  4.02669  2.62458  3.67618  3.10678  3.13603  1.78066  2.19387  3.07630  5.42433  4.14656    435 s - - -
      2.68618  4.42225  2.77519  2.73123  3.46354  2.40513  3.72494  3.29354  2.67741  2.69355  4.24690  2.90347  2.73739  3.18146  2.89801  2.37887  2.77519  2.98518  4.58477  3.61503
      0.03268  3.83303  4.55537  0.61958  0.77255  0.48576  0.95510
436   2.71632  4.39926  3.60326  3.19235  3.33686  3.55517  4.06965  2.57326  3.03246  1.36565  3.35448  3.52392  3.12238  3.43140  3.29962  2.97315  3.02362  2.47605  4.99865  3.72594    436 l - - -
      2.68618  4.42225  2.77519  2.73123  3.46354  2.40513  3.72494  3.29354  2.67741  2.69355  4.24690  2.90347  2.73739  3.18146  2.89801  2.37887  2.77519  2.98518  4.58477  3.61503
      0.03268  3.83303  4.55537  0.61958  0.77255  0.48576  0.95510
437   2.96214  4.80276  3.44889  2.96907  4.14736  3.45685  3.79770  3.68689  2.22438  3.21005  4.21188  3.33151  3.96192  3.02053  1.04043  3.04858  3.23116  3.41270  5.24678  4.08393    437 r - - -
      2.68618  4.42225  2.77519  2.73123  3.46354  2.40513  3.72494  3.29354  2.67741  2.69355  4.24690  2.90347  2.73739  3.18146  2.89801  2.37887  2.77519  2.98518  4.58477  3.61503
      0.03268  3.83303  4.55537  0.61958  0.77255  0.48576  0.95510
438   2.87606  4.34702  4.23479  3.75722  2.70830  3.95734  4.37747  2.01849  3.65226  1.93706  3.22436  3.99208  4.36390  3.91099  3.87731  3.33194  3.16084  1.32028  4.94730  3.58783    438 v - - -
      2.68618  4.42225  2.77519  2.73123  3.46354  2.40513  3.72494  3.29354  2.67741  2.69355  4.24690  2.90347  2.73739  3.18146  2.89801  2.37887  2.77519  2.98518  4.58477  3.61503
      0.03268  3.83303  4.55537  0.61958  0.77255  0.48576  0.95510
439   2.79165  4.62319  3.27166  2.83755  3.04992  3.54686  2.06751  3.11303  2.58257  2.18928  3.70939  3.23625  3.98514  3.08081  2.87293  2.90970  3.04226  2.92115  4.59610  3.06627    439 h - - -
      2.68618  4.42225  2.77519  2.73123  3.46354  2.40513  3.72494  3.29354  2.67741  2.69355  4.24690  2.90347  2.73739  3.18146  2.89801  2.37887  2.77519  2.98518  4.58477  3.61503
      0.03268  3.83303  4.55537  0.61958  0.77255  0.48576  0.95510
440   2.91965  5.02079  2.47306  1.08285  4.36227  3.24954  3.83381  3.80837  2.70946  3.43216  4.40865  2.91254  3.85246  3.05076  3.11366  2.90651  3.22382  3.49656  5.54134  4.26436    440 e - - -
      2.68618  4.42225  2.77519  2.73123  3.46354  2.40513  3.72494  3.29354  2.67741  2.69355  4.24690  2.90347  2.73739  3.18146  2.89801  2.37887  2.77519  2.98518  4.58477  3.61503
      0.03268  3.83303  4.55537  0.61958  0.77255  0.48576  0.95510
441   2.50532  4.36025  3.86472  3.37655  1.90483  3.77326  3.66474  2.72325  3.28378  2.31021  3.40243  3.59295  4.15453  3.51461  3.52220  3.08353  3.06943  2.57765  4.11054  2.03849    441 f - - -
      2.68618  4.42225  2.77519  2.73123  3.46354  2.40513  3.72494  3.29354  2.67741  2.69355  4.24690  2.90347  2.73739  3.18146  2.89801  2.37887  2.77519  2.98518  4.58477  3.61503
      0.03268  3.83303  4.55537  0.61958  0.77255  0.48576  0.95510
442   2.69182  4.68600  2.68850  2.57033  4.04344  3.15836  3.89101  3.74595  2.79175  3.39751  4.35350  1.22987  3.80860  3.16424  3.14460  2.76087  3.07272  3.37215  5.35398  3.99503    442 n - - -
      2.68618  4.42225  2.77519  2.73123  3.46354  2.40513  3.72494  3.29354  2.67741  2.69355  4.24690  2.90347  2.73739  3.18146  2.89801  2.37887  2.77519  2.98518  4.58477  3.61503
      0.03268  3.83303  4.55537  0.61958  0.77255  0.48576  0.95510
443   2.61765  4.39623  3.28327  3.17805  4.31966  0.79266  4.30013  3.91025  3.35388  3.56921  4.54515  3.44774  3.78610  3.68127  3.59365  2.78855  3.09584  3.44624  5.42460  4.42045    443 g - - -
```

	2.68618	4.42225	2.77519	2.73123	3.46354	2.40513	3.72494	3.29354	2.67741	2.69355	4.24690	2.90347	2.73739	3.18146	2.89801	2.37887	2.77519	2.98518	4.58477	3.61503	
	0.03268	3.83303	4.55537	0.61958	0.77255	0.48576	0.95510														
444	2.84332	5.07663	2.81432	2.28650	4.42901	3.42434	3.57730	3.82360	1.53755	3.32596	4.17697	2.92876	3.86896	2.43561	2.35774	2.82148	3.05440	3.48742	5.44693	4.17383	444 k - - -
	2.68618	4.42225	2.77519	2.73123	3.46354	2.40513	3.72494	3.29354	2.67741	2.69355	4.24690	2.90347	2.73739	3.18146	2.89801	2.37887	2.77519	2.98518	4.58477	3.61503	
	0.03268	3.83303	4.55537	0.61958	0.77255	0.48576	0.95510														
445	2.74526	4.79809	3.22953	2.62514	4.06565	3.50322	3.58797	3.42254	1.62903	3.00126	3.25297	3.07033	3.88601	2.75196	2.18348	2.79858	2.95301	3.14456	5.23118	3.98563	445 k - - -
	2.68618	4.42225	2.77519	2.73123	3.46354	2.40513	3.72494	3.29354	2.67741	2.69355	4.24690	2.90347	2.73739	3.18146	2.89801	2.37887	2.77519	2.98518	4.58477	3.61503	
	0.03268	3.83303	4.55537	0.61958	0.77255	0.48576	0.95510														
446	2.89033	4.64423	3.40056	2.98898	2.26632	3.64223	1.82246	3.19358	2.87278	2.69386	3.76733	3.33464	4.07280	3.24265	3.17835	2.99804	3.14529	3.00453	4.16807	2.52619	446 h - - -
	2.68618	4.42225	2.77519	2.73123	3.46354	2.40513	3.72494	3.29354	2.67741	2.69355	4.24690	2.90347	2.73739	3.18146	2.89801	2.37887	2.77519	2.98518	4.58477	3.61503	
	0.03268	3.83303	4.55537	0.61958	0.77255	0.48576	0.95510														
447	1.94377	4.11077	3.29401	2.98907	4.12509	2.90321	4.06816	3.43660	3.01554	3.19486	4.05172	3.19019	3.62688	3.33399	3.33284	1.53770	2.28571	2.96863	5.48521	4.26000	447 s - - -
	2.68618	4.42225	2.77519	2.73123	3.46354	2.40513	3.72494	3.29354	2.67741	2.69355	4.24690	2.90347	2.73739	3.18146	2.89801	2.37887	2.77519	2.98518	4.58477	3.61503	
	0.03268	3.83303	4.55537	0.61958	0.77255	0.48576	0.95510														
448	3.14980	4.55087	4.39430	3.91858	1.78697	4.18711	4.10769	2.33204	3.77728	1.35123	3.03811	4.09533	4.48616	3.94670	3.93894	3.55080	3.39063	2.38646	4.41610	2.89487	448 l - - -
	2.68618	4.42225	2.77519	2.73123	3.46354	2.40513	3.72494	3.29354	2.67741	2.69355	4.24690	2.90347	2.73739	3.18146	2.89801	2.37887	2.77519	2.98518	4.58477	3.61503	
	0.03268	3.83303	4.55537	0.61958	0.77255	0.48576	0.95510														
449	2.21044	4.14592	3.25552	2.95895	4.08042	2.93456	4.04303	3.40805	2.97033	3.16500	4.03897	3.18370	3.64789	3.31088	3.28430	1.62966	1.93819	2.96327	5.44908	4.20463	449 s - - -
	2.68618	4.42225	2.77519	2.73123	3.46354	2.40513	3.72494	3.29354	2.67741	2.69355	4.24690	2.90347	2.73739	3.18146	2.89801	2.37887	2.77519	2.98518	4.58477	3.61503	
	0.03268	3.83303	4.55537	0.61958	0.77255	0.48576	0.95510														
450	2.87759	5.33786	1.33387	1.86786	4.63127	3.15364	3.72174	4.12359	2.74483	3.67936	4.56019	2.64720	3.78205	2.90177	3.31840	2.77979	3.17457	3.72921	5.83535	4.36780	450 d - - -
	2.68618	4.42225	2.77519	2.73123	3.46354	2.40513	3.72494	3.29354	2.67741	2.69355	4.24690	2.90347	2.73739	3.18146	2.89801	2.37887	2.77519	2.98518	4.58477	3.61503	
	0.03268	3.83303	4.55537	0.61958	0.77255	0.48576	0.95510														
451	2.71287	5.10136	1.82269	2.16084	4.41211	3.21626	3.63998	3.89188	2.52064	3.43173	4.25769	2.72043	3.13503	2.32168	3.02618	2.66567	2.98291	3.50452	5.61220	4.19353	451 d - - -
	2.68618	4.42225	2.77519	2.73123	3.46354	2.40513	3.72494	3.29354	2.67741	2.69355	4.24690	2.90347	2.73739	3.18146	2.89801	2.37887	2.77519	2.98518	4.58477	3.61503	
	0.03268	3.83303	4.55537	0.61958	0.77255	0.48576	0.95510														
452	3.20630	4.63349	4.10661	3.68461	2.06886	3.99217	3.46070	3.12413	3.44188	2.54856	3.71924	3.74111	4.35515	3.69093	3.60684	3.36753	3.44015	3.00025	1.68288	1.81536	452 w - - -
	2.68618	4.42225	2.77519	2.73123	3.46354	2.40513	3.72494	3.29354	2.67741	2.69355	4.24690	2.90347	2.73739	3.18146	2.89801	2.37887	2.77519	2.98518	4.58477	3.61503	
	0.03268	3.83303	4.55537	0.61958	0.77255	0.48576	0.95510														
453	2.61765	4.39623	3.28327	3.17805	4.31966	0.79266	4.30013	3.91025	3.35388	3.56921	4.54515	3.44774	3.78610	3.68127	3.59365	2.78855	3.09584	3.44624	5.42460	4.42045	453 g - - -
	2.68618	4.42225	2.77519	2.73123	3.46354	2.40513	3.72494	3.29354	2.67741	2.69355	4.24690	2.90347	2.73739	3.18146	2.89801	2.37887	2.77519	2.98518	4.58477	3.61503	
	0.03268	3.83303	4.55537	0.61958	0.77255	0.48576	0.95510														
454	2.91965	5.02079	2.47306	1.08285	4.36227	3.24954	3.83381	3.80837	2.70946	3.43216	4.40865	2.91254	3.85246	3.05076	3.11366	2.90651	3.22382	3.49656	5.54134	4.26436	454 e - - -
	2.68618	4.42225	2.77519	2.73123	3.46354	2.40513	3.72494	3.29354	2.67741	2.69355	4.24690	2.90347	2.73739	3.18146	2.89801	2.37887	2.77519	2.98518	4.58477	3.61503	
	0.03268	3.83303	4.55537	0.61958	0.77255	0.48576	0.95510														
455	2.96214	4.80276	3.44889	2.96907	4.14736	3.45685	3.79770	3.68689	2.22438	3.21005	4.21188	3.33151	3.96192	3.02053	1.04043	3.04858	3.23116	3.41270	5.24678	4.08393	455 r - - -
	2.68618	4.42225	2.77519	2.73123	3.46354	2.40513	3.72494	3.29354	2.67741	2.69355	4.24690	2.90347	2.73739	3.18146	2.89801	2.37887	2.77519	2.98518	4.58477	3.61503	
	0.03268	3.83303	4.55537	0.61958	0.77255	0.48576	0.95510														
456	2.69767	4.54958	3.27264	2.78731	3.52708	3.52748	3.78424	2.88666	2.54762	2.37684	2.40666	3.21934	3.94760	2.27356	2.85744	2.84811	2.94477	2.74532	5.04303	3.76374	456 q - - -
	2.68618	4.42225	2.77519	2.73123	3.46354	2.40513	3.72494	3.29354	2.67741	2.69355	4.24690	2.90347	2.73739	3.18146	2.89801	2.37887	2.77519	2.98518	4.58477	3.61503	
	0.03268	3.83303	4.55537	0.61958	0.77255	0.48576	0.95510														
457	3.27202	4.66041	4.03210	3.72890	2.62865	3.68698	3.85349	3.29453	3.45219	2.69596	3.93010	3.88606	4.20159	3.84191	3.58729	3.47606	3.57058	3.18378	0.96876	2.63291	457 w - - -
	2.68618	4.42225	2.77519	2.73123	3.46354	2.40513	3.72494	3.29354	2.67741	2.69355	4.24690	2.90347	2.73739	3.18146	2.89801	2.37887	2.77519	2.98518	4.58477	3.61503	
	0.03268	3.83303	4.55537	0.61958	0.77255	0.48576	0.95510														
458	3.11165	4.58599	4.12585	3.76620	3.12182	3.93147	4.43434	2.32453	3.53431	0.92536	3.15834	4.04543	4.37407	3.91210	3.71656	3.49871	3.40796	2.35149	4.98612	3.70011	458 l - - -
	2.68618	4.42225	2.77519	2.73123	3.46354	2.40513	3.72494	3.29354	2.67741	2.69355	4.24690	2.90347	2.73739	3.18146	2.89801	2.37887	2.77519	2.98518	4.58477	3.61503	
	0.03268	3.83303	4.55537	0.61958	0.77255	0.48576	0.95510														
459	2.30261	4.39107	3.15252	2.67756	3.69626	3.32654	3.74614	2.59432	2.61797	2.73328	3.62811	3.10230	3.80572	2.70698	2.98748	2.17741	2.77624	2.74092	5.08659	3.81943	459 s - - -
	2.68618	4.42225	2.77519	2.73123	3.46354	2.40513	3.72494	3.29354	2.67741	2.69355	4.24690	2.90347	2.73739	3.18146	2.89801	2.37887	2.77519	2.98518	4.58477	3.61503	
	0.03268	3.83303	4.55537	0.61958	0.77255	0.48576	0.95510														
460	2.91965	5.02079	2.47306	1.08285	4.36227	3.24954	3.83381	3.80837	2.70946	3.43216	4.40865	2.91254	3.85246	3.05076	3.11366	2.90651	3.22382	3.49656	5.54134	4.26436	460 e - - -
	2.68618	4.42225	2.77519	2.73123	3.46354	2.40513	3.72494	3.29354	2.67741	2.69355	4.24690	2.90347	2.73739	3.18146	2.89801	2.37887	2.77519	2.98518	4.58477	3.61503	
	0.03268	3.83303	4.55537	0.61958	0.77255	0.48576	0.95510														
461	2.70125	4.46443	3.30428	3.13815	4.13555	3.15421	4.21835	3.67141	3.19219	3.30231	4.35093	3.44851	0.90433	3.58220	3.44165	2.86766	3.13986	3.32122	5.33234	4.25589	461 p - - -
	2.68618	4.42225	2.77519	2.73123	3.46354	2.40513	3.72494	3.29354	2.67741	2.69355	4.24690	2.90347	2.73739	3.18146	2.89801	2.37887	2.77519	2.98518	4.58477	3.61503	
	0.03268	3.83303	4.55537	0.61958	0.77255	0.48576	0.95510														
462	2.91965	5.02079	2.47306	1.08285	4.36227	3.24954	3.83381	3.80837	2.70946	3.43216	4.40865	2.91254	3.85246	3.05076	3.11366	2.90651	3.22382	3.49656	5.54134	4.26436	462 e - - -
	2.68618	4.42225	2.77519	2.73123	3.46354	2.40513	3.72494	3.29354	2.67741	2.69355	4.24690	2.90347	2.73739	3.18146	2.89801	2.37887	2.77519	2.98518	4.58477	3.61503	
	0.03268	3.83303	4.55537	0.61958	0.77255	0.48576	0.95510														
463	2.96214	4.80276	3.44889	2.96907	4.14736	3.45685	3.79770	3.68689	2.22438	3.21005	4.21188	3.33151	3.96192	3.02053	1.04043	3.04858	3.23116	3.41270	5.24678	4.08393	463 r - - -
	2.68618	4.42225	2.77519	2.73123	3.46354	2.40513	3.72494	3.29354	2.67741	2.69355	4.24690	2.90347	2.73739	3.18146	2.89801	2.37887	2.77519	2.98518	4.58477	3.61503	
	0.03268	3.83303	4.55537	0.61958	0.77255	0.48576	0.95510														
464	3.19134	4.69630	3.78054	3.50310	2.29611	3.78460	3.60201	3.20618	3.36176	2.67014	3.88549	3.67575	4.25966	3.67727	3.55331	3.33175	3.47801	3.07495	3.93816	1.03399	464 y - - -
	2.68618	4.42225	2.77519	2.73123	3.46354	2.40513	3.72494	3.29354	2.67741	2.69355	4.24690	2.90347	2.73739	3.18146	2.89801	2.37887	2.77519	2.98518	4.58477	3.61503	
	0.03268	3.83303	4.55537	0.61958	0.77255	0.48576	0.95510														
465	2.91965	5.02079	2.47306	1.08285	4.36227	3.24954	3.83381	3.80837	2.70946	3.43216	4.40865	2.91254	3.85246	3.05076	3.11366	2.90651	3.22382	3.49656	5.54134	4.26436	465 e - - -
	2.68618	4.42225	2.77519	2.73123	3.46354	2.40513	3.72494	3.29354	2.67741	2.69355	4.24690	2.90347	2.73739	3.18146	2.89801	2.37887	2.77519	2.98518	4.58477	3.61503	

0.03268 3.83303 4.55537 0.61958 0.77255 0.48576 0.95510
466 2.38791 2.08265 3.97247 3.48647 2.78058 3.42421 4.11767 2.41519 3.36318 2.28057 3.36304 3.63158 3.97562 3.63090 3.58143 2.81037 2.83665 2.23109 4.85401 3.54499 466 c - - -
2.68618 4.42225 2.77519 2.73123 3.46354 2.40513 3.72494 3.29354 2.67741 2.69355 4.24690 2.90347 2.73739 3.18146 2.89801 2.37887 2.77519 2.98518 4.58477 3.61503
0.03268 3.83303 4.55537 0.61958 0.77255 0.48576 0.95510
467 2.78178 4.92937 2.81366 2.46057 3.89077 3.39721 2.61691 3.65946 2.24594 3.18368 4.07075 2.94976 3.86116 1.93984 2.56361 2.78911 3.01769 3.34706 5.17770 3.76769 467 q - - -
2.68618 4.42225 2.77519 2.73123 3.46354 2.40513 3.72494 3.29354 2.67741 2.69355 4.24690 2.90347 2.73739 3.18146 2.89801 2.37887 2.77519 2.98518 4.58477 3.61503
0.03268 3.83303 4.55537 0.61958 0.77255 0.48576 0.95510
468 2.52002 4.59958 2.61481 2.46351 4.15147 3.08559 3.81757 3.72971 2.69538 3.37324 4.24827 1.59503 3.73665 3.04295 3.08219 2.35480 2.90519 3.30496 5.47110 4.08565 468 n - - -
2.68618 4.42225 2.77519 2.73123 3.46354 2.40513 3.72494 3.29354 2.67741 2.69355 4.24690 2.90347 2.73739 3.18146 2.89801 2.37887 2.77519 2.98518 4.58477 3.61503
0.03268 3.83303 4.55537 0.61958 0.77255 0.48576 0.95510
469 2.98194 4.35591 4.48219 3.92982 2.75513 4.21989 4.54548 1.68776 3.82899 1.61066 3.04647 4.18915 4.49725 4.02771 4.02415 3.54678 3.21906 1.58865 5.05459 3.85038 469 v - - -
2.68618 4.42225 2.77519 2.73123 3.46354 2.40513 3.72494 3.29354 2.67741 2.69355 4.24690 2.90347 2.73739 3.18146 2.89801 2.37887 2.77519 2.98518 4.58477 3.61503
0.03268 3.83303 4.55537 0.61958 0.77255 0.48576 0.95510
470 3.20912 4.62273 4.18444 3.89199 1.07857 3.90702 3.86137 2.73830 3.80015 2.11778 3.47472 3.97513 4.36694 3.97918 3.92960 3.48072 3.51047 2.72599 4.12320 2.49559 470 f - - -
2.68618 4.42225 2.77519 2.73123 3.46354 2.40513 3.72494 3.29354 2.67741 2.69355 4.24690 2.90347 2.73739 3.18146 2.89801 2.37887 2.77519 2.98518 4.58477 3.61503
0.03268 3.83303 4.55537 0.61958 0.77255 0.48576 0.95510
471 2.91965 5.02079 2.47306 1.08285 4.36227 3.24954 3.83381 3.80837 2.70946 3.43216 4.40865 2.91254 3.85246 3.05076 3.11366 2.90651 3.22382 3.49656 5.54134 4.26436 471 e - - -
2.68618 4.42225 2.77519 2.73123 3.46354 2.40513 3.72494 3.29354 2.67741 2.69355 4.24690 2.90347 2.73739 3.18146 2.89801 2.37887 2.77519 2.98518 4.58477 3.61503
0.03268 3.83303 4.55537 0.61958 0.77255 0.48576 0.95510
472 2.85084 4.83409 2.87832 2.63320 3.99721 3.34074 3.79090 3.62260 2.45691 3.12880 4.15852 3.09230 3.89411 1.30591 2.74155 2.90061 3.14819 3.35495 5.27660 3.96796 472 q - - -
2.68618 4.42225 2.77519 2.73123 3.46354 2.40513 3.72494 3.29354 2.67741 2.69355 4.24690 2.90347 2.73739 3.18146 2.89801 2.37887 2.77519 2.98518 4.58477 3.61503
0.03268 3.83303 4.55537 0.61958 0.77255 0.48576 0.95510
473 3.28721 4.68247 4.16936 3.78810 1.78716 4.07978 3.44718 3.07007 3.66031 2.47715 3.70821 3.79946 4.43049 3.79350 3.81617 3.44017 3.52462 2.97669 3.66742 1.19862 473 y - - -
2.68618 4.42225 2.77519 2.73123 3.46354 2.40513 3.72494 3.29354 2.67741 2.69355 4.24690 2.90347 2.73739 3.18146 2.89801 2.37887 2.77519 2.98518 4.58477 3.61503
0.03268 3.83303 4.55537 0.61958 0.77255 0.48576 0.95510
474 2.68614 4.87716 2.54309 1.59290 4.17622 3.25906 3.67978 3.56769 2.48821 3.03260 4.09898 2.84059 3.79540 2.85991 2.90446 2.49468 2.96648 3.24291 5.44704 4.07886 474 e - - -
2.68618 4.42225 2.77519 2.73123 3.46354 2.40513 3.72494 3.29354 2.67741 2.69355 4.24690 2.90347 2.73739 3.18146 2.89801 2.37887 2.77519 2.98518 4.58477 3.61503
0.03268 3.83303 4.55537 0.61958 0.77255 0.48576 0.95510
475 2.54751 4.51246 2.51399 2.63379 4.29559 1.22911 3.99865 3.80152 3.00002 3.46263 4.35765 3.05587 3.74262 3.26159 3.38853 2.65121 2.96908 3.35656 5.51719 4.30108 475 g - - -
2.68618 4.42225 2.77519 2.73123 3.46354 2.40513 3.72494 3.29354 2.67741 2.69355 4.24690 2.90347 2.73739 3.18146 2.89801 2.37887 2.77519 2.98518 4.58477 3.61503
0.03268 3.83303 4.55537 0.61958 0.77255 0.48576 0.95510
476 2.81017 4.66711 3.46293 2.85619 3.45980 3.56029 3.64017 3.28257 2.25166 2.83549 3.79647 3.24128 3.95956 2.92929 1.57719 2.91768 3.03042 3.05000 3.43979 3.45422 476 r - - -
2.68618 4.42225 2.77519 2.73123 3.46354 2.40513 3.72494 3.29354 2.67741 2.69355 4.24690 2.90347 2.73739 3.18146 2.89801 2.37887 2.77519 2.98518 4.58477 3.61503
0.03268 3.83303 4.55537 0.61958 0.77255 0.48576 0.95510
477 2.91965 5.02079 2.47306 1.08285 4.36227 3.24954 3.83381 3.80837 2.70946 3.43216 4.40865 2.91254 3.85246 3.05076 3.11366 2.90651 3.22382 3.49656 5.54134 4.26436 477 e - - -
2.68618 4.42225 2.77519 2.73123 3.46354 2.40513 3.72494 3.29354 2.67741 2.69355 4.24690 2.90347 2.73739 3.18146 2.89801 2.37887 2.77519 2.98518 4.58477 3.61503
0.03268 3.83303 4.55537 0.61958 0.77255 0.48576 0.95510
478 3.11165 4.58599 4.12585 3.76620 3.12182 3.93147 4.43434 2.32453 3.53431 0.92536 3.15834 4.04543 4.37407 3.91210 3.71656 3.49871 3.40796 2.35149 4.98612 3.70011 478 l - - -
2.68618 4.42225 2.77519 2.73123 3.46354 2.40513 3.72494 3.29354 2.67741 2.69355 4.24690 2.90347 2.73739 3.18146 2.89801 2.37887 2.77519 2.98518 4.58477 3.61503
0.03268 3.83303 4.55537 0.61958 0.77255 0.48576 0.95510
479 2.29672 4.21624 3.17324 2.98785 4.03735 2.94690 4.09847 3.55494 3.05893 3.27847 4.20833 3.21852 3.68259 3.41149 3.34573 1.15212 2.77402 3.09870 5.41316 4.12083 479 s - - -
2.68618 4.42225 2.77519 2.73123 3.46354 2.40513 3.72494 3.29354 2.67741 2.69355 4.24690 2.90347 2.73739 3.18146 2.89801 2.37887 2.77519 2.98518 4.58477 3.61503
0.03268 3.83303 4.55537 0.61958 0.77255 0.48576 0.95510
480 2.85135 5.33661 1.75604 1.47332 4.62456 3.16627 3.69440 4.11466 2.68229 3.65281 4.51312 2.64681 3.77559 2.86431 3.24536 2.75489 3.13744 3.71498 5.81435 4.34659 480 e - - -
2.68618 4.42225 2.77519 2.73123 3.46354 2.40513 3.72494 3.29354 2.67741 2.69355 4.24690 2.90347 2.73739 3.18146 2.89801 2.37887 2.77519 2.98518 4.58477 3.61503
0.03268 3.83303 4.55537 0.61958 0.77255 0.48576 0.95510
481 2.89507 4.36874 4.24868 3.78761 3.36571 3.98405 4.54844 1.91805 3.61702 1.81558 3.28181 4.04311 4.40936 3.95828 3.84886 3.38250 3.19214 1.24551 5.23093 3.96224 481 v - - -
2.68618 4.42225 2.77519 2.73123 3.46354 2.40513 3.72494 3.29354 2.67741 2.69355 4.24690 2.90347 2.73739 3.18146 2.89801 2.37887 2.77519 2.98518 4.58477 3.61503
0.03268 3.83303 4.55537 0.61958 0.77255 0.48576 0.95510
482 2.98044 4.37817 4.46740 3.98940 3.38614 4.18006 4.73478 1.25382 3.84270 1.90638 3.23588 4.23899 4.54687 4.14343 4.06059 3.57123 3.26057 1.57023 5.32759 4.10374 482 i - - -
2.68618 4.42225 2.77519 2.73123 3.46354 2.40513 3.72494 3.29354 2.67741 2.69355 4.24690 2.90347 2.73739 3.18146 2.89801 2.37887 2.77519 2.98518 4.58477 3.61503
0.03268 3.83303 4.55537 0.61958 0.77255 0.48576 0.95510
483 1.72432 4.35716 3.20091 2.78041 3.84067 3.24086 3.84633 2.99550 2.39943 2.82685 3.75681 3.15538 3.79835 3.08629 2.99504 2.61357 2.79341 2.50725 5.23187 3.97433 483 a - - -
2.68618 4.42225 2.77519 2.73123 3.46354 2.40513 3.72494 3.29354 2.67741 2.69355 4.24690 2.90347 2.73739 3.18146 2.89801 2.37887 2.77519 2.98518 4.58477 3.61503
0.03268 3.83303 4.55537 0.61958 0.77255 0.48576 0.95510
484 2.39139 4.89437 2.45700 1.48390 4.29794 3.21261 3.73566 3.60609 2.58753 3.29280 4.20136 2.82675 3.79398 2.92017 3.01863 2.71572 3.01321 3.27684 5.56057 4.20076 484 e - - -
2.68618 4.42225 2.77519 2.73123 3.46354 2.40513 3.72494 3.29354 2.67741 2.69355 4.24690 2.90347 2.73739 3.18146 2.89801 2.37887 2.77519 2.98518 4.58477 3.61503
0.03268 3.83303 4.55537 0.61958 0.77255 0.48576 0.95510
485 2.10480 4.24451 3.21311 2.87075 3.87533 1.90135 3.94956 3.13709 2.86441 2.52283 3.84836 3.17963 3.73239 3.21984 3.19595 2.51206 2.74108 2.80452 5.28615 4.02154 485 g - - -
2.68618 4.42225 2.77519 2.73123 3.46354 2.40513 3.72494 3.29354 2.67741 2.69355 4.24690 2.90347 2.73739 3.18146 2.89801 2.37887 2.77519 2.98518 4.58477 3.61503
0.03268 3.83303 4.55537 0.61958 0.77255 0.48576 0.95510
486 2.63280 4.54628 3.09997 2.66952 2.88036 2.95481 2.21177 3.19350 2.62870 2.81381 3.73418 3.10401 3.87336 3.00137 2.99666 2.73967 2.89744 2.92972 4.70034 3.19878 486 h - - -
2.68618 4.42225 2.77519 2.73123 3.46354 2.40513 3.72494 3.29354 2.67741 2.69355 4.24690 2.90347 2.73739 3.18146 2.89801 2.37887 2.77519 2.98518 4.58477 3.61503
0.03268 3.83303 4.55537 0.61958 0.77255 0.48576 0.95510
487 2.61765 4.39623 3.28327 3.17805 4.31966 0.79266 4.30013 3.91025 3.35388 3.56921 4.54515 3.44774 3.78610 3.68127 3.59365 2.78855 3.09584 3.44624 5.42460 4.42045 487 g - - -
2.68618 4.42225 2.77519 2.73123 3.46354 2.40513 3.72494 3.29354 2.67741 2.69355 4.24690 2.90347 2.73739 3.18146 2.89801 2.37887 2.77519 2.98518 4.58477 3.61503
0.03268 3.83303 4.55537 0.61958 0.77255 0.48576 0.95510

488	2.89702	4.36953	4.25107	3.78862	3.36325	3.98788	4.54868	1.91784	3.61819	1.80707	3.27836	4.04489	4.41114	3.95852	3.84999	3.38537	3.19322	1.24951	5.22954	3.96184	488 v - - -
	2.68618	4.42225	2.77519	2.73123	3.46354	2.40513	3.72494	3.29354	2.67741	2.69355	4.24690	2.90347	2.73739	3.18146	2.89801	2.37887	2.77519	2.98518	4.58477	3.61503	
	0.03268	3.83303	4.55537	0.61958	0.77255	0.48576	0.95510														
489	2.96214	4.80276	3.44889	2.96907	4.14736	3.45685	3.79770	3.68689	2.22438	3.21005	4.21188	3.33151	3.96192	3.02053	1.04043	3.04858	3.23116	3.41270	5.24678	4.08393	489 r - - -
	2.68618	4.42225	2.77519	2.73123	3.46354	2.40513	3.72494	3.29354	2.67741	2.69355	4.24690	2.90347	2.73739	3.18146	2.89801	2.37887	2.77519	2.98518	4.58477	3.61503	
	0.03268	3.83303	4.55537	0.61958	0.77255	0.48576	0.95510														
490	2.00928	4.13755	3.20261	2.97665	4.13564	2.89492	4.09397	3.49720	3.04523	3.25424	4.13807	3.18334	3.63593	3.37313	3.34871	1.32846	2.67722	3.02023	5.50996	4.25179	490 s - - -
	2.68618	4.42225	2.77519	2.73123	3.46354	2.40513	3.72494	3.29354	2.67741	2.69355	4.24690	2.90347	2.73739	3.18146	2.89801	2.37887	2.77519	2.98518	4.58477	3.61503	
	0.03268	3.83303	4.55537	0.61958	0.77255	0.48576	0.95510														
491	2.94831	5.06869	0.97632	2.31258	4.44069	3.18175	3.89799	4.02012	2.97493	3.63580	4.61368	2.86669	3.83184	3.13755	3.48525	2.91560	3.28628	3.66374	5.61691	4.32095	491 d - - -
	2.68618	4.42225	2.77519	2.73123	3.46354	2.40513	3.72494	3.29354	2.67741	2.69355	4.24690	2.90347	2.73739	3.18146	2.89801	2.37887	2.77519	2.98518	4.58477	3.61503	
	0.03268	3.83303	4.55537	0.61958	0.77255	0.48576	0.95510														
492	2.61765	4.39623	3.28327	3.17805	4.31966	0.79266	4.30013	3.91025	3.35388	3.56921	4.54515	3.44774	3.78610	3.68127	3.59365	2.78855	3.09584	3.44624	5.42460	4.42045	492 g - - -
	2.68618	4.42225	2.77519	2.73123	3.46354	2.40513	3.72494	3.29354	2.67741	2.69355	4.24690	2.90347	2.73739	3.18146	2.89801	2.37887	2.77519	2.98518	4.58477	3.61503	
	0.03268	3.83303	4.55537	0.61958	0.77255	0.48576	0.95510														
493	2.93703	4.89791	3.11032	2.74002	4.27531	3.43004	3.73906	3.70226	1.09362	3.27630	4.24050	3.16601	3.93369	2.93324	2.42654	2.98198	3.19357	3.41249	5.34669	4.15401	493 k - - -
	2.68618	4.42225	2.77519	2.73123	3.46354	2.40513	3.72494	3.29354	2.67741	2.69355	4.24690	2.90347	2.73739	3.18146	2.89801	2.37887	2.77519	2.98518	4.58477	3.61503	
	0.03268	3.83303	4.55537	0.61958	0.77255	0.48576	0.95510														
494	2.39269	4.23746	3.39425	3.14521	3.93517	3.07784	4.16539	3.05754	3.08739	2.92240	3.97601	3.36046	3.77873	3.48436	3.34758	2.59359	1.24059	2.75673	5.37648	4.15199	494 t - - -
	2.68618	4.42225	2.77519	2.73123	3.46354	2.40513	3.72494	3.29354	2.67741	2.69355	4.24690	2.90347	2.73739	3.18146	2.89801	2.37887	2.77519	2.98518	4.58477	3.61503	
	0.03268	3.83303	4.55537	0.61958	0.77255	0.48576	0.95510														
495	3.11165	4.58599	4.12585	3.76620	3.12182	3.93147	4.43434	2.32453	3.53431	0.92536	3.15834	4.04543	4.37407	3.91210	3.71656	3.49871	3.40796	2.35149	4.98612	3.70011	495 l - - -
	2.68618	4.42225	2.77519	2.73123	3.46354	2.40513	3.72494	3.29354	2.67741	2.69355	4.24690	2.90347	2.73739	3.18146	2.89801	2.37887	2.77519	2.98518	4.58477	3.61503	
	0.03268	3.83303	4.55537	0.61958	0.77255	0.48576	0.95510														
496	3.02617	4.42174	4.44914	3.92784	3.23346	4.22538	4.62859	1.32984	3.75728	1.72151	2.67581	4.20743	4.53104	4.03743	3.96502	3.58482	3.27910	1.85040	5.18446	3.99861	496 i - - -
	2.68618	4.42225	2.77519	2.73123	3.46354	2.40513	3.72494	3.29354	2.67741	2.69355	4.24690	2.90347	2.73739	3.18146	2.89801	2.37887	2.77519	2.98518	4.58477	3.61503	
	0.03268	3.83303	4.55537	0.61958	0.77255	0.48576	0.95510														
497	1.34986	4.08260	3.27419	3.01899	4.15975	2.53320	4.11089	3.51876	3.09641	3.25674	4.10171	3.19448	3.60946	3.39092	3.40402	2.10215	2.63092	3.01421	5.51808	4.29977	497 a - - -
	2.68618	4.42225	2.77519	2.73123	3.46354	2.40513	3.72494	3.29354	2.67741	2.69355	4.24690	2.90347	2.73739	3.18146	2.89801	2.37887	2.77519	2.98518	4.58477	3.61503	
	0.03268	3.83303	4.55537	0.61958	0.77255	0.48576	0.95510														
498	2.85084	4.83409	2.87832	2.63320	3.99721	3.34074	3.79090	3.62260	2.45691	3.12880	4.15852	3.09230	3.89411	1.30591	2.74155	2.90061	3.14819	3.35495	5.27660	3.96796	498 q - - -
	2.68618	4.42225	2.77519	2.73123	3.46354	2.40513	3.72494	3.29354	2.67741	2.69355	4.24690	2.90347	2.73739	3.18146	2.89801	2.37887	2.77519	2.98518	4.58477	3.61503	
	0.03268	3.83303	4.55537	0.61958	0.77255	0.48576	0.95510														
499	2.70125	4.46443	3.30428	3.13815	4.13555	3.15421	4.21835	3.67141	3.19219	3.30231	4.35093	3.44851	0.90433	3.58220	3.44165	2.86766	3.13986	3.32122	5.33234	4.25589	499 p - - -
	2.68618	4.42225	2.77519	2.73123	3.46354	2.40513	3.72494	3.29354	2.67741	2.69355	4.24690	2.90347	2.73739	3.18146	2.89801	2.37887	2.77519	2.98518	4.58477	3.61503	
	0.03268	3.83303	4.55537	0.61958	0.77255	0.48576	0.95510														
500	2.95371	4.77708	3.08911	2.84570	3.25855	3.41543	1.22850	3.65045	2.68990	3.14913	4.18951	3.25638	3.96612	3.21160	2.95766	3.02494	3.25600	3.38639	4.72403	3.21065	500 h - - -
	2.68618	4.42225	2.77519	2.73123	3.46354	2.40513	3.72494	3.29354	2.67741	2.69355	4.24690	2.90347	2.73739	3.18146	2.89801	2.37887	2.77519	2.98518	4.58477	3.61503	
	0.03268	3.83303	4.55537	0.61958	0.77255	0.48576	0.95510														
501	2.64659	4.26585	3.84209	3.34167	3.48845	3.63710	4.21033	2.16265	3.21630	2.06097	3.38787	3.64041	4.11354	3.55898	3.50308	2.98964	2.29612	1.64680	5.15437	3.92552	501 v - - -
	2.68618	4.42225	2.77519	2.73123	3.46354	2.40513	3.72494	3.29354	2.67741	2.69355	4.24690	2.90347	2.73739	3.18146	2.89801	2.37887	2.77519	2.98518	4.58477	3.61503	
	0.03268	3.83303	4.55537	0.61958	0.77255	0.48576	0.95510														
502	2.56925	4.84941	3.20082	2.65631	4.25214	3.46851	3.60150	3.60262	1.90416	3.16279	4.03826	3.08013	3.89529	2.76000	1.61126	2.83217	3.01355	3.29366	5.32043	4.09866	502 r - - -
	2.68618	4.42225	2.77519	2.73123	3.46354	2.40513	3.72494	3.29354	2.67741	2.69355	4.24690	2.90347	2.73739	3.18146	2.89801	2.37887	2.77519	2.98518	4.58477	3.61503	
	0.03268	3.83303	4.55537	0.61958	0.77255	0.48576	0.95510														
503	2.44017	4.48072	2.79776	2.55256	4.18254	1.87577	3.81530	3.60895	2.39380	3.24591	4.09338	2.97262	3.71713	3.02342	3.00650	2.26318	2.81826	3.19130	5.45904	4.16589	503 g - - -
	2.68618	4.42225	2.77519	2.73123	3.46354	2.40513	3.72494	3.29354	2.67741	2.69355	4.24690	2.90347	2.73739	3.18146	2.89801	2.37887	2.77519	2.98518	4.58477	3.61503	
	0.03268	3.83303	4.55537	0.61958	0.77255	0.48576	0.95510														
504	2.62047	4.86359	1.64640	2.23659	4.36101	3.13853	3.72664	3.77494	2.66815	3.40637	4.25443	2.76730	3.74016	2.90431	3.17309	2.41863	2.58471	3.37838	5.62289	4.22285	504 d - - -
	2.68618	4.42225	2.77520	2.73124	3.46354	2.40507	3.72495	3.29354	2.67741	2.69355	4.24690	2.90347	2.73740	3.18147	2.89801	2.37887	2.77520	2.98519	4.58477	3.61503	
	0.08092	2.69977	4.55537	0.48428	0.95747	0.48576	0.95510														
505	2.73184	5.11135	2.18766	2.18296	4.42080	3.23919	3.62956	3.89069	2.09796	3.42897	4.25418	2.03675	3.77132	2.78246	2.91898	2.68448	2.99394	3.50827	5.59555	4.19192	506 n - - -
	2.68618	4.42225	2.77519	2.73123	3.46354	2.40513	3.72494	3.29354	2.67741	2.69355	4.24690	2.90347	2.73739	3.18146	2.89801	2.37887	2.77519	2.98518	4.58477	3.61503	
	0.03268	3.83303	4.55537	0.61958	0.77255	0.48576	0.95510														
506	3.11774	4.55883	4.37251	3.84746	3.03916	4.22177	4.52024	2.14998	3.62540	1.05398	2.54259	4.15657	4.50445	3.91447	3.82537	3.58143	3.36051	2.21949	5.03480	3.84727	507 l - - -
	2.68618	4.42225	2.77519	2.73123	3.46354	2.40513	3.72494	3.29354	2.67741	2.69355	4.24690	2.90347	2.73739	3.18146	2.89801	2.37887	2.77519	2.98518	4.58477	3.61503	
	0.03268	3.83303	4.55537	0.61958	0.77255	0.48576	0.95510														
507	3.27202	4.66041	4.03210	3.72890	2.62865	3.68698	3.85349	3.29453	3.45219	2.69596	3.93010	3.88606	4.20159	3.84191	3.58729	3.47606	3.57058	3.18378	0.96876	2.63291	508 w - - -
	2.68618	4.42225	2.77519	2.73123	3.46354	2.40513	3.72494	3.29354	2.67741	2.69355	4.24690	2.90347	2.73739	3.18146	2.89801	2.37887	2.77519	2.98518	4.58477	3.61503	
	0.03268	3.83303	4.55537	0.61958	0.77255	0.48576	0.95510														
508	2.39269	4.23746	3.39425	3.14521	3.93517	3.07784	4.16539	3.05754	3.08739	2.92240	3.97601	3.36046	3.77873	3.48436	3.34758	2.59359	1.24059	2.75673	5.37648	4.15199	509 t - - -
	2.68618	4.42225	2.77519	2.73123	3.46354	2.40513	3.72494	3.29354	2.67741	2.69355	4.24690	2.90347	2.73739	3.18146	2.89801	2.37887	2.77519	2.98518	4.58477	3.61503	
	0.03268	3.83303	4.55537	0.61958	0.77255	0.48576	0.95510														
509	2.99120	4.45279	4.30366	3.81435	3.18378	4.08481	4.52958	2.03294	3.63175	1.24608	3.07474	4.09270	4.44813	3.93600	3.84782	3.46483	3.26511	1.73037	5.11979	3.89466	510 l - - -
	2.68618	4.42225	2.77519	2.73123	3.46354	2.40513	3.72494	3.29354	2.67741	2.69355	4.24690	2.90347	2.73739	3.18146	2.89801	2.37887	2.77519	2.98518	4.58477	3.61503	
	0.03268	3.83303	4.55537	0.61958	0.77255	0.48576	0.95510														
510	2.85732	5.35578	1.64392	1.55065	4.64227	3.16195	3.69569	4.13637	2.69245	3.67043	4.53110	2.63967	3.77492	2.86575	3.26288	2.75689	3.14446	3.73324	5.83145	4.35698	511 e - - -

```
          2.68618  4.42225  2.77519  2.73123  3.46354  2.40513  3.72494  3.29354  2.67741  2.69355  4.24690  2.90347  2.73739  3.18146  2.89801  2.37887  2.77519  2.98518  4.58477  3.61503
          0.03268  3.83303  4.55537  0.61958  0.77255  0.48576  0.95510
    511   2.94831  5.06869  0.97632  2.31258  4.44069  3.18175  3.89799  4.02012  2.97493  3.63580  4.61368  2.86669  3.83184  3.13755  3.48525  2.91560  3.28628  3.66374  5.61691  4.32095    512 d - - -
          2.68618  4.42225  2.77519  2.73123  3.46354  2.40513  3.72494  3.29354  2.67741  2.69355  4.24690  2.90347  2.73739  3.18146  2.89801  2.37887  2.77519  2.98518  4.58477  3.61503
          0.03268  3.83303  4.55537  0.61958  0.77255  0.48576  0.95510
    512   3.07605  4.45331  4.49535  3.99042  3.21279  4.28500  4.66995  1.36797  3.80844  1.50665  3.08467  4.26780  4.58111  4.09498  4.00979  3.65951  3.32907  1.83421  5.18409  3.96301    513 i - - -
          2.68618  4.42225  2.77519  2.73123  3.46354  2.40513  3.72494  3.29354  2.67741  2.69355  4.24690  2.90347  2.73739  3.18146  2.89801  2.37887  2.77519  2.98518  4.58477  3.61503
          0.03268  3.83303  4.55537  0.61958  0.77255  0.48576  0.95510
    513   2.88537  5.00048  2.89893  2.42454  4.37643  3.42871  3.63363  3.74285  1.31285  3.29948  4.19673  3.00932  3.90110  2.79601  2.35417  2.89015  3.11333  3.43132  5.41727  4.17649    514 k - - -
          2.68618  4.42225  2.77519  2.73123  3.46354  2.40513  3.72494  3.29354  2.67741  2.69355  4.24690  2.90347  2.73739  3.18146  2.89801  2.37887  2.77519  2.98518  4.58477  3.61503
          0.03268  3.83303  4.55537  0.61958  0.77255  0.48576  0.95510
    514   2.96214  4.80276  3.44889  2.96907  4.14736  3.45685  3.79770  3.68689  2.22438  3.21005  4.21188  3.33151  3.96192  3.02053  1.04043  3.04858  3.23116  3.41270  5.24678  4.08393    515 r - - -
          2.68618  4.42225  2.77519  2.73123  3.46354  2.40513  3.72494  3.29354  2.67741  2.69355  4.24690  2.90347  2.73739  3.18146  2.89801  2.37887  2.77519  2.98518  4.58477  3.61503
          0.03268  3.83303  4.55537  0.61958  0.77255  0.48576  0.95510
    515   2.01385  4.29370  3.84822  3.33939  3.42545  3.71024  4.20338  1.88111  3.22334  1.98661  3.32684  3.65979  4.15055  3.55660  3.51320  3.04834  2.98269  2.00713  5.11529  3.88573    516 i - - -
          2.68618  4.42225  2.77519  2.73123  3.46354  2.40513  3.72494  3.29354  2.67741  2.69355  4.24690  2.90347  2.73739  3.18146  2.89801  2.37887  2.77519  2.98518  4.58477  3.61503
          0.03268  3.83303  4.55537  0.61958  0.77255  0.48576  0.95510
    516   2.62317  4.81085  2.57494  2.33855  4.27804  2.34592  3.68721  3.72534  2.24644  3.30801  4.14695  2.17709  3.76361  2.86066  2.88132  2.64408  2.92619  3.34347  5.49389  4.15101    517 n - - -
          2.68618  4.42225  2.77519  2.73123  3.46354  2.40513  3.72494  3.29354  2.67741  2.69355  4.24690  2.90347  2.73739  3.18146  2.89801  2.37887  2.77519  2.98518  4.58477  3.61503
          0.03268  3.83303  4.55537  0.61958  0.77255  0.48576  0.95510
    517   2.58649  1.86576  4.05392  3.58898  2.63270  3.49369  4.12321  2.43090  3.45347  2.23792  3.37822  3.71663  4.03905  3.71685  3.65253  2.91263  2.93215  2.27028  4.75115  3.36959    518 c - - -
          2.68618  4.42225  2.77519  2.73123  3.46354  2.40513  3.72494  3.29354  2.67741  2.69355  4.24690  2.90347  2.73739  3.18146  2.89801  2.37887  2.77519  2.98518  4.58477  3.61503
          0.03268  3.83303  4.55537  0.61958  0.77255  0.48576  0.95510
    518   2.59084  4.23004  3.91003  3.51121  3.61948  3.51913  4.38316  2.12280  3.38997  2.34517  3.51940  3.71710  4.09862  3.74298  3.64338  2.94156  2.56552  1.30673  5.32127  4.08472    519 v - - -
          2.68618  4.42225  2.77519  2.73123  3.46354  2.40513  3.72494  3.29354  2.67741  2.69355  4.24690  2.90347  2.73739  3.18146  2.89801  2.37887  2.77519  2.98518  4.58477  3.61503
          0.03268  3.83303  4.55537  0.61958  0.77255  0.48576  0.95510
    519   3.07422  4.49292  4.34097  3.87343  1.58581  4.10685  4.07178  2.07226  3.74317  1.76636  3.11885  4.03752  4.44102  3.92382  3.91316  3.47085  3.32744  2.29841  4.40239  2.86585    520 f - - -
          2.68618  4.42225  2.77519  2.73123  3.46354  2.40513  3.72494  3.29354  2.67741  2.69355  4.24690  2.90347  2.73739  3.18146  2.89801  2.37887  2.77519  2.98518  4.58477  3.61503
          0.03268  3.83303  4.55537  0.61958  0.77255  0.48576  0.95510
    520   2.41486  4.66827  2.84739  2.44503  4.03017  3.29051  3.42466  3.44380  2.23508  3.05766  3.89660  2.93134  2.56896  2.82721  2.80654  2.44956  2.83496  3.11159  5.29410  3.98596    521 k - - -
          2.68618  4.42225  2.77519  2.73123  3.46354  2.40513  3.72494  3.29354  2.67741  2.69355  4.24690  2.90347  2.73739  3.18146  2.89801  2.37887  2.77519  2.98518  4.58477  3.61503
          0.03268  3.83303  4.55537  0.61958  0.77255  0.48576  0.95510
    521   2.81280  5.21935  1.40982  2.09543  4.53727  3.14050  3.72205  4.09316  2.73672  3.64859  4.51700  2.24873  3.76971  2.90583  3.29723  2.73892  3.12337  3.68200  5.77385  4.29983    522 d - - -
          2.68618  4.42225  2.77519  2.73123  3.46354  2.40513  3.72494  3.29354  2.67741  2.69355  4.24690  2.90347  2.73739  3.18146  2.89801  2.37887  2.77519  2.98518  4.58477  3.61503
          0.03268  3.83303  4.55537  0.61958  0.77255  0.48576  0.95510
    522   2.70125  4.46443  3.30428  3.13815  4.13555  3.15421  4.21835  3.67141  3.19219  3.30231  4.35093  3.44851  0.90433  3.58220  3.44165  2.86766  3.13986  3.32122  5.33234  4.25589    523 p - - -
          2.68618  4.42225  2.77519  2.73123  3.46354  2.40513  3.72494  3.29354  2.67741  2.69355  4.24690  2.90347  2.73739  3.18146  2.89801  2.37887  2.77519  2.98518  4.58477  3.61503
          0.03268  3.83303  4.55537  0.61958  0.77255  0.48576  0.95510
    523   3.05234  4.47968  4.39107  3.84101  3.10920  4.21299  4.54385  2.06576  3.68366  1.19161  2.58465  4.14940  4.48981  3.92953  3.89590  3.54628  3.29196  1.91115  5.08653  3.94249    524 l - - -
          2.68618  4.42225  2.77519  2.73123  3.46354  2.40513  3.72494  3.29354  2.67741  2.69355  4.24690  2.90347  2.73739  3.18146  2.89801  2.37887  2.77519  2.98518  4.58477  3.61503
          0.03268  3.83303  4.55537  0.61958  0.77255  0.48576  0.95510
    524   1.89847  4.59503  2.98084  2.60798  4.09184  3.26601  3.73687  3.37781  1.86956  3.07820  3.97288  3.03842  3.80432  2.92903  2.71506  2.66607  2.88817  3.05660  5.34748  4.07730    525 k - - -
          2.68618  4.42225  2.77519  2.73123  3.46354  2.40513  3.72494  3.29354  2.67741  2.69355  4.24690  2.90347  2.73739  3.18146  2.89801  2.37887  2.77519  2.98518  4.58477  3.61503
          0.03268  3.83303  4.55537  0.61958  0.77255  0.48576  0.95510
    525   2.15820  4.49560  3.09133  2.60246  3.81794  3.05883  3.69698  2.80816  2.13706  2.83876  3.71319  3.05118  3.80401  2.90758  2.85409  2.64262  2.79970  2.83581  5.15552  3.88220    526 k - - -
          2.68618  4.42225  2.77519  2.73123  3.46354  2.40513  3.72494  3.29354  2.67741  2.69355  4.24690  2.90347  2.73739  3.18146  2.89801  2.37887  2.77519  2.98518  4.58477  3.61503
          0.03268  3.83303  4.55537  0.61958  0.77255  0.48576  0.95510
    526   3.15472  4.55840  4.36920  3.90908  1.63239  4.16341  4.03194  2.37729  3.76570  1.47455  3.09170  4.06434  4.47652  3.93613  3.92547  3.53355  3.39939  2.41984  4.32719  2.77107    527 l - - -
          2.68618  4.42225  2.77519  2.73123  3.46354  2.40513  3.72494  3.29354  2.67741  2.69355  4.24690  2.90347  2.73739  3.18146  2.89801  2.37887  2.77519  2.98518  4.58477  3.61503
          0.02212  3.82246        *  0.61958  0.77255  0.00000        *
//
HMMER3/f [3.1b2 | February 2015]
NAME  PmoA
ACC   PF14100.5
DESC  Methane oxygenase PmoA
LENG  271
ALPH  amino
RF    no
MM    no
CONS  yes
CS    no
MAP   yes
DATE  Thu Jan 19 06:32:35 2017
NSEQ  101
EFFN  4.663483
CKSUM 3333536686
GA    27.00 27.00;
```

```
TC    27.70 42.50;
NC    18.60 22.50;
BM    hmmbuild HMM.ann SEED.ann
SM    hmmsearch -Z 26740544 -E 1000 --cpu 4 HMM pfamseq
STATS LOCAL MSV      -11.0667  0.70215
STATS LOCAL VITERBI  -11.8736  0.70215
STATS LOCAL FORWARD   -5.1113  0.70215
HMM          A        C        D        E        F        G        H        I        K        L        M        N        P        Q        R        S        T        V        W        Y
            m->m     m->i     m->d     i->m     i->i     d->m     d->d
  COMPO   2.52515  4.76848  2.92134  2.63783  3.37938  2.73487  3.36412  3.09915  2.72502  2.57730  3.87693  3.08304  3.14670  3.10187  2.81265  2.68435  2.70685  2.71821  3.89961  3.50278
          2.68618  4.42225  2.77519  2.73123  3.46354  2.40513  3.72494  3.29354  2.67741  2.69355  4.24690  2.90347  2.73739  3.18146  2.89801  2.37887  2.77519  2.98518  4.58477  3.61503
          0.27282  5.28723  1.45366  0.61958  0.77255  0.00000        *
      1   2.07421  4.30993  3.02561  2.29320  4.70871  3.36655  3.71951  4.18784  2.05176  3.54979  4.41656  3.13473  4.05670  2.62078  2.33612  2.30323  2.53370  3.75876  5.81183  3.75914      1 k - - -
          2.68618  4.42225  2.77519  2.73123  3.46354  2.40513  3.72494  3.29354  2.67741  2.69355  4.24690  2.90347  2.73739  3.18146  2.89801  2.37887  2.77519  2.98518  4.58477  3.61503
          0.00982  5.02423  5.74657  0.61958  0.77255  0.51518  0.90979
      2   2.59420  4.40308  4.81486  4.20970  3.49819  4.15575  4.48151  1.88165  4.02705  1.82883  3.52113  4.26669  3.80409  3.50361  4.08917  3.14136  2.58966  1.43687  4.42164  3.84358      2 v - - -
          2.68618  4.42225  2.77519  2.73123  3.46354  2.40513  3.72494  3.29354  2.67741  2.69355  4.24690  2.90347  2.73739  3.18146  2.89801  2.37887  2.77519  2.98518  4.58477  3.61503
          0.00836  5.18425  5.90660  0.61958  0.77255  0.51661  0.90768
      3   2.39114  4.86811  2.12932  2.03811  4.82119  3.75879  3.94639  3.96401  2.41476  3.28951  4.52336  2.94119  3.67936  2.84028  2.79685  2.27342  2.59281  2.89175  5.91714  4.50935      3 e - - -
          2.68618  4.42225  2.77519  2.73123  3.46354  2.40513  3.72494  3.29354  2.67741  2.69355  4.24690  2.90347  2.73739  3.18146  2.89801  2.37887  2.77519  2.98518  4.58477  3.61503
          0.00789  5.24176  5.96410  0.61958  0.77255  0.58196  0.81826
      4   2.92376  4.42627  5.00576  4.14645  2.87629  4.22106  4.16748  1.75317  4.17897  1.50282  3.16554  4.38035  4.58384  4.31163  4.19063  3.41663  3.28329  1.53547  5.04973  3.55217      4 l - - -
          2.68618  4.42225  2.77519  2.73123  3.46354  2.40513  3.72494  3.29354  2.67741  2.69355  4.24690  2.90347  2.73739  3.18146  2.89801  2.37887  2.77519  2.98518  4.58477  3.61503
          0.00781  5.25199  5.97433  0.61958  0.77255  0.41623  1.07743
      5   2.45432  5.46088  2.88326  2.38023  4.78314  3.41368  3.58825  4.08600  2.42822  2.35987  3.61149  3.07911  4.18176  2.66804  2.57664  2.48713  2.28219  2.99809  5.90745  3.84116      5 t - - -
          2.68618  4.42225  2.77519  2.73123  3.46354  2.40513  3.72494  3.29354  2.67741  2.69355  4.24690  2.90347  2.73739  3.18146  2.89801  2.37887  2.77519  2.98518  4.58477  3.61503
          0.00754  5.28723  6.00957  0.61958  0.77255  0.48576  0.95510
      6   2.44027  3.78377  3.64863  3.72703  3.21681  4.11388  2.79101  1.97850  3.37710  2.39271  3.56713  3.75850  4.48646  3.36256  3.00321  2.80676  3.11568  1.80881  5.19308  3.29139      6 v - - -
          2.68618  4.42225  2.77519  2.73123  3.46354  2.40513  3.72494  3.29354  2.67741  2.69355  4.24690  2.90347  2.73739  3.18146  2.89801  2.37887  2.77519  2.98518  4.58477  3.61503
          0.00754  5.28723  6.00957  0.61958  0.77255  0.48576  0.95510
      7   2.73677  5.03111  1.63170  2.23565  4.87645  1.85781  3.56889  4.36261  2.59263  3.83762  4.57489  2.72977  4.18449  2.89487  3.05200  2.85621  3.18134  3.92294  5.96609  4.55384      7 d - - -
          2.68618  4.42225  2.77519  2.73123  3.46354  2.40513  3.72494  3.29354  2.67741  2.69355  4.24690  2.90347  2.73739  3.18146  2.89801  2.37887  2.77519  2.98518  4.58477  3.61503
          0.00754  5.28723  6.00957  0.61958  0.77255  0.48576  0.95510
      8   3.30444  5.85169  2.13800  2.61188  5.17610  0.99919  3.79187  4.67536  3.03719  4.14880  4.91402  2.66185  4.37546  3.20359  3.13796  2.61736  3.55190  4.23432  6.28481  4.83945      8 g - - -
          2.68607  4.42226  2.77520  2.73124  3.46355  2.40513  3.72495  3.29355  2.67741  2.69356  4.24690  2.90347  2.73740  3.18147  2.89801  2.37887  2.77520  2.98519  4.58478  3.61504
          0.03478  3.93610  4.22291  0.44930  1.01632  0.48576  0.95510
      9   2.86975  4.87640  3.18382  2.37695  4.79585  3.68094  3.59698  4.01164  1.78593  3.12878  4.51345  3.10277  4.17339  2.37547  2.34309  2.57674  2.42675  2.71752  4.67110  4.51123     10 k - - -
          2.68618  4.42225  2.77519  2.73123  3.46354  2.40513  3.72494  3.29354  2.67741  2.69355  4.24690  2.90347  2.73739  3.18146  2.89801  2.37887  2.77519  2.98518  4.58477  3.61503
          0.00763  5.27495  5.99730  0.61958  0.77255  0.53059  0.88735
     10   2.74577  5.33856  3.03900  2.13368  3.81447  3.80772  3.38664  3.66844  2.66224  2.49616  4.39727  3.03204  1.85869  3.06678  2.96616  2.89129  2.70984  2.91548  5.81303  4.18379     11 p - - -
          2.68618  4.42225  2.77519  2.73123  3.46354  2.40513  3.72494  3.29354  2.67741  2.69355  4.24690  2.90347  2.73739  3.18146  2.89801  2.37887  2.77519  2.98518  4.58477  3.61503
          0.00763  5.27495  5.99730  0.61958  0.77255  0.53059  0.88735
     11   3.18746  4.65023  5.23002  4.62442  1.83097  4.49099  4.82746  2.42712  4.42492  1.36690  3.60078  4.64202  4.82557  4.54473  3.28996  3.80901  3.52426  1.45397  5.28457  4.12461     12 l - - -
          2.68618  4.42225  2.77519  2.73123  3.46354  2.40513  3.72494  3.29354  2.67741  2.69355  4.24690  2.90347  2.73739  3.18146  2.89801  2.37887  2.77519  2.98518  4.58477  3.61503
          0.00763  5.27495  5.99730  0.61958  0.77255  0.53059  0.88735
     12   1.84825  4.22756  4.91071  4.30259  2.56587  3.49844  4.53887  2.87829  4.11054  1.73303  3.21676  4.33766  4.57549  4.26167  4.15631  3.51730  1.71984  2.45676  3.45384  3.88676     13 t - - -
          2.68618  4.42225  2.77519  2.73123  3.46354  2.40513  3.72494  3.29354  2.67741  2.69355  4.24690  2.90347  2.73739  3.18146  2.89801  2.37887  2.77519  2.98518  4.58477  3.61503
          0.00763  5.27495  5.99730  0.61958  0.77255  0.53059  0.88735
     13   2.62282  5.48120  3.16895  2.45635  3.71942  3.49324  3.86357  4.29168  2.30136  3.78128  4.52507  3.25031  4.17126  2.58035  1.92801  2.09755  2.08737  3.30713  5.92112  4.51767     14 r - - -
          2.68618  4.42225  2.77519  2.73123  3.46354  2.40513  3.72494  3.29354  2.67741  2.69355  4.24690  2.90347  2.73739  3.18146  2.89801  2.37887  2.77519  2.98518  4.58477  3.61503
          0.00763  5.27495  5.99730  0.61958  0.77255  0.53059  0.88735
     14   4.32040  5.53889  5.72876  5.38746  2.01947  5.21122  3.70693  4.06785  5.18162  2.24702  4.68602  5.00637  4.42016  5.07865  5.11108  4.56165  4.53848  3.57859  3.80759  0.55808     15 Y - - -
          2.68618  4.42225  2.77519  2.73123  3.46354  2.40513  3.72494  3.29354  2.67741  2.69355  4.24690  2.90347  2.73739  3.18146  2.89801  2.37887  2.77519  2.98518  4.58477  3.61503
          0.00763  5.27495  5.99730  0.61958  0.77255  0.53059  0.88735
     15   2.92904  3.72807  3.66401  3.11364  3.42102  3.88435  2.65158  2.69171  3.26493  2.57020  3.07970  2.50145  4.37991  2.34290  3.12120  3.19111  2.90476  1.98155  5.35000  3.60935     16 v - - -
          2.68618  4.42225  2.77519  2.73123  3.46354  2.40513  3.72494  3.29354  2.67741  2.69355  4.24690  2.90347  2.73739  3.18146  2.89801  2.37887  2.77519  2.98518  4.58477  3.61503
          0.01969  5.27495  4.24169  0.61958  0.77255  0.53059  0.88735
     16   2.88322  4.79298  2.87153  3.25635  2.58817  3.98007  2.99795  3.19991  2.99907  2.54679  3.43624  3.38732  4.12682  3.10113  3.59200  2.93994  2.70732  2.90944  4.19833  1.69182     17 y - - -
          2.68618  4.42225  2.77519  2.73123  3.46354  2.40513  3.72494  3.29354  2.67741  2.69355  4.24690  2.90347  2.73739  3.18146  2.89801  2.37887  2.77519  2.98518  4.58477  3.61503
          0.00772  5.26298  5.98533  0.61958  0.77255  0.50263  0.92871
     17   1.86588  5.49866  2.74206  2.51363  4.83856  2.10372  3.96383  4.32080  2.54450  3.67679  4.54101  3.24514  2.75444  2.99486  2.29360  2.50473  3.06260  3.74732  5.93466  4.52690     18 a - - -
          2.68616  4.42279  2.77521  2.73144  3.46407  2.40566  3.72526  3.29375  2.67794  2.69365  4.24436  2.90392  2.73557  3.18164  2.89854  2.37889  2.77517  2.98448  4.58530  3.61422
          0.84703  1.41681  1.11222  1.36478  0.29496  0.53059  0.88735
     18   2.61656  5.28893  2.61212  2.22360  4.61334  3.31779  3.43085  3.59407  2.44671  3.31641  4.33513  2.97127  2.01217  2.83697  3.03218  2.56809  2.49625  3.46745  5.73417  4.33594     24 p - - -
          2.68618  4.42225  2.77519  2.73123  3.46354  2.40513  3.72494  3.29354  2.67741  2.69355  4.24690  2.90347  2.73739  3.18146  2.89801  2.37887  2.77519  2.98518  4.58477  3.61503
          0.01135  4.87992  5.60226  0.61958  0.77255  1.29993  0.31821
     19   2.69917  5.32265  2.10927  2.32160  4.66111  2.46101  3.57299  4.14247  2.42346  3.52952  4.36506  2.70822  2.84369  2.79235  2.91354  2.51642  2.81061  3.44259  5.11964  3.92694     25 d - - -
```

```
    2.68618  4.42225  2.77519  2.73123  3.46354  2.40513  3.72494  3.29354  2.67741  2.69355  4.24690  2.90347  2.73739  3.18146  2.89801  2.37887  2.77519  2.98518  4.58477  3.61503
    0.02326  4.87992  4.17375  0.61958  0.77255  1.29993  0.31821
20  2.35444  4.74713  2.95494  2.46187  3.91819  3.73895  3.96718  2.82442  2.54236  2.11064  3.24001  3.36683  3.43011  3.20204  3.28861  2.90083  2.61788  2.27800  5.30066  4.02797    26 l - - -
    2.68618  4.42225  2.77519  2.73123  3.46354  2.40513  3.72494  3.29354  2.67741  2.69355  4.24690  2.90347  2.73739  3.18146  2.89801  2.37887  2.77519  2.98518  4.58477  3.61503
    0.04360  4.86814  3.35315  0.61958  0.77255  1.11940  0.39523
21  2.46818  5.32196  2.08832  2.23179  4.28876  3.03923  3.61102  4.13866  2.25348  3.62364  3.95887  3.07906  2.84993  2.81720  2.75749  2.51906  2.95155  3.03856  5.75972  4.35389    27 d - - -
    2.68618  4.42225  2.77519  2.73123  3.46354  2.40513  3.72494  3.29354  2.67741  2.69355  4.24690  2.90347  2.73739  3.18146  2.89801  2.37887  2.77519  2.98518  4.58477  3.61503
    0.01124  4.88978  5.61213  0.61958  0.77255  1.01746  0.44865
22  2.01938  5.34600  2.81300  2.37578  4.68028  3.02549  3.82743  4.15867  2.56665  3.29665  4.38972  2.92324  2.37901  2.58368  2.70978  2.55345  2.52291  3.64780  5.78536  4.38100    28 a - - -
    2.68618  4.42225  2.77519  2.73123  3.46354  2.40513  3.72494  3.29354  2.67741  2.69355  4.24690  2.90347  2.73739  3.18146  2.89801  2.37887  2.77519  2.98518  4.58477  3.61503
    0.01044  4.96300  5.68535  0.61958  0.77255  0.31777  1.30112
23  2.08407  5.26006  2.54590  2.49248  3.18873  3.50798  3.63526  3.38176  2.59524  3.12356  4.09998  3.24046  3.59987  2.87057  2.99579  2.60774  2.37866  2.99320  3.92892  3.40447    29 a - - -
    2.68618  4.42225  2.77519  2.73123  3.46354  2.40513  3.72494  3.29354  2.67741  2.69355  4.24690  2.90347  2.73739  3.18146  2.89801  2.37887  2.77519  2.98518  4.58477  3.61503
    0.00790  5.24073  5.96307  0.61958  0.77255  0.46835  0.98360
24  2.63099  5.46177  2.48732  1.87693  3.56436  3.61597  2.94960  4.25953  2.59516  2.96146  4.50764  2.96662  4.17343  2.77382  3.20176  2.41758  2.97565  3.28483  5.90640  2.54917    30 e - - -
    2.68618  4.42225  2.77519  2.73123  3.46354  2.40513  3.72494  3.29354  2.67741  2.69355  4.24690  2.90347  2.73739  3.18146  2.89801  2.37887  2.77519  2.98518  4.58477  3.61503
    0.00765  5.27301  5.99536  0.61958  0.77255  0.45475  1.00678
25  2.14055  5.45861  3.08606  2.37080  4.77996  3.08038  3.97900  3.55771  2.35351  2.32432  4.50557  3.10045  3.59103  2.86325  2.92011  2.00687  2.70117  3.57237  5.90576  4.03836    31 s - - -
    2.68618  4.42225  2.77519  2.73123  3.46354  2.40513  3.72494  3.29354  2.67741  2.69355  4.24690  2.90347  2.73739  3.18146  2.89801  2.37887  2.77519  2.98518  4.58477  3.61503
    0.00754  5.28723  6.00957  0.61958  0.77255  0.48576  0.95510
26  2.70332  5.39238  3.16684  2.70105  3.64527  2.88451  3.99649  4.14076  2.12174  3.67634  4.02401  3.15321  1.89141  3.11159  1.96941  2.99726  3.15196  3.75628  5.10126  3.21654    32 p - - -
    2.68618  4.42225  2.77519  2.73123  3.46354  2.40513  3.72494  3.29354  2.67741  2.69355  4.24690  2.90347  2.73739  3.18146  2.89801  2.37887  2.77519  2.98518  4.58477  3.61503
    0.00754  5.28723  6.00957  0.61958  0.77255  0.48576  0.95510
27  2.79473  4.46496  3.18265  2.74467  4.79486  3.62846  3.99137  4.26094  1.40259  3.76439  4.51913  3.28578  4.19897  3.09680  1.74374  2.17398  3.13603  3.85017  5.91434  3.88585    33 k - - -
    2.68618  4.42225  2.77519  2.73123  3.46354  2.40513  3.72494  3.29354  2.67741  2.69355  4.24690  2.90347  2.73739  3.18146  2.89801  2.37887  2.77519  2.98518  4.58477  3.61503
    0.00754  5.28723  6.00957  0.61958  0.77255  0.48576  0.95510
28  2.67346  5.25205  3.73260  3.40630  5.09694  2.09127  4.66614  4.54015  3.54486  4.14703  4.95232  2.89297  0.78022  3.47780  3.97248  3.27102  3.56557  3.52557  6.36114  5.05926    34 p - - -
    2.68618  4.42225  2.77519  2.73123  3.46354  2.40513  3.72494  3.29354  2.67741  2.69355  4.24690  2.90347  2.73739  3.18146  2.89801  2.37887  2.77519  2.98518  4.58477  3.61503
    0.00754  5.28723  6.00957  0.61958  0.77255  0.48576  0.95510
29  3.00471  4.29590  4.85448  4.25121  1.89749  3.89130  3.66121  2.83212  4.07215  2.65836  3.42679  4.31600  4.57869  4.23518  4.00146  3.37607  3.13016  2.11052  5.07539  1.27823    35 y - - -
    2.68618  4.42225  2.77519  2.73123  3.46354  2.40513  3.72494  3.29354  2.67741  2.69355  4.24690  2.90347  2.73739  3.18146  2.89801  2.37887  2.77519  2.98518  4.58477  3.61503
    0.00754  5.28723  6.00957  0.61958  0.77255  0.48576  0.95510
30  2.85182  4.52518  5.08416  4.47306  2.51694  4.32536  3.56416  1.75404  4.26706  1.04240  3.56642  4.47646  4.67840  4.39443  4.28384  3.63762  3.38374  2.43724  5.13235  3.76092    36 l - - -
    2.68618  4.42225  2.77519  2.73123  3.46354  2.40513  3.72494  3.29354  2.67741  2.69355  4.24690  2.90347  2.73739  3.18146  2.89801  2.37887  2.77519  2.98518  4.58477  3.61503
    0.00754  5.28723  6.00957  0.61958  0.77255  0.48576  0.95510
31  3.46767  5.17656  3.90615  4.04489  2.34433  4.55172  0.85119  3.69541  3.97376  3.31343  4.30121  4.29012  4.91082  3.75624  4.24509  3.44624  3.83928  3.49946  3.43438  2.08768    37 h - - -
    2.68618  4.42225  2.77519  2.73123  3.46354  2.40513  3.72494  3.29354  2.67741  2.69355  4.24690  2.90347  2.73739  3.18146  2.89801  2.37887  2.77519  2.98518  4.58477  3.61503
    0.03960  5.28723  3.38818  0.61958  0.77255  0.48576  0.95510
32  3.68513  5.51603  5.13628  5.10595  5.60969  4.28558  5.95861  5.09492  5.11422  4.77610  5.85521  4.98452  0.18215  5.42940  5.21605  3.90102  4.24331  3.61488  6.95149  5.83942    38 P - - -
    2.68618  4.42225  2.77519  2.73123  3.46354  2.40513  3.72494  3.29354  2.67741  2.69355  4.24690  2.90347  2.73739  3.18146  2.89801  2.37887  2.77519  2.98518  4.58477  3.61503
    0.00778  5.25541  5.97776  0.61958  0.77255  0.42199  1.06636
33  3.62185  5.17203  5.96122  5.40312  3.49468  5.32218  5.74705  1.67467  5.26138  1.00374  3.33592  5.49120  5.52693  5.31362  5.27477  4.69206  4.15542  1.29245  5.99871  4.91094    39 l - - -
    2.68618  4.42225  2.77519  2.73123  3.46354  2.40513  3.72494  3.29354  2.67741  2.69355  4.24690  2.90347  2.73739  3.18146  2.89801  2.37887  2.77519  2.98518  4.58477  3.61503
    0.00754  5.28723  6.00957  0.61958  0.77255  0.48576  0.95510
34  2.74584  4.69309  4.03971  3.46899  2.69353  3.64121  3.39536  2.32527  3.14193  2.54952  3.79983  2.80065  4.42777  3.69775  1.73387  3.17388  2.87094  2.90263  3.50938  2.96262    40 r - - -
    2.68618  4.42225  2.77519  2.73123  3.46354  2.40513  3.72494  3.29354  2.67741  2.69355  4.24690  2.90347  2.73739  3.18146  2.89801  2.37887  2.77519  2.98518  4.58477  3.61503
    0.00754  5.28723  6.00957  0.61958  0.77255  0.48576  0.95510
35  2.13905  5.31439  2.53296  3.08712  5.00413  2.49494  4.34310  4.46961  3.14380  4.01555  4.78199  3.56293  3.75501  3.47596  3.61434  2.14913  1.15722  3.99220  6.18634  4.83057    41 t - - -
    2.68618  4.42225  2.77519  2.73123  3.46354  2.40513  3.72494  3.29354  2.67741  2.69355  4.24690  2.90347  2.73739  3.18146  2.89801  2.37887  2.77519  2.98518  4.58477  3.61503
    0.00754  5.28723  6.00957  0.61958  0.77255  0.48576  0.95510
36  2.05558  4.74052  3.94871  3.12191  3.76297  3.62650  4.28385  3.07813  3.33167  1.65633  3.63194  3.77715  1.74092  3.62992  3.47341  2.98748  2.80751  3.01301  5.32615  4.09544    42 l - - -
    2.68618  4.42225  2.77519  2.73123  3.46354  2.40513  3.72494  3.29354  2.67741  2.69355  4.24690  2.90347  2.73739  3.18146  2.89801  2.37887  2.77519  2.98518  4.58477  3.61503
    0.00754  5.28723  6.00957  0.61958  0.77255  0.48576  0.95510
37  1.84542  5.51890  2.58998  2.41492  4.86300  2.48028  3.80984  4.34865  2.42032  3.82380  4.56006  2.54622  4.17555  2.81965  3.19925  2.07559  2.64780  3.80781  5.95205  4.54105    43 a - - -
    2.68618  4.42225  2.77519  2.73123  3.46354  2.40513  3.72494  3.29354  2.67741  2.69355  4.24690  2.90347  2.73739  3.18146  2.89801  2.37887  2.77519  2.98518  4.58477  3.61503
    0.00754  5.28723  6.00957  0.61958  0.77255  0.48576  0.95510
38  3.92918  6.49039  2.84719  2.86964  5.82204  0.41617  4.17052  5.34818  3.38055  4.82687  5.72233  3.24551  4.75552  3.69202  4.25975  3.79285  4.23740  4.91614  6.98222  5.46792    44 G - - -
    2.68618  4.42225  2.77519  2.73123  3.46354  2.40513  3.72494  3.29354  2.67741  2.69355  4.24690  2.90347  2.73739  3.18146  2.89801  2.37887  2.77519  2.98518  4.58477  3.61503
    0.00754  5.28723  6.00957  0.61958  0.77255  0.48576  0.95510
39  2.29421  5.49444  2.37789  2.25431  4.29041  3.78231  3.88708  3.79965  2.39562  3.15388  4.53745  2.73152  4.17557  2.69267  2.63049  2.91022  2.00074  2.99360  5.93250  4.52700    45 t - - -
    2.68620  4.42227  2.77522  2.73125  3.46356  2.40508  3.72497  3.29356  2.67730  2.69357  4.24692  2.90349  2.73742  3.18149  2.89803  2.37889  2.77522  2.98504  4.58479  3.61505
    0.05237  3.02472  6.00957  0.29080  1.37701  0.48576  0.95510
40  2.54715  4.80415  3.44667  2.77353  3.66102  3.99360  3.82977  2.45431  3.23334  2.32901  3.35971  3.69781  2.66651  3.37328  3.11249  2.54157  2.48792  1.96327  5.37928  4.13561    47 v - - -
    2.68618  4.42225  2.77519  2.73123  3.46354  2.40513  3.72494  3.29354  2.67741  2.69355  4.24690  2.90347  2.73739  3.18146  2.89801  2.37887  2.77519  2.98518  4.58477  3.61503
    0.00754  5.28723  6.00957  0.61958  0.77255  0.48576  0.95510
41  3.81732  5.08319  5.84958  5.28515  3.21883  5.18465  5.60152  1.74680  5.13225  1.33698  3.15252  5.35264  5.42237  5.20902  5.14978  4.54437  3.76223  1.01144  5.90719  4.79621    48 v - - -
    2.68618  4.42225  2.77519  2.73123  3.46354  2.40513  3.72494  3.29354  2.67741  2.69355  4.24690  2.90347  2.73739  3.18146  2.89801  2.37887  2.77519  2.98518  4.58477  3.61503
```

```
      0.00754  5.28723  6.00957  0.61958  0.77255  0.48576  0.95510
 42   3.12926  4.95977  4.94874  4.59544  4.93188  3.91459  5.34384  3.52681  4.48046  3.96702  4.88994  4.48789  4.67493  4.74111  4.67221  2.56447  0.40114  3.19998  6.40222  5.23239     49 T - - -
      2.68618  4.42225  2.77519  2.73123  3.46354  2.40513  3.72494  3.29354  2.67741  2.69355  4.24690  2.90347  2.73739  3.18146  2.89801  2.37887  2.77519  2.98518  4.58477  3.61503
      0.00754  5.28723  6.00957  0.61958  0.77255  0.48576  0.95510
 43   2.54751  5.50863  2.33015  1.95107  4.85074  3.03646  3.96724  4.13160  2.40631  3.54731  4.55009  3.01603  4.17331  3.06455  1.63139  2.79948  3.14310  3.61182  4.65817  4.53395     50 r - - -
      2.68618  4.42225  2.77519  2.73123  3.46354  2.40513  3.72494  3.29354  2.67741  2.69355  4.24690  2.90347  2.73739  3.18146  2.89801  2.37887  2.77519  2.98518  4.58477  3.61503
      0.02738  5.28723  3.81903  0.61958  0.77255  0.48576  0.95510
 44   2.25650  4.10130  2.83098  3.12479  3.33242  2.01486  3.59632  2.60540  3.38613  2.31864  3.37019  3.81762  3.93908  3.67493  3.58935  2.88060  3.21487  2.52466  5.27996  2.76456     51 g - - -
      2.68656  4.42292  2.77536  2.73120  3.46313  2.40538  3.72561  3.29343  2.67775  2.69373  4.24402  2.90390  2.73531  3.18181  2.89853  2.37939  2.77561  2.98523  4.58208  3.61350
      0.39818  1.28230  2.97469  1.45974  0.26435  0.55646  0.85152
 45   2.68412  5.28990  3.31421  2.56268  3.03355  3.07976  3.07971  3.64425  2.54104  2.96804  3.41798  3.19624  4.17626  2.56551  1.68652  2.63701  3.09974  3.47070  5.76912  4.20950     61 r - - -
      2.68618  4.42225  2.77519  2.73123  3.46354  2.40513  3.72494  3.29354  2.67741  2.69355  4.24690  2.90347  2.73739  3.18146  2.89801  2.37887  2.77519  2.98518  4.58477  3.61503
      0.01561  5.21776  4.59834  0.61958  0.77255  0.45537  1.00571
 46   2.57217  5.45236  3.15905  2.67250  4.76198  3.47285  3.80238  4.19913  2.86936  3.77189  4.57740  3.40581  0.86875  3.32497  3.47627  3.04385  3.42003  3.55405  5.98656  4.62178     62 p - - -
      2.68617  4.42230  2.77525  2.73118  3.46359  2.40489  3.72500  3.29359  2.67733  2.69360  4.24695  2.90352  2.73745  3.18151  2.89806  2.37892  2.77514  2.98523  4.58482  3.61508
      0.14442  2.27551  3.45042  0.17941  1.80646  0.43079  1.04980
 47   2.05419  5.46937  2.73244  2.29589  4.80083  2.07550  2.90313  4.27732  2.48091  3.76884  3.88320  3.00206  3.18218  3.05987  3.18964  2.61735  2.93078  3.38343  4.09641  4.14117     64 a - - -
      2.68618  4.42225  2.77519  2.73123  3.46354  2.40513  3.72494  3.29354  2.67741  2.69355  4.24690  2.90347  2.73739  3.18146  2.89801  2.37887  2.77519  2.98518  4.58477  3.61503
      0.00776  5.25838  5.98072  0.61958  0.77255  0.42713  1.05664
 48   3.03676  5.54713  1.20915  1.86150  4.88885  3.28890  3.52922  4.37486  2.65417  3.54279  4.58991  3.15619  3.87395  2.92858  3.00957  2.99698  3.07442  3.78021  5.98039  4.56742     65 d - - -
      2.68637  4.42272  2.77361  2.73153  3.46370  2.40518  3.72469  3.29376  2.67757  2.69402  4.24736  2.90361  2.73723  3.18183  2.89714  2.37925  2.77500  2.98504  4.58524  3.61550
      0.35739  1.21050  6.00957  1.03565  0.43850  0.48576  0.95510
 49   3.61189  5.39601  3.82877  3.36431  2.87983  4.41656  0.61279  3.97222  3.59091  3.54277  4.51442  4.02569  4.81130  3.92619  3.68046  3.71551  3.84082  3.74666  4.98194  2.67942     69 H - - -
      2.68618  4.42225  2.77519  2.73123  3.46354  2.40513  3.72494  3.29354  2.67741  2.69355  4.24690  2.90347  2.73739  3.18146  2.89801  2.37887  2.77519  2.98518  4.58477  3.61503
      0.00754  5.28723  6.00957  0.61958  0.77255  0.48576  0.95510
 50   2.74213  4.88436  3.46588  3.06293  3.61361  3.95787  3.89839  3.19699  2.99534  2.62133  3.38279  3.31032  1.78084  3.33937  2.45318  2.92031  2.72465  2.39870  4.80529  3.19122     70 p - - -
      2.68618  4.42225  2.77519  2.73123  3.46354  2.40513  3.72494  3.29354  2.67741  2.69355  4.24690  2.90347  2.73739  3.18146  2.89801  2.37887  2.77519  2.98518  4.58477  3.61503
      0.00754  5.28723  6.00957  0.61958  0.77255  0.48576  0.95510
 51   4.16387  6.04851  5.24825  4.98522  3.14223  5.16232  0.54984  4.85098  4.46056  4.12076  5.36256  4.94048  5.60443  4.91072  4.57384  4.67846  4.92127  4.70051  1.59444  3.06765     71 H - - -
      2.68618  4.42225  2.77519  2.73123  3.46354  2.40513  3.72494  3.29354  2.67741  2.69355  4.24690  2.90347  2.73739  3.18146  2.89801  2.37887  2.77519  2.98518  4.58477  3.61503
      0.00754  5.28723  6.00957  0.61958  0.77255  0.48576  0.95510
 52   4.27541  6.29883  3.90996  3.74703  4.78469  4.56830  0.40457  5.33030  3.37087  4.67349  5.67790  4.19757  5.12275  2.20549  3.59340  4.23425  4.53135  5.00123  6.13098  4.62422     72 H - - -
      2.68618  4.42225  2.77519  2.73123  3.46354  2.40513  3.72494  3.29354  2.67741  2.69355  4.24690  2.90347  2.73739  3.18146  2.89801  2.37887  2.77519  2.98518  4.58477  3.61503
      0.00754  5.28723  6.00957  0.61958  0.77255  0.48576  0.95510
 53   2.84024  4.86568  3.74858  3.18476  3.25578  3.96595  2.91387  3.23491  2.23418  2.17269  3.63568  3.15080  4.34855  3.26668  2.03097  2.97367  3.10760  2.42676  4.81550  2.93442     73 r - - -
      2.68618  4.42225  2.77519  2.73123  3.46354  2.40513  3.72494  3.29354  2.67741  2.69355  4.24690  2.90347  2.73739  3.18146  2.89801  2.37887  2.77519  2.98518  4.58477  3.61503
      0.00754  5.28723  6.00957  0.61958  0.77255  0.48576  0.95510
 54   2.34178  5.03644  5.01492  4.94578  5.85061  0.41861  5.81411  5.36149  5.07746  5.03666  5.77495  4.59158  3.90947  5.18819  5.19143  2.13557  3.68968  4.44098  7.17631  6.11853     74 G - - -
      2.68618  4.42225  2.77519  2.73123  3.46354  2.40513  3.72494  3.29354  2.67741  2.69355  4.24690  2.90347  2.73739  3.18146  2.89801  2.37887  2.77519  2.98518  4.58477  3.61503
      0.00754  5.28723  6.00957  0.61958  0.77255  0.48576  0.95510
 55   2.45442  4.80433  5.45736  4.85763  3.02762  4.71136  5.06297  1.68455  4.66409  1.16998  2.60107  4.87569  5.01832  4.75918  4.66879  4.03920  3.70470  1.84007  5.46986  4.33161     75 l - - -
      2.68618  4.42225  2.77519  2.73123  3.46354  2.40513  3.72494  3.29354  2.67741  2.69355  4.24690  2.90347  2.73739  3.18146  2.89801  2.37887  2.77519  2.98518  4.58477  3.61503
      0.00754  5.28723  6.00957  0.61958  0.77255  0.48576  0.95510
 56   2.70402  4.39113  4.38873  3.80713  2.31754  3.07501  4.42395  3.02297  3.70618  2.76479  3.18457  4.06302  4.50444  2.90949  3.92721  2.33403  3.28523  2.82415  1.54029  3.18761     76 w - - -
      2.68618  4.42225  2.77519  2.73123  3.46354  2.40513  3.72494  3.29354  2.67741  2.69355  4.24690  2.90347  2.73739  3.18146  2.89801  2.37887  2.77519  2.98518  4.58477  3.61503
      0.00754  5.28723  6.00957  0.61958  0.77255  0.48576  0.95510
 57   3.06117  4.43866  5.00678  4.39275  1.60337  3.63106  4.56067  2.29967  4.18159  1.96631  2.63224  3.24941  4.59234  4.31611  4.19608  3.34372  3.29334  2.15242  2.77387  3.56523     77 f - - -
      2.68618  4.42225  2.77519  2.73123  3.46354  2.40513  3.72494  3.29354  2.67741  2.69355  4.24690  2.90347  2.73739  3.18146  2.89801  2.37887  2.77519  2.98518  4.58477  3.61503
      0.00754  5.28723  6.00957  0.61958  0.77255  0.48576  0.95510
 58   1.32703  5.21746  3.75200  3.24484  5.03453  2.59058  4.45056  4.49683  3.25571  4.05896  4.82510  2.35722  2.80796  3.59249  3.70780  1.97511  2.07888  3.99206  6.23463  4.90480     78 a - - -
      2.68618  4.42225  2.77519  2.73123  3.46354  2.40513  3.72494  3.29354  2.67741  2.69355  4.24690  2.90347  2.73739  3.18146  2.89801  2.37887  2.77519  2.98518  4.58477  3.61503
      0.00754  5.28723  6.00957  0.61958  0.77255  0.48576  0.95510
 59   2.64470  3.77635  5.00399  4.39021  2.80561  4.22826  2.63427  2.46719  4.17967  2.13657  3.42541  4.38369  4.59212  4.31468  4.19513  3.38391  3.29346  2.10906  1.89957  2.33984     79 w - - -
      2.68618  4.42225  2.77519  2.73123  3.46354  2.40513  3.72494  3.29354  2.67741  2.69355  4.24690  2.90347  2.73739  3.18146  2.89801  2.37887  2.77519  2.98518  4.58477  3.61503
      0.00754  5.28723  6.00957  0.61958  0.77255  0.48576  0.95510
 60   2.83920  5.51258  2.71012  2.35318  4.85643  2.52876  3.06496  4.34156  2.40074  3.81734  4.06744  2.91448  2.58047  2.61386  3.19394  2.25910  2.43751  3.00495  5.94598  4.53574     80 s - - -
      2.68618  4.42225  2.77519  2.73123  3.46354  2.40513  3.72494  3.29354  2.67741  2.69355  4.24690  2.90347  2.73739  3.18146  2.89801  2.37887  2.77519  2.98518  4.58477  3.61503
      0.00754  5.28723  6.00957  0.61958  0.77255  0.48576  0.95510
 61   2.95401  5.51396  1.82810  2.42432  4.22556  3.77868  2.54662  4.02945  2.23165  3.81899  4.55478  2.21349  4.17214  2.76820  2.59456  2.61915  3.23586  3.64204  5.30663  4.15890     81 d - - -
      2.68618  4.42225  2.77519  2.73123  3.46354  2.40513  3.72494  3.29354  2.67741  2.69355  4.24690  2.90347  2.73739  3.18146  2.89801  2.37887  2.77519  2.98518  4.58477  3.61503
      0.00754  5.28723  6.00957  0.61958  0.77255  0.48576  0.95510
 62   2.94466  4.89440  5.61523  5.06657  3.97528  4.98037  5.45325  1.77040  4.92466  2.23826  4.04965  5.13069  5.28895  5.11031  5.00246  4.33454  2.40522  0.77722  5.91160  4.71544     82 v - - -
      2.68618  4.42225  2.77519  2.73123  3.46354  2.40513  3.72494  3.29354  2.67741  2.69355  4.24690  2.90347  2.73739  3.18146  2.89801  2.37887  2.77519  2.98518  4.58477  3.61503
      0.00754  5.28723  6.00957  0.61958  0.77255  0.48576  0.95510
 63   2.76206  5.50797  2.83826  2.36036  4.84962  2.80963  3.96771  4.33321  2.57200  3.66676  4.54956  1.54186  4.17364  2.72464  3.02161  2.14382  2.94768  3.70750  3.98254  4.53376     83 n - - -
      2.68666  4.42279  2.77481  2.73059  3.46273  2.40440  3.72512  3.29365  2.67730  2.69387  4.24744  2.90319  2.73794  3.18201  2.89767  2.37936  2.77560  2.98501  4.58531  3.61479
      0.30304  1.35103  6.00957  1.30976  0.31455  0.48576  0.95510
```

64	2.80235	5.51270	3.04697	2.27195	4.85478	1.34051	3.42992	4.33853	2.24647	3.81651	4.55441	3.25064	3.88426	2.89163	2.81164	2.76363	2.87349	3.74395	5.94691	4.53786	88 g - - -
	2.68618	4.42228	2.77519	2.73123	3.46354	2.40513	3.72494	3.29354	2.67741	2.69355	4.24690	2.90347	2.73739	3.18146	2.89801	2.37887	2.77519	2.98518	4.58477	3.61503	
	0.00754	5.28723	6.00957	0.61958	0.77255	0.48576	0.95510														
65	2.83346	4.70720	3.73071	3.32740	2.97739	3.57344	3.03456	2.35492	3.00141	2.31137	3.81317	3.51945	4.06657	2.88864	3.42014	2.83314	2.52277	1.97991	4.53132	2.87577	89 v - - -
	2.68608	4.42228	2.77522	2.73126	3.46356	2.40495	3.72497	3.29356	2.67743	2.69357	4.24692	2.90349	2.73742	3.18149	2.89803	2.37889	2.77522	2.98521	4.58479	3.61506	
	0.05733	2.93244	6.00957	0.27517	1.42479	0.48576	0.95510														
66	3.43201	5.98267	1.08001	2.82401	5.31599	3.93267	4.32598	4.82206	2.92397	3.84006	5.08165	1.46659	4.46132	3.45662	3.74271	2.41344	3.47430	4.37842	6.44032	4.97822	91 d - - -
	2.68618	4.42225	2.77519	2.73123	3.46354	2.40513	3.72494	3.29354	2.67741	2.69355	4.24690	2.90347	2.73739	3.18146	2.89801	2.37887	2.77519	2.98518	4.58477	3.61503	
	0.00754	5.28723	6.00957	0.61958	0.77255	0.48576	0.95510														
67	3.09039	4.03204	4.76406	4.16994	0.99652	4.22093	3.79717	2.95409	4.00871	2.22707	3.63040	4.27901	3.53469	4.19086	3.66589	3.51987	2.96771	2.76534	5.06827	2.69016	92 f - - -
	2.68618	4.42225	2.77519	2.73123	3.46354	2.40513	3.72494	3.29354	2.67741	2.69355	4.24690	2.90347	2.73739	3.18146	2.89801	2.37887	2.77519	2.98518	4.58477	3.61503	
	0.00754	5.28723	6.00957	0.61958	0.77255	0.48576	0.95510														
68	5.06051	6.19073	5.96221	5.81049	2.86540	5.49710	4.65084	4.82433	5.35855	3.97774	5.34989	5.35738	5.89458	5.44708	4.50000	5.09084	5.33196	4.79430	0.23743	3.02811	93 W - - -
	2.68618	4.42225	2.77519	2.73123	3.46354	2.40513	3.72494	3.29354	2.67741	2.69355	4.24690	2.90347	2.73739	3.18146	2.89801	2.37887	2.77519	2.98518	4.58477	3.61503	
	0.00754	5.28723	6.00957	0.61958	0.77255	0.48576	0.95510														
69	2.64374	5.44913	3.28338	2.31208	4.76594	1.83452	2.86884	3.75212	2.73047	3.34709	4.03236	1.76683	4.18610	2.84285	3.21780	2.77504	3.24007	3.82455	5.89917	4.50602	94 n - - -
	2.68618	4.42225	2.77519	2.73123	3.46354	2.40513	3.72494	3.29354	2.67741	2.69355	4.24690	2.90347	2.73739	3.18146	2.89801	2.37887	2.77519	2.98518	4.58477	3.61503	
	0.01417	5.28723	4.70878	0.61958	0.77255	0.48576	0.95510														
70	2.78090	3.91704	2.91887	2.11054	4.73712	1.87882	3.70425	3.49547	2.73313	2.77924	3.95059	2.16696	4.18458	2.80075	3.21933	2.60528	3.23521	3.79873	5.88124	4.49241	95 g - - -
	2.68618	4.42225	2.77519	2.73123	3.46354	2.40513	3.72494	3.29354	2.67741	2.69355	4.24690	2.90347	2.73739	3.18146	2.89801	2.37887	2.77519	2.98518	4.58477	3.61503	
	0.00759	5.28065	6.00299	0.61958	0.77255	0.47083	0.97946														
71	2.78012	5.51703	2.66650	2.59577	4.86275	2.75082	3.09379	4.34918	2.45699	3.82258	4.55753	2.57858	3.06866	2.71709	1.98090	2.08452	2.98588	3.90802	5.94930	4.01529	96 r - - -
	2.68671	4.42383	2.77517	2.73109	3.46330	2.40400	3.72531	3.29363	2.67845	2.69442	4.24847	2.90443	2.73838	3.18192	2.89708	2.37990	2.77336	2.98393	4.58425	3.61141	
	1.89568	0.63051	1.14740	1.66073	0.21072	0.48576	0.95510														
72	2.69100	5.10592	3.13394	2.53576	4.36163	2.90621	3.86391	3.78870	2.41403	3.37226	4.17216	3.17875	2.27198	2.91621	3.01760	2.58650	3.07760	2.94272	5.59659	2.05921	111 y - - -
	2.68618	4.42225	2.77519	2.73123	3.46354	2.40513	3.72494	3.29354	2.67741	2.69355	4.24690	2.90347	2.73739	3.18146	2.89801	2.37887	2.77519	2.98518	4.58477	3.61503	
	0.15084	4.90888	2.02019	0.61958	0.77255	1.26683	0.33090														
73	2.32871	5.18646	2.85555	2.39431	4.48772	3.24209	3.58328	3.31850	2.35094	3.16153	4.23890	2.91927	3.25323	2.74186	2.58569	2.65851	2.31594	2.76310	5.64628	4.26343	112 t - - -
	2.68618	4.42225	2.77519	2.73123	3.46354	2.40513	3.72494	3.29354	2.67741	2.69355	4.24690	2.90347	2.73739	3.18146	2.89801	2.37887	2.77519	2.98518	4.58477	3.61503	
	0.01267	4.77071	5.49306	0.61958	0.77255	1.36914	0.29347														
74	2.33921	5.24019	2.74360	2.16948	4.56030	3.42491	3.45898	4.02904	2.28189	2.81401	4.08074	2.83862	3.50080	2.63423	2.86489	2.55623	2.76589	3.61735	3.15071	3.86923	113 e - - -
	2.68618	4.42225	2.77519	2.73123	3.46354	2.40513	3.72494	3.29354	2.67741	2.69355	4.24690	2.90347	2.73739	3.18146	2.89801	2.37887	2.77519	2.98518	4.58477	3.61503	
	0.01253	4.78165	5.50400	0.61958	0.77255	1.33996	0.30362														
75	2.72963	5.12768	3.04885	2.53534	4.40462	3.60718	3.28782	3.84404	2.22508	1.87851	4.18731	2.90474	3.15061	2.80989	2.45199	2.75193	2.83167	3.14026	5.60268	4.23904	114 l - - -
	2.68618	4.42225	2.77519	2.73123	3.46354	2.40513	3.72494	3.29354	2.67741	2.69355	4.24690	2.90347	2.73739	3.18146	2.89801	2.37887	2.77519	2.98518	4.58477	3.61503	
	0.01233	4.79790	5.52025	0.61958	0.77255	1.20291	0.35713														
76	2.40478	5.32772	2.41824	2.32337	4.67121	2.77177	3.59436	4.15633	1.91681	3.63227	4.36906	2.78309	2.74621	2.87756	2.70228	2.67564	2.84655	3.71756	5.76114	4.35065	115 k - - -
	2.68618	4.42225	2.77519	2.73123	3.46354	2.40513	3.72494	3.29354	2.67741	2.69355	4.24690	2.90347	2.73739	3.18146	2.89801	2.37887	2.77519	2.98518	4.58477	3.61503	
	0.01170	4.84943	5.57177	0.61958	0.77255	0.47207	0.97739														
77	2.84957	5.45238	2.19687	2.21063	4.79774	3.42569	3.46221	4.28405	1.90705	3.25127	4.49305	2.08917	3.20147	2.99729	2.94555	2.53270	2.98252	3.84319	5.88479	4.47345	116 k - - -
	2.68618	4.42225	2.77519	2.73123	3.46354	2.40513	3.72494	3.29354	2.67741	2.69355	4.24690	2.90347	2.73739	3.18146	2.89801	2.37887	2.77519	2.98518	4.58477	3.61503	
	0.00868	5.14664	5.86898	0.61958	0.77255	0.79157	0.60355														
78	2.68181	5.45986	2.83771	2.20960	4.80561	2.86871	2.18993	4.29204	2.02501	3.48582	4.50036	2.58947	3.82437	2.73379	2.52163	2.65374	3.11529	3.74286	5.89209	4.48075	117 k - - -
	2.68618	4.42225	2.77519	2.73123	3.46354	2.40513	3.72494	3.29354	2.67741	2.69355	4.24690	2.90347	2.73739	3.18146	2.89801	2.37887	2.77519	2.98518	4.58477	3.61503	
	0.30556	5.16522	1.35643	0.61958	0.77255	0.60228	0.79310														
79	2.85192	5.20195	2.92722	2.40041	3.67905	3.11075	3.68103	3.62960	2.20436	3.35212	3.34342	3.14946	3.70068	2.33191	2.93671	2.51195	2.76930	3.03223	5.67302	2.56192	118 k - - -
	2.68618	4.42225	2.77519	2.73123	3.46354	2.40513	3.72494	3.29354	2.67741	2.69355	4.24690	2.90347	2.73739	3.18146	2.89801	2.37887	2.77519	2.98518	4.58477	3.61503	
	0.01072	4.93694	5.65929	0.61958	0.77255	0.30474	1.33681														
80	3.00311	5.37262	3.60210	3.20262	5.43842	0.38781	4.98330	4.20774	3.97892	4.52548	5.37149	3.52270	4.65034	4.22210	4.37025	3.45322	3.80588	4.32963	6.73140	5.43605	119 G - - -
	2.68618	4.42225	2.77519	2.73123	3.46354	2.40513	3.72494	3.29354	2.67741	2.69355	4.24690	2.90347	2.73739	3.18146	2.89801	2.37887	2.77519	2.98518	4.58477	3.61503	
	0.00790	5.24104	5.96339	0.61958	0.77255	0.64245	0.74656														
81	2.78863	5.41296	3.26735	2.39252	4.72507	3.77182	3.50345	3.44153	2.51828	3.23885	4.46243	3.16997	3.53824	2.56495	2.04479	2.22304	1.99239	3.46883	4.63863	4.11504	120 t - - -
	2.68618	4.42225	2.77519	2.73123	3.46354	2.40513	3.72494	3.29354	2.67741	2.69355	4.24690	2.90347	2.73739	3.18146	2.89801	2.37887	2.77519	2.98518	4.58477	3.61503	
	0.00790	5.24104	5.96339	0.61958	0.77255	0.64245	0.74656														
82	2.80171	4.45913	4.82205	3.86119	3.09154	4.20559	4.53095	1.40528	4.04823	2.64017	2.45461	4.30024	4.56981	2.53793	4.12727	3.50883	2.92809	1.72196	5.08742	3.90625	121 i - - -
	2.68618	4.42225	2.77519	2.73123	3.46354	2.40513	3.72494	3.29354	2.67741	2.69355	4.24690	2.90347	2.73739	3.18146	2.89801	2.37887	2.77519	2.98518	4.58477	3.61503	
	0.00790	5.24104	5.96339	0.61958	0.77255	0.64245	0.74656														
83	2.64862	5.44851	2.92974	2.45411	4.77468	3.53058	3.58528	3.60480	2.22531	3.50044	4.49421	2.98990	4.15832	2.80974	1.75538	2.95572	2.93321	2.07657	5.89256	4.49355	122 r - - -
	2.68618	4.42225	2.77519	2.73123	3.46354	2.40513	3.72494	3.29354	2.67741	2.69355	4.24690	2.90347	2.73739	3.18146	2.89801	2.37887	2.77519	2.98518	4.58477	3.61503	
	0.01749	5.24104	4.41928	0.61958	0.77255	0.64245	0.74656														
84	2.87732	5.00971	3.52802	2.89917	2.68167	3.87173	1.71060	3.37829	2.95175	2.60355	3.72577	2.85309	3.48827	2.87294	3.19594	2.44377	2.75665	3.12392	5.54668	4.25296	123 h - - -
	2.68618	4.42225	2.77519	2.73123	3.46354	2.40513	3.72494	3.29354	2.67741	2.69355	4.24690	2.90347	2.73739	3.18146	2.89801	2.37887	2.77519	2.98518	4.58477	3.61503	
	0.00797	5.23152	5.95387	0.61958	0.77255	0.67109	0.71570														
85	2.55624	5.45964	2.50211	2.34991	4.79238	3.60090	3.38031	3.81062	2.17342	2.79107	4.50355	3.13564	3.90646	2.58570	2.45664	2.85812	2.36577	2.74983	4.43187	4.49637	124 k - - -
	2.68618	4.42225	2.77519	2.73123	3.46354	2.40513	3.72494	3.29354	2.67741	2.69355	4.24690	2.90347	2.73739	3.18146	2.89801	2.37887	2.77519	2.98518	4.58477	3.61503	
	0.00797	5.23152	5.95387	0.61958	0.77255	0.67109	0.71570														
86	2.64147	5.49118	3.22788	2.20167	4.83642	2.10191	3.79122	4.32205	2.30901	3.79620	4.53201	2.77727	3.77430	2.52474	2.12882	2.18305	3.10958	3.88196	5.92342	4.51298	125 g - - -

	2.68620	4.42227	2.77521	2.73114	3.46356	2.40515	3.72496	3.29356	2.67730	2.69357	4.24692	2.90349	2.73741	3.18148	2.89803	2.37889	2.77521	2.98512	4.58479	3.61505	
	0.07061	3.12230	3.72481	0.31731	1.30233	0.38519	1.14043														
87	2.40914	4.42551	5.00165	4.38707	1.94873	4.21714	3.85884	2.01485	4.17437	2.01663	3.15174	4.37594	4.58093	4.30764	3.83470	3.52795	2.80033	2.09416	2.63765	3.40131	127 f - - -
	2.68618	4.42225	2.77519	2.73123	3.46354	2.40513	3.72494	3.29354	2.67741	2.69355	4.24690	2.90347	2.73739	3.18146	2.89801	2.37887	2.77519	2.98518	4.58477	3.61503	
	0.00770	5.26666	5.98901	0.61958	0.77255	0.44228	1.02881														
88	2.64797	5.48757	2.37033	2.00396	4.82080	3.63791	3.97197	3.53849	2.23940	2.47986	4.53135	2.79508	3.37042	3.03635	3.02116	2.59981	2.53662	2.91252	5.92740	4.52369	128 e - - -
	2.68618	4.42225	2.77519	2.73123	3.46354	2.40513	3.72494	3.29354	2.67741	2.69355	4.24690	2.90347	2.73739	3.18146	2.89801	2.37887	2.77519	2.98518	4.58477	3.61503	
	0.00754	5.28723	6.00957	0.61958	0.77255	0.48576	0.95510														
89	2.67006	5.51047	2.59970	2.07160	4.20480	3.42530	3.24317	3.31102	2.11922	3.24020	4.55167	2.83938	3.98919	2.77308	2.36720	2.31051	3.11604	3.24925	5.94439	4.16959	129 e - - -
	2.68618	4.42225	2.77519	2.73123	3.46354	2.40513	3.72494	3.29354	2.67741	2.69355	4.24690	2.90347	2.73739	3.18146	2.89801	2.37887	2.77519	2.98518	4.58477	3.61503	
	0.00754	5.28723	6.00957	0.61958	0.77255	0.48576	0.95510														
90	2.47522	4.65385	3.52918	2.65406	3.67856	3.60281	3.95611	2.37952	2.90775	2.54084	3.81297	3.63929	4.35103	3.02471	2.67007	2.89868	2.55235	1.87774	5.42609	3.22884	130 v - - -
	2.68618	4.42225	2.77519	2.73123	3.46354	2.40513	3.72494	3.29354	2.67741	2.69355	4.24690	2.90347	2.73739	3.18146	2.89801	2.37887	2.77519	2.98518	4.58477	3.61503	
	0.00754	5.28723	6.00957	0.61958	0.77255	0.48576	0.95510														
91	2.56880	5.51500	2.18955	2.08281	4.85996	3.50153	3.46731	3.66501	2.16597	3.37793	4.55568	3.09693	3.54188	2.36914	3.01193	2.34571	2.68877	3.35631	5.94774	4.53685	131 e - - -
	2.68618	4.42225	2.77519	2.73123	3.46354	2.40513	3.72494	3.29354	2.67741	2.69355	4.24690	2.90347	2.73739	3.18146	2.89801	2.37887	2.77519	2.98518	4.58477	3.61503	
	0.04332	5.28723	3.28773	0.61958	0.77255	0.48576	0.95510														
92	1.94331	5.47136	2.44371	2.47796	4.80482	3.34107	3.95521	4.28266	2.69471	3.48747	4.51521	2.82647	2.93717	2.89887	3.02349	1.98878	2.66711	2.81049	5.91118	4.50732	132 a - - -
	2.68618	4.42225	2.77519	2.73123	3.46354	2.40513	3.72494	3.29354	2.67741	2.69355	4.24690	2.90347	2.73739	3.18146	2.89801	2.37887	2.77519	2.98518	4.58477	3.61503	
	0.10897	5.25172	2.32276	0.61958	0.77255	0.60895	0.78510														
93	2.30349	5.44053	2.67018	2.43255	4.77819	1.61139	3.91231	4.25895	2.40840	3.61343	4.48462	2.70813	3.63058	3.01173	3.14424	2.47016	2.86699	3.60391	5.87889	4.47205	133 g - - -
	2.68618	4.42225	2.77519	2.73123	3.46354	2.40513	3.72494	3.29354	2.67741	2.69355	4.24690	2.90347	2.73739	3.18146	2.89801	2.37887	2.77519	2.98518	4.58477	3.61503	
	0.03557	5.15139	3.53523	0.61958	0.77255	0.87599	0.53863														
94	2.48335	5.43860	2.25970	2.42639	4.78300	2.64713	3.89054	4.26852	2.19737	3.51229	4.47947	2.51590	2.60199	2.90933	2.81890	2.53089	2.77508	3.57098	5.87168	4.46109	134 k - - -
	2.68618	4.42225	2.77519	2.73123	3.46354	2.40513	3.72494	3.29354	2.67741	2.69355	4.24690	2.90347	2.73739	3.18146	2.89801	2.37887	2.77519	2.98518	4.58477	3.61503	
	0.02873	5.12470	3.79968	0.61958	0.77255	0.93283	0.49995														
95	2.86488	5.39413	2.05886	2.33642	4.11235	3.02779	3.88969	3.58563	2.32616	3.55493	4.43893	2.64507	3.72195	2.72995	3.12087	2.33066	3.07983	2.29595	5.83627	4.43490	135 d - - -
	2.68608	4.42235	2.77529	2.73133	3.46364	2.40523	3.72504	3.29328	2.67731	2.69365	4.24700	2.90332	2.73734	3.18124	2.89811	2.37882	2.77513	2.98528	4.58487	3.61513	
	0.22119	2.86546	1.95558	1.19663	0.35984	0.85396	0.55465														
96	2.40916	5.31249	2.99579	2.25626	3.51330	3.17034	3.15206	4.09711	2.13045	3.26015	3.84777	3.04529	3.89900	2.83998	2.48804	2.39816	2.67173	3.68827	4.85413	3.25442	142 k - - -
	2.68577	4.42248	2.77521	2.73126	3.46377	2.40499	3.72518	3.29377	2.67764	2.69358	4.24713	2.90370	2.73727	3.18138	2.89758	2.37896	2.77510	2.98522	4.58500	3.61526	
	0.07000	2.74447	5.70638	1.90925	0.16039	0.29666	1.35982														
97	1.61510	4.02196	2.66693	3.04979	3.96919	2.19304	3.75194	2.95118	2.90401	2.88395	4.03850	3.45372	4.07970	3.35976	3.24048	2.87042	3.23966	2.35652	5.49798	4.22030	153 a - - -
	2.68618	4.42225	2.77519	2.73123	3.46354	2.40513	3.72494	3.29354	2.67741	2.69355	4.24690	2.90347	2.73739	3.18146	2.89801	2.37887	2.77519	2.98518	4.58477	3.61503	
	0.00780	5.25361	5.97595	0.61958	0.77255	0.48052	0.96355														
98	2.72887	5.44503	3.03321	2.42027	4.76325	2.67089	3.54841	3.50338	2.61116	3.11084	4.49283	3.09711	4.17792	2.51862	2.28410	2.39691	2.20627	2.83206	4.60932	4.22474	154 t - - -
	2.68618	4.42225	2.77519	2.73123	3.46354	2.40513	3.72494	3.29354	2.67741	2.69355	4.24690	2.90347	2.73739	3.18146	2.89801	2.37887	2.77519	2.98518	4.58477	3.61503	
	0.00763	5.27541	5.99775	0.61958	0.77255	0.52898	0.88966														
99	2.72488	4.43480	4.97879	4.36613	2.08583	4.21918	3.94714	1.87469	3.78963	1.39972	2.95753	4.36875	4.58319	4.29810	3.50230	3.44277	3.28623	2.31525	5.05590	3.53814	155 l - - -
	2.68618	4.42225	2.77519	2.73123	3.46354	2.40513	3.72494	3.29354	2.67741	2.69355	4.24690	2.90347	2.73739	3.18146	2.89801	2.37887	2.77519	2.98518	4.58477	3.61503	
	0.00763	5.27541	5.99775	0.61958	0.77255	0.52898	0.88966														
100	2.41523	5.49561	2.70963	2.21957	4.49516	3.27448	3.64995	4.07811	2.18451	3.67006	4.53785	3.08208	4.16860	2.83823	2.68709	2.67909	2.03177	2.41319	5.93190	4.52459	156 t - - -
	2.68618	4.42225	2.77519	2.73123	3.46354	2.40513	3.72494	3.29354	2.67741	2.69355	4.24690	2.90347	2.73739	3.18146	2.89801	2.37887	2.77519	2.98518	4.58477	3.61503	
	0.00763	5.27541	5.99775	0.61958	0.77255	0.45965	0.99832														
101	2.07071	5.40472	3.07277	1.96394	4.70517	3.41422	3.07818	3.84502	2.74783	3.27869	3.98905	3.05967	3.85892	2.78063	3.23185	2.54834	2.52350	2.27407	5.86508	3.93362	157 e - - -
	2.68618	4.42225	2.77519	2.73123	3.46354	2.40513	3.72494	3.29354	2.67741	2.69355	4.24690	2.90347	2.73739	3.18146	2.89801	2.37887	2.77519	2.98518	4.58477	3.61503	
	0.00754	5.28723	6.00957	0.61958	0.77255	0.48576	0.95510														
102	2.83553	5.50422	2.90912	1.78203	3.93271	3.78049	3.54128	4.32712	2.34522	2.93262	4.54614	2.91921	4.17385	2.84585	2.11172	2.67181	2.30000	3.24720	5.93977	4.53172	158 e - - -
	2.68618	4.42225	2.77519	2.73123	3.46354	2.40513	3.72494	3.29354	2.67741	2.69355	4.24690	2.90347	2.73739	3.18146	2.89801	2.37887	2.77519	2.98518	4.58477	3.61503	
	0.00754	5.28723	6.00957	0.61958	0.77255	0.48576	0.95510														
103	2.39301	3.65782	4.31561	4.06682	2.88478	4.17983	3.39261	2.42049	3.75364	1.38989	3.39114	3.45289	4.54748	3.26087	4.05629	2.71819	3.07131	2.15017	5.11166	3.92544	159 l - - -
	2.68618	4.42225	2.77519	2.73123	3.46354	2.40513	3.72494	3.29354	2.67741	2.69355	4.24690	2.90347	2.73739	3.18146	2.89801	2.37887	2.77519	2.98518	4.58477	3.61503	
	0.00754	5.28723	6.00957	0.61958	0.77255	0.48576	0.95510														
104	2.51257	5.50771	2.21436	1.99872	4.84952	3.62638	3.24683	3.37427	2.49575	2.99819	4.54923	2.80671	4.17317	2.89194	2.73940	2.39705	2.43897	3.53951	5.94235	4.27541	160 e - - -
	2.68618	4.42225	2.77519	2.73123	3.46354	2.40513	3.72494	3.29354	2.67741	2.69355	4.24690	2.90347	2.73739	3.18146	2.89801	2.37887	2.77519	2.98518	4.58477	3.61503	
	0.00754	5.28723	6.00957	0.61958	0.77255	0.48576	0.95510														
105	5.27939	6.21900	6.03527	5.92565	2.77915	5.73471	2.02374	4.92239	5.60126	3.58629	5.42884	5.24905	5.98937	5.39525	5.46928	5.13939	5.46632	4.86068	0.51675	2.14983	161 W - - -
	2.68618	4.42225	2.77519	2.73123	3.46354	2.40513	3.72494	3.29354	2.67741	2.69355	4.24690	2.90347	2.73739	3.18146	2.89801	2.37887	2.77519	2.98518	4.58477	3.61503	
	0.00754	5.28723	6.00957	0.61958	0.77255	0.48576	0.95510														
106	2.64183	3.76519	4.15550	2.86976	3.48525	4.07975	2.99978	2.42728	3.05503	2.00919	3.54826	3.55458	4.45477	3.39735	2.98993	2.95768	2.40151	2.02451	4.62230	3.53322	162 l - - -
	2.68619	4.42226	2.77520	2.73116	3.46355	2.40514	3.72495	3.29355	2.67742	2.69348	4.24691	2.90347	2.73740	3.18147	2.89802	2.37888	2.77520	2.98519	4.58478	3.61504	
	0.11661	4.25847	2.34416	0.63802	0.75150	0.48576	0.95510														
107	2.08737	5.45012	1.96512	2.53552	4.78708	2.57396	3.92459	3.84216	2.66291	3.75217	4.49344	2.56273	3.26753	3.02375	2.83669	2.58347	2.51203	3.53892	5.88826	4.48235	165 d - - -
	2.68618	4.42225	2.77519	2.73123	3.46354	2.40513	3.72494	3.29354	2.67741	2.69355	4.24690	2.90347	2.73739	3.18146	2.89801	2.37887	2.77519	2.98518	4.58477	3.61503	
	0.04553	5.18909	3.24595	0.61958	0.77255	0.33512	1.25615														
108	2.13392	5.45479	2.63556	2.54300	3.58216	2.89134	3.13696	4.25413	2.38052	2.93883	3.81899	3.01389	2.34655	2.61125	3.14025	2.76231	3.22088	3.58853	5.89884	3.73958	166 a - - -
	2.68621	4.42281	2.77494	2.73131	3.46389	2.40486	3.72531	3.29367	2.67689	2.69391	4.24745	2.90369	2.73696	3.18143	2.89837	2.37896	2.77473	2.98549	4.58454	3.61534	

	0.28707	1.39837	5.97619	1.40434	0.28174	0.41932	1.07146														
109	2.67713	5.51593	1.99074	2.08986	4.86110	2.62529	3.10770	3.90875	2.23918	3.82125	4.55657	2.91124	3.48808	2.95450	3.19326	2.46096	2.56023	3.54099	5.94853	4.53747	173 d - - -
	2.68618	4.42225	2.77519	2.73123	3.46354	2.40513	3.72494	3.29354	2.67741	2.69355	4.24690	2.90347	2.73739	3.18146	2.89801	2.37887	2.77519	2.98518	4.58477	3.61503	
	0.03706	5.28723	3.46334	0.61958	0.77255	0.48576	0.95510														
110	2.77263	5.59141	3.00944	2.41881	4.92636	1.09874	4.04325	4.40998	2.62740	3.89497	4.64522	2.64104	3.65559	3.14951	3.09261	2.64768	3.33010	3.97877	6.03351	4.61828	174 g - - -
	2.68618	4.42225	2.77519	2.73123	3.46354	2.40513	3.72494	3.29354	2.67741	2.69355	4.24690	2.90347	2.73739	3.18146	2.89801	2.37887	2.77519	2.98518	4.58477	3.61503	
	0.00776	5.25793	5.98028	0.61958	0.77255	0.58876	0.80971														
111	2.38702	5.50232	2.88404	2.01885	4.84792	3.54645	3.75826	4.11078	1.86585	3.67527	4.54285	2.74308	3.15888	2.54868	2.68358	2.62405	2.62632	3.42284	5.93469	4.22017	175 k - - -
	2.68618	4.42225	2.77519	2.73123	3.46354	2.40513	3.72494	3.29354	2.67741	2.69355	4.24690	2.90347	2.73739	3.18146	2.89801	2.37887	2.77519	2.98518	4.58477	3.61503	
	0.00776	5.25793	5.98028	0.61958	0.77255	0.58876	0.80971														
112	2.52532	5.47334	2.89605	2.18229	4.80654	3.76950	3.56381	3.57845	2.48708	3.06782	4.51712	3.00585	2.67719	2.63962	2.49798	2.78448	2.41471	2.49003	5.91318	4.50949	176 e - - -
	2.68603	4.42233	2.77527	2.73131	3.46362	2.40521	3.72502	3.29344	2.67734	2.69351	4.24650	2.90355	2.73730	3.18154	2.89809	2.37895	2.77517	2.98495	4.58485	3.61511	
	0.17323	1.85452	5.98028	0.12944	2.10855	0.42635	1.05810														
113	2.29704	4.43946	4.99730	4.38381	3.01537	4.22697	4.55911	2.30224	4.17464	1.49897	3.00932	4.38053	3.85199	3.57926	3.82588	3.53709	3.21700	1.71737	3.09795	3.54365	178 l - - -
	2.68618	4.42225	2.77519	2.73123	3.46354	2.40513	3.72494	3.29354	2.67741	2.69355	4.24690	2.90347	2.73739	3.18146	2.89801	2.37887	2.77519	2.98518	4.58477	3.61503	
	0.00754	5.28723	6.00957	0.61958	0.77255	0.48576	0.95510														
114	2.37165	5.14148	5.79722	5.20101	3.43695	5.12099	5.45161	2.00412	5.02865	0.63694	2.99577	5.27886	5.33907	5.04234	5.01561	4.46389	4.06701	2.73003	5.72136	4.65740	179 L - - -
	2.68618	4.42225	2.77519	2.73123	3.46354	2.40513	3.72494	3.29354	2.67741	2.69355	4.24690	2.90347	2.73739	3.18146	2.89801	2.37887	2.77519	2.98518	4.58477	3.61503	
	0.00754	5.28723	6.00957	0.61958	0.77255	0.48576	0.95510														
115	2.75249	5.51522	2.93749	1.96969	4.86029	3.61425	3.23108	3.92161	2.21913	3.42976	4.55589	2.78713	4.17188	2.69236	2.05656	2.43749	2.42642	3.42932	5.94791	4.53698	180 e - - -
	2.68618	4.42225	2.77519	2.73123	3.46354	2.40513	3.72494	3.29354	2.67741	2.69355	4.24690	2.90347	2.73739	3.18146	2.89801	2.37887	2.77519	2.98518	4.58477	3.61503	
	0.00754	5.28723	6.00957	0.61958	0.77255	0.48576	0.95510														
116	4.04350	6.85106	2.32100	0.37273	6.09356	4.08274	4.74613	5.70253	3.92668	5.12069	6.03855	3.51859	4.78151	3.13016	4.64189	3.85029	4.36279	5.21093	7.25951	5.62130	181 E - - -
	2.68618	4.42225	2.77519	2.73123	3.46354	2.40513	3.72494	3.29354	2.67741	2.69355	4.24690	2.90347	2.73739	3.18146	2.89801	2.37887	2.77519	2.98518	4.58477	3.61503	
	0.00754	5.28723	6.00957	0.61958	0.77255	0.48576	0.95510														
117	2.81742	5.51005	2.97248	2.17818	4.05844	3.77936	3.35613	3.99449	2.26143	3.56610	4.55130	2.68651	4.17278	2.35151	2.12779	2.71656	2.20017	3.05218	5.94408	4.53449	182 r - - -
	2.68618	4.42225	2.77519	2.73123	3.46354	2.40513	3.72494	3.29354	2.67741	2.69355	4.24690	2.90347	2.73739	3.18146	2.89801	2.37887	2.77519	2.98518	4.58477	3.61503	
	0.00754	5.28723	6.00957	0.61958	0.77255	0.48576	0.95510														
118	3.10787	5.01130	3.75844	3.17649	4.19011	4.00265	4.19733	3.56710	3.00447	2.65500	4.10493	3.63208	4.38287	2.92533	1.22936	3.08593	2.06796	3.11227	2.89840	4.28804	183 r - - -
	2.68618	4.42225	2.77519	2.73123	3.46354	2.40513	3.72494	3.29354	2.67741	2.69355	4.24690	2.90347	2.73739	3.18146	2.89801	2.37887	2.77519	2.98518	4.58477	3.61503	
	0.00754	5.28723	6.00957	0.61958	0.77255	0.48576	0.95510														
119	2.75635	4.60715	2.95112	2.32922	4.78452	3.46724	3.62167	3.42409	2.44577	3.49206	4.50847	3.15984	4.18160	2.81400	2.49407	2.62396	1.53590	3.03387	5.90819	4.51118	184 t - - -
	2.68618	4.42225	2.77519	2.73123	3.46354	2.40513	3.72494	3.29354	2.67741	2.69355	4.24690	2.90347	2.73739	3.18146	2.89801	2.37887	2.77519	2.98518	4.58477	3.61503	
	0.00754	5.28723	6.00957	0.61958	0.77255	0.48576	0.95510														
120	2.98152	4.43780	5.03676	4.42091	2.51466	4.23497	4.31721	1.81492	4.20388	1.87524	2.87788	4.40013	4.59818	4.33302	4.20885	3.54671	3.04802	1.70122	3.20759	2.61247	185 v - - -
	2.68618	4.42225	2.77519	2.73123	3.46354	2.40513	3.72494	3.29354	2.67741	2.69355	4.24690	2.90347	2.73739	3.18146	2.89801	2.37887	2.77519	2.98518	4.58477	3.61503	
	0.00754	5.28723	6.00957	0.61958	0.77255	0.48576	0.95510														
121	2.33098	5.38366	3.31528	2.37558	4.67636	3.26147	3.42595	3.59189	2.28608	3.36376	4.13876	3.10185	4.19705	3.05470	2.44700	2.56613	1.83892	2.94972	4.43278	4.18314	186 t - - -
	2.68618	4.42225	2.77519	2.73123	3.46354	2.40513	3.72494	3.29354	2.67741	2.69355	4.24690	2.90347	2.73739	3.18146	2.89801	2.37887	2.77519	2.98518	4.58477	3.61503	
	0.00754	5.28723	6.00957	0.61958	0.77255	0.48576	0.95510														
122	2.23527	4.05186	5.02429	4.40898	1.84429	4.04013	4.56379	2.14165	4.19389	2.28252	3.33713	4.39256	4.34643	4.32515	4.20216	3.39719	3.10307	1.51167	4.06444	3.47297	187 v - - -
	2.68618	4.42225	2.77519	2.73123	3.46354	2.40513	3.72494	3.29354	2.67741	2.69355	4.24690	2.90347	2.73739	3.18146	2.89801	2.37887	2.77519	2.98518	4.58477	3.61503	
	0.02124	5.28723	4.13786	0.61958	0.77255	0.48576	0.95510														
123	2.62291	5.30723	3.26426	2.63113	3.55422	3.54265	2.71320	3.85251	2.50886	3.45492	3.90209	3.32061	4.20539	2.77128	2.34738	2.22768	2.35186	3.38628	4.27163	2.60396	188 s - - -
	2.68621	4.42239	2.77508	2.73118	3.46368	2.40516	3.72456	3.29307	2.67742	2.69343	4.24704	2.90343	2.73713	3.18161	2.89815	2.37901	2.77534	2.98533	4.58491	3.61518	
	0.28948	2.49667	1.77791	1.11390	0.39791	0.53526	0.88073														
124	2.00743	5.40344	2.92277	2.44551	4.73932	2.53134	3.31927	4.05296	2.47625	3.34379	4.44652	2.74263	2.67603	2.80417	2.84829	2.53915	2.87802	3.07669	5.84166	4.43631	193 a - - -
	2.68618	4.42225	2.77519	2.73123	3.46354	2.40513	3.72494	3.29354	2.67741	2.69355	4.24690	2.90347	2.73739	3.18146	2.89801	2.37887	2.77519	2.98518	4.58477	3.61503	
	0.00918	5.09151	5.81386	0.61958	0.77255	0.47806	0.96755														
125	2.62332	5.41636	2.26911	2.55946	4.73398	3.26590	3.77252	3.61531	2.56333	2.41404	4.31448	3.05318	2.99483	2.79972	2.74977	2.27762	2.83627	2.68739	5.86584	4.47207	194 d - - -
	2.68618	4.42225	2.77519	2.73123	3.46354	2.40513	3.72494	3.29354	2.67741	2.69355	4.24690	2.90347	2.73739	3.18146	2.89801	2.37887	2.77519	2.98518	4.58477	3.61503	
	0.03487	5.21865	3.54548	0.61958	0.77255	0.62893	0.76177														
126	2.47063	4.82371	2.29338	2.56001	4.78549	2.40442	3.70585	4.26380	2.35786	3.75199	4.49495	2.85389	2.60431	2.96126	3.16321	2.58753	2.44257	2.96421	5.89070	3.88720	195 d - - -
	2.68618	4.42225	2.77519	2.73123	3.46354	2.40513	3.72494	3.29354	2.67741	2.69355	4.24690	2.90347	2.73739	3.18146	2.89801	2.37887	2.77519	2.98518	4.58477	3.61503	
	0.00817	5.20681	5.92916	0.61958	0.77255	0.35409	1.21003														
127	2.63435	5.51762	2.10153	2.01735	4.86366	2.54956	3.54214	4.35032	2.03254	3.44448	4.29269	2.86771	3.45738	2.75253	2.91871	2.57814	3.01048	3.90878	5.94972	4.53817	196 e - - -
	2.68574	4.42313	2.77403	2.73125	3.46403	2.40423	3.72526	3.29432	2.67818	2.69421	4.24778	2.90393	2.73642	3.18208	2.89783	2.37884	2.77557	2.98498	4.58432	3.61592	
	0.58643	0.86047	3.87624	1.31638	0.31212	0.48576	0.95510														
128	2.15429	4.50484	2.97153	2.49623	4.38932	2.38329	3.80430	3.48078	2.53978	3.18160	4.04509	2.84762	3.87425	2.86070	2.47908	2.48474	2.53387	3.46879	5.88115	3.60207	216 a - - -
	2.68618	4.42225	2.77519	2.73123	3.46354	2.40513	3.72494	3.29354	2.67741	2.69355	4.24690	2.90347	2.73739	3.18146	2.89801	2.37887	2.77519	2.98518	4.58477	3.61503	
	0.01665	5.26878	4.47710	0.61958	0.77255	0.55218	0.85730														
129	2.95935	4.65012	4.09961	3.15240	2.84015	3.82154	4.32541	3.12582	3.45584	2.60850	3.75858	3.59997	4.43277	3.73694	1.96980	3.31666	2.81886	2.83560	1.79903	2.40033	217 w - - -
	2.68618	4.42225	2.77519	2.73123	3.46354	2.40513	3.72494	3.29354	2.67741	2.69355	4.24690	2.90347	2.73739	3.18146	2.89801	2.37887	2.77519	2.98518	4.58477	3.61503	
	0.00775	5.25988	5.98223	0.61958	0.77255	0.53053	0.88745														
130	2.20641	4.45377	4.82388	4.06972	3.03054	3.67717	4.51850	2.04510	3.77515	1.83964	2.99605	4.29378	4.56092	3.52083	2.99583	3.03448	2.53071	2.24182	3.79173	3.05114	218 l - - -
	2.68615	4.42229	2.77523	2.73127	3.46358	2.40517	3.72498	3.29358	2.67745	2.69339	4.24694	2.90351	2.73743	3.18124	2.89790	2.37891	2.77523	2.98513	4.58481	3.61507	
	0.08740	2.51097	5.99113	0.21134	1.65809	0.44636	1.02152														

131	3.08595	4.21267	5.16668	4.55394	2.58077	4.37673	4.71275	1.48890	4.34194	1.23978	3.34387	4.54229	4.72456	4.45958	4.34499	3.69223	3.33891	2.09256	4.08871	4.01475	220 l - - -
	2.68618	4.42225	2.77519	2.73123	3.46354	2.40513	3.72494	3.29354	2.67741	2.69355	4.24690	2.90347	2.73739	3.18146	2.89801	2.37887	2.77519	2.98518	4.58477	3.61503	
	0.00754	5.28723	6.00957	0.61958	0.77255	0.48576	0.95510														
132	2.79972	5.48890	1.16085	2.28757	4.81223	3.66797	3.56715	4.28434	2.74348	3.41206	4.53649	3.27209	4.19613	3.01710	3.07890	2.99803	2.40735	3.34240	5.93434	4.53457	221 d - - -
	2.68618	4.42225	2.77519	2.73123	3.46354	2.40513	3.72494	3.29354	2.67741	2.69355	4.24690	2.90347	2.73739	3.18146	2.89801	2.37887	2.77519	2.98518	4.58477	3.61503	
	0.00754	5.28723	6.00957	0.61958	0.77255	0.48576	0.95510														
133	2.90893	3.69187	4.99901	4.09604	1.91967	4.22698	4.55919	2.40434	4.17579	2.01556	3.22420	4.38116	4.59092	4.31173	2.56889	3.23117	3.29256	2.22719	2.23820	3.14941	222 f - - -
	2.68618	4.42225	2.77519	2.73123	3.46354	2.40513	3.72494	3.29354	2.67741	2.69355	4.24690	2.90347	2.73739	3.18146	2.89801	2.37887	2.77519	2.98518	4.58477	3.61503	
	0.00754	5.28723	6.00957	0.61958	0.77255	0.48576	0.95510														
134	2.58826	5.50280	2.03814	2.20191	4.84241	3.67057	3.42035	3.19107	2.65899	3.80575	4.54490	3.25038	4.17421	2.53464	2.84805	2.16867	2.10776	3.75195	5.93875	4.10388	223 d - - -
	2.68618	4.42225	2.77519	2.73123	3.46354	2.40513	3.72494	3.29354	2.67741	2.69355	4.24690	2.90347	2.73739	3.18146	2.89801	2.37887	2.77519	2.98518	4.58477	3.61503	
	0.00754	5.28723	6.00957	0.61958	0.77255	0.48576	0.95510														
135	2.75354	4.45658	4.89029	4.28388	2.78909	4.21468	4.54110	1.84806	4.09755	2.31183	3.39183	4.33204	4.57964	4.25377	4.15298	1.73184	1.83441	2.49265	4.33020	3.74057	224 s - - -
	2.68618	4.42225	2.77519	2.73123	3.46354	2.40513	3.72494	3.29354	2.67741	2.69355	4.24690	2.90347	2.73739	3.18146	2.89801	2.37887	2.77519	2.98518	4.58477	3.61503	
	0.00754	5.28723	6.00957	0.61958	0.77255	0.48576	0.95510														
136	2.59521	5.44554	3.00815	2.23014	4.76163	3.79211	3.55028	4.22764	2.63261	3.15592	4.24663	3.17632	4.18486	2.78194	2.73885	2.63093	1.54150	2.55707	5.89595	4.50322	225 t - - -
	2.68618	4.42225	2.77519	2.73123	3.46354	2.40513	3.72494	3.29354	2.67741	2.69355	4.24690	2.90347	2.73739	3.18146	2.89801	2.37887	2.77519	2.98518	4.58477	3.61503	
	0.00754	5.28723	6.00957	0.61958	0.77255	0.48576	0.95510														
137	3.32353	4.69862	5.16359	4.55793	2.76563	3.97017	4.81611	2.75197	4.37353	0.70377	2.88113	4.61856	4.82504	3.11386	4.41497	3.80953	3.55163	2.58693	5.28308	4.13641	226 l - - -
	2.68618	4.42225	2.77519	2.73123	3.46354	2.40513	3.72494	3.29354	2.67741	2.69355	4.24690	2.90347	2.73739	3.18146	2.89801	2.37887	2.77519	2.98518	4.58477	3.61503	
	0.00754	5.28723	6.00957	0.61958	0.77255	0.48576	0.95510														
138	2.92971	5.00883	3.26690	2.30150	4.81994	3.65365	3.13890	4.29719	2.14997	3.29070	4.16143	3.08346	4.17771	2.56521	2.55392	2.79016	1.50976	3.51427	5.92691	4.52376	227 t - - -
	2.68618	4.42225	2.77519	2.73123	3.46354	2.40513	3.72494	3.29354	2.67741	2.69355	4.24690	2.90347	2.73739	3.18146	2.89801	2.37887	2.77519	2.98518	4.58477	3.61503	
	0.00754	5.28723	6.00957	0.61958	0.77255	0.48576	0.95510														
139	1.45808	3.08070	3.67710	3.12233	4.27727	3.89900	4.22116	3.66545	3.10268	3.22476	3.78720	1.99001	2.43166	3.43486	3.52641	2.56826	2.93972	3.26870	5.63467	4.35313	228 a - - -
	2.68618	4.42225	2.77519	2.73123	3.46354	2.40513	3.72494	3.29354	2.67741	2.69355	4.24690	2.90347	2.73739	3.18146	2.89801	2.37887	2.77519	2.98518	4.58477	3.61503	
	0.00754	5.28723	6.00957	0.61958	0.77255	0.48576	0.95510														
140	2.15475	5.08871	3.16727	2.68356	4.03775	3.88307	4.09683	3.30572	2.88884	2.01905	4.00028	3.21551	3.20492	2.83966	2.99151	2.87002	2.35804	2.19380	5.61635	4.31101	229 l - - -
	2.68618	4.42225	2.77519	2.73123	3.46354	2.40513	3.72494	3.29354	2.67741	2.69355	4.24690	2.90347	2.73739	3.18146	2.89801	2.37887	2.77519	2.98518	4.58477	3.61503	
	0.00754	5.28723	6.00957	0.61958	0.77255	0.48576	0.95510														
141	2.44167	5.50080	2.48530	2.48231	4.83965	2.81772	3.70720	4.10287	2.42471	3.28846	3.99577	2.93594	3.96011	2.93982	2.55607	2.11810	2.36381	2.83441	5.93723	4.53005	230 s - - -
	2.68618	4.42225	2.77519	2.73123	3.46354	2.40513	3.72494	3.29354	2.67741	2.69355	4.24690	2.90347	2.73739	3.18146	2.89801	2.37887	2.77519	2.98518	4.58477	3.61503	
	0.00754	5.28723	6.00957	0.61958	0.77255	0.48576	0.95510														
142	2.74301	5.52134	1.78668	2.07871	4.86685	2.34046	3.96851	4.07973	2.14914	3.82680	4.56205	2.95748	3.25595	2.83901	3.07007	2.57028	3.09530	3.91225	5.95359	4.54183	231 d - - -
	2.68599	4.42247	2.77501	2.73069	3.46376	2.40515	3.72486	3.29376	2.67763	2.69377	4.24712	2.90362	2.73762	3.18149	2.89757	2.37869	2.77533	2.98530	4.58499	3.61525	
	0.73800	0.91142	2.12048	0.07681	2.60457	0.48576	0.95510														
143	2.70740	4.90023	2.25671	2.28834	4.78521	3.03192	3.90951	3.83878	2.43503	3.63840	4.48714	2.85228	1.93196	2.74011	3.13820	2.60040	2.66334	3.58405	5.88083	4.47288	234 p - - -
	2.68618	4.42225	2.77519	2.73123	3.46354	2.40513	3.72494	3.29354	2.67741	2.69355	4.24690	2.90347	2.73739	3.18146	2.89801	2.37887	2.77519	2.98518	4.58477	3.61503	
	0.00855	5.16221	5.88456	0.61958	0.77255	0.31047	1.32088														
144	3.28163	5.01426	5.81401	5.27953	3.86774	5.19262	5.69874	1.82380	5.15119	1.43928	4.05030	5.35405	5.46318	5.31838	5.22309	4.56389	3.62511	0.84352	6.08551	4.90874	235 v - - -
	2.68618	4.42225	2.77519	2.73123	3.46354	2.40513	3.72494	3.29354	2.67741	2.69355	4.24690	2.90347	2.73739	3.18146	2.89801	2.37887	2.77519	2.98518	4.58477	3.61503	
	0.00754	5.28723	6.00957	0.61958	0.77255	0.48576	0.95510														
145	2.88971	5.34335	3.12775	2.46625	4.02661	3.81404	3.26980	3.74189	2.25703	3.00712	4.22441	3.24653	3.63738	2.96904	2.46259	2.55790	2.05508	2.24666	4.85642	3.92783	236 t - - -
	2.68618	4.42225	2.77519	2.73123	3.46354	2.40513	3.72494	3.29354	2.67741	2.69355	4.24690	2.90347	2.73739	3.18146	2.89801	2.37887	2.77519	2.98518	4.58477	3.61503	
	0.00754	5.28723	6.00957	0.61958	0.77255	0.48576	0.95510														
146	3.93691	5.22368	5.90843	5.30136	1.43500	5.19823	5.48707	1.75540	5.11764	1.01638	3.02507	5.36842	5.38562	5.07758	5.06873	4.54279	4.15006	2.74930	4.75974	4.64404	237 l - - -
	2.68618	4.42225	2.77519	2.73123	3.46354	2.40513	3.72494	3.29354	2.67741	2.69355	4.24690	2.90347	2.73739	3.18146	2.89801	2.37887	2.77519	2.98518	4.58477	3.61503	
	0.00754	5.28723	6.00957	0.61958	0.77255	0.48576	0.95510														
147	3.01039	5.50991	2.78278	2.34638	4.84998	1.52103	3.69252	4.33264	2.25669	2.80351	4.55212	2.77599	4.02613	2.99399	3.03662	2.88540	2.51025	3.89791	5.94531	4.53699	238 g - - -
	2.68618	4.42225	2.77519	2.73123	3.46354	2.40513	3.72494	3.29354	2.67741	2.69355	4.24690	2.90347	2.73739	3.18146	2.89801	2.37887	2.77519	2.98518	4.58477	3.61503	
	0.01417	5.28723	4.70878	0.61958	0.77255	0.48576	0.95510														
148	2.64833	5.52382	1.93779	2.49434	4.86865	2.72335	3.97081	4.35503	2.08148	3.82893	4.56507	3.24674	3.53889	2.88204	2.76947	1.72663	3.24345	3.91443	5.95627	4.54442	239 s - - -
	2.68618	4.42225	2.77519	2.73123	3.46354	2.40513	3.72494	3.29354	2.67741	2.69355	4.24690	2.90347	2.73739	3.18146	2.89801	2.37887	2.77519	2.98518	4.58477	3.61503	
	0.00759	5.28065	6.00299	0.61958	0.77255	0.51012	0.91735														
149	2.81229	5.33924	2.67599	2.77843	4.61759	3.65940	3.42257	3.87163	2.77714	3.61594	4.39890	2.86014	1.96720	3.13247	3.25533	2.62591	1.97601	3.19978	5.81499	2.53015	240 p - - -
	2.68618	4.42225	2.77519	2.73123	3.46354	2.40513	3.72494	3.29354	2.67741	2.69355	4.24690	2.90347	2.73739	3.18146	2.89801	2.37887	2.77519	2.98518	4.58477	3.61503	
	0.00759	5.28065	6.00299	0.61958	0.77255	0.47083	0.97946														
150	2.20673	5.51560	2.72017	2.57430	4.86055	3.13934	3.37720	4.34638	1.74566	3.82079	4.55635	2.77729	2.81039	3.06249	2.40617	2.96769	2.39541	3.74804	5.94831	4.53749	241 k - - -
	2.68469	4.42368	2.77595	2.73149	3.46473	2.40174	3.72532	3.29482	2.67811	2.69477	4.24761	2.90314	2.73693	3.18262	2.89666	2.37986	2.77461	2.98644	4.58620	3.61621	
	0.42908	1.06006	6.00957	1.97524	0.14935	0.48576	0.95510														
151	3.14781	5.49761	2.55361	1.87451	3.30555	3.88632	3.46839	4.22803	2.91496	3.76314	4.56222	3.37629	4.30630	2.87650	3.40055	3.13250	3.38317	3.85987	5.79530	1.23128	251 y - - -
	2.68619	4.42232	2.77526	2.73130	3.46361	2.40476	3.72393	3.29361	2.67748	2.69362	4.24696	2.90353	2.73746	3.18153	2.89807	2.37894	2.77526	2.98525	4.58484	3.61510	
	0.14304	2.03392	6.00957	0.14601	1.99619	0.48576	0.95510														
152	2.37514	5.05750	4.68235	4.49130	5.61270	0.45721	5.50147	5.08819	4.60336	4.75155	5.52350	4.42060	4.64577	4.14229	4.84518	2.56869	2.64074	4.32446	6.93453	5.79621	253 G - - -
	2.68592	4.42229	2.77523	2.73127	3.46358	2.40517	3.72498	3.29358	2.67745	2.69359	4.24694	2.90351	2.73743	3.18150	2.89805	2.37891	2.77523	2.98522	4.58481	3.61432	
	0.04522	3.17595	6.00957	0.66184	0.72547	0.48576	0.95510														
153	2.87298	4.51642	4.68318	4.09188	3.01653	1.11541	4.51990	2.87455	3.94801	2.00399	2.63081	4.23798	4.56559	4.14418	4.08096	3.35770	2.95990	2.70151	5.13848	3.95313	256 g - - -

	2.68618	4.42225	2.77519	2.73123	3.46354	2.40513	3.72494	3.29354	2.67741	2.69355	4.24690	2.90347	2.73739	3.18146	2.89801	2.37887	2.77519	2.98518	4.58477	3.61503	
	0.00754	5.28723	6.00957	0.61958	0.77255	0.48576	0.95510														
154	3.53368	4.87198	5.48200	4.87283	1.38488	4.73654	5.03602	2.40801	4.67335	1.01875	2.98812	4.89412	4.50902	4.73194	4.66212	4.06046	3.75758	2.82055	5.40101	3.39551	257 l - - -
	2.68618	4.42225	2.77519	2.73123	3.46354	2.40513	3.72494	3.29354	2.67741	2.69355	4.24690	2.90347	2.73739	3.18146	2.89801	2.37887	2.77519	2.98518	4.58477	3.61503	
	0.00754	5.28723	6.00957	0.61958	0.77255	0.48576	0.95510														
155	2.00952	3.48697	3.80354	3.23933	1.91005	2.06445	3.68472	3.34840	3.20410	2.98195	3.93074	3.50993	4.36545	2.85409	3.58552	2.37031	3.26615	3.11190	5.40258	3.95370	258 f - - -
	2.68618	4.42225	2.77519	2.73123	3.46354	2.40513	3.72494	3.29354	2.67741	2.69355	4.24690	2.90347	2.73739	3.18146	2.89801	2.37887	2.77519	2.98518	4.58477	3.61503	
	0.00754	5.28723	6.00957	0.61958	0.77255	0.48576	0.95510														
156	3.06625	4.44089	5.02823	4.41321	2.51242	3.82358	4.56819	1.99866	4.19851	2.00842	3.21752	4.39746	3.88733	4.32942	4.20706	3.54707	3.29838	1.84304	1.79660	3.38016	259 w - - -
	2.68618	4.42225	2.77519	2.73123	3.46354	2.40513	3.72494	3.29354	2.67741	2.69355	4.24690	2.90347	2.73739	3.18146	2.89801	2.37887	2.77519	2.98518	4.58477	3.61503	
	0.00754	5.28723	6.00957	0.61958	0.77255	0.48576	0.95510														
157	5.29544	6.67499	5.88820	5.34481	6.44237	5.25966	5.78928	6.29247	4.05847	5.54718	6.62453	5.56986	5.80517	5.07070	0.07776	5.41013	5.54666	5.96626	7.03466	6.28171	260 R - - -
	2.68618	4.42225	2.77519	2.73123	3.46354	2.40513	3.72494	3.29354	2.67741	2.69355	4.24690	2.90347	2.73739	3.18146	2.89801	2.37887	2.77519	2.98518	4.58477	3.61503	
	0.00754	5.28723	6.00957	0.61958	0.77255	0.48576	0.95510														
158	1.95351	3.99862	4.92382	4.31539	3.07155	2.17546	4.54746	2.75488	4.12220	1.91997	2.47278	4.34772	3.94602	4.27235	4.16600	3.42738	3.09259	1.76178	5.07212	3.89374	261 v - - -
	2.68618	4.42225	2.77519	2.73123	3.46354	2.40513	3.72494	3.29354	2.67741	2.69355	4.24690	2.90347	2.73739	3.18146	2.89801	2.37887	2.77519	2.98518	4.58477	3.61503	
	0.00754	5.28723	6.00957	0.61958	0.77255	0.48576	0.95510														
159	1.77411	4.89630	2.86810	2.73091	4.75469	3.57325	3.72239	3.82235	2.52755	3.73254	4.20984	2.95449	2.04408	3.09086	2.58174	2.58376	2.64252	3.31544	5.89218	4.50069	262 a - - -
	2.68618	4.42225	2.77519	2.73123	3.46354	2.40513	3.72494	3.29354	2.67741	2.69355	4.24690	2.90347	2.73739	3.18146	2.89801	2.37887	2.77519	2.98518	4.58477	3.61503	
	0.00754	5.28723	6.00957	0.61958	0.77255	0.48576	0.95510														
160	2.36633	5.51506	2.68014	2.20444	4.86006	3.33815	3.52458	4.16763	2.10739	3.27244	4.55574	3.06354	3.22313	2.63297	1.97874	2.69790	2.89483	3.62889	4.76347	4.53688	263 r - - -
	2.68618	4.42225	2.77519	2.73123	3.46354	2.40513	3.72494	3.29354	2.67741	2.69355	4.24690	2.90347	2.73739	3.18146	2.89801	2.37887	2.77519	2.98518	4.58477	3.61503	
	0.00754	5.28723	6.00957	0.61958	0.77255	0.48576	0.95510														
161	2.32129	4.50095	2.60556	1.94091	4.29277	3.05365	3.96781	4.32834	2.48722	3.52598	4.54673	2.77804	3.60732	2.81123	2.95494	2.00270	2.70982	3.29091	5.08295	4.53202	264 e - - -
	2.68618	4.42225	2.77519	2.73123	3.46354	2.40513	3.72494	3.29354	2.67741	2.69355	4.24690	2.90347	2.73739	3.18146	2.89801	2.37887	2.77519	2.98518	4.58477	3.61503	
	0.00754	5.28723	6.00957	0.61958	0.77255	0.48576	0.95510														
162	2.67274	4.48889	3.11888	3.19993	2.35416	3.89895	4.50092	2.53463	3.95301	1.73315	2.53885	4.23598	3.99625	4.14267	4.07317	3.07749	3.19778	2.39248	2.40466	3.91988	265 l - - -
	2.68574	4.42407	2.77409	2.73043	3.46379	2.40567	3.72579	3.29389	2.67666	2.69457	4.24651	2.90363	2.73718	3.18175	2.89918	2.37924	2.77417	2.98380	4.58659	3.61633	
	0.92087	1.36062	1.06327	2.48744	0.08678	0.48576	0.95510														
163	2.23867	5.31193	2.63027	2.35122	4.64773	3.35792	3.59220	4.12721	2.32854	3.46307	4.35510	2.24821	3.99374	2.88799	2.53869	2.64277	2.27823	3.14597	5.75017	4.34486	290 a - - -
	2.68618	4.42225	2.77519	2.73123	3.46354	2.40513	3.72494	3.29354	2.67741	2.69355	4.24690	2.90347	2.73739	3.18146	2.89801	2.37887	2.77519	2.98518	4.58477	3.61503	
	0.26679	4.86735	1.48514	0.61958	0.77255	0.75951	0.63091														
164	2.34915	5.15244	2.68259	2.22930	4.07894	3.45181	3.64146	3.09271	2.46903	3.05962	4.21036	2.65636	3.78283	2.83932	2.84230	2.57932	2.45084	2.82139	4.53865	4.25593	291 e - - -
	2.68618	4.42225	2.77519	2.73123	3.46354	2.40513	3.72494	3.29354	2.67741	2.69355	4.24690	2.90347	2.73739	3.18146	2.89801	2.37887	2.77519	2.98518	4.58477	3.61503	
	0.01196	4.82834	5.55069	0.61958	0.77255	1.24232	0.34068														
165	2.10930	5.22790	2.42037	2.55557	3.74284	2.52885	3.80270	3.44651	2.30356	3.27838	4.27948	2.83219	3.67955	2.84547	2.64774	2.57685	2.95157	3.32685	5.68572	4.06738	292 a - - -
	2.68618	4.42225	2.77519	2.73123	3.46354	2.40513	3.72494	3.29354	2.67741	2.69355	4.24690	2.90347	2.73739	3.18146	2.89801	2.37887	2.77519	2.98518	4.58477	3.61503	
	0.01160	4.85852	5.58087	0.61958	0.77255	0.97891	0.47116														
166	2.17355	5.30985	2.46910	2.53721	4.63078	2.69948	3.59719	3.33252	2.29106	3.23232	4.35692	2.67076	3.89736	2.68340	2.81281	2.71055	2.81151	2.82469	5.75725	4.07217	293 a - - -
	2.68618	4.42225	2.77519	2.73123	3.46354	2.40513	3.72494	3.29354	2.67741	2.69355	4.24690	2.90347	2.73739	3.18146	2.89801	2.37887	2.77519	2.98518	4.58477	3.61503	
	0.01051	4.95680	5.67914	0.61958	0.77255	0.23407	1.56689														
167	2.78600	5.51469	2.60363	2.45361	4.85938	1.93959	3.96389	4.34546	2.45416	3.81982	4.55568	2.58947	2.92196	2.69329	3.05407	2.55534	2.14023	3.90517	5.94745	4.53608	294 g - - -
	2.68618	4.42225	2.77519	2.73123	3.46354	2.40513	3.72494	3.29354	2.67741	2.69355	4.24690	2.90347	2.73739	3.18146	2.89801	2.37887	2.77519	2.98518	4.58477	3.61503	
	0.00763	5.27529	5.99763	0.61958	0.77255	0.52940	0.88906														
168	2.47161	4.17225	3.85623	3.29005	3.66411	1.32965	3.64439	3.28944	3.24954	2.90039	3.74318	3.70969	2.93531	3.55702	3.43696	2.66871	2.66457	2.68885	4.68917	4.12016	295 g - - -
	2.68618	4.42225	2.77519	2.73123	3.46354	2.40513	3.72494	3.29354	2.67741	2.69355	4.24690	2.90347	2.73739	3.18146	2.89801	2.37887	2.77519	2.98518	4.58477	3.61503	
	0.00763	5.27529	5.99763	0.61958	0.77255	0.52940	0.88906														
169	2.80575	5.50475	2.81018	2.12397	4.84778	3.12454	3.56145	4.07740	2.31845	3.61010	4.05864	3.00651	3.99195	2.72415	2.02236	2.74903	2.10877	3.13085	5.93865	4.00331	296 r - - -
	2.68618	4.42225	2.77519	2.73123	3.46354	2.40513	3.72494	3.29354	2.67741	2.69355	4.24690	2.90347	2.73739	3.18146	2.89801	2.37887	2.77519	2.98518	4.58477	3.61503	
	0.00763	5.27529	5.99763	0.61958	0.77255	0.45941	0.99874														
170	2.92903	4.46216	5.05588	4.44223	2.48594	3.92412	4.60041	1.88892	4.22877	2.25953	2.84274	4.42757	4.62641	4.35949	4.23765	3.57778	3.32301	1.46027	5.09033	2.08687	297 v - - -
	2.68618	4.42225	2.77519	2.73123	3.46354	2.40513	3.72494	3.29354	2.67741	2.69355	4.24690	2.90347	2.73739	3.18146	2.89801	2.37887	2.77519	2.98518	4.58477	3.61503	
	0.00754	5.28723	6.00957	0.61958	0.77255	0.48576	0.95510														
171	2.91383	4.46516	4.82853	4.22563	2.46243	4.20489	3.57136	2.16320	3.87224	1.58210	3.58286	4.30137	4.57059	3.69967	2.72355	2.88578	2.51824	2.21698	4.07827	3.27864	298 l - - -
	2.68618	4.42225	2.77519	2.73123	3.46354	2.40513	3.72494	3.29354	2.67741	2.69355	4.24690	2.90347	2.73739	3.18146	2.89801	2.37887	2.77519	2.98518	4.58477	3.61503	
	0.00754	5.28723	6.00957	0.61958	0.77255	0.48576	0.95510														
172	2.57549	4.32865	2.91412	2.75187	4.07416	2.92151	3.67543	4.15044	2.75144	3.49399	4.45179	2.09295	4.19431	3.10918	2.90272	2.22404	1.67723	3.58823	5.86039	4.47982	299 t - - -
	2.68618	4.42225	2.77519	2.73123	3.46354	2.40513	3.72494	3.29354	2.67741	2.69355	4.24690	2.90347	2.73739	3.18146	2.89801	2.37887	2.77519	2.98518	4.58477	3.61503	
	0.01471	5.28723	4.65139	0.61958	0.77255	0.48576	0.95510														
173	1.72764	5.48665	3.19998	2.46374	4.81300	3.71670	3.85791	4.28537	2.54960	3.78533	4.53672	3.13475	2.48773	2.99204	3.23154	1.50824	3.25810	3.72956	5.93413	4.53441	300 s - - -
	2.68618	4.42225	2.77519	2.73123	3.46354	2.40513	3.72494	3.29354	2.67741	2.69355	4.24690	2.90347	2.73739	3.18146	2.89801	2.37887	2.77519	2.98518	4.58477	3.61503	
	0.27831	5.28011	1.43613	0.61958	0.77255	0.51207	0.91443														
174	2.46319	5.41040	2.63179	1.41064	4.20803	3.33906	3.86010	4.23715	2.48063	3.71416	4.45314	2.42200	4.06272	2.75823	3.09746	2.40004	3.06909	3.79924	5.84447	4.43225	301 e - - -
	2.68618	4.42225	2.77519	2.73123	3.46354	2.40513	3.72494	3.29354	2.67741	2.69355	4.24690	2.90347	2.73739	3.18146	2.89801	2.37887	2.77519	2.98518	4.58477	3.61503	
	0.01639	5.01174	4.64667	0.61958	0.77255	0.25638	1.48655														
175	3.01912	5.59599	2.38155	2.67451	4.92971	1.13517	3.74885	4.41456	2.66681	3.89633	4.64421	2.81034	4.23195	2.88569	3.13484	2.86178	2.80844	3.98141	6.03215	4.61618	302 g - - -
	2.68618	4.42225	2.77519	2.73123	3.46354	2.40513	3.72494	3.29354	2.67741	2.69355	4.24690	2.90347	2.73739	3.18146	2.89801	2.37887	2.77519	2.98518	4.58477	3.61503	

	0.00767	5.27000	5.99235	0.61958	0.77255	0.54796	0.86305															
176	2.38751	5.40075	2.85717	2.48612	3.84989	2.53783	3.82105	3.30502	1.94915	2.32385	4.45296	3.04615	4.18343	2.76001	2.80343	2.73547	3.23052	3.22332	4.62220	4.47659	303 k - - -
	2.68618	4.42225	2.77519	2.73123	3.46354	2.40513	3.72494	3.29354	2.67741	2.69355	4.24690	2.90347	2.73739	3.18146	2.89801	2.37887	2.77519	2.98518	4.58477	3.61503	
	0.00767	5.27000	5.99235	0.61958	0.77255	0.54796	0.86305														
177	2.69976	5.50371	2.54166	1.91615	4.84737	3.51520	3.80371	3.51621	2.35451	3.80839	4.54474	3.05060	3.55854	2.66981	2.58844	2.58435	2.10490	2.90966	5.93724	4.52713	304 e - - -
	2.68618	4.42225	2.77519	2.73123	3.46354	2.40513	3.72494	3.29354	2.67741	2.69355	4.24690	2.90347	2.73739	3.18146	2.89801	2.37887	2.77519	2.98518	4.58477	3.61503	
	0.00767	5.27000	5.99235	0.61958	0.77255	0.47581	0.97122														
178	2.97367	5.53636	2.95133	2.71899	4.87941	1.37997	3.99158	4.36136	2.32106	3.83953	4.58116	2.53334	4.19737	2.92489	2.21306	2.75824	2.94062	3.92668	5.96975	4.56281	305 g - - -
	2.68613	4.42244	2.77508	2.73108	3.46373	2.40488	3.72489	3.29373	2.67747	2.69347	4.24709	2.90366	2.73745	3.18144	2.89808	2.37894	2.77514	2.98512	4.58496	3.61522	
	0.63255	2.52465	0.94501	1.36905	0.29350	0.50375	0.92701														
179	2.46546	5.26461	2.88524	1.41004	4.59102	3.05818	3.78529	4.06146	2.53621	3.56571	4.07732	2.91422	2.83192	2.89144	3.02649	2.78526	2.78025	3.47382	5.71806	4.32001	313 e - - -
	2.68618	4.42225	2.77519	2.73123	3.46354	2.40513	3.72494	3.29354	2.67741	2.69355	4.24690	2.90347	2.73739	3.18146	2.89801	2.37887	2.77519	2.98518	4.58477	3.61503	
	0.01237	4.79429	5.51663	0.61958	0.77255	0.61243	0.78095														
180	2.12491	5.43988	2.17689	1.77826	4.78414	2.97987	3.60380	4.27159	2.17194	3.74482	4.48166	2.69609	3.52421	2.83143	3.11761	2.72034	3.15709	3.83026	5.87207	4.45823	314 e - - -
	2.68618	4.42225	2.77519	2.73123	3.46354	2.40513	3.72494	3.29354	2.67741	2.69355	4.24690	2.90347	2.73739	3.18146	2.89801	2.37887	2.77519	2.98518	4.58477	3.61503	
	0.00941	5.06695	5.78929	0.61958	0.77255	1.04136	0.43537														
181	1.87839	5.41699	2.37269	1.81759	4.75976	3.19042	3.87068	4.24467	2.48953	3.42644	4.45857	2.69718	4.07589	2.74756	3.02921	2.56913	2.73598	3.80645	5.85082	4.44031	315 e - - -
	2.68618	4.42225	2.77519	2.73123	3.46354	2.40513	3.72494	3.29354	2.67741	2.69355	4.24690	2.90347	2.73739	3.18146	2.89801	2.37887	2.77519	2.98518	4.58477	3.61503	
	0.00941	5.06695	5.78929	0.61958	0.77255	1.04136	0.43537														
182	1.97650	3.67005	4.86478	4.25573	3.08313	4.13188	4.46567	2.48380	4.05668	1.92095	3.16248	4.27280	4.09240	4.20121	4.08933	3.44153	2.54950	1.38861	4.97776	3.79999	316 v - - -
	2.68614	4.42233	2.77528	2.73118	3.46362	2.40512	3.72503	3.29362	2.67749	2.69363	4.24698	2.90343	2.73732	3.18133	2.89797	2.37895	2.77528	2.98475	4.58485	3.61511	
	0.10883	2.30204	5.78929	0.67362	0.71307	0.33160	1.26506														
183	2.73982	5.05687	3.39534	2.65210	3.17797	3.67788	2.54911	3.65168	2.54811	2.64633	2.86618	2.78480	4.26131	3.27788	2.76402	2.89829	3.23718	3.20461	2.19004	3.71113	319 w - - -
	2.68618	4.42225	2.77519	2.73123	3.46354	2.40513	3.72494	3.29354	2.67741	2.69355	4.24690	2.90347	2.73739	3.18146	2.89801	2.37887	2.77519	2.98518	4.58477	3.61503	
	0.00776	5.25831	5.98065	0.61958	0.77255	0.51414	0.91134														
184	2.63307	5.47925	2.71361	2.54660	4.80599	1.31612	3.98313	4.27931	2.64060	3.77778	4.52769	2.68822	3.84078	3.08706	3.22095	2.39790	2.93896	3.86062	4.21715	4.52477	320 g - - -
	2.68539	4.42262	2.77492	2.73150	3.46391	2.40450	3.72342	3.29391	2.67778	2.69392	4.24681	2.90302	2.73742	3.18171	2.89825	2.37912	2.77549	2.98555	4.58431	3.61540	
	0.31973	1.43680	3.32512	1.03893	0.43670	0.45083	1.01363														
185	2.60327	4.10789	3.24676	2.46209	4.81550	3.76662	3.78633	4.29523	1.82656	3.78058	4.52209	3.09161	3.74888	2.54706	2.75628	2.00769	2.00202	3.52900	5.91692	4.51150	324 k - - -
	2.68618	4.42225	2.77519	2.73123	3.46354	2.40513	3.72494	3.29354	2.67741	2.69355	4.24690	2.90347	2.73739	3.18146	2.89801	2.37887	2.77519	2.98518	4.58477	3.61503	
	0.00780	5.25374	5.97608	0.61958	0.77255	0.60246	0.79289														
186	2.44445	5.46735	3.15808	2.63600	4.79874	3.76958	3.71331	3.94423	2.18822	3.14441	4.51171	3.08824	2.69144	2.70310	1.54442	2.82907	2.86314	3.51050	5.90809	4.50609	325 r - - -
	2.68602	4.42237	2.77504	2.73123	3.46366	2.40525	3.72435	3.29366	2.67727	2.69367	4.24702	2.90327	2.73744	3.18159	2.89797	2.37894	2.77532	2.98514	4.58489	3.61515	
	0.06915	2.74454	5.97608	1.19145	0.36209	0.41915	1.07179														
187	1.34643	5.41113	2.93556	2.68309	4.71241	2.64932	3.79615	4.16845	2.75393	3.20995	4.46483	3.28985	3.35611	2.90250	3.23837	2.03474	3.24497	3.77828	5.87210	3.95555	330 a - - -
	2.68618	4.42225	2.77519	2.73123	3.46354	2.40513	3.72494	3.29354	2.67741	2.69355	4.24690	2.90347	2.73739	3.18146	2.89801	2.37887	2.77519	2.98518	4.58477	3.61503	
	0.00754	5.28723	6.00957	0.61958	0.77255	0.48576	0.95510														
188	2.53155	5.51621	2.46178	2.34374	4.86138	3.32684	3.27756	4.17109	2.44860	3.82152	4.55688	2.76124	1.97526	3.00558	2.09214	2.75656	3.14455	3.69642	5.94879	4.53775	331 p - - -
	2.68618	4.42225	2.77519	2.73123	3.46354	2.40513	3.72494	3.29354	2.67741	2.69355	4.24690	2.90347	2.73739	3.18146	2.89801	2.37887	2.77519	2.98518	4.58477	3.61503	
	0.00754	5.28723	6.00957	0.61958	0.77255	0.48576	0.95510														
189	3.84669	5.74868	5.61428	5.41456	2.80816	5.01157	4.57206	4.43938	5.11919	3.77928	5.04786	5.04757	5.52877	5.19513	5.11773	4.08426	4.68042	4.30797	0.31245	2.99859	332 W - - -
	2.68618	4.42225	2.77519	2.73123	3.46354	2.40513	3.72494	3.29354	2.67741	2.69355	4.24690	2.90347	2.73739	3.18146	2.89801	2.37887	2.77519	2.98518	4.58477	3.61503	
	0.00754	5.28723	6.00957	0.61958	0.77255	0.48576	0.95510														
190	3.06055	2.35422	5.12844	4.52230	3.64062	4.36044	4.70895	2.16507	4.31769	1.60459	2.78133	3.75536	4.71657	4.45570	4.33599	3.67657	3.31190	1.34660	5.19932	4.02185	333 v - - -
	2.68618	4.42225	2.77519	2.73123	3.46354	2.40513	3.72494	3.29354	2.67741	2.69355	4.24690	2.90347	2.73739	3.18146	2.89801	2.37887	2.77519	2.98518	4.58477	3.61503	
	0.00754	5.28723	6.00957	0.61958	0.77255	0.48576	0.95510														
191	1.74910	5.39053	1.87209	2.67294	4.68572	3.09599	3.99668	3.37978	2.51830	3.08599	3.78560	2.60164	4.19565	3.11204	3.10013	2.84283	3.00870	3.33746	5.85428	4.01644	334 a - - -
	2.68618	4.42225	2.77519	2.73123	3.46354	2.40513	3.72494	3.29354	2.67741	2.69355	4.24690	2.90347	2.73739	3.18146	2.89801	2.37887	2.77519	2.98518	4.58477	3.61503	
	0.00754	5.28723	6.00957	0.61958	0.77255	0.48576	0.95510														
192	2.68337	4.17347	5.03059	3.85186	2.27509	4.23265	4.56626	2.32490	4.19892	1.59061	2.71895	4.39642	4.59607	4.32909	4.20553	3.54415	3.29493	2.15950	3.67187	2.11227	335 l - - -
	2.68618	4.42225	2.77519	2.73123	3.46354	2.40513	3.72494	3.29354	2.67741	2.69355	4.24690	2.90347	2.73739	3.18146	2.89801	2.37887	2.77519	2.98518	4.58477	3.61503	
	0.00754	5.28723	6.00957	0.61958	0.77255	0.48576	0.95510														
193	2.54773	4.91634	3.07244	2.51597	4.31890	3.47392	3.71176	4.22419	2.42991	3.73590	4.49200	3.17644	4.18511	2.74837	3.01176	1.70412	1.95466	2.84874	5.89433	3.63300	336 s - - -
	2.68618	4.42225	2.77519	2.73123	3.46354	2.40513	3.72494	3.29354	2.67741	2.69355	4.24690	2.90347	2.73739	3.18146	2.89801	2.37887	2.77519	2.98518	4.58477	3.61503	
	0.00754	5.28723	6.00957	0.61958	0.77255	0.48576	0.95510														
194	2.64287	4.93234	3.67030	3.10861	3.41508	1.03818	3.87707	3.47950	3.01118	3.06674	3.72089	3.57540	4.32569	3.16323	3.34827	2.80726	3.06121	3.22500	5.48798	3.76267	337 g - - -
	2.68618	4.42225	2.77519	2.73123	3.46354	2.40513	3.72494	3.29354	2.67741	2.69355	4.24690	2.90347	2.73739	3.18146	2.89801	2.37887	2.77519	2.98518	4.58477	3.61503	
	0.00754	5.28723	6.00957	0.61958	0.77255	0.48576	0.95510														
195	2.87272	5.51287	2.99949	2.11352	4.22470	3.57879	3.66822	4.34214	2.06892	3.67942	4.55381	3.06393	2.37185	2.59212	2.21218	2.74438	2.59595	3.01374	5.94617	4.53586	338 k - - -
	2.68618	4.42225	2.77519	2.73123	3.46354	2.40513	3.72494	3.29354	2.67741	2.69355	4.24690	2.90347	2.73739	3.18146	2.89801	2.37887	2.77519	2.98518	4.58477	3.61503	
	0.04752	5.28723	3.18550	0.61958	0.77255	0.48576	0.95510														
196	2.63610	4.76146	3.73124	2.81449	2.99913	3.77147	2.57031	2.09192	3.05426	2.37342	3.53521	3.55195	4.12131	3.38070	3.12417	2.38863	2.91925	2.26677	5.33977	4.10049	339 i - - -
	2.68618	4.42225	2.77519	2.73123	3.46354	2.40513	3.72494	3.29354	2.67741	2.69355	4.24690	2.90347	2.73739	3.18146	2.89801	2.37887	2.77519	2.98518	4.58477	3.61503	
	0.36987	5.24755	1.19101	0.61958	0.77255	0.62221	0.76950														
197	2.49947	5.31395	2.59991	2.31269	4.03008	2.23705	3.57344	4.12498	2.48953	3.61420	3.87878	2.82254	2.58149	2.79937	2.92067	2.42028	2.80118	3.69824	5.75376	4.34996	340 g - - -
	2.68618	4.42225	2.77519	2.73123	3.46354	2.40513	3.72494	3.29354	2.67741	2.69355	4.24690	2.90347	2.73739	3.18146	2.89801	2.37887	2.77519	2.98518	4.58477	3.61503	
	0.04586	4.88893	3.28902	0.61958	0.77255	0.60377	0.79131														

198	2.63889	5.41638	2.11447	1.98286	3.93351	2.48513	3.60515	4.24545	2.14633	3.72116	4.45736	2.82310	3.55137	2.82068	2.87916	2.87118	2.88911	3.27452	5.84974	4.43944	341 e - - -
	2.68618	4.42225	2.77519	2.73123	3.46354	2.40513	3.72494	3.29354	2.67741	2.69355	4.24690	2.90347	2.73739	3.18146	2.89801	2.37887	2.77519	2.98518	4.58477	3.61503	
	0.00931	5.07686	5.79920	0.61958	0.77255	1.02398	0.44498														
199	2.63177	5.41195	2.35230	2.51241	4.75383	1.82605	3.72148	4.06148	2.46303	3.58091	4.45349	2.72937	3.85748	2.61285	2.80813	2.52728	2.64336	3.65460	5.84652	4.43744	342 g - - -
	2.68618	4.42225	2.77519	2.73123	3.46354	2.40513	3.72494	3.29354	2.67741	2.69355	4.24690	2.90347	2.73739	3.18146	2.89801	2.37887	2.77519	2.98518	4.58477	3.61503	
	0.03234	5.07686	3.66587	0.61958	0.77255	1.02398	0.44498														
200	2.63249	5.40588	2.73275	1.86107	4.36303	3.10257	3.85959	4.23480	2.06750	3.71063	4.17875	2.79094	3.11008	2.44027	2.70936	2.65419	2.92972	3.52700	4.20286	4.15032	343 e - - -
	2.68618	4.42225	2.77519	2.73123	3.46354	2.40513	3.72494	3.29354	2.67741	2.69355	4.24690	2.90347	2.73739	3.18146	2.89801	2.37887	2.77519	2.98518	4.58477	3.61503	
	0.22562	5.05405	1.63170	0.61958	0.77255	0.37095	1.17143														
201	2.35806	5.38401	1.99933	2.56307	4.71608	2.76645	3.87198	3.58197	2.29928	3.58221	4.42818	2.78920	3.14049	2.97264	2.94454	2.46790	2.57421	3.13988	5.82463	4.42166	344 d - - -
	2.68480	4.42306	2.77519	2.73125	3.46453	2.40455	3.72466	3.29420	2.67783	2.69415	4.24753	2.90419	2.73610	3.18160	2.89832	2.37937	2.77444	2.98570	4.58262	3.61592	
	0.58725	0.97514	2.70298	1.83051	0.17475	0.24986	1.50918														
202	2.13494	4.26480	4.24245	2.95160	3.51731	2.82177	3.82429	2.43974	3.57963	2.66789	3.46199	3.63141	3.81606	3.83596	3.83559	2.22600	2.99980	2.11756	3.13316	2.79864	371 v - - -
	2.68618	4.42225	2.77519	2.73123	3.46354	2.40513	3.72494	3.29354	2.67741	2.69355	4.24690	2.90347	2.73739	3.18146	2.89801	2.37887	2.77519	2.98518	4.58477	3.61503	
	0.00795	5.23451	5.95686	0.61958	0.77255	0.38939	1.13155														
203	2.75728	4.13644	4.02552	3.52441	4.84625	1.32599	3.99050	4.26827	3.51340	3.90675	4.72084	3.42507	4.44929	3.83386	3.91937	2.04012	1.34161	3.82952	6.15642	4.88761	372 g - - -
	2.68618	4.42225	2.77519	2.73123	3.46354	2.40513	3.72494	3.29354	2.67741	2.69355	4.24690	2.90347	2.73739	3.18146	2.89801	2.37887	2.77519	2.98518	4.58477	3.61503	
	0.00754	5.28723	6.00957	0.61958	0.77255	0.48576	0.95510														
204	3.35446	5.16903	6.04963	5.54224	4.30536	5.49136	6.07026	1.18076	5.44804	1.23455	3.81643	5.65795	5.69986	5.59738	5.53110	4.89396	4.20316	1.32938	6.34741	5.19958	373 i - - -
	2.68618	4.42225	2.77519	2.73123	3.46354	2.40513	3.72494	3.29354	2.67741	2.69355	4.24690	2.90347	2.73739	3.18146	2.89801	2.37887	2.77519	2.98518	4.58477	3.61503	
	0.00754	5.28723	6.00957	0.61958	0.77255	0.48576	0.95510														
205	1.72505	3.50055	4.95948	4.07694	3.59708	4.27109	4.60716	2.67441	4.16790	1.79534	3.42691	4.39656	4.63397	4.32285	4.21905	3.57992	2.25465	1.48950	5.13438	3.95477	374 v - - -
	2.68618	4.42225	2.77519	2.73123	3.46354	2.40513	3.72494	3.29354	2.67741	2.69355	4.24690	2.90347	2.73739	3.18146	2.89801	2.37887	2.77519	2.98518	4.58477	3.61503	
	0.00754	5.28723	6.00957	0.61958	0.77255	0.48576	0.95510														
206	3.04241	4.47952	5.07002	4.45577	1.72274	4.28016	3.94725	1.56392	4.24200	1.89981	2.57659	4.44166	4.63830	4.36788	4.24897	3.59250	3.33956	2.28989	5.09177	3.14226	375 i - - -
	2.68618	4.42225	2.77519	2.73123	3.46354	2.40513	3.72494	3.29354	2.67741	2.69355	4.24690	2.90347	2.73739	3.18146	2.89801	2.37887	2.77519	2.98518	4.58477	3.61503	
	0.00754	5.28723	6.00957	0.61958	0.77255	0.48576	0.95510														
207	2.24848	4.44070	5.00446	4.06030	2.52880	4.23008	4.56264	2.29513	4.18063	1.44374	2.34802	4.38525	4.59377	4.31595	4.19665	3.07196	3.03475	2.05738	5.06263	3.61884	376 l - - -
	2.68618	4.42225	2.77519	2.73123	3.46354	2.40513	3.72494	3.29354	2.67741	2.69355	4.24690	2.90347	2.73739	3.18146	2.89801	2.37887	2.77519	2.98518	4.58477	3.61503	
	0.00754	5.28723	6.00957	0.61958	0.77255	0.48576	0.95510														
208	2.76220	4.85220	1.44308	2.54962	4.85765	2.63407	3.18432	4.34263	2.70398	3.81881	4.55541	2.75217	3.55815	2.45910	3.10490	2.56419	3.13562	3.90399	5.94782	4.16618	377 d - - -
	2.68618	4.42225	2.77519	2.73123	3.46354	2.40513	3.72494	3.29354	2.67741	2.69355	4.24690	2.90347	2.73739	3.18146	2.89801	2.37887	2.77519	2.98518	4.58477	3.61503	
	0.00754	5.28723	6.00957	0.61958	0.77255	0.48576	0.95510														
209	2.33208	5.45768	2.62030	2.59577	4.12258	3.39907	1.50820	4.24771	2.72458	3.14470	4.50481	2.78995	3.71397	3.08436	2.98348	2.59728	3.09371	3.83522	5.90516	3.91004	378 h - - -
	2.68618	4.42225	2.77519	2.73123	3.46354	2.40513	3.72494	3.29354	2.67741	2.69355	4.24690	2.90347	2.73739	3.18146	2.89801	2.37887	2.77519	2.98518	4.58477	3.61503	
	0.00754	5.28723	6.00957	0.61958	0.77255	0.48576	0.95510														
210	2.90360	5.56162	3.08146	2.61136	4.88761	2.76127	4.07928	4.35840	2.67294	3.86703	4.63028	3.20589	1.07519	3.01079	3.34907	2.77098	2.78837	3.94679	6.02487	4.62217	379 p - - -
	2.68618	4.42225	2.77519	2.73123	3.46354	2.40513	3.72494	3.29354	2.67741	2.69355	4.24690	2.90347	2.73739	3.18146	2.89801	2.37887	2.77519	2.98518	4.58477	3.61503	
	0.04503	5.28723	3.24473	0.61958	0.77255	0.48576	0.95510														
211	2.47761	5.49777	2.44262	2.02568	4.84270	2.92325	3.94813	4.32861	2.30956	3.62649	4.53851	2.97098	3.39189	2.68147	3.17587	1.91273	2.60161	3.44519	5.93053	4.51958	380 s - - -
	2.68618	4.42225	2.77519	2.73123	3.46354	2.40513	3.72494	3.29354	2.67741	2.69355	4.24690	2.90347	2.73739	3.18146	2.89801	2.37887	2.77519	2.98518	4.58477	3.61503	
	0.01612	5.25002	4.53340	0.61958	0.77255	0.43158	1.04835														
212	2.72581	5.48366	3.08509	2.55854	4.80774	3.12544	3.81811	4.27985	2.60708	3.47373	4.53240	1.05572	3.79051	3.09619	3.23072	2.72644	3.19431	3.55255	5.93027	4.53049	381 n - - -
	2.68625	4.42237	2.77472	2.73118	3.46366	2.40500	3.72441	3.29366	2.67742	2.69356	4.24702	2.90359	2.73752	3.18151	2.89813	2.37891	2.77519	2.98531	4.58489	3.61515	
	0.31741	2.71594	1.58074	1.15451	0.37864	0.52837	0.89053														
213	2.56921	4.84332	3.23920	2.80257	2.76027	3.16344	3.78524	3.24316	2.73711	2.40277	3.93382	3.43120	1.97458	3.26536	2.82834	3.03292	2.92165	2.64420	5.39386	3.59379	388 p - - -
	2.68618	4.42225	2.77519	2.73123	3.46354	2.40513	3.72494	3.29354	2.67741	2.69355	4.24690	2.90347	2.73739	3.18146	2.89801	2.37887	2.77519	2.98518	4.58477	3.61503	
	0.03805	5.04825	3.47644	0.61958	0.77255	0.88056	0.53537														
214	2.61711	5.31141	2.83562	2.55986	3.75539	2.37351	3.88726	3.70395	2.43621	3.35208	4.11398	2.00232	4.08777	2.99865	2.31135	2.75078	2.71907	3.54879	4.28939	4.38463	389 n - - -
	2.68618	4.42225	2.77519	2.73123	3.46354	2.40513	3.72494	3.29354	2.67741	2.69355	4.24690	2.90347	2.73739	3.18146	2.89801	2.37887	2.77519	2.98518	4.58477	3.61503	
	0.00943	5.06441	5.78676	0.61958	0.77255	0.93991	0.49538														
215	2.68784	4.61252	3.89848	3.32881	2.59121	3.65523	2.07646	3.10013	3.27258	2.65175	3.71842	3.71066	4.31690	3.56595	2.69333	2.93311	2.92088	2.69444	5.19623	1.91011	390 y - - -
	2.68618	4.42225	2.77519	2.73123	3.46354	2.40513	3.72494	3.29354	2.67741	2.69355	4.24690	2.90347	2.73739	3.18146	2.89801	2.37887	2.77519	2.98518	4.58477	3.61503	
	0.00922	5.08699	5.80934	0.61958	0.77255	0.55617	0.85191														
216	3.00265	5.84906	2.97567	2.47935	5.21406	3.94832	4.37550	4.67302	3.20416	4.20046	5.02974	3.45772	0.65420	3.31933	3.27727	3.42468	3.74614	4.28066	6.37505	4.97542	391 P - - -
	2.68625	4.42238	2.77532	2.73101	3.46366	2.40510	3.72507	3.29367	2.67715	2.69367	4.24702	2.90359	2.73725	3.18146	2.89804	2.37889	2.77518	2.98521	4.58490	3.61478	
	0.36087	2.15521	1.67636	0.79267	0.60264	0.34067	1.24234														
217	2.45304	4.60418	2.21638	2.64691	4.66452	3.25856	3.89889	3.59627	2.64753	3.52444	4.40094	3.11361	3.41436	2.61026	2.80100	2.31006	1.83655	3.48623	5.80414	4.41351	395 t - - -
	2.68618	4.42225	2.77519	2.73123	3.46354	2.40513	3.72494	3.29354	2.67741	2.69355	4.24690	2.90347	2.73739	3.18146	2.89801	2.37887	2.77519	2.98518	4.58477	3.61503	
	0.00907	5.10326	5.82561	0.61958	0.77255	0.97525	0.47336														
218	2.69680	5.21790	3.27242	2.67695	3.83252	3.38967	2.71778	3.91682	2.59607	3.48849	4.28048	3.05987	2.35053	2.78435	2.30972	2.93453	2.87823	3.35125	4.88421	2.25420	396 y - - -
	2.68618	4.42225	2.77519	2.73123	3.46354	2.40513	3.72494	3.29354	2.67741	2.69355	4.24690	2.90347	2.73739	3.18146	2.89801	2.37887	2.77519	2.98518	4.58477	3.61503	
	0.00907	5.10326	5.82561	0.61958	0.77255	0.26898	1.44459														
219	3.04465	4.91709	3.49512	2.48470	3.05673	3.96281	4.18865	3.45565	3.12383	2.97446	4.01190	3.60915	4.34725	2.78325	3.52691	2.92479	3.15168	3.20693	1.17590	3.55097	397 w - - -
	2.68618	4.42225	2.77519	2.73123	3.46354	2.40513	3.72494	3.29354	2.67741	2.69355	4.24690	2.90347	2.73739	3.18146	2.89801	2.37887	2.77519	2.98518	4.58477	3.61503	
	0.01991	5.28723	4.22291	0.61958	0.77255	0.48576	0.95510														
220	2.87116	4.27532	4.31066	3.73132	1.69865	4.10999	1.87010	2.71512	3.41769	2.67779	3.69163	4.01242	2.66729	3.67761	3.88207	2.87978	3.27440	2.75143	5.18282	3.01313	398 f - - -

```
        2.68618  4.42225  2.77519  2.73123  3.46354  2.40513  3.72494  3.29354  2.67741  2.69355  4.24690  2.90347  2.73739  3.18146  2.89801  2.37887  2.77519  2.98518  4.58477  3.61503
        0.00763  5.27495  5.99730  0.61958  0.77255  0.53059  0.88735
  221   2.00097  4.47021  4.80385  3.41129  3.56839  4.20469  4.52728  2.57389  4.03436  1.90313  3.34758  4.29163  4.57022  4.20682  4.12038  3.29747  2.74897  1.24295  5.09253  3.53370    399 v - - -
        2.68618  4.42225  2.77519  2.73123  3.46354  2.40513  3.72494  3.29354  2.67741  2.69355  4.24690  2.90347  2.73739  3.18146  2.89801  2.37887  2.77519  2.98518  4.58477  3.61503
        0.01801  5.27495  4.36410  0.61958  0.77255  0.45871  0.99993
  222   4.09056  6.10011  3.71131  3.77851  5.12253  4.56395  4.56980  4.99810  2.86883  4.39725  5.32732  4.15958  4.99700  3.76315  0.32932  4.08373  4.27512  4.68913  6.18959  3.94753    400 R - - -
        2.68618  4.42225  2.77519  2.73123  3.46354  2.40513  3.72494  3.29354  2.67741  2.69355  4.24690  2.90347  2.73739  3.18146  2.89801  2.37887  2.77519  2.98518  4.58477  3.61503
        0.02039  5.27706  4.19465  0.61958  0.77255  0.52307  0.89819
  223   2.53990  5.43237  2.55439  2.56283  4.74767  2.32809  3.74943  3.40438  2.50183  2.95933  4.48097  2.81456  3.75309  2.94514  3.20650  2.41228  2.22734  2.71788  5.88330  4.49104    401 t - - -
        2.68618  4.42225  2.77519  2.73123  3.46354  2.40513  3.72494  3.29354  2.67741  2.69355  4.24690  2.90347  2.73739  3.18146  2.89801  2.37887  2.77519  2.98518  4.58477  3.61503
        0.00771  5.26438  5.98673  0.61958  0.77255  0.56722  0.83724
  224   2.58096  5.43022  2.55416  2.21121  4.74459  3.43213  2.93016  4.20947  2.61594  3.72232  4.47908  3.26225  4.17464  3.02256  3.00589  2.58414  2.86860  3.52757  3.11589  1.85213    402 y - - -
        2.68618  4.42225  2.77519  2.73123  3.46354  2.40513  3.72494  3.29354  2.67741  2.69355  4.24690  2.90347  2.73739  3.18146  2.89801  2.37887  2.77519  2.98518  4.58477  3.61503
        0.00771  5.26438  5.98673  0.61958  0.77255  0.56722  0.83724
  225   2.76135  5.47080  2.87694  2.33102  4.79252  1.50417  3.82929  4.10211  2.73692  3.76697  3.55089  2.77006  2.14588  3.09247  3.22734  2.94341  3.18165  3.84939  5.91932  4.52108    403 g - - -
        2.68618  4.42225  2.77519  2.73123  3.46354  2.40513  3.72494  3.29354  2.67741  2.69355  4.24690  2.90347  2.73739  3.18146  2.89801  2.37887  2.77519  2.98518  4.58477  3.61503
        0.01991  5.26439  4.23112  0.61958  0.77255  0.56722  0.83724
  226   2.95490  4.84055  3.20290  2.78822  2.56555  3.95063  3.98073  2.97161  3.05979  1.80093  3.93768  3.16188  4.09590  3.11834  2.90747  2.82836  2.96369  3.12437  3.40145  2.35987    404 l - - -
        2.68618  4.42225  2.77519  2.73123  3.46354  2.40513  3.72494  3.29354  2.67741  2.69355  4.24690  2.90347  2.73739  3.18146  2.89801  2.37887  2.77519  2.98518  4.58477  3.61503
        0.00781  5.25229  5.97463  0.61958  0.77255  0.60713  0.78727
  227   2.45658  4.70060  3.39099  3.27819  1.75562  3.64215  4.27767  3.10554  3.09532  2.56499  2.37982  3.48407  2.29150  3.50125  3.68122  3.22178  3.15674  2.66628  5.28785  4.06102    405 f - - -
        2.68618  4.42225  2.77519  2.73123  3.46354  2.40513  3.72494  3.29354  2.67741  2.69355  4.24690  2.90347  2.73739  3.18146  2.89801  2.37887  2.77519  2.98518  4.58477  3.61503
        0.00781  5.25229  5.97463  0.61958  0.77255  0.60713  0.78727
  228   1.72099  3.58676  3.47139  2.77599  4.31292  1.86783  4.06963  3.56569  2.90109  3.21757  3.82670  3.30236  3.08911  2.80532  3.35099  2.52636  2.94849  3.15149  5.62380  4.31191    406 a - - -
        2.68618  4.42225  2.77519  2.73123  3.46354  2.40513  3.72494  3.29354  2.67741  2.69355  4.24690  2.90347  2.73739  3.18146  2.89801  2.37887  2.77519  2.98518  4.58477  3.61503
        0.12678  5.25229  2.17295  0.61958  0.77255  0.60713  0.78727
  229   1.61612  4.50403  4.23673  3.49875  3.50569  4.03519  4.32353  2.48941  3.56625  2.36125  3.61643  3.01291  3.39740  3.81601  3.80828  2.89189  2.89633  1.71820  5.10896  3.53092    407 a - - -
        2.68618  4.42225  2.77519  2.73123  3.46354  2.40513  3.72494  3.29354  2.67741  2.69355  4.24690  2.90347  2.73739  3.18146  2.89801  2.37887  2.77519  2.98518  4.58477  3.61503
        0.08556  5.13429  2.57555  0.61958  0.77255  0.91295  0.51306
  230   2.51867  3.07627  3.22408  2.77983  3.44586  2.25213  3.95821  3.70106  2.65192  3.31834  4.13993  1.71780  4.13932  3.01379  3.22990  2.66876  2.91424  3.39004  5.57697  3.77988    408 n - - -
        2.68618  4.42225  2.77519  2.73123  3.46354  2.40513  3.72494  3.29354  2.67741  2.69355  4.24690  2.90347  2.73739  3.18146  2.89801  2.37887  2.77519  2.98518  4.58477  3.61503
        0.00949  5.05822  5.78057  0.61958  0.77255  0.48634  0.95418
  231   2.86184  4.63303  4.03187  3.46018  2.55815  3.36927  4.28420  3.02112  3.11497  2.46969  3.74069  3.63617  1.54510  3.68062  3.29401  2.70018  3.08100  2.47730  5.22603  2.94853    409 p - - -
        2.68661  4.42299  2.77537  2.73180  3.45830  2.40432  3.72467  3.29384  2.67782  2.69295  4.24372  2.90434  2.73821  3.18216  2.89787  2.37952  2.77629  2.98467  4.58392  3.61456
        1.85310  0.74763  0.99490  1.49500  0.25392  0.56219  0.84388
  232   2.79960  3.92997  2.74194  2.35630  3.81901  3.59887  3.79337  3.89254  2.50486  3.23893  4.21492  2.09250  3.99081  2.77235  3.03673  1.94249  2.62703  3.35888  5.62756  3.64807    416 s - - -
        2.68618  4.42248  2.77533  2.73146  3.46189  2.40536  3.72491  3.29355  2.67744  2.69358  4.24643  2.90276  2.73727  3.18169  2.89824  2.37910  2.77543  2.98510  4.58500  3.61490
        1.19448  0.36651  5.52489  0.05239  2.97517  0.26227  1.46665
  233   1.71780  4.03086  2.88116  2.77411  4.56125  2.32674  3.99637  3.99678  2.62571  3.56469  4.35419  3.05545  4.18903  3.03903  3.24649  1.87749  2.74351  3.36827  4.34166  4.41442    418 a - - -
        2.68618  4.42225  2.77519  2.73123  3.46354  2.40513  3.72494  3.29354  2.67741  2.69355  4.24690  2.90347  2.73739  3.18146  2.89801  2.37887  2.77519  2.98518  4.58477  3.61503
        0.00792  5.23844  5.96079  0.61958  0.77255  0.65037  0.73784
  234   2.59795  4.75403  3.46546  3.28438  2.97299  3.97970  4.22952  3.09476  2.34637  1.86867  3.55742  3.70036  2.01713  3.11971  3.38645  3.07200  3.05583  2.86410  3.36014  3.95991    419 l - - -
        2.68618  4.42225  2.77519  2.73123  3.46354  2.40513  3.72494  3.29354  2.67741  2.69355  4.24690  2.90347  2.73739  3.18146  2.89801  2.37887  2.77519  2.98518  4.58477  3.61503
        0.00792  5.23844  5.96079  0.61958  0.77255  0.65037  0.73784
  235   1.60123  4.34976  3.12029  2.30367  3.43564  3.83799  3.61400  3.37991  2.76370  2.93973  3.82655  3.26614  3.06898  3.06657  3.32034  2.74784  2.39333  3.00650  5.64722  4.32593    420 a - - -
        2.68618  4.42225  2.77519  2.73123  3.46354  2.40513  3.72494  3.29354  2.67741  2.69355  4.24690  2.90347  2.73739  3.18146  2.89801  2.37887  2.77519  2.98518  4.58477  3.61503
        0.13321  5.23844  2.12520  0.61958  0.77255  0.65037  0.73784
  236   2.68088  5.20881  3.04687  2.44530  2.15192  3.01037  3.63586  3.90062  2.42339  3.15544  4.27265  3.16692  3.30288  2.73995  2.71019  2.87799  3.16079  3.09055  2.92093  3.63048    421 f - - -
        2.68618  4.42225  2.77519  2.73123  3.46354  2.40513  3.72494  3.29354  2.67741  2.69355  4.24690  2.90347  2.73739  3.18146  2.89801  2.37887  2.77519  2.98518  4.58477  3.61503
        0.00897  5.11421  5.83655  0.61958  0.77255  0.43535  1.04140
  237   2.22351  5.48811  2.05963  2.07461  3.87602  3.68184  2.94578  4.31640  2.47803  3.29596  4.23549  2.62854  3.94087  2.80558  2.91418  2.39761  3.01842  3.79090  5.92177  4.14188    422 d - - -
        2.68618  4.42225  2.77519  2.73123  3.46354  2.40513  3.72494  3.29354  2.67741  2.69355  4.24690  2.90347  2.73739  3.18146  2.89801  2.37887  2.77519  2.98518  4.58477  3.61503
        0.00792  5.23844  5.96079  0.61958  0.77255  0.65037  0.73784
  238   2.21580  5.48546  2.98290  1.91137  4.82774  2.53145  3.51269  4.31172  2.08889  2.80060  4.12700  3.06223  4.14969  2.89471  2.41998  2.85287  2.91938  3.70314  5.91980  4.51058    423 e - - -
        2.68618  4.42225  2.77519  2.73123  3.46354  2.40513  3.72494  3.29354  2.67741  2.69355  4.24690  2.90347  2.73739  3.18146  2.89801  2.37887  2.77519  2.98518  4.58477  3.61503
        0.00792  5.23844  5.96079  0.61958  0.77255  0.39508  1.11971
  239   2.59826  5.51790  2.78275  1.85983  4.86241  3.23439  3.96841  4.34821  2.59573  3.82296  4.55888  2.44637  1.86618  2.98669  2.77152  2.64766  2.91327  3.67290  5.95084  4.53983    424 e - - -
        2.68618  4.42225  2.77519  2.73123  3.46354  2.40513  3.72494  3.29354  2.67741  2.69355  4.24690  2.90347  2.73739  3.18146  2.89801  2.37887  2.77519  2.98518  4.58477  3.61503
        0.00754  5.28723  6.00957  0.61958  0.77255  0.48576  0.95510
  240   2.55053  4.44257  4.97437  4.36243  2.12590  4.22406  3.67431  2.49739  4.15817  1.65327  3.42428  4.37011  4.58823  4.29871  2.99729  3.53319  2.70843  2.16669  2.91814  2.83875    425 l - - -
        2.68618  4.42225  2.77519  2.73123  3.46354  2.40513  3.72494  3.29354  2.67741  2.69355  4.24690  2.90347  2.73739  3.18146  2.89801  2.37887  2.77519  2.98518  4.58477  3.61503
        0.00754  5.28723  6.00957  0.61958  0.77255  0.48576  0.95510
  241   2.76685  5.45532  2.81779  2.16774  4.77535  3.62493  3.71206  3.85916  2.42038  2.90646  4.01901  3.26774  2.90947  2.73206  2.90780  2.82508  1.86012  2.63834  5.90329  4.19166    426 t - - -
        2.68618  4.42225  2.77519  2.73123  3.46354  2.40513  3.72494  3.29354  2.67741  2.69355  4.24690  2.90347  2.73739  3.18146  2.89801  2.37887  2.77519  2.98518  4.58477  3.61503
        0.00754  5.28723  6.00957  0.61958  0.77255  0.48576  0.95510
  242   3.17743  4.87502  5.52058  4.91682  3.01778  4.77845  5.11492  1.52431  4.72289  0.88347  3.29555  4.94247  5.06774  4.79373  4.71797  4.10685  3.77408  2.26204  5.48982  3.88089    427 l - - -
        2.68618  4.42225  2.77519  2.73123  3.46354  2.40513  3.72494  3.29354  2.67741  2.69355  4.24690  2.90347  2.73739  3.18146  2.89801  2.37887  2.77519  2.98518  4.58477  3.61503
```

	0.00754	5.28723	6.00957	0.61958	0.77255	0.48576	0.95510														
243	2.03736	5.51408	2.73260	1.94199	4.85855	3.26099	3.81365	4.34411	2.09690	3.39215	4.55490	2.98317	2.31587	2.81477	3.10877	2.76190	3.03347	3.74689	5.94711	4.04176	428 e - - -
	2.68609	4.42227	2.77522	2.73125	3.46356	2.40507	3.72497	3.29356	2.67743	2.69357	4.24692	2.90349	2.73742	3.18148	2.89789	2.37889	2.77522	2.98520	4.58479	3.61505	
	0.02498	3.98865	5.09265	0.80074	0.59602	0.48576	0.95510														
244	2.06524	5.47765	3.27370	2.59979	4.80690	3.78809	3.97566	4.28060	2.04627	3.77549	4.52297	3.17725	1.68018	2.61222	2.78428	2.79235	2.83817	3.49781	5.91941	4.23218	432 p - - -
	2.68618	4.42225	2.77519	2.73123	3.46354	2.40513	3.72494	3.29354	2.67741	2.69355	4.24690	2.90347	2.73739	3.18146	2.89801	2.37887	2.77519	2.98518	4.58477	3.61503	
	0.00757	5.28353	6.00588	0.61958	0.77255	0.49952	0.93349														
245	2.76322	5.68659	2.36643	2.70351	5.02625	1.00849	4.14112	4.51115	2.71134	4.00198	4.76345	2.99855	4.31210	3.25579	3.33252	2.58731	3.20352	4.08161	6.14754	4.72533	433 g - - -
	2.68618	4.42225	2.77519	2.73123	3.46354	2.40513	3.72494	3.29354	2.67741	2.69355	4.24690	2.90347	2.73739	3.18146	2.89801	2.37887	2.77519	2.98518	4.58477	3.61503	
	0.00757	5.28353	6.00588	0.61958	0.77255	0.47725	0.96886														
246	2.71807	5.51980	2.75012	1.57672	4.86587	3.21363	3.35158	4.35252	2.03859	3.82541	4.56031	3.24614	4.17307	2.15979	2.65071	2.74103	2.76392	3.91100	5.95174	4.54017	434 e - - -
	2.68618	4.42225	2.77519	2.73123	3.46354	2.40513	3.72494	3.29354	2.67741	2.69355	4.24690	2.90347	2.73739	3.18146	2.89801	2.37887	2.77519	2.98518	4.58477	3.61503	
	0.00754	5.28723	6.00957	0.61958	0.77255	0.48576	0.95510														
247	2.49310	5.47946	3.13283	2.49950	4.80919	3.69129	3.74613	4.28445	2.50528	3.77817	4.52422	3.25910	2.17223	2.89380	2.91875	1.87740	2.00939	3.33375	5.92143	4.23362	435 s - - -
	2.68618	4.42225	2.77519	2.73123	3.46354	2.40513	3.72494	3.29354	2.67741	2.69355	4.24690	2.90347	2.73739	3.18146	2.89801	2.37887	2.77519	2.98518	4.58477	3.61503	
	0.00754	5.28723	6.00957	0.61958	0.77255	0.48576	0.95510														
248	2.76053	4.44974	4.92492	3.89826	2.96346	4.21778	4.19848	2.34380	3.88119	1.20654	3.39089	4.34737	4.58246	4.27201	3.43911	3.38004	2.97249	2.05143	5.06996	2.55931	436 l - - -
	2.68618	4.42225	2.77519	2.73123	3.46354	2.40513	3.72494	3.29354	2.67741	2.69355	4.24690	2.90347	2.73739	3.18146	2.89801	2.37887	2.77519	2.98518	4.58477	3.61503	
	0.00754	5.28723	6.00957	0.61958	0.77255	0.48576	0.95510														
249	2.52212	5.48857	3.05656	2.45360	4.82223	3.52646	3.01266	3.78278	2.17029	3.78898	4.00680	3.12135	4.17667	2.90487	2.27236	2.40862	2.08637	2.61242	5.92814	4.52418	437 t - - -
	2.68618	4.42225	2.77519	2.73123	3.46354	2.40513	3.72494	3.29354	2.67741	2.69355	4.24690	2.90347	2.73739	3.18146	2.89801	2.37887	2.77519	2.98518	4.58477	3.61503	
	0.00754	5.28723	6.00957	0.61958	0.77255	0.48576	0.95510														
250	3.05510	4.50233	4.63437	4.04174	2.02672	3.97552	4.48643	2.74121	3.90245	1.38945	3.25400	3.46256	4.54363	3.68045	2.52661	3.15703	2.75795	2.62167	5.11667	3.02761	438 l - - -
	2.68618	4.42225	2.77519	2.73123	3.46354	2.40513	3.72494	3.29354	2.67741	2.69355	4.24690	2.90347	2.73739	3.18146	2.89801	2.37887	2.77519	2.98518	4.58477	3.61503	
	0.00754	5.28723	6.00957	0.61958	0.77255	0.48576	0.95510														
251	2.80558	5.50443	3.07791	2.70795	4.84420	3.42191	3.25652	4.07276	2.01341	3.80676	4.54687	3.00140	4.17799	3.01632	1.35562	2.43342	2.77662	3.68371	5.93973	4.53371	439 r - - -
	2.68618	4.42225	2.77519	2.73123	3.46354	2.40513	3.72494	3.29354	2.67741	2.69355	4.24690	2.90347	2.73739	3.18146	2.89801	2.37887	2.77519	2.98518	4.58477	3.61503	
	0.00754	5.28723	6.00957	0.61958	0.77255	0.48576	0.95510														
252	3.52340	4.87976	5.21860	4.68772	2.39137	4.60774	3.19091	2.98912	4.50067	2.51984	4.00632	3.78180	4.95672	4.58700	4.52939	3.92442	3.75228	2.63900	3.99944	0.77263	440 y - - -
	2.68618	4.42225	2.77519	2.73123	3.46354	2.40513	3.72494	3.29354	2.67741	2.69355	4.24690	2.90347	2.73739	3.18146	2.89801	2.37887	2.77519	2.98518	4.58477	3.61503	
	0.00754	5.28723	6.00957	0.61958	0.77255	0.48576	0.95510														
253	2.64464	5.33293	3.36649	2.80708	4.60698	3.02448	3.63757	3.92943	2.69445	3.22096	4.39462	3.33686	4.22233	2.72994	1.16761	2.88924	2.80488	3.04624	5.80949	4.45191	441 r - - -
	2.68618	4.42225	2.77519	2.73123	3.46354	2.40513	3.72494	3.29354	2.67741	2.69355	4.24690	2.90347	2.73739	3.18146	2.89801	2.37887	2.77519	2.98518	4.58477	3.61503	
	0.00754	5.28723	6.00957	0.61958	0.77255	0.48576	0.95510														
254	2.73019	4.45796	5.05032	4.43660	2.60989	4.03508	4.59645	2.00322	4.22331	1.75774	2.74169	4.42244	4.62202	4.35485	4.23278	3.57270	2.80206	1.39334	5.08815	3.69563	442 v - - -
	2.68618	4.42225	2.77519	2.73123	3.46354	2.40513	3.72494	3.29354	2.67741	2.69355	4.24690	2.90347	2.73739	3.18146	2.89801	2.37887	2.77519	2.98518	4.58477	3.61503	
	0.00754	5.28723	6.00957	0.61958	0.77255	0.48576	0.95510														
255	2.65593	4.43822	4.60517	4.14916	2.68857	4.22904	4.00765	2.16376	4.18428	1.63478	3.55688	4.38667	4.59283	4.31812	4.19749	3.53973	3.29346	1.40223	4.03636	2.86427	443 v - - -
	2.68618	4.42225	2.77519	2.73123	3.46354	2.40513	3.72494	3.29354	2.67741	2.69355	4.24690	2.90347	2.73739	3.18146	2.89801	2.37887	2.77519	2.98518	4.58477	3.61503	
	0.00754	5.28723	6.00957	0.61958	0.77255	0.48576	0.95510														
256	2.67400	4.57404	5.14546	4.54605	3.19625	4.41947	4.77448	1.67326	4.35375	2.25533	3.69345	4.56564	4.77215	4.00131	4.38901	3.73679	2.68756	1.05151	5.27342	4.09389	444 v - - -
	2.68618	4.42225	2.77519	2.73123	3.46354	2.40513	3.72494	3.29354	2.67741	2.69355	4.24690	2.90347	2.73739	3.18146	2.89801	2.37887	2.77519	2.98518	4.58477	3.61503	
	0.00754	5.28723	6.00957	0.61958	0.77255	0.48576	0.95510														
257	1.84552	4.88483	3.53258	3.09926	2.80586	3.67747	1.89786	3.31020	3.04194	2.50025	3.98031	3.61438	4.05002	3.27063	3.41419	2.76074	2.70726	2.99400	5.44762	3.21991	445 a - - -
	2.68618	4.42225	2.77519	2.73123	3.46354	2.40513	3.72494	3.29354	2.67741	2.69355	4.24690	2.90347	2.73739	3.18146	2.89801	2.37887	2.77519	2.98518	4.58477	3.61503	
	0.01869	5.28723	4.30831	0.61958	0.77255	0.48576	0.95510														
258	2.77727	5.51493	1.51082	2.34361	4.85818	3.62192	3.96731	4.09598	2.70459	3.81950	4.55650	2.85778	2.99389	2.83182	2.94273	2.19262	2.48328	3.90468	5.94858	4.53774	446 d - - -
	2.68618	4.42225	2.77519	2.73123	3.46354	2.40513	3.72494	3.29354	2.67741	2.69355	4.24690	2.90347	2.73739	3.18146	2.89801	2.37887	2.77519	2.98518	4.58477	3.61503	
	0.00762	5.27617	5.99851	0.61958	0.77255	0.52627	0.89356														
259	3.15481	5.66303	2.67358	2.47874	4.99675	0.98248	3.71130	4.48178	2.50999	3.96114	4.71493	3.20365	4.27785	3.19596	2.86926	2.91200	3.23534	4.04920	6.09540	4.67797	447 g - - -
	2.68614	4.42229	2.77523	2.73114	3.46358	2.40516	3.72498	3.29358	2.67739	2.69346	4.24693	2.90350	2.73743	3.18133	2.89804	2.37890	2.77515	2.98522	4.58481	3.61507	
	0.08234	2.99376	3.54240	0.51665	0.90762	0.52627	0.89356														
260	2.14028	5.00251	2.19999	2.19015	4.36562	3.57103	3.35259	3.72556	2.32198	3.60104	4.18474	2.80192	3.72407	2.90879	2.63880	2.69709	2.61489	3.04929	5.92196	4.51374	450 a - - -
	2.68618	4.42225	2.77519	2.73123	3.46354	2.40513	3.72494	3.29354	2.67741	2.69355	4.24690	2.90347	2.73739	3.18146	2.89801	2.37887	2.77519	2.98518	4.58477	3.61503	
	0.00783	5.24935	5.97169	0.61958	0.77255	0.56941	0.83438														
261	2.48429	4.25559	4.59931	3.42839	3.40426	4.15596	3.62882	2.50040	3.60076	1.44477	2.82904	3.88862	2.93211	3.85984	3.87600	2.73424	3.02285	2.35130	3.45885	3.71408	451 l - - -
	2.68622	4.42229	2.77518	2.73127	3.46358	2.40517	3.72481	3.29358	2.67745	2.69353	4.24694	2.90351	2.73717	3.18129	2.89805	2.37885	2.77524	2.98523	4.58481	3.61507	
	0.09447	2.43479	5.97986	0.20189	1.69930	0.52510	0.89525														
262	2.74846	5.50779	1.86782	2.27618	4.85282	3.54580	3.95758	4.12794	2.35164	3.67675	4.54852	2.61977	3.98873	2.92197	2.88110	2.05336	2.22038	3.89842	5.94045	4.52937	453 d - - -
	2.68618	4.42225	2.77519	2.73123	3.46354	2.40513	3.72494	3.29354	2.67741	2.69355	4.24690	2.90347	2.73739	3.18146	2.89801	2.37887	2.77519	2.98518	4.58477	3.61503	
	0.00768	5.26879	5.99113	0.61958	0.77255	0.55218	0.85730														
263	1.85887	5.48956	2.50671	2.55764	4.82740	3.39171	3.96044	3.81605	2.60210	3.14059	4.53212	2.86356	2.77984	2.70245	2.34054	2.61254	2.70833	3.37683	5.92659	4.52003	454 a - - -
	2.68618	4.42225	2.77519	2.73123	3.46354	2.40513	3.72494	3.29354	2.67741	2.69355	4.24690	2.90347	2.73739	3.18146	2.89801	2.37887	2.77519	2.98518	4.58477	3.61503	
	0.00768	5.26879	5.99113	0.61958	0.77255	0.55218	0.85730														
264	1.89325	5.50194	2.46506	1.63335	4.84496	3.03275	3.73486	4.13976	2.51453	3.53533	4.54315	3.23870	4.02845	2.79868	2.97153	2.67008	2.65534	3.63451	5.93584	4.52610	455 e - - -
	2.68618	4.42225	2.77519	2.73123	3.46354	2.40513	3.72494	3.29354	2.67741	2.69355	4.24690	2.90347	2.73739	3.18146	2.89801	2.37887	2.77519	2.98518	4.58477	3.61503	
	0.01393	5.26879	4.74688	0.61958	0.77255	0.55218	0.85730														

```
    265   2.53108  5.50309  2.69364  1.79494  4.84802  3.21484  3.73106  4.33389  2.22719  3.47001  4.54379  2.89924  3.86263  2.80686  2.27838  2.76533  2.60371  2.83775  5.93586  4.52499    456 e - - -
          2.68618  4.42225  2.77519  2.73123  3.46354  2.40513  3.72494  3.29354  2.67741  2.69355  4.24690  2.90347  2.73739  3.18146  2.89801  2.37887  2.77519  2.98518  4.58477  3.61503
          0.00773  5.26259  5.98493  0.61958  0.77255  0.57328  0.82936
    266   1.71678  4.44907  4.92177  4.31469  3.54717  3.86849  4.55177  1.57353  4.12285  1.87978  2.90505  3.99455  4.58470  4.27435  4.16844  3.25913  3.14872  2.03241  5.07695  3.89821    457 i - - -
          2.68618  4.42225  2.77519  2.73123  3.46354  2.40513  3.72494  3.29354  2.67741  2.69355  4.24690  2.90347  2.73739  3.18146  2.89801  2.37887  2.77519  2.98518  4.58477  3.61503
          0.03058  5.26259  3.69137  0.61958  0.77255  0.57328  0.82936
    267   1.70495  5.50163  2.53149  1.71108  4.84613  3.39976  3.94977  4.33237  2.59263  3.80667  4.54280  2.41342  3.74235  2.66618  2.95705  2.90655  3.07982  3.73938  5.93435  4.52261    458 a - - -
          2.68618  4.42225  2.77519  2.73123  3.46354  2.40513  3.72494  3.29354  2.67741  2.69355  4.24690  2.90347  2.73739  3.18146  2.89801  2.37887  2.77519  2.98518  4.58477  3.61503
          0.03324  5.23991  3.59742  0.61958  0.77255  0.64590  0.74274
    268   1.94681  5.48211  2.88052  2.22380  4.82745  3.14624  3.93139  4.31358  1.98573  3.78747  4.52275  2.89806  4.13781  2.15103  2.61075  2.50751  3.08628  3.43739  5.91459  4.50349    459 a - - -
          2.68618  4.42225  2.77519  2.73123  3.46354  2.40513  3.72494  3.29354  2.67741  2.69355  4.24690  2.90347  2.73739  3.18146  2.89801  2.37887  2.77519  2.98518  4.58477  3.61503
          0.00811  5.21478  5.93712  0.61958  0.77255  0.71897  0.66798
    269   2.24225  4.55769  4.24267  3.15213  3.52240  4.06947  3.19658  2.61458  3.45156  1.35571  3.44854  3.95783  4.44242  3.42698  3.32437  2.97162  3.15829  2.41674  5.16085  2.87926    460 l - - -
          2.68618  4.42225  2.77519  2.73123  3.46354  2.40513  3.72494  3.29354  2.67741  2.69355  4.24690  2.90347  2.73739  3.18146  2.89801  2.37887  2.77519  2.98518  4.58477  3.61503
          0.05862  5.21478  2.96614  0.61958  0.77255  0.71897  0.66798
    270   1.66140  4.38291  4.86333  4.25453  2.28811  4.15222  4.00305  2.51095  4.05971  2.21770  3.32601  4.28309  4.51678  4.20826  4.10082  3.10505  3.15596  2.48829  2.24813  2.73641    461 a - - -
          2.68618  4.42225  2.77519  2.73123  3.46354  2.40513  3.72494  3.29354  2.67741  2.69355  4.24690  2.90347  2.73739  3.18146  2.89801  2.37887  2.77519  2.98518  4.58477  3.61503
          0.09924  5.16468  2.42183  0.61958  0.77255  0.74001  0.64838
    271   2.03041  5.42403  2.41274  2.11742  4.76762  3.46929  3.78584  3.82985  2.18942  3.49802  3.85401  2.80192  4.08462  2.56320  2.61626  2.62931  2.70742  3.65497  5.85759  4.44753    462 a - - -
          2.68618  4.42225  2.77519  2.73123  3.46354  2.40513  3.72494  3.29354  2.67741  2.69355  4.24690  2.90347  2.73739  3.18146  2.89801  2.37887  2.77519  2.98518  4.58477  3.61503
          0.00615  5.09415        *  0.61958  0.77255  0.00000        *
//
```
