# Supplementary material for: Unique high Arctic methane metabolizing community revealed through in situ 13CH4-DNA-SIP enrichment in concert with genome binning
Source: Sci Rep. 2022 Jan 21;12:1160. doi: 10.1038/s41598-021-04486-z (PMC8782848; doi:10.1038/s41598-021-04486-z)
Supplement: Supplementary file 2 — Supplementary Information 2. [file 41598_2021_4486_MOESM2_ESM.pdf]

RID: 4XBYRU9G016
Job Title:2824025549 16S ribosomal RNA [Ga0376100_395...
Program: BLASTN
Database: nt Nucleotide collection (nt)
Query #1: 2824025549 16S ribosomal RNA [Ga0376100_395 : Chloroflexi bacterium B_21] (+)strand Query ID: lcl|Query_23531
Length: 147

Sequences producing significant alignments:

Description	Max Score	Total Score	Query cover	E Value	Per. Ident	Accession
Uncultured bacterium clone Bfb08-02-E7 16S ribosomal RNA gene,...	187	187	95%	1e-43	90.71	JF910611.1
Uncultured bacterium clone BFO1035 16S ribosomal RNA gene,...	182	182	95%	5e-42	90.21	KT460527.1
Uncultured bacterium clone OTU2149 16S ribosomal RNA gene,...	176	176	95%	2e-40	89.29	KY545276.1
Uncultured bacterium clone GB7N87004JVMK8 small subunit...	176	176	82%	2e-40	92.62	HM720389.1
Uncultured bacterium clone GB7N87004JH4RH small subunit...	176	176	82%	2e-40	92.62	HM716556.1
Uncultured bacterium clone GB7N87004IO73K small subunit...	176	176	82%	2e-40	92.62	HM715742.1
Uncultured bacterium clone GB7N87004H9AT2 small subunit...	176	176	82%	2e-40	92.62	HM713917.1
Uncultured bacterium clone GB7N87004I4MHY small subunit...	176	176	82%	2e-40	92.62	HM713504.1
Uncultured bacterium clone GB7N87004ISPQK small subunit...	176	176	82%	2e-40	92.62	HM709438.1
Uncultured bacterium clone GB7N87004IG12I small subunit...	171	171	82%	1e-38	91.80	HM715920.1
Uncultured bacterium clone GB7N87004H5S5B small subunit...	171	171	82%	1e-38	91.80	HM714733.1
Uncultured bacterium clone GB7N87004I58D4 small subunit...	171	171	82%	1e-38	91.80	HM713896.1
Uncultured bacterium clone GB7N87004IN8XF small subunit...	171	171	82%	1e-38	91.80	HM713793.1
Uncultured bacterium clone GB7N87004IQBTD small subunit...	171	171	82%	1e-38	91.80	HM713668.1
Uncultured bacterium clone GB7N87003F5Y2E small subunit...	171	171	82%	1e-38	91.80	HM658570.1
Uncultured bacterium clone GB7N87003G6C5Q small subunit...	171	171	82%	1e-38	91.80	HM654716.1
Uncultured bacterium clone GB7N87003GMUPF small subunit...	171	171	82%	1e-38	91.80	HM654342.1
Uncultured bacterium clone GB7N87003G17CM small subunit...	171	171	82%	1e-38	91.80	HM651539.1
Uncultured bacterium clone GB7N87003FJ4FB small subunit...	171	171	82%	1e-38	91.80	HM649901.1
Uncultured bacterium clone OTU7640 16S ribosomal RNA gene,...	169	169	95%	4e-38	88.65	KY547645.1
Uncultured bacterium clone OTU5903 16S ribosomal RNA gene,...	167	167	95%	1e-37	88.11	KY545877.1
Uncultured bacterium clone OTU804 16S ribosomal RNA gene,...	167	167	95%	1e-37	88.03	KY544053.1
Uncultured bacterium clone OTU1732 16S ribosomal RNA gene,...	167	167	95%	1e-37	88.03	KY543657.1
Uncultured bacterium clone OTU4307 16S ribosomal RNA gene,...	165	165	95%	5e-37	88.03	KY545803.1
Uncultured bacterium clone OTU5395 16S ribosomal RNA gene,...	165	165	95%	5e-37	87.94	KY545512.1
Uncultured bacterium clone OTU1283 16S ribosomal RNA gene,...	165	165	95%	5e-37	87.67	KY543062.1
Uncultured bacterium clone KH-T5-171 16S ribosomal RNA gene,...	165	165	95%	5e-37	88.11	KU930024.1
Uncultured bacterium clone QM-CK-95 16S ribosomal RNA gene,...	165	165	93%	5e-37	88.49	KU929206.1
Uncultured bacterium clone BFO15046 16S ribosomal RNA gene,...	165	165	95%	5e-37	88.11	KT460604.1
Uncultured bacterium clone GB7N87003FWKPN small subunit...	165	165	82%	5e-37	90.98	HM723702.1
Uncultured bacterium clone GB7N87004IK9IA small subunit...	165	165	82%	5e-37	90.98	HM709136.1
Uncultured bacterium clone OTU9719 16S ribosomal RNA gene,...	163	163	95%	2e-36	87.86	KY546786.1
Uncultured bacterium clone OTU2887 16S ribosomal RNA gene,...	163	163	95%	2e-36	87.94	KY545320.1
Uncultured bacterium clone OTU4720 16S ribosomal RNA gene,...	163	163	94%	2e-36	87.94	KY545273.1
Uncultured bacterium clone OTU2179 16S ribosomal RNA gene,...	163	163	95%	2e-36	87.94	KY544094.1
Uncultured bacterium clone OTU1947 16S ribosomal RNA gene,...	163	163	95%	2e-36	88.03	KY543692.1
Uncultured bacterium clone QM-T2-28 16S ribosomal RNA gene,...	163	163	94%	2e-36	88.03	KU928505.1
Uncultured bacterium clone BFO15038 16S ribosomal RNA gene,...	163	163	95%	2e-36	88.11	KT460596.1
Uncultured bacterium clone BFO15009 16S ribosomal RNA gene,...	163	163	95%	2e-36	88.11	KT460567.1
Uncultured bacterium clone BTE15052 16S ribosomal RNA gene,...	163	163	94%	2e-36	88.03	KT460480.1
Uncultured bacterium clone LG16 16S ribosomal RNA gene, partia...	163	163	94%	2e-36	88.03	JX133504.1
Uncultured Chloroflexi bacterium clone TSD_20 16S ribosomal RN...	161	161	84%	6e-36	90.32	MH174131.1
Uncultured bacterium clone OTU3995 16S ribosomal RNA gene,...	161	161	94%	6e-36	87.94	KY546566.1
Uncultured bacterium clone OTU1423 16S ribosomal RNA gene,...	161	161	95%	6e-36	87.50	KY543117.1
Uncultured bacterium clone OTU4253 16S ribosomal RNA gene,...	159	159	95%	2e-35	87.14	KY546520.1
Uncultured bacterium clone OTU3038 16S ribosomal RNA gene,...	159	159	95%	2e-35	86.99	KY545602.1
Uncultured bacterium clone BFO15013 16S ribosomal RNA gene,...	159	159	95%	2e-35	87.41	KT460571.1
Uncultured bacterium clone BFO1057 16S ribosomal RNA gene,...	159	159	95%	2e-35	87.41	KT460549.1

Select Alignments:
>Uncultured bacterium clone Bfb08-02-E7 16S ribosomal RNA gene, partial sequence
Sequence ID: JF910611.1 Length: 331
Range 1: 13 to 152

Score:187 bits(101), Expect:1e-43,
Identities:127/140(91%), Gaps:0/140(0%), Strand: Plus/Plus

```
Query  8    AGAGTTTGATCCTGGCTCAGGACAAACGCTGGCGGCGTGCATAACACATGCAAGTCGAAC  67
            |||||||||||||||||||||||||||||||||||||||| |||||||||||||||| ||
Sbjct  13   AGAGTTTGATCCTGGCTCAGGACAAACGCTGGCGGCGTGCGTAACACATGCAAGTCGGAC  72

Query  68   GAGTGTAGCGGCTTCGGCTGGTACATGAGTGGCGGACGGGTGAGTAACACGTGGATGACC  127
            ||| |  | ||||||||||| | |  |||||||||||||||| |||||||||||||||||
Sbjct  73   GAGCGGGGTGGCTTCGGCTGCTTCGCGAGTGGCGGACGGGTGCGTAACACGTGGATGACC  132

Query  128  TGCCTTGCGATGGGGGATAC  147
            ||||| ||| ||||||||||
Sbjct  133  TGCCTCGCGGTGGGGGATAC  152
```

>Uncultured bacterium clone BFO1035 16S ribosomal RNA gene, partial sequence
Sequence ID: KT460527.1 Length: 902
Range 1: 1 to 140

Score:182 bits(98), Expect:5e-42,
Identities:129/143(90%), Gaps:6/143(4%), Strand: Plus/Plus

```
Query  8    AGAGTTTGATCCTGGCTCAGGACAAACGCTGGCGGCGTGCATAACACATGCAAGTCGAAC  67
            |||||||||||||||||||||||||||||||||||||||| |||||||||||||||||||
Sbjct  1    AGAGTTTGATCCTGGCTCAGGACAAACGCTGGCGGCGTGCGTAACACATGCAAGTCGAAC  60

Query  68   GAGTGTAGCGGC-TTCGGCTGGTACAT-GAGTGGCGGACGGGTGAGTAACACGTGGATGA  125
            | | || ||| | |||||   || ||| ||||||||||||||||||||||||||||||||
Sbjct  61   G-GGGTGGCGTCTTTCGGGATGT-CATCGAGTGGCGGACGGGTGAGTAACACGTGGATGA  118

Query  126  CCTGCCT-TGCGATGGGGGATAC  147
            ||||||  |||| ||||||||||
Sbjct  119  CCTGCCGATGCG-TGGGGGATAC  140
```

>Uncultured Chloroflexi bacterium clone TSD_20 16S ribosomal RNA gene, partial sequence
Sequence ID: MH174131.1 Length: 785
Range 1: 1 to 122

Score:161 bits(87), Expect:6e-36,
Identities:112/124(90%), Gaps:2/124(1%), Strand: Plus/Plus

```
Query  8    AGAGTTTGATCCTGGCTCAGGACAAACGCTGGCGGCGTGCATAACACATGCAAGTCGAAC  67
            |||||||||||||||||||||||||||||||||||||||| | | |||||||||||||||
Sbjct  1    AGAGTTTGATCCTGGCTCAGGACAAACGCTGGCGGCGTGCGTTAAACATGCAAGTCGAAC  60

Query  68   GAGTGTAGCGGCTTCGGCTGGTACATGAGTGGCGGACGGGTGAGTAACACGTGGATGACC  127
            | |||||  |||||||| | || ||| |||||||||||| |||||| |||||||||||||
Sbjct  61   G-GTGTATGGGCTTCGGTTCGTGCAT-AGTGGCGGACGGCTGAGTAGCACGTGGATGACC  118

Query  128  TGCC  131
            ||||
Sbjct  119  TGCC  122
```
