# Supplementary material for: Unique high Arctic methane metabolizing community revealed through in situ 13CH4-DNA-SIP enrichment in concert with genome binning
Source: Sci Rep. 2022 Jan 21;12:1160. doi: 10.1038/s41598-021-04486-z (PMC8782848; doi:10.1038/s41598-021-04486-z)
Supplement: Supplementary file 3 — Supplementary Information 3. [file 41598_2021_4486_MOESM3_ESM.pdf]

Supplementary File S3- 3D Modeling Results

MAG #21 3D protein modeling of the AA sequences of gene 573136 (*mmoX*) and two genes of interest (573135 and 573134) that were directly upstream of *mmoX*. The modeling was performed with SWISS-MODEL (Waterhouse et al 2018) and I-TASSER (Yang et al 2015). The three SWISS-MODEL reports are on pages 2-15 of this PDF document, followed by the three I-TASSER reports on pages 16-18. Overall the 3D modeling results corroborated the Pfam analysis/annotation results for all three MAG #21 genes.

For MAG #21's *mmoX* gene the modeling results based on SWISS-MODEL identified several possible PDB (protein data base) templates that were most closely related to the 3D structure of *mmoX* on MAG #21. These included several templates, with methane monooxygenase (sMMO) having the highest sequence similarity of all the templates to our *mmoX* gene (see graphic below). The MAG #21 *mmoX*'s 3D models that were based on these matched templates in the SWISS-MODEL are reported on pages 2-5 of this PDF document.

Sequence similarity of top matching PDB 3D templates (circled in blue) to the MAG #21's *mmox* (red dot) 3D model. Graphic adapted from the SWISS-MODEL 3D protein modeling results.

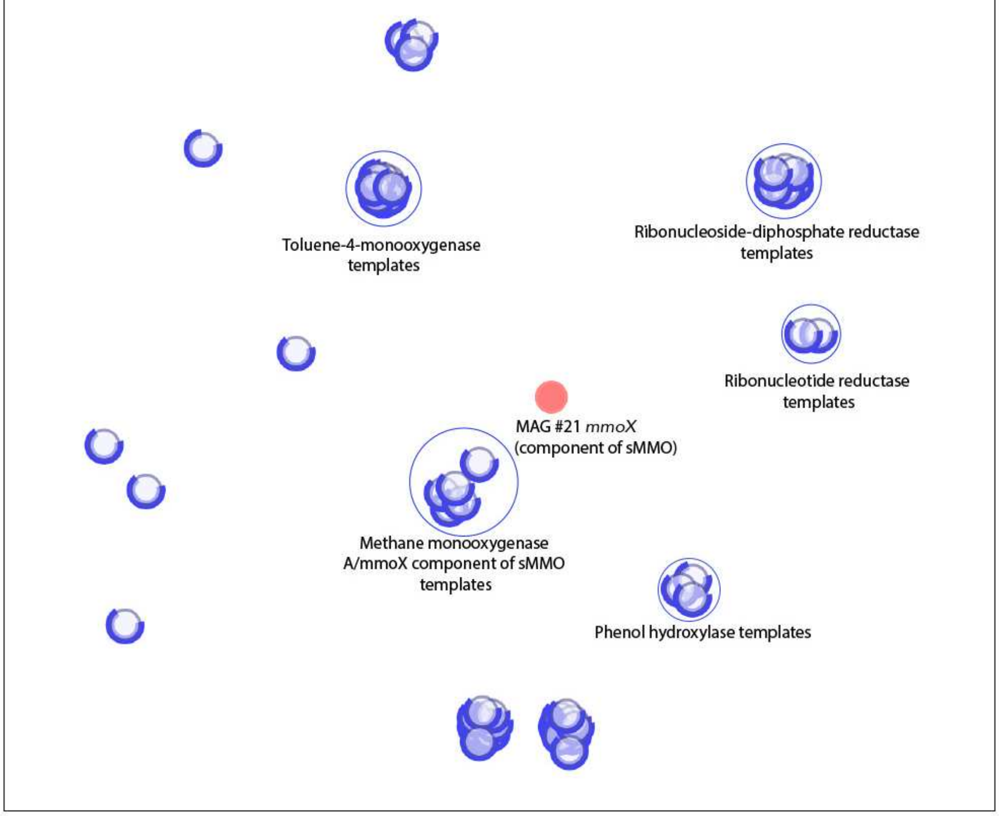

For MAG #21's *mmoX* modeling with I-TASSER, the top PDB template hits included an aldehyde deformylating oxidase, R2-like ligand-binding oxidase, methane monooxygenase, and arylamine oxygenase. While MAG #21's *mmoX* could be structurally similar to any of these matches, the biological annotations and predicted function using the subsequent COACH/COFACTOR analysis (Zhang et al 2017) inferred the top function of MAG #21's *mmoX* to be a methane monooxygenase based on Enzyme Commission (EC) number and active site. The I-TASSER and COACH/COFACTOR results are in the report on page 16 of this PDF.

References:

Waterhouse A, Bertoni M, Bienert S, Studer G, Tauriello G, Gumienny R *et al* (2018). SWISS-MODEL: homology modelling of protein structures and complexes. *Nucleic acids research* **46:** W296-W303.

Yang J, Yan R, Roy A, Xu D, Poisson J, Zhang Y (2015). The I-TASSER Suite: protein structure and function prediction. *Nature methods* **12:** 7.

Zhang C, Freddolino PL, Zhang Y (2017). COFACTOR: improved protein function prediction by combining structure, sequence and protein–protein interaction information. *Nucleic acids research* **45:** W291-W299.

SWISS-MODEL Homology Modelling Report

Model Building Report

This document lists the results for the homology modelling project "gene_573136_mmox" submitted to SWISS-MODEL workspace on April 27, 2020, 6:25 p.m..The submitted primary amino acid sequence is given in Table T1.

If you use any results in your research, please cite the relevant publications:

- Waterhouse, A., Bertoni, M., Bienert, S., Studer, G., Tauriello, G., Gumienny, R., Heer, F.T., de Beer, T.A.P., Rempfer, C., Bordoli, L., Lepore, R., Schwede, T. SWISS-MODEL: homology modelling of protein structures and complexes. Nucleic Acids Res. 46(W1), W296-W303 (2018). doi>
- Guex, N., Peitsch, M.C., Schwede, T. Automated comparative protein structure modeling with SWISS-MODEL and Swiss-PdbViewer: A historical perspective. Electrophoresis 30, S162-S173 (2009). doi>
- Bienert, S., Waterhouse, A., de Beer, T.A.P., Tauriello, G., Studer, G., Bordoli, L., Schwede, T. The SWISS-MODEL Repository - new features and functionality. Nucleic Acids Res. 45, D313-D319 (2017). doi>
- Studer, G., Rempfer, C., Waterhouse, A.M., Gumienny, G., Haas, J., Schwede, T. QMEANDisCo - distance constraints applied on model quality estimation. Bioinformatics 36, 1765-1771 (2020). doi>
- Bertoni, M., Kiefer, F., Biasini, M., Bordoli, L., Schwede, T. Modeling protein quaternary structure of homo- and hetero-oligomers beyond binary interactions by homology. Scientific Reports 7 (2017). doi>

Results

The SWISS-MODEL template library (SMTL version 2020-04-22, PDB release 2020-04-17) was searched with BLAST (Camacho et al.) and HHBlits (Remmert et al.) for evolutionary related structures matching the target sequence in Table T1. For details on the template search, see Materials and Methods. Overall 149 templates were found (Table T2).

Models

The following models were built (see Materials and Methods "Model Building"):

Model #01	File	Built with	Oligo-State	Ligands	GMQE	QMEAN
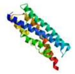	PDB	ProMod3 3.0.0	monomer (matching prediction)	2 x FE: FE (III) ION;	0.35	-4.17

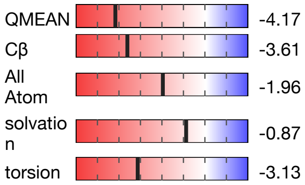

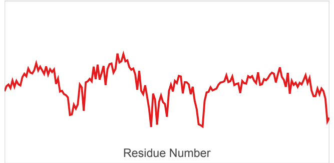

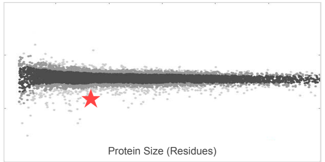

Template	Seq Identity	Oligo-state	QSQE	Found by	Method	Resolution	Seq Similarity	Range	Coverage	Description
1mmo.1.D	18.99	homo-dimer	0.15	HHblits	X-ray	2.20Å	0.28	55 - 219	0.63	METHANE MONOOXYGENASE HYDROLASE (ALPHA CHAIN)

Included Ligands

Ligand	Description
2 x FE	FE (III) ION

Excluded ligands

Ligand Name.Number	Reason for Exclusion	Description
ACY.3	Not biologically relevant.	ACETIC ACID
ACY.6	Not biologically relevant.	ACETIC ACID
FE.1	Binding site not conserved.	FE (III) ION
FE.2	Binding site not conserved.	FE (III) ION

```
Target     MSTAFAEQVRTLVSRRDPAYRPAMFIDEAEKKALAEKLGDASNYDLGLLREMVAQDPDNYEFRKALVSGIVSAEYAGIDA
1mmo.1.D   ------------------------------------------------------VHPKWNETMKVVSNFLEVGEYNAIAA

Target     FSRKVCEWQTWPVPWELIMAMARQTWDEVRHAQLGTELLKSYGGEV---GEYPD---TLAGSPPNQATAAV-AQGQGADV
1mmo.1.D   TGMLW---DSAQAA-EQKNGYLAQVLDEIRHTHQCAYVNYYFAKNGQDPAGHNDARRTRTIGPLWKGMKRVFSDGFIS--

Target     MRDPVISLSVTNVALEGAALD-LFKGTSDLGRKVNDDLMRLVYDYNWADEVTHTAIGDYFVKVLCDGNPAEEARALRAHA
1mmo.1.D   -GDAVECSLNLQLVGEACFTNPLIVAVTEWAAANGDEITPTVFLSIETDELRHMANGYQTVVSIAND-------------

Target     MFEGMRERLSGQQSAEIR
1mmo.1.D   ------------------
```

Model #02	File	Built with	Oligo-State	Ligands	GMQE	QMEAN
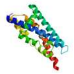	PDB	ProMod3 3.0.0	monomer	2 x FE: FE (III) ION;	0.33	-4.80

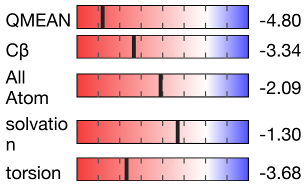

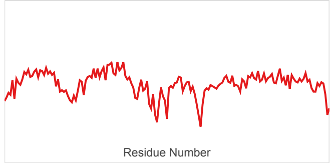

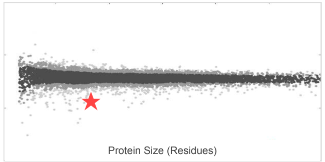

Template	Seq Identity	Oligo-state	QSQE	Found by	Method	Resolution	Seq Similarity	Range	Coverage	Description
6d7k.1.A	20.25	monomer	0.00	HHblits	X-ray	2.60Å	0.28	55 - 219	0.63	Methane monooxygenase hydroxylase, MmoX1

Included Ligands

Ligand	Description
2 x FE	FE (III) ION

Excluded ligands

Ligand Name.Number	Reason for Exclusion	Description
FE.5	Binding site not conserved.	FE (III) ION
FE.6	Binding site not conserved.	FE (III) ION

FMT.4	Not biologically relevant.	FORMIC ACID
FMT.8	Not biologically relevant.	FORMIC ACID
HEZ.3	Binding site not conserved.	HEXANE-1,6-DIOL
HEZ.7	Binding site not conserved.	HEXANE-1,6-DIOL

```
Target    MSTAFAEQVRTLVSRRDPAYRPAMFIDEAEKKALAEKLGDASNYDLGLLREMVAQDPDNYEFRKALVSGIVSAEYAGIDA
6d7k.1.A  ------------------------------------------------------VHPRWGETMKVISNFLEVGEYNAIAA

Target    FSRKVCEWQTWPVPWELIMAMARQTWDEVRHAQLGTELLKSYGGEV---GEY---PDTLAGSPPNQATAAV-AQGQGADV
6d7k.1.A  SAMLW---DSATAA-EQKNGYLAQVLDEIRHTHQCAFINHYYSKHYHDPAGHNDARRTRAIGPLWKGMKRVFADGFIS--

Target    MRDPVISLSVTNVALEGAALD-LFKGTSDLGRKVNDDLMRLVYDYNWADEVTHTAIGDYFVKVLCDGNPAEEARALRAHA
6d7k.1.A  -GDAVECSVNLQLVGEACFTNPLIVAVTEWASANGDEITPTVFLSVETDELRHMANGYQTVVSIAND-------------

Target    MFEGMRERLSGQQSAEIR
6d7k.1.A  ------------------
```

Model #04	File	Built with	Oligo-State	Ligands	GMQE	QMEAN
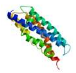	PDB	ProMod3 3.0.0	monomer (matching prediction)	2 x FE: FE (III) ION;	0.32	-4.39

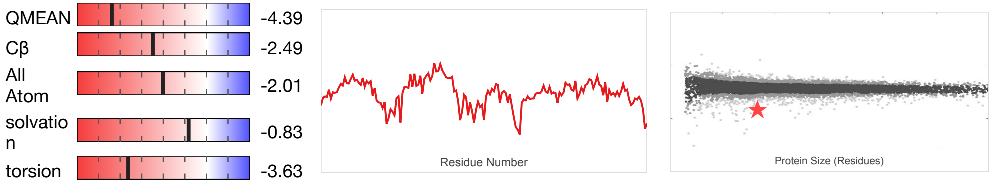

Template	Seq Identity	Oligo-state	QSQE	Found by	Method	Resolution	Seq Similarity	Range	Coverage	Description
1fz4.1.A	18.35	homo-dimer	0.16	HHblits	X-ray	2.38Å	0.28	55 - 219	0.63	METHANE MONOOXY-GENASE COMPONENT A, ALPHA CHAIN

Included Ligands

Ligand	Description
2 x FE	FE (III) ION

Excluded ligands

Ligand Name.Number	Reason for Exclusion	Description
CA.5	Binding site not conserved.	CALCIUM ION
CA.6	Not in contact with model.	CALCIUM ION
CA.7	Binding site not conserved.	CALCIUM ION
FE.3	Binding site not conserved.	FE (III) ION
FE.4	Binding site not conserved.	FE (III) ION

FMT.8	Not biologically relevant.	FORMIC ACID

```
Target    MSTAFAEQVRTLVSRRDPAYRPAMFIDEAEKKALAEKLGDASNYDLGLLREMVAQDPDNYEFRKALVSGIVSAEYAGIDA
1fz4.1.A  ------------------------------------------------------VHPKWNETMKVVSNFLEVGEYNAIAA

Target    FSRKVCEWQTWPVPWELIMAMARQTWDEVRHAQLGTELLKSYGGEV---GEYP---DTLAGSPPNQATAAV-AQGQGADV
1fz4.1.A  TGMLW---DSAQAA-EQKNGYLAQVLDEIRHTHQCAYVNYYFAKNGQDPAGHNDARRTRTIGPLWKGMKRVFSDGFIS--

Target    MRDPVISLSVTNVALEGAALD-LFKGTSDLGRKVNDDLMRLVYDYNWADEVTHTAIGDYFVKVLCDGNPAEEARALRAHA
1fz4.1.A  -GDAVECSLNLQLVGEACFTNPLIVAVTEWAAANGDEITPTVFLSIETDELRHMANGYQTVVSIAND-------------

Target    MFEGMRERLSGQQSAEIR
1fz4.1.A  ------------------
```

Model #03	File	Built with	Oligo-State	Ligands	GMQE	QMEAN
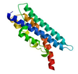	PDB	ProMod3 3.0.0	monomer (matching prediction)	2 x FE: FE (III) ION;	0.32	-5.05

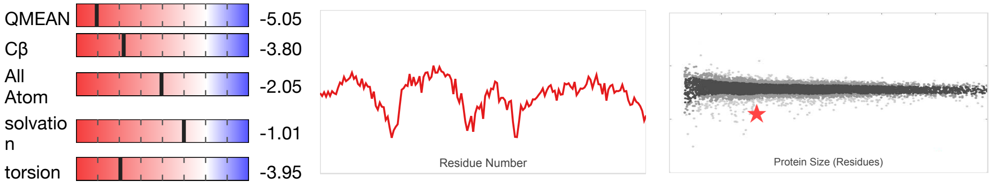

Template	Seq Identity	Oligo-state	QSQE	Found by	Method	Resolution	Seq Similarity	Range	Coverage	Description
4gam.1.B	18.35	homo-dimer	0.00	HHblits	X-ray	2.90Å	0.28	55 - 219	0.63	Methane monooxygenase component A alpha chain

Included Ligands

Ligand	Description
2 x FE	FE (III) ION

Excluded ligands

Ligand Name.Number	Reason for Exclusion	Description
FE.3	Binding site not conserved.	FE (III) ION
FE.4	Binding site not conserved.	FE (III) ION

```
Target    MSTAFAEQVRTLVSRRDPAYRPAMFIDEAEKKALAEKLGDASNYDLGLLREMVAQDPDNYEFRKALVSGIVSAEYAGIDA
4gam.1.B  ------------------------------------------------------VHPKWNETMKVVSNFLEVGEYNAIAA

Target    FSRKVCEWQTWPVPWELIMAMARQTWDEVRHAQLGTELLKSYGGEV---GEYP---DTLAGSPPNQATAAV-AQGQGADV
4gam.1.B  TGMLW---DSAQAA-EQKNGYLAQVLDEIRHTHQCAYVNYYFAKNGQDPAGHNDARRTRTIGPLWKGMKRVFSDGFIS--

Target    MRDPVISLSVTNVALEGAALD-LFKGTSDLGRKVNDDLMRLVYDYNWADEVTHTAIGDYFVKVLCDGNPAEEARALRAHA
```

```
4gam.1.B   -GDAVECSLNLQLVGEACFTNPLIVAVTEWAAANGDEITPTVFLSIETDELRHMANGYQTVVSIAND-------------

Target     MFEGMRERLSGQQSAEIR
4gam.1.B   ------------------
```

Model #05	File	Built with	Oligo-State	Ligands	GMQE	QMEAN
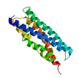	PDB	ProMod3 3.0.0	monomer (matching prediction)	2 x FE: FE (III) ION;	0.32	-4.40

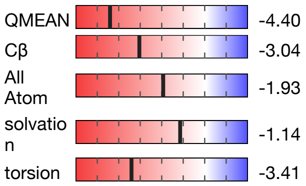

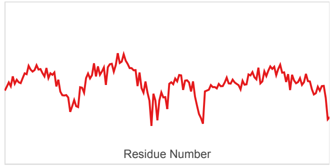

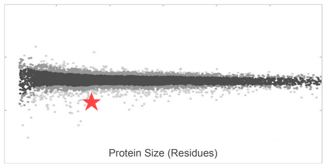

Template	Seq Identity	Oligo-state	QSQE	Found by	Method	Resolution	Seq Similarity	Range	Coverage	Description
1mty.1.A	18.99	homo-dimer	0.08	HHblits	X-ray	1.70Å	0.28	55 - 219	0.63	METHANE MONOOXYGENASE HYDROXYLASE

Included Ligands

Ligand	Description
2 x FE	FE (III) ION

Excluded ligands

Ligand Name.Number	Reason for Exclusion	Description
FE.3	Binding site not conserved.	FE (III) ION
FE.4	Binding site not conserved.	FE (III) ION

```
Target     MSTAFAEQVRTLVSRRDPAYRPAMFIDEAEKKALAEKLGDASNYDLGLLREMVAQDPDNYEFRKALVSGIVSAEYAGIDA
1mty.1.A   ------------------------------------------------------VHPKWNETMKVVSNFLEVGEYNAIAA

Target     FSRKVCEWQTWPVPWELIMAMARQTWDEVRHAQLGTELLKSYGGEV---GEYPD---TLAGSPPNQATAAV-AQGQGADV
1mty.1.A   TGMLW---DSAQAA-EQKNGYLAQVLDEIRHTHQCAYVNYYFAKNGQDPAGHNDARRTRTIGPLWKGMKRVFSDGFIS--

Target     MRDPVISLSVTNVALEGAALD-LFKGTSDLGRKVNDDLMRLVYDYNWADEVTHTAIGDYFVKVLCDGNPAEEARALRAHA
1mty.1.A   -GDAVECSLNLQLVGEACFTNPLIVAVTEWAAANGDEITPTVFLSIETDELRHMANGYQTVVSIAND-------------

Target     MFEGMRERLSGQQSAEIR
1mty.1.A   ------------------
```

Materials and Methods

Template Search

Template search with BLAST and HHBlits has been performed against the SWISS-MODEL template library (SMTL, last update: 2020-04-22, last included PDB release: 2020-04-17).

The target sequence was searched with BLAST against the primary amino acid sequence contained in the SMTL.

An initial HHblits profile has been built using the procedure outlined in (Remmert et al.), followed by 1 iteration of HHblits against NR20. The obtained profile has then be searched against all profiles of the SMTL. A total of 149 templates were found.

Model Building

Models are built based on the target-template alignment using ProMod3. Coordinates which are conserved between the target and the template are copied from the template to the model. Insertions and deletions are remodelled using a fragment library. Side chains are then rebuilt. Finally, the geometry of the resulting model is regularized by using a force field. In case loop modelling with ProMod3 fails, an alternative model is built with PROMOD-II (Guex et al.).

Model Quality Estimation

The global and per-residue model quality has been assessed using the QMEAN scoring function (Studer et al.).

Ligand Modelling

Ligands present in the template structure are transferred by homology to the model when the following criteria are met: (a) The ligands are annotated as biologically relevant in the template library, (b) the ligand is in contact with the model, (c) the ligand is not clashing with the protein, (d) the residues in contact with the ligand are conserved between the target and the template. If any of these four criteria is not satisfied, a certain ligand will not be included in the model. The model summary includes information on why and which ligand has not been included.

Oligomeric State Conservation

The quaternary structure annotation of the template is used to model the target sequence in its oligomeric form. The method (Bertoni et al.) is based on a supervised machine learning algorithm, Support Vector Machines (SVM), which combines interface conservation, structural clustering, and other template features to provide a quaternary structure quality estimate (QSQE). The QSQE score is a number between 0 and 1, reflecting the expected accuracy of the interchain contacts for a model built based a given alignment and template. Higher numbers indicate higher reliability. This complements the GMQE score which estimates the accuracy of the tertiary structure of the resulting model.

References

- **BLAST**
  Camacho, C., Coulouris, G., Avagyan, V., Ma, N., Papadopoulos, J., Bealer, K., Madden, T.L. BLAST+: architecture and applications. BMC Bioinformatics 10, 421-430 (2009). doi>
- **HHblits**
  Remmert, M., Biegert, A., Hauser, A., Söding, J. HHblits: lightning-fast iterative protein sequence searching by HMM-HMM alignment. Nat Methods 9, 173-175 (2012). doi>

Table T1:

Primary amino acid sequence for which templates were searched and models were built.

```
MSTAFAEQVRTLVSRRDPAYRPAMFIDEAEKKALAEKLGDASNYDLGLLREMVAQDPDNYEFRKALVSGIVSAEYAGIDAFSRKVCEWQTWPVPWELIMA
MARQTWDEVRHAQLGTELLKSYGGEVGEYPDTLAGSPPNQATAAVAQGQGADVMRDPVISLSVTNVALEGAALDLFKGTSDLGRKVNDDLMRLVYDYNWA
DEVTHTAIGDYFVKVLCDGNPAEEARALRAHAMFEGMRERLSGQQSAEIR
```

Table T2:

Template	Seq Identity	Oligo-state	QSQE	Found by	Method	Resolution	Seq Similarity	Coverage	Description
6sf5.1.B	18.13	homo-dimer	0.12	HHblits	X-ray	1.90Å	0.28	0.64	Ribonucleoside-diphosphate reductase, beta subunit 1
6sf4.1.A	18.13	homo-dimer	0.08	HHblits	X-ray	1.70Å	0.28	0.64	Ribonucleoside-diphosphate reductase, beta subunit 1
2inp.1.A	16.85	homo-dimer	-	HHblits	X-ray	2.30Å	0.27	0.71	Phenol hydroxylase component phN
3dhg.2.B	14.29	monomer	-	HHblits	X-ray	1.85Å	0.25	0.73	toluene 4-monooxygenase hydroxylase beta subunit
3dhg.1.B	14.29	monomer	-	HHblits	X-ray	1.85Å	0.25	0.73	toluene 4-monooxygenase hydroxylase beta subunit
3dhh.1.B	14.29	monomer	-	HHblits	X-ray	1.94Å	0.25	0.73	toluene 4-monooxygenase hydroxylase beta subunit
5tdv.1.B	14.29	homo-dimer	0.24	HHblits	X-ray	2.00Å	0.25	0.73	Toluene-4-monooxygenase system protein E
5tds.1.B	14.29	homo-dimer	0.22	HHblits	X-ray	1.72Å	0.25	0.73	Toluene-4-monooxygenase system protein E
3ge8.1.B	14.29	homo-dimer	0.23	HHblits	X-ray	2.19Å	0.25	0.73	Toluene-4-monooxygenase system protein E
1t0s.1.B	11.54	homo-dimer	0.09	HHblits	X-ray	2.20Å	0.25	0.73	toluene, o-xylene monooxygenase oxygenase subunit
3rmk.1.B	13.33	monomer	-	HHblits	X-ray	1.95Å	0.25	0.72	Toluene-4-monooxygenase system protein E
3q3o.1.B	13.33	homo-dimer	0.26	HHblits	X-ray	1.95Å	0.25	0.72	Toluene-4-monooxygenase system protein E
2inc.1.B	11.48	homo-dimer	0.11	HHblits	X-ray	1.85Å	0.25	0.73	Toluene, o-xylene monooxygenase oxygenase subunit
2ind.1.B	11.54	homo-dimer	0.20	HHblits	X-ray	2.20Å	0.25	0.73	Toluene, o-xylene monooxygenase oxygenase subunit
6cwp.1.A	18.13	homo-dimer	0.05	HHblits	X-ray	1.92Å	0.27	0.64	Ribonucleotide reductase
6qrz.1.A	16.88	homo-dimer	0.03	HHblits	2DX	3.00Å	0.29	0.64	Ribonucleoside-diphosphate reductase
3u52.1.C	10.67	homo-dimer	0.02	HHblits	X-ray	1.95Å	0.24	0.71	Phenol hydroxylase component phL
1mmo.1.D	18.99	homo-dimer	0.15	HHblits	X-ray	2.20Å	0.28	0.63	METHANE MONOOXYGENASE HYDROLASE (ALPHA CHAIN)
2inn.1.A	18.13	monomer	-	HHblits	X-ray	2.70Å	0.28	0.64	Phenol hydroxylase component phN
4p1b.1.A	18.87	homo-dimer	0.07	HHblits	X-ray	2.05Å	0.28	0.64	Toluene-4-monooxygenase system protein A
3rmk.1.A	18.87	monomer	-	HHblits	X-ray	1.95Å	0.28	0.64	Toluene-4-monooxygenase system protein A
3i5j.1.A	18.75	homo-dimer	-	HHblits	X-ray	1.90Å	0.28	0.64	Toluene-4-monooxygenase system protein A
3dhg.2.A	18.75	monomer	-	HHblits	X-ray	1.85Å	0.28	0.64	toluene 4-monooxygenase hydroxylase alpha subunit
5tdv.1.A	18.87	homo-dimer	0.02	HHblits	X-ray	2.00Å	0.28	0.64	Toluene-4-monooxygenase system protein A
3ge8.1.A	18.87	homo-dimer	-	HHblits	X-ray	2.19Å	0.28	0.64	Toluene-4-monooxygenase system protein A
5tdt.1.D	18.87	homo-dimer	0.04	HHblits	X-ray	1.82Å	0.28	0.64	Toluene-4-monooxygenase system protein A
5tds.1.A	18.87	homo-dimer	-	HHblits	X-ray	1.72Å	0.28	0.64	Toluene-4-monooxygenase system protein A
5olk.1.D	15.52	homo-tetramer	0.00	HHblits	X-ray	2.45Å	0.27	0.70	Ribonucleoside-diphosphate reductase, beta subunit 1
5olk.1.A	15.52	homo-tetramer	0.00	HHblits	X-ray	2.45Å	0.27	0.70	Ribonucleoside-diphosphate reductase, beta subunit 1
5olk.1.B	15.52	homo-tetramer	0.00	HHblits	X-ray	2.45Å	0.27	0.70	Ribonucleoside-diphosphate reductase, beta subunit 1
5olk.1.C	15.52	homo-tetramer	0.00	HHblits	X-ray	2.45Å	0.27	0.70	Ribonucleoside-diphosphate reductase, beta subunit 1
3rng.1.A	19.75	homo-dimer	-	HHblits	X-ray	2.81Å	0.28	0.63	Toluene o-xylene monooxygenase component
3ri7.1.A	18.24	homo-dimer	0.06	HHblits	X-ray	2.10Å	0.28	0.64	Toluene-4-monooxygenase system protein A
6d7k.1.A	20.25	monomer	-	HHblits	X-ray	2.60Å	0.28	0.63	Methane monooxygenase hydroxylase, MmoX1
6cwo.1.A	18.13	homo-dimer	0.02	HHblits	X-ray	1.87Å	0.27	0.64	Ribonucleotide reductase
3rne.1.A	18.99	homo-dimer	-	HHblits	X-ray	2.50Å	0.28	0.63	Toluene o-xylene monooxygenase component
1t0s.1.A	18.99	homo-dimer	-	HHblits	X-ray	2.20Å	0.28	0.63	toluene, o-xylene monooxygenase oxygenase subunit
3u52.1.A	17.50	homo-dimer	-	HHblits	X-ray	1.95Å	0.28	0.64	Phenol hydroxylase component phN
3rnb.1.A	18.99	homo-dimer	-	HHblits	X-ray	2.64Å	0.28	0.63	Toluene o-xylene monooxygenase component
3rnf.1.A	18.99	homo-dimer	0.03	HHblits	X-ray	2.20Å	0.28	0.63	Toluene o-xylene monooxygenase component
3rn9.1.A	18.99	homo-dimer	-	HHblits	X-ray	2.80Å	0.28	0.63	Toluene o-xylene monooxygenase component
3n20.1.A	18.99	homo-dimer	-	HHblits	X-ray	1.90Å	0.28	0.63	Toluene o-xylene monooxygenase component
3n1x.1.A	18.99	homo-dimer	-	HHblits	X-ray	2.40Å	0.28	0.63	Toluene o-xylene monooxygenase component
3n1z.1.A	18.99	homo-dimer	0.01	HHblits	X-ray	2.90Å	0.28	0.63	Toluene o-xylene monooxygenase component
4gam.1.B	18.35	homo-dimer	-	HHblits	X-ray	2.90Å	0.28	0.63	Methane monooxygenase component A alpha chain
6f65.1.A	13.66	homo-dimer	0.02	HHblits	X-ray	1.95Å	0.27	0.64	Ribonucleotide reductase small subunit
1fz4.1.A	18.35	homo-dimer	0.16	HHblits	X-ray	2.38Å	0.28	0.63	METHANE MONOOXYGENASE COMPONENT A, ALPHA CHAIN
6ebo.1.A	12.73	homo-dimer	0.01	HHblits	X-ray	1.58Å	0.25	0.66	Ribonucleoside-diphosphate reductase, beta subunit
1mty.1.A	18.99	homo-dimer	0.08	HHblits	X-ray	1.70Å	0.28	0.63	METHANE MONOOXYGENASE HYDROXYLASE
4n83.1.A	12.05	homo-dimer	-	HHblits	X-ray	2.65Å	0.25	0.66	Ribonucleoside-diphosphate reductase subunit beta

The table above shows the top 50 filtered templates. A further 99 templates were found which were considered to be less suitable for modelling than the filtered list.

4n7r.1.A, 6f6h.1.A, 3n1y.1.A, 6qk1.1.A, 5x1e.1.B, 2o1z.1.A, 4bmr.2.A, 2ani.1.A, 2uw2.1.A, 1oxz.1.A, 1xsm.1.A, 4tod.1.A, 6gp3.1.A, 3rna.1.A, 4toh.1.B, 4bmp.1.A, 6f6l.1.A, 2bq1.1.D, 6gp2.1.A, 4djn.1.B, 1w68.1.A, 4bmq.1.A, 6qk0.1.B, 1jk0.1.A, 1mhy.1.A, 6tqx.1.A, 4hr5.1.A, 3oql.1.A, 6i92.1.A, 2inp.1.C, 3rnc.1.A, 5omj.1.A, 6qk0.1.A, 2bq1.1.C, 2inc.1.A, 1xmh.1.C, 5uxg.1.A, 3olj.1.A, 3hf1.1.B, 4tof.1.A, 6f6f.1.A, 5yjl.1.A, 1smq.1.A, 4gam.2.E, 1kgo.1.A, 3vpm.1.A, 3hf1.1.A, 5xnb.1.C, 3ee4.1.A, 6i93.1.A, 6f6c.1.A, 1syy.1.A, 6i90.1.A, 5yjl.1.B, 6qjv.1.A, 3qk9.1.B, 1nwm.1.A, 4m1h.1.B, 6cwq.1.A, 5x90.1.B, 3qk9.1.A, 4hr0.1.A, 1mmo.1.A, 5tdu.1.B, 1uzr.1.A, 2rdb.1.A, 2vux.1.A, 4d8f.1.B, 4bmo.1.A, 4djn.1.A, 1mhy.1.B, 4n7r.1.C, 6gp3.1.B, 4mud.1.A, 6ebp.1.A, 3vpn.1.A, 6qo5.1.A, 3ri7.1.B, 5che.1.B, 5x90.2.B, 5che.1.A, 3vpo.1.A, 3mjo.1.A, 6gp2.1.B, 1za0.1.A, 2p1i.4.A, 2r2f.1.A, 4bmq.2.A, 1sms.1.A, 1mty.1.C, 6d7k.1.B, 2p1i.1.A, 4iit.1.C, 1jk0.1.B, 1tuw.1.A, 6d9f.1.A, 3dhz.1.B, 3n39.1.A, 1fz4.1.C

SWISS-MODEL Homology Modelling Report

Model Building Report

This document lists the results for the homology modelling project "gene_573135" submitted to SWISS-MODEL workspace on Jan. 23, 2020, 8:41 p.m..The submitted primary amino acid sequence is given in Table T1.

If you use any results in your research, please cite the relevant publications:

- Waterhouse, A., Bertoni, M., Bienert, S., Studer, G., Tauriello, G., Gumienny, R., Heer, F.T., de Beer, T.A.P., Rempfer, C., Bordoli, L., Lepore, R., Schwede, T. SWISS-MODEL: homology modelling of protein structures and complexes. Nucleic Acids Res. 46(W1), W296-W303 (2018).
- Guex, N., Peitsch, M.C., Schwede, T. Automated comparative protein structure modeling with SWISS-MODEL and Swiss-PdbViewer: A historical perspective. Electrophoresis 30, S162-S173 (2009).
- Bienert, S., Waterhouse, A., de Beer, T.A.P., Tauriello, G., Studer, G., Bordoli, L., Schwede, T. The SWISS-MODEL Repository - new features and functionality. Nucleic Acids Res. 45, D313-D319 (2017).
- Benkert, P., Biasini, M., Schwede, T. Toward the estimation of the absolute quality of individual protein structure models. Bioinformatics 27, 343-350 (2011).
- Bertoni, M., Kiefer, F., Biasini, M., Bordoli, L., Schwede, T. Modeling protein quaternary structure of homo- and hetero-oligomers beyond binary interactions by homology. Scientific Reports 7 (2017).

Results

The SWISS-MODEL template library (SMTL version 2020-01-15, PDB release 2020-01-10) was searched with BLAST (Camacho et al.) and HHBlits (Remmert et al.) for evolutionary related structures matching the target sequence in Table T1. For details on the template search, see Materials and Methods. Overall 478 templates were found (Table T2).

Models

The following models were built (see Materials and Methods "Model Building"):

Model #01	File	Built with	Oligo-State	Ligands	GMQE	QMEAN
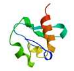	PDB	ProMod3 2.0.0	monomer	None	0.51	-3.88

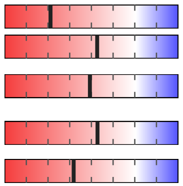

QMEAN	-3.88
Cβ	-1.73
All Atom	-2.07
solvation	-1.72
torsion	-2.82

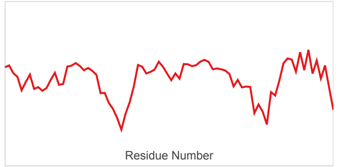

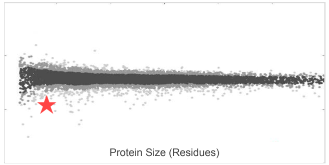

Template	Seq Identity	Oligo-state	QSQE	Found by	Method	Resolution	Seq Similarity	Range	Coverage	Description
1bwe.1.A	36.49	monomer	0.00	HHblits	NMR	-	0.41	4 - 83	0.70	PROTEIN (FERREDOXIN)

Excluded ligands

Ligand Name.Number	Reason for Exclusion	Description
SF4.1	Binding site not conserved.	IRON/SULFUR CLUSTER
SF4.2	Binding site not conserved.	IRON/SULFUR CLUSTER

```
Target     VSVKILLEPCIN--CGLCRKACPTETIHFFTTQHRTHVVEPAGCIDCDICVPICPEKCIVHDDDYVHEPSELAAAKVRAR
1bwe.1.A   ---YVITEPCIGTKCASCVEVCPVDCIHEGEDQ---YYIDPDVCIDCGACEAVCPVSAIYHEDFV---PEEWKSYIQKNR

Target     DWAKRQNVLRQRVKDRAAAAVMAVRRGA
1bwe.1.A   DFFKK-----------------------
```

Model #03	File	Built with	Oligo-State	Ligands	GMQE	QMEAN
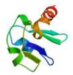	PDB	ProMod3 2.0.0	monomer	None	0.45	-4.10

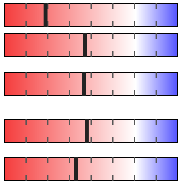

QMEAN	-4.10
Cβ	-2.29
All Atom	-2.32
solvation	-2.20
torsion	-2.70

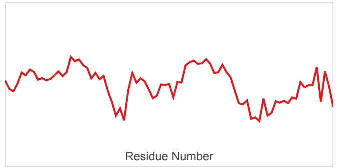

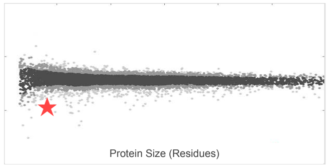

Template	Seq Identity	Oligo-state	QSQE	Found by	Method	Resolution	Seq Similarity	Range	Coverage	Description
2c42.1.B	26.32	monomer	0.00	HHblits	X-ray	1.78Å	0.33	3 - 83	0.72	PYRUVATE-FERREDOXIN OXIDOREDUCTASE

Excluded ligands

Ligand Name.Number	Reason for Exclusion	Description
CA.7	Binding site not conserved.	CALCIUM ION
CA.14	Binding site not conserved.	CALCIUM ION
MG.6	Binding site not conserved.	MAGNESIUM ION
MG.13	Binding site not conserved.	MAGNESIUM ION
PYR.5	Binding site not conserved.	PYRUVIC ACID
PYR.12	Binding site not conserved.	PYRUVIC ACID
SF4.1	Binding site not conserved.	IRON/SULFUR CLUSTER
SF4.2	Binding site not conserved.	IRON/SULFUR CLUSTER
SF4.3	Binding site not conserved.	IRON/SULFUR CLUSTER
SF4.8	Binding site not conserved.	IRON/SULFUR CLUSTER
SF4.9	Binding site not conserved.	IRON/SULFUR CLUSTER
SF4.10	Binding site not conserved.	IRON/SULFUR CLUSTER
TPP.4	Binding site not conserved.	THIAMINE DIPHOSPHATE
TPP.11	Binding site not conserved.	THIAMINE DIPHOSPHATE

```
Target     VSVKILLEPCINCGLCRKACPTETIHFF---T---------------------TQHRTHVVEPAGCIDCDICVPICPE--
```

```
2c42.1.B  --PQWVPENCIQCNQCAFVCPHSAILPVLAKEEELVGAPANFTALEAKGKELKGYKFRIQINTLDCMGCGNCADICPPKE

Target    KCIVHDDDYVHEPSELAAAKVRARDWAKRQNVLRQRVKDRAAAAVMAVRRGA
2c42.1.B  KALVMQPLD-----TQRDAQVPNLEYAAR-----------------------
```

Model #02	File	Built with	Oligo-State	Ligands	GMQE	QMEAN
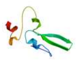	PDB	ProMod3 2.0.0	monomer	None	0.44	-3.44

QMEAN -3.44
Cβ -3.22
All Atom -2.51
solvation -4.13
torsion -0.69

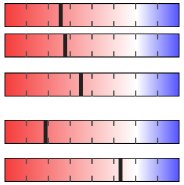

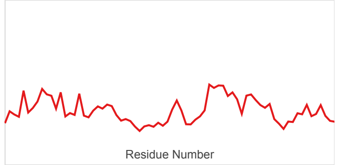

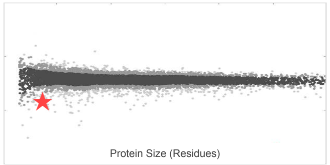

Template	Seq Identity	Oligo-state	QSQE	Found by	Method	Resolution	Seq Similarity	Range	Coverage	Description
2vpz.1.B	30.77	monomer	0.00	HHblits	X-ray	2.40Å	0.35	3 - 74	0.61	NRFC PROTEIN

Excluded ligands

Ligand Name.Number	Reason for Exclusion	Description
MGD.2	Binding site not conserved.	2-AMINO-5,6-DIMERCAPTO-7-METHYL-3,7,8A,9-TETRAHYDRO-8-OXA-1,3,9,10-TETRAAZA-ANTHRACEN-4-ONE GUANOSINE DINUCLEOTIDE
MGD.3	Binding site not conserved.	2-AMINO-5,6-DIMERCAPTO-7-METHYL-3,7,8A,9-TETRAHYDRO-8-OXA-1,3,9,10-TETRAAZA-ANTHRACEN-4-ONE GUANOSINE DINUCLEOTIDE
MGD.10	Binding site not conserved.	2-AMINO-5,6-DIMERCAPTO-7-METHYL-3,7,8A,9-TETRAHYDRO-8-OXA-1,3,9,10-TETRAAZA-ANTHRACEN-4-ONE GUANOSINE DINUCLEOTIDE
MGD.11	Binding site not conserved.	2-AMINO-5,6-DIMERCAPTO-7-METHYL-3,7,8A,9-TETRAHYDRO-8-OXA-1,3,9,10-TETRAAZA-ANTHRACEN-4-ONE GUANOSINE DINUCLEOTIDE
MO.4	Not in contact with model.	MOLYBDENUM ATOM
MO.12	Not in contact with model.	MOLYBDENUM ATOM
SF4.1	Binding site not conserved.	IRON/SULFUR CLUSTER
SF4.5	Binding site not conserved.	IRON/SULFUR CLUSTER
SF4.6	Binding site not conserved.	IRON/SULFUR CLUSTER
SF4.7	Binding site not conserved.	IRON/SULFUR CLUSTER
SF4.8	Binding site not conserved.	IRON/SULFUR CLUSTER
SF4.9	Binding site not conserved.	IRON/SULFUR CLUSTER
SF4.13	Binding site not conserved.	IRON/SULFUR CLUSTER
SF4.14	Binding site not conserved.	IRON/SULFUR CLUSTER
SF4.15	Binding site not conserved.	IRON/SULFUR CLUSTER
SF4.16	Binding site not conserved.	IRON/SULFUR CLUSTER

```
Target    VSVKILLEPCINCGLCRKACPTETIHFFTTQHRTHVVEPAGCIDCD---------ICVPICPEKCIVHDDDYVHEPSELA
2vpz.1.B  --VLVDPKKCIACGACIAACPYDARYLHPA------GYVSKCTFCAHRLEKGKVPACVETCPTYCRTFGDLEDP-ESPVA

Target    AAKVRARDWAKRQNVLRQRVKDRAAAAVMAVRRGA
2vpz.1.B  KAL--------------------------------
```

Model #04	File	Built with	Oligo-State	Ligands	GMQE	QMEAN
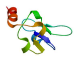	PDB	ProMod3 2.0.0	monomer	None	0.43	-2.10

QMEAN -2.10
Cβ -1.32
All Atom -1.59
solvation -2.14
torsion -0.82

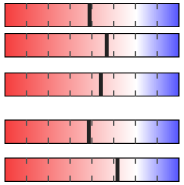

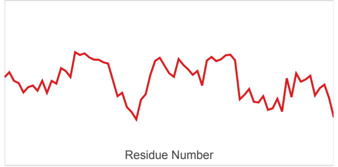

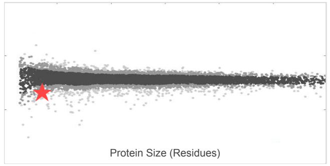

Template	Seq Identity	Oligo-state	QSQE	Found by	Method	Resolution	Seq Similarity	Range	Coverage	Description
2zvs.1.A	27.94	monomer	0.00	HHblits	X-ray	1.65Å	0.36	4 - 74	0.64	Uncharacterized ferredoxin-like protein yfhL

Excluded ligands

Ligand Name.Number	Reason for Exclusion	Description
SF4.1	Binding site not conserved.	IRON/SULFUR CLUSTER
SF4.2	Binding site not conserved.	IRON/SULFUR CLUSTER

```
Target    VSVKILLEPCINCGLCRKACPTETIHFFTTQHRTHVVEPAGCIDCD------ICVPICPE-KCIVHDDDYVHEPSELAAA
2zvs.1.A  ---LLITKKCINCDMCEPECPNEAISMGDHI---YEINSDKCTECVGHYETPTCQKVCPIPNTIVKDPAHVETEEQLWDK

Target    KVRARDWAKRQNVLRQRVKDRAAAAVMAVRRGA
2zvs.1.A  F--------------------------------
```

Model #05	File	Built with	Oligo-State	Ligands	GMQE	QMEAN
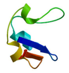	PDB	ProMod3 2.0.0	monomer	None	0.38	-2.89

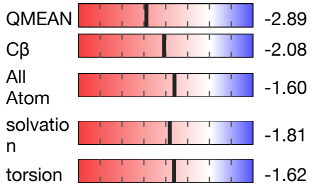

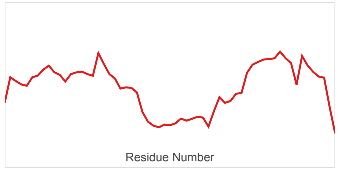

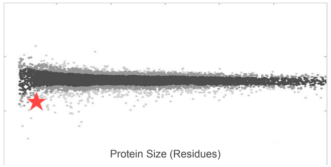

Template	Seq Identity	Oligo-state	QSQE	Found by	Method	Resolution	Seq Similarity	Range	Coverage	Description
6gm0.1.A	21.31	monomer	0.00	HHblits	X-ray	2.11Å	0.34	2 - 62	0.58	Iron hydro-genase 1

Excluded ligands

Ligand Name.Number	Reason for Exclusion	Description
402.1	Binding site not conserved.	dicarbonyl[bis(cyanide-kappaC)]-mu-(iminodimethanethiolatato-1kappaS:2kappaS)-mu-(oxomethylidene)diiron(2+)
FES.6	Binding site not conserved.	FE2/S2 (INORGANIC) CLUSTER
MG.7	Not in contact with model.	MAGNESIUM ION
SF4.2	Binding site not conserved.	IRON/SULFUR CLUSTER
SF4.3	Binding site not conserved.	IRON/SULFUR CLUSTER
SF4.4	Binding site not conserved.	IRON/SULFUR CLUSTER
SF4.5	Binding site not conserved.	IRON/SULFUR CLUSTER

```
Target     VSVKILLEPCINCGLCRKACPT----ETIHFFTTQHR-------THVVEPAGCIDCDICVPICPEKCIVHDDDYVHEPSE
6gm0.1.A   -SLTVDRTKCLLCGRCVNACGKNTETYAMKFLNKNGKTIIGAEDEKCFDDTNCLLCGQCIIACPVAALSEKSH-------

Target     LAAAKVRARDWAKRQNVLRQRVKDRAAAAVMAVRRGA
6gm0.1.A   -------------------------------------
```

Materials and Methods

Template Search

Template search with BLAST and HHBlits has been performed against the SWISS-MODEL template library (SMTL, last update: 2020-01-15, last included PDB release: 2020-01-10).

The target sequence was searched with BLAST against the primary amino acid sequence contained in the SMTL.

An initial HHblits profile has been built using the procedure outlined in (Remmert et al.), followed by 1 iteration of HHblits against NR20. The obtained profile has then be searched against all profiles of the SMTL. A total of 487 templates were found.

Template Selection

For each identified template, the template's quality has been predicted from features of the target-template alignment. The templates with the highest quality have then been selected for model building.

Model Building

Models are built based on the target-template alignment using ProMod3. Coordinates which are conserved between the target and the template are copied from the template to the model. Insertions and deletions are remodelled using a fragment library. Side chains are then rebuilt. Finally, the geometry of the resulting model is regularized by using a force field. In case loop modelling with ProMod3 fails, an alternative model is built with PROMOD-II (Guex et al.).

Model Quality Estimation

The global and per-residue model quality has been assessed using the QMEAN scoring function (Benkert et al.) . For improved performance, weights of the individual QMEAN terms have been trained specifically for SWISS-MODEL.

Ligand Modelling

Ligands present in the template structure are transferred by homology to the model when the following criteria are met: (a) The ligands are annotated as biologically relevant in the template library, (b) the ligand is in contact with the model, (c) the ligand is not clashing with the protein, (d) the residues in contact with the ligand are conserved between the target and the template. If any of these four criteria is not satisfied, a certain ligand will not be included in the model. The model summary includes information on why and which ligand has not been included.

Oligomeric State Conservation

The quaternary structure annotation of the template is used to model the target sequence in its oligomeric form. The method (Bertoni et al.) is based on a supervised machine learning algorithm, Support Vector Machines (SVM), which combines interface conservation, structural clustering, and other template features to provide a quaternary structure quality estimate (QSQE). The QSQE score is a number between 0 and 1, reflecting the expected accuracy of the interchain contacts for a model built based a given alignment and template. Higher numbers indicate higher reliability. This complements the GMQE score which estimates the accuracy of the tertiary structure of the resulting model.

References

- **BLAST**
  Camacho, C., Coulouris, G., Avagyan, V., Ma, N., Papadopoulos, J., Bealer, K., Madden, T.L. BLAST+: architecture and applications. BMC Bioinformatics 10, 421-430 (2009). doi>
- **HHblits**
  Remmert, M., Biegert, A., Hauser, A., Söding, J. HHblits: lightning-fast iterative protein sequence searching by HMM-HMM alignment. Nat Methods 9, 173-175 (2012). doi>

Table T1:

Primary amino acid sequence for which templates were searched and models were built.

```
VSVKILLEPCINCGLCRKACPTETIHFFTTQHRTHVVEPAGCIDCDICVPICPEKCIVHDDDYVHEPSELAAAKVRARDWAKRQNVLRQRVKDRAAAAVM
AVRRGA
```

Table T2:

Template	Seq Identity	Oligo-state	QSQE	Found by	Method	Resolution	Seq Similarity	Coverage	Description
1bwe.1.A	36.49	monomer	-	HHblits	NMR	NA	0.41	0.70	PROTEIN (FERREDOXIN)
1bqx.1.A	36.49	monomer	-	HHblits	NMR	NA	0.41	0.70	PROTEIN (FERREDOXIN)
1bd6.1.A	35.14	monomer	-	HHblits	NMR	NA	0.40	0.70	7-FE FERREDOXIN
1bc6.1.A	35.14	monomer	-	HHblits	NMR	NA	0.40	0.70	7-FE FERREDOXIN
1h98.1.A	33.33	monomer	-	HHblits	X-ray	1.64Å	0.38	0.71	FERREDOXIN
2v2k.1.A	25.97	monomer	-	HHblits	X-ray	1.60Å	0.37	0.73	FERREDOXIN
1kek.1.A	26.32	monomer	-	HHblits	X-ray	1.90Å	0.33	0.72	Pyruvate-Ferredoxin Oxidoreductase
2c42.1.B	26.32	monomer	-	HHblits	X-ray	1.78Å	0.33	0.72	PYRUVATE-FERREDOXIN OXIDOREDUCTASE
3exy.1.A	34.33	monomer	-	HHblits	X-ray	1.48Å	0.39	0.63	Ferredoxin
2vpz.1.B	30.77	monomer	-	HHblits	X-ray	2.40Å	0.35	0.61	NRFC PROTEIN
2fgo.1.A	30.88	monomer	-	HHblits	X-ray	1.32Å	0.37	0.64	Ferredoxin
1rgv.1.A	29.41	monomer	-	HHblits	X-ray	2.90Å	0.36	0.64	ferredoxin
6ciq.2.B	25.33	monomer	-	HHblits	X-ray	3.30Å	0.34	0.71	PYRUVATE-FERREDOXIN OXIDOREDUCTASE
6cin.1.A	25.33	monomer	-	HHblits	X-ray	2.60Å	0.34	0.71	PYRUVATE-FERREDOXIN OXIDOREDUCTASE
1blu.1.A	34.33	monomer	-	HHblits	X-ray	2.10Å	0.40	0.63	FERREDOXIN
1h7x.1.A	30.65	monomer	-	HHblits	X-ray	2.01Å	0.35	0.58	DIHYDROPYRIMIDINE DEHYDROGENASE
5gpn.27.A	28.33	monomer	-	HHblits	EM	NA	0.35	0.57	NADH dehydrogenase [ubi-quinone] iron-sulfur protein 8
3eun.1.A	33.33	monomer	-	HHblits	X-ray	1.05Å	0.39	0.62	Ferredoxin
1h7w.2.A	31.15	monomer	-	HHblits	X-ray	1.90Å	0.36	0.58	DIHYDROPYRIMIDINE DEHYDROGENASE
1gth.1.A	31.15	monomer	-	HHblits	X-ray	2.25Å	0.36	0.58	DIHYDROPYRIMIDINE DEHYDROGENASE
1gt8.1.A	31.15	monomer	-	HHblits	X-ray	3.30Å	0.36	0.58	DIHYDROPYRIMIDINE DEHYDROGENASE
2zvs.2.A	27.94	monomer	-	HHblits	X-ray	1.65Å	0.36	0.64	Uncharacterized ferredoxin-like protein yfhL
2zvs.3.A	27.94	monomer	-	HHblits	X-ray	1.65Å	0.36	0.64	Uncharacterized ferredoxin-like protein yfhL
2zvs.1.A	27.94	monomer	-	HHblits	X-ray	1.65Å	0.36	0.64	Uncharacterized ferredoxin-like protein yfhL
1xer.1.A	30.00	homo-dimer	-	HHblits	X-ray	2.00Å	0.36	0.57	FERREDOXIN
1frm.1.A	30.00	monomer	-	HHblits	X-ray	2.30Å	0.38	0.57	FERREDOXIN
1ftc.1.A	30.51	monomer	-	HHblits	X-ray	2.35Å	0.38	0.56	FERREDOXIN
1fdd.1.A	30.51	monomer	-	HHblits	X-ray	1.90Å	0.38	0.56	FERREDOXIN
6cfw.1.N	20.97	monomer	-	HHblits	EM	3.70Å	0.35	0.58	NADH-plastoquinone oxidore-ductase subunit
1b0t.1.A	30.51	monomer	-	HHblits	X-ray	2.10Å	0.39	0.56	PROTEIN (FERREDOXIN I)
1frl.1.A	30.51	monomer	-	HHblits	X-ray	2.30Å	0.39	0.56	FERREDOXIN
1d3w.1.A	30.51	monomer	-	HHblits	X-ray	1.70Å	0.38	0.56	FERREDOXIN 1
1a6l.1.A	30.00	monomer	-	HHblits	X-ray	2.10Å	0.38	0.57	FERREDOXIN
2fja.1.D	24.59	monomer	-	HHblits	X-ray	2.00Å	0.33	0.58	adenylylsulfate reductase, sub-unit B
2vkr.1.A	28.81	monomer	-	HHblits	X-ray	2.01Å	0.36	0.56	ZINC-CONTAINING FERREDOXIN
6cz7.1.B	27.42	monomer	-	HHblits	X-ray	1.62Å	0.33	0.58	4Fe-4S ferredoxin, iron-sulfur binding domain protein
6gm0.1.A	21.31	monomer	-	HHblits	X-ray	2.11Å	0.34	0.58	Iron hydrogenase 1
6gm2.2.A	21.31	monomer	-	HHblits	X-ray	2.76Å	0.34	0.58	Iron hydrogenase 1
6gm2.1.A	21.31	monomer	-	HHblits	X-ray	2.76Å	0.34	0.58	Iron hydrogenase 1
6hum.1.J	24.59	monomer	-	HHblits	EM	NA	0.36	0.58	NAD(P)H-quinone oxidore-ductase subunit I
6nbq.1.D	24.59	monomer	-	HHblits	EM	NA	0.36	0.58	NAD(P)H-quinone oxidore-ductase subunit I
3lw5.1.C	21.31	monomer	-	HHblits	X-ray	3.30Å	0.33	0.58	Photosystem I iron-sulfur center
4y28.1.H	21.31	monomer	-	HHblits	X-ray	2.80Å	0.33	0.58	Photosystem I iron-sulfur center
1y4z.1.B	20.31	monomer	-	HHblits	X-ray	2.00Å	0.30	0.60	Respiratory nitrate reductase 1 beta chain
1jnz.1.B	24.59	monomer	-	HHblits	X-ray	2.50Å	0.33	0.58	adenylylsulfate reductase
4rku.1.C	20.97	monomer	-	HHblits	X-ray	3.00Å	0.32	0.58	Photosystem I iron-sulfur center
2o01.1.C	20.97	monomer	-	HHblits	X-ray	3.40Å	0.32	0.58	Photosystem I iron-sulfur center
5t5i.1.F	27.87	monomer	-	HHblits	X-ray	1.90Å	0.34	0.58	Tungsten formylmethanofuran dehydrogenase subunit fwdG
6igz.1.C	21.31	monomer	-	HHblits	EM	NA	0.33	0.58	PsaC
1frk.1.A	30.51	monomer	-	HHblits	X-ray	2.10Å	0.39	0.56	FERREDOXIN

The table above shows the top 50 filtered templates. A further 245 templates were found which were considered to be less suitable for modelling than the filtered list.
1fxr.1.B, 5lnk.1.C, 1fxr.1.A, 5lnk.1.G, 2gmh.1.A, 1frl.1.A, 4wz7.1.G, 1bc6.1.A, 1fdn.1.A, 4ytp.1.B, 5ldw.1.G, 1siw.1.B, 1gt8.1.A, 2fgo.1.A, 1rof.1.A, 5la3.1.A, 6gly.1.A, 6gcs.1.I, 1bd6.1.A, 6glz.1.A, 6g2j.1.I, 3mmc.1.A, 3mmc.1.B, 3exy.1.A, 2vpz.1.B, 6g2j.1.G, 6gm3.1.A, 5xmj.1.E, 6kmw.1.C, 1h98.1.A, 1l0v.1.B, 6hqb.1.C, 6h8k.7.A, 2c42.1.B, 6nbq.1.D, 3gyx.1.B, 2z8q.1.A, 4ydd.1.B, 6hum.1.J, 1hfe.1.B, 5zji.1.G, 1pc4.1.A, 1g6b.1.A, 1d3w.1.A, 1ff2.1.A, 5odh.1.A, 3eun.1.A, 5xmj.2.B, 5odh.1.G, 1kek.1.A, 5t61.1.X, 6ijj.1.C, 6pfy.1.C, 4qjv.2.A, 3gyx.3.B, 6igz.1.C, 4qjv.1.A, 3gyx.5.B, 6gcs.1.A, 6qc5.1.C, 5xwd.1.A, 5odc.1.C, 5ma1.1.A, 5zgb.1.C, 1k8v.1.A, 2zvs.2.A, 5xtb.1.L, 1e08.1.A, 4qiw.2.D, 6hcm.35.A, 3p4r.2.B, 6gly.2.A, 1blu.1.A, 3gyx.6.B, 5xtb.1.B, 5lc5.1.G, 3m9s.1.C, 3m9s.1.G, 1dax.1.A, 1frj.1.A, 1gth.1.A, 6gm0.1.A, 4id8.1.A, 5m2g.1.A, 4hea.1.G, 2vkr.1.A, 1pc5.1.A, 6qgr.1.A, 3or1.1.A, 3or1.1.B, 1fd2.1.A, 4xdd.1.A, 6jeo.1.O, 1qlb.1.B, 3bk7.1.A, 3j15.1.B, 1h7w.2.A, 1h0h.1.B, 2o01.1.C, 4fe1.1.C, 4xdd.2.A, 1siz.1.A, 5u8q.1.A, 2fug.2.C, 6cfw.1.N, 4kro.1.A, 4rku.1.C, 1fxd.1.A, 2z8q.2.A, 1bwe.1.A, 1wtf.1.A, 4ras.1.A, 1gao.1.A, 1frx.1.A, 5lzv.79.A, 6cz7.1.B, 4p6v.1.A, 1rgv.1.A, 1g3o.1.A, 6gm2.1.A, 4omf.1.D, 3gyx.4.B, 2ivf.1.B, 3sfd.1.B, 1dur.1.A, 6f0k.1.B, 4ayb.1.D, 6ciq.2.B, 3vr8.1.B, 1f5b.1.A, 4krp.1.A, 5t5i.1.E, 5t5i.1.F, 5xf9.1.B, 1dfd.1.A, 1kqf.1.B, 5t5i.1.K, 2fja.1.D, 1a6l.1.A, 2pa8.1.A, 5u8r.1.D, 1clf.1.A, 1fdd.1.A, 1vjw.1.A, 1y4z.1.B, 1xer.1.A, 1k0t.1.A, 6gm1.1.A, 1dwl.1.A, 1frh.1.A, 6fah.1.E, 5c4i.1.E, 5o31.1.8, 2zvs.1.A, 5odh.1.I, 5d6s.1.A, 3qwq.1.A, 6gm3.2.A, 5ll6.1.T, 4crm.1.A, 1frm.1.A, 2v4j.1.B, 2v4j.1.A, 2b76.2.B, 5exe.1.B, 4ysz.1.B, 2fd2.1.A, 6fah.1.A, 3p4q.2.B, 5exe.1.E, 1f5c.1.A, 1feh.1.A, 5odc.1.A, 2vkr.6.A, 1fca.1.A, 6gm8.1.A, 6btm.1.B, 1frk.1.A, 5d08.1.A, 5t5m.1.E, 5byr.1.A, 3jag.79.A, 2vkr.4.A, 3j16.1.B, 6gm4.1.A, 3egw.1.B, 5lw7.1.A, 1b0v.1.A, 2fbw.1.B, 2xsj.1.A, 4y28.1.H, 1jnz.1.B, 6qii.1.A, 2pmz.2.D, 3abv.1.B, 1h7x.1.A, 2wb1.1.D, 4wz7.1.M, 2bs2.1.B, 1b0t.1.A, 6qcf.1.G, 5gpn.27.A, 6gm1.2.A, 1gx7.1.A, 6qcf.1.C, 6gm2.2.A, 1f2g.1.A, 6h8k.13.A, 4dhv.1.B, 4dhv.1.A, 5xmj.2.E, 3cf4.1.A, 5oef.2.A, 6cin.1.A, 1igr.1.A, 5t8y.2.A, 4v4c.1.B, 1axq.1.A, 5ldx.1.I, 3or2.1.B, 3or2.1.A, 2wp9.1.B, 6fos.1.F, 4z3w.1.E, 5ldx.1.G, 4z3w.1.F, 1y5n.2.E, 1fri.1.A, 4ci0.1.B, 6rfr.1.A, 5dqr.1.A, 3p4s.2.B, 2v2k.1.A, 6rfr.1.I, 4kt0.1.C, 2xsj.1.B, 2acz.1.B, 1bqx.1.A, 2zvs.3.A, 1ftc.1.A, 6rfq.1.I, 3lw5.1.C, 5gpn.24.A, 5xmj.1.B, 6rfq.1.A

SWISS-MODEL Homology Modelling Report

Model Building Report

This document lists the results for the homology modelling project "gene_573134" submitted to SWISS-MODEL workspace on Jan. 23, 2020, 8:40 p.m..The submitted primary amino acid sequence is given in Table T1.

If you use any results in your research, please cite the relevant publications:

- Waterhouse, A., Bertoni, M., Bienert, S., Studer, G., Tauriello, G., Gumienny, R., Heer, F.T., de Beer, T.A.P., Rempfer, C., Bordoli, L., Lepore, R., Schwede, T. SWISS-MODEL: homology modelling of protein structures and complexes. Nucleic Acids Res. 46(W1), W296-W303 (2018). doi>
- Guex, N., Peitsch, M.C., Schwede, T. Automated comparative protein structure modeling with SWISS-MODEL and Swiss-PdbViewer: A historical perspective. Electrophoresis 30, S162-S173 (2009). doi>
- Bienert, S., Waterhouse, A., de Beer, T.A.P., Tauriello, G., Studer, G., Bordoli, L., Schwede, T. The SWISS-MODEL Repository - new features and functionality. Nucleic Acids Res. 45, D313-D319 (2017). doi>
- Benkert, P., Biasini, M., Schwede, T. Toward the estimation of the absolute quality of individual protein structure models. Bioinformatics 27, 343-350 (2011). doi>
- Bertoni, M., Kiefer, F., Biasini, M., Bordoli, L., Schwede, T. Modeling protein quaternary structure of homo- and hetero-oligomers beyond binary interactions by homology. Scientific Reports 7 (2017). doi>

Results

The SWISS-MODEL template library (SMTL version 2020-01-15, PDB release 2020-01-10) was searched with BLAST (Camacho et al.) and HHBlits (Remmert et al.) for evolutionary related structures matching the target sequence in Table T1. For details on the template search, see Materials and Methods. Overall 43 templates were found (Table T2).

Models

The following models were built (see Materials and Methods "Model Building"):

Model #01	File	Built with	Oligo-State	Ligands	GMQE	QMEAN
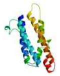	PDB	ProMod3 2.0.0	monomer	None	0.41	-3.70

QMEAN -3.70
Cβ -2.95
All Atom -2.91
solvation -0.49
torsion -2.89

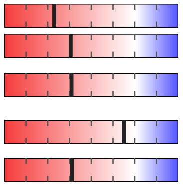

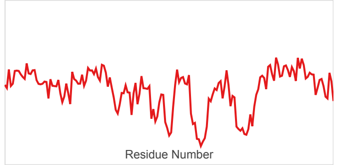

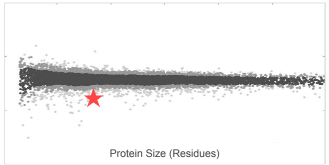

Template	Seq Identity	Oligo-state	QSQE	Found by	Method	Resolution	Seq Similarity	Range	Coverage	Description
4ii4.1.A	17.12	monomer	0.00	HHblits	X-ray	2.80Å	0.28	19 - 185	0.71	1,2-phenylacetyl-CoA epoxidase, subunit A

Excluded ligands

Ligand Name.Number	Reason for Exclusion	Description
BYC.1	Binding site not conserved.	benzoyl coenzyme A
BYC.2	Binding site not conserved.	benzoyl coenzyme A

```
Target    MPELALDPRTIGGAFSVDECARRIINYRFAENVCMTTEGGWASSTPSIKVKFALGEHCYHDSIHSFWLGQRLPELRQ--T
4ii4.1.A  ------------------TLIRQIGQHAHSEIVGMLPQGNWITRAPTLRRKAILLAQVQDEAGHGLYLYSAAETLGCARE

Target    EKMD---YESPPTFRTSYLAEPPNEAFLKFCETMQLQTDQLLRVVGLYRVLKTHLAVYYRHHIQVTDQVSDAPSVRILNH
4ii4.1.A  DIYQKMLDGR-MKYSSI-F-NYPTLS---WADIGVIG--------WLVDGAAIVNQVALC-------RTSYGPYARAMVK

Target    VLLEEEQHLKWGQAMYEELAD-TPERRRAALSWQMELEDLLVKSGGVTGGR
4ii4.1.A  ICKEESFHQRQGFEACMALAQGSEAQKQMLQ--------------------
```

Model #04	File	Built with	Oligo-State	Ligands	GMQE	QMEAN
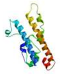	PDB	ProMod3 2.0.0	monomer	None	0.36	-4.69

QMEAN -4.69
Cβ -4.85
All Atom -3.66
solvation -0.72
torsion -3.45

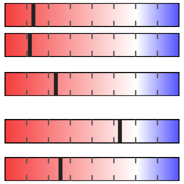

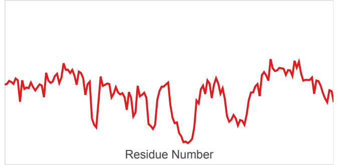

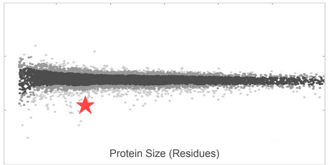

Template	Seq Identity	Oligo-state	QSQE	Found by	Method	Resolution	Seq Similarity	Range	Coverage	Description
3pvt.1.B	17.91	monomer	0.00	HHblits	X-ray	2.03Å	0.29	34 - 185	0.65	Phenylacetic acid degradation protein paaC

Excluded ligands

Ligand Name.Number	Reason for Exclusion	Description
3HC.1	Binding site not conserved.	3-HYDROXYBUTANOYL-COENZYME A
3HC.6	Binding site not conserved.	3-HYDROXYBUTANOYL-COENZYME A
GOL.2	Not biologically relevant.	GLYCEROL
GOL.3	Not biologically relevant.	GLYCEROL
GOL.4	Not biologically relevant.	GLYCEROL
GOL.5	Not biologically relevant.	GLYCEROL
GOL.7	Not biologically relevant.	GLYCEROL
GOL.8	Not biologically relevant.	GLYCEROL
GOL.9	Not biologically relevant.	GLYCEROL
GOL.10	Not biologically relevant.	GLYCEROL

```
Target    MPELALDPRTIGGAFSVDECARRIINYRFAENVCMTTEGGWASSTPSIKVKFALGEHCYHDSIHSFWLGQRLPELR---Q
3pvt.1.B  ---------------------------------LSQRLGEWCGHAPELEIDLALANIGLDLLGQARNFLSYAAELAGEGD
```

```
Target    TEKMDYESPP-TFRTSYLAEPPNEAFLKFCETMQLQTDQLLRVVGLYRVLKTHLAVYYRHHIQVTDQVSDAPSVRILNHV
3pvt.1.B  EDTLAFTRDERQFSNLLLVEQPNGNFA---DTIAR---QY-----FIDAWHVALFTRLM-------ESRDPQLAAISAKA

Target    LLEEEQHLKWGQAMYEELAD-TPERRRAALSWQMELEDLLVKSGGVTGGR
3pvt.1.B  IKEARYHLRFSRGWLERLGNGTDVSGQKMQ--------------------
```

Model #05	File	Built with	Oligo-State	Ligands	GMQE	QMEAN
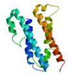	PDB	ProMod3 2.0.0	monomer (matching prediction)	None	0.36	-4.03

QMEAN -4.03
Cβ -3.27
All Atom -2.07
solvation -1.17
torsion -3.02

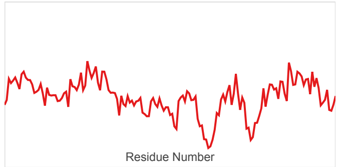

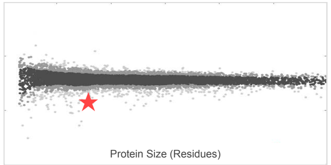

Template	Seq Identity	Oligo-state	QSQE	Found by	Method	Resolution	Seq Similarity	Range	Coverage	Description
4mud.1.A	13.48	homo-dimer	0.01	HHblits	X-ray	2.43Å	0.27	22 - 178	0.69	Ring oxydation complex/ phenylacetic acid degradation related protein

The template contained no ligands.

```
Target    MPELALDPRTIGGAFSVDECARRIINYRFAENVCMTTEGGWASSTPSIKVKFALGEHCYHDSIHSFWLGQRLPELRQTEK
4mud.1.A  ---------------------NIIELRADFELAMVEQYSPWLVNAPTVDSRLFVAKLVSDELNHGWQLVRLLEEFKVKDV

Target    MDYESPPTFRTSYLAEPPNEAFLKFCETMQLQTDQLLRVVGLYRVLKTHLAVYYRHHIQVTDQVSDAPSVRILNHVLLEE
4mud.1.A  IERISNA-RLGIHKLEVSNLPLFNWEDVIAFT--------FLVDGAGLYQLKILK-------DCSFEPLSTLASSMIKEE

Target    EQHLKWGQAMYEELADTPERRRAALSWQMELEDLLVKSGGVTGGR
4mud.1.A  ESHIFFSQNELRNYQNKN---------------------------
```

Model #03	File	Built with	Oligo-State	Ligands	GMQE	QMEAN
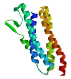	PDB	ProMod3 2.0.0	monomer (matching prediction)	None	0.35	-4.15

QMEAN -4.15

Cβ -3.54
All Atom -1.11
solvation -0.06
torsion -3.49

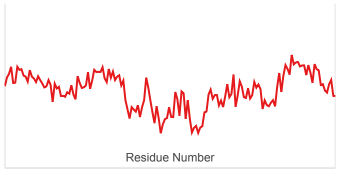

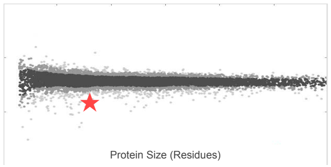

Template	Seq Identity	Oligo-state	QSQE	Found by	Method	Resolution	Seq Similarity	Range	Coverage	Description
3ogh.1.A	11.19	homo-dimer	0.05	HHblits	X-ray	1.65Å	0.26	18 - 177	0.70	Protein yciE

Excluded ligands

Ligand Name.Number	Reason for Exclusion	Description
CL.3	Not biologically relevant.	CHLORIDE ION
FE.2	Binding site not conserved.	FE (III) ION
FE.5	Binding site not conserved.	FE (III) ION
MG.1	Binding site not conserved.	MAGNESIUM ION
MG.4	Binding site not conserved.	MAGNESIUM ION

```
Target    MPELALDPRTIGGAFSVDECARRIINYRFAENVCMTTEGGWAS-STPSIKVKFALGEHCYHDSIHSFWLGQRLPELRQTE
3ogh.1.A  -----------------EHYHDWLRDAHAMEKQAESMLESMASRIDNYPELRARIEQHLSETKNQIVQLETILDRNDI--

Target    KMDYESPPTFRTSYLAEPPNEAFLKFCETMQLQ-----TDQLLRVVGLYRVLKTHLA-VYYRHHIQVTDQVSDAPSVRIL
3ogh.1.A  ------SRS----V---IK-DSMSKMAALGQSIGGIFPSDEIVKGS-ISGYVFEQFEIACYTSLLAAAKNAGDTASIPTI

Target    NHVLLEEEQHLKWGQAMYEELADTPERRRAALSWQMELEDLLVKSGGVTGGR
3ogh.1.A  EAILNEEKHMADWLIQHIPQTTEK----------------------------
```

Model #02	File	Built with	Oligo-State	Ligands	GMQE	QMEAN
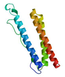	PDB	ProMod3 2.0.0	monomer (matching prediction)	None	0.31	-4.91

QMEAN -4.91
Cβ -2.44
All Atom -1.62
solvation 0.05
torsion -4.64

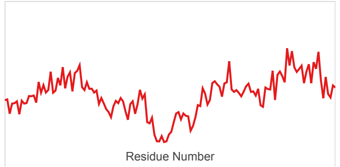

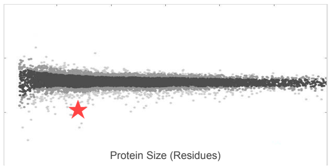

Template	Seq Identity	Oligo-state	QSQE	Found by	Method	Resolution	Seq Similarity	Range	Coverage	Description
		homo-						39 -		Alr3090

4r42.1.A	12.12	hexamer	0.00	HHblits	X-ray	1.90Å	0.26	176	0.64	protein

Excluded ligands

Ligand Name.Number	Reason for Exclusion	Description
CA.3	Binding site not conserved.	CALCIUM ION
CA.4	Binding site not conserved.	CALCIUM ION
CA.9	Binding site not conserved.	CALCIUM ION
CA.10	Binding site not conserved.	CALCIUM ION
CA.14	Binding site not conserved.	CALCIUM ION
CA.19	Binding site not conserved.	CALCIUM ION
CA.20	Binding site not conserved.	CALCIUM ION
CA.25	Binding site not conserved.	CALCIUM ION
CA.26	Binding site not conserved.	CALCIUM ION
CA.30	Binding site not conserved.	CALCIUM ION
MN.1	Binding site not conserved.	MANGANESE (II) ION
MN.2	Binding site not conserved.	MANGANESE (II) ION
MN.7	Binding site not conserved.	MANGANESE (II) ION
MN.8	Binding site not conserved.	MANGANESE (II) ION
MN.12	Binding site not conserved.	MANGANESE (II) ION
MN.13	Binding site not conserved.	MANGANESE (II) ION
MN.17	Binding site not conserved.	MANGANESE (II) ION
MN.18	Binding site not conserved.	MANGANESE (II) ION
MN.23	Binding site not conserved.	MANGANESE (II) ION
MN.24	Binding site not conserved.	MANGANESE (II) ION
MN.28	Binding site not conserved.	MANGANESE (II) ION
MN.29	Binding site not conserved.	MANGANESE (II) ION
P6G.16	Not biologically relevant.	HEXAETHYLENE GLYCOL
P6G.32	Not biologically relevant.	HEXAETHYLENE GLYCOL
PEG.11	Not biologically relevant.	DI(HYDROXYETHYL)ETHER
PEG.27	Not biologically relevant.	DI(HYDROXYETHYL)ETHER
PG4.6	Not biologically relevant.	TETRAETHYLENE GLYCOL
PG4.22	Not biologically relevant.	TETRAETHYLENE GLYCOL
PGE.5	Not biologically relevant.	TRIETHYLENE GLYCOL
PGE.15	Not biologically relevant.	TRIETHYLENE GLYCOL
PGE.21	Not biologically relevant.	TRIETHYLENE GLYCOL
PGE.31	Not biologically relevant.	TRIETHYLENE GLYCOL

```
Target     MPELALDPRTIGGAFSVDECARRIINYRFAENVCMTTEGGWASSTPSIKVKFALGEHCYHDSIHSFWLGQRLPELRQTEK
4r42.1.A   --------------------------------------QSFHVENA--GIKDMLQDIAIEEFSHLEMVGKLIEAHTKNVD

Target     MD-YESPPTFRTSYLAEPPNEAFL-KFC-ETMQLQTDQLLRVVGLYRVLKTHLAVYYRHHIQVTDQVSDAPSVRILNHVL
4r42.1.A   QTEAYKSTLFAVRGMGPHFLDSQGNAWTASYLNEGG-D---VVRDLRANIAAEAGARQTYEELIKLSPDEGTKQTLVHLL

Target     LEEEQHLKWGQAMYEELADTPERRRAALSWQMELEDLLVKSGGVTGGR
4r42.1.A   TREISHTQMFMKALDSLGK-----------------------------
```

Model #06	File	Built with	Oligo-State	Ligands	GMQE	QMEAN
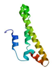	PDB	ProMod3 2.0.0	monomer (matching prediction)	None	0.25	-2.94

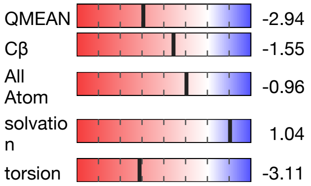

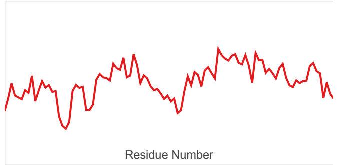

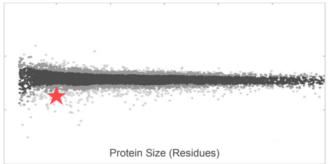

Template	Seq Identity	Oligo-state	QSQE	Found by	Method	Resolution	Seq Similarity	Range	Coverage	Description
1t0s.1.B	12.50	homo-dimer	0.04	HHblits	X-ray	2.20Å	0.29	93 - 190	0.47	toluene, o-xylene monooxygenase oxygenase subunit

Excluded ligands

Ligand Name.Number	Reason for Exclusion	Description
BML.5	Binding site not conserved.	4-BROMOPHENOL
BML.6	Binding site not conserved.	4-BROMOPHENOL
BML.7	Binding site not conserved.	4-BROMOPHENOL
BML.8	Binding site not conserved.	4-BROMOPHENOL
BML.9	Binding site not conserved.	4-BROMOPHENOL
BML.10	Binding site not conserved.	4-BROMOPHENOL
BML.15	Binding site not conserved.	4-BROMOPHENOL
BML.16	Binding site not conserved.	4-BROMOPHENOL
BML.17	Binding site not conserved.	4-BROMOPHENOL
BML.18	Binding site not conserved.	4-BROMOPHENOL
BML.19	Binding site not conserved.	4-BROMOPHENOL
BML.20	Binding site not conserved.	4-BROMOPHENOL
FE.1	Binding site not conserved.	FE (III) ION
FE.2	Binding site not conserved.	FE (III) ION
FE.11	Binding site not conserved.	FE (III) ION
FE.12	Binding site not conserved.	FE (III) ION
MCR.4	Binding site not conserved.	SULFANYLACETIC ACID
MCR.14	Binding site not conserved.	SULFANYLACETIC ACID
OH.3	Binding site not conserved.	HYDROXIDE ION
OH.13	Binding site not conserved.	HYDROXIDE ION

```
Target     MPELALDPRTIGGAFSVDECARRIINYRFAENVCMTTEGGWASSTPSIKVKFALGEHCYHDSIHSFWLGQRLPELRQTEK
1t0s.1.B   --------------------------------------------------------------------------------

Target     MDYESPPTFRTSYLAEPPNEAFLKFCETMQLQ--TDQLLRVVGLYRVLKTHLAVYYRHHIQ-VTDQVSDAPSVRILNHVL
```

```
1t0s.1.B   ------------WENDPAWQGFRELIEKALIAWDWGEA--FTAINLVTKPAVEEALLQQLGSLAQSEGDTLLGLLAQAQK

Target     LEEEQHLKWGQAMYEELADTPERRRAALSWQMELEDLLVKSGGVTGGR
1t0s.1.B   RDAERHRRWSSALVKMALEKEGNREVLQKWVAK---------------
```

Materials and Methods

Template Search

Template search with BLAST and HHBlits has been performed against the SWISS-MODEL template library (SMTL, last update: 2020-01-15, last included PDB release: 2020-01-10).

The target sequence was searched with BLAST against the primary amino acid sequence contained in the SMTL.

An initial HHblits profile has been built using the procedure outlined in (Remmert et al.), followed by 1 iteration of HHblits against NR20. The obtained profile has then be searched against all profiles of the SMTL. A total of 44 templates were found.

Template Selection

For each identified template, the template's quality has been predicted from features of the target-template alignment. The templates with the highest quality have then been selected for model building.

Model Building

Models are built based on the target-template alignment using ProMod3. Coordinates which are conserved between the target and the template are copied from the template to the model. Insertions and deletions are remodelled using a fragment library. Side chains are then rebuilt. Finally, the geometry of the resulting model is regularized by using a force field. In case loop modelling with ProMod3 fails, an alternative model is built with PROMOD-II (Guex et al.).

Model Quality Estimation

The global and per-residue model quality has been assessed using the QMEAN scoring function (Benkert et al.) . For improved performance, weights of the individual QMEAN terms have been trained specifically for SWISS-MODEL.

Ligand Modelling

Ligands present in the template structure are transferred by homology to the model when the following criteria are met: (a) The ligands are annotated as biologically relevant in the template library, (b) the ligand is in contact with the model, (c) the ligand is not clashing with the protein, (d) the residues in contact with the ligand are conserved between the target and the template. If any of these four criteria is not satisfied, a certain ligand will not be included in the model. The model summary includes information on why and which ligand has not been included.

Oligomeric State Conservation

The quaternary structure annotation of the template is used to model the target sequence in its oligomeric form. The method (Bertoni et al.) is based on a supervised machine learning algorithm, Support Vector Machines (SVM), which combines interface conservation, structural clustering, and other template features to provide a quaternary structure quality estimate (QSQE). The QSQE score is a number between 0 and 1, reflecting the expected accuracy of the interchain contacts for a model built based a given alignment and template. Higher numbers indicate higher reliability. This complements the GMQE score which estimates the accuracy of the tertiary structure of the resulting model.

References

- **BLAST**
  Camacho, C., Coulouris, G., Avagyan, V., Ma, N., Papadopoulos, J., Bealer, K., Madden, T.L. BLAST+: architecture and applications. BMC Bioinformatics 10, 421-430 (2009). doi>
- **HHblits**
  Remmert, M., Biegert, A., Hauser, A., Söding, J. HHblits: lightning-fast iterative protein sequence searching by HMM-HMM alignment. Nat Methods 9, 173-175 (2012). doi>

Table T1:

Primary amino acid sequence for which templates were searched and models were built.

```
MPELALDPRTIGGAFSVDECARRIINYRFAENVCMTTEGGWASSTPSIKVKFALGEHCYHDSIHSFWLGQRLPELRQTEKMDYESPPTFRTSYLAEPPNE
AFLKFCETMQLQTDQLLRVVGLYRVLKTHLAVYYRHHIQVTDQVSDAPSVRILNHVLLEEEQHLKWGQAMYEELADTPERRRAALSWQMELEDLLVKSGG
VTGGR
```

Table T2:

Template	Seq Identity	Oligo-state	QSQE	Found by	Method	Resolution	Seq Similarity	Coverage	Description
4ii4.1.A	17.12	monomer	-	HHblits	X-ray	2.80Å	0.28	0.71	1,2-phenylacetyl-CoA epoxidase, subunit A
4iit.1.A	18.49	monomer	-	HHblits	X-ray	4.30Å	0.29	0.71	Phenylacetate-CoA oxygenase subunit PaaA
3pvt.1.A	17.81	monomer	-	HHblits	X-ray	2.03Å	0.28	0.71	Phenylacetic acid degradation protein paaA
3pvt.1.B	17.91	monomer	-	HHblits	X-ray	2.03Å	0.29	0.65	Phenylacetic acid degradation protein paaC
3pvt.2.A	17.91	monomer	-	HHblits	X-ray	2.03Å	0.29	0.65	Phenylacetic acid degradation protein paaC
4iit.1.C	20.30	monomer	-	HHblits	X-ray	4.30Å	0.29	0.65	Phenylacetate-CoA oxygenase subunit PaaC
1otk.1.A	18.18	homo-dimer	-	HHblits	X-ray	2.00Å	0.29	0.64	Phenylacetic acid degradation protein paaC
4mud.1.A	13.48	homo-dimer	0.01	HHblits	X-ray	2.43Å	0.27	0.69	Ring oxydation complex/ phenylacetic acid degradation re-lated protein
3ogh.1.A	11.19	homo-dimer	0.05	HHblits	X-ray	1.65Å	0.26	0.70	Protein yciE
3ogh.1.B	11.19	homo-dimer	0.03	HHblits	X-ray	1.65Å	0.26	0.70	Protein yciE
4r42.1.A	12.12	homo-hexamer	-	HHblits	X-ray	1.90Å	0.26	0.64	Alr3090 protein
6j42.1.A	12.12	homo-hexamer	-	HHblits	X-ray	2.49Å	0.26	0.64	Alr3090 protein
3ge8.1.B	16.67	homo-dimer	0.08	HHblits	X-ray	2.19Å	0.30	0.47	Toluene-4-monooxygenase sys-tem protein E
5tdv.1.B	16.67	homo-dimer	0.06	HHblits	X-ray	2.00Å	0.30	0.47	Toluene-4-monooxygenase sys-tem protein E
1t0s.1.B	12.50	homo-dimer	0.04	HHblits	X-ray	2.20Å	0.29	0.47	toluene, o-xylene monooxy-genase oxygenase subunit
3rmk.1.B	16.49	monomer	-	HHblits	X-ray	1.95Å	0.30	0.47	Toluene-4-monooxygenase sys-tem protein E
3q3o.1.B	16.49	homo-dimer	0.11	HHblits	X-ray	1.95Å	0.30	0.47	Toluene-4-monooxygenase sys-tem protein E
5tdu.1.B	16.67	homo-dimer	0.08	HHblits	X-ray	1.74Å	0.30	0.47	Toluene-4-monooxygenase sys-tem protein E
									toluene 4-monooxygenase hy-

3dhg.2.B	16.67	monomer	-	HHblits	X-ray	1.85Å	0.30	0.47	droxylase beta subunit
3dhg.1.B	16.67	monomer	-	HHblits	X-ray	1.85Å	0.30	0.47	toluene 4-monooxygenase hy-droxylase beta subunit
3dhh.1.B	16.67	monomer	-	HHblits	X-ray	1.94Å	0.30	0.47	toluene 4-monooxygenase hy-droxylase beta subunit
2inc.1.B	12.50	homo-dimer	0.04	HHblits	X-ray	1.85Å	0.29	0.47	Toluene, o-xylene monooxy-genase oxygenase subunit
2ind.1.B	12.50	homo-dimer	-	HHblits	X-ray	2.20Å	0.29	0.47	Toluene, o-xylene monooxy-genase oxygenase subunit
1cnt.2.B	14.46	homo-dimer	-	HHblits	X-ray	2.40Å	0.25	0.40	CILIARY NEUROTROPHIC FACTOR
1cnt.1.A	14.46	homo-dimer	-	HHblits	X-ray	2.40Å	0.25	0.40	CILIARY NEUROTROPHIC FACTOR
1cnt.1.B	14.46	homo-dimer	-	HHblits	X-ray	2.40Å	0.25	0.40	CILIARY NEUROTROPHIC FACTOR
1cnt.2.A	14.46	homo-dimer	-	HHblits	X-ray	2.40Å	0.25	0.40	CILIARY NEUROTROPHIC FACTOR
1u7m.1.A	15.38	homo-dimer	0.01	HHblits	NMR	NA	0.31	0.19	Four-helix bundle model
1u7m.1.B	15.38	homo-dimer	0.01	HHblits	NMR	NA	0.31	0.19	Four-helix bundle model
5vju.1.A	15.00	monomer	-	HHblits	X-ray	2.08Å	0.30	0.20	Reaction Center Maquette Leu71His variant
1mft.1.A	15.38	homo-dimer	0.11	HHblits	X-ray	2.50Å	0.31	0.19	Four-helix bundle model
1mft.1.B	15.38	homo-dimer	0.11	HHblits	X-ray	2.50Å	0.31	0.19	Four-helix bundle model
5c39.1.A	15.79	homo-dimer	0.12	HHblits	X-ray	1.75Å	0.31	0.19	P0 Manganese cluster peptide
5c39.1.B	15.79	homo-dimer	0.12	HHblits	X-ray	1.75Å	0.31	0.19	P0 Manganese cluster peptide
5vjs.1.A	15.00	monomer	-	HHblits	X-ray	2.00Å	0.29	0.20	Reaction Center Maquette
5vjt.1.A	15.00	monomer	-	HHblits	X-ray	1.45Å	0.29	0.20	Reaction Center Maquette
2lfd.1.A	13.16	monomer	-	HHblits	NMR	NA	0.32	0.19	Diiron protein
2lff.1.A	13.16	monomer	-	HHblits	NMR	NA	0.32	0.19	Diiron protein
1umq.1.A	17.74	monomer	-	HHblits	NMR	NA	0.28	0.30	PHOTOSYNTHETIC APPARATUS REGULATORY PROTEIN

The table above shows the top 39 filtered templates. A further 4 templates were found which were considered to be less suitable for modelling than the filtered list.
3ri7.1.B, 5grq.1.C, 5tds.1.B, 5grq.1.D

I-TASSER results for job id S520517

(Click on S520517_results.tar.bz2 to download the tarball file including all modeling results listed on this page. Click on Annotation of I-TASSER Output to read the instructions for how to interpret the results on this page. Model results are kept on the server for 60 days, there is no way to retrieve the modeling data older than 2 months)

Submitted Sequence in FASTA format

```
>Gene_573136_mmoX
MSTAFAEQVRTLVSRRDPAYRPAMFIDEAEKKALAEKLGDASNYDLGLLREMVAQDPDNY
EFRKALVSGIVSAEYAGIDAFSRKVCEWQTWPVPWELIMAMARQTWDEVRHAQLGTELLK
SYGGEVGEYPDTLAGSPPNQATAAVAQGQGADVMRDPVISLSVTNVALEGAALDLFKGTS
DLGRKVNDDLMRLVYDYNWADEVTHTAIGDYFVKVLCDGNPAEEARALRAHAMFEGMRER
LSGQQSAEIREFFAEEIERGTAALAGNG
```

Predicted Secondary Structure

```
                   20                  40                  60                  80                 100                 120                 140                 160                 180                 200                 220                 240                 260
                    |                   |                   |                   |                   |                   |                   |                   |                   |                   |                   |                   |                   |
Sequence   MSTAFAEQVRTLVSRRDPAYRPAMFIDEAEKKALAEKLGDASNYDLGLLREMVAQDPDNYEFRKALVSGIVSAEYAGIDAFSRKVCEWQTWPVPWELIMAMARQTWDEVRHAQLGTELLKSYGGEVGEYPDTLAGSPPNQATAAVAQGQGADVMRDPVISLSVTNVALEGAALDLFKGTSDLGRKVNDDLMRLVYDYNWADEVTHTAIGDYFVKVLCDGNPAEEARALRAHAMFEGMRERLSGQQSAEIREFFAEEIERGTAALAGNG
Prediction CCHHHHCCCCCCCCCCCCCCCCCCCCCCCCCCCCCCCCCCCCCCCCCHHHHCCCCCCCCHHHHHHHHHHHHHHHHHHHHHHHHHHHHCCCCCCCHHHHHHHHHHHHHHHHHHHHHHHHHHHHCCCCCCCCCCCCHHHHHHHHHCCCCCCCCCCCCCHHHHHHHHHHHHHHHCCCCCHHHHHHHHHCCCHHHHHHHHHHHHHHHHHHHHHHHHHHHHHCCCCHHHHHHHHHHHHCCCCCCCCCHHHHHHHHHHHHHHHHHHHHHHCCCC
Conf.Score 9357752254434557987686545566789998555897311121026651677899877899999999999999899999999997067788699999999999999999999999998299868863313289999898500000010010059999999999999972788768999999978988899999998999999999889999999983694899999999982678999999899999995789988501666 52589
           H:Helix; S:Strand; C:Coil
```

Predicted Solvent Accessibility

```
                   20                  40                  60                  80                 100                 120                 140                 160                 180                 200                 220                 240                 260
                    |                   |                   |                   |                   |                   |                   |                   |                   |                   |                   |                   |                   |
Sequence   MSTAFAEQVRTLVSRRDPAYRPAMFIDEAEKKALAEKLGDASNYDLGLLREMVAQDPDNYEFRKALVSGIVSAEYAGIDAFSRKVCEWQTWPVPWELIMAMARQTWDEVRHAQLGTELLKSYGGEVGEYPDTLAGSPPNQATAAVAQGQGADVMRDPVISLSVTNVALEGAALDLFKGTSDLGRKVNDDLMRLVYDYNWADEVTHTAIGDYFVKVLCDGNPAEEARALRAHAMFEGMRERLSGQQSAEIREFFAEEIERGTAALAGNG
Prediction 6431335414432443412342443263673254263263664352333451344447346210000000001102100000000000114737113300320040031003003002400461324001002333333333113210443154135202110001000310221021331142047262630130042016100300220120021006542362034004412445033212462045036114430443343237 58
           Values range from 0 (buried residue) to 9 (highly exposed residue)
```

Predicted normalized B-factor

(B-factor is a value to indicate the extent of the inherent thermal mobility of residues/atoms in proteins. In I-TASSER, this value is deduced from threading template proteins from the PDB in combination with the sequence profiles derived from sequence databases. The reported B-factor profile in the figure below corresponds to the normalized B-factor of the target protein, defined by B=(B'-u)/s, where B' is the raw B-factor value, u and s are respectively the mean and standard deviation of the raw B-factors along the sequence. Click here to read more about predicted normalized B-factor)

Top 10 threading templates used by I-TASSER

(I-TASSER modeling starts from the structure templates identified by LOMETS from the PDB library. LOMETS is a meta-server threading approach containing multiple threading programs, where each threading program can generate tens of thousands of template alignments. I-TASSER only uses the templates of the highest significance in the threading alignments, the significance of which are measured by the Z-score, i.e. the difference between the raw and average scores in the unit of standard deviation. The templates in this section are the 10 best templates selected from the LOMETS threading programs. Usually, one template of the highest Z-score is selected from each threading program, where the threading programs are sorted by the average performance in the large-scale benchmark test experiments.)

Rank	PDB Hit	Iden1	Iden2	Cov	Norm. Z-score	Download Align.	Alignment
						Sec.Str	CCHHHHCCCCCCCCCCCCCCCCCCCCCCCCCCCCCCCCCCCCCCCCCHHHHCCCCCCCCHHHHHHHHHHHHHHHHHHHHHHHHHHHHCCCCCCCHHHHHHHHHHHHHHHHHHHHHHHHHHHHCCCCCCCCCCCCHHHHHHHHHCCCCCCCCCCCCCHHHHHHHHHHHHHHHCCCCCHHHHHHHHHCCCHHHHHHHHHHHHHHHHHHHHHHHHHHHHHCCCCHHHHHHHHHHHHCCCCCCCCCHHHHHHHHHHHHHHHHHHHHHHCCCC
						Seq	MSTAFAEQVRTLVSRRDPAYRPAMFIDEAEKKALAEKLGDASNYDLGLLREMVAQDPDNYEFRKALVSGIVSAEYAGIDAFSRKVCEWQTWPVPWELIMAMARQTWDEVRHAQLGTELLKSYGGEVGEYPDTLAGSPPNQATAAVAQGQGADVMRDPVISLSVTNVALEGAALDLFKGTSDLGRKVNDDLMRLVYDYNWADEVTHTAIGDYFVKVLCDGNPAEEARALRAHAMFEGMRERLSGQQSAEIREFFAEEIERGTAALAGNG
1	6d9fA	0.15	0.20	0.97	0.95	Download	SAPGLLEGAMTLDLTPE-QCDLGYWLRGVAQGTLAGRAETGHTDAEPTPEHM-RADGPLRDAQVLELSCRSVAEAQATRVLAHYVAQAPD----IVELEFFTTQLVDEARHSMVFRRHLLAMGVPADRLSIAEVSAEYRREVLEPILDFALTTEGDFVGGVAVFTIIIEGVLAPAAELSERKWNLL-DPAAGAIARGAAIDEVRHLTVGSSVVRRHLLRRPKAALLDIVRRGR-EIWDGIPDRKHVLRREELFQAGMREHADLLAGPG
2	6d9fA	0.15	0.20	0.77	1.76	Download	-------------------------------AGRAETGHTDA----EPTPEHMRADGPLRDAQVLELSCRSVAEAQATRVLAHYVAQAPD----IVELEFFTTQLVDEARHSMVFRRHLLAMGVPADRLHRREVLEPILDFALTTVRDE-----GDFVGGVAVFTIIIEGVLAPAAELSERKWNL-LDPAAGAIARGAAIDEVRHLTVGSSVVRRHLLRRPEAALLDIVRRGR------------EIWDGIPDRKHVLRREEL----
3	6d9fA	0.14	0.20	0.96	0.95	Download	SAPGLLEGAMTL--DLTPEQCDLGYWLRGVAQGTLAGRAETGHTDAEPTPEHMRADGPLRDAQVLELSCRSVAEAQATRVLAHYVAQAPD----IVELEFFTTQLVDEARHSMVFRRHLLAMGVPVSAEYRREVLEPILDFALTTVRDEG-----DFVGGVAVFTIIIEGVLAPAAELSERKWNLL-DPAAGAIARGAAIDEVRHLTVGSSVVRRHLLRRPKAALLDIVRRGREIWPDRKHVLRELFQAGMREHEQRYAMAEQWTDRM
4	4hr0	0.12	0.19	0.94	1.96	Download	-----------VHHDGFQTVKATIDWEHPMFKLYEKAKRNGKWNPA-DIDFSQDTSEEKISALPLVAGFSAGEEAVTLDILPMAHALARQGR-LEDVLFLTTFMHDEAKHVEMFSRWQQAVGIGQMDLSVNDHYKEALP-EAMNRLYA-DDSPEAVIRAATVYNMIVEGTLESGYYTFRQIYKAGLFPGLLQGIDYLNMDEGRHIQFGIYTIQRIVNEDERYYELFIRYMDELWPHVIGYVDYLTEYALEIDYDLLRHYVIKKKQIS
5	5hyg	0.11	0.22	0.87	1.14	Download	LVGNWHRRAAVK------------FD-PGRPDFREDMIPFR--------GHPIWERLSDETWVAYNRNTVLIEQRIANPAFELVIGGAYGLGGQQLELAVAQAMVDEQYHTLMHINGSAVTRSDFPDSHITTIHQEHLDRCE--------EPWQRSLTTLGFA-TVAEIS---IN-AYLDLADQEIQVVNSTTVKLHNRDEYCHASISGEMMKQVYEALADRFLLEKVVAGLEAFVAPDFTTWESIVAFEGVPGKAAAEVREAQ-GG
6	6d9fA	0.14	0.20	0.98	1.25	Download	-DSAPGLLEGAMTLDLTPEQCDLGYWLRGVAQGTLAGRAETGHTDAEPTPEHMRADGPLRDAQVLELSCRSVAEAQATRVLAHYVAQAPD----IVELEFFTTQLVDEARHSMVFRRHLLAMGVPASIAEVSAEYRREVLEPILDFALTTVRDEGDFVGGVAVFTIIIEGVLAPAAELSERKWNLL-DPAAGAIARGAAIDEVRHLTVGSSVVRRHLLRRPERKAALLDIVRRGREIWDGIPDRKHVLRREELFQAGMREHADLLAGY
7	1mhz	0.16	0.21	0.59	1.73	Download	---HPRWGETMKNFLEVGEYNAIAASAMLWDSATA----AEQKNGYLAQVLDEIRHTHQCAFINHYYSKHYHDPAGHND-ARRTRAIGPLWKGMADGFSGDAVECSVNLQLVGEACFTNPLIAVTEWASANGDEITPTVFLSVETDELRHMANGYQTVVSIA-NDP---
8	6d9fA	0.14	0.20	0.89	1.21	Download	EQCDLGYWLRGVAQGTLAGRAETGHTDAEPTPEH-----------------MRADGPLRDAQVLELSCRSVAEAQATRVLAHYVAQAPD----IVELEFFTTQLVDEARHSMVFRRHLLAMGVPADRLHASIAEVSAEYRREVDFALTTVRDEGDFVGGVAVFTIIIEGVLAPAA-ELSERKWNLLDPAAGAIARGAAIDEVRHLTVGSSVVRRHLLRRPERKAALLDIVRRGREIWDGIP------DRKHVLRREELFQAGMREHA
9	6d9fA	0.13	0.20	0.96	0.99	Download	IPAETDSAPGLLEGAMTLDLTPE-QCDLAQGTLAGRAETGHTDAEPTPEHM--RADGPLRDAQVLELSCRSVAEAQATRVLAHYVAQAPD----IVELEFFTTQLVDEARHSMVFRRHLLAMGVPADR--LHASIAEVSAEYRREVLEPILDDEGDFVGGVAVFTIIIEGVLAPA-AELSERKWNLLDPAAGAIARGAAIDEVRHLTVGSSVVRRHLLRRPKAALLDIVRRGREGIPDRKHVLRREELFQAGMRE--ADLLAGYVWPG
10	5hygA	0.12	0.22	0.93	1.26	Download	NAVIVGNWHRRAAVKFDP-----------GRPDFREDMIPFRGHPIWERLSDETRSRLLSWGWVAYNRNTVLIEQRIANPAFELVIGGAPGLGGQQLELAVAQAMVDEQYHTLGSAVTRRMRRSDFSDRVLPDSHITTIHQEHLDRCEEPWQR---SLTTLGFATVAEISI-----NAYLDLLADDQEVVNSTTVKLHNRDEYCHASISGEMMKQVYEALPADRLLEKVVAGLEAFVAPDFTTWESIVAFEGVWEKAAAEVREAQGGT

(a) All the residues are colored in black; however, those residues in template which are identical to the residue in the query sequence are highlighted in color. Coloring scheme is based on the property of amino acids, where polar are brightly coloured while non-polar residues are colored in dark shade. (more about the colors used)
(b) Rank of templates represents the top ten threading templates used by I-TASSER.
(c) Ident1 is the percentage sequence identity of the templates in the threading aligned region with the query sequence.
(d) Ident2 is the percentage sequence identity of the whole template chains with query sequence.
(e) Cov represents the coverage of the threading alignment and is equal to the number of aligned residues divided by the length of query protein.
(f) Norm. Z-score is the normalized Z-score of the threading alignments. Alignment with a Normalized Z-score >1 mean a good alignment and vice versa.
(g) Download Align. provides the 3D structure of the aligned regions of the threading templates.
(h) The top 10 alignments reported above (in order of their ranking) are from the following threading programs:
1: MUSTER 2: FFAS-3D 3: SPARKS-X 4: HHSEARCH2 5: HHSEARCH I 6: Neff-PPAS 7: HHSEARCH 8: pGenTHREADER 9: wdPPAS 10: PROSPECT2

Top 5 final models predicted by I-TASSER

(For each target, I-TASSER simulations generate a large ensemble of structural conformations, called decoys. To select the final models, I-TASSER uses the SPICKER program to cluster all the decoys based on the pair-wise structure similarity, and reports up to five models which corresponds to the five largest structure clusters. The confidence of each model is quantitatively measured by C-score that is calculated based on the significance of threading template alignments and the convergence parameters of the structure assembly simulations. C-score is typically in the range of [-5, 2], where a C-score of a higher value signifies a model with a higher confidence and vice-versa. TM-score and RMSD are estimated based on C-score and protein length following the correlation observed between these qualities. Since the top 5 models are ranked by the cluster size, it is possible that the lower-rank models have a higher C-score in rare cases. Although the first model has a better quality in most cases, it is also possible that the lower-rank models have a better quality than the higher-rank models as seen in our benchmark tests. If the I-TASSER simulations converge, it is possible to have less than 5 clusters generated; this is usually an indication that the models have a good quality because of the converged simulations.)

- More about C-score
- Local structure accuracy profile of the top five models

(By right-click on the images, you can export image file or change the configurations, e.g. modifying the background color or stopping the spin of your models)

- Download Model 1
- C-score=-1.24 (Read more about C-score)
- Estimated TM-score = 0.56±0.15
- Estimated RMSD = 8.7±4.6Å

- Download Model 2
- C-score = -1.85

- Download Model 3
- C-score = -3.50

- Download Model 4
- C-score = -4.17

- Download Model 5
- C-score = -4.55

Proteins structurally close to the target in the PDB (as identified by TM-align)

(After the structure assembly simulation, I-TASSER uses the TM-align structural alignment program to match the first I-TASSER model to all structures in the PDB library. This section reports the top 10 proteins from the PDB that have the closest structural similarity, i.e. the highest TM-score, to the predicted I-TASSER model. Due to the structural similarity, these proteins often have similar function to the target. However, users are encouraged to use the data in the next section 'Predicted function using COACH' to infer the function of the target protein, since COACH has been extensively trained to derive biological functions from multi-source of sequence and structure features which has on average a higher accuracy than the function annotations derived only from the global structure comparison.)

Top 10 Identified stuctural analogs in PDB

Click to view	Rank	PDB Hit	TM-score	RMSD[a]	IDEN[a]	Cov	Alignment
	1	6d9fA	0.890	1.98	0.139	0.966	Download
	2	4hr0A	0.766	3.16	0.113	0.907	Download
	3	3ee4A	0.763	3.10	0.109	0.907	Download
	4	1mhyB	0.757	2.96	0.110	0.899	Download
	5	1mxrA	0.756	2.95	0.083	0.892	Download
	6	1fz0D	0.754	3.02	0.102	0.899	Download
	7	4bmoA	0.750	3.01	0.084	0.892	Download
	8	4d8gA	0.748	3.06	0.106	0.895	Download
	9	4dr0A	0.746	3.05	0.059	0.892	Download
	10	1uzrB	0.741	2.85	0.065	0.869	Download

(a) Query structure is shown in cartoon, while the structural analog is displayed using backbone trace.
(b) Ranking of proteins is based on TM-score of the structural alignment between the query structure and known structures in the PDB library.
(c) RMSD[a] is the RMSD between residues that are structurally aligned by TM-align.
(d) IDEN[a] is the percentage sequence identity in the structurally aligned region.
(e) Cov represents the coverage of the alignment by TM-align and is equal to the number of structurally aligned residues divided by length of the query protein.

Predicted function using COFACTOR and COACH

(This section reports biological annotations of the target protein by COFACTOR and COACH based on the I-TASSER structure prediction. While COFACTOR deduces protein functions (ligand-binding sites, EC and GO) using structure comparison and protein-protein networks, COACH is a meta-server approach that combines multiple function annotation results (on ligand-binding sites) from the COFACTOR, TM-SITE and S-SITE programs.)

Ligand binding sites

Click to view	Rank	C-score	Cluster size	PDB Hit	Lig Name	Download Complex	Ligand Binding Site Residues
	1	0.35	208	1afrA	FE2	Rep, Mult	104,108,169,202,205
	2	0.31	163	1bcfA	MN	Rep, Mult	74,108,111,202
	3	0.07	38	4kvsA	6NA	Rep, Mult	73,74,77,108,164,168,169,172,202
	4	0.03	19	4pgkA	Y69	Rep, Mult	69,73,74,77,108,164,168,169,171,172,175,176,236,242,245
	5	0.02	11	3chtA	4NB	Rep, Mult	66,70,73,74,108,169,173,176,198,202

Download the residue-specific ligand binding probability, which is estimated by SVM.
Download the all possible binding ligands and detailed prediction summary.
Download the templates clustering results.
(a) **C-score** is the confidence score of the prediction. C-score ranges [0-1], where a higher score indicates a more reliable prediction.
(b) **Cluster size** is the total number of templates in a cluster.
(c) **Lig Name** is name of possible binding ligand. Click the name to view its information in the BioLiP database.
(d) **Rep** is a single complex structure with the most representative ligand in the cluster, i.e., the one listed in the **Lig Name** column.
Mult is the complex structures with all potential binding ligands in the cluster.

Enzyme Commission (EC) numbers and active sites

Click to view	Rank	$Cscore^{EC}$	PDB Hit	TM-score	RMSD[a]	IDEN[a]	Cov	EC Number	Active Site Residues
	1	0.284	1xvgC	0.752	3.05	0.102	0.899	1.14.13.25	31,75,112
	2	0.279	1mhyB	0.757	2.96	0.110	0.899	1.14.13.25	75,112
	3	0.261	1fziA	0.720	3.44	0.122	0.895	1.14.13.25	NA
	4	0.260	1mhyD	0.720	3.44	0.134	0.895	1.14.13.25	NA
	5	0.248	3ee4A	0.763	3.10	0.109	0.907	1.17.4.1	NA

Click on the radio buttons to visualize predicted active site residues.
(a) $Cscore^{EC}$ is the confidence score for the EC number prediction. $Cscore^{EC}$ values range in between [0-1]; where a higher score indicates a more reliable EC number prediction.
(b) TM-score is a measure of global structural similarity between query and template protein.
(c) RMSD[a] is the RMSD between residues that are structurally aligned by TM-align.
(d) IDEN[a] is the percentage sequence identity in the structurally aligned region.
(e) Cov represents the coverage of global structural alignment and is equal to the number of structurally aligned residues divided by length of the query protein.

Gene Ontology (GO) terms

Top 10 homologous GO templates in PDB

Rank	$Cscore^{GO}$	TM-score	RMSD[a]	IDEN[a]	Cov	PDB Hit	Associated GO Terms
1	0.37	0.7191	3.11	0.09	0.87	1h0nA	GO:0009186 GO:0005515 GO:0051290 GO:0046914 GO:0051259 GO:0009262 GO:0046872 GO:0055114 GO:0004748 GO:0005737 GO:0016491 GO:0006260 GO:0009263
2	0.36	0.7523	3.05	0.10	0.90	1fyzC	GO:0055114 GO:0006730 GO:0015049 GO:0006725 GO:0016709 GO:0004497 GO:0016491 GO:0046914
3	0.33	0.7371	2.94	0.08	0.87	3n37A	GO:0006260 GO:0055114 GO:0046914 GO:0004748 GO:0009186 GO:0016491 GO:0030145 GO:0046872
4	0.29	0.7203	3.44	0.12	0.90	1fziA	GO:0016491 GO:0015049 GO:0055114 GO:0004497 GO:0046872 GO:0006730 GO:0006725 GO:0046914
5	0.26	0.7630	3.10	0.11	0.91	3ee4A	GO:0046872 GO:0005829 GO:0016491 GO:0055114 GO:0004748 GO:0005506 GO:0009186 GO:0030145 GO:0042803 GO:0046914
6	0.25	0.7360	3.00	0.07	0.88	1oquC	GO:0009186 GO:0016491 GO:0046872 GO:0055114 GO:0004748 GO:0046914
7	0.25	0.7283	3.13	0.08	0.88	2bq1I	GO:0009186 GO:0006260 GO:0046872 GO:0016491 GO:0004748 GO:0046914 GO:0055114
8	0.24	0.7568	2.96	0.11	0.90	1mhyB	GO:0016491 GO:0004497 GO:0006730 GO:0015049 GO:0055114 GO:0006725 GO:0016709 GO:0046914
9	0.24	0.7482	3.09	0.07	0.90	1biqB	GO:0004748 GO:0006260 GO:0055114 GO:0005829 GO:0005515 GO:0016491 GO:0046872 GO:0009186 GO:0046914
10	0.23	0.7368	3.11	0.10	0.87	3n1xB	GO:0046914 GO:0055114 GO:0016709 GO:0004497 GO:0016491 GO:0006725

Consensus prediction of GO terms

**Molecular Function**	GO:0004748	GO:0015049	GO:0042802	GO:0030145			
**GO-Score**	0.69	0.54	0.51	0.50			
**Biological Process**	GO:0055114	GO:0009186	GO:0006260	GO:0006725	GO:0006730	GO:0009263	GO:0051290
**GO-Score**	0.86	0.69	0.58	0.54	0.54	0.36	0.36
**Cellular Component**	GO:0044444						
**GO-Score**	0.51						

(a) $Cscore^{GO}$ is a combined measure for evaluating global and local similarity between query and template protein. It's range is [0-1] and higher values indicate more confident predictions.
(b) TM-score is a measure of global structural similarity between query and template protein.
(c) RMSD[a] is the RMSD between residues that are structurally aligned by TM-align.
(d) IDEN[a] is the percentage sequence identity in the structurally aligned region.
(e) Cov represents the coverage of global structural alignment and is equal to the number of structurally aligned residues divided by length of the query protein.
(f) The second table shows a consensus GO terms amongst the top scoring templates. The GO-Score associated with each prediction is defined as the average weight of the GO term, where the weights are assigned based on $Cscore^{GO}$ of the template.

[Click on S520517_results.tar.bz2 to download the tarball file including all modeling results listed on this page]

Please cite the following articles when you use the I-TASSER server:

1. J Yang, Y Zhang. I-TASSER server: new development for protein structure and function predictions, Nucleic Acids Research, 43: W174-W181, 2015.
2. C Zhang, PL Freddolino, Y Zhang. COFACTOR: improved protein function prediction by combining structure, sequence and protein–protein interaction information. Nucleic Acids Research, 45: W291-W299, 2017.

I-TASSER results for job id S523043

(Click on S523043_results.tar.bz2 to download the tarball file including all modeling results listed on this page. Click on Annotation of I-TASSER Output to read the instructions for how to interpret the results on this page. Model results are kept on the server for 60 days, there is no way to retrieve the modeling data older than 2 months)

Submitted Sequence in FASTA format

```
Gene_573135
VSVKILLEPCINCGLCRKACPTETIHFFTTQHRTHVVEPAGCIDCDICVPICPEKCIVHD
DDYVHEPSELAAAKVRARDWAKRQNVLRQRVKDRAAAAVMAVRRGA
```

Predicted Secondary Structure

```
                              20                  40                  60                  80                 100
                               |                   |                   |                   |                   |
Sequence   VSVKILLEPCINCGLCRKACPTETIHFFTTQHRTHVVEPAGCIDCDICVPICPEKCIVHDDDYVHEPSELAAAKVRARDWAKRQNVLRQRVKDRAAAAVMAVRRGA
Prediction CSSSSCCCCCCCCCHHHHHCCHHHCCCCCCCCSSSSSCHHHCCCCCCCCCCCCCCSSSCCCCCCCCHHHHHHHHHHHHHHHHHHHHHHHHHHHHHCCCHHHHCCCC
Conf.Score 9448887259883964524928433403688732898830377998462408807345578877899999998999999898589999888766315255542389
           H:Helix; S:Strand; C:Coil
```

Predicted Solvent Accessibility

```
                              20                  40                  60                  80                 100
                               |                   |                   |                   |                   |
Sequence   VSVKILLEPCINCGLCRKACPTETIHFFTTQHRTHVVEPAGCIDCDICVPICPEKCIVHDDDYVHEPSELAAAKVRARDWAKRQNVLRQRVKDRAAAAVMAVRRGA
Prediction 7534035551341534364321400333467633130236443737363653236104347444544541344444254356445334444645454534546678
           Values range from 0 (buried residue) to 9 (highly exposed residue)
```

Predicted normalized B-factor

(B-factor is a value to indicate the extent of the inherent thermal mobility of residues/atoms in proteins. In I-TASSER, this value is deduced from threading template proteins from the PDB in combination with the sequence profiles derived from sequence databases. The reported B-factor profile in the figure below corresponds to the normalized B-factor of the target protein, defined by B=(B'-u)/s, where B' is the raw B-factor value, u and s are respectively the mean and standard deviation of the raw B-factors along the sequence. Click here to read more about predicted normalized B-factor)

Top 10 threading templates used by I-TASSER

(I-TASSER modeling starts from the structure templates identified by LOMETS from the PDB library. LOMETS is a meta-server threading approach containing multiple threading programs, where each threading program can generate tens of thousands of template alignments. I-TASSER only uses the templates of the highest significance in the threading alignments, the significance of which are measured by the Z-score, i.e. the difference between the raw and average scores in the unit of standard deviation. The templates in this section are the 10 best templates selected from the LOMETS threading programs. Usually, one template of the highest Z-score is selected from each threading program, where the threading programs are sorted by the average performance in the large-scale benchmark test experiments.)

Rank	PDB Hit	Iden1	Iden2	Cov	Norm. Z-score	Download Align.	Alignment (20 / 40 / 60 / 80 / 100)
						Sec.Str	CSSSSCCCCCCCCCHHHHHCCHHHCCCCCCCCSSSSSCHHHCCCCCCCCCCCCCCSSSCCCCCCCCHHHHHHHHHHHHHHHHHHHHHHHHHHHHHCCCHHHHCCCC
						Seq	VSVKILLEPCINCGLCRKACPTETIHFFTTQHRTHVVEPAGCIDCDICVPICPEKCIVHDDDYVHEPSELAAAKVRARDWAKRQNVLRQRVKDRAAAAVMAVRRGA
1	1axqA	0.21	0.24	0.92	3.32	Download	--AFVVTDNCIKCKYCVEVCPVDCFYEGP---NFLVIHPDECIDCALCEPECPAQAIFSEDEVPEDMQEFIQLNAELAEV---WPNITEKKDPLPDAEDWDGVKGK
2	1fd2A	0.21	0.23	0.91	1.19	Download	---FVVTDNCIKCKYCVEVAPVDCFY---EGPNFLVIHPDECIDCALCEPECPAQAIFSEDEVPEDMQEFIQLNAELA---EVWPNITEKKDPLPDAEDWDGVKG-
3	3eunA	0.33	0.24	0.71	2.73	Download	-ALMIT-DECINCDVCEPECPNGAISQGDE---TYVIEPSLCTECSQCVEVCPVDAIIKDPSHEETEDELRAKYERITGE-------------------------
4	1kek	0.18	0.26	0.96	1.28	Download	NVPQWVPENCIQCNQCAFVCPHSAILPVLAGYFRIQINTLDCMGCGNCADICPPKALVMQPLDTQRDAQVPNL----EYAARIPVKGETPYVRVITQLFGERATGP
5	1kek	0.18	0.26	0.98	0.97	Download	NVPQWVPENCIQCNQCAFVCPHSAILPVLGYKFRIQINTLDCMGCGNCADICPPKALVMQPLDTQRDA--QVPNLEYAARIPVKSEVLPRDSLKGSQEPLMEFSGA
6	1pc5A	0.20	0.23	0.92	4.19	Download	--AFVVTDNCIKCTDCVEVCPVDCFYEGPN---FLVIHPDECIDCALCEPECGAQAIFSEDEVPEDMQEFIQLNAELAEV---WPNITEKKDPLPDAEDWDGVKGK
7	1kek	0.25	0.26	0.59	1.33	Download	--PQWVPENCIQCNQCAFVCPHSAILPVLGYKFRIQINTLDCMGCGNCADICPPKALVMQPLDTQ--
8	7fd1A	0.21	0.24	0.92	1.11	Download	--AFVVTDNCIKCKYCVEVCPVDCFYEG---PNFLVIHPDECIDCALCEPECPAQAIFSEDEVPEDMQ---EFIQLNAELAEVWPNITEKKDPLPDAEDWDGVKGK
9	2v2kA	0.22	0.22	0.94	3.12	Download	--TYVIAEPCVDVKDCIEECPVDCIY---EGARMLYIHPDECV-DGACEPVCPVEAIYYEDDVPDQWSSYAQANADFFAEGSPGGASKVGQTDNDPQAIKDLPPQG
10	1bluA	0.34	0.24	0.70	1.43	Download	A--LMITDECINCDVCEPECPNGAIS---QGDETYVIEPSLCTECSQCVEVCPVDCIIKDPSHEETEDELRAKYERIT-------------------------G

(a) All the residues are colored in black; however, those residues in template which are identical to the residue in the query sequence are highlighted in color. Coloring scheme is based on the property of amino acids, where polar are brightly coloured while non-polar residues are colored in dark shade. (more about the colors used)
(b) Rank of templates represents the top ten threading templates used by I-TASSER.
(c) Ident1 is the percentage sequence identity of the templates in the threading aligned region with the query sequence.
(d) Ident2 is the percentage sequence identity of the whole template chains with query sequence.
(e) Cov represents the coverage of the threading alignment and is equal to the number of aligned residues divided by the length of query protein.
(f) Norm. Z-score is the normalized Z-score of the threading alignments. Alignment with a Normalized Z-score >1 mean a good alignment and vice versa.
(g) Download Align. provides the 3D structure of the aligned regions of the threading templates.
(h) The top 10 alignments reported above (in order of their ranking) are from the following threading programs:
1: MUSTER 2: FFAS-3D 3: SPARKS-X 4: HHSEARCH2 5: HHSEARCH I 6: Neff-PPAS 7: HHSEARCH 8: pGenTHREADER 9: wdPPAS 10: PROSPECT2

Top 5 final models predicted by I-TASSER

(For each target, I-TASSER simulations generate a large ensemble of structural conformations, called decoys. To select the final models, I-TASSER uses the SPICKER program to cluster all the decoys based on the pair-wise structure similarity, and reports up to five models which corresponds to the five largest structure clusters. The confidence of each model is quantitatively measured by C-score that is calculated based on the significance of threading template alignments and the convergence parameters of the structure assembly simulations. C-score is typically in the range of [-5, 2], where a C-score of a higher value signifies a model with a higher confidence and vice-versa. TM-score and RMSD are estimated based on C-score and protein length following the correlation observed between these qualities. Since the top 5 models are ranked by the cluster size, it is possible that the lower-rank models have a higher C-score in rare cases. Although the first model has a better quality in most cases, it is also possible that the lower-rank models have a better quality than the higher-rank models as seen in our benchmark tests. If the I-TASSER simulations converge, it is possible to have less than 5 clusters generated; this is usually an indication that the models have a good quality because of the converged simulations.)

- More about C-score
- Local structure accuracy profile of the top five models

(By right-click on the images, you can export image file or change the configurations, e.g. modifying the background color or stopping the spin of your models)

- Download Model 1
- C-score=-0.43 (Read more about C-score)
- Estimated TM-score = 0.66±0.13
- Estimated RMSD = 4.9±3.2Å

- Download Model 2
- C-score = -2.27

- Download Model 3
- C-score = -1.74

- Download Model 4
- C-score = -3.17

- Download Model 5
- C-score = -3.58

Proteins structurally close to the target in the PDB (as identified by TM-align)

(After the structure assembly simulation, I-TASSER uses the TM-align structural alignment program to match the first I-TASSER model to all structures in the PDB library. This section reports the top 10 proteins from the PDB that have the closest structural similarity, i.e. the highest TM-score, to the predicted I-TASSER model. Due to the structural similarity, these proteins often have similar function to the target. However, users are encouraged to use the data in the next section 'Predicted function using COACH' to infer the function of the target protein, since COACH has been extensively trained to derive biological functions from multi-source of sequence and structure features which has on average a higher accuracy than the function annotations derived only from the global structure comparison.)

Top 10 Identified stuctural analogs in PDB

Click to view	Rank	PDB Hit	TM-score	RMSD[a]	IDEN[a]	Cov	Alignment
	1	1a6l_	0.787	1.76	0.202	0.887	Download
	2	2v2kB	0.705	2.39	0.210	0.830	Download
	3	1h98A	0.664	0.89	0.267	0.698	Download
	4	2c3yA2	0.627	3.19	0.210	0.821	Download
	5	2c3pB	0.625	3.18	0.210	0.821	Download
	6	1bqxA	0.616	1.69	0.280	0.708	Download
	7	6cinA2	0.614	2.95	0.198	0.783	Download
	8	1h0hB	0.531	3.27	0.134	0.755	Download
	9	2vpxF	0.530	3.06	0.157	0.726	Download
	10	1durA	0.492	0.93	0.273	0.519	Download

(a) Query structure is shown in cartoon, while the structural analog is displayed using backbone trace.
(b) Ranking of proteins is based on TM-score of the structural alignment between the query structure and known structures in the PDB library.
(c) RMSD[a] is the RMSD between residues that are structurally aligned by TM-align.
(d) IDEN[a] is the percentage sequence identity in the structurally aligned region.
(e) Cov represents the coverage of the alignment by TM-align and is equal to the number of structurally aligned residues divided by length of the query protein.

Predicted function using COFACTOR and COACH

(This section reports biological annotations of the target protein by COFACTOR and COACH based on the I-TASSER structure prediction. While COFACTOR deduces protein functions (ligand-binding sites, EC and GO) using structure comparison and protein-protein networks, COACH is a meta-server approach that combines multiple function annotation results (on ligand-binding sites) from the COFACTOR, TM-SITE and S-SITE programs.)

Ligand binding sites

Click to view	Rank	C-score	Cluster size	PDB Hit	Lig Name	Download Complex	Ligand Binding Site Residues
	1	0.49	124	1durA	SF4	Rep, Mult	6,10,11,12,13,14,15,16,35,52,53,54,56,57
	2	0.48	111	2zvsC	SF4	Rep, Mult	4,20,21,22,24,25,37,42,43,44,45,46,47,48
	3	0.01	2	1gx7A	HEC	N/A	11,13,31,53
	4	0.00	1	1GT8A	1GT8A01	Rep, Mult	1,43,44
	5	0.00	1	1E08A	1E08A00	Rep, Mult	93,94,96,97,98,99,101,102

Download the residue-specific ligand binding probability, which is estimated by SVM.
Download the all possible binding ligands and detailed prediction summary.
Download the templates clustering results.
(a) **C-score** is the confidence score of the prediction. C-score ranges [0-1], where a higher score indicates a more reliable prediction.
(b) **Cluster size** is the total number of templates in a cluster.
(c) **Lig Name** is name of possible binding ligand. Click the name to view its information in the BioLiP database.
(d) **Rep** is a single complex structure with the most representative ligand in the cluster, i.e., the one listed in the **Lig Name** column.
Mult is the complex structures with all potential binding ligands in the cluster.

Enzyme Commission (EC) numbers and active sites

Click to view	Rank	$Cscore^{EC}$	PDB Hit	TM-score	RMSD[a]	IDEN[a]	Cov	EC Number	Active Site Residues
	1	0.431	2fugA	0.493	3.14	0.198	0.670	1.6.99.5	43
	2	0.390	1kqfB	0.495	3.84	0.118	0.717	1.2.1.2	43
	3	0.242	1b0pA	0.626	3.06	0.210	0.811	1.2.7.1	NA
	4	0.241	2pdaA	0.623	3.06	0.210	0.811	1.2.7.1	NA
	5	0.225	1jnrB	0.512	1.65	0.140	0.566	1.8.99.2	NA

Click on the radio buttons to visualize predicted active site residues.
(a) $Cscore^{EC}$ is the confidence score for the EC number prediction. $Cscore^{EC}$ values range in between [0-1]; where a higher score indicates a more reliable EC number prediction.
(b) TM-score is a measure of global structural similarity between query and template protein.
(c) RMSD[a] is the RMSD between residues that are structurally aligned by TM-align.
(d) IDEN[a] is the percentage sequence identity in the structurally aligned region.
(e) Cov represents the coverage of global structural alignment and is equal to the number of structurally aligned residues divided by length of the query protein.

Gene Ontology (GO) terms

Top 10 homologous GO templates in PDB

Rank	$Cscore^{GO}$	TM-score	RMSD[a]	IDEN[a]	Cov	PDB Hit	Associated GO Terms
1	0.46	0.6639	0.89	0.27	0.70	1h98A	GO:0051536 GO:0006810 GO:0051539 GO:0046872 GO:0022900 GO:0051538 GO:0009055
2	0.42	0.5279	3.08	0.16	0.73	2vpwB	GO:0046872 GO:0009055 GO:0051536 GO:0051539
3	0.32	0.6060	1.91	0.28	0.71	1bc6A	GO:0051539 GO:0046872 GO:0051538 GO:0051536 GO:0006810 GO:0022900 GO:0009055
4	0.31	0.7872	1.76	0.20	0.89	1a6lA	GO:0006810 GO:0051536 GO:0051538 GO:0046872 GO:0051539 GO:0003677 GO:0022900 GO:0009055
5	0.29	0.4626	2.04	0.12	0.55	1sizA	GO:0046872 GO:0006810 GO:0051539 GO:0051538 GO:0022900 GO:0051536 GO:0005506 GO:0009055
6	0.29	0.7126	2.42	0.22	0.84	2v2kA	GO:0006810 GO:0022900 GO:0046872 GO:0051536 GO:0051538 GO:0051539 GO:0009055
7	0.24	0.6258	3.06	0.21	0.81	1b0pA	GO:0055114 GO:0051536 GO:0022900 GO:0006810 GO:0051539 GO:0046872 GO:0016491 GO:0003824 GO:0005506 GO:0008152 GO:0009055 GO:0016903 GO:0030976
8	0.20	0.5314	3.27	0.13	0.75	1h0hB	GO:0042597 GO:0046872 GO:0051539 GO:0022900 GO:0006810 GO:0051536
9	0.19	0.4923	0.93	0.27	0.52	1durA	GO:0009055 GO:0051536
10	0.19	0.4878	1.00	0.33	0.52	1fcaA	GO:0051539 GO:0022900 GO:0046872 GO:0006810 GO:0009055 GO:0051536

Consensus prediction of GO terms

**Molecular Function**	GO:0051539	GO:0009055	GO:0051538	GO:0046914	GO:0003677
**GO-Score**	0.90	0.90	0.82	0.45	0.31
**Biological Process**	GO:0006810	GO:0022900			
**GO-Score**	0.82	0.82			
**Cellular Component**	None was predicted				

(a) $Cscore^{GO}$ is a combined measure for evaluating global and local similarity between query and template protein. It's range is [0-1] and higher values indicate more confident predictions.
(b) TM-score is a measure of global structural similarity between query and template protein.
(c) RMSD[a] is the RMSD between residues that are structurally aligned by TM-align.
(d) IDEN[a] is the percentage sequence identity in the structurally aligned region.
(e) Cov represents the coverage of global structural alignment and is equal to the number of structurally aligned residues divided by length of the query protein.
(f) The second table shows a consensus GO terms amongst the top scoring templates. The GO-Score associated with each prediction is defined as the average weight of the GO term, where the weights are assigned based on $Cscore^{GO}$ of the template.

[Click on S523043_results.tar.bz2 to download the tarball file including all modeling results listed on this page]

Please cite the following articles when you use the I-TASSER server:

1. J Yang, Y Zhang. I-TASSER server: new development for protein structure and function predictions, Nucleic Acids Research, 43: W174-W181, 2015.
2. C Zhang, PL Freddolino, Y Zhang. COFACTOR: improved protein function prediction by combining structure, sequence and protein–protein interaction information. Nucleic Acids Research, 45: W291-W299, 2017.

[Home] [Server] [Queue] [About] [Remove] [Statistics]

I-TASSER results for job id S522614

(Click on S522614_results.tar.bz2 to download the tarball file including all modeling results listed on this page. Click on Annotation of I-TASSER Output to read the instructions for how to interpret the results on this page. Model results are kept on the server for 60 days, there is no way to retrieve the modeling data older than 2 months)

Submitted Sequence in FASTA format

```
>Gene_573134
MPELALDPRTIGGAFSVDECARRIINYRFAENVCMTTEGGWASSTPSIKVKFALGEHCYH
DSIHSFWLGQRLPELRQTEKMDYESPPTFRTSYLAEPPNEAFLKFCETMQLQTDQLLRVV
GLYRVLKTHLAVYYRHHIQVTDQVSDAPSVRILNHVLLEEEQHLKWGQAMYEELADTPER
RRAALSWQMELEDLLVKSGGVTGGR
```

Predicted Secondary Structure

```
                    20                  40                  60                  80                 100                 120                 140                 160                 180                 200
                     |                   |                   |                   |                   |                   |                   |                   |                   |                   |
Sequence   MPELALDPRTIGGAFSVDECARRIINYRFAENVCMTTEGGWASSTPSIKVKFALGEHCYHDSIHSFWLGQRLPELRQTEKMDYESPPTFRTSYLAEPPNEAFLKFCETMQLQTDQLLRVVGLYRVLKTHLAVYYRHHIQVTDQVSDAPSVRILNHVLLEEEQHLKWGQAMYEELADTPERRRAALSWQMELEDLLVKSGGVTGGR
Prediction CCCCCCCHHHHCCCCCHHHHHHHHHHHHHHHHHHHHHHHHHHHHCCCCHHHHHHHHHHHHHHHHHHHHHHHHHHHHHHHHHHHHCCCCCCCCCCCCCCCCHHCCCCHHHHHHHHHHHHHCCCCCHHHHHHHHHHHHHHHHHHHHHHHHHHHHHHCCCCCCCCHHHHHHHHHHHHHHHHHHHHHHHHHHHHHHCCCHHHHHHHHHHHHHHHHHHHHHHHHCCCCCCC
Conf.Score 9511137554078774999999999999999999999998720245418999999999999999899999999999989852455565346998672005787188999999998506870689999999999999999999999986365444547999999998599999999999999998616277889999999999999999826987899
           H:Helix; S:Strand; C:Coil
```

Predicted Solvent Accessibility

```
                    20                  40                  60                  80                 100                 120                 140                 160                 180                 200
                     |                   |                   |                   |                   |                   |                   |                   |                   |                   |
Sequence   MPELALDPRTIGGAFSVDECARRIINYRFAENVCMTTEGGWASSTPSIKVKFALGEHCYHDSIHSFWLGQRLPELRQTEKMDYESPPTFRTSYLAEPPNEAFLKFCETMQLQTDQLLRVVGLYRVLKTHLAVYYRHHIQVTDQVSDAPSVRILNHVLLEEEQHLKWGQAMYEELADTPERRRAALSWQMELEDLLVKSGGVTGGR
Prediction 6443343462243213044004203412301320242033313534313022000310130142042036314403434436344443231442343324122312422434443222000014302420240144026303403532013004302343663352134114412447434623340252034004313526458
           Values range from 0 (buried residue) to 9 (highly exposed residue)
```

Predicted normalized B-factor

(B-factor is a value to indicate the extent of the inherent thermal mobility of residues/atoms in proteins. In I-TASSER, this value is deduced from threading template proteins from the PDB in combination with the sequence profiles derived from sequence databases. The reported B-factor profile in the figure below corresponds to the normalized B-factor of the target protein, defined by B=(B'-u)/s, where B' is the raw B-factor value, u and s are respectively the mean and standard deviation of the raw B-factors along the sequence. Click here to read more about predicted normalized B-factor)

Top 10 threading templates used by I-TASSER

(I-TASSER modeling starts from the structure templates identified by LOMETS from the PDB library. LOMETS is a meta-server threading approach containing multiple threading programs, where each threading program can generate tens of thousands of template alignments. I-TASSER only uses the templates of the highest significance in the threading alignments, the significance of which are measured by the Z-score, i.e. the difference between the raw and average scores in the unit of standard deviation. The templates in this section are the 10 best templates selected from the LOMETS threading programs. Usually, one template of the highest Z-score is selected from each threading program, where the threading programs are sorted by the average performance in the large-scale benchmark test experiments.)

Rank	PDB Hit	Iden1	Iden2	Cov	Norm. Z-score	Download Align.
1	2vzbA	0.14	0.17	0.78	1.46	Download
2	1mhz	0.11	0.18	0.98	1.65	Download
3	1mhz	0.10	0.18	0.94	1.21	Download
4	1mhz	0.10	0.18	0.86	2.19	Download
5	4wjgE	0.07	0.18	0.95	1.25	Download
6	2clbA	0.17	0.13	0.70	1.00	Download
7	2gs4A	0.10	0.14	0.75	1.45	Download
8	1mhzA	0.10	0.14	1.00	1.54	Download
9	1mhzA	0.10	0.14	0.96	1.06	Download
10	1mhzA	0.09	0.14	0.86	1.78	Download

```
                 20                  40                  60                  80                 100                 120                 140                 160                 180                 200
                  |                   |                   |                   |                   |                   |                   |                   |                   |                   |
Sec.Str CCCCCCCHHHHCCCCCHHHHHHHHHHHHHHHHHHHHHHHHHHHHCCCCHHHHHHHHHHHHHHHHHHHHHHHHHHHHHHHHHHHHCCCCCCCCCCCCCCCCHHCCCCHHHHHHHHHHHHHCCCCCHHHHHHHHHHHHHHHHHHHHHHHHHHHHHHCCCCCCCCHHHHHHHHHHHHHHHHHHHHHHHHHHHHHHCCCHHHHHHHHHHHHHHHHHHHHHHHHCCCCCCC
    Seq MPELALDPRTIGGAFSVDECARRIINYRFAENVCMTTEGGWASSTPSIKVKFALGEHCYHDSIHSFWLGQRLPELRQTEKMDYESPPTFRTSYLAEPPNEAFLKFCETMQLQTDQLLRVVGLYRVLKTHLAVYYRHHIQVTDQVSDAPSVRILNHVLLEEEQHLKWGQAMYEELADTPERRRAALSWQMELEDLLVKSGGVTGGR
1       -AKESV--KILQGKLDVKSLIDQLNAAEWLAYYQYWVGALVVEGAMRADVQGEFEEHAEEERHHAQLIADRIIELEG---------------VPVLDPKKWFELARCKSPTAFDSVSLLNQNVSSERCAILRYQEIANFTNG-KDYTTCDIAKHILAEEEHEQDLQDYLTDIARMKES-----------------------
2       FGTLDGLRLGAGNKVHPETMKVISNFLEVGEYNAIAASAMLWDSATAAEQKNGYLAQVLDEIRHTHQCAFINHYYSKHY----HDPAGHNDTRAIGPLWKGKRVFADGFI-SGDAVECSVNLQLVGEACFTNPLIVVTEWASANGDEITPTVFLSVETDELRHMANGYQTVVSIANDPASAKFLNTDLNNAFTQQKYFTPVLGYK
3       LGAGNKVHPR-----WGETMKVISNFLEVGEYNAIAASAMLWDSATAAEQKNGYLAQVLDEIRHTHQCAFINHYYSKHY------HDPAGHNRAIGPLWKGMRVFADGFI-SGDAVECSVNLQLVGEACFTNPLIVATEWASANGDEITPTVFLSVETDELRHMANGYQTVVSIANDPASAKFLNTDLNAFWTQQKYFTPVLGYL
4       ---------------GETMKVISNFLEVGEYNAIAASAMLWDSATAAEQKNGYLAQVLDEIRHTHQCAFINHYYSK-------HYHDAGHNDAIGPLKGMKRVFADGFI-SGDAVECSVNLQLVGEACFTNPLIVATEWASANGDEITPTVFLSVETDELRHMANGYQTVVSIANDPASAKFLNTDLNAFWTQQKYFTPV----
5       ----------AEGLKTKDEVEKACHLAQQLKEVSITLGVIYRTTERHSVQVEAHKTAIDKHADASRAVEALTRVDVAELGKANDTKAVKIIENITSARENLALFNNETQAVLTARDHVHKHRAAALQGWSDAVWVLLNAAKKGNGSADVKKFKLSNASVGAVRDTSGRGGKHMEKVNNVAKLLKDAEVSLAAAAAEIEEVKQEE
6       -----------SGLDIKKLVDKLVKATAAEFTTYYYYTILRHLTGEGEGLKEIAEDARLEDRLHFELTQRIYELGGGLPRD-------------IRQLADISACSDA-YLPENWKDPKEILKVLLEAEQCAIRTWKEVCDTYKDPRTYDLAQRILQEEIEHEAWFLELL------------------------------
7       -------------TIEDVIHLLSDTYSAEKQLTRALAKLARATSNEKLSQAFHAHLEETHGQIERIDQVVESESNLKIKR-KCVA-EGL-------IEEANEVIESTEKNEVRDAALIAAAQKVEHYEIASYGTLATLAEQLGYRKAAKLLKETLEEEKATDIKLTDLAINNVNKK-------------------------
8       RQFGDGLRLGAGNKVHPRTMKVISNFLEVGEYNAIAASAMLWDSATAAEQKNGYLAQVLDEIRHTHQCAFINHYYSKHYHDPAGHNDA-RRTRAIGPLWKGMKRVFADGFISGDAVECSVNLQLVGEACFNPLIVAVTEWASANGDEITPTVFLSVETDELRHMANGYQTVVSIANDPASAKFLNTDLNNAWTQQKYFTPVLGYK
9       LGAGNKVHPR-----WGETMKVISNFLEVGEYNAIAASAMLWDSATAAEQKNGYLAQVLDEIRHTHQCAFINHYYSKHYHAGH---NDARRTRAIGPLWKGMKRVFADGFISGDAVECSVNLQLVGEACFTNPLIAVTEWASANGDEITPTVFLSVETDELRHMANGYQTVVSIANDPASAKFLNTDLNAFWTQQKYFTPVLGYL
10      ---------------ETMKVISNFLEVGEYNAIAASAMLWDSATAAEQKNGYLAQVLDEIRHTHQCAFINHYYSKH-------YHDPGHNRAIGPLWKGMKRVFADGFISGDAVECSVNLQLVGEACFTNPLIAVTEWASANGDEITPTVFLSVETDELRHMANGYQTVVSIANDPASAKFLNTDLNAFWTQQKYFTP-----
```

(a) All the residues are colored in black; however, those residues in template which are identical to the residue in the query sequence are highlighted in color. Coloring scheme is based on the property of amino acids, where polar are brightly coloured while non-polar residues are colored in dark shade. (more about the colors used)
(b) Rank of templates represents the top ten threading templates used by I-TASSER.
(c) Ident1 is the percentage sequence identity of the templates in the threading aligned region with the query sequence.
(d) Ident2 is the percentage sequence identity of the whole template chains with query sequence.
(e) Cov represents the coverage of the threading alignment and is equal to the number of aligned residues divided by the length of query protein.
(f) Norm. Z-score is the normalized Z-score of the threading alignments. Alignment with a Normalized Z-score >1 mean a good alignment and vice versa.
(g) Download Align. provides the 3D structure of the aligned regions of the threading templates.
(h) The top 10 alignments reported above (in order of their ranking) are from the following threading programs:
1: FFAS-3D 2: HHSEARCH2 3: HHSEARCH I 4: HHSEARCH 5: PROSPECT2 6: PRC 7: FFAS-3D 8: HHSEARCH2 9: HHSEARCH I 10: HHSEARCH

Top 5 final models predicted by I-TASSER

(For each target, I-TASSER simulations generate a large ensemble of structural conformations, called decoys. To select the final models, I-TASSER uses the SPICKER program to cluster all the decoys based on the pair-wise structure similarity, and reports up to five models which corresponds to the five largest structure clusters. The confidence of each model is quantitatively measured by C-score that is calculated based on the significance of threading template alignments and the convergence parameters of the structure assembly simulations. C-score is typically in the range of [-5, 2], where a C-score of a higher value signifies a model with a higher confidence and vice-versa. TM-score and RMSD are estimated based on C-score and protein length following the correlation observed between these qualities. Since the top 5 models are ranked by the cluster size, it is possible that the lower-rank models have a higher C-score in rare cases. Although the first model has a better quality in most cases, it is also possible that the lower-rank models have a better quality than the higher-rank models as seen in our benchmark tests. If the I-TASSER simulations converge, it is possible to have less than 5 clusters generated; this is usually an indication that the models have a good quality because of the converged simulations.)

- More about C-score
- Local structure accuracy profile of the top five models

(By right-click on the images, you can export image file or change the configurations, e.g. modifying the background color or stopping the spin of your models)

- Download Model 1
- C-score=-0.58 (Read more about C-score)
- Estimated TM-score = 0.64±0.13
- Estimated RMSD = 6.6±4.0Å

- Download Model 2
- C-score = -3.70

- Download Model 3
- C-score = -3.93

- Download Model 4
- C-score = -2.23

- Download Model 5
- C-score = -4.44

Proteins structurally close to the target in the PDB (as identified by TM-align)

(After the structure assembly simulation, I-TASSER uses the TM-align structural alignment program to match the first I-TASSER model to all structures in the PDB library. This section reports the top 10 proteins from the PDB that have the closest structural similarity, i.e. the highest TM-score, to the predicted I-TASSER model. Due to the structural similarity, these proteins often have similar function to the target. However, users are encouraged to use the data in the next section 'Predicted function using COACH' to infer the function of the target protein, since COACH has been extensively trained to derive biological functions from multi-source of sequence and structure features which has on average a higher accuracy than the function annotations derived only from the global structure comparison.)

Top 10 Identified stuctural analogs in PDB

Click to view	Rank	PDB Hit	TM-score	RMSD[a]	IDEN[a]	Cov	Alignment
	1	1fz3B	0.925	1.59	0.099	0.990	Download
	2	1mhzD1	0.924	1.66	0.103	0.995	Download
	3	3dhgD	0.868	2.11	0.130	0.976	Download
	4	1t0qA	0.865	2.13	0.125	0.976	Download
	5	3ge8B	0.857	2.34	0.127	0.995	Download
	6	2incB	0.850	2.43	0.127	0.995	Download
	7	1mhyB	0.839	2.62	0.073	0.995	Download
	8	2inpD	0.833	2.58	0.089	0.976	Download
	9	2vzbA	0.620	3.32	0.154	0.766	Download
	10	2clbA	0.588	3.47	0.119	0.737	Download

(a) Query structure is shown in cartoon, while the structural analog is displayed using backbone trace.
(b) Ranking of proteins is based on TM-score of the structural alignment between the query structure and known structures in the PDB library.
(c) RMSD[a] is the RMSD between residues that are structurally aligned by TM-align.
(d) IDEN[a] is the percentage sequence identity in the structurally aligned region.
(e) Cov represents the coverage of the alignment by TM-align and is equal to the number of structurally aligned residues divided by length of the query protein.

Predicted function using COFACTOR and COACH

(This section reports biological annotations of the target protein by COFACTOR and COACH based on the I-TASSER structure prediction. While COFACTOR deduces protein functions (ligand-binding sites, EC and GO) using structure comparison and protein-protein networks, COACH is a meta-server approach that combines multiple function annotation results (on ligand-binding sites) from the COFACTOR, TM-SITE and S-SITE programs.)

Ligand binding sites

Click to view	Rank	C-score	Cluster size	PDB Hit	Lig Name	Download Complex	Ligand Binding Site Residues
	1	0.36	183	1jprA	MN	Rep, Mult	31,61,64,160
	2	0.18	95	2wtlD	FE	Rep, Mult	57,61,127,160,163
	3	0.15	48	4kvsA	6NA	Rep, Mult	30,31,34,61,122,126,127,130,160
	4	0.08	29	1fz9B	ETI	Rep, Mult	23,26,27,102,133,202,205
	5	0.06	23	4rc5A	PL3	Rep, Mult	20,23,26,27,30,31,34,61,102,126,127,129,130,133,134,137

Download the residue-specific ligand binding probability, which is estimated by SVM.
Download the all possible binding ligands and detailed prediction summary.
Download the templates clustering results.
(a) **C-score** is the confidence score of the prediction. C-score ranges [0-1], where a higher score indicates a more reliable prediction.
(b) **Cluster size** is the total number of templates in a cluster.
(c) **Lig Name** is name of possible binding ligand. Click the name to view its information in the BioLiP database.
(d) **Rep** is a single complex structure with the most representative ligand in the cluster, i.e., the one listed in the **Lig Name** column.
Mult is the complex structures with all potential binding ligands in the cluster.

Enzyme Commission (EC) numbers and active sites

Click to view	Rank	$Cscore^{EC}$	PDB Hit	TM-score	RMSD[a]	IDEN[a]	Cov	EC Number	Active Site Residues
	1	0.358	1fziA	0.924	1.67	0.098	0.995	1.14.13.25	NA
	2	0.357	1mhyD	0.925	1.60	0.103	0.990	1.14.13.25	NA
	3	0.350	3ee4A	0.817	2.43	0.108	0.946	1.17.4.1	NA
	4	0.339	1syyA	0.816	2.62	0.106	0.966	1.17.4.1	NA
	5	0.334	1biqB	0.813	2.55	0.071	0.956	1.17.4.1	NA

Click on the radio buttons to visualize predicted active site residues.
(a) $Cscore^{EC}$ is the confidence score for the EC number prediction. $Cscore^{EC}$ values range in between [0-1]; where a higher score indicates a more reliable EC number prediction.
(b) TM-score is a measure of global structural similarity between query and template protein.
(c) RMSD[a] is the RMSD between residues that are structurally aligned by TM-align.
(d) IDEN[a] is the percentage sequence identity in the structurally aligned region.
(e) Cov represents the coverage of global structural alignment and is equal to the number of structurally aligned residues divided by length of the query protein.

Gene Ontology (GO) terms

Top 10 homologous GO templates in PDB

Rank	$Cscore^{GO}$	TM-score	RMSD[a]	IDEN[a]	Cov	PDB Hit	Associated GO Terms
1	0.33	0.9240	1.67	0.10	1.00	1fziA	GO:0016491 GO:0015049 GO:0055114 GO:0004497 GO:0046872 GO:0006730 GO:0006725 GO:0046914
2	0.33	0.9246	1.60	0.10	0.99	1mhyD	GO:0006730 GO:0055114 GO:0015049 GO:0046872 GO:0004497 GO:0016491 GO:0006725 GO:0046914
3	0.32	0.8654	2.13	0.12	0.98	1t0qA	GO:0016491 GO:0004497 GO:0046872 GO:0046914 GO:0055114 GO:0006725
4	0.31	0.8660	2.11	0.14	0.98	3dhiA	GO:0055114 GO:0016491 GO:0019439 GO:0004497 GO:0006725 GO:0046914 GO:0046872
5	0.31	0.8386	2.62	0.07	1.00	1mhyB	GO:0016491 GO:0004497 GO:0006730 GO:0015049 GO:0055114 GO:0006725 GO:0016709 GO:0046914
6	0.31	0.8331	2.58	0.09	0.98	2inpD	GO:0046914 GO:0016709 GO:0055114 GO:0006725 GO:0016491
7	0.31	0.8557	2.35	0.13	1.00	3dhiB	GO:0004497 GO:0016491 GO:0019439 GO:0046914 GO:0055114 GO:0006725 GO:0016709
8	0.30	0.8484	2.44	0.13	1.00	3n1xB	GO:0046914 GO:0055114 GO:0016709 GO:0004497 GO:0016491 GO:0006725
9	0.27	0.6195	3.32	0.15	0.77	2vzbA	GO:0046872 GO:0016491 GO:0006879 GO:0008199 GO:0046914 GO:0055114
10	0.23	0.5656	3.00	0.13	0.69	1jigA	GO:0055114 GO:0046872 GO:0005737 GO:0006879 GO:0016491 GO:0006950 GO:0008199 GO:0046914

Consensus prediction of GO terms

**Molecular Function**	GO:0046914	GO:0015049	
**GO-Score**	0.85	0.69	
**Biological Process**	GO:0055114	GO:0006730	GO:0019439
**GO-Score**	0.85	0.69	0.31
**Cellular Component**	None was predicted		

(a) $Cscore^{GO}$ is a combined measure for evaluating global and local similarity between query and template protein. It's range is [0-1] and higher values indicate more confident predictions.
(b) TM-score is a measure of global structural similarity between query and template protein.
(c) RMSD[a] is the RMSD between residues that are structurally aligned by TM-align.
(d) IDEN[a] is the percentage sequence identity in the structurally aligned region.
(e) Cov represents the coverage of global structural alignment and is equal to the number of structurally aligned residues divided by length of the query protein.
(f) The second table shows a consensus GO terms amongst the top scoring templates. The GO-Score associated with each prediction is defined as the average weight of the GO term, where the weights are assigned based on $Cscore^{GO}$ of the template.

[Click on S522614_results.tar.bz2 to download the tarball file including all modeling results listed on this page]

Please cite the following articles when you use the I-TASSER server:
1. J Yang, Y Zhang. I-TASSER server: new development for protein structure and function predictions, Nucleic Acids Research, 43: W174-W181, 2015.
2. C Zhang, PL Freddolino, Y Zhang. COFACTOR: improved protein function prediction by combining structure, sequence and protein–protein interaction information. Nucleic Acids Research, 45: W291-W299, 2017.
