# Supplementary material for: Unique high Arctic methane metabolizing community revealed through in situ 13CH4-DNA-SIP enrichment in concert with genome binning
Source: Sci Rep. 2022 Jan 21;12:1160. doi: 10.1038/s41598-021-04486-z (PMC8782848; doi:10.1038/s41598-021-04486-z)

Supplementary Figures

Figure S1. Polygon pattern of ice-wedge polygon terrain. On the right is the birds eye view, on the left is the ground view.


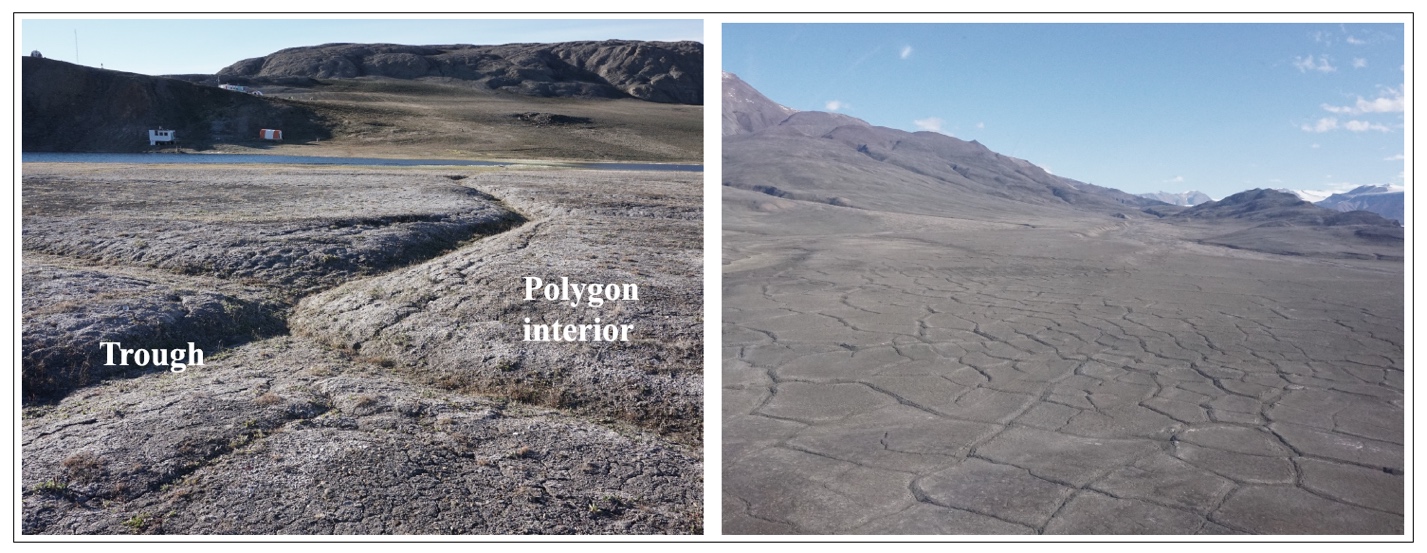


Figure S2. Visualization of the heavy and light bands. The heavy band is the ^13^C labelled DNA. Control is the soil that was not exposed to the ^13^C.





Figure S3. The microbial community composition. The community composition is based on 16S profiling in the heavy and light bands from the ^13^CH_4_ SIP enrichment at 100 ppm and 1000 ppm, as well as the composition of the control soils that were not enriched in CH_4_.


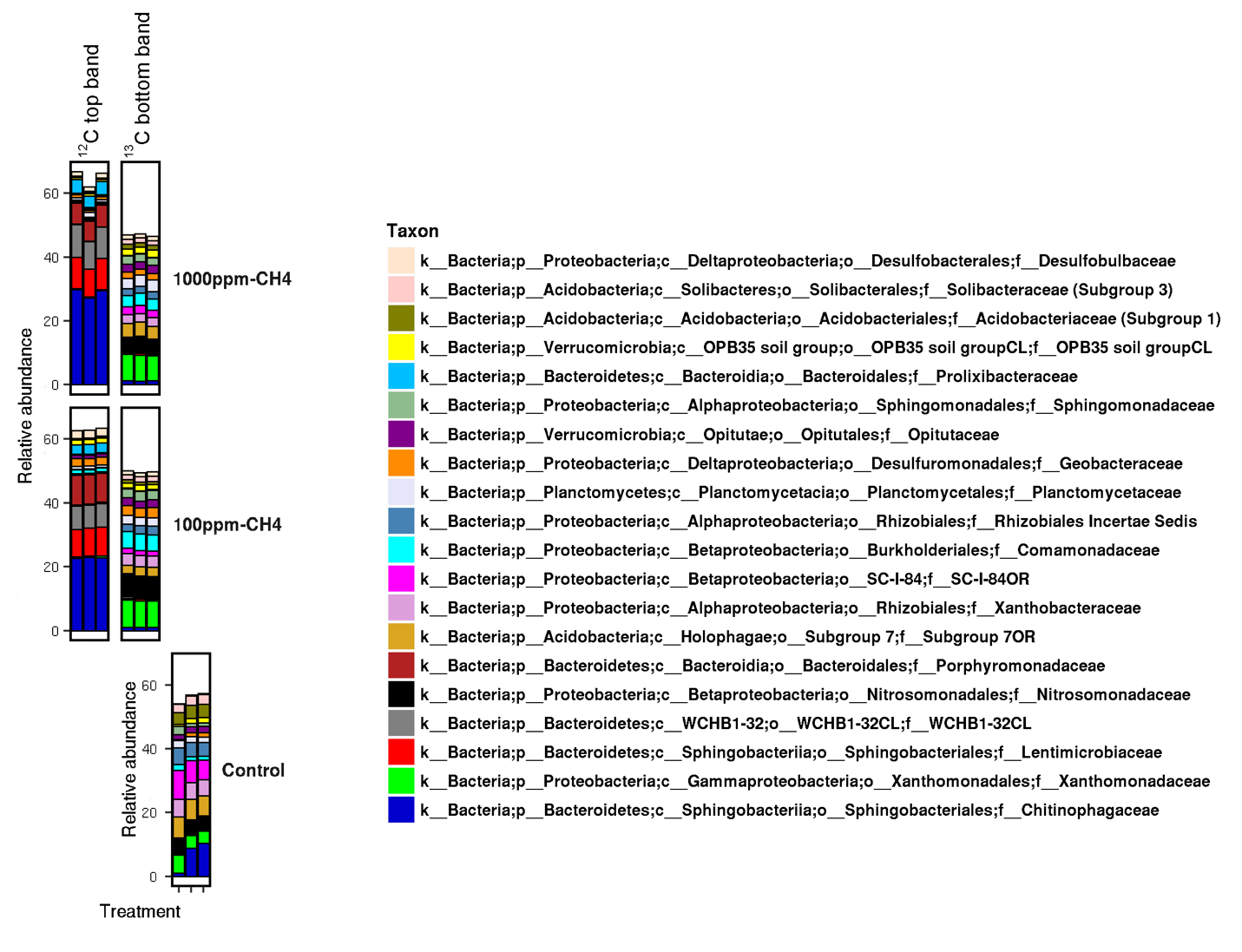

Supplement: Supplementary file 4 — Supplementary Figures. [file 41598_2021_4486_MOESM4_ESM.docx]
