# Supplementary material for: Unique high Arctic methane metabolizing community revealed through in situ 13CH4-DNA-SIP enrichment in concert with genome binning
Source: Sci Rep. 2022 Jan 21;12:1160. doi: 10.1038/s41598-021-04486-z (PMC8782848; doi:10.1038/s41598-021-04486-z)
Supplement: Supplementary file 5 — Supplementary Tables. [file 41598_2021_4486_MOESM5_ESM.docx]

Supplementary Tables:

**Table S1**. ANOVA and Tukey T-test analysis of the *pmoA* qPCR.

| **ANOVA** |  |  |  | **TUKEY T-test** |  |  |
| --- | --- | --- | --- | --- | --- | --- |
| ***Source of Variation*** | ***df*** | ***F*** | ***P-value*** | ***Soils compared*** | | ***P-value*** |
| Depth (5cm/25cm) | 1 | 23.9858 | **0.0012** | Trough 5cm : Polygon 5cm | | **0.0006** |
| Terrain (Polygon/Trough) | 1 | 42.4757 | **0.0002** | Trough 5cm : Trough 25cm | | **0.0019** |
| Interaction of Depth and Terrain | 1 | 10.7185 | **0.0113** | Trough 5cm : Polygon 25cm | | **0.0002** |
| Within | 8 |  |  | Polygon 5cm : Trough 25cm | | 0.6750 |
| Total | 11 |  |  | Polygon 5cm : Polygon 25cm | | 0.6725 |
|  |  |  |  | Trough 25cm : Polygon 25cm | | 0.1789 |

**Table S2.** Sequencing summary of the three ^13^CH_4_ SIP metagenomes.

| Metagenome | Public MGRAST accession # | Base Pairs | Sequences | GC content |
| --- | --- | --- | --- | --- |
| ^13^CH_4_-SIP1 | mgm4798482.3 | 2,617,877,087 | 9,460,463 | 65 ± 6 % |
| ^13^CH_4_-SIP2 | mgm4798483.3 | 2,082,553,587 | 7,559,115 | 64 ± 6 % |
| ^13^CH_4_-SIP3 | mgm4798481.3 | 2,643,747,297 | 9,518,034 | 66 ± 5 % |

**Table S3.** MAGs taxonomic novelty at each taxonomic level, p values represent how likely the MAGs are novel at each taxonomic classification, calculated with MiGA Genomic Atlas. Bold p values indicate that the MAG has a ≥70% chance of being novel at that classification rank.

| MAG | Closest (%AAI) relative | Phylum | Class | Order | Family | Genus | Species |
| --- | --- | --- | --- | --- | --- | --- | --- |
| #6 | Betaproteobacteria bacterium GR16 43 (59.97%) | 0.972 | 0.927 | 0.751 | 0.48 | **0.275** | **0.00385** |
| #8 | Sulfuricella denitrificans skB26 (52.06%) | 0.935 | 0.829 | 0.577 | **0.235** | **0.0756** | **0.00154** |
| #15 | Ramlibacter tataouinensis (67.9%) | 0.984 | 0.958 | 0.854 | 0.695 | 0.487 | **0.00924** |
| #16 | Thiobacillus denitrificans ATCC 25259 (64.25%) | 0.977 | 0.94 | 0.794 | 0.569 | 0.368 | **0.00539** |
| #20 | Gemmatirosa kalamazoonesis (47.7%) | 0.866 | 0.645 | 0.329 | **0.143** | **0.0208** | **0.00077** |
| #21 | Sphaerobacter thermophilus DSM 20745 (40.91%) | 0.545 | **0.117** | **0.0521** | **0.0123** | **0** | **0** |
| #22 | Azoarcus sp. BH72 (42.61%) | 0.599 | **0.166** | **0.107** | **0.00373** | **0.00132** | **0** |
| #24 | Burkholderiales bacterium JOSHI 001 (54.42%) | 0.953 | 0.876 | 0.651 | 0.321 | **0.139** | **0.00385** |
| #27 | Candidatus Solibacter usitatus Ellin6076 (39.75% AAI) | 0.497 | **0.0908** | **0.0336** | **0.0098** | **0** | **0** |

**Table S4.** Methane pathway genes found in the 100ppm ^13^CH_4_-SIP metagenomes. Annotated with DOE-JGI Metagenome Annotation Pipeline.

| JGI-IMG Metagenome and Gene ID | Gene Product Name | DNA Sequence Length (bp) | Amino Acid Sequence Length (AA) | Enzyme |
| --- | --- | --- | --- | --- |
| 3300034949 assembled Ga0374202_0815625_1_495 | methane monooxygenase component A alpha chain/propane monooxygenase large subunit | 495 | 165 | EC:1.14.13.25 - Methane monooxygenase (soluble) |
| 3300034422 assembled Ga0374207_656181_3_374 | methane monooxygenase component A alpha chain/propane monooxygenase large subunit | 372 | 124 | EC:1.14.13.25 - Methane monooxygenase (soluble) |
| 3300034949 assembled Ga0374202_0223846_3_344 | methane monooxygenase component A beta chain/propane monooxygenase small subunit | 342 | 114 | EC:1.14.13.25 - Methane monooxygenase (soluble) |
| 3300034949 assembled Ga0374202_1374523_1_453 | methane monooxygenase component A alpha chain/propane monooxygenase large subunit | 453 | 150 | EC:1.14.13.- - Oxidoreductases |
| 3300034422 assembled Ga0374207_891519_409_642 | methane monooxygenase component A alpha chain/propane monooxygenase large subunit | 234 | 78 | EC:1.14.13.25 - Methane monooxygenase (soluble) |
| 3300034949 assembled Ga0374202_0019601_95_331 | methane monooxygenase component A alpha chain/propane monooxygenase large subunit | 237 | 79 | EC:1.14.13.25 - Methane monooxygenase (soluble) |
| 3300034385 assembled Ga0374210_0496428_3_299 | methane monooxygenase component A alpha chain/propane monooxygenase large subunit | 297 | 99 | EC:1.14.13.- - Oxidoreductases |
| 3300034385 assembled Ga0374210_0338907_3_419 | methane monooxygenase component A alpha chain/propane monooxygenase large subunit | 417 | 139 | EC:1.14.13.25 - Methane monooxygenase (soluble) |
| 3300034949 assembled Ga0374202_0414519_4_1110 | methane monooxygenase component A alpha chain/propane monooxygenase large subunit | 1107 | 368 | EC:1.14.13.25 - Methane monooxygenase (soluble) |
| 3300034385 assembled Ga0374210_1249541_3_299 | methane monooxygenase component A beta chain/propane monooxygenase small subunit | 297 | 99 | EC:1.14.13.25 - Methane monooxygenase (soluble) |
| 3300034949 assembled Ga0374202_0043021_3_389 | methane/ammonia monooxygenase subunit B | 387 | 129 | EC:1.14.18.3 - pmoB-amoB; methane/ammonia monooxygenase subunit B |
| 3300034385 assembled Ga0374210_0519114_83_382 | mxaJ protein | 300 | 100 | EC:1.1.2.7methanol dehydrogenase (cytochrome c) |
| 3300034422 assembled Ga0374207_187712_1_360 | mxaD protein | 360 | 120 | EC:1.1.2.7methanol dehydrogenase (cytochrome c) |
| 3300034422 assembled Ga0374207_903718_3_530 | mxaJ protein | 528 | 176 | EC:1.1.2.7methanol dehydrogenase (cytochrome c) |
| 3300034949 assembled Ga0374202_0286878_168_305 | mxaJ protein | 138 | 45 | EC:1.1.2.7methanol dehydrogenase (cytochrome c) |
| 3300034385 assembled Ga0374210_0323618_4_420 | mxaK protein | 417 | 139 | EC:1.1.2.7methanol dehydrogenase (cytochrome c) |
| 3300034385 assembled Ga0374210_1327404_294_614 | mxaJ protein | 321 | 107 | EC:1.1.2.7methanol dehydrogenase (cytochrome c) |
| 3300034949 assembled Ga0374202_0899177_38_229 | mxaD protein | 192 | 63 | EC:1.1.2.7methanol dehydrogenase (cytochrome c) |
| 3300034422 assembled Ga0374207_405631_91_303 | mxaJ protein | 213 | 70 | EC:1.1.2.7methanol dehydrogenase (cytochrome c) |
| 3300034385 assembled Ga0374210_0904853_1_288 | mxaJ protein | 288 | 96 | EC:1.1.2.7methanol dehydrogenase (cytochrome c) |
| 3300034949 assembled Ga0374202_0466335_1_291 | mxaJ protein | 291 | 97 | EC:1.1.2.7methanol dehydrogenase (cytochrome c) |
| 3300034385 assembled Ga0374210_1348456_84_437 | mxaJ protein | 354 | 117 | EC:1.1.2.7methanol dehydrogenase (cytochrome c) |
| 3300034385 assembled Ga0374210_0090887_80_574 | mxaJ protein | 495 | 165 | EC:1.1.2.7methanol dehydrogenase (cytochrome c) |
| 3300034949 assembled Ga0374202_0157762_91_738 | mxaJ protein | 648 | 216 | EC:1.1.2.7methanol dehydrogenase (cytochrome c) |
| 3300034422 assembled Ga0374207_380423_2_289 | mxaJ protein | 288 | 96 | EC:1.1.2.7methanol dehydrogenase (cytochrome c) |
| 3300034385 assembled Ga0374210_1218021_1_435 | mxaJ protein | 435 | 144 | EC:1.1.2.7methanol dehydrogenase (cytochrome c) |
| 3300034422 assembled Ga0374207_628487_99_347 | mxaK protein | 249 | 82 | EC:1.1.2.7methanol dehydrogenase (cytochrome c) |
| 3300034422 assembled Ga0374207_527965_282_899 | mxaJ protein | 618 | 206 | EC:1.1.2.7methanol dehydrogenase (cytochrome c) |
| 3300034949 assembled Ga0374202_0110390_3_134 | mxaC protein | 132 | 44 | EC:1.1.2.7methanol dehydrogenase (cytochrome c) |
| 3300034385 assembled Ga0374210_1377247_47_472 | mxaJ protein | 426 | 142 | EC:1.1.2.7methanol dehydrogenase (cytochrome c) |
| 3300034385 assembled Ga0374210_0545166_355_969 | mxaJ protein | 615 | 204 | EC:1.1.2.7methanol dehydrogenase (cytochrome c) |
| 3300034422 assembled Ga0374207_464698_70_465 | mxaJ protein | 396 | 131 | EC:1.1.2.7methanol dehydrogenase (cytochrome c) |
| 3300034422 assembled Ga0374207_886775_1_402 | mxaD protein | 402 | 134 | EC:1.1.2.7methanol dehydrogenase (cytochrome c) |
| 3300034385 assembled Ga0374210_0199729_73_426 | mxaJ protein | 354 | 118 | EC:1.1.2.7methanol dehydrogenase (cytochrome c) |
| 3300034422 assembled Ga0374207_213021_3_224 | mxaD protein | 222 | 73 | EC:1.1.2.7methanol dehydrogenase (cytochrome c) |
| 3300034385 assembled Ga0374210_0507117_2_343 | mxaC protein | 342 | 114 | EC:1.1.2.7methanol dehydrogenase (cytochrome c) |
| 3300034949 assembled Ga0374202_1131304_1_435 | mxaA protein | 435 | 144 | EC:1.1.2.7methanol dehydrogenase (cytochrome c) |
| 3300034949 assembled Ga0374202_1299989_709_1152 | mxaJ protein | 444 | 148 | EC:1.1.2.7methanol dehydrogenase (cytochrome c) |
| 3300034949 assembled Ga0374202_0110390_131_481 | mxaA protein | 351 | 116 | EC:1.1.2.7methanol dehydrogenase (cytochrome c) |
| 3300034949 assembled Ga0374202_0627508_2_187 | mxaJ protein | 186 | 62 | EC:1.1.2.7methanol dehydrogenase (cytochrome c) |
| 3300034949 assembled Ga0374202_0841042_1_213 | mxaJ protein | 213 | 71 | EC:1.1.2.7methanol dehydrogenase (cytochrome c) |
| 3300034422 assembled Ga0374207_707686_4_333 | fae (5,6,7,8-tetrahydromethanopterin hydro-lyase) | 330 | 110 | EC:4.2.1.147 - 5,6,7,8-tetrahydromethanopterin hydro-lyase |
| 3300034949 assembled Ga0374202_0498226_1_231 | fae (5,6,7,8-tetrahydromethanopterin hydro-lyase) | 231 | 77 | EC:4.2.1.147 - 5,6,7,8-tetrahydromethanopterin hydro-lyase |
| 3300034385 assembled Ga0374210_1532266_155_400 | fae (5,6,7,8-tetrahydromethanopterin hydro-lyase) | 246 | 81 | EC:4.2.1.147 - 5,6,7,8-tetrahydromethanopterin hydro-lyase |
| 3300034385 assembled Ga0374210_0791302_194_370 | fae (5,6,7,8-tetrahydromethanopterin hydro-lyase) | 177 | 59 | EC:4.2.1.147 - 5,6,7,8-tetrahydromethanopterin hydro-lyase |
| 3300034949 assembled Ga0374202_0532079_4_171 | fae (5,6,7,8-tetrahydromethanopterin hydro-lyase) | 168 | 55 | EC:4.2.1.147 - 5,6,7,8-tetrahydromethanopterin hydro-lyase |
| 3300034385 assembled Ga0374210_0235104_289_516 | fae (5,6,7,8-tetrahydromethanopterin hydro-lyase) | 228 | 76 | EC:4.2.1.147 - 5,6,7,8-tetrahydromethanopterin hydro-lyase |
| 3300034422 assembled Ga0374207_166716_22_315 | fae (5,6,7,8-tetrahydromethanopterin hydro-lyase) | 294 | 98 | EC:4.2.1.147 - 5,6,7,8-tetrahydromethanopterin hydro-lyase |
| 3300034385 assembled Ga0374210_0182770_109_606 | fae (5,6,7,8-tetrahydromethanopterin hydro-lyase) | 498 | 165 | EC:4.2.1.147 - 5,6,7,8-tetrahydromethanopterin hydro-lyase |
| 3300034422 assembled Ga0374207_339391_141_656 | fae (5,6,7,8-tetrahydromethanopterin hydro-lyase) | 516 | 171 | EC:4.2.1.147 - 5,6,7,8-tetrahydromethanopterin hydro-lyase |
| 3300034385 assembled Ga0374210_0675128_188_349 | fae (5,6,7,8-tetrahydromethanopterin hydro-lyase) | 162 | 54 | EC:4.2.1.147 - 5,6,7,8-tetrahydromethanopterin hydro-lyase |
| 3300034949 assembled Ga0374202_0866529_2_388 | fae (5,6,7,8-tetrahydromethanopterin hydro-lyase) | 387 | 129 | EC:4.2.1.147 - 5,6,7,8-tetrahydromethanopterin hydro-lyase |
| 3300034949 assembled Ga0374202_0098959_16_360 | fae (5,6,7,8-tetrahydromethanopterin hydro-lyase) | 345 | 115 | EC:4.2.1.147 - 5,6,7,8-tetrahydromethanopterin hydro-lyase |
| 3300034949 assembled Ga0374202_0954254_2_469 | fae (5,6,7,8-tetrahydromethanopterin hydro-lyase) | 468 | 156 | EC:4.2.1.147 - 5,6,7,8-tetrahydromethanopterin hydro-lyase |
| 3300034949 assembled Ga0374202_0991088_1_399 | fae (5,6,7,8-tetrahydromethanopterin hydro-lyase) | 399 | 133 | EC:4.2.1.147 - 5,6,7,8-tetrahydromethanopterin hydro-lyase |
| 3300034385 assembled Ga0374210_1122284_88_579 | fae (5,6,7,8-tetrahydromethanopterin hydro-lyase) | 492 | 163 | EC:4.2.1.147 - 5,6,7,8-tetrahydromethanopterin hydro-lyase |
| 3300034949 assembled Ga0374202_0136983_382_609 | fae (5,6,7,8-tetrahydromethanopterin hydro-lyase) | 228 | 76 | EC:4.2.1.147 - 5,6,7,8-tetrahydromethanopterin hydro-lyase |
| 3300034422 assembled Ga0374207_129523_3_314 | fae (5,6,7,8-tetrahydromethanopterin hydro-lyase) | 312 | 103 | EC:4.2.1.147 - 5,6,7,8-tetrahydromethanopterin hydro-lyase |
| 3300034422 assembled Ga0374207_040794_86_307 | fae (5,6,7,8-tetrahydromethanopterin hydro-lyase) | 222 | 74 | EC:4.2.1.147 - 5,6,7,8-tetrahydromethanopterin hydro-lyase |
| 3300034385 assembled Ga0374210_0283342_509_679 | fae (5,6,7,8-tetrahydromethanopterin hydro-lyase) | 171 | 57 | EC:4.2.1.147 - 5,6,7,8-tetrahydromethanopterin hydro-lyase |
| 3300034385 assembled Ga0374210_1086803_63_374 | fae (5,6,7,8-tetrahydromethanopterin hydro-lyase) | 312 | 103 | EC:4.2.1.147 - 5,6,7,8-tetrahydromethanopterin hydro-lyase |
| 3300034422 assembled Ga0374207_801367_13_276 | fae (5,6,7,8-tetrahydromethanopterin hydro-lyase) | 264 | 87 | EC:4.2.1.147 - 5,6,7,8-tetrahydromethanopterin hydro-lyase |
| 3300034949 assembled Ga0374202_0113232_300_572 | fae (5,6,7,8-tetrahydromethanopterin hydro-lyase) | 273 | 90 | EC:4.2.1.147 - 5,6,7,8-tetrahydromethanopterin hydro-lyase |
| 3300034385 assembled Ga0374210_0736982_1_447 | fae (5,6,7,8-tetrahydromethanopterin hydro-lyase) | 447 | 148 | EC:4.2.1.147 - 5,6,7,8-tetrahydromethanopterin hydro-lyase |
| 3300034422 assembled Ga0374207_875003_50_442 | fae (5,6,7,8-tetrahydromethanopterin hydro-lyase) | 393 | 131 | EC:4.2.1.147 - 5,6,7,8-tetrahydromethanopterin hydro-lyase |
| 3300034949 assembled Ga0374202_1307597_3_428 | fae (5,6,7,8-tetrahydromethanopterin hydro-lyase) | 426 | 141 | EC:4.2.1.147 - 5,6,7,8-tetrahydromethanopterin hydro-lyase |
| 3300034385 assembled Ga0374210_1062091_240_755 | fae (5,6,7,8-tetrahydromethanopterin hydro-lyase) | 516 | 171 | EC:4.2.1.147 - 5,6,7,8-tetrahydromethanopterin hydro-lyase |
| 3300034385 assembled Ga0374210_1355718_3_425 | fae (5,6,7,8-tetrahydromethanopterin hydro-lyase) | 423 | 141 | EC:4.2.1.147 - 5,6,7,8-tetrahydromethanopterin hydro-lyase |
| 3300034385 assembled Ga0374210_0259930_2_289 | fae (5,6,7,8-tetrahydromethanopterin hydro-lyase) | 288 | 96 | EC:4.2.1.147 - 5,6,7,8-tetrahydromethanopterin hydro-lyase |
| 3300034385 assembled Ga0374210_0461605_204_431 | fae (5,6,7,8-tetrahydromethanopterin hydro-lyase) | 228 | 75 | EC:4.2.1.147 - 5,6,7,8-tetrahydromethanopterin hydro-lyase |
| 3300034385 assembled Ga0374210_1431118_3_224 | fae (5,6,7,8-tetrahydromethanopterin hydro-lyase) | 222 | 74 | EC:4.2.1.147 - 5,6,7,8-tetrahydromethanopterin hydro-lyase |
| 3300034422 assembled Ga0374207_219383_88_300 | fae (5,6,7,8-tetrahydromethanopterin hydro-lyase) | 213 | 71 | EC:4.2.1.147 - 5,6,7,8-tetrahydromethanopterin hydro-lyase |
| 3300034385 assembled Ga0374210_0111584_7_162 | fae (5,6,7,8-tetrahydromethanopterin hydro-lyase) | 156 | 51 | EC:4.2.1.147 - 5,6,7,8-tetrahydromethanopterin hydro-lyase |
| 3300034385 assembled Ga0374210_0241272_1_381 | fae (5,6,7,8-tetrahydromethanopterin hydro-lyase) | 381 | 126 | EC:4.2.1.147 - 5,6,7,8-tetrahydromethanopterin hydro-lyase |
| 3300034385 assembled Ga0374210_0842544_156_338 | fae (5,6,7,8-tetrahydromethanopterin hydro-lyase) | 183 | 61 | EC:4.2.1.147 - 5,6,7,8-tetrahydromethanopterin hydro-lyase |
| 3300034385 assembled Ga0374210_1466434_240_425 | fae (5,6,7,8-tetrahydromethanopterin hydro-lyase) | 186 | 62 | EC:4.2.1.147 - 5,6,7,8-tetrahydromethanopterin hydro-lyase |
| 3300034949 assembled Ga0374202_0529722_3_338 | fae (5,6,7,8-tetrahydromethanopterin hydro-lyase) | 336 | 112 | EC:4.2.1.147 - 5,6,7,8-tetrahydromethanopterin hydro-lyase |
| 3300034422 assembled Ga0374207_302401_269_589 | fae (5,6,7,8-tetrahydromethanopterin hydro-lyase) | 321 | 107 | EC:4.2.1.147 - 5,6,7,8-tetrahydromethanopterin hydro-lyase |
| 3300034385 assembled Ga0374210_1049477_126_320 | fae (5,6,7,8-tetrahydromethanopterin hydro-lyase) | 195 | 65 | EC:4.2.1.147 - 5,6,7,8-tetrahydromethanopterin hydro-lyase |
| 3300034422 assembled Ga0374207_416886_1_228 | glutathione-independent formaldehyde dehydrogenase | 228 | 75 | EC:1.2.1.46 - Formaldehyde dehydrogenase |
| 3300034949 assembled Ga0374202_1120849_3_458 | glutathione-independent formaldehyde dehydrogenase | 456 | 152 | EC:1.2.1.46 - Formaldehyde dehydrogenase |
| 3300034422 assembled Ga0374207_825822_1_399 | glutathione-independent formaldehyde dehydrogenase | 399 | 133 | EC:1.2.1.46 - Formaldehyde dehydrogenase |
| 3300034949 assembled Ga0374202_1111944_1_387 | glutathione-independent formaldehyde dehydrogenase | 387 | 129 | EC:1.2.1.46 - Formaldehyde dehydrogenase |
| 3300034949 assembled Ga0374202_0203384_95_355 | glutathione-independent formaldehyde dehydrogenase | 261 | 87 | EC:1.2.1.46 - Formaldehyde dehydrogenase |
| 3300034949 assembled Ga0374202_0163949_1_300 | glutathione-independent formaldehyde dehydrogenase | 300 | 100 | EC:1.2.1.46 - Formaldehyde dehydrogenase |
| 3300034949 assembled Ga0374202_0002352_2_355 | glutathione-independent formaldehyde dehydrogenase | 354 | 118 | EC:1.2.1.46 - Formaldehyde dehydrogenase |
| 3300034422 assembled Ga0374207_248024_13_381 | glutathione-independent formaldehyde dehydrogenase | 369 | 123 | EC:1.2.1.46 - Formaldehyde dehydrogenase |
| 3300034422 assembled Ga0374207_500893_65_370 | glutathione-independent formaldehyde dehydrogenase | 306 | 102 | EC:1.2.1.46 - Formaldehyde dehydrogenase |
| 3300034949 assembled Ga0374202_0812485_3_365 | glutathione-independent formaldehyde dehydrogenase | 363 | 121 | EC:1.2.1.46 - Formaldehyde dehydrogenase |
| 3300034422 assembled Ga0374207_688915_1_465 | glutathione-independent formaldehyde dehydrogenase | 465 | 155 | EC:1.2.1.46 - Formaldehyde dehydrogenase |
| 3300034422 assembled Ga0374207_320338_1_162 | glutathione-independent formaldehyde dehydrogenase | 162 | 54 | EC:1.2.1.46 - Formaldehyde dehydrogenase |
| 3300034949 assembled Ga0374202_0814046_45_473 | glutathione-independent formaldehyde dehydrogenase | 429 | 143 | EC:1.2.1.46 - Formaldehyde dehydrogenase |
| 3300034949 assembled Ga0374202_0432018_78_353 | glutathione-independent formaldehyde dehydrogenase | 276 | 92 | EC:1.2.1.46 - Formaldehyde dehydrogenase |
| 3300034949 assembled Ga0374202_0326245_2_457 | glutathione-independent formaldehyde dehydrogenase | 456 | 152 | EC:1.2.1.46 - Formaldehyde dehydrogenase |
| 3300034385 assembled Ga0374210_0470481_31_330 | glutathione-independent formaldehyde dehydrogenase | 300 | 100 | EC:1.2.1.46 - Formaldehyde dehydrogenase |
| 3300034385 assembled Ga0374210_0421317_89_964 | glutathione-independent formaldehyde dehydrogenase | 876 | 292 | EC:1.2.1.46 - Formaldehyde dehydrogenase |
| 3300034422 assembled Ga0374207_277196_3_305 | glutathione-independent formaldehyde dehydrogenase | 303 | 101 | EC:1.2.1.46 - Formaldehyde dehydrogenase |
| 3300034949 assembled Ga0374202_0278763_13_393 | glutathione-independent formaldehyde dehydrogenase | 381 | 127 | EC:1.2.1.46 - Formaldehyde dehydrogenase |
| 3300034422 assembled Ga0374207_810787_2_376 | glutathione-independent formaldehyde dehydrogenase | 375 | 125 | EC:1.2.1.46 - Formaldehyde dehydrogenase |
| 3300034949 assembled Ga0374202_0638910_2_481 | glutathione-independent formaldehyde dehydrogenase | 480 | 160 | EC:1.2.1.46 - Formaldehyde dehydrogenase |
| 3300034949 assembled Ga0374202_0754095_1_447 | glutathione-independent formaldehyde dehydrogenase | 447 | 149 | EC:1.2.1.46 - Formaldehyde dehydrogenase |
| 3300034949 assembled Ga0374202_0335270_3_383 | glutathione-independent formaldehyde dehydrogenase | 381 | 127 | EC:1.2.1.46 - Formaldehyde dehydrogenase |
| 3300034385 assembled Ga0374210_1372241_27_329 | glutathione-independent formaldehyde dehydrogenase | 303 | 101 | EC:1.2.1.46 - Formaldehyde dehydrogenase |
| 3300034949 assembled Ga0374202_0204320_5_325 | glutathione-independent formaldehyde dehydrogenase | 321 | 107 | EC:1.2.1.46 - Formaldehyde dehydrogenase |
| 3300034385 assembled Ga0374210_0464847_3_323 | glutathione-independent formaldehyde dehydrogenase | 321 | 107 | EC:1.2.1.46 - Formaldehyde dehydrogenase |
| 3300034422 assembled Ga0374207_429286_105_767 | glutathione-independent formaldehyde dehydrogenase | 663 | 221 | EC:1.2.1.46 - Formaldehyde dehydrogenase |
| 3300034385 assembled Ga0374210_0649899_9_182 | glutathione-independent formaldehyde dehydrogenase | 174 | 57 | EC:1.2.1.46 - Formaldehyde dehydrogenase |
| 3300034949 assembled Ga0374202_0503408_2_262 | glutathione-independent formaldehyde dehydrogenase | 261 | 87 | EC:1.2.1.46 - Formaldehyde dehydrogenase |
| 3300034385 assembled Ga0374210_0796723_274_615 | glutathione-independent formaldehyde dehydrogenase | 342 | 113 | EC:1.2.1.46 - Formaldehyde dehydrogenase |
| 3300034422 assembled Ga0374207_130865_8_193 | glutathione-independent formaldehyde dehydrogenase | 186 | 61 | EC:1.2.1.46 - Formaldehyde dehydrogenase |
| 3300034385 assembled Ga0374210_0591018_4_651 | glutathione-independent formaldehyde dehydrogenase | 648 | 216 | EC:1.2.1.46 - Formaldehyde dehydrogenase |
| 3300034385 assembled Ga0374210_0546205_108_320 | glutathione-independent formaldehyde dehydrogenase | 213 | 70 | EC:1.2.1.46 - Formaldehyde dehydrogenase |
| 3300034385 assembled Ga0374210_0935152_2_271 | glutathione-independent formaldehyde dehydrogenase | 270 | 90 | EC:1.2.1.46 - Formaldehyde dehydrogenase |
| 3300034385 assembled Ga0374210_0282017_3_377 | glutathione-independent formaldehyde dehydrogenase | 375 | 125 | EC:1.2.1.46 - Formaldehyde dehydrogenase |
| 3300034422 assembled Ga0374207_186670_18_551 | glutathione-independent formaldehyde dehydrogenase | 534 | 178 | EC:1.2.1.46 - Formaldehyde dehydrogenase |
| 3300034949 assembled Ga0374202_0042833_2_418 | glutathione-independent formaldehyde dehydrogenase | 417 | 139 | EC:1.2.1.46 - Formaldehyde dehydrogenase |
| 3300034949 assembled Ga0374202_0213242_2_337 | glutathione-independent formaldehyde dehydrogenase | 336 | 112 | EC:1.2.1.46 - Formaldehyde dehydrogenase |
| 3300034949 assembled Ga0374202_0495810_3_419 | glutathione-independent formaldehyde dehydrogenase | 417 | 139 | EC:1.2.1.46 - Formaldehyde dehydrogenase |
| 3300034422 assembled Ga0374207_110100_99_434 | glutathione-independent formaldehyde dehydrogenase | 336 | 111 | EC:1.2.1.46 - Formaldehyde dehydrogenase |
| 3300034385 assembled Ga0374210_0991082_2_301 | glutathione-independent formaldehyde dehydrogenase | 300 | 100 | EC:1.2.1.46 - Formaldehyde dehydrogenase |
| 3300034949 assembled Ga0374202_0834863_1_282 | glutathione-independent formaldehyde dehydrogenase | 282 | 94 | EC:1.2.1.46 - Formaldehyde dehydrogenase |
| 3300034949 assembled Ga0374202_0473193_6_314 | glutathione-independent formaldehyde dehydrogenase | 309 | 103 | EC:1.2.1.46 - Formaldehyde dehydrogenase |
| 3300034385 assembled Ga0374210_1181924_17_334 | glutathione-independent formaldehyde dehydrogenase | 318 | 106 | EC:1.2.1.46 - Formaldehyde dehydrogenase |
| 3300034949 assembled Ga0374202_0149201_287_466 | glutathione-independent formaldehyde dehydrogenase | 180 | 59 | EC:1.2.1.46 - Formaldehyde dehydrogenase |
| 3300034385 assembled Ga0374210_0821086_3_308 | glutathione-independent formaldehyde dehydrogenase | 306 | 102 | EC:1.2.1.46 - Formaldehyde dehydrogenase |
| 3300034422 assembled Ga0374207_169001_2_151 | glutathione-independent formaldehyde dehydrogenase | 150 | 49 | EC:1.2.1.46 - Formaldehyde dehydrogenase |
| 3300034385 assembled Ga0374210_1184041_28_300 | glutathione-independent formaldehyde dehydrogenase | 273 | 91 | EC:1.2.1.46 - Formaldehyde dehydrogenase |
| 3300034385 assembled Ga0374210_1374583_2_334 | glutathione-independent formaldehyde dehydrogenase | 333 | 111 | EC:1.2.1.46 - Formaldehyde dehydrogenase |
| 3300034949 assembled Ga0374202_1234907_35_319 | glutathione-independent formaldehyde dehydrogenase | 285 | 94 | EC:1.2.1.46 - Formaldehyde dehydrogenase |
| 3300034385 assembled Ga0374210_0062706_3_311 | glutathione-independent formaldehyde dehydrogenase | 309 | 102 | EC:1.2.1.46 - Formaldehyde dehydrogenase |
| 3300034385 assembled Ga0374210_0279586_3_419 | glutathione-independent formaldehyde dehydrogenase | 417 | 139 | EC:1.2.1.46 - Formaldehyde dehydrogenase |
| 3300034385 assembled Ga0374210_0950915_1_429 | glutathione-independent formaldehyde dehydrogenase | 429 | 143 | EC:1.2.1.46 - Formaldehyde dehydrogenase |
| 3300034385 assembled Ga0374210_1575839_3_356 | glutathione-independent formaldehyde dehydrogenase | 354 | 117 | EC:1.2.1.46 - Formaldehyde dehydrogenase |
| 3300034949 assembled Ga0374202_0365022_382_495 | glutathione-independent formaldehyde dehydrogenase | 114 | 37 | EC:1.2.1.46 - Formaldehyde dehydrogenase |
| 3300034422 assembled Ga0374207_231872_3_347 | glutathione-independent formaldehyde dehydrogenase | 345 | 115 | EC:1.2.1.46 - Formaldehyde dehydrogenase |
| 3300034949 assembled Ga0374202_0741736_47_355 | glutathione-independent formaldehyde dehydrogenase | 309 | 103 | EC:1.2.1.46 - Formaldehyde dehydrogenase |
| 3300034385 assembled Ga0374210_0199596_1_306 | glutathione-independent formaldehyde dehydrogenase | 306 | 101 | EC:1.2.1.46 - Formaldehyde dehydrogenase |
| 3300034949 assembled Ga0374202_0520665_3_425 | glutathione-independent formaldehyde dehydrogenase | 423 | 141 | EC:1.2.1.46 - Formaldehyde dehydrogenase |
| 3300034422 assembled Ga0374207_501766_49_390 | glutathione-independent formaldehyde dehydrogenase | 342 | 113 | EC:1.2.1.46 - Formaldehyde dehydrogenase |
| 3300034385 assembled Ga0374210_0799273_1_345 | glutathione-independent formaldehyde dehydrogenase | 345 | 115 | EC:1.2.1.46 - Formaldehyde dehydrogenase |
| 3300034385 assembled Ga0374210_1411341_2_565 | glutathione-independent formaldehyde dehydrogenase | 564 | 188 | EC:1.2.1.46 - Formaldehyde dehydrogenase |
| 3300034949 assembled Ga0374202_0558031_1_234 | glutathione-independent formaldehyde dehydrogenase | 234 | 77 | EC:1.2.1.46 - Formaldehyde dehydrogenase |
| 3300034949 assembled Ga0374202_0803272_250_426 | glutathione-independent formaldehyde dehydrogenase | 177 | 59 | EC:1.2.1.46 - Formaldehyde dehydrogenase |
| 3300034385 assembled Ga0374210_1242996_41_562 | glutathione-independent formaldehyde dehydrogenase | 522 | 173 | EC:1.2.1.46 - Formaldehyde dehydrogenase |
| 3300034949 assembled Ga0374202_1333717_1_336 | glutathione-independent formaldehyde dehydrogenase | 336 | 112 | EC:1.2.1.46 - Formaldehyde dehydrogenase |
| 3300034949 assembled Ga0374202_0782301_10_471 | glutathione-independent formaldehyde dehydrogenase | 462 | 154 | EC:1.2.1.46 - Formaldehyde dehydrogenase |
| 3300034949 assembled Ga0374202_0812198_74_385 | glutathione-independent formaldehyde dehydrogenase | 312 | 104 | EC:1.2.1.46 - Formaldehyde dehydrogenase |
| 3300034385 assembled Ga0374210_0850400_232_408 | glutathione-independent formaldehyde dehydrogenase | 177 | 58 | EC:1.2.1.46 - Formaldehyde dehydrogenase |
| 3300034385 assembled Ga0374210_0917027_455_550 | glutathione-independent formaldehyde dehydrogenase | 96 | 32 | EC:1.2.1.46 - Formaldehyde dehydrogenase |
| 3300034385 assembled Ga0374210_0851386_3_140 | glutathione-independent formaldehyde dehydrogenase | 138 | 45 | EC:1.2.1.46 - Formaldehyde dehydrogenase |
| 3300034385 assembled Ga0374210_1294442_3_371 | glutathione-independent formaldehyde dehydrogenase | 369 | 123 | EC:1.2.1.46 - Formaldehyde dehydrogenase |
| 3300034385 assembled Ga0374210_1014222_1_309 | glutathione-independent formaldehyde dehydrogenase | 309 | 103 | EC:1.2.1.46 - Formaldehyde dehydrogenase |
| 3300034385 assembled Ga0374210_0724873_149_427 | glutathione-independent formaldehyde dehydrogenase | 279 | 93 | EC:1.2.1.46 - Formaldehyde dehydrogenase |
| 3300034385 assembled Ga0374210_0350229_3_506 | glutathione-independent formaldehyde dehydrogenase | 504 | 168 | EC:1.2.1.46 - Formaldehyde dehydrogenase |
| 3300034949 assembled Ga0374202_1159002_177_425 | glutathione-independent formaldehyde dehydrogenase | 249 | 83 | EC:1.2.1.46 - Formaldehyde dehydrogenase |
| 3300034385 assembled Ga0374210_0696159_2_412 | glutathione-independent formaldehyde dehydrogenase | 411 | 136 | EC:1.2.1.46 - Formaldehyde dehydrogenase |
| 3300034422 assembled Ga0374207_649988_1_474 | glutathione-independent formaldehyde dehydrogenase | 474 | 158 | EC:1.2.1.46 - Formaldehyde dehydrogenase |
| 3300034385 assembled Ga0374210_1258686_2_319 | glutathione-independent formaldehyde dehydrogenase | 318 | 106 | EC:1.2.1.46 - Formaldehyde dehydrogenase |
| 3300034949 assembled Ga0374202_0301851_19_384 | glutathione-independent formaldehyde dehydrogenase | 366 | 122 | EC:1.2.1.46 - Formaldehyde dehydrogenase |
| 3300034949 assembled Ga0374202_0500757_370_483 | glutathione-independent formaldehyde dehydrogenase | 114 | 37 | EC:1.2.1.46 - Formaldehyde dehydrogenase |
| 3300034949 assembled Ga0374202_1374523_1_453 | methane monooxygenase component A alpha chain/propane monooxygenase large subunit | 453 | 150 | EC:1.14.13.- - Oxidoreductases |

­­­­­

**Table S5.** Genes upstream and downstream of *mmoX* in MAG #21, contig k141_435455, as predicted using both the Pfam database and NCBI Blastp with default parameters.

| Gene Location | Pfam number | Pfam Protein match | e-value | BLASTp organism match | BLAST match | Percent identity | Query Cover |
| --- | --- | --- | --- | --- | --- | --- | --- |
| gene_573132\|GeneMark.hmm\|177_aa\|-\|277\|810 | PF01797.15 | Transposase IS200 like | 3.7e-06 | Planctomycetes bacterium/ Chloroflexi bacterium | transposase/ hypothetical protein | 49.38%/ 48.17% | 89%/ 92% |
| gene_573133\|GeneMark.hmm\|284_aa\|-\|889\|1743 | PF04321.16 | RmlD substrate binding domain | 9.5e-81 | Dehalococcoidia bacterium (Chlorofexi) | dTDP-4-dehydrorhamnose reductase | 77.03% | 99% |
| gene_573134\|GeneMark.hmm\|205_aa\|-\|1787\|2404 | PF05138.11 | Phenylacetic acid catabolic protein | 0.0064 | Chloroflexi bacterium | hypothetical protein | 85.85% | 100% |
| gene_573135\|GeneMark.hmm\|106_aa\|-\|2404\|2724 | PF12838.6 | 4Fe-4S dicluster domain | 2e-09 | Dehalococcoidia bacterium (Chloroflexi) | 4Fe-4S dicluster domain-containing protein | 85.39% | 83% |
|  | PF13484.5 | 4Fe-4S double cluster binding domain | 5.5e-09 |  |  |  |  |
| gene_573136\|GeneMark.hmm\|268_aa\|-\|2728\|3534 | Custom Pfam database | **Methane monooxygenase (mmoX)** | 6.6e-07 | Chloroflexi bacterium | hypothetical protein | 62.83% | 97% |
|  | PF04305.13 | Protein of unknown function (DUF455) | 4.2e-07 |  |  |  |  |
|  | PF02332.17 | Methane/Phenol/Toluene Hydroxylase | 0.021 |  |  |  |  |
| gene_573137\|GeneMark.hmm\|210_aa\|+\|3684\|4316 | PF04263.15 | Thiamin pyrophosphokinase, catalytic domain | 4.5e-19 | Dehalococcoidia bacterium (Chloroflexi) | thiamine diphosphokinase | 68.57% | 100% |
| gene_573138\|GeneMark.hmm\|266_aa\|+\|4419\|5219 | PF14357.5 | Domain of unknown function (DUF4404) | 0.018 | Dehalococcoidia bacterium (Chloroflexi) | hypothetical protein | 61.45% | 93% |
| gene_573139\|GeneMark.hmm\|302_aa\|+\|5422\|6330 | PF00701.21 | Dihydrodipicolinate synthetase family (DHDPS) | 8.1e-81 | Chloroflexi bacterium | 4-hydroxy-tetrahydrodipicolinate synthase | 88.53% | 98% |
| gene_573140\|GeneMark.hmm\|124_aa\|+\|6337\|6711 | PF09413.9 | Putative prokaryotic signal transducing protein (DUF2007) | 8.6e-06 | Dehalococcoidia bacterium | hypothetical protein | 40.54% | 89% |
| gene_573141\|GeneMark.hmm\|178_aa\|-\|6708\|7244 | PF11495.7 | Archaeal transcriptional regulator TrmB | 8.4e-06 | Bacteria | hypothetical protein | 65.32% | 97% |
| gene_573142\|GeneMark.hmm\|190_aa\|-\|7311\|7883 | PF00132.23 | Bacterial transferase hexapeptide (six repeats) | 3.6e-15 | Dehalococcoidia bacterium (Chloroflexi) | gamma carbonic anhydrase family protein | 91.21% | 95% |
| gene_573143\|GeneMark.hmm\|611_aa\|-\|7910\|9745 | PF00005.26 | ABC transporter | 3.2e-16 | Micromonospora inaquosa | ABC subunit UvrA | 83.63% | 100% |
| gene_573144\|GeneMark.hmm\|191_aa\|-\|9688\|10263 | PF03193.15 | RsgA GTPase | 6e-05 | Dehalococcoidia bacterium (Chloroflexi) | ABC subunit UvrA | 90.73% | 79% |
| gene_573145\|GeneMark.hmm\|131_aa\|-\|10305\|10700 | PF00903.24 | Glyoxalase/Bleomycin resistance protein/Dioxygenase superfamily | 3.4e-15 | Candidatus Eisenbacteria bacterium | VOC family protein | 86.61% | 96% |
| gene_573146\|GeneMark.hmm\|206_aa\|+\|10780\|11397 | PF04542.13 | Sigma-70, region 2 | 1e-15 | Dehalococcoidia bacterium (Chloroflexi) | sigma-70 family RNA polymerase sigma factor | 77.78% | 100% |
|  | PF08281.11 | Sigma-70, region 4 | 1.3e-11 |  |  |  |  |

**Table S6.** Genes upstream and downstream of *pmoB* on MAGs #8 and #16, contigs k141_54367 and k141_62597, respectively, as predicted using the Pfam database.

| Gene Location | Pfam number | Pfam protein match | e-value |
| --- | --- | --- | --- |
| **MAG 8** |  |  |  |
| gene_71784\|GeneMark.hmm\|87_aa\|-\|3\|263 - | PF03971.13 | IDH Monomeric isocitrate dehydrogenase | 5.80E-33 |
| **gene_71785\|GeneMark.hmm\|163_aa\|-\|515\|1006 -** | PF02036.16 | SCP2 sterol transfer family | 3.00E-08 |
|  | **-** | **PmoB** | **0.2** |
| gene_71786\|GeneMark.hmm\|292_aa\|-\|1003\|1881 - | PF01136.18 | Peptidase_U32 | 2.90E-18 |
| gene_71787\|GeneMark.hmm\|357_aa\|-\|1997\|3070 - | PF01136.18 | Peptidase_U32 | 1.30E-65 |
|  | PF04445.12 | SAM_MT SAM-dependent methyltransferase | 0.0074 |
| gene_71788\|GeneMark.hmm\|95_aa\|+\|3232\|3519 - | PF08369.9 | PCP_red Proto-chlorophyllide reductase 57 kD subunit | 0.011 |
|  | PF01402.20 | RHH_1 Ribbon-helix-helix protein, copG family | 0.032 |
| gene_71789\|GeneMark.hmm\|186_aa\|-\|3494\|4054 - | PF07152.11 | YaeQ | 7.10E-82 |
| gene_71790\|GeneMark.hmm\|266_aa\|-\|4222\|5022 - | PF13616.5 | Rotamase_3 domain | 2.40E-21 |
|  | PF13145.5 | Rotamase_2 domain | 2.10E-19 |
|  | PF00639.20 | Rotamase domain | 2.50E-15 |
| gene_71791\|GeneMark.hmm\|90_aa\|-\|5051\|5323 - | PF01722.17 | BolA-like protein family | 6.50E-29 |
| gene_71792\|GeneMark.hmm\|179_aa\|-\|5331\|5870 - | PF04279.14 | IspA Intracellular septation protein A | 8.90E-72 |
|  | PF03203.13 | MerC mercury resistance protein | 0.034 |
|  | PF11222.7 | DUF3017 protein of unknown function | 0.47 |
| gene_71793\|GeneMark.hmm\|130_aa\|-\|5967\|6359 - | PF01641.17 | SelR domain | 4.60E-55 |
|  | PF03226.13 | Yippee-Mis18 zinc-binding/DNA-binding, centromere assembly | 0.00077 |
| gene_71794\|GeneMark.hmm\|118_aa\|+\|6509\|6865 - | PF02627.19 | CMD Carboxymuconolactone decarboxylase family | 3.00E-19 |
|  | PF10778.8 | DehI Halocarboxylic acid dehydrogenase DehI | 0.01 |
| gene_71795\|GeneMark.hmm\|345_aa\|-\|7012\|8049 - | PF02696.13 | YdiU/UPF0061 family, Uncharacterized ACR | 2.00E-96 |
| **MAG 16** |  |  |  |
| gene_82597\|GeneMark.hmm\|65_aa\|+\|2\|199 | PF13192.5 | Thioredoxin_3 domain | 0.036 |
| gene_82598\|GeneMark.hmm\|69_aa\|+\|614\|823 | PF11455.7 | DUF3018 Protein of unknown function | 9.50E-31 |
| gene_82599\|GeneMark.hmm\|54_aa\|+\|820\|984 | PF02452.16 | PemK-like, MazF-like toxin of type II toxin-antitoxin system | 1.60E-07 |
| gene_82600\|GeneMark.hmm\|77_aa\|+\|1182\|1415 | PF14178.5 | YppF-like protein | 0.0022 |
| gene_82601\|GeneMark.hmm\|313_aa\|-\|1695\|2636 | PF07804.11 | HipA_C HipA-like C-terminal domain | 1.80E-21 |
| **gene_82604\|GeneMark.hmm\|272_aa\|-\|3767\|4585** | PF07804.11 | HipA_C HipA-like C-terminal domain | 3.20E-42 |
|  | - | **PmoB** | **0.088** |
|  | **PF04744.11** | **Monooxygenase_B** | **0.19** |

**Table S7.** Ammonia assimilation, nitrate and nitrite ammonification, and denitrification genes found in MAG #8 of the from the ^13^CH_4_-SIP metagenomes based on the Pfam database and HMMER assignments.

| **Gene** | **Process** |
| --- | --- |
| Ferredoxin-dependent glutamate synthase | Ammonia Assimilation |
| Nitrogen regulatory protein P-II | Ammonia Assimilation |
| Glutamate-ammonia-ligase adenylyltransferase | Ammonia Assimilation |
| Ammonium transporter | Ammonia Assimilation |
| Glutamate synthase [NADPH] large chain | Ammonia Assimilation |
| Glutamine synthetase type I | Ammonia Assimilation |
| [Protein-PII] uridylyltransferase | Ammonia Assimilation |
| Glutamate synthase [NADPH] small chain | Ammonia Assimilation |
| NnrS protein involved in response to NO | Nitrate and nitrite ammonification |
| Nitrite-sensitive transcriptional repressor NsrR | Nitrate and nitrite ammonification |
| Nitric-oxide reductase, quinol-dependent | Nitrate and nitrite ammonification |
| Nitrite reductase probable [NAD(P)H] subunit | Nitrate and nitrite ammonification |
| Nitrate/nitrite sensor protein | Nitrate and nitrite ammonification |
| Nitrate/nitrite response regulator protein | Nitrate and nitrite ammonification |
| NnrS protein involved in response to NO | Denitrification |
| NnrU family prot. required for expression of NO & NO2^−^ reductases (Nir & Nor) | Denitrification |
| Nitric oxide reductase activation protein NorQ | Denitrification |
| Nitric oxide responding transcriptional regulator Dnr (Crp/Fnr family) | Denitrification |
| Nitric oxide reductase, quinol-dependent | Denitrification |
| Nitric oxide reductase activation protein NorD | Denitrification |
| Nitric oxide reductase activation protein NorQ | Denitrification |
